# Protein markers of ovarian cancer and its subtypes: insights from proteome-wide Mendelian randomisation analysis

**DOI:** 10.1038/s41416-025-03143-w

**Published:** 2025-08-28

**Authors:** Anwar Mulugeta, David Stacey, Amanda L. Lumsden, Iqbal Madakkatel, S. Hong Lee, Johanna Mäenpää, Martin K. Oehler, Elina Hyppönen

**Affiliations:** 1 Australian Centre for Precision Health, Unit of Clinical and Health Sciences, University of South Australia, Adelaide, SA Australia; 2 South Australian Health and Medical Research Institute, Adelaide, SA Australia; 3 Department of Pharmacology and Clinical Pharmacy, College of Health Sciences, Addis Ababa University, Addis Ababa, Ethiopia; 4 UniSA Allied Health & Human Performance, University of South Australia, Adelaide, SA Australia; 5 Faculty of Medicine and Medical Technology, Tampere University, Tampere, Finland; 6 Cancer Centre, Tampere University Hospital, Tampere, Finland; 7 Department of Gynaecological Oncology, Royal Adelaide Hospital, Adelaide, SA Australia; 8 Adelaide Medical School, Robinson Research Institute, University of Adelaide, Adelaide, SA Australia

**Keywords:** Predictive markers, Ovarian cancer, Target identification

## Abstract

**Background:**

Ovarian cancer (OC) is often diagnosed at an advanced stage when prognosis is poor. We aimed to identify blood plasma proteins predictive of OC risk.

**Methods:**

We conducted proteome-wide Mendelian randomisation (MR) analyses using summary-level protein quantitative trait locus data covering 2337 plasma proteins, and genome-wide association data on OC and its subtypes (up to 25,509 cases) from the Ovarian Cancer Association Consortium. Wald ratio or inverse-variance weighted MR analysis was used as the primary method, depending on the number of instruments. We evaluated pleiotropy using MR-Egger intercept test and leave-one-out analysis.

**Results:**

From 2337 plasma proteins, 12 were associated (*p* < 7.4 × 10^−5^) with OC or its subtypes. Robust evidence linked follitropin subunit beta (FSHB) with endometrioid OC (per SD higher, OR 2.41, 95% CI 1.56, 3.71). Associations for the other 11 proteins could be explained by pleiotropy from *ABO* or *MAPT-AS1* loci. We identified 12 suggestive associations with OC or its subtypes at nominal threshold (*p* < 0.05), involving 11 plasma proteins, with no evidence of pleiotropy from leave-one-out and MR-Egger intercept tests (*P*_intercept_ > 0.17). Potential drug targets were identified for follitropin receptor and eight other proteins.

**Conclusion:**

Our study suggests FSHB and 11 additional plasma proteins as of potential interest in OC (or subtypes) prognosis, mostly representing potentially druggable targets.

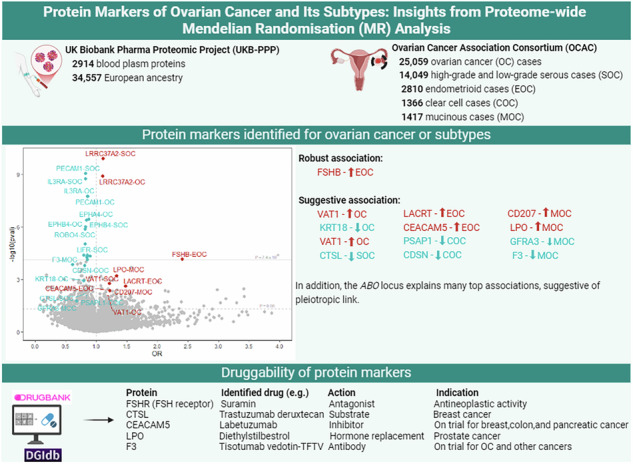

## Introduction

Ovarian cancer (OC) is the most lethal gynaecological cancer, representing 47% of all deaths within female genital tract cancers [1]. As the disease is commonly diagnosed at a late stage, survival rates for OC remain low, with five-year survival less than 30% among those with stage III and IV cancer at diagnosis [2]. In contrast, over 90% of women diagnosed at stage I survive for the next five years, underscoring the importance of early detection in reducing OC mortality [2]. Current screening practices involve a combination of blood-based biomarkers, such as cancer antigen 125 (CA125), and transvaginal ultrasound imaging, especially for individuals with a strong family history such as due to or germline mutations in *BRCA1* and *BRCA2* genes [3]. However, as CA125 is not always elevated at an early stage of OC, and as ultrasound imaging may fail to detect very small tumours [3] there is a need to enhance existing screening approaches. Beyond screening, identifying protein markers predictive of OC can contribute to a deeper understanding of the underlying biology of OC and may help to discover promising targets for pharmacological intervention.

Proteome-wide association studies have played an important role in uncovering protein markers associated with diseases, providing insight into the underlying biology of these conditions and further facilitating drug repositioning [4, 5]. For example, a proteome-wide screen identified an association between plasma kallikrein (KLKB1) and venous thromboembolism, suggesting potential to repurpose ecallantide, a drug that targets this protein and is currently used in the treatment of hereditary angioedema [4]. Recent applications have also extended the use of the Mendelian randomisation (MR) approach to the context of proteome-wide analyses [4]. MR is a genetic approach which can be used to assess evidence for a causal association [6], and in the context of proteomic studies, this method utilises genetic predictors of protein levels (protein quantitative trait loci, pQTLs) to instrument the actual circulating concentrations. A valid analysis requires three core assumptions [6]; (1) pQTLs should be associated with blood plasma protein levels, (2) pQTLs should not be associated with confounders of the plasma protein-OC association, and (3) pQTLs should affect OC only through influencing plasma protein level. A recent proteome-wide MR study utilised 1276 proteins from the ARIC (Atherosclerosis Risk in Communities) study, identifying 93 proteins associated with at least one of the four cancers investigated [7]. However, none of these proteins were found to be associated with OC [7]. Another MR used 1329 proteins from 3301 healthy individuals in the INTERVAL study, identifying 26 proteins associated with OC, of which 22 were explained by the pleiotropic genetic variant from *ABO* locus [8]. For the remaining four proteins (MFAP2, SEMG2, DLK1, and NTNG1), a potential causal link with OC was identified. In this study, we extend on these previous analyses in three ways. First, we seek to identify proteins not only associated with epithelial OC but also several OC subtypes. Second, we utilise pQTL summary data for 2414 blood plasma proteins measured in over 54,000 UK Biobank participants, which is the most highly powered pQTL study to date thus enabling us to instrument additional protein markers with higher precision. Third, given that proteins are key targets for most drugs and act as proximal effectors of biological processes encoded by the genome, we searched multiple drug databases indexing the identified protein markers to explore opportunities for drug repurposing.

## Methods

### Data sources for ovarian cancer summary results

We used summary-level genetic data for epithelial OC from the largest genome-wide association study (GWAS) dataset provided by the Ovarian Cancer Association Consortium (OCAC) [9]. This dataset was generated from a European ancestry population, comprising 25,509 epithelial OC cases and 40,941 controls. The OCAC GWAS also includes histotype-specific summary results, covering high-grade and low-grade serous OC (14,049 cases, here after referred as serous OC), endometrioid OC (2810 cases), clear cell OC (1366 cases), and mucinous OC (1417 cases).

### Data source for blood plasma protein summary results

We derived GWAS summary-level data on proteome from the UK Biobank Pharma Proteomics Project (UKB-PPP) [10]. The UKB-PPP used blood plasma samples from 54,219 participants, covering 2923 proteomic markers acquired through the Olink platform (Olink Explore 3072 antibody-based proximal extension assay). The discovery analysis involved 34,557 participants of European ancestry from the UK Biobank, identifying genetic variant associations with 2414 unique proteins under significance threshold of *p* < 1.7 × 10^−11^ [10]. This threshold was established to accommodate the multiple tests performed for the 2923 proteins on the conventional GWAS threshold (5 × 10^−08^/2923). For the MR analysis, we used both cis-pQTL (within 1 Mb from the gene encoding the protein) and trans-pQTL (>1 Mb from the gene encoding proteins) (Supplementary Table 1). This approach enables us to test over 600 proteins that have only trans-pQTLs (Supplementary Table 1), also enhancing the analytical power. Previous studies have typically been conducted using a single instrument [8], while using a broader range of instruments will allow us to assess horizontal pleiotropy potentially explaining associations across multiple loci.

### Statistical analysis

We examined evidence for a causal association between blood plasma protein levels and the risk of epithelial OC and its subtypes using a two-sample MR approach. We evaluated the strength of the genetic instruments (pQTLs) by calculating F-statistics, ensuring that all variants had an F-statistic of 10 or above to mitigate potential weak instrument bias [11]. We extracted the pQTL-OC associations from the OCAC database. If data were unavailable, we either used a proxy in high Linkage Disequilibrium (LD, r^2^ ≥ 0.8) or excluded the variant from the analysis. We used Wald ratio or inverse-variance weighted (IVW) MR analyses for single or two and above pQTLs, respectively. Where proteins had three or more pQTLs, we performed sensitivity analyses using weighted median [12], weighted mode [13], and MR-Egger [14] approaches, allowing us to obtain causal estimates under different pleiotropic assumptions. We assessed pleiotropy using the MR-Egger intercept test, while appreciating that the ability of the method is limited when using a small number of instruments [14]. We additionally conducted leave-one-out analyses to assess the sensitivity of the observed association on the influences by individual variants. For protein markers identified, we further repeated the MR analysis using cis-pQTLs to replicate the findings under a reduced likelihood of pleiotropic effects. We also conducted the MR-Steiger test to confirm the directionality of the association, testing that the directionality of the genetic instrument is to the exposure rather than the outcome [15]. We corrected for multiple testing using False discovery rate (FDR) when selecting the protein signals associated with OC and the subtypes. For protein markers that passed the FDR threshold and passed the pleiotropy tests, we further analyse to examine evidence on the robustness of the association. First, we conducted colocalisation analysis to minimise the bias coming from linkage disequilibrium. We used the *coloc* package in R for testing the following five hypotheses: (1) no association with any trait (posterior probability hypothesis 0, PPH0); (2) Only associated with trait 1 (PPH1); (3) Only associated with trait 2 (PPH2); (4) Two traits are correlated, but two traits have different causal variants (PPH3); (5) Two traits are related, and share the same causal variants (PPH4). With the evidence of PPH4 > 0.8 considered as high support of evidence for colocalisation while values between 0.5 and 0.8 considered as suggestive evidence of colocalisation. Second, we repeated the analyses using independent Olink-based proteome data generated from KARMA study [16], and four other independent SomaScan-based data from the INTERVAL, KORA, Fenland and deCODE studies, where valid genetic instruments were available [17–20]. Protein signals above the FDR threshold and below the nominal significance level (*p* < 0.05) were classified as having “suggestive evidence of association”. The effect estimates for the causal association between blood plasma protein and OC are given as odds ratios (ORs) with 95% confidence intervals (CIs) per one standard deviation (SD) higher blood plasma protein level.

Given drug targets with human genetic evidence of disease association are twice as likely to lead to approved drugs [21], and most drug targets are proteins [22], we conducted a druggability evaluation for proteins showing evidence for a potential causal association. To assess the suitability of these proteins as potential drug targets for OC management, we explored drugs (approved or investigational) targeting these proteins using DrugBank [23] and the Drug-Gene Interaction Database (DGIdb) [24].

Statistical analyses were performed using STATA SE version 17.1 and R version 4.2.1.

## Results

**Fig. 1:**
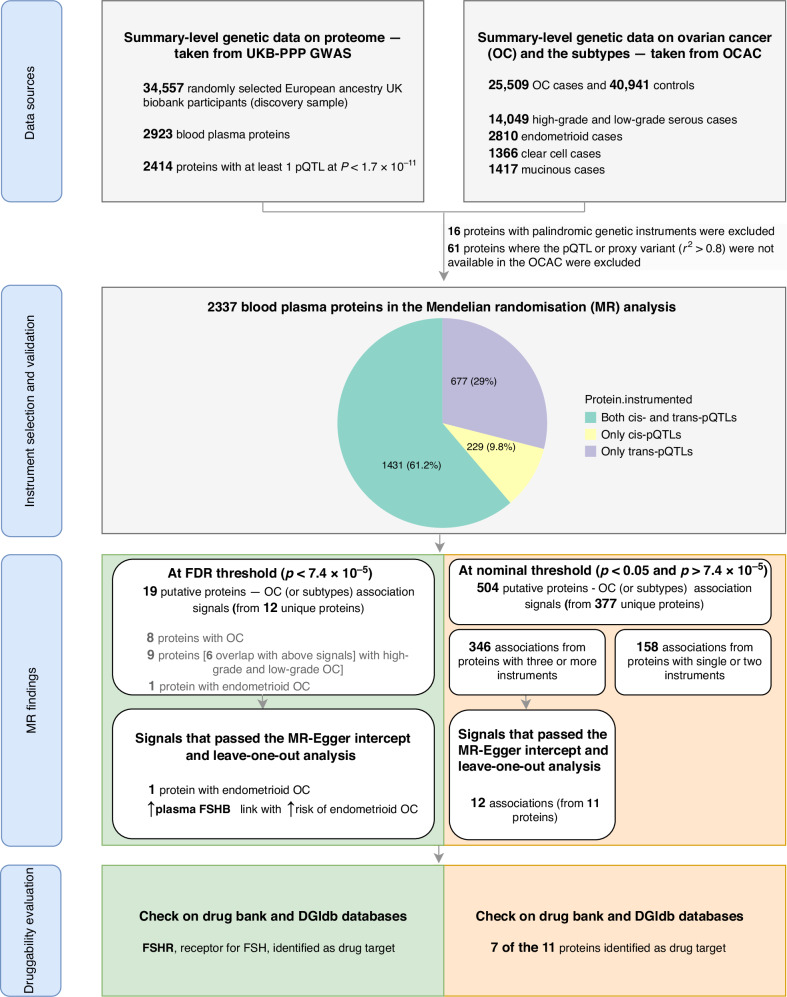
Overview of the study design.

The study’s overview, illustrating the data sources, instrument selection strategy, and analysis approach, is presented in Fig. 1. Out of the 2414 proteins with at least one pQTL, we included 2337 proteins with valid instruments (Supplementary Table 1), excluding 16 proteins with palindromic instruments and 66 proteins where the pQTL or proxy variant (r^2^ ≥ 0.8) were not available in the OCAC. Of the included proteins, 1431 proteins were instrumented by both cis- and trans-pQTLs, 677 proteins by only trans-pQTLs, and 229 proteins by only cis-pQTLs (Fig. 1). The median number of pQTLs used per protein was five (with interquartile range of three to eight). The F-statistic for all included pQTls was 45 or higher.

### Mendelian randomisation

**Fig. 2:**
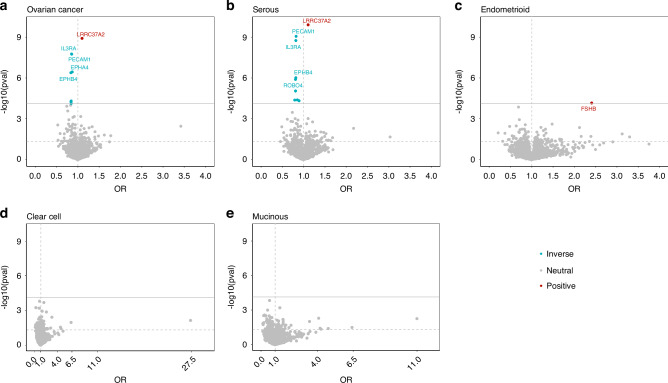
Volcano plot showing the proteome-wide Mendelian randomisation findings. The *Y*-axis represents *p* values on a log_10_ scale for the association between the blood plasma proteome and ovarian cancer (or its subtypes), while the *X*-axis represents the odds ratio effect estimates. Volcano plots in panels **a**–**e** represent ovarian cancer, serous ovarian cancer, endometrioid ovarian cancer, clear cell ovarian cancer, and mucinous ovarian cancer, respectively.

Figure 2 illustrates the distribution of the MR estimates along with their corresponding *p*-values for each protein-OC (or subtype) association. Signals that passed the FDR correction (*p* < 7.4 × 10^−5^) are annotated with the respective protein names. Using the IVW method, we identified a total of 18 signals for proteins associated with OC. Among these signals, eight proteins were associated with epithelial OC, nine proteins (six of which overlapped with epithelial OC signals) were associated with serous OC, and one protein was associated with endometrioid OC (Supplementary Table 2). All the proteins linked with serous OC were also identified in high-grade serous OC (13,037 cases), while none were identified for low-grade serous OC (1012 cases, Supplementary Table 3).

**Fig. 3:**
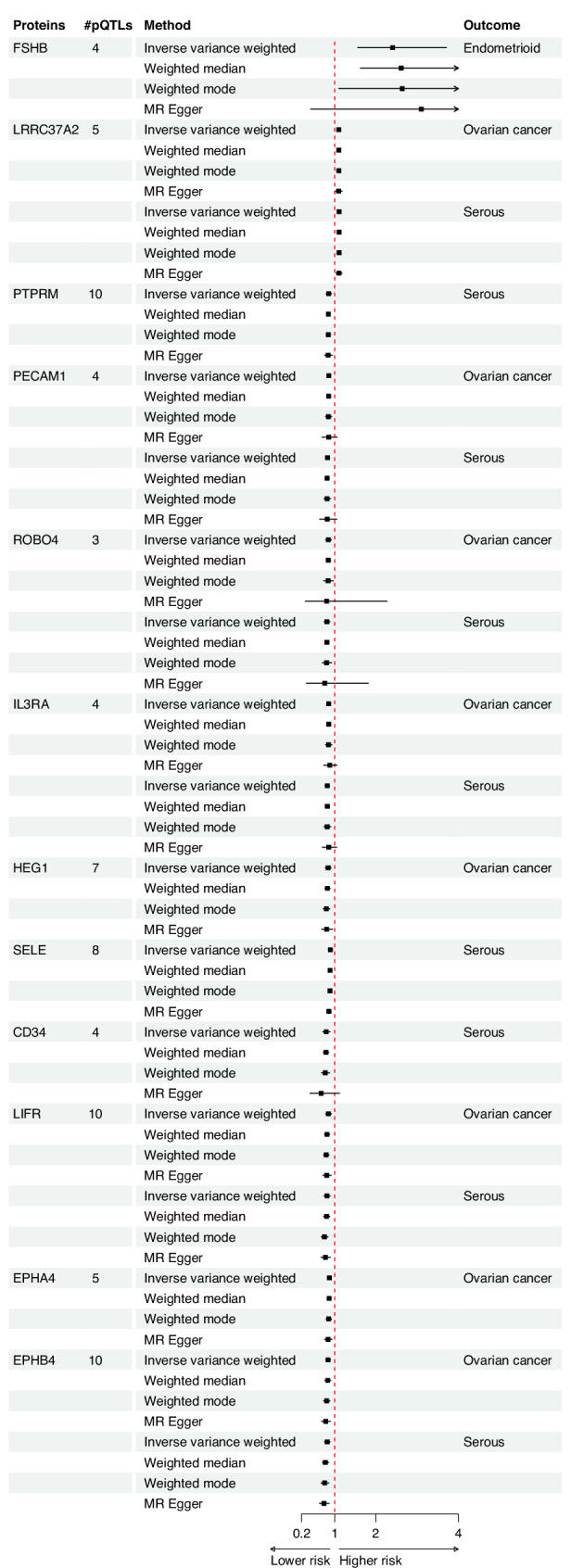
Forest plot showing the association between blood plasma protein and ovarian cancer (or its subtypes), using different Mendelian randomisation methods. #pQTLs stands for number of protein quantitative trait locus used as instruments.

**Fig. 4:**
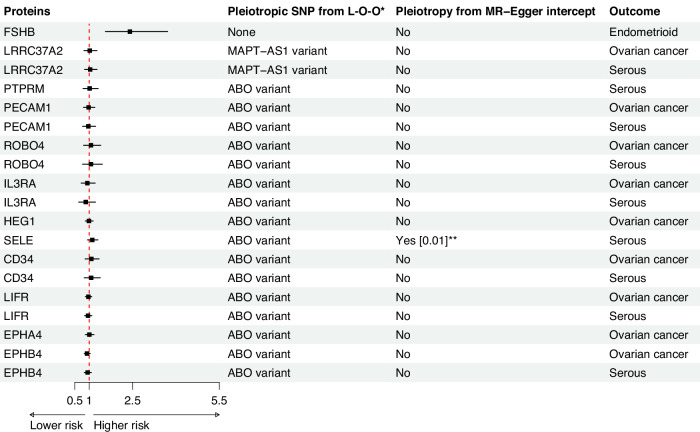
Forest plot showing the Mendelian randomisation estimates after correcting for pleiotropy identified from leave-one-out analysis, with additional finding from MR-Egger intercept. #pQTLs stands for number of protein quantitative trait locus used as instruments. * L-O-O stands for leave-one-out analysis.

We observed robust evidence for a link between follitropin subunit beta, also known as Follicle Stimulating Hormone subunit beta (FSHB), and endometrioid OC (OR per genetically instrumented 1 SD higher 2.41, 95% CI 1.56, 3.71). Evidence was consistent when using weighted median (2.61, 95% CI 1.63, 4.2) and weighted mode (2.64, 95% CI 1.10, 6.33). MR analyses, with similar but less precise estimates using MR-Egger (Fig. 3). No evidence of pleiotropy was found from the MR-Egger intercept test (p_intercept_ = 0.60) or leave-one-out analyses (Fig. 4). Analyses only using the cis-pQTL variant of FSHB (rs11031006), which accounts for 1.2% of the variability in blood FSHB level (r^2^ = 1.2%), supported the main finding (2.57, 95% CI 1.46, 4.52, Supplementary Table 4). While we did not observe strong evidence for colocalization, we found suggestive evidence of shared genetic architecture between FSHB and endometrioid OC, with 65% posterior probability of colocalisation at the *FSHB* locus (Supplementary Fig. 1). Furthermore, leveraging the cis- pQTL variant (rs11031006, *p* = 5.2 × 10^−05^) obtained from an independent Olink-based cohort (KARMA study, Supplementary Table 5), we found a consistent association between FSHB and endometrioid OC (OR 1.64, 95% CI: 1.22, 2.21). However, using the aptamer-based SomaScan measurements, this variant (or a proxy, r^2^ > 0.8) showed no association with plasma FSHB levels in two independent cohorts (deCODE and Fenland; *p* > 0.73). Consequently, MR analyses using weight from these data sources were not performed. In contrast, the instrument (rs11031006) was associated with FSH, the clinically relevant protein complex that includes FSHB, in three SomaScan-based cohorts (INTERVAL, KORA, and Fenland). MR analyses using these FSH measures supported the primary finding (Supplementary Table 5). Beyond endometrioid OC, FSHB also showed suggestive evidence for an association (at a nominal threshold of *p* < 0.05) with other types of epithelial OC, including serous OC, and clear cell OC subtypes (Supplementary Table 2). MR-Steiger test supported the directionality of the association in all cases (Supplementary Table 6).

Leucine-rich repeat-containing protein 37A2 (LRRC37A2) was the top signal for epithelial OC and the serous OC subtype. Higher blood plasma levels of this protein were associated with an increased risk of these cancers. These associations were supported by weighted median, weighted mode, and MR-Egger methods (Fig. 3), with support for directionality of association from MR-Steiger test (Supplementary Table 6). There were five instruments for LRRC37A2, and the MR-Egger intercept test did not identify evidence for pleiotropy (*P* ≥ 0.80 across all OC outcomes). Pleiotropy assessment using the leave-one-out analyses indicated this signal might be driven by a potential pleiotropic variant (rs62057151) located at the *MAPT-*AS1 locus. After excluding this variant, the MR estimates for both epithelial OC and serous OC crossed the null (Fig. 4). In a single pQTL MR analysis, we observed an association specifically with this SNP (rs62057151, in the *MAPT-AS1* locus), a cis-pQTL for LRRC37A2 that explains approximately 30% of the variation in its plasma protein levels (Supplementary Tables 7 and 8).

Platelet endothelial cell adhesion molecule (PECAM1), Roundabout homolog 4 (ROBO4), Interleukin-3 receptor subunit alpha (IL3RA), Leukemia inhibitory factor receptor (LIFR), and Ephrin type-B receptor 4 (EPHB4) were also associated with OC, as well as serous OC (Fig. 3 and Supplementary Table 2). Protein HEG homolog 1 (HEG1) and Ephrin type-A receptor 4 (EPHA4) were linked to epithelial OC only, while Receptor-type tyrosine-protein phosphatase mu (PTPRM), Hematopoietic progenitor cell antigen CD34 (CD34) and E-selectin (SELE) were associated with serous OC. Higher blood plasma levels of these proteins were generally associated with a lower risk of OC and/or serous OC. Estimates from all MR methods were consistent, although confidence intervals from the MR-Egger analyses crossed the null for associations with PECAM1, ROBO4, CD34 and IL3RA (Fig. 3). Further leave-one-out analyses suggested that the observed associations were primarily due to instrumental variants at the *ABO* locus, specifically rs2519093, rs532436, or rs507666 (with LD r^2^ values of ≥0.993), although the MR-Steiger test supported the directionality of the association (Supplementary Table 6). In all cases, exclusion of these variants from *ABO* locus resulted in estimates that crossed the null (Fig. 4). In the single pQTL MR analysis, we observed associations exclusively with the *ABO* variants for each plasma protein, which explained approximately 4.2% to 23% of the variability in plasma protein levels (Supplementary Tables 7 and 8).

**Fig. 5:**
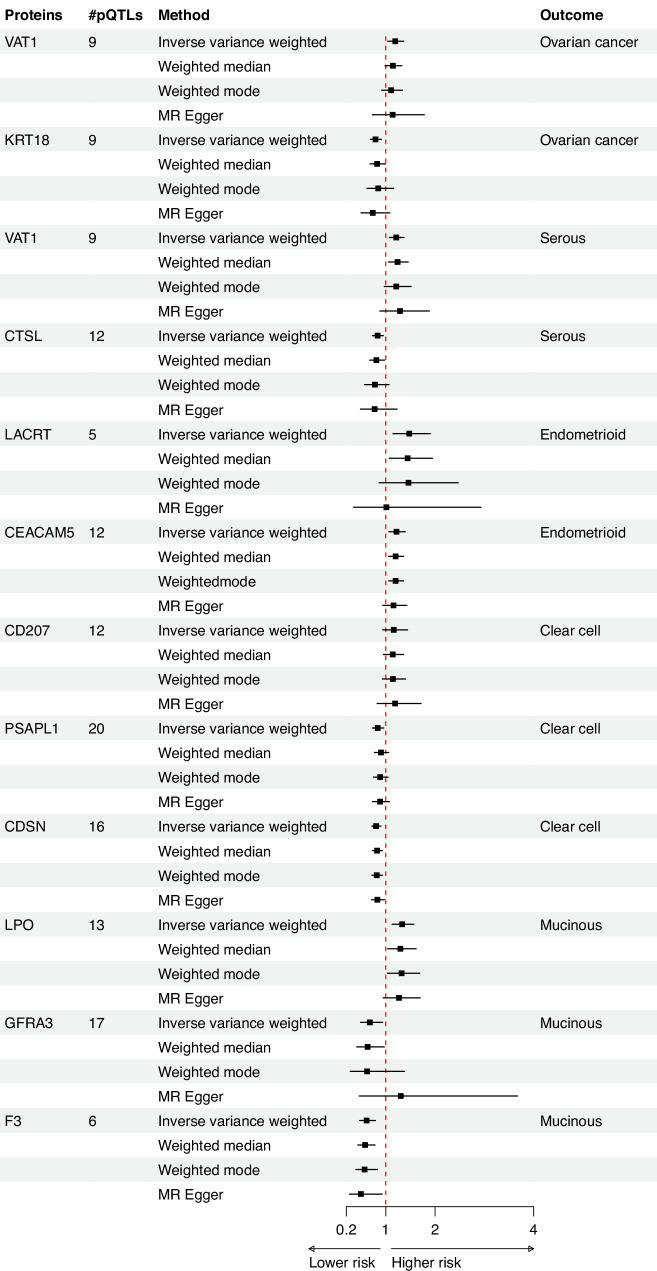
Forest plot showing suggestive evidence of association (*p* < 0.05 and *p* > 7.4 × 10^−5^) between blood plasma proteins and ovarian cancer (or its subtypes), using different Mendelian randomisation methods. #pQTLs stands for number of protein quantitative trait locus used as instruments.

We identified 504 suggestive protein-OC (or subtype) association signals, involving 377 proteins, at the nominal significance threshold (*p* < 0.05 and above the FDR threshold). Among these, 346 associations were from plasma proteins with at least three pQTLs, for which we were able to conduct MR-Egger intercept and leave-one-out analysis. Of these, 12 associations (from 11 proteins) passed both the MR-Egger intercept and leave-one-out pleiotropy tests, suggesting evidence of a causal link (Fig. 5 and Supplementary Fig. 2). MR-Steiger test confirmed the directionality for all the 12 associations (Supplementary Table 6). One of the proteins that did not pass the leave-one-out analyses, was Mucin-16 (MUC16), also known as CA125. This protein, a known marker for OC, was associated with a higher risk of OC and its subtypes, including serous OC, endometrioid OC, and clear cell OC, at nominal significance (Supplementary Table 2). However, after excluding rs62193070 (*GAL3ST2*), a trans-pQTL that explains 1.8% of the variability, the association was lost. Of the 504 suggestive protein-OC associations, 158 were derived from analyses using either a single or two instruments, making it impossible to perform MR-Egger intercept test and leave-one-out analyses. This includes CGA (Glycoprotein hormones alpha chain), a component of Follicular stimulating hormone (FSH), which is linked with a higher risk of OC, as well as with serous and clear cell OC subtypes (Supplementary Table 2).

We conducted a follow-up search for FSHB (and its receptor FSHR), and for the 11 protein signals with suggestive MR evidence, using DrugBank and DGIdb (Drug-Gene Interaction Database) to explore their potential as drug targets. According to these databases, eight of these proteins have been identified as potential drug targets. For example, we found six FSHR agonists commonly used to treat or diagnose infertility (Supplementary Table 9). Interestingly, we identified one FSHR antagonist: suramin, a polyanionic compound with demonstrated antineoplastic properties, that is currently under investigation in clinical trials for cancers including prostate cancer, brain tumour, multiple myeloma, COVID-19, autism spectrum disorder, and acute kidney injury. The effect of this antagonist reflects lower serum levels of FSHB, relevant to our finding for an association between higher plasma FSHB level and higher risk of endometrioid OC. We also identified two approved and one investigational drug targeting Keratin, type I cytoskeletal 18 (KRT18); three approved and five investigational drugs targeting CTSL; and multiple investigational drugs, including labetuzumab (currently in clinical trials) and diagnostic agent Technetium Tc-99m arcitumomab (removed from the market), targeting Carcinoembryonic antigen-related cell adhesion molecule 5 (CEACAM5). Additionally, we identified two approved drugs targeting Corneodesmosin (CDSN), two approved and three investigational drugs targeting Lactoperoxidase (LPO), and one investigational drug targeting GDNF family receptor alpha-3 (GFRA3). F3, a tissue factor, is another target for three approved drugs, including tisotumab vedotin-TFTV, a tissue factor-directed antibody used to treat multiple cancers, including OC. F3 is also targeted by multiple investigational drugs (Supplementary Table 9).

## Discussion

In this study, we conducted a comprehensive proteome-wide MR analysis to identify potential protein markers and druggable targets for epithelial OC and its various subtypes. We found robust evidence of an association between plasma FSHB (and more notably, FSH) levels and endometrioid OC, suggesting that FSHB, more relevantly FSH, may serve as a potential serum marker for early detection. Importantly, we report that the downstream target of this protein, the FSH receptor (FSHR), has been suggested as drug target for multiple chemicals, including approved receptor agonist drugs primarily used for managing female infertility and an investigational receptor antagonist, suramin, for anticancer activity [25]. We also identified eight other proteins associated with epithelial OC, and nine proteins (six of which overlapped with OC) associated with serous OC. However, these associations were attributed to pleiotropic variants primarily from the *ABO* and *MAPT-AS1* loci, regions that were also identified as OC risk loci in OC GWAS.

Our finding for a link between FSHB and endometrioid OC is consistent with the hypothesis suggesting that conditions such as late menopause, ovulation, and infertility therapy, which lead to excess exposure of the ovarian surface epithelium to gonadotropins (FSH and luteinizing hormone (LH)), increase the risk of OC [26, 27]. This is in line our prior machine learning-based study, where oral contraceptive use and higher parity (conditions that lead to lower ovulations) were observed to be protective against OC, while later age at menopause was associated with a higher risk of clear cell and endometrioid OC [28]. The identification of *FSHB* as a shared risk-associated gene for endometriosis and endometrioid OC in cross-trait meta-analyses, alongside evidence of a genetic correlation and causal link between endometriosis and OC subtypes, particularly endometrioid and clear cell OC [29], support the importance of the *FSHB* genes and its encoded protein in the progression of both endometrioid OC and endometriosis. FSH is a glycoprotein composed of two subunits: FSHB which is specific to FSH, and CGA, which is nonspecific and shared with other hormones such as LH, thyroid-stimulating hormone, and human chorionic gonadotropin [27]. Consistent with the effect of FSHB, we also found suggestive evidence for an association between CGA and higher risk of OC, including serous OC, and clear cell OC subtypes. However, as there was only a single instrument available for this protein, we were unable to test the robustness of this association. Through binding to the FSHR on ovarian follicles, FSH regulates sexual development, reproduction, follicular development, and ovulation [27]. FSHR is present in ovarian granulosa cells and has also been found to be expressed in normal ovarian surface epithelial cells as well as in epithelial OC cells [30]. FSH binding to FSHR leads to upregulation of multiple signalling pathways, including the Notch signalling, protein kinase C and sphingosine kinase pathways, which results in the proliferation of OC cells and the progression of the cancer [31, 32]. Furthermore, ascites, a common condition during the late stage of OC, contains cellular aggregates detached from the primary tumour, known as spheroids [31]. These spheroids express *FSHB* mRNA and secrete FSH, further promoting cancer cell proliferation and metastasis through activation of the FSHR in the tumour cell [31]. The majority of approved drugs targeting the FSHR mimic the function of FSH and are commonly used for diagnosing and treating infertility [25]. We identified one FSHR antagonist, suramin, which was originally developed for treating African trypanosomiasis [25]. Suramin’s antineoplastic effects have been tested in clinical trials for a variety of human cancers, including prostate cancer, OC, breast cancer, bladder cancer, brain and central nervous system tumours, multiple myeloma, and plasma cell neoplasm, although it has not yet been approved for treatment of any cancer [25, 33].

In addition to the robust association observed for FSHB, we identified 11 proteins with suggestive evidence of associations with OC or its subtypes, most of which have been identified as druggable targets by approved or investigational drugs. Among these, CTSL is a lysosomal cysteine protease primarily involved in the terminal degradation of intracellular and endocytosed proteins [34]. This protein has been found overexpressed in OC, with its expression level correlated with disease progression and metastasis, while downregulation was shown to inhibit OC cell growth and migration [34]. We found approved drugs (such as fostamatinib [for chronic immune thrompocytopenia], trastuzumab deruxtecan [for breast cancer] and bortezomib [for multiple myeloma]) and investigational drugs (such as Felbinac and) that target CTSL, indicating its potential role in cancer treatment. In our study, however, we found an inverse association between CTSL and serous OC, which requires further investigation.

CEACAM5, originally known as a gastrointestinal oncofetal antigen, is now recognised as being expressed in the majority of carcinomas of the gastrointestinal, respiratory, and genitourinary systems [35]. CEACAM5 is a cell surface glycoprotein that plays a role in cell adhesion and intracellular signalling, and is highly expressed in tumours of epithelial origin including OC, particularly in mucinous and endometrioid tumours [35, 36]. Our study found that higher plasma levels of CEACAM5 are associated with higher risk of endometrioid OC, with a potential link also with a higher risk of mucinous OC, although the confidence interval crossed the null, possibly due to limited power (*n* = 1417 cases). CEACAM5 is being investigated as a potential drug target for various agents due to its role in cancer progression and metastasis [36]. Potential drugs such as labetuzumab, a humanised IgG1 monoclonal antibody, through directly binding with CEACAM5, inhibits tumour growth [37]. Given the higher expression of CEACAM5 in tumour compared to healthy cells, CEACAM5 can facilitate the delivery of cytotoxic agents to tumour cells as “antibody-drug – conjugates” [38], such as the delivery of SN-38 (a topoisomerase I inhibitor) to CEACAM5-expressing tumour cells as SN-38-labetuzumab conjugates (labetuzumab govitecan). Labetezumab and its conjugate have been investigated for the treatment of breast cancer, colorectal cancer, lung cancer and pancreatic cancer and could also be explored as a potential treatment for OC including endometrioid and mucinous OC [39].

F3, also referred to as coagulation factor III, tissue factor, thromboplastin, or CD142, is a transmembrane glycoprotein that plays a crucial role in the activation of the coagulation extrinsic cascade [38]. F3 is overexpressed in tumour cells and is involved in tumour growth and metastasis mainly by promoting inflammation, and angiogenesis by increasing expression of vascular endothelial growth factor (VEGF) [40]. It has been targeted by tissue factor-directed antibodies such as tisotumab vedotin, antibody-drug conjugates comprised of anti-tissue factor human IgG1-kappa antibody conjugated to monomethyl auristatin E, a cytotoxic agent that disrupts the microtubule of the tumour cells [41]. This drug is used to treat recurrent and metastatic cervical cancer and is being investigated in clinical trials for its potential use in treating multiple cancers, including OC, fallopian tube cancer, endometrial cancer and prostate cancer [42]. In our study, we found a lower risk of mucinous OC with higher plasma F3 levels, which warrants further investigation given the unexpected directionality of the association. However, population-based studies have shown that plasma F3 levels are elevated in patients with OC, contributing to the higher risk of venous thromboembolism in these patients [40]. Our study also identified other protein markers, including KRT18, CDSN, LPO, and GFRA3, as druggable targets, and their potential use in OC treatment should be explored in future studies.

The primary strength of our proteome-wide MR analyses lies in the large-scale design, which allowed us to screen known and potential novel drug targets and biomarkers using a hypothesis-free approach. By including both cis-pQTLs and trans-pQTLs, we were able to test for horizontal pleiotropy, and demonstrate that many apparent blood protein signals for OC or subtypes were driven by the *ABO* locus. Our approach allow us to re-evaluate the previously identified 26 proteins associated with invasive epithelial OC in the INTERVAL study, which included 1329 proteins from 3301 healthy participants [8]. However, this earlier study employed only a single instrument for each protein and did not include pleiotropy tests. Of the 26 identified proteins, we tested 16 proteins that were available in our dataset for their association with OC, but found evidence suggesting that the associations in each case were driven by pleiotropy. These associations were mostly driven by the *ABO* locus, suggesting that the observed associations in the prior MR study are likely attributable to pleiotropic relationships rather than causal links. Indeed, the *ABO* is a well-known pleiotropic locus that was associated with over 300 proteins in the latest proteome GWAS [10] and it was also identified as a risk locus in the OC GWAS conducted by the OCAC [9]. Furthermore, blood groups defined by the *ABO* locus differentially associate with the risk of OC, particularly high-grade serous OC, with the variants linked to the “A1” blood group antigen (rs507666) associated with a higher risk, while variants indexing the “O” blood group antigen (rs687289) linked with a lower risk. Although our blood protein panel includes ABO, we did not observe an association between ABO protein abundance and OC. This may reflect an importance of the blood type itself, rather than the abundance of ABO protein, in OC risk. An additional strength of our study was that we accounted for potential protein assay effects by replicating the analysis with data from SomaScan [17].

However, our study also has some limitations. First, despite including a large set of proteomic markers from the largest population cohort available at present, due to the limited number of OC cases and its subtypes among UK Biobank participants with proteomic data, our study was unable to conduct and confirm the predictive and prognostic impact of the identified protein markers in the observational analysis. Second, some of our MR analyses, particularly those involving OC subtypes such as endometrioid, clear cell, and mucinous OC, were likely underpowered due to limited number of cases (*n* < 2810). This limitation may have been further exacerbated in the leave-one-out analyses by the exclusion of key pQTLs that contribute substantially to the heritability of protein expression. Mucin-16 (MUC16), also known as CA125, a well-known OC tumour marker, was initially associated with a higher risk of OC and its subtypes in our study at the nominally significant threshold. However, after excluding rs62193070 (*GAL3ST2*), a trans-pQTL that explains 1.8% of the variability, the association was lost (power dropped from 0.96 to 0.45 for detecting OR = 1.22). Similarly, of the top signals identified at FDR threshold, the lack of association involving CD34, IL3RA, PECAM1, ROBO4, PTPRM, ROBO4 and SELE after excluding the pleiotropic variants might reflect lack of power (Supplementary Table 10). Third, the lack of a robust association with established OC markers, such as CA125 and the latest approved Human Epididymis protein 4 (HE4, also known as WAP four-disulfide core domain protein 2 or WFDC2) [43], could be partly attributed to the use of genetic variants derived from a sex-combined population, as the female-specific summary results were unavailable. Fourth, while our study encompassed 2337 proteins with at least one instrumental variable, it is important to acknowledge that certain relevant plasma proteins may not have been captured. This limitation underscores the need for future research employing broader and more comprehensive proteomic platforms to ensure a more exhaustive representation of the plasma proteome. Fifth, it is also important to note that our proteome-wide analysis focuses on protein abundance measured in plasma rather than tissue-specific proteins, that are often more directly relevant to disease progression and therapeutic targeting. For instance, a previous study identified an expression quantitative trait locus (eQTL) for OC associated with the expression of LRRC37A2 in 47 tissues, including the endometrium and ovary [44]. In our study we observed an association between LRRC37A2 and OC, in cis-pQTL but not trans-pQTL analyses (Supplementary Tables 7 and 8), which is relevant as cis-pQTL are more likely to reflect tissue-specific expression differences in the ovary compared to trans-pQTL which typically capture pleiotropic influences. Further studies incorporating tissue-specific protein data are warranted to provide more comprehensive insights. Sixth, we applied FDR correction to account for multiple testing across 2337 proteins and five outcomes, and also reported nominally significant signals as ‘suggestive associations, with related caution required when interpreting these findings. Seventh, we did not observe strong evidence for colocalisation between blood FSHB level and endometrioid OC association (PPH4 = 0.65), although this represents suggestive support. Eighth, although we replicated the association between FSHB and endometrioid OC with an independent data measured with the Olink platform, which has demonstrated superior protein target specificity compared to SomaScan [45, 46], we were unable to replicate this finding using SomaScan-based FSHB data. Future replication using mass spectrometry-based protein measurement, an orthogonal method to affinity-based platforms, may therefore be warranted. However, for FSH, the clinically relevant and predominant form of the protein complex containing FSHB, we found consistent supporting evidence for an association with endometrioid OC across three independent cohorts. In light of replication and the biological relevance of FSH in the ovarian tissue, we believe this finding is likely to be robust. Nineth, given that our analysis was restricted to a European ancestry population, the findings of this study may not be representative of other population groups.

In conclusion, our study identified association between plasma FSHB, along with 11 other proteins, with OC or its subtypes. These findings suggest that these serum biomarkers could be useful for the early detection of OC. Notably, most of these proteins have already been recognised as drug targets, underscoring the need for further investigation into their roles in OC development and treatment.

### Reporting summary

Further information on research design is available in the Nature Research Reporting Summary linked to this article.

## Supplementary information

Supplementary materials

## Data Availability

This research has been conducted using publically available GWAS data accessed through UKB-PPP (http://ukb-ppp.gwas.eu) and OpenGWAS (https://gwas.mrcieu.ac.uk/datasets/). The codes for conducting this study can be provided upon request.
