# Supplementary material for: Protein markers of ovarian cancer and its subtypes: insights from proteome-wide Mendelian randomisation analysis
Source: Br J Cancer. 2025 Aug 28;133(8):1208–17. doi: 10.1038/s41416-025-03143-w (PMC12532995; doi:10.1038/s41416-025-03143-w)
Supplement: Supplementary file 1 — Supplementary materials [file 41416_2025_3143_MOESM1_ESM.pdf]

Supplementary materials

Protein Markers of Ovarian Cancer and Its Subtypes: Insights from Proteome-wide Mendelian Randomisation Analysis

Anwar Mulugeta[1,2&3*], David Stacey[1&2], Amanda L. Lumsden[1&2], Iqbal Madakkatel[1&2], S.Hong Lee[1,2&4], Johanna Mäenpää[5&6], Martin K. Oehler[7&8], Elina Hyppönen[1&2]

[1]Australian Centre for Precision Health, Unit of Clinical and Health Sciences, University of South Australia, Adelaide, Australia

[2]South Australian Health and Medical Research Institute, Adelaide, Australia

[3]Department of Pharmacology and Clinical Pharmacy, College of Health Sciences, Addis Ababa University, Addis Ababa, Ethiopia

[4]UniSA Allied Health & Human Performance, University of South Australia, Adelaide, SA, Australia

[5]Faculty of Medicine and Medical Technology, Tampere University, Tampere, Finland

[6]Cancer Centre, Tampere University Hospital, Tampere, Finland

[7]Department of Gynaecological Oncology, Royal Adelaide Hospital, Adelaide, SA, Australia

[8]Adelaide Medical School, Robinson Research Institute, University of Adelaide, Adelaide, SA, Australia

Contents

Supplementary Table 1. Genetic instruments for blood plasma proteins used in the MR analysis on ovarian cancer and the subtypes

Supplementary Table 2. Association between blood plasma protein and ovarian cancer or its subtypes, with estimates from MR analysis

Supplementary Table 3. Sensitivity analysis of the association of selected blood plasma protein with high-grade serous and low-grade serous ovarian cancer, with estimates from MR analysis

Supplementary Table 4. Instrument strength, single SNP MR estimates and the respective power (for detecting odd ratio of 1.2 (logOR=0.2) at p=0.05) between FSHB and endometrioid cancer (2810 cases and 40941 controls).

Supplementary Table 5. MR association between FSHB (or FSH) and endometrioid OC using cis-pQTL based summary data from different sources (with proteome data coming either from Olink or SomaScan platforms).

Supplementary Table 6. MR Steiger test for directionality of the association for blood protein - OC outcome associations

Supplementary Table 7. Instrument strength, single SNP MR estimates and the respective power (for detecting odd ratio of 1.2 (logOR=0.2) at p=0.05) between selected serum proteins and ovarian cancer.

Supplementary Table 8. Instrument strength, single SNP MR estimates and the respective power (for detecting odd ratio of 1.2 (logOR=0.2) at p=0.05) between selected serum proteins and high and low grade serous ovarian cancer.

Supplementary Table 9. Drug target identification from DrugBank and DGIdb (Drug-Gene Interaction Database) using protein signals associated with OC or its subtype in the MR analysis [Search on the databases till October 11, 2024]

Supplementary Table 10. Power analysis for detecting an effect estimate (logOR) of 0.10 at alpha=0.05, before (main) and after removing the outlier variants detected in the leave-one-out analysis (LOO).

Supplementary Figure 1. Regional association plot for FSHB locus against blood plasma FSHB level and endometrioid ovarian cancer

Supplementary Figure 2. Volcano plot showing the phenome-wide MR findings, annotation of association included suggestive evidence that passed pleiotropy tests. The Y-axis represents p-values on a -log10 scale for the association between the blood plasma proteome and ovarian cancer (or its subtypes), while the X-axis represents the odds ratio effect estimates.

Supplementary Table 1. Genetic instruments for blood plasma proteins used in the MR analysis on ovarian cancer and the subtypes

S.No	Protein	UniProt	Protein fullname	rsid	Pos_hg37	Chr.	EA	OA	EAF	Beta	SE	P	cis/trans
1	A1BG	P04217	Alpha-1B-glycoprotein	rs1260326	27730940	2	T	C	0.392	0.137	0.007	5.7E-80	trans
1	A1BG	P04217	Alpha-1B-glycoprotein	rs35332062	73012042	7	G	A	0.873	0.125	0.011	3.1E-32	trans
2	AAMDC	Q9H7C9	Mth938 domain-containing protein	rs1354034	56849749	3	C	T	0.605	0.055	0.007	2.7E-15	trans
2	AAMDC	Q9H7C9	Mth938 domain-containing protein	rs2186564	77583266	11	G	A	0.894	1.064	0.012	0.0E+00	cis
3	AARSD1	Q9BTE6	Alanyl-tRNA editing protein Aarsd1	rs1354034	56849749	3	C	T	0.605	0.067	0.007	8.2E-20	trans
3	AARSD1	Q9BTE6	Alanyl-tRNA editing protein Aarsd1	rs323500	41140545	17	T	C	0.290	0.132	0.008	2.7E-61	cis
4	ABHD14B	Q96IU4	Protein ABHD14B	rs1354034	56849749	3	C	T	0.604	0.056	0.008	3.3E-13	trans
4	ABHD14B	Q96IU4	Protein ABHD14B	rs6993770	106581528	8	A	T	0.714	0.059	0.008	8.3E-13	trans
4	ABHD14B	Q96IU4	Protein ABHD14B	rs7080386	65048306	10	A	C	0.412	0.058	0.008	9.3E-15	trans
5	ABL1	P00519	Tyrosine-protein kinase ABL1	rs1354034	56849749	3	C	T	0.605	0.082	0.008	3.5E-27	trans
6	ABO	P16442	Histo-blood group ABO system transferase	rs17855739	5831840	19	T	C	0.041	0.087	0.012	1.1E-12	trans
7	ACAA1	P09110	3-ketoacyl-CoA thiolase, peroxisomal	rs156265	38170810	3	C	G	0.856	0.125	0.011	4.5E-31	cis
7	ACAA1	P09110	3-ketoacyl-CoA thiolase, peroxisomal	rs4835265	146821410	4	A	C	0.156	0.085	0.010	7.8E-16	trans
7	ACAA1	P09110	3-ketoacyl-CoA thiolase, peroxisomal	rs11456266	56090801	18	G	GT	0.244	0.072	0.009	7.5E-16	trans
8	ACADM	P11310	Medium-chain specific acyl-CoA dehydrogenase, mitochondrial	rs61797339	76189946	1	T	G	0.308	0.142	0.008	6.5E-66	cis
9	ACADSB	P45954	Short/branched chain specific acyl-CoA dehydrogenase, mitochondrial	rs1354034	56849749	3	C	T	0.604	0.059	0.008	7.3E-15	trans
9	ACADSB	P45954	Short/branched chain specific acyl-CoA dehydrogenase, mitochondrial	rs11242109	131677047	5	G	T	0.519	0.097	0.008	3.1E-38	trans
9	ACADSB	P45954	Short/branched chain specific acyl-CoA dehydrogenase, mitochondrial	rs342293	106372219	7	C	G	0.541	0.059	0.007	2.6E-15	trans
9	ACADSB	P45954	Short/branched chain specific acyl-CoA dehydrogenase, mitochondrial	rs6993770	106581528	8	A	T	0.713	0.090	0.008	1.2E-27	trans
9	ACADSB	P45954	Short/branched chain specific acyl-CoA dehydrogenase, mitochondrial	rs7896518	65104500	10	G	A	0.424	0.052	0.008	1.5E-11	trans
9	ACADSB	P45954	Short/branched chain specific acyl-CoA dehydrogenase, mitochondrial	rs7913063	124798683	10	T	A	0.085	0.374	0.014	3.5E-167	cis
10	ACAN	P16112	Aggrecan core protein	rs1337526	47965130	1	G	A	0.803	0.061	0.009	3.7E-12	trans
10	ACAN	P16112	Aggrecan core protein	rs828602	98473318	3	A	G	0.414	0.070	0.007	7.3E-23	trans
10	ACAN	P16112	Aggrecan core protein	rs6461354	17914600	7	C	T	0.607	0.050	0.007	1.8E-12	trans
10	ACAN	P16112	Aggrecan core protein	rs12684083	79148591	9	G	A	0.762	0.106	0.008	1.3E-37	trans
10	ACAN	P16112	Aggrecan core protein	rs2519093	136141870	9	T	C	0.184	0.092	0.009	2.7E-24	trans
10	ACAN	P16112	Aggrecan core protein	rs56278466	17875857	10	G	T	0.661	0.064	0.007	5.9E-18	trans
10	ACAN	P16112	Aggrecan core protein	rs11000138	73782022	10	A	T	0.606	0.196	0.007	4.6E-160	trans
10	ACAN	P16112	Aggrecan core protein	rs895734	45684254	11	T	C	0.588	0.090	0.007	7.3E-37	trans
10	ACAN	P16112	Aggrecan core protein	rs59379014	126228000	11	T	C	0.072	0.102	0.014	4.6E-14	trans
10	ACAN	P16112	Aggrecan core protein	rs200201338	81241246	15	T	TGAA	0.023	0.207	0.024	1.2E-18	trans
10	ACAN	P16112	Aggrecan core protein	rs4932196	89253268	15	C	T	0.191	0.134	0.009	8.9E-52	cis
10	ACAN	P16112	Aggrecan core protein	rs186021206	7069412	17	A	G	0.006	0.355	0.048	1.3E-13	trans
10	ACAN	P16112	Aggrecan core protein	rs55639531	394341	18	G	C	0.285	0.075	0.008	6.5E-22	trans
10	ACAN	P16112	Aggrecan core protein	rs17549173	36400231	19	A	G	0.072	0.108	0.014	2.6E-15	trans
11	ACE	P12821	Angiotensin-converting enzyme	rs34434834	126307796	11	A	G	0.033	0.169	0.020	3.0E-17	trans
11	ACE	P12821	Angiotensin-converting enzyme	rs186021206	7069412	17	A	G	0.006	0.400	0.052	1.2E-14	trans
11	ACE	P12821	Angiotensin-converting enzyme	rs4362	61573761	17	T	C	0.518	0.448	0.008	0.0E+00	cis
12	ACE2	Q9BYF1	Angiotensin-converting enzyme 2	rs1047891	211540507	2	A	C	0.313	0.052	0.007	9.5E-13	trans
12	ACE2	Q9BYF1	Angiotensin-converting enzyme 2	rs439651	29903267	6	G	A	0.512	0.053	0.007	5.5E-14	trans
12	ACE2	Q9BYF1	Angiotensin-converting enzyme 2	rs112875651	126506694	8	G	A	0.609	0.078	0.007	1.3E-28	trans
12	ACE2	Q9BYF1	Angiotensin-converting enzyme 2	rs72694393	24874193	14	T	G	0.479	0.060	0.007	2.2E-18	trans
12	ACE2	Q9BYF1	Angiotensin-converting enzyme 2	rs28929474	94844947	14	T	C	0.021	0.219	0.024	2.8E-20	trans
12	ACE2	Q9BYF1	Angiotensin-converting enzyme 2	rs72787359	51431230	16	A	C	0.927	0.096	0.013	3.1E-13	trans
12	ACE2	Q9BYF1	Angiotensin-converting enzyme 2	rs5934255	15664386	X	A	G	0.294	0.149	0.006	5.0E-129	cis
13	ACHE	P22303	Acetylcholinesterase	rs2269615	172415451	1	C	T	0.440	0.070	0.007	3.1E-23	trans
13	ACHE	P22303	Acetylcholinesterase	rs7636227	52566682	3	A	G	0.553	0.051	0.007	4.3E-13	trans
13	ACHE	P22303	Acetylcholinesterase	rs35762167	186654985	3	T	C	0.417	0.107	0.007	3.2E-51	trans
13	ACHE	P22303	Acetylcholinesterase	rs6976053	100512119	7	T	C	0.471	0.313	0.007	0.0E+00	cis
13	ACHE	P22303	Acetylcholinesterase	rs2519093	136141870	9	C	T	0.815	0.173	0.009	3.2E-81	trans
13	ACHE	P22303	Acetylcholinesterase	rs186021206	7069412	17	A	G	0.006	0.496	0.048	3.7E-25	trans
14	ACOT13	Q9NPJ3	Acyl-coenzyme A thioesterase 13	rs1354034	56849749	3	C	T	0.605	0.068	0.008	7.7E-19	trans
14	ACOT13	Q9NPJ3	Acyl-coenzyme A thioesterase 13	rs10058074	131686146	5	G	A	0.525	0.056	0.008	1.7E-13	trans
14	ACOT13	Q9NPJ3	Acyl-coenzyme A thioesterase 13	rs143667676	24698187	6	G	T	0.998	1.055	0.082	1.9E-37	cis
14	ACOT13	Q9NPJ3	Acyl-coenzyme A thioesterase 13	rs6993770	106581528	8	A	T	0.713	0.072	0.008	5.9E-18	trans
15	ACOX1	Q15067	Peroxisomal acyl-coenzyme A oxidase 1	rs10852766	73951864	17	C	T	0.659	0.078	0.008	7.6E-22	cis
16	ACP1	P24666	Low molecular weight phosphotyrosine protein phosphatase	rs11553746	272203	2	C	T	0.645	0.379	0.008	0.0E+00	cis
17	ACP5	P13686	Tartrate-resistant acid phosphatase type 5	rs10813949	33123464	9	G	A	0.690	0.057	0.007	1.5E-14	trans
17	ACP5	P13686	Tartrate-resistant acid phosphatase type 5	rs35382467	128199283	11	G	A	0.902	0.093	0.011	3.3E-16	trans
17	ACP5	P13686	Tartrate-resistant acid phosphatase type 5	rs1958559	66052055	14	T	C	0.602	0.055	0.007	3.1E-15	trans
17	ACP5	P13686	Tartrate-resistant acid phosphatase type 5	rs2073333	94844562	14	T	C	0.253	0.109	0.008	9.1E-44	trans
17	ACP5	P13686	Tartrate-resistant acid phosphatase type 5	rs78755089	79576621	16	T	G	0.084	0.108	0.012	1.7E-18	trans
17	ACP5	P13686	Tartrate-resistant acid phosphatase type 5	rs186021206	7069412	17	A	G	0.006	0.332	0.047	2.0E-12	trans
17	ACP5	P13686	Tartrate-resistant acid phosphatase type 5	rs2305799	11687351	19	C	T	0.877	0.504	0.011	0.0E+00	cis
18	ACP6	Q9NPH0	Lysophosphatidic acid phosphatase type 6	rs77542178	120515032	1	T	C	0.979	0.181	0.027	1.0E-11	trans
19	ACRBP	Q8NEB7	Acrosin-binding protein	rs342298	106373646	7	C	T	0.545	0.053	0.008	2.4E-12	trans
19	ACRBP	Q8NEB7	Acrosin-binding protein	rs6993770	106581528	8	A	T	0.714	0.065	0.008	3.9E-15	trans
19	ACRBP	Q8NEB7	Acrosin-binding protein	rs892090	55539072	19	G	T	0.834	0.069	0.010	9.2E-12	trans
20	ACRV1	P26436	Acrosomal protein SP-10	rs369963387	44646182	17	G	A	0.271	0.042	0.006	1.7E-11	trans
21	ACSL1	P33121	Long-chain-fatty-acid--CoA ligase 1	rs56295533	107152516	14	A	G	0.206	0.096	0.011	1.3E-18	trans
22	ACTA2	P62736	Actin, aortic smooth muscle	rs1324551	90751516	10	C	T	0.501	0.060	0.007	1.1E-18	cis
23	ACVRL1	P37023	Serine/threonine-protein kinase receptor R3	rs75581187	150742542	1	C	T	0.928	0.094	0.014	4.7E-12	trans
23	ACVRL1	P37023	Serine/threonine-protein kinase receptor R3	rs9264623	31237950	6	T	C	0.361	0.057	0.007	7.1E-15	trans
23	ACVRL1	P37023	Serine/threonine-protein kinase receptor R3	rs11244079	136184526	9	A	G	0.050	0.110	0.016	6.6E-12	trans
23	ACVRL1	P37023	Serine/threonine-protein kinase receptor R3	rs2837988	42619544	21	A	C	0.362	0.082	0.007	1.5E-28	trans
24	ACY1	Q03154	Aminoacylase-1	rs1260326	27730940	2	T	C	0.392	0.071	0.007	2.4E-22	trans
24	ACY1	Q03154	Aminoacylase-1	rs121912698	52022837	3	C	T	0.996	1.692	0.054	8.9E-218	cis
24	ACY1	Q03154	Aminoacylase-1	rs3747207	44324855	22	A	G	0.215	0.077	0.009	5.5E-19	trans
25	ACY3	Q96HD9	N-acyl-aromatic-L-amino acid amidohydrolase	rs6894249	131797547	5	A	G	0.613	0.063	0.008	6.4E-17	trans
25	ACY3	Q96HD9	N-acyl-aromatic-L-amino acid amidohydrolase	rs1964528	76390058	8	C	T	0.887	0.091	0.012	6.4E-15	trans
25	ACY3	Q96HD9	N-acyl-aromatic-L-amino acid amidohydrolase	rs112875651	126506694	8	G	A	0.608	0.053	0.008	4.6E-12	trans
25	ACY3	Q96HD9	N-acyl-aromatic-L-amino acid amidohydrolase	rs3758938	67402362	11	G	T	0.312	0.274	0.008	5.4E-248	cis
25	ACY3	Q96HD9	N-acyl-aromatic-L-amino acid amidohydrolase	rs6057570	30988098	20	T	A	0.828	0.073	0.010	8.1E-14	trans
25	ACY3	Q96HD9	N-acyl-aromatic-L-amino acid amidohydrolase	rs738409	44324727	22	G	C	0.217	0.064	0.009	4.5E-13	trans
26	ACYP1	P07311	Acylphosphatase-1	rs1354034	56849749	3	C	T	0.604	0.066	0.007	6.5E-19	trans
26	ACYP1	P07311	Acylphosphatase-1	rs6993770	106581528	8	A	T	0.713	0.058	0.008	6.0E-13	trans
26	ACYP1	P07311	Acylphosphatase-1	rs2393967	65133156	10	C	A	0.314	0.056	0.008	1.6E-12	trans
26	ACYP1	P07311	Acylphosphatase-1	rs75948138	75530041	14	C	G	0.989	1.263	0.036	1.0E-272	cis
27	ADA	P00813	Adenosine deaminase	rs140373387	162868934	2	C	G	0.005	0.441	0.049	3.9E-19	trans
27	ADA	P00813	Adenosine deaminase	rs11555566	43255220	20	C	T	0.063	1.280	0.015	0.0E+00	cis
28	ADA2	Q9NZK5	Adenosine deaminase 2	rs72930772	203619677	2	T	C	0.335	0.079	0.008	7.9E-25	trans
28	ADA2	Q9NZK5	Adenosine deaminase 2	rs148819155	158344981	3	A	G	0.997	0.431	0.064	1.3E-11	trans
28	ADA2	Q9NZK5	Adenosine deaminase 2	rs2903386	106099809	4	C	T	0.371	0.058	0.007	8.4E-15	trans
28	ADA2	Q9NZK5	Adenosine deaminase 2	rs2548999	131831058	5	T	G	0.662	0.051	0.008	1.3E-11	trans
28	ADA2	Q9NZK5	Adenosine deaminase 2	rs535777	32577633	6	C	G	0.143	0.156	0.010	2.8E-50	trans
28	ADA2	Q9NZK5	Adenosine deaminase 2	rs56278466	17875857	10	G	T	0.661	0.076	0.008	1.0E-23	trans
28	ADA2	Q9NZK5	Adenosine deaminase 2	rs12575367	17360590	11	G	A	0.299	0.062	0.008	2.6E-15	trans
28	ADA2	Q9NZK5	Adenosine deaminase 2	rs76428106	28604007	13	C	T	0.013	0.224	0.033	1.4E-11	trans
28	ADA2	Q9NZK5	Adenosine deaminase 2	rs145078947	93652974	14	T	G	0.003	0.554	0.072	1.1E-14	trans
28	ADA2	Q9NZK5	Adenosine deaminase 2	rs186021206	7069412	17	A	G	0.006	0.669	0.049	8.6E-42	trans
28	ADA2	Q9NZK5	Adenosine deaminase 2	rs485186	49207206	19	G	A	0.538	0.050	0.007	4.7E-12	trans
28	ADA2	Q9NZK5	Adenosine deaminase 2	rs737963	17709771	22	C	T	0.612	0.207	0.008	3.7E-164	cis
28	ADA2	Q9NZK5	Adenosine deaminase 2	rs738409	44324727	22	G	C	0.217	0.078	0.009	1.9E-18	trans
29	ADAM12	O43184	Disintegrin and metalloproteinase domain-containing protein 12	rs11244909	127944593	10	C	T	0.609	0.129	0.008	9.4E-62	cis
30	ADAM15	Q13444	Disintegrin and metalloproteinase domain-containing protein 15	rs11589479	155033308	1	A	G	0.163	1.024	0.010	0.0E+00	cis
30	ADAM15	Q13444	Disintegrin and metalloproteinase domain-containing protein 15	rs146142570	50354287	3	A	G	0.002	0.513	0.065	2.0E-15	trans
30	ADAM15	Q13444	Disintegrin and metalloproteinase domain-containing protein 15	rs77468121	31345484	6	A	G	0.044	0.117	0.015	1.9E-15	trans
30	ADAM15	Q13444	Disintegrin and metalloproteinase domain-containing protein 15	rs62571868	113126211	9	T	A	0.232	0.049	0.007	1.1E-11	trans
30	ADAM15	Q13444	Disintegrin and metalloproteinase domain-containing protein 15	rs8176759	136129647	9	A	G	0.061	0.507	0.013	0.0E+00	trans
30	ADAM15	Q13444	Disintegrin and metalloproteinase domain-containing protein 15	rs56278466	17875857	10	G	T	0.661	0.043	0.006	7.7E-12	trans
30	ADAM15	Q13444	Disintegrin and metalloproteinase domain-containing protein 15	rs186021206	7069412	17	A	G	0.006	0.687	0.042	3.5E-61	trans
31	ADAM22	Q9P0K1	Disintegrin and metalloproteinase domain-containing protein 22	rs61747728	179526214	1	T	C	0.038	0.169	0.018	6.9E-22	trans
31	ADAM22	Q9P0K1	Disintegrin and metalloproteinase domain-containing protein 22	rs75166367	162964301	2	G	A	0.940	0.112	0.014	2.3E-15	trans
31	ADAM22	Q9P0K1	Disintegrin and metalloproteinase domain-containing protein 22	rs2279542	87564497	7	C	G	0.495	0.565	0.008	0.0E+00	cis
31	ADAM22	Q9P0K1	Disintegrin and metalloproteinase domain-containing protein 22	rs186021206	7069412	17	A	G	0.006	0.332	0.046	7.6E-13	trans
31	ADAM22	Q9P0K1	Disintegrin and metalloproteinase domain-containing protein 22	rs2659005	79218714	17	T	C	0.441	0.051	0.007	9.8E-14	trans
32	ADAM23	O75077	Disintegrin and metalloproteinase domain-containing protein 23	rs61747728	179526214	1	T	C	0.038	0.121	0.016	1.1E-13	trans
32	ADAM23	O75077	Disintegrin and metalloproteinase domain-containing protein 23	rs72933203	207311349	2	A	T	0.103	0.878	0.012	0.0E+00	cis
32	ADAM23	O75077	Disintegrin and metalloproteinase domain-containing protein 23	rs1294438	6752059	6	T	C	0.359	0.056	0.007	2.6E-17	trans
32	ADAM23	O75077	Disintegrin and metalloproteinase domain-containing protein 23	rs8176741	136131461	9	A	G	0.061	0.222	0.013	8.1E-64	trans
32	ADAM23	O75077	Disintegrin and metalloproteinase domain-containing protein 23	rs186021206	7069412	17	A	G	0.006	0.482	0.043	5.9E-29	trans
33	ADAM8	P78325	Disintegrin and metalloproteinase domain-containing protein 8	rs11806326	25899165	1	G	T	0.478	0.070	0.007	6.8E-22	trans
33	ADAM8	P78325	Disintegrin and metalloproteinase domain-containing protein 8	rs2596530	31387373	6	G	A	0.506	0.059	0.007	1.7E-16	trans
33	ADAM8	P78325	Disintegrin and metalloproteinase domain-containing protein 8	rs2995310	135082810	10	C	T	0.106	0.709	0.012	0.0E+00	cis
33	ADAM8	P78325	Disintegrin and metalloproteinase domain-containing protein 8	rs4055121	126232337	11	C	T	0.865	0.086	0.011	2.3E-16	trans
33	ADAM8	P78325	Disintegrin and metalloproteinase domain-containing protein 8	rs76428106	28604007	13	C	T	0.013	0.255	0.033	8.3E-15	trans
33	ADAM8	P78325	Disintegrin and metalloproteinase domain-containing protein 8	rs186021206	7069412	17	A	G	0.006	0.567	0.049	5.5E-31	trans
33	ADAM8	P78325	Disintegrin and metalloproteinase domain-containing protein 8	rs2227319	38170845	17	G	A	0.629	0.076	0.007	4.6E-25	trans
34	ADAM9	Q13443	Disintegrin and metalloproteinase domain-containing protein 9	rs9267123	31427395	6	C	G	0.158	0.137	0.010	4.6E-41	trans
34	ADAM9	Q13443	Disintegrin and metalloproteinase domain-containing protein 9	rs61753672	38869207	8	A	G	0.005	0.923	0.053	2.7E-67	cis
34	ADAM9	Q13443	Disintegrin and metalloproteinase domain-containing protein 9	rs507666	136149399	9	G	A	0.816	0.080	0.010	1.3E-16	trans
34	ADAM9	Q13443	Disintegrin and metalloproteinase domain-containing protein 9	rs34307716	20199378	10	A	G	0.276	0.132	0.008	4.2E-56	trans
34	ADAM9	Q13443	Disintegrin and metalloproteinase domain-containing protein 9	rs877081	94843083	14	G	A	0.862	0.083	0.011	1.4E-14	trans
34	ADAM9	Q13443	Disintegrin and metalloproteinase domain-containing protein 9	rs186021206	7069412	17	A	G	0.006	0.545	0.051	8.4E-27	trans
35	ADAMTS13	Q76LX8	A disintegrin and metalloproteinase with thrombospondin motifs 13	rs4282054	52566065	3	C	T	0.559	0.049	0.007	1.2E-11	trans
35	ADAMTS13	Q76LX8	A disintegrin and metalloproteinase with thrombospondin motifs 13	rs5023357	124770793	4	G	T	0.108	0.103	0.011	1.8E-19	trans
35	ADAMTS13	Q76LX8	A disintegrin and metalloproteinase with thrombospondin motifs 13	rs2910686	96252589	5	C	T	0.450	0.075	0.007	1.7E-25	trans
35	ADAMTS13	Q76LX8	A disintegrin and metalloproteinase with thrombospondin motifs 13	rs9266217	31325092	6	G	A	0.608	0.052	0.008	6.4E-12	trans
35	ADAMTS13	Q76LX8	A disintegrin and metalloproteinase with thrombospondin motifs 13	rs143073120	147665936	6	C	CACTT	0.438	0.049	0.007	1.1E-11	trans
35	ADAMTS13	Q76LX8	A disintegrin and metalloproteinase with thrombospondin motifs 13	rs10253113	130009311	7	G	A	0.307	0.066	0.008	1.4E-17	trans

35	ADAMTS13	Q76LX8	A disintegrin and metalloproteinase with thrombospondin motifs 13	rs9987289	9183358	8	A	G	0.092	0.096	0.012	6.4E-15	trans
35	ADAMTS13	Q76LX8	A disintegrin and metalloproteinase with thrombospondin motifs 13	rs74715985	136304114	9	A	G	0.983	1.459	0.029	0.0E+00	cis
35	ADAMTS13	Q76LX8	A disintegrin and metalloproteinase with thrombospondin motifs 13	rs738408	44324730	22	T	C	0.217	0.064	0.009	1.3E-13	trans
36	ADAMTS15	Q8TE58	A disintegrin and metalloproteinase with thrombospondin motifs 15	rs5030062	186454180	3	A	C	0.627	0.067	0.008	1.3E-17	trans
36	ADAMTS15	Q8TE58	A disintegrin and metalloproteinase with thrombospondin motifs 15	rs4861708	187157233	4	G	A	0.488	0.124	0.008	2.6E-60	trans
36	ADAMTS15	Q8TE58	A disintegrin and metalloproteinase with thrombospondin motifs 15	rs2545801	176841339	5	T	C	0.255	0.097	0.009	2.8E-29	trans
36	ADAMTS15	Q8TE58	A disintegrin and metalloproteinase with thrombospondin motifs 15	rs73044885	130344063	11	A	G	0.055	0.193	0.017	9.4E-29	cis
36	ADAMTS15	Q8TE58	A disintegrin and metalloproteinase with thrombospondin motifs 15	rs186021206	7069412	17	A	G	0.006	0.522	0.052	1.6E-23	trans
37	ADAMTS16	Q8TE57	A disintegrin and metalloproteinase with thrombospondin motifs 16	rs40468	4982814	5	A	G	0.662	0.123	0.007	5.3E-61	cis
38	ADAMTS4	O75173	A disintegrin and metalloproteinase with thrombospondin motifs 4	rs4656993	161176137	1	A	G	0.414	0.118	0.008	8.4E-54	cis
38	ADAMTS4	O75173	A disintegrin and metalloproteinase with thrombospondin motifs 4	rs61658003	55544203	19	A	C	0.842	0.070	0.010	1.6E-11	trans
39	ADAMTS8	Q9UP79	A disintegrin and metalloproteinase with thrombospondin motifs 8	rs7529464	230412836	1	C	G	0.249	0.065	0.008	4.4E-16	trans
39	ADAMTS8	Q9UP79	A disintegrin and metalloproteinase with thrombospondin motifs 8	rs13107325	103188709	4	C	T	0.925	0.144	0.013	3.2E-28	trans
39	ADAMTS8	Q9UP79	A disintegrin and metalloproteinase with thrombospondin motifs 8	rs2131535	130281488	11	A	G	0.731	0.493	0.008	0.0E+00	cis
39	ADAMTS8	Q9UP79	A disintegrin and metalloproteinase with thrombospondin motifs 8	rs7209484	46143042	17	C	T	0.242	0.060	0.008	9.0E-14	trans
40	ADAMTSL2	Q86TH1	ADAMTS-like protein 2	rs61830291	221001142	1	C	A	0.097	0.141	0.013	2.9E-29	trans
40	ADAMTSL2	Q86TH1	ADAMTS-like protein 2	rs34466290	35399393	7	T	TA	0.331	0.061	0.008	1.8E-14	trans
40	ADAMTSL2	Q86TH1	ADAMTS-like protein 2	rs9987289	9183358	8	A	G	0.092	0.090	0.013	2.0E-12	trans
40	ADAMTSL2	Q86TH1	ADAMTS-like protein 2	rs1008250	136397834	9	G	A	0.282	0.106	0.009	1.7E-35	cis
40	ADAMTSL2	Q86TH1	ADAMTS-like protein 2	rs168960	69225124	14	A	G	0.080	0.104	0.013	1.6E-14	trans
40	ADAMTSL2	Q86TH1	ADAMTS-like protein 2	rs28929474	94844947	14	T	C	0.021	0.242	0.026	9.5E-21	trans
40	ADAMTSL2	Q86TH1	ADAMTS-like protein 2	rs1801689	64210580	17	C	A	0.030	0.148	0.022	8.2E-12	trans
40	ADAMTSL2	Q86TH1	ADAMTS-like protein 2	rs77542162	67081278	17	G	A	0.023	0.167	0.025	1.6E-11	trans
40	ADAMTSL2	Q86TH1	ADAMTS-like protein 2	rs73045269	41825191	19	T	C	0.171	0.096	0.010	2.2E-22	trans
41	ADAMTSL4	Q6UY14	ADAMTS-like protein 4	rs6746339	163087425	2	C	T	0.989	0.309	0.036	9.6E-18	trans
41	ADAMTSL4	Q6UY14	ADAMTS-like protein 4	rs13107325	103188709	4	C	T	0.925	0.105	0.014	6.2E-14	trans
41	ADAMTSL4	Q6UY14	ADAMTS-like protein 4	rs2954017	126476873	8	T	C	0.481	0.064	0.007	5.5E-18	trans
42	ADAMTSL5	Q6ZMM2	ADAMTS-like protein 5	rs791461	1507535	19	C	T	0.532	0.406	0.008	0.0E+00	cis
43	ADD1	P35611	Alpha-adducin	rs1354034	56849749	3	C	T	0.604	0.074	0.007	1.2E-23	trans
43	ADD1	P35611	Alpha-adducin	rs1263339	2917974	4	G	C	0.814	0.177	0.009	7.0E-80	cis
43	ADD1	P35611	Alpha-adducin	rs2501577	111846687	10	A	G	0.831	0.135	0.010	7.4E-44	trans
44	ADGRB3	O60242	Adhesion G protein-coupled receptor B3	rs3798969	69671585	6	A	G	0.377	0.941	0.008	0.0E+00	cis
44	ADGRB3	O60242	Adhesion G protein-coupled receptor B3	rs200489612	7106378	17	A	G	0.005	0.294	0.038	8.3E-15	trans
44	ADGRB3	O60242	Adhesion G protein-coupled receptor B3	rs573431210	67276383	17	G	T	0.027	0.117	0.016	8.6E-14	trans
45	ADGRD1	Q6QNK2	Adhesion G-protein coupled receptor D1	rs1260326	27730940	2	T	C	0.392	0.064	0.006	3.6E-23	trans
45	ADGRD1	Q6QNK2	Adhesion G-protein coupled receptor D1	rs6706968	121310269	2	A	C	0.419	0.051	0.006	1.5E-15	trans
45	ADGRD1	Q6QNK2	Adhesion G-protein coupled receptor D1	rs687339	135932359	3	T	C	0.771	0.086	0.008	4.1E-30	trans
45	ADGRD1	Q6QNK2	Adhesion G-protein coupled receptor D1	rs35332062	73012042	7	G	A	0.873	0.085	0.009	2.4E-19	trans
45	ADGRD1	Q6QNK2	Adhesion G-protein coupled receptor D1	rs28601761	126500031	8	C	G	0.581	0.057	0.006	1.9E-18	trans
45	ADGRD1	Q6QNK2	Adhesion G-protein coupled receptor D1	rs1870138	82269611	10	G	A	0.796	0.070	0.008	2.2E-19	trans
45	ADGRD1	Q6QNK2	Adhesion G-protein coupled receptor D1	rs174533	61549025	11	A	G	0.347	0.069	0.007	2.1E-25	trans
45	ADGRD1	Q6QNK2	Adhesion G-protein coupled receptor D1	rs2239222	73011885	14	G	A	0.348	0.047	0.007	1.5E-12	trans
45	ADGRD1	Q6QNK2	Adhesion G-protein coupled receptor D1	rs186021206	7069412	17	A	G	0.006	0.430	0.043	4.0E-23	trans
45	ADGRD1	Q6QNK2	Adhesion G-protein coupled receptor D1	rs1801689	64210580	17	C	A	0.030	0.144	0.018	6.8E-15	trans
45	ADGRD1	Q6QNK2	Adhesion G-protein coupled receptor D1	rs1800961	43042364	20	C	T	0.968	0.165	0.018	2.4E-20	trans
46	ADGRE1	Q14246	Adhesion G protein-coupled receptor E1	rs12616639	62529925	2	G	A	0.611	0.088	0.007	3.5E-32	trans
46	ADGRE1	Q14246	Adhesion G protein-coupled receptor E1	rs1257169	134963862	2	C	A	0.501	0.113	0.007	1.5E-54	trans
46	ADGRE1	Q14246	Adhesion G protein-coupled receptor E1	rs6440016	98374622	3	C	A	0.851	0.083	0.010	3.9E-16	trans
46	ADGRE1	Q14246	Adhesion G protein-coupled receptor E1	rs35005076	38789072	4	GC	G	0.198	0.066	0.009	3.7E-13	trans
46	ADGRE1	Q14246	Adhesion G protein-coupled receptor E1	rs115216147	70665526	5	A	T	0.067	0.118	0.015	1.7E-15	trans
46	ADGRE1	Q14246	Adhesion G protein-coupled receptor E1	rs4934470	91067438	10	T	C	0.274	0.059	0.008	4.0E-13	trans
46	ADGRE1	Q14246	Adhesion G protein-coupled receptor E1	rs4601794	126245145	11	A	G	0.941	0.184	0.015	4.4E-33	trans
46	ADGRE1	Q14246	Adhesion G protein-coupled receptor E1	rs186021206	7069412	17	A	G	0.006	1.096	0.050	4.5E-108	trans
46	ADGRE1	Q14246	Adhesion G protein-coupled receptor E1	rs2305482	38140927	17	A	C	0.461	0.050	0.007	4.4E-12	trans
46	ADGRE1	Q14246	Adhesion G protein-coupled receptor E1	rs1292061	57911230	17	G	A	0.554	0.076	0.007	7.9E-26	trans
46	ADGRE1	Q14246	Adhesion G protein-coupled receptor E1	rs2725405	79220224	17	C	G	0.441	0.054	0.007	1.5E-13	trans
46	ADGRE1	Q14246	Adhesion G protein-coupled receptor E1	rs59055395	6873522	19	C	T	0.113	0.444	0.012	0.0E+00	cis
47	ADGRE2	Q9UHX3	Adhesion G protein-coupled receptor E2	rs1260326	27730940	2	C	T	0.609	0.054	0.007	9.2E-14	trans
47	ADGRE2	Q9UHX3	Adhesion G protein-coupled receptor E2	rs78776915	32469380	6	T	C	0.415	0.057	0.008	1.1E-13	trans
47	ADGRE2	Q9UHX3	Adhesion G protein-coupled receptor E2	rs56278466	17875857	10	G	T	0.661	0.119	0.007	1.2E-57	trans
47	ADGRE2	Q9UHX3	Adhesion G protein-coupled receptor E2	rs950802	60152584	11	G	A	0.694	0.060	0.008	6.5E-15	trans
47	ADGRE2	Q9UHX3	Adhesion G protein-coupled receptor E2	rs186021206	7069412	17	A	G	0.006	1.090	0.048	4.4E-112	trans
47	ADGRE2	Q9UHX3	Adhesion G protein-coupled receptor E2	rs7251607	14506991	19	C	A	0.753	0.287	0.008	1.2E-254	cis
48	ADGRE5	P48960	Adhesion G protein-coupled receptor E5	rs1260326	27730940	2	C	T	0.608	0.056	0.007	2.8E-14	trans
48	ADGRE5	P48960	Adhesion G protein-coupled receptor E5	rs34458031	98752035	3	A	G	0.086	0.087	0.013	8.3E-12	trans
48	ADGRE5	P48960	Adhesion G protein-coupled receptor E5	rs10093	32609173	6	G	C	0.377	0.065	0.008	1.0E-17	trans
48	ADGRE5	P48960	Adhesion G protein-coupled receptor E5	rs56278466	17875857	10	G	T	0.662	0.138	0.008	6.6E-75	trans
48	ADGRE5	P48960	Adhesion G protein-coupled receptor E5	rs950802	60152584	11	G	A	0.693	0.069	0.008	7.0E-19	trans
48	ADGRE5	P48960	Adhesion G protein-coupled receptor E5	rs186021206	7069412	17	A	G	0.006	1.178	0.049	1.9E-126	trans
48	ADGRE5	P48960	Adhesion G protein-coupled receptor E5	rs139113505	14499616	19	C	T	0.983	0.715	0.028	2.0E-143	cis
48	ADGRE5	P48960	Adhesion G protein-coupled receptor E5	rs6065904	44534651	20	A	G	0.215	0.065	0.009	7.6E-14	trans
49	ADGRF5	Q8IZF2	Adhesion G protein-coupled receptor F5	rs76183323	236230317	1	G	A	0.989	0.844	0.038	6.7E-108	trans
49	ADGRF5	Q8IZF2	Adhesion G protein-coupled receptor F5	rs13412535	224874874	2	G	A	0.769	0.060	0.009	4.4E-12	trans
49	ADGRF5	Q8IZF2	Adhesion G protein-coupled receptor F5	rs11719450	58456811	3	T	C	0.370	0.074	0.008	2.0E-22	trans
49	ADGRF5	Q8IZF2	Adhesion G protein-coupled receptor F5	rs34716199	72193315	5	AT	A	0.536	0.052	0.007	1.3E-12	trans
49	ADGRF5	Q8IZF2	Adhesion G protein-coupled receptor F5	rs4098923	31193756	6	A	G	0.552	0.079	0.008	8.4E-25	trans
49	ADGRF5	Q8IZF2	Adhesion G protein-coupled receptor F5	rs342299	106373718	7	C	T	0.545	0.063	0.007	6.2E-18	trans
49	ADGRF5	Q8IZF2	Adhesion G protein-coupled receptor F5	rs927826	20220846	10	T	G	0.705	0.187	0.008	5.7E-121	trans
49	ADGRF5	Q8IZF2	Adhesion G protein-coupled receptor F5	rs7896518	65104500	10	G	A	0.424	0.089	0.007	1.3E-32	trans
49	ADGRF5	Q8IZF2	Adhesion G protein-coupled receptor F5	rs12445050	81870969	16	T	C	0.138	0.088	0.010	4.4E-17	trans
49	ADGRF5	Q8IZF2	Adhesion G protein-coupled receptor F5	rs892090	55539072	19	G	T	0.834	0.123	0.010	2.6E-36	trans
49	ADGRF5	Q8IZF2	Adhesion G protein-coupled receptor F5	rs1033549	19271940	20	T	G	0.378	0.053	0.007	2.1E-12	trans
49	ADGRF5	Q8IZF2	Adhesion G protein-coupled receptor F5	rs28391150	69673464	X	T	C	0.195	0.052	0.008	5.7E-12	trans
50	ADGRG1	Q9Y653	Adhesion G-protein coupled receptor G1	rs1047891	211540507	2	A	C	0.312	0.063	0.008	4.7E-16	trans
50	ADGRG1	Q9Y653	Adhesion G-protein coupled receptor G1	rs10935473	98416900	3	G	T	0.560	0.079	0.007	9.0E-28	trans
50	ADGRG1	Q9Y653	Adhesion G-protein coupled receptor G1	rs2519093	136141870	9	C	T	0.816	0.113	0.009	6.5E-34	trans
50	ADGRG1	Q9Y653	Adhesion G-protein coupled receptor G1	rs56278466	17875857	10	G	T	0.661	0.133	0.008	3.3E-69	trans
50	ADGRG1	Q9Y653	Adhesion G-protein coupled receptor G1	rs733464	57682275	16	T	C	0.407	0.071	0.007	4.5E-22	cis
50	ADGRG1	Q9Y653	Adhesion G-protein coupled receptor G1	rs186021206	7069412	17	A	G	0.006	0.473	0.050	1.7E-21	trans
50	ADGRG1	Q9Y653	Adhesion G-protein coupled receptor G1	rs738409	44324727	22	G	C	0.217	0.060	0.009	3.9E-12	trans
51	ADGRG2	Q8IZP9	Adhesion G-protein coupled receptor G2	rs13107325	103188709	4	T	C	0.075	0.098	0.013	7.9E-14	trans
51	ADGRG2	Q8IZP9	Adhesion G-protein coupled receptor G2	rs115912456	82815158	5	A	G	0.959	0.148	0.017	2.0E-17	trans
51	ADGRG2	Q8IZP9	Adhesion G-protein coupled receptor G2	rs8176719	136132908	9	TC	T	0.340	0.051	0.007	1.4E-12	trans
51	ADGRG2	Q8IZP9	Adhesion G-protein coupled receptor G2	rs74612335	126238633	11	T	C	0.867	0.090	0.010	9.7E-19	trans
51	ADGRG2	Q8IZP9	Adhesion G-protein coupled receptor G2	rs186021206	7069412	17	A	G	0.006	0.520	0.047	2.7E-28	trans
52	ADGRV1	Q8WXG9	Adhesion G-protein coupled receptor V1	rs1031101	54533360	10	A	G	0.858	0.165	0.011	4.0E-50	trans
53	ADH1B	P00325	All-trans-retinol dehydrogenase	rs41275699	100239268	4	A	G	0.998	1.293	0.090	3.2E-47	cis
53	ADH1B	P00325	All-trans-retinol dehydrogenase	rs4979373	117144504	9	C	T	0.510	0.071	0.007	1.0E-21	trans
53	ADH1B	P00325	All-trans-retinol dehydrogenase	rs10883451	101924418	10	T	C	0.498	0.053	0.007	6.6E-13	trans
53	ADH1B	P00325	All-trans-retinol dehydrogenase	rs10405357	54759666	19	T	C	0.565	0.069	0.007	4.8E-20	trans
53	ADH1B	P00325	All-trans-retinol dehydrogenase	rs3747207	44324855	22	A	G	0.215	0.098	0.009	5.1E-28	trans
54	ADH4	P08319	All-trans-retinol dehydrogenase [NAD(+)] ADH4	rs1800759	100065509	4	G	T	0.613	0.130	0.007	5.4E-68	cis
54	ADH4	P08319	All-trans-retinol dehydrogenase [NAD(+)] ADH4	rs112875651	126506694	8	G	A	0.609	0.056	0.008	1.2E-13	trans
54	ADH4	P08319	All-trans-retinol dehydrogenase [NAD(+)] ADH4	rs10733608	117148430	9	G	T	0.511	0.063	0.007	6.1E-18	trans
54	ADH4	P08319	All-trans-retinol dehydrogenase [NAD(+)] ADH4	rs10883451	101924418	10	T	C	0.498	0.051	0.007	2.5E-12	trans
54	ADH4	P08319	All-trans-retinol dehydrogenase [NAD(+)] ADH4	rs3747207	44324855	22	A	G	0.216	0.102	0.009	3.1E-31	trans
55	ADIPOQ	Q15848	Adiponectin	rs72657272	31407314	1	T	C	0.153	0.063	0.009	1.8E-12	trans
55	ADIPOQ	Q15848	Adiponectin	rs66782572	52567617	3	G	A	0.539	0.059	0.007	1.7E-19	trans
55	ADIPOQ	Q15848	Adiponectin	rs143257534	186551888	3	C	T	0.974	0.615	0.020	2.1E-199	cis
55	ADIPOQ	Q15848	Adiponectin	rs7859344	131848382	9	T	A	0.285	0.048	0.007	6.1E-12	trans
55	ADIPOQ	Q15848	Adiponectin	rs2925979	81534790	16	C	T	0.698	0.092	0.007	5.8E-40	trans
55	ADIPOQ	Q15848	Adiponectin	rs10422861	33894846	19	T	C	0.667	0.065	0.007	5.5E-22	trans
56	AFAP1	Q8N556	Actin filament-associated protein 1	rs41264705	7780582	4	T	C	0.298	1.083	0.008	0.0E+00	cis
56	AFAP1	Q8N556	Actin filament-associated protein 1	rs3134935	32210355	6	C	T	0.280	0.046	0.007	5.4E-12	trans
56	AFAP1	Q8N556	Actin filament-associated protein 1	rs36051450	123344959	11	T	C	0.804	0.048	0.007	7.6E-12	trans
57	AFM	P43652	Afamin	rs200293726	79754440	16	A	T	0.694	0.070	0.008	6.9E-18	trans
58	AFP	P02771	Alpha-fetoprotein	rs12059956	171063262	1	G	A	0.578	0.047	0.007	1.3E-11	trans
58	AFP	P02771	Alpha-fetoprotein	rs4149909	242023898	1	A	G	0.968	0.144	0.020	2.7E-13	trans
58	AFP	P02771	Alpha-fetoprotein	rs11900031	17930874	2	A	G	0.895	0.414	0.012	6.4E-282	trans
58	AFP	P02771	Alpha-fetoprotein	rs62280667	101084604	3	T	C	0.659	0.129	0.007	2.2E-69	trans
58	AFP	P02771	Alpha-fetoprotein	rs59950280	3452345	4	A	G	0.330	0.092	0.008	4.5E-34	trans
58	AFP	P02771	Alpha-fetoprotein	rs12506899	74319283	4	T	G	0.603	0.186	0.007	4.8E-150	cis
58	AFP	P02771	Alpha-fetoprotein	rs2045028	27922723	8	A	G	0.519	0.047	0.007	1.5E-11	trans
58	AFP	P02771	Alpha-fetoprotein	rs62576492	135218708	9	G	A	0.113	0.134	0.011	8.7E-33	trans
58	AFP	P02771	Alpha-fetoprotein	rs35751397	64914016	10	T	TA	0.531	0.049	0.007	8.7E-13	trans
58	AFP	P02771	Alpha-fetoprotein	rs10883567	102755779	10	T	C	0.509	0.060	0.007	3.0E-18	trans
58	AFP	P02771	Alpha-fetoprotein	rs560191	43767774	15	C	G	0.299	0.122	0.008	5.2E-59	trans
58	AFP	P02771	Alpha-fetoprotein	rs17689024	79739978	16	G	C	0.686	0.053	0.007	1.3E-12	trans
58	AFP	P02771	Alpha-fetoprotein	rs11642010	89845111	16	T	C	0.911	0.084	0.012	5.0E-12	trans
58	AFP	P02771	Alpha-fetoprotein	rs4794048	45763073	17	C	A	0.481	0.071	0.007	1.1E-24	trans
58	AFP	P02771	Alpha-fetoprotein	rs34349423	47860063	17	A	AC	0.375	0.058	0.007	2.3E-15	trans
58	AFP	P02771	Alpha-fetoprotein	rs1801689	64210580	17	A	C	0.970	0.170	0.020	4.1E-17	trans
58	AFP	P02771	Alpha-fetoprotein	rs35103294	73810937	17	T	C	0.631	0.050	0.007	4.1E-12	trans
58	AFP	P02771	Alpha-fetoprotein	rs2548458	49209325	19	C	T	0.461	0.049	0.007	2.3E-12	trans
58	AFP	P02771	Alpha-fetoprotein	rs6116934	5731803	20	C	T	0.215	0.095	0.008	1.2E-29	trans
58	AFP	P02771	Alpha-fetoprotein	rs113844904	30836608	20	C	T	0.843	0.069	0.009	2.6E-13	trans
58	AFP	P02771	Alpha-fetoprotein	rs6007594	45728370	22	A	G	0.257	0.131	0.008	4.9E-62	trans
59	AGER	Q15109	Advanced glycosylation end product-specific receptor	rs66906568	10722299	1	T	C	0.701	0.078	0.008	6.7E-22	trans
59	AGER	Q15109	Advanced glycosylation end product-specific receptor	rs10753239	31362224	1	T	C	0.540	0.055	0.007	2.7E-13	trans
59	AGER	Q15109	Advanced glycosylation end product-specific receptor	rs6664840	175128726	1	A	G	0.529	0.077	0.007	8.1E-26	trans
59	AGER	Q15109	Advanced glycosylation end product-specific receptor	rs2137715	90010460	4	C	A	0.387	0.065	0.007	4.3E-18	trans
59	AGER	Q15109	Advanced glycosylation end product-specific receptor	rs204993	32155581	6	A	G	0.680	0.170	0.008	2.5E-103	cis
59	AGER	Q15109	Advanced glycosylation end product-specific receptor	rs7823498	32403573	8	T	C	0.780	0.073	0.009	9.1E-17	trans

59	AGER	Q15109	Advanced glycosylation end product-specific receptor	rs11243421	134426038	9	T	C	0.438	0.131	0.007	1.4E-70	trans
59	AGER	Q15109	Advanced glycosylation end product-specific receptor	rs2487928	30323892	10	G	A	0.552	0.056	0.007	2.9E-14	trans
59	AGER	Q15109	Advanced glycosylation end product-specific receptor	rs55703124	109922674	13	T	C	0.268	0.064	0.008	1.2E-14	trans
59	AGER	Q15109	Advanced glycosylation end product-specific receptor	rs10152355	58671178	15	A	C	0.453	0.065	0.008	1.2E-17	trans
59	AGER	Q15109	Advanced glycosylation end product-specific receptor	rs34262842	20355811	16	A	G	0.834	0.068	0.010	5.6E-12	trans
60	AGR2	O95994	Anterior gradient protein 2 homolog	rs115317719	32412005	6	T	G	0.187	0.078	0.010	1.8E-15	trans
60	AGR2	O95994	Anterior gradient protein 2 homolog	rs7807123	16856549	7	T	C	0.566	0.107	0.008	8.7E-44	cis
60	AGR2	O95994	Anterior gradient protein 2 homolog	rs1580180	59387337	8	C	T	0.666	0.060	0.008	7.9E-14	trans
60	AGR2	O95994	Anterior gradient protein 2 homolog	rs28633254	1220599	11	T	C	0.583	0.057	0.008	2.7E-13	trans
60	AGR2	O95994	Anterior gradient protein 2 homolog	rs601338	49206674	19	G	A	0.491	0.059	0.008	1.9E-14	trans
61	AGR3	Q8TD06	Anterior gradient protein 3	rs58198139	156399039	5	T	C	0.638	0.079	0.008	3.5E-23	trans
61	AGR3	Q8TD06	Anterior gradient protein 3	rs9271956	32597313	6	C	T	0.834	0.072	0.010	7.1E-12	trans
61	AGR3	Q8TD06	Anterior gradient protein 3	rs12983058	51642784	19	G	T	0.673	0.058	0.008	1.0E-12	trans
62	AGRN	O00468	Agrin	rs3121560	968226	1	C	A	0.660	0.378	0.008	0.0E+00	cis
62	AGRN	O00468	Agrin	rs13107325	103188709	4	T	C	0.075	0.098	0.014	8.4E-13	trans
62	AGRN	O00468	Agrin	rs7654228	119299978	4	T	C	0.590	0.087	0.007	1.7E-32	trans
62	AGRN	O00468	Agrin	rs4841133	9183664	8	A	G	0.092	0.086	0.012	2.7E-12	trans
62	AGRN	O00468	Agrin	rs881930	76484241	11	G	A	0.828	0.085	0.010	2.5E-19	trans
62	AGRN	O00468	Agrin	rs8668	60964064	20	G	A	0.303	0.120	0.008	4.1E-53	trans
63	AGRP	O00253	Agouti-related protein	rs12239737	63086001	1	T	A	0.663	0.053	0.008	1.2E-11	trans
63	AGRP	O00253	Agouti-related protein	rs1260326	27730940	2	T	C	0.392	0.053	0.007	1.8E-12	trans
63	AGRP	O00253	Agouti-related protein	rs56393506	161089307	6	T	C	0.172	0.092	0.010	1.4E-20	trans
63	AGRP	O00253	Agouti-related protein	rs80026582	19932863	8	C	T	0.916	0.112	0.013	1.5E-17	trans
63	AGRP	O00253	Agouti-related protein	rs7850767	127254523	9	T	C	0.487	0.052	0.007	1.8E-12	trans
63	AGRP	O00253	Agouti-related protein	rs964184	116648917	11	G	C	0.133	0.148	0.011	2.1E-43	trans
63	AGRP	O00253	Agouti-related protein	rs188707867	67515388	16	C	G	0.993	0.469	0.046	1.1E-24	cis
63	AGRP	O00253	Agouti-related protein	rs740516	67082962	17	C	G	0.852	0.077	0.010	1.0E-13	trans
64	AGT	P01019	Angiotensinogen	rs4762	230845977	1	A	G	0.117	0.744	0.012	0.0E+00	cis
64	AGT	P01019	Angiotensinogen	rs62466318	73042085	7	C	T	0.798	0.085	0.009	6.0E-22	trans
65	AGXT	P21549	Serine--pyruvate aminotransferase	rs1497406	16505320	1	G	A	0.580	0.064	0.007	2.1E-18	trans
65	AGXT	P21549	Serine--pyruvate aminotransferase	rs4361977	155076079	1	C	T	0.566	0.063	0.007	7.7E-18	trans
65	AGXT	P21549	Serine--pyruvate aminotransferase	rs55649245	241793545	2	G	A	0.690	0.288	0.008	1.8E-281	cis
65	AGXT	P21549	Serine--pyruvate aminotransferase	rs4835265	146821410	4	A	C	0.156	0.130	0.010	8.4E-39	trans
65	AGXT	P21549	Serine--pyruvate aminotransferase	rs112875651	126506694	8	G	A	0.609	0.053	0.008	3.0E-12	trans
65	AGXT	P21549	Serine--pyruvate aminotransferase	rs10883451	101924418	10	T	C	0.498	0.051	0.007	1.9E-12	trans
65	AGXT	P21549	Serine--pyruvate aminotransferase	rs28929474	94844947	14	T	C	0.021	0.198	0.025	6.6E-15	trans
65	AGXT	P21549	Serine--pyruvate aminotransferase	rs4503880	56084054	18	T	C	0.201	0.102	0.009	6.3E-29	trans
65	AGXT	P21549	Serine--pyruvate aminotransferase	rs10405357	54759666	19	T	C	0.565	0.057	0.007	1.5E-14	trans
65	AGXT	P21549	Serine--pyruvate aminotransferase	rs3747207	44324855	22	A	G	0.215	0.080	0.009	8.6E-20	trans
66	AHCY	P23526	Adenosylhomocysteinase	rs13043752	32883308	20	G	A	0.984	1.375	0.030	0.0E+00	cis
67	AHNAK	Q09666	Neuroblast differentiation-associated protein AHNAK	rs74853209	62296601	11	T	C	0.972	0.473	0.022	6.8E-99	cis
68	AHNAK2	Q8IVF2	Protein AHNAK2	rs9805957	105437057	14	C	A	0.642	0.216	0.008	1.8E-163	cis
69	AHSG	P02765	Alpha-2-HS-glycoprotein	rs1900618	186338135	3	T	C	0.640	0.676	0.008	0.0E+00	cis
69	AHSG	P02765	Alpha-2-HS-glycoprotein	rs28929474	94844947	14	T	C	0.021	0.164	0.023	6.3E-13	trans
70	AHSP	Q9NZD4	Alpha-hemoglobin-stabilizing protein	rs875741	173287851	5	G	A	0.593	0.052	0.007	5.1E-13	trans
70	AHSP	Q9NZD4	Alpha-hemoglobin-stabilizing protein	rs80215559	25918225	6	C	T	0.077	0.133	0.013	1.1E-23	trans
70	AHSP	Q9NZD4	Alpha-hemoglobin-stabilizing protein	rs11759553	135422296	6	A	T	0.728	0.060	0.008	1.0E-13	trans
70	AHSP	Q9NZD4	Alpha-hemoglobin-stabilizing protein	rs590856	139844429	6	G	A	0.447	0.079	0.007	7.1E-28	trans
70	AHSP	Q9NZD4	Alpha-hemoglobin-stabilizing protein	rs6592965	50427982	7	G	A	0.546	0.067	0.007	1.0E-20	trans
70	AHSP	Q9NZD4	Alpha-hemoglobin-stabilizing protein	rs10742583	5248641	11	A	G	0.847	0.117	0.010	2.0E-30	trans
70	AHSP	Q9NZD4	Alpha-hemoglobin-stabilizing protein	rs1000821	74434003	17	C	T	0.535	0.112	0.007	1.2E-55	trans
70	AHSP	Q9NZD4	Alpha-hemoglobin-stabilizing protein	rs855791	37462936	22	G	A	0.561	0.079	0.007	1.0E-28	trans
71	AIF1	P55008	Allograft inflammatory factor 1	rs2261033	31603591	6	A	G	0.569	0.111	0.008	7.5E-47	cis
72	AIF1L	Q9BQI0	Allograft inflammatory factor 1-like	rs9275036	32645319	6	T	C	0.330	0.059	0.008	2.0E-12	trans
72	AIF1L	Q9BQI0	Allograft inflammatory factor 1-like	rs7856365	133934926	9	G	A	0.316	0.311	0.008	1.9E-307	cis
73	AIFM1	O95831	Apoptosis-inducing factor 1, mitochondrial	rs1354034	56849749	3	C	T	0.605	0.053	0.008	4.0E-12	trans
73	AIFM1	O95831	Apoptosis-inducing factor 1, mitochondrial	rs6993770	106581528	8	A	T	0.714	0.058	0.008	3.7E-12	trans
74	AKR1B1	P15121	Aldo-keto reductase family 1 member B1	rs2229542	134135621	7	T	C	0.986	0.587	0.033	3.1E-69	cis
74	AKR1B1	P15121	Aldo-keto reductase family 1 member B1	rs6993770	106581528	8	A	T	0.713	0.068	0.008	1.4E-15	trans
74	AKR1B1	P15121	Aldo-keto reductase family 1 member B1	rs72481564	106352251	14	T	C	0.227	0.083	0.010	4.6E-16	trans
75	AKR1C4	P17516	Aldo-keto reductase family 1 member C4	rs11253048	5261159	10	A	G	0.823	0.068	0.010	1.5E-11	cis
76	AKR7L	Q8NHP1	Aflatoxin B1 aldehyde reductase member 4	rs59622577	19609013	1	C	T	0.903	0.317	0.013	5.4E-133	cis
76	AKR7L	Q8NHP1	Aflatoxin B1 aldehyde reductase member 4	rs58534292	106483584	14	T	C	0.350	0.066	0.008	2.0E-16	trans
76	AKR7L	Q8NHP1	Aflatoxin B1 aldehyde reductase member 4	rs34160075	106453965	14	G	A	0.372	0.074	0.008	1.2E-18	trans
76	AKR7L	Q8NHP1	Aflatoxin B1 aldehyde reductase member 4	rs3747207	44324855	22	A	G	0.215	0.076	0.009	1.2E-16	trans
77	AKT1S1	Q96B36	Proline-rich AKT1 substrate 1	rs1354034	56849749	3	C	T	0.604	0.067	0.008	3.3E-18	trans
77	AKT1S1	Q96B36	Proline-rich AKT1 substrate 1	rs342298	106373646	7	C	T	0.546	0.056	0.008	1.5E-13	trans
77	AKT1S1	Q96B36	Proline-rich AKT1 substrate 1	rs10733789	64948684	10	C	T	0.313	0.066	0.008	4.3E-16	trans
78	AKT2	P31751	RAC-beta serine/threonine-protein kinase	rs1354034	56849749	3	C	T	0.604	0.057	0.008	1.4E-13	trans
78	AKT2	P31751	RAC-beta serine/threonine-protein kinase	rs2706396	131796809	5	C	A	0.571	0.056	0.008	2.7E-13	trans
78	AKT2	P31751	RAC-beta serine/threonine-protein kinase	rs6993770	106581528	8	A	T	0.714	0.068	0.008	1.7E-16	trans
79	AKT3	Q9Y243	RAC-gamma serine/threonine-protein kinase	rs4515770	243686913	1	C	T	0.218	0.065	0.009	3.0E-12	cis
79	AKT3	Q9Y243	RAC-gamma serine/threonine-protein kinase	rs7916868	64988931	10	T	A	0.502	0.055	0.008	8.5E-13	trans
80	ALCAM	Q13740	CD166 antigen	rs34926152	105264176	3	G	T	0.951	0.303	0.016	5.5E-80	cis
80	ALCAM	Q13740	CD166 antigen	rs9274626	32636040	6	T	C	0.321	0.058	0.008	1.9E-14	trans
80	ALCAM	Q13740	CD166 antigen	rs10901252	136128000	9	C	G	0.061	0.102	0.014	1.3E-12	trans
80	ALCAM	Q13740	CD166 antigen	rs59822672	126270975	11	G	C	0.924	0.094	0.013	6.8E-13	trans
80	ALCAM	Q13740	CD166 antigen	rs12448760	16239539	16	A	G	0.286	0.096	0.008	1.9E-36	trans
80	ALCAM	Q13740	CD166 antigen	rs186021206	7069412	17	A	G	0.006	0.850	0.047	3.6E-72	trans
80	ALCAM	Q13740	CD166 antigen	rs7224668	79235788	17	T	C	0.443	0.051	0.007	2.9E-13	trans
80	ALCAM	Q13740	CD166 antigen	rs2638282	49213833	19	A	G	0.533	0.059	0.007	1.2E-17	trans
80	ALCAM	Q13740	CD166 antigen	rs2294915	44340904	22	T	C	0.232	0.078	0.008	8.2E-22	trans
81	ALDH1A1	P00352	Retinal dehydrogenase 1	rs112596047	75548526	9	T	A	0.265	0.091	0.008	9.9E-29	cis
81	ALDH1A1	P00352	Retinal dehydrogenase 1	rs3747207	44324855	22	A	G	0.215	0.074	0.009	1.4E-17	trans
82	ALDH3A1	P30838	Aldehyde dehydrogenase, dimeric NADP-preferring	rs887241	19645938	17	C	A	0.658	0.484	0.008	0.0E+00	cis
83	ALDH5A1	P51649	Succinate-semialdehyde dehydrogenase, mitochondrial	rs2744581	24502484	6	A	G	0.481	0.141	0.008	5.5E-77	cis
84	ALPI	P09923	Intestinal-type alkaline phosphatase	rs11216930	118488782	11	A	C	0.774	0.056	0.007	2.3E-14	trans
84	ALPI	P09923	Intestinal-type alkaline phosphatase	rs186021206	7069412	17	A	G	0.006	0.751	0.043	1.2E-69	trans
84	ALPI	P09923	Intestinal-type alkaline phosphatase	rs79008529	5816953	19	A	G	0.046	0.119	0.017	1.4E-12	trans
84	ALPI	P09923	Intestinal-type alkaline phosphatase	rs681343	49206462	19	C	T	0.491	0.611	0.007	0.0E+00	trans
85	ALPP	P05187	Alkaline phosphatase, placental type	rs761164	40351651	1	G	A	0.149	0.069	0.009	1.6E-13	trans
85	ALPP	P05187	Alkaline phosphatase, placental type	rs12743084	155161168	1	G	C	0.542	0.063	0.007	8.0E-21	trans
85	ALPP	P05187	Alkaline phosphatase, placental type	rs12478529	233286654	2	T	C	0.235	0.506	0.009	0.0E+00	cis
85	ALPP	P05187	Alkaline phosphatase, placental type	rs2076295	7563232	6	G	T	0.448	0.046	0.007	1.2E-11	trans
85	ALPP	P05187	Alkaline phosphatase, placental type	rs55792059	53352206	6	A	C	0.191	0.075	0.009	1.2E-17	trans
85	ALPP	P05187	Alkaline phosphatase, placental type	rs117847945	79862330	6	G	A	0.019	0.175	0.024	7.6E-13	trans
85	ALPP	P05187	Alkaline phosphatase, placental type	rs3936578	109482027	9	A	C	0.510	0.080	0.007	4.8E-32	trans
85	ALPP	P05187	Alkaline phosphatase, placental type	rs7167092	31626873	15	T	C	0.649	0.052	0.007	1.1E-13	trans
85	ALPP	P05187	Alkaline phosphatase, placental type	rs186021206	7069412	17	A	G	0.006	0.463	0.046	1.2E-23	trans
85	ALPP	P05187	Alkaline phosphatase, placental type	rs516246	49206172	19	C	T	0.491	0.117	0.007	1.2E-67	trans
85	ALPP	P05187	Alkaline phosphatase, placental type	rs5913932	56319136	X	G	A	0.775	0.049	0.007	1.2E-13	trans
86	AMBP	P02760	Protein AMBP	rs61747728	179526214	1	T	C	0.038	0.149	0.018	6.6E-16	trans
86	AMBP	P02760	Protein AMBP	rs75166367	162964301	2	G	A	0.940	0.102	0.015	7.5E-12	trans
86	AMBP	P02760	Protein AMBP	rs406658	31996524	6	A	C	0.109	0.152	0.012	4.3E-38	trans
86	AMBP	P02760	Protein AMBP	rs10265221	151414329	7	C	T	0.287	0.053	0.008	6.8E-12	trans
86	AMBP	P02760	Protein AMBP	rs147941577	116831989	9	A	G	0.997	1.690	0.067	2.8E-142	cis
86	AMBP	P02760	Protein AMBP	rs28929474	94844947	14	T	C	0.021	0.180	0.025	4.4E-13	trans
86	AMBP	P02760	Protein AMBP	rs34262842	20355811	16	A	G	0.834	0.064	0.010	1.5E-11	trans
86	AMBP	P02760	Protein AMBP	rs704	26694861	17	G	A	0.527	0.109	0.007	8.4E-54	trans
87	AMIGO2	Q86SJ2	Amphoterin-induced protein 2	rs3791101	44366250	1	A	G	0.308	0.061	0.008	4.0E-14	trans
87	AMIGO2	Q86SJ2	Amphoterin-induced protein 2	rs1524668	9697372	2	C	A	0.667	0.062	0.008	3.5E-15	trans
87	AMIGO2	Q86SJ2	Amphoterin-induced protein 2	rs1354034	56849749	3	T	C	0.395	0.146	0.008	4.6E-83	trans
87	AMIGO2	Q86SJ2	Amphoterin-induced protein 2	rs10935473	98416900	3	G	T	0.560	0.178	0.008	2.6E-123	trans
87	AMIGO2	Q86SJ2	Amphoterin-induced protein 2	rs1281937	32578143	6	T	C	0.725	0.062	0.008	8.6E-14	trans
87	AMIGO2	Q86SJ2	Amphoterin-induced protein 2	rs385893	4763176	9	C	T	0.524	0.061	0.007	3.3E-16	trans
87	AMIGO2	Q86SJ2	Amphoterin-induced protein 2	rs2519093	136141870	9	C	T	0.816	0.169	0.010	1.8E-69	trans
87	AMIGO2	Q86SJ2	Amphoterin-induced protein 2	rs174546	61569830	11	T	C	0.344	0.063	0.008	4.2E-16	trans
87	AMIGO2	Q86SJ2	Amphoterin-induced protein 2	rs4055121	126232337	11	C	T	0.865	0.118	0.011	2.3E-27	trans
87	AMIGO2	Q86SJ2	Amphoterin-induced protein 2	rs682619	59098773	15	C	T	0.359	0.052	0.008	1.3E-11	trans
87	AMIGO2	Q86SJ2	Amphoterin-induced protein 2	rs186021206	7069412	17	A	G	0.006	0.981	0.051	2.9E-81	trans
88	AMN	Q9BXJ7	Protein amnionless	rs12740374	109817590	1	T	G	0.221	0.070	0.008	1.4E-16	trans
88	AMN	Q9BXJ7	Protein amnionless	rs28929474	94844947	14	T	C	0.021	0.682	0.027	3.2E-140	trans
88	AMN	Q9BXJ7	Protein amnionless	rs2295829	103389003	14	C	G	0.390	0.770	0.009	0.0E+00	cis
88	AMN	Q9BXJ7	Protein amnionless	rs1801689	64210580	17	C	A	0.030	0.238	0.021	5.6E-31	trans
89	AMOT	Q4VCS5	Angiomotin	rs4846913	230294715	1	A	C	0.607	0.049	0.007	1.4E-11	trans
89	AMOT	Q4VCS5	Angiomotin	rs35719237	148981178	4	C	CGA	0.256	0.064	0.008	3.1E-15	trans
89	AMOT	Q4VCS5	Angiomotin	rs2954021	126482077	8	G	A	0.504	0.054	0.007	1.1E-14	trans
89	AMOT	Q4VCS5	Angiomotin	rs533333382	94572076	11	A	G	0.190	0.251	0.009	1.5E-168	trans
89	AMOT	Q4VCS5	Angiomotin	rs340005	60878030	15	A	G	0.624	0.049	0.007	7.3E-12	trans
90	AMOTL2	Q9Y2J4	Angiomotin-like protein 2	rs1353778	134088443	3	T	A	0.613	0.061	0.008	5.9E-15	cis
90	AMOTL2	Q9Y2J4	Angiomotin-like protein 2	rs3780420	100677166	9	C	T	0.364	0.058	0.008	1.8E-13	trans
91	AMPD3	Q01432	AMP deaminase 3	rs117706710	10508903	11	G	T	0.991	1.717	0.038	0.0E+00	cis
92	AMY1A_AMY1B_AMY1(	P0DUB6,P0DTE7,P0DTE8	Alpha-amylase 1A_Alpha-amylase 1B_Alpha-amylase 1C	rs72694438	104364878	1	A	G	0.207	0.403	0.010	0.0E+00	cis
92	AMY1A_AMY1B_AMY1(	P0DUB6,P0DTE7,P0DTE8	Alpha-amylase 1A_Alpha-amylase 1B_Alpha-amylase 1C	rs291091	207113679	1	C	T	0.453	0.065	0.008	1.0E-16	trans
92	AMY1A_AMY1B_AMY1(	P0DUB6,P0DTE7,P0DTE8	Alpha-amylase 1A_Alpha-amylase 1B_Alpha-amylase 1C	rs13159800	44325469	5	T	C	0.331	0.073	0.008	1.7E-21	trans
92	AMY1A_AMY1B_AMY1(	P0DUB6,P0DTE7,P0DTE8	Alpha-amylase 1A_Alpha-amylase 1B_Alpha-amylase 1C	rs888686	134510772	5	A	T	0.408	0.052	0.007	1.6E-12	trans
92	AMY1A_AMY1B_AMY1(	P0DUB6,P0DTE7,P0DTE8	Alpha-amylase 1A_Alpha-amylase 1B_Alpha-amylase 1C	rs56278466	17875857	10	G	T	0.660	0.051	0.008	1.4E-11	trans
92	AMY1A_AMY1B_AMY1(	P0DUB6,P0DTE7,P0DTE8	Alpha-amylase 1A_Alpha-amylase 1B_Alpha-amylase 1C	rs2638282	49213833	19	A	G	0.533	0.072	0.007	1.6E-23	trans
93	AMY2A	P04746	Pancreatic alpha-amylase	rs78245241	104335726	1	T	G	0.963	0.964	0.021	0.0E+00	cis
93	AMY2A	P04746	Pancreatic alpha-amylase	rs55901622	127157438	6	G	A	0.269	0.056	0.008	3.5E-12	trans
93	AMY2A	P04746	Pancreatic alpha-amylase	rs1561929	129567373	8	T	C	0.883	0.076	0.011	1.4E-11	trans
93	AMY2A	P04746	Pancreatic alpha-amylase	rs533406	88974860	16	A	G	0.617	0.071	0.007	1.4E-22	trans
93	AMY2A	P04746	Pancreatic alpha-amylase	rs2638282	49213833	19	A	G	0.533	0.052	0.007	4.2E-13	trans

94	AMY2B	P19961	Alpha-amylase 2B	rs12080068	104329823	1	A	C	0.092	0.659	0.014	0.0E+00	cis
94	AMY2B	P19961	Alpha-amylase 2B	rs1561929	129567373	8	T	C	0.883	0.081	0.011	1.8E-12	trans
94	AMY2B	P19961	Alpha-amylase 2B	rs56278466	17875857	10	G	T	0.662	0.055	0.008	4.6E-13	trans
94	AMY2B	P19961	Alpha-amylase 2B	rs28456	61589481	11	G	A	0.314	0.054	0.008	3.4E-12	trans
94	AMY2B	P19961	Alpha-amylase 2B	rs533406	88974860	16	A	G	0.617	0.078	0.007	1.4E-25	trans
94	AMY2B	P19961	Alpha-amylase 2B	rs2638282	49213833	19	A	G	0.532	0.053	0.007	6.6E-13	trans
95	ANG	P03950	Angiogenin	rs61791757	135905325	3	G	A	0.769	0.060	0.008	1.7E-14	trans
95	ANG	P03950	Angiogenin	rs8008227	21156304	14	A	G	0.117	0.667	0.011	0.0E+00	cis
96	ANGPT1	Q15389	Angiopoietin-1	rs12041331	156869714	1	G	A	0.914	0.103	0.013	1.2E-14	trans
96	ANGPT1	Q15389	Angiopoietin-1	rs13412535	224874874	2	G	A	0.769	0.078	0.009	1.1E-18	trans
96	ANGPT1	Q15389	Angiopoietin-1	rs7618405	18250509	3	C	A	0.795	0.071	0.009	2.0E-14	trans
96	ANGPT1	Q15389	Angiopoietin-1	rs1354034	56849749	3	T	C	0.396	0.087	0.008	1.2E-30	trans
96	ANGPT1	Q15389	Angiopoietin-1	rs3792366	122839876	3	G	A	0.407	0.055	0.008	6.2E-13	trans
96	ANGPT1	Q15389	Angiopoietin-1	rs10058074	131686146	5	G	A	0.524	0.065	0.007	1.9E-18	trans
96	ANGPT1	Q15389	Angiopoietin-1	rs2516471	31400292	6	G	A	0.936	0.138	0.015	3.5E-19	trans
96	ANGPT1	Q15389	Angiopoietin-1	rs146842698	71328273	6	T	TAAATA	0.695	0.069	0.008	3.6E-17	trans
96	ANGPT1	Q15389	Angiopoietin-1	rs6961069	80218961	7	T	C	0.402	0.077	0.008	2.5E-24	trans
96	ANGPT1	Q15389	Angiopoietin-1	rs6993770	106581528	8	A	T	0.713	0.195	0.008	1.2E-122	cis
96	ANGPT1	Q15389	Angiopoietin-1	rs2047792	91444677	9	G	A	0.938	0.105	0.016	1.1E-11	trans
96	ANGPT1	Q15389	Angiopoietin-1	rs61751937	113312231	9	C	G	0.028	0.154	0.022	4.5E-12	trans
96	ANGPT1	Q15389	Angiopoietin-1	rs113344952	3139180	10	G	C	0.647	0.058	0.008	5.7E-13	trans
96	ANGPT1	Q15389	Angiopoietin-1	rs7080386	65048306	10	A	C	0.412	0.098	0.008	1.2E-38	trans
96	ANGPT1	Q15389	Angiopoietin-1	rs61978213	70653758	14	A	G	0.042	0.141	0.019	3.4E-14	trans
96	ANGPT1	Q15389	Angiopoietin-1	rs59001897	65160392	15	A	T	0.174	0.081	0.010	2.0E-16	trans
96	ANGPT1	Q15389	Angiopoietin-1	rs151234	28505660	16	C	G	0.129	0.076	0.011	8.4E-12	trans
96	ANGPT1	Q15389	Angiopoietin-1	rs12445050	81870969	16	T	C	0.138	0.121	0.011	1.8E-29	trans
96	ANGPT1	Q15389	Angiopoietin-1	rs1654425	55538980	19	C	T	0.834	0.186	0.010	9.5E-76	trans
96	ANGPT1	Q15389	Angiopoietin-1	rs3827978	19281291	20	T	C	0.354	0.081	0.008	3.1E-25	trans
96	ANGPT1	Q15389	Angiopoietin-1	rs5967171	99957136	X	C	T	0.334	0.060	0.007	1.9E-20	trans
97	ANGPT2	O15123	Angiopoietin-2	rs13107325	103188709	4	T	C	0.075	0.121	0.014	4.4E-18	trans
97	ANGPT2	O15123	Angiopoietin-2	rs1968586	6279511	8	C	T	0.645	0.157	0.008	1.2E-89	cis
97	ANGPT2	O15123	Angiopoietin-2	rs4841133	9183664	8	A	G	0.092	0.108	0.013	3.8E-17	trans
97	ANGPT2	O15123	Angiopoietin-2	rs34434834	126307796	11	A	G	0.033	0.238	0.021	4.3E-31	trans
97	ANGPT2	O15123	Angiopoietin-2	rs186021206	7069412	17	A	G	0.006	0.862	0.050	1.9E-65	trans
98	ANGPTL1	O95841	Angiopoietin-related protein 1	rs12753251	178529898	1	G	A	0.466	0.284	0.008	0.0E+00	cis
98	ANGPTL1	O95841	Angiopoietin-related protein 1	rs1137654	101570336	9	A	T	0.906	0.093	0.013	4.8E-13	trans
98	ANGPTL1	O95841	Angiopoietin-related protein 1	rs28929474	94844947	14	T	C	0.021	0.195	0.025	1.4E-14	trans
98	ANGPTL1	O95841	Angiopoietin-related protein 1	rs149394327	64228995	17	C	G	0.030	0.245	0.021	8.7E-31	trans
98	ANGPTL1	O95841	Angiopoietin-related protein 1	rs8103017	55999142	19	G	C	0.291	0.061	0.008	4.6E-14	trans
99	ANGPTL2	Q9UKU9	Angiopoietin-related protein 2	rs4141100	7276954	7	A	G	0.614	0.064	0.008	2.7E-17	trans
99	ANGPTL2	Q9UKU9	Angiopoietin-related protein 2	rs55799523	55999545	19	A	C	0.265	0.264	0.009	3.6E-209	trans
99	ANGPTL2	Q9UKU9	Angiopoietin-related protein 2	rs181210490	3695977	20	A	G	0.012	0.573	0.034	5.1E-62	trans
100	ANGPTL3	Q9Y5C1	Angiopoietin-related protein 3	rs3916027	19824868	8	A	G	0.264	0.085	0.008	1.3E-28	trans
100	ANGPTL3	Q9Y5C1	Angiopoietin-related protein 3	rs10808546	126495818	8	T	C	0.446	0.051	0.007	6.0E-14	trans
100	ANGPTL3	Q9Y5C1	Angiopoietin-related protein 3	rs964184	116648917	11	G	C	0.133	0.118	0.010	3.6E-32	trans
100	ANGPTL3	Q9Y5C1	Angiopoietin-related protein 3	rs28929474	94844947	14	T	C	0.021	0.239	0.024	9.4E-24	trans
100	ANGPTL3	Q9Y5C1	Angiopoietin-related protein 3	rs72786786	56985514	16	A	G	0.313	0.051	0.007	9.4E-12	trans
100	ANGPTL3	Q9Y5C1	Angiopoietin-related protein 3	rs1065853	45413233	19	T	G	0.081	0.131	0.012	1.3E-25	trans
101	ANGPTL4	Q9BY76	Angiopoietin-related protein 4	rs2278236	8431581	19	A	G	0.514	0.280	0.008	1.7E-303	cis
102	ANGPTL7	O43827	Angiopoietin-related protein 7	rs28991009	11253684	1	G	T	0.992	2.155	0.040	0.0E+00	cis
102	ANGPTL7	O43827	Angiopoietin-related protein 7	rs6764769	141100280	3	A	G	0.556	0.060	0.007	2.8E-17	trans
102	ANGPTL7	O43827	Angiopoietin-related protein 7	rs7019896	33113196	9	T	G	0.933	0.108	0.014	2.6E-14	trans
102	ANGPTL7	O43827	Angiopoietin-related protein 7	rs112771035	126225876	11	C	G	0.929	0.118	0.014	1.1E-17	trans
102	ANGPTL7	O43827	Angiopoietin-related protein 7	rs186021206	7069412	17	A	G	0.006	0.759	0.049	1.5E-54	trans
102	ANGPTL7	O43827	Angiopoietin-related protein 7	rs704	26694861	17	A	G	0.473	0.058	0.007	2.6E-16	trans
103	ANK2	Q01484	Ankyrin-2	rs144646268	98062753	10	T	G	0.039	0.160	0.020	2.2E-15	trans
104	ANKMY2	Q8IV38	Ankyrin repeat and MYND domain-containing protein 2	rs1354034	56849749	3	C	T	0.604	0.085	0.008	1.2E-28	trans
104	ANKMY2	Q8IV38	Ankyrin repeat and MYND domain-containing protein 2	rs4719474	16676955	7	A	C	0.376	0.082	0.008	1.4E-24	cis
104	ANKMY2	Q8IV38	Ankyrin repeat and MYND domain-containing protein 2	rs7080386	65048306	10	A	C	0.412	0.054	0.008	3.0E-12	trans
104	ANKMY2	Q8IV38	Ankyrin repeat and MYND domain-containing protein 2	rs11502185	180258	11	C	T	0.260	0.072	0.010	6.8E-14	trans
105	ANKRD54	Q6NXT1	Ankyrin repeat domain-containing protein 54	rs35496925	38424607	22	G	A	0.059	0.143	0.016	5.4E-19	cis
106	ANPEP	P15144	Aminopeptidase N	rs13107325	103188709	4	T	C	0.075	0.094	0.013	1.4E-12	trans
106	ANPEP	P15144	Aminopeptidase N	rs4835265	146821410	4	A	C	0.157	0.071	0.010	1.2E-13	trans
106	ANPEP	P15144	Aminopeptidase N	rs10142200	103568729	14	G	A	0.409	0.053	0.007	4.0E-14	trans
106	ANPEP	P15144	Aminopeptidase N	rs1042499	90328305	15	C	T	0.057	0.819	0.016	0.0E+00	cis
106	ANPEP	P15144	Aminopeptidase N	rs186021206	7069412	17	A	G	0.006	0.639	0.048	9.3E-41	trans
107	ANXA1	P04083	Annexin A1	rs35923643	123355391	11	A	G	0.804	0.093	0.009	5.9E-23	trans
107	ANXA1	P04083	Annexin A1	rs58534292	106483584	14	C	T	0.650	0.053	0.008	1.6E-11	trans
107	ANXA1	P04083	Annexin A1	rs12885254	106487017	14	A	T	0.705	0.064	0.009	2.4E-13	trans
107	ANXA1	P04083	Annexin A1	rs34436714	54327313	19	A	C	0.209	0.129	0.009	2.2E-44	trans
108	ANXA11	P50995	Annexin A11	rs2784773	81919800	10	C	T	0.644	0.066	0.008	4.5E-17	cis
109	ANXA2	P07355	Annexin A2	rs342298	106373646	7	C	T	0.545	0.056	0.007	1.9E-15	trans
109	ANXA2	P07355	Annexin A2	rs8033800	60689179	15	T	A	0.398	0.461	0.008	0.0E+00	cis
109	ANXA2	P07355	Annexin A2	rs4632248	54324995	19	T	G	0.209	0.119	0.009	3.1E-42	trans
110	ANXA3	P12429	Annexin A3	rs146728751	79500235	4	G	A	0.998	1.222	0.093	2.7E-39	cis
110	ANXA3	P12429	Annexin A3	rs6993770	106581528	8	A	T	0.713	0.067	0.008	2.3E-16	trans
110	ANXA3	P12429	Annexin A3	rs7896518	65104500	10	G	A	0.424	0.059	0.008	7.8E-15	trans
110	ANXA3	P12429	Annexin A3	rs10418046	54327869	19	G	T	0.211	0.065	0.009	9.1E-13	trans
111	ANXA4	P09525	Annexin A4	rs1354034	56849749	3	C	T	0.604	0.053	0.008	3.7E-12	trans
111	ANXA4	P09525	Annexin A4	rs7896518	65104500	10	G	A	0.423	0.069	0.008	2.6E-19	trans
112	ANXA5	P08758	Annexin A5	rs2306411	122598635	4	A	G	0.709	0.117	0.008	2.2E-43	cis
113	AOC1	P19801	Amiloride-sensitive amine oxidase [copper-containing]	rs1049742	150554553	7	T	C	0.081	0.495	0.014	1.2E-270	cis
114	AOC3	Q16853	Membrane primary amine oxidase	rs2270894	9975386	3	G	C	0.203	0.081	0.008	1.5E-21	trans
114	AOC3	Q16853	Membrane primary amine oxidase	rs13135092	103198082	4	G	A	0.084	0.086	0.012	7.4E-13	trans
114	AOC3	Q16853	Membrane primary amine oxidase	rs75901433	32499150	6	G	A	0.558	0.054	0.007	2.5E-15	trans
114	AOC3	Q16853	Membrane primary amine oxidase	rs767298	7274584	7	G	T	0.366	0.065	0.007	4.2E-21	trans
114	AOC3	Q16853	Membrane primary amine oxidase	rs9411365	136118268	9	G	C	0.072	0.098	0.013	2.8E-14	trans
114	AOC3	Q16853	Membrane primary amine oxidase	rs56278466	17875857	10	G	T	0.661	0.067	0.007	3.7E-22	trans
114	AOC3	Q16853	Membrane primary amine oxidase	rs3967200	126232385	11	C	T	0.865	0.101	0.010	1.6E-25	trans
114	AOC3	Q16853	Membrane primary amine oxidase	rs1042704	23312594	14	G	A	0.785	0.092	0.008	8.8E-31	trans
114	AOC3	Q16853	Membrane primary amine oxidase	rs12913657	51000968	15	C	T	0.147	0.079	0.009	1.7E-17	trans
114	AOC3	Q16853	Membrane primary amine oxidase	rs186021206	7069412	17	A	G	0.006	0.591	0.050	1.3E-32	trans
114	AOC3	Q16853	Membrane primary amine oxidase	rs33986943	41004637	17	G	A	0.889	0.867	0.012	0.0E+00	cis
115	AP1G2	O75843	AP-1 complex subunit gamma-like 2	rs28403575	24025340	14	C	T	0.912	0.192	0.013	2.0E-48	cis
116	AP2B1	P63010	AP-2 complex subunit beta	rs3811444	248039451	1	C	T	0.668	0.082	0.008	4.0E-24	trans
116	AP2B1	P63010	AP-2 complex subunit beta	rs1354034	56849749	3	C	T	0.604	0.058	0.008	6.8E-14	trans
117	AP3B1	O00203	AP-3 complex subunit beta-1	rs1354034	56849749	3	C	T	0.605	0.077	0.008	5.1E-24	trans
117	AP3B1	O00203	AP-3 complex subunit beta-1	rs6993770	106581528	8	A	T	0.713	0.058	0.008	1.7E-12	trans
118	AP3S2	P59780	AP-3 complex subunit sigma-2	rs1354034	56849749	3	C	T	0.604	0.063	0.008	1.9E-16	trans
119	APBB1IP	Q7Z5R6	Amyloid beta A4 precursor protein-binding family B member 1-interacting protein	rs7461753	144981498	8	C	A	0.420	0.057	0.007	2.7E-14	trans
119	APBB1IP	Q7Z5R6	Amyloid beta A4 precursor protein-binding family B member 1-interacting protein	rs2992335	26727934	10	C	G	0.399	0.217	0.008	4.4E-179	cis
119	APBB1IP	Q7Z5R6	Amyloid beta A4 precursor protein-binding family B member 1-interacting protein	rs11671654	12014355	19	G	C	0.495	0.150	0.007	9.0E-90	trans
119	APBB1IP	Q7Z5R6	Amyloid beta A4 precursor protein-binding family B member 1-interacting protein	rs10418046	54327869	19	G	T	0.211	0.113	0.009	2.3E-35	trans
120	APCS	P02743	Serum amyloid P-component	rs36126250	159563701	1	T	G	0.977	0.535	0.024	7.5E-114	cis
120	APCS	P02743	Serum amyloid P-component	rs2842700	207282149	1	A	C	0.054	0.155	0.016	5.0E-23	trans
120	APCS	P02743	Serum amyloid P-component	rs1260326	27730940	2	T	C	0.392	0.095	0.007	1.7E-40	trans
121	APEX1	P27695	DNA-(apurinic or apyrimidinic site) endonuclease	rs1354034	56849749	3	C	T	0.604	0.057	0.007	1.3E-15	trans
121	APEX1	P27695	DNA-(apurinic or apyrimidinic site) endonuclease	rs1130409	20925154	14	T	G	0.520	0.389	0.007	0.0E+00	cis
121	APEX1	P27695	DNA-(apurinic or apyrimidinic site) endonuclease	rs4632248	54324995	19	T	G	0.210	0.067	0.009	1.5E-14	trans
122	APLP1	P51693	Amyloid-like protein 1	rs13412535	224874874	2	A	G	0.231	0.074	0.009	9.4E-17	trans
122	APLP1	P51693	Amyloid-like protein 1	rs4602861	106590706	8	G	A	0.274	0.097	0.008	1.5E-30	trans
122	APLP1	P51693	Amyloid-like protein 1	rs2229498	70856852	10	G	A	0.164	0.112	0.010	8.7E-29	trans
122	APLP1	P51693	Amyloid-like protein 1	rs12609736	55545284	19	A	G	0.168	0.104	0.010	4.3E-25	trans
122	APLP1	P51693	Amyloid-like protein 1	rs6045989	19292926	20	G	A	0.644	0.057	0.008	2.9E-13	trans
123	APOA1	P02647	Apolipoprotein A-I	rs62568183	107650345	9	T	G	0.883	0.083	0.011	6.8E-14	trans
123	APOA1	P02647	Apolipoprotein A-I	rs2727784	116711141	11	C	T	0.350	0.064	0.007	1.8E-17	cis
123	APOA1	P02647	Apolipoprotein A-I	rs1077835	58723426	15	G	A	0.222	0.135	0.009	1.2E-56	trans
123	APOA1	P02647	Apolipoprotein A-I	rs3764261	56993324	16	A	C	0.325	0.161	0.008	1.1E-99	trans
123	APOA1	P02647	Apolipoprotein A-I	rs77960347	47109955	18	G	A	0.013	0.307	0.031	6.1E-23	trans
123	APOA1	P02647	Apolipoprotein A-I	rs4420638	45422946	19	A	G	0.810	0.083	0.009	4.4E-20	trans
124	APOA2	P02652	Apolipoprotein A-II	rs4073054	161200487	1	A	C	0.626	0.067	0.008	5.6E-19	cis
124	APOA2	P02652	Apolipoprotein A-II	rs6682862	177938437	1	G	A	0.833	0.076	0.010	7.9E-15	trans
124	APOA2	P02652	Apolipoprotein A-II	rs11096641	20374158	2	G	A	0.529	0.089	0.007	1.4E-34	trans
124	APOA2	P02652	Apolipoprotein A-II	rs13107325	103188709	4	C	T	0.925	0.145	0.014	1.6E-25	trans
124	APOA2	P02652	Apolipoprotein A-II	rs4240624	9184231	8	A	G	0.908	0.121	0.013	1.4E-21	trans
124	APOA2	P02652	Apolipoprotein A-II	rs28601761	126500031	8	G	C	0.420	0.055	0.007	1.3E-13	trans
124	APOA2	P02652	Apolipoprotein A-II	rs62568182	107650343	9	T	G	0.879	0.086	0.011	1.9E-14	trans
124	APOA2	P02652	Apolipoprotein A-II	rs12555241	139387676	9	G	A	0.722	0.059	0.008	8.2E-13	trans
124	APOA2	P02652	Apolipoprotein A-II	rs2727784	116711141	11	C	T	0.350	0.057	0.008	1.3E-13	trans
124	APOA2	P02652	Apolipoprotein A-II	rs339969	60883281	15	A	C	0.617	0.052	0.007	2.9E-12	trans
124	APOA2	P02652	Apolipoprotein A-II	rs56228609	56987765	16	T	C	0.316	0.095	0.008	1.6E-33	trans
124	APOA2	P02652	Apolipoprotein A-II	rs58542926	19379549	19	T	C	0.075	0.103	0.014	8.1E-14	trans
124	APOA2	P02652	Apolipoprotein A-II	rs429358	45411941	19	C	T	0.156	0.127	0.010	2.2E-36	trans
125	APOA4	P06727	Apolipoprotein A-IV	rs150816167	179571862	1	C	T	0.042	0.167	0.019	9.8E-19	trans
125	APOA4	P06727	Apolipoprotein A-IV	rs9273206	32613573	6	C	T	0.835	0.087	0.010	2.2E-17	trans
125	APOA4	P06727	Apolipoprotein A-IV	rs1729407	116677370	11	G	C	0.549	0.141	0.008	6.3E-77	cis
126	APOBR	Q0VD83	Apolipoprotein B receptor	rs12132412	21820042	1	A	G	0.610	0.079	0.005	6.1E-54	trans
126	APOBR	Q0VD83	Apolipoprotein B receptor	rs149271	28506872	16	A	G	0.590	1.123	0.008	0.0E+00	cis
127	APOC1	P02654	Apolipoprotein C-I	rs12124281	62934002	1	G	A	0.648	0.055	0.007	8.4E-14	trans
127	APOC1	P02654	Apolipoprotein C-I	rs55776147	156400808	5	G	A	0.638	0.063	0.007	3.8E-18	trans
127	APOC1	P02654	Apolipoprotein C-I	rs17489373	19855344	8	A	G	0.265	0.082	0.008	4.4E-25	trans
127	APOC1	P02654	Apolipoprotein C-I	rs3764261	56993324	16	A	C	0.325	0.145	0.008	1.8E-82	trans
127	APOC1	P02654	Apolipoprotein C-I	rs77960347	47109955	18	G	A	0.013	0.282	0.031	8.6E-20	trans

127	APOC1	P02654	Apolipoprotein C-I	rs8107974	19388500	19	A	T	0.924	0.103	0.013	2.8E-14	trans
127	APOC1	P02654	Apolipoprotein C-I	rs5112	45430280	19	G	C	0.533	0.345	0.008	0.0E+00	cis
128	APOD	P05090	Apolipoprotein D	rs139828053	195298892	3	T	C	0.969	0.537	0.022	2.8E-130	cis
128	APOD	P05090	Apolipoprotein D	rs894211	19865747	8	T	C	0.265	0.075	0.008	1.2E-18	trans
128	APOD	P05090	Apolipoprotein D	rs10644928	59960726	11	CTT	C	0.388	0.063	0.008	6.1E-16	trans
128	APOD	P05090	Apolipoprotein D	rs1800775	56995236	16	A	C	0.487	0.077	0.008	7.2E-25	trans
128	APOD	P05090	Apolipoprotein D	rs5167	45448465	19	G	T	0.352	0.058	0.008	8.0E-14	trans
129	APOE	P02649	Apolipoprotein E	rs2740488	107661742	9	A	C	0.733	0.058	0.007	1.5E-16	trans
129	APOE	P02649	Apolipoprotein E	rs99780	61596633	11	C	T	0.649	0.068	0.007	7.9E-26	trans
129	APOE	P02649	Apolipoprotein E	rs429358	45411941	19	T	C	0.844	1.012	0.010	0.0E+00	cis
130	APOF	Q13790	Apolipoprotein F	rs112875651	126506694	8	A	G	0.391	0.063	0.008	4.6E-17	trans
130	APOF	Q13790	Apolipoprotein F	rs147233090	44028047	15	C	T	0.976	0.202	0.024	5.5E-17	trans
130	APOF	Q13790	Apolipoprotein F	rs11076175	57006378	16	A	G	0.824	0.077	0.010	1.1E-15	trans
130	APOF	Q13790	Apolipoprotein F	rs3809868	45750596	17	G	A	0.482	0.066	0.007	1.4E-19	trans
130	APOF	Q13790	Apolipoprotein F	rs112001035	66823805	17	A	G	0.061	0.126	0.016	6.3E-16	trans
130	APOF	Q13790	Apolipoprotein F	rs429358	45411941	19	C	T	0.156	0.117	0.010	4.9E-31	trans
131	APOH	P02749	Beta-2-glycoprotein 1	rs1801689	64210580	17	A	C	0.970	1.462	0.022	0.0E+00	cis
132	APOL1	O14791	Apolipoprotein L1	rs5030062	186454180	3	C	A	0.373	0.063	0.007	1.4E-19	trans
132	APOL1	O14791	Apolipoprotein L1	rs4861708	187157233	4	A	G	0.512	0.201	0.007	1.9E-190	trans
132	APOL1	O14791	Apolipoprotein L1	rs1801020	176836532	5	G	A	0.745	0.202	0.008	4.3E-149	trans
132	APOL1	O14791	Apolipoprotein L1	rs9275282	32662974	6	T	C	0.488	0.047	0.007	3.5E-12	trans
132	APOL1	O14791	Apolipoprotein L1	rs550478926	117085186	9	C	G	0.916	0.092	0.013	5.8E-13	trans
132	APOL1	O14791	Apolipoprotein L1	rs9930957	72149923	16	T	C	0.160	0.509	0.010	0.0E+00	trans
132	APOL1	O14791	Apolipoprotein L1	rs1800961	43042364	20	T	C	0.032	0.130	0.019	8.4E-12	trans
132	APOL1	O14791	Apolipoprotein L1	rs136168	36660842	22	G	A	0.187	0.318	0.009	6.9E-286	cis
133	APOM	O95445	Apolipoprotein M	rs1260326	27730940	2	T	C	0.392	0.093	0.007	3.0E-36	trans
133	APOM	O95445	Apolipoprotein M	rs805258	31633552	6	C	T	0.970	0.784	0.022	6.6E-286	cis
133	APOM	O95445	Apolipoprotein M	rs115849089	19912370	8	A	G	0.116	0.085	0.011	4.0E-14	trans
133	APOM	O95445	Apolipoprotein M	rs56332871	96714816	15	A	C	0.270	0.055	0.008	1.6E-11	trans
133	APOM	O95445	Apolipoprotein M	rs247616	56989590	16	T	C	0.325	0.131	0.008	3.4E-63	trans
133	APOM	O95445	Apolipoprotein M	rs112001035	66823805	17	A	G	0.061	0.134	0.015	4.9E-18	trans
133	APOM	O95445	Apolipoprotein M	rs1800961	43042364	20	C	T	0.968	0.244	0.020	6.0E-33	trans
133	APOM	O95445	Apolipoprotein M	rs738408	44324730	22	C	T	0.783	0.061	0.009	2.0E-12	trans
134	APP	P05067	Amyloid-beta precursor protein	rs12086222	156869630	1	C	G	0.111	0.087	0.012	4.1E-13	trans
134	APP	P05067	Amyloid-beta precursor protein	rs13412535	224874874	2	G	A	0.769	0.062	0.009	2.5E-12	trans
134	APP	P05067	Amyloid-beta precursor protein	rs1354034	56849749	3	T	C	0.396	0.101	0.008	2.7E-40	trans
134	APP	P05067	Amyloid-beta precursor protein	rs274555	131722951	5	T	C	0.399	0.066	0.008	1.4E-17	trans
134	APP	P05067	Amyloid-beta precursor protein	rs1917342	71349754	6	A	G	0.697	0.060	0.008	3.4E-13	trans
134	APP	P05067	Amyloid-beta precursor protein	rs6961069	80218961	7	T	C	0.402	0.069	0.008	2.7E-19	trans
134	APP	P05067	Amyloid-beta precursor protein	rs6993770	106581528	8	A	T	0.714	0.084	0.008	2.5E-24	trans
134	APP	P05067	Amyloid-beta precursor protein	rs11794772	99118445	9	G	A	0.741	0.068	0.009	2.4E-15	trans
134	APP	P05067	Amyloid-beta precursor protein	rs7896518	65104500	10	G	A	0.424	0.104	0.008	8.5E-42	trans
134	APP	P05067	Amyloid-beta precursor protein	rs2229498	70856852	10	A	G	0.836	0.113	0.010	5.6E-29	trans
134	APP	P05067	Amyloid-beta precursor protein	rs61978213	70653758	14	A	G	0.042	0.129	0.019	6.1E-12	trans
134	APP	P05067	Amyloid-beta precursor protein	rs59001897	65160392	15	A	T	0.175	0.079	0.010	1.8E-15	trans
134	APP	P05067	Amyloid-beta precursor protein	rs12445050	81870969	16	T	C	0.138	0.112	0.011	5.6E-25	trans
134	APP	P05067	Amyloid-beta precursor protein	rs892090	55539072	19	G	T	0.834	0.170	0.010	4.5E-63	trans
134	APP	P05067	Amyloid-beta precursor protein	rs6081565	19287904	20	A	G	0.353	0.079	0.008	8.2E-24	trans
134	APP	P05067	Amyloid-beta precursor protein	rs8131895	27503527	21	C	A	0.346	0.073	0.008	3.7E-20	cis
134	APP	P05067	Amyloid-beta precursor protein	rs5967171	99957136	X	C	T	0.333	0.051	0.007	7.1E-15	trans
135	APRT	P07741	Adenine phosphoribosyltransferase	rs1354034	56849749	3	C	T	0.604	0.098	0.008	1.7E-38	trans
135	APRT	P07741	Adenine phosphoribosyltransferase	rs854572	94954696	7	G	C	0.527	0.051	0.008	9.4E-12	trans
135	APRT	P07741	Adenine phosphoribosyltransferase	rs74403919	92243978	14	A	T	0.172	0.067	0.010	1.3E-11	trans
135	APRT	P07741	Adenine phosphoribosyltransferase	rs111433410	88869195	16	C	T	0.990	0.351	0.038	4.3E-20	cis
135	APRT	P07741	Adenine phosphoribosyltransferase	rs704	26694861	17	G	A	0.527	0.081	0.007	5.0E-28	trans
136	AREG	P15514	Amphiregulin	rs1691273	75323645	4	C	T	0.308	0.177	0.008	9.1E-105	cis
136	AREG	P15514	Amphiregulin	rs712831	55242782	7	C	T	0.785	0.071	0.009	4.8E-15	trans
136	AREG	P15514	Amphiregulin	rs492602	49206417	19	A	G	0.491	0.069	0.007	4.5E-20	trans
137	ARF6	P62330	ADP-ribosylation factor 6	rs1354034	56849749	3	C	T	0.605	0.059	0.008	1.0E-14	trans
138	ARFIP1	P53367	Arfaptin-1	rs1354034	56849749	3	C	T	0.604	0.059	0.008	7.2E-15	trans
138	ARFIP1	P53367	Arfaptin-1	rs4619875	153701130	4	T	C	0.404	0.159	0.008	2.4E-95	cis
139	ARG1	P05089	Arginase-1	rs2781668	131897278	6	C	T	0.833	0.237	0.010	5.7E-128	cis
139	ARG1	P05089	Arginase-1	rs590856	139844429	6	A	G	0.553	0.094	0.007	1.5E-36	trans
139	ARG1	P05089	Arginase-1	rs6592965	50427982	7	A	G	0.454	0.057	0.007	3.9E-15	trans
139	ARG1	P05089	Arginase-1	rs62108438	13000247	19	C	T	0.392	0.094	0.007	1.7E-36	trans
139	ARG1	P05089	Arginase-1	rs12850852	70339498	X	C	T	0.683	0.081	0.006	2.4E-35	trans
140	ARG2	P78540	Arginase-2, mitochondrial	rs13257521	9031995	8	G	C	0.211	0.084	0.009	2.4E-19	trans
140	ARG2	P78540	Arginase-2, mitochondrial	rs140881784	68132084	14	G	C	0.992	0.404	0.047	4.3E-18	cis
141	ARHGAP1	Q07960	Rho GTPase-activating protein 1	rs6993770	106581528	8	A	T	0.713	0.092	0.008	8.1E-28	trans
141	ARHGAP1	Q07960	Rho GTPase-activating protein 1	rs12245149	65321147	10	C	A	0.512	0.062	0.008	2.2E-16	trans
142	ARHGAP25	P42331	Rho GTPase-activating protein 25	rs13413887	68996668	2	G	A	0.758	0.106	0.009	1.2E-32	cis
142	ARHGAP25	P42331	Rho GTPase-activating protein 25	rs4602861	106590706	8	A	G	0.726	0.067	0.009	5.8E-15	trans
143	ARHGAP45	Q92619	Rho GTPase-activating protein 45	rs1354034	56849749	3	C	T	0.604	0.075	0.008	7.3E-23	trans
143	ARHGAP45	Q92619	Rho GTPase-activating protein 45	rs34593562	1083556	19	A	G	0.551	0.071	0.008	3.9E-21	cis
144	ARHGEF1	Q92888	Rho guanine nucleotide exchange factor 1	rs1354034	56849749	3	C	T	0.604	0.064	0.008	3.9E-17	trans
144	ARHGEF1	Q92888	Rho guanine nucleotide exchange factor 1	rs6993770	106581528	8	A	T	0.713	0.056	0.008	1.6E-11	trans
145	ARHGEF10	O15013	Rho guanine nucleotide exchange factor 10	rs1354034	56849749	3	C	T	0.604	0.074	0.007	2.4E-24	trans
145	ARHGEF10	O15013	Rho guanine nucleotide exchange factor 10	rs11428326	178786875	3	G	GT	0.253	0.066	0.008	1.9E-15	trans
145	ARHGEF10	O15013	Rho guanine nucleotide exchange factor 10	rs342293	106372219	7	C	G	0.540	0.077	0.007	5.7E-27	trans
145	ARHGEF10	O15013	Rho guanine nucleotide exchange factor 10	rs13281104	1811923	8	G	A	0.244	0.317	0.009	2.1E-291	cis
145	ARHGEF10	O15013	Rho guanine nucleotide exchange factor 10	rs6993770	106581528	8	A	T	0.714	0.079	0.008	5.6E-22	trans
146	ARHGEF5	Q12774	Rho guanine nucleotide exchange factor 5	rs17881210	31324448	6	G	A	0.929	0.106	0.014	4.1E-14	trans
147	ARL13B	Q3SXY8	ADP-ribosylation factor-like protein 13B	rs13412535	224874874	2	A	G	0.231	0.068	0.009	9.0E-14	trans
147	ARL13B	Q3SXY8	ADP-ribosylation factor-like protein 13B	rs1354034	56849749	3	C	T	0.604	0.060	0.008	8.5E-15	trans
148	ARL2BP	Q9Y2Y0	ADP-ribosylation factor-like protein 2-binding protein	rs1354034	56849749	3	C	T	0.604	0.110	0.008	2.1E-46	trans
148	ARL2BP	Q9Y2Y0	ADP-ribosylation factor-like protein 2-binding protein	rs72767003	127913157	9	T	C	0.146	0.075	0.011	2.7E-12	trans
148	ARL2BP	Q9Y2Y0	ADP-ribosylation factor-like protein 2-binding protein	rs7198865	57283730	16	G	A	0.965	0.746	0.021	7.7E-281	cis
149	ARSA	P15289	Arylsulfatase A	rs114165349	27021913	1	C	G	0.023	0.168	0.022	6.9E-15	trans
149	ARSA	P15289	Arylsulfatase A	rs687339	135932359	3	T	C	0.771	0.063	0.008	3.6E-16	trans
149	ARSA	P15289	Arylsulfatase A	rs145078947	93652974	14	T	G	0.003	0.634	0.064	3.4E-23	trans
149	ARSA	P15289	Arylsulfatase A	rs58542926	19379549	19	T	C	0.076	0.184	0.012	2.9E-50	trans
149	ARSA	P15289	Arylsulfatase A	rs429358	45411941	19	T	C	0.845	0.062	0.009	5.4E-12	trans
149	ARSA	P15289	Arylsulfatase A	rs873697	51064169	22	A	G	0.045	1.499	0.018	0.0E+00	cis
150	ARSB	P15848	Arylsulfatase B	rs1065757	78181477	5	C	T	0.560	0.294	0.008	0.0E+00	cis
150	ARSB	P15848	Arylsulfatase B	rs1293303	11720227	8	C	G	0.446	0.073	0.007	4.8E-23	trans
150	ARSB	P15848	Arylsulfatase B	rs6993770	106581528	8	A	T	0.714	0.086	0.008	1.9E-26	trans
150	ARSB	P15848	Arylsulfatase B	rs10968020	27628440	9	T	C	0.736	0.067	0.008	9.7E-16	trans
150	ARSB	P15848	Arylsulfatase B	rs7896518	65104500	10	G	A	0.424	0.083	0.007	3.2E-28	trans
150	ARSB	P15848	Arylsulfatase B	rs145078947	93652974	14	T	G	0.003	0.590	0.072	2.5E-16	trans
150	ARSB	P15848	Arylsulfatase B	rs8072896	55082832	17	T	G	0.515	0.051	0.007	8.8E-12	trans
150	ARSB	P15848	Arylsulfatase B	rs892090	55539072	19	G	T	0.834	0.099	0.010	1.9E-23	trans
151	ART3	Q13508	Ecto-ADP-ribosyltransferase 3	rs150816167	179571862	1	C	T	0.042	0.151	0.017	4.8E-19	trans
151	ART3	Q13508	Ecto-ADP-ribosyltransferase 3	rs1868164	134369420	3	G	A	0.369	0.061	0.007	2.0E-18	trans
151	ART3	Q13508	Ecto-ADP-ribosyltransferase 3	rs11649031	427516	16	T	C	0.413	0.053	0.007	4.7E-15	trans
151	ART3	Q13508	Ecto-ADP-ribosyltransferase 3	rs12934455	20357281	16	C	T	0.834	0.080	0.009	6.7E-19	trans
152	ART5	Q96L15	Ecto-ADP-ribosyltransferase 5	rs75902876	3663021	11	A	G	0.081	0.350	0.015	1.4E-115	cis
153	ARTN	Q5T4W7	Artemin	rs112597211	106369433	14	C	T	0.136	0.092	0.011	5.7E-16	trans
154	ASAH1	Q13510	Acid ceramidase	rs2472205	17924739	8	A	T	0.959	1.099	0.019	0.0E+00	cis
154	ASAH1	Q13510	Acid ceramidase	rs4734879	106583124	8	A	G	0.726	0.062	0.008	3.0E-13	trans
154	ASAH1	Q13510	Acid ceramidase	rs2127868	65794327	14	A	G	0.795	0.078	0.009	8.8E-19	trans
154	ASAH1	Q13510	Acid ceramidase	rs72701845	93217023	14	G	A	0.962	0.214	0.019	8.9E-29	trans
155	ASAH2	Q9NR71	Neutral ceramidase	rs12032634	26007568	1	T	A	0.484	0.081	0.007	1.4E-33	trans
155	ASAH2	Q9NR71	Neutral ceramidase	rs7518301	200260339	1	G	T	0.212	0.056	0.008	9.7E-12	trans
155	ASAH2	Q9NR71	Neutral ceramidase	rs3732356	119529113	3	G	T	0.065	0.112	0.014	3.4E-16	trans
155	ASAH2	Q9NR71	Neutral ceramidase	rs890793	153573938	5	C	T	0.618	0.048	0.007	2.2E-12	trans
155	ASAH2	Q9NR71	Neutral ceramidase	rs804267	11629241	8	G	A	0.317	0.054	0.007	7.4E-14	trans
155	ASAH2	Q9NR71	Neutral ceramidase	rs12056768	116988527	8	G	T	0.583	0.065	0.007	6.8E-22	trans
155	ASAH2	Q9NR71	Neutral ceramidase	rs56278466	17875857	10	G	T	0.661	0.068	0.008	6.8E-18	trans
155	ASAH2	Q9NR71	Neutral ceramidase	rs2842126	52018482	10	G	A	0.805	0.691	0.009	0.0E+00	cis
155	ASAH2	Q9NR71	Neutral ceramidase	rs1519125	16244588	11	C	G	0.386	0.053	0.007	1.1E-14	trans
155	ASAH2	Q9NR71	Neutral ceramidase	rs28929474	94844947	14	T	C	0.021	0.162	0.023	3.4E-12	trans
155	ASAH2	Q9NR71	Neutral ceramidase	rs77542162	67081278	17	G	A	0.023	0.290	0.022	5.5E-39	trans
156	ASGR1	P07306	Asialoglycoprotein receptor 1	rs12740374	109817590	1	T	G	0.221	0.127	0.009	2.5E-46	trans
156	ASGR1	P07306	Asialoglycoprotein receptor 1	rs11405616	141510261	5	CA	C	0.606	0.053	0.008	5.2E-12	trans
156	ASGR1	P07306	Asialoglycoprotein receptor 1	rs7012814	9173358	8	G	A	0.528	0.054	0.007	5.1E-13	trans
156	ASGR1	P07306	Asialoglycoprotein receptor 1	rs2622935	117010758	11	A	G	0.192	0.141	0.009	4.2E-51	trans
156	ASGR1	P07306	Asialoglycoprotein receptor 1	rs28929474	94844947	14	T	C	0.021	0.313	0.026	8.8E-34	trans
156	ASGR1	P07306	Asialoglycoprotein receptor 1	rs55714927	7080316	17	C	T	0.805	0.193	0.009	5.5E-94	cis
156	ASGR1	P07306	Asialoglycoprotein receptor 1	rs1801689	64210580	17	C	A	0.030	0.190	0.022	2.3E-18	trans
157	ASGR2	P07307	Asialoglycoprotein receptor 2	rs61747728	179526214	1	T	C	0.038	0.128	0.019	6.4E-12	trans
157	ASGR2	P07307	Asialoglycoprotein receptor 2	rs529541	196719716	1	G	A	0.173	0.088	0.010	1.8E-20	trans
157	ASGR2	P07307	Asialoglycoprotein receptor 2	rs12621059	47149658	2	C	T	0.294	0.079	0.008	1.5E-23	trans
157	ASGR2	P07307	Asialoglycoprotein receptor 2	rs35457250	186338564	3	T	C	0.009	0.346	0.038	2.5E-19	trans
157	ASGR2	P07307	Asialoglycoprotein receptor 2	rs1611185	29768344	6	T	C	0.583	0.050	0.007	6.5E-12	trans
157	ASGR2	P07307	Asialoglycoprotein receptor 2	rs4240624	9184231	8	A	G	0.908	0.107	0.012	1.1E-17	trans
157	ASGR2	P07307	Asialoglycoprotein receptor 2	rs28929474	94844947	14	T	C	0.021	0.740	0.025	3.1E-188	trans
157	ASGR2	P07307	Asialoglycoprotein receptor 2	rs2304978	7012079	17	T	C	0.290	0.325	0.008	0.0E+00	cis
157	ASGR2	P07307	Asialoglycoprotein receptor 2	rs1801689	64210580	17	C	A	0.030	0.841	0.022	0.0E+00	trans
157	ASGR2	P07307	Asialoglycoprotein receptor 2	rs2733739	36045772	19	A	G	0.372	0.052	0.007	1.6E-12	trans
158	ASPN	Q9BXN1	Asporin	rs74617384	160997118	6	A	T	0.919	0.114	0.013	4.7E-18	trans
158	ASPN	Q9BXN1	Asporin	rs2295941	95036987	9	A	G	0.673	0.257	0.008	6.3E-238	cis
158	ASPN	Q9BXN1	Asporin	rs67852477	70856963	10	G	C	0.010	0.243	0.036	1.5E-11	trans
158	ASPN	Q9BXN1	Asporin	rs35887873	126219396	11	C	T	0.228	0.076	0.009	5.3E-19	trans

158	ASPN	Q9BXN1	Asporin	rs1303	94844843	14	G	T	0.255	0.083	0.008	1.9E-23	trans
158	ASPN	Q9BXN1	Asporin	rs186021206	7069412	17	A	G	0.006	0.617	0.050	1.8E-35	trans
159	ASPSCR1	Q9BZE9	Tether containing UBX domain for GLUT4	rs1354034	56849749	3	C	T	0.604	0.072	0.007	4.1E-22	trans
159	ASPSCR1	Q9BZE9	Tether containing UBX domain for GLUT4	rs342293	106372219	7	C	G	0.540	0.057	0.007	8.3E-15	trans
159	ASPSCR1	Q9BZE9	Tether containing UBX domain for GLUT4	rs8074498	79954544	17	T	A	0.419	0.213	0.008	1.1E-174	cis
160	ASRGL1	Q7L266	Isoaspartyl peptidase/L-asparaginase	rs7896518	65104500	10	G	A	0.424	0.069	0.007	6.7E-21	trans
160	ASRGL1	Q7L266	Isoaspartyl peptidase/L-asparaginase	rs1043785	62160723	11	C	T	0.715	0.372	0.008	0.0E+00	cis
160	ASRGL1	Q7L266	Isoaspartyl peptidase/L-asparaginase	rs11671654	12014355	19	G	C	0.495	0.288	0.007	0.0E+00	trans
161	ASS1	P00966	Argininosuccinate synthase	rs1047891	211540507	2	A	C	0.313	0.078	0.008	5.4E-23	trans
161	ASS1	P00966	Argininosuccinate synthase	rs71633359	88183820	4	T	C	0.679	0.055	0.008	9.1E-12	trans
161	ASS1	P00966	Argininosuccinate synthase	rs112875651	126506694	8	G	A	0.608	0.056	0.008	1.3E-13	trans
161	ASS1	P00966	Argininosuccinate synthase	rs10883451	101924418	10	T	C	0.498	0.057	0.007	3.2E-15	trans
161	ASS1	P00966	Argininosuccinate synthase	rs7117339	93870338	11	C	T	0.883	0.084	0.011	1.2E-13	trans
161	ASS1	P00966	Argininosuccinate synthase	rs3747207	44324855	22	A	G	0.216	0.143	0.009	2.1E-58	trans
162	ATF2	P15336	Cyclic AMP-dependent transcription factor ATF-2	rs58534292	106483584	14	T	C	0.350	0.070	0.008	2.4E-17	trans
162	ATF2	P15336	Cyclic AMP-dependent transcription factor ATF-2	rs2857339	106382687	14	T	C	0.498	0.135	0.009	2.2E-51	trans
163	ATG16L1	Q676U5	Autophagy-related protein 16-1	rs1354034	56849749	3	C	T	0.605	0.052	0.008	6.5E-12	trans
163	ATG16L1	Q676U5	Autophagy-related protein 16-1	rs342293	106372219	7	C	G	0.540	0.058	0.007	1.1E-14	trans
163	ATG16L1	Q676U5	Autophagy-related protein 16-1	rs7896518	65104500	10	G	A	0.424	0.056	0.008	3.0E-13	trans
164	ATG4A	Q8WYN0	Cysteine protease ATG4A	rs1354034	56849749	3	C	T	0.604	0.053	0.008	1.7E-12	trans
164	ATG4A	Q8WYN0	Cysteine protease ATG4A	rs749679	88570816	16	G	A	0.297	0.064	0.008	3.4E-15	trans
164	ATG4A	Q8WYN0	Cysteine protease ATG4A	rs8887	4502201	19	C	T	0.574	0.064	0.008	3.9E-17	trans
164	ATG4A	Q8WYN0	Cysteine protease ATG4A	rs5973846	107382374	X	C	T	0.464	0.072	0.006	1.8E-31	cis
165	ATOX1	O00244	Copper transport protein ATOX1	rs1354034	56849749	3	C	T	0.604	0.060	0.008	5.8E-15	trans
165	ATOX1	O00244	Copper transport protein ATOX1	rs1050152	131676320	5	C	T	0.566	0.052	0.008	5.3E-12	trans
165	ATOX1	O00244	Copper transport protein ATOX1	rs58534292	106483584	14	T	C	0.351	0.110	0.008	1.1E-43	trans
165	ATOX1	O00244	Copper transport protein ATOX1	rs73365419	106481112	14	T	C	0.384	0.117	0.008	6.5E-49	trans
165	ATOX1	O00244	Copper transport protein ATOX1	rs892090	55539072	19	G	T	0.834	0.079	0.010	6.0E-15	trans
166	ATP5IF1	Q9UII2	ATPase inhibitor, mitochondrial	rs510379	28535233	1	C	T	0.360	0.112	0.008	1.2E-45	cis
166	ATP5IF1	Q9UII2	ATPase inhibitor, mitochondrial	rs1354034	56849749	3	C	T	0.604	0.061	0.008	1.2E-15	trans
166	ATP5IF1	Q9UII2	ATPase inhibitor, mitochondrial	rs10733789	64948684	10	C	T	0.313	0.060	0.008	2.1E-13	trans
167	ATP6AP2	O75787	Renin receptor	rs76415507	32492589	6	T	G	0.440	0.060	0.008	1.3E-12	trans
168	ATP6V1D	Q9Y5K8	V-type proton ATPase subunit D	rs189049184	32487474	6	G	A	0.176	0.091	0.010	1.8E-18	trans
169	ATP6V1F	Q16864	V-type proton ATPase subunit F	rs1354034	56849749	3	C	T	0.603	0.063	0.008	2.7E-16	trans
170	ATP6V1G1	O75348	V-type proton ATPase subunit G 1	rs1354034	56849749	3	C	T	0.605	0.052	0.008	1.5E-11	trans
171	ATP6V1G2	O95670	V-type proton ATPase subunit G 2	rs2239705	31513402	6	A	G	0.157	0.204	0.011	8.2E-82	cis
172	ATRAID	Q6UW56	All-trans retinoic acid-induced differentiation factor	rs61747728	179526214	1	T	C	0.038	0.155	0.019	3.2E-16	trans
172	ATRAID	Q6UW56	All-trans retinoic acid-induced differentiation factor	rs78444298	184672098	1	A	G	0.019	0.434	0.027	5.1E-60	trans
172	ATRAID	Q6UW56	All-trans retinoic acid-induced differentiation factor	rs1275530	27432547	2	A	G	0.293	0.279	0.008	6.7E-259	cis
173	ATRN	O75882	Attractin, Isoform 2	rs9427104	154589232	1	T	C	0.480	0.047	0.007	2.5E-12	trans
173	ATRN	O75882	Attractin, Isoform 2	rs1260326	27730940	2	T	C	0.392	0.060	0.007	6.2E-18	trans
173	ATRN	O75882	Attractin, Isoform 2	rs11845606	24895010	14	C	G	0.939	0.106	0.014	9.3E-14	trans
173	ATRN	O75882	Attractin, Isoform 2	rs78971233	3543542	20	T	A	0.947	1.293	0.016	0.0E+00	cis
174	ATXN10	Q9UBB4	Ataxin-10	rs1354034	56849749	3	C	T	0.604	0.074	0.008	1.1E-21	trans
174	ATXN10	Q9UBB4	Ataxin-10	rs342298	106373646	7	C	T	0.546	0.054	0.008	1.4E-12	trans
174	ATXN10	Q9UBB4	Ataxin-10	rs6993770	106581528	8	A	T	0.713	0.064	0.008	3.1E-14	trans
174	ATXN10	Q9UBB4	Ataxin-10	rs7080386	65048306	10	A	C	0.412	0.056	0.008	4.6E-13	trans
174	ATXN10	Q9UBB4	Ataxin-10	rs138153	46107078	22	G	C	0.293	0.106	0.008	9.0E-37	cis
175	ATXN2L	Q8WWM7	Ataxin-2-like protein	rs1354034	56849749	3	C	T	0.604	0.073	0.008	1.2E-21	trans
176	ATXN3	P54252	Ataxin-3	rs1354034	56849749	3	C	T	0.604	0.051	0.007	3.1E-12	trans
176	ATXN3	P54252	Ataxin-3	rs55656743	92540054	14	A	G	0.755	0.307	0.008	1.4E-285	cis
177	AXIN1	O15169	Axin-1	rs1354034	56849749	3	C	T	0.604	0.057	0.008	4.1E-14	trans
177	AXIN1	O15169	Axin-1	rs6993770	106581528	8	A	T	0.713	0.062	0.008	1.7E-14	trans
178	AXL	P30530	Tyrosine-protein kinase receptor UFO	rs79412885	9241839	1	G	A	0.926	0.164	0.014	1.2E-30	trans
178	AXL	P30530	Tyrosine-protein kinase receptor UFO	rs2470855	98704133	3	G	A	0.426	0.054	0.007	2.6E-13	trans
178	AXL	P30530	Tyrosine-protein kinase receptor UFO	rs7140110	114544024	13	C	T	0.296	0.127	0.008	4.6E-56	trans
178	AXL	P30530	Tyrosine-protein kinase receptor UFO	rs186021206	7069412	17	A	G	0.006	0.991	0.050	2.0E-86	trans
178	AXL	P30530	Tyrosine-protein kinase receptor UFO	rs7224668	79235788	17	T	C	0.443	0.053	0.007	8.1E-13	trans
178	AXL	P30530	Tyrosine-protein kinase receptor UFO	rs66841352	41739180	19	G	C	0.597	0.174	0.008	2.0E-118	cis
179	AZI2	Q9H6S1	5-azacytidine-induced protein 2	rs1354034	56849749	3	C	T	0.604	0.085	0.008	6.3E-30	trans
180	AZU1	P20160	Azurocidin	rs2210918	157692985	1	C	T	0.471	0.051	0.007	8.4E-12	trans
180	AZU1	P20160	Azurocidin	rs2951842	6826521	8	C	G	0.496	0.062	0.008	6.3E-16	trans
180	AZU1	P20160	Azurocidin	rs744781	144579215	8	G	C	0.526	0.059	0.007	2.5E-15	trans
181	B2M	P61769	Beta-2-microglobulin	rs74439742	57059427	16	C	T	0.828	0.087	0.009	4.1E-20	trans
182	B3GNT7	Q8NFL0	UDP-GlcNAc:betaGal beta-1,3-N-acetylglucosaminyltransferase 7	rs583104	109821307	1	T	G	0.773	0.069	0.008	2.2E-19	trans
182	B3GNT7	Q8NFL0	UDP-GlcNAc:betaGal beta-1,3-N-acetylglucosaminyltransferase 7	rs2290130	232263127	2	G	A	0.770	0.747	0.009	0.0E+00	cis
182	B3GNT7	Q8NFL0	UDP-GlcNAc:betaGal beta-1,3-N-acetylglucosaminyltransferase 7	rs13107325	103188709	4	T	C	0.075	0.114	0.012	2.5E-20	trans
182	B3GNT7	Q8NFL0	UDP-GlcNAc:betaGal beta-1,3-N-acetylglucosaminyltransferase 7	rs6908045	10534503	6	C	T	0.471	0.053	0.006	5.5E-16	trans
182	B3GNT7	Q8NFL0	UDP-GlcNAc:betaGal beta-1,3-N-acetylglucosaminyltransferase 7	rs111630162	33071267	6	T	C	0.992	0.441	0.037	5.6E-33	trans
182	B3GNT7	Q8NFL0	UDP-GlcNAc:betaGal beta-1,3-N-acetylglucosaminyltransferase 7	rs79578725	73002455	7	A	G	0.042	0.170	0.016	3.8E-25	trans
182	B3GNT7	Q8NFL0	UDP-GlcNAc:betaGal beta-1,3-N-acetylglucosaminyltransferase 7	rs7865362	33117965	9	C	T	0.638	0.070	0.007	1.5E-24	trans
182	B3GNT7	Q8NFL0	UDP-GlcNAc:betaGal beta-1,3-N-acetylglucosaminyltransferase 7	rs56278466	17875857	10	G	T	0.661	0.096	0.007	2.2E-44	trans
182	B3GNT7	Q8NFL0	UDP-GlcNAc:betaGal beta-1,3-N-acetylglucosaminyltransferase 7	rs1955512	33175822	14	A	G	0.572	0.148	0.007	2.0E-108	trans
182	B3GNT7	Q8NFL0	UDP-GlcNAc:betaGal beta-1,3-N-acetylglucosaminyltransferase 7	rs12881755	65801811	14	A	G	0.315	0.050	0.007	7.6E-13	trans
182	B3GNT7	Q8NFL0	UDP-GlcNAc:betaGal beta-1,3-N-acetylglucosaminyltransferase 7	rs201796794	50883037	15	A	AG	0.750	0.064	0.007	8.5E-18	trans
182	B3GNT7	Q8NFL0	UDP-GlcNAc:betaGal beta-1,3-N-acetylglucosaminyltransferase 7	rs200489612	7106378	17	A	G	0.005	0.413	0.048	1.5E-17	trans
182	B3GNT7	Q8NFL0	UDP-GlcNAc:betaGal beta-1,3-N-acetylglucosaminyltransferase 7	rs34321000	12713088	19	C	T	0.329	0.054	0.007	3.4E-15	trans
182	B3GNT7	Q8NFL0	UDP-GlcNAc:betaGal beta-1,3-N-acetylglucosaminyltransferase 7	rs516246	49206172	19	C	T	0.491	0.044	0.007	8.6E-12	trans
183	B4GALT1	P15291	Beta-1,4-galactosyltransferase 1	rs61747728	179526214	1	T	C	0.038	0.164	0.019	3.2E-18	trans
183	B4GALT1	P15291	Beta-1,4-galactosyltransferase 1	rs56249455	32644378	6	T	A	0.886	0.097	0.012	1.8E-16	trans
183	B4GALT1	P15291	Beta-1,4-galactosyltransferase 1	rs7019909	33113322	9	T	C	0.102	0.332	0.012	3.4E-167	cis
183	B4GALT1	P15291	Beta-1,4-galactosyltransferase 1	rs7247412	52313868	19	T	C	0.733	0.323	0.008	0.0E+00	trans
184	B4GAT1	O43505	Beta-1,4-glucuronyltransferase 1	rs10168551	168597986	2	T	C	0.795	0.060	0.009	9.8E-12	trans
184	B4GAT1	O43505	Beta-1,4-glucuronyltransferase 1	rs6800514	58358717	3	T	C	0.371	0.057	0.007	1.6E-14	trans
184	B4GAT1	O43505	Beta-1,4-glucuronyltransferase 1	rs9274678	32636646	6	A	G	0.847	0.094	0.010	1.4E-21	trans
184	B4GAT1	O43505	Beta-1,4-glucuronyltransferase 1	rs10758194	33157419	9	G	T	0.579	0.078	0.007	2.8E-27	trans
184	B4GAT1	O43505	Beta-1,4-glucuronyltransferase 1	rs56278466	17875857	10	G	T	0.661	0.100	0.008	2.0E-40	trans
184	B4GAT1	O43505	Beta-1,4-glucuronyltransferase 1	rs10896113	66117111	11	C	T	0.778	0.316	0.009	3.4E-287	cis
184	B4GAT1	O43505	Beta-1,4-glucuronyltransferase 1	rs74611001	94220519	11	C	A	0.976	0.205	0.024	1.4E-17	trans
184	B4GAT1	O43505	Beta-1,4-glucuronyltransferase 1	rs28873836	52314655	19	G	C	0.734	0.198	0.008	1.3E-130	trans
184	B4GAT1	O43505	Beta-1,4-glucuronyltransferase 1	rs12975366	54759361	19	T	C	0.605	0.054	0.007	2.5E-13	trans
185	BACH1	O14867	Transcription regulator protein BACH1	rs342293	106372219	7	C	G	0.541	0.050	0.007	1.5E-11	trans
185	BACH1	O14867	Transcription regulator protein BACH1	rs6993770	106581528	8	A	T	0.713	0.061	0.008	6.3E-14	trans
186	BAG3	O95817	BAG family molecular chaperone regulator 3	rs710446	186459927	3	T	C	0.591	0.066	0.008	6.8E-18	trans
186	BAG3	O95817	BAG family molecular chaperone regulator 3	rs3733402	187158034	4	G	A	0.488	0.091	0.008	3.2E-33	trans
186	BAG3	O95817	BAG family molecular chaperone regulator 3	rs2545801	176841339	5	T	C	0.256	0.069	0.009	1.0E-15	trans
186	BAG3	O95817	BAG family molecular chaperone regulator 3	rs35434411	121429394	10	A	G	0.031	0.178	0.022	2.1E-16	cis
186	BAG3	O95817	BAG family molecular chaperone regulator 3	rs55714647	35546307	19	G	GA	0.066	0.107	0.015	3.6E-12	trans
187	BAG4	O95429	BAG family molecular chaperone regulator 4	rs1354034	56849749	3	C	T	0.604	0.065	0.008	3.6E-17	trans
187	BAG4	O95429	BAG family molecular chaperone regulator 4	rs4632248	54324995	19	T	G	0.210	0.079	0.009	2.8E-17	trans
188	BAIAP2	Q9UQB8	Brain-specific angiogenesis inhibitor 1-associated protein 2	rs28456	61589481	11	G	A	0.315	0.055	0.008	1.3E-11	trans
188	BAIAP2	Q9UQB8	Brain-specific angiogenesis inhibitor 1-associated protein 2	rs147121761	103570134	14	T	C	0.282	0.078	0.009	1.9E-19	trans
188	BAIAP2	Q9UQB8	Brain-specific angiogenesis inhibitor 1-associated protein 2	rs9989483	78971536	17	T	C	0.173	0.095	0.010	2.2E-21	cis
188	BAIAP2	Q9UQB8	Brain-specific angiogenesis inhibitor 1-associated protein 2	rs189448562	3705115	20	C	G	0.011	0.406	0.037	9.3E-29	trans
189	BAMBI	Q13145	BMP and activin membrane-bound inhibitor homolog	rs1354034	56849749	3	T	C	0.396	0.064	0.008	1.3E-16	trans
190	BANK1	Q8NDB2	B-cell scaffold protein with ankyrin repeats	rs1354034	56849749	3	C	T	0.604	0.073	0.008	2.8E-22	trans
190	BANK1	Q8NDB2	B-cell scaffold protein with ankyrin repeats	rs3113676	102965043	4	C	T	0.985	0.478	0.030	6.6E-56	cis
190	BANK1	Q8NDB2	B-cell scaffold protein with ankyrin repeats	rs342293	106372219	7	C	G	0.541	0.057	0.007	1.6E-14	trans
191	BAP18	Q8IXM2	Chromatin complexes subunit BAP18	rs1354034	56849749	3	C	T	0.604	0.083	0.008	5.0E-28	trans
191	BAP18	Q8IXM2	Chromatin complexes subunit BAP18	rs76483802	32454913	6	A	G	0.661	0.075	0.008	3.7E-20	trans
191	BAP18	Q8IXM2	Chromatin complexes subunit BAP18	rs8065014	6888605	17	G	A	0.921	0.112	0.014	3.2E-16	cis
192	BAX	Q07812	Apoptosis regulator BAX	rs1354034	56849749	3	C	T	0.604	0.059	0.008	2.8E-14	trans
192	BAX	Q07812	Apoptosis regulator BAX	rs12245367	65321464	10	C	T	0.519	0.052	0.008	1.2E-11	trans
193	BCAM	P50895	Basal cell adhesion molecule	rs539657009	161628456	1	T	C	0.130	0.078	0.011	5.6E-13	trans
193	BCAM	P50895	Basal cell adhesion molecule	rs4681846	58332543	3	T	C	0.371	0.070	0.007	3.7E-22	trans
193	BCAM	P50895	Basal cell adhesion molecule	rs11100831	145059643	4	C	T	0.441	0.124	0.007	4.3E-67	trans
193	BCAM	P50895	Basal cell adhesion molecule	rs73080552	39435433	5	G	A	0.889	0.081	0.011	4.9E-13	trans
193	BCAM	P50895	Basal cell adhesion molecule	rs12522289	177640864	5	A	G	0.920	0.091	0.013	4.7E-12	trans
193	BCAM	P50895	Basal cell adhesion molecule	rs9270591	32561424	6	C	T	0.470	0.065	0.007	2.3E-20	trans
193	BCAM	P50895	Basal cell adhesion molecule	rs6592965	50427982	7	G	A	0.546	0.083	0.007	4.9E-32	trans
193	BCAM	P50895	Basal cell adhesion molecule	rs8176672	136142185	9	T	C	0.061	0.663	0.015	0.0E+00	trans
193	BCAM	P50895	Basal cell adhesion molecule	rs56278466	17875857	10	G	T	0.661	0.159	0.007	1.6E-101	trans
193	BCAM	P50895	Basal cell adhesion molecule	rs186021206	7069412	17	A	G	0.006	0.378	0.048	3.3E-15	trans
193	BCAM	P50895	Basal cell adhesion molecule	rs11085824	13001547	19	G	A	0.378	0.117	0.007	2.8E-56	trans
193	BCAM	P50895	Basal cell adhesion molecule	rs28399654	45316588	19	G	A	0.967	0.654	0.020	3.3E-235	cis
193	BCAM	P50895	Basal cell adhesion molecule	rs2638282	49213833	19	A	G	0.534	0.051	0.007	1.5E-12	trans
193	BCAM	P50895	Basal cell adhesion molecule	rs2410405	42580363	21	T	C	0.378	0.111	0.007	1.1E-52	trans
194	BCAN	Q96GW7	Brevican core protein	rs2365715	156615114	1	A	G	0.610	0.231	0.008	1.7E-191	cis
194	BCAN	Q96GW7	Brevican core protein	rs13086758	24422109	3	T	A	0.786	0.066	0.009	1.3E-13	trans
194	BCAN	Q96GW7	Brevican core protein	rs2071592	31515340	6	T	A	0.653	0.061	0.008	1.3E-15	trans
194	BCAN	Q96GW7	Brevican core protein	rs73086586	20394948	7	T	C	0.061	0.154	0.015	3.6E-24	trans
194	BCAN	Q96GW7	Brevican core protein	rs61738649	73010754	7	C	T	0.929	0.130	0.014	2.7E-20	trans
194	BCAN	Q96GW7	Brevican core protein	rs56278466	17875857	10	G	T	0.661	0.128	0.008	2.1E-62	trans
195	BCAT1	P54687	Branched-chain-amino-acid aminotransferase, cytosolic	rs111466273	52539677	3	G	A	0.006	0.479	0.047	3.2E-24	trans
195	BCAT1	P54687	Branched-chain-amino-acid aminotransferase, cytosolic	rs1354034	56849749	3	C	T	0.604	0.055	0.007	7.2E-14	trans
195	BCAT1	P54687	Branched-chain-amino-acid aminotransferase, cytosolic	rs6993770	106581528	8	A	T	0.713	0.067	0.008	2.0E-17	trans
196	BCHE	P06276	Cholinesterase	rs70620	196704997	1	A	G	0.172	0.067	0.009	6.5E-14	trans
196	BCHE	P06276	Cholinesterase	rs1260326	27730940	2	T	C	0.392	0.066	0.007	5.1E-21	trans
196	BCHE	P06276	Cholinesterase	rs11687186	204319839	2	G	C	0.552	0.161	0.007	5.3E-121	trans
196	BCHE	P06276	Cholinesterase	rs111696008	217487709	2	A	G	0.017	0.650	0.027	8.3E-133	trans
196	BCHE	P06276	Cholinesterase	rs887829	234668570	2	C	T	0.686	0.075	0.007	1.2E-24	trans
196	BCHE	P06276	Cholinesterase	rs73165060	165481418	3	C	A	0.790	0.585	0.009	0.0E+00	cis
196	BCHE	P06276	Cholinesterase	rs183545662	140852055	5	T	C	0.006	0.413	0.045	6.4E-20	trans

196	BCHE	P06276	Cholinesterase	rs2516448	31390410	6	C	T	0.494	0.057	0.007	3.2E-17	trans
196	BCHE	P06276	Cholinesterase	rs10502	90039670	6	G	C	0.619	0.053	0.007	2.3E-14	trans
196	BCHE	P06276	Cholinesterase	rs72941804	105855659	6	G	A	0.049	0.118	0.016	4.9E-14	trans
196	BCHE	P06276	Cholinesterase	rs35493868	73039406	7	C	G	0.799	0.078	0.008	2.0E-20	trans
196	BCHE	P06276	Cholinesterase	rs4841132	9183596	8	G	A	0.908	0.155	0.012	6.5E-40	trans
196	BCHE	P06276	Cholinesterase	rs7896518	65104500	10	A	G	0.576	0.065	0.007	5.0E-21	trans
196	BCHE	P06276	Cholinesterase	rs174568	61593816	11	T	C	0.344	0.070	0.007	7.0E-23	trans
197	BCL2	P10415	Apoptosis regulator Bcl-2	rs72700845	150568749	1	T	C	0.017	0.225	0.029	8.6E-15	trans
197	BCL2	P10415	Apoptosis regulator Bcl-2	rs445925	45415640	19	A	G	0.109	0.084	0.012	2.4E-12	trans
198	BCL2L1	Q07817	Bcl-2-like protein 1	rs1354034	56849749	3	C	T	0.604	0.060	0.008	3.6E-15	trans
198	BCL2L1	Q07817	Bcl-2-like protein 1	rs6993770	106581528	8	A	T	0.713	0.065	0.008	6.3E-15	trans
199	BCL2L11	O43521	Bcl-2-like protein 11, Isoform BimL	rs13412535	224874874	2	G	A	0.769	0.064	0.009	2.1E-13	trans
199	BCL2L11	O43521	Bcl-2-like protein 11, Isoform BimL	rs1354034	56849749	3	T	C	0.396	0.054	0.007	4.5E-13	trans
199	BCL2L11	O43521	Bcl-2-like protein 11, Isoform BimL	rs5030062	186454180	3	A	C	0.627	0.157	0.008	1.2E-94	trans
199	BCL2L11	O43521	Bcl-2-like protein 11, Isoform BimL	rs66530140	187161211	4	T	C	0.486	0.293	0.007	0.0E+00	trans
199	BCL2L11	O43521	Bcl-2-like protein 11, Isoform BimL	rs1801020	176836532	5	A	G	0.255	0.154	0.008	6.9E-76	trans
199	BCL2L11	O43521	Bcl-2-like protein 11, Isoform BimL	rs1050328	31238155	6	G	A	0.734	0.056	0.008	1.2E-11	trans
199	BCL2L11	O43521	Bcl-2-like protein 11, Isoform BimL	rs6993770	106581528	8	A	T	0.713	0.104	0.008	3.5E-38	trans
199	BCL2L11	O43521	Bcl-2-like protein 11, Isoform BimL	rs10761737	65052205	10	C	T	0.413	0.081	0.007	4.9E-28	trans
199	BCL2L11	O43521	Bcl-2-like protein 11, Isoform BimL	rs71354995	38791841	19	A	G	0.744	0.212	0.008	3.8E-142	trans
199	BCL2L11	O43521	Bcl-2-like protein 11, Isoform BimL	rs892090	55539072	19	G	T	0.834	0.108	0.010	5.8E-28	trans
200	BCL2L15	Q5TBC7	Bcl-2-like protein 15	rs1230715	114428086	1	C	A	0.673	0.177	0.008	1.6E-108	cis
200	BCL2L15	Q5TBC7	Bcl-2-like protein 15	rs35440906	61646351	8	CA	C	0.186	0.087	0.010	1.4E-19	trans
200	BCL2L15	Q5TBC7	Bcl-2-like protein 15	rs34436714	54327313	19	A	C	0.209	0.090	0.009	8.0E-23	trans
201	BCR	P11274	Breakpoint cluster region protein	rs149005797	23485100	22	T	C	0.996	0.541	0.066	3.5E-16	cis
202	BDNF	P23560	Brain-derived neurotrophic factor	rs6671171	156871318	1	A	G	0.111	0.084	0.012	1.1E-12	trans
202	BDNF	P23560	Brain-derived neurotrophic factor	rs13412535	224874874	2	G	A	0.769	0.061	0.009	4.2E-12	trans
202	BDNF	P23560	Brain-derived neurotrophic factor	rs1354034	56849749	3	T	C	0.395	0.114	0.008	2.9E-51	trans
202	BDNF	P23560	Brain-derived neurotrophic factor	rs2278668	122835232	3	T	C	0.407	0.052	0.008	1.2E-11	trans
202	BDNF	P23560	Brain-derived neurotrophic factor	rs274555	131722951	5	T	C	0.399	0.057	0.008	5.4E-14	trans
202	BDNF	P23560	Brain-derived neurotrophic factor	rs2516471	31400292	6	G	A	0.936	0.121	0.015	3.3E-15	trans
202	BDNF	P23560	Brain-derived neurotrophic factor	rs146842698	71328273	6	T	TAAATA	0.695	0.059	0.008	6.4E-13	trans
202	BDNF	P23560	Brain-derived neurotrophic factor	rs6961069	80218961	7	T	C	0.402	0.063	0.008	9.4E-17	trans
202	BDNF	P23560	Brain-derived neurotrophic factor	rs6993770	106581528	8	A	T	0.714	0.132	0.008	2.4E-58	trans
202	BDNF	P23560	Brain-derived neurotrophic factor	rs9410207	91404799	9	T	C	0.935	0.104	0.015	6.3E-12	trans
202	BDNF	P23560	Brain-derived neurotrophic factor	rs61751937	113312231	9	C	G	0.028	0.158	0.022	1.3E-12	trans
202	BDNF	P23560	Brain-derived neurotrophic factor	rs7080386	65048306	10	A	C	0.412	0.114	0.008	2.8E-51	trans
202	BDNF	P23560	Brain-derived neurotrophic factor	rs12445050	81870969	16	T	C	0.138	0.105	0.011	1.1E-22	trans
202	BDNF	P23560	Brain-derived neurotrophic factor	rs1654425	55538980	19	C	T	0.834	0.160	0.010	1.9E-57	trans
202	BDNF	P23560	Brain-derived neurotrophic factor	rs3827978	19281291	20	T	C	0.353	0.075	0.008	5.8E-22	trans
202	BDNF	P23560	Brain-derived neurotrophic factor	rs5967171	99957136	X	C	T	0.333	0.046	0.006	2.1E-12	trans
203	BECN1	Q14457	Beclin-1	rs1354034	56849749	3	C	T	0.605	0.071	0.008	1.5E-20	trans
203	BECN1	Q14457	Beclin-1	rs1801020	176836532	5	G	A	0.745	0.062	0.009	3.8E-13	trans
203	BECN1	Q14457	Beclin-1	rs72829144	64916651	10	A	C	0.033	0.216	0.021	2.3E-25	trans
204	BGLAP	P02818	Osteocalcin	rs541618	170663475	1	G	A	0.459	0.052	0.007	3.1E-12	trans
204	BGLAP	P02818	Osteocalcin	rs12331051	187139807	4	T	C	0.428	0.052	0.008	1.7E-11	trans
204	BGLAP	P02818	Osteocalcin	rs12209125	25769789	6	A	C	0.595	0.052	0.008	3.1E-12	trans
204	BGLAP	P02818	Osteocalcin	rs13264172	120012861	8	A	T	0.528	0.072	0.007	2.4E-22	trans
204	BGLAP	P02818	Osteocalcin	rs12597511	31145219	16	C	T	0.647	0.069	0.008	4.1E-19	trans
204	BGLAP	P02818	Osteocalcin	rs7412	45412079	19	C	T	0.919	0.109	0.014	6.7E-16	trans
204	BGLAP	P02818	Osteocalcin	rs35143646	2856155	X	T	C	0.658	0.077	0.006	4.5E-33	trans
205	BGN	P21810	Biglycan	rs744233	126249265	3	A	G	0.295	0.065	0.008	4.3E-15	trans
205	BGN	P21810	Biglycan	rs3756074	74848065	4	C	G	0.049	0.153	0.018	6.4E-18	trans
205	BGN	P21810	Biglycan	rs4463068	102801474	4	T	A	0.398	0.060	0.008	4.5E-15	trans
205	BGN	P21810	Biglycan	rs9266095	31321327	6	G	A	0.212	0.090	0.009	3.7E-22	trans
205	BGN	P21810	Biglycan	rs6993770	106581528	8	T	A	0.287	0.096	0.008	1.1E-30	trans
205	BGN	P21810	Biglycan	rs7080386	65048306	10	C	A	0.588	0.068	0.008	8.6E-19	trans
205	BGN	P21810	Biglycan	rs2511241	72945341	11	C	T	0.070	0.232	0.015	1.5E-55	trans
205	BGN	P21810	Biglycan	rs77242536	88446140	14	C	T	0.144	0.075	0.011	5.4E-12	trans
205	BGN	P21810	Biglycan	rs61758388	17564311	16	C	A	0.968	0.368	0.021	1.2E-65	trans
205	BGN	P21810	Biglycan	rs1654425	55538980	19	T	C	0.166	0.115	0.010	2.5E-29	trans
205	BGN	P21810	Biglycan	rs6081555	19245723	20	G	T	0.657	0.057	0.008	1.5E-12	trans
205	BGN	P21810	Biglycan	rs184339396	49043608	X	G	C	0.967	0.138	0.018	2.8E-14	trans
206	BID	P55957	BH3-interacting domain death agonist	rs10418046	54327869	19	G	T	0.211	0.096	0.009	2.9E-25	trans
206	BID	P55957	BH3-interacting domain death agonist	rs183393610	18252442	22	G	A	0.993	0.413	0.048	1.1E-17	cis
207	BIN2	Q9UBW5	Bridging integrator 2	rs1354034	56849749	3	C	T	0.604	0.063	0.008	7.8E-17	trans
207	BIN2	Q9UBW5	Bridging integrator 2	rs2631367	131705458	5	G	C	0.519	0.054	0.007	6.6E-13	trans
207	BIN2	Q9UBW5	Bridging integrator 2	rs6993770	106581528	8	A	T	0.714	0.063	0.008	1.2E-14	trans
208	BIRC2	Q13490	Baculoviral IAP repeat-containing protein 2	rs1354034	56849749	3	C	T	0.604	0.053	0.008	5.5E-12	trans
208	BIRC2	Q13490	Baculoviral IAP repeat-containing protein 2	rs10761785	65318766	10	T	G	0.511	0.051	0.008	1.3E-11	trans
208	BIRC2	Q13490	Baculoviral IAP repeat-containing protein 2	rs61754131	102248406	11	A	G	0.998	0.677	0.085	1.2E-15	cis
209	BLMH	Q13867	Bleomycin hydrolase	rs1250258	216300185	2	T	C	0.737	0.062	0.008	8.3E-16	trans
209	BLMH	Q13867	Bleomycin hydrolase	rs915651	31749184	6	G	A	0.869	0.083	0.010	1.6E-16	trans
209	BLMH	Q13867	Bleomycin hydrolase	rs1050565	28576076	17	C	T	0.332	0.579	0.008	0.0E+00	cis
210	BLNK	Q8WV28	B-cell linker protein	rs4954569	136913375	2	C	T	0.748	0.090	0.009	4.5E-24	trans
210	BLNK	Q8WV28	B-cell linker protein	rs4566648	84159219	4	T	G	0.662	0.107	0.008	8.1E-40	trans
210	BLNK	Q8WV28	B-cell linker protein	rs55769428	98005835	10	A	C	0.962	0.149	0.020	1.2E-13	cis
210	BLNK	Q8WV28	B-cell linker protein	rs76428106	28604007	13	C	T	0.013	0.240	0.035	9.3E-12	trans
211	BLVRB	P30043	Flavin reductase	rs11759553	135422296	6	A	T	0.728	0.078	0.008	1.6E-21	trans
211	BLVRB	P30043	Flavin reductase	rs149698066	40964064	19	G	A	0.994	1.466	0.052	8.7E-172	cis
212	BMP10	O95393	Bone morphogenetic protein 10	rs34008398	69093413	2	G	A	0.987	2.019	0.032	0.0E+00	cis
212	BMP10	O95393	Bone morphogenetic protein 10	rs7848055	95115492	9	A	C	0.671	0.153	0.007	4.9E-97	trans
212	BMP10	O95393	Bone morphogenetic protein 10	rs10740131	65271488	10	T	A	0.471	0.057	0.007	9.2E-17	trans
212	BMP10	O95393	Bone morphogenetic protein 10	rs67852477	70856963	10	G	C	0.010	0.298	0.034	2.9E-18	trans
212	BMP10	O95393	Bone morphogenetic protein 10	rs8022092	69265564	14	C	A	0.497	0.051	0.007	9.7E-14	trans
212	BMP10	O95393	Bone morphogenetic protein 10	rs1956713	94932919	14	G	A	0.149	0.130	0.010	3.6E-41	trans
212	BMP10	O95393	Bone morphogenetic protein 10	rs4702	91426560	15	A	G	0.558	0.056	0.007	3.1E-16	trans
212	BMP10	O95393	Bone morphogenetic protein 10	rs186021206	7069412	17	A	G	0.006	0.458	0.047	1.8E-22	trans
213	BMP4	P12644	Bone morphogenetic protein 4	rs535025308	74432188	11	G	C	0.959	0.351	0.019	1.4E-75	trans
213	BMP4	P12644	Bone morphogenetic protein 4	rs12284180	126265501	11	C	A	0.246	0.067	0.009	3.4E-14	trans
213	BMP4	P12644	Bone morphogenetic protein 4	rs186021206	7069412	17	A	G	0.006	0.754	0.052	2.4E-48	trans
213	BMP4	P12644	Bone morphogenetic protein 4	rs704	26694861	17	G	A	0.527	0.056	0.008	1.3E-13	trans
214	BMP6	P22004	Bone morphogenetic protein 6	rs13412535	224874874	2	G	A	0.769	0.063	0.009	1.4E-12	trans
214	BMP6	P22004	Bone morphogenetic protein 6	rs1354034	56849749	3	T	C	0.396	0.209	0.008	5.1E-164	trans
214	BMP6	P22004	Bone morphogenetic protein 6	rs3792386	122853680	3	G	A	0.413	0.053	0.008	1.0E-11	trans
214	BMP6	P22004	Bone morphogenetic protein 6	rs5030062	186454180	3	A	C	0.626	0.085	0.008	2.4E-27	trans
214	BMP6	P22004	Bone morphogenetic protein 6	rs71640034	187161048	4	G	A	0.486	0.148	0.008	1.2E-85	trans
214	BMP6	P22004	Bone morphogenetic protein 6	rs2545801	176841339	5	T	C	0.256	0.095	0.009	1.4E-28	trans
214	BMP6	P22004	Bone morphogenetic protein 6	rs71542879	7728162	6	CT	C	0.467	0.135	0.008	5.4E-71	cis
214	BMP6	P22004	Bone morphogenetic protein 6	rs6993770	106581528	8	A	T	0.713	0.080	0.008	3.4E-22	trans
214	BMP6	P22004	Bone morphogenetic protein 6	rs10820606	99192919	9	A	C	0.770	0.109	0.009	3.6E-33	trans
214	BMP6	P22004	Bone morphogenetic protein 6	rs12762934	104359884	10	T	C	0.325	0.102	0.008	1.8E-37	trans
214	BMP6	P22004	Bone morphogenetic protein 6	rs535025308	74432188	11	G	C	0.959	0.183	0.019	8.0E-22	trans
214	BMP6	P22004	Bone morphogenetic protein 6	rs186021206	7069412	17	A	G	0.006	0.441	0.052	1.5E-17	trans
214	BMP6	P22004	Bone morphogenetic protein 6	rs892090	55539072	19	G	T	0.834	0.096	0.010	2.8E-21	trans
214	BMP6	P22004	Bone morphogenetic protein 6	rs2834720	36376839	21	A	G	0.273	0.062	0.008	2.0E-13	trans
215	BMPER	Q8N8U9	BMP-binding endothelial regulator protein	rs79419884	184103722	3	G	A	0.110	0.085	0.012	8.3E-13	trans
215	BMPER	Q8N8U9	BMP-binding endothelial regulator protein	rs201897458	33783208	7	C	T	0.922	0.324	0.014	5.3E-114	cis
215	BMPER	Q8N8U9	BMP-binding endothelial regulator protein	rs4418728	94839724	10	T	G	0.451	0.064	0.007	1.9E-17	trans
215	BMPER	Q8N8U9	BMP-binding endothelial regulator protein	rs186021206	7069412	17	A	G	0.006	0.444	0.051	4.2E-18	trans
216	BNIP2	Q12982	BCL2/adenovirus E1B 19 kDa protein-interacting protein 2	rs6993770	106581528	8	A	T	0.713	0.057	0.008	9.5E-12	trans
217	BNIP3L	O60238	BCL2/adenovirus E1B 19 kDa protein-interacting protein 3-like	rs7080536	115348046	10	G	A	0.956	0.154	0.019	1.4E-16	trans
218	BOC	Q9BWV1	Brother of CDO	rs1260326	27730940	2	C	T	0.609	0.064	0.008	8.0E-17	trans
218	BOC	Q9BWV1	Brother of CDO	rs3856718	112996218	3	A	G	0.171	0.207	0.010	1.5E-94	cis
218	BOC	Q9BWV1	Brother of CDO	rs34437827	72923338	7	G	A	0.195	0.066	0.009	2.7E-12	trans
218	BOC	Q9BWV1	Brother of CDO	rs56408342	22048490	8	G	A	0.947	0.144	0.017	6.0E-18	trans
218	BOC	Q9BWV1	Brother of CDO	rs8176749	136131188	9	T	C	0.061	0.129	0.016	1.0E-16	trans
218	BOC	Q9BWV1	Brother of CDO	rs1037117	102068658	15	G	A	0.748	0.063	0.009	2.4E-13	trans
218	BOC	Q9BWV1	Brother of CDO	rs186021206	7069412	17	A	G	0.006	0.555	0.052	5.6E-27	trans
219	BOLA1	Q9Y3E2	BolA-like protein 1	rs72708157	149874606	1	G	A	0.918	0.192	0.014	1.5E-45	cis
219	BOLA1	Q9Y3E2	BolA-like protein 1	rs1354034	56849749	3	C	T	0.604	0.060	0.008	2.0E-15	trans
220	BOLA2_BOLA2B	Q9H3K6	BolA-like protein 2	rs1354034	56849749	3	C	T	0.605	0.057	0.008	3.2E-14	trans
220	BOLA2_BOLA2B	Q9H3K6	BolA-like protein 2	rs111894927	30361101	16	A	C	0.990	0.383	0.041	3.3E-21	cis
221	BPIFA2	Q96DR5	BPI fold-containing family A member 2	rs6427943	202186945	1	A	G	0.413	0.060	0.007	3.3E-16	trans
221	BPIFA2	Q96DR5	BPI fold-containing family A member 2	rs1624793	207067194	1	G	A	0.465	0.067	0.007	3.7E-20	trans
221	BPIFA2	Q96DR5	BPI fold-containing family A member 2	rs103855	109532717	2	G	A	0.100	0.081	0.012	1.5E-11	trans
221	BPIFA2	Q96DR5	BPI fold-containing family A member 2	rs1839091	44313303	5	T	C	0.331	0.072	0.008	5.7E-21	trans
221	BPIFA2	Q96DR5	BPI fold-containing family A member 2	rs10435096	26099770	7	C	T	0.095	0.091	0.012	2.2E-13	trans
221	BPIFA2	Q96DR5	BPI fold-containing family A member 2	rs67740686	107639180	9	G	A	0.885	0.078	0.011	8.2E-12	trans
221	BPIFA2	Q96DR5	BPI fold-containing family A member 2	rs247617	56990716	16	A	C	0.324	0.114	0.008	6.2E-49	trans
221	BPIFA2	Q96DR5	BPI fold-containing family A member 2	rs4843719	87882695	16	A	C	0.309	0.072	0.008	6.1E-20	trans
221	BPIFA2	Q96DR5	BPI fold-containing family A member 2	rs17855739	5831840	19	T	C	0.041	0.146	0.019	1.8E-14	trans
221	BPIFA2	Q96DR5	BPI fold-containing family A member 2	rs395908	45373565	19	A	G	0.170	0.075	0.010	1.5E-13	trans
221	BPIFA2	Q96DR5	BPI fold-containing family A member 2	rs516316	49206145	19	C	G	0.509	0.434	0.008	0.0E+00	trans
221	BPIFA2	Q96DR5	BPI fold-containing family A member 2	rs6059134	31754236	20	T	A	0.963	0.350	0.019	2.6E-73	cis
221	BPIFA2	Q96DR5	BPI fold-containing family A member 2	rs4810479	44545048	20	C	T	0.250	0.073	0.008	2.2E-18	trans
222	BPIFB1	Q8TDL5	BPI fold-containing family B member 1	rs1619599	12504290	3	A	G	0.583	0.084	0.007	1.9E-34	trans
222	BPIFB1	Q8TDL5	BPI fold-containing family B member 1	rs7429705	72291224	3	T	C	0.704	0.064	0.007	1.1E-17	trans
222	BPIFB1	Q8TDL5	BPI fold-containing family B member 1	rs791361	173208633	5	C	A	0.773	0.069	0.008	1.6E-17	trans
222	BPIFB1	Q8TDL5	BPI fold-containing family B member 1	rs34978289	2504622	6	CT	C	0.517	0.084	0.007	1.9E-35	trans
222	BPIFB1	Q8TDL5	BPI fold-containing family B member 1	rs9399137	135419018	6	C	T	0.258	0.104	0.008	4.6E-41	trans
222	BPIFB1	Q8TDL5	BPI fold-containing family B member 1	rs1822200	19852829	8	T	G	0.262	0.079	0.008	5.6E-25	trans
222	BPIFB1	Q8TDL5	BPI fold-containing family B member 1	rs2721961	116657911	8	T	G	0.717	0.058	0.008	2.9E-14	trans

222	BPIFB1	Q8TDL5	BPI fold-containing family B member 1	rs2980888	126507308	8	C	T	0.698	0.053	0.007	8.4E-13	trans
222	BPIFB1	Q8TDL5	BPI fold-containing family B member 1	rs7040302	89344224	9	T	G	0.593	0.054	0.007	2.1E-15	trans
222	BPIFB1	Q8TDL5	BPI fold-containing family B member 1	rs247617	56990716	16	A	C	0.324	0.111	0.007	7.3E-53	trans
222	BPIFB1	Q8TDL5	BPI fold-containing family B member 1	rs12597477	78334871	16	A	G	0.804	0.068	0.009	2.8E-15	trans
222	BPIFB1	Q8TDL5	BPI fold-containing family B member 1	rs116843064	8429323	19	A	G	0.020	0.199	0.024	2.6E-16	trans
222	BPIFB1	Q8TDL5	BPI fold-containing family B member 1	rs681343	49206462	19	T	C	0.509	0.084	0.007	2.1E-35	trans
222	BPIFB1	Q8TDL5	BPI fold-containing family B member 1	rs2424963	31694448	20	T	C	0.418	0.422	0.007	0.0E+00	cis
223	BPIFB2	Q8N4F0	BPI fold-containing family B member 2	rs469864	91542517	1	T	C	0.790	0.062	0.009	1.3E-12	trans
223	BPIFB2	Q8N4F0	BPI fold-containing family B member 2	rs553427	234852760	1	T	C	0.520	0.053	0.007	5.2E-14	trans
223	BPIFB2	Q8N4F0	BPI fold-containing family B member 2	rs11709077	12336507	3	G	A	0.880	0.074	0.011	1.3E-11	trans
223	BPIFB2	Q8N4F0	BPI fold-containing family B member 2	rs17145750	73026378	7	T	C	0.160	0.073	0.010	3.9E-14	trans
223	BPIFB2	Q8N4F0	BPI fold-containing family B member 2	rs174535	61551356	11	C	T	0.351	0.064	0.007	3.6E-18	trans
223	BPIFB2	Q8N4F0	BPI fold-containing family B member 2	rs62011287	63791228	15	G	A	0.342	0.060	0.007	1.0E-15	trans
223	BPIFB2	Q8N4F0	BPI fold-containing family B member 2	rs11444334	45720627	17	AC	A	0.502	0.080	0.007	1.5E-29	trans
223	BPIFB2	Q8N4F0	BPI fold-containing family B member 2	rs1801689	64210580	17	C	A	0.030	0.155	0.021	6.9E-14	trans
223	BPIFB2	Q8N4F0	BPI fold-containing family B member 2	rs429358	45411941	19	T	C	0.845	0.149	0.010	1.1E-51	trans
223	BPIFB2	Q8N4F0	BPI fold-containing family B member 2	rs2377736	31582444	20	C	T	0.403	0.265	0.008	1.4E-272	cis
224	BRAP	Q7Z569	BRCA1-associated protein	rs1354034	56849749	3	C	T	0.604	0.076	0.008	1.2E-23	trans
224	BRAP	Q7Z569	BRCA1-associated protein	rs342298	106373646	7	C	T	0.545	0.064	0.007	1.0E-17	trans
224	BRAP	Q7Z569	BRCA1-associated protein	rs7896518	65104500	10	G	A	0.424	0.052	0.008	7.2E-12	trans
225	BRDT	Q58F21	Bromodomain testis-specific protein	rs25640	118811533	5	G	A	0.544	0.167	0.008	5.5E-104	trans
225	BRDT	Q58F21	Bromodomain testis-specific protein	rs11974227	80222408	7	T	C	0.063	0.115	0.016	4.7E-13	trans
225	BRDT	Q58F21	Bromodomain testis-specific protein	rs6993770	106581528	8	A	T	0.714	0.077	0.008	5.6E-20	trans
225	BRDT	Q58F21	Bromodomain testis-specific protein	rs10822145	64934548	10	T	C	0.473	0.056	0.008	3.7E-13	trans
226	BRK1	Q8WUW1	Protein BRICK1	rs1354034	56849749	3	C	T	0.604	0.131	0.008	1.7E-65	trans
226	BRK1	Q8WUW1	Protein BRICK1	rs6054	155489608	4	T	C	0.006	0.398	0.051	3.3E-15	trans
226	BRK1	Q8WUW1	Protein BRICK1	rs342293	106372219	7	C	G	0.541	0.075	0.008	1.7E-23	trans
226	BRK1	Q8WUW1	Protein BRICK1	rs7896518	65104500	10	G	A	0.424	0.071	0.008	3.3E-20	trans
226	BRK1	Q8WUW1	Protein BRICK1	rs707236	83803160	16	T	A	0.654	0.148	0.008	9.9E-78	trans
226	BRK1	Q8WUW1	Protein BRICK1	rs9897552	33974481	17	A	G	0.177	0.072	0.010	2.2E-13	trans
226	BRK1	Q8WUW1	Protein BRICK1	rs1065853	45413233	19	G	T	0.919	0.251	0.014	1.7E-72	trans
227	BRSK2	Q8IWQ3	Serine/threonine-protein kinase BRSK2	rs7395567	1413056	11	T	C	0.265	0.606	0.009	0.0E+00	cis
228	BSG	P35613	Basigin	rs61747728	179526214	1	T	C	0.038	0.184	0.019	1.7E-22	trans
228	BSG	P35613	Basigin	rs78444298	184672098	1	A	G	0.019	0.208	0.026	4.6E-15	trans
228	BSG	P35613	Basigin	rs1260326	27730940	2	C	T	0.608	0.059	0.007	3.2E-15	trans
228	BSG	P35613	Basigin	rs700753	46753684	7	G	C	0.654	0.060	0.008	2.0E-15	trans
228	BSG	P35613	Basigin	rs2519093	136141870	9	C	T	0.816	0.144	0.009	7.9E-53	trans
228	BSG	P35613	Basigin	rs77924615	20392332	16	G	A	0.803	0.072	0.009	6.2E-15	trans
228	BSG	P35613	Basigin	rs200489612	7106378	17	A	G	0.005	0.417	0.054	1.9E-14	trans
228	BSG	P35613	Basigin	rs56101188	586745	19	C	T	0.091	0.241	0.013	6.3E-79	cis
229	BST1	Q10588	ADP-ribosyl cyclase/cyclic ADP-ribose hydrolase 2	rs2302465	15709192	4	G	A	0.870	1.492	0.011	0.0E+00	cis
229	BST1	Q10588	ADP-ribosyl cyclase/cyclic ADP-ribose hydrolase 2	rs7952602	126233669	11	G	C	0.865	0.103	0.007	3.8E-49	trans
229	BST1	Q10588	ADP-ribosyl cyclase/cyclic ADP-ribose hydrolase 2	rs186021206	7069412	17	A	G	0.006	0.529	0.033	5.5E-59	trans
229	BST1	Q10588	ADP-ribosyl cyclase/cyclic ADP-ribose hydrolase 2	rs4760	44153100	19	A	G	0.844	0.214	0.007	1.1E-230	trans
230	BST2	Q10589	Bone marrow stromal antigen 2	rs1063412	172410967	1	G	A	0.441	0.052	0.007	2.0E-12	trans
230	BST2	Q10589	Bone marrow stromal antigen 2	rs2111485	163110536	2	G	A	0.610	0.061	0.007	2.8E-16	trans
230	BST2	Q10589	Bone marrow stromal antigen 2	rs4835265	146821410	4	A	C	0.156	0.073	0.010	5.5E-13	trans
230	BST2	Q10589	Bone marrow stromal antigen 2	rs6926219	122720806	6	G	A	0.455	0.057	0.007	8.6E-15	trans
230	BST2	Q10589	Bone marrow stromal antigen 2	rs17157266	62199817	11	T	C	0.820	0.067	0.010	2.3E-12	trans
230	BST2	Q10589	Bone marrow stromal antigen 2	rs12373325	56087648	18	T	C	0.202	0.062	0.009	1.2E-11	trans
230	BST2	Q10589	Bone marrow stromal antigen 2	rs35251378	10459969	19	G	A	0.706	0.082	0.008	2.2E-24	trans
230	BST2	Q10589	Bone marrow stromal antigen 2	rs8101243	17488793	19	T	C	0.043	0.344	0.018	8.5E-79	cis
230	BST2	Q10589	Bone marrow stromal antigen 2	rs5992933	18457043	22	G	T	0.757	0.060	0.009	2.1E-12	trans
231	BTC	P35070	Probetacellulin	rs1354034	56849749	3	C	T	0.604	0.053	0.007	7.7E-13	trans
231	BTC	P35070	Probetacellulin	rs28549760	75719517	4	A	C	0.868	0.598	0.011	0.0E+00	cis
231	BTC	P35070	Probetacellulin	rs342296	106372903	7	G	A	0.543	0.050	0.007	5.9E-12	trans
231	BTC	P35070	Probetacellulin	rs7044478	100621002	9	C	G	0.667	0.057	0.008	6.0E-13	trans
232	BTD	P43251	Biotinidase	rs1260326	27730940	2	T	C	0.392	0.076	0.007	2.0E-30	trans
232	BTD	P43251	Biotinidase	rs13078881	15686693	3	G	C	0.957	1.618	0.018	0.0E+00	cis
232	BTD	P43251	Biotinidase	rs73015965	161127501	6	G	A	0.006	0.394	0.044	1.4E-19	trans
232	BTD	P43251	Biotinidase	rs10761760	65179152	10	A	C	0.571	0.055	0.007	2.2E-16	trans
232	BTD	P43251	Biotinidase	rs148480921	41652040	13	CA	C	0.075	0.085	0.012	1.0E-11	trans
232	BTD	P43251	Biotinidase	rs4788460	72154509	16	T	C	0.294	0.064	0.007	1.5E-19	trans
232	BTD	P43251	Biotinidase	rs1801689	64210580	17	C	A	0.030	0.328	0.019	5.2E-67	trans
232	BTD	P43251	Biotinidase	rs12019136	5835677	19	A	G	0.041	0.129	0.016	3.6E-15	trans
233	BTN1A1	Q13410	Butyrophilin subfamily 1 member A1	rs12211201	26461461	6	G	A	0.106	0.300	0.012	3.2E-130	cis
234	BTN2A1	Q7KYR7	Butyrophilin subfamily 2 member A1	rs61747728	179526214	1	T	C	0.038	0.192	0.019	5.2E-25	trans
234	BTN2A1	Q7KYR7	Butyrophilin subfamily 2 member A1	rs143048713	46893173	3	GGT	G	0.100	0.083	0.012	4.1E-12	trans
234	BTN2A1	Q7KYR7	Butyrophilin subfamily 2 member A1	rs72843784	26498758	6	T	G	0.110	0.472	0.012	0.0E+00	cis
234	BTN2A1	Q7KYR7	Butyrophilin subfamily 2 member A1	rs77924615	20392332	16	G	A	0.803	0.076	0.009	4.2E-17	trans
234	BTN2A1	Q7KYR7	Butyrophilin subfamily 2 member A1	rs186021206	7069412	17	A	G	0.006	0.527	0.049	3.7E-27	trans
234	BTN2A1	Q7KYR7	Butyrophilin subfamily 2 member A1	rs2659005	79218714	17	T	C	0.441	0.065	0.007	2.1E-19	trans
235	BTN3A2	P78410	Butyrophilin subfamily 3 member A2	rs9393710	26367833	6	A	G	0.866	0.956	0.011	0.0E+00	cis
235	BTN3A2	P78410	Butyrophilin subfamily 3 member A2	rs74439742	57059427	16	C	T	0.828	0.094	0.009	4.2E-27	trans
236	C19orf12	Q9NSK7	Protein C19orf12	rs142552223	172234017	3	G	A	0.962	0.136	0.020	1.4E-11	trans
236	C19orf12	Q9NSK7	Protein C19orf12	rs705379	94953895	7	A	G	0.477	0.087	0.008	3.6E-30	trans
236	C19orf12	Q9NSK7	Protein C19orf12	rs16919942	93840415	11	C	T	0.894	0.085	0.012	5.4E-12	trans
236	C19orf12	Q9NSK7	Protein C19orf12	rs28929474	94844947	14	T	C	0.021	0.195	0.027	2.1E-13	trans
236	C19orf12	Q9NSK7	Protein C19orf12	rs738409	44324727	22	G	C	0.217	0.097	0.009	3.8E-26	trans
237	C1GALT1C1	Q96EU7	C1GALT1-specific chaperone 1	rs149340486	119760720	X	C	T	0.990	0.717	0.032	3.1E-112	cis
238	C1QA	P02745	Complement C1q subcomponent subunit A	rs12058824	22963050	1	G	A	0.917	0.631	0.013	0.0E+00	cis
238	C1QA	P02745	Complement C1q subcomponent subunit A	rs188468174	25291697	1	T	C	0.014	0.215	0.032	1.1E-11	trans
238	C1QA	P02745	Complement C1q subcomponent subunit A	rs2765501	157804648	1	A	G	0.401	0.066	0.008	2.3E-18	trans
238	C1QA	P02745	Complement C1q subcomponent subunit A	rs6532770	99783669	4	A	C	0.244	0.151	0.008	6.3E-73	trans
238	C1QA	P02745	Complement C1q subcomponent subunit A	rs4868218	172257971	5	C	A	0.248	0.078	0.008	2.4E-21	trans
238	C1QA	P02745	Complement C1q subcomponent subunit A	rs2734975	29833253	6	C	A	0.540	0.062	0.007	3.4E-18	trans
238	C1QA	P02745	Complement C1q subcomponent subunit A	rs1727	91066769	10	C	A	0.275	0.111	0.008	1.5E-44	trans
238	C1QA	P02745	Complement C1q subcomponent subunit A	rs28372834	49749142	13	T	TA	0.836	0.066	0.010	5.9E-12	trans
238	C1QA	P02745	Complement C1q subcomponent subunit A	rs2665399	57866422	17	A	C	0.159	0.090	0.010	9.6E-21	trans
238	C1QA	P02745	Complement C1q subcomponent subunit A	rs117981338	71088843	17	T	C	0.014	0.387	0.030	5.4E-37	trans
238	C1QA	P02745	Complement C1q subcomponent subunit A	rs55841020	45821172	18	T	A	0.826	0.074	0.009	2.0E-15	trans
238	C1QA	P02745	Complement C1q subcomponent subunit A	rs28659908	35693120	19	C	T	0.405	0.064	0.007	1.7E-18	trans
239	C1QBP	Q07021	Complement component 1 Q subcomponent-binding protein, mitochondrial	rs74839303	106362295	14	C	T	0.883	0.121	0.013	1.2E-19	trans
240	C1QL2	Q7Z5L3	Complement C1q-like protein 2	rs4844573	207304900	1	T	C	0.651	0.260	0.007	1.3E-266	trans
240	C1QL2	Q7Z5L3	Complement C1q-like protein 2	rs34475538	119918265	2	G	A	0.798	0.279	0.009	9.5E-210	cis
240	C1QL2	Q7Z5L3	Complement C1q-like protein 2	rs876036	50307710	7	T	C	0.681	0.052	0.008	5.5E-12	trans
240	C1QL2	Q7Z5L3	Complement C1q-like protein 2	rs1420299	54436541	16	T	C	0.345	0.096	0.007	1.5E-37	trans
240	C1QL2	Q7Z5L3	Complement C1q-like protein 2	rs11149803	75195765	16	G	C	0.315	0.150	0.008	4.2E-86	trans
240	C1QL2	Q7Z5L3	Complement C1q-like protein 2	rs2957150	60080311	18	A	G	0.356	0.062	0.007	3.7E-17	trans
240	C1QL2	Q7Z5L3	Complement C1q-like protein 2	rs571497	7827830	19	G	A	0.847	0.092	0.010	1.0E-20	trans
241	C1QTNF1	Q9BXJ1	Complement C1q tumor necrosis factor-related protein 1	rs2498323	3451109	4	A	G	0.101	0.134	0.012	7.5E-28	trans
241	C1QTNF1	Q9BXJ1	Complement C1q tumor necrosis factor-related protein 1	rs116539064	77018400	17	T	C	0.097	0.398	0.013	5.8E-212	cis
241	C1QTNF1	Q9BXJ1	Complement C1q tumor necrosis factor-related protein 1	rs2838952	46930163	21	C	T	0.965	0.252	0.020	1.5E-35	trans
242	C1QTNF5	Q9BXJ0	Complement C1q tumor necrosis factor-related protein 5	rs12756819	159532524	1	T	C	0.977	0.178	0.024	2.8E-13	trans
242	C1QTNF5	Q9BXJ0	Complement C1q tumor necrosis factor-related protein 5	rs35677470	58183636	3	G	A	0.917	0.098	0.013	1.7E-13	trans
242	C1QTNF5	Q9BXJ0	Complement C1q tumor necrosis factor-related protein 5	rs10513546	159551651	3	A	G	0.297	0.063	0.008	3.4E-15	trans
242	C1QTNF5	Q9BXJ0	Complement C1q tumor necrosis factor-related protein 5	rs674980	72365332	5	T	C	0.534	0.062	0.007	2.1E-17	trans
242	C1QTNF5	Q9BXJ0	Complement C1q tumor necrosis factor-related protein 5	rs7720230	131592405	5	T	G	0.537	0.051	0.007	2.6E-12	trans
242	C1QTNF5	Q9BXJ0	Complement C1q tumor necrosis factor-related protein 5	rs2856694	32652281	6	T	C	0.814	0.076	0.010	2.2E-14	trans
242	C1QTNF5	Q9BXJ0	Complement C1q tumor necrosis factor-related protein 5	rs41341748	16012594	8	A	G	0.011	1.759	0.037	0.0E+00	trans
242	C1QTNF5	Q9BXJ0	Complement C1q tumor necrosis factor-related protein 5	rs35184771	47475189	11	G	T	0.649	0.060	0.008	9.0E-15	trans
242	C1QTNF5	Q9BXJ0	Complement C1q tumor necrosis factor-related protein 5	rs7232	59940599	11	T	A	0.627	0.063	0.008	9.8E-17	trans
242	C1QTNF5	Q9BXJ0	Complement C1q tumor necrosis factor-related protein 5	rs2509656	119208576	11	A	G	0.143	0.163	0.010	4.0E-55	cis
243	C1QTNF9	P0C862	Complement C1q and tumor necrosis factor-related protein 9A	rs72550870	11106666	1	T	C	0.970	0.124	0.017	6.6E-14	trans
243	C1QTNF9	P0C862	Complement C1q and tumor necrosis factor-related protein 9A	rs10510410	12346738	3	A	C	0.757	0.045	0.007	1.1E-11	trans
243	C1QTNF9	P0C862	Complement C1q and tumor necrosis factor-related protein 9A	rs142021145	71756519	5	C	A	0.997	0.356	0.048	1.6E-13	trans
243	C1QTNF9	P0C862	Complement C1q and tumor necrosis factor-related protein 9A	rs27289	96346278	5	G	T	0.447	0.061	0.006	2.8E-26	trans
243	C1QTNF9	P0C862	Complement C1q and tumor necrosis factor-related protein 9A	rs2524096	31236467	6	G	T	0.580	0.070	0.006	1.6E-33	trans
243	C1QTNF9	P0C862	Complement C1q and tumor necrosis factor-related protein 9A	rs11259539	15239029	10	C	T	0.409	0.067	0.006	8.6E-31	trans
243	C1QTNF9	P0C862	Complement C1q and tumor necrosis factor-related protein 9A	rs1961993	24891017	13	A	C	0.803	1.151	0.010	0.0E+00	cis
243	C1QTNF9	P0C862	Complement C1q and tumor necrosis factor-related protein 9A	rs13333582	67549547	16	C	T	0.043	0.097	0.014	5.1E-12	trans
243	C1QTNF9	P0C862	Complement C1q and tumor necrosis factor-related protein 9A	rs12975366	54759361	19	T	C	0.605	0.042	0.006	3.2E-13	trans
244	C1R	P00736	Complement C1r subcomponent	rs12722051	32609147	6	T	A	0.204	0.064	0.009	3.8E-12	trans
244	C1R	P00736	Complement C1r subcomponent	rs12433460	106262680	14	C	A	0.731	0.191	0.009	1.3E-94	trans
245	C1RL	Q9NZP8	Complement C1r subcomponent-like protein	rs35447916	64933592	10	C	CA	0.530	0.065	0.007	3.8E-20	trans
245	C1RL	Q9NZP8	Complement C1r subcomponent-like protein	rs28362944	57365723	11	C	T	0.049	0.124	0.016	2.6E-14	trans
245	C1RL	Q9NZP8	Complement C1r subcomponent-like protein	rs174566	61592362	11	G	A	0.350	0.071	0.007	4.2E-22	trans
245	C1RL	Q9NZP8	Complement C1r subcomponent-like protein	rs149394327	64228995	17	C	G	0.030	0.350	0.021	1.0E-63	trans
246	C1S	P09871	Complement C1s subcomponent	rs73480556	57400462	11	G	C	0.729	0.080	0.008	4.6E-23	trans
246	C1S	P09871	Complement C1s subcomponent	rs12433460	106262680	14	C	A	0.731	0.199	0.009	4.5E-108	trans
247	C2	P06681	Complement C2	rs1260326	27730940	2	T	C	0.392	0.069	0.007	1.6E-21	trans
247	C2	P06681	Complement C2	rs6984305	9178268	8	T	A	0.885	0.075	0.011	1.5E-11	trans
247	C2	P06681	Complement C2	rs56278466	17875857	10	G	T	0.661	0.104	0.007	5.2E-44	trans
248	C2CD2L	O14523	Phospholipid transfer protein C2CD2L	rs640603	118964330	11	G	A	0.648	0.056	0.008	5.5E-12	cis
249	C2orf69	Q8N8R5	UPF0565 protein C2orf69	rs17629007	200721555	2	C	T	0.032	0.253	0.022	3.1E-31	cis
249	C2orf69	Q8N8R5	UPF0565 protein C2orf69	rs1354034	56849749	3	C	T	0.604	0.063	0.008	1.1E-15	trans
250	C3	P01024	Complement C3	rs3753396	196695742	1	A	G	0.828	0.128	0.010	3.3E-36	trans
250	C3	P01024	Complement C3	rs1260326	27730940	2	T	C	0.392	0.065	0.008	1.5E-16	trans
250	C3	P01024	Complement C3	rs556679	31894355	6	T	C	0.121	0.107	0.012	1.2E-19	trans
250	C3	P01024	Complement C3	rs12975366	54759361	19	C	T	0.396	0.072	0.008	8.0E-20	trans
251	C4BPB	P20851	C4b-binding protein beta chain	rs140584594	110232983	1	G	A	0.732	0.060	0.008	6.8E-14	trans
251	C4BPB	P20851	C4b-binding protein beta chain	rs2316653	90442925	3	A	G	0.501	0.060	0.007	3.3E-17	trans
251	C4BPB	P20851	C4b-binding protein beta chain	rs9822589	93931141	3	A	G	0.468	0.060	0.007	2.8E-17	trans

251	C4BPB	P20851	C4b-binding protein beta chain	rs687339	135932359	3	C	T	0.229	0.060	0.008	1.5E-12	trans
251	C4BPB	P20851	C4b-binding protein beta chain	rs28507491	77197651	4	G	A	0.623	0.055	0.007	5.3E-14	trans
251	C4BPB	P20851	C4b-binding protein beta chain	rs601020	32573179	6	G	A	0.755	0.064	0.008	3.1E-14	trans
251	C4BPB	P20851	C4b-binding protein beta chain	rs2068888	94839642	10	G	A	0.550	0.049	0.007	5.9E-12	trans
251	C4BPB	P20851	C4b-binding protein beta chain	rs11621792	24871926	14	C	T	0.547	0.049	0.007	4.1E-12	trans
251	C4BPB	P20851	C4b-binding protein beta chain	rs28929474	94844947	14	T	C	0.021	0.364	0.025	9.0E-49	trans
251	C4BPB	P20851	C4b-binding protein beta chain	rs9302635	72144174	16	T	C	0.818	0.137	0.009	2.3E-50	trans
251	C4BPB	P20851	C4b-binding protein beta chain	rs8178824	64224775	17	T	C	0.030	0.815	0.021	0.0E+00	trans
252	C5	P01031	Complement C5	rs780094	27741237	2	T	C	0.381	0.062	0.008	8.8E-16	trans
252	C5	P01031	Complement C5	rs4837810	123881199	9	T	C	0.423	0.143	0.008	6.5E-78	cis
253	C7	P10643	Complement component C7	rs74480769	40972211	5	A	G	0.971	1.498	0.023	0.0E+00	cis
253	C7	P10643	Complement component C7	rs56278466	17875857	10	G	T	0.661	0.077	0.007	5.9E-28	trans
253	C7	P10643	Complement component C7	rs738409	44324727	22	G	C	0.217	0.069	0.008	1.7E-17	trans
254	C7orf50	Q9BRJ6	Uncharacterized protein C7orf50	rs200169409	1037382	7	GC	G	0.972	0.948	0.023	0.0E+00	cis
255	C8B	P07358	Complement component C8 beta chain	rs626457	57407484	1	T	C	0.330	0.519	0.008	0.0E+00	cis
255	C8B	P07358	Complement component C8 beta chain	rs1260326	27730940	2	T	C	0.392	0.055	0.007	9.7E-16	trans
256	C9	P02748	Complement component C9	rs6734238	113841030	2	G	A	0.403	0.053	0.008	3.8E-12	trans
256	C9	P02748	Complement component C9	rs34000044	39342214	5	G	T	0.999	1.405	0.098	8.7E-47	cis
257	C9orf40	Q8IXQ3	Uncharacterized protein C9orf40	rs11759553	135422296	6	A	T	0.728	0.066	0.008	1.5E-15	trans
257	C9orf40	Q8IXQ3	Uncharacterized protein C9orf40	rs1879154	78537155	15	T	C	0.605	0.060	0.007	1.0E-15	trans
257	C9orf40	Q8IXQ3	Uncharacterized protein C9orf40	rs75372982	33081333	19	A	G	0.712	0.065	0.008	1.8E-15	trans
258	CA1	P00915	Carbonic anhydrase 1	rs3811444	248039451	1	T	C	0.332	0.080	0.008	8.8E-25	trans
258	CA1	P00915	Carbonic anhydrase 1	rs11759553	135422296	6	A	T	0.728	0.075	0.008	6.9E-20	trans
258	CA1	P00915	Carbonic anhydrase 1	rs12544332	86256210	8	C	A	0.411	0.089	0.007	5.8E-33	cis
258	CA1	P00915	Carbonic anhydrase 1	rs67544533	75361539	15	A	G	0.817	0.132	0.009	3.0E-44	trans
259	CA11	O75493	Carbonic anhydrase-related protein 11	rs646776	109818530	1	T	C	0.778	0.186	0.009	1.7E-89	trans
259	CA11	O75493	Carbonic anhydrase-related protein 11	rs4747198	73568406	10	C	G	0.715	0.059	0.009	3.4E-12	trans
260	CA12	O43570	Carbonic anhydrase 12	rs61747728	179526214	1	T	C	0.038	0.169	0.019	8.1E-19	trans
260	CA12	O43570	Carbonic anhydrase 12	rs1260326	27730940	2	C	T	0.608	0.075	0.008	3.2E-23	trans
260	CA12	O43570	Carbonic anhydrase 12	rs13107325	103188709	4	T	C	0.075	0.134	0.014	1.2E-21	trans
260	CA12	O43570	Carbonic anhydrase 12	rs12341245	128340941	9	A	G	0.398	0.056	0.007	6.8E-14	trans
260	CA12	O43570	Carbonic anhydrase 12	rs12902855	53975914	15	A	G	0.483	0.062	0.008	1.9E-16	trans
260	CA12	O43570	Carbonic anhydrase 12	rs12916532	63674171	15	T	C	0.726	0.294	0.008	7.3E-268	cis
260	CA12	O43570	Carbonic anhydrase 12	rs186021206	7069412	17	A	G	0.006	0.639	0.051	3.1E-36	trans
260	CA12	O43570	Carbonic anhydrase 12	rs708686	5840619	19	T	C	0.267	0.112	0.008	8.6E-42	trans
261	CA13	Q8N1Q1	Carbonic anhydrase 13	rs1354034	56849749	3	C	T	0.604	0.053	0.008	1.3E-12	trans
261	CA13	Q8N1Q1	Carbonic anhydrase 13	rs56072918	86209408	8	C	T	0.932	0.526	0.015	3.5E-273	cis
262	CA14	Q9ULX7	Carbonic anhydrase 14	rs1122967	150258838	1	G	C	0.510	0.174	0.007	2.3E-129	cis
262	CA14	Q9ULX7	Carbonic anhydrase 14	rs61431557	34717578	6	C	T	0.863	0.071	0.010	4.0E-12	trans
262	CA14	Q9ULX7	Carbonic anhydrase 14	rs2954021	126482077	8	G	A	0.504	0.075	0.007	2.3E-26	trans
263	CA2	P00918	Carbonic anhydrase 2	rs2980370	86860127	8	G	A	0.521	0.102	0.007	6.2E-45	cis
264	CA3	P07451	Carbonic anhydrase 3	rs3811444	248039451	1	T	C	0.332	0.112	0.008	6.2E-50	trans
264	CA3	P07451	Carbonic anhydrase 3	rs1047891	211540507	2	A	C	0.313	0.086	0.008	5.3E-29	trans
264	CA3	P07451	Carbonic anhydrase 3	rs11759553	135422296	6	A	T	0.728	0.114	0.008	1.1E-45	trans
264	CA3	P07451	Carbonic anhydrase 3	rs2072696	86351051	8	G	C	0.757	0.174	0.008	8.0E-97	cis
264	CA3	P07451	Carbonic anhydrase 3	rs1867146	75354971	15	C	G	0.818	0.089	0.009	3.4E-22	trans
264	CA3	P07451	Carbonic anhydrase 3	rs1799918	13002400	19	G	C	0.621	0.067	0.007	1.2E-19	trans
265	CA4	P22748	Carbonic anhydrase 4	rs184775562	197569112	2	C	T	0.002	0.818	0.092	8.2E-19	trans
265	CA4	P22748	Carbonic anhydrase 4	rs78058190	219699999	2	G	A	0.949	0.189	0.019	3.2E-24	trans
265	CA4	P22748	Carbonic anhydrase 4	rs115007843	83575212	4	A	C	0.824	0.072	0.010	8.0E-14	trans
265	CA4	P22748	Carbonic anhydrase 4	rs9270588	32561327	6	T	C	0.531	0.059	0.007	1.1E-15	trans
265	CA4	P22748	Carbonic anhydrase 4	rs117154637	58234154	17	A	T	0.965	0.825	0.020	0.0E+00	cis
265	CA4	P22748	Carbonic anhydrase 4	rs10642898	76393675	17	CCCG	C	0.403	0.055	0.008	7.9E-13	trans
265	CA4	P22748	Carbonic anhydrase 4	rs4760	44153100	19	A	G	0.844	0.170	0.010	1.4E-64	trans
265	CA4	P22748	Carbonic anhydrase 4	rs12975366	54759361	19	T	C	0.605	0.073	0.007	1.3E-22	trans
266	CA5A	P35218	Carbonic anhydrase 5A, mitochondrial	rs4835265	146821410	4	A	C	0.156	0.071	0.009	2.8E-15	trans
266	CA5A	P35218	Carbonic anhydrase 5A, mitochondrial	rs4979373	117144504	9	C	T	0.509	0.048	0.007	3.4E-13	trans
266	CA5A	P35218	Carbonic anhydrase 5A, mitochondrial	rs10883451	101924418	10	T	C	0.498	0.045	0.006	4.8E-12	trans
266	CA5A	P35218	Carbonic anhydrase 5A, mitochondrial	rs55870502	87927222	16	C	T	0.193	0.812	0.009	0.0E+00	cis
266	CA5A	P35218	Carbonic anhydrase 5A, mitochondrial	rs738409	44324727	22	G	C	0.217	0.054	0.008	8.9E-12	trans
267	CA6	P23280	Carbonic anhydrase 6	rs3765963	9034598	1	G	A	0.412	0.683	0.007	0.0E+00	cis
267	CA6	P23280	Carbonic anhydrase 6	rs946527	46485970	1	T	C	0.292	0.056	0.008	4.6E-13	trans
267	CA6	P23280	Carbonic anhydrase 6	rs351388	212422580	1	C	G	0.408	0.051	0.007	2.2E-12	trans
267	CA6	P23280	Carbonic anhydrase 6	rs891499	10114230	2	C	G	0.327	0.065	0.007	9.8E-24	trans
267	CA6	P23280	Carbonic anhydrase 6	rs836143	34535585	11	G	C	0.328	0.054	0.007	2.7E-16	trans
267	CA6	P23280	Carbonic anhydrase 6	rs4256938	94323577	11	G	A	0.905	0.123	0.010	5.5E-32	trans
267	CA6	P23280	Carbonic anhydrase 6	rs290371	95761108	15	G	A	0.645	0.071	0.006	4.3E-28	trans
267	CA6	P23280	Carbonic anhydrase 6	rs173539	56988044	16	T	C	0.332	0.057	0.007	1.7E-18	trans
267	CA6	P23280	Carbonic anhydrase 6	rs1523135	47183320	17	G	C	0.716	0.055	0.007	1.0E-15	trans
267	CA6	P23280	Carbonic anhydrase 6	rs7250028	13953120	19	G	T	0.237	0.058	0.007	9.3E-16	trans
267	CA6	P23280	Carbonic anhydrase 6	rs601338	49206674	19	A	G	0.510	0.063	0.006	1.8E-24	trans
268	CA9	Q16790	Carbonic anhydrase 9	rs183985235	35677780	9	C	A	0.998	1.479	0.085	2.5E-67	cis
268	CA9	Q16790	Carbonic anhydrase 9	rs56278466	17875857	10	G	T	0.661	0.142	0.008	2.5E-74	trans
268	CA9	Q16790	Carbonic anhydrase 9	rs616114	29797921	18	T	C	0.390	0.082	0.008	1.4E-27	trans
268	CA9	Q16790	Carbonic anhydrase 9	rs2608894	5848000	19	T	C	0.235	0.063	0.009	6.3E-13	trans
268	CA9	Q16790	Carbonic anhydrase 9	rs492602	49206417	19	A	G	0.491	0.052	0.007	3.3E-12	trans
269	CABP2	Q9NPB3	Calcium-binding protein 2	rs607929	32586998	6	C	G	0.551	0.059	0.008	2.2E-14	trans
269	CABP2	Q9NPB3	Calcium-binding protein 2	rs79034686	107097568	14	T	C	0.252	0.070	0.009	3.0E-15	trans
269	CABP2	Q9NPB3	Calcium-binding protein 2	rs62129286	48554875	19	T	C	0.231	0.161	0.009	1.9E-69	trans
270	CACNB1	Q02641	Voltage-dependent L-type calcium channel subunit beta-1	rs58534292	106483584	14	T	C	0.350	0.091	0.008	7.5E-29	trans
270	CACNB1	Q02641	Voltage-dependent L-type calcium channel subunit beta-1	rs57523015	106486754	14	C	T	0.338	0.094	0.008	1.7E-29	trans
271	CACNB3	P54284	Voltage-dependent L-type calcium channel subunit beta-3	rs1354034	56849749	3	C	T	0.604	0.096	0.008	8.7E-36	trans
271	CACNB3	P54284	Voltage-dependent L-type calcium channel subunit beta-3	rs11502185	180258	11	C	T	0.260	0.072	0.010	8.8E-14	trans
272	CACYBP	Q9HB71	Calcyclin-binding protein	rs1354034	56849749	3	C	T	0.604	0.097	0.008	1.6E-36	trans
272	CACYBP	Q9HB71	Calcyclin-binding protein	rs342298	106373646	7	C	T	0.545	0.057	0.008	6.2E-14	trans
272	CACYBP	Q9HB71	Calcyclin-binding protein	rs73396521	267568	11	A	G	0.245	0.060	0.009	1.3E-11	trans
273	CALB1	P05937	Calbindin	rs143241372	91123583	8	G	A	0.009	0.655	0.040	5.4E-60	cis
273	CALB1	P05937	Calbindin	rs9529913	72345089	13	C	T	0.397	0.085	0.008	3.8E-29	trans
273	CALB1	P05937	Calbindin	rs6127099	52731402	20	A	T	0.722	0.063	0.008	9.4E-14	trans
274	CALB2	P22676	Calretinin	rs1476089	71153772	16	G	A	0.712	0.069	0.008	1.7E-16	cis
275	CALCA	P01258	Calcitonin	rs3748916	113984033	2	A	G	0.472	0.067	0.006	7.1E-28	trans
275	CALCA	P01258	Calcitonin	rs73995235	233196537	2	G	T	0.940	0.112	0.013	9.2E-18	trans
275	CALCA	P01258	Calcitonin	rs7008914	25880400	8	T	C	0.745	0.054	0.007	2.3E-14	trans
275	CALCA	P01258	Calcitonin	rs138903858	105634487	14	G	T	0.972	0.140	0.019	2.1E-13	trans
275	CALCA	P01258	Calcitonin	rs3790163	10647951	20	G	A	0.793	0.069	0.008	7.8E-19	trans
276	CALCB	P10092	Calcitonin gene-related peptide 2	rs4757282	15126744	11	G	T	0.240	0.127	0.009	9.7E-47	cis
277	CALCOCO1	Q9P1Z2	Calcium-binding and coiled-coil domain-containing protein 1	rs1354034	56849749	3	C	T	0.604	0.062	0.007	3.6E-17	trans
277	CALCOCO1	Q9P1Z2	Calcium-binding and coiled-coil domain-containing protein 1	rs342293	106372219	7	C	G	0.541	0.054	0.007	7.5E-14	trans
277	CALCOCO1	Q9P1Z2	Calcium-binding and coiled-coil domain-containing protein 1	rs6993770	106581528	8	A	T	0.714	0.072	0.008	5.2E-19	trans
278	CALCOCO2	Q13137	Calcium-binding and coiled-coil domain-containing protein 2	rs1354034	56849749	3	C	T	0.605	0.195	0.008	6.8E-143	trans
278	CALCOCO2	Q13137	Calcium-binding and coiled-coil domain-containing protein 2	rs3792382	122850938	3	T	C	0.407	0.055	0.008	1.1E-12	trans
278	CALCOCO2	Q13137	Calcium-binding and coiled-coil domain-containing protein 2	rs11242109	131677047	5	G	T	0.519	0.057	0.007	4.3E-14	trans
278	CALCOCO2	Q13137	Calcium-binding and coiled-coil domain-containing protein 2	rs342298	106373646	7	C	T	0.545	0.083	0.008	3.4E-28	trans
278	CALCOCO2	Q13137	Calcium-binding and coiled-coil domain-containing protein 2	rs200309755	27845774	17	T	C	0.537	0.054	0.008	2.3E-12	trans
278	CALCOCO2	Q13137	Calcium-binding and coiled-coil domain-containing protein 2	rs606911	46908228	17	A	G	0.159	0.075	0.010	1.4E-13	cis
279	CAMKK1	Q8N5S9	Calcium/calmodulin-dependent protein kinase kinase 1	rs342296	106372903	7	G	A	0.543	0.063	0.008	1.5E-16	trans
279	CAMKK1	Q8N5S9	Calcium/calmodulin-dependent protein kinase kinase 1	rs753513	3793472	17	C	T	0.713	0.297	0.008	4.7E-272	cis
280	CAMSAP1	Q5T5Y3	Calmodulin-regulated spectrin-associated protein 1	rs1354034	56849749	3	C	T	0.604	0.077	0.007	6.8E-25	trans
281	CANT1	Q8WVQ1	Soluble calcium-activated nucleotidase 1	rs61747728	179526214	1	T	C	0.038	0.142	0.019	3.1E-13	trans
281	CANT1	Q8WVQ1	Soluble calcium-activated nucleotidase 1	rs1863622	186396616	3	T	C	0.778	0.079	0.009	2.7E-18	trans
281	CANT1	Q8WVQ1	Soluble calcium-activated nucleotidase 1	rs56278466	17875857	10	G	T	0.662	0.088	0.008	7.8E-29	trans
281	CANT1	Q8WVQ1	Soluble calcium-activated nucleotidase 1	rs423135	89740873	16	A	G	0.518	0.059	0.008	2.7E-15	trans
281	CANT1	Q8WVQ1	Soluble calcium-activated nucleotidase 1	rs17739056	76995205	17	T	C	0.884	0.215	0.012	3.0E-75	cis
281	CANT1	Q8WVQ1	Soluble calcium-activated nucleotidase 1	rs1654425	55538980	19	C	T	0.834	0.104	0.010	1.0E-24	trans
282	CAPG	P40121	Macrophage-capping protein	rs6886	85622059	2	T	C	0.333	0.872	0.008	0.0E+00	cis
282	CAPG	P40121	Macrophage-capping protein	rs9268925	32432969	6	A	C	0.145	0.080	0.008	1.2E-22	trans
282	CAPG	P40121	Macrophage-capping protein	rs4632248	54324995	19	T	G	0.210	0.186	0.007	5.9E-151	trans
283	CAPN3	P20807	Calpain-3	rs710446	186459927	3	C	T	0.409	0.074	0.008	1.4E-21	trans
284	CAPS	Q13938	Calcyphosin	rs113534515	32460798	6	C	G	0.291	0.273	0.008	2.6E-237	trans
284	CAPS	Q13938	Calcyphosin	rs147363394	5915300	19	C	A	0.999	2.202	0.096	1.8E-116	cis
285	CARHSP1	Q9Y2V2	Calcium-regulated heat-stable protein 1	rs12597479	8903589	16	G	T	0.474	0.078	0.007	9.3E-26	cis
286	CASC3	O15234	Protein CASC3	rs1354034	56849749	3	C	T	0.604	0.109	0.008	1.9E-44	trans
286	CASC3	O15234	Protein CASC3	rs11502185	180258	11	C	T	0.260	0.071	0.010	4.9E-13	trans
287	CASP1	P29466	Caspase-1	rs141551019	150297098	7	A	G	0.774	0.066	0.009	3.7E-13	trans
287	CASP1	P29466	Caspase-1	rs34436714	54327313	19	A	C	0.209	0.325	0.009	3.2E-263	trans
288	CASP10	Q92851	Caspase-10	rs7576306	202074528	2	G	A	0.494	0.182	0.008	2.9E-126	cis
288	CASP10	Q92851	Caspase-10	rs6993770	106581528	8	A	T	0.713	0.093	0.008	4.2E-29	trans
289	CASP2	P42575	Caspase-2	rs1354034	56849749	3	C	T	0.604	0.097	0.008	3.7E-37	trans
289	CASP2	P42575	Caspase-2	rs139141690	101499930	7	A	G	0.005	0.382	0.055	5.6E-12	trans
289	CASP2	P42575	Caspase-2	rs342299	106373718	7	C	T	0.545	0.051	0.008	1.1E-11	trans
289	CASP2	P42575	Caspase-2	rs11502185	180258	11	C	T	0.260	0.068	0.010	1.8E-12	trans
290	CASP3	P42574	Caspase-3	rs62339863	185570467	4	A	C	0.739	0.128	0.008	2.7E-51	cis
290	CASP3	P42574	Caspase-3	rs6993770	106581528	8	A	T	0.713	0.078	0.008	1.1E-21	trans
291	CASP4	P49662	Caspase-4	rs117001788	106830448	14	T	C	0.032	0.348	0.023	3.0E-51	trans
292	CASP7	P55210	Caspase-7	rs1354034	56849749	3	C	T	0.604	0.074	0.008	3.8E-22	trans
292	CASP7	P55210	Caspase-7	rs6993770	106581528	8	A	T	0.713	0.057	0.008	6.2E-12	trans
292	CASP7	P55210	Caspase-7	rs141266925	115485285	10	T	C	0.997	1.151	0.070	1.3E-61	cis
293	CASP8	Q14790	Caspase-8	rs34841024	202151140	2	G	T	0.136	0.310	0.011	5.3E-183	cis
293	CASP8	Q14790	Caspase-8	rs1354034	56849749	3	C	T	0.604	0.065	0.007	1.6E-18	trans
293	CASP8	Q14790	Caspase-8	rs3847326	65331859	10	A	G	0.518	0.051	0.007	1.6E-12	trans
294	CASP9	P55211	Caspase-9	rs12024833	15866416	1	A	G	0.475	0.081	0.008	2.8E-26	cis
295	CAT	P04040	Catalase	rs2284368	34469295	11	T	C	0.779	0.184	0.009	1.2E-100	cis
296	CBLIF	P27352	Cobalamin binding intrinsic factor	rs333947	110470764	1	G	A	0.852	0.071	0.010	6.2E-13	trans

296	CBLIF	P27352	Cobalamin binding intrinsic factor	rs2348194	12575590	3	T	A	0.381	0.050	0.007	6.7E-12	trans
296	CBLIF	P27352	Cobalamin binding intrinsic factor	rs13088531	150977363	3	T	A	0.755	0.060	0.008	2.7E-13	trans
296	CBLIF	P27352	Cobalamin binding intrinsic factor	rs1290790	169091569	3	A	T	0.553	0.060	0.007	1.3E-17	trans
296	CBLIF	P27352	Cobalamin binding intrinsic factor	rs72704793	548008	5	A	G	0.330	0.065	0.007	1.8E-18	trans
296	CBLIF	P27352	Cobalamin binding intrinsic factor	rs7718236	180254869	5	G	A	0.174	0.088	0.010	1.6E-20	trans
296	CBLIF	P27352	Cobalamin binding intrinsic factor	rs1634716	30969754	6	G	A	0.850	0.111	0.010	4.9E-27	trans
296	CBLIF	P27352	Cobalamin binding intrinsic factor	rs807309	119965256	6	C	T	0.262	0.465	0.008	0.0E+00	trans
296	CBLIF	P27352	Cobalamin binding intrinsic factor	rs2976397	143764613	8	G	T	0.581	0.054	0.007	1.7E-14	trans
296	CBLIF	P27352	Cobalamin binding intrinsic factor	rs532436	136149830	9	G	A	0.815	0.211	0.009	9.5E-119	trans
296	CBLIF	P27352	Cobalamin binding intrinsic factor	rs56278466	17875857	10	G	T	0.661	0.069	0.007	9.5E-21	trans
296	CBLIF	P27352	Cobalamin binding intrinsic factor	rs2472632	27395875	11	C	A	0.640	0.062	0.007	3.1E-17	trans
296	CBLIF	P27352	Cobalamin binding intrinsic factor	rs7929203	68924739	11	T	C	0.281	0.054	0.008	6.7E-12	trans
296	CBLIF	P27352	Cobalamin binding intrinsic factor	rs4925105	17629285	17	T	C	0.473	0.054	0.007	9.1E-15	trans
296	CBLIF	P27352	Cobalamin binding intrinsic factor	rs61580404	74638043	17	T	G	0.101	0.085	0.012	6.3E-13	trans
296	CBLIF	P27352	Cobalamin binding intrinsic factor	rs584768	49213284	19	A	G	0.533	0.052	0.007	1.3E-13	trans
297	CBLN4	Q9NTU7	Cerebellin-4	rs7684253	57727311	4	C	T	0.447	0.095	0.007	7.1E-38	trans
297	CBLN4	Q9NTU7	Cerebellin-4	rs7116008	126282606	11	A	G	0.433	0.067	0.007	1.4E-19	trans
297	CBLN4	Q9NTU7	Cerebellin-4	rs186021206	7069412	17	A	G	0.006	0.492	0.050	1.2E-22	trans
297	CBLN4	Q9NTU7	Cerebellin-4	rs12624596	54290016	20	A	G	0.286	0.188	0.008	3.3E-113	cis
298	CBS	P35520	Cystathionine beta-synthase	rs4835265	146821410	4	A	C	0.156	0.082	0.010	4.1E-15	trans
298	CBS	P35520	Cystathionine beta-synthase	rs6586281	44478393	21	A	G	0.176	0.156	0.010	4.2E-55	cis
298	CBS	P35520	Cystathionine beta-synthase	rs3747207	44324855	22	A	G	0.216	0.105	0.009	4.6E-30	trans
299	CC2D1A	Q6P1N0	Coiled-coil and C2 domain-containing protein 1A	rs61740117	14024269	19	C	T	0.986	0.325	0.032	2.2E-24	cis
300	CCDC134	Q9H6E4	Coiled-coil domain-containing protein 134	rs10733789	64948684	10	C	T	0.313	0.061	0.008	1.7E-13	trans
300	CCDC134	Q9H6E4	Coiled-coil domain-containing protein 134	rs78290111	42280064	22	T	TG	0.643	0.060	0.008	4.2E-14	cis
301	CCDC50	Q8IVM0	Coiled-coil domain-containing protein 50	rs35380043	191087740	3	A	T	0.952	0.419	0.018	1.6E-125	cis
301	CCDC50	Q8IVM0	Coiled-coil domain-containing protein 50	rs5859867	84161920	4	C	CT	0.714	0.102	0.008	3.1E-34	trans
301	CCDC50	Q8IVM0	Coiled-coil domain-containing protein 50	rs62445350	50278187	7	A	T	0.692	0.071	0.008	1.9E-18	trans
301	CCDC50	Q8IVM0	Coiled-coil domain-containing protein 50	rs2583285	107137412	14	G	A	0.715	0.184	0.009	4.2E-103	trans
302	CCDC80	Q76M96	Coiled-coil domain-containing protein 80	rs6772337	112362188	3	A	G	0.376	0.070	0.007	9.3E-23	cis
302	CCDC80	Q76M96	Coiled-coil domain-containing protein 80	rs13107325	103188709	4	T	C	0.075	0.138	0.013	4.9E-26	trans
302	CCDC80	Q76M96	Coiled-coil domain-containing protein 80	rs41265930	160964135	6	T	C	0.926	0.146	0.013	7.6E-29	trans
302	CCDC80	Q76M96	Coiled-coil domain-containing protein 80	rs2142306	134470631	8	C	T	0.412	0.131	0.007	2.6E-78	trans
302	CCDC80	Q76M96	Coiled-coil domain-containing protein 80	rs186021206	7069412	17	A	G	0.006	0.908	0.047	2.2E-83	trans
303	CCER2	I3L3R5	Coiled-coil domain-containing glutamate-rich protein 2	rs905938	154991389	1	C	T	0.267	0.097	0.008	2.6E-32	trans
303	CCER2	I3L3R5	Coiled-coil domain-containing glutamate-rich protein 2	rs57060715	88594147	2	A	G	0.271	0.058	0.009	1.0E-11	trans
303	CCER2	I3L3R5	Coiled-coil domain-containing glutamate-rich protein 2	rs1561477	111787474	2	T	C	0.108	0.109	0.012	2.1E-20	trans
303	CCER2	I3L3R5	Coiled-coil domain-containing glutamate-rich protein 2	rs17400325	178565913	2	C	T	0.041	0.160	0.018	2.8E-18	trans
303	CCER2	I3L3R5	Coiled-coil domain-containing glutamate-rich protein 2	rs9290890	189196565	3	A	C	0.417	0.061	0.007	2.1E-16	trans
303	CCER2	I3L3R5	Coiled-coil domain-containing glutamate-rich protein 2	rs2870749	94820512	4	A	G	0.565	0.320	0.008	0.0E+00	trans
303	CCER2	I3L3R5	Coiled-coil domain-containing glutamate-rich protein 2	rs13168098	67437942	5	G	A	0.619	0.054	0.008	1.3E-12	trans
303	CCER2	I3L3R5	Coiled-coil domain-containing glutamate-rich protein 2	rs9392840	6901582	6	T	C	0.272	0.060	0.008	6.8E-13	trans
303	CCER2	I3L3R5	Coiled-coil domain-containing glutamate-rich protein 2	rs4837917	124709309	9	T	C	0.707	0.096	0.008	4.0E-33	trans
303	CCER2	I3L3R5	Coiled-coil domain-containing glutamate-rich protein 2	rs7083373	18579862	10	C	G	0.516	0.060	0.007	1.3E-16	trans
303	CCER2	I3L3R5	Coiled-coil domain-containing glutamate-rich protein 2	rs11148502	61165538	13	T	A	0.488	0.100	0.007	1.5E-42	trans
303	CCER2	I3L3R5	Coiled-coil domain-containing glutamate-rich protein 2	rs879627	88522541	16	G	C	0.437	0.062	0.007	5.4E-17	trans
303	CCER2	I3L3R5	Coiled-coil domain-containing glutamate-rich protein 2	rs7236418	10726142	18	C	G	0.557	0.052	0.007	1.1E-12	trans
303	CCER2	I3L3R5	Coiled-coil domain-containing glutamate-rich protein 2	rs2596606	77162816	18	G	A	0.182	0.095	0.010	2.0E-23	trans
303	CCER2	I3L3R5	Coiled-coil domain-containing glutamate-rich protein 2	rs190921775	39402296	19	G	T	0.995	0.917	0.053	9.5E-68	cis
303	CCER2	I3L3R5	Coiled-coil domain-containing glutamate-rich protein 2	rs5751435	43513156	22	T	C	0.124	0.078	0.011	2.1E-12	trans
304	CCL11	P51671	Eotaxin	rs12075	159175354	1	A	G	0.583	0.080	0.007	2.2E-29	trans
304	CCL11	P51671	Eotaxin	rs2228467	42906116	3	C	T	0.061	0.410	0.015	6.7E-168	trans
304	CCL11	P51671	Eotaxin	rs1979671	46274215	3	C	T	0.313	0.079	0.008	1.3E-24	trans
304	CCL11	P51671	Eotaxin	rs1354034	56849749	3	T	C	0.396	0.052	0.007	7.4E-13	trans
304	CCL11	P51671	Eotaxin	rs2516471	31400292	6	G	A	0.936	0.116	0.015	1.6E-15	trans
304	CCL11	P51671	Eotaxin	rs2868169	75464349	7	C	T	0.104	0.202	0.011	8.5E-70	trans
304	CCL11	P51671	Eotaxin	rs4602861	106590706	8	A	G	0.726	0.056	0.008	1.8E-12	trans
304	CCL11	P51671	Eotaxin	rs7080386	65048306	10	A	C	0.412	0.066	0.007	2.1E-20	trans
304	CCL11	P51671	Eotaxin	rs140905292	32515534	17	G	A	0.776	0.121	0.009	2.8E-45	cis
304	CCL11	P51671	Eotaxin	rs1613662	55536595	19	A	G	0.834	0.092	0.009	3.0E-22	trans
304	CCL11	P51671	Eotaxin	rs5980962	69764100	X	C	A	0.336	0.042	0.006	1.6E-11	trans
305	CCL13	Q99616	C-C motif chemokine 13	rs12075	159175354	1	A	G	0.583	0.223	0.007	9.9E-206	trans
305	CCL13	Q99616	C-C motif chemokine 13	rs13412535	224874874	2	G	A	0.769	0.070	0.008	1.4E-16	trans
305	CCL13	Q99616	C-C motif chemokine 13	rs12495098	46342502	3	G	T	0.069	0.105	0.014	1.3E-13	trans
305	CCL13	Q99616	C-C motif chemokine 13	rs2522051	131797578	5	C	T	0.452	0.052	0.007	4.2E-13	trans
305	CCL13	Q99616	C-C motif chemokine 13	rs3117110	32340176	6	T	C	0.213	0.066	0.009	5.9E-14	trans
305	CCL13	Q99616	C-C motif chemokine 13	rs826732	59742639	8	C	G	0.499	0.051	0.007	2.8E-12	trans
305	CCL13	Q99616	C-C motif chemokine 13	rs7080386	65048306	10	A	C	0.412	0.073	0.007	1.1E-23	trans
305	CCL13	Q99616	C-C motif chemokine 13	rs59001897	65160392	15	A	T	0.175	0.073	0.010	1.1E-14	trans
305	CCL13	Q99616	C-C motif chemokine 13	rs12445050	81870969	16	T	C	0.138	0.080	0.010	8.3E-15	trans
305	CCL13	Q99616	C-C motif chemokine 13	rs3136674	32682616	17	T	C	0.946	0.462	0.016	9.9E-179	cis
305	CCL13	Q99616	C-C motif chemokine 13	rs892090	55539072	19	G	T	0.834	0.136	0.010	5.3E-45	trans
306	CCL14	Q16627	C-C motif chemokine 14	rs13962	159175527	1	A	G	0.177	0.169	0.009	2.6E-72	trans
306	CCL14	Q16627	C-C motif chemokine 14	rs13412535	224874874	2	G	A	0.769	0.066	0.008	1.9E-15	trans
306	CCL14	Q16627	C-C motif chemokine 14	rs1354034	56849749	3	T	C	0.396	0.065	0.007	1.2E-19	trans
306	CCL14	Q16627	C-C motif chemokine 14	rs6993770	106581528	8	A	T	0.714	0.073	0.008	3.9E-21	trans
306	CCL14	Q16627	C-C motif chemokine 14	rs9903158	34312337	17	T	C	0.947	1.013	0.017	0.0E+00	cis
306	CCL14	Q16627	C-C motif chemokine 14	rs1671152	55526345	19	G	T	0.841	0.103	0.010	2.1E-26	trans
306	CCL14	Q16627	C-C motif chemokine 14	rs182320903	69721267	X	C	G	0.168	0.057	0.008	1.1E-11	trans
307	CCL15	Q16663	C-C motif chemokine 15	rs28929474	94844947	14	T	C	0.021	0.261	0.022	2.6E-33	trans
307	CCL15	Q16663	C-C motif chemokine 15	rs601338	49206674	19	G	A	0.491	0.181	0.006	5.7E-184	trans
308	CCL16	O15467	C-C motif chemokine 16	rs11127048	27752463	2	G	A	0.382	0.043	0.006	5.5E-12	trans
308	CCL16	O15467	C-C motif chemokine 16	rs1047891	211540507	2	C	A	0.687	0.049	0.006	3.7E-14	trans
308	CCL16	O15467	C-C motif chemokine 16	rs3134984	32645029	6	T	A	0.790	0.074	0.008	2.4E-21	trans
308	CCL16	O15467	C-C motif chemokine 16	rs34958196	73043101	7	G	A	0.871	0.067	0.009	3.7E-14	trans
308	CCL16	O15467	C-C motif chemokine 16	rs112689088	34307457	17	T	C	0.904	1.332	0.012	0.0E+00	cis
309	CCL17	Q92583	C-C motif chemokine 17	rs12075	159175354	1	A	G	0.583	0.054	0.007	1.1E-13	trans
309	CCL17	Q92583	C-C motif chemokine 17	rs142289859	16954941	3	T	C	0.967	0.557	0.021	3.3E-162	trans
309	CCL17	Q92583	C-C motif chemokine 17	rs116135711	33072469	3	T	C	0.964	0.171	0.020	1.9E-17	trans
309	CCL17	Q92583	C-C motif chemokine 17	rs2228467	42906116	3	C	T	0.061	0.425	0.015	4.2E-168	trans
309	CCL17	Q92583	C-C motif chemokine 17	rs11925560	47259460	3	T	A	0.388	0.061	0.008	8.7E-16	trans
309	CCL17	Q92583	C-C motif chemokine 17	rs771327	101534500	3	G	A	0.559	0.056	0.007	6.7E-14	trans
309	CCL17	Q92583	C-C motif chemokine 17	rs3804749	122833003	3	C	T	0.408	0.053	0.008	3.9E-12	trans
309	CCL17	Q92583	C-C motif chemokine 17	rs2073643	131723288	5	T	C	0.448	0.059	0.007	2.6E-16	trans
309	CCL17	Q92583	C-C motif chemokine 17	rs3763287	31370329	6	C	A	0.935	0.102	0.015	2.1E-12	trans
309	CCL17	Q92583	C-C motif chemokine 17	rs6993770	106581528	8	A	T	0.713	0.132	0.008	4.0E-62	trans
309	CCL17	Q92583	C-C motif chemokine 17	rs61469632	135861990	9	T	C	0.937	0.114	0.015	9.0E-15	trans
309	CCL17	Q92583	C-C motif chemokine 17	rs10886430	121010256	10	A	G	0.873	0.365	0.011	7.7E-239	trans
309	CCL17	Q92583	C-C motif chemokine 17	rs9302690	57442968	16	A	G	0.081	0.381	0.013	7.8E-179	cis
309	CCL17	Q92583	C-C motif chemokine 17	rs1654425	55538980	19	C	T	0.834	0.085	0.010	9.4E-19	trans
310	CCL18	P55774	C-C motif chemokine 18	rs6715862	42328575	2	A	G	0.498	0.077	0.007	8.2E-29	trans
310	CCL18	P55774	C-C motif chemokine 18	rs2015086	34391617	17	G	A	0.133	0.706	0.011	0.0E+00	cis
310	CCL18	P55774	C-C motif chemokine 18	rs56298400	39124802	20	A	G	0.083	0.089	0.012	8.7E-13	trans
311	CCL19	Q99731	C-C motif chemokine 19	rs2476601	114377568	1	A	G	0.101	0.113	0.012	1.4E-20	trans
311	CCL19	Q99731	C-C motif chemokine 19	rs2305625	132241510	3	C	T	0.888	0.148	0.012	3.8E-37	trans
311	CCL19	Q99731	C-C motif chemokine 19	rs201980192	32609063	6	T	C	0.019	0.888	0.028	1.9E-228	trans
311	CCL19	Q99731	C-C motif chemokine 19	rs10972202	34707814	9	T	C	0.123	0.105	0.011	7.7E-21	cis
311	CCL19	Q99731	C-C motif chemokine 19	rs2412973	30529631	22	C	A	0.557	0.053	0.007	3.9E-13	trans
312	CCL2	P13500	C-C motif chemokine 2	rs12075	159175354	1	A	G	0.583	0.247	0.007	2.7E-251	trans
312	CCL2	P13500	C-C motif chemokine 2	rs2228467	42906116	3	C	T	0.061	0.137	0.015	2.9E-20	trans
312	CCL2	P13500	C-C motif chemokine 2	rs10510751	46346025	3	G	T	0.071	0.281	0.014	1.1E-89	trans
312	CCL2	P13500	C-C motif chemokine 2	rs4541868	106590705	8	C	A	0.726	0.057	0.008	1.3E-12	trans
312	CCL2	P13500	C-C motif chemokine 2	rs7080386	65048306	10	A	C	0.412	0.067	0.007	1.5E-20	trans
312	CCL2	P13500	C-C motif chemokine 2	rs76428106	28604007	13	T	C	0.987	0.277	0.033	2.2E-17	trans
312	CCL2	P13500	C-C motif chemokine 2	rs12601658	32533423	17	T	A	0.771	0.068	0.008	8.6E-16	cis
312	CCL2	P13500	C-C motif chemokine 2	rs1671152	55526345	19	G	T	0.841	0.096	0.010	8.9E-23	trans
312	CCL2	P13500	C-C motif chemokine 2	rs5980952	69710823	X	T	C	0.203	0.050	0.007	6.0E-12	trans
313	CCL20	P78556	C-C motif chemokine 20	rs1395338	228648323	2	A	G	0.601	0.093	0.008	1.7E-34	cis
313	CCL20	P78556	C-C motif chemokine 20	rs3129754	32583046	6	G	A	0.420	0.082	0.008	4.5E-27	trans
313	CCL20	P78556	C-C motif chemokine 20	rs742493	40998167	6	T	C	0.887	0.118	0.012	1.3E-23	trans
313	CCL20	P78556	C-C motif chemokine 20	rs77542162	67081278	17	G	A	0.023	0.270	0.025	2.0E-27	trans
313	CCL20	P78556	C-C motif chemokine 20	rs117900576	72761195	17	G	C	0.034	0.172	0.021	5.5E-17	trans
314	CCL21	O00585	C-C motif chemokine 21	rs4671127	65562970	2	C	G	0.665	0.054	0.008	4.4E-12	trans
314	CCL21	O00585	C-C motif chemokine 21	rs1354034	56849749	3	T	C	0.396	0.065	0.008	5.2E-18	trans
314	CCL21	O00585	C-C motif chemokine 21	rs11924792	132240100	3	A	C	0.888	0.177	0.012	9.3E-52	trans
314	CCL21	O00585	C-C motif chemokine 21	rs885122	115331335	5	A	G	0.605	0.062	0.008	6.1E-16	trans
314	CCL21	O00585	C-C motif chemokine 21	rs17764730	127357526	5	C	T	0.751	0.087	0.009	3.3E-24	trans
314	CCL21	O00585	C-C motif chemokine 21	rs12722013	33048460	6	C	T	0.047	0.315	0.017	3.4E-73	trans
314	CCL21	O00585	C-C motif chemokine 21	rs10814138	34723035	9	C	A	0.683	0.103	0.008	1.5E-38	cis
314	CCL21	O00585	C-C motif chemokine 21	rs12935829	54435965	16	G	T	0.344	0.053	0.008	1.1E-11	trans
314	CCL21	O00585	C-C motif chemokine 21	rs62054652	90125822	16	C	T	0.929	0.107	0.014	1.1E-13	trans
314	CCL21	O00585	C-C motif chemokine 21	rs2412973	30529631	22	C	A	0.557	0.070	0.007	3.3E-21	trans
315	CCL22	O00626	C-C motif chemokine 22	rs13098877	188133163	3	C	T	0.556	0.052	0.008	4.1E-12	trans
315	CCL22	O00626	C-C motif chemokine 22	rs9296027	32393062	6	G	C	0.122	0.091	0.011	9.3E-16	trans
315	CCL22	O00626	C-C motif chemokine 22	rs1504215	91006227	6	G	A	0.649	0.065	0.008	9.9E-17	trans
315	CCL22	O00626	C-C motif chemokine 22	rs41398344	57394393	16	T	C	0.990	0.824	0.038	1.7E-104	cis
315	CCL22	O00626	C-C motif chemokine 22	rs77542162	67081278	17	G	A	0.023	0.512	0.025	1.1E-92	trans
315	CCL22	O00626	C-C motif chemokine 22	rs11670056	18589943	19	T	C	0.074	0.107	0.014	4.0E-14	trans
316	CCL23	P55773	C-C motif chemokine 23	rs61804164	161623025	1	G	C	0.876	0.082	0.011	3.6E-14	trans
316	CCL23	P55773	C-C motif chemokine 23	rs780093	27742603	2	T	C	0.380	0.066	0.007	7.8E-20	trans
316	CCL23	P55773	C-C motif chemokine 23	rs142021145	71756519	5	A	C	0.004	0.580	0.059	1.3E-22	trans
316	CCL23	P55773	C-C motif chemokine 23	rs9260620	29923091	6	T	G	0.671	0.055	0.008	2.8E-13	trans
316	CCL23	P55773	C-C motif chemokine 23	rs964184	116648917	11	G	C	0.133	0.104	0.010	7.5E-24	trans

316	CCL23	P55773	C-C motif chemokine 23	rs11545007	30544041	16	C	A	0.991	0.353	0.040	1.9E-18	trans
316	CCL23	P55773	C-C motif chemokine 23	rs712048	34326215	17	C	A	0.869	0.569	0.011	0.0E+00	cis
316	CCL23	P55773	C-C motif chemokine 23	rs1065853	45413233	19	T	G	0.081	0.113	0.013	3.5E-18	trans
317	CCL24	O00175	C-C motif chemokine 24	rs12083692	115032389	1	A	T	0.741	0.052	0.005	5.7E-24	trans
317	CCL24	O00175	C-C motif chemokine 24	rs2228467	42906116	3	C	T	0.061	0.173	0.009	1.1E-74	trans
317	CCL24	O00175	C-C motif chemokine 24	rs115216147	70665526	5	T	A	0.933	0.076	0.009	2.7E-16	trans
317	CCL24	O00175	C-C motif chemokine 24	rs111872865	360124	6	A	G	0.013	0.152	0.022	4.6E-12	trans
317	CCL24	O00175	C-C motif chemokine 24	rs2024050	75460393	7	A	G	0.105	1.375	0.012	0.0E+00	cis
317	CCL24	O00175	C-C motif chemokine 24	rs2737246	116659578	8	G	C	0.718	0.037	0.005	1.6E-13	trans
317	CCL24	O00175	C-C motif chemokine 24	rs13294683	82279767	9	T	C	0.101	0.071	0.007	4.3E-21	trans
317	CCL24	O00175	C-C motif chemokine 24	rs10769256	47378396	11	T	C	0.400	0.060	0.005	1.2E-37	trans
317	CCL24	O00175	C-C motif chemokine 24	rs201510256	54792639	11	C	T	0.526	0.043	0.005	2.0E-15	trans
317	CCL24	O00175	C-C motif chemokine 24	rs8012229	64911562	14	T	C	0.912	0.056	0.008	3.9E-12	trans
317	CCL24	O00175	C-C motif chemokine 24	rs2939849	90619082	15	G	C	0.396	0.056	0.005	2.1E-33	trans
317	CCL24	O00175	C-C motif chemokine 24	rs1121985	79363079	16	C	A	0.622	0.048	0.005	5.1E-24	trans
317	CCL24	O00175	C-C motif chemokine 24	rs113374757	47256542	19	C	T	0.834	0.042	0.006	1.1E-11	trans
317	CCL24	O00175	C-C motif chemokine 24	rs11480320	52532696	20	AT	A	0.584	0.034	0.005	1.3E-12	trans
318	CCL25	O15444	C-C motif chemokine 25	rs9275576	32679326	6	T	C	0.144	0.062	0.008	2.5E-13	trans
318	CCL25	O15444	C-C motif chemokine 25	rs78061563	10579332	8	A	G	0.073	0.094	0.011	2.2E-16	trans
318	CCL25	O15444	C-C motif chemokine 25	rs118062058	42103378	8	A	G	0.944	0.150	0.013	5.4E-31	trans
318	CCL25	O15444	C-C motif chemokine 25	rs186021206	7069412	17	A	G	0.006	0.387	0.041	3.0E-21	trans
318	CCL25	O15444	C-C motif chemokine 25	rs7223866	9785346	17	G	C	0.330	0.044	0.006	6.1E-12	trans
318	CCL25	O15444	C-C motif chemokine 25	rs112001035	66823805	17	G	A	0.939	0.100	0.013	2.2E-15	trans
318	CCL25	O15444	C-C motif chemokine 25	rs2032887	8121360	19	G	A	0.237	0.770	0.009	0.0E+00	cis
318	CCL25	O15444	C-C motif chemokine 25	rs601338	49206674	19	G	A	0.491	0.390	0.007	0.0E+00	trans
318	CCL25	O15444	C-C motif chemokine 25	rs34710186	30987905	20	TA	T	0.811	0.067	0.008	1.1E-17	trans
319	CCL26	Q9Y258	C-C motif chemokine 26	rs12075	159175354	1	A	G	0.583	0.192	0.008	1.7E-144	trans
319	CCL26	Q9Y258	C-C motif chemokine 26	rs13412535	224874874	2	G	A	0.769	0.061	0.009	5.3E-12	trans
319	CCL26	Q9Y258	C-C motif chemokine 26	rs17622656	131820997	5	G	A	0.611	0.053	0.008	4.0E-12	trans
319	CCL26	Q9Y258	C-C motif chemokine 26	rs41463245	75401263	7	C	T	0.991	1.014	0.039	6.8E-147	cis
319	CCL26	Q9Y258	C-C motif chemokine 26	rs6993770	106581528	8	A	T	0.713	0.067	0.008	2.1E-16	trans
319	CCL26	Q9Y258	C-C motif chemokine 26	rs10761731	65027610	10	T	A	0.415	0.057	0.007	2.3E-14	trans
319	CCL26	Q9Y258	C-C motif chemokine 26	rs1654425	55538980	19	C	T	0.834	0.096	0.010	7.0E-22	trans
320	CCL27	Q9Y4X3	C-C motif chemokine 27	rs1150756	32035694	6	C	T	0.869	0.095	0.011	2.6E-19	trans
320	CCL27	Q9Y4X3	C-C motif chemokine 27	rs118039278	160985526	6	A	G	0.082	0.108	0.013	1.7E-16	trans
320	CCL27	Q9Y4X3	C-C motif chemokine 27	rs2070074	34649442	9	A	G	0.903	0.328	0.012	1.4E-159	cis
320	CCL27	Q9Y4X3	C-C motif chemokine 27	rs964184	116648917	11	G	C	0.133	0.106	0.010	5.1E-24	trans
320	CCL27	Q9Y4X3	C-C motif chemokine 27	rs77542162	67081278	17	G	A	0.023	0.306	0.024	2.2E-37	trans
320	CCL27	Q9Y4X3	C-C motif chemokine 27	rs7412	45412079	19	T	C	0.082	0.148	0.013	1.0E-29	trans
320	CCL27	Q9Y4X3	C-C motif chemokine 27	rs2569491	51584916	19	G	A	0.702	0.058	0.008	1.2E-13	trans
320	CCL27	Q9Y4X3	C-C motif chemokine 27	rs5942986	109849194	X	C	T	0.624	0.044	0.006	1.9E-12	trans
321	CCL28	Q9NRJ3	C-C motif chemokine 28	rs7535275	150354210	1	A	G	0.868	0.078	0.011	4.7E-13	trans
321	CCL28	Q9NRJ3	C-C motif chemokine 28	rs41267433	156874421	1	G	C	0.111	0.085	0.012	3.3E-13	trans
321	CCL28	Q9NRJ3	C-C motif chemokine 28	rs3732083	207041053	2	T	C	0.570	0.182	0.007	1.2E-131	trans
321	CCL28	Q9NRJ3	C-C motif chemokine 28	rs13412535	224874874	2	G	A	0.769	0.150	0.009	2.4E-65	trans
321	CCL28	Q9NRJ3	C-C motif chemokine 28	rs2278668	122835232	3	T	C	0.407	0.068	0.008	1.1E-19	trans
321	CCL28	Q9NRJ3	C-C motif chemokine 28	rs55874224	142820470	3	C	G	0.392	0.067	0.007	2.8E-19	trans
321	CCL28	Q9NRJ3	C-C motif chemokine 28	rs11734132	6891519	4	G	C	0.826	0.065	0.010	1.3E-11	trans
321	CCL28	Q9NRJ3	C-C motif chemokine 28	rs114694170	88180196	5	C	T	0.060	0.122	0.016	4.6E-15	trans
321	CCL28	Q9NRJ3	C-C motif chemokine 28	rs77024756	32498558	6	A	G	0.297	0.072	0.009	8.3E-15	trans
321	CCL28	Q9NRJ3	C-C motif chemokine 28	rs1331309	135406178	6	G	T	0.261	0.066	0.008	3.8E-15	trans
321	CCL28	Q9NRJ3	C-C motif chemokine 28	rs139141690	101499930	7	G	A	0.995	0.648	0.054	5.0E-33	trans
321	CCL28	Q9NRJ3	C-C motif chemokine 28	rs2519093	136141870	9	C	T	0.816	0.173	0.009	1.4E-74	trans
321	CCL28	Q9NRJ3	C-C motif chemokine 28	rs73000929	113953622	11	G	A	0.963	0.247	0.019	1.4E-37	trans
321	CCL28	Q9NRJ3	C-C motif chemokine 28	rs4965385	101996034	15	A	G	0.716	0.057	0.008	1.6E-12	trans
321	CCL28	Q9NRJ3	C-C motif chemokine 28	rs892090	55539072	19	G	T	0.834	0.080	0.010	3.8E-16	trans
321	CCL28	Q9NRJ3	C-C motif chemokine 28	rs71329093	36392564	21	A	G	0.944	0.130	0.016	3.2E-16	trans
322	CCL3	P10147	C-C motif chemokine 3	rs60939770	46337606	3	A	G	0.684	0.054	0.008	1.1E-12	trans
322	CCL3	P10147	C-C motif chemokine 3	rs1354034	56849749	3	T	C	0.396	0.051	0.007	8.6E-13	trans
322	CCL3	P10147	C-C motif chemokine 3	rs13153461	138852369	5	A	G	0.743	0.072	0.008	2.1E-19	trans
322	CCL3	P10147	C-C motif chemokine 3	rs9268644	32408044	6	C	A	0.547	0.055	0.007	3.8E-15	trans
322	CCL3	P10147	C-C motif chemokine 3	rs1634486	34410091	17	C	A	0.224	0.420	0.009	0.0E+00	cis
323	CCL4	P13236	C-C motif chemokine 4	rs113341849	46384204	3	A	G	0.119	0.615	0.011	0.0E+00	trans
323	CCL4	P13236	C-C motif chemokine 4	rs9405083	31319489	6	C	T	0.749	0.057	0.008	1.1E-12	trans
323	CCL4	P13236	C-C motif chemokine 4	rs13338037	81874986	16	G	C	0.141	0.079	0.010	1.4E-15	trans
323	CCL4	P13236	C-C motif chemokine 4	rs1015673	34477386	17	C	T	0.575	0.314	0.008	0.0E+00	cis
323	CCL4	P13236	C-C motif chemokine 4	rs892090	55539072	19	G	T	0.834	0.070	0.009	2.8E-14	trans
324	CCL5	P13501	C-C motif chemokine 5	rs1354034	56849749	3	T	C	0.396	0.071	0.007	2.1E-21	trans
324	CCL5	P13501	C-C motif chemokine 5	rs6961069	80218961	7	T	C	0.402	0.063	0.008	3.3E-17	trans
324	CCL5	P13501	C-C motif chemokine 5	rs6993770	106581528	8	A	T	0.714	0.093	0.008	4.6E-30	trans
324	CCL5	P13501	C-C motif chemokine 5	rs35754228	38203811	10	A	AT	0.676	0.077	0.008	6.3E-22	trans
324	CCL5	P13501	C-C motif chemokine 5	rs10761731	65027610	10	T	A	0.415	0.128	0.007	2.5E-65	trans
324	CCL5	P13501	C-C motif chemokine 5	rs12445050	81870969	16	T	C	0.138	0.081	0.011	2.8E-14	trans
324	CCL5	P13501	C-C motif chemokine 5	rs2107538	34207780	17	C	T	0.820	0.220	0.010	6.8E-115	cis
324	CCL5	P13501	C-C motif chemokine 5	rs1654425	55538980	19	C	T	0.835	0.126	0.010	6.2E-37	trans
324	CCL5	P13501	C-C motif chemokine 5	rs3827978	19281291	20	T	C	0.354	0.064	0.008	9.9E-17	trans
325	CCL7	P80098	C-C motif chemokine 7	rs12075	159175354	1	G	A	0.417	0.272	0.007	7.9E-294	trans
325	CCL7	P80098	C-C motif chemokine 7	rs2228467	42906116	3	C	T	0.061	0.301	0.015	9.1E-88	trans
325	CCL7	P80098	C-C motif chemokine 7	rs28380853	31311604	6	A	T	0.145	0.094	0.011	8.3E-17	trans
325	CCL7	P80098	C-C motif chemokine 7	rs6505397	32517996	17	A	T	0.423	0.105	0.007	4.6E-47	cis
325	CCL7	P80098	C-C motif chemokine 7	rs1654425	55538980	19	C	T	0.834	0.081	0.010	1.0E-16	trans
326	CCL8	P80075	C-C motif chemokine 8	rs12075	159175354	1	G	A	0.417	0.200	0.006	8.9E-223	trans
326	CCL8	P80075	C-C motif chemokine 8	rs35173808	31326148	6	C	A	0.904	0.088	0.010	2.8E-17	trans
326	CCL8	P80075	C-C motif chemokine 8	rs2190504	30507380	7	C	T	0.444	0.043	0.006	4.0E-12	trans
326	CCL8	P80075	C-C motif chemokine 8	rs2737245	116658583	8	G	T	0.718	0.053	0.007	2.0E-14	trans
326	CCL8	P80075	C-C motif chemokine 8	rs61469632	135861990	9	T	C	0.937	0.086	0.013	1.2E-11	trans
326	CCL8	P80075	C-C motif chemokine 8	rs3740688	47380340	11	G	T	0.457	0.046	0.006	1.7E-13	trans
326	CCL8	P80075	C-C motif chemokine 8	rs1133763	32647831	17	A	C	0.846	1.074	0.010	0.0E+00	cis
326	CCL8	P80075	C-C motif chemokine 8	rs62125968	3422261	19	A	G	0.235	0.054	0.007	1.3E-13	trans
326	CCL8	P80075	C-C motif chemokine 8	rs892090	55539072	19	G	T	0.834	0.089	0.008	1.1E-26	trans
327	CCN1	O00622	CCN family member 1	rs4949896	86062336	1	G	T	0.738	0.118	0.008	9.9E-46	cis
327	CCN1	O00622	CCN family member 1	rs1354034	56849749	3	T	C	0.395	0.058	0.007	6.3E-15	trans
327	CCN1	O00622	CCN family member 1	rs55730499	161005610	6	T	C	0.082	0.117	0.013	1.2E-18	trans
327	CCN1	O00622	CCN family member 1	rs328	19819724	8	C	G	0.899	0.126	0.012	1.4E-25	trans
327	CCN1	O00622	CCN family member 1	rs7848055	95115492	9	A	C	0.671	0.082	0.008	4.8E-26	trans
327	CCN1	O00622	CCN family member 1	rs964184	116648917	11	G	C	0.133	0.133	0.011	3.0E-35	trans
327	CCN1	O00622	CCN family member 1	rs11846959	94846316	14	A	G	0.253	0.080	0.008	1.2E-21	trans
327	CCN1	O00622	CCN family member 1	rs186021206	7069412	17	A	G	0.006	0.419	0.050	1.1E-16	trans
327	CCN1	O00622	CCN family member 1	rs72654473	45414399	19	A	C	0.109	0.122	0.012	2.2E-25	trans
328	CCN2	P29279	CCN family member 2	rs1434282	199010721	1	C	T	0.273	0.078	0.008	6.4E-21	trans
328	CCN2	P29279	CCN family member 2	rs13412535	224874874	2	G	A	0.769	0.060	0.009	9.6E-12	trans
328	CCN2	P29279	CCN family member 2	rs1354034	56849749	3	T	C	0.396	0.097	0.007	3.1E-38	trans
328	CCN2	P29279	CCN family member 2	rs28625045	102772190	4	G	A	0.607	0.059	0.008	5.1E-15	trans
328	CCN2	P29279	CCN family member 2	rs2631367	131705458	5	G	C	0.518	0.055	0.007	7.0E-14	trans
328	CCN2	P29279	CCN family member 2	rs2516471	31400292	6	G	A	0.936	0.114	0.016	2.3E-13	trans
328	CCN2	P29279	CCN family member 2	rs1917342	71349754	6	A	G	0.696	0.057	0.008	3.7E-12	trans
328	CCN2	P29279	CCN family member 2	rs7767139	132292781	6	C	G	0.784	0.355	0.009	0.0E+00	cis
328	CCN2	P29279	CCN family member 2	rs13236689	80236014	7	G	T	0.408	0.066	0.007	1.0E-18	trans
328	CCN2	P29279	CCN family member 2	rs4631576	100705210	9	T	C	0.361	0.058	0.008	2.3E-14	trans
328	CCN2	P29279	CCN family member 2	rs10761731	65027610	10	T	A	0.415	0.122	0.007	5.6E-60	trans
328	CCN2	P29279	CCN family member 2	rs59001897	65160392	15	A	T	0.174	0.076	0.010	9.7E-15	trans
328	CCN2	P29279	CCN family member 2	rs11075045	9034716	16	G	A	0.182	0.067	0.010	2.4E-12	trans
328	CCN2	P29279	CCN family member 2	rs12445050	81870969	16	T	C	0.138	0.110	0.011	3.4E-25	trans
328	CCN2	P29279	CCN family member 2	rs1654425	55538980	19	C	T	0.834	0.152	0.010	1.4E-52	trans
328	CCN2	P29279	CCN family member 2	rs6081565	19287904	20	A	G	0.352	0.066	0.008	7.4E-18	trans
328	CCN2	P29279	CCN family member 2	rs56045941	36421331	21	C	T	0.170	0.073	0.010	1.5E-13	trans
328	CCN2	P29279	CCN family member 2	rs28391150	69673464	X	T	C	0.195	0.073	0.008	3.3E-21	trans
328	CCN2	P29279	CCN family member 2	rs5921632	99955419	X	G	T	0.352	0.045	0.006	8.5E-13	trans
329	CCN3	P48745	CCN family member 3	rs2279112	120429024	8	G	A	0.741	0.274	0.008	1.9E-253	cis
329	CCN3	P48745	CCN family member 3	rs7827593	134024462	8	A	G	0.135	0.078	0.010	3.4E-14	trans
330	CCN4	O95388	CCN family member 4	rs2274319	156450873	1	T	C	0.347	0.051	0.007	5.9E-13	trans
330	CCN4	O95388	CCN family member 4	rs68066031	224880498	2	T	C	0.770	0.096	0.008	4.2E-33	trans
330	CCN4	O95388	CCN family member 4	rs1354034	56849749	3	T	C	0.396	0.079	0.007	4.1E-31	trans
330	CCN4	O95388	CCN family member 4	rs10033707	121708442	4	T	G	0.663	0.051	0.007	1.3E-12	trans
330	CCN4	O95388	CCN family member 4	rs114694170	88180196	5	C	T	0.060	0.129	0.014	1.6E-19	trans
330	CCN4	O95388	CCN family member 4	rs139141690	101499930	7	A	G	0.005	0.366	0.050	1.5E-13	trans
330	CCN4	O95388	CCN family member 4	rs4541868	106590705	8	C	A	0.726	0.063	0.008	5.6E-14	trans
330	CCN4	O95388	CCN family member 4	rs12156037	134204458	8	A	G	0.646	0.439	0.008	0.0E+00	cis
330	CCN4	O95388	CCN family member 4	rs150813342	135864513	9	T	C	0.008	0.294	0.038	6.7E-15	trans
330	CCN4	O95388	CCN family member 4	rs11591571	104342804	10	A	G	0.341	0.057	0.007	9.2E-16	trans
330	CCN4	O95388	CCN family member 4	rs10424665	32895631	19	C	A	0.171	0.069	0.009	8.3E-15	trans
330	CCN4	O95388	CCN family member 4	rs1654425	55538980	19	C	T	0.834	0.079	0.009	2.3E-18	trans
331	CCN5	O76076	CCN family member 5	rs4859682	77410318	4	A	C	0.457	0.047	0.007	3.4E-12	trans
332	CCNE1	P24864	G1/S-specific cyclin-E1	rs139299944	32602665	6	CT	C	0.344	0.110	0.008	2.3E-41	trans
333	CCS	O14618	Copper chaperone for superoxide dismutase	rs498045	66362997	11	G	A	0.526	0.481	0.007	0.0E+00	cis
334	CD101	Q93033	Immunoglobulin superfamily member 2	rs12130298	117544331	1	A	C	0.925	1.278	0.014	0.0E+00	cis
334	CD101	Q93033	Immunoglobulin superfamily member 2	rs9271374	32587041	6	G	A	0.194	0.096	0.007	8.7E-39	trans
334	CD101	Q93033	Immunoglobulin superfamily member 2	rs12667978	128777461	7	C	T	0.429	0.041	0.006	2.6E-12	trans
334	CD101	Q93033	Immunoglobulin superfamily member 2	rs2082209	71195853	10	G	A	0.445	0.068	0.006	8.6E-32	trans
334	CD101	Q93033	Immunoglobulin superfamily member 2	rs4893	48833608	19	A	G	0.960	0.170	0.015	6.9E-31	trans
334	CD101	Q93033	Immunoglobulin superfamily member 2	rs150358287	3687141	20	A	C	0.012	0.333	0.027	6.4E-36	trans
335	CD109	Q6YHK3	CD109 antigen	rs1260326	27730940	2	C	T	0.608	0.047	0.006	9.8E-16	trans
335	CD109	Q6YHK3	CD109 antigen	rs8176749	136131188	9	T	C	0.061	0.367	0.012	6.7E-205	trans

335	CD109	Q6YHK3	CD109 antigen	rs56278466	17875857	10	G	T	0.661	0.096	0.006	4.0E-57	trans
335	CD109	Q6YHK3	CD109 antigen	rs186021206	7069412	17	A	G	0.006	0.554	0.039	5.9E-46	trans
335	CD109	Q6YHK3	CD109 antigen	rs868875	7831166	19	A	G	0.699	0.049	0.006	4.4E-15	trans
336	CD14	P08571	Monocyte differentiation antigen CD14	rs3213563	172412995	1	C	T	0.559	0.096	0.007	1.0E-38	trans
336	CD14	P08571	Monocyte differentiation antigen CD14	rs5744441	140016847	5	G	A	0.767	0.321	0.009	2.6E-288	cis
336	CD14	P08571	Monocyte differentiation antigen CD14	rs28601761	126500031	8	C	G	0.581	0.075	0.008	2.8E-23	trans
336	CD14	P08571	Monocyte differentiation antigen CD14	rs56278466	17875857	10	G	T	0.661	0.090	0.008	4.4E-31	trans
336	CD14	P08571	Monocyte differentiation antigen CD14	rs9414801	65149089	10	G	A	0.467	0.062	0.007	8.1E-17	trans
336	CD14	P08571	Monocyte differentiation antigen CD14	rs149394327	64228995	17	C	G	0.030	0.171	0.022	2.8E-15	trans
337	CD160	O95971	CD160 antigen	rs6699680	25238631	1	A	G	0.585	0.055	0.007	1.8E-13	trans
337	CD160	O95971	CD160 antigen	rs12562475	121268653	1	C	T	0.897	0.128	0.013	1.1E-22	trans
337	CD160	O95971	CD160 antigen	rs9728526	145716763	1	G	A	0.560	0.394	0.008	0.0E+00	cis
337	CD160	O95971	CD160 antigen	rs370856537	145359251	1	A	C	0.920	0.211	0.015	4.7E-43	trans
337	CD160	O95971	CD160 antigen	rs2009581	111807677	2	G	A	0.728	0.162	0.008	7.7E-89	trans
337	CD160	O95971	CD160 antigen	rs186535521	197791434	2	C	T	0.983	0.285	0.030	6.0E-22	trans
337	CD160	O95971	CD160 antigen	rs2844616	31242639	6	C	T	0.414	0.109	0.007	4.1E-50	trans
337	CD160	O95971	CD160 antigen	rs8177590	6027302	10	A	G	0.568	0.060	0.007	1.6E-16	trans
337	CD160	O95971	CD160 antigen	rs2497318	94432000	10	T	C	0.452	0.094	0.007	1.6E-38	trans
337	CD160	O95971	CD160 antigen	rs3016275	128067404	11	C	T	0.663	0.086	0.008	6.6E-30	trans
337	CD160	O95971	CD160 antigen	rs58745116	33803592	17	G	A	0.610	0.075	0.007	3.8E-24	trans
337	CD160	O95971	CD160 antigen	rs601338	49206674	19	G	A	0.491	0.066	0.007	3.8E-20	trans
338	CD163	Q86VB7	Scavenger receptor cysteine-rich type 1 protein M130	rs2099684	161500130	1	G	A	0.315	0.060	0.008	6.0E-15	trans
338	CD163	Q86VB7	Scavenger receptor cysteine-rich type 1 protein M130	rs62165726	134966562	2	C	A	0.963	0.689	0.019	2.1E-281	trans
338	CD163	Q86VB7	Scavenger receptor cysteine-rich type 1 protein M130	rs190303734	203491226	2	T	A	0.297	0.062	0.008	8.4E-15	trans
338	CD163	Q86VB7	Scavenger receptor cysteine-rich type 1 protein M130	rs11923060	186589390	3	C	T	0.843	0.077	0.010	1.9E-15	trans
338	CD163	Q86VB7	Scavenger receptor cysteine-rich type 1 protein M130	rs115216147	70665526	5	A	T	0.066	0.166	0.015	4.2E-30	trans
338	CD163	Q86VB7	Scavenger receptor cysteine-rich type 1 protein M130	rs11381821	109641874	6	C	CA	0.532	0.055	0.007	2.0E-14	trans
338	CD163	Q86VB7	Scavenger receptor cysteine-rich type 1 protein M130	rs10267707	150510973	7	G	C	0.454	0.053	0.007	1.4E-13	trans
338	CD163	Q86VB7	Scavenger receptor cysteine-rich type 1 protein M130	rs2737231	116650219	8	A	G	0.695	0.058	0.008	5.2E-14	trans
338	CD163	Q86VB7	Scavenger receptor cysteine-rich type 1 protein M130	rs2031902	33117524	9	C	T	0.413	0.091	0.007	9.8E-36	trans
338	CD163	Q86VB7	Scavenger receptor cysteine-rich type 1 protein M130	rs635634	136155000	9	T	C	0.184	0.093	0.009	4.3E-24	trans
338	CD163	Q86VB7	Scavenger receptor cysteine-rich type 1 protein M130	rs11220505	126334805	11	T	C	0.223	0.130	0.009	1.0E-51	trans
338	CD163	Q86VB7	Scavenger receptor cysteine-rich type 1 protein M130	rs217184	72105965	16	T	C	0.804	0.080	0.009	2.8E-19	trans
338	CD163	Q86VB7	Scavenger receptor cysteine-rich type 1 protein M130	rs186021206	7069412	17	A	G	0.006	1.793	0.049	5.5E-291	trans
338	CD163	Q86VB7	Scavenger receptor cysteine-rich type 1 protein M130	rs601338	49206674	19	A	G	0.509	0.059	0.007	1.2E-16	trans
339	CD164	Q04900	Sialomucin core protein 24	rs4950771	202111779	1	T	G	0.066	0.166	0.015	5.0E-28	trans
339	CD164	Q04900	Sialomucin core protein 24	rs45492299	226054333	1	G	A	0.825	0.075	0.010	2.7E-14	trans
339	CD164	Q04900	Sialomucin core protein 24	rs9857570	58280690	3	A	G	0.676	0.094	0.008	5.7E-32	trans
339	CD164	Q04900	Sialomucin core protein 24	rs12213313	109757893	6	C	G	0.670	0.149	0.008	7.8E-78	cis
339	CD164	Q04900	Sialomucin core protein 24	rs4734879	106583124	8	A	G	0.726	0.058	0.008	5.9E-12	trans
339	CD164	Q04900	Sialomucin core protein 24	rs10820606	99192919	9	A	C	0.770	0.073	0.009	5.8E-16	trans
339	CD164	Q04900	Sialomucin core protein 24	rs72701845	93217023	14	G	A	0.962	0.202	0.020	1.5E-23	trans
339	CD164	Q04900	Sialomucin core protein 24	rs892090	55539072	19	G	T	0.834	0.096	0.010	2.5E-21	trans
340	CD164L2	Q6UWJ8	CD164 sialomucin-like 2 protein	rs734725	27709858	1	T	G	0.086	0.792	0.014	0.0E+00	cis
341	CD177	Q8N6Q3	CD177 antigen	rs78530667	43900697	19	T	C	0.872	0.740	0.012	0.0E+00	cis
342	CD1C	P29017	T-cell surface glycoprotein CD1c	rs61788913	60091168	1	G	T	0.872	0.100	0.011	4.6E-20	trans
342	CD1C	P29017	T-cell surface glycoprotein CD1c	rs115248892	158262619	1	G	T	0.997	1.624	0.069	2.1E-121	cis
342	CD1C	P29017	T-cell surface glycoprotein CD1c	rs4384720	145425401	2	G	C	0.920	0.105	0.013	5.7E-16	trans
342	CD1C	P29017	T-cell surface glycoprotein CD1c	rs10189685	203488449	2	G	A	0.703	0.059	0.008	4.8E-14	trans
342	CD1C	P29017	T-cell surface glycoprotein CD1c	rs4698932	106049147	4	A	G	0.494	0.053	0.007	1.6E-13	trans
342	CD1C	P29017	T-cell surface glycoprotein CD1c	rs9265969	31315706	6	A	G	0.163	0.229	0.010	2.6E-121	trans
342	CD1C	P29017	T-cell surface glycoprotein CD1c	rs11963621	44591006	6	C	T	0.104	0.115	0.012	2.7E-22	trans
342	CD1C	P29017	T-cell surface glycoprotein CD1c	rs1504215	91006227	6	G	A	0.650	0.097	0.008	1.1E-37	trans
342	CD1C	P29017	T-cell surface glycoprotein CD1c	rs2721934	116628403	8	C	T	0.441	0.051	0.007	1.9E-12	trans
342	CD1C	P29017	T-cell surface glycoprotein CD1c	rs572608	118801640	11	T	A	0.547	0.059	0.007	2.7E-16	trans
342	CD1C	P29017	T-cell surface glycoprotein CD1c	rs11218738	122524951	11	A	G	0.249	0.069	0.008	6.2E-17	trans
342	CD1C	P29017	T-cell surface glycoprotein CD1c	rs76428106	28604007	13	C	T	0.013	0.376	0.033	1.3E-30	trans
342	CD1C	P29017	T-cell surface glycoprotein CD1c	rs12599288	11822066	16	T	C	0.704	0.113	0.008	2.4E-47	trans
342	CD1C	P29017	T-cell surface glycoprotein CD1c	rs60969446	33759573	19	T	C	0.430	0.056	0.007	7.6E-15	trans
342	CD1C	P29017	T-cell surface glycoprotein CD1c	rs17272847	49979398	19	A	G	0.087	0.095	0.013	3.8E-14	trans
342	CD1C	P29017	T-cell surface glycoprotein CD1c	rs6063502	48955595	20	G	A	0.347	0.067	0.007	3.0E-19	trans
343	CD200	P41217	OX-2 membrane glycoprotein	rs1260326	27730940	2	C	T	0.609	0.058	0.007	3.2E-15	trans
343	CD200	P41217	OX-2 membrane glycoprotein	rs6775517	58358967	3	T	G	0.367	0.070	0.008	8.8E-20	trans
343	CD200	P41217	OX-2 membrane glycoprotein	rs13318932	98396350	3	T	C	0.105	0.084	0.012	3.5E-12	trans
343	CD200	P41217	OX-2 membrane glycoprotein	rs2399420	112069765	3	A	C	0.903	0.274	0.012	9.5E-109	cis
343	CD200	P41217	OX-2 membrane glycoprotein	rs8176747	136131315	9	G	C	0.061	0.286	0.015	4.2E-80	trans
343	CD200	P41217	OX-2 membrane glycoprotein	rs56278466	17875857	10	G	T	0.662	0.099	0.008	3.4E-38	trans
343	CD200	P41217	OX-2 membrane glycoprotein	rs35830321	20353027	16	C	CA	0.833	0.067	0.010	7.0E-12	trans
343	CD200	P41217	OX-2 membrane glycoprotein	rs2008174	39860130	22	C	T	0.261	0.086	0.008	1.9E-24	trans
344	CD200R1	Q8TD46	Cell surface glycoprotein CD200 receptor 1	rs72899532	62615124	2	G	T	0.806	0.058	0.007	9.1E-15	trans
344	CD200R1	Q8TD46	Cell surface glycoprotein CD200 receptor 1	rs10935473	98416900	3	G	T	0.560	0.317	0.008	0.0E+00	trans
344	CD200R1	Q8TD46	Cell surface glycoprotein CD200 receptor 1	rs16860233	112642122	3	T	C	0.039	1.825	0.020	0.0E+00	cis
344	CD200R1	Q8TD46	Cell surface glycoprotein CD200 receptor 1	rs3129782	32648719	6	G	A	0.323	0.076	0.006	2.7E-32	trans
344	CD200R1	Q8TD46	Cell surface glycoprotein CD200 receptor 1	rs544503442	105972546	8	A	G	0.143	0.072	0.009	1.0E-15	trans
344	CD200R1	Q8TD46	Cell surface glycoprotein CD200 receptor 1	rs10823374	71201716	10	G	A	0.445	0.057	0.006	5.3E-22	trans
344	CD200R1	Q8TD46	Cell surface glycoprotein CD200 receptor 1	rs4938939	60160838	11	A	G	0.289	0.047	0.006	4.8E-13	trans
344	CD200R1	Q8TD46	Cell surface glycoprotein CD200 receptor 1	rs75071241	126232186	11	G	A	0.940	0.116	0.012	1.5E-20	trans
344	CD200R1	Q8TD46	Cell surface glycoprotein CD200 receptor 1	rs186021206	7069412	17	A	G	0.006	0.916	0.041	1.2E-112	trans
345	CD207	Q9UJ71	C-type lectin domain family 4 member K	rs35533397	12700857	2	T	C	0.226	0.092	0.009	2.4E-24	trans
345	CD207	Q9UJ71	C-type lectin domain family 4 member K	rs17718987	71060936	2	C	G	0.325	0.473	0.008	0.0E+00	cis
345	CD207	Q9UJ71	C-type lectin domain family 4 member K	rs7432658	185048314	3	T	C	0.211	0.070	0.008	5.9E-17	trans
345	CD207	Q9UJ71	C-type lectin domain family 4 member K	rs3129843	32395726	6	A	G	0.871	0.181	0.010	1.5E-70	trans
345	CD207	Q9UJ71	C-type lectin domain family 4 member K	rs62408218	90931858	6	C	T	0.640	0.102	0.007	2.2E-46	trans
345	CD207	Q9UJ71	C-type lectin domain family 4 member K	rs989437	28830498	7	G	A	0.618	0.077	0.007	8.4E-28	trans
345	CD207	Q9UJ71	C-type lectin domain family 4 member K	rs2721926	116608228	8	T	G	0.442	0.082	0.007	7.6E-33	trans
345	CD207	Q9UJ71	C-type lectin domain family 4 member K	rs17450261	90815495	10	C	T	0.200	0.061	0.008	6.4E-13	trans
345	CD207	Q9UJ71	C-type lectin domain family 4 member K	rs34593439	79234957	15	G	A	0.894	0.091	0.011	1.8E-16	trans
345	CD207	Q9UJ71	C-type lectin domain family 4 member K	rs4985556	70694000	16	C	A	0.879	0.082	0.010	2.7E-15	trans
345	CD207	Q9UJ71	C-type lectin domain family 4 member K	rs2139493	86021624	16	T	C	0.733	0.053	0.008	6.0E-12	trans
345	CD207	Q9UJ71	C-type lectin domain family 4 member K	rs7247412	52313868	19	T	C	0.734	0.065	0.008	2.4E-17	trans
346	CD209	Q9NNX6	CD209 antigen	rs7528419	109817192	1	A	G	0.778	0.102	0.007	1.8E-47	trans
346	CD209	Q9NNX6	CD209 antigen	rs9677444	183613678	2	T	C	0.380	0.044	0.006	1.8E-13	trans
346	CD209	Q9NNX6	CD209 antigen	rs10769256	47378396	11	T	C	0.400	0.057	0.006	5.1E-21	trans
346	CD209	Q9NNX6	CD209 antigen	rs183130	56991363	16	T	C	0.325	0.045	0.006	7.3E-13	trans
346	CD209	Q9NNX6	CD209 antigen	rs16950595	79580990	16	G	C	0.905	0.069	0.010	1.0E-11	trans
346	CD209	Q9NNX6	CD209 antigen	rs77542162	67081278	17	G	A	0.023	0.150	0.020	2.2E-14	trans
346	CD209	Q9NNX6	CD209 antigen	rs4804774	7772173	19	G	C	0.763	0.414	0.008	0.0E+00	cis
346	CD209	Q9NNX6	CD209 antigen	rs35164803	14929375	X	G	A	0.963	0.096	0.013	4.5E-14	trans
347	CD22	P20273	B-cell receptor CD22	rs4848370	111811665	2	C	T	0.728	0.071	0.008	2.5E-19	trans
347	CD22	P20273	B-cell receptor CD22	rs967367	186734466	3	G	A	0.612	0.120	0.007	1.2E-61	trans
347	CD22	P20273	B-cell receptor CD22	rs304154	88120416	5	C	T	0.309	0.053	0.008	2.2E-12	trans
347	CD22	P20273	B-cell receptor CD22	rs111672628	158256903	5	A	G	0.924	0.123	0.013	1.6E-20	trans
347	CD22	P20273	B-cell receptor CD22	rs9271065	32575619	6	C	G	0.146	0.247	0.010	3.5E-134	trans
347	CD22	P20273	B-cell receptor CD22	rs12554596	37002142	9	A	G	0.796	0.066	0.009	3.2E-14	trans
347	CD22	P20273	B-cell receptor CD22	rs12784975	98380137	10	T	C	0.808	0.062	0.009	4.0E-12	trans
347	CD22	P20273	B-cell receptor CD22	rs76428106	28604007	13	C	T	0.013	0.229	0.032	1.3E-12	trans
347	CD22	P20273	B-cell receptor CD22	rs374039502	108960385	13	A	T	0.021	0.337	0.026	9.8E-38	trans
347	CD22	P20273	B-cell receptor CD22	rs9939427	86016091	16	A	G	0.231	0.074	0.008	1.1E-18	trans
347	CD22	P20273	B-cell receptor CD22	rs186021206	7069412	17	A	G	0.006	0.995	0.048	5.1E-94	trans
347	CD22	P20273	B-cell receptor CD22	rs34557412	16852187	17	G	A	0.007	0.713	0.043	1.3E-62	trans
347	CD22	P20273	B-cell receptor CD22	rs35222145	38007321	17	T	G	0.508	0.054	0.007	2.0E-14	trans
347	CD22	P20273	B-cell receptor CD22	rs10411704	35800662	19	G	T	0.214	0.295	0.009	3.0E-252	cis
347	CD22	P20273	B-cell receptor CD22	rs492602	49206417	19	G	A	0.509	0.062	0.007	2.8E-18	trans
347	CD22	P20273	B-cell receptor CD22	rs6065926	44735854	20	A	G	0.246	0.072	0.008	1.8E-18	trans
347	CD22	P20273	B-cell receptor CD22	rs73167342	39834102	22	C	G	0.258	0.061	0.008	4.0E-14	trans
347	CD22	P20273	B-cell receptor CD22	rs73165110	42291856	22	G	A	0.921	0.124	0.013	2.6E-21	trans
348	CD226	Q15762	CD226 antigen	rs2278668	122835232	3	T	C	0.406	0.062	0.008	1.3E-15	trans
348	CD226	Q15762	CD226 antigen	rs6993770	106581528	8	A	T	0.713	0.102	0.008	6.0E-35	trans
348	CD226	Q15762	CD226 antigen	rs72850966	1793552	11	C	T	0.913	0.197	0.013	2.2E-49	trans
348	CD226	Q15762	CD226 antigen	rs9635249	65797784	14	G	A	0.794	0.109	0.009	2.1E-31	trans
348	CD226	Q15762	CD226 antigen	rs151234	28505660	16	C	G	0.129	0.079	0.011	1.7E-12	trans
348	CD226	Q15762	CD226 antigen	rs13381239	67526725	18	T	A	0.528	0.264	0.008	5.6E-261	cis
348	CD226	Q15762	CD226 antigen	rs1654425	55538980	19	C	T	0.834	0.101	0.010	1.2E-23	trans
349	CD244	Q9BZW8	Natural killer cell receptor 2B4	rs37456	44292989	1	T	C	0.353	0.071	0.008	2.5E-20	trans
349	CD244	Q9BZW8	Natural killer cell receptor 2B4	rs115868021	160811374	1	C	G	0.994	2.164	0.049	0.0E+00	cis
349	CD244	Q9BZW8	Natural killer cell receptor 2B4	rs12616639	62529925	2	G	A	0.611	0.110	0.007	1.7E-50	trans
349	CD244	Q9BZW8	Natural killer cell receptor 2B4	rs2009581	111807677	2	G	A	0.728	0.089	0.008	7.9E-28	trans
349	CD244	Q9BZW8	Natural killer cell receptor 2B4	rs34458031	98752035	3	A	G	0.086	0.090	0.013	2.8E-12	trans
349	CD244	Q9BZW8	Natural killer cell receptor 2B4	rs6993770	106581528	8	T	A	0.287	0.077	0.008	3.0E-22	trans
349	CD244	Q9BZW8	Natural killer cell receptor 2B4	rs10815098	4865338	9	T	C	0.208	0.074	0.009	5.9E-17	trans
349	CD244	Q9BZW8	Natural killer cell receptor 2B4	rs113315674	100720053	9	A	G	0.365	0.082	0.007	6.4E-28	trans
349	CD244	Q9BZW8	Natural killer cell receptor 2B4	rs12574844	126273386	11	G	A	0.929	0.131	0.014	1.3E-20	trans
349	CD244	Q9BZW8	Natural killer cell receptor 2B4	rs10146962	101170540	14	T	C	0.662	0.055	0.008	6.9E-13	trans
349	CD244	Q9BZW8	Natural killer cell receptor 2B4	rs186021206	7069412	17	A	G	0.006	1.292	0.050	1.8E-149	trans
349	CD244	Q9BZW8	Natural killer cell receptor 2B4	rs2659005	79218714	17	T	C	0.441	0.087	0.007	4.1E-33	trans
350	CD248	Q9HCU0	Endosialin	rs333947	110470764	1	G	A	0.852	0.069	0.010	5.9E-12	trans
350	CD248	Q9HCU0	Endosialin	rs1260326	27730940	2	C	T	0.608	0.057	0.007	7.3E-15	trans
350	CD248	Q9HCU0	Endosialin	rs67409736	52551284	3	T	G	0.558	0.101	0.007	4.5E-44	trans
350	CD248	Q9HCU0	Endosialin	rs78526588	132217957	3	T	G	0.912	0.109	0.013	5.9E-18	trans
350	CD248	Q9HCU0	Endosialin	rs73080552	39435433	5	G	A	0.890	0.087	0.011	2.1E-14	trans
350	CD248	Q9HCU0	Endosialin	rs6913309	32339840	6	A	T	0.280	0.056	0.008	2.1E-12	trans
350	CD248	Q9HCU0	Endosialin	rs11766489	18003670	7	C	T	0.517	0.065	0.007	1.3E-19	trans
350	CD248	Q9HCU0	Endosialin	rs17657502	18227452	10	T	C	0.180	0.068	0.009	2.5E-13	trans

350	CD248	Q9HCU0	Endosialin	rs182547559	66082215	11	C	G	0.996	0.763	0.056	2.4E-42	cis
351	CD27	P26842	CD27 antigen	rs61747728	179526214	1	T	C	0.038	0.137	0.019	2.9E-13	trans
351	CD27	P26842	CD27 antigen	rs4722557	26019556	7	G	A	0.741	0.063	0.008	3.8E-14	trans
351	CD27	P26842	CD27 antigen	rs150861794	109003805	13	T	C	0.018	0.210	0.029	9.5E-13	trans
351	CD27	P26842	CD27 antigen	rs34562254	16842991	17	A	G	0.097	0.100	0.012	1.3E-16	trans
352	CD274	Q9NZQ7	Programmed cell death 1 ligand 1	rs4657041	161478859	1	C	T	0.536	0.061	0.007	6.9E-17	trans
352	CD274	Q9NZQ7	Programmed cell death 1 ligand 1	rs78444298	184672098	1	A	G	0.019	0.196	0.026	1.0E-13	trans
352	CD274	Q9NZQ7	Programmed cell death 1 ligand 1	rs6993770	106581528	8	A	T	0.713	0.061	0.008	2.3E-14	trans
352	CD274	Q9NZQ7	Programmed cell death 1 ligand 1	rs822340	5453260	9	G	A	0.745	0.341	0.009	0.0E+00	cis
352	CD274	Q9NZQ7	Programmed cell death 1 ligand 1	rs112635299	94838142	14	T	G	0.022	0.216	0.025	2.2E-18	trans
352	CD274	Q9NZQ7	Programmed cell death 1 ligand 1	rs8178824	64224775	17	T	C	0.030	0.151	0.021	1.1E-12	trans
353	CD276	Q5ZPR3	CD276 antigen	rs4665972	27598097	2	C	T	0.609	0.055	0.006	3.0E-18	trans
353	CD276	Q5ZPR3	CD276 antigen	rs1042704	23312594	14	A	G	0.215	0.058	0.007	7.5E-15	trans
353	CD276	Q5ZPR3	CD276 antigen	rs2239651	94848547	14	T	C	0.735	0.072	0.007	8.2E-25	trans
353	CD276	Q5ZPR3	CD276 antigen	rs2291014	73996359	15	G	A	0.902	1.337	0.012	0.0E+00	cis
353	CD276	Q5ZPR3	CD276 antigen	rs186021206	7069412	17	A	G	0.006	0.414	0.042	1.0E-22	trans
353	CD276	Q5ZPR3	CD276 antigen	rs2837988	42619544	21	A	C	0.362	0.068	0.006	8.6E-26	trans
354	CD28	P10747	T-cell-specific surface glycoprotein CD28	rs37459	44289397	1	G	A	0.353	0.110	0.008	4.8E-46	trans
354	CD28	P10747	T-cell-specific surface glycoprotein CD28	rs13406632	204649338	2	G	C	0.832	0.261	0.010	5.6E-147	cis
354	CD28	P10747	T-cell-specific surface glycoprotein CD28	rs9838308	169751292	3	T	C	0.635	0.115	0.008	1.0E-48	trans
354	CD28	P10747	T-cell-specific surface glycoprotein CD28	rs6796	6502367	7	C	T	0.279	0.059	0.008	1.1E-12	trans
354	CD28	P10747	T-cell-specific surface glycoprotein CD28	rs1461729	9187242	8	A	G	0.103	0.090	0.012	2.0E-13	trans
354	CD28	P10747	T-cell-specific surface glycoprotein CD28	rs2293579	47440758	11	G	A	0.614	0.055	0.008	5.4E-13	trans
354	CD28	P10747	T-cell-specific surface glycoprotein CD28	rs78689694	126234820	11	G	C	0.867	0.096	0.011	3.7E-18	trans
354	CD28	P10747	T-cell-specific surface glycoprotein CD28	rs7161799	58770523	15	T	C	0.076	0.131	0.014	1.5E-20	trans
354	CD28	P10747	T-cell-specific surface glycoprotein CD28	rs186021206	7069412	17	A	G	0.006	1.362	0.052	2.8E-152	trans
354	CD28	P10747	T-cell-specific surface glycoprotein CD28	rs2725405	79220224	17	C	G	0.441	0.065	0.008	5.1E-18	trans
355	CD2AP	Q9Y5K6	CD2-associated protein	rs1354034	56849749	3	C	T	0.604	0.063	0.007	6.5E-18	trans
355	CD2AP	Q9Y5K6	CD2-associated protein	rs1385742	47595155	6	A	T	0.355	0.286	0.008	3.8E-300	cis
356	CD300A	Q9UGN4	CMRF35-like molecule 8	rs11888817	62530980	2	C	T	0.643	0.107	0.007	2.5E-47	trans
356	CD300A	Q9UGN4	CMRF35-like molecule 8	rs6532770	99783669	4	A	C	0.244	0.059	0.008	1.3E-12	trans
356	CD300A	Q9UGN4	CMRF35-like molecule 8	rs13107325	103188709	4	T	C	0.075	0.214	0.014	3.8E-56	trans
356	CD300A	Q9UGN4	CMRF35-like molecule 8	rs509227	10535591	6	C	A	0.523	0.051	0.007	8.4E-13	trans
356	CD300A	Q9UGN4	CMRF35-like molecule 8	rs950802	60152584	11	A	G	0.306	0.065	0.008	2.6E-17	trans
356	CD300A	Q9UGN4	CMRF35-like molecule 8	rs186021206	7069412	17	A	G	0.006	0.870	0.049	3.6E-69	trans
356	CD300A	Q9UGN4	CMRF35-like molecule 8	rs4794824	38179290	17	A	G	0.391	0.060	0.007	6.6E-16	trans
356	CD300A	Q9UGN4	CMRF35-like molecule 8	rs62087202	72459919	17	G	A	0.951	0.623	0.017	8.2E-304	cis
357	CD300C	Q08708	CMRF35-like molecule 6	rs11888817	62530980	2	C	T	0.642	0.120	0.007	1.8E-63	trans
357	CD300C	Q08708	CMRF35-like molecule 6	rs79935131	98498567	3	T	G	0.086	0.144	0.012	1.1E-31	trans
357	CD300C	Q08708	CMRF35-like molecule 6	rs13107325	103188709	4	T	C	0.075	0.143	0.013	7.7E-28	trans
357	CD300C	Q08708	CMRF35-like molecule 6	rs509227	10535591	6	C	A	0.523	0.065	0.007	2.1E-21	trans
357	CD300C	Q08708	CMRF35-like molecule 6	rs17843620	32620800	6	G	A	0.424	0.050	0.007	7.8E-12	trans
357	CD300C	Q08708	CMRF35-like molecule 6	rs4413619	96082979	6	T	C	0.107	0.078	0.011	2.0E-12	trans
357	CD300C	Q08708	CMRF35-like molecule 6	rs143110592	7236131	7	A	T	0.805	0.062	0.009	8.9E-13	trans
357	CD300C	Q08708	CMRF35-like molecule 6	rs635634	136155000	9	T	C	0.184	0.060	0.009	1.7E-11	trans
357	CD300C	Q08708	CMRF35-like molecule 6	rs181897168	126227095	11	A	G	0.010	0.257	0.037	1.9E-12	trans
357	CD300C	Q08708	CMRF35-like molecule 6	rs186021206	7069412	17	A	G	0.006	1.070	0.050	1.8E-102	trans
357	CD300C	Q08708	CMRF35-like molecule 6	rs58440339	72539474	17	T	C	0.092	0.682	0.013	0.0E+00	cis
357	CD300C	Q08708	CMRF35-like molecule 6	rs2659005	79218714	17	T	C	0.441	0.058	0.007	1.6E-15	trans
358	CD300E	Q496F6	CMRF35-like molecule 2	rs111344982	32473588	6	A	T	0.734	0.075	0.009	1.6E-17	trans
358	CD300E	Q496F6	CMRF35-like molecule 2	rs3778752	128580047	7	T	G	0.460	0.080	0.007	2.1E-30	trans
358	CD300E	Q496F6	CMRF35-like molecule 2	rs76428106	28604007	13	C	T	0.013	0.299	0.032	3.3E-21	trans
358	CD300E	Q496F6	CMRF35-like molecule 2	rs1987102	80265888	15	T	C	0.740	0.060	0.008	1.2E-14	trans
358	CD300E	Q496F6	CMRF35-like molecule 2	rs1719465	72576391	17	T	C	0.313	0.361	0.008	0.0E+00	cis
358	CD300E	Q496F6	CMRF35-like molecule 2	rs4760	44153100	19	G	A	0.156	0.133	0.009	7.7E-45	trans
359	CD300LF	Q8TDQ1	CMRF35-like molecule 1	rs12667978	128777461	7	C	T	0.428	0.039	0.005	4.3E-14	trans
359	CD300LF	Q8TDQ1	CMRF35-like molecule 1	rs3809111	307029	11	C	T	0.936	0.084	0.011	1.4E-15	trans
359	CD300LF	Q8TDQ1	CMRF35-like molecule 1	rs4997081	20365234	16	G	C	0.808	0.051	0.007	1.7E-14	trans
359	CD300LF	Q8TDQ1	CMRF35-like molecule 1	rs2241244	38153554	17	G	C	0.463	0.060	0.007	1.6E-15	trans
359	CD300LF	Q8TDQ1	CMRF35-like molecule 1	rs35489971	72700943	17	G	A	0.812	1.295	0.010	0.0E+00	cis
360	CD300LG	Q6UXG3	CMRF35-like molecule 9	rs61747728	179526214	1	T	C	0.038	0.145	0.018	6.4E-16	trans
360	CD300LG	Q6UXG3	CMRF35-like molecule 9	rs78058190	219699999	2	G	A	0.949	0.134	0.018	2.4E-14	trans
360	CD300LG	Q6UXG3	CMRF35-like molecule 9	rs7647481	12391813	3	A	G	0.122	0.078	0.011	1.1E-13	trans
360	CD300LG	Q6UXG3	CMRF35-like molecule 9	rs13107325	103188709	4	T	C	0.075	0.094	0.013	8.5E-13	trans
360	CD300LG	Q6UXG3	CMRF35-like molecule 9	rs11967262	43760327	6	C	G	0.514	0.087	0.007	7.4E-37	trans
360	CD300LG	Q6UXG3	CMRF35-like molecule 9	rs28588745	10647044	8	A	T	0.793	0.107	0.009	1.9E-36	trans
360	CD300LG	Q6UXG3	CMRF35-like molecule 9	rs10901252	136128000	9	C	G	0.061	0.231	0.014	2.3E-58	trans
360	CD300LG	Q6UXG3	CMRF35-like molecule 9	rs2068888	94839642	10	A	G	0.450	0.051	0.007	1.9E-13	trans
360	CD300LG	Q6UXG3	CMRF35-like molecule 9	rs71468663	64018104	11	A	AC	0.953	0.135	0.016	1.6E-16	trans
360	CD300LG	Q6UXG3	CMRF35-like molecule 9	rs174418	58687603	15	T	C	0.402	0.058	0.007	2.6E-16	trans
360	CD300LG	Q6UXG3	CMRF35-like molecule 9	rs12446515	56987015	16	T	C	0.324	0.062	0.007	5.0E-17	trans
360	CD300LG	Q6UXG3	CMRF35-like molecule 9	rs72836561	41926126	17	C	T	0.968	1.072	0.020	0.0E+00	cis
361	CD302	Q8IX05	CD302 antigen	rs2556106	160654524	2	G	T	0.127	0.419	0.011	0.0E+00	cis
361	CD302	Q8IX05	CD302 antigen	rs10073754	179306517	5	G	A	0.125	0.099	0.011	1.1E-20	trans
361	CD302	Q8IX05	CD302 antigen	rs9260603	29922754	6	C	T	0.559	0.096	0.007	2.2E-42	trans
361	CD302	Q8IX05	CD302 antigen	rs9302635	72144174	16	T	C	0.818	0.065	0.009	6.5E-13	trans
361	CD302	Q8IX05	CD302 antigen	rs8178824	64224775	17	T	C	0.030	0.300	0.021	9.0E-48	trans
362	CD33	P20138	Myeloid cell surface antigen CD33	rs62165726	134966562	2	C	A	0.963	0.101	0.010	2.4E-24	trans
362	CD33	P20138	Myeloid cell surface antigen CD33	rs7865362	33117965	9	T	C	0.363	0.029	0.004	1.1E-13	trans
362	CD33	P20138	Myeloid cell surface antigen CD33	rs10998781	71203641	10	G	A	0.444	0.049	0.004	9.5E-39	trans
362	CD33	P20138	Myeloid cell surface antigen CD33	rs4055121	126232337	11	C	T	0.866	0.037	0.006	1.5E-11	trans
362	CD33	P20138	Myeloid cell surface antigen CD33	rs186021206	7069412	17	A	G	0.006	0.633	0.026	1.5E-133	trans
362	CD33	P20138	Myeloid cell surface antigen CD33	rs2455069	51728641	19	G	A	0.427	1.177	0.008	0.0E+00	cis
363	CD34	P28906	Hematopoietic progenitor cell antigen CD34	rs6671850	208057800	1	T	C	0.064	0.123	0.015	4.1E-16	cis
363	CD34	P28906	Hematopoietic progenitor cell antigen CD34	rs532436	136149830	9	G	A	0.815	0.461	0.010	0.0E+00	trans
363	CD34	P28906	Hematopoietic progenitor cell antigen CD34	rs34434834	126307796	11	A	G	0.033	0.219	0.020	8.2E-27	trans
363	CD34	P28906	Hematopoietic progenitor cell antigen CD34	rs186021206	7069412	17	A	G	0.006	0.721	0.050	1.1E-46	trans
364	CD36	P16671	Platelet glycoprotein 4	rs3811444	248039451	1	C	T	0.668	0.055	0.008	1.2E-12	trans
364	CD36	P16671	Platelet glycoprotein 4	rs7618405	18250509	3	C	A	0.795	0.177	0.009	3.1E-83	trans
364	CD36	P16671	Platelet glycoprotein 4	rs62248908	47297060	3	A	G	0.826	0.071	0.010	4.0E-13	trans
364	CD36	P16671	Platelet glycoprotein 4	rs1354034	56849749	3	T	C	0.396	0.062	0.008	2.4E-16	trans
364	CD36	P16671	Platelet glycoprotein 4	rs139596721	154461313	4	CT	C	0.033	0.144	0.021	5.1E-12	trans
364	CD36	P16671	Platelet glycoprotein 4	rs4705938	131694077	5	T	C	0.522	0.054	0.007	1.9E-13	trans
364	CD36	P16671	Platelet glycoprotein 4	rs511515	33541507	6	G	A	0.699	0.056	0.008	2.1E-12	trans
364	CD36	P16671	Platelet glycoprotein 4	rs6927569	109621494	6	C	T	0.522	0.057	0.007	8.0E-15	trans
364	CD36	P16671	Platelet glycoprotein 4	rs10224886	38242166	7	T	A	0.749	0.105	0.009	4.4E-34	trans
364	CD36	P16671	Platelet glycoprotein 4	rs6961069	80218961	7	T	C	0.403	0.273	0.008	3.6E-279	cis
364	CD36	P16671	Platelet glycoprotein 4	rs139141690	101499930	7	A	G	0.005	0.494	0.055	1.9E-19	trans
364	CD36	P16671	Platelet glycoprotein 4	rs1293303	11720227	8	C	G	0.446	0.063	0.007	3.0E-17	trans
364	CD36	P16671	Platelet glycoprotein 4	rs6993770	106581528	8	A	T	0.713	0.209	0.008	2.5E-145	trans
364	CD36	P16671	Platelet glycoprotein 4	rs10820606	99192919	9	A	C	0.771	0.065	0.009	1.8E-13	trans
364	CD36	P16671	Platelet glycoprotein 4	rs7080386	65048306	10	A	C	0.412	0.091	0.007	5.6E-34	trans
364	CD36	P16671	Platelet glycoprotein 4	rs116052829	81164146	10	T	C	0.103	0.086	0.012	1.0E-12	trans
364	CD36	P16671	Platelet glycoprotein 4	rs34377578	104336426	10	C	A	0.253	0.064	0.008	6.3E-14	trans
364	CD36	P16671	Platelet glycoprotein 4	rs113473196	1794144	11	A	G	0.913	0.195	0.013	8.1E-51	trans
364	CD36	P16671	Platelet glycoprotein 4	rs73000929	113953622	11	G	A	0.963	0.142	0.019	2.5E-13	trans
364	CD36	P16671	Platelet glycoprotein 4	rs2588833	68599492	14	C	A	0.836	0.069	0.010	3.6E-12	trans
364	CD36	P16671	Platelet glycoprotein 4	rs12719735	101513378	15	G	T	0.289	0.068	0.008	5.2E-17	trans
364	CD36	P16671	Platelet glycoprotein 4	rs1654425	55538980	19	C	T	0.834	0.095	0.010	7.4E-22	trans
364	CD36	P16671	Platelet glycoprotein 4	rs111527738	36259324	21	G	A	0.021	0.217	0.026	3.6E-17	trans
365	CD38	P28907	ADP-ribosyl cyclase/cyclic ADP-ribose hydrolase 1	rs6449182	15780453	4	C	G	0.776	0.423	0.007	0.0E+00	cis
366	CD4	P01730	T-cell surface glycoprotein CD4	rs147599819	32594816	6	G	GGAT	0.602	0.122	0.008	3.3E-53	trans
366	CD4	P01730	T-cell surface glycoprotein CD4	rs964184	116648917	11	G	C	0.133	0.095	0.011	4.2E-19	trans
366	CD4	P01730	T-cell surface glycoprotein CD4	rs72654473	45414399	19	A	C	0.110	0.096	0.012	1.1E-16	trans
367	CD40	P25942	Tumor necrosis factor receptor superfamily member 5	rs1354034	56849749	3	C	T	0.604	0.090	0.007	2.7E-34	trans
367	CD40	P25942	Tumor necrosis factor receptor superfamily member 5	rs342293	106372219	7	C	G	0.541	0.067	0.007	4.9E-20	trans
367	CD40	P25942	Tumor necrosis factor receptor superfamily member 5	rs6993770	106581528	8	A	T	0.713	0.076	0.008	3.2E-21	trans
367	CD40	P25942	Tumor necrosis factor receptor superfamily member 5	rs7896518	65104500	10	G	A	0.424	0.074	0.007	1.5E-23	trans
367	CD40	P25942	Tumor necrosis factor receptor superfamily member 5	rs11603942	266804	11	T	C	0.247	0.059	0.008	3.0E-12	trans
367	CD40	P25942	Tumor necrosis factor receptor superfamily member 5	rs892090	55539072	19	G	T	0.834	0.074	0.010	3.9E-14	trans
367	CD40	P25942	Tumor necrosis factor receptor superfamily member 5	rs4810485	44747947	20	G	T	0.752	0.424	0.009	0.0E+00	cis
368	CD40LG	P29965	CD40 ligand	rs13084580	39188182	3	T	C	0.114	0.087	0.012	1.4E-13	trans
368	CD40LG	P29965	CD40 ligand	rs1354034	56849749	3	T	C	0.396	0.069	0.008	2.2E-19	trans
368	CD40LG	P29965	CD40 ligand	rs139596721	154461313	4	CT	C	0.032	0.147	0.021	6.8E-12	trans
368	CD40LG	P29965	CD40 ligand	rs2248116	131804347	5	A	C	0.571	0.057	0.008	5.9E-14	trans
368	CD40LG	P29965	CD40 ligand	rs6961069	80218961	7	T	C	0.402	0.052	0.008	9.9E-12	trans
368	CD40LG	P29965	CD40 ligand	rs6993770	106581528	8	A	T	0.713	0.131	0.008	2.3E-56	trans
368	CD40LG	P29965	CD40 ligand	rs151234	28505660	16	C	G	0.129	0.076	0.011	9.4E-12	trans
368	CD40LG	P29965	CD40 ligand	rs12445050	81870969	16	T	C	0.138	0.091	0.011	3.9E-17	trans
368	CD40LG	P29965	CD40 ligand	rs1654425	55538980	19	C	T	0.834	0.136	0.010	5.4E-41	trans
368	CD40LG	P29965	CD40 ligand	rs6081565	19287904	20	A	G	0.353	0.058	0.008	1.3E-13	trans
368	CD40LG	P29965	CD40 ligand	rs4239702	44749251	20	T	C	0.278	0.116	0.008	2.1E-43	trans
368	CD40LG	P29965	CD40 ligand	rs3002416	39710195	X	C	T	0.592	0.045	0.007	8.3E-12	trans
368	CD40LG	P29965	CD40 ligand	rs36206512	135730217	X	A	C	0.965	0.552	0.017	2.0E-223	cis
369	CD46	P15529	Membrane cofactor protein	rs61747728	179526214	1	T	C	0.038	0.160	0.019	1.9E-16	trans
369	CD46	P15529	Membrane cofactor protein	rs1970530	207924066	1	G	A	0.405	0.089	0.008	3.5E-31	cis
369	CD46	P15529	Membrane cofactor protein	rs6993770	106581528	8	A	T	0.714	0.066	0.008	1.0E-15	trans
369	CD46	P15529	Membrane cofactor protein	rs579459	136154168	9	T	C	0.793	0.063	0.009	7.3E-12	trans
369	CD46	P15529	Membrane cofactor protein	rs10740131	65271488	10	T	A	0.470	0.060	0.008	1.1E-15	trans
369	CD46	P15529	Membrane cofactor protein	rs972570	71272018	10	T	C	0.487	0.079	0.008	9.7E-26	trans
369	CD46	P15529	Membrane cofactor protein	rs1654425	55538980	19	C	T	0.835	0.073	0.010	8.2E-13	trans
370	CD48	P09326	CD48 antigen	rs2295615	160654758	1	C	G	0.863	0.933	0.011	0.0E+00	cis
370	CD48	P09326	CD48 antigen	rs6705658	62539316	2	T	C	0.642	0.060	0.007	1.3E-18	trans

370	CD48	P09326	CD48 antigen	rs9822939	169748550	3	T	C	0.634	0.075	0.007	1.8E-27	trans
370	CD48	P09326	CD48 antigen	rs4012172	186741511	3	C	T	0.632	0.055	0.007	6.2E-16	trans
370	CD48	P09326	CD48 antigen	rs35467801	35881130	5	G	GT	0.728	0.050	0.007	1.3E-11	trans
370	CD48	P09326	CD48 antigen	rs2844622	31240060	6	G	A	0.659	0.072	0.007	3.6E-25	trans
370	CD48	P09326	CD48 antigen	rs60843925	126238832	11	T	C	0.864	0.084	0.010	4.3E-18	trans
370	CD48	P09326	CD48 antigen	rs76428106	28604007	13	C	T	0.013	0.258	0.030	1.4E-17	trans
370	CD48	P09326	CD48 antigen	rs186021206	7069412	17	A	G	0.006	1.178	0.045	3.6E-149	trans
370	CD48	P09326	CD48 antigen	rs2659005	79218714	17	T	C	0.441	0.098	0.007	2.1E-49	trans
370	CD48	P09326	CD48 antigen	rs78530667	43900697	19	T	C	0.871	0.072	0.010	1.0E-12	trans
371	CD5	P06127	T-cell surface glycoprotein CD5	rs10214237	35883734	5	T	C	0.727	0.077	0.008	7.8E-21	trans
371	CD5	P06127	T-cell surface glycoprotein CD5	rs952579	21884440	6	A	G	0.145	0.071	0.010	5.4E-12	trans
371	CD5	P06127	T-cell surface glycoprotein CD5	rs2844541	31347542	6	T	A	0.227	0.074	0.009	4.2E-17	trans
371	CD5	P06127	T-cell surface glycoprotein CD5	rs4939490	60793651	11	C	G	0.609	0.136	0.008	2.8E-73	cis
371	CD5	P06127	T-cell surface glycoprotein CD5	rs7161799	58770523	15	T	C	0.077	0.138	0.014	1.8E-23	trans
371	CD5	P06127	T-cell surface glycoprotein CD5	rs7208422	76130575	17	A	T	0.523	0.050	0.007	4.7E-12	trans
371	CD5	P06127	T-cell surface glycoprotein CD5	rs4893	48833608	19	A	G	0.960	0.130	0.019	3.9E-12	trans
372	CD55	P08174	Complement decay-accelerating factor	rs1583090	207435783	1	T	C	0.777	0.487	0.009	0.0E+00	cis
372	CD55	P08174	Complement decay-accelerating factor	rs13107325	103188709	4	T	C	0.075	0.135	0.014	1.3E-22	trans
372	CD55	P08174	Complement decay-accelerating factor	rs767298	7274584	7	T	G	0.634	0.061	0.008	8.6E-16	trans
372	CD55	P08174	Complement decay-accelerating factor	rs2142306	134470631	8	C	T	0.412	0.074	0.007	7.1E-24	trans
372	CD55	P08174	Complement decay-accelerating factor	rs186021206	7069412	17	A	G	0.006	1.070	0.050	4.0E-101	trans
372	CD55	P08174	Complement decay-accelerating factor	rs3814995	36342212	19	T	C	0.315	0.057	0.008	1.4E-13	trans
373	CD58	P19256	Lymphocyte function-associated antigen 3	rs10801908	117090493	1	C	T	0.880	0.258	0.011	2.8E-115	cis
373	CD58	P19256	Lymphocyte function-associated antigen 3	rs3213563	172412995	1	T	C	0.441	0.178	0.007	2.6E-128	trans
373	CD58	P19256	Lymphocyte function-associated antigen 3	rs1260326	27730940	2	C	T	0.609	0.056	0.007	1.0E-13	trans
373	CD58	P19256	Lymphocyte function-associated antigen 3	rs10935473	98416900	3	G	T	0.560	0.062	0.007	2.8E-17	trans
373	CD58	P19256	Lymphocyte function-associated antigen 3	rs13064814	186654766	3	A	G	0.286	0.064	0.008	1.4E-15	trans
373	CD58	P19256	Lymphocyte function-associated antigen 3	rs3967200	126232385	11	C	T	0.865	0.103	0.011	4.6E-22	trans
373	CD58	P19256	Lymphocyte function-associated antigen 3	rs186021206	7069412	17	A	G	0.006	1.295	0.050	5.6E-147	trans
373	CD58	P19256	Lymphocyte function-associated antigen 3	rs2659005	79218714	17	T	C	0.441	0.054	0.007	2.6E-13	trans
373	CD58	P19256	Lymphocyte function-associated antigen 3	rs4760	44153100	19	G	A	0.156	0.085	0.010	2.6E-17	trans
374	CD59	P13987	CD59 glycoprotein	rs34331968	196659753	1	T	C	0.534	0.070	0.007	1.8E-23	trans
374	CD59	P13987	CD59 glycoprotein	rs1398018	77372923	4	C	T	0.443	0.050	0.007	1.2E-12	trans
374	CD59	P13987	CD59 glycoprotein	rs704701	33743679	11	T	G	0.679	0.246	0.008	6.5E-229	cis
374	CD59	P13987	CD59 glycoprotein	rs77924615	20392332	16	G	A	0.804	0.071	0.009	1.1E-15	trans
374	CD59	P13987	CD59 glycoprotein	rs407307	37827163	17	G	A	0.679	0.053	0.008	2.2E-12	trans
375	CD5L	O43866	CD5 antigen-like	rs188468174	25291697	1	T	C	0.014	0.441	0.032	1.6E-43	trans
375	CD5L	O43866	CD5 antigen-like	rs1925678	39325063	1	C	T	0.666	0.058	0.008	9.3E-14	trans
375	CD5L	O43866	CD5 antigen-like	rs2476601	114377568	1	G	A	0.899	0.083	0.012	1.5E-11	trans
375	CD5L	O43866	CD5 antigen-like	rs2765501	157804648	1	A	G	0.401	0.200	0.008	2.9E-152	cis
375	CD5L	O43866	CD5 antigen-like	rs3766370	160711477	1	C	T	0.671	0.070	0.008	9.2E-19	trans
375	CD5L	O43866	CD5 antigen-like	rs17031793	106413031	2	C	T	0.839	0.069	0.010	3.8E-12	trans
375	CD5L	O43866	CD5 antigen-like	rs1898846	111814093	2	T	C	0.142	0.075	0.010	6.7E-13	trans
375	CD5L	O43866	CD5 antigen-like	rs617119	32455767	3	T	C	0.513	0.066	0.007	1.3E-19	trans
375	CD5L	O43866	CD5 antigen-like	rs9307257	102396292	4	G	A	0.498	0.061	0.007	1.1E-16	trans
375	CD5L	O43866	CD5 antigen-like	rs6891054	124340703	5	A	G	0.760	0.061	0.009	1.6E-12	trans
375	CD5L	O43866	CD5 antigen-like	rs116446171	484453	6	G	C	0.021	0.187	0.026	5.4E-13	trans
375	CD5L	O43866	CD5 antigen-like	rs1264457	30458064	6	G	A	0.394	0.107	0.008	2.3E-45	trans
375	CD5L	O43866	CD5 antigen-like	rs114373434	50474931	7	C	G	0.727	0.062	0.008	9.0E-14	trans
375	CD5L	O43866	CD5 antigen-like	rs112348182	150954562	7	A	G	0.114	0.085	0.012	1.6E-13	trans
375	CD5L	O43866	CD5 antigen-like	rs4840568	11351019	8	A	G	0.268	0.057	0.008	6.0E-12	trans
375	CD5L	O43866	CD5 antigen-like	rs2436887	103577741	8	C	T	0.736	0.087	0.008	8.5E-26	trans
375	CD5L	O43866	CD5 antigen-like	rs7903926	3928528	10	G	C	0.506	0.050	0.007	1.5E-11	trans
375	CD5L	O43866	CD5 antigen-like	rs9658720	90760669	10	T	TG	0.446	0.069	0.007	2.5E-20	trans
375	CD5L	O43866	CD5 antigen-like	rs35923643	123355391	11	G	A	0.197	0.089	0.009	8.7E-22	trans
375	CD5L	O43866	CD5 antigen-like	rs117972357	96043546	14	A	G	0.003	0.534	0.073	3.5E-13	trans
375	CD5L	O43866	CD5 antigen-like	rs12880641	103251452	14	T	G	0.339	0.056	0.008	6.0E-13	trans
375	CD5L	O43866	CD5 antigen-like	rs200123836	106200601	14	C	T	0.728	0.056	0.008	1.5E-11	trans
375	CD5L	O43866	CD5 antigen-like	rs6497185	94802545	15	A	C	0.726	0.063	0.008	1.5E-14	trans
375	CD5L	O43866	CD5 antigen-like	rs12924236	10974423	16	C	G	0.241	0.059	0.009	3.4E-12	trans
375	CD5L	O43866	CD5 antigen-like	rs9901675	7484812	17	A	G	0.050	0.147	0.017	1.2E-18	trans
375	CD5L	O43866	CD5 antigen-like	rs34557412	16852187	17	A	G	0.993	0.301	0.044	1.2E-11	trans
375	CD5L	O43866	CD5 antigen-like	rs6037651	3686436	20	T	C	0.584	0.094	0.007	1.7E-36	trans
376	CD6	P30203	T-cell differentiation antigen CD6	rs7578575	25488819	2	A	T	0.290	0.048	0.007	1.0E-12	trans
376	CD6	P30203	T-cell differentiation antigen CD6	rs4672497	62523565	2	G	C	0.222	0.054	0.007	2.5E-13	trans
376	CD6	P30203	T-cell differentiation antigen CD6	rs62246406	47097985	3	G	A	0.824	0.054	0.008	1.6E-11	trans
376	CD6	P30203	T-cell differentiation antigen CD6	rs62454712	6498057	7	C	T	0.243	0.049	0.007	7.4E-12	trans
376	CD6	P30203	T-cell differentiation antigen CD6	rs1483573	79623617	8	A	G	0.251	0.048	0.007	6.8E-12	trans
376	CD6	P30203	T-cell differentiation antigen CD6	rs11230563	60776209	11	C	T	0.652	0.810	0.008	0.0E+00	cis
376	CD6	P30203	T-cell differentiation antigen CD6	rs7161799	58770523	15	T	C	0.077	0.100	0.011	1.6E-18	trans
376	CD6	P30203	T-cell differentiation antigen CD6	rs200489612	7106378	17	A	G	0.005	0.522	0.046	2.0E-30	trans
376	CD6	P30203	T-cell differentiation antigen CD6	rs7208422	76130575	17	A	T	0.522	0.076	0.006	1.5E-35	trans
376	CD6	P30203	T-cell differentiation antigen CD6	rs28413174	17516620	19	T	C	0.123	0.110	0.009	1.7E-32	trans
377	CD63	P08962	CD63 antigen	rs2278668	122835232	3	T	C	0.406	0.060	0.008	5.1E-15	trans
377	CD63	P08962	CD63 antigen	rs78217698	148706064	3	G	C	0.967	0.143	0.021	1.1E-11	trans
377	CD63	P08962	CD63 antigen	rs342299	106373718	7	C	T	0.545	0.052	0.008	3.8E-12	trans
377	CD63	P08962	CD63 antigen	rs6993770	106581528	8	A	T	0.714	0.174	0.008	7.0E-97	trans
377	CD63	P08962	CD63 antigen	rs10761756	65172328	10	T	C	0.470	0.054	0.008	4.7E-13	trans
377	CD63	P08962	CD63 antigen	rs12445050	81870969	16	T	C	0.138	0.082	0.011	3.8E-14	trans
377	CD63	P08962	CD63 antigen	rs35496032	38817628	19	A	G	0.014	0.363	0.033	7.0E-29	trans
377	CD63	P08962	CD63 antigen	rs892090	55539072	19	G	T	0.834	0.144	0.010	1.0E-45	trans
377	CD63	P08962	CD63 antigen	rs4813637	3682762	20	G	A	0.415	0.111	0.008	7.9E-48	trans
377	CD63	P08962	CD63 antigen	rs78107116	99954540	X	G	A	0.333	0.064	0.007	3.4E-22	trans
378	CD69	Q07108	Early activation antigen CD69	rs1354034	56849749	3	C	T	0.604	0.088	0.008	4.6E-30	trans
378	CD69	Q07108	Early activation antigen CD69	rs3804749	122833003	3	C	T	0.407	0.054	0.008	5.8E-12	trans
378	CD69	Q07108	Early activation antigen CD69	rs7758845	135428537	6	C	A	0.272	0.058	0.008	8.1E-12	trans
378	CD69	Q07108	Early activation antigen CD69	rs6993770	106581528	8	A	T	0.713	0.124	0.008	1.5E-49	trans
378	CD69	Q07108	Early activation antigen CD69	rs7896518	65104500	10	G	A	0.423	0.093	0.008	2.8E-33	trans
379	CD7	P09564	T-cell antigen CD7	rs2548993	131808869	5	A	G	0.722	0.061	0.008	3.0E-14	trans
379	CD7	P09564	T-cell antigen CD7	rs2516501	31356598	6	C	T	0.737	0.057	0.008	2.6E-12	trans
379	CD7	P09564	T-cell antigen CD7	rs3176831	80272491	17	C	T	0.957	1.132	0.019	0.0E+00	cis
380	CD70	P32970	CD70 antigen	rs17118876	59616910	1	C	G	0.430	0.039	0.006	3.2E-12	trans
380	CD70	P32970	CD70 antigen	rs12740374	109817590	1	G	T	0.778	0.303	0.007	0.0E+00	trans
380	CD70	P32970	CD70 antigen	rs57894427	29906044	6	C	T	0.093	0.068	0.009	2.2E-13	trans
380	CD70	P32970	CD70 antigen	rs7254538	6611336	19	C	G	0.543	0.750	0.007	0.0E+00	cis
381	CD72	P21854	B-cell differentiation antigen CD72	rs4848370	111811665	2	C	T	0.729	0.060	0.008	7.5E-13	trans
381	CD72	P21854	B-cell differentiation antigen CD72	rs56364901	158280972	5	C	A	0.923	0.107	0.014	2.0E-14	trans
381	CD72	P21854	B-cell differentiation antigen CD72	rs9269573	32544697	6	T	A	0.146	0.151	0.011	3.8E-46	trans
381	CD72	P21854	B-cell differentiation antigen CD72	rs869487	35615896	9	C	G	0.780	0.166	0.009	6.3E-75	cis
381	CD72	P21854	B-cell differentiation antigen CD72	rs76428106	28604007	13	C	T	0.013	0.231	0.034	1.3E-11	trans
381	CD72	P21854	B-cell differentiation antigen CD72	rs374039502	108960385	13	A	T	0.021	0.211	0.028	3.3E-14	trans
381	CD72	P21854	B-cell differentiation antigen CD72	rs34557412	16852187	17	G	A	0.007	0.420	0.045	8.7E-21	trans
381	CD72	P21854	B-cell differentiation antigen CD72	rs35222145	38007321	17	T	G	0.507	0.060	0.008	1.8E-15	trans
382	CD74	P04233	HLA class II histocompatibility antigen gamma chain	rs79078220	149793650	5	G	A	0.890	0.087	0.011	7.0E-15	cis
382	CD74	P04233	HLA class II histocompatibility antigen gamma chain	rs9273368	32626475	6	A	G	0.304	0.177	0.008	2.1E-115	trans
382	CD74	P04233	HLA class II histocompatibility antigen gamma chain	rs7194862	10965115	16	C	T	0.290	0.138	0.008	1.0E-70	trans
382	CD74	P04233	HLA class II histocompatibility antigen gamma chain	rs186021206	7069412	17	A	G	0.006	0.386	0.048	1.1E-15	trans
382	CD74	P04233	HLA class II histocompatibility antigen gamma chain	rs4969145	76406170	17	T	C	0.417	0.049	0.007	1.4E-11	trans
382	CD74	P04233	HLA class II histocompatibility antigen gamma chain	rs3827211	42619254	21	A	G	0.548	0.050	0.007	2.8E-12	trans
383	CD79B	P40259	B-cell antigen receptor complex-associated protein beta chain	rs61747728	179526214	1	T	C	0.038	0.131	0.019	4.6E-12	trans
383	CD79B	P40259	B-cell antigen receptor complex-associated protein beta chain	rs78444298	184672098	1	A	G	0.019	0.308	0.027	2.6E-31	trans
383	CD79B	P40259	B-cell antigen receptor complex-associated protein beta chain	rs112151347	227695446	2	G	A	0.973	0.209	0.023	3.3E-19	trans
383	CD79B	P40259	B-cell antigen receptor complex-associated protein beta chain	rs77397204	186738899	3	A	C	0.615	0.066	0.008	1.6E-18	trans
383	CD79B	P40259	B-cell antigen receptor complex-associated protein beta chain	rs78777785	158317592	5	A	T	0.924	0.118	0.014	6.5E-18	trans
383	CD79B	P40259	B-cell antigen receptor complex-associated protein beta chain	rs55654737	179218842	5	G	A	0.657	0.085	0.008	1.7E-28	trans
383	CD79B	P40259	B-cell antigen receptor complex-associated protein beta chain	rs2523583	31327895	6	G	A	0.295	0.054	0.008	8.7E-12	trans
383	CD79B	P40259	B-cell antigen receptor complex-associated protein beta chain	rs2395904	130617819	8	T	G	0.726	0.057	0.008	1.8E-12	trans
383	CD79B	P40259	B-cell antigen receptor complex-associated protein beta chain	rs507666	136149399	9	G	A	0.815	0.246	0.009	5.5E-148	trans
383	CD79B	P40259	B-cell antigen receptor complex-associated protein beta chain	rs7087507	63745689	10	G	A	0.349	0.059	0.008	4.8E-15	trans
383	CD79B	P40259	B-cell antigen receptor complex-associated protein beta chain	rs76428106	28604007	13	C	T	0.013	0.261	0.033	4.9E-15	trans
383	CD79B	P40259	B-cell antigen receptor complex-associated protein beta chain	rs13333054	86011033	16	T	C	0.228	0.067	0.009	1.1E-14	trans
383	CD79B	P40259	B-cell antigen receptor complex-associated protein beta chain	rs186021206	7069412	17	A	G	0.006	0.797	0.050	1.4E-56	trans
383	CD79B	P40259	B-cell antigen receptor complex-associated protein beta chain	rs12946510	37912377	17	C	T	0.524	0.059	0.007	7.5E-16	trans
383	CD79B	P40259	B-cell antigen receptor complex-associated protein beta chain	rs12946669	62006007	17	C	T	0.606	0.163	0.007	3.3E-107	cis
383	CD79B	P40259	B-cell antigen receptor complex-associated protein beta chain	rs7213540	79255912	17	C	T	0.461	0.055	0.007	4.5E-14	trans
384	CD80	P33681	T-lymphocyte activation antigen CD80	rs56078908	119286521	3	T	A	0.106	0.605	0.012	0.0E+00	cis
384	CD80	P33681	T-lymphocyte activation antigen CD80	rs3936289	186754957	3	C	T	0.238	0.068	0.008	1.1E-15	trans
384	CD80	P33681	T-lymphocyte activation antigen CD80	rs9270747	32568292	6	A	G	0.342	0.086	0.007	1.1E-31	trans
384	CD80	P33681	T-lymphocyte activation antigen CD80	rs75071241	126232186	11	G	A	0.940	0.120	0.015	1.2E-16	trans
384	CD80	P33681	T-lymphocyte activation antigen CD80	rs186021206	7069412	17	A	G	0.006	1.069	0.047	1.6E-112	trans
384	CD80	P33681	T-lymphocyte activation antigen CD80	rs738409	44324727	22	G	C	0.217	0.073	0.008	9.1E-19	trans
385	CD82	P27701	CD82 antigen	rs34118068	106434607	14	A	G	0.105	0.110	0.013	8.6E-18	trans
386	CD83	Q01151	CD83 antigen	rs35332487	62510388	2	C	A	0.695	0.057	0.008	8.4E-13	trans
386	CD83	Q01151	CD83 antigen	rs12526086	14105897	6	A	G	0.223	0.200	0.009	1.6E-113	cis
386	CD83	Q01151	CD83 antigen	rs17843620	32620800	6	G	A	0.423	0.062	0.008	3.0E-15	trans
386	CD83	Q01151	CD83 antigen	rs7801921	6390817	7	G	C	0.458	0.050	0.007	1.0E-11	trans
386	CD83	Q01151	CD83 antigen	rs7869	73576307	10	T	C	0.291	0.100	0.008	1.6E-35	trans
386	CD83	Q01151	CD83 antigen	rs4753126	93862020	11	T	C	0.501	0.051	0.007	2.3E-12	trans
386	CD83	Q01151	CD83 antigen	rs76428106	28604007	13	C	T	0.013	0.354	0.033	2.5E-26	trans
386	CD83	Q01151	CD83 antigen	rs186021206	7069412	17	A	G	0.006	0.447	0.050	4.6E-19	trans
386	CD83	Q01151	CD83 antigen	rs2150879	57859210	17	A	G	0.553	0.050	0.007	7.3E-12	trans
386	CD83	Q01151	CD83 antigen	rs34878396	3546264	19	T	C	0.013	0.302	0.033	2.0E-20	trans
387	CD84	Q9UIB8	SLAM family member 5	rs41315846	247712303	1	T	C	0.523	0.055	0.008	2.3E-13	trans

387	CD84	Q9UIB8	SLAM family member 5	rs1354034	56849749	3	C	T	0.604	0.084	0.008	2.2E-28	trans
387	CD84	Q9UIB8	SLAM family member 5	rs114694170	88180196	5	C	T	0.060	0.132	0.016	1.2E-16	trans
387	CD84	Q9UIB8	SLAM family member 5	rs6993770	106581528	8	A	T	0.713	0.078	0.008	3.6E-21	trans
387	CD84	Q9UIB8	SLAM family member 5	rs409801	4744743	9	C	T	0.507	0.066	0.007	6.8E-19	trans
387	CD84	Q9UIB8	SLAM family member 5	rs7080386	65048306	10	A	C	0.412	0.079	0.008	2.2E-25	trans
387	CD84	Q9UIB8	SLAM family member 5	rs10131298	103568614	14	A	T	0.245	0.215	0.009	6.4E-133	trans
387	CD84	Q9UIB8	SLAM family member 5	rs151165225	45760920	19	C	T	0.745	0.094	0.009	6.3E-28	trans
388	CD86	P42081	T-lymphocyte activation antigen CD86	rs139795227	92842367	1	A	C	0.985	0.341	0.031	4.2E-28	trans
388	CD86	P42081	T-lymphocyte activation antigen CD86	rs151088139	134893133	2	C	T	0.855	0.092	0.010	4.9E-19	trans
388	CD86	P42081	T-lymphocyte activation antigen CD86	rs13063578	47087837	3	T	A	0.602	0.084	0.007	5.5E-29	trans
388	CD86	P42081	T-lymphocyte activation antigen CD86	rs192337168	121817928	3	G	T	0.997	1.369	0.074	5.3E-76	cis
388	CD86	P42081	T-lymphocyte activation antigen CD86	rs6796	6502367	7	C	T	0.279	0.056	0.008	5.5E-12	trans
388	CD86	P42081	T-lymphocyte activation antigen CD86	rs635634	136155000	9	T	C	0.184	0.073	0.009	1.2E-14	trans
388	CD86	P42081	T-lymphocyte activation antigen CD86	rs60194243	126247299	11	C	T	0.941	0.283	0.015	2.4E-74	trans
388	CD86	P42081	T-lymphocyte activation antigen CD86	rs76428106	28604007	13	C	T	0.013	0.528	0.033	3.4E-56	trans
388	CD86	P42081	T-lymphocyte activation antigen CD86	rs186021206	7069412	17	A	G	0.006	1.220	0.050	9.0E-132	trans
389	CD8A	P01732	T-cell surface glycoprotein CD8 alpha chain	rs3020726	87016506	2	G	A	0.161	0.362	0.010	1.2E-292	cis
389	CD8A	P01732	T-cell surface glycoprotein CD8 alpha chain	rs35467801	35881130	5	G	GT	0.728	0.069	0.008	1.6E-17	trans
389	CD8A	P01732	T-cell surface glycoprotein CD8 alpha chain	rs12211087	31269946	6	A	T	0.092	0.472	0.013	2.0E-304	trans
389	CD8A	P01732	T-cell surface glycoprotein CD8 alpha chain	rs12360863	11311191	11	C	T	0.749	0.057	0.008	8.9E-12	trans
390	CD93	Q9NPY3	Complement component C1q receptor	rs61747728	179526214	1	T	C	0.038	0.177	0.020	2.7E-19	trans
390	CD93	Q9NPY3	Complement component C1q receptor	rs1260326	27730940	2	C	T	0.608	0.060	0.008	1.2E-14	trans
390	CD93	Q9NPY3	Complement component C1q receptor	rs9275180	32654278	6	C	T	0.527	0.052	0.008	6.7E-12	trans
390	CD93	Q9NPY3	Complement component C1q receptor	rs7821812	10644101	8	G	C	0.792	0.081	0.009	5.4E-18	trans
390	CD93	Q9NPY3	Complement component C1q receptor	rs10901252	136128000	9	C	G	0.061	0.140	0.016	1.4E-18	trans
391	CD99	P14209	CD99 antigen	rs1260326	27730940	2	C	T	0.608	0.057	0.007	7.9E-18	trans
391	CD99	P14209	CD99 antigen	rs12149545	56993161	16	A	G	0.316	0.049	0.007	1.4E-12	trans
391	CD99	P14209	CD99 antigen	rs704	26694861	17	A	G	0.473	0.062	0.006	4.7E-22	trans
391	CD99	P14209	CD99 antigen	rs673408	29800119	18	A	G	0.544	0.107	0.006	6.6E-61	trans
392	CD99L2	Q8TCZ2	CD99 antigen-like protein 2	rs4665710	21221035	2	A	C	0.207	0.066	0.009	7.6E-13	trans
392	CD99L2	Q8TCZ2	CD99 antigen-like protein 2	rs139039156	122842256	3	G	A	0.991	0.294	0.042	3.7E-12	trans
392	CD99L2	Q8TCZ2	CD99 antigen-like protein 2	rs6054	155489608	4	T	C	0.006	0.387	0.050	1.5E-14	trans
392	CD99L2	Q8TCZ2	CD99 antigen-like protein 2	rs73015965	161127501	6	G	A	0.006	0.349	0.050	3.7E-12	trans
392	CD99L2	Q8TCZ2	CD99 antigen-like protein 2	rs896173	35735537	7	G	A	0.578	0.058	0.008	2.1E-14	trans
392	CD99L2	Q8TCZ2	CD99 antigen-like protein 2	rs11438680	65174698	10	GA	G	0.461	0.053	0.008	1.8E-12	trans
392	CD99L2	Q8TCZ2	CD99 antigen-like protein 2	rs174536	61551927	11	C	A	0.348	0.123	0.008	1.9E-55	trans
392	CD99L2	Q8TCZ2	CD99 antigen-like protein 2	rs1801689	64210580	17	C	A	0.030	0.481	0.022	1.4E-106	trans
392	CD99L2	Q8TCZ2	CD99 antigen-like protein 2	rs1065853	45413233	19	T	G	0.081	0.132	0.014	8.5E-22	trans
392	CD99L2	Q8TCZ2	CD99 antigen-like protein 2	rs7058184	150068138	X	T	C	0.871	0.204	0.009	6.1E-106	cis
393	CDA	P32320	Cytidine deaminase	rs2072671	20915701	1	A	C	0.656	0.489	0.008	0.0E+00	cis
393	CDA	P32320	Cytidine deaminase	rs6762442	15833903	3	G	C	0.676	0.052	0.007	1.8E-12	trans
393	CDA	P32320	Cytidine deaminase	rs10770	186389559	3	T	C	0.881	0.112	0.011	1.7E-26	trans
393	CDA	P32320	Cytidine deaminase	rs11775560	61660163	8	G	A	0.186	0.112	0.009	1.1E-36	trans
393	CDA	P32320	Cytidine deaminase	rs11993233	145002283	8	G	A	0.426	0.149	0.007	2.7E-101	trans
393	CDA	P32320	Cytidine deaminase	rs34433840	980416	10	C	T	0.760	0.066	0.008	1.3E-16	trans
393	CDA	P32320	Cytidine deaminase	rs61744929	2325427	11	C	T	0.019	0.172	0.025	5.7E-12	trans
393	CDA	P32320	Cytidine deaminase	rs9303283	38192633	17	C	T	0.436	0.064	0.007	2.1E-20	trans
393	CDA	P32320	Cytidine deaminase	rs4807399	1004710	19	T	C	0.533	0.054	0.007	6.7E-15	trans
394	CDAN1	Q8IWY9	Codanin-1	rs575220	24295103	22	G	A	0.540	0.055	0.008	8.4E-12	trans
395	CDC26	Q8NHZ8	Anaphase-promoting complex subunit CDC26	rs1354034	56849749	3	C	T	0.605	0.068	0.008	2.6E-19	trans
395	CDC26	Q8NHZ8	Anaphase-promoting complex subunit CDC26	rs7916868	64988931	10	T	A	0.502	0.051	0.007	1.1E-11	trans
395	CDC26	Q8NHZ8	Anaphase-promoting complex subunit CDC26	rs11502185	180258	11	C	T	0.260	0.068	0.010	1.5E-12	trans
396	CDC27	P30260	Cell division cycle protein 27 homolog	rs79702286	45296616	17	G	A	0.102	0.136	0.013	2.7E-27	cis
397	CDC37	Q16543	Hsp90 co-chaperone Cdc37	rs1354034	56849749	3	C	T	0.604	0.059	0.008	5.6E-15	trans
397	CDC37	Q16543	Hsp90 co-chaperone Cdc37	rs342298	106373646	7	C	T	0.545	0.061	0.008	4.0E-16	trans
397	CDC37	Q16543	Hsp90 co-chaperone Cdc37	rs10733789	64948684	10	C	T	0.313	0.055	0.008	1.4E-11	trans
398	CDC42BPB	Q9Y5S2	Serine/threonine-protein kinase MRCK beta	rs1354034	56849749	3	C	T	0.605	0.068	0.008	1.2E-19	trans
398	CDC42BPB	Q9Y5S2	Serine/threonine-protein kinase MRCK beta	rs342293	106372219	7	C	G	0.540	0.050	0.007	1.2E-11	trans
399	CDCP1	Q9H5V8	CUB domain-containing protein 1	rs2276862	45187785	3	C	G	0.828	0.303	0.009	7.1E-266	cis
399	CDCP1	Q9H5V8	CUB domain-containing protein 1	rs113787140	32502901	6	T	A	0.496	0.084	0.006	2.5E-39	trans
399	CDCP1	Q9H5V8	CUB domain-containing protein 1	rs77029323	64449879	10	T	G	0.404	0.055	0.007	9.2E-17	trans
399	CDCP1	Q9H5V8	CUB domain-containing protein 1	rs683486	126226663	11	C	A	0.597	0.127	0.007	3.4E-80	trans
399	CDCP1	Q9H5V8	CUB domain-containing protein 1	rs186021206	7069412	17	A	G	0.006	0.561	0.044	4.4E-37	trans
399	CDCP1	Q9H5V8	CUB domain-containing protein 1	rs738409	44324727	22	G	C	0.217	0.064	0.008	2.5E-16	trans
400	CDH1	P12830	Cadherin-1	rs532086	31881309	6	C	T	0.162	0.066	0.010	1.2E-11	trans
400	CDH1	P12830	Cadherin-1	rs2519093	136141870	9	C	T	0.816	0.352	0.009	2.1E-303	trans
400	CDH1	P12830	Cadherin-1	rs10749609	82249752	10	A	G	0.794	0.068	0.009	1.4E-14	trans
400	CDH1	P12830	Cadherin-1	rs186021206	7069412	17	A	G	0.006	0.439	0.049	3.6E-19	trans
400	CDH1	P12830	Cadherin-1	rs708686	5840619	19	T	C	0.268	0.097	0.008	4.1E-30	trans
400	CDH1	P12830	Cadherin-1	rs3814995	36342212	19	T	C	0.315	0.059	0.008	1.6E-13	trans
400	CDH1	P12830	Cadherin-1	rs681343	49206462	19	C	T	0.490	0.433	0.008	0.0E+00	trans
401	CDH15	P55291	Cadherin-15	rs10923344	118096628	1	A	G	0.618	0.049	0.006	7.8E-15	trans
401	CDH15	P55291	Cadherin-15	rs11646135	89199108	16	A	G	0.142	0.438	0.009	0.0E+00	cis
401	CDH15	P55291	Cadherin-15	rs2240715	19960606	22	C	G	0.299	0.062	0.007	2.2E-20	trans
402	CDH17	Q12864	Cadherin-17	rs12084158	120608444	1	G	A	0.098	0.093	0.012	2.2E-15	trans
402	CDH17	Q12864	Cadherin-17	rs12479244	107647860	2	T	A	0.288	0.146	0.007	3.2E-93	trans
402	CDH17	Q12864	Cadherin-17	rs9815073	188115682	3	C	A	0.657	0.061	0.007	1.4E-17	trans
402	CDH17	Q12864	Cadherin-17	rs3128921	33070749	6	A	C	0.259	0.091	0.007	1.4E-34	trans
402	CDH17	Q12864	Cadherin-17	rs4556017	100632790	7	C	T	0.146	0.120	0.009	5.6E-39	trans
402	CDH17	Q12864	Cadherin-17	rs12801491	126346868	11	A	G	0.195	0.061	0.008	7.6E-14	trans
402	CDH17	Q12864	Cadherin-17	rs2280381	86018633	16	C	T	0.386	0.051	0.007	2.4E-14	trans
402	CDH17	Q12864	Cadherin-17	rs186021206	7069412	17	A	G	0.006	0.543	0.044	2.2E-34	trans
402	CDH17	Q12864	Cadherin-17	rs708686	5840619	19	T	C	0.268	0.154	0.008	7.4E-90	trans
402	CDH17	Q12864	Cadherin-17	rs492602	49206417	19	A	G	0.491	0.382	0.007	0.0E+00	trans
403	CDH2	P19022	Cadherin-2	rs4074793	52193125	5	G	A	0.074	0.119	0.013	2.8E-19	trans
403	CDH2	P19022	Cadherin-2	rs62383006	156709342	5	A	G	0.946	0.187	0.015	2.5E-34	trans
403	CDH2	P19022	Cadherin-2	rs112875651	126506694	8	G	A	0.609	0.059	0.007	2.6E-16	trans
403	CDH2	P19022	Cadherin-2	rs181242111	17865664	10	A	G	0.118	0.089	0.011	1.3E-14	trans
403	CDH2	P19022	Cadherin-2	rs9633740	82265271	10	G	A	0.796	0.107	0.009	1.2E-35	trans
403	CDH2	P19022	Cadherin-2	rs7108216	702097	11	C	T	0.598	0.049	0.007	3.7E-12	trans
403	CDH2	P19022	Cadherin-2	rs28929474	94844947	14	T	C	0.021	0.281	0.024	3.5E-31	trans
403	CDH2	P19022	Cadherin-2	rs3785307	583360	16	T	C	0.885	0.146	0.011	8.8E-41	trans
403	CDH2	P19022	Cadherin-2	rs56192742	9595314	17	G	A	0.237	0.087	0.008	8.0E-27	trans
403	CDH2	P19022	Cadherin-2	rs12454164	25690927	18	G	A	0.689	0.101	0.007	5.1E-42	cis
403	CDH2	P19022	Cadherin-2	rs5927408	35475489	X	G	A	0.460	0.065	0.006	5.1E-30	trans
404	CDH23	Q9H251	Cadherin-23	rs2166631	73472882	10	C	T	0.786	0.168	0.009	4.6E-72	cis
405	CDH3	P22223	Cadherin-3	rs12137270	117264336	1	T	C	0.245	0.066	0.009	1.9E-14	trans
405	CDH3	P22223	Cadherin-3	rs61747728	179526214	1	T	C	0.038	0.141	0.019	3.0E-13	trans
405	CDH3	P22223	Cadherin-3	rs1260326	27730940	2	C	T	0.608	0.058	0.008	1.9E-14	trans
405	CDH3	P22223	Cadherin-3	rs4934189	82214792	10	T	C	0.536	0.090	0.007	1.5E-33	trans
405	CDH3	P22223	Cadherin-3	rs28640218	20359267	16	G	T	0.809	0.071	0.009	5.5E-14	trans
405	CDH3	P22223	Cadherin-3	rs3814995	36342212	19	T	C	0.315	0.064	0.008	1.2E-15	trans
406	CDH5	P33151	Cadherin-5	rs61802301	161575149	1	C	T	0.566	0.052	0.007	1.0E-13	trans
406	CDH5	P33151	Cadherin-5	rs1260326	27730940	2	C	T	0.608	0.060	0.007	9.0E-18	trans
406	CDH5	P33151	Cadherin-5	rs13107325	103188709	4	T	C	0.075	0.127	0.013	6.3E-23	trans
406	CDH5	P33151	Cadherin-5	rs10093	32609173	6	G	C	0.377	0.059	0.007	3.6E-16	trans
406	CDH5	P33151	Cadherin-5	rs8176743	136131415	9	T	C	0.061	1.205	0.015	0.0E+00	trans
406	CDH5	P33151	Cadherin-5	rs35166255	126301756	11	A	G	0.034	0.295	0.019	8.9E-56	trans
406	CDH5	P33151	Cadherin-5	rs16956504	66432381	16	C	T	0.104	0.308	0.011	7.3E-169	cis
406	CDH5	P33151	Cadherin-5	rs186021206	7069412	17	A	G	0.006	0.582	0.047	7.3E-36	trans
406	CDH5	P33151	Cadherin-5	rs3814995	36342212	19	T	C	0.315	0.052	0.007	7.4E-13	trans
406	CDH5	P33151	Cadherin-5	rs660745	49219459	19	T	C	0.455	0.051	0.007	5.2E-14	trans
407	CDH6	P55285	Cadherin-6	rs4626350	30839451	5	A	G	0.555	0.510	0.008	0.0E+00	cis
407	CDH6	P55285	Cadherin-6	rs6993770	106581528	8	T	A	0.287	0.073	0.007	2.4E-24	trans
407	CDH6	P55285	Cadherin-6	rs60757417	135864436	9	G	C	0.061	0.095	0.014	3.9E-12	trans
407	CDH6	P55285	Cadherin-6	rs186021206	7069412	17	A	G	0.006	0.550	0.045	9.1E-35	trans
407	CDH6	P55285	Cadherin-6	rs9306336	39873470	22	T	A	0.487	0.052	0.007	1.9E-15	trans
408	CDHR1	Q96JP9	Cadherin-related family member 1	rs61816761	152285861	1	A	G	0.023	0.200	0.026	4.4E-14	trans
408	CDHR1	Q96JP9	Cadherin-related family member 1	rs12330170	98506498	3	C	A	0.450	0.074	0.008	2.8E-22	trans
408	CDHR1	Q96JP9	Cadherin-related family member 1	rs73186030	122013465	3	T	C	0.128	0.105	0.011	1.7E-20	trans
408	CDHR1	Q96JP9	Cadherin-related family member 1	rs7916271	85954220	10	T	C	0.499	0.226	0.008	1.5E-187	cis
409	CDHR2	Q9BYE9	Cadherin-related family member 2	rs36086195	16510894	1	T	C	0.580	0.053	0.006	1.9E-16	trans
409	CDHR2	Q9BYE9	Cadherin-related family member 2	rs4835265	146821410	4	A	C	0.156	0.063	0.009	7.0E-13	trans
409	CDHR2	Q9BYE9	Cadherin-related family member 2	rs2963680	175942948	5	C	T	0.498	0.102	0.006	6.1E-57	cis
409	CDHR2	Q9BYE9	Cadherin-related family member 2	rs11621792	24871926	14	T	C	0.452	0.046	0.006	9.2E-13	trans
409	CDHR2	Q9BYE9	Cadherin-related family member 2	rs28929474	94844947	14	T	C	0.021	0.155	0.023	6.6E-12	trans
409	CDHR2	Q9BYE9	Cadherin-related family member 2	rs151184170	103570132	14	T	C	0.245	0.090	0.007	1.6E-33	trans
410	CDHR5	Q9HBB8	Cadherin-related family member 5	rs5993	197030829	1	T	A	0.161	0.256	0.009	1.7E-171	trans
410	CDHR5	Q9HBB8	Cadherin-related family member 5	rs35457250	186338564	3	T	C	0.009	0.305	0.036	1.3E-17	trans
410	CDHR5	Q9HBB8	Cadherin-related family member 5	rs3130297	32198981	6	C	T	0.867	0.071	0.010	7.3E-13	trans
410	CDHR5	Q9HBB8	Cadherin-related family member 5	rs10264306	28239466	7	G	A	0.196	0.073	0.008	4.2E-18	trans
410	CDHR5	Q9HBB8	Cadherin-related family member 5	rs12360820	620927	11	C	A	0.829	0.690	0.010	0.0E+00	cis
410	CDHR5	Q9HBB8	Cadherin-related family member 5	rs174566	61592362	11	G	A	0.350	0.056	0.008	1.1E-12	trans
410	CDHR5	Q9HBB8	Cadherin-related family member 5	rs28929474	94844947	14	T	C	0.021	0.231	0.023	7.4E-23	trans
410	CDHR5	Q9HBB8	Cadherin-related family member 5	rs8178824	64224775	17	T	C	0.030	0.257	0.020	7.2E-39	trans
411	CDK5RAP3	Q96JB5	CDK5 regulatory subunit-associated protein 3	rs1354034	56849749	3	C	T	0.604	0.083	0.008	2.5E-27	trans
411	CDK5RAP3	Q96JB5	CDK5 regulatory subunit-associated protein 3	rs9897552	33974481	17	A	G	0.177	0.086	0.010	2.5E-18	trans
412	CDKN1A	P38936	Cyclin-dependent kinase inhibitor 1	rs1354034	56849749	3	T	C	0.395	0.081	0.008	7.2E-27	trans
412	CDKN1A	P38936	Cyclin-dependent kinase inhibitor 1	rs3176345	36650565	6	G	C	0.935	0.268	0.015	8.1E-70	cis
412	CDKN1A	P38936	Cyclin-dependent kinase inhibitor 1	rs139141690	101499930	7	G	A	0.995	0.418	0.055	3.1E-14	trans
412	CDKN1A	P38936	Cyclin-dependent kinase inhibitor 1	rs6993770	106581528	8	A	T	0.714	0.066	0.008	1.3E-15	trans
412	CDKN1A	P38936	Cyclin-dependent kinase inhibitor 1	rs7080386	65048306	10	A	C	0.412	0.055	0.008	2.9E-13	trans
412	CDKN1A	P38936	Cyclin-dependent kinase inhibitor 1	rs964184	116648917	11	G	C	0.133	0.078	0.011	1.1E-12	trans

412	CDKN1A	P38936	Cyclin-dependent kinase inhibitor 1	rs58345103	69203039	14	G	A	0.813	0.077	0.010	1.1E-15	trans
413	CDKN2D	P55273	Cyclin-dependent kinase 4 inhibitor D	rs1354034	56849749	3	C	T	0.605	0.109	0.008	4.3E-46	trans
413	CDKN2D	P55273	Cyclin-dependent kinase 4 inhibitor D	rs17622656	131820997	5	G	A	0.611	0.056	0.008	6.3E-13	trans
413	CDKN2D	P55273	Cyclin-dependent kinase 4 inhibitor D	rs342298	106373646	7	C	T	0.546	0.051	0.008	1.5E-11	trans
413	CDKN2D	P55273	Cyclin-dependent kinase 4 inhibitor D	rs6993770	106581528	8	A	T	0.714	0.084	0.008	7.7E-24	trans
413	CDKN2D	P55273	Cyclin-dependent kinase 4 inhibitor D	rs544626513	180302	11	C	G	0.229	0.067	0.009	6.1E-13	trans
414	CDNF	Q49AH0	Cerebral dopamine neurotrophic factor	rs61738953	14862082	10	C	G	0.965	1.129	0.021	0.0E+00	cis
415	CDON	Q4KMG0	Cell adhesion molecule-related/down-regulated by oncogenes	rs607980	126002683	11	G	A	0.095	0.181	0.013	4.8E-47	cis
415	CDON	Q4KMG0	Cell adhesion molecule-related/down-regulated by oncogenes	rs186021206	7069412	17	A	G	0.006	0.631	0.050	2.0E-36	trans
416	CDSN	Q15517	Corneodesmosin	rs72674184	59058094	1	T	C	0.921	0.092	0.012	8.2E-15	trans
416	CDSN	Q15517	Corneodesmosin	rs61816761	152285861	1	A	G	0.024	0.554	0.022	9.6E-136	trans
416	CDSN	Q15517	Corneodesmosin	rs7545538	209979613	1	C	G	0.574	0.046	0.007	1.9E-12	trans
416	CDSN	Q15517	Corneodesmosin	rs1436662	190021928	3	G	A	0.594	0.071	0.006	3.7E-28	trans
416	CDSN	Q15517	Corneodesmosin	rs75278193	7433851	4	A	C	0.812	0.070	0.008	6.8E-18	trans
416	CDSN	Q15517	Corneodesmosin	rs3095306	31063601	6	A	T	0.610	0.644	0.008	0.0E+00	cis
416	CDSN	Q15517	Corneodesmosin	rs7815687	6723173	8	T	C	0.163	0.086	0.009	2.8E-23	trans
416	CDSN	Q15517	Corneodesmosin	rs3758294	107664815	9	T	C	0.796	0.054	0.008	8.1E-12	trans
416	CDSN	Q15517	Corneodesmosin	rs17658212	41145919	15	C	T	0.940	0.268	0.014	4.9E-85	trans
416	CDSN	Q15517	Corneodesmosin	rs77924615	20392332	16	G	A	0.803	0.055	0.008	6.8E-12	trans
416	CDSN	Q15517	Corneodesmosin	rs35187787	68855966	16	G	A	0.994	0.387	0.044	1.9E-18	trans
416	CDSN	Q15517	Corneodesmosin	rs729988	45926457	17	G	C	0.631	0.050	0.007	1.9E-14	trans
416	CDSN	Q15517	Corneodesmosin	rs7253823	38784550	19	C	T	0.832	0.185	0.009	3.2E-105	trans
416	CDSN	Q15517	Corneodesmosin	rs16981329	50097932	19	C	G	0.227	0.060	0.008	3.4E-15	trans
416	CDSN	Q15517	Corneodesmosin	rs12625565	39986085	20	A	C	0.776	0.051	0.008	1.6E-11	trans
416	CDSN	Q15517	Corneodesmosin	rs10521460	49759745	X	T	A	0.077	0.071	0.010	7.2E-13	trans
416	CDSN	Q15517	Corneodesmosin	rs2145205	115383943	X	A	G	0.276	0.044	0.006	9.8E-14	trans
417	CEACAM1	P13688	Carcinoembryonic antigen-related cell adhesion molecule 1	rs36086195	16510894	1	T	C	0.579	0.055	0.007	2.4E-13	trans
417	CEACAM1	P13688	Carcinoembryonic antigen-related cell adhesion molecule 1	rs114165349	27021913	1	C	G	0.023	0.169	0.025	6.7E-12	trans
417	CEACAM1	P13688	Carcinoembryonic antigen-related cell adhesion molecule 1	rs4835265	146821410	4	A	C	0.156	0.076	0.010	6.1E-14	trans
417	CEACAM1	P13688	Carcinoembryonic antigen-related cell adhesion molecule 1	rs56956502	103565044	14	A	G	0.248	0.067	0.009	7.7E-15	trans
417	CEACAM1	P13688	Carcinoembryonic antigen-related cell adhesion molecule 1	rs10416089	43002369	19	C	G	0.915	0.483	0.013	5.9E-282	cis
418	CEACAM16	Q2WEN9	Carcinoembryonic antigen-related cell adhesion molecule 16	rs12747765	27195907	1	C	A	0.842	0.074	0.009	5.2E-15	trans
418	CEACAM16	Q2WEN9	Carcinoembryonic antigen-related cell adhesion molecule 16	rs1260326	27730940	2	C	T	0.608	0.085	0.007	2.9E-33	trans
418	CEACAM16	Q2WEN9	Carcinoembryonic antigen-related cell adhesion molecule 16	rs6460047	73042443	7	C	T	0.208	0.058	0.008	6.0E-12	trans
418	CEACAM16	Q2WEN9	Carcinoembryonic antigen-related cell adhesion molecule 16	rs28601761	126500031	8	G	C	0.419	0.105	0.007	8.6E-50	trans
418	CEACAM16	Q2WEN9	Carcinoembryonic antigen-related cell adhesion molecule 16	rs2068888	94839642	10	G	A	0.549	0.051	0.007	2.6E-13	trans
418	CEACAM16	Q2WEN9	Carcinoembryonic antigen-related cell adhesion molecule 16	rs17715069	45209443	19	G	C	0.619	0.335	0.008	0.0E+00	cis
418	CEACAM16	Q2WEN9	Carcinoembryonic antigen-related cell adhesion molecule 16	rs200991074	109888230	X	GCCT	G	0.391	0.102	0.006	1.5E-66	trans
419	CEACAM19	Q7Z692	Carcinoembryonic antigen-related cell adhesion molecule 19	rs12738484	118091945	1	C	G	0.769	0.067	0.009	2.1E-14	trans
419	CEACAM19	Q7Z692	Carcinoembryonic antigen-related cell adhesion molecule 19	rs61816761	152285861	1	A	G	0.023	0.205	0.026	1.8E-15	trans
419	CEACAM19	Q7Z692	Carcinoembryonic antigen-related cell adhesion molecule 19	rs776021	37936464	9	A	C	0.737	0.084	0.008	1.4E-23	trans
419	CEACAM19	Q7Z692	Carcinoembryonic antigen-related cell adhesion molecule 19	rs35673728	41047777	15	T	C	0.937	0.106	0.015	2.0E-12	trans
419	CEACAM19	Q7Z692	Carcinoembryonic antigen-related cell adhesion molecule 19	rs150985681	1236396	19	G	A	0.974	0.159	0.023	7.5E-12	trans
419	CEACAM19	Q7Z692	Carcinoembryonic antigen-related cell adhesion molecule 19	rs62119322	45175069	19	C	G	0.029	0.623	0.022	2.1E-176	cis
420	CEACAM20	Q6UY09	Carcinoembryonic antigen-related cell adhesion molecule 20	rs10414398	45021210	19	G	A	0.920	0.283	0.014	3.8E-91	cis
420	CEACAM20	Q6UY09	Carcinoembryonic antigen-related cell adhesion molecule 20	rs681343	49206462	19	C	T	0.491	0.161	0.008	6.4E-98	trans
421	CEACAM3	P40198	Carcinoembryonic antigen-related cell adhesion molecule 3	rs139026664	89936251	2	A	C	0.084	0.187	0.015	2.5E-37	trans
421	CEACAM3	P40198	Carcinoembryonic antigen-related cell adhesion molecule 3	rs62148538	95371194	2	C	T	0.044	0.183	0.020	1.1E-20	trans
422	CEACAM5	P06731	Carcinoembryonic antigen-related cell adhesion molecule 5	rs12523278	60563907	5	G	A	0.502	0.052	0.007	2.0E-13	trans
422	CEACAM5	P06731	Carcinoembryonic antigen-related cell adhesion molecule 5	rs60750824	99927436	7	A	G	0.816	0.065	0.009	3.4E-12	trans
422	CEACAM5	P06731	Carcinoembryonic antigen-related cell adhesion molecule 5	rs4242701	36267261	9	A	G	0.565	0.057	0.007	4.2E-15	trans
422	CEACAM5	P06731	Carcinoembryonic antigen-related cell adhesion molecule 5	rs375524395	109507030	9	A	G	0.544	0.104	0.007	1.2E-46	trans
422	CEACAM5	P06731	Carcinoembryonic antigen-related cell adhesion molecule 5	rs12799864	126353484	11	T	C	0.196	0.080	0.009	2.5E-19	trans
422	CEACAM5	P06731	Carcinoembryonic antigen-related cell adhesion molecule 5	rs7332827	109923414	13	A	G	0.711	0.056	0.008	6.4E-13	trans
422	CEACAM5	P06731	Carcinoembryonic antigen-related cell adhesion molecule 5	rs186021206	7069412	17	A	G	0.006	0.341	0.049	4.6E-12	trans
422	CEACAM5	P06731	Carcinoembryonic antigen-related cell adhesion molecule 5	rs117990138	46116462	17	G	A	0.930	0.099	0.014	6.5E-13	trans
422	CEACAM5	P06731	Carcinoembryonic antigen-related cell adhesion molecule 5	rs708686	5840619	19	T	C	0.267	0.121	0.009	3.3E-45	trans
422	CEACAM5	P06731	Carcinoembryonic antigen-related cell adhesion molecule 5	rs9621	42231159	19	A	G	0.043	0.963	0.019	0.0E+00	cis
422	CEACAM5	P06731	Carcinoembryonic antigen-related cell adhesion molecule 5	rs681343	49206462	19	T	C	0.509	0.302	0.008	0.0E+00	trans
422	CEACAM5	P06731	Carcinoembryonic antigen-related cell adhesion molecule 5	rs441810	42698907	21	G	A	0.215	0.096	0.009	6.6E-29	trans
423	CEACAM6	P40199	Carcinoembryonic antigen-related cell adhesion molecule 6	rs11127048	27752463	2	A	G	0.618	0.055	0.008	1.6E-12	trans
423	CEACAM6	P40199	Carcinoembryonic antigen-related cell adhesion molecule 6	rs113542380	43464818	2	A	G	0.074	0.115	0.014	7.8E-16	trans
423	CEACAM6	P40199	Carcinoembryonic antigen-related cell adhesion molecule 6	rs12619285	213824045	2	A	G	0.734	0.070	0.008	5.2E-17	trans
423	CEACAM6	P40199	Carcinoembryonic antigen-related cell adhesion molecule 6	rs55734389	7172221	6	G	C	0.139	0.075	0.011	1.8E-12	trans
423	CEACAM6	P40199	Carcinoembryonic antigen-related cell adhesion molecule 6	rs73404209	31405671	6	C	T	0.026	0.192	0.023	2.3E-16	trans
423	CEACAM6	P40199	Carcinoembryonic antigen-related cell adhesion molecule 6	rs11775560	61660163	8	G	A	0.186	0.148	0.009	5.5E-55	trans
423	CEACAM6	P40199	Carcinoembryonic antigen-related cell adhesion molecule 6	rs375524395	109507030	9	A	G	0.544	0.061	0.007	2.8E-16	trans
423	CEACAM6	P40199	Carcinoembryonic antigen-related cell adhesion molecule 6	rs73017385	126179575	11	G	A	0.760	0.133	0.009	4.5E-53	trans
423	CEACAM6	P40199	Carcinoembryonic antigen-related cell adhesion molecule 6	rs186021206	7069412	17	A	G	0.006	0.684	0.051	4.2E-41	trans
423	CEACAM6	P40199	Carcinoembryonic antigen-related cell adhesion molecule 6	rs12453334	38153473	17	C	T	0.634	0.076	0.008	2.3E-23	trans
423	CEACAM6	P40199	Carcinoembryonic antigen-related cell adhesion molecule 6	rs56378716	56356502	17	G	A	0.013	0.516	0.032	7.1E-58	trans
423	CEACAM6	P40199	Carcinoembryonic antigen-related cell adhesion molecule 6	rs12459454	42263143	19	A	G	0.708	0.120	0.008	6.8E-50	cis
423	CEACAM6	P40199	Carcinoembryonic antigen-related cell adhesion molecule 6	rs492602	49206417	19	G	A	0.509	0.107	0.007	3.8E-47	trans
423	CEACAM6	P40199	Carcinoembryonic antigen-related cell adhesion molecule 6	rs28398212	61206323	20	T	C	0.574	0.052	0.008	7.7E-12	trans
423	CEACAM6	P40199	Carcinoembryonic antigen-related cell adhesion molecule 6	rs7275212	39852551	21	T	A	0.008	0.512	0.041	1.2E-36	trans
424	CEACAM8	P31997	Carcinoembryonic antigen-related cell adhesion molecule 8	rs3917932	36943916	1	C	G	0.425	0.056	0.007	8.8E-14	trans
424	CEACAM8	P31997	Carcinoembryonic antigen-related cell adhesion molecule 8	rs112694524	43453721	2	A	G	0.070	0.100	0.015	1.0E-11	trans
424	CEACAM8	P31997	Carcinoembryonic antigen-related cell adhesion molecule 8	rs2236947	50371432	3	A	C	0.482	0.086	0.007	3.6E-31	trans
424	CEACAM8	P31997	Carcinoembryonic antigen-related cell adhesion molecule 8	rs3774298	187460099	3	A	G	0.687	0.071	0.008	6.9E-18	trans
424	CEACAM8	P31997	Carcinoembryonic antigen-related cell adhesion molecule 8	rs2738144	6826043	8	A	T	0.665	0.065	0.008	8.4E-16	trans
424	CEACAM8	P31997	Carcinoembryonic antigen-related cell adhesion molecule 8	rs35440906	61646351	8	CA	C	0.185	0.209	0.009	2.2E-107	trans
424	CEACAM8	P31997	Carcinoembryonic antigen-related cell adhesion molecule 8	rs1058900	308290	11	C	T	0.566	0.058	0.008	2.3E-14	trans
424	CEACAM8	P31997	Carcinoembryonic antigen-related cell adhesion molecule 8	rs2511241	72945341	11	T	C	0.929	0.165	0.014	1.6E-30	trans
424	CEACAM8	P31997	Carcinoembryonic antigen-related cell adhesion molecule 8	rs6503533	38184580	17	C	T	0.383	0.106	0.008	3.4E-44	trans
424	CEACAM8	P31997	Carcinoembryonic antigen-related cell adhesion molecule 8	rs114641762	42417164	17	C	A	0.896	0.248	0.012	1.5E-88	trans
424	CEACAM8	P31997	Carcinoembryonic antigen-related cell adhesion molecule 8	rs12459454	42263143	19	A	G	0.708	0.131	0.008	3.6E-59	cis
425	CEBPA	P49715	CCAAT/enhancer-binding protein alpha	rs28929474	94844947	14	T	C	0.021	0.194	0.027	8.4E-13	trans
426	CEBPB	P17676	CCAAT/enhancer-binding protein beta	rs1045929	38175426	17	T	C	0.387	0.066	0.008	5.3E-18	trans
426	CEBPB	P17676	CCAAT/enhancer-binding protein beta	rs10404947	1081617	19	G	A	0.802	0.070	0.009	9.1E-14	trans
427	CELA2A	P08217	Chymotrypsin-like elastase family member 2A	rs188524036	15798603	1	T	G	0.988	0.535	0.035	6.3E-52	cis
427	CELA2A	P08217	Chymotrypsin-like elastase family member 2A	rs187351243	22292098	1	A	G	0.021	0.260	0.026	2.0E-23	trans
427	CELA2A	P08217	Chymotrypsin-like elastase family member 2A	rs35004807	51108411	1	TGTAA	T	0.344	0.074	0.008	4.1E-21	trans
427	CELA2A	P08217	Chymotrypsin-like elastase family member 2A	rs17032925	67266483	2	T	C	0.918	0.168	0.013	1.4E-35	trans
427	CELA2A	P08217	Chymotrypsin-like elastase family member 2A	rs2842895	7106316	6	G	C	0.449	0.053	0.007	9.8E-13	trans
427	CELA2A	P08217	Chymotrypsin-like elastase family member 2A	rs9388490	126704795	6	C	T	0.560	0.066	0.007	7.5E-19	trans
427	CELA2A	P08217	Chymotrypsin-like elastase family member 2A	rs13221897	97719699	7	G	A	0.436	0.055	0.007	2.0E-13	trans
427	CELA2A	P08217	Chymotrypsin-like elastase family member 2A	rs13226219	130019491	7	T	C	0.871	0.110	0.011	3.6E-23	trans
427	CELA2A	P08217	Chymotrypsin-like elastase family member 2A	rs77643237	135889210	9	A	G	0.055	0.242	0.017	2.2E-48	trans
427	CELA2A	P08217	Chymotrypsin-like elastase family member 2A	rs77958433	49371043	10	T	C	0.366	0.067	0.008	1.3E-16	trans
427	CELA2A	P08217	Chymotrypsin-like elastase family member 2A	rs10994860	52645424	10	C	T	0.823	0.067	0.010	3.3E-12	trans
427	CELA2A	P08217	Chymotrypsin-like elastase family member 2A	rs4246215	61564299	11	T	G	0.360	0.061	0.008	2.2E-15	trans
427	CELA2A	P08217	Chymotrypsin-like elastase family member 2A	rs28929474	94844947	14	C	T	0.979	0.662	0.026	3.3E-144	trans
427	CELA2A	P08217	Chymotrypsin-like elastase family member 2A	rs28365941	333138	16	T	C	0.864	0.109	0.011	7.6E-23	trans
427	CELA2A	P08217	Chymotrypsin-like elastase family member 2A	rs2287796	25240163	16	T	G	0.407	0.054	0.008	1.1E-12	trans
427	CELA2A	P08217	Chymotrypsin-like elastase family member 2A	rs72802342	75234872	16	C	A	0.924	0.116	0.014	4.7E-16	trans
427	CELA2A	P08217	Chymotrypsin-like elastase family member 2A	rs529900	88976968	16	G	C	0.621	0.169	0.008	1.9E-109	trans
427	CELA2A	P08217	Chymotrypsin-like elastase family member 2A	rs8088688	42394381	18	T	A	0.077	0.108	0.014	6.4E-15	trans
428	CELA3A	P09093	Chymotrypsin-like elastase family member 3A	rs34262568	22313371	1	A	G	0.231	0.237	0.009	4.1E-167	cis
428	CELA3A	P09093	Chymotrypsin-like elastase family member 3A	rs333947	110470764	1	G	A	0.852	0.076	0.010	8.8E-14	trans
428	CELA3A	P09093	Chymotrypsin-like elastase family member 3A	rs17032925	67266483	2	T	C	0.917	0.099	0.013	3.1E-14	trans
428	CELA3A	P09093	Chymotrypsin-like elastase family member 3A	rs6571015	96534478	6	A	G	0.790	0.157	0.009	1.1E-70	trans
428	CELA3A	P09093	Chymotrypsin-like elastase family member 3A	rs11778310	18774902	8	G	A	0.294	0.072	0.008	5.0E-20	trans
428	CELA3A	P09093	Chymotrypsin-like elastase family member 3A	rs8176747	136131315	9	G	C	0.061	0.500	0.015	2.2E-243	trans
428	CELA3A	P09093	Chymotrypsin-like elastase family member 3A	rs56278466	17875857	10	G	T	0.661	0.060	0.008	2.2E-15	trans
428	CELA3A	P09093	Chymotrypsin-like elastase family member 3A	rs61910478	100592308	11	A	T	0.731	0.070	0.008	2.7E-18	trans
428	CELA3A	P09093	Chymotrypsin-like elastase family member 3A	rs6575449	95097303	14	C	T	0.815	0.110	0.009	2.9E-32	trans
428	CELA3A	P09093	Chymotrypsin-like elastase family member 3A	rs28365941	333138	16	T	C	0.864	0.082	0.011	3.0E-14	trans
428	CELA3A	P09093	Chymotrypsin-like elastase family member 3A	rs72802342	75234872	16	C	A	0.925	0.257	0.014	6.7E-77	trans
428	CELA3A	P09093	Chymotrypsin-like elastase family member 3A	rs529900	88976968	16	G	C	0.622	0.101	0.007	1.1E-42	trans
428	CELA3A	P09093	Chymotrypsin-like elastase family member 3A	rs681343	49206462	19	T	C	0.509	0.197	0.007	2.1E-163	trans
429	CELSR2	Q9HCU4	Cadherin EGF LAG seven-pass G-type receptor 2	rs12740374	109817590	1	T	G	0.221	0.460	0.009	0.0E+00	cis
429	CELSR2	Q9HCU4	Cadherin EGF LAG seven-pass G-type receptor 2	rs6637830	130423543	X	C	G	0.815	0.119	0.008	7.0E-54	trans
430	CEMIP2	Q9UHN6	Cell surface hyaluronidase	rs28601761	126500031	8	C	G	0.581	0.054	0.008	4.7E-12	trans
430	CEMIP2	Q9UHN6	Cell surface hyaluronidase	rs191912523	74346987	9	G	T	0.083	0.254	0.014	9.0E-73	cis
431	CEND1	Q8N111	Cell cycle exit and neuronal differentiation protein 1	rs429358	45411941	19	C	T	0.156	0.078	0.011	3.4E-13	trans
432	CEP112	Q8N8E3	Centrosomal protein of 112 kDa	rs12938334	63934200	17	A	C	0.567	0.229	0.008	4.0E-185	cis
433	CEP152	O94986	Centrosomal protein of 152 kDa	rs77379665	49082509	15	T	C	0.033	0.157	0.022	4.3E-13	cis
434	CEP170	Q5SW79	Centrosomal protein of 170 kDa	rs1354034	56849749	3	C	T	0.605	0.062	0.008	4.3E-16	trans
435	CEP20	Q96NB1	Centrosomal protein 20	rs1354034	56849749	3	C	T	0.604	0.101	0.008	1.7E-38	trans
435	CEP20	Q96NB1	Centrosomal protein 20	rs342298	106373646	7	C	T	0.545	0.057	0.008	9.2E-14	trans
435	CEP20	Q96NB1	Centrosomal protein 20	rs73396521	267568	11	A	G	0.245	0.063	0.009	1.2E-12	trans
435	CEP20	Q96NB1	Centrosomal protein 20	rs74009487	15965897	16	C	T	0.944	0.216	0.017	1.4E-38	cis
435	CEP20	Q96NB1	Centrosomal protein 20	rs200309755	27845774	17	T	C	0.537	0.054	0.008	9.4E-12	trans
436	CEP43	O95684	Centrosomal protein 43	rs2501620	179944928	1	A	C	0.350	0.064	0.008	4.4E-16	trans
436	CEP43	O95684	Centrosomal protein 43	rs2237276	167442115	6	T	C	0.455	0.065	0.008	7.5E-18	cis
436	CEP43	O95684	Centrosomal protein 43	rs342298	106373646	7	C	T	0.545	0.063	0.008	7.6E-17	trans
437	CEP85	Q6P2H3	Centrosomal protein of 85 kDa	rs1354034	56849749	3	C	T	0.604	0.117	0.008	4.4E-53	trans
437	CEP85	Q6P2H3	Centrosomal protein of 85 kDa	rs342293	106372219	7	C	G	0.540	0.064	0.007	1.8E-17	trans
437	CEP85	Q6P2H3	Centrosomal protein of 85 kDa	rs11604127	196944	11	T	C	0.231	0.062	0.009	4.9E-12	trans
438	CERT	Q9Y5P4	Ceramide transfer protein	rs1354034	56849749	3	C	T	0.604	0.084	0.008	2.5E-27	trans
438	CERT	Q9Y5P4	Ceramide transfer protein	rs10942737	74682813	5	C	T	0.082	0.101	0.014	4.6E-13	cis
438	CERT	Q9Y5P4	Ceramide transfer protein	rs342298	106373646	7	C	T	0.546	0.054	0.008	1.5E-12	trans
439	CES1	P23141	Liver carboxylesterase 1	rs12129540	224625334	1	T	C	0.043	0.117	0.017	4.7E-12	trans

439	CES1	P23141	Liver carboxylesterase 1	rs1260326	27730940	2	T	C	0.392	0.097	0.007	3.8E-43	trans
439	CES1	P23141	Liver carboxylesterase 1	rs7640956	57634135	3	A	G	0.178	0.126	0.009	3.9E-45	trans
439	CES1	P23141	Liver carboxylesterase 1	rs17259257	119778889	4	T	C	0.056	0.136	0.015	1.2E-19	trans
439	CES1	P23141	Liver carboxylesterase 1	rs187515714	126880275	5	T	G	0.026	0.147	0.021	8.0E-12	trans
439	CES1	P23141	Liver carboxylesterase 1	rs13240994	73016862	7	T	C	0.804	0.066	0.009	1.7E-14	trans
439	CES1	P23141	Liver carboxylesterase 1	rs75231243	127122272	7	G	A	0.932	0.135	0.014	6.6E-23	trans
439	CES1	P23141	Liver carboxylesterase 1	rs112875651	126506694	8	G	A	0.609	0.059	0.007	9.2E-17	trans
439	CES1	P23141	Liver carboxylesterase 1	rs72775768	139324574	9	C	T	0.711	0.065	0.008	1.1E-17	trans
439	CES1	P23141	Liver carboxylesterase 1	rs2393969	65140440	10	A	C	0.532	0.062	0.007	1.7E-19	trans
439	CES1	P23141	Liver carboxylesterase 1	rs143168962	55849073	16	T	G	0.943	1.152	0.016	0.0E+00	cis
439	CES1	P23141	Liver carboxylesterase 1	rs58542926	19379549	19	T	C	0.076	0.191	0.013	3.1E-49	trans
439	CES1	P23141	Liver carboxylesterase 1	rs7256200	45415935	19	G	T	0.872	0.098	0.010	1.8E-21	trans
439	CES1	P23141	Liver carboxylesterase 1	rs641738	54676763	19	C	T	0.558	0.050	0.007	5.3E-13	trans
440	CES2	O00748	Cocaine esterase	rs140461033	66971964	16	G	A	0.996	1.050	0.062	9.7E-65	cis
441	CES3	Q6UWW8	Carboxylesterase 3	rs193084249	26987646	1	G	A	0.023	0.229	0.025	2.6E-20	trans
441	CES3	Q6UWW8	Carboxylesterase 3	rs62260502	57559369	3	A	G	0.179	0.133	0.010	1.9E-43	trans
441	CES3	Q6UWW8	Carboxylesterase 3	rs73015965	161127501	6	G	A	0.006	0.581	0.050	9.0E-32	trans
441	CES3	Q6UWW8	Carboxylesterase 3	rs17715183	127195197	7	G	C	0.933	0.150	0.015	5.4E-24	trans
441	CES3	Q6UWW8	Carboxylesterase 3	rs9987289	9183358	8	A	G	0.092	0.176	0.013	2.0E-43	trans
441	CES3	Q6UWW8	Carboxylesterase 3	rs71647892	66997756	16	T	C	0.987	0.854	0.033	2.3E-145	cis
441	CES3	Q6UWW8	Carboxylesterase 3	rs11668882	54675097	19	T	C	0.559	0.060	0.007	6.5E-16	trans
442	CETN2	P41208	Centrin-2	rs1354034	56849749	3	C	T	0.604	0.085	0.008	1.2E-27	trans
442	CETN2	P41208	Centrin-2	rs342296	106372903	7	G	A	0.544	0.065	0.008	1.1E-17	trans
442	CETN2	P41208	Centrin-2	rs117564216	31949927	22	G	A	0.984	0.248	0.031	4.2E-16	trans
443	CETN3	O15182	Centrin-3	rs1354034	56849749	3	C	T	0.605	0.108	0.008	2.6E-46	trans
443	CETN3	O15182	Centrin-3	rs4873	89703641	5	C	G	0.723	0.173	0.008	4.0E-93	cis
443	CETN3	O15182	Centrin-3	rs958976	108747204	5	C	T	0.588	0.073	0.008	7.4E-22	trans
443	CETN3	O15182	Centrin-3	rs342298	106373646	7	C	T	0.545	0.069	0.007	3.0E-20	trans
443	CETN3	O15182	Centrin-3	rs113315674	100720053	9	A	G	0.365	0.054	0.008	3.7E-12	trans
443	CETN3	O15182	Centrin-3	rs5998067	32043630	22	G	A	0.293	0.138	0.008	1.2E-63	trans
444	CFB	P00751	Complement factor B	rs2228145	154426970	1	A	C	0.594	0.049	0.007	5.6E-12	trans
444	CFB	P00751	Complement factor B	rs10801552	196609262	1	C	T	0.229	0.185	0.008	2.1E-109	trans
444	CFB	P00751	Complement factor B	rs1260326	27730940	2	T	C	0.392	0.067	0.007	7.9E-21	trans
444	CFB	P00751	Complement factor B	rs10033900	110659067	4	C	T	0.516	0.050	0.007	1.0E-12	trans
444	CFB	P00751	Complement factor B	rs641153	31914180	6	A	G	0.096	0.630	0.013	0.0E+00	cis
444	CFB	P00751	Complement factor B	rs28601761	126500031	8	G	C	0.420	0.060	0.007	2.8E-17	trans
444	CFB	P00751	Complement factor B	rs12975366	54759361	19	T	C	0.604	0.049	0.007	6.5E-12	trans
445	CFC1	P0CG37	Cryptic protein	rs11708067	123065778	3	G	A	0.245	0.068	0.009	1.3E-14	trans
445	CFC1	P0CG37	Cryptic protein	rs6790951	160025287	3	T	C	0.479	0.060	0.008	4.1E-15	trans
445	CFC1	P0CG37	Cryptic protein	rs2517546	31009055	6	C	T	0.829	0.075	0.010	9.5E-14	trans
445	CFC1	P0CG37	Cryptic protein	rs35510806	126678331	6	T	TA	0.529	0.052	0.008	7.8E-12	trans
445	CFC1	P0CG37	Cryptic protein	rs2168101	8255408	11	A	C	0.311	0.062	0.008	3.3E-13	trans
445	CFC1	P0CG37	Cryptic protein	rs8038040	62394339	15	A	G	0.427	0.069	0.008	2.8E-19	trans
445	CFC1	P0CG37	Cryptic protein	rs28461437	423813	16	C	G	0.404	0.098	0.008	1.8E-36	trans
445	CFC1	P0CG37	Cryptic protein	rs34931250	66879927	17	T	C	0.061	0.108	0.016	1.6E-11	trans
446	CFD	P00746	Complement factor D	rs698084	186997648	3	T	C	0.182	0.069	0.009	2.6E-13	trans
446	CFD	P00746	Complement factor D	rs704	26694861	17	G	A	0.526	0.065	0.007	2.9E-19	trans
446	CFD	P00746	Complement factor D	rs71335276	859368	19	G	T	0.773	0.185	0.009	1.4E-91	cis
447	CFH	P08603	Complement factor H	rs731557	196705015	1	A	C	0.194	0.421	0.010	0.0E+00	cis
447	CFH	P08603	Complement factor H	rs36019311	73051990	7	G	GTA	0.871	0.076	0.011	9.3E-12	trans
447	CFH	P08603	Complement factor H	rs73201506	33044408	21	A	G	0.955	0.137	0.018	2.3E-14	trans
448	CFHR2	P36980	Complement factor H-related protein 2	rs4085749	196920148	1	C	T	0.773	1.037	0.009	0.0E+00	cis
448	CFHR2	P36980	Complement factor H-related protein 2	rs1260326	27730940	2	T	C	0.392	0.031	0.005	8.7E-12	trans
449	CFHR4	Q92496	Complement factor H-related protein 4	rs4915363	196905205	1	T	C	0.764	0.793	0.009	0.0E+00	cis
449	CFHR4	Q92496	Complement factor H-related protein 4	rs141880390	172362200	5	G	A	0.997	0.506	0.043	5.2E-32	trans
449	CFHR4	Q92496	Complement factor H-related protein 4	rs1461729	9187242	8	G	A	0.897	0.054	0.008	3.2E-12	trans
449	CFHR4	Q92496	Complement factor H-related protein 4	rs41274050	52573772	10	T	C	0.010	0.174	0.023	1.3E-13	trans
449	CFHR4	Q92496	Complement factor H-related protein 4	rs664034	59930966	11	T	C	0.576	0.040	0.005	3.5E-17	trans
449	CFHR4	Q92496	Complement factor H-related protein 4	rs28929474	94844947	14	T	C	0.021	0.113	0.016	4.0E-12	trans
449	CFHR4	Q92496	Complement factor H-related protein 4	rs149394327	64228995	17	C	G	0.030	0.145	0.014	5.0E-26	trans
450	CFHR5	Q9BXR6	Complement factor H-related protein 5	rs165316	91533297	1	G	A	0.198	0.066	0.010	3.9E-12	trans
450	CFHR5	Q9BXR6	Complement factor H-related protein 5	rs72732234	196303889	1	T	C	0.983	1.589	0.030	0.0E+00	cis
450	CFHR5	Q9BXR6	Complement factor H-related protein 5	rs55709272	113867288	2	C	T	0.439	0.051	0.007	3.2E-13	trans
450	CFHR5	Q9BXR6	Complement factor H-related protein 5	rs28601761	126500031	8	C	G	0.580	0.060	0.007	2.6E-17	trans
450	CFHR5	Q9BXR6	Complement factor H-related protein 5	rs10740131	65271488	10	T	A	0.470	0.062	0.007	6.7E-19	trans
450	CFHR5	Q9BXR6	Complement factor H-related protein 5	rs72731579	69300821	14	A	G	0.835	0.067	0.010	3.5E-12	trans
451	CFI	P05156	Complement factor I	rs1260326	27730940	2	T	C	0.392	0.093	0.007	8.6E-36	trans
451	CFI	P05156	Complement factor I	rs10033900	110659067	4	C	T	0.516	0.216	0.007	1.3E-188	cis
451	CFI	P05156	Complement factor I	rs204886	32029415	6	C	T	0.506	0.053	0.007	2.2E-13	trans
452	CFP	P27918	Properdin	rs10737680	196679455	1	C	A	0.403	0.203	0.007	2.1E-163	trans
452	CFP	P27918	Properdin	rs4151667	31914024	6	A	T	0.046	0.399	0.017	6.5E-119	trans
452	CFP	P27918	Properdin	rs4752973	47269159	11	T	C	0.811	0.070	0.009	3.5E-14	trans
452	CFP	P27918	Properdin	rs186021206	7069412	17	A	G	0.006	0.424	0.049	1.0E-17	trans
452	CFP	P27918	Properdin	rs2230199	6718387	19	G	C	0.784	0.149	0.009	4.1E-63	trans
452	CFP	P27918	Properdin	rs61737993	47486217	X	C	T	0.999	1.425	0.084	6.6E-64	cis
453	CGA	P01215	Glycoprotein hormones alpha chain	rs11091775	50207907	X	C	T	0.289	0.039	0.005	3.5E-15	trans
454	CGB3_CGB5_CGB8	P0DN86	Choriogonadotropin subunit beta 3	rs16854211	169338409	3	T	G	0.158	0.077	0.010	1.3E-15	trans
454	CGB3_CGB5_CGB8	P0DN86	Choriogonadotropin subunit beta 3	rs7252920	49504260	19	A	C	0.514	0.061	0.007	3.4E-18	cis
455	CGREF1	Q99674	Cell growth regulator with EF hand domain protein 1	rs2384572	27324751	2	G	T	0.375	0.561	0.008	0.0E+00	cis
455	CGREF1	Q99674	Cell growth regulator with EF hand domain protein 1	rs9273206	32613573	6	C	T	0.835	0.078	0.009	4.6E-18	trans
455	CGREF1	Q99674	Cell growth regulator with EF hand domain protein 1	rs4940867	57043904	18	T	C	0.144	0.079	0.009	6.2E-17	trans
455	CGREF1	Q99674	Cell growth regulator with EF hand domain protein 1	rs492602	49206417	19	A	G	0.491	0.281	0.007	0.0E+00	trans
456	CHAC2	Q8WUX2	Glutathione-specific gamma-glutamylcyclotransferase 2	rs78694551	53978813	2	A	G	0.730	0.149	0.009	8.5E-69	cis
457	CHAD	O15335	Chondroadherin	rs2476601	114377568	1	G	A	0.899	0.084	0.012	3.3E-12	trans
457	CHAD	O15335	Chondroadherin	rs1386625	38361120	4	A	G	0.098	0.102	0.012	2.5E-16	trans
457	CHAD	O15335	Chondroadherin	rs1485307	120007395	8	C	T	0.542	0.088	0.007	2.7E-33	trans
457	CHAD	O15335	Chondroadherin	rs9594738	42952145	13	T	C	0.488	0.125	0.007	2.0E-65	trans
457	CHAD	O15335	Chondroadherin	rs11625758	75428234	14	T	C	0.505	0.056	0.007	1.5E-14	trans
457	CHAD	O15335	Chondroadherin	rs144030994	48542714	17	G	T	0.994	1.630	0.048	1.7E-257	cis
457	CHAD	O15335	Chondroadherin	rs884205	60054857	18	A	C	0.254	0.061	0.008	5.3E-13	trans
457	CHAD	O15335	Chondroadherin	rs12975366	54759361	19	T	C	0.605	0.097	0.007	1.7E-38	trans
458	CHCHD10	Q8WYQ3	Coiled-coil-helix-coiled-coil-helix domain-containing protein 10, mitochondrial	rs10502	90039670	6	G	C	0.619	0.050	0.007	1.2E-12	trans
458	CHCHD10	Q8WYQ3	Coiled-coil-helix-coiled-coil-helix domain-containing protein 10, mitochondrial	rs62143206	54326212	19	T	G	0.209	0.132	0.009	7.0E-54	trans
458	CHCHD10	Q8WYQ3	Coiled-coil-helix-coiled-coil-helix domain-containing protein 10, mitochondrial	rs34284791	24166789	22	G	A	0.893	0.118	0.011	8.5E-26	cis
459	CHCHD6	Q9BRQ6	MICOS complex subunit MIC25	rs34723687	86261291	2	G	C	0.492	0.052	0.007	2.7E-12	trans
459	CHCHD6	Q9BRQ6	MICOS complex subunit MIC25	rs9863101	126408304	3	T	C	0.827	0.306	0.010	6.2E-209	cis
460	CHGA	P10645	Chromogranin-A	rs56092644	95774809	5	T	G	0.245	0.097	0.009	1.9E-29	trans
460	CHGA	P10645	Chromogranin-A	rs1801020	176836532	5	A	G	0.255	0.069	0.008	2.3E-16	trans
460	CHGA	P10645	Chromogranin-A	rs17489891	20007573	8	G	C	0.888	0.160	0.012	9.0E-43	trans
460	CHGA	P10645	Chromogranin-A	rs11786721	143760179	8	C	T	0.410	0.054	0.007	5.1E-13	trans
460	CHGA	P10645	Chromogranin-A	rs9581957	28557889	13	T	C	0.328	0.054	0.008	5.2E-12	trans
460	CHGA	P10645	Chromogranin-A	rs729940	93399101	14	C	T	0.843	0.246	0.010	2.3E-129	cis
461	CHGB	P05060	Secretogranin-1	rs2509902	158514969	2	T	C	0.143	0.081	0.010	4.3E-17	trans
461	CHGB	P05060	Secretogranin-1	rs13159918	157892732	5	C	A	0.700	0.050	0.007	7.3E-12	trans
461	CHGB	P05060	Secretogranin-1	rs11154902	137301298	6	C	T	0.537	0.089	0.007	1.1E-39	trans
461	CHGB	P05060	Secretogranin-1	rs17092104	20005554	8	C	G	0.109	0.132	0.011	1.7E-34	trans
461	CHGB	P05060	Secretogranin-1	rs28688991	20367550	16	C	T	0.808	0.062	0.009	2.9E-13	trans
461	CHGB	P05060	Secretogranin-1	rs236153	5903894	20	A	G	0.628	0.611	0.008	0.0E+00	cis
462	CHI3L1	P36222	Chitinase-3-like protein 1	rs4950928	203155882	1	C	G	0.793	0.672	0.009	0.0E+00	cis
462	CHI3L1	P36222	Chitinase-3-like protein 1	rs11127048	27752463	2	G	A	0.382	0.059	0.007	9.8E-18	trans
462	CHI3L1	P36222	Chitinase-3-like protein 1	rs28601761	126500031	8	C	G	0.581	0.119	0.007	1.1E-69	trans
462	CHI3L1	P36222	Chitinase-3-like protein 1	rs339969	60883281	15	C	A	0.383	0.056	0.007	6.8E-17	trans
462	CHI3L1	P36222	Chitinase-3-like protein 1	rs62117298	53918063	19	T	C	0.519	0.062	0.007	6.1E-21	trans
463	CHIT1	Q13231	Chitotriosidase-1	rs872583	203184766	1	T	C	0.797	1.092	0.009	0.0E+00	cis
463	CHIT1	Q13231	Chitotriosidase-1	rs9272672	32608849	6	T	C	0.682	0.049	0.006	8.0E-16	trans
463	CHIT1	Q13231	Chitotriosidase-1	rs34743976	85611817	15	T	TA	0.721	0.049	0.006	8.7E-16	trans
464	CHL1	O00533	Neural cell adhesion molecule L1-like protein	rs931679	259525	3	A	T	0.087	0.536	0.013	0.0E+00	cis
464	CHL1	O00533	Neural cell adhesion molecule L1-like protein	rs12574844	126273386	11	G	A	0.929	0.116	0.014	9.7E-17	trans
464	CHL1	O00533	Neural cell adhesion molecule L1-like protein	rs186021206	7069412	17	A	G	0.006	0.716	0.049	3.7E-48	trans
464	CHL1	O00533	Neural cell adhesion molecule L1-like protein	rs8105767	22215441	19	A	G	0.713	0.054	0.008	5.0E-12	trans
465	CHM	P24386	Rab proteins geranylgeranyltransferase component A 1	rs1354034	56849749	3	C	T	0.604	0.061	0.008	5.1E-15	trans
466	CHMP1A	Q9HD42	Charged multivesicular body protein 1a	rs7080386	65048306	10	A	C	0.412	0.055	0.008	1.6E-12	trans
466	CHMP1A	Q9HD42	Charged multivesicular body protein 1a	rs460879	89712889	16	C	T	0.573	0.095	0.008	4.5E-35	cis
466	CHMP1A	Q9HD42	Charged multivesicular body protein 1a	rs1671152	55526345	19	G	T	0.841	0.073	0.010	3.1E-12	trans
467	CHMP6	Q96FZ7	Charged multivesicular body protein 6	rs1260326	27730940	2	T	C	0.391	0.058	0.008	5.4E-14	trans
467	CHMP6	Q96FZ7	Charged multivesicular body protein 6	rs1050152	131676320	5	C	T	0.566	0.053	0.008	2.0E-12	trans
467	CHMP6	Q96FZ7	Charged multivesicular body protein 6	rs342298	106373646	7	C	T	0.545	0.055	0.008	2.1E-13	trans
467	CHMP6	Q96FZ7	Charged multivesicular body protein 6	rs56239087	78970499	17	T	C	0.172	0.203	0.010	3.3E-93	cis
468	CHRDL1	Q9BU40	Chordin-like protein 1	rs186021206	7069412	17	A	G	0.006	0.432	0.046	6.9E-21	trans
468	CHRDL1	Q9BU40	Chordin-like protein 1	rs1065853	45413233	19	T	G	0.081	0.107	0.012	4.1E-18	trans
468	CHRDL1	Q9BU40	Chordin-like protein 1	rs2899319	39874314	22	C	A	0.616	0.058	0.007	1.4E-16	trans
469	CHRDL2	Q6WN34	Chordin-like protein 2	rs9810189	186641212	3	A	C	0.285	0.056	0.008	1.4E-12	trans
469	CHRDL2	Q6WN34	Chordin-like protein 2	rs6590199	126235667	11	A	G	0.158	0.085	0.010	2.4E-17	trans
469	CHRDL2	Q6WN34	Chordin-like protein 2	rs138903858	105634487	14	T	G	0.028	0.193	0.022	5.8E-18	trans
469	CHRDL2	Q6WN34	Chordin-like protein 2	rs200489612	7106378	17	A	G	0.005	0.649	0.053	2.4E-34	trans
470	CIAPIN1	Q6FI81	Anamorsin	rs1354034	56849749	3	C	T	0.604	0.072	0.008	3.1E-21	trans
470	CIAPIN1	Q6FI81	Anamorsin	rs342298	106373646	7	C	T	0.545	0.052	0.007	4.9E-12	trans
470	CIAPIN1	Q6FI81	Anamorsin	rs10733789	64948684	10	C	T	0.313	0.056	0.008	5.8E-12	trans
471	CILP	O75339	Cartilage intermediate layer protein 1	rs56278466	17875857	10	G	T	0.661	0.085	0.008	1.3E-29	trans
471	CILP	O75339	Cartilage intermediate layer protein 1	rs35887873	126219396	11	C	T	0.228	0.076	0.008	3.4E-19	trans
471	CILP	O75339	Cartilage intermediate layer protein 1	rs2585035	65496475	15	T	G	0.367	0.292	0.008	0.0E+00	cis
472	CIRBP	Q14011	Cold-inducible RNA-binding protein	rs1354034	56849749	3	C	T	0.605	0.109	0.008	2.1E-46	trans
472	CIRBP	Q14011	Cold-inducible RNA-binding protein	rs342298	106373646	7	C	T	0.545	0.060	0.008	2.2E-15	trans

472	CIRBP	Q14011	Cold-inducible RNA-binding protein	rs6993770	106581528	8	A	T	0.713	0.058	0.008	1.5E-12	trans
473	CIT	O14578	Citron Rho-interacting kinase	rs3811444	248039451	1	C	T	0.668	0.058	0.008	5.3E-13	trans
474	CKAP4	Q07065	Cytoskeleton-associated protein 4	rs61747728	179526214	1	T	C	0.038	0.135	0.019	2.8E-13	trans
474	CKAP4	Q07065	Cytoskeleton-associated protein 4	rs28929474	94844947	14	T	C	0.021	1.318	0.026	0.0E+00	trans
474	CKAP4	Q07065	Cytoskeleton-associated protein 4	rs28640218	20359267	16	G	T	0.808	0.064	0.009	1.7E-12	trans
475	CKB	P12277	Creatine kinase B-type	rs1260326	27730940	2	C	T	0.608	0.064	0.008	4.4E-17	trans
475	CKB	P12277	Creatine kinase B-type	rs4674100	217480424	2	G	A	0.707	0.072	0.009	8.5E-17	trans
475	CKB	P12277	Creatine kinase B-type	rs13108218	3443931	4	A	G	0.381	0.057	0.008	1.5E-13	trans
475	CKB	P12277	Creatine kinase B-type	rs10027182	148981406	4	A	G	0.744	0.058	0.008	1.1E-11	trans
476	CKMT1A_CKMT1B	P12532	Creatine kinase U-type, mitochondrial	rs700882	101731397	2	T	C	0.151	0.071	0.011	1.3E-11	trans
476	CKMT1A_CKMT1B	P12532	Creatine kinase U-type, mitochondrial	rs34248556	69354678	4	T	C	0.344	0.070	0.008	3.0E-18	trans
476	CKMT1A_CKMT1B	P12532	Creatine kinase U-type, mitochondrial	rs73082019	24756643	7	A	G	0.899	0.094	0.013	6.8E-14	trans
476	CKMT1A_CKMT1B	P12532	Creatine kinase U-type, mitochondrial	rs573943463	71583942	8	C	T	0.494	0.078	0.008	1.5E-24	trans
476	CKMT1A_CKMT1B	P12532	Creatine kinase U-type, mitochondrial	rs690604	43834864	15	G	T	0.919	0.291	0.014	1.4E-90	cis
476	CKMT1A_CKMT1B	P12532	Creatine kinase U-type, mitochondrial	rs601338	49206674	19	G	A	0.490	0.089	0.008	4.4E-32	trans
477	CLASP1	Q7Z460	CLIP-associating protein 1	rs371337007	32493146	6	G	A	0.354	0.082	0.009	4.2E-19	trans
478	CLC	Q05315	Galectin-10	rs1186706	61673394	2	G	T	0.509	0.050	0.007	1.0E-11	trans
478	CLC	Q05315	Galectin-10	rs10208293	102966310	2	G	A	0.720	0.065	0.008	2.2E-15	trans
478	CLC	Q05315	Galectin-10	rs4328821	128316435	3	A	G	0.893	0.150	0.012	2.2E-36	trans
478	CLC	Q05315	Galectin-10	rs11242115	131826413	5	G	C	0.611	0.066	0.008	3.7E-18	trans
478	CLC	Q05315	Galectin-10	rs448277	29901806	6	C	T	0.711	0.061	0.008	3.0E-13	trans
478	CLC	Q05315	Galectin-10	rs6467885	75469451	7	T	C	0.104	0.186	0.012	2.0E-54	trans
478	CLC	Q05315	Galectin-10	rs55646585	144999621	8	C	T	0.586	0.073	0.007	1.3E-22	trans
478	CLC	Q05315	Galectin-10	rs1888909	6197392	9	T	C	0.251	0.067	0.008	2.9E-15	trans
478	CLC	Q05315	Galectin-10	rs34210653	4535314	17	G	A	0.979	0.181	0.026	1.8E-12	trans
478	CLC	Q05315	Galectin-10	rs143121234	40226530	19	C	G	0.998	1.357	0.091	1.1E-50	cis
478	CLC	Q05315	Galectin-10	rs34436714	54327313	19	A	C	0.209	0.140	0.009	5.8E-54	trans
479	CLEC10A	Q8IUN9	C-type lectin domain family 10 member A	rs9271378	32587300	6	G	A	0.490	0.069	0.007	1.0E-23	trans
479	CLEC10A	Q8IUN9	C-type lectin domain family 10 member A	rs971481	7243033	7	C	G	0.386	0.120	0.007	1.6E-64	trans
479	CLEC10A	Q8IUN9	C-type lectin domain family 10 member A	rs13294683	82279767	9	T	C	0.102	0.203	0.011	9.1E-72	trans
479	CLEC10A	Q8IUN9	C-type lectin domain family 10 member A	rs2519093	136141870	9	T	C	0.184	0.130	0.009	2.6E-48	trans
479	CLEC10A	Q8IUN9	C-type lectin domain family 10 member A	rs12771902	72002499	10	G	A	0.675	0.056	0.007	2.2E-14	trans
479	CLEC10A	Q8IUN9	C-type lectin domain family 10 member A	rs56156922	56987369	16	C	T	0.325	0.061	0.007	5.7E-17	trans
479	CLEC10A	Q8IUN9	C-type lectin domain family 10 member A	rs90951	6981397	17	G	A	0.333	0.544	0.008	0.0E+00	cis
479	CLEC10A	Q8IUN9	C-type lectin domain family 10 member A	rs12938273	57901652	17	A	G	0.554	0.093	0.007	2.0E-35	trans
480	CLEC11A	Q9Y240	C-type lectin domain family 11 member A	rs13063578	47087837	3	T	A	0.602	0.074	0.008	2.9E-22	trans
480	CLEC11A	Q9Y240	C-type lectin domain family 11 member A	rs4407843	130609368	8	A	C	0.432	0.061	0.008	8.8E-16	trans
480	CLEC11A	Q9Y240	C-type lectin domain family 11 member A	rs2282683	79117751	9	A	G	0.790	0.074	0.009	2.1E-16	trans
480	CLEC11A	Q9Y240	C-type lectin domain family 11 member A	rs12050709	50359224	15	T	C	0.756	0.059	0.009	7.5E-12	trans
480	CLEC11A	Q9Y240	C-type lectin domain family 11 member A	rs2179768	540500	16	G	A	0.487	0.065	0.007	2.2E-18	trans
480	CLEC11A	Q9Y240	C-type lectin domain family 11 member A	rs141369989	51226507	19	A	G	0.029	0.820	0.023	2.9E-286	cis
480	CLEC11A	Q9Y240	C-type lectin domain family 11 member A	rs13036464	17665867	20	C	G	0.407	0.057	0.008	3.8E-14	trans
481	CLEC12A	Q5QGZ9	C-type lectin domain family 12 member A	rs11260012	7759569	19	T	C	0.513	0.081	0.007	1.8E-28	trans
482	CLEC14A	Q86T13	C-type lectin domain family 14 member A	rs6664906	31354813	1	T	C	0.536	0.102	0.007	5.5E-45	trans
482	CLEC14A	Q86T13	C-type lectin domain family 14 member A	rs61747728	179526214	1	T	C	0.038	0.169	0.019	8.9E-20	trans
482	CLEC14A	Q86T13	C-type lectin domain family 14 member A	rs75166367	162964301	2	G	A	0.940	0.113	0.015	4.1E-14	trans
482	CLEC14A	Q86T13	C-type lectin domain family 14 member A	rs13107325	103188709	4	T	C	0.075	0.161	0.014	5.2E-32	trans
482	CLEC14A	Q86T13	C-type lectin domain family 14 member A	rs507666	136149399	9	G	A	0.815	0.343	0.009	1.2E-290	trans
482	CLEC14A	Q86T13	C-type lectin domain family 14 member A	rs10151239	38972370	14	A	C	0.404	0.084	0.007	1.1E-30	cis
482	CLEC14A	Q86T13	C-type lectin domain family 14 member A	rs186021206	7069412	17	A	G	0.006	0.432	0.049	9.6E-19	trans
482	CLEC14A	Q86T13	C-type lectin domain family 14 member A	rs33950747	36339247	19	T	C	0.075	0.152	0.014	3.2E-29	trans
483	CLEC1A	Q8NC01	C-type lectin domain family 1 member A	rs7669607	9997801	4	C	T	0.791	0.059	0.009	3.6E-12	trans
483	CLEC1A	Q8NC01	C-type lectin domain family 1 member A	rs10982156	117088064	9	A	T	0.070	0.227	0.015	5.8E-52	trans
483	CLEC1A	Q8NC01	C-type lectin domain family 1 member A	rs10901252	136128000	9	C	G	0.061	0.780	0.015	0.0E+00	trans
483	CLEC1A	Q8NC01	C-type lectin domain family 1 member A	rs77924615	20392332	16	G	A	0.803	0.066	0.009	5.9E-14	trans
483	CLEC1A	Q8NC01	C-type lectin domain family 1 member A	rs186021206	7069412	17	A	G	0.006	0.443	0.048	3.3E-20	trans
484	CLEC1B	Q9P126	C-type lectin domain family 1 member B	rs3804749	122833003	3	C	T	0.407	0.081	0.008	6.0E-26	trans
484	CLEC1B	Q9P126	C-type lectin domain family 1 member B	rs11242109	131677047	5	G	T	0.519	0.065	0.008	7.6E-18	trans
484	CLEC1B	Q9P126	C-type lectin domain family 1 member B	rs6961069	80218961	7	T	C	0.402	0.057	0.008	6.4E-14	trans
484	CLEC1B	Q9P126	C-type lectin domain family 1 member B	rs6993770	106581528	8	A	T	0.714	0.131	0.008	4.4E-56	trans
484	CLEC1B	Q9P126	C-type lectin domain family 1 member B	rs7896518	65104500	10	G	A	0.424	0.092	0.008	7.5E-33	trans
484	CLEC1B	Q9P126	C-type lectin domain family 1 member B	rs1654425	55538980	19	C	T	0.834	0.112	0.010	1.6E-28	trans
485	CLEC3B	P05452	Tetranectin	rs13065490	45057108	3	T	A	0.269	0.238	0.008	5.7E-177	cis
485	CLEC3B	P05452	Tetranectin	rs13107325	103188709	4	T	C	0.075	0.122	0.014	2.6E-18	trans
485	CLEC3B	P05452	Tetranectin	rs77542162	67081278	17	G	A	0.023	0.209	0.025	1.5E-17	trans
486	CLEC4A	Q9UMR7	C-type lectin domain family 4 member A	rs200688856	161599779	1	C	G	0.342	0.129	0.007	3.7E-69	trans
486	CLEC4A	Q9UMR7	C-type lectin domain family 4 member A	rs791357	173205225	5	T	C	0.280	0.058	0.008	1.7E-13	trans
486	CLEC4A	Q9UMR7	C-type lectin domain family 4 member A	rs1634791	31276777	6	G	A	0.542	0.072	0.007	6.0E-24	trans
486	CLEC4A	Q9UMR7	C-type lectin domain family 4 member A	rs11775560	61660163	8	G	A	0.186	0.069	0.009	9.3E-15	trans
486	CLEC4A	Q9UMR7	C-type lectin domain family 4 member A	rs7074896	99071282	10	T	C	0.689	0.054	0.008	1.1E-12	trans
486	CLEC4A	Q9UMR7	C-type lectin domain family 4 member A	rs11603123	126305495	11	A	G	0.034	0.467	0.019	9.9E-128	trans
486	CLEC4A	Q9UMR7	C-type lectin domain family 4 member A	rs174418	58687603	15	T	C	0.402	0.088	0.007	1.2E-34	trans
486	CLEC4A	Q9UMR7	C-type lectin domain family 4 member A	rs247617	56990716	16	A	C	0.324	0.066	0.007	1.0E-18	trans
486	CLEC4A	Q9UMR7	C-type lectin domain family 4 member A	rs11654208	38196493	17	G	A	0.633	0.049	0.007	1.2E-11	trans
486	CLEC4A	Q9UMR7	C-type lectin domain family 4 member A	rs77960347	47109955	18	G	A	0.013	0.215	0.031	2.2E-12	trans
486	CLEC4A	Q9UMR7	C-type lectin domain family 4 member A	rs4760	44153100	19	A	G	0.844	0.108	0.010	2.4E-29	trans
486	CLEC4A	Q9UMR7	C-type lectin domain family 4 member A	rs184339396	49043608	X	C	G	0.033	0.138	0.017	2.1E-16	trans
487	CLEC4C	Q8WTT0	C-type lectin domain family 4 member C	rs174847	65605194	2	T	C	0.455	0.067	0.006	2.1E-29	trans
487	CLEC4C	Q8WTT0	C-type lectin domain family 4 member C	rs11899659	99363790	2	G	A	0.407	0.045	0.006	7.7E-14	trans
487	CLEC4C	Q8WTT0	C-type lectin domain family 4 member C	rs1439287	111871897	2	A	G	0.484	0.098	0.006	2.8E-60	trans
487	CLEC4C	Q8WTT0	C-type lectin domain family 4 member C	rs7595770	145460864	2	G	T	0.922	0.104	0.011	3.2E-21	trans
487	CLEC4C	Q8WTT0	C-type lectin domain family 4 member C	rs7658518	84160279	4	G	A	0.717	0.079	0.007	1.1E-32	trans
487	CLEC4C	Q8WTT0	C-type lectin domain family 4 member C	rs6818511	105949268	4	C	T	0.659	0.117	0.006	2.3E-77	trans
487	CLEC4C	Q8WTT0	C-type lectin domain family 4 member C	rs17329170	9547242	5	G	A	0.902	0.071	0.010	8.7E-13	trans
487	CLEC4C	Q8WTT0	C-type lectin domain family 4 member C	rs4713570	32626040	6	T	C	0.266	0.065	0.007	4.1E-22	trans
487	CLEC4C	Q8WTT0	C-type lectin domain family 4 member C	rs2347784	6524843	7	C	G	0.728	0.072	0.007	5.8E-27	trans
487	CLEC4C	Q8WTT0	C-type lectin domain family 4 member C	rs876038	50308527	7	C	T	0.684	0.206	0.006	2.3E-227	trans
487	CLEC4C	Q8WTT0	C-type lectin domain family 4 member C	rs10107630	130603635	8	T	C	0.574	0.062	0.006	8.1E-25	trans
487	CLEC4C	Q8WTT0	C-type lectin domain family 4 member C	rs10971417	33121480	9	C	T	0.747	0.056	0.007	1.3E-16	trans
487	CLEC4C	Q8WTT0	C-type lectin domain family 4 member C	rs10441775	94481908	9	G	A	0.692	0.046	0.006	1.2E-12	trans
487	CLEC4C	Q8WTT0	C-type lectin domain family 4 member C	rs7873862	116113396	9	T	C	0.186	0.083	0.008	8.4E-28	trans
487	CLEC4C	Q8WTT0	C-type lectin domain family 4 member C	rs11789604	139599129	9	G	T	0.453	0.040	0.006	9.3E-12	trans
487	CLEC4C	Q8WTT0	C-type lectin domain family 4 member C	rs10838702	47410888	11	T	G	0.386	0.058	0.006	2.1E-21	trans
487	CLEC4C	Q8WTT0	C-type lectin domain family 4 member C	rs7129402	121211756	11	C	A	0.280	0.138	0.007	1.7E-94	trans
487	CLEC4C	Q8WTT0	C-type lectin domain family 4 member C	rs76428106	28604007	13	C	T	0.013	0.507	0.027	1.1E-77	trans
487	CLEC4C	Q8WTT0	C-type lectin domain family 4 member C	rs62045817	89045233	16	C	T	0.850	0.083	0.008	7.8E-23	trans
487	CLEC4C	Q8WTT0	C-type lectin domain family 4 member C	rs62057151	43903842	17	C	T	0.767	0.054	0.007	7.2E-15	trans
487	CLEC4C	Q8WTT0	C-type lectin domain family 4 member C	rs16947425	62144344	17	C	A	0.841	0.068	0.008	2.8E-17	trans
488	CLEC4D	Q8WXI8	C-type lectin domain family 4 member D	rs2070901	161185058	1	G	T	0.730	0.205	0.008	1.1E-151	trans
488	CLEC4D	Q8WXI8	C-type lectin domain family 4 member D	rs6429432	236107241	1	A	C	0.106	0.092	0.011	1.4E-16	trans
488	CLEC4D	Q8WXI8	C-type lectin domain family 4 member D	rs149110519	144385777	6	T	C	0.036	0.140	0.018	2.4E-14	trans
488	CLEC4D	Q8WXI8	C-type lectin domain family 4 member D	rs445	92408370	7	C	T	0.905	0.083	0.012	1.0E-12	trans
488	CLEC4D	Q8WXI8	C-type lectin domain family 4 member D	rs2511241	72945341	11	T	C	0.929	0.188	0.013	1.1E-44	trans
488	CLEC4D	Q8WXI8	C-type lectin domain family 4 member D	rs3826331	38150492	17	T	C	0.402	0.119	0.007	6.0E-65	trans
488	CLEC4D	Q8WXI8	C-type lectin domain family 4 member D	rs872629	52130638	19	C	A	0.886	0.152	0.011	3.0E-45	trans
489	CLEC4G	Q6UXB4	C-type lectin domain family 4 member G	rs10935473	98416900	3	G	T	0.560	0.107	0.007	1.2E-48	trans
489	CLEC4G	Q6UXB4	C-type lectin domain family 4 member G	rs7865362	33117965	9	T	C	0.363	0.069	0.008	9.2E-19	trans
489	CLEC4G	Q6UXB4	C-type lectin domain family 4 member G	rs56278466	17875857	10	T	G	0.338	0.087	0.008	8.1E-30	trans
489	CLEC4G	Q6UXB4	C-type lectin domain family 4 member G	rs10830278	89216425	11	A	T	0.799	0.086	0.009	3.7E-21	trans
489	CLEC4G	Q6UXB4	C-type lectin domain family 4 member G	rs12709103	88712782	16	A	G	0.324	0.055	0.008	8.6E-13	trans
489	CLEC4G	Q6UXB4	C-type lectin domain family 4 member G	rs17701598	66320445	17	G	A	0.749	0.061	0.008	2.4E-13	trans
489	CLEC4G	Q6UXB4	C-type lectin domain family 4 member G	rs76560987	7799452	19	T	C	0.071	0.374	0.014	2.3E-151	cis
490	CLEC4M	Q9H2X3	C-type lectin domain family 4 member M	rs7528419	109817192	1	A	G	0.778	0.096	0.007	2.8E-43	trans
490	CLEC4M	Q9H2X3	C-type lectin domain family 4 member M	rs10769256	47378396	11	T	C	0.400	0.049	0.006	8.1E-17	trans
490	CLEC4M	Q9H2X3	C-type lectin domain family 4 member M	rs56156922	56987369	16	C	T	0.325	0.046	0.006	1.3E-13	trans
490	CLEC4M	Q9H2X3	C-type lectin domain family 4 member M	rs77542162	67081278	17	G	A	0.023	0.132	0.019	8.4E-12	trans
490	CLEC4M	Q9H2X3	C-type lectin domain family 4 member M	rs4804774	7772173	19	G	C	0.763	0.364	0.008	0.0E+00	cis
491	CLEC5A	Q9NY25	C-type lectin domain family 5 member A	rs10801568	196791881	1	T	G	0.752	0.071	0.009	1.3E-16	trans
491	CLEC5A	Q9NY25	C-type lectin domain family 5 member A	rs13107325	103188709	4	T	C	0.075	0.096	0.013	1.3E-12	trans
491	CLEC5A	Q9NY25	C-type lectin domain family 5 member A	rs9273113	32612655	6	A	G	0.669	0.074	0.009	7.2E-18	trans
491	CLEC5A	Q9NY25	C-type lectin domain family 5 member A	rs11769630	50257703	7	A	T	0.073	0.131	0.014	8.0E-21	trans
491	CLEC5A	Q9NY25	C-type lectin domain family 5 member A	rs6962383	141661450	7	C	A	0.297	0.286	0.008	1.7E-285	cis
491	CLEC5A	Q9NY25	C-type lectin domain family 5 member A	rs10838702	47410888	11	G	T	0.615	0.059	0.007	3.6E-16	trans
491	CLEC5A	Q9NY25	C-type lectin domain family 5 member A	rs588136	58730498	15	C	T	0.216	0.071	0.009	1.9E-16	trans
491	CLEC5A	Q9NY25	C-type lectin domain family 5 member A	rs186021206	7069412	17	A	G	0.006	0.442	0.048	7.9E-20	trans
491	CLEC5A	Q9NY25	C-type lectin domain family 5 member A	rs3826331	38150492	17	T	C	0.402	0.071	0.007	3.0E-23	trans
491	CLEC5A	Q9NY25	C-type lectin domain family 5 member A	rs143333049	19800289	19	G	A	0.040	0.132	0.019	1.0E-12	trans
491	CLEC5A	Q9NY25	C-type lectin domain family 5 member A	rs2254448	1551097	20	G	C	0.778	0.181	0.009	2.0E-99	trans
492	CLEC6A	Q6EIG7	C-type lectin domain family 6 member A	rs188468174	25291697	1	T	C	0.014	0.215	0.030	5.4E-13	trans
492	CLEC6A	Q6EIG7	C-type lectin domain family 6 member A	rs2070901	161185058	1	G	T	0.730	0.120	0.008	1.5E-52	trans
492	CLEC6A	Q6EIG7	C-type lectin domain family 6 member A	rs10186921	43644556	2	C	T	0.430	0.053	0.007	3.9E-14	trans
492	CLEC6A	Q6EIG7	C-type lectin domain family 6 member A	rs9880192	128297569	3	G	C	0.586	0.051	0.007	5.6E-13	trans
492	CLEC6A	Q6EIG7	C-type lectin domain family 6 member A	rs2158799	28277107	7	G	C	0.610	0.050	0.007	4.9E-12	trans
492	CLEC6A	Q6EIG7	C-type lectin domain family 6 member A	rs13277237	130604563	8	G	A	0.432	0.049	0.007	2.8E-12	trans
492	CLEC6A	Q6EIG7	C-type lectin domain family 6 member A	rs76428106	28604007	13	C	T	0.013	0.269	0.032	3.3E-17	trans
492	CLEC6A	Q6EIG7	C-type lectin domain family 6 member A	rs2239630	23589349	14	A	G	0.438	0.049	0.007	3.1E-12	trans
492	CLEC6A	Q6EIG7	C-type lectin domain family 6 member A	rs1147128	51207983	15	G	A	0.458	0.085	0.007	5.9E-34	trans
492	CLEC6A	Q6EIG7	C-type lectin domain family 6 member A	rs8081692	38154595	17	G	A	0.393	0.055	0.007	1.7E-14	trans
492	CLEC6A	Q6EIG7	C-type lectin domain family 6 member A	rs872629	52130638	19	C	A	0.887	0.132	0.011	4.4E-33	trans
493	CLEC7A	Q9BXN2	C-type lectin domain family 7 member A	rs2647011	32661915	6	C	A	0.461	0.041	0.006	6.9E-12	trans
494	CLGN	O14967	Calmegin	rs6852397	141348748	4	T	G	0.094	1.175	0.013	0.0E+00	cis
495	CLIC5	Q9NZA1	Chloride intracellular channel protein 5	rs2022423	46046859	6	A	G	0.641	0.121	0.008	8.0E-51	cis
496	CLIP2	Q9UDT6	CAP-Gly domain-containing linker protein 2	rs117471007	73696488	7	T	C	0.063	0.250	0.015	7.1E-59	cis

496	CLIP2	Q9UDT6	CAP-Gly domain-containing linker protein 2	rs6993770	106581528	8	A	T	0.713	0.067	0.008	2.2E-16	trans
497	CLMP	Q9H6B4	CXADR-like membrane protein	rs1260326	27730940	2	C	T	0.608	0.050	0.007	2.4E-13	trans
497	CLMP	Q9H6B4	CXADR-like membrane protein	rs73017877	123063062	11	G	C	0.552	0.336	0.007	0.0E+00	cis
497	CLMP	Q9H6B4	CXADR-like membrane protein	rs77924615	20392332	16	G	A	0.803	0.075	0.009	1.6E-18	trans
498	CLNS1A	P54105	Methylosome subunit pICln	rs6695321	196675861	1	G	A	0.381	0.060	0.008	1.0E-14	trans
499	CLPS	P04118	Colipase	rs2766588	35753227	6	C	T	0.607	0.550	0.008	0.0E+00	cis
499	CLPS	P04118	Colipase	rs2653414	17726069	8	A	C	0.013	0.256	0.029	3.3E-18	trans
499	CLPS	P04118	Colipase	rs7015116	129539982	8	T	G	0.857	0.072	0.010	3.0E-14	trans
499	CLPS	P04118	Colipase	rs7068821	90051035	10	G	T	0.741	0.053	0.008	1.2E-12	trans
499	CLPS	P04118	Colipase	rs2286779	118394551	10	G	C	0.481	0.048	0.007	1.9E-13	trans
499	CLPS	P04118	Colipase	rs9652674	75255473	16	A	G	0.691	0.089	0.007	2.8E-35	trans
499	CLPS	P04118	Colipase	rs35866622	49218060	19	T	C	0.500	0.051	0.007	2.9E-14	trans
500	CLSPN	Q9HAW4	Claspin	rs1008982	131445731	10	T	C	0.627	0.104	0.008	6.2E-38	trans
501	CLSTN2	Q9H4D0	Calsyntenin-2	rs41265459	140167490	3	A	T	0.007	2.283	0.046	0.0E+00	cis
501	CLSTN2	Q9H4D0	Calsyntenin-2	rs6984305	9178268	8	A	T	0.115	0.103	0.011	1.5E-21	trans
501	CLSTN2	Q9H4D0	Calsyntenin-2	rs77542162	67081278	17	G	A	0.023	0.202	0.023	1.2E-18	trans
501	CLSTN2	Q9H4D0	Calsyntenin-2	rs329721	53600422	19	C	T	0.665	0.086	0.007	7.2E-31	trans
502	CLSTN3	Q9BQT9	Calsyntenin-3	rs6984305	9178268	8	A	T	0.115	0.108	0.011	1.1E-22	trans
502	CLSTN3	Q9BQT9	Calsyntenin-3	rs2519093	136141870	9	C	T	0.816	0.237	0.009	7.4E-149	trans
502	CLSTN3	Q9BQT9	Calsyntenin-3	rs2035675	6463088	11	G	A	0.245	0.059	0.008	4.0E-13	trans
502	CLSTN3	Q9BQT9	Calsyntenin-3	rs28929474	94844947	14	T	C	0.021	0.198	0.025	6.3E-16	trans
502	CLSTN3	Q9BQT9	Calsyntenin-3	rs11075921	72132129	16	T	C	0.160	0.065	0.010	8.5E-12	trans
502	CLSTN3	Q9BQT9	Calsyntenin-3	rs77542162	67081278	17	G	A	0.023	0.254	0.023	1.7E-27	trans
503	CLTA	P09496	Clathrin light chain A	rs3811444	248039451	1	C	T	0.667	0.118	0.008	1.1E-47	trans
504	CLU	P10909	Clusterin	rs1260326	27730940	2	T	C	0.392	0.054	0.008	1.0E-12	trans
504	CLU	P10909	Clusterin	rs553014	27566514	8	T	C	0.513	0.094	0.008	5.2E-36	cis
504	CLU	P10909	Clusterin	rs7247412	52313868	19	T	C	0.733	0.063	0.008	4.8E-14	trans
505	CLUL1	Q15846	Clusterin-like protein 1	rs34881159	161590503	1	A	T	0.491	0.042	0.006	1.2E-11	trans
505	CLUL1	Q15846	Clusterin-like protein 1	rs17482753	19832646	8	T	G	0.101	0.079	0.010	1.8E-15	trans
505	CLUL1	Q15846	Clusterin-like protein 1	rs174553	61575158	11	A	G	0.656	0.049	0.006	3.7E-15	trans
505	CLUL1	Q15846	Clusterin-like protein 1	rs1601935	58671765	15	G	T	0.344	0.051	0.006	9.3E-16	trans
505	CLUL1	Q15846	Clusterin-like protein 1	rs12149545	56993161	16	A	G	0.316	0.074	0.006	3.2E-30	trans
505	CLUL1	Q15846	Clusterin-like protein 1	rs186021206	7069412	17	A	G	0.006	0.347	0.041	2.4E-17	trans
505	CLUL1	Q15846	Clusterin-like protein 1	rs12957470	591690	18	C	G	0.286	0.614	0.008	0.0E+00	cis
505	CLUL1	Q15846	Clusterin-like protein 1	rs4465830	44585420	20	A	G	0.813	0.118	0.008	1.4E-53	trans
506	CMC1	Q7Z7K0	COX assembly mitochondrial protein homolog	rs370517451	28283101	3	G	A	0.985	0.314	0.034	9.6E-21	cis
506	CMC1	Q7Z7K0	COX assembly mitochondrial protein homolog	rs1354034	56849749	3	C	T	0.604	0.059	0.008	6.9E-15	trans
507	CMIP	Q8IY22	C-Maf-inducing protein	rs342298	106373646	7	C	T	0.545	0.056	0.008	9.4E-14	trans
508	CNDP1	Q96KN2	Beta-Ala-His dipeptidase	rs645040	135926622	3	T	G	0.772	0.113	0.008	1.0E-40	trans
508	CNDP1	Q96KN2	Beta-Ala-His dipeptidase	rs1030431	59311697	8	A	G	0.331	0.056	0.008	1.2E-13	trans
508	CNDP1	Q96KN2	Beta-Ala-His dipeptidase	rs28601761	126500031	8	C	G	0.581	0.049	0.007	9.9E-12	trans
508	CNDP1	Q96KN2	Beta-Ala-His dipeptidase	rs139974673	44027885	15	T	C	0.976	0.214	0.023	8.7E-21	trans
508	CNDP1	Q96KN2	Beta-Ala-His dipeptidase	rs17817077	72209543	18	A	G	0.393	0.294	0.008	0.0E+00	cis
508	CNDP1	Q96KN2	Beta-Ala-His dipeptidase	rs8102710	23870110	19	G	T	0.755	0.364	0.008	0.0E+00	trans
508	CNDP1	Q96KN2	Beta-Ala-His dipeptidase	rs12609475	27770761	19	C	A	0.830	0.244	0.010	2.4E-134	trans
508	CNDP1	Q96KN2	Beta-Ala-His dipeptidase	rs2294915	44340904	22	C	T	0.769	0.060	0.008	1.1E-12	trans
509	CNP	P09543	2',3'-cyclic-nucleotide 3'-phosphodiesterase	rs1354034	56849749	3	C	T	0.605	0.061	0.008	9.5E-16	trans
509	CNP	P09543	2',3'-cyclic-nucleotide 3'-phosphodiesterase	rs342298	106373646	7	C	T	0.545	0.065	0.008	9.3E-18	trans
510	CNPY2	Q9Y2B0	Protein canopy homolog 2	rs28929474	94844947	14	T	C	0.021	0.475	0.027	2.4E-69	trans
511	CNPY4	Q8N129	Protein canopy homolog 4	rs144305120	99745585	7	T	G	0.929	0.335	0.015	3.2E-113	cis
511	CNPY4	Q8N129	Protein canopy homolog 4	rs342298	106373646	7	C	T	0.546	0.053	0.008	3.0E-12	trans
512	CNTN1	Q12860	Contactin-1	rs1814170	138794149	7	A	T	0.900	0.152	0.011	3.4E-43	trans
512	CNTN1	Q12860	Contactin-1	rs56278466	17875857	10	G	T	0.661	0.059	0.007	1.2E-17	trans
512	CNTN1	Q12860	Contactin-1	rs2071916	426476	16	T	C	0.414	0.301	0.007	0.0E+00	trans
512	CNTN1	Q12860	Contactin-1	rs186021206	7069412	17	A	G	0.006	0.481	0.045	2.4E-26	trans
513	CNTN2	Q02246	Contactin-2	rs2071533	205012198	1	T	G	0.134	1.175	0.011	0.0E+00	cis
513	CNTN2	Q02246	Contactin-2	rs912057	6736931	6	A	G	0.390	0.074	0.006	1.7E-32	trans
513	CNTN2	Q02246	Contactin-2	rs56278466	17875857	10	G	T	0.661	0.105	0.006	3.2E-60	trans
513	CNTN2	Q02246	Contactin-2	rs74612335	126238633	11	T	C	0.867	0.077	0.009	5.8E-18	trans
513	CNTN2	Q02246	Contactin-2	rs186021206	7069412	17	A	G	0.006	0.462	0.042	1.5E-28	trans
514	CNTN3	Q9P232	Contactin-3	rs114165349	27021913	1	C	G	0.023	0.261	0.022	1.5E-31	trans
514	CNTN3	Q9P232	Contactin-3	rs3213563	172412995	1	C	T	0.559	0.053	0.007	3.0E-15	trans
514	CNTN3	Q9P232	Contactin-3	rs1260326	27730940	2	T	C	0.392	0.086	0.007	5.7E-36	trans
514	CNTN3	Q9P232	Contactin-3	rs4677414	74607020	3	A	G	0.071	0.512	0.014	4.9E-306	cis
514	CNTN3	Q9P232	Contactin-3	rs1154988	135925191	3	T	A	0.227	0.084	0.008	8.6E-24	trans
514	CNTN3	Q9P232	Contactin-3	rs33951980	73029437	7	C	T	0.871	0.117	0.010	9.8E-32	trans
514	CNTN3	Q9P232	Contactin-3	rs4921914	18272438	8	T	C	0.779	0.071	0.008	1.2E-18	trans
514	CNTN3	Q9P232	Contactin-3	rs10822145	64934548	10	C	T	0.528	0.057	0.007	2.3E-17	trans
514	CNTN3	Q9P232	Contactin-3	rs2030291	16251251	11	T	A	0.387	0.048	0.007	4.5E-12	trans
514	CNTN3	Q9P232	Contactin-3	rs147233090	44028047	15	T	C	0.024	0.305	0.022	7.9E-43	trans
514	CNTN3	Q9P232	Contactin-3	rs11646097	425605	16	T	C	0.408	0.074	0.007	1.3E-27	trans
514	CNTN3	Q9P232	Contactin-3	rs11647695	80779331	16	C	A	0.554	0.058	0.007	5.7E-18	trans
514	CNTN3	Q9P232	Contactin-3	rs34088055	88535670	16	T	C	0.316	0.053	0.007	6.2E-13	trans
514	CNTN3	Q9P232	Contactin-3	rs1292066	57932314	17	A	T	0.215	0.061	0.008	5.6E-14	trans
514	CNTN3	Q9P232	Contactin-3	rs77542162	67081278	17	G	A	0.023	0.210	0.022	1.0E-20	trans
514	CNTN3	Q9P232	Contactin-3	rs55714572	58502950	19	T	C	0.147	0.065	0.010	1.2E-11	trans
515	CNTN4	Q8IWV2	Contactin-4	rs163352	3098041	3	G	C	0.185	0.316	0.010	1.3E-241	cis
515	CNTN4	Q8IWV2	Contactin-4	rs28366036	186647763	3	A	C	0.656	0.055	0.008	8.4E-13	trans
515	CNTN4	Q8IWV2	Contactin-4	rs11730129	16032948	4	T	C	0.217	0.079	0.008	2.1E-21	trans
515	CNTN4	Q8IWV2	Contactin-4	rs9270487	32559076	6	A	G	0.664	0.053	0.008	1.7E-12	trans
515	CNTN4	Q8IWV2	Contactin-4	rs9987289	9183358	8	A	G	0.092	0.091	0.012	2.0E-14	trans
515	CNTN4	Q8IWV2	Contactin-4	rs174583	61609750	11	T	C	0.354	0.053	0.007	2.2E-13	trans
515	CNTN4	Q8IWV2	Contactin-4	rs4055121	126232337	11	C	T	0.865	0.185	0.010	2.1E-73	trans
515	CNTN4	Q8IWV2	Contactin-4	rs186021206	7069412	17	A	G	0.006	0.888	0.047	1.3E-78	trans
515	CNTN4	Q8IWV2	Contactin-4	rs2899319	39874314	22	C	A	0.617	0.049	0.007	1.2E-11	trans
516	CNTN5	O94779	Contactin-5	rs780094	27741237	2	C	T	0.620	0.044	0.006	2.2E-12	trans
516	CNTN5	O94779	Contactin-5	rs13133886	16035795	4	A	C	0.218	0.126	0.007	3.3E-65	trans
516	CNTN5	O94779	Contactin-5	rs10993170	97108540	9	C	G	0.044	0.229	0.015	1.5E-50	trans
516	CNTN5	O94779	Contactin-5	rs72767003	127913157	9	C	T	0.854	0.065	0.009	9.2E-14	trans
516	CNTN5	O94779	Contactin-5	rs898776	99089041	11	G	A	0.078	0.792	0.013	0.0E+00	cis
516	CNTN5	O94779	Contactin-5	rs186021206	7069412	17	A	G	0.006	0.421	0.042	1.3E-23	trans
516	CNTN5	O94779	Contactin-5	rs134077	28063227	22	G	A	0.562	0.071	0.006	9.4E-30	trans
517	CNTNAP2	Q9UHC6	Contactin-associated protein-like 2	rs7810370	145366002	7	A	G	0.286	0.554	0.008	0.0E+00	cis
517	CNTNAP2	Q9UHC6	Contactin-associated protein-like 2	rs56278466	17875857	10	G	T	0.662	0.122	0.007	1.8E-65	trans
517	CNTNAP2	Q9UHC6	Contactin-associated protein-like 2	rs1033415	39888774	22	G	A	0.646	0.059	0.007	1.3E-16	trans
518	CNTNAP4	Q9C0A0	Contactin-associated protein-like 4	rs71534539	32512754	6	G	A	0.535	0.107	0.009	6.7E-36	trans
519	COCH	O43405	Cochlin	rs13412535	224874874	2	G	A	0.769	0.237	0.008	1.1E-178	trans
519	COCH	O43405	Cochlin	rs7618405	18250509	3	C	A	0.795	0.077	0.009	5.3E-19	trans
519	COCH	O43405	Cochlin	rs1354034	56849749	3	T	C	0.396	0.056	0.007	2.2E-15	trans
519	COCH	O43405	Cochlin	rs139141690	101499930	7	A	G	0.005	0.654	0.051	3.3E-37	trans
519	COCH	O43405	Cochlin	rs342292	106370644	7	G	C	0.457	0.053	0.007	2.1E-14	trans
519	COCH	O43405	Cochlin	rs6993770	106581528	8	A	T	0.713	0.193	0.008	2.2E-137	trans
519	COCH	O43405	Cochlin	rs663939	128597046	11	A	G	0.354	0.051	0.007	2.0E-12	trans
519	COCH	O43405	Cochlin	rs28400019	31343494	14	A	G	0.115	0.510	0.012	0.0E+00	cis
519	COCH	O43405	Cochlin	rs1552948	101993989	15	C	T	0.705	0.115	0.008	7.9E-51	trans
519	COCH	O43405	Cochlin	rs892090	55539072	19	G	T	0.834	0.103	0.009	5.7E-28	trans
520	COL15A1	P39059	Collagen alpha-1(XV) chain	rs1398018	77372923	4	C	T	0.442	0.056	0.007	1.1E-14	trans
520	COL15A1	P39059	Collagen alpha-1(XV) chain	rs10819566	101763477	9	C	T	0.365	0.189	0.008	1.2E-135	cis
520	COL15A1	P39059	Collagen alpha-1(XV) chain	rs77924615	20392332	16	G	A	0.803	0.073	0.009	1.6E-15	trans
521	COL18A1	P39060	Collagen alpha-1(XVIII) chain	rs1260326	27730940	2	T	C	0.391	0.065	0.007	3.0E-19	trans
521	COL18A1	P39060	Collagen alpha-1(XVIII) chain	rs116539064	77018400	17	T	C	0.097	0.115	0.012	3.3E-21	trans
521	COL18A1	P39060	Collagen alpha-1(XVIII) chain	rs9976834	46897214	21	C	T	0.816	0.197	0.009	5.9E-98	cis
522	COL1A1	P02452	Collagen alpha-1(I) chain	rs1386625	38361120	4	A	G	0.098	0.128	0.013	1.7E-24	trans
522	COL1A1	P02452	Collagen alpha-1(I) chain	rs2023472	30075864	6	G	A	0.588	0.050	0.007	1.2E-11	trans
522	COL1A1	P02452	Collagen alpha-1(I) chain	rs10955915	119963547	8	G	A	0.540	0.098	0.007	7.0E-40	trans
522	COL1A1	P02452	Collagen alpha-1(I) chain	rs58973023	42959133	13	T	A	0.487	0.131	0.007	1.6E-70	trans
522	COL1A1	P02452	Collagen alpha-1(I) chain	rs12879725	75428248	14	C	T	0.505	0.056	0.007	3.3E-14	trans
522	COL1A1	P02452	Collagen alpha-1(I) chain	rs117068593	93118229	14	C	T	0.813	0.074	0.009	3.5E-15	trans
522	COL1A1	P02452	Collagen alpha-1(I) chain	rs147266928	48263206	17	C	T	0.001	1.425	0.111	8.3E-38	cis
522	COL1A1	P02452	Collagen alpha-1(I) chain	rs146385050	60637258	17	A	C	0.183	0.133	0.010	8.3E-44	trans
522	COL1A1	P02452	Collagen alpha-1(I) chain	rs2957137	60066888	18	A	T	0.360	0.059	0.008	1.8E-14	trans
523	COL24A1	Q17RW2	Collagen alpha-1(XXIV) chain	rs11161756	86616339	1	T	C	0.908	0.257	0.013	8.8E-86	cis
523	COL24A1	Q17RW2	Collagen alpha-1(XXIV) chain	rs62059251	75187465	16	A	G	0.309	0.076	0.008	2.6E-20	trans
524	COL28A1	Q2UY09	Collagen alpha-1(XXVIII) chain	rs6775517	58358967	3	T	G	0.367	0.055	0.007	2.5E-16	trans
524	COL28A1	Q2UY09	Collagen alpha-1(XXVIII) chain	rs7810134	7542265	7	A	G	0.232	0.905	0.009	0.0E+00	cis
524	COL28A1	Q2UY09	Collagen alpha-1(XXVIII) chain	rs804289	11617916	8	A	C	0.672	0.047	0.007	8.3E-12	trans
524	COL28A1	Q2UY09	Collagen alpha-1(XXVIII) chain	rs62509471	105968927	8	A	G	0.823	0.081	0.009	6.2E-20	trans
524	COL28A1	Q2UY09	Collagen alpha-1(XXVIII) chain	rs56278466	17875857	10	G	T	0.661	0.092	0.007	1.5E-41	trans
524	COL28A1	Q2UY09	Collagen alpha-1(XXVIII) chain	rs35772006	75180124	16	A	G	0.413	0.066	0.007	1.0E-23	trans
524	COL28A1	Q2UY09	Collagen alpha-1(XXVIII) chain	rs55682587	2917689	19	T	C	0.022	0.164	0.022	6.5E-14	trans
525	COL2A1	P02458	Collagen alpha-1(II) chain	rs154507	122200907	5	G	C	0.444	0.066	0.006	4.3E-25	trans
525	COL2A1	P02458	Collagen alpha-1(II) chain	rs10075801	131677642	5	A	G	0.512	0.056	0.006	2.4E-18	trans
525	COL2A1	P02458	Collagen alpha-1(II) chain	rs3823764	27187753	7	T	C	0.842	0.070	0.009	7.1E-16	trans
525	COL2A1	P02458	Collagen alpha-1(II) chain	rs6993770	106581528	8	A	T	0.713	0.104	0.007	2.1E-50	trans
525	COL2A1	P02458	Collagen alpha-1(II) chain	rs61269268	92241352	14	A	C	0.827	0.105	0.008	1.7E-36	trans
525	COL2A1	P02458	Collagen alpha-1(II) chain	rs11382616	103081465	14	G	GC	0.297	0.089	0.007	9.1E-38	trans
525	COL2A1	P02458	Collagen alpha-1(II) chain	rs12445050	81870969	16	T	C	0.139	0.074	0.009	3.5E-16	trans
525	COL2A1	P02458	Collagen alpha-1(II) chain	rs35005573	5202839	17	G	T	0.520	0.064	0.006	2.5E-24	trans
525	COL2A1	P02458	Collagen alpha-1(II) chain	rs892090	55539072	19	G	T	0.834	0.116	0.009	9.3E-42	trans
525	COL2A1	P02458	Collagen alpha-1(II) chain	rs1033549	19271940	20	T	G	0.378	0.050	0.007	1.2E-14	trans
526	COL3A1	P02461	Collagen alpha-1(III) chain	rs146385050	60637258	17	A	C	0.183	0.239	0.010	8.9E-131	trans
527	COL4A1	P02462	Collagen alpha-1(IV) chain	rs2498323	3451109	4	G	A	0.899	0.145	0.012	4.4E-33	trans
527	COL4A1	P02462	Collagen alpha-1(IV) chain	rs72793280	131562900	5	T	C	0.462	0.052	0.007	1.3E-12	trans

527	COL4A1	P02462	Collagen alpha-1(IV) chain	rs35493868	73039406	7	G	C	0.201	0.076	0.009	5.2E-17	trans
527	COL4A1	P02462	Collagen alpha-1(IV) chain	rs28600710	55436793	8	A	G	0.209	0.082	0.009	5.0E-20	trans
527	COL4A1	P02462	Collagen alpha-1(IV) chain	rs652572	110819457	13	G	A	0.661	0.184	0.008	2.9E-123	cis
527	COL4A1	P02462	Collagen alpha-1(IV) chain	rs116539064	77018400	17	T	C	0.097	0.189	0.013	2.1E-51	trans
527	COL4A1	P02462	Collagen alpha-1(IV) chain	rs12483377	46931109	21	A	G	0.089	0.415	0.013	3.1E-221	trans
527	COL4A1	P02462	Collagen alpha-1(IV) chain	rs738408	44324730	22	T	C	0.217	0.074	0.009	6.5E-17	trans
528	COL5A1	P20908	Collagen alpha-1(V) chain	rs9821691	72396902	3	T	G	0.429	0.056	0.008	1.3E-13	trans
528	COL5A1	P20908	Collagen alpha-1(V) chain	rs114694170	88180196	5	C	T	0.060	0.115	0.016	2.6E-13	trans
528	COL5A1	P20908	Collagen alpha-1(V) chain	rs4541868	106590705	8	C	A	0.726	0.072	0.008	6.1E-18	trans
528	COL5A1	P20908	Collagen alpha-1(V) chain	rs11995824	120012700	8	G	C	0.543	0.058	0.007	9.3E-15	trans
528	COL5A1	P20908	Collagen alpha-1(V) chain	rs12002679	137533634	9	G	C	0.282	0.236	0.009	2.8E-168	cis
528	COL5A1	P20908	Collagen alpha-1(V) chain	rs56278466	17875857	10	G	T	0.661	0.103	0.008	1.0E-39	trans
528	COL5A1	P20908	Collagen alpha-1(V) chain	rs12358789	65121485	10	G	A	0.436	0.058	0.008	3.7E-14	trans
528	COL5A1	P20908	Collagen alpha-1(V) chain	rs2509582	128607609	11	C	T	0.744	0.073	0.009	9.6E-18	trans
528	COL5A1	P20908	Collagen alpha-1(V) chain	rs9594738	42952145	13	T	C	0.488	0.089	0.007	3.5E-33	trans
528	COL5A1	P20908	Collagen alpha-1(V) chain	rs892090	55539072	19	G	T	0.834	0.072	0.010	6.8E-13	trans
529	COL6A3	P12111	Collagen alpha-3(VI) chain	rs1050785	238232752	2	A	C	0.601	0.092	0.008	2.4E-33	cis
529	COL6A3	P12111	Collagen alpha-3(VI) chain	rs186021206	7069412	17	A	G	0.006	0.389	0.050	9.9E-15	trans
529	COL6A3	P12111	Collagen alpha-3(VI) chain	rs3088026	47549613	21	C	T	0.911	0.113	0.013	1.7E-18	trans
530	COL9A1	P20849	Collagen alpha-1(IX) chain	rs79220007	26098474	6	C	T	0.077	0.113	0.014	1.5E-16	trans
530	COL9A1	P20849	Collagen alpha-1(IX) chain	rs34072915	4973113	10	T	G	0.885	0.101	0.011	3.0E-19	trans
531	COLEC12	Q5KU26	Collectin-12	rs11606287	47407439	11	C	A	0.636	0.053	0.007	1.1E-13	trans
531	COLEC12	Q5KU26	Collectin-12	rs34262842	20355811	16	A	G	0.834	0.069	0.009	8.2E-14	trans
531	COLEC12	Q5KU26	Collectin-12	rs149622251	331717	18	C	T	0.994	0.849	0.045	4.7E-78	cis
532	COMMD1	Q8N668	COMM domain-containing protein 1	rs55677935	62228013	2	C	T	0.983	0.673	0.030	3.7E-114	cis
532	COMMD1	Q8N668	COMM domain-containing protein 1	rs1354034	56849749	3	C	T	0.605	0.080	0.008	2.0E-25	trans
532	COMMD1	Q8N668	COMM domain-containing protein 1	rs150753801	47455100	4	T	C	0.993	0.447	0.046	3.3E-22	trans
533	COMMD9	Q9P000	COMM domain-containing protein 9	rs1354034	56849749	3	C	T	0.604	0.063	0.008	3.2E-16	trans
533	COMMD9	Q9P000	COMM domain-containing protein 9	rs2868346	44547970	20	C	T	0.241	0.074	0.009	2.4E-17	trans
534	COMP	P49747	Cartilage oligomeric matrix protein	rs4233367	161163037	1	T	C	0.391	0.066	0.007	1.7E-19	trans
534	COMP	P49747	Cartilage oligomeric matrix protein	rs56278466	17875857	10	G	T	0.661	0.124	0.008	1.1E-60	trans
534	COMP	P49747	Cartilage oligomeric matrix protein	rs60648773	126237281	11	G	A	0.772	0.067	0.009	5.4E-15	trans
534	COMP	P49747	Cartilage oligomeric matrix protein	rs186021206	7069412	17	A	G	0.006	0.987	0.049	1.8E-90	trans
534	COMP	P49747	Cartilage oligomeric matrix protein	rs12974746	18906548	19	A	G	0.970	0.579	0.022	3.7E-157	cis
535	COMT	P21964	Catechol O-methyltransferase	rs1354034	56849749	3	C	T	0.604	0.051	0.007	4.2E-12	trans
535	COMT	P21964	Catechol O-methyltransferase	rs4680	19951271	22	G	A	0.482	0.445	0.008	0.0E+00	cis
536	COQ7	Q99807	5-demethoxyubiquinone hydroxylase, mitochondrial	rs4665972	27598097	2	T	C	0.392	0.062	0.008	1.7E-15	trans
536	COQ7	Q99807	5-demethoxyubiquinone hydroxylase, mitochondrial	rs4835265	146821410	4	A	C	0.156	0.132	0.010	7.4E-37	trans
536	COQ7	Q99807	5-demethoxyubiquinone hydroxylase, mitochondrial	rs11074359	19085298	16	C	T	0.361	0.121	0.008	6.6E-54	cis
536	COQ7	Q99807	5-demethoxyubiquinone hydroxylase, mitochondrial	rs7189522	80498094	16	T	C	0.273	0.057	0.008	1.1E-11	trans
536	COQ7	Q99807	5-demethoxyubiquinone hydroxylase, mitochondrial	rs9959832	56086820	18	C	T	0.202	0.106	0.009	3.0E-29	trans
536	COQ7	Q99807	5-demethoxyubiquinone hydroxylase, mitochondrial	rs7256200	45415935	19	G	T	0.872	0.081	0.011	1.0E-12	trans
537	CORO1A	P31146	Coronin-1A	rs342299	106373718	7	C	T	0.545	0.063	0.008	7.9E-17	trans
537	CORO1A	P31146	Coronin-1A	rs10432210	22861794	18	T	C	0.209	0.064	0.009	3.5E-12	trans
538	COX5B	P10606	Cytochrome c oxidase subunit 5B, mitochondrial	rs112327139	48859320	4	C	T	0.993	0.689	0.045	1.4E-53	trans
538	COX5B	P10606	Cytochrome c oxidase subunit 5B, mitochondrial	rs201327644	52729913	4	C	T	0.993	0.617	0.050	8.4E-35	trans
538	COX5B	P10606	Cytochrome c oxidase subunit 5B, mitochondrial	rs17569603	45008501	7	C	T	0.174	0.069	0.010	8.3E-12	trans
539	CPA1	P15085	Carboxypeptidase A1	rs35004807	51108411	1	TGTAA	T	0.345	0.067	0.008	1.3E-17	trans
539	CPA1	P15085	Carboxypeptidase A1	rs2641348	120437884	1	A	G	0.889	0.095	0.012	5.6E-16	trans
539	CPA1	P15085	Carboxypeptidase A1	rs13103023	39457617	4	G	A	0.672	0.073	0.008	1.5E-20	trans
539	CPA1	P15085	Carboxypeptidase A1	rs11724862	186572503	4	T	C	0.764	0.063	0.009	1.7E-12	trans
539	CPA1	P15085	Carboxypeptidase A1	rs11759956	7109097	6	T	C	0.549	0.058	0.007	4.8E-15	trans
539	CPA1	P15085	Carboxypeptidase A1	rs13226219	130019491	7	C	T	0.129	0.256	0.011	9.2E-118	cis
539	CPA1	P15085	Carboxypeptidase A1	rs4921967	18719629	8	G	T	0.313	0.073	0.008	5.4E-20	trans
539	CPA1	P15085	Carboxypeptidase A1	rs10901250	136121626	9	A	G	0.121	0.091	0.011	6.5E-16	trans
539	CPA1	P15085	Carboxypeptidase A1	rs56278466	17875857	10	G	T	0.661	0.055	0.008	7.4E-13	trans
539	CPA1	P15085	Carboxypeptidase A1	rs174548	61571348	11	G	C	0.314	0.074	0.008	3.7E-21	trans
539	CPA1	P15085	Carboxypeptidase A1	rs686056	100646689	11	G	A	0.678	0.093	0.008	6.9E-33	trans
539	CPA1	P15085	Carboxypeptidase A1	rs72802342	75234872	16	C	A	0.924	0.367	0.014	3.7E-146	trans
539	CPA1	P15085	Carboxypeptidase A1	rs1673931	88976477	16	T	C	0.622	0.115	0.008	4.8E-51	trans
539	CPA1	P15085	Carboxypeptidase A1	rs17138478	36073320	17	A	C	0.131	0.082	0.011	3.4E-14	trans
539	CPA1	P15085	Carboxypeptidase A1	rs1698113	49259856	19	C	T	0.484	0.060	0.008	2.0E-14	trans
539	CPA1	P15085	Carboxypeptidase A1	rs4817932	39619023	21	C	G	0.637	0.060	0.008	7.5E-14	trans
539	CPA1	P15085	Carboxypeptidase A1	rs80184311	37908610	22	C	G	0.106	0.080	0.012	1.5E-11	trans
540	CPA2	P48052	Carboxypeptidase A2	rs6952090	129922498	7	T	C	0.652	0.369	0.008	0.0E+00	cis
540	CPA2	P48052	Carboxypeptidase A2	rs56057779	10516880	8	C	G	0.564	0.063	0.007	2.9E-18	trans
540	CPA2	P48052	Carboxypeptidase A2	rs2519093	136141870	9	C	T	0.815	0.111	0.009	8.7E-33	trans
540	CPA2	P48052	Carboxypeptidase A2	rs56278466	17875857	10	G	T	0.661	0.085	0.008	6.2E-29	trans
540	CPA2	P48052	Carboxypeptidase A2	rs72802342	75234872	16	C	A	0.924	0.229	0.014	1.4E-61	trans
541	CPA4	Q9UI42	Carboxypeptidase A4	rs80293268	8207579	1	G	C	0.952	0.157	0.017	1.4E-20	trans
541	CPA4	Q9UI42	Carboxypeptidase A4	rs11384422	60499861	2	AG	A	0.582	0.047	0.007	1.4E-11	trans
541	CPA4	Q9UI42	Carboxypeptidase A4	rs34587586	129938598	7	G	T	0.603	0.535	0.008	0.0E+00	cis
541	CPA4	Q9UI42	Carboxypeptidase A4	rs186021206	7069412	17	A	G	0.006	0.343	0.047	2.7E-13	trans
541	CPA4	Q9UI42	Carboxypeptidase A4	rs12986064	54755133	19	T	C	0.493	0.082	0.007	5.2E-31	trans
542	CPB1	P15086	Carboxypeptidase B	rs333947	110470764	1	G	A	0.852	0.076	0.010	2.0E-13	trans
542	CPB1	P15086	Carboxypeptidase B	rs2027447	120440029	1	T	C	0.889	0.097	0.012	9.3E-17	trans
542	CPB1	P15086	Carboxypeptidase B	rs17032925	67266483	2	T	C	0.917	0.101	0.013	4.2E-14	trans
542	CPB1	P15086	Carboxypeptidase B	rs13318853	148562399	3	A	G	0.237	0.129	0.009	4.4E-50	cis
542	CPB1	P15086	Carboxypeptidase B	rs35400735	39447306	4	T	TG	0.733	0.064	0.009	5.4E-13	trans
542	CPB1	P15086	Carboxypeptidase B	rs9482772	127446790	6	C	T	0.451	0.075	0.007	5.2E-24	trans
542	CPB1	P15086	Carboxypeptidase B	rs4921967	18719629	8	G	T	0.313	0.079	0.008	3.0E-23	trans
542	CPB1	P15086	Carboxypeptidase B	rs56278466	17875857	10	G	T	0.661	0.055	0.008	1.2E-12	trans
542	CPB1	P15086	Carboxypeptidase B	rs174548	61571348	11	G	C	0.314	0.072	0.008	8.6E-20	trans
542	CPB1	P15086	Carboxypeptidase B	rs686056	100646689	11	G	A	0.678	0.089	0.008	4.2E-30	trans
542	CPB1	P15086	Carboxypeptidase B	rs72802342	75234872	16	C	A	0.924	0.407	0.014	5.4E-178	trans
542	CPB1	P15086	Carboxypeptidase B	rs1673931	88976477	16	T	C	0.622	0.160	0.008	1.2E-96	trans
542	CPB1	P15086	Carboxypeptidase B	rs2211864	39626474	21	A	T	0.507	0.058	0.007	2.4E-15	trans
542	CPB1	P15086	Carboxypeptidase B	rs80184311	37908610	22	C	G	0.106	0.080	0.012	1.6E-11	trans
543	CPB2	Q96IY4	Carboxypeptidase B2	rs1260326	27730940	2	T	C	0.392	0.088	0.007	7.8E-38	trans
543	CPB2	Q96IY4	Carboxypeptidase B2	rs192519670	161160094	6	T	C	0.996	0.398	0.058	9.1E-12	trans
543	CPB2	Q96IY4	Carboxypeptidase B2	rs2126263	9181611	8	A	G	0.899	0.084	0.011	6.1E-14	trans
543	CPB2	Q96IY4	Carboxypeptidase B2	rs78689694	126234820	11	C	G	0.133	0.079	0.010	1.4E-15	trans
543	CPB2	Q96IY4	Carboxypeptidase B2	rs6561283	46640837	13	G	A	0.735	0.674	0.008	0.0E+00	cis
543	CPB2	Q96IY4	Carboxypeptidase B2	rs28929474	94844947	14	T	C	0.021	0.161	0.023	6.3E-12	trans
544	CPE	P16870	Carboxypeptidase E	rs1550270	166261800	4	C	T	0.301	0.256	0.008	7.0E-211	cis
544	CPE	P16870	Carboxypeptidase E	rs34958196	73043101	7	A	G	0.129	0.083	0.011	1.0E-13	trans
544	CPE	P16870	Carboxypeptidase E	rs532436	136149830	9	G	A	0.815	0.083	0.010	1.2E-17	trans
544	CPE	P16870	Carboxypeptidase E	rs56278466	17875857	10	G	T	0.661	0.063	0.008	1.9E-15	trans
544	CPE	P16870	Carboxypeptidase E	rs184458383	75249552	16	C	T	0.924	0.125	0.014	8.7E-19	trans
545	CPM	P14384	Carboxypeptidase M	rs11118611	220990473	1	G	A	0.291	0.089	0.008	4.4E-29	trans
545	CPM	P14384	Carboxypeptidase M	rs1862069	169933741	2	G	A	0.463	0.054	0.007	1.6E-13	trans
545	CPM	P14384	Carboxypeptidase M	rs28601761	126500031	8	C	G	0.581	0.083	0.007	4.8E-29	trans
545	CPM	P14384	Carboxypeptidase M	rs56278466	17875857	10	G	T	0.661	0.066	0.008	2.9E-18	trans
545	CPM	P14384	Carboxypeptidase M	rs10751647	306884	11	C	T	0.402	0.052	0.007	2.6E-12	trans
545	CPM	P14384	Carboxypeptidase M	rs1601935	58671765	15	T	G	0.656	0.077	0.008	4.7E-24	trans
545	CPM	P14384	Carboxypeptidase M	rs200293726	79754440	16	T	A	0.306	0.057	0.008	6.8E-13	trans
545	CPM	P14384	Carboxypeptidase M	rs186021206	7069412	17	A	G	0.006	0.347	0.050	2.7E-12	trans
545	CPM	P14384	Carboxypeptidase M	rs5112	45430280	19	C	G	0.466	0.058	0.008	6.2E-14	trans
545	CPM	P14384	Carboxypeptidase M	rs11668882	54675097	19	C	T	0.441	0.054	0.007	1.4E-13	trans
545	CPM	P14384	Carboxypeptidase M	rs6115156	25333189	20	C	G	0.522	0.078	0.007	2.7E-27	trans
546	CPOX	P36551	Oxygen-dependent coproporphyrinogen-III oxidase, mitochondrial	rs35393423	5174908	3	A	AT	0.676	0.062	0.008	2.2E-14	trans
546	CPOX	P36551	Oxygen-dependent coproporphyrinogen-III oxidase, mitochondrial	rs1729988	98318393	3	C	T	0.869	0.157	0.011	4.9E-44	cis
546	CPOX	P36551	Oxygen-dependent coproporphyrinogen-III oxidase, mitochondrial	rs1800562	26093141	6	A	G	0.078	0.193	0.014	4.3E-42	trans
546	CPOX	P36551	Oxygen-dependent coproporphyrinogen-III oxidase, mitochondrial	rs855791	37462936	22	G	A	0.561	0.063	0.008	9.0E-17	trans
547	CPPED1	Q9BRF8	Serine/threonine-protein phosphatase CPPED1	rs8062313	12899766	16	C	T	0.173	0.828	0.010	0.0E+00	cis
548	CPQ	Q9Y646	Carboxypeptidase Q	rs687339	135932359	3	T	C	0.771	0.078	0.008	2.7E-22	trans
548	CPQ	Q9Y646	Carboxypeptidase Q	rs13107325	103188709	4	C	T	0.925	0.244	0.013	1.0E-80	trans
548	CPQ	Q9Y646	Carboxypeptidase Q	rs56055826	119789424	4	T	A	0.939	0.105	0.014	1.8E-13	trans
548	CPQ	Q9Y646	Carboxypeptidase Q	rs148684432	98041637	8	G	A	0.991	1.949	0.038	0.0E+00	cis
548	CPQ	Q9Y646	Carboxypeptidase Q	rs2272662	145639726	8	T	C	0.422	0.054	0.007	7.4E-14	trans
548	CPQ	Q9Y646	Carboxypeptidase Q	rs11622951	39722682	14	A	G	0.462	0.055	0.007	2.9E-16	trans
548	CPQ	Q9Y646	Carboxypeptidase Q	rs145078947	93652974	14	T	G	0.003	1.185	0.067	1.4E-70	trans
548	CPQ	Q9Y646	Carboxypeptidase Q	rs55707100	43820717	15	T	C	0.024	0.158	0.022	3.6E-13	trans
548	CPQ	Q9Y646	Carboxypeptidase Q	rs58542926	19379549	19	T	C	0.076	0.196	0.013	2.6E-53	trans
549	CPVL	Q9H3G5	Probable serine carboxypeptidase CPVL	rs710446	186459927	3	T	C	0.591	0.053	0.006	1.5E-18	trans
549	CPVL	Q9H3G5	Probable serine carboxypeptidase CPVL	rs2545801	176841339	5	T	C	0.256	0.050	0.007	2.0E-13	trans
549	CPVL	Q9H3G5	Probable serine carboxypeptidase CPVL	rs993632	46086257	6	C	T	0.357	0.050	0.007	1.9E-14	trans
549	CPVL	Q9H3G5	Probable serine carboxypeptidase CPVL	rs34219043	29160604	7	C	T	0.800	0.842	0.009	0.0E+00	cis
549	CPVL	Q9H3G5	Probable serine carboxypeptidase CPVL	rs56278466	17875857	10	G	T	0.661	0.063	0.006	4.8E-24	trans
549	CPVL	Q9H3G5	Probable serine carboxypeptidase CPVL	rs145078947	93652974	14	T	G	0.003	1.492	0.059	7.9E-143	trans
549	CPVL	Q9H3G5	Probable serine carboxypeptidase CPVL	rs15951	5075248	16	G	A	0.467	0.088	0.006	3.2E-48	trans
549	CPVL	Q9H3G5	Probable serine carboxypeptidase CPVL	rs186021206	7069412	17	A	G	0.006	0.325	0.041	1.6E-15	trans
549	CPVL	Q9H3G5	Probable serine carboxypeptidase CPVL	rs74181667	12758723	19	G	A	0.366	0.055	0.006	5.0E-19	trans
550	CPXM1	Q96SM3	Probable carboxypeptidase X1	rs1434282	199010721	1	C	T	0.274	0.084	0.008	1.0E-24	trans
550	CPXM1	Q96SM3	Probable carboxypeptidase X1	rs13412535	224874874	2	G	A	0.769	0.058	0.009	1.7E-11	trans
550	CPXM1	Q96SM3	Probable carboxypeptidase X1	rs7618405	18250509	3	C	A	0.795	0.064	0.009	1.0E-12	trans
550	CPXM1	Q96SM3	Probable carboxypeptidase X1	rs1354034	56849749	3	T	C	0.396	0.065	0.007	1.2E-18	trans
550	CPXM1	Q96SM3	Probable carboxypeptidase X1	rs3016013	31351242	6	A	G	0.721	0.094	0.008	4.0E-31	trans
550	CPXM1	Q96SM3	Probable carboxypeptidase X1	rs6961069	80218961	7	T	C	0.402	0.055	0.007	1.1E-13	trans
550	CPXM1	Q96SM3	Probable carboxypeptidase X1	rs6972167	100080013	7	G	T	0.802	0.063	0.009	3.7E-12	trans
550	CPXM1	Q96SM3	Probable carboxypeptidase X1	rs6993770	106581528	8	A	T	0.714	0.164	0.008	8.7E-94	trans
550	CPXM1	Q96SM3	Probable carboxypeptidase X1	rs692502	4745645	9	A	G	0.556	0.051	0.007	4.3E-12	trans
550	CPXM1	Q96SM3	Probable carboxypeptidase X1	rs61469632	135861990	9	T	C	0.937	0.115	0.015	1.3E-14	trans

550	CPXM1	Q96SM3	Probable carboxypeptidase X1	rs10740118	65101207	10	C	G	0.414	0.069	0.007	6.2E-21	trans
550	CPXM1	Q96SM3	Probable carboxypeptidase X1	rs12445050	81870969	16	T	C	0.138	0.109	0.010	1.5E-25	trans
550	CPXM1	Q96SM3	Probable carboxypeptidase X1	rs57839578	5268337	17	A	T	0.521	0.052	0.007	1.1E-12	trans
550	CPXM1	Q96SM3	Probable carboxypeptidase X1	rs892090	55539072	19	G	T	0.834	0.162	0.010	4.5E-62	trans
550	CPXM1	Q96SM3	Probable carboxypeptidase X1	rs215545	2782015	20	C	T	0.765	0.363	0.009	0.0E+00	cis
550	CPXM1	Q96SM3	Probable carboxypeptidase X1	rs4814837	19241680	20	T	C	0.343	0.070	0.008	7.6E-19	trans
550	CPXM1	Q96SM3	Probable carboxypeptidase X1	rs16989483	43537200	20	T	C	0.728	0.059	0.008	1.4E-12	trans
550	CPXM1	Q96SM3	Probable carboxypeptidase X1	rs2027828	99958809	X	G	T	0.333	0.048	0.006	3.2E-14	trans
551	CPXM2	Q8N436	Inactive carboxypeptidase-like protein X2	rs28741041	125650709	10	G	C	0.613	0.477	0.008	0.0E+00	cis
552	CR1	P17927	Complement receptor type 1	rs679515	207750568	1	T	C	0.174	0.540	0.010	0.0E+00	cis
552	CR1	P17927	Complement receptor type 1	rs1257169	134963862	2	C	A	0.501	0.096	0.007	8.3E-45	trans
552	CR1	P17927	Complement receptor type 1	rs13064814	186654766	3	A	G	0.286	0.127	0.008	1.3E-62	trans
552	CR1	P17927	Complement receptor type 1	rs1800759	100065509	4	T	G	0.387	0.048	0.007	6.5E-12	trans
552	CR1	P17927	Complement receptor type 1	rs6910861	10538183	6	A	G	0.545	0.048	0.007	3.0E-12	trans
552	CR1	P17927	Complement receptor type 1	rs13204265	32420502	6	C	G	0.161	0.082	0.009	9.2E-19	trans
552	CR1	P17927	Complement receptor type 1	rs4841132	9183596	8	A	G	0.092	0.085	0.012	8.5E-13	trans
552	CR1	P17927	Complement receptor type 1	rs2031902	33117524	9	C	T	0.412	0.050	0.007	5.1E-13	trans
552	CR1	P17927	Complement receptor type 1	rs74622686	134264227	11	G	A	0.147	0.078	0.010	8.5E-16	trans
552	CR1	P17927	Complement receptor type 1	rs186021206	7069412	17	A	G	0.006	1.661	0.047	6.2E-271	trans
552	CR1	P17927	Complement receptor type 1	rs34557412	16852187	17	G	A	0.007	0.358	0.041	4.5E-18	trans
552	CR1	P17927	Complement receptor type 1	rs77542162	67081278	17	G	A	0.023	0.175	0.023	2.4E-14	trans
552	CR1	P17927	Complement receptor type 1	rs35106244	49203829	19	T	C	0.463	0.074	0.007	1.2E-25	trans
553	CR2	P20023	Complement receptor type 2	rs4656100	86917944	1	C	T	0.545	0.065	0.007	7.9E-19	trans
553	CR2	P20023	Complement receptor type 2	rs61821113	207596464	1	C	T	0.886	0.283	0.011	2.8E-134	cis
553	CR2	P20023	Complement receptor type 2	rs11585945	214409010	1	T	A	0.596	0.078	0.007	1.6E-25	trans
553	CR2	P20023	Complement receptor type 2	rs7634389	186738421	3	T	C	0.610	0.068	0.007	2.9E-20	trans
553	CR2	P20023	Complement receptor type 2	rs10012161	177966780	4	A	G	0.184	0.093	0.009	2.2E-23	trans
553	CR2	P20023	Complement receptor type 2	rs1047437	122685727	5	C	G	0.831	0.068	0.010	1.9E-12	trans
553	CR2	P20023	Complement receptor type 2	rs9460100	170583022	6	C	T	0.553	0.059	0.007	8.2E-16	trans
553	CR2	P20023	Complement receptor type 2	rs2389581	16118171	7	G	C	0.348	0.093	0.008	2.6E-34	trans
553	CR2	P20023	Complement receptor type 2	rs7798316	47626397	7	T	A	0.428	0.051	0.007	2.4E-12	trans
553	CR2	P20023	Complement receptor type 2	rs12535578	55154586	7	A	G	0.279	0.080	0.008	4.4E-23	trans
553	CR2	P20023	Complement receptor type 2	rs12554848	36981822	9	T	C	0.728	0.062	0.008	2.2E-14	trans
553	CR2	P20023	Complement receptor type 2	rs4055121	126232337	11	C	T	0.865	0.081	0.011	2.0E-14	trans
553	CR2	P20023	Complement receptor type 2	rs3751437	41133080	13	G	A	0.238	0.061	0.008	5.1E-13	trans
553	CR2	P20023	Complement receptor type 2	rs374039502	108960385	13	A	T	0.020	0.243	0.027	2.2E-19	trans
553	CR2	P20023	Complement receptor type 2	rs186021206	7069412	17	A	G	0.006	1.021	0.050	9.7E-94	trans
553	CR2	P20023	Complement receptor type 2	rs34557412	16852187	17	G	A	0.007	0.805	0.044	4.1E-75	trans
553	CR2	P20023	Complement receptor type 2	rs12950377	81011652	17	T	C	0.843	0.108	0.010	1.3E-27	trans
553	CR2	P20023	Complement receptor type 2	rs660637	57170266	18	G	C	0.584	0.072	0.007	1.1E-22	trans
553	CR2	P20023	Complement receptor type 2	rs200437269	36444600	21	CTG	C	0.110	0.093	0.012	1.6E-14	trans
553	CR2	P20023	Complement receptor type 2	rs73165110	42291856	22	G	A	0.921	0.098	0.013	3.1E-13	trans
554	CRACR2A	Q9BSW2	EF-hand calcium-binding domain-containing protein 4B	rs1354034	56849749	3	C	T	0.604	0.052	0.008	4.7E-12	trans
554	CRACR2A	Q9BSW2	EF-hand calcium-binding domain-containing protein 4B	rs6993770	106581528	8	A	T	0.713	0.059	0.008	9.8E-13	trans
554	CRACR2A	Q9BSW2	EF-hand calcium-binding domain-containing protein 4B	rs11227136	64931964	11	G	A	0.757	0.063	0.009	5.9E-13	trans
555	CRADD	P78560	Death domain-containing protein CRADD	rs1354034	56849749	3	C	T	0.604	0.055	0.008	7.5E-13	trans
555	CRADD	P78560	Death domain-containing protein CRADD	rs342298	106373646	7	C	T	0.546	0.052	0.008	5.9E-12	trans
555	CRADD	P78560	Death domain-containing protein CRADD	rs7896518	65104500	10	G	A	0.424	0.054	0.008	2.5E-12	trans
556	CREG1	O75629	Protein CREG1	rs7513428	167515272	1	T	C	0.159	0.254	0.010	3.2E-146	cis
556	CREG1	O75629	Protein CREG1	rs687339	135932359	3	T	C	0.771	0.068	0.009	1.2E-15	trans
556	CREG1	O75629	Protein CREG1	rs13107325	103188709	4	T	C	0.075	0.122	0.014	3.2E-19	trans
556	CREG1	O75629	Protein CREG1	rs77945361	154254534	4	G	A	0.852	0.069	0.010	7.4E-12	trans
556	CREG1	O75629	Protein CREG1	rs4594967	130366161	6	G	A	0.675	0.060	0.008	4.7E-15	trans
556	CREG1	O75629	Protein CREG1	rs9414801	65149089	10	G	A	0.467	0.049	0.007	1.3E-11	trans
556	CREG1	O75629	Protein CREG1	rs150641790	41687844	13	TG	T	0.075	0.104	0.014	1.9E-14	trans
556	CREG1	O75629	Protein CREG1	rs145078947	93652974	14	T	G	0.003	1.005	0.071	3.9E-45	trans
556	CREG1	O75629	Protein CREG1	rs58542926	19379549	19	T	C	0.075	0.211	0.014	2.5E-54	trans
556	CREG1	O75629	Protein CREG1	rs429358	45411941	19	T	C	0.844	0.121	0.010	2.5E-34	trans
556	CREG1	O75629	Protein CREG1	rs3747207	44324855	22	A	G	0.215	0.068	0.009	3.9E-15	trans
557	CRELD1	Q96HD1	Protein disulfide isomerase CRELD1	rs61747728	179526214	1	T	C	0.038	0.114	0.016	1.8E-12	trans
557	CRELD1	Q96HD1	Protein disulfide isomerase CRELD1	rs9878047	9985457	3	C	T	0.249	0.832	0.009	0.0E+00	cis
557	CRELD1	Q96HD1	Protein disulfide isomerase CRELD1	rs204893	32094593	6	C	T	0.431	0.045	0.006	6.7E-13	trans
557	CRELD1	Q96HD1	Protein disulfide isomerase CRELD1	rs28929474	94844947	14	T	C	0.021	0.209	0.022	9.7E-22	trans
558	CRELD2	Q6UXH1	Protein disulfide isomerase CRELD2	rs1295638	227069267	1	G	A	0.516	0.063	0.007	9.4E-19	trans
558	CRELD2	Q6UXH1	Protein disulfide isomerase CRELD2	rs9852409	57623541	3	T	A	0.178	0.185	0.009	1.6E-87	trans
558	CRELD2	Q6UXH1	Protein disulfide isomerase CRELD2	rs6796	6502367	7	C	T	0.279	0.083	0.008	2.7E-25	trans
558	CRELD2	Q6UXH1	Protein disulfide isomerase CRELD2	rs3752617	127220917	7	G	T	0.932	0.150	0.014	7.3E-26	trans
558	CRELD2	Q6UXH1	Protein disulfide isomerase CRELD2	rs9987289	9183358	8	A	G	0.092	0.172	0.012	2.3E-44	trans
558	CRELD2	Q6UXH1	Protein disulfide isomerase CRELD2	rs10488686	32129891	11	A	G	0.366	0.056	0.007	3.6E-14	trans
558	CRELD2	Q6UXH1	Protein disulfide isomerase CRELD2	rs1801689	64210580	17	C	A	0.030	0.155	0.021	6.0E-14	trans
558	CRELD2	Q6UXH1	Protein disulfide isomerase CRELD2	rs2659005	79218714	17	T	C	0.441	0.049	0.007	5.1E-12	trans
558	CRELD2	Q6UXH1	Protein disulfide isomerase CRELD2	rs74510325	50315382	22	C	G	0.965	0.813	0.020	0.0E+00	cis
559	CRH	P06850	Corticoliberin	rs12740374	109817590	1	G	T	0.779	0.106	0.009	8.7E-34	trans
559	CRH	P06850	Corticoliberin	rs10183338	111610816	2	G	T	0.708	0.058	0.008	4.0E-13	trans
559	CRH	P06850	Corticoliberin	rs4561605	144272150	2	G	C	0.461	0.071	0.007	5.2E-22	trans
559	CRH	P06850	Corticoliberin	rs13017862	200449588	2	T	C	0.817	0.076	0.009	7.3E-16	trans
559	CRH	P06850	Corticoliberin	rs7687767	57824932	4	G	A	0.184	0.084	0.009	2.9E-19	trans
559	CRH	P06850	Corticoliberin	rs6887216	76211295	5	A	T	0.638	0.065	0.008	1.0E-17	trans
559	CRH	P06850	Corticoliberin	rs331700	124826611	5	A	G	0.455	0.050	0.007	8.8E-12	trans
559	CRH	P06850	Corticoliberin	rs12518248	173081460	5	C	T	0.417	0.059	0.007	7.5E-16	trans
559	CRH	P06850	Corticoliberin	rs9503212	2501535	6	G	A	0.522	0.109	0.007	2.6E-50	trans
559	CRH	P06850	Corticoliberin	rs9264839	31270949	6	C	T	0.833	0.096	0.010	6.6E-23	trans
559	CRH	P06850	Corticoliberin	rs1229027	39023276	7	G	A	0.757	0.090	0.008	4.6E-26	trans
559	CRH	P06850	Corticoliberin	rs111632177	150931805	7	A	G	0.114	0.092	0.011	7.8E-16	trans
559	CRH	P06850	Corticoliberin	rs59104426	67172949	8	T	A	0.967	0.681	0.021	8.1E-235	cis
559	CRH	P06850	Corticoliberin	rs4263799	72394991	8	T	C	0.480	0.164	0.007	1.2E-105	trans
559	CRH	P06850	Corticoliberin	rs7387462	100603910	8	T	C	0.043	0.440	0.018	5.1E-125	trans
559	CRH	P06850	Corticoliberin	rs5898348	77001834	9	GA	G	0.273	0.080	0.008	2.0E-22	trans
559	CRH	P06850	Corticoliberin	rs59585065	108735211	9	GT	G	0.712	0.094	0.008	4.9E-29	trans
559	CRH	P06850	Corticoliberin	rs1463597	81139141	10	A	G	0.541	0.067	0.007	2.8E-20	trans
559	CRH	P06850	Corticoliberin	rs7192602	78346429	16	G	A	0.703	0.058	0.008	5.4E-13	trans
559	CRH	P06850	Corticoliberin	rs77542162	67081278	17	G	A	0.023	0.164	0.024	1.4E-11	trans
559	CRH	P06850	Corticoliberin	rs7412	45412079	19	T	C	0.081	0.094	0.013	1.8E-12	trans
560	CRHBP	P24387	Corticotropin-releasing factor-binding protein	rs11162598	79496383	1	A	G	0.357	0.053	0.007	1.8E-13	trans
560	CRHBP	P24387	Corticotropin-releasing factor-binding protein	rs6798915	194497262	3	G	C	0.302	0.052	0.007	2.2E-12	trans
560	CRHBP	P24387	Corticotropin-releasing factor-binding protein	rs6896257	76210596	5	G	A	0.362	0.588	0.008	0.0E+00	cis
560	CRHBP	P24387	Corticotropin-releasing factor-binding protein	rs6924387	137082948	6	A	G	0.594	0.058	0.007	6.0E-17	trans
560	CRHBP	P24387	Corticotropin-releasing factor-binding protein	rs10104003	55422440	8	T	C	0.210	0.151	0.008	1.7E-73	trans
561	CRIM1	Q9NZV1	Cysteine-rich motor neuron 1 protein	rs201258494	36583342	2	T	TGCG	0.808	0.153	0.009	3.1E-61	cis
561	CRIM1	Q9NZV1	Cysteine-rich motor neuron 1 protein	rs6474527	38938365	8	G	C	0.349	0.102	0.008	9.4E-40	trans
561	CRIM1	Q9NZV1	Cysteine-rich motor neuron 1 protein	rs11244061	136153981	9	C	T	0.885	0.094	0.011	1.6E-16	trans
562	CRIP2	P52943	Cysteine-rich protein 2	rs705379	94953895	7	A	G	0.477	0.090	0.007	7.9E-36	trans
562	CRIP2	P52943	Cysteine-rich protein 2	rs495203	136145240	9	T	C	0.318	0.073	0.008	1.9E-21	trans
562	CRIP2	P52943	Cysteine-rich protein 2	rs80324969	105917816	14	C	G	0.171	0.183	0.010	9.3E-75	cis
563	CRISP2	P16562	Cysteine-rich secretory protein 2	rs9287979	152356361	2	A	G	0.375	0.066	0.006	1.4E-32	trans
563	CRISP2	P16562	Cysteine-rich secretory protein 2	rs3087450	127327309	3	G	A	0.987	0.167	0.024	2.0E-12	trans
563	CRISP2	P16562	Cysteine-rich secretory protein 2	rs34687149	138997538	5	TA	T	0.052	0.103	0.012	2.4E-17	trans
563	CRISP2	P16562	Cysteine-rich secretory protein 2	rs555247	49710105	6	A	G	0.456	0.489	0.007	0.0E+00	cis
563	CRISP2	P16562	Cysteine-rich secretory protein 2	rs72558770	87960011	6	A	ATAG	0.480	0.049	0.007	5.2E-14	trans
563	CRISP2	P16562	Cysteine-rich secretory protein 2	rs6460105	63502692	7	A	C	0.303	0.135	0.006	3.4E-116	trans
563	CRISP2	P16562	Cysteine-rich secretory protein 2	rs10993994	51549496	10	T	C	0.392	0.041	0.005	1.1E-13	trans
563	CRISP2	P16562	Cysteine-rich secretory protein 2	rs74973131	135045332	10	G	A	0.968	0.169	0.015	9.3E-29	trans
563	CRISP2	P16562	Cysteine-rich secretory protein 2	rs1766138	36688560	14	G	C	0.675	0.040	0.006	3.1E-12	trans
563	CRISP2	P16562	Cysteine-rich secretory protein 2	rs8030179	41929220	15	A	T	0.201	0.045	0.007	1.0E-11	trans
563	CRISP2	P16562	Cysteine-rich secretory protein 2	rs4911257	31359574	20	T	C	0.604	0.049	0.005	7.6E-19	trans
563	CRISP2	P16562	Cysteine-rich secretory protein 2	rs67610389	43294820	21	A	G	0.288	0.045	0.006	2.9E-14	trans
564	CRISP3	P54108	Cysteine-rich secretory protein 3	rs3917932	36943916	1	C	G	0.425	0.084	0.007	3.2E-38	trans
564	CRISP3	P54108	Cysteine-rich secretory protein 3	rs149290349	43451957	2	A	G	0.074	0.087	0.012	2.3E-12	trans
564	CRISP3	P54108	Cysteine-rich secretory protein 3	rs778139	61684564	2	G	A	0.511	0.052	0.006	2.3E-16	trans
564	CRISP3	P54108	Cysteine-rich secretory protein 3	rs35987226	101636366	2	TA	T	0.221	0.066	0.008	5.4E-18	trans
564	CRISP3	P54108	Cysteine-rich secretory protein 3	rs62246445	47175194	3	A	G	0.175	0.065	0.008	8.9E-15	trans
564	CRISP3	P54108	Cysteine-rich secretory protein 3	rs2734031	128301390	3	T	C	0.090	0.101	0.011	2.2E-19	trans
564	CRISP3	P54108	Cysteine-rich secretory protein 3	rs218264	55408875	4	T	A	0.247	0.080	0.007	6.2E-27	trans
564	CRISP3	P54108	Cysteine-rich secretory protein 3	rs7705526	1285974	5	A	C	0.325	0.064	0.007	1.3E-20	trans
564	CRISP3	P54108	Cysteine-rich secretory protein 3	rs114300451	31234537	6	G	A	0.932	0.147	0.015	2.3E-23	trans
564	CRISP3	P54108	Cysteine-rich secretory protein 3	rs1025347	49652977	6	G	A	0.504	0.571	0.007	0.0E+00	cis
564	CRISP3	P54108	Cysteine-rich secretory protein 3	rs149110519	144385777	6	T	C	0.036	0.250	0.020	5.4E-36	trans
564	CRISP3	P54108	Cysteine-rich secretory protein 3	rs2158799	28277107	7	G	C	0.609	0.049	0.007	6.6E-14	trans
564	CRISP3	P54108	Cysteine-rich secretory protein 3	rs11769630	50257703	7	A	T	0.073	0.094	0.012	2.4E-14	trans
564	CRISP3	P54108	Cysteine-rich secretory protein 3	rs45577137	48651633	8	G	A	0.045	0.127	0.016	9.2E-15	trans
564	CRISP3	P54108	Cysteine-rich secretory protein 3	rs10107630	130603635	8	C	T	0.426	0.065	0.006	6.3E-24	trans
564	CRISP3	P54108	Cysteine-rich secretory protein 3	rs150813342	135864513	9	C	T	0.991	0.264	0.036	1.7E-13	trans
564	CRISP3	P54108	Cysteine-rich secretory protein 3	rs55742421	961037	10	A	C	0.795	0.057	0.008	6.5E-13	trans
564	CRISP3	P54108	Cysteine-rich secretory protein 3	rs2074038	44087989	11	T	G	0.100	0.083	0.011	7.4E-15	trans
564	CRISP3	P54108	Cysteine-rich secretory protein 3	rs7989654	28625831	13	A	G	0.629	0.049	0.007	1.7E-13	trans
564	CRISP3	P54108	Cysteine-rich secretory protein 3	rs34460966	57011555	15	C	CA	0.376	0.060	0.007	2.5E-19	trans
564	CRISP3	P54108	Cysteine-rich secretory protein 3	rs391855	85928621	16	T	A	0.424	0.047	0.006	6.5E-13	trans
564	CRISP3	P54108	Cysteine-rich secretory protein 3	rs186021206	7069412	17	A	G	0.006	0.364	0.044	1.5E-16	trans
564	CRISP3	P54108	Cysteine-rich secretory protein 3	rs11655264	38138995	17	G	C	0.549	0.108	0.006	8.2E-64	trans
564	CRISP3	P54108	Cysteine-rich secretory protein 3	rs893182	58865068	19	C	T	0.951	0.411	0.015	6.4E-163	trans
564	CRISP3	P54108	Cysteine-rich secretory protein 3	rs243587	19154417	21	A	C	0.521	0.060	0.006	1.5E-20	trans
564	CRISP3	P54108	Cysteine-rich secretory protein 3	rs1978082	147583958	X	G	A	0.425	0.042	0.005	5.1E-15	trans
565	CRKL	P46109	Crk-like protein	rs6993770	106581528	8	A	T	0.713	0.071	0.008	1.3E-17	trans

566	CRLF1	O75462	Cytokine receptor-like factor 1	rs72538440	65225088	2	G	GC	0.576	0.050	0.007	1.3E-11	trans
566	CRLF1	O75462	Cytokine receptor-like factor 1	rs11676298	227291731	2	C	G	0.808	0.069	0.009	9.6E-14	trans
566	CRLF1	O75462	Cytokine receptor-like factor 1	rs1126464	89704365	16	G	C	0.756	0.065	0.008	1.6E-14	trans
566	CRLF1	O75462	Cytokine receptor-like factor 1	rs12986064	54755133	19	T	C	0.492	0.073	0.008	2.4E-22	trans
567	CRNN	Q9UBG3	Cornulin	rs10494275	152391567	1	G	A	0.929	1.187	0.014	0.0E+00	cis
567	CRNN	Q9UBG3	Cornulin	rs9274369	32632523	6	G	A	0.783	0.074	0.008	1.6E-19	trans
567	CRNN	Q9UBG3	Cornulin	rs704	26694861	17	A	G	0.473	0.092	0.007	2.5E-45	trans
568	CRTAC1	Q9NQ79	Cartilage acidic protein 1	rs1042445	186395436	3	T	C	0.222	0.492	0.008	0.0E+00	trans
568	CRTAC1	Q9NQ79	Cartilage acidic protein 1	rs56007204	99625319	10	C	T	0.788	0.778	0.008	0.0E+00	cis
569	CRTAM	O95727	Cytotoxic and regulatory T-cell molecule	rs13401811	111616104	2	G	A	0.818	0.068	0.009	1.1E-13	trans
569	CRTAM	O95727	Cytotoxic and regulatory T-cell molecule	rs1047891	211540507	2	A	C	0.313	0.060	0.008	1.1E-14	trans
569	CRTAM	O95727	Cytotoxic and regulatory T-cell molecule	rs13063578	47087837	3	T	A	0.602	0.100	0.007	1.8E-43	trans
569	CRTAM	O95727	Cytotoxic and regulatory T-cell molecule	rs35829610	131823862	5	C	CTG	0.662	0.057	0.008	3.2E-14	trans
569	CRTAM	O95727	Cytotoxic and regulatory T-cell molecule	rs9267084	31409605	6	A	G	0.154	0.125	0.010	1.5E-36	trans
569	CRTAM	O95727	Cytotoxic and regulatory T-cell molecule	rs2327832	137973068	6	G	A	0.223	0.061	0.009	9.0E-13	trans
569	CRTAM	O95727	Cytotoxic and regulatory T-cell molecule	rs3181356	117692882	9	T	C	0.229	0.062	0.009	1.6E-12	trans
569	CRTAM	O95727	Cytotoxic and regulatory T-cell molecule	rs11215416	115077327	11	A	G	0.743	0.066	0.008	9.2E-15	trans
569	CRTAM	O95727	Cytotoxic and regulatory T-cell molecule	rs2370794	122714782	11	G	A	0.337	0.313	0.008	0.0E+00	cis
569	CRTAM	O95727	Cytotoxic and regulatory T-cell molecule	rs186021206	7069412	17	A	G	0.006	0.627	0.049	1.3E-37	trans
569	CRTAM	O95727	Cytotoxic and regulatory T-cell molecule	rs2725405	79220224	17	C	G	0.441	0.050	0.007	2.5E-12	trans
570	CRYBB1	P53674	Beta-crystallin B1	rs1354034	56849749	3	C	T	0.604	0.063	0.008	1.1E-16	trans
570	CRYBB1	P53674	Beta-crystallin B1	rs9402686	135427817	6	A	G	0.271	0.070	0.008	6.2E-17	trans
570	CRYBB1	P53674	Beta-crystallin B1	rs342298	106373646	7	C	T	0.545	0.081	0.007	7.2E-28	trans
570	CRYBB1	P53674	Beta-crystallin B1	rs6993770	106581528	8	A	T	0.713	0.199	0.008	4.1E-129	trans
570	CRYBB1	P53674	Beta-crystallin B1	rs4631576	100705210	9	T	C	0.361	0.066	0.008	1.2E-17	trans
570	CRYBB1	P53674	Beta-crystallin B1	rs4783183	85415465	16	C	G	0.126	0.088	0.011	2.0E-15	trans
570	CRYBB1	P53674	Beta-crystallin B1	rs4822754	27012894	22	A	G	0.472	0.157	0.007	1.1E-98	cis
571	CRYBB2	P43320	Beta-crystallin B2	rs327189	120542784	1	T	C	0.882	0.083	0.012	5.8E-12	trans
571	CRYBB2	P43320	Beta-crystallin B2	rs11586604	227183865	1	T	G	0.472	0.059	0.007	8.0E-16	trans
571	CRYBB2	P43320	Beta-crystallin B2	rs56257618	107020339	6	T	C	0.105	0.088	0.012	1.9E-13	trans
571	CRYBB2	P43320	Beta-crystallin B2	rs78247684	28388615	7	A	G	0.246	0.071	0.009	1.0E-16	trans
571	CRYBB2	P43320	Beta-crystallin B2	rs34481144	320836	11	C	T	0.498	0.076	0.008	5.8E-24	trans
571	CRYBB2	P43320	Beta-crystallin B2	rs6578313	3026934	11	C	T	0.795	0.063	0.009	5.2E-12	trans
571	CRYBB2	P43320	Beta-crystallin B2	rs523175	98680662	13	A	G	0.566	0.058	0.007	5.3E-15	trans
571	CRYBB2	P43320	Beta-crystallin B2	rs12050132	73276250	14	T	A	0.240	0.112	0.009	8.3E-39	trans
571	CRYBB2	P43320	Beta-crystallin B2	rs5742915	74336633	15	T	C	0.538	0.052	0.007	1.2E-12	trans
571	CRYBB2	P43320	Beta-crystallin B2	rs7242807	61084959	18	G	C	0.507	0.081	0.007	7.6E-29	trans
571	CRYBB2	P43320	Beta-crystallin B2	rs45506792	59066558	19	C	T	0.993	0.391	0.051	1.8E-14	trans
571	CRYBB2	P43320	Beta-crystallin B2	rs56084239	32387102	20	T	C	0.172	0.358	0.010	4.2E-286	trans
572	CRYGD	P07320	Gamma-crystallin D	rs201408120	46768864	1	G	C	0.979	0.154	0.023	1.0E-11	trans
572	CRYGD	P07320	Gamma-crystallin D	rs1434282	199010721	1	T	C	0.726	0.133	0.007	4.9E-79	trans
572	CRYGD	P07320	Gamma-crystallin D	rs34570682	208978937	2	G	A	0.690	0.710	0.008	0.0E+00	cis
572	CRYGD	P07320	Gamma-crystallin D	rs72627509	57839051	4	G	C	0.184	0.075	0.008	2.0E-20	trans
572	CRYGD	P07320	Gamma-crystallin D	rs9374080	109616420	6	C	T	0.457	0.058	0.006	3.0E-20	trans
572	CRYGD	P07320	Gamma-crystallin D	rs60757417	135864436	9	C	G	0.940	0.134	0.013	3.5E-24	trans
572	CRYGD	P07320	Gamma-crystallin D	rs10883359	101274033	10	G	A	0.285	0.049	0.007	1.9E-12	trans
572	CRYGD	P07320	Gamma-crystallin D	rs2295125	23755607	14	A	G	0.290	0.053	0.007	1.2E-14	trans
572	CRYGD	P07320	Gamma-crystallin D	rs7256400	53411448	19	G	A	0.782	0.084	0.008	6.1E-29	trans
573	CRYM	Q14894	Ketimine reductase mu-crystallin	rs2733910	21285281	16	C	T	0.743	0.415	0.009	0.0E+00	cis
574	CRYZL1	O95825	Quinone oxidoreductase-like protein 1	rs1354034	56849749	3	C	T	0.605	0.087	0.008	8.2E-30	trans
574	CRYZL1	O95825	Quinone oxidoreductase-like protein 1	rs6993770	106581528	8	A	T	0.713	0.058	0.008	3.4E-12	trans
574	CRYZL1	O95825	Quinone oxidoreductase-like protein 1	rs7896518	65104500	10	G	A	0.424	0.067	0.008	5.3E-18	trans
574	CRYZL1	O95825	Quinone oxidoreductase-like protein 1	rs11502185	180258	11	C	T	0.260	0.068	0.010	2.0E-12	trans
574	CRYZL1	O95825	Quinone oxidoreductase-like protein 1	rs13050238	34997018	21	C	T	0.968	0.370	0.021	1.6E-66	cis
575	CSDE1	O75534	Cold shock domain-containing protein E1	rs1354034	56849749	3	C	T	0.604	0.073	0.008	4.0E-21	trans
575	CSDE1	O75534	Cold shock domain-containing protein E1	rs607929	32586998	6	C	G	0.551	0.096	0.008	3.3E-35	trans
576	CSF1	P09603	Macrophage colony-stimulating factor 1	rs11577194	110500175	1	T	C	0.476	0.187	0.007	7.5E-138	cis
576	CSF1	P09603	Macrophage colony-stimulating factor 1	rs17315646	230295307	1	G	C	0.606	0.065	0.008	1.7E-17	trans
576	CSF1	P09603	Macrophage colony-stimulating factor 1	rs3132455	31487954	6	C	G	0.348	0.060	0.008	6.7E-15	trans
576	CSF1	P09603	Macrophage colony-stimulating factor 1	rs423135	89740873	16	A	G	0.519	0.055	0.007	1.4E-13	trans
577	CSF1R	P07333	Macrophage colony-stimulating factor 1 receptor	rs79412885	9241839	1	G	A	0.926	0.130	0.014	8.0E-21	trans
577	CSF1R	P07333	Macrophage colony-stimulating factor 1 receptor	rs11899659	99363790	2	A	G	0.594	0.052	0.007	1.1E-12	trans
577	CSF1R	P07333	Macrophage colony-stimulating factor 1 receptor	rs62165726	134966562	2	C	A	0.963	0.381	0.019	1.8E-89	trans
577	CSF1R	P07333	Macrophage colony-stimulating factor 1 receptor	rs111772691	203608349	2	CAAAT	C	0.348	0.056	0.008	2.3E-13	trans
577	CSF1R	P07333	Macrophage colony-stimulating factor 1 receptor	rs2470750	98690592	3	A	T	0.607	0.131	0.007	4.9E-71	trans
577	CSF1R	P07333	Macrophage colony-stimulating factor 1 receptor	rs2293329	143103785	3	A	G	0.826	0.087	0.009	3.1E-20	trans
577	CSF1R	P07333	Macrophage colony-stimulating factor 1 receptor	rs35829610	131823862	5	C	CTG	0.662	0.053	0.008	5.8E-12	trans
577	CSF1R	P07333	Macrophage colony-stimulating factor 1 receptor	rs56048668	149460542	5	A	C	0.993	1.538	0.044	2.8E-262	cis
577	CSF1R	P07333	Macrophage colony-stimulating factor 1 receptor	rs1281944	32597379	6	C	T	0.130	0.090	0.011	9.0E-16	trans
577	CSF1R	P07333	Macrophage colony-stimulating factor 1 receptor	rs4841132	9183596	8	A	G	0.092	0.093	0.012	4.3E-14	trans
577	CSF1R	P07333	Macrophage colony-stimulating factor 1 receptor	rs7865362	33117965	9	T	C	0.363	0.086	0.008	1.8E-30	trans
577	CSF1R	P07333	Macrophage colony-stimulating factor 1 receptor	rs635634	136155000	9	T	C	0.184	0.122	0.009	1.2E-39	trans
577	CSF1R	P07333	Macrophage colony-stimulating factor 1 receptor	rs75071241	126232186	11	G	A	0.940	0.168	0.015	4.9E-29	trans
577	CSF1R	P07333	Macrophage colony-stimulating factor 1 receptor	rs186021206	7069412	17	A	G	0.006	1.563	0.049	2.3E-220	trans
577	CSF1R	P07333	Macrophage colony-stimulating factor 1 receptor	rs61267327	74473212	17	C	T	0.975	0.161	0.023	2.1E-12	trans
577	CSF1R	P07333	Macrophage colony-stimulating factor 1 receptor	rs516316	49206145	19	C	G	0.509	0.055	0.007	2.4E-14	trans
577	CSF1R	P07333	Macrophage colony-stimulating factor 1 receptor	rs738409	44324727	22	G	C	0.217	0.066	0.009	2.1E-14	trans
578	CSF2	P04141	Granulocyte-macrophage colony-stimulating factor	rs2009581	111807677	2	G	A	0.727	0.086	0.009	5.7E-24	trans
578	CSF2	P04141	Granulocyte-macrophage colony-stimulating factor	rs25882	131411460	5	C	T	0.207	0.244	0.009	7.4E-149	cis
578	CSF2	P04141	Granulocyte-macrophage colony-stimulating factor	rs9270604	32564800	6	C	T	0.319	0.063	0.008	2.2E-14	trans
578	CSF2	P04141	Granulocyte-macrophage colony-stimulating factor	rs2967683	8785951	19	T	C	0.763	0.071	0.009	9.3E-16	trans
579	CSF2RA	P15509	Granulocyte-macrophage colony-stimulating factor receptor subunit alpha	rs9394107	32620720	6	G	A	0.230	0.069	0.010	1.2E-11	trans
579	CSF2RA	P15509	Granulocyte-macrophage colony-stimulating factor receptor subunit alpha	rs200489612	7106378	17	A	G	0.005	0.541	0.058	5.7E-21	trans
580	CSF2RB	P32927	Cytokine receptor common subunit beta	rs8176741	136131461	9	A	G	0.061	0.098	0.010	1.4E-22	trans
580	CSF2RB	P32927	Cytokine receptor common subunit beta	rs200489612	7106378	17	A	G	0.005	0.302	0.036	5.8E-17	trans
580	CSF2RB	P32927	Cytokine receptor common subunit beta	rs11655264	38138995	17	G	C	0.549	0.041	0.005	1.1E-17	trans
580	CSF2RB	P32927	Cytokine receptor common subunit beta	rs2239749	37331305	22	A	G	0.541	1.122	0.008	0.0E+00	cis
581	CSF3	P09919	Granulocyte colony-stimulating factor	rs3917932	36943916	1	C	G	0.425	0.174	0.008	7.3E-116	trans
581	CSF3	P09919	Granulocyte colony-stimulating factor	rs41348	28716848	7	A	G	0.582	0.053	0.008	4.5E-12	trans
581	CSF3	P09919	Granulocyte colony-stimulating factor	rs14408	308314	11	T	C	0.634	0.069	0.008	1.3E-18	trans
582	CSF3R	Q99062	Granulocyte colony-stimulating factor receptor	rs3917999	36933317	1	T	C	0.011	1.132	0.038	1.9E-199	cis
582	CSF3R	Q99062	Granulocyte colony-stimulating factor receptor	rs11888817	62530980	2	C	T	0.642	0.056	0.008	6.2E-13	trans
582	CSF3R	Q99062	Granulocyte colony-stimulating factor receptor	rs2853951	31236115	6	T	C	0.589	0.066	0.008	6.6E-18	trans
582	CSF3R	Q99062	Granulocyte colony-stimulating factor receptor	rs445	92408370	7	C	T	0.905	0.093	0.013	2.4E-13	trans
582	CSF3R	Q99062	Granulocyte colony-stimulating factor receptor	rs10107630	130603635	8	C	T	0.426	0.062	0.008	3.5E-16	trans
582	CSF3R	Q99062	Granulocyte colony-stimulating factor receptor	rs240556	126193807	11	T	C	0.239	0.146	0.009	6.3E-61	trans
582	CSF3R	Q99062	Granulocyte colony-stimulating factor receptor	rs186021206	7069412	17	A	G	0.006	0.657	0.052	1.0E-36	trans
582	CSF3R	Q99062	Granulocyte colony-stimulating factor receptor	rs3826331	38150492	17	T	C	0.402	0.128	0.008	1.0E-62	trans
583	CSNK1D	P48730	Casein kinase I isoform delta	rs9273369	32626484	6	T	C	0.854	0.095	0.011	3.0E-18	trans
584	CSNK2A1	P68400	Casein kinase II subunit alpha	rs1354034	56849749	3	C	T	0.604	0.053	0.008	3.5E-12	trans
584	CSNK2A1	P68400	Casein kinase II subunit alpha	rs7896518	65104500	10	G	A	0.424	0.055	0.008	4.8E-13	trans
584	CSNK2A1	P68400	Casein kinase II subunit alpha	rs61269268	92241352	14	C	A	0.173	0.135	0.010	2.3E-42	trans
584	CSNK2A1	P68400	Casein kinase II subunit alpha	rs429358	45411941	19	C	T	0.156	0.214	0.010	1.6E-94	trans
585	CSPG4	Q6UVK1	Chondroitin sulfate proteoglycan 4	rs333947	110470764	1	G	A	0.852	0.085	0.010	1.1E-17	trans
585	CSPG4	Q6UVK1	Chondroitin sulfate proteoglycan 4	rs13303	52558008	3	C	T	0.560	0.103	0.007	3.4E-47	trans
585	CSPG4	Q6UVK1	Chondroitin sulfate proteoglycan 4	rs76967933	132406895	3	C	A	0.913	0.090	0.013	6.7E-13	trans
585	CSPG4	Q6UVK1	Chondroitin sulfate proteoglycan 4	rs1188865	139234628	6	G	C	0.181	0.062	0.009	1.5E-11	trans
585	CSPG4	Q6UVK1	Chondroitin sulfate proteoglycan 4	rs2389858	17908130	7	T	A	0.510	0.073	0.007	5.3E-25	trans
585	CSPG4	Q6UVK1	Chondroitin sulfate proteoglycan 4	rs56278466	17875857	10	G	T	0.661	0.061	0.007	1.5E-16	trans
585	CSPG4	Q6UVK1	Chondroitin sulfate proteoglycan 4	rs7318906	74106131	13	A	G	0.429	0.056	0.007	8.7E-15	trans
585	CSPG4	Q6UVK1	Chondroitin sulfate proteoglycan 4	rs11854097	75995221	15	A	G	0.744	0.134	0.008	1.4E-61	cis
585	CSPG4	Q6UVK1	Chondroitin sulfate proteoglycan 4	rs200489612	7106378	17	A	G	0.005	0.472	0.053	5.6E-19	trans
586	CSRP3	P50461	Cysteine and glycine-rich protein 3	rs4408310	22221240	11	A	C	0.540	0.057	0.008	1.5E-13	trans
587	CST1	P01037	Cystatin-SN	rs72694438	104364878	1	G	A	0.793	0.075	0.009	2.9E-17	trans
587	CST1	P01037	Cystatin-SN	rs61685000	202181185	1	G	C	0.591	0.102	0.007	7.0E-45	trans
587	CST1	P01037	Cystatin-SN	rs4976271	134511366	5	G	T	0.403	0.078	0.007	2.0E-26	trans
587	CST1	P01037	Cystatin-SN	rs745377	34858413	11	T	G	0.523	0.069	0.007	3.8E-22	trans
587	CST1	P01037	Cystatin-SN	rs1998764	37230561	14	T	C	0.559	0.064	0.007	4.5E-19	trans
587	CST1	P01037	Cystatin-SN	rs67020211	63639644	15	T	G	0.446	0.085	0.007	1.2E-32	trans
587	CST1	P01037	Cystatin-SN	rs601338	49206674	19	A	G	0.509	0.067	0.007	1.1E-20	trans
587	CST1	P01037	Cystatin-SN	rs4549181	23728873	20	C	T	0.700	0.583	0.009	0.0E+00	cis
588	CST3	P01034	Cystatin-C	rs13146355	77412140	4	A	G	0.457	0.065	0.007	4.1E-21	trans
588	CST3	P01034	Cystatin-C	rs77924615	20392332	16	G	A	0.803	0.062	0.009	1.8E-12	trans
588	CST3	P01034	Cystatin-C	rs911119	23612737	20	T	C	0.777	0.328	0.008	0.0E+00	cis
589	CST5	P28325	Cystatin-D	rs72694438	104364878	1	G	A	0.793	0.052	0.007	4.3E-12	trans
589	CST5	P28325	Cystatin-D	rs4950775	202192423	1	C	T	0.582	0.050	0.006	2.2E-16	trans
589	CST5	P28325	Cystatin-D	rs34639880	207124079	1	GT	G	0.455	0.060	0.006	4.5E-23	trans
589	CST5	P28325	Cystatin-D	rs2576233	216991932	1	T	G	0.244	0.051	0.007	6.1E-13	trans
589	CST5	P28325	Cystatin-D	rs3820640	226868918	1	C	T	0.159	0.058	0.008	1.7E-12	trans
589	CST5	P28325	Cystatin-D	rs7428376	18857812	3	T	C	0.770	0.052	0.007	4.6E-13	trans
589	CST5	P28325	Cystatin-D	rs17637318	23880985	4	T	C	0.708	0.055	0.007	6.8E-17	trans
589	CST5	P28325	Cystatin-D	rs4976271	134511366	5	G	T	0.403	0.066	0.006	1.5E-26	trans
589	CST5	P28325	Cystatin-D	rs745377	34858413	11	T	G	0.524	0.053	0.006	2.5E-18	trans
589	CST5	P28325	Cystatin-D	rs67020211	63639644	15	T	G	0.446	0.056	0.006	2.3E-20	trans
589	CST5	P28325	Cystatin-D	rs601338	49206674	19	A	G	0.509	0.097	0.006	5.8E-58	trans
589	CST5	P28325	Cystatin-D	rs1799841	23860178	20	G	A	0.436	0.731	0.007	0.0E+00	cis
590	CST6	Q15828	Cystatin-M	rs80293268	8207579	1	G	C	0.952	0.387	0.018	5.3E-107	trans
590	CST6	Q15828	Cystatin-M	rs28635651	218856502	1	G	A	0.330	0.067	0.008	1.3E-18	trans
590	CST6	Q15828	Cystatin-M	rs1260326	27730940	2	C	T	0.608	0.059	0.007	9.5E-16	trans
590	CST6	Q15828	Cystatin-M	rs13015258	162930725	2	G	T	0.621	0.056	0.007	6.5E-14	trans
590	CST6	Q15828	Cystatin-M	rs121908120	219755011	2	T	A	0.972	0.191	0.022	6.2E-18	trans
590	CST6	Q15828	Cystatin-M	rs833270	181520166	3	C	T	0.617	0.050	0.007	9.5E-12	trans
590	CST6	Q15828	Cystatin-M	rs75680898	107786548	4	G	A	0.145	0.071	0.010	3.6E-12	trans

590	CST6	Q15828	Cystatin-M	rs114964313	78433572	5	A	G	0.319	0.062	0.008	1.3E-14	trans
590	CST6	Q15828	Cystatin-M	rs155942	139532538	5	T	C	0.298	0.059	0.008	4.5E-14	trans
590	CST6	Q15828	Cystatin-M	rs7905367	54334653	10	C	G	0.798	0.086	0.009	1.5E-22	trans
590	CST6	Q15828	Cystatin-M	rs12577165	65776429	11	C	T	0.024	0.613	0.024	1.4E-148	cis
590	CST6	Q15828	Cystatin-M	rs36034702	90734627	15	C	T	0.831	0.114	0.010	5.6E-32	trans
591	CST7	O76096	Cystatin-F	rs114694170	88180196	5	C	T	0.060	0.123	0.012	4.0E-23	trans
591	CST7	O76096	Cystatin-F	rs6993770	106581528	8	A	T	0.713	0.152	0.006	7.3E-122	trans
591	CST7	O76096	Cystatin-F	rs7098181	65027143	10	G	T	0.586	0.065	0.006	3.5E-28	trans
591	CST7	O76096	Cystatin-F	rs12767683	104316581	10	A	C	0.325	0.086	0.006	1.3E-43	trans
591	CST7	O76096	Cystatin-F	rs2511241	72945341	11	T	C	0.929	0.089	0.011	4.3E-15	trans
591	CST7	O76096	Cystatin-F	rs145078947	93652974	14	T	G	0.003	0.420	0.058	2.9E-13	trans
591	CST7	O76096	Cystatin-F	rs4783183	85415465	16	C	G	0.126	0.079	0.009	1.5E-19	trans
591	CST7	O76096	Cystatin-F	rs1045929	38175426	17	T	C	0.388	0.077	0.006	4.3E-38	trans
591	CST7	O76096	Cystatin-F	rs227651	24929834	20	A	G	0.769	0.836	0.009	0.0E+00	cis
592	CTBS	Q01459	Di-N-acetylchitobiase	rs114165349	27021913	1	C	G	0.023	0.226	0.024	8.8E-21	trans
592	CTBS	Q01459	Di-N-acetylchitobiase	rs79723172	85012695	1	A	G	0.030	1.378	0.021	0.0E+00	cis
592	CTBS	Q01459	Di-N-acetylchitobiase	rs687339	135932359	3	T	C	0.771	0.155	0.008	9.8E-84	trans
592	CTBS	Q01459	Di-N-acetylchitobiase	rs9857914	194063611	3	A	G	0.279	0.051	0.007	7.8E-12	trans
592	CTBS	Q01459	Di-N-acetylchitobiase	rs72729623	154208278	4	C	T	0.853	0.074	0.009	4.1E-15	trans
592	CTBS	Q01459	Di-N-acetylchitobiase	rs2169387	9181395	8	A	G	0.101	0.085	0.011	1.9E-14	trans
592	CTBS	Q01459	Di-N-acetylchitobiase	rs145078947	93652974	14	T	G	0.003	0.687	0.066	4.7E-25	trans
592	CTBS	Q01459	Di-N-acetylchitobiase	rs139974673	44027885	15	C	T	0.024	0.276	0.022	1.7E-37	trans
592	CTBS	Q01459	Di-N-acetylchitobiase	rs58542926	19379549	19	T	C	0.075	0.111	0.013	1.0E-18	trans
592	CTBS	Q01459	Di-N-acetylchitobiase	rs429358	45411941	19	T	C	0.844	0.062	0.009	1.5E-11	trans
592	CTBS	Q01459	Di-N-acetylchitobiase	rs1800961	43042364	20	T	C	0.032	0.165	0.019	1.1E-18	trans
593	CTF1	Q16619	Cardiotrophin-1	rs342298	106373646	7	C	T	0.545	0.054	0.008	1.9E-12	trans
594	CTHRC1	Q96CG8	Collagen triple helix repeat-containing protein 1	rs827592	104364637	8	C	G	0.456	0.162	0.007	1.1E-124	cis
594	CTHRC1	Q96CG8	Collagen triple helix repeat-containing protein 1	rs1562782	10342711	11	A	G	0.591	0.046	0.007	1.5E-11	trans
595	CTRB1	P17538	Chymotrypsinogen B	rs11759956	7109097	6	T	C	0.549	0.050	0.007	7.1E-13	trans
595	CTRB1	P17538	Chymotrypsinogen B	rs3132568	31101917	6	A	G	0.744	0.057	0.008	1.2E-12	trans
595	CTRB1	P17538	Chymotrypsinogen B	rs7766106	127455138	6	T	C	0.495	0.074	0.007	1.7E-26	trans
595	CTRB1	P17538	Chymotrypsinogen B	rs2329566	51017655	7	A	G	0.954	0.151	0.017	7.3E-20	trans
595	CTRB1	P17538	Chymotrypsinogen B	rs6666	142460313	7	T	C	0.421	0.054	0.007	1.5E-14	trans
595	CTRB1	P17538	Chymotrypsinogen B	rs4921967	18719629	8	G	T	0.313	0.068	0.007	1.0E-19	trans
595	CTRB1	P17538	Chymotrypsinogen B	rs2519093	136141870	9	C	T	0.816	0.100	0.009	4.9E-29	trans
595	CTRB1	P17538	Chymotrypsinogen B	rs4751995	118397884	10	A	G	0.481	0.047	0.007	1.1E-11	trans
595	CTRB1	P17538	Chymotrypsinogen B	rs174544	61567753	11	A	C	0.309	0.067	0.007	2.7E-19	trans
595	CTRB1	P17538	Chymotrypsinogen B	rs61910478	100592308	11	A	T	0.731	0.057	0.008	1.8E-13	trans
595	CTRB1	P17538	Chymotrypsinogen B	rs8051363	75255217	16	G	A	0.715	0.574	0.008	0.0E+00	cis
595	CTRB1	P17538	Chymotrypsinogen B	rs1126464	89704365	16	G	C	0.756	0.086	0.009	1.6E-22	trans
595	CTRB1	P17538	Chymotrypsinogen B	rs17138478	36073320	17	A	C	0.130	0.070	0.010	1.1E-11	trans
596	CTRC	Q99895	Chymotrypsin-C	rs497078	15767036	1	C	T	0.902	0.569	0.012	0.0E+00	cis
596	CTRC	Q99895	Chymotrypsin-C	rs6571015	96534478	6	A	G	0.791	0.205	0.009	1.7E-127	trans
596	CTRC	Q99895	Chymotrypsin-C	rs112166936	126733140	6	A	C	0.550	0.078	0.007	4.6E-29	trans
596	CTRC	Q99895	Chymotrypsin-C	rs11778310	18774902	8	G	A	0.294	0.064	0.008	4.3E-17	trans
596	CTRC	Q99895	Chymotrypsin-C	rs8176743	136131415	9	T	C	0.061	0.885	0.015	0.0E+00	trans
596	CTRC	Q99895	Chymotrypsin-C	rs56278466	17875857	10	G	T	0.661	0.060	0.007	1.8E-16	trans
596	CTRC	Q99895	Chymotrypsin-C	rs9581943	28493997	13	A	G	0.404	0.049	0.007	2.8E-12	trans
596	CTRC	Q99895	Chymotrypsin-C	rs35775091	21275985	14	A	G	0.274	0.061	0.008	2.1E-15	trans
596	CTRC	Q99895	Chymotrypsin-C	rs72802342	75234872	16	C	A	0.924	0.238	0.013	1.0E-71	trans
596	CTRC	Q99895	Chymotrypsin-C	rs529900	88976968	16	G	C	0.622	0.099	0.007	5.7E-44	trans
596	CTRC	Q99895	Chymotrypsin-C	rs200489612	7106378	17	A	G	0.005	0.521	0.052	6.4E-24	trans
596	CTRC	Q99895	Chymotrypsin-C	rs601338	49206674	19	A	G	0.510	0.110	0.007	9.8E-57	trans
597	CTRL	P40313	Chymotrypsin-like protease CTRL-1	rs61200828	22314444	1	A	C	0.102	0.133	0.012	3.2E-28	trans
597	CTRL	P40313	Chymotrypsin-like protease CTRL-1	rs2816941	199990779	1	G	A	0.228	0.094	0.009	1.7E-27	trans
597	CTRL	P40313	Chymotrypsin-like protease CTRL-1	rs1260326	27730940	2	C	T	0.608	0.062	0.007	4.1E-17	trans
597	CTRL	P40313	Chymotrypsin-like protease CTRL-1	rs11967891	32500957	6	A	T	0.816	0.075	0.010	2.4E-14	trans
597	CTRL	P40313	Chymotrypsin-like protease CTRL-1	rs6571015	96534478	6	A	G	0.791	0.132	0.009	6.1E-50	trans
597	CTRL	P40313	Chymotrypsin-like protease CTRL-1	rs1578060	126712247	6	G	C	0.544	0.062	0.007	1.6E-17	trans
597	CTRL	P40313	Chymotrypsin-like protease CTRL-1	rs3812304	158871744	7	G	A	0.768	0.068	0.009	1.8E-15	trans
597	CTRL	P40313	Chymotrypsin-like protease CTRL-1	rs608337	102511718	8	G	A	0.069	0.170	0.014	1.2E-32	trans
597	CTRL	P40313	Chymotrypsin-like protease CTRL-1	rs8176743	136131415	9	T	C	0.062	0.493	0.015	4.7E-232	trans
597	CTRL	P40313	Chymotrypsin-like protease CTRL-1	rs56278466	17875857	10	G	T	0.661	0.060	0.008	2.7E-15	trans
597	CTRL	P40313	Chymotrypsin-like protease CTRL-1	rs1946515	49336477	10	A	T	0.376	0.062	0.008	1.8E-13	trans
597	CTRL	P40313	Chymotrypsin-like protease CTRL-1	rs7142320	21273751	14	A	C	0.273	0.057	0.008	3.5E-12	trans
597	CTRL	P40313	Chymotrypsin-like protease CTRL-1	rs28929474	94844947	14	C	T	0.979	0.943	0.026	1.7E-293	trans
597	CTRL	P40313	Chymotrypsin-like protease CTRL-1	rs4983549	105228216	14	A	G	0.321	0.106	0.008	6.6E-42	trans
597	CTRL	P40313	Chymotrypsin-like protease CTRL-1	rs20549	67969930	16	G	A	0.167	0.089	0.010	9.4E-20	cis
597	CTRL	P40313	Chymotrypsin-like protease CTRL-1	rs8051363	75255217	16	G	A	0.715	0.224	0.008	2.0E-166	trans
597	CTRL	P40313	Chymotrypsin-like protease CTRL-1	rs529900	88976968	16	G	C	0.621	0.082	0.008	5.6E-28	trans
597	CTRL	P40313	Chymotrypsin-like protease CTRL-1	rs6501457	69209252	17	T	C	0.775	0.090	0.009	2.6E-25	trans
597	CTRL	P40313	Chymotrypsin-like protease CTRL-1	rs681343	49206462	19	T	C	0.509	0.137	0.007	2.1E-78	trans
597	CTRL	P40313	Chymotrypsin-like protease CTRL-1	rs6020369	48835972	20	T	C	0.534	0.050	0.007	3.3E-12	trans
598	CTSB	P07858	Cathepsin B	rs4713570	32626040	6	T	C	0.266	0.068	0.008	4.1E-18	trans
598	CTSB	P07858	Cathepsin B	rs709821	11702594	8	C	G	0.262	0.591	0.009	0.0E+00	cis
598	CTSB	P07858	Cathepsin B	rs10740131	65271488	10	T	A	0.470	0.048	0.007	2.4E-12	trans
598	CTSB	P07858	Cathepsin B	rs41281340	73571619	10	C	G	0.906	0.128	0.012	1.2E-27	trans
598	CTSB	P07858	Cathepsin B	rs145078947	93652974	14	T	G	0.003	0.531	0.069	1.0E-14	trans
598	CTSB	P07858	Cathepsin B	rs8107974	19388500	19	T	A	0.077	0.127	0.013	1.6E-22	trans
599	CTSC	P53634	Dipeptidyl peptidase 1	rs2854275	32628428	6	A	C	0.147	0.118	0.009	1.6E-41	trans
599	CTSC	P53634	Dipeptidyl peptidase 1	rs9457800	160411747	6	C	T	0.865	0.072	0.009	2.0E-15	trans
599	CTSC	P53634	Dipeptidyl peptidase 1	rs10822143	64887856	10	T	C	0.501	0.045	0.006	2.0E-13	trans
599	CTSC	P53634	Dipeptidyl peptidase 1	rs11600158	88070914	11	G	A	0.098	0.989	0.013	0.0E+00	cis
599	CTSC	P53634	Dipeptidyl peptidase 1	rs145078947	93652974	14	T	G	0.003	0.855	0.061	6.7E-45	trans
599	CTSC	P53634	Dipeptidyl peptidase 1	rs139974673	44027885	15	C	T	0.024	0.136	0.020	9.0E-12	trans
600	CTSD	P07339	Cathepsin D	rs2298632	23710475	1	C	T	0.500	0.051	0.007	2.2E-12	trans
600	CTSD	P07339	Cathepsin D	rs1260326	27730940	2	T	C	0.392	0.070	0.007	2.6E-21	trans
600	CTSD	P07339	Cathepsin D	rs55861089	1783757	11	A	G	0.896	0.507	0.012	0.0E+00	cis
600	CTSD	P07339	Cathepsin D	rs145078947	93652974	14	T	G	0.003	0.491	0.071	5.3E-12	trans
600	CTSD	P07339	Cathepsin D	rs58542926	19379549	19	T	C	0.075	0.105	0.014	1.7E-14	trans
601	CTSE	P14091	Cathepsin E	rs2422141	172402127	1	C	G	0.511	0.076	0.008	5.8E-24	trans
601	CTSE	P14091	Cathepsin E	rs41302235	206328803	1	T	C	0.812	0.643	0.010	0.0E+00	cis
601	CTSE	P14091	Cathepsin E	rs7606173	60725451	2	G	C	0.568	0.111	0.007	1.4E-54	trans
601	CTSE	P14091	Cathepsin E	rs36124964	69354362	4	C	T	0.656	0.056	0.008	8.4E-14	trans
601	CTSE	P14091	Cathepsin E	rs535777	32577633	6	G	C	0.857	0.117	0.010	6.8E-30	trans
601	CTSE	P14091	Cathepsin E	rs2519093	136141870	9	C	T	0.815	0.269	0.009	1.1E-185	trans
601	CTSE	P14091	Cathepsin E	rs56278466	17875857	10	G	T	0.661	0.065	0.007	4.5E-18	trans
601	CTSE	P14091	Cathepsin E	rs681343	49206462	19	C	T	0.491	0.092	0.007	3.1E-38	trans
601	CTSE	P14091	Cathepsin E	rs200756900	107304707	X	G	A	0.004	0.897	0.049	2.3E-76	trans
602	CTSF	Q9UBX1	Cathepsin F	rs1260326	27730940	2	T	C	0.391	0.103	0.007	1.1E-44	trans
602	CTSF	Q9UBX1	Cathepsin F	rs59916403	242370751	2	G	T	0.651	0.054	0.007	5.2E-13	trans
602	CTSF	Q9UBX1	Cathepsin F	rs187325356	110266913	4	A	T	0.008	0.336	0.042	1.5E-15	trans
602	CTSF	Q9UBX1	Cathepsin F	rs115850161	119732447	4	T	C	0.939	0.178	0.015	4.6E-32	trans
602	CTSF	Q9UBX1	Cathepsin F	rs41280463	154191226	4	G	A	0.832	0.066	0.010	5.0E-12	trans
602	CTSF	Q9UBX1	Cathepsin F	rs17145750	73026378	7	C	T	0.840	0.071	0.010	3.3E-13	trans
602	CTSF	Q9UBX1	Cathepsin F	rs2001945	126477978	8	G	C	0.481	0.051	0.007	1.1E-12	trans
602	CTSF	Q9UBX1	Cathepsin F	rs102275	61557803	11	T	C	0.649	0.057	0.008	2.1E-14	trans
602	CTSF	Q9UBX1	Cathepsin F	rs1044522	66335832	11	A	G	0.241	0.217	0.008	2.2E-146	cis
602	CTSF	Q9UBX1	Cathepsin F	rs145078947	93652974	14	T	G	0.003	0.780	0.071	4.8E-28	trans
602	CTSF	Q9UBX1	Cathepsin F	rs139974673	44027885	15	C	T	0.024	0.242	0.023	8.5E-26	trans
602	CTSF	Q9UBX1	Cathepsin F	rs429358	45411941	19	T	C	0.844	0.077	0.010	4.9E-15	trans
603	CTSL	P07711	Cathepsin L1	rs144274836	186422367	3	C	T	0.886	0.092	0.011	1.6E-15	trans
603	CTSL	P07711	Cathepsin L1	rs3129938	32336495	6	T	A	0.166	0.085	0.010	4.1E-18	trans
603	CTSL	P07711	Cathepsin L1	rs9497576	146925676	6	T	G	0.075	0.106	0.014	1.9E-14	trans
603	CTSL	P07711	Cathepsin L1	rs17151689	76950686	7	C	A	0.954	0.130	0.017	4.3E-14	trans
603	CTSL	P07711	Cathepsin L1	rs2282240	27572634	9	T	C	0.252	0.091	0.008	4.8E-27	trans
603	CTSL	P07711	Cathepsin L1	rs868561	90349202	9	G	C	0.496	0.148	0.007	2.6E-92	cis
603	CTSL	P07711	Cathepsin L1	rs150370599	60718792	11	T	C	0.081	0.210	0.013	1.8E-56	trans
603	CTSL	P07711	Cathepsin L1	rs140735783	95100401	14	T	TC	0.816	0.107	0.010	3.9E-29	trans
603	CTSL	P07711	Cathepsin L1	rs8176396	103601138	14	C	T	0.976	0.161	0.024	1.3E-11	trans
603	CTSL	P07711	Cathepsin L1	rs9901675	7484812	17	G	A	0.950	0.140	0.016	2.1E-17	trans
603	CTSL	P07711	Cathepsin L1	rs77733715	3537184	19	G	A	0.009	0.303	0.038	3.2E-15	trans
603	CTSL	P07711	Cathepsin L1	rs10469365	12731874	19	A	G	0.891	0.138	0.012	2.3E-32	trans
603	CTSL	P07711	Cathepsin L1	rs200373	18286546	19	A	T	0.489	0.130	0.007	1.1E-70	trans
603	CTSL	P07711	Cathepsin L1	rs1065853	45413233	19	T	G	0.081	0.095	0.013	1.3E-12	trans
604	CTSO	P43234	Cathepsin O	rs583104	109821307	1	T	G	0.773	0.074	0.009	1.7E-17	trans
604	CTSO	P43234	Cathepsin O	rs687339	135932359	3	T	C	0.771	0.088	0.009	6.4E-24	trans
604	CTSO	P43234	Cathepsin O	rs2334114	156856193	4	C	A	0.787	0.218	0.009	1.5E-128	cis
604	CTSO	P43234	Cathepsin O	rs116105128	32511456	6	G	C	0.881	0.101	0.012	5.4E-16	trans
604	CTSO	P43234	Cathepsin O	rs75474551	160409894	6	G	T	0.907	0.244	0.013	1.0E-83	trans
604	CTSO	P43234	Cathepsin O	rs56278466	17875857	10	G	T	0.661	0.055	0.008	7.0E-13	trans
604	CTSO	P43234	Cathepsin O	rs174560	61581764	11	C	T	0.315	0.067	0.008	1.2E-17	trans
604	CTSO	P43234	Cathepsin O	rs58542926	19379549	19	T	C	0.075	0.254	0.014	1.6E-75	trans
604	CTSO	P43234	Cathepsin O	rs738409	44324727	22	G	C	0.217	0.063	0.009	8.1E-13	trans
605	CTSS	P25774	Cathepsin S	rs9365125	160450402	6	C	T	0.141	0.098	0.011	1.6E-18	trans
605	CTSS	P25774	Cathepsin S	rs79751758	101256377	10	A	C	0.106	0.094	0.013	6.4E-14	trans
605	CTSS	P25774	Cathepsin S	rs3829241	68855363	11	G	A	0.594	0.060	0.008	2.7E-14	trans
606	CTSV	O60911	Cathepsin L2	rs113292219	29486128	1	T	C	0.053	0.106	0.015	8.9E-12	trans
606	CTSV	O60911	Cathepsin L2	rs13407583	113967740	2	A	G	0.210	0.063	0.009	1.9E-13	trans
606	CTSV	O60911	Cathepsin L2	rs28493806	106106709	4	G	A	0.371	0.050	0.007	3.2E-12	trans
606	CTSV	O60911	Cathepsin L2	rs35716097	176806636	5	T	C	0.296	0.073	0.008	6.6E-22	trans
606	CTSV	O60911	Cathepsin L2	rs260237	42392578	6	C	T	0.888	0.090	0.011	3.0E-16	trans
606	CTSV	O60911	Cathepsin L2	rs9382146	52702085	6	G	A	0.426	0.112	0.007	3.7E-58	trans

606	CTSV	O60911	Cathepsin L2	rs12686511	99909044	9	T	C	0.207	0.257	0.009	4.1E-187	cis
606	CTSV	O60911	Cathepsin L2	rs532436	136149830	9	G	A	0.815	0.112	0.009	4.7E-35	trans
606	CTSV	O60911	Cathepsin L2	rs1427204	60267640	10	C	A	0.454	0.073	0.007	3.4E-26	trans
606	CTSV	O60911	Cathepsin L2	rs4077451	79931720	16	A	T	0.855	0.100	0.010	2.1E-24	trans
606	CTSV	O60911	Cathepsin L2	rs123698	807442	19	C	G	0.605	0.049	0.007	2.3E-12	trans
606	CTSV	O60911	Cathepsin L2	rs12019136	5835677	19	A	G	0.041	0.134	0.017	1.1E-14	trans
606	CTSV	O60911	Cathepsin L2	rs78581838	16236580	21	T	C	0.090	0.094	0.012	1.4E-14	trans
607	CTSZ	Q9UBR2	Cathepsin Z	rs41271951	150737220	1	A	G	0.915	0.110	0.013	7.1E-18	trans
607	CTSZ	Q9UBR2	Cathepsin Z	rs687339	135932359	3	T	C	0.771	0.075	0.009	2.0E-18	trans
607	CTSZ	Q9UBR2	Cathepsin Z	rs3021302	32623150	6	C	T	0.168	0.069	0.009	3.9E-13	trans
607	CTSZ	Q9UBR2	Cathepsin Z	rs76778371	160418050	6	G	A	0.907	0.083	0.012	1.4E-11	trans
607	CTSZ	Q9UBR2	Cathepsin Z	rs150641790	41687844	13	TG	T	0.075	0.097	0.014	1.3E-12	trans
607	CTSZ	Q9UBR2	Cathepsin Z	rs145078947	93652974	14	T	G	0.002	0.773	0.071	2.3E-27	trans
607	CTSZ	Q9UBR2	Cathepsin Z	rs200210321	19393890	19	AG	A	0.074	0.169	0.014	5.8E-35	trans
607	CTSZ	Q9UBR2	Cathepsin Z	rs429358	45411941	19	T	C	0.844	0.087	0.010	8.2E-19	trans
607	CTSZ	Q9UBR2	Cathepsin Z	rs199825459	57572709	20	A	G	0.992	1.954	0.040	0.0E+00	cis
608	CUZD1	Q86UP6	CUB and zona pellucida-like domain-containing protein 1	rs6571015	96534478	6	A	G	0.791	0.123	0.009	2.9E-41	trans
608	CUZD1	Q86UP6	CUB and zona pellucida-like domain-containing protein 1	rs708686	5840619	19	T	C	0.268	0.080	0.008	2.7E-21	trans
609	CWC15	Q9P013	Spliceosome-associated protein CWC15 homolog	rs16921241	94707809	11	A	G	0.318	0.058	0.008	8.8E-13	cis
610	CX3CL1	P78423	Fractalkine	rs114921587	21671973	1	A	G	0.097	0.093	0.013	2.5E-13	trans
610	CX3CL1	P78423	Fractalkine	rs3732378	39307162	3	A	G	0.175	0.084	0.010	1.0E-17	trans
610	CX3CL1	P78423	Fractalkine	rs9264666	31239296	6	G	C	0.445	0.089	0.007	1.1E-32	trans
610	CX3CL1	P78423	Fractalkine	rs502417	79104873	9	C	T	0.740	0.059	0.008	3.4E-12	trans
610	CX3CL1	P78423	Fractalkine	rs507666	136149399	9	G	A	0.815	0.124	0.010	4.3E-38	trans
610	CX3CL1	P78423	Fractalkine	rs9414801	65149089	10	G	A	0.467	0.055	0.007	1.9E-13	trans
610	CX3CL1	P78423	Fractalkine	rs602662	49206985	19	A	G	0.537	0.055	0.007	1.9E-13	trans
611	CXADR	P78310	Coxsackievirus and adenovirus receptor	rs6705147	133196926	2	C	T	0.670	0.058	0.008	1.6E-13	trans
611	CXADR	P78310	Coxsackievirus and adenovirus receptor	rs56398830	103701690	13	G	A	0.989	0.414	0.035	1.3E-32	trans
611	CXADR	P78310	Coxsackievirus and adenovirus receptor	rs12938714	28645110	17	A	G	0.330	0.086	0.008	1.9E-28	trans
611	CXADR	P78310	Coxsackievirus and adenovirus receptor	rs681343	49206462	19	C	T	0.491	0.177	0.007	4.8E-126	trans
611	CXADR	P78310	Coxsackievirus and adenovirus receptor	rs2849900	18936119	21	T	C	0.332	0.124	0.008	7.6E-56	cis
612	CXCL1	P09341	Growth-regulated alpha protein	rs12075	159175354	1	A	G	0.583	0.063	0.007	5.2E-20	trans
612	CXCL1	P09341	Growth-regulated alpha protein	rs3097411	74738454	4	G	C	0.236	0.674	0.009	0.0E+00	cis
612	CXCL1	P09341	Growth-regulated alpha protein	rs16870187	32498584	6	C	A	0.262	0.108	0.009	2.4E-33	trans
612	CXCL1	P09341	Growth-regulated alpha protein	rs7080386	65048306	10	A	C	0.412	0.090	0.007	3.5E-38	trans
613	CXCL10	P02778	C-X-C motif chemokine 10	rs113010081	46457412	3	T	C	0.881	0.081	0.011	6.0E-13	trans
613	CXCL10	P02778	C-X-C motif chemokine 10	rs11548618	76943947	4	A	G	0.007	1.730	0.044	0.0E+00	cis
613	CXCL10	P02778	C-X-C motif chemokine 10	rs28366364	32565532	6	C	G	0.598	0.064	0.007	1.0E-17	trans
614	CXCL11	O14625	C-X-C motif chemokine 11	rs76036957	113496453	2	A	G	0.009	0.278	0.038	5.1E-13	trans
614	CXCL11	O14625	C-X-C motif chemokine 11	rs35170645	76964956	4	A	G	0.486	0.224	0.007	2.8E-197	cis
614	CXCL11	O14625	C-X-C motif chemokine 11	rs13181561	138850905	5	A	G	0.745	0.063	0.008	1.2E-13	trans
614	CXCL11	O14625	C-X-C motif chemokine 11	rs11962818	31320176	6	C	T	0.841	0.083	0.010	5.3E-16	trans
614	CXCL11	O14625	C-X-C motif chemokine 11	rs139141690	101499930	7	A	G	0.005	1.157	0.054	6.6E-102	trans
614	CXCL11	O14625	C-X-C motif chemokine 11	rs7080386	65048306	10	A	C	0.412	0.101	0.007	9.4E-42	trans
614	CXCL11	O14625	C-X-C motif chemokine 11	rs11596235	104391034	10	C	T	0.688	0.090	0.008	3.9E-30	trans
614	CXCL11	O14625	C-X-C motif chemokine 11	rs1654425	55538980	19	C	T	0.834	0.099	0.010	9.7E-24	trans
615	CXCL12	P48061	Stromal cell-derived factor 1	rs6542680	3640142	2	C	T	0.179	0.073	0.010	2.0E-13	trans
615	CXCL12	P48061	Stromal cell-derived factor 1	rs6768977	12508169	3	G	A	0.534	0.052	0.008	8.2E-12	trans
615	CXCL12	P48061	Stromal cell-derived factor 1	rs113760175	22343592	6	A	G	0.071	0.133	0.015	2.0E-19	trans
615	CXCL12	P48061	Stromal cell-derived factor 1	rs3131626	31415150	6	G	A	0.325	0.061	0.008	4.9E-13	trans
615	CXCL12	P48061	Stromal cell-derived factor 1	rs7028191	137117496	9	C	T	0.563	0.053	0.008	3.1E-12	trans
615	CXCL12	P48061	Stromal cell-derived factor 1	rs11239028	44883165	10	T	C	0.339	0.195	0.008	2.9E-128	cis
615	CXCL12	P48061	Stromal cell-derived factor 1	rs7896518	65104500	10	G	A	0.424	0.070	0.008	4.8E-19	trans
615	CXCL12	P48061	Stromal cell-derived factor 1	rs892090	55539072	19	G	T	0.834	0.073	0.010	6.2E-13	trans
616	CXCL13	O43927	C-X-C motif chemokine 13	rs2053526	78426029	4	A	G	0.603	0.055	0.008	1.5E-12	cis
616	CXCL13	O43927	C-X-C motif chemokine 13	rs13204736	32582603	6	A	G	0.348	0.061	0.008	6.6E-14	trans
616	CXCL13	O43927	C-X-C motif chemokine 13	rs55730499	161005610	6	T	C	0.082	0.106	0.014	9.2E-15	trans
616	CXCL13	O43927	C-X-C motif chemokine 13	rs34557412	16852187	17	G	A	0.007	0.360	0.046	2.8E-15	trans
616	CXCL13	O43927	C-X-C motif chemokine 13	rs77542162	67081278	17	G	A	0.023	0.305	0.025	7.8E-34	trans
616	CXCL13	O43927	C-X-C motif chemokine 13	rs190712692	45425178	19	A	G	0.054	0.142	0.017	1.3E-16	trans
616	CXCL13	O43927	C-X-C motif chemokine 13	rs2412973	30529631	22	C	A	0.557	0.069	0.008	4.4E-20	trans
617	CXCL14	O95715	C-X-C motif chemokine 14	rs4744	20304926	1	G	A	0.773	0.070	0.008	1.1E-16	trans
617	CXCL14	O95715	C-X-C motif chemokine 14	rs13171268	134955385	5	C	G	0.643	0.072	0.007	2.9E-22	cis
618	CXCL16	Q9H2A7	C-X-C motif chemokine 16	rs2009581	111807677	2	A	G	0.273	0.176	0.008	1.5E-105	trans
618	CXCL16	Q9H2A7	C-X-C motif chemokine 16	rs4347833	198557822	2	T	G	0.528	0.050	0.007	4.5E-12	trans
618	CXCL16	Q9H2A7	C-X-C motif chemokine 16	rs79806033	46094602	3	G	A	0.805	0.122	0.009	4.9E-42	trans
618	CXCL16	Q9H2A7	C-X-C motif chemokine 16	rs4449583	1284135	5	C	T	0.673	0.054	0.008	1.8E-12	trans
618	CXCL16	Q9H2A7	C-X-C motif chemokine 16	rs117030384	32573945	6	A	T	0.978	0.292	0.024	6.8E-33	trans
618	CXCL16	Q9H2A7	C-X-C motif chemokine 16	rs748113	73508791	10	T	C	0.565	0.103	0.007	1.1E-46	trans
618	CXCL16	Q9H2A7	C-X-C motif chemokine 16	rs1250567	81046265	10	T	C	0.547	0.078	0.007	1.1E-27	trans
618	CXCL16	Q9H2A7	C-X-C motif chemokine 16	rs748988	99786241	14	G	A	0.614	0.058	0.007	2.9E-15	trans
618	CXCL16	Q9H2A7	C-X-C motif chemokine 16	rs78086290	4641370	17	T	A	0.529	0.189	0.007	1.4E-148	cis
618	CXCL16	Q9H2A7	C-X-C motif chemokine 16	rs77542162	67081278	17	G	A	0.023	0.304	0.024	2.1E-36	trans
618	CXCL16	Q9H2A7	C-X-C motif chemokine 16	rs6031302	42656310	20	C	G	0.274	0.085	0.008	1.5E-26	trans
619	CXCL17	Q6UXB2	C-X-C motif chemokine 17	rs1124	22021517	8	G	A	0.677	0.070	0.007	3.3E-23	trans
619	CXCL17	Q6UXB2	C-X-C motif chemokine 17	rs9987332	120933963	8	G	A	0.575	0.048	0.007	9.0E-13	trans
619	CXCL17	Q6UXB2	C-X-C motif chemokine 17	rs1366050	81388375	10	C	T	0.658	0.049	0.007	1.8E-12	trans
619	CXCL17	Q6UXB2	C-X-C motif chemokine 17	rs117753190	67136325	17	G	C	0.037	0.171	0.018	5.7E-22	trans
619	CXCL17	Q6UXB2	C-X-C motif chemokine 17	rs4807612	1156853	19	T	C	0.678	0.064	0.007	3.9E-19	trans
619	CXCL17	Q6UXB2	C-X-C motif chemokine 17	rs11668599	42955136	19	G	A	0.078	0.118	0.012	1.6E-21	cis
620	CXCL3	P19876	C-X-C motif chemokine 3	rs12640189	6893181	4	T	C	0.173	0.068	0.010	1.4E-11	trans
620	CXCL3	P19876	C-X-C motif chemokine 3	rs3104369	32602482	6	T	C	0.230	0.101	0.009	2.2E-29	trans
620	CXCL3	P19876	C-X-C motif chemokine 3	rs7896518	65104500	10	G	A	0.424	0.070	0.008	6.1E-20	trans
621	CXCL5	P42830	C-X-C motif chemokine 5	rs62191340	242645466	2	A	G	0.543	0.052	0.007	1.3E-13	trans
621	CXCL5	P42830	C-X-C motif chemokine 5	rs1354034	56849749	3	T	C	0.396	0.120	0.007	3.6E-62	trans
621	CXCL5	P42830	C-X-C motif chemokine 5	rs12640189	6893181	4	T	C	0.173	0.084	0.010	3.7E-18	trans
621	CXCL5	P42830	C-X-C motif chemokine 5	rs352045	74864687	4	T	G	0.117	0.588	0.011	0.0E+00	cis
621	CXCL5	P42830	C-X-C motif chemokine 5	rs6993770	106581528	8	T	A	0.287	0.081	0.008	5.8E-25	trans
621	CXCL5	P42830	C-X-C motif chemokine 5	rs4631576	100705210	9	T	C	0.361	0.064	0.007	3.4E-18	trans
621	CXCL5	P42830	C-X-C motif chemokine 5	rs10821545	136934101	9	T	G	0.682	0.091	0.008	7.7E-33	trans
621	CXCL5	P42830	C-X-C motif chemokine 5	rs10761731	65027610	10	T	A	0.414	0.216	0.007	6.9E-196	trans
621	CXCL5	P42830	C-X-C motif chemokine 5	rs1654425	55538980	19	C	T	0.834	0.090	0.010	3.4E-21	trans
622	CXCL6	P80162	C-X-C motif chemokine 6	rs12075	159175354	1	A	G	0.583	0.086	0.007	7.1E-34	trans
622	CXCL6	P80162	C-X-C motif chemokine 6	rs1260326	27730940	2	T	C	0.392	0.052	0.007	3.4E-13	trans
622	CXCL6	P80162	C-X-C motif chemokine 6	rs16858768	219026698	2	C	A	0.003	0.441	0.064	5.3E-12	trans
622	CXCL6	P80162	C-X-C motif chemokine 6	rs16850073	74703999	4	T	C	0.376	0.560	0.008	0.0E+00	cis
622	CXCL6	P80162	C-X-C motif chemokine 6	rs10761737	65052205	10	C	T	0.413	0.066	0.007	1.5E-20	trans
622	CXCL6	P80162	C-X-C motif chemokine 6	rs892090	55539072	19	G	T	0.834	0.107	0.009	1.0E-29	trans
623	CXCL8	P10145	Interleukin-8	rs2241244	38153554	17	C	G	0.537	0.078	0.007	4.7E-26	trans
623	CXCL8	P10145	Interleukin-8	rs5902673	69929588	X	T	TA	0.257	0.048	0.007	6.4E-12	trans
623	CXCL8	P10145	Interleukin-8	rs892090	55539072	19	G	T	0.834	0.109	0.010	3.5E-27	trans
623	CXCL8	P10145	Interleukin-8	rs12075	159175354	1	A	G	0.583	0.138	0.007	6.7E-76	trans
623	CXCL8	P10145	Interleukin-8	rs16858768	219026698	2	C	A	0.003	0.962	0.068	4.3E-46	trans
623	CXCL8	P10145	Interleukin-8	rs74468557	74591403	4	G	A	0.946	0.180	0.017	2.2E-27	cis
623	CXCL8	P10145	Interleukin-8	rs4541868	106590705	8	C	A	0.726	0.062	0.008	7.6E-14	trans
623	CXCL8	P10145	Interleukin-8	rs7080386	65048306	10	A	C	0.412	0.084	0.007	4.8E-29	trans
623	CXCL8	P10145	Interleukin-8	rs184339396	49043608	X	C	G	0.032	0.123	0.018	3.7E-12	trans
624	CXCL9	Q07325	C-X-C motif chemokine 9	rs2476601	114377568	1	A	G	0.101	0.091	0.011	1.8E-15	trans
624	CXCL9	Q07325	C-X-C motif chemokine 9	rs13118503	76860470	4	C	T	0.759	0.149	0.008	5.0E-77	cis
624	CXCL9	Q07325	C-X-C motif chemokine 9	rs2858864	32578229	6	T	C	0.218	0.073	0.009	5.9E-17	trans
624	CXCL9	Q07325	C-X-C motif chemokine 9	rs4252129	161152905	6	C	T	0.984	0.312	0.027	1.1E-30	trans
625	CYB5R2	Q6BCY4	NADH-cytochrome b5 reductase 2	rs342293	106372219	7	C	G	0.540	0.071	0.007	3.2E-22	trans
625	CYB5R2	Q6BCY4	NADH-cytochrome b5 reductase 2	rs7080386	65048306	10	A	C	0.412	0.052	0.007	3.3E-12	trans
625	CYB5R2	Q6BCY4	NADH-cytochrome b5 reductase 2	rs61683338	7686858	11	C	G	0.888	0.385	0.012	8.3E-230	cis
626	CYTL1	Q9NRR1	Cytokine-like protein 1	rs11722554	5016883	4	G	A	0.962	1.317	0.019	0.0E+00	cis
626	CYTL1	Q9NRR1	Cytokine-like protein 1	rs7193058	20360101	16	A	G	0.758	0.056	0.008	2.2E-13	trans
627	DAAM1	Q9Y4D1	Disheveled-associated activator of morphogenesis 1	rs11242109	131677047	5	G	T	0.519	0.057	0.008	7.6E-14	trans
627	DAAM1	Q9Y4D1	Disheveled-associated activator of morphogenesis 1	rs342293	106372219	7	C	G	0.540	0.057	0.008	3.9E-14	trans
627	DAAM1	Q9Y4D1	Disheveled-associated activator of morphogenesis 1	rs1151519	65645831	11	C	G	0.797	0.106	0.009	1.7E-29	trans
627	DAAM1	Q9Y4D1	Disheveled-associated activator of morphogenesis 1	rs7160523	55594560	14	T	G	0.440	0.056	0.008	3.7E-13	trans
627	DAAM1	Q9Y4D1	Disheveled-associated activator of morphogenesis 1	rs55656227	55535326	19	A	G	0.811	0.067	0.010	4.3E-12	trans
628	DAB2	P98082	Disabled homolog 2	rs6993770	106581528	8	A	T	0.714	0.076	0.008	3.1E-20	trans
628	DAB2	P98082	Disabled homolog 2	rs7916868	64988931	10	T	A	0.501	0.058	0.007	1.3E-14	trans
629	DAG1	Q14118	Dystroglycan	rs1354034	56849749	3	C	T	0.604	0.053	0.008	2.4E-12	trans
629	DAG1	Q14118	Dystroglycan	rs892090	55539072	19	G	T	0.834	0.074	0.010	3.0E-13	trans
630	DAPK2	Q9UIK4	Death-associated protein kinase 2	rs34772505	64276772	15	T	C	0.430	0.387	0.008	0.0E+00	cis
631	DAPP1	Q9UN19	Dual adapter for phosphotyrosine and 3-phosphotyrosine and 3-phosphoinositide	rs1354034	56849749	3	C	T	0.604	0.059	0.008	1.0E-14	trans
631	DAPP1	Q9UN19	Dual adapter for phosphotyrosine and 3-phosphotyrosine and 3-phosphoinositide	rs7688733	100752652	4	A	G	0.522	0.100	0.008	6.2E-40	cis
632	DARS1	P14868	Aspartate--tRNA ligase, cytoplasmic	rs149170955	136691500	2	G	A	0.998	0.954	0.089	1.0E-26	cis
632	DARS1	P14868	Aspartate--tRNA ligase, cytoplasmic	rs78909033	241510903	2	G	A	0.864	0.100	0.011	5.9E-20	trans
632	DARS1	P14868	Aspartate--tRNA ligase, cytoplasmic	rs1354034	56849749	3	C	T	0.604	0.068	0.008	6.7E-19	trans
632	DARS1	P14868	Aspartate--tRNA ligase, cytoplasmic	rs342298	106373646	7	C	T	0.546	0.057	0.008	5.1E-14	trans
633	DBH	P09172	Dopamine beta-hydroxylase	rs61830291	221001142	1	C	A	0.097	0.054	0.008	5.7E-12	trans
633	DBH	P09172	Dopamine beta-hydroxylase	rs1611115	136500515	9	C	T	0.794	1.170	0.009	0.0E+00	cis
633	DBH	P09172	Dopamine beta-hydroxylase	rs2420915	122840277	10	G	A	0.914	0.064	0.008	7.9E-15	trans
633	DBH	P09172	Dopamine beta-hydroxylase	rs7944748	122506308	11	G	T	0.376	0.039	0.005	2.4E-16	trans
633	DBH	P09172	Dopamine beta-hydroxylase	rs2160725	66395726	17	C	A	0.485	0.040	0.005	2.7E-18	trans
633	DBH	P09172	Dopamine beta-hydroxylase	rs56254331	41826020	19	C	A	0.171	0.072	0.006	2.6E-32	trans
633	DBH	P09172	Dopamine beta-hydroxylase	rs754623	57467412	19	T	C	0.883	0.054	0.007	4.3E-14	trans
634	DBI	P07108	Acyl-CoA-binding protein	rs72833212	120136467	2	T	G	0.795	0.326	0.009	6.1E-263	cis
634	DBI	P07108	Acyl-CoA-binding protein	rs1354034	56849749	3	C	T	0.604	0.051	0.008	1.7E-11	trans
634	DBI	P07108	Acyl-CoA-binding protein	rs1671152	55526345	19	G	T	0.841	0.070	0.010	5.6E-12	trans
635	DBN1	Q16643	Drebrin	rs1354034	56849749	3	C	T	0.604	0.063	0.008	3.0E-16	trans

635	DBN1	Q16643	Drebrin	rs342293	106372219	7	C	G	0.541	0.052	0.008	4.6E-12	trans
636	DBNL	Q9UJU6	Drebrin-like protein	rs1354034	56849749	3	C	T	0.604	0.055	0.008	3.5E-13	trans
636	DBNL	Q9UJU6	Drebrin-like protein	rs7799945	44109802	7	G	A	0.327	0.058	0.008	3.7E-13	cis
636	DBNL	Q9UJU6	Drebrin-like protein	rs342298	106373646	7	C	T	0.546	0.055	0.007	3.0E-13	trans
636	DBNL	Q9UJU6	Drebrin-like protein	rs6993770	106581528	8	A	T	0.713	0.064	0.008	6.6E-15	trans
637	DCBLD2	Q96PD2	Discoidin, CUB and LCCL domain-containing protein 2	rs12487717	98605007	3	G	A	0.987	1.091	0.032	4.8E-253	cis
637	DCBLD2	Q96PD2	Discoidin, CUB and LCCL domain-containing protein 2	rs7356034	170732599	3	G	A	0.718	0.062	0.008	8.8E-15	trans
637	DCBLD2	Q96PD2	Discoidin, CUB and LCCL domain-containing protein 2	rs28601761	126500031	8	C	G	0.581	0.063	0.007	2.2E-18	trans
637	DCBLD2	Q96PD2	Discoidin, CUB and LCCL domain-containing protein 2	rs115478735	136149711	9	A	T	0.816	0.063	0.009	4.8E-12	trans
637	DCBLD2	Q96PD2	Discoidin, CUB and LCCL domain-containing protein 2	rs56278466	17875857	10	G	T	0.661	0.085	0.007	1.9E-30	trans
637	DCBLD2	Q96PD2	Discoidin, CUB and LCCL domain-containing protein 2	rs174581	61606683	11	A	G	0.354	0.056	0.007	2.7E-14	trans
637	DCBLD2	Q96PD2	Discoidin, CUB and LCCL domain-containing protein 2	rs28929474	94844947	14	T	C	0.021	0.501	0.025	3.5E-90	trans
637	DCBLD2	Q96PD2	Discoidin, CUB and LCCL domain-containing protein 2	rs11078597	1618363	17	C	T	0.188	0.077	0.009	1.3E-17	trans
637	DCBLD2	Q96PD2	Discoidin, CUB and LCCL domain-containing protein 2	rs9906593	9588715	17	C	A	0.237	0.062	0.008	5.5E-14	trans
637	DCBLD2	Q96PD2	Discoidin, CUB and LCCL domain-containing protein 2	rs77542162	67081278	17	A	G	0.977	0.199	0.024	2.7E-17	trans
637	DCBLD2	Q96PD2	Discoidin, CUB and LCCL domain-containing protein 2	rs58542926	19379549	19	C	T	0.925	0.114	0.013	9.5E-18	trans
637	DCBLD2	Q96PD2	Discoidin, CUB and LCCL domain-containing protein 2	rs1672992	35556729	19	T	C	0.861	0.144	0.010	6.7E-46	trans
637	DCBLD2	Q96PD2	Discoidin, CUB and LCCL domain-containing protein 2	rs9980195	46268783	21	T	C	0.474	0.048	0.007	6.3E-12	trans
638	DCC	P43146	Netrin receptor DCC	rs9807459	49481736	18	A	G	0.307	0.247	0.008	4.1E-196	cis
639	DCN	P07585	Decorin	rs1260326	27730940	2	C	T	0.609	0.059	0.007	7.4E-16	trans
640	DCTD	P32321	Deoxycytidylate deaminase	rs1354034	56849749	3	C	T	0.604	0.062	0.008	5.9E-16	trans
641	DCTN1	Q14203	Dynactin subunit 1	rs1339847	248039294	1	G	A	0.891	0.107	0.012	1.9E-19	trans
641	DCTN1	Q14203	Dynactin subunit 1	rs35505243	98263327	2	A	AT	0.916	0.100	0.014	3.2E-13	trans
642	DCTPP1	Q9H773	dCTP pyrophosphatase 1	rs1354034	56849749	3	C	T	0.604	0.055	0.007	9.0E-14	trans
642	DCTPP1	Q9H773	dCTP pyrophosphatase 1	rs12342201	95894964	9	G	A	0.523	0.054	0.007	1.0E-13	trans
642	DCTPP1	Q9H773	dCTP pyrophosphatase 1	rs56278466	17875857	10	G	T	0.662	0.095	0.008	3.6E-35	trans
642	DCTPP1	Q9H773	dCTP pyrophosphatase 1	rs146094440	30435601	16	C	G	0.987	0.520	0.035	7.2E-50	cis
643	DCXR	Q7Z4W1	L-xylulose reductase	rs4979373	117144504	9	C	T	0.509	0.074	0.007	7.3E-25	trans
643	DCXR	Q7Z4W1	L-xylulose reductase	rs10883451	101924418	10	T	C	0.499	0.058	0.007	1.4E-16	trans
643	DCXR	Q7Z4W1	L-xylulose reductase	rs11604580	93852880	11	G	A	0.893	0.091	0.011	1.6E-15	trans
643	DCXR	Q7Z4W1	L-xylulose reductase	rs61746217	79993919	17	C	T	0.997	0.767	0.065	1.4E-32	cis
643	DCXR	Q7Z4W1	L-xylulose reductase	rs3747207	44324855	22	A	G	0.215	0.081	0.009	3.2E-21	trans
644	DDAH1	O94760	N(G),N(G)-dimethylarginine dimethylaminohydrolase 1	rs1498374	85787334	1	C	T	0.793	0.186	0.009	5.9E-90	cis
644	DDAH1	O94760	N(G),N(G)-dimethylarginine dimethylaminohydrolase 1	rs1434282	199010721	1	T	C	0.726	0.088	0.009	5.5E-25	trans
644	DDAH1	O94760	N(G),N(G)-dimethylarginine dimethylaminohydrolase 1	rs342293	106372219	7	C	G	0.541	0.052	0.007	4.0E-12	trans
644	DDAH1	O94760	N(G),N(G)-dimethylarginine dimethylaminohydrolase 1	rs61905116	116649538	11	G	A	0.064	0.171	0.015	3.2E-29	trans
645	DDC	P20711	Aromatic-L-amino-acid decarboxylase	rs34103191	69359280	4	A	G	0.343	0.053	0.008	7.1E-12	trans
645	DDC	P20711	Aromatic-L-amino-acid decarboxylase	rs12112308	50603487	7	T	C	0.984	1.435	0.030	0.0E+00	cis
645	DDC	P20711	Aromatic-L-amino-acid decarboxylase	rs12342201	95894964	9	G	A	0.523	0.057	0.007	3.6E-15	trans
645	DDC	P20711	Aromatic-L-amino-acid decarboxylase	rs601338	49206674	19	G	A	0.491	0.087	0.007	6.9E-33	trans
645	DDC	P20711	Aromatic-L-amino-acid decarboxylase	rs3747207	44324855	22	A	G	0.215	0.077	0.009	1.7E-18	trans
646	DDHD2	O94830	Phospholipase DDHD2	rs342298	106373646	7	C	T	0.545	0.061	0.007	2.3E-16	trans
647	DDI2	Q5TDH0	Protein DDI1 homolog 2	rs41270291	15994251	1	G	A	0.989	0.264	0.035	2.5E-14	cis
647	DDI2	Q5TDH0	Protein DDI1 homolog 2	rs34315295	106471685	14	A	G	0.148	0.086	0.010	1.6E-16	trans
648	DDR1	Q08345	Epithelial discoidin domain-containing receptor 1	rs3095353	30803808	6	T	A	0.414	0.347	0.007	0.0E+00	cis
648	DDR1	Q08345	Epithelial discoidin domain-containing receptor 1	rs4841132	9183596	8	A	G	0.092	0.132	0.012	2.2E-27	trans
648	DDR1	Q08345	Epithelial discoidin domain-containing receptor 1	rs56278466	17875857	10	G	T	0.661	0.061	0.007	1.4E-16	trans
648	DDR1	Q08345	Epithelial discoidin domain-containing receptor 1	rs78689694	126234820	11	G	C	0.867	0.087	0.010	4.6E-17	trans
648	DDR1	Q08345	Epithelial discoidin domain-containing receptor 1	rs139097404	43933941	15	T	C	0.977	0.163	0.024	9.5E-12	trans
648	DDR1	Q08345	Epithelial discoidin domain-containing receptor 1	rs186021206	7069412	17	A	G	0.006	0.841	0.048	3.9E-68	trans
648	DDR1	Q08345	Epithelial discoidin domain-containing receptor 1	rs708686	5840619	19	T	C	0.268	0.055	0.008	5.8E-12	trans
648	DDR1	Q08345	Epithelial discoidin domain-containing receptor 1	rs73930562	38758471	19	G	A	0.848	0.072	0.010	1.9E-13	trans
648	DDR1	Q08345	Epithelial discoidin domain-containing receptor 1	rs681343	49206462	19	T	C	0.510	0.128	0.007	2.3E-73	trans
648	DDR1	Q08345	Epithelial discoidin domain-containing receptor 1	rs55953905	42882671	21	T	C	0.247	0.115	0.008	3.7E-45	trans
649	DDT	P30046	D-dopachrome decarboxylase	rs1006771	24314006	22	G	T	0.355	0.206	0.008	1.3E-158	cis
650	DDX58	O95786	Antiviral innate immune response receptor RIG-I	rs1133071	32455674	9	G	A	0.299	0.289	0.008	1.1E-263	cis
651	DECR1	Q16698	2,4-dienoyl-CoA reductase, mitochondrial	rs189045259	91051553	8	G	A	0.995	0.953	0.056	6.3E-64	cis
651	DECR1	Q16698	2,4-dienoyl-CoA reductase, mitochondrial	rs6993770	106581528	8	A	T	0.713	0.065	0.008	5.6E-15	trans
651	DECR1	Q16698	2,4-dienoyl-CoA reductase, mitochondrial	rs1671152	55526345	19	G	T	0.841	0.075	0.010	5.4E-13	trans
652	DEFA1_DEFA1B	P59665	Neutrophil defensin 1	rs3917932	36943916	1	C	G	0.424	0.078	0.007	2.9E-25	trans
652	DEFA1_DEFA1B	P59665	Neutrophil defensin 1	rs4927110	54890664	1	A	G	0.522	0.051	0.007	3.6E-12	trans
652	DEFA1_DEFA1B	P59665	Neutrophil defensin 1	rs61740288	71762413	2	A	G	0.022	0.216	0.026	4.1E-17	trans
652	DEFA1_DEFA1B	P59665	Neutrophil defensin 1	rs13063578	47087837	3	T	A	0.603	0.056	0.007	7.9E-14	trans
652	DEFA1_DEFA1B	P59665	Neutrophil defensin 1	rs77547572	98451375	5	A	G	0.081	0.099	0.014	8.3E-13	trans
652	DEFA1_DEFA1B	P59665	Neutrophil defensin 1	rs3128959	33048380	6	A	G	0.123	0.192	0.011	2.7E-64	trans
652	DEFA1_DEFA1B	P59665	Neutrophil defensin 1	rs915125	82463376	6	T	C	0.278	0.065	0.008	4.1E-15	trans
652	DEFA1_DEFA1B	P59665	Neutrophil defensin 1	rs210962	135503785	6	C	T	0.762	0.099	0.009	2.0E-30	trans
652	DEFA1_DEFA1B	P59665	Neutrophil defensin 1	rs116236945	6876927	8	C	T	0.614	0.274	0.009	7.7E-218	cis
652	DEFA1_DEFA1B	P59665	Neutrophil defensin 1	rs35440906	61646351	8	CA	C	0.186	0.130	0.010	5.0E-41	trans
652	DEFA1_DEFA1B	P59665	Neutrophil defensin 1	rs10103048	130602281	8	A	C	0.405	0.058	0.008	6.8E-14	trans
652	DEFA1_DEFA1B	P59665	Neutrophil defensin 1	rs59974297	101246301	10	G	GT	0.110	0.094	0.012	1.2E-15	trans
652	DEFA1_DEFA1B	P59665	Neutrophil defensin 1	rs2241244	38153554	17	G	C	0.463	0.113	0.007	7.5E-54	trans
653	DEFB103A_DEFB103B	P81534	Beta-defensin 103	rs34931250	66879927	17	T	C	0.061	0.135	0.016	1.3E-16	trans
654	DEFB104A_DEFB104B	Q8WTQ1	Beta-defensin 104	rs183772362	7243016	8	T	C	0.031	0.400	0.021	2.2E-80	cis
654	DEFB104A_DEFB104B	Q8WTQ1	Beta-defensin 104	rs144072245	12477657	8	C	G	0.012	0.819	0.035	2.5E-122	trans
655	DEFB118	Q96PH6	Beta-defensin 118	rs77587796	106371016	14	T	C	0.117	0.105	0.014	9.9E-15	trans
656	DEFB4A_DEFB4B	O15263	Beta-defensin 4A	rs117797877	8164592	8	T	C	0.024	0.233	0.026	3.6E-19	cis
656	DEFB4A_DEFB4B	O15263	Beta-defensin 4A	rs4831643	12477559	8	G	A	0.720	0.126	0.008	3.1E-50	trans
657	DFFA	O00273	DNA fragmentation factor subunit alpha	rs183281838	10038213	1	G	T	0.997	0.649	0.080	5.6E-16	cis
657	DFFA	O00273	DNA fragmentation factor subunit alpha	rs1354034	56849749	3	C	T	0.604	0.065	0.008	6.9E-18	trans
657	DFFA	O00273	DNA fragmentation factor subunit alpha	rs10733789	64948684	10	C	T	0.313	0.057	0.008	1.3E-12	trans
658	DGCR6	Q14129	Protein DGCR6	rs62143198	54320939	19	A	G	0.210	0.066	0.010	4.1E-12	trans
658	DGCR6	Q14129	Protein DGCR6	rs430899	18896530	22	C	A	0.628	0.131	0.008	1.4E-60	cis
659	DHRS4L2	Q6PKH6	Dehydrogenase/reductase SDR family member 4-like 2	rs146691898	24477942	14	A	G	0.948	0.651	0.017	0.0E+00	cis
660	DIABLO	Q9NR28	Diablo homolog, mitochondrial	rs1354034	56849749	3	C	T	0.604	0.053	0.008	4.8E-12	trans
660	DIABLO	Q9NR28	Diablo homolog, mitochondrial	rs10733789	64948684	10	C	T	0.312	0.061	0.008	1.3E-13	trans
661	DIPK2B	Q9H7Y0	Divergent protein kinase domain 2B	rs77515410	149829934	5	T	C	0.041	0.140	0.018	3.9E-15	trans
661	DIPK2B	Q9H7Y0	Divergent protein kinase domain 2B	rs56278466	17875857	10	G	T	0.661	0.139	0.008	1.0E-75	trans
661	DIPK2B	Q9H7Y0	Divergent protein kinase domain 2B	rs12975366	54759361	19	T	C	0.605	0.055	0.007	2.6E-14	trans
661	DIPK2B	Q9H7Y0	Divergent protein kinase domain 2B	rs4498684	45059412	X	C	A	0.792	0.145	0.007	2.8E-87	cis
662	DKK1	O94907	Dickkopf-related protein 1	rs12407843	156873727	1	A	G	0.110	0.081	0.012	8.3E-12	trans
662	DKK1	O94907	Dickkopf-related protein 1	rs7618405	18250509	3	C	A	0.795	0.075	0.009	5.3E-16	trans
662	DKK1	O94907	Dickkopf-related protein 1	rs1354034	56849749	3	T	C	0.396	0.087	0.008	1.0E-30	trans
662	DKK1	O94907	Dickkopf-related protein 1	rs3804753	122851840	3	A	G	0.407	0.052	0.008	5.3E-12	trans
662	DKK1	O94907	Dickkopf-related protein 1	rs142178438	83919199	4	G	GTA	0.187	0.067	0.010	2.6E-12	trans
662	DKK1	O94907	Dickkopf-related protein 1	rs35173808	31326148	6	C	A	0.903	0.087	0.012	4.2E-12	trans
662	DKK1	O94907	Dickkopf-related protein 1	rs6961069	80218961	7	T	C	0.402	0.067	0.008	4.5E-19	trans
662	DKK1	O94907	Dickkopf-related protein 1	rs6993770	106581528	8	A	T	0.714	0.185	0.008	8.2E-113	trans
662	DKK1	O94907	Dickkopf-related protein 1	rs7097068	54119880	10	T	G	0.565	0.103	0.008	4.9E-42	cis
662	DKK1	O94907	Dickkopf-related protein 1	rs59001897	65160392	15	A	T	0.175	0.072	0.010	1.7E-13	trans
662	DKK1	O94907	Dickkopf-related protein 1	rs12445050	81870969	16	T	C	0.138	0.101	0.011	4.7E-21	trans
662	DKK1	O94907	Dickkopf-related protein 1	rs34416903	88559245	16	C	T	0.280	0.072	0.008	1.2E-17	trans
662	DKK1	O94907	Dickkopf-related protein 1	rs892090	55539072	19	G	T	0.834	0.165	0.010	1.1E-61	trans
662	DKK1	O94907	Dickkopf-related protein 1	rs6081565	19287904	20	A	G	0.352	0.068	0.008	1.8E-18	trans
662	DKK1	O94907	Dickkopf-related protein 1	rs5967171	99957136	X	C	T	0.333	0.049	0.006	4.2E-14	trans
663	DKK3	Q9UBP4	Dickkopf-related protein 3	rs539657009	161628456	1	T	C	0.130	0.100	0.011	2.9E-21	trans
663	DKK3	Q9UBP4	Dickkopf-related protein 3	rs2389858	17908130	7	T	A	0.509	0.065	0.007	1.1E-21	trans
663	DKK3	Q9UBP4	Dickkopf-related protein 3	rs507666	136149399	9	G	A	0.815	0.103	0.009	6.8E-31	trans
663	DKK3	Q9UBP4	Dickkopf-related protein 3	rs196255	121480161	10	C	T	0.587	0.065	0.007	5.8E-21	trans
663	DKK3	Q9UBP4	Dickkopf-related protein 3	rs11022114	12038874	11	A	G	0.315	0.372	0.008	0.0E+00	cis
663	DKK3	Q9UBP4	Dickkopf-related protein 3	rs186021206	7069412	17	A	G	0.006	0.443	0.047	6.3E-21	trans
664	DKK4	Q9UBT3	Dickkopf-related protein 4	rs77203322	42147164	8	T	C	0.950	0.550	0.017	1.3E-228	cis
664	DKK4	Q9UBT3	Dickkopf-related protein 4	rs1964528	76390058	8	C	T	0.887	0.080	0.012	8.1E-12	trans
664	DKK4	Q9UBT3	Dickkopf-related protein 4	rs7232	59940599	11	A	T	0.373	0.073	0.008	6.9E-22	trans
664	DKK4	Q9UBT3	Dickkopf-related protein 4	rs77542162	67081278	17	G	A	0.023	0.545	0.025	1.9E-108	trans
665	DKKL1	Q9UK85	Dickkopf-like protein 1	rs2740488	107661742	9	A	C	0.734	0.037	0.004	3.9E-19	trans
665	DKKL1	Q9UK85	Dickkopf-like protein 1	rs12149545	56993161	16	A	G	0.316	0.048	0.004	2.9E-34	trans
665	DKKL1	Q9UK85	Dickkopf-like protein 1	rs77542162	67081278	17	G	A	0.023	0.390	0.012	2.0E-215	trans
665	DKKL1	Q9UK85	Dickkopf-like protein 1	rs149615216	47106028	18	T	C	0.011	0.132	0.018	5.9E-14	trans
665	DKKL1	Q9UK85	Dickkopf-like protein 1	rs2303759	49869051	19	T	G	0.748	1.260	0.009	0.0E+00	cis
666	DLG4	P78352	Disks large homolog 4	rs1354034	56849749	3	C	T	0.604	0.059	0.008	1.2E-14	trans
666	DLG4	P78352	Disks large homolog 4	rs9271344	32583328	6	T	C	0.642	0.058	0.008	1.7E-13	trans
667	DLK1	P80370	Protein delta homolog 1	rs6772323	156227643	3	C	A	0.374	0.050	0.007	1.4E-12	trans
667	DLK1	P80370	Protein delta homolog 1	rs2282802	139712239	5	G	A	0.440	0.049	0.007	8.4E-13	trans
667	DLK1	P80370	Protein delta homolog 1	rs732194	100626344	6	T	C	0.477	0.056	0.007	2.0E-16	trans
667	DLK1	P80370	Protein delta homolog 1	rs10823391	71226444	10	C	T	0.533	0.053	0.007	8.8E-15	trans
667	DLK1	P80370	Protein delta homolog 1	rs10893501	126251033	11	G	A	0.133	0.070	0.010	2.8E-12	trans
667	DLK1	P80370	Protein delta homolog 1	rs12881545	101176212	14	C	G	0.669	0.492	0.008	0.0E+00	cis
667	DLK1	P80370	Protein delta homolog 1	rs112001035	66823805	17	G	A	0.939	0.223	0.015	1.4E-52	trans
668	DLL1	O00548	Delta-like protein 1	rs61747728	179526214	1	T	C	0.038	0.178	0.019	2.7E-21	trans
668	DLL1	O00548	Delta-like protein 1	rs9264625	31237960	6	G	C	0.368	0.081	0.008	4.7E-26	trans
668	DLL1	O00548	Delta-like protein 1	rs9356631	170587470	6	G	A	0.409	0.157	0.007	9.2E-100	cis
668	DLL1	O00548	Delta-like protein 1	rs8176672	136142185	9	T	C	0.061	0.181	0.015	7.0E-34	trans
668	DLL1	O00548	Delta-like protein 1	rs3814995	36342212	19	T	C	0.315	0.061	0.008	1.7E-15	trans
668	DLL1	O00548	Delta-like protein 1	rs2837988	42619544	21	A	C	0.362	0.092	0.008	4.3E-34	trans
669	DMP1	Q13316	Dentin matrix acidic phosphoprotein 1	rs3001032	219727779	1	C	T	0.320	0.064	0.008	2.5E-16	trans
669	DMP1	Q13316	Dentin matrix acidic phosphoprotein 1	rs13303	52558008	3	C	T	0.560	0.062	0.007	3.7E-17	trans
669	DMP1	Q13316	Dentin matrix acidic phosphoprotein 1	rs139276089	187160394	4	G	C	0.487	0.083	0.007	2.9E-29	trans
669	DMP1	Q13316	Dentin matrix acidic phosphoprotein 1	rs2731672	176842474	5	T	C	0.256	0.071	0.008	1.8E-17	trans
669	DMP1	Q13316	Dentin matrix acidic phosphoprotein 1	rs11995824	120012700	8	G	C	0.544	0.068	0.007	2.3E-20	trans
669	DMP1	Q13316	Dentin matrix acidic phosphoprotein 1	rs2519093	136141870	9	C	T	0.816	0.072	0.010	3.4E-14	trans
669	DMP1	Q13316	Dentin matrix acidic phosphoprotein 1	rs12789028	65326154	11	G	A	0.800	0.072	0.009	6.5E-15	trans
669	DMP1	Q13316	Dentin matrix acidic phosphoprotein 1	rs9594738	42952145	13	T	C	0.488	0.105	0.007	3.5E-46	trans

669	DMP1	Q13316	Dentin matrix acidic phosphoprotein 1	rs806295	50815467	13	G	A	0.469	0.053	0.007	4.8E-13	trans
669	DMP1	Q13316	Dentin matrix acidic phosphoprotein 1	rs117068593	93118229	14	C	T	0.813	0.066	0.009	2.7E-12	trans
669	DMP1	Q13316	Dentin matrix acidic phosphoprotein 1	rs8085237	77223783	18	T	C	0.687	0.058	0.008	2.3E-13	trans
669	DMP1	Q13316	Dentin matrix acidic phosphoprotein 1	rs913678	48955424	20	T	C	0.670	0.057	0.008	3.4E-13	trans
670	DNAJA2	O60884	DnaJ homolog subfamily A member 2	rs342298	106373646	7	C	T	0.545	0.062	0.008	2.1E-16	trans
671	DNAJA4	Q8WW22	DnaJ homolog subfamily A member 4	rs11759553	135422296	6	A	T	0.727	0.086	0.008	7.5E-26	trans
671	DNAJA4	Q8WW22	DnaJ homolog subfamily A member 4	rs11635166	78538899	15	T	C	0.179	0.157	0.009	3.1E-61	cis
672	DNAJB1	P25685	DnaJ homolog subfamily B member 1	rs3811444	248039451	1	C	T	0.668	0.070	0.008	1.2E-18	trans
672	DNAJB1	P25685	DnaJ homolog subfamily B member 1	rs1354034	56849749	3	C	T	0.605	0.082	0.008	1.4E-27	trans
672	DNAJB1	P25685	DnaJ homolog subfamily B member 1	rs342298	106373646	7	C	T	0.546	0.053	0.007	1.8E-12	trans
672	DNAJB1	P25685	DnaJ homolog subfamily B member 1	rs10733789	64948684	10	C	T	0.313	0.057	0.008	1.0E-12	trans
672	DNAJB1	P25685	DnaJ homolog subfamily B member 1	rs9612021	43583598	22	T	C	0.331	0.086	0.008	4.0E-27	trans
673	DNAJB14	Q8TBM8	DnaJ homolog subfamily B member 14	rs1354034	56849749	3	C	T	0.605	0.090	0.008	6.9E-32	trans
673	DNAJB14	Q8TBM8	DnaJ homolog subfamily B member 14	rs342299	106373718	7	C	T	0.545	0.052	0.008	5.6E-12	trans
673	DNAJB14	Q8TBM8	DnaJ homolog subfamily B member 14	rs6993770	106581528	8	A	T	0.713	0.057	0.008	1.1E-11	trans
674	DNAJB6	O75190	DnaJ homolog subfamily B member 6	rs342298	106373646	7	C	T	0.546	0.053	0.008	4.4E-12	trans
674	DNAJB6	O75190	DnaJ homolog subfamily B member 6	rs12668458	157145474	7	C	T	0.458	0.191	0.008	5.1E-134	cis
674	DNAJB6	O75190	DnaJ homolog subfamily B member 6	rs6993770	106581528	8	A	T	0.713	0.061	0.008	2.0E-13	trans
675	DNAJB8	Q8NHS0	DnaJ homolog subfamily B member 8	rs12668458	157145474	7	C	T	0.457	0.082	0.008	1.2E-25	trans
675	DNAJB8	Q8NHS0	DnaJ homolog subfamily B member 8	rs112988467	107072766	14	T	C	0.269	0.063	0.009	1.1E-12	trans
676	DNAJC21	Q5F1R6	DnaJ homolog subfamily C member 21	rs1354034	56849749	3	C	T	0.604	0.055	0.008	6.6E-13	trans
676	DNAJC21	Q5F1R6	DnaJ homolog subfamily C member 21	rs35176895	34930387	5	T	C	0.558	0.107	0.008	6.9E-46	cis
676	DNAJC21	Q5F1R6	DnaJ homolog subfamily C member 21	rs9271344	32583328	6	T	C	0.642	0.056	0.008	7.5E-13	trans
676	DNAJC21	Q5F1R6	DnaJ homolog subfamily C member 21	rs342296	106372903	7	G	A	0.543	0.056	0.007	5.9E-14	trans
677	DNAJC6	O75061	Putative tyrosine-protein phosphatase auxilin	rs7527768	65758036	1	G	A	0.585	0.079	0.008	1.2E-25	cis
677	DNAJC6	O75061	Putative tyrosine-protein phosphatase auxilin	rs342293	106372219	7	C	G	0.540	0.090	0.008	9.9E-33	trans
677	DNAJC6	O75061	Putative tyrosine-protein phosphatase auxilin	rs7080386	65048306	10	A	C	0.412	0.089	0.008	4.3E-31	trans
677	DNAJC6	O75061	Putative tyrosine-protein phosphatase auxilin	rs34416903	88559245	16	C	T	0.279	0.071	0.009	7.7E-17	trans
677	DNAJC6	O75061	Putative tyrosine-protein phosphatase auxilin	rs10418205	6100433	19	A	G	0.627	0.108	0.008	9.1E-44	trans
678	DNAJC9	Q8WXX5	DnaJ homolog subfamily C member 9	rs7681303	83895219	4	T	C	0.180	0.066	0.010	5.0E-12	trans
679	DNER	Q8NFT8	Delta and Notch-like epidermal growth factor-related receptor	rs34412673	230592359	2	A	T	0.668	0.280	0.008	6.1E-275	cis
679	DNER	Q8NFT8	Delta and Notch-like epidermal growth factor-related receptor	rs1713987	57785149	4	T	A	0.185	0.081	0.009	3.1E-18	trans
679	DNER	Q8NFT8	Delta and Notch-like epidermal growth factor-related receptor	rs9379084	7231843	6	A	G	0.114	0.085	0.012	3.6E-13	trans
679	DNER	Q8NFT8	Delta and Notch-like epidermal growth factor-related receptor	rs4741849	3733660	9	G	T	0.472	0.050	0.007	4.9E-12	trans
679	DNER	Q8NFT8	Delta and Notch-like epidermal growth factor-related receptor	rs62625031	71243629	10	T	A	0.039	0.145	0.019	1.0E-14	trans
679	DNER	Q8NFT8	Delta and Notch-like epidermal growth factor-related receptor	rs186021206	7069412	17	A	G	0.006	0.455	0.049	3.1E-20	trans
679	DNER	Q8NFT8	Delta and Notch-like epidermal growth factor-related receptor	rs8127156	42543403	21	G	C	0.256	0.102	0.008	1.3E-35	trans
680	DNM1	Q05193	Dynamin-1	rs6993770	106581528	8	T	A	0.287	0.056	0.008	6.4E-12	trans
680	DNM1	Q05193	Dynamin-1	rs3003599	130979181	9	G	A	0.803	0.321	0.009	2.6E-252	cis
680	DNM1	Q05193	Dynamin-1	rs7896518	65104500	10	G	A	0.424	0.079	0.008	1.2E-25	trans
681	DNM3	Q9UQ16	Dynamin-3	rs6427756	199000727	1	A	G	0.265	0.072	0.008	9.8E-18	trans
681	DNM3	Q9UQ16	Dynamin-3	rs13062095	101267385	3	T	C	0.662	0.053	0.008	1.2E-11	trans
681	DNM3	Q9UQ16	Dynamin-3	rs707388	74208459	7	A	T	0.158	0.075	0.010	4.5E-13	trans
681	DNM3	Q9UQ16	Dynamin-3	rs342296	106372903	7	G	A	0.543	0.140	0.007	4.7E-80	trans
681	DNM3	Q9UQ16	Dynamin-3	rs7896518	65104500	10	G	A	0.424	0.065	0.008	5.6E-18	trans
681	DNM3	Q9UQ16	Dynamin-3	rs12957249	5699128	18	A	G	0.723	0.263	0.008	1.4E-217	trans
682	DNMBP	Q6XZF7	Dynamin-binding protein	rs114694170	88180196	5	C	T	0.060	0.124	0.016	4.8E-15	trans
682	DNMBP	Q6XZF7	Dynamin-binding protein	rs7069556	101770377	10	C	G	0.922	0.211	0.014	1.4E-50	cis
683	DNPEP	Q9ULA0	Aspartyl aminopeptidase	rs115897075	220250232	2	C	G	0.018	0.330	0.028	4.4E-32	cis
683	DNPEP	Q9ULA0	Aspartyl aminopeptidase	rs71309976	56728081	3	T	C	0.011	0.291	0.039	4.2E-14	trans
684	DNPH1	O43598	2'-deoxynucleoside 5'-phosphate N-hydrolase 1	rs77321231	43200203	6	C	T	0.896	0.460	0.012	0.0E+00	cis
685	DOC2B	Q14184	Double C2-like domain-containing protein beta	rs8069133	32311	17	G	A	0.674	0.067	0.008	8.9E-16	cis
686	DOCK9	Q9BZ29	Dedicator of cytokinesis protein 9	rs61997961	106864531	14	C	T	0.804	0.117	0.011	5.5E-27	trans
686	DOCK9	Q9BZ29	Dedicator of cytokinesis protein 9	rs10427551	22662770	22	T	A	0.832	0.165	0.010	8.4E-57	trans
687	DOK2	O60496	Docking protein 2	rs342298	106373646	7	C	T	0.545	0.054	0.007	5.6E-13	trans
687	DOK2	O60496	Docking protein 2	rs67385415	21853733	8	C	T	0.446	0.062	0.008	2.0E-16	cis
687	DOK2	O60496	Docking protein 2	rs6993770	106581528	8	A	T	0.713	0.090	0.008	1.5E-27	trans
688	DPEP1	P16444	Dipeptidase 1	rs12508709	145319544	4	T	C	0.514	0.044	0.006	1.1E-13	trans
688	DPEP1	P16444	Dipeptidase 1	rs10807578	14588147	6	C	G	0.803	0.048	0.007	4.8E-12	trans
688	DPEP1	P16444	Dipeptidase 1	rs79137699	104249070	9	T	C	0.967	0.129	0.016	3.4E-16	trans
688	DPEP1	P16444	Dipeptidase 1	rs115478735	136149711	9	T	A	0.184	0.053	0.007	2.9E-13	trans
688	DPEP1	P16444	Dipeptidase 1	rs78689694	126234820	11	G	C	0.867	0.097	0.008	4.7E-32	trans
688	DPEP1	P16444	Dipeptidase 1	rs409170	89682350	16	G	A	0.597	0.799	0.008	0.0E+00	cis
688	DPEP1	P16444	Dipeptidase 1	rs186021206	7069412	17	A	G	0.006	0.990	0.038	1.5E-146	trans
688	DPEP1	P16444	Dipeptidase 1	rs8111600	5821968	19	T	C	0.040	0.110	0.014	2.1E-14	trans
689	DPEP2	Q9H4A9	Dipeptidase 2	rs148866368	32531582	6	G	A	0.450	0.054	0.008	1.4E-11	trans
689	DPEP2	Q9H4A9	Dipeptidase 2	rs144339907	68025998	16	A	C	0.996	2.280	0.061	4.3E-304	cis
689	DPEP2	Q9H4A9	Dipeptidase 2	rs186021206	7069412	17	A	G	0.006	0.661	0.050	2.8E-39	trans
690	DPP10	Q8N608	Inactive dipeptidyl peptidase 10	rs17355498	115937924	2	G	T	0.504	0.261	0.007	3.3E-268	cis
690	DPP10	Q8N608	Inactive dipeptidyl peptidase 10	rs73165969	57750725	4	A	G	0.181	0.064	0.009	9.1E-12	trans
690	DPP10	Q8N608	Inactive dipeptidyl peptidase 10	rs4552882	55462324	8	G	A	0.799	0.092	0.009	1.0E-24	trans
690	DPP10	Q8N608	Inactive dipeptidyl peptidase 10	rs2519093	136141870	9	C	T	0.816	0.122	0.009	1.3E-38	trans
690	DPP10	Q8N608	Inactive dipeptidyl peptidase 10	rs56278466	17875857	10	G	T	0.662	0.119	0.008	3.8E-55	trans
691	DPP4	P27487	Dipeptidyl peptidase 4	rs13015258	162930725	2	T	G	0.379	0.229	0.008	9.0E-204	cis
691	DPP4	P27487	Dipeptidyl peptidase 4	rs3807865	12250402	7	A	G	0.412	0.076	0.007	1.2E-25	trans
691	DPP4	P27487	Dipeptidyl peptidase 4	rs1617484	65463095	7	A	G	0.545	0.059	0.007	1.7E-16	trans
692	DPP6	P42658	Dipeptidyl aminopeptidase-like protein 6	rs2071592	31515340	6	T	A	0.653	0.060	0.007	6.5E-16	trans
692	DPP6	P42658	Dipeptidyl aminopeptidase-like protein 6	rs2240046	20371273	7	G	C	0.062	0.108	0.015	9.2E-13	trans
692	DPP6	P42658	Dipeptidyl aminopeptidase-like protein 6	rs3734960	154684153	7	C	T	0.262	0.261	0.008	7.6E-225	cis
692	DPP6	P42658	Dipeptidyl aminopeptidase-like protein 6	rs507666	136149399	9	G	A	0.815	0.122	0.009	4.9E-40	trans
692	DPP6	P42658	Dipeptidyl aminopeptidase-like protein 6	rs56278466	17875857	10	G	T	0.661	0.179	0.008	6.9E-124	trans
692	DPP6	P42658	Dipeptidyl aminopeptidase-like protein 6	rs2289702	79237293	15	T	C	0.106	0.093	0.012	1.0E-15	trans
693	DPP7	Q9UHL4	Dipeptidyl peptidase 2	rs35457250	186338564	3	T	C	0.009	0.374	0.036	2.6E-25	trans
693	DPP7	Q9UHL4	Dipeptidyl peptidase 2	rs13253029	17749622	8	T	C	0.691	0.088	0.007	2.3E-33	trans
693	DPP7	Q9UHL4	Dipeptidyl peptidase 2	rs9414801	65149089	10	G	A	0.466	0.062	0.007	8.5E-20	trans
693	DPP7	Q9UHL4	Dipeptidyl peptidase 2	rs145078947	93652974	14	T	G	0.003	0.540	0.066	3.3E-16	trans
693	DPP7	Q9UHL4	Dipeptidyl peptidase 2	rs147233090	44028047	15	T	C	0.023	0.164	0.022	2.3E-13	trans
693	DPP7	Q9UHL4	Dipeptidyl peptidase 2	rs58542926	19379549	19	T	C	0.075	0.148	0.013	7.9E-31	trans
694	DPT	Q07507	Dermatopontin	rs1018454	168697761	1	C	A	0.579	0.330	0.007	0.0E+00	cis
694	DPT	Q07507	Dermatopontin	rs7639267	52568805	3	T	G	0.560	0.051	0.007	1.4E-14	trans
695	DPY30	Q9C005	Protein dpy-30 homolog	rs72794684	31986545	2	G	A	0.048	0.133	0.018	3.5E-14	cis
695	DPY30	Q9C005	Protein dpy-30 homolog	rs6993770	106581528	8	A	T	0.714	0.059	0.008	8.1E-13	trans
696	DRAXIN	Q8NBI3	Draxin	rs6679089	11752140	1	G	A	0.944	0.605	0.016	3.0E-304	cis
696	DRAXIN	Q8NBI3	Draxin	rs76529516	60583370	2	G	A	0.027	0.225	0.021	5.1E-26	trans
696	DRAXIN	Q8NBI3	Draxin	rs13063578	47087837	3	T	A	0.602	0.073	0.007	2.2E-24	trans
696	DRAXIN	Q8NBI3	Draxin	rs10002742	141020165	4	G	A	0.558	0.049	0.007	6.3E-12	trans
696	DRAXIN	Q8NBI3	Draxin	rs17361097	153001219	4	A	G	0.438	0.057	0.007	5.5E-16	trans
696	DRAXIN	Q8NBI3	Draxin	rs304153	88121188	5	A	T	0.308	0.068	0.008	3.0E-19	trans
696	DRAXIN	Q8NBI3	Draxin	rs180798958	143188976	5	G	A	0.982	0.194	0.027	4.1E-13	trans
696	DRAXIN	Q8NBI3	Draxin	rs4704963	158247378	5	T	C	0.924	0.192	0.013	1.8E-47	trans
696	DRAXIN	Q8NBI3	Draxin	rs652951	44564008	6	C	A	0.508	0.054	0.007	1.7E-14	trans
696	DRAXIN	Q8NBI3	Draxin	rs9483788	135435501	6	T	C	0.743	0.058	0.008	5.9E-13	trans
696	DRAXIN	Q8NBI3	Draxin	rs60160896	56047572	7	T	C	0.413	0.061	0.007	8.7E-18	trans
696	DRAXIN	Q8NBI3	Draxin	rs62621812	127015083	7	A	G	0.021	0.272	0.025	1.2E-27	trans
696	DRAXIN	Q8NBI3	Draxin	rs2217715	130615008	8	A	C	0.571	0.080	0.007	8.4E-30	trans
696	DRAXIN	Q8NBI3	Draxin	rs1250546	81032532	10	G	A	0.439	0.048	0.007	6.6E-12	trans
696	DRAXIN	Q8NBI3	Draxin	rs11410806	81844771	15	TA	T	0.186	0.072	0.009	2.5E-15	trans
696	DRAXIN	Q8NBI3	Draxin	rs9927316	86016401	16	G	C	0.239	0.088	0.008	8.1E-27	trans
696	DRAXIN	Q8NBI3	Draxin	rs7508294	33743697	19	A	G	0.336	0.061	0.007	2.3E-16	trans
696	DRAXIN	Q8NBI3	Draxin	rs681343	49206462	19	T	C	0.510	0.063	0.007	4.5E-19	trans
697	DRG2	P55039	Developmentally-regulated GTP-binding protein 2	rs1354034	56849749	3	C	T	0.605	0.065	0.008	1.4E-17	trans
697	DRG2	P55039	Developmentally-regulated GTP-binding protein 2	rs147899431	116892681	6	C	G	0.044	0.270	0.018	1.1E-48	trans
698	DSC2	Q02487	Desmocollin-2	rs61747728	179526214	1	T	C	0.038	0.188	0.019	2.8E-23	trans
698	DSC2	Q02487	Desmocollin-2	rs8176741	136131461	9	A	G	0.061	0.130	0.015	7.0E-18	trans
698	DSC2	Q02487	Desmocollin-2	rs186021206	7069412	17	A	G	0.006	0.416	0.050	8.2E-17	trans
698	DSC2	Q02487	Desmocollin-2	rs2665405	57875292	17	A	G	0.548	0.103	0.007	1.0E-45	trans
698	DSC2	Q02487	Desmocollin-2	rs4799305	28689144	18	G	A	0.116	0.342	0.012	3.8E-193	cis
698	DSC2	Q02487	Desmocollin-2	rs3814995	36342212	19	T	C	0.315	0.089	0.008	3.7E-30	trans
698	DSC2	Q02487	Desmocollin-2	rs516316	49206145	19	G	C	0.490	0.092	0.007	1.3E-36	trans
699	DSCAM	O60469	Down syndrome cell adhesion molecule	rs148701924	158539799	3	T	A	0.997	0.453	0.067	1.4E-11	trans
699	DSCAM	O60469	Down syndrome cell adhesion molecule	rs9274454	32633371	6	T	G	0.813	0.107	0.010	1.6E-28	trans
699	DSCAM	O60469	Down syndrome cell adhesion molecule	rs56278466	17875857	10	G	T	0.661	0.177	0.008	3.0E-120	trans
699	DSCAM	O60469	Down syndrome cell adhesion molecule	rs186021206	7069412	17	A	G	0.006	0.497	0.049	6.0E-24	trans
699	DSCAM	O60469	Down syndrome cell adhesion molecule	rs78111814	42060498	21	C	T	0.038	0.950	0.020	0.0E+00	cis
700	DSG2	Q14126	Desmoglein-2	rs61747728	179526214	1	T	C	0.038	0.155	0.018	5.5E-17	trans
700	DSG2	Q14126	Desmoglein-2	rs13127398	102921704	4	A	T	0.072	0.111	0.014	1.3E-15	trans
700	DSG2	Q14126	Desmoglein-2	rs998584	43757896	6	C	A	0.520	0.084	0.007	2.4E-32	trans
700	DSG2	Q14126	Desmoglein-2	rs648802	138413333	6	G	C	0.460	0.138	0.007	4.4E-83	trans
700	DSG2	Q14126	Desmoglein-2	rs8176741	136131461	9	A	G	0.061	0.432	0.015	4.3E-182	trans
700	DSG2	Q14126	Desmoglein-2	rs2848680	29093317	18	A	G	0.524	0.218	0.007	1.1E-198	cis
700	DSG2	Q14126	Desmoglein-2	rs708686	5840619	19	T	C	0.268	0.112	0.008	3.8E-44	trans
700	DSG2	Q14126	Desmoglein-2	rs3814995	36342212	19	T	C	0.315	0.061	0.008	1.7E-15	trans
701	DSG3	P32926	Desmoglein-3	rs80293268	8207579	1	G	C	0.952	0.183	0.017	4.4E-27	trans
701	DSG3	P32926	Desmoglein-3	rs61816761	152285861	1	A	G	0.024	0.243	0.024	4.9E-24	trans
701	DSG3	P32926	Desmoglein-3	rs1260326	27730940	2	C	T	0.608	0.078	0.007	2.3E-28	trans
701	DSG3	P32926	Desmoglein-3	rs833270	181520166	3	C	T	0.617	0.060	0.007	4.3E-17	trans
701	DSG3	P32926	Desmoglein-3	rs13107325	103188709	4	T	C	0.075	0.166	0.013	1.7E-36	trans
701	DSG3	P32926	Desmoglein-3	rs7905367	54334653	10	C	G	0.797	0.073	0.009	6.6E-18	trans
701	DSG3	P32926	Desmoglein-3	rs683486	126226663	11	A	C	0.403	0.064	0.007	4.0E-19	trans
701	DSG3	P32926	Desmoglein-3	rs34177108	89893375	16	A	C	0.273	0.058	0.008	9.3E-14	trans
701	DSG3	P32926	Desmoglein-3	rs186021206	7069412	17	A	G	0.006	0.795	0.047	2.7E-63	trans
701	DSG3	P32926	Desmoglein-3	rs7234091	29019829	18	C	T	0.819	0.308	0.009	8.1E-252	cis
701	DSG3	P32926	Desmoglein-3	rs12457893	60926161	18	A	C	0.542	0.049	0.007	4.5E-12	trans

701	DSG3	P32926	Desmoglein-3	rs3814995	36342212	19	T	C	0.315	0.066	0.007	6.1E-19	trans
701	DSG3	P32926	Desmoglein-3	rs2682581	43966506	19	C	G	0.471	0.071	0.007	4.5E-25	trans
702	DSG4	Q86SJ6	Desmoglein-4	rs80293268	8207579	1	G	C	0.952	0.209	0.018	1.1E-31	trans
702	DSG4	Q86SJ6	Desmoglein-4	rs1707115	117873222	1	T	C	0.752	0.058	0.008	8.5E-12	trans
702	DSG4	Q86SJ6	Desmoglein-4	rs11589479	155033308	1	G	A	0.837	0.078	0.010	4.4E-15	trans
702	DSG4	Q86SJ6	Desmoglein-4	rs1260326	27730940	2	C	T	0.608	0.062	0.007	1.2E-16	trans
702	DSG4	Q86SJ6	Desmoglein-4	rs1345417	181511951	3	G	C	0.604	0.082	0.008	3.9E-27	trans
702	DSG4	Q86SJ6	Desmoglein-4	rs13107325	103188709	4	T	C	0.075	0.121	0.014	2.7E-18	trans
702	DSG4	Q86SJ6	Desmoglein-4	rs190638675	53094074	5	C	T	0.878	0.083	0.011	1.5E-13	trans
702	DSG4	Q86SJ6	Desmoglein-4	rs2436396	139533563	5	A	G	0.299	0.087	0.008	1.9E-27	trans
702	DSG4	Q86SJ6	Desmoglein-4	rs12203592	396321	6	C	T	0.782	0.069	0.009	1.8E-14	trans
702	DSG4	Q86SJ6	Desmoglein-4	rs9358325	10259737	6	A	T	0.169	0.080	0.010	2.1E-16	trans
702	DSG4	Q86SJ6	Desmoglein-4	rs1918719	117305398	8	T	C	0.804	0.082	0.009	2.4E-19	trans
702	DSG4	Q86SJ6	Desmoglein-4	rs7905367	54334653	10	C	G	0.797	0.102	0.009	1.2E-29	trans
702	DSG4	Q86SJ6	Desmoglein-4	rs9896243	44826056	17	G	C	0.216	0.096	0.009	1.9E-27	trans
702	DSG4	Q86SJ6	Desmoglein-4	rs55842605	76937691	17	T	C	0.553	0.071	0.007	3.1E-22	trans
702	DSG4	Q86SJ6	Desmoglein-4	rs34620697	28983529	18	C	T	0.994	0.368	0.047	5.8E-15	cis
702	DSG4	Q86SJ6	Desmoglein-4	rs12457893	60926161	18	A	C	0.542	0.078	0.007	4.5E-26	trans
702	DSG4	Q86SJ6	Desmoglein-4	rs10417997	41902361	19	G	A	0.395	0.051	0.007	5.8E-12	trans
702	DSG4	Q86SJ6	Desmoglein-4	rs2473881	66593211	X	A	G	0.175	0.062	0.008	4.0E-15	trans
703	DTD1	Q8TEA8	D-aminoacyl-tRNA deacylase 1	rs1354034	56849749	3	C	T	0.604	0.079	0.008	1.3E-24	trans
703	DTD1	Q8TEA8	D-aminoacyl-tRNA deacylase 1	rs6993770	106581528	8	A	T	0.713	0.061	0.008	1.7E-13	trans
703	DTD1	Q8TEA8	D-aminoacyl-tRNA deacylase 1	rs6081238	18577212	20	G	A	0.624	0.166	0.008	2.6E-100	cis
704	DTX3	Q8N9I9	Probable E3 ubiquitin-protein ligase DTX3	rs542212699	223900610	1	G	A	0.991	0.680	0.037	1.5E-73	trans
704	DTX3	Q8N9I9	Probable E3 ubiquitin-protein ligase DTX3	rs151828	96060262	5	C	T	0.547	0.157	0.007	1.4E-111	trans
704	DTX3	Q8N9I9	Probable E3 ubiquitin-protein ligase DTX3	rs2232659	105209413	10	T	C	0.017	0.184	0.027	4.8E-12	trans
705	DTYMK	P23919	Thymidylate kinase	rs28630677	242625961	2	C	T	0.958	0.138	0.019	7.7E-14	cis
705	DTYMK	P23919	Thymidylate kinase	rs1354034	56849749	3	C	T	0.604	0.066	0.008	2.7E-18	trans
705	DTYMK	P23919	Thymidylate kinase	rs6993770	106581528	8	A	T	0.713	0.060	0.008	3.3E-13	trans
706	DUSP13	Q6B8I1	Dual specificity protein phosphatase 13 isoform A	rs6480771	76861680	10	T	C	0.589	0.265	0.008	1.2E-264	cis
707	DUSP29	Q68J44	Dual specificity phosphatase 29	rs4745762	76828323	10	C	A	0.661	0.140	0.008	1.8E-67	cis
708	DUSP3	P51452	Dual specificity protein phosphatase 3	rs7080386	65048306	10	A	C	0.412	0.066	0.008	4.5E-17	trans
708	DUSP3	P51452	Dual specificity protein phosphatase 3	rs141728140	106363115	14	A	G	0.161	0.093	0.011	1.3E-17	trans
709	DUT	P33316	Deoxyuridine 5'-triphosphate nucleotidohydrolase, mitochondrial	rs1354034	56849749	3	C	T	0.605	0.066	0.008	6.8E-18	trans
709	DUT	P33316	Deoxyuridine 5'-triphosphate nucleotidohydrolase, mitochondrial	rs10075801	131677642	5	A	G	0.512	0.058	0.008	3.3E-14	trans
709	DUT	P33316	Deoxyuridine 5'-triphosphate nucleotidohydrolase, mitochondrial	rs6993770	106581528	8	A	T	0.713	0.069	0.008	1.1E-16	trans
709	DUT	P33316	Deoxyuridine 5'-triphosphate nucleotidohydrolase, mitochondrial	rs247617	56990716	16	C	A	0.676	0.057	0.008	1.5E-12	trans
709	DUT	P33316	Deoxyuridine 5'-triphosphate nucleotidohydrolase, mitochondrial	rs892090	55539072	19	G	T	0.834	0.071	0.010	3.6E-12	trans
710	DXO	O77932	Decapping and exoribonuclease protein	rs45531831	31938120	6	C	T	0.971	1.087	0.022	0.0E+00	cis
710	DXO	O77932	Decapping and exoribonuclease protein	rs342298	106373646	7	C	T	0.545	0.053	0.007	2.6E-13	trans
710	DXO	O77932	Decapping and exoribonuclease protein	rs6993770	106581528	8	A	T	0.713	0.068	0.008	1.4E-17	trans
711	DYNC1H1	Q14204	Cytoplasmic dynein 1 heavy chain 1	rs1339847	248039294	1	A	G	0.109	0.371	0.012	3.0E-203	trans
711	DYNC1H1	Q14204	Cytoplasmic dynein 1 heavy chain 1	rs1354034	56849749	3	C	T	0.604	0.072	0.008	9.1E-21	trans
712	DYNLT1	P63172	Dynein light chain Tctex-type 1	rs61840351	249058569	1	C	G	0.147	0.121	0.011	8.6E-29	trans
712	DYNLT1	P63172	Dynein light chain Tctex-type 1	rs1339847	248039294	1	A	G	0.109	1.041	0.012	0.0E+00	trans
712	DYNLT1	P63172	Dynein light chain Tctex-type 1	rs78909033	241510903	2	G	A	0.864	0.065	0.010	8.4E-12	trans
712	DYNLT1	P63172	Dynein light chain Tctex-type 1	rs1354034	56849749	3	C	T	0.604	0.225	0.007	2.0E-245	trans
712	DYNLT1	P63172	Dynein light chain Tctex-type 1	rs2306751	159065758	6	G	C	0.579	0.081	0.007	5.5E-34	cis
712	DYNLT1	P63172	Dynein light chain Tctex-type 1	rs1824872	95401698	7	T	C	0.293	0.050	0.007	2.1E-12	trans
712	DYNLT1	P63172	Dynein light chain Tctex-type 1	rs139141690	101499930	7	A	G	0.005	0.356	0.048	1.9E-13	trans
712	DYNLT1	P63172	Dynein light chain Tctex-type 1	rs342296	106372903	7	G	A	0.543	0.063	0.007	6.6E-22	trans
712	DYNLT1	P63172	Dynein light chain Tctex-type 1	rs11604127	196944	11	T	C	0.231	0.097	0.008	1.4E-35	trans
712	DYNLT1	P63172	Dynein light chain Tctex-type 1	rs5963356	37732384	X	C	T	0.967	0.230	0.015	5.0E-51	trans
713	DYNLT3	P51808	Dynein light chain Tctex-type 3	rs1339847	248039294	1	A	G	0.109	0.387	0.012	1.8E-225	trans
713	DYNLT3	P51808	Dynein light chain Tctex-type 3	rs1354034	56849749	3	C	T	0.604	0.108	0.008	4.2E-46	trans
714	EBAG9	O00559	Receptor-binding cancer antigen expressed on SiSo cells	rs1354034	56849749	3	C	T	0.604	0.080	0.008	1.1E-25	trans
714	EBAG9	O00559	Receptor-binding cancer antigen expressed on SiSo cells	rs11950562	131652529	5	A	C	0.521	0.052	0.008	5.7E-12	trans
714	EBAG9	O00559	Receptor-binding cancer antigen expressed on SiSo cells	rs342293	106372219	7	C	G	0.541	0.052	0.008	4.4E-12	trans
714	EBAG9	O00559	Receptor-binding cancer antigen expressed on SiSo cells	rs6993770	106581528	8	A	T	0.713	0.070	0.008	5.4E-17	trans
714	EBAG9	O00559	Receptor-binding cancer antigen expressed on SiSo cells	rs2022934	110567774	8	G	A	0.614	0.069	0.008	6.5E-19	cis
715	EBI3_IL27	Q14213,Q8NEV9	Interleukin-27	rs4970836	109821797	1	A	G	0.773	0.071	0.008	8.4E-18	trans
715	EBI3_IL27	Q14213,Q8NEV9	Interleukin-27	rs10183338	111610816	2	G	T	0.708	0.054	0.008	1.8E-12	trans
715	EBI3_IL27	Q14213,Q8NEV9	Interleukin-27	rs62165726	134966562	2	C	A	0.963	0.277	0.018	1.9E-51	trans
715	EBI3_IL27	Q14213,Q8NEV9	Interleukin-27	rs11456863	12271918	3	AT	A	0.352	0.051	0.007	2.8E-12	trans
715	EBI3_IL27	Q14213,Q8NEV9	Interleukin-27	rs4234589	185818882	3	G	A	0.139	0.070	0.010	3.9E-12	trans
715	EBI3_IL27	Q14213,Q8NEV9	Interleukin-27	rs11713634	194061578	3	G	A	0.721	0.131	0.008	6.7E-65	trans
715	EBI3_IL27	Q14213,Q8NEV9	Interleukin-27	rs5743618	38798648	4	A	C	0.224	0.116	0.008	2.0E-43	trans
715	EBI3_IL27	Q14213,Q8NEV9	Interleukin-27	rs4713572	32626952	6	C	T	0.400	0.066	0.007	1.3E-20	trans
715	EBI3_IL27	Q14213,Q8NEV9	Interleukin-27	rs2529440	30511794	7	T	C	0.444	0.051	0.007	2.9E-13	trans
715	EBI3_IL27	Q14213,Q8NEV9	Interleukin-27	rs339060	128375603	7	G	A	0.553	0.062	0.007	4.9E-19	trans
715	EBI3_IL27	Q14213,Q8NEV9	Interleukin-27	rs4986790	120475302	9	A	G	0.940	0.118	0.015	6.8E-16	trans
715	EBI3_IL27	Q14213,Q8NEV9	Interleukin-27	rs4486555	101799302	10	G	T	0.590	0.194	0.007	2.8E-164	trans
715	EBI3_IL27	Q14213,Q8NEV9	Interleukin-27	rs6589939	122518525	11	A	G	0.622	0.058	0.007	2.8E-16	trans
715	EBI3_IL27	Q14213,Q8NEV9	Interleukin-27	rs186021206	7069412	17	A	G	0.006	0.826	0.048	1.9E-67	trans
715	EBI3_IL27	Q14213,Q8NEV9	Interleukin-27	rs704	26694861	17	G	A	0.527	0.098	0.007	2.7E-45	trans
715	EBI3_IL27	Q14213	Interleukin-27	rs8109160	4247476	19	C	T	0.710	0.413	0.008	0.0E+00	cis
715	EBI3_IL27	Q14213,Q8NEV9	Interleukin-27	rs4804669	12502457	19	A	G	0.220	0.099	0.009	8.1E-30	trans
715	EBI3_IL27	Q14213,Q8NEV9	Interleukin-27	rs7264079	34154937	20	C	G	0.137	0.098	0.010	1.8E-22	trans
716	ECE1	P42892	Endothelin-converting enzyme 1	rs181637959	21546033	1	A	T	0.994	1.028	0.048	5.7E-101	cis
716	ECE1	P42892	Endothelin-converting enzyme 1	rs181242111	17865664	10	A	G	0.118	0.086	0.013	7.2E-12	trans
716	ECE1	P42892	Endothelin-converting enzyme 1	rs35166255	126301756	11	A	G	0.034	0.227	0.021	2.2E-27	trans
716	ECE1	P42892	Endothelin-converting enzyme 1	rs186021206	7069412	17	A	G	0.006	0.538	0.052	3.1E-25	trans
717	ECHDC3	Q96DC8	Enoyl-CoA hydratase domain-containing protein 3, mitochondrial	rs1497406	16505320	1	G	A	0.580	0.047	0.007	7.3E-12	trans
717	ECHDC3	Q96DC8	Enoyl-CoA hydratase domain-containing protein 3, mitochondrial	rs1260326	27730940	2	T	C	0.391	0.051	0.007	2.9E-13	trans
717	ECHDC3	Q96DC8	Enoyl-CoA hydratase domain-containing protein 3, mitochondrial	rs4835265	146821410	4	A	C	0.156	0.116	0.009	2.0E-34	trans
717	ECHDC3	Q96DC8	Enoyl-CoA hydratase domain-containing protein 3, mitochondrial	rs72781910	11802849	10	G	A	0.908	0.955	0.013	0.0E+00	cis
717	ECHDC3	Q96DC8	Enoyl-CoA hydratase domain-containing protein 3, mitochondrial	rs12373325	56087648	18	T	C	0.202	0.086	0.009	1.0E-23	trans
718	ECHS1	P30084	Enoyl-CoA hydratase, mitochondrial	rs6993770	106581528	8	A	T	0.713	0.057	0.008	7.1E-12	trans
718	ECHS1	P30084	Enoyl-CoA hydratase, mitochondrial	rs1049951	135184126	10	G	A	0.056	0.447	0.017	2.8E-160	cis
719	ECI2	O75521	Enoyl-CoA delta isomerase 2	rs660560	4125941	6	A	G	0.675	0.278	0.008	5.3E-249	cis
720	ECM1	Q16610	Extracellular matrix protein 1	rs78448142	146844998	1	C	T	0.968	0.148	0.022	1.1E-11	trans
720	ECM1	Q16610	Extracellular matrix protein 1	rs3737240	150483355	1	C	T	0.606	0.677	0.008	0.0E+00	cis
720	ECM1	Q16610	Extracellular matrix protein 1	rs111856038	124745030	4	A	G	0.108	0.075	0.011	1.0E-11	trans
721	EDA2R	Q9HAV5	Tumor necrosis factor receptor superfamily member 27	rs4335411	249191706	1	G	A	0.240	0.062	0.007	2.6E-17	trans
721	EDA2R	Q9HAV5	Tumor necrosis factor receptor superfamily member 27	rs10069690	1279790	5	C	T	0.741	0.050	0.007	8.7E-14	trans
721	EDA2R	Q9HAV5	Tumor necrosis factor receptor superfamily member 27	rs78378222	7571752	17	T	G	0.987	0.260	0.027	2.8E-22	trans
721	EDA2R	Q9HAV5	Tumor necrosis factor receptor superfamily member 27	rs73221564	65954295	X	C	T	0.991	1.304	0.026	0.0E+00	cis
722	EDAR	Q9UNE0	Tumor necrosis factor receptor superfamily member EDAR	rs72665955	54841656	1	A	G	0.119	0.129	0.011	9.7E-32	trans
722	EDAR	Q9UNE0	Tumor necrosis factor receptor superfamily member EDAR	rs12712870	43161180	2	G	A	0.690	0.092	0.008	4.6E-31	trans
722	EDAR	Q9UNE0	Tumor necrosis factor receptor superfamily member EDAR	rs140661471	109603739	2	A	G	0.014	1.660	0.032	0.0E+00	cis
722	EDAR	Q9UNE0	Tumor necrosis factor receptor superfamily member EDAR	rs28362642	39149503	3	G	A	0.112	0.078	0.011	7.1E-12	trans
722	EDAR	Q9UNE0	Tumor necrosis factor receptor superfamily member EDAR	rs1420476	168864550	3	T	A	0.045	0.141	0.017	3.0E-16	trans
722	EDAR	Q9UNE0	Tumor necrosis factor receptor superfamily member EDAR	rs10446711	105970284	4	G	A	0.174	0.065	0.009	5.2E-12	trans
722	EDAR	Q9UNE0	Tumor necrosis factor receptor superfamily member EDAR	rs114694170	88180196	5	T	C	0.940	0.206	0.015	5.6E-42	trans
722	EDAR	Q9UNE0	Tumor necrosis factor receptor superfamily member EDAR	rs139141690	101499930	7	G	A	0.995	0.379	0.053	5.9E-13	trans
722	EDAR	Q9UNE0	Tumor necrosis factor receptor superfamily member EDAR	rs67036916	106367636	7	A	G	0.456	0.072	0.007	1.2E-23	trans
722	EDAR	Q9UNE0	Tumor necrosis factor receptor superfamily member EDAR	rs6993770	106581528	8	T	A	0.287	0.227	0.008	5.6E-180	trans
722	EDAR	Q9UNE0	Tumor necrosis factor receptor superfamily member EDAR	rs4742045	4746363	9	T	G	0.587	0.053	0.007	2.4E-13	trans
722	EDAR	Q9UNE0	Tumor necrosis factor receptor superfamily member EDAR	rs10820606	99192919	9	A	C	0.771	0.060	0.009	3.6E-12	trans
722	EDAR	Q9UNE0	Tumor necrosis factor receptor superfamily member EDAR	rs10821548	136937263	9	C	T	0.676	0.106	0.008	2.5E-44	trans
722	EDAR	Q9UNE0	Tumor necrosis factor receptor superfamily member EDAR	rs7080386	65048306	10	A	C	0.412	0.117	0.007	3.1E-58	trans
722	EDAR	Q9UNE0	Tumor necrosis factor receptor superfamily member EDAR	rs12762934	104359884	10	C	T	0.675	0.147	0.008	1.2E-82	trans
722	EDAR	Q9UNE0	Tumor necrosis factor receptor superfamily member EDAR	rs652963	32911737	11	T	C	0.843	0.084	0.010	9.8E-18	trans
722	EDAR	Q9UNE0	Tumor necrosis factor receptor superfamily member EDAR	rs1820994	57297779	15	G	A	0.787	0.104	0.009	6.9E-33	trans
722	EDAR	Q9UNE0	Tumor necrosis factor receptor superfamily member EDAR	rs8057254	9052989	16	A	T	0.190	0.077	0.009	5.3E-17	trans
722	EDAR	Q9UNE0	Tumor necrosis factor receptor superfamily member EDAR	rs74035509	88567333	16	C	T	0.922	0.205	0.013	1.2E-52	trans
722	EDAR	Q9UNE0	Tumor necrosis factor receptor superfamily member EDAR	rs111527738	36259324	21	A	G	0.979	0.235	0.025	7.1E-21	trans
723	EDDM3B	P56851	Epididymal secretory protein E3-beta	rs3827905	21238318	14	G	A	0.332	0.160	0.005	1.4E-203	cis
724	EDIL3	O43854	EGF-like repeat and discoidin I-like domain-containing protein 3	rs78606665	66146314	17	A	G	0.061	0.108	0.016	5.6E-12	trans
724	EDIL3	O43854	EGF-like repeat and discoidin I-like domain-containing protein 3	rs112001035	66823805	17	A	G	0.061	0.259	0.016	2.2E-59	trans
725	EDN1	P05305	Endothelin-1	rs5370	12296255	6	T	G	0.220	0.272	0.009	5.3E-198	cis
726	EEF1D	P29692	Elongation factor 1-delta	rs3811444	248039451	1	C	T	0.668	0.123	0.008	2.5E-55	trans
726	EEF1D	P29692	Elongation factor 1-delta	rs1354034	56849749	3	C	T	0.604	0.080	0.008	1.8E-26	trans
726	EEF1D	P29692	Elongation factor 1-delta	rs342298	106373646	7	C	T	0.545	0.050	0.007	1.1E-11	trans
727	EFCAB14	O75071	EF-hand calcium-binding domain-containing protein 14	rs35117964	32568146	6	A	G	0.809	0.102	0.010	3.7E-26	trans
727	EFCAB14	O75071	EF-hand calcium-binding domain-containing protein 14	rs7166599	91403674	15	G	A	0.334	0.056	0.008	5.5E-13	trans
728	EFCAB2	Q5VUJ9	Dynein regulatory complex protein 8	rs61844237	245133662	1	G	C	0.531	0.384	0.008	0.0E+00	cis
728	EFCAB2	Q5VUJ9	Dynein regulatory complex protein 8	rs1354034	56849749	3	C	T	0.604	0.106	0.007	2.7E-45	trans
728	EFCAB2	Q5VUJ9	Dynein regulatory complex protein 8	rs342298	106373646	7	C	T	0.545	0.056	0.007	4.7E-14	trans
728	EFCAB2	Q5VUJ9	Dynein regulatory complex protein 8	rs11502185	180258	11	C	T	0.260	0.076	0.009	9.6E-16	trans
729	EFEMP1	Q12805	EGF-containing fibulin-like extracellular matrix protein 1	rs188468174	25291697	1	C	T	0.986	0.244	0.029	5.7E-17	trans
729	EFEMP1	Q12805	EGF-containing fibulin-like extracellular matrix protein 1	rs1260326	27730940	2	C	T	0.608	0.053	0.007	3.6E-14	trans
729	EFEMP1	Q12805	EGF-containing fibulin-like extracellular matrix protein 1	rs3791679	56096892	2	G	A	0.226	0.226	0.008	1.2E-166	cis
729	EFEMP1	Q12805	EGF-containing fibulin-like extracellular matrix protein 1	rs2070634	186336027	3	G	T	0.515	0.050	0.007	1.5E-13	trans
730	EFHD1	Q9BUP0	EF-hand domain-containing protein D1	rs146096867	233520579	2	T	C	0.084	0.325	0.013	4.0E-137	cis
731	EFNA1	P20827	Ephrin-A1	rs4390169	155106054	1	G	A	0.519	0.317	0.008	0.0E+00	cis
731	EFNA1	P20827	Ephrin-A1	rs174578	61605499	11	A	T	0.353	0.055	0.008	4.3E-13	trans
731	EFNA1	P20827	Ephrin-A1	rs28929474	94844947	14	T	C	0.021	0.184	0.026	6.7E-13	trans
732	EFNA4	P52798	Ephrin-A4	rs6737671	222386932	2	C	T	0.513	0.059	0.007	1.3E-15	trans
732	EFNA4	P52798	Ephrin-A4	rs7187875	413747	16	A	G	0.459	0.060	0.007	4.2E-16	trans
732	EFNA4	P52798	Ephrin-A4	rs12934455	20357281	16	C	T	0.833	0.078	0.010	8.8E-15	trans
732	EFNA4	P52798	Ephrin-A4	rs4760	44153100	19	G	A	0.156	0.089	0.010	1.6E-18	trans
733	EFNB2	P52799	Ephrin-B2	rs2845705	120101092	11	T	A	0.797	0.186	0.010	3.5E-83	trans

734	EGF	P01133	Pro-epidermal growth factor	rs3831508	110837903	4	GT	G	0.402	0.134	0.008	4.0E-67	cis
734	EGF	P01133	Pro-epidermal growth factor	rs2631367	131705458	5	G	C	0.518	0.063	0.008	7.9E-17	trans
734	EGF	P01133	Pro-epidermal growth factor	rs6961069	80218961	7	T	C	0.402	0.065	0.008	3.1E-17	trans
734	EGF	P01133	Pro-epidermal growth factor	rs6993770	106581528	8	A	T	0.713	0.095	0.008	3.6E-30	trans
734	EGF	P01133	Pro-epidermal growth factor	rs10820606	99192919	9	A	C	0.770	0.069	0.009	3.0E-14	trans
734	EGF	P01133	Pro-epidermal growth factor	rs7896518	65104500	10	G	A	0.424	0.089	0.008	9.1E-31	trans
734	EGF	P01133	Pro-epidermal growth factor	rs12445050	81870969	16	T	C	0.138	0.091	0.011	7.1E-17	trans
734	EGF	P01133	Pro-epidermal growth factor	rs892090	55539072	19	G	T	0.834	0.147	0.010	4.3E-47	trans
734	EGF	P01133	Pro-epidermal growth factor	rs6081565	19287904	20	A	G	0.353	0.061	0.008	1.5E-14	trans
735	EGFL7	Q9UHF1	Epidermal growth factor-like protein 7	rs78945826	26057416	1	A	C	0.969	0.152	0.022	6.8E-12	trans
735	EGFL7	Q9UHF1	Epidermal growth factor-like protein 7	rs823066	205771173	1	A	G	0.051	0.115	0.017	3.5E-12	trans
735	EGFL7	Q9UHF1	Epidermal growth factor-like protein 7	rs78909033	241510903	2	G	A	0.864	0.074	0.011	3.0E-12	trans
735	EGFL7	Q9UHF1	Epidermal growth factor-like protein 7	rs114694170	88180196	5	C	T	0.060	0.108	0.015	3.0E-12	trans
735	EGFL7	Q9UHF1	Epidermal growth factor-like protein 7	rs3131622	31420500	6	G	T	0.450	0.066	0.007	1.2E-19	trans
735	EGFL7	Q9UHF1	Epidermal growth factor-like protein 7	rs6796	6502367	7	T	C	0.721	0.166	0.008	7.9E-92	trans
735	EGFL7	Q9UHF1	Epidermal growth factor-like protein 7	rs10259992	35697347	7	G	A	0.626	0.083	0.008	1.9E-28	trans
735	EGFL7	Q9UHF1	Epidermal growth factor-like protein 7	rs17154155	80234243	7	T	G	0.407	0.051	0.007	4.1E-12	trans
735	EGFL7	Q9UHF1	Epidermal growth factor-like protein 7	rs339061	128373828	7	T	C	0.445	0.056	0.007	3.0E-14	trans
735	EGFL7	Q9UHF1	Epidermal growth factor-like protein 7	rs883006	33294427	8	C	G	0.438	0.069	0.007	8.3E-21	trans
735	EGFL7	Q9UHF1	Epidermal growth factor-like protein 7	rs8193003	135865496	9	C	T	0.608	0.058	0.008	2.0E-14	trans
735	EGFL7	Q9UHF1	Epidermal growth factor-like protein 7	rs74557797	139550503	9	G	T	0.907	0.285	0.014	6.9E-99	cis
735	EGFL7	Q9UHF1	Epidermal growth factor-like protein 7	rs10761731	65027610	10	T	A	0.415	0.068	0.007	4.9E-20	trans
735	EGFL7	Q9UHF1	Epidermal growth factor-like protein 7	rs55646096	64918514	11	C	T	0.823	0.163	0.010	4.9E-65	trans
735	EGFL7	Q9UHF1	Epidermal growth factor-like protein 7	rs9635249	65797784	14	G	A	0.794	0.076	0.009	9.6E-17	trans
735	EGFL7	Q9UHF1	Epidermal growth factor-like protein 7	rs74035509	88567333	16	T	C	0.078	0.107	0.014	4.6E-15	trans
735	EGFL7	Q9UHF1	Epidermal growth factor-like protein 7	rs892090	55539072	19	G	T	0.834	0.115	0.010	1.0E-31	trans
736	EGFLAM	Q63HQ2	Pikachurin	rs113400125	38406251	5	T	C	0.026	1.456	0.023	0.0E+00	cis
736	EGFLAM	Q63HQ2	Pikachurin	rs628064	29807966	18	A	T	0.543	0.050	0.007	8.7E-14	trans
736	EGFLAM	Q63HQ2	Pikachurin	rs12975366	54759361	19	C	T	0.395	0.089	0.007	3.7E-39	trans
737	EGFR	P00533	Epidermal growth factor receptor	rs10081281	55236796	7	T	C	0.453	0.171	0.007	8.5E-121	cis
737	EGFR	P00533	Epidermal growth factor receptor	rs3967200	126232385	11	C	T	0.865	0.090	0.011	2.5E-17	trans
737	EGFR	P00533	Epidermal growth factor receptor	rs186021206	7069412	17	A	G	0.006	0.622	0.049	2.6E-36	trans
738	EGLN1	Q9GZT9	Egl nine homolog 1	rs1434282	199010721	1	T	C	0.726	0.083	0.008	1.7E-23	trans
738	EGLN1	Q9GZT9	Egl nine homolog 1	rs77466488	231624369	1	G	A	0.036	0.337	0.020	6.6E-65	cis
738	EGLN1	Q9GZT9	Egl nine homolog 1	rs915652	31749142	6	G	A	0.869	0.075	0.011	4.4E-12	trans
738	EGLN1	Q9GZT9	Egl nine homolog 1	rs342296	106372903	7	G	A	0.544	0.076	0.007	3.9E-25	trans
738	EGLN1	Q9GZT9	Egl nine homolog 1	rs3002416	39710195	X	T	C	0.409	0.058	0.006	1.0E-19	trans
739	EHBP1	Q8NDI1	EH domain-binding protein 1	rs1354034	56849749	3	C	T	0.604	0.055	0.008	2.8E-13	trans
739	EHBP1	Q8NDI1	EH domain-binding protein 1	rs6993770	106581528	8	A	T	0.713	0.071	0.008	2.0E-18	trans
740	EHD3	Q9NZN3	EH domain-containing protein 3	rs592039	31480967	2	G	A	0.291	0.193	0.009	1.1E-113	cis
740	EHD3	Q9NZN3	EH domain-containing protein 3	rs10820606	99192919	9	A	C	0.771	0.078	0.009	3.4E-17	trans
741	EIF1AX	P47813	Eukaryotic translation initiation factor 1A, X-chromosomal	rs9271459	32588457	6	T	C	0.498	0.074	0.008	6.7E-20	trans
741	EIF1AX	P47813	Eukaryotic translation initiation factor 1A, X-chromosomal	rs12895651	106494269	14	T	C	0.299	0.064	0.009	2.7E-12	trans
742	EIF2AK2	P19525	Interferon-induced, double-stranded RNA-activated protein kinase	rs10187017	37386731	2	C	T	0.473	0.150	0.008	2.1E-88	cis
742	EIF2AK2	P19525	Interferon-induced, double-stranded RNA-activated protein kinase	rs1354034	56849749	3	C	T	0.605	0.083	0.008	1.2E-27	trans
743	EIF2AK3	Q9NZJ5	Eukaryotic translation initiation factor 2-alpha kinase 3	rs6547787	88929736	2	T	G	0.660	0.121	0.008	7.8E-52	cis
743	EIF2AK3	Q9NZJ5	Eukaryotic translation initiation factor 2-alpha kinase 3	rs1354034	56849749	3	C	T	0.604	0.071	0.008	3.9E-20	trans
743	EIF2AK3	Q9NZJ5	Eukaryotic translation initiation factor 2-alpha kinase 3	rs6993770	106581528	8	A	T	0.714	0.062	0.008	7.6E-14	trans
744	EIF4B	P23588	Eukaryotic translation initiation factor 4B	rs1354034	56849749	3	C	T	0.604	0.084	0.008	4.9E-28	trans
744	EIF4B	P23588	Eukaryotic translation initiation factor 4B	rs342298	106373646	7	C	T	0.546	0.059	0.008	7.6E-15	trans
744	EIF4B	P23588	Eukaryotic translation initiation factor 4B	rs10733789	64948684	10	C	T	0.313	0.060	0.008	1.4E-13	trans
744	EIF4B	P23588	Eukaryotic translation initiation factor 4B	rs11604127	196944	11	T	C	0.231	0.069	0.009	2.2E-14	trans
745	EIF4E	P06730	Eukaryotic translation initiation factor 4E	rs1354034	56849749	3	C	T	0.604	0.065	0.008	3.3E-17	trans
745	EIF4E	P06730	Eukaryotic translation initiation factor 4E	rs342298	106373646	7	C	T	0.545	0.056	0.008	1.3E-13	trans
745	EIF4E	P06730	Eukaryotic translation initiation factor 4E	rs6993770	106581528	8	A	T	0.713	0.063	0.008	6.5E-14	trans
746	EIF4G1	Q04637	Eukaryotic translation initiation factor 4 gamma 1	rs1354034	56849749	3	C	T	0.604	0.091	0.008	1.4E-33	trans
746	EIF4G1	Q04637	Eukaryotic translation initiation factor 4 gamma 1	rs342298	106373646	7	C	T	0.545	0.058	0.007	8.9E-15	trans
746	EIF4G1	Q04637	Eukaryotic translation initiation factor 4 gamma 1	rs6993770	106581528	8	A	T	0.713	0.057	0.008	4.6E-12	trans
747	EIF4G3	O43432	Eukaryotic translation initiation factor 4 gamma 3	rs6993770	106581528	8	A	T	0.714	0.062	0.008	5.3E-14	trans
748	EIF5	P55010	Eukaryotic translation initiation factor 5	rs138711694	103812439	14	T	C	0.982	0.250	0.031	3.7E-16	cis
749	ELAC1	Q9H777	Zinc phosphodiesterase ELAC protein 1	rs1354034	56849749	3	C	T	0.604	0.100	0.008	1.4E-39	trans
749	ELAC1	Q9H777	Zinc phosphodiesterase ELAC protein 1	rs6993770	106581528	8	A	T	0.714	0.080	0.008	6.3E-22	trans
749	ELAC1	Q9H777	Zinc phosphodiesterase ELAC protein 1	rs11502185	180258	11	C	T	0.260	0.081	0.010	2.9E-17	trans
749	ELAC1	Q9H777	Zinc phosphodiesterase ELAC protein 1	rs142132015	48510696	18	T	C	0.995	0.861	0.054	1.1E-57	cis
749	ELAC1	Q9H777	Zinc phosphodiesterase ELAC protein 1	rs892090	55539072	19	G	T	0.834	0.069	0.010	1.1E-11	trans
750	ELN	P15502	Elastin	rs4252125	161152240	6	A	G	0.295	0.061	0.006	2.7E-23	trans
750	ELN	P15502	Elastin	rs17855988	73474825	7	C	G	0.100	0.144	0.009	1.8E-52	cis
750	ELN	P15502	Elastin	rs964184	116648917	11	G	C	0.134	0.084	0.008	9.1E-25	trans
750	ELN	P15502	Elastin	rs28929474	94844947	14	T	C	0.021	0.210	0.019	3.6E-27	trans
750	ELN	P15502	Elastin	rs72802342	75234872	16	A	C	0.076	0.159	0.011	9.9E-50	trans
750	ELN	P15502	Elastin	rs584007	45416478	19	G	A	0.642	0.043	0.006	8.6E-14	trans
750	ELN	P15502	Elastin	rs2569488	51579646	19	T	C	0.683	0.064	0.006	5.7E-26	trans
751	ELOA	Q14241	Elongin-A	rs35863927	24051934	1	C	T	0.597	0.086	0.008	3.9E-30	cis
751	ELOA	Q14241	Elongin-A	rs10418046	54327869	19	G	T	0.211	0.143	0.009	5.0E-57	trans
752	ENAH	Q8N8S7	Protein enabled homolog	rs10799316	225668031	1	A	G	0.755	0.124	0.009	1.8E-44	cis
753	ENDOU	P21128	Poly(U)-specific endoribonuclease	rs78924983	8204596	1	C	T	0.949	0.129	0.016	5.8E-16	trans
753	ENDOU	P21128	Poly(U)-specific endoribonuclease	rs61816761	152285861	1	A	G	0.023	0.247	0.023	1.8E-26	trans
753	ENDOU	P21128	Poly(U)-specific endoribonuclease	rs12029741	218848361	1	A	G	0.353	0.053	0.007	4.7E-14	trans
753	ENDOU	P21128	Poly(U)-specific endoribonuclease	rs955658	60501421	2	C	T	0.588	0.120	0.007	2.2E-70	trans
753	ENDOU	P21128	Poly(U)-specific endoribonuclease	rs121908120	219755011	2	T	A	0.973	0.168	0.021	2.7E-16	trans
753	ENDOU	P21128	Poly(U)-specific endoribonuclease	rs12203057	1687710	6	C	T	0.606	0.055	0.007	3.2E-16	trans
753	ENDOU	P21128	Poly(U)-specific endoribonuclease	rs7808585	73240296	7	T	C	0.758	0.068	0.008	7.8E-19	trans
753	ENDOU	P21128	Poly(U)-specific endoribonuclease	rs35673728	41047777	15	T	C	0.937	0.103	0.014	4.6E-14	trans
753	ENDOU	P21128	Poly(U)-specific endoribonuclease	rs34560261	90734426	15	C	T	0.831	0.113	0.009	6.7E-36	trans
753	ENDOU	P21128	Poly(U)-specific endoribonuclease	rs9939286	31162303	16	T	G	0.366	0.057	0.007	3.1E-16	trans
754	ENG	P17813	Endoglin	rs61762319	154801978	3	A	G	0.970	0.213	0.020	1.5E-25	trans
754	ENG	P17813	Endoglin	rs6437412	194468413	3	C	T	0.286	0.065	0.008	1.0E-16	trans
754	ENG	P17813	Endoglin	rs9987289	9183358	8	A	G	0.092	0.082	0.012	1.1E-11	trans
754	ENG	P17813	Endoglin	rs11794565	130601277	9	T	C	0.463	0.259	0.008	5.3E-250	cis
754	ENG	P17813	Endoglin	rs2519093	136141870	9	C	T	0.816	0.504	0.010	0.0E+00	trans
754	ENG	P17813	Endoglin	rs35166255	126301756	11	A	G	0.034	0.293	0.019	8.3E-52	trans
754	ENG	P17813	Endoglin	rs186021206	7069412	17	A	G	0.006	0.732	0.048	2.7E-52	trans
754	ENG	P17813	Endoglin	rs4760	44153100	19	G	A	0.156	0.235	0.010	3.1E-131	trans
755	ENO1	P06733	Alpha-enolase	rs11544513	8926474	1	G	T	0.997	1.322	0.066	4.3E-90	cis
755	ENO1	P06733	Alpha-enolase	rs1354034	56849749	3	C	T	0.604	0.064	0.008	5.8E-17	trans
756	ENO2	P09104	Gamma-enolase	rs1354034	56849749	3	C	T	0.604	0.057	0.008	4.0E-14	trans
756	ENO2	P09104	Gamma-enolase	rs6993770	106581528	8	A	T	0.714	0.147	0.008	2.8E-71	trans
756	ENO2	P09104	Gamma-enolase	rs60757417	135864436	9	C	G	0.939	0.107	0.016	8.1E-12	trans
756	ENO2	P09104	Gamma-enolase	rs7896518	65104500	10	G	A	0.424	0.106	0.008	6.6E-44	trans
756	ENO2	P09104	Gamma-enolase	rs11502185	180258	11	C	T	0.260	0.065	0.009	7.1E-12	trans
757	ENO3	P13929	Beta-enolase	rs74434374	31850308	6	A	C	0.044	0.120	0.018	1.1E-11	trans
757	ENO3	P13929	Beta-enolase	rs2243090	4835217	17	T	C	0.330	0.090	0.008	1.4E-32	cis
757	ENO3	P13929	Beta-enolase	rs12975366	54759361	19	T	C	0.605	0.094	0.007	1.5E-37	trans
758	ENOX2	Q16206	Ecto-NOX disulfide-thiol exchanger 2	rs1354034	56849749	3	C	T	0.604	0.070	0.008	2.4E-19	trans
759	ENPEP	Q07075	Glutamyl aminopeptidase	rs33966350	111431444	4	G	A	0.987	0.323	0.034	4.4E-21	cis
760	ENPP2	Q13822	Ectonucleotide pyrophosphatase/phosphodiesterase family member 2	rs34707604	69491456	4	C	T	0.258	0.057	0.007	2.0E-16	trans
760	ENPP2	Q13822	Ectonucleotide pyrophosphatase/phosphodiesterase family member 2	rs35570672	18272635	8	C	T	0.781	0.068	0.007	2.0E-20	trans
760	ENPP2	Q13822	Ectonucleotide pyrophosphatase/phosphodiesterase family member 2	rs13267597	120654225	8	T	G	0.673	0.262	0.007	0.0E+00	cis
760	ENPP2	Q13822	Ectonucleotide pyrophosphatase/phosphodiesterase family member 2	rs10901252	136128000	9	C	G	0.061	0.101	0.012	9.0E-17	trans
760	ENPP2	Q13822	Ectonucleotide pyrophosphatase/phosphodiesterase family member 2	rs186021206	7069412	17	A	G	0.006	0.338	0.040	4.5E-17	trans
760	ENPP2	Q13822	Ectonucleotide pyrophosphatase/phosphodiesterase family member 2	rs77542162	67081278	17	G	A	0.023	0.149	0.020	2.3E-14	trans
761	ENPP5	Q9UJA9	Ectonucleotide pyrophosphatase/phosphodiesterase family member 5	rs4665972	27598097	2	C	T	0.609	0.038	0.005	4.4E-14	trans
761	ENPP5	Q9UJA9	Ectonucleotide pyrophosphatase/phosphodiesterase family member 5	rs9472701	46116449	6	G	A	0.357	0.778	0.007	0.0E+00	cis
761	ENPP5	Q9UJA9	Ectonucleotide pyrophosphatase/phosphodiesterase family member 5	rs1299525	11722125	8	T	C	0.431	0.039	0.005	1.9E-15	trans
761	ENPP5	Q9UJA9	Ectonucleotide pyrophosphatase/phosphodiesterase family member 5	rs75491173	16368494	11	A	G	0.056	0.074	0.011	4.1E-12	trans
761	ENPP5	Q9UJA9	Ectonucleotide pyrophosphatase/phosphodiesterase family member 5	rs11072817	79236815	15	A	G	0.701	0.051	0.005	8.5E-22	trans
761	ENPP5	Q9UJA9	Ectonucleotide pyrophosphatase/phosphodiesterase family member 5	rs12903256	85308141	15	G	T	0.657	0.042	0.005	1.8E-16	trans
761	ENPP5	Q9UJA9	Ectonucleotide pyrophosphatase/phosphodiesterase family member 5	rs200489612	7106378	17	A	G	0.005	0.349	0.037	1.5E-21	trans
762	ENPP6	Q6UWR7	Glycerophosphocholine cholinephosphodiesterase ENPP6	rs317900	16135475	4	T	C	0.331	0.137	0.008	5.9E-70	trans
762	ENPP6	Q6UWR7	Glycerophosphocholine cholinephosphodiesterase ENPP6	rs78293932	185139101	4	T	C	0.024	0.953	0.024	0.0E+00	cis
762	ENPP6	Q6UWR7	Glycerophosphocholine cholinephosphodiesterase ENPP6	rs9266366	31333032	6	C	T	0.851	0.330	0.010	2.0E-240	trans
762	ENPP6	Q6UWR7	Glycerophosphocholine cholinephosphodiesterase ENPP6	rs17256042	150490238	7	G	A	0.948	0.114	0.016	3.5E-13	trans
762	ENPP6	Q6UWR7	Glycerophosphocholine cholinephosphodiesterase ENPP6	rs186021206	7069412	17	A	G	0.006	0.564	0.047	1.4E-32	trans
763	ENPP7	Q6UWV6	Ectonucleotide pyrophosphatase/phosphodiesterase family member 7	rs114165349	27021913	1	C	G	0.023	0.151	0.019	3.4E-15	trans
763	ENPP7	Q6UWV6	Ectonucleotide pyrophosphatase/phosphodiesterase family member 7	rs28601761	126500031	8	C	G	0.580	0.042	0.006	6.4E-13	trans
763	ENPP7	Q6UWV6	Ectonucleotide pyrophosphatase/phosphodiesterase family member 7	rs28929474	94844947	14	T	C	0.021	0.141	0.020	2.5E-12	trans
763	ENPP7	Q6UWV6	Ectonucleotide pyrophosphatase/phosphodiesterase family member 7	rs147233090	44028047	15	T	C	0.024	0.143	0.019	7.2E-14	trans
763	ENPP7	Q6UWV6	Ectonucleotide pyrophosphatase/phosphodiesterase family member 7	rs8076533	77704314	17	T	C	0.367	0.950	0.008	0.0E+00	cis
763	ENPP7	Q6UWV6	Ectonucleotide pyrophosphatase/phosphodiesterase family member 7	rs6050555	25328543	20	G	A	0.540	0.040	0.006	9.1E-12	trans
764	ENSA	O43768	Alpha-endosulfine	rs190018150	150607787	1	A	G	0.041	0.131	0.019	1.3E-11	cis
764	ENSA	O43768	Alpha-endosulfine	rs1354034	56849749	3	C	T	0.604	0.061	0.008	3.8E-15	trans
765	ENTPD2	Q9Y5L3	Ectonucleoside triphosphate diphosphohydrolase 2	rs56278466	17875857	10	G	T	0.661	0.050	0.007	4.3E-12	trans
765	ENTPD2	Q9Y5L3	Ectonucleoside triphosphate diphosphohydrolase 2	rs5848	42430244	17	T	C	0.274	0.062	0.008	2.6E-15	trans
766	ENTPD5	O75356	Ectonucleoside triphosphate diphosphohydrolase 5	rs35457250	186338564	3	T	C	0.009	0.759	0.038	3.1E-88	trans
766	ENTPD5	O75356	Ectonucleoside triphosphate diphosphohydrolase 5	rs6054	155489608	4	T	C	0.006	0.344	0.048	9.9E-13	trans
766	ENTPD5	O75356	Ectonucleoside triphosphate diphosphohydrolase 5	rs174574	61600342	11	A	C	0.353	0.066	0.007	2.0E-18	trans
766	ENTPD5	O75356	Ectonucleoside triphosphate diphosphohydrolase 5	rs146086623	74467222	14	G	A	0.947	0.973	0.017	0.0E+00	cis
766	ENTPD5	O75356	Ectonucleoside triphosphate diphosphohydrolase 5	rs28929474	94844947	14	T	C	0.021	0.552	0.027	1.2E-95	trans
766	ENTPD5	O75356	Ectonucleoside triphosphate diphosphohydrolase 5	rs763665	72078043	16	T	C	0.160	0.080	0.010	4.3E-16	trans
766	ENTPD5	O75356	Ectonucleoside triphosphate diphosphohydrolase 5	rs149394327	64228995	17	C	G	0.030	0.412	0.021	4.5E-85	trans
766	ENTPD5	O75356	Ectonucleoside triphosphate diphosphohydrolase 5	rs7236989	57010453	18	T	C	0.372	0.052	0.007	2.1E-12	trans
767	ENTPD6	O75354	Ectonucleoside triphosphate diphosphohydrolase 6	rs61804164	161623025	1	C	G	0.124	0.069	0.010	8.2E-12	trans
767	ENTPD6	O75354	Ectonucleoside triphosphate diphosphohydrolase 6	rs66824127	52551265	3	C	T	0.557	0.045	0.007	1.3E-11	trans

767	ENTPD6	O75354	Ectonucleoside triphosphate diphosphohydrolase 6	rs28929474	94844947	14	T	C	0.021	0.187	0.023	2.5E-16	trans
767	ENTPD6	O75354	Ectonucleoside triphosphate diphosphohydrolase 6	rs704	26694861	17	G	A	0.526	0.051	0.007	5.5E-15	trans
767	ENTPD6	O75354	Ectonucleoside triphosphate diphosphohydrolase 6	rs17695224	52324216	19	G	A	0.734	0.096	0.007	3.3E-38	trans
767	ENTPD6	O75354	Ectonucleoside triphosphate diphosphohydrolase 6	rs6050446	25195509	20	G	A	0.967	1.864	0.021	0.0E+00	cis
767	ENTPD6	O75354	Ectonucleoside triphosphate diphosphohydrolase 6	rs6061054	29811317	20	G	T	0.965	1.572	0.021	0.0E+00	trans
768	ENTR1	Q96C92	Endosome-associated-trafficking regulator 1	rs11145904	139297946	9	A	C	0.827	0.297	0.010	5.0E-195	cis
768	ENTR1	Q96C92	Endosome-associated-trafficking regulator 1	rs3747207	44324855	22	A	G	0.216	0.071	0.009	2.7E-15	trans
769	EPCAM	P16422	Epithelial cell adhesion molecule	rs2241764	133174764	2	C	T	0.662	0.084	0.008	6.9E-28	trans
769	EPCAM	P16422	Epithelial cell adhesion molecule	rs35853577	233558879	2	T	C	0.575	0.067	0.007	1.5E-19	trans
769	EPCAM	P16422	Epithelial cell adhesion molecule	rs1713810	153876826	3	A	G	0.148	0.084	0.010	2.8E-16	trans
769	EPCAM	P16422	Epithelial cell adhesion molecule	rs939885	195955762	3	A	G	0.486	0.060	0.007	1.3E-16	trans
769	EPCAM	P16422	Epithelial cell adhesion molecule	rs10066308	131790616	5	G	A	0.691	0.053	0.008	1.6E-11	trans
769	EPCAM	P16422	Epithelial cell adhesion molecule	rs6911827	22130601	6	T	C	0.452	0.049	0.007	9.8E-12	trans
769	EPCAM	P16422	Epithelial cell adhesion molecule	rs9478784	151020330	6	T	C	0.095	0.120	0.012	1.9E-22	trans
769	EPCAM	P16422	Epithelial cell adhesion molecule	rs931493	71145692	9	G	A	0.968	0.185	0.021	1.9E-18	trans
769	EPCAM	P16422	Epithelial cell adhesion molecule	rs2291428	45958856	10	G	C	0.756	0.096	0.008	5.9E-30	trans
769	EPCAM	P16422	Epithelial cell adhesion molecule	rs56398830	103701690	13	G	A	0.989	0.845	0.035	8.3E-132	trans
769	EPCAM	P16422	Epithelial cell adhesion molecule	rs35193388	4420817	16	G	GC	0.265	0.072	0.008	5.8E-18	trans
769	EPCAM	P16422	Epithelial cell adhesion molecule	rs12938714	28645110	17	A	G	0.330	0.164	0.008	2.9E-100	trans
769	EPCAM	P16422	Epithelial cell adhesion molecule	rs12453447	37648267	17	A	T	0.406	0.070	0.008	9.7E-17	trans
769	EPCAM	P16422	Epithelial cell adhesion molecule	rs2318997	55529230	18	T	G	0.567	0.054	0.007	1.5E-13	trans
769	EPCAM	P16422	Epithelial cell adhesion molecule	rs681343	49206462	19	C	T	0.490	0.234	0.007	4.0E-222	trans
769	EPCAM	P16422	Epithelial cell adhesion molecule	rs3746778	61341472	20	A	G	0.421	0.060	0.008	1.7E-15	trans
770	EPHA1	P21709	Ephrin type-A receptor 1	rs1497406	16505320	1	G	A	0.579	0.061	0.007	9.0E-19	trans
770	EPHA1	P21709	Ephrin type-A receptor 1	rs61747728	179526214	1	T	C	0.038	0.133	0.018	1.0E-13	trans
770	EPHA1	P21709	Ephrin type-A receptor 1	rs75045569	143109208	7	G	T	0.183	0.254	0.009	1.9E-172	cis
770	EPHA1	P21709	Ephrin type-A receptor 1	rs10740131	65271488	10	T	A	0.471	0.096	0.007	2.6E-44	trans
770	EPHA1	P21709	Ephrin type-A receptor 1	rs28929474	94844947	14	T	C	0.021	0.290	0.024	2.0E-33	trans
770	EPHA1	P21709	Ephrin type-A receptor 1	rs3814995	36342212	19	T	C	0.315	0.057	0.007	7.4E-15	trans
771	EPHA2	P29317	Ephrin type-A receptor 2	rs924204	16513926	1	A	G	0.410	0.133	0.007	3.1E-72	cis
771	EPHA2	P29317	Ephrin type-A receptor 2	rs61747728	179526214	1	T	C	0.038	0.151	0.019	1.3E-15	trans
771	EPHA2	P29317	Ephrin type-A receptor 2	rs11689257	162958353	2	G	A	0.931	0.111	0.015	3.8E-14	trans
771	EPHA2	P29317	Ephrin type-A receptor 2	rs8176746	136131322	9	T	G	0.062	0.141	0.015	6.2E-21	trans
771	EPHA2	P29317	Ephrin type-A receptor 2	rs12934455	20357281	16	C	T	0.834	0.066	0.010	1.2E-11	trans
771	EPHA2	P29317	Ephrin type-A receptor 2	rs679574	49206108	19	C	G	0.491	0.069	0.007	2.2E-21	trans
772	EPHA4	P54764	Ephrin type-A receptor 4	rs1260326	27730940	2	C	T	0.608	0.063	0.007	1.4E-17	trans
772	EPHA4	P54764	Ephrin type-A receptor 4	rs10932916	222389971	2	A	G	0.514	0.139	0.007	1.4E-84	cis
772	EPHA4	P54764	Ephrin type-A receptor 4	rs2519093	136141870	9	C	T	0.815	0.612	0.010	0.0E+00	trans
772	EPHA4	P54764	Ephrin type-A receptor 4	rs186021206	7069412	17	A	G	0.006	0.655	0.049	3.5E-41	trans
772	EPHA4	P54764	Ephrin type-A receptor 4	rs284662	41932275	19	C	T	0.622	0.054	0.007	1.1E-13	trans
773	EPHB4	P54760	Ephrin type-B receptor 4	rs61747728	179526214	1	T	C	0.038	0.154	0.019	2.3E-16	trans
773	EPHB4	P54760	Ephrin type-B receptor 4	rs314361	100413005	7	G	A	0.520	0.154	0.007	5.1E-100	cis
773	EPHB4	P54760	Ephrin type-B receptor 4	rs11782073	146300287	8	G	A	0.742	0.056	0.008	1.6E-11	trans
773	EPHB4	P54760	Ephrin type-B receptor 4	rs507666	136149399	9	G	A	0.815	0.368	0.010	0.0E+00	trans
773	EPHB4	P54760	Ephrin type-B receptor 4	rs7099071	28993896	10	C	T	0.169	0.067	0.010	4.7E-12	trans
773	EPHB4	P54760	Ephrin type-B receptor 4	rs7909516	77885337	10	C	T	0.776	0.093	0.009	5.4E-27	trans
773	EPHB4	P54760	Ephrin type-B receptor 4	rs9558661	106650093	13	C	T	0.799	0.133	0.009	2.1E-48	trans
773	EPHB4	P54760	Ephrin type-B receptor 4	rs28640218	20359267	16	G	T	0.808	0.079	0.009	1.3E-17	trans
773	EPHB4	P54760	Ephrin type-B receptor 4	rs186021206	7069412	17	A	G	0.006	0.395	0.050	2.0E-15	trans
773	EPHB4	P54760	Ephrin type-B receptor 4	rs1137844	36343079	19	G	C	0.315	0.064	0.008	2.7E-16	trans
774	EPHB6	O15197	Ephrin type-B receptor 6	rs61747728	179526214	1	T	C	0.038	0.199	0.019	5.1E-25	trans
774	EPHB6	O15197	Ephrin type-B receptor 6	rs7789303	142552547	7	A	G	0.734	0.318	0.009	1.1E-299	cis
774	EPHB6	O15197	Ephrin type-B receptor 6	rs13329952	20366507	16	T	C	0.808	0.081	0.009	6.6E-18	trans
775	EPO	P01588	Erythropoietin	rs218264	55408875	4	T	A	0.248	0.078	0.009	6.2E-19	trans
775	EPO	P01588	Erythropoietin	rs2032444	26046744	6	T	G	0.648	0.074	0.008	1.3E-20	trans
775	EPO	P01588	Erythropoietin	rs1546723	109625879	6	A	G	0.450	0.058	0.008	2.9E-14	trans
775	EPO	P01588	Erythropoietin	rs34164109	135421176	6	T	C	0.260	0.208	0.009	4.1E-126	trans
775	EPO	P01588	Erythropoietin	rs78744187	33754548	19	C	T	0.918	0.114	0.014	1.3E-16	trans
775	EPO	P01588	Erythropoietin	rs4811073	49099809	20	C	T	0.457	0.053	0.008	2.1E-12	trans
775	EPO	P01588	Erythropoietin	rs855791	37462936	22	A	G	0.439	0.078	0.008	1.0E-24	trans
776	EPPK1	P58107	Epiplakin	rs1434282	199010721	1	C	T	0.274	0.100	0.008	2.3E-37	trans
776	EPPK1	P58107	Epiplakin	rs342293	106372219	7	C	G	0.540	0.052	0.007	5.9E-14	trans
776	EPPK1	P58107	Epiplakin	rs73375129	144950276	8	A	G	0.863	0.544	0.011	0.0E+00	cis
776	EPPK1	P58107	Epiplakin	rs60757417	135864436	9	C	G	0.939	0.110	0.015	6.0E-14	trans
776	EPPK1	P58107	Epiplakin	rs695113	128562098	11	T	C	0.695	0.057	0.008	2.9E-14	trans
777	EPS8L2	Q9H6S3	Epidermal growth factor receptor kinase substrate 8-like protein 2	rs9271657	32592582	6	C	T	0.214	0.065	0.009	1.4E-12	trans
777	EPS8L2	Q9H6S3	Epidermal growth factor receptor kinase substrate 8-like protein 2	rs1106192	95896586	9	C	G	0.954	0.118	0.017	9.5E-12	trans
777	EPS8L2	Q9H6S3	Epidermal growth factor receptor kinase substrate 8-like protein 2	rs11246276	706284	11	G	T	0.841	0.394	0.010	0.0E+00	cis
777	EPS8L2	Q9H6S3	Epidermal growth factor receptor kinase substrate 8-like protein 2	rs3747207	44324855	22	A	G	0.215	0.062	0.009	1.4E-12	trans
778	ERBB2	P04626	Receptor tyrosine-protein kinase erbB-2	rs9270464	32558914	6	A	G	0.661	0.068	0.008	4.2E-18	trans
778	ERBB2	P04626	Receptor tyrosine-protein kinase erbB-2	rs1461729	9187242	8	A	G	0.103	0.090	0.012	7.8E-15	trans
778	ERBB2	P04626	Receptor tyrosine-protein kinase erbB-2	rs8176749	136131188	9	T	C	0.061	0.110	0.015	4.4E-14	trans
778	ERBB2	P04626	Receptor tyrosine-protein kinase erbB-2	rs28929474	94844947	14	T	C	0.021	0.244	0.025	3.8E-23	trans
778	ERBB2	P04626	Receptor tyrosine-protein kinase erbB-2	rs4788460	72154509	16	T	C	0.294	0.052	0.008	1.4E-11	trans
778	ERBB2	P04626	Receptor tyrosine-protein kinase erbB-2	rs3764354	37916823	17	T	C	0.146	0.200	0.010	1.4E-88	cis
778	ERBB2	P04626	Receptor tyrosine-protein kinase erbB-2	rs1801689	64210580	17	C	A	0.030	0.309	0.021	2.9E-50	trans
779	ERBB3	P21860	Receptor tyrosine-protein kinase erbB-3	rs1260326	27730940	2	T	C	0.391	0.131	0.007	1.2E-74	trans
779	ERBB3	P21860	Receptor tyrosine-protein kinase erbB-3	rs13234131	73025975	7	A	G	0.873	0.085	0.010	3.6E-16	trans
779	ERBB3	P21860	Receptor tyrosine-protein kinase erbB-3	rs4731549	128741913	7	C	T	0.450	0.102	0.007	1.6E-47	trans
779	ERBB3	P21860	Receptor tyrosine-protein kinase erbB-3	rs28601761	126500031	8	C	G	0.581	0.056	0.007	3.2E-15	trans
779	ERBB3	P21860	Receptor tyrosine-protein kinase erbB-3	rs10761737	65052205	10	T	C	0.587	0.049	0.007	8.6E-12	trans
779	ERBB3	P21860	Receptor tyrosine-protein kinase erbB-3	rs150844304	43726625	15	C	A	0.024	0.205	0.023	1.9E-19	trans
779	ERBB3	P21860	Receptor tyrosine-protein kinase erbB-3	rs149394327	64228995	17	C	G	0.030	0.190	0.020	1.5E-20	trans
780	ERBB4	Q15303	Receptor tyrosine-protein kinase erbB-4	rs9787076	44141149	1	C	A	0.329	0.064	0.008	5.9E-17	trans
780	ERBB4	Q15303	Receptor tyrosine-protein kinase erbB-4	rs35383942	201437832	1	T	C	0.062	0.127	0.015	1.1E-17	trans
780	ERBB4	Q15303	Receptor tyrosine-protein kinase erbB-4	rs12692385	9694620	2	T	C	0.671	0.082	0.008	2.7E-26	trans
780	ERBB4	Q15303	Receptor tyrosine-protein kinase erbB-4	rs4665972	27598097	2	C	T	0.609	0.064	0.008	1.1E-17	trans
780	ERBB4	Q15303	Receptor tyrosine-protein kinase erbB-4	rs6735267	213403863	2	T	C	0.269	0.219	0.008	2.7E-153	cis
780	ERBB4	Q15303	Receptor tyrosine-protein kinase erbB-4	rs34673027	128739025	7	C	A	0.553	0.076	0.007	4.9E-26	trans
780	ERBB4	Q15303	Receptor tyrosine-protein kinase erbB-4	rs2519093	136141870	9	C	T	0.816	0.136	0.009	7.1E-49	trans
780	ERBB4	Q15303	Receptor tyrosine-protein kinase erbB-4	rs56278466	17875857	10	G	T	0.661	0.110	0.008	2.3E-48	trans
780	ERBB4	Q15303	Receptor tyrosine-protein kinase erbB-4	rs11227279	65495211	11	G	A	0.656	0.051	0.008	9.0E-12	trans
780	ERBB4	Q15303	Receptor tyrosine-protein kinase erbB-4	rs9567991	48654526	13	T	C	0.656	0.052	0.007	3.8E-12	trans
780	ERBB4	Q15303	Receptor tyrosine-protein kinase erbB-4	rs2541593	103423	16	A	C	0.810	0.068	0.009	1.1E-13	trans
780	ERBB4	Q15303	Receptor tyrosine-protein kinase erbB-4	rs186021206	7069412	17	A	G	0.006	0.975	0.049	1.5E-87	trans
780	ERBB4	Q15303	Receptor tyrosine-protein kinase erbB-4	rs72835417	47241642	17	A	G	0.110	0.152	0.011	1.9E-40	trans
780	ERBB4	Q15303	Receptor tyrosine-protein kinase erbB-4	rs485186	49207206	19	G	A	0.538	0.097	0.007	9.4E-42	trans
781	ERBIN	Q96RT1	Erbin	rs342296	106372903	7	G	A	0.544	0.052	0.008	5.3E-12	trans
781	ERBIN	Q96RT1	Erbin	rs10822145	64934548	10	T	C	0.472	0.055	0.008	2.0E-13	trans
782	ERC2	O15083	ERC protein 2	rs79784038	56133667	3	G	C	0.974	0.170	0.025	5.1E-12	cis
783	EREG	O14944	Proepiregulin	rs12655145	150835349	5	G	A	0.616	0.075	0.008	3.8E-22	trans
783	EREG	O14944	Proepiregulin	rs2516471	31400292	6	G	A	0.936	0.110	0.016	2.3E-12	trans
783	EREG	O14944	Proepiregulin	rs6993770	106581528	8	A	T	0.714	0.093	0.008	2.1E-28	trans
783	EREG	O14944	Proepiregulin	rs10820606	99192919	9	A	C	0.770	0.110	0.009	2.2E-33	trans
783	EREG	O14944	Proepiregulin	rs7080386	65048306	10	A	C	0.412	0.070	0.008	1.4E-19	trans
783	EREG	O14944	Proepiregulin	rs892090	55539072	19	G	T	0.834	0.119	0.010	3.2E-31	trans
783	EREG	O14944	Proepiregulin	rs6081569	19303786	20	C	G	0.369	0.055	0.008	2.1E-12	trans
784	ERI1	Q8IV48	3'-5' exoribonuclease 1	rs1354034	56849749	3	C	T	0.604	0.052	0.008	1.5E-11	trans
784	ERI1	Q8IV48	3'-5' exoribonuclease 1	rs5985	6318795	6	C	A	0.742	0.084	0.009	4.5E-22	trans
784	ERI1	Q8IV48	3'-5' exoribonuclease 1	rs7814328	8876229	8	T	C	0.539	0.071	0.008	9.8E-21	cis
785	ERMAP	Q96PL5	Erythroid membrane-associated protein	rs28594470	25736299	1	A	T	0.581	0.087	0.007	4.2E-34	trans
785	ERMAP	Q96PL5	Erythroid membrane-associated protein	rs11210710	43209941	1	G	T	0.524	0.091	0.007	1.7E-38	cis
785	ERMAP	Q96PL5	Erythroid membrane-associated protein	rs4672497	62523565	2	C	G	0.779	0.070	0.008	4.6E-17	trans
785	ERMAP	Q96PL5	Erythroid membrane-associated protein	rs11736498	145058284	4	G	A	0.448	0.270	0.007	0.0E+00	trans
785	ERMAP	Q96PL5	Erythroid membrane-associated protein	rs28749166	29920951	6	C	T	0.633	0.058	0.007	2.2E-15	trans
785	ERMAP	Q96PL5	Erythroid membrane-associated protein	rs6592965	50427982	7	G	A	0.546	0.099	0.007	2.5E-46	trans
785	ERMAP	Q96PL5	Erythroid membrane-associated protein	rs6993770	106581528	8	A	T	0.713	0.061	0.008	1.0E-15	trans
785	ERMAP	Q96PL5	Erythroid membrane-associated protein	rs746064	42581448	21	A	G	0.380	0.449	0.008	0.0E+00	trans
786	ERN1	O75460	Serine/threonine-protein kinase/endoribonuclease IRE1	rs1260326	27730940	2	T	C	0.392	0.059	0.008	1.3E-14	trans
786	ERN1	O75460	Serine/threonine-protein kinase/endoribonuclease IRE1	rs6054	155489608	4	T	C	0.006	0.382	0.050	1.5E-14	trans
786	ERN1	O75460	Serine/threonine-protein kinase/endoribonuclease IRE1	rs8176746	136131322	9	T	G	0.061	0.204	0.015	5.2E-40	trans
786	ERN1	O75460	Serine/threonine-protein kinase/endoribonuclease IRE1	rs174566	61592362	11	G	A	0.350	0.104	0.008	4.2E-41	trans
786	ERN1	O75460	Serine/threonine-protein kinase/endoribonuclease IRE1	rs28929474	94844947	14	T	C	0.021	0.222	0.026	1.3E-17	trans
786	ERN1	O75460	Serine/threonine-protein kinase/endoribonuclease IRE1	rs217184	72105965	16	T	C	0.804	0.085	0.009	1.8E-19	trans
786	ERN1	O75460	Serine/threonine-protein kinase/endoribonuclease IRE1	rs1801689	64210580	17	C	A	0.030	0.526	0.022	1.9E-128	cis
786	ERN1	O75460	Serine/threonine-protein kinase/endoribonuclease IRE1	rs429358	45411941	19	T	C	0.845	0.147	0.010	5.7E-46	trans
786	ERN1	O75460	Serine/threonine-protein kinase/endoribonuclease IRE1	rs681343	49206462	19	C	T	0.491	0.124	0.007	1.6E-61	trans
787	ERP29	P30040	Endoplasmic reticulum resident protein 29	rs1354034	56849749	3	C	T	0.604	0.068	0.008	8.7E-19	trans
787	ERP29	P30040	Endoplasmic reticulum resident protein 29	rs342298	106373646	7	C	T	0.545	0.058	0.008	1.7E-14	trans
787	ERP29	P30040	Endoplasmic reticulum resident protein 29	rs6993770	106581528	8	A	T	0.713	0.065	0.008	5.6E-15	trans
788	ERP44	Q9BS26	Endoplasmic reticulum resident protein 44	rs4846923	230307222	1	T	G	0.296	0.059	0.008	2.2E-13	trans
788	ERP44	Q9BS26	Endoplasmic reticulum resident protein 44	rs1260326	27730940	2	T	C	0.392	0.055	0.007	1.9E-13	trans
788	ERP44	Q9BS26	Endoplasmic reticulum resident protein 44	rs7614709	186393786	3	G	A	0.778	0.250	0.009	2.7E-176	trans
788	ERP44	Q9BS26	Endoplasmic reticulum resident protein 44	rs6993770	106581528	8	A	T	0.714	0.061	0.008	2.2E-14	trans
788	ERP44	Q9BS26	Endoplasmic reticulum resident protein 44	rs36115375	7777799	10	A	C	0.063	0.723	0.015	0.0E+00	trans
788	ERP44	Q9BS26	Endoplasmic reticulum resident protein 44	rs17580	94847262	14	T	A	0.951	0.132	0.017	3.3E-15	trans
789	ESAM	Q96AP7	Endothelial cell-selective adhesion molecule	rs6961069	80218961	7	T	C	0.403	0.060	0.007	2.1E-15	trans
789	ESAM	Q96AP7	Endothelial cell-selective adhesion molecule	rs6993770	106581528	8	A	T	0.713	0.105	0.008	4.4E-38	trans
789	ESAM	Q96AP7	Endothelial cell-selective adhesion molecule	rs8176672	136142185	9	T	C	0.061	0.205	0.015	4.4E-41	trans
789	ESAM	Q96AP7	Endothelial cell-selective adhesion molecule	rs7080386	65048306	10	A	C	0.412	0.086	0.007	1.4E-30	trans
789	ESAM	Q96AP7	Endothelial cell-selective adhesion molecule	rs61753651	124626569	11	G	A	0.996	1.890	0.059	1.7E-226	cis
789	ESAM	Q96AP7	Endothelial cell-selective adhesion molecule	rs2127870	65796846	14	C	G	0.795	0.087	0.009	2.6E-21	trans
789	ESAM	Q96AP7	Endothelial cell-selective adhesion molecule	rs188942006	65003826	15	C	G	0.065	0.109	0.015	7.1E-13	trans

789	ESAM	Q96AP7	Endothelial cell-selective adhesion molecule	rs1654425	55538980	19	C	T	0.834	0.133	0.010	1.0E-40	trans
790	ESM1	Q9NQ30	Endothelial cell-specific molecule 1	rs4242051	54198775	5	C	T	0.746	0.192	0.009	5.3E-111	cis
790	ESM1	Q9NQ30	Endothelial cell-specific molecule 1	rs224916	81722747	5	A	G	0.274	0.077	0.008	3.3E-20	trans
790	ESM1	Q9NQ30	Endothelial cell-specific molecule 1	rs113760175	22343592	6	A	G	0.071	0.136	0.014	2.4E-21	trans
790	ESM1	Q9NQ30	Endothelial cell-specific molecule 1	rs9269891	32551017	6	C	T	0.539	0.068	0.008	1.3E-16	trans
790	ESM1	Q9NQ30	Endothelial cell-specific molecule 1	rs1475718	137118170	9	G	A	0.563	0.081	0.007	9.2E-28	trans
790	ESM1	Q9NQ30	Endothelial cell-specific molecule 1	rs10761782	65287064	10	G	A	0.475	0.060	0.007	1.8E-16	trans
791	ESR1	P03372	Estrogen receptor	rs373801706	32606560	6	G	A	0.053	0.149	0.017	1.3E-17	trans
792	ESYT2	A0FGR8	Extended synaptotagmin-2	rs1354034	56849749	3	C	T	0.604	0.078	0.008	1.1E-24	trans
792	ESYT2	A0FGR8	Extended synaptotagmin-2	rs2709865	158539572	7	T	C	0.058	0.291	0.016	2.0E-73	cis
793	EVI5	O60447	Ecotropic viral integration site 5 protein homolog	rs11808092	93073228	1	C	A	0.745	0.145	0.009	1.7E-63	cis
794	EXOSC10	Q01780	Exosome component 10	rs79874727	106370437	14	A	G	0.106	0.107	0.014	2.9E-14	trans
795	EXTL1	Q92935	Exostosin-like 1	rs2736831	26357656	1	C	A	0.307	0.412	0.008	0.0E+00	cis
796	EZR	P15311	Ezrin	rs3734470	159187347	6	T	A	0.697	0.097	0.008	3.2E-32	cis
796	EZR	P15311	Ezrin	rs7208422	76130575	17	A	T	0.523	0.056	0.007	8.0E-14	trans
796	EZR	P15311	Ezrin	rs10405357	54759666	19	T	C	0.564	0.062	0.008	2.5E-16	trans
797	F10	P00742	Coagulation factor X	rs35797675	72878044	7	T	G	0.785	0.075	0.009	1.3E-16	trans
797	F10	P00742	Coagulation factor X	rs28601761	126500031	8	C	G	0.580	0.053	0.008	1.4E-12	trans
797	F10	P00742	Coagulation factor X	rs10982156	117088064	9	T	A	0.931	0.454	0.015	1.5E-195	trans
797	F10	P00742	Coagulation factor X	rs6479877	64878118	10	C	G	0.560	0.080	0.007	3.6E-27	trans
797	F10	P00742	Coagulation factor X	rs547138	113792170	13	A	T	0.605	0.179	0.008	1.1E-118	cis
797	F10	P00742	Coagulation factor X	rs924135	16123459	16	T	A	0.611	0.057	0.008	6.0E-14	trans
797	F10	P00742	Coagulation factor X	rs2330795	24996582	22	G	A	0.662	0.073	0.008	5.0E-21	trans
798	F11	P03951	Coagulation factor XI	rs1260326	27730940	2	T	C	0.392	0.066	0.007	1.3E-22	trans
798	F11	P03951	Coagulation factor XI	rs710446	186459927	3	C	T	0.409	0.474	0.007	0.0E+00	trans
798	F11	P03951	Coagulation factor XI	rs2289252	187207381	4	T	C	0.399	0.385	0.007	0.0E+00	cis
798	F11	P03951	Coagulation factor XI	rs77542162	67081278	17	G	A	0.023	0.158	0.022	4.9E-13	trans
799	F11R	Q9Y624	Junctional adhesion molecule A	rs60315407	161032805	1	C	T	0.291	0.063	0.008	5.7E-14	cis
799	F11R	Q9Y624	Junctional adhesion molecule A	rs1354034	56849749	3	C	T	0.604	0.075	0.008	2.3E-22	trans
799	F11R	Q9Y624	Junctional adhesion molecule A	rs3804753	122851840	3	A	G	0.406	0.055	0.008	7.3E-13	trans
799	F11R	Q9Y624	Junctional adhesion molecule A	rs11242109	131677047	5	G	T	0.519	0.064	0.008	3.5E-17	trans
799	F11R	Q9Y624	Junctional adhesion molecule A	rs6993770	106581528	8	A	T	0.714	0.069	0.008	1.5E-16	trans
799	F11R	Q9Y624	Junctional adhesion molecule A	rs409801	4744743	9	C	T	0.507	0.053	0.008	1.9E-12	trans
799	F11R	Q9Y624	Junctional adhesion molecule A	rs10733789	64948684	10	C	T	0.313	0.059	0.008	5.0E-13	trans
799	F11R	Q9Y624	Junctional adhesion molecule A	rs1654425	55538980	19	C	T	0.834	0.088	0.010	6.7E-18	trans
800	F12	P00748	Coagulation factor XII	rs1260326	27730940	2	T	C	0.392	0.068	0.005	8.8E-37	trans
800	F12	P00748	Coagulation factor XII	rs1801020	176836532	5	G	A	0.745	1.182	0.009	0.0E+00	cis
800	F12	P00748	Coagulation factor XII	rs204886	32029415	6	C	T	0.506	0.037	0.005	5.1E-13	trans
800	F12	P00748	Coagulation factor XII	rs13234378	73026151	7	A	T	0.873	0.061	0.008	8.0E-15	trans
800	F12	P00748	Coagulation factor XII	rs7924036	65191645	10	G	T	0.499	0.047	0.005	1.6E-19	trans
800	F12	P00748	Coagulation factor XII	rs12975366	54759361	19	C	T	0.395	0.047	0.005	2.0E-18	trans
801	F13B	P05160	Coagulation factor XIII B chain	rs12134960	197009508	1	G	C	0.841	0.559	0.010	0.0E+00	cis
801	F13B	P05160	Coagulation factor XIII B chain	rs1047891	211540507	2	C	A	0.687	0.070	0.008	2.6E-20	trans
801	F13B	P05160	Coagulation factor XIII B chain	rs12204494	6333084	6	G	T	0.382	0.236	0.007	5.8E-228	trans
802	F2	P00734	Prothrombin	rs10982156	117088064	9	T	A	0.931	0.111	0.015	6.7E-13	trans
802	F2	P00734	Prothrombin	rs1799963	46761055	11	A	G	0.011	0.731	0.037	8.1E-86	cis
802	F2	P00734	Prothrombin	rs151130188	51486440	11	T	G	0.008	0.490	0.049	8.2E-24	trans
803	F2R	P25116	Proteinase-activated receptor 1	rs13088560	17151126	3	G	T	0.824	0.080	0.010	7.9E-15	trans
803	F2R	P25116	Proteinase-activated receptor 1	rs1354034	56849749	3	C	T	0.604	0.094	0.008	1.9E-34	trans
803	F2R	P25116	Proteinase-activated receptor 1	rs168753	76028124	5	A	T	0.827	0.152	0.010	6.4E-52	cis
803	F2R	P25116	Proteinase-activated receptor 1	rs11759553	135422296	6	T	A	0.272	0.061	0.008	5.5E-13	trans
803	F2R	P25116	Proteinase-activated receptor 1	rs6993770	106581528	8	A	T	0.713	0.082	0.008	3.4E-23	trans
803	F2R	P25116	Proteinase-activated receptor 1	rs579459	136154168	9	T	C	0.792	0.063	0.009	1.2E-11	trans
803	F2R	P25116	Proteinase-activated receptor 1	rs7080386	65048306	10	A	C	0.412	0.112	0.008	1.2E-48	trans
803	F2R	P25116	Proteinase-activated receptor 1	rs17655730	270715	11	C	T	0.245	0.060	0.009	9.0E-12	trans
803	F2R	P25116	Proteinase-activated receptor 1	rs892090	55539072	19	G	T	0.834	0.085	0.010	4.7E-17	trans
804	F3	P13726	Tissue factor	rs6666213	95274365	1	G	A	0.373	0.185	0.007	7.8E-145	cis
804	F3	P13726	Tissue factor	rs780094	27741237	2	C	T	0.620	0.051	0.007	5.3E-13	trans
804	F3	P13726	Tissue factor	rs2519093	136141870	9	C	T	0.816	0.276	0.009	1.3E-209	trans
804	F3	P13726	Tissue factor	rs488703	113770876	13	A	G	0.101	0.097	0.011	7.0E-18	trans
804	F3	P13726	Tissue factor	rs200489612	7106378	17	A	G	0.005	0.485	0.051	2.3E-21	trans
804	F3	P13726	Tissue factor	rs2659005	79218714	17	T	C	0.441	0.048	0.007	4.1E-12	trans
804	F3	P13726	Tissue factor	rs516316	49206145	19	G	C	0.491	0.283	0.007	0.0E+00	trans
805	F7	P08709	Coagulation factor VII	rs1260326	27730940	2	T	C	0.392	0.126	0.007	4.8E-73	trans
805	F7	P08709	Coagulation factor VII	rs35732917	73013269	7	T	C	0.715	0.066	0.008	1.2E-18	trans
805	F7	P08709	Coagulation factor VII	rs7075901	65280994	10	C	A	0.532	0.057	0.007	3.6E-17	trans
805	F7	P08709	Coagulation factor VII	rs7232	59940599	11	A	T	0.374	0.059	0.007	5.6E-17	trans
805	F7	P08709	Coagulation factor VII	rs510335	113759755	13	G	T	0.888	0.779	0.012	0.0E+00	cis
805	F7	P08709	Coagulation factor VII	rs28929474	94844947	14	T	C	0.021	0.162	0.024	8.4E-12	trans
806	F9	P00740	Coagulation factor IX	rs1260326	27730940	2	T	C	0.392	0.110	0.008	2.5E-47	trans
806	F9	P00740	Coagulation factor IX	rs422187	138632859	X	C	A	0.303	0.355	0.007	0.0E+00	cis
807	FABP1	P07148	Fatty acid-binding protein, liver	rs2241883	88424066	2	T	C	0.693	0.237	0.008	6.3E-195	cis
807	FABP1	P07148	Fatty acid-binding protein, liver	rs17655730	270715	11	C	T	0.245	0.060	0.009	2.6E-12	trans
808	FABP2	P12104	Fatty acid-binding protein, intestinal	rs1375131	135954797	2	C	T	0.266	0.058	0.009	1.5E-11	trans
808	FABP2	P12104	Fatty acid-binding protein, intestinal	rs17009129	120280617	4	C	T	0.675	0.213	0.008	3.1E-156	cis
808	FABP2	P12104	Fatty acid-binding protein, intestinal	rs6472539	71629810	8	G	C	0.484	0.071	0.007	1.4E-21	trans
808	FABP2	P12104	Fatty acid-binding protein, intestinal	rs17655730	270715	11	C	T	0.245	0.086	0.009	4.5E-23	trans
808	FABP2	P12104	Fatty acid-binding protein, intestinal	rs8111874	49168942	19	G	A	0.535	0.057	0.008	9.5E-14	trans
809	FABP3	P05413	Fatty acid-binding protein, heart	rs61780805	31877051	1	C	T	0.952	0.192	0.016	3.3E-32	cis
809	FABP3	P05413	Fatty acid-binding protein, heart	rs12463674	179432185	2	G	A	0.316	0.064	0.007	9.0E-18	trans
810	FABP4	P15090	Fatty acid-binding protein, adipocyte	rs7647481	12391813	3	A	G	0.121	0.104	0.011	7.2E-23	trans
810	FABP4	P15090	Fatty acid-binding protein, adipocyte	rs112313579	82391183	8	T	TG	0.841	0.118	0.009	1.7E-35	cis
811	FABP5	Q01469	Fatty acid-binding protein 5	rs118019635	82193282	8	C	A	0.926	0.200	0.014	7.4E-44	cis
811	FABP5	Q01469	Fatty acid-binding protein 5	rs6993770	106581528	8	A	T	0.714	0.058	0.008	2.8E-12	trans
812	FABP6	P51161	Gastrotropin	rs1130435	159659273	5	C	T	0.599	0.131	0.008	1.7E-62	cis
812	FABP6	P51161	Gastrotropin	rs6472539	71629810	8	G	C	0.485	0.209	0.008	1.1E-167	trans
812	FABP6	P51161	Gastrotropin	rs35866622	49218060	19	C	T	0.500	0.075	0.008	5.0E-23	trans
813	FABP9	Q0Z7S8	Fatty acid-binding protein 9	rs72646785	17603472	1	T	G	0.166	0.156	0.010	1.6E-58	trans
813	FABP9	Q0Z7S8	Fatty acid-binding protein 9	rs36010924	152088844	1	G	A	0.184	0.371	0.009	0.0E+00	trans
813	FABP9	Q0Z7S8	Fatty acid-binding protein 9	rs774004	138177132	3	A	G	0.740	0.076	0.008	1.3E-21	trans
813	FABP9	Q0Z7S8	Fatty acid-binding protein 9	rs1344815	149340813	3	A	G	0.346	0.050	0.007	8.2E-12	trans
813	FABP9	Q0Z7S8	Fatty acid-binding protein 9	rs1345417	181511951	3	G	C	0.603	0.072	0.007	1.2E-22	trans
813	FABP9	Q0Z7S8	Fatty acid-binding protein 9	rs57866025	189957369	3	T	C	0.266	0.055	0.008	6.0E-12	trans
813	FABP9	Q0Z7S8	Fatty acid-binding protein 9	rs2436396	139533563	5	A	G	0.299	0.064	0.008	7.1E-17	trans
813	FABP9	Q0Z7S8	Fatty acid-binding protein 9	rs28497692	82412691	8	G	T	0.962	0.224	0.019	1.5E-33	cis
813	FABP9	Q0Z7S8	Fatty acid-binding protein 9	rs7905367	54334653	10	C	G	0.798	0.081	0.009	8.3E-21	trans
813	FABP9	Q0Z7S8	Fatty acid-binding protein 9	rs12457893	60926161	18	A	C	0.542	0.068	0.007	1.3E-21	trans
813	FABP9	Q0Z7S8	Fatty acid-binding protein 9	rs867348	39687121	19	A	T	0.322	0.091	0.008	2.1E-32	trans
813	FABP9	Q0Z7S8	Fatty acid-binding protein 9	rs73207128	16785127	X	T	C	0.455	0.044	0.006	9.4E-14	trans
814	FADD	Q13158	FAS-associated death domain protein	rs342298	106373646	7	C	T	0.545	0.051	0.008	1.3E-11	trans
814	FADD	Q13158	FAS-associated death domain protein	rs3847326	65331859	10	A	G	0.518	0.051	0.007	6.3E-12	trans
814	FADD	Q13158	FAS-associated death domain protein	rs10898847	70045922	11	A	G	0.408	0.080	0.008	1.9E-25	cis
815	FAM13A	O94988	Protein FAM13A	rs1354034	56849749	3	C	T	0.605	0.072	0.008	7.4E-22	trans
815	FAM13A	O94988	Protein FAM13A	rs34262577	89932374	4	A	AAGGG	0.831	0.167	0.010	7.2E-63	cis
815	FAM13A	O94988	Protein FAM13A	rs342293	106372219	7	G	C	0.460	0.113	0.007	1.3E-52	trans
815	FAM13A	O94988	Protein FAM13A	rs60757417	135864436	9	C	G	0.940	0.132	0.016	3.1E-17	trans
816	FAM171A2	A8MVW0	Protein FAM171A2	rs139026664	89936251	2	A	C	0.084	0.129	0.014	4.4E-19	trans
817	FAM171B	Q6P995	Protein FAM171B	rs3886170	187557294	2	C	T	0.721	0.377	0.008	0.0E+00	cis
817	FAM171B	Q6P995	Protein FAM171B	rs7635884	186341191	3	T	C	0.640	0.056	0.008	2.7E-13	trans
817	FAM171B	Q6P995	Protein FAM171B	rs8176741	136131461	9	A	G	0.061	0.110	0.015	8.9E-13	trans
818	FAM172A	Q8WUF8	Cotranscriptional regulator FAM172A	rs1354034	56849749	3	C	T	0.605	0.053	0.008	4.5E-12	trans
818	FAM172A	Q8WUF8	Cotranscriptional regulator FAM172A	rs1470150	93138654	5	C	T	0.132	0.191	0.011	9.9E-66	cis
818	FAM172A	Q8WUF8	Cotranscriptional regulator FAM172A	rs113708600	31376217	6	C	A	0.067	0.119	0.015	4.8E-15	trans
819	FAM20A	Q96MK3	Pseudokinase FAM20A	rs1260326	27730940	2	T	C	0.391	0.096	0.007	1.3E-40	trans
819	FAM20A	Q96MK3	Pseudokinase FAM20A	rs249641	141525305	5	G	A	0.618	0.052	0.007	9.1E-13	trans
819	FAM20A	Q96MK3	Pseudokinase FAM20A	rs150231592	299843	7	G	A	0.956	0.180	0.019	3.7E-21	trans
819	FAM20A	Q96MK3	Pseudokinase FAM20A	rs35332062	73012042	7	G	A	0.873	0.099	0.011	1.3E-20	trans
819	FAM20A	Q96MK3	Pseudokinase FAM20A	rs2001846	126478450	8	T	C	0.480	0.055	0.007	9.9E-15	trans
819	FAM20A	Q96MK3	Pseudokinase FAM20A	rs2622935	117010758	11	A	G	0.193	0.138	0.009	1.2E-53	trans
819	FAM20A	Q96MK3	Pseudokinase FAM20A	rs28929474	94844947	14	T	C	0.021	0.167	0.025	1.1E-11	trans
819	FAM20A	Q96MK3	Pseudokinase FAM20A	rs704	26694861	17	G	A	0.527	0.084	0.007	2.8E-31	trans
819	FAM20A	Q96MK3	Pseudokinase FAM20A	rs2952271	66529779	17	G	A	0.347	0.239	0.008	2.2E-214	cis
819	FAM20A	Q96MK3	Pseudokinase FAM20A	rs7247412	52313868	19	T	C	0.733	0.068	0.008	1.3E-17	trans
820	FAM3B	P58499	Protein FAM3B	rs3129777	32646184	6	C	A	0.785	0.115	0.009	5.8E-37	trans
820	FAM3B	P58499	Protein FAM3B	rs2519093	136141870	9	C	T	0.816	0.108	0.009	5.5E-34	trans
820	FAM3B	P58499	Protein FAM3B	rs17743980	9793839	17	A	G	0.314	0.058	0.007	3.7E-15	trans
820	FAM3B	P58499	Protein FAM3B	rs708686	5840619	19	T	C	0.267	0.253	0.008	1.5E-221	trans
820	FAM3B	P58499	Protein FAM3B	rs602662	49206985	19	A	G	0.536	0.250	0.007	1.8E-271	trans
820	FAM3B	P58499	Protein FAM3B	rs57529409	42718262	21	C	T	0.953	1.153	0.017	0.0E+00	cis
821	FAM3C	Q92520	Protein FAM3C	rs36198735	121080192	7	A	AAAC	0.267	0.114	0.008	4.6E-43	cis
821	FAM3C	Q92520	Protein FAM3C	rs36060036	20361950	16	C	T	0.832	0.073	0.010	7.1E-14	trans
821	FAM3C	Q92520	Protein FAM3C	rs9889902	74648034	17	C	T	0.342	0.059	0.008	1.4E-14	trans
822	FAM3D	Q96BQ1	Protein FAM3D	rs12493131	58649978	3	G	T	0.904	0.492	0.011	0.0E+00	cis
822	FAM3D	Q96BQ1	Protein FAM3D	rs4556017	100632790	7	C	T	0.147	0.089	0.009	2.7E-23	trans
822	FAM3D	Q96BQ1	Protein FAM3D	rs708686	5840619	19	T	C	0.268	0.122	0.008	1.2E-57	trans
822	FAM3D	Q96BQ1	Protein FAM3D	rs681343	49206462	19	C	T	0.491	0.478	0.007	0.0E+00	trans
823	FAP	Q12884	Prolyl endopeptidase FAP	rs1751479	66016205	1	G	A	0.354	0.064	0.008	5.2E-17	trans
823	FAP	Q12884	Prolyl endopeptidase FAP	rs4664454	163071161	2	G	C	0.010	1.075	0.037	3.9E-187	cis
823	FAP	Q12884	Prolyl endopeptidase FAP	rs9266252	31325822	6	A	G	0.701	0.118	0.008	2.6E-49	trans
823	FAP	Q12884	Prolyl endopeptidase FAP	rs74044565	326384	11	C	G	0.139	0.087	0.011	2.7E-16	trans
823	FAP	Q12884	Prolyl endopeptidase FAP	rs34885055	51013520	15	G	C	0.119	0.160	0.011	1.4E-46	trans
823	FAP	Q12884	Prolyl endopeptidase FAP	rs740516	67082962	17	C	G	0.851	0.149	0.010	2.0E-47	trans
824	FAS	P25445	Tumor necrosis factor receptor superfamily member 6	rs113705304	32590453	6	G	C	0.089	0.152	0.012	2.4E-36	trans

824	FAS	P25445	Tumor necrosis factor receptor superfamily member 6	rs10887882	90782922	10	T	A	0.415	0.257	0.007	1.0E-285	cis
824	FAS	P25445	Tumor necrosis factor receptor superfamily member 6	rs123698	807442	19	C	G	0.604	0.051	0.007	3.0E-13	trans
824	FAS	P25445	Tumor necrosis factor receptor superfamily member 6	rs738409	44324727	22	G	C	0.217	0.060	0.008	2.8E-13	trans
825	FASLG	P48023	Tumor necrosis factor ligand superfamily member 6	rs79419452	172672473	1	T	A	0.844	0.099	0.009	1.5E-26	cis
825	FASLG	P48023	Tumor necrosis factor ligand superfamily member 6	rs11127048	27752463	2	G	A	0.382	0.051	0.007	3.2E-13	trans
825	FASLG	P48023	Tumor necrosis factor ligand superfamily member 6	rs13063578	47087837	3	T	A	0.602	0.169	0.007	1.0E-132	trans
825	FASLG	P48023	Tumor necrosis factor ligand superfamily member 6	rs13111860	142612879	4	A	G	0.288	0.054	0.007	5.3E-13	trans
825	FASLG	P48023	Tumor necrosis factor ligand superfamily member 6	rs9259888	29898381	6	T	C	0.353	0.079	0.008	1.0E-21	trans
825	FASLG	P48023	Tumor necrosis factor ligand superfamily member 6	rs117343599	38855747	8	G	C	0.977	0.174	0.023	3.5E-14	trans
825	FASLG	P48023	Tumor necrosis factor ligand superfamily member 6	rs3927680	16887366	9	A	T	0.620	0.054	0.007	9.6E-15	trans
825	FASLG	P48023	Tumor necrosis factor ligand superfamily member 6	rs8177655	6015119	10	A	G	0.329	0.075	0.007	3.1E-24	trans
825	FASLG	P48023	Tumor necrosis factor ligand superfamily member 6	rs2646421	8467155	10	C	G	0.379	0.052	0.007	5.6E-13	trans
825	FASLG	P48023	Tumor necrosis factor ligand superfamily member 6	rs978522	90748338	10	C	T	0.405	0.291	0.007	0.0E+00	trans
825	FASLG	P48023	Tumor necrosis factor ligand superfamily member 6	rs648710	126176376	11	A	T	0.257	0.109	0.008	5.0E-45	trans
825	FASLG	P48023	Tumor necrosis factor ligand superfamily member 6	rs748988	99786241	14	G	A	0.614	0.065	0.007	1.3E-20	trans
825	FASLG	P48023	Tumor necrosis factor ligand superfamily member 6	rs186021206	7069412	17	A	G	0.006	0.611	0.047	1.8E-39	trans
825	FASLG	P48023	Tumor necrosis factor ligand superfamily member 6	rs9916257	33797371	17	G	T	0.561	0.123	0.007	1.8E-73	trans
825	FASLG	P48023	Tumor necrosis factor ligand superfamily member 6	rs7221605	38070789	17	T	C	0.499	0.053	0.007	4.5E-15	trans
825	FASLG	P48023	Tumor necrosis factor ligand superfamily member 6	rs1205340	32923871	20	A	G	0.503	0.073	0.007	5.2E-27	trans
825	FASLG	P48023	Tumor necrosis factor ligand superfamily member 6	rs35477491	15273817	21	C	T	0.776	0.091	0.010	1.8E-21	trans
825	FASLG	P48023	Tumor necrosis factor ligand superfamily member 6	rs12628239	29096361	22	G	A	0.679	0.051	0.007	2.2E-12	trans
825	FASLG	P48023	Tumor necrosis factor ligand superfamily member 6	rs739141	39824450	22	C	T	0.338	0.062	0.007	3.8E-18	trans
826	FBLN2	P98095	Fibulin-2	rs333947	110470764	1	G	A	0.852	0.070	0.010	2.5E-12	trans
826	FBLN2	P98095	Fibulin-2	rs1260326	27730940	2	C	T	0.608	0.054	0.007	1.4E-13	trans
826	FBLN2	P98095	Fibulin-2	rs7623097	13613064	3	A	G	0.994	1.563	0.046	2.4E-252	cis
826	FBLN2	P98095	Fibulin-2	rs1010554	52542919	3	T	C	0.529	0.096	0.007	2.4E-39	trans
826	FBLN2	P98095	Fibulin-2	rs10769256	47378396	11	T	C	0.400	0.050	0.007	8.2E-12	trans
827	FBN2	P35556	Fibrillin-2	rs78821460	127874149	5	C	G	0.083	0.545	0.014	0.0E+00	cis
828	FBP1	P09467	Fructose-1,6-bisphosphatase 1	rs499206	97359479	9	G	A	0.925	0.575	0.014	0.0E+00	cis
828	FBP1	P09467	Fructose-1,6-bisphosphatase 1	rs10883451	101924418	10	T	C	0.498	0.057	0.007	7.3E-15	trans
828	FBP1	P09467	Fructose-1,6-bisphosphatase 1	rs10405357	54759666	19	T	C	0.565	0.074	0.007	2.3E-23	trans
828	FBP1	P09467	Fructose-1,6-bisphosphatase 1	rs738409	44324727	22	G	C	0.217	0.072	0.009	4.3E-16	trans
829	FCAMR	Q8WWV6	High affinity immunoglobulin alpha and immunoglobulin mu Fc receptor	rs188468174	25291697	1	T	C	0.014	0.238	0.031	2.6E-14	trans
829	FCAMR	Q8WWV6	High affinity immunoglobulin alpha and immunoglobulin mu Fc receptor	rs646776	109818530	1	T	C	0.778	0.163	0.009	1.4E-77	trans
829	FCAMR	Q8WWV6	High affinity immunoglobulin alpha and immunoglobulin mu Fc receptor	rs11119933	207140440	1	T	C	0.178	0.447	0.010	0.0E+00	cis
829	FCAMR	Q8WWV6	High affinity immunoglobulin alpha and immunoglobulin mu Fc receptor	rs1047891	211540507	2	A	C	0.313	0.058	0.008	2.7E-14	trans
829	FCAMR	Q8WWV6	High affinity immunoglobulin alpha and immunoglobulin mu Fc receptor	rs56819515	170712913	3	T	A	0.875	0.083	0.011	8.2E-15	trans
829	FCAMR	Q8WWV6	High affinity immunoglobulin alpha and immunoglobulin mu Fc receptor	rs2884401	148984082	4	G	C	0.256	0.055	0.008	8.2E-12	trans
829	FCAMR	Q8WWV6	High affinity immunoglobulin alpha and immunoglobulin mu Fc receptor	rs11154519	130362577	6	A	G	0.574	0.056	0.007	5.9E-15	trans
829	FCAMR	Q8WWV6	High affinity immunoglobulin alpha and immunoglobulin mu Fc receptor	rs4418728	94839724	10	G	T	0.549	0.070	0.007	2.5E-23	trans
829	FCAMR	Q8WWV6	High affinity immunoglobulin alpha and immunoglobulin mu Fc receptor	rs118088990	65473175	11	G	A	0.939	0.119	0.015	5.9E-16	trans
829	FCAMR	Q8WWV6	High affinity immunoglobulin alpha and immunoglobulin mu Fc receptor	rs28929474	94844947	14	T	C	0.021	0.203	0.025	1.6E-16	trans
829	FCAMR	Q8WWV6	High affinity immunoglobulin alpha and immunoglobulin mu Fc receptor	rs78357146	64305051	17	G	A	0.030	0.143	0.020	2.4E-12	trans
829	FCAMR	Q8WWV6	High affinity immunoglobulin alpha and immunoglobulin mu Fc receptor	rs344560	6665020	19	C	T	0.945	0.121	0.016	7.4E-15	trans
829	FCAMR	Q8WWV6	High affinity immunoglobulin alpha and immunoglobulin mu Fc receptor	rs2207132	39142516	20	G	A	0.967	0.159	0.020	1.1E-15	trans
829	FCAMR	Q8WWV6	High affinity immunoglobulin alpha and immunoglobulin mu Fc receptor	rs1800961	43042364	20	T	C	0.032	0.155	0.020	4.5E-15	trans
829	FCAMR	Q8WWV6	High affinity immunoglobulin alpha and immunoglobulin mu Fc receptor	rs2266959	21922904	22	T	G	0.187	0.086	0.009	1.4E-21	trans
829	FCAMR	Q8WWV6	High affinity immunoglobulin alpha and immunoglobulin mu Fc receptor	rs738409	44324727	22	G	C	0.217	0.074	0.008	2.0E-18	trans
830	FCAR	P24071	Immunoglobulin alpha Fc receptor	rs188468174	25291697	1	C	T	0.986	0.579	0.027	1.4E-105	trans
830	FCAR	P24071	Immunoglobulin alpha Fc receptor	rs2070901	161185058	1	T	G	0.270	0.052	0.007	7.9E-14	trans
830	FCAR	P24071	Immunoglobulin alpha Fc receptor	rs9862795	49915506	3	T	A	0.530	0.045	0.006	5.2E-13	trans
830	FCAR	P24071	Immunoglobulin alpha Fc receptor	rs2853950	31236175	6	T	C	0.437	0.060	0.006	6.1E-22	trans
830	FCAR	P24071	Immunoglobulin alpha Fc receptor	rs11155297	143825104	6	G	T	0.736	0.048	0.007	1.6E-11	trans
830	FCAR	P24071	Immunoglobulin alpha Fc receptor	rs6796	6502367	7	C	T	0.279	0.093	0.007	1.1E-40	trans
830	FCAR	P24071	Immunoglobulin alpha Fc receptor	rs73017385	126179575	11	A	G	0.240	0.093	0.007	2.2E-37	trans
830	FCAR	P24071	Immunoglobulin alpha Fc receptor	rs186021206	7069412	17	A	G	0.006	0.648	0.043	1.2E-51	trans
830	FCAR	P24071	Immunoglobulin alpha Fc receptor	rs3826331	38150492	17	T	C	0.402	0.081	0.006	4.4E-37	trans
830	FCAR	P24071	Immunoglobulin alpha Fc receptor	rs12608573	55391374	19	G	C	0.218	0.844	0.009	0.0E+00	cis
831	FCER1A	P12319	High affinity immunoglobulin epsilon receptor subunit alpha	rs61828219	159258641	1	A	C	0.166	0.174	0.010	8.9E-65	cis
832	FCER2	P06734	Low affinity immunoglobulin epsilon Fc receptor	rs1432296	61068167	2	T	C	0.142	0.074	0.010	5.6E-13	trans
832	FCER2	P06734	Low affinity immunoglobulin epsilon Fc receptor	rs112824187	158265308	5	G	A	0.924	0.149	0.014	2.0E-28	trans
832	FCER2	P06734	Low affinity immunoglobulin epsilon Fc receptor	rs2844606	31248432	6	A	G	0.582	0.089	0.007	2.7E-34	trans
832	FCER2	P06734	Low affinity immunoglobulin epsilon Fc receptor	rs9488914	116690849	6	T	C	0.357	0.095	0.008	7.4E-37	trans
832	FCER2	P06734	Low affinity immunoglobulin epsilon Fc receptor	rs17494629	7871531	7	T	G	0.798	0.060	0.009	1.4E-11	trans
832	FCER2	P06734	Low affinity immunoglobulin epsilon Fc receptor	rs62502726	24175523	8	T	C	0.849	0.077	0.010	1.0E-14	trans
832	FCER2	P06734	Low affinity immunoglobulin epsilon Fc receptor	rs7087507	63745689	10	G	A	0.349	0.053	0.007	1.3E-12	trans
832	FCER2	P06734	Low affinity immunoglobulin epsilon Fc receptor	rs12784975	98380137	10	T	C	0.808	0.078	0.009	4.3E-18	trans
832	FCER2	P06734	Low affinity immunoglobulin epsilon Fc receptor	rs374039502	108960385	13	A	T	0.021	0.193	0.027	4.7E-13	trans
832	FCER2	P06734	Low affinity immunoglobulin epsilon Fc receptor	rs9939427	86016091	16	A	G	0.231	0.069	0.009	5.6E-16	trans
832	FCER2	P06734	Low affinity immunoglobulin epsilon Fc receptor	rs35222145	38007321	17	T	G	0.508	0.092	0.007	6.5E-37	trans
832	FCER2	P06734	Low affinity immunoglobulin epsilon Fc receptor	rs2277989	7761812	19	A	G	0.513	0.292	0.007	0.0E+00	cis
833	FCGR2A	P12318	Low affinity immunoglobulin gamma Fc region receptor II-a	rs11810143	161480649	1	A	G	0.883	1.474	0.012	0.0E+00	cis
833	FCGR2A	P12318	Low affinity immunoglobulin gamma Fc region receptor II-a	rs62165726	134966562	2	C	A	0.963	0.116	0.015	3.2E-14	trans
833	FCGR2A	P12318	Low affinity immunoglobulin gamma Fc region receptor II-a	rs75071241	126232186	11	G	A	0.940	0.086	0.012	1.4E-12	trans
833	FCGR2A	P12318	Low affinity immunoglobulin gamma Fc region receptor II-a	rs186021206	7069412	17	A	G	0.006	1.087	0.040	1.1E-164	trans
834	FCGR2B	P31994	Low affinity immunoglobulin gamma Fc region receptor II-b	rs1801274	161479745	1	A	G	0.466	0.451	0.008	0.0E+00	cis
834	FCGR2B	P31994	Low affinity immunoglobulin gamma Fc region receptor II-b	rs4240356	145042250	4	C	G	0.452	0.049	0.007	1.3E-11	trans
834	FCGR2B	P31994	Low affinity immunoglobulin gamma Fc region receptor II-b	rs6115999	3699741	20	T	C	0.012	0.298	0.034	1.3E-18	trans
835	FCGR3B	O75015	Low affinity immunoglobulin gamma Fc region receptor III-B	rs3762281	156304782	1	G	A	0.505	0.080	0.007	3.5E-27	trans
835	FCGR3B	O75015	Low affinity immunoglobulin gamma Fc region receptor III-B	rs200688856	161599779	1	G	C	0.659	0.862	0.008	0.0E+00	cis
835	FCGR3B	O75015	Low affinity immunoglobulin gamma Fc region receptor III-B	rs34860591	98607464	3	C	T	0.085	0.087	0.012	5.1E-14	trans
835	FCGR3B	O75015	Low affinity immunoglobulin gamma Fc region receptor III-B	rs28371261	32658727	6	G	A	0.887	0.076	0.010	5.8E-14	trans
835	FCGR3B	O75015	Low affinity immunoglobulin gamma Fc region receptor III-B	rs6796	6502367	7	C	T	0.279	0.061	0.007	1.3E-17	trans
835	FCGR3B	O75015	Low affinity immunoglobulin gamma Fc region receptor III-B	rs7795319	50353851	7	C	T	0.655	0.058	0.007	1.3E-17	trans
835	FCGR3B	O75015	Low affinity immunoglobulin gamma Fc region receptor III-B	rs73017399	126186312	11	C	G	0.239	0.200	0.008	1.6E-152	trans
835	FCGR3B	O75015	Low affinity immunoglobulin gamma Fc region receptor III-B	rs10138570	65860038	14	A	G	0.468	0.070	0.006	3.1E-27	trans
835	FCGR3B	O75015	Low affinity immunoglobulin gamma Fc region receptor III-B	rs36082322	101749641	15	C	T	0.110	0.079	0.010	1.4E-14	trans
835	FCGR3B	O75015	Low affinity immunoglobulin gamma Fc region receptor III-B	rs186021206	7069412	17	A	G	0.006	0.612	0.044	1.3E-43	trans
835	FCGR3B	O75015	Low affinity immunoglobulin gamma Fc region receptor III-B	rs2241244	38153554	17	G	C	0.463	0.084	0.006	6.8E-39	trans
835	FCGR3B	O75015	Low affinity immunoglobulin gamma Fc region receptor III-B	rs4760	44153100	19	A	G	0.844	0.279	0.009	1.2E-216	trans
836	FCN1	O00602	Ficolin-1	rs72550870	11106666	1	C	T	0.030	0.173	0.020	1.4E-17	trans
836	FCN1	O00602	Ficolin-1	rs6542680	3640142	2	T	C	0.821	0.158	0.009	5.2E-67	trans
836	FCN1	O00602	Ficolin-1	rs1486236	180739450	2	A	C	0.373	0.140	0.007	5.9E-81	trans
836	FCN1	O00602	Ficolin-1	rs2304456	186445052	3	G	T	0.111	0.147	0.011	2.2E-39	trans
836	FCN1	O00602	Ficolin-1	rs115216147	70665526	5	T	A	0.933	0.105	0.014	1.9E-13	trans
836	FCN1	O00602	Ficolin-1	rs4585607	32624407	6	G	A	0.498	0.064	0.008	8.0E-17	trans
836	FCN1	O00602	Ficolin-1	rs199980439	73007087	7	C	CA	0.878	0.075	0.011	1.7E-12	trans
836	FCN1	O00602	Ficolin-1	rs41341748	16012594	8	A	G	0.011	0.335	0.033	1.1E-23	trans
836	FCN1	O00602	Ficolin-1	rs11103604	137857446	9	T	G	0.266	0.442	0.008	0.0E+00	cis
836	FCN1	O00602	Ficolin-1	rs2511241	72945341	11	T	C	0.930	0.105	0.014	2.0E-14	trans
836	FCN1	O00602	Ficolin-1	rs112771035	126225876	11	C	G	0.929	0.248	0.014	6.2E-74	trans
836	FCN1	O00602	Ficolin-1	rs6503533	38184580	17	C	T	0.384	0.052	0.007	6.3E-13	trans
836	FCN1	O00602	Ficolin-1	rs12986064	54755133	19	T	C	0.492	0.055	0.007	2.3E-14	trans
836	FCN1	O00602	Ficolin-1	rs6115999	3699741	20	T	C	0.012	0.293	0.033	3.3E-19	trans
837	FCN2	Q15485	Ficolin-2	rs72550870	11106666	1	T	C	0.970	0.216	0.018	2.6E-32	trans
837	FCN2	Q15485	Ficolin-2	rs1260326	27730940	2	T	C	0.392	0.045	0.006	5.2E-12	trans
837	FCN2	Q15485	Ficolin-2	rs35677470	58183636	3	A	G	0.083	0.128	0.011	5.3E-29	trans
837	FCN2	Q15485	Ficolin-2	rs62276455	98417481	3	T	A	0.558	0.047	0.006	2.9E-13	trans
837	FCN2	Q15485	Ficolin-2	rs11386832	96121370	5	TA	T	0.738	0.140	0.007	5.4E-86	trans
837	FCN2	Q15485	Ficolin-2	rs111331197	7883468	6	T	C	0.011	0.278	0.030	3.4E-20	trans
837	FCN2	Q15485	Ficolin-2	rs7041446	137776054	9	G	A	0.540	0.620	0.007	0.0E+00	cis
837	FCN2	Q15485	Ficolin-2	rs174533	61549025	11	A	G	0.346	0.047	0.007	3.9E-12	trans
837	FCN2	Q15485	Ficolin-2	rs7122830	114444127	11	G	A	0.867	0.473	0.010	0.0E+00	trans
838	FCRL1	Q96LA6	Fc receptor-like protein 1	rs4971154	157771880	1	C	T	0.497	0.358	0.007	0.0E+00	cis
838	FCRL1	Q96LA6	Fc receptor-like protein 1	rs2009581	111807677	2	G	A	0.727	0.055	0.008	4.8E-12	trans
838	FCRL1	Q96LA6	Fc receptor-like protein 1	rs62292644	186738896	3	A	C	0.615	0.068	0.007	2.7E-20	trans
838	FCRL1	Q96LA6	Fc receptor-like protein 1	rs17361097	153001219	4	A	G	0.438	0.056	0.007	1.1E-14	trans
838	FCRL1	Q96LA6	Fc receptor-like protein 1	rs112824187	158265308	5	G	A	0.924	0.160	0.013	1.7E-32	trans
838	FCRL1	Q96LA6	Fc receptor-like protein 1	rs1226013	10539289	6	G	T	0.551	0.097	0.007	7.2E-42	trans
838	FCRL1	Q96LA6	Fc receptor-like protein 1	rs9280782	29905513	6	C	CA	0.492	0.068	0.007	9.3E-20	trans
838	FCRL1	Q96LA6	Fc receptor-like protein 1	rs4245597	63725942	10	G	A	0.346	0.111	0.007	2.7E-50	trans
838	FCRL1	Q96LA6	Fc receptor-like protein 1	rs12784975	98380137	10	T	C	0.808	0.072	0.009	1.3E-15	trans
838	FCRL1	Q96LA6	Fc receptor-like protein 1	rs76428106	28604007	13	C	T	0.013	0.298	0.033	5.3E-20	trans
838	FCRL1	Q96LA6	Fc receptor-like protein 1	rs374039502	108960385	13	A	T	0.021	0.311	0.027	1.5E-31	trans
838	FCRL1	Q96LA6	Fc receptor-like protein 1	rs9927316	86016401	16	G	C	0.239	0.092	0.008	8.4E-28	trans
838	FCRL1	Q96LA6	Fc receptor-like protein 1	rs186021206	7069412	17	A	G	0.006	0.711	0.049	4.1E-48	trans
838	FCRL1	Q96LA6	Fc receptor-like protein 1	rs34557412	16852187	17	G	A	0.007	0.662	0.043	2.2E-53	trans
838	FCRL1	Q96LA6	Fc receptor-like protein 1	rs35222145	38007321	17	T	G	0.508	0.082	0.007	2.7E-30	trans
838	FCRL1	Q96LA6	Fc receptor-like protein 1	rs77542162	67081278	17	G	A	0.023	0.174	0.024	4.0E-13	trans
838	FCRL1	Q96LA6	Fc receptor-like protein 1	rs79966604	33752658	19	GGT	G	0.337	0.070	0.008	3.3E-20	trans
838	FCRL1	Q96LA6	Fc receptor-like protein 1	rs73165110	42291856	22	G	A	0.921	0.123	0.013	1.1E-20	trans
839	FCRL2	Q96LA5	Fc receptor-like protein 2	rs188468174	25291697	1	C	T	0.986	0.298	0.031	1.1E-21	trans
839	FCRL2	Q96LA5	Fc receptor-like protein 2	rs12568320	157744004	1	G	A	0.168	0.767	0.010	0.0E+00	cis
839	FCRL2	Q96LA5	Fc receptor-like protein 2	rs72832854	111590770	2	T	G	0.975	0.150	0.021	1.7E-12	trans
839	FCRL2	Q96LA5	Fc receptor-like protein 2	rs5001409	186735690	3	A	C	0.609	0.071	0.007	6.8E-27	trans
839	FCRL2	Q96LA5	Fc receptor-like protein 2	rs9295574	10531244	6	A	G	0.434	0.067	0.007	6.9E-24	trans
839	FCRL2	Q96LA5	Fc receptor-like protein 2	rs114619738	29884644	6	C	T	0.010	1.111	0.034	1.1E-237	trans
839	FCRL2	Q96LA5	Fc receptor-like protein 2	rs12554596	37002142	9	A	G	0.796	0.071	0.008	1.4E-18	trans
839	FCRL2	Q96LA5	Fc receptor-like protein 2	rs10791824	65559266	11	G	A	0.566	0.061	0.007	7.9E-21	trans
839	FCRL2	Q96LA5	Fc receptor-like protein 2	rs374039502	108960385	13	A	T	0.021	0.408	0.024	1.4E-63	trans
839	FCRL2	Q96LA5	Fc receptor-like protein 2	rs186021206	7069412	17	A	G	0.006	0.605	0.045	4.3E-41	trans
839	FCRL2	Q96LA5	Fc receptor-like protein 2	rs34557412	16852187	17	G	A	0.007	0.820	0.040	1.5E-94	trans
839	FCRL2	Q96LA5	Fc receptor-like protein 2	rs34931250	66879927	17	T	C	0.061	0.404	0.014	1.0E-189	trans

839	FCRL2	Q96LA5	Fc receptor-like protein 2	rs11085015	3369572	19	G	T	0.801	0.095	0.008	9.0E-30	trans
839	FCRL2	Q96LA5	Fc receptor-like protein 2	rs73165110	42291856	22	G	A	0.921	0.102	0.012	2.2E-17	trans
840	FCRL3	Q96P31	Fc receptor-like protein 3	rs7522061	157668390	1	C	T	0.483	0.965	0.008	0.0E+00	cis
840	FCRL3	Q96P31	Fc receptor-like protein 3	rs2009581	111807677	2	G	A	0.728	0.044	0.006	3.5E-12	trans
840	FCRL3	Q96P31	Fc receptor-like protein 3	rs186021206	7069412	17	A	G	0.006	0.535	0.039	1.8E-43	trans
840	FCRL3	Q96P31	Fc receptor-like protein 3	rs34557412	16852187	17	G	A	0.007	0.329	0.034	6.4E-22	trans
841	FCRL5	Q96RD9	Fc receptor-like protein 5	rs188468174	25291697	1	C	T	0.986	0.534	0.031	2.2E-67	trans
841	FCRL5	Q96RD9	Fc receptor-like protein 5	rs61804164	161623025	1	G	C	0.876	0.078	0.011	2.5E-12	trans
841	FCRL5	Q96RD9	Fc receptor-like protein 5	rs7452226	31303394	6	A	G	0.471	0.065	0.007	3.8E-19	trans
841	FCRL5	Q96RD9	Fc receptor-like protein 5	rs6583437	50350267	7	G	A	0.623	0.057	0.007	1.6E-16	trans
841	FCRL5	Q96RD9	Fc receptor-like protein 5	rs76428106	28604007	13	C	T	0.013	0.233	0.031	8.8E-14	trans
841	FCRL5	Q96RD9	Fc receptor-like protein 5	rs3803286	103246470	14	A	G	0.334	0.066	0.007	3.9E-20	trans
841	FCRL5	Q96RD9	Fc receptor-like protein 5	rs11624491	106188045	14	T	C	0.656	0.168	0.007	2.9E-118	trans
841	FCRL5	Q96RD9	Fc receptor-like protein 5	rs3803800	7462969	17	A	G	0.211	0.075	0.008	1.4E-19	trans
841	FCRL5	Q96RD9	Fc receptor-like protein 5	rs34562254	16842991	17	A	G	0.097	0.164	0.011	2.2E-47	trans
841	FCRL5	Q96RD9	Fc receptor-like protein 5	rs754292	16699813	19	C	T	0.455	0.046	0.007	8.0E-12	trans
841	FCRL5	Q96RD9	Fc receptor-like protein 5	rs5906672	48326779	X	T	C	0.042	0.132	0.014	4.7E-21	trans
842	FCRL6	Q6DN72	Fc receptor-like protein 6	rs55650803	159783148	1	C	T	0.808	0.891	0.009	0.0E+00	cis
842	FCRL6	Q6DN72	Fc receptor-like protein 6	rs2549006	131827065	5	G	A	0.667	0.059	0.007	2.5E-18	trans
842	FCRL6	Q6DN72	Fc receptor-like protein 6	rs9270747	32568292	6	G	A	0.658	0.126	0.007	5.1E-75	trans
842	FCRL6	Q6DN72	Fc receptor-like protein 6	rs482246	128060772	11	A	G	0.661	0.074	0.007	1.1E-28	trans
842	FCRL6	Q6DN72	Fc receptor-like protein 6	rs186021206	7069412	17	A	G	0.006	0.312	0.044	9.6E-13	trans
842	FCRL6	Q6DN72	Fc receptor-like protein 6	rs58745116	33803592	17	G	A	0.610	0.085	0.007	1.0E-38	trans
842	FCRL6	Q6DN72	Fc receptor-like protein 6	rs2255166	79213562	17	C	T	0.437	0.044	0.006	3.7E-12	trans
843	FCRLB	Q6BAA4	Fc receptor-like B	rs188468174	25291697	1	C	T	0.986	0.322	0.032	2.4E-23	trans
843	FCRLB	Q6BAA4	Fc receptor-like B	rs61801180	161688764	1	C	G	0.061	1.363	0.016	0.0E+00	cis
843	FCRLB	Q6BAA4	Fc receptor-like B	rs1973612	187169167	4	C	T	0.487	0.060	0.006	1.0E-22	trans
843	FCRLB	Q6BAA4	Fc receptor-like B	rs7789913	50352695	7	T	C	0.401	0.063	0.006	2.9E-24	trans
843	FCRLB	Q6BAA4	Fc receptor-like B	rs73169662	150922306	7	C	T	0.115	0.102	0.010	1.4E-26	trans
843	FCRLB	Q6BAA4	Fc receptor-like B	rs7232	59940599	11	A	T	0.373	0.044	0.006	3.2E-12	trans
843	FCRLB	Q6BAA4	Fc receptor-like B	rs186021206	7069412	17	A	G	0.006	0.340	0.042	1.0E-15	trans
844	FEN1	P39748	Flap endonuclease 1	rs10418046	54327869	19	G	T	0.212	0.064	0.009	2.4E-12	trans
845	FES	P07332	Tyrosine-protein kinase Fes/Fps	rs1894401	91429042	15	A	G	0.523	0.133	0.008	1.0E-67	cis
846	FETUB	Q9UGM5	Fetuin-B	rs1862069	169933741	2	G	A	0.462	0.050	0.007	1.4E-12	trans
846	FETUB	Q9UGM5	Fetuin-B	rs114780909	186360255	3	T	A	0.991	2.139	0.040	0.0E+00	cis
846	FETUB	Q9UGM5	Fetuin-B	rs28601761	126500031	8	C	G	0.581	0.118	0.007	1.3E-60	trans
846	FETUB	Q9UGM5	Fetuin-B	rs11438680	65174698	10	GA	G	0.461	0.080	0.007	8.5E-30	trans
846	FETUB	Q9UGM5	Fetuin-B	rs150844304	43726625	15	A	C	0.976	0.265	0.023	2.1E-31	trans
847	FGA	P02671	Fibrinogen alpha chain	rs2228145	154426970	1	A	C	0.594	0.051	0.007	4.1E-12	trans
847	FGA	P02671	Fibrinogen alpha chain	rs55709272	113867288	2	C	T	0.439	0.055	0.007	4.9E-14	trans
847	FGA	P02671	Fibrinogen alpha chain	rs140473879	155479065	4	C	T	0.996	1.307	0.061	7.6E-101	cis
847	FGA	P02671	Fibrinogen alpha chain	rs10900807	131757480	5	G	C	0.816	0.079	0.009	5.4E-17	trans
848	FGD3	Q5JSP0	FYVE, RhoGEF and PH domain-containing protein 3	rs6993770	106581528	8	A	T	0.714	0.056	0.008	9.6E-12	trans
849	FGF19	O95750	Fibroblast growth factor 19	rs2276890	39460490	4	G	C	0.328	0.106	0.008	4.0E-40	trans
849	FGF19	O95750	Fibroblast growth factor 19	rs10504255	59398461	8	A	G	0.665	0.098	0.008	3.9E-35	trans
849	FGF19	O95750	Fibroblast growth factor 19	rs8176741	136131461	9	A	G	0.061	0.166	0.016	1.5E-26	trans
849	FGF19	O95750	Fibroblast growth factor 19	rs708686	5840619	19	T	C	0.268	0.075	0.009	8.3E-19	trans
849	FGF19	O95750	Fibroblast growth factor 19	rs492602	49206417	19	A	G	0.490	0.225	0.008	3.5E-193	trans
850	FGF2	P09038	Fibroblast growth factor 2	rs1434282	199010721	1	T	C	0.726	0.058	0.008	1.3E-14	trans
850	FGF2	P09038	Fibroblast growth factor 2	rs1354034	56849749	3	C	T	0.604	0.056	0.007	1.4E-16	trans
850	FGF2	P09038	Fibroblast growth factor 2	rs2922979	123748616	4	C	G	0.691	0.711	0.008	0.0E+00	cis
850	FGF2	P09038	Fibroblast growth factor 2	rs6993770	106581528	8	A	T	0.713	0.124	0.007	2.3E-62	trans
850	FGF2	P09038	Fibroblast growth factor 2	rs3780420	100677166	9	C	T	0.364	0.053	0.007	4.9E-14	trans
850	FGF2	P09038	Fibroblast growth factor 2	rs34416903	88559245	16	C	T	0.280	0.053	0.008	1.8E-12	trans
851	FGF21	Q9NSA1	Fibroblast growth factor 21	rs1260326	27730940	2	T	C	0.391	0.136	0.008	8.2E-71	trans
851	FGF21	Q9NSA1	Fibroblast growth factor 21	rs1047891	211540507	2	C	A	0.687	0.064	0.008	2.5E-15	trans
851	FGF21	Q9NSA1	Fibroblast growth factor 21	rs13234378	73026151	7	A	T	0.873	0.157	0.011	4.2E-45	trans
851	FGF21	Q9NSA1	Fibroblast growth factor 21	rs3779195	97993362	7	T	A	0.815	0.079	0.010	2.6E-16	trans
851	FGF21	Q9NSA1	Fibroblast growth factor 21	rs2954021	126482077	8	A	G	0.496	0.055	0.007	1.1E-13	trans
851	FGF21	Q9NSA1	Fibroblast growth factor 21	rs62074055	45771933	17	G	C	0.497	0.054	0.007	3.1E-13	trans
851	FGF21	Q9NSA1	Fibroblast growth factor 21	rs838131	49260677	19	A	C	0.543	0.123	0.008	4.7E-58	cis
852	FGF23	Q9GZV9	Fibroblast growth factor 23	rs17199004	190452957	2	G	T	0.264	0.077	0.008	1.0E-19	trans
852	FGF23	Q9GZV9	Fibroblast growth factor 23	rs6862195	176822512	5	G	T	0.689	0.070	0.008	3.8E-18	trans
852	FGF23	Q9GZV9	Fibroblast growth factor 23	rs6561643	33509079	13	T	A	0.347	0.077	0.008	4.4E-22	trans
852	FGF23	Q9GZV9	Fibroblast growth factor 23	rs6127099	52731402	20	A	T	0.722	0.096	0.008	8.9E-30	trans
853	FGF5	P12034	Fibroblast growth factor 5	rs12509595	81182554	4	C	T	0.293	0.682	0.008	0.0E+00	cis
853	FGF5	P12034	Fibroblast growth factor 5	rs17580	94847262	14	A	T	0.050	0.112	0.014	4.8E-15	trans
854	FGFBP1	Q14512	Fibroblast growth factor-binding protein 1	rs7528419	109817192	1	A	G	0.778	0.106	0.009	2.0E-34	trans
854	FGFBP1	Q14512	Fibroblast growth factor-binding protein 1	rs61816761	152285861	1	A	G	0.024	0.175	0.025	3.4E-12	trans
854	FGFBP1	Q14512	Fibroblast growth factor-binding protein 1	rs1260326	27730940	2	C	T	0.609	0.051	0.007	7.7E-12	trans
854	FGFBP1	Q14512	Fibroblast growth factor-binding protein 1	rs6800909	187501571	3	G	T	0.295	0.064	0.008	6.0E-16	trans
854	FGFBP1	Q14512	Fibroblast growth factor-binding protein 1	rs9968276	15949551	4	G	A	0.798	0.121	0.009	6.1E-41	cis
854	FGFBP1	Q14512	Fibroblast growth factor-binding protein 1	rs61738275	31380198	6	T	C	0.152	0.071	0.010	2.0E-12	trans
854	FGFBP1	Q14512	Fibroblast growth factor-binding protein 1	rs1532085	58683366	15	A	G	0.383	0.051	0.007	7.8E-12	trans
854	FGFBP1	Q14512	Fibroblast growth factor-binding protein 1	rs77542162	67081278	17	G	A	0.023	0.179	0.024	1.0E-13	trans
854	FGFBP1	Q14512	Fibroblast growth factor-binding protein 1	rs151113958	11191201	19	A	AG	0.880	0.083	0.011	9.4E-14	trans
854	FGFBP1	Q14512	Fibroblast growth factor-binding protein 1	rs1065853	45413233	19	G	T	0.918	0.282	0.013	6.1E-100	trans
854	FGFBP1	Q14512	Fibroblast growth factor-binding protein 1	rs2074735	31535872	22	C	G	0.064	0.145	0.015	8.3E-23	trans
855	FGFBP2	Q9BYJ0	Fibroblast growth factor-binding protein 2	rs900400	156798775	3	T	C	0.602	0.056	0.007	1.1E-17	trans
855	FGFBP2	Q9BYJ0	Fibroblast growth factor-binding protein 2	rs35700679	15960246	4	G	A	0.148	0.719	0.010	0.0E+00	cis
855	FGFBP2	Q9BYJ0	Fibroblast growth factor-binding protein 2	rs9272302	32603854	6	C	T	0.835	0.081	0.009	9.8E-20	trans
855	FGFBP2	Q9BYJ0	Fibroblast growth factor-binding protein 2	rs836468	6436722	7	A	T	0.312	0.048	0.007	1.6E-12	trans
855	FGFBP2	Q9BYJ0	Fibroblast growth factor-binding protein 2	rs2740488	107661742	9	A	C	0.733	0.058	0.007	8.3E-16	trans
855	FGFBP2	Q9BYJ0	Fibroblast growth factor-binding protein 2	rs2070895	58723939	15	A	G	0.221	0.076	0.008	4.2E-23	trans
855	FGFBP2	Q9BYJ0	Fibroblast growth factor-binding protein 2	rs36229491	56994244	16	TA	T	0.324	0.092	0.007	6.8E-42	trans
855	FGFBP2	Q9BYJ0	Fibroblast growth factor-binding protein 2	rs34931250	66879927	17	T	C	0.061	1.176	0.015	0.0E+00	trans
855	FGFBP2	Q9BYJ0	Fibroblast growth factor-binding protein 2	rs77960347	47109955	18	G	A	0.013	0.209	0.028	8.7E-14	trans
855	FGFBP2	Q9BYJ0	Fibroblast growth factor-binding protein 2	rs1065853	45413233	19	T	G	0.081	0.099	0.012	3.0E-17	trans
856	FGFBP3	Q8TAT2	Fibroblast growth factor-binding protein 3	rs12740374	109817590	1	G	T	0.778	0.097	0.008	4.4E-31	trans
856	FGFBP3	Q8TAT2	Fibroblast growth factor-binding protein 3	rs1434282	199010721	1	C	T	0.274	0.055	0.008	2.4E-12	trans
856	FGFBP3	Q8TAT2	Fibroblast growth factor-binding protein 3	rs11711157	194061826	3	C	T	0.722	0.068	0.008	2.2E-18	trans
856	FGFBP3	Q8TAT2	Fibroblast growth factor-binding protein 3	rs11734099	6891435	4	A	G	0.174	0.065	0.009	2.0E-12	trans
856	FGFBP3	Q8TAT2	Fibroblast growth factor-binding protein 3	rs6923313	31241370	6	C	T	0.344	0.055	0.008	3.1E-13	trans
856	FGFBP3	Q8TAT2	Fibroblast growth factor-binding protein 3	rs4252117	161143376	6	A	G	0.705	0.345	0.008	0.0E+00	trans
856	FGFBP3	Q8TAT2	Fibroblast growth factor-binding protein 3	rs13269700	22570608	8	G	A	0.323	0.054	0.007	5.4E-13	trans
856	FGFBP3	Q8TAT2	Fibroblast growth factor-binding protein 3	rs6993770	106581528	8	A	T	0.713	0.080	0.008	2.3E-25	trans
856	FGFBP3	Q8TAT2	Fibroblast growth factor-binding protein 3	rs61751507	101829514	10	C	T	0.957	0.252	0.018	4.2E-46	trans
856	FGFBP3	Q8TAT2	Fibroblast growth factor-binding protein 3	rs17127600	92279983	14	T	G	0.830	0.078	0.009	2.5E-17	trans
856	FGFBP3	Q8TAT2	Fibroblast growth factor-binding protein 3	rs61658003	55544203	19	A	C	0.843	0.087	0.010	3.9E-19	trans
857	FGFR2	P21802	Fibroblast growth factor receptor 2	rs924204	16513926	1	G	A	0.589	0.059	0.007	2.2E-16	trans
857	FGFR2	P21802	Fibroblast growth factor receptor 2	rs6968170	73020676	7	G	A	0.717	0.058	0.008	1.5E-13	trans
857	FGFR2	P21802	Fibroblast growth factor receptor 2	rs7826120	59371725	8	C	T	0.670	0.058	0.008	1.1E-14	trans
857	FGFR2	P21802	Fibroblast growth factor receptor 2	rs2001945	126477978	8	G	C	0.481	0.057	0.007	6.0E-16	trans
857	FGFR2	P21802	Fibroblast growth factor receptor 2	rs2981430	123311698	10	G	A	0.420	0.113	0.007	5.5E-56	cis
857	FGFR2	P21802	Fibroblast growth factor receptor 2	rs111632263	68233122	16	A	T	0.128	0.074	0.011	3.1E-12	trans
857	FGFR2	P21802	Fibroblast growth factor receptor 2	rs186021206	7069412	17	A	G	0.006	0.519	0.048	8.4E-27	trans
858	FGFR4	P22455	Fibroblast growth factor receptor 4	rs10753239	31362224	1	T	C	0.540	0.043	0.005	1.6E-16	trans
858	FGFR4	P22455	Fibroblast growth factor receptor 4	rs5402	170727739	3	T	A	0.880	0.086	0.008	1.7E-27	trans
858	FGFR4	P22455	Fibroblast growth factor receptor 4	rs6855868	39454293	4	A	G	0.328	0.397	0.006	0.0E+00	trans
858	FGFR4	P22455	Fibroblast growth factor receptor 4	rs351855	176520243	5	A	G	0.296	1.009	0.008	0.0E+00	cis
858	FGFR4	P22455	Fibroblast growth factor receptor 4	rs2253491	31249217	6	A	G	0.205	0.089	0.006	6.9E-44	trans
858	FGFR4	P22455	Fibroblast growth factor receptor 4	rs7799998	128738950	7	A	G	0.550	0.054	0.005	9.5E-26	trans
858	FGFR4	P22455	Fibroblast growth factor receptor 4	rs11783515	59320389	8	G	A	0.680	0.043	0.006	5.2E-15	trans
858	FGFR4	P22455	Fibroblast growth factor receptor 4	rs1131773	95840256	9	G	A	0.144	0.058	0.007	1.6E-15	trans
858	FGFR4	P22455	Fibroblast growth factor receptor 4	rs9633740	82265271	10	G	A	0.796	0.068	0.006	5.8E-27	trans
858	FGFR4	P22455	Fibroblast growth factor receptor 4	rs174581	61606683	11	A	G	0.354	0.055	0.005	6.8E-25	trans
858	FGFR4	P22455	Fibroblast growth factor receptor 4	rs112001035	66823805	17	G	A	0.939	0.105	0.011	5.5E-22	trans
858	FGFR4	P22455	Fibroblast growth factor receptor 4	rs41292412	56118358	18	T	C	0.013	0.506	0.023	5.5E-104	trans
858	FGFR4	P22455	Fibroblast growth factor receptor 4	rs33950747	36339247	19	T	C	0.075	0.078	0.010	1.1E-15	trans
859	FGL1	Q08830	Fibrinogen-like protein 1	rs1260326	27730940	2	T	C	0.391	0.093	0.006	1.9E-53	trans
859	FGL1	Q08830	Fibrinogen-like protein 1	rs3739404	17739843	8	A	C	0.691	0.657	0.008	0.0E+00	cis
859	FGL1	Q08830	Fibrinogen-like protein 1	rs2351958	16248020	11	A	C	0.387	0.048	0.006	2.6E-15	trans
859	FGL1	Q08830	Fibrinogen-like protein 1	rs12444166	79709950	16	A	G	0.322	0.046	0.006	4.5E-13	trans
859	FGL1	Q08830	Fibrinogen-like protein 1	rs186021206	7069412	17	G	A	0.994	0.276	0.041	1.3E-11	trans
859	FGL1	Q08830	Fibrinogen-like protein 1	rs731170	55176262	19	A	G	0.313	0.043	0.006	1.0E-11	trans
860	FGR	P09769	Tyrosine-protein kinase Fgr	rs3737803	27960792	1	G	A	0.927	0.179	0.014	4.8E-36	cis
861	FH	P07954	Fumarate hydratase, mitochondrial	rs141575023	106370370	14	T	C	0.138	0.087	0.011	1.2E-14	trans
862	FHIT	P49789	Bis(5'-adenosyl)-triphosphatase	rs1354034	56849749	3	C	T	0.604	0.063	0.008	3.5E-16	trans
862	FHIT	P49789	Bis(5'-adenosyl)-triphosphatase	rs13070475	59969336	3	A	G	0.644	0.089	0.008	4.7E-29	cis
863	FIS1	Q9Y3D6	Mitochondrial fission 1 protein	rs1354034	56849749	3	C	T	0.604	0.063	0.008	2.1E-16	trans
863	FIS1	Q9Y3D6	Mitochondrial fission 1 protein	rs7778271	100874354	7	T	C	0.946	0.346	0.017	1.5E-97	cis
863	FIS1	Q9Y3D6	Mitochondrial fission 1 protein	rs342293	106372219	7	C	G	0.541	0.051	0.008	1.3E-11	trans
863	FIS1	Q9Y3D6	Mitochondrial fission 1 protein	rs6993770	106581528	8	A	T	0.713	0.056	0.008	1.6E-11	trans
863	FIS1	Q9Y3D6	Mitochondrial fission 1 protein	rs10733789	64948684	10	C	T	0.313	0.060	0.008	2.2E-13	trans
863	FIS1	Q9Y3D6	Mitochondrial fission 1 protein	rs58807415	268718	11	G	A	0.244	0.059	0.009	1.5E-11	trans
864	FKBP14	Q9NWM8	Peptidyl-prolyl cis-trans isomerase FKBP14	rs1354034	56849749	3	C	T	0.604	0.111	0.008	9.9E-48	trans
864	FKBP14	Q9NWM8	Peptidyl-prolyl cis-trans isomerase FKBP14	rs114694170	88180196	5	C	T	0.060	0.113	0.016	1.3E-12	trans
864	FKBP14	Q9NWM8	Peptidyl-prolyl cis-trans isomerase FKBP14	rs181397928	30136191	7	G	C	0.999	1.018	0.142	6.7E-13	cis
864	FKBP14	Q9NWM8	Peptidyl-prolyl cis-trans isomerase FKBP14	rs6993770	106581528	8	A	T	0.714	0.068	0.008	4.1E-16	trans
864	FKBP14	Q9NWM8	Peptidyl-prolyl cis-trans isomerase FKBP14	rs10820606	99192919	9	A	C	0.771	0.068	0.009	8.3E-14	trans
864	FKBP14	Q9NWM8	Peptidyl-prolyl cis-trans isomerase FKBP14	rs11502185	180258	11	C	T	0.260	0.073	0.010	3.7E-14	trans
864	FKBP14	Q9NWM8	Peptidyl-prolyl cis-trans isomerase FKBP14	rs351978	806256	19	G	A	0.583	0.052	0.008	1.4E-11	trans
865	FKBP1B	P68106	Peptidyl-prolyl cis-trans isomerase FKBP1B	rs872169	24259188	2	C	G	0.795	0.255	0.009	5.3E-164	cis

865	FKBP1B	P68106	Peptidyl-prolyl cis-trans isomerase FKBP1B	rs1354034	56849749	3	C	T	0.604	0.081	0.008	9.6E-27	trans
865	FKBP1B	P68106	Peptidyl-prolyl cis-trans isomerase FKBP1B	rs342296	106372903	7	G	A	0.543	0.069	0.007	1.9E-20	trans
865	FKBP1B	P68106	Peptidyl-prolyl cis-trans isomerase FKBP1B	rs7916868	64988931	10	T	A	0.502	0.072	0.007	6.3E-22	trans
865	FKBP1B	P68106	Peptidyl-prolyl cis-trans isomerase FKBP1B	rs11502185	180258	11	C	T	0.260	0.073	0.010	2.7E-14	trans
865	FKBP1B	P68106	Peptidyl-prolyl cis-trans isomerase FKBP1B	rs200309755	27845774	17	T	C	0.537	0.066	0.008	1.6E-17	trans
866	FKBP4	Q02790	Peptidyl-prolyl cis-trans isomerase FKBP4	rs1354034	56849749	3	C	T	0.604	0.084	0.008	9.4E-28	trans
866	FKBP4	Q02790	Peptidyl-prolyl cis-trans isomerase FKBP4	rs342298	106373646	7	C	T	0.546	0.057	0.008	6.6E-14	trans
866	FKBP4	Q02790	Peptidyl-prolyl cis-trans isomerase FKBP4	rs6993770	106581528	8	A	T	0.713	0.062	0.008	7.9E-14	trans
866	FKBP4	Q02790	Peptidyl-prolyl cis-trans isomerase FKBP4	rs201265508	180231	11	G	A	0.294	0.071	0.009	5.6E-14	trans
867	FKBP5	Q13451	Peptidyl-prolyl cis-trans isomerase FKBP5	rs1354034	56849749	3	C	T	0.604	0.052	0.008	7.4E-12	trans
867	FKBP5	Q13451	Peptidyl-prolyl cis-trans isomerase FKBP5	rs17622656	131820997	5	G	A	0.611	0.052	0.008	8.7E-12	trans
867	FKBP5	Q13451	Peptidyl-prolyl cis-trans isomerase FKBP5	rs2817032	35688619	6	C	T	0.287	0.209	0.008	5.5E-138	cis
867	FKBP5	Q13451	Peptidyl-prolyl cis-trans isomerase FKBP5	rs342293	106372219	7	C	G	0.541	0.051	0.007	8.5E-12	trans
868	FKBPL	Q9UIM3	FK506-binding protein-like	rs1354034	56849749	3	C	T	0.605	0.055	0.008	4.6E-13	trans
868	FKBPL	Q9UIM3	FK506-binding protein-like	rs9469087	32129465	6	A	G	0.996	0.620	0.061	5.7E-24	cis
869	FLI1	Q01543	Friend leukemia integration 1 transcription factor	rs1354034	56849749	3	C	T	0.604	0.060	0.008	1.4E-14	trans
869	FLI1	Q01543	Friend leukemia integration 1 transcription factor	rs6993770	106581528	8	A	T	0.713	0.075	0.008	5.3E-19	trans
870	FLRT2	O43155	Leucine-rich repeat transmembrane protein FLRT2	rs3748770	82265905	1	A	G	0.267	0.249	0.008	3.6E-220	trans
870	FLRT2	O43155	Leucine-rich repeat transmembrane protein FLRT2	rs1260326	27730940	2	C	T	0.608	0.052	0.007	2.0E-13	trans
870	FLRT2	O43155	Leucine-rich repeat transmembrane protein FLRT2	rs10935473	98416900	3	G	T	0.560	0.056	0.007	3.6E-16	trans
870	FLRT2	O43155	Leucine-rich repeat transmembrane protein FLRT2	rs1363490	166821663	5	A	C	0.716	0.055	0.008	4.0E-13	trans
870	FLRT2	O43155	Leucine-rich repeat transmembrane protein FLRT2	rs4841132	9183596	8	A	G	0.092	0.117	0.012	1.2E-22	trans
870	FLRT2	O43155	Leucine-rich repeat transmembrane protein FLRT2	rs10901252	136128000	9	C	G	0.061	0.103	0.014	5.6E-13	trans
870	FLRT2	O43155	Leucine-rich repeat transmembrane protein FLRT2	rs17646457	86089315	14	G	A	0.849	0.699	0.010	0.0E+00	cis
870	FLRT2	O43155	Leucine-rich repeat transmembrane protein FLRT2	rs77542162	67081278	17	G	A	0.023	0.213	0.023	1.1E-20	trans
870	FLRT2	O43155	Leucine-rich repeat transmembrane protein FLRT2	rs6135225	14678135	20	G	T	0.695	0.195	0.008	3.3E-148	trans
871	FLT1	P17948	Vascular endothelial growth factor receptor 1	rs13088856	159554958	3	G	T	0.298	0.065	0.008	2.7E-16	trans
871	FLT1	P17948	Vascular endothelial growth factor receptor 1	rs13107325	103188709	4	T	C	0.075	0.106	0.014	2.7E-14	trans
871	FLT1	P17948	Vascular endothelial growth factor receptor 1	rs6921438	43925607	6	A	G	0.491	0.345	0.008	0.0E+00	trans
871	FLT1	P17948	Vascular endothelial growth factor receptor 1	rs6993770	106581528	8	T	A	0.286	0.102	0.008	3.6E-36	trans
871	FLT1	P17948	Vascular endothelial growth factor receptor 1	rs34881325	2622134	9	T	C	0.376	0.059	0.008	2.0E-14	trans
871	FLT1	P17948	Vascular endothelial growth factor receptor 1	rs2519093	136141870	9	C	T	0.815	0.183	0.010	1.4E-82	trans
871	FLT1	P17948	Vascular endothelial growth factor receptor 1	rs200489612	7106378	17	A	G	0.005	0.508	0.055	3.7E-20	trans
872	FLT3	P36888	Receptor-type tyrosine-protein kinase FLT3	rs2736100	1286516	5	C	A	0.501	0.053	0.008	4.0E-12	trans
872	FLT3	P36888	Receptor-type tyrosine-protein kinase FLT3	rs1847472	90973159	6	C	A	0.646	0.059	0.008	6.4E-14	trans
872	FLT3	P36888	Receptor-type tyrosine-protein kinase FLT3	rs9554228	28637838	13	A	G	0.373	0.084	0.008	8.4E-27	cis
872	FLT3	P36888	Receptor-type tyrosine-protein kinase FLT3	rs186021206	7069412	17	A	G	0.006	0.639	0.053	1.0E-33	trans
873	FLT3LG	P49771	Fms-related tyrosine kinase 3 ligand	rs17534670	65615428	2	A	G	0.493	0.093	0.007	4.9E-40	trans
873	FLT3LG	P49771	Fms-related tyrosine kinase 3 ligand	rs2001703	128383097	3	A	G	0.381	0.117	0.007	1.5E-59	trans
873	FLT3LG	P49771	Fms-related tyrosine kinase 3 ligand	rs6784404	141073675	3	G	A	0.583	0.058	0.007	3.1E-16	trans
873	FLT3LG	P49771	Fms-related tyrosine kinase 3 ligand	rs218265	55408999	4	T	C	0.847	0.076	0.010	8.4E-15	trans
873	FLT3LG	P49771	Fms-related tyrosine kinase 3 ligand	rs7705526	1285974	5	C	A	0.675	0.123	0.008	3.0E-59	trans
873	FLT3LG	P49771	Fms-related tyrosine kinase 3 ligand	rs56353819	91012867	6	T	C	0.351	0.071	0.007	6.1E-22	trans
873	FLT3LG	P49771	Fms-related tyrosine kinase 3 ligand	rs13219432	109614905	6	C	T	0.457	0.065	0.007	4.5E-20	trans
873	FLT3LG	P49771	Fms-related tyrosine kinase 3 ligand	rs42032	92237426	7	G	A	0.738	0.057	0.008	9.2E-13	trans
873	FLT3LG	P49771	Fms-related tyrosine kinase 3 ligand	rs2256187	77919768	11	G	A	0.846	0.068	0.010	2.5E-12	trans
873	FLT3LG	P49771	Fms-related tyrosine kinase 3 ligand	rs1800056	108138003	11	T	C	0.986	0.245	0.030	2.1E-16	trans
873	FLT3LG	P49771	Fms-related tyrosine kinase 3 ligand	rs4936290	114009255	11	C	A	0.348	0.062	0.007	5.6E-17	trans
873	FLT3LG	P49771	Fms-related tyrosine kinase 3 ligand	rs76428106	28604007	13	C	T	0.013	1.309	0.033	0.0E+00	trans
873	FLT3LG	P49771	Fms-related tyrosine kinase 3 ligand	rs78378222	7571752	17	T	G	0.987	0.220	0.032	5.3E-12	trans
873	FLT3LG	P49771	Fms-related tyrosine kinase 3 ligand	rs80277818	42017901	18	A	G	0.875	0.073	0.011	5.8E-12	trans
873	FLT3LG	P49771	Fms-related tyrosine kinase 3 ligand	rs17758695	60920854	18	T	C	0.029	0.194	0.021	8.8E-21	trans
873	FLT3LG	P49771	Fms-related tyrosine kinase 3 ligand	rs117955557	45755368	19	T	G	0.255	0.057	0.008	1.7E-12	trans
873	FLT3LG	P49771	Fms-related tyrosine kinase 3 ligand	rs62237617	28761148	22	C	T	0.997	0.586	0.075	6.4E-15	trans
874	FLT4	P35916	Vascular endothelial growth factor receptor 3	rs11162623	79559634	1	T	C	0.340	0.066	0.007	7.7E-23	trans
874	FLT4	P35916	Vascular endothelial growth factor receptor 3	rs10935473	98416900	3	G	T	0.560	0.694	0.007	0.0E+00	trans
874	FLT4	P35916	Vascular endothelial growth factor receptor 3	rs34221241	180057293	5	T	C	0.892	0.443	0.010	0.0E+00	cis
874	FLT4	P35916	Vascular endothelial growth factor receptor 3	rs6913664	32295874	6	C	T	0.527	0.063	0.006	1.8E-23	trans
874	FLT4	P35916	Vascular endothelial growth factor receptor 3	rs7019909	33113322	9	T	C	0.103	0.083	0.011	1.1E-14	trans
874	FLT4	P35916	Vascular endothelial growth factor receptor 3	rs10981199	114766367	9	C	T	0.356	0.053	0.007	8.5E-15	trans
874	FLT4	P35916	Vascular endothelial growth factor receptor 3	rs2519093	136141870	9	C	T	0.816	0.380	0.008	0.0E+00	trans
874	FLT4	P35916	Vascular endothelial growth factor receptor 3	rs186021206	7069412	17	A	G	0.006	0.510	0.044	1.1E-31	trans
874	FLT4	P35916	Vascular endothelial growth factor receptor 3	rs1012383	59196065	17	G	T	0.173	0.057	0.008	1.2E-11	trans
874	FLT4	P35916	Vascular endothelial growth factor receptor 3	rs67525224	42627220	21	C	G	0.185	0.080	0.008	9.0E-23	trans
874	FLT4	P35916	Vascular endothelial growth factor receptor 3	rs4611747	29757090	22	T	C	0.288	0.062	0.007	7.2E-19	trans
874	FLT4	P35916	Vascular endothelial growth factor receptor 3	rs132653	36556823	22	T	G	0.818	0.056	0.008	7.6E-12	trans
875	FN1	P02751	Fibronectin	rs1250259	216300482	2	A	T	0.737	0.448	0.008	0.0E+00	cis
875	FN1	P02751	Fibronectin	rs13108218	3443931	4	A	G	0.381	0.051	0.008	1.2E-11	trans
875	FN1	P02751	Fibronectin	rs41341748	16012594	8	A	G	0.011	0.265	0.035	2.9E-14	trans
876	FNDC1	Q4ZHG4	Fibronectin type III domain-containing protein 1	rs73588939	159948120	6	A	G	0.733	0.428	0.008	0.0E+00	cis
876	FNDC1	Q4ZHG4	Fibronectin type III domain-containing protein 1	rs7047089	95117980	9	T	C	0.371	0.076	0.007	6.2E-26	trans
876	FNDC1	Q4ZHG4	Fibronectin type III domain-containing protein 1	rs7178458	101991773	15	C	T	0.716	0.138	0.008	2.1E-70	trans
877	FNTA	P49354	Protein farnesyltransferase/geranylgeranyltransferase type-1 subunit alpha	rs12614	31914179	6	T	C	0.085	0.144	0.014	3.0E-26	trans
878	FOLH1	Q04609	Glutamate carboxypeptidase 2	rs139299944	32602665	6	CT	C	0.344	0.113	0.008	4.3E-44	trans
878	FOLH1	Q04609	Glutamate carboxypeptidase 2	rs61886488	49180460	11	T	G	0.058	0.191	0.017	8.0E-29	cis
878	FOLH1	Q04609	Glutamate carboxypeptidase 2	rs139654989	51574001	11	C	G	0.062	0.166	0.016	4.6E-25	trans
879	FOLR1	P15328	Folate receptor alpha	rs423144	155169355	1	T	G	0.426	0.057	0.007	3.5E-15	trans
879	FOLR1	P15328	Folate receptor alpha	rs61747728	179526214	1	T	C	0.038	0.139	0.019	6.3E-14	trans
879	FOLR1	P15328	Folate receptor alpha	rs13107325	103188709	4	T	C	0.075	0.106	0.014	7.2E-15	trans
879	FOLR1	P15328	Folate receptor alpha	rs10740131	65271488	10	T	A	0.470	0.049	0.007	4.0E-12	trans
879	FOLR1	P15328	Folate receptor alpha	rs186021206	7069412	17	A	G	0.006	0.725	0.049	1.3E-49	trans
879	FOLR1	P15328	Folate receptor alpha	rs78060698	5832773	19	A	G	0.041	0.208	0.018	1.0E-30	trans
879	FOLR1	P15328	Folate receptor alpha	rs681343	49206462	19	T	C	0.509	0.116	0.007	8.9E-59	trans
880	FOLR2	P14207	Folate receptor beta	rs333947	110470764	1	A	G	0.148	0.082	0.010	2.4E-16	trans
880	FOLR2	P14207	Folate receptor beta	rs1260326	27730940	2	C	T	0.608	0.058	0.007	3.7E-15	trans
880	FOLR2	P14207	Folate receptor beta	rs2470853	98704822	3	A	G	0.425	0.068	0.007	6.7E-21	trans
880	FOLR2	P14207	Folate receptor beta	rs509227	10535591	6	C	A	0.523	0.058	0.007	8.2E-16	trans
880	FOLR2	P14207	Folate receptor beta	rs80177780	32490421	6	A	C	0.752	0.112	0.009	3.2E-34	trans
880	FOLR2	P14207	Folate receptor beta	rs17138358	17920253	7	G	C	0.606	0.066	0.007	2.7E-19	trans
880	FOLR2	P14207	Folate receptor beta	rs7865362	33117965	9	T	C	0.363	0.084	0.008	7.9E-29	trans
880	FOLR2	P14207	Folate receptor beta	rs2519093	136141870	9	T	C	0.185	0.065	0.009	2.4E-12	trans
880	FOLR2	P14207	Folate receptor beta	rs603424	102075479	10	G	A	0.834	0.107	0.010	4.0E-29	trans
880	FOLR2	P14207	Folate receptor beta	rs950803	60152563	11	A	T	0.629	0.106	0.007	3.4E-46	trans
880	FOLR2	P14207	Folate receptor beta	rs146893981	71917353	11	T	C	0.029	0.321	0.021	1.3E-51	cis
880	FOLR2	P14207	Folate receptor beta	rs4760	44153100	19	G	A	0.156	0.144	0.010	2.3E-48	trans
880	FOLR2	P14207	Folate receptor beta	rs12975366	54759361	19	T	C	0.604	0.143	0.007	1.6E-84	trans
881	FOLR3	P41439	Folate receptor gamma	rs333947	110470764	1	A	G	0.148	0.055	0.008	1.4E-11	trans
881	FOLR3	P41439	Folate receptor gamma	rs62235167	31052683	3	G	C	0.306	0.044	0.006	3.9E-12	trans
881	FOLR3	P41439	Folate receptor gamma	rs2470872	98688564	3	A	T	0.426	0.053	0.006	8.2E-20	trans
881	FOLR3	P41439	Folate receptor gamma	rs10242866	17920613	7	C	T	0.607	0.050	0.006	3.3E-17	trans
881	FOLR3	P41439	Folate receptor gamma	rs2031902	33117524	9	C	T	0.413	0.060	0.006	1.8E-24	trans
881	FOLR3	P41439	Folate receptor gamma	rs603424	102075479	10	G	A	0.834	0.071	0.008	5.9E-20	trans
881	FOLR3	P41439	Folate receptor gamma	rs950802	60152584	11	A	G	0.306	0.077	0.008	1.5E-21	trans
881	FOLR3	P41439	Folate receptor gamma	rs4760	44153100	19	G	A	0.156	0.080	0.008	1.1E-23	trans
881	FOLR3	P41439	Folate receptor gamma	rs12975366	54759361	19	T	C	0.605	0.094	0.006	7.6E-57	trans
882	FOXJ3	Q9UPW0	Forkhead box protein J3	rs1139978	42657196	1	T	G	0.861	0.103	0.011	1.4E-20	cis
882	FOXJ3	Q9UPW0	Forkhead box protein J3	rs1354034	56849749	3	C	T	0.604	0.060	0.008	6.5E-15	trans
883	FOXO1	Q12778	Forkhead box protein O1	rs342298	106373646	7	C	T	0.545	0.084	0.008	4.3E-29	trans
883	FOXO1	Q12778	Forkhead box protein O1	rs10820606	99192919	9	A	C	0.771	0.126	0.009	4.2E-44	trans
883	FOXO1	Q12778	Forkhead box protein O1	rs17061453	41184358	13	T	A	0.043	0.280	0.018	4.5E-52	cis
884	FOXO3	O43524	Forkhead box protein O3	rs34136174	32583682	6	C	A	0.425	0.055	0.008	1.7E-12	trans
885	FRMD7	Q6ZUT3	FERM domain-containing protein 7	rs147252207	43233182	22	T	C	0.997	0.931	0.080	2.3E-31	trans
886	FRZB	Q92765	Secreted frizzled-related protein 3	rs13009	183699364	2	C	T	0.918	0.817	0.014	0.0E+00	cis
886	FRZB	Q92765	Secreted frizzled-related protein 3	rs2856694	32652281	6	T	C	0.814	0.153	0.009	5.8E-63	trans
886	FRZB	Q92765	Secreted frizzled-related protein 3	rs6018652	46340974	20	G	A	0.209	0.058	0.008	1.6E-12	trans
887	FSHB	P01225	Follitropin subunit beta	rs10429910	197141253	1	C	A	0.141	0.090	0.007	4.2E-34	trans
887	FSHB	P01225	Follitropin subunit beta	rs9362387	87793401	6	C	T	0.739	0.049	0.006	4.8E-17	trans
887	FSHB	P01225	Follitropin subunit beta	rs11031006	30226528	11	G	A	0.857	0.147	0.007	5.9E-91	cis
887	FSHB	P01225	Follitropin subunit beta	rs2159436	30388987	X	A	G	0.745	0.039	0.005	4.5E-16	trans
888	FST	P19883	Follistatin	rs1260326	27730940	2	T	C	0.391	0.110	0.008	7.1E-48	trans
888	FST	P19883	Follistatin	rs31226	53327571	5	T	C	0.397	0.142	0.008	1.9E-77	cis
888	FST	P19883	Follistatin	rs139974673	44027885	15	C	T	0.024	0.178	0.024	7.6E-14	trans
889	FSTL1	Q12841	Follistatin-related protein 1	rs1260326	27730940	2	C	T	0.608	0.066	0.008	2.2E-17	trans
889	FSTL1	Q12841	Follistatin-related protein 1	rs1147707	120169248	3	C	T	0.605	0.149	0.008	7.5E-80	cis
889	FSTL1	Q12841	Follistatin-related protein 1	rs7042335	95122547	9	C	T	0.671	0.059	0.008	2.7E-13	trans
889	FSTL1	Q12841	Follistatin-related protein 1	rs2519093	136141870	9	C	T	0.816	0.076	0.010	1.2E-14	trans
889	FSTL1	Q12841	Follistatin-related protein 1	rs11075921	72132129	16	T	C	0.160	0.082	0.010	2.3E-15	trans
889	FSTL1	Q12841	Follistatin-related protein 1	rs200489612	7106378	17	A	G	0.005	0.476	0.057	5.2E-17	trans
889	FSTL1	Q12841	Follistatin-related protein 1	rs10418205	6100433	19	A	G	0.627	0.057	0.008	7.1E-13	trans
890	FSTL3	O95633	Follistatin-related protein 3	rs77924615	20392332	16	G	A	0.803	0.077	0.009	4.0E-17	trans
890	FSTL3	O95633	Follistatin-related protein 3	rs916904	663929	19	G	A	0.634	0.087	0.008	2.2E-30	cis
891	FTCD	O95954	Formimidoyltransferase-cyclodeaminase	rs7041363	117146043	9	C	G	0.510	0.065	0.007	2.8E-18	trans
891	FTCD	O95954	Formimidoyltransferase-cyclodeaminase	rs10883451	101924418	10	T	C	0.499	0.052	0.007	2.1E-12	trans
891	FTCD	O95954	Formimidoyltransferase-cyclodeaminase	rs58542926	19379549	19	T	C	0.075	0.111	0.014	1.6E-15	trans
891	FTCD	O95954	Formimidoyltransferase-cyclodeaminase	rs3747207	44324855	22	A	G	0.216	0.111	0.009	1.3E-35	trans
892	FUCA1	P04066	Tissue alpha-L-fucosidase	rs13551	24180962	1	T	C	0.661	0.382	0.005	0.0E+00	cis
892	FUCA1	P04066	Tissue alpha-L-fucosidase	rs11155297	143825104	6	G	T	0.736	1.300	0.008	0.0E+00	trans
892	FUCA1	P04066	Tissue alpha-L-fucosidase	rs148815576	93685488	14	G	T	0.002	0.391	0.050	5.2E-15	trans
892	FUCA1	P04066	Tissue alpha-L-fucosidase	rs339969	60883281	15	A	C	0.617	0.028	0.004	9.7E-12	trans
893	FUOM	A2VDF0	Fucose mutarotase	rs7041363	117146043	9	C	G	0.510	0.068	0.007	4.1E-20	trans
893	FUOM	A2VDF0	Fucose mutarotase	rs10883451	101924418	10	T	C	0.498	0.060	0.007	2.1E-16	trans
893	FUOM	A2VDF0	Fucose mutarotase	rs11816588	135174600	10	C	T	0.033	0.503	0.021	2.1E-130	cis
893	FUOM	A2VDF0	Fucose mutarotase	rs4753124	93823235	11	C	G	0.893	0.082	0.012	3.7E-12	trans

893	FUOM	A2VDF0	Fucose mutarotase	rs28929474	94844947	14	T	C	0.021	0.190	0.026	1.1E-13	trans
893	FUOM	A2VDF0	Fucose mutarotase	rs200210321	19393890	19	AG	A	0.074	0.109	0.014	5.1E-15	trans
893	FUOM	A2VDF0	Fucose mutarotase	rs3747207	44324855	22	A	G	0.215	0.111	0.009	4.5E-36	trans
894	FURIN	P09958	Furin	rs1260326	27730940	2	T	C	0.391	0.102	0.008	1.5E-41	trans
894	FURIN	P09958	Furin	rs34430945	73043665	7	T	C	0.871	0.080	0.011	3.1E-13	trans
894	FURIN	P09958	Furin	rs2071410	91420940	15	G	C	0.326	0.194	0.008	3.5E-135	cis
895	FUS	P35637	RNA-binding protein FUS	rs10418046	54327869	19	G	T	0.212	0.103	0.009	2.0E-28	trans
896	FUT3_FUT5	P21217,Q11128	3-galactosyl-N-acetylglucosaminide 4-alpha-L-fucosyltransferase FUT3_4-galactosyl-N-acetylglucosaminide 3-alpha-L-fucosyltransferase FUT5	rs1800759	100065509	4	T	G	0.387	0.061	0.006	8.0E-21	trans
896	FUT3_FUT5	P21217,Q11128	3-galactosyl-N-acetylglucosaminide 4-alpha-L-fucosyltransferase FUT3_4-galactosyl-N-acetylglucosaminide 3-alpha-L-fucosyltransferase FUT5	rs2106854	131769174	5	C	T	0.815	0.056	0.008	9.2E-12	trans
896	FUT3_FUT5	P21217,Q11128	3-galactosyl-N-acetylglucosaminide 4-alpha-L-fucosyltransferase FUT3_4-galactosyl-N-acetylglucosaminide 3-alpha-L-fucosyltransferase FUT5	rs34434863	32559673	6	G	T	0.309	0.053	0.007	7.8E-14	trans
896	FUT3_FUT5	P21217,Q11128	3-galactosyl-N-acetylglucosaminide 4-alpha-L-fucosyltransferase FUT3_4-galactosyl-N-acetylglucosaminide 3-alpha-L-fucosyltransferase FUT5	rs28601761	126500031	8	G	C	0.419	0.071	0.006	6.1E-28	trans
896	FUT3_FUT5	P21217,Q11128	3-galactosyl-N-acetylglucosaminide 4-alpha-L-fucosyltransferase FUT3_4-galactosyl-N-acetylglucosaminide 3-alpha-L-fucosyltransferase FUT5	rs62542743	33385241	9	A	C	0.053	0.101	0.014	2.5E-12	trans
896	FUT3_FUT5	P21217,Q11128	3-galactosyl-N-acetylglucosaminide 4-alpha-L-fucosyltransferase FUT3_4-galactosyl-N-acetylglucosaminide 3-alpha-L-fucosyltransferase FUT5	rs140107293	5267191	10	G	A	0.156	0.061	0.009	2.4E-12	trans
896	FUT3_FUT5	P21217,Q11128	3-galactosyl-N-acetylglucosaminide 4-alpha-L-fucosyltransferase FUT3_4-galactosyl-N-acetylglucosaminide 3-alpha-L-fucosyltransferase FUT5	rs56278466	17875857	10	G	T	0.661	0.074	0.007	2.8E-28	trans
896	FUT3_FUT5	P21217,Q11128	3-galactosyl-N-acetylglucosaminide 4-alpha-L-fucosyltransferase FUT3_4-galactosyl-N-acetylglucosaminide 3-alpha-L-fucosyltransferase FUT5	rs178765	23706738	14	A	G	0.283	0.068	0.007	3.1E-22	trans
896	FUT3_FUT5	P21217,Q11128	3-galactosyl-N-acetylglucosaminide 4-alpha-L-fucosyltransferase FUT3_4-galactosyl-N-acetylglucosaminide 3-alpha-L-fucosyltransferase FUT5	rs3760775	5841356	19	G	T	0.940	1.068	0.015	0.0E+00	cis
896	FUT3_FUT5	P21217,Q11128	3-galactosyl-N-acetylglucosaminide 4-alpha-L-fucosyltransferase FUT3_4-galactosyl-N-acetylglucosaminide 3-alpha-L-fucosyltransferase FUT5	rs58542926	19379549	19	T	C	0.075	0.114	0.014	5.9E-17	trans
896	FUT3_FUT5	P21217,Q11128	3-galactosyl-N-acetylglucosaminide 4-alpha-L-fucosyltransferase FUT3_4-galactosyl-N-acetylglucosaminide 3-alpha-L-fucosyltransferase FUT5	rs492602	49206417	19	A	G	0.490	0.081	0.007	7.8E-29	trans
896	FUT3_FUT5	P21217,Q11128	3-galactosyl-N-acetylglucosaminide 4-alpha-L-fucosyltransferase FUT3_4-galactosyl-N-acetylglucosaminide 3-alpha-L-fucosyltransferase FUT5	rs6073431	43040569	20	T	C	0.532	0.047	0.006	3.8E-13	trans
896	FUT3_FUT5	P21217,Q11128	3-galactosyl-N-acetylglucosaminide 4-alpha-L-fucosyltransferase FUT3_4-galactosyl-N-acetylglucosaminide 3-alpha-L-fucosyltransferase FUT5	rs56692788	109933231	X	A	C	0.376	0.052	0.006	1.2E-20	trans
897	FUT8	Q9BYC5	Alpha-(1,6)-fucosyltransferase	rs7618405	18250509	3	C	A	0.795	0.055	0.008	7.5E-12	trans
897	FUT8	Q9BYC5	Alpha-(1,6)-fucosyltransferase	rs1354034	56849749	3	T	C	0.396	0.065	0.007	1.6E-22	trans
897	FUT8	Q9BYC5	Alpha-(1,6)-fucosyltransferase	rs3792386	122853680	3	G	A	0.413	0.047	0.007	1.8E-12	trans
897	FUT8	Q9BYC5	Alpha-(1,6)-fucosyltransferase	rs10016018	102794621	4	A	T	0.603	0.053	0.007	1.9E-15	trans
897	FUT8	Q9BYC5	Alpha-(1,6)-fucosyltransferase	rs10075801	131677642	5	A	G	0.511	0.049	0.007	1.7E-13	trans
897	FUT8	Q9BYC5	Alpha-(1,6)-fucosyltransferase	rs6961069	80218961	7	T	C	0.402	0.052	0.007	5.2E-15	trans
897	FUT8	Q9BYC5	Alpha-(1,6)-fucosyltransferase	rs6993770	106581528	8	A	T	0.713	0.147	0.007	2.3E-93	trans
897	FUT8	Q9BYC5	Alpha-(1,6)-fucosyltransferase	rs11591571	104342804	10	A	G	0.341	0.055	0.007	8.9E-16	trans
897	FUT8	Q9BYC5	Alpha-(1,6)-fucosyltransferase	rs2127870	65796846	14	G	C	0.205	0.915	0.009	0.0E+00	cis
897	FUT8	Q9BYC5	Alpha-(1,6)-fucosyltransferase	rs12445050	81870969	16	T	C	0.138	0.089	0.009	4.1E-21	trans
897	FUT8	Q9BYC5	Alpha-(1,6)-fucosyltransferase	rs892090	55539072	19	G	T	0.834	0.125	0.009	2.2E-46	trans
897	FUT8	Q9BYC5	Alpha-(1,6)-fucosyltransferase	rs6112319	19317065	20	A	G	0.353	0.054	0.007	1.6E-15	trans
898	FXN	Q16595	Frataxin, mitochondrial	rs1354034	56849749	3	C	T	0.604	0.055	0.008	5.4E-13	trans
898	FXN	Q16595	Frataxin, mitochondrial	rs6993770	106581528	8	A	T	0.714	0.064	0.008	9.7E-15	trans
898	FXN	Q16595	Frataxin, mitochondrial	rs10733789	64948684	10	C	T	0.313	0.060	0.008	3.0E-13	trans
899	FXYD5	Q96DB9	FXYD domain-containing ion transport regulator 5	rs1354034	56849749	3	C	T	0.604	0.078	0.008	9.8E-25	trans
899	FXYD5	Q96DB9	FXYD domain-containing ion transport regulator 5	rs7896518	65104500	10	G	A	0.424	0.055	0.008	9.1E-13	trans
899	FXYD5	Q96DB9	FXYD domain-containing ion transport regulator 5	rs756845	35663250	19	A	G	0.220	0.380	0.009	0.0E+00	cis
900	FYB1	O15117	FYN-binding protein 1	rs1354034	56849749	3	C	T	0.604	0.074	0.008	5.4E-22	trans
900	FYB1	O15117	FYN-binding protein 1	rs8100773	55533479	19	C	T	0.801	0.069	0.010	7.1E-13	trans
901	GABARAP	O95166	Gamma-aminobutyric acid receptor-associated protein	rs11967437	32681927	6	T	C	0.007	0.793	0.046	2.7E-67	trans
902	GADD45GIP1	Q8TAE8	Growth arrest and DNA damage-inducible proteins-interacting protein 1	rs1354034	56849749	3	C	T	0.605	0.063	0.008	3.8E-16	trans
902	GADD45GIP1	Q8TAE8	Growth arrest and DNA damage-inducible proteins-interacting protein 1	rs10058074	131686146	5	G	A	0.525	0.056	0.008	2.6E-13	trans
902	GADD45GIP1	Q8TAE8	Growth arrest and DNA damage-inducible proteins-interacting protein 1	rs342299	106373718	7	C	T	0.545	0.055	0.008	8.2E-13	trans
902	GADD45GIP1	Q8TAE8	Growth arrest and DNA damage-inducible proteins-interacting protein 1	rs6993770	106581528	8	A	T	0.713	0.068	0.008	8.4E-16	trans
902	GADD45GIP1	Q8TAE8	Growth arrest and DNA damage-inducible proteins-interacting protein 1	rs11085147	5711930	19	T	C	0.099	0.150	0.013	8.8E-30	trans
903	GAL	P22466	Galanin peptides	rs320366	118128145	1	C	T	0.251	0.059	0.008	5.7E-13	trans
903	GAL	P22466	Galanin peptides	rs760077	155178782	1	A	T	0.395	0.080	0.007	6.0E-29	trans
903	GAL	P22466	Galanin peptides	rs28631481	218852147	1	T	C	0.383	0.053	0.007	1.7E-13	trans
903	GAL	P22466	Galanin peptides	rs11680123	60514748	2	G	A	0.593	0.196	0.007	2.3E-165	trans
903	GAL	P22466	Galanin peptides	rs121908120	219755011	2	T	A	0.972	0.243	0.022	4.5E-29	trans
903	GAL	P22466	Galanin peptides	rs1530018	224925121	2	A	G	0.767	0.078	0.008	7.2E-21	trans
903	GAL	P22466	Galanin peptides	rs3773364	12189968	3	G	A	0.161	0.196	0.010	2.7E-94	trans
903	GAL	P22466	Galanin peptides	rs34826710	1666657	6	T	C	0.105	0.146	0.011	1.6E-37	trans
903	GAL	P22466	Galanin peptides	rs565427	65532825	11	A	G	0.358	0.063	0.007	3.8E-18	trans
903	GAL	P22466	Galanin peptides	rs76634175	68441727	11	A	T	0.078	0.152	0.013	1.9E-31	cis
903	GAL	P22466	Galanin peptides	rs9601479	81442736	13	A	G	0.509	0.051	0.007	3.4E-13	trans
903	GAL	P22466	Galanin peptides	rs36034702	90734627	15	C	T	0.831	0.088	0.009	8.5E-21	trans
903	GAL	P22466	Galanin peptides	rs720130	11132633	16	G	T	0.592	0.055	0.007	4.7E-15	trans
903	GAL	P22466	Galanin peptides	rs56228609	56987765	16	T	C	0.316	0.075	0.007	1.8E-23	trans
903	GAL	P22466	Galanin peptides	rs77542162	67081278	17	G	A	0.023	0.738	0.024	2.4E-215	trans
903	GAL	P22466	Galanin peptides	rs55949690	70054424	17	A	G	0.523	0.051	0.007	6.6E-13	trans
903	GAL	P22466	Galanin peptides	rs6028484	38156052	20	C	T	0.544	0.055	0.007	2.1E-15	trans
903	GAL	P22466	Galanin peptides	rs6024978	55165067	20	A	C	0.774	0.066	0.008	3.9E-15	trans
904	GALNT10	Q86SR1	Polypeptide N-acetylgalactosaminyltransferase 10	rs4672497	62523565	2	G	C	0.222	0.070	0.008	2.1E-16	trans
904	GALNT10	Q86SR1	Polypeptide N-acetylgalactosaminyltransferase 10	rs890793	153573938	5	T	C	0.382	0.384	0.008	0.0E+00	cis
904	GALNT10	Q86SR1	Polypeptide N-acetylgalactosaminyltransferase 10	rs635634	136155000	9	T	C	0.184	0.260	0.009	4.5E-176	trans
904	GALNT10	Q86SR1	Polypeptide N-acetylgalactosaminyltransferase 10	rs56278466	17875857	10	G	T	0.661	0.057	0.007	2.4E-14	trans
904	GALNT10	Q86SR1	Polypeptide N-acetylgalactosaminyltransferase 10	rs145078947	93652974	14	T	G	0.003	0.570	0.069	1.7E-16	trans
904	GALNT10	Q86SR1	Polypeptide N-acetylgalactosaminyltransferase 10	rs704	26694861	17	A	G	0.473	0.086	0.007	1.0E-34	trans
904	GALNT10	Q86SR1	Polypeptide N-acetylgalactosaminyltransferase 10	rs601338	49206674	19	A	G	0.509	0.052	0.007	2.1E-13	trans
905	GALNT2	Q10471	Polypeptide N-acetylgalactosaminyltransferase 2	rs4846914	230295691	1	A	G	0.606	0.293	0.007	0.0E+00	cis
905	GALNT2	Q10471	Polypeptide N-acetylgalactosaminyltransferase 2	rs9987289	9183358	8	A	G	0.092	0.085	0.012	1.6E-12	trans
905	GALNT2	Q10471	Polypeptide N-acetylgalactosaminyltransferase 2	rs704	26694861	17	A	G	0.473	0.083	0.007	1.0E-32	trans
905	GALNT2	Q10471	Polypeptide N-acetylgalactosaminyltransferase 2	rs12975366	54759361	19	T	C	0.605	0.145	0.007	3.2E-91	trans
906	GALNT3	Q14435	Polypeptide N-acetylgalactosaminyltransferase 3	rs2116546	166723533	2	T	C	0.702	0.389	0.008	0.0E+00	cis
906	GALNT3	Q14435	Polypeptide N-acetylgalactosaminyltransferase 3	rs9310400	12289449	3	T	C	0.328	0.055	0.008	8.2E-13	trans
906	GALNT3	Q14435	Polypeptide N-acetylgalactosaminyltransferase 3	rs13107325	103188709	4	C	T	0.925	0.121	0.014	1.3E-18	trans
906	GALNT3	Q14435	Polypeptide N-acetylgalactosaminyltransferase 3	rs9391787	32448616	6	C	T	0.593	0.062	0.008	9.9E-15	trans
906	GALNT3	Q14435	Polypeptide N-acetylgalactosaminyltransferase 3	rs7791957	7270034	7	C	T	0.431	0.096	0.007	3.8E-40	trans
906	GALNT3	Q14435	Polypeptide N-acetylgalactosaminyltransferase 3	rs145078947	93652974	14	T	G	0.003	0.509	0.071	6.9E-13	trans
906	GALNT3	Q14435	Polypeptide N-acetylgalactosaminyltransferase 3	rs146261845	7012254	17	T	C	0.007	0.334	0.047	8.1E-13	trans
906	GALNT3	Q14435	Polypeptide N-acetylgalactosaminyltransferase 3	rs492602	49206417	19	A	G	0.491	0.183	0.007	9.1E-140	trans
906	GALNT3	Q14435	Polypeptide N-acetylgalactosaminyltransferase 3	rs149340486	119760720	X	C	T	0.990	0.315	0.029	6.1E-27	trans
907	GALNT5	Q7Z7M9	Polypeptide N-acetylgalactosaminyltransferase 5	rs1427407	60718043	2	T	G	0.152	0.120	0.010	5.1E-31	trans
907	GALNT5	Q7Z7M9	Polypeptide N-acetylgalactosaminyltransferase 5	rs714649	158141092	2	G	T	0.982	0.941	0.028	2.7E-250	cis
907	GALNT5	Q7Z7M9	Polypeptide N-acetylgalactosaminyltransferase 5	rs3864106	186656602	3	G	C	0.416	0.117	0.007	2.6E-56	trans
907	GALNT5	Q7Z7M9	Polypeptide N-acetylgalactosaminyltransferase 5	rs6592965	50427982	7	G	A	0.546	0.089	0.007	1.2E-34	trans
907	GALNT5	Q7Z7M9	Polypeptide N-acetylgalactosaminyltransferase 5	rs507666	136149399	9	G	A	0.815	0.155	0.009	8.5E-61	trans
907	GALNT5	Q7Z7M9	Polypeptide N-acetylgalactosaminyltransferase 5	rs74035509	88567333	16	C	T	0.922	0.142	0.014	1.6E-25	trans
907	GALNT5	Q7Z7M9	Polypeptide N-acetylgalactosaminyltransferase 5	rs186021206	7069412	17	A	G	0.006	0.582	0.050	2.0E-31	trans
908	GALNT7	Q86SF2	N-acetylgalactosaminyltransferase 7	rs13107325	103188709	4	C	T	0.925	0.140	0.014	1.1E-22	trans
908	GALNT7	Q86SF2	N-acetylgalactosaminyltransferase 7	rs144873913	174235304	4	C	A	0.998	0.855	0.077	7.1E-29	cis
908	GALNT7	Q86SF2	N-acetylgalactosaminyltransferase 7	rs11751024	32586236	6	A	C	0.398	0.072	0.008	8.3E-21	trans
908	GALNT7	Q86SF2	N-acetylgalactosaminyltransferase 7	rs2691582	17829247	7	C	T	0.494	0.057	0.008	2.1E-14	trans
908	GALNT7	Q86SF2	N-acetylgalactosaminyltransferase 7	rs117905808	100509130	7	A	G	0.020	0.727	0.027	5.5E-157	trans
908	GALNT7	Q86SF2	N-acetylgalactosaminyltransferase 7	rs9534300	46629225	13	A	T	0.292	0.120	0.008	7.0E-49	trans
908	GALNT7	Q86SF2	N-acetylgalactosaminyltransferase 7	rs12975366	54759361	19	T	C	0.605	0.217	0.008	5.0E-175	trans
909	GAPDH	P04406	Glyceraldehyde-3-phosphate dehydrogenase	rs1354034	56849749	3	C	T	0.604	0.061	0.008	5.5E-15	trans
910	GART	P22102	Trifunctional purine biosynthetic protein adenosine-3	rs35923643	123355391	11	A	G	0.804	0.071	0.010	1.7E-13	trans
911	GAS2	O43903	Growth arrest-specific protein 2	rs2279240	22696890	11	C	T	0.707	0.114	0.008	6.6E-42	cis
912	GAS6	Q14393	Growth arrest-specific protein 6	rs56073752	9617361	2	A	G	0.513	0.049	0.007	6.0E-12	trans
912	GAS6	Q14393	Growth arrest-specific protein 6	rs1260326	27730940	2	C	T	0.608	0.057	0.007	4.2E-15	trans
912	GAS6	Q14393	Growth arrest-specific protein 6	rs62165726	134966562	2	C	A	0.963	0.129	0.019	8.5E-12	trans
912	GAS6	Q14393	Growth arrest-specific protein 6	rs1983009	98558048	3	T	C	0.101	0.099	0.012	8.5E-16	trans
912	GAS6	Q14393	Growth arrest-specific protein 6	rs13108218	3443931	4	G	A	0.619	0.050	0.007	1.1E-11	trans
912	GAS6	Q14393	Growth arrest-specific protein 6	rs6840282	99913471	4	C	T	0.386	0.051	0.007	4.6E-12	trans
912	GAS6	Q14393	Growth arrest-specific protein 6	rs112840324	32478658	6	G	A	0.709	0.076	0.009	1.1E-17	trans
912	GAS6	Q14393	Growth arrest-specific protein 6	rs17138358	17920253	7	G	C	0.606	0.054	0.007	1.3E-13	trans
912	GAS6	Q14393	Growth arrest-specific protein 6	rs17145750	73026378	7	T	C	0.159	0.074	0.010	3.0E-14	trans
912	GAS6	Q14393	Growth arrest-specific protein 6	rs12667978	128777461	7	T	C	0.571	0.060	0.007	7.1E-17	trans
912	GAS6	Q14393	Growth arrest-specific protein 6	rs4841133	9183664	8	A	G	0.092	0.131	0.012	1.4E-26	trans
912	GAS6	Q14393	Growth arrest-specific protein 6	rs4876611	116671848	8	G	A	0.718	0.074	0.008	1.5E-20	trans
912	GAS6	Q14393	Growth arrest-specific protein 6	rs11230313	60204041	11	A	C	0.672	0.056	0.008	4.7E-13	trans
912	GAS6	Q14393	Growth arrest-specific protein 6	rs6602909	114551993	13	T	C	0.676	0.338	0.008	0.0E+00	cis
912	GAS6	Q14393	Growth arrest-specific protein 6	rs150844304	43726625	15	A	C	0.976	0.157	0.023	9.7E-12	trans
912	GAS6	Q14393	Growth arrest-specific protein 6	rs186021206	7069412	17	A	G	0.006	1.078	0.049	5.1E-107	trans
912	GAS6	Q14393	Growth arrest-specific protein 6	rs1688030	35556744	19	T	C	0.066	0.106	0.014	1.8E-13	trans
912	GAS6	Q14393	Growth arrest-specific protein 6	rs74816457	41739534	19	G	C	0.597	0.221	0.007	1.1E-200	trans
912	GAS6	Q14393	Growth arrest-specific protein 6	rs3747207	44324855	22	A	G	0.215	0.080	0.009	1.3E-20	trans
913	GASK1A	Q9UFP1	Golgi-associated kinase 1A	rs493412	43126405	3	T	C	0.629	0.730	0.008	0.0E+00	cis
914	GAST	P01350	Gastrin	rs72704793	548008	5	A	G	0.330	0.071	0.008	1.1E-19	trans
914	GAST	P01350	Gastrin	rs56092644	95774809	5	T	G	0.245	0.130	0.009	2.3E-52	trans
914	GAST	P01350	Gastrin	rs2395184	32432646	6	G	A	0.833	0.125	0.010	2.6E-37	trans
914	GAST	P01350	Gastrin	rs2472632	27395875	11	A	C	0.360	0.073	0.008	1.5E-21	trans
915	GATD3	P0DPI2	Glutamine amidotransferase-like class 1 domain-containing protein 3B, mitochondrial	rs11242109	131677047	5	G	T	0.519	0.052	0.008	1.2E-11	trans
915	GATD3	P0DPI2	Glutamine amidotransferase-like class 1 domain-containing protein 3B, mitochondrial	rs342298	106373646	7	C	T	0.545	0.059	0.008	2.2E-14	trans
915	GATD3	P0DPI2	Glutamine amidotransferase-like class 1 domain-containing protein 3B, mitochondrial	rs6993770	106581528	8	A	T	0.713	0.065	0.008	1.0E-14	trans
915	GATD3	P0DPI2	Glutamine amidotransferase-like class 1 domain-containing protein 3B, mitochondrial	rs2187313	45544594	21	T	C	0.182	0.124	0.010	3.7E-36	cis
916	GBP1	P32455	Guanylate-binding protein 1	rs148526074	89523756	1	G	T	0.972	1.314	0.023	0.0E+00	cis
917	GBP2	P32456	Guanylate-binding protein 2	rs7531439	89554224	1	C	A	0.790	0.124	0.009	1.7E-39	cis
918	GC	P02774	Vitamin D-binding protein	rs1260326	27730940	2	T	C	0.392	0.033	0.005	9.2E-12	trans
918	GC	P02774	Vitamin D-binding protein	rs1352846	72617775	4	G	A	0.292	1.202	0.008	0.0E+00	cis
918	GC	P02774	Vitamin D-binding protein	rs28601761	126500031	8	C	G	0.581	0.042	0.005	1.4E-17	trans
918	GC	P02774	Vitamin D-binding protein	rs7080386	65048306	10	C	A	0.588	0.033	0.005	5.2E-12	trans
919	GCC1	Q96CN9	GRIP and coiled-coil domain-containing protein 1	rs1354034	56849749	3	C	T	0.604	0.071	0.008	7.0E-21	trans
919	GCC1	Q96CN9	GRIP and coiled-coil domain-containing protein 1	rs342298	106373646	7	C	T	0.545	0.057	0.007	2.1E-14	trans
919	GCC1	Q96CN9	GRIP and coiled-coil domain-containing protein 1	rs6993770	106581528	8	A	T	0.713	0.064	0.008	9.3E-15	trans
919	GCC1	Q96CN9	GRIP and coiled-coil domain-containing protein 1	rs11502185	180258	11	C	T	0.260	0.069	0.010	4.3E-13	trans
920	GCG	P01275	Pro-glucagon	rs17145750	73026378	7	C	T	0.840	0.084	0.010	3.7E-16	trans
920	GCG	P01275	Pro-glucagon	rs39281	89778489	7	G	A	0.527	0.055	0.008	3.9E-13	trans
920	GCG	P01275	Pro-glucagon	rs964184	116648917	11	G	C	0.133	0.082	0.011	2.8E-13	trans
920	GCG	P01275	Pro-glucagon	rs72654473	45414399	19	A	C	0.109	0.089	0.012	3.2E-13	trans

921	GCHFR	P30047	GTP cyclohydrolase 1 feedback regulatory protein	rs1427407	60718043	2	T	G	0.152	0.112	0.010	4.1E-28	trans
921	GCHFR	P30047	GTP cyclohydrolase 1 feedback regulatory protein	rs9399137	135419018	6	C	T	0.258	0.071	0.008	7.2E-17	trans
921	GCHFR	P30047	GTP cyclohydrolase 1 feedback regulatory protein	rs2289218	41065726	15	A	T	0.458	0.111	0.007	4.6E-51	cis
922	GCLM	P48507	Glutamate--cysteine ligase regulatory subunit	rs12563495	94384624	1	G	A	0.491	0.060	0.008	3.8E-15	cis
923	GCNT1	Q02742	Beta-1,3-galactosyl-O-glycosyl-glycoprotein beta-1,6-N-acetylglucosaminyltransferase	rs2009581	111807677	2	G	A	0.727	0.061	0.008	4.2E-15	trans
923	GCNT1	Q02742	Beta-1,3-galactosyl-O-glycosyl-glycoprotein beta-1,6-N-acetylglucosaminyltransferase	rs35829610	131823862	5	C	CTG	0.662	0.065	0.007	6.2E-19	trans
923	GCNT1	Q02742	Beta-1,3-galactosyl-O-glycosyl-glycoprotein beta-1,6-N-acetylglucosaminyltransferase	rs2282683	79117751	9	A	G	0.790	0.496	0.009	0.0E+00	cis
923	GCNT1	Q02742	Beta-1,3-galactosyl-O-glycosyl-glycoprotein beta-1,6-N-acetylglucosaminyltransferase	rs117163080	17845995	10	C	T	0.138	0.076	0.010	2.5E-14	trans
923	GCNT1	Q02742	Beta-1,3-galactosyl-O-glycosyl-glycoprotein beta-1,6-N-acetylglucosaminyltransferase	rs492602	49206417	19	A	G	0.491	0.072	0.007	1.0E-24	trans
924	GDF15	Q99988	Growth/differentiation factor 15	rs1054221	18499858	19	C	T	0.146	0.392	0.009	0.0E+00	cis
925	GDF2	Q9UK05	Growth/differentiation factor 2	rs34008398	69093413	2	G	A	0.987	0.225	0.031	2.8E-13	trans
925	GDF2	Q9UK05	Growth/differentiation factor 2	rs68066031	224880498	2	C	T	0.230	0.167	0.008	2.4E-90	trans
925	GDF2	Q9UK05	Growth/differentiation factor 2	rs1912826	187149540	4	A	G	0.513	0.092	0.007	1.3E-40	trans
925	GDF2	Q9UK05	Growth/differentiation factor 2	rs78408340	102338739	5	C	G	0.990	0.309	0.035	1.4E-18	trans
925	GDF2	Q9UK05	Growth/differentiation factor 2	rs10820966	95055429	9	A	T	0.668	0.181	0.007	2.2E-131	trans
925	GDF2	Q9UK05	Growth/differentiation factor 2	rs9414801	65149089	10	G	A	0.466	0.062	0.007	3.6E-19	trans
925	GDF2	Q9UK05	Growth/differentiation factor 2	rs67852477	70856963	10	G	C	0.010	0.328	0.034	1.3E-21	trans
925	GDF2	Q9UK05	Growth/differentiation factor 2	rs1956713	94932919	14	G	A	0.148	0.152	0.010	3.2E-54	trans
925	GDF2	Q9UK05	Growth/differentiation factor 2	rs4702	91426560	15	A	G	0.558	0.056	0.007	1.1E-15	trans
925	GDF2	Q9UK05	Growth/differentiation factor 2	rs12441560	101985923	15	A	G	0.275	0.062	0.008	1.3E-15	trans
925	GDF2	Q9UK05	Growth/differentiation factor 2	rs77303550	72079657	16	C	T	0.803	0.073	0.009	7.4E-17	trans
925	GDF2	Q9UK05	Growth/differentiation factor 2	rs186021206	7069412	17	A	G	0.006	0.479	0.047	4.4E-24	trans
926	GDNF	P39905	Glial cell line-derived neurotrophic factor	rs62360373	37820741	5	G	A	0.905	0.393	0.013	7.5E-209	cis
926	GDNF	P39905	Glial cell line-derived neurotrophic factor	rs4808305	14800957	19	C	T	0.323	0.066	0.008	5.8E-17	trans
927	GET3	O43681	ATPase GET3	rs58534292	106483584	14	C	T	0.650	0.064	0.008	1.5E-15	trans
927	GET3	O43681	ATPase GET3	rs8009574	106462295	14	G	T	0.628	0.072	0.008	2.7E-19	trans
928	GFAP	P14136	Glial fibrillary acidic protein	rs150394890	223864380	1	G	T	0.991	0.469	0.038	5.9E-35	trans
928	GFAP	P14136	Glial fibrillary acidic protein	rs6713456	86507653	2	A	T	0.435	0.051	0.007	3.8E-13	trans
928	GFAP	P14136	Glial fibrillary acidic protein	rs5011436	12268758	7	A	C	0.585	0.059	0.007	4.3E-17	trans
928	GFAP	P14136	Glial fibrillary acidic protein	rs3132880	101673916	8	T	C	0.460	0.058	0.007	2.3E-16	trans
928	GFAP	P14136	Glial fibrillary acidic protein	rs7114754	64971396	11	T	C	0.922	0.145	0.013	3.1E-29	trans
928	GFAP	P14136	Glial fibrillary acidic protein	rs59291670	42991449	17	T	C	0.021	0.278	0.024	4.8E-31	cis
928	GFAP	P14136	Glial fibrillary acidic protein	rs429358	45411941	19	C	T	0.156	0.103	0.010	8.5E-27	trans
929	GFER	P55789	FAD-linked sulfhydryl oxidase ALR	rs61516948	2023322	16	C	T	0.874	0.104	0.011	1.2E-19	cis
930	GFRA1	P56159	GDNF family receptor alpha-1	rs10982156	117088064	9	A	T	0.069	0.185	0.014	2.1E-37	trans
930	GFRA1	P56159	GDNF family receptor alpha-1	rs11197603	118001771	10	T	C	0.852	0.303	0.010	2.0E-197	cis
930	GFRA1	P56159	GDNF family receptor alpha-1	rs6041	113772707	13	G	A	0.900	0.216	0.012	1.8E-75	trans
930	GFRA1	P56159	GDNF family receptor alpha-1	rs12934455	20357281	16	C	T	0.834	0.070	0.010	2.2E-13	trans
931	GFRA2	O00451	GDNF family receptor alpha-2	rs13107325	103188709	4	T	C	0.075	0.108	0.014	7.5E-15	trans
931	GFRA2	O00451	GDNF family receptor alpha-2	rs28478097	70309998	5	C	G	0.384	0.055	0.007	1.9E-13	trans
931	GFRA2	O00451	GDNF family receptor alpha-2	rs9275580	32679462	6	A	G	0.772	0.090	0.009	1.5E-25	trans
931	GFRA2	O00451	GDNF family receptor alpha-2	rs1480999	21474088	8	A	G	0.562	0.309	0.008	0.0E+00	cis
931	GFRA2	O00451	GDNF family receptor alpha-2	rs603424	102075479	10	G	A	0.834	0.067	0.010	6.5E-12	trans
931	GFRA2	O00451	GDNF family receptor alpha-2	rs186021206	7069412	17	A	G	0.006	0.834	0.050	1.9E-61	trans
932	GFRA3	O60609	GDNF family receptor alpha-3	rs12027119	60152685	1	A	G	0.333	0.053	0.008	4.9E-12	trans
932	GFRA3	O60609	GDNF family receptor alpha-3	rs35383942	201437832	1	T	C	0.062	0.111	0.015	1.1E-13	trans
932	GFRA3	O60609	GDNF family receptor alpha-3	rs12710696	19320803	2	T	C	0.360	0.061	0.007	3.5E-16	trans
932	GFRA3	O60609	GDNF family receptor alpha-3	rs73158489	153757862	3	T	C	0.160	0.075	0.010	2.6E-14	trans
932	GFRA3	O60609	GDNF family receptor alpha-3	rs13107325	103188709	4	T	C	0.075	0.140	0.014	2.7E-24	trans
932	GFRA3	O60609	GDNF family receptor alpha-3	rs3756599	109026663	5	G	C	0.675	0.064	0.008	1.0E-16	trans
932	GFRA3	O60609	GDNF family receptor alpha-3	rs56259296	137480609	5	A	T	0.365	0.092	0.008	1.9E-29	cis
932	GFRA3	O60609	GDNF family receptor alpha-3	rs62405538	10324696	6	A	T	0.788	0.063	0.009	7.7E-13	trans
932	GFRA3	O60609	GDNF family receptor alpha-3	rs10956755	135302956	8	A	G	0.700	0.054	0.008	7.8E-12	trans
932	GFRA3	O60609	GDNF family receptor alpha-3	rs56278466	17875857	10	G	T	0.661	0.081	0.008	1.4E-26	trans
932	GFRA3	O60609	GDNF family receptor alpha-3	rs10887793	89990905	10	T	G	0.449	0.065	0.007	4.0E-19	trans
932	GFRA3	O60609	GDNF family receptor alpha-3	rs10742826	48093445	11	T	C	0.684	0.060	0.008	8.1E-15	trans
932	GFRA3	O60609	GDNF family receptor alpha-3	rs112771035	126225876	11	C	G	0.929	0.097	0.014	4.3E-12	trans
932	GFRA3	O60609	GDNF family receptor alpha-3	rs12901860	51189326	15	A	G	0.166	0.071	0.010	1.7E-13	trans
932	GFRA3	O60609	GDNF family receptor alpha-3	rs186021206	7069412	17	A	G	0.006	0.536	0.049	2.2E-27	trans
932	GFRA3	O60609	GDNF family receptor alpha-3	rs11655511	59287269	17	T	C	0.767	0.104	0.009	2.4E-33	trans
932	GFRA3	O60609	GDNF family receptor alpha-3	rs3744052	74558057	17	T	C	0.205	0.074	0.009	1.3E-16	trans
933	GFRAL	Q6UXV0	GDNF family receptor alpha-like	rs11695471	25457708	2	T	A	0.667	0.052	0.007	6.3E-13	trans
933	GFRAL	Q6UXV0	GDNF family receptor alpha-like	rs893832	98475083	3	A	G	0.548	0.072	0.007	2.8E-26	trans
933	GFRAL	Q6UXV0	GDNF family receptor alpha-like	rs4710973	21385702	6	T	C	0.372	0.068	0.008	2.3E-19	trans
933	GFRAL	Q6UXV0	GDNF family receptor alpha-like	rs72988065	55484581	6	G	A	0.154	0.370	0.010	3.1E-285	cis
933	GFRAL	Q6UXV0	GDNF family receptor alpha-like	rs4263799	72394991	8	T	C	0.480	0.058	0.007	6.0E-18	trans
933	GFRAL	Q6UXV0	GDNF family receptor alpha-like	rs8176747	136131315	9	G	C	0.062	0.506	0.014	2.9E-279	trans
933	GFRAL	Q6UXV0	GDNF family receptor alpha-like	rs11603123	126305495	11	A	G	0.033	0.439	0.019	8.4E-122	trans
933	GFRAL	Q6UXV0	GDNF family receptor alpha-like	rs7147346	35500702	14	C	T	0.536	0.046	0.007	6.3E-12	trans
933	GFRAL	Q6UXV0	GDNF family receptor alpha-like	rs186021206	7069412	17	A	G	0.007	0.371	0.046	4.6E-16	trans
933	GFRAL	Q6UXV0	GDNF family receptor alpha-like	rs17265513	39832628	20	C	T	0.200	0.078	0.008	1.5E-20	trans
934	GGACT	Q9BVM4	Gamma-glutamylaminecyclotransferase	rs1354034	56849749	3	C	T	0.604	0.055	0.007	7.3E-14	trans
934	GGACT	Q9BVM4	Gamma-glutamylaminecyclotransferase	rs6993770	106581528	8	A	T	0.713	0.090	0.008	8.1E-30	trans
934	GGACT	Q9BVM4	Gamma-glutamylaminecyclotransferase	rs7896518	65104500	10	G	A	0.424	0.055	0.007	5.1E-14	trans
934	GGACT	Q9BVM4	Gamma-glutamylaminecyclotransferase	rs4772304	101195523	13	G	A	0.487	0.445	0.008	0.0E+00	cis
935	GGCT	O75223	Gamma-glutamylcyclotransferase	rs1354034	56849749	3	C	T	0.604	0.069	0.008	2.6E-19	trans
935	GGCT	O75223	Gamma-glutamylcyclotransferase	rs4259	30543734	7	G	A	0.273	0.119	0.008	2.9E-45	cis
936	GGH	Q92820	Gamma-glutamyl hydrolase	rs114165349	27021913	1	C	G	0.023	0.203	0.024	8.1E-18	trans
936	GGH	Q92820	Gamma-glutamyl hydrolase	rs1513275	28259233	7	C	T	0.192	0.070	0.009	2.7E-14	trans
936	GGH	Q92820	Gamma-glutamyl hydrolase	rs74329104	63931196	8	A	G	0.066	0.723	0.015	0.0E+00	cis
936	GGH	Q92820	Gamma-glutamyl hydrolase	rs28601761	126500031	8	G	C	0.419	0.052	0.008	9.5E-12	trans
936	GGH	Q92820	Gamma-glutamyl hydrolase	rs117230571	41689067	13	G	A	0.076	0.099	0.013	1.5E-13	trans
936	GGH	Q92820	Gamma-glutamyl hydrolase	rs139974673	44027885	15	C	T	0.024	0.169	0.023	1.5E-13	trans
936	GGH	Q92820	Gamma-glutamyl hydrolase	rs12970396	60109169	18	A	G	0.301	0.056	0.008	6.5E-13	trans
936	GGH	Q92820	Gamma-glutamyl hydrolase	rs58542926	19379549	19	T	C	0.075	0.102	0.013	3.4E-14	trans
936	GGH	Q92820	Gamma-glutamyl hydrolase	rs12975366	54759361	19	T	C	0.604	0.097	0.007	3.1E-41	trans
937	GGT1	P19440	Glutathione hydrolase 1 proenzyme	rs924204	16513926	1	G	A	0.589	0.064	0.007	1.7E-20	trans
937	GGT1	P19440	Glutathione hydrolase 1 proenzyme	rs4665972	27598097	2	T	C	0.392	0.048	0.007	9.2E-12	trans
937	GGT1	P19440	Glutathione hydrolase 1 proenzyme	rs4835265	146821410	4	A	C	0.156	0.090	0.009	7.7E-22	trans
937	GGT1	P19440	Glutathione hydrolase 1 proenzyme	rs4998040	56093999	18	G	A	0.212	0.061	0.008	3.2E-13	trans
937	GGT1	P19440	Glutathione hydrolase 1 proenzyme	rs28509371	24997846	22	C	T	0.337	0.254	0.007	1.8E-263	cis
938	GGT5	P36269	Glutathione hydrolase 5 proenzyme	rs74612335	126238633	11	T	C	0.867	0.104	0.011	1.2E-21	trans
938	GGT5	P36269	Glutathione hydrolase 5 proenzyme	rs186021206	7069412	17	A	G	0.006	0.635	0.051	3.7E-36	trans
938	GGT5	P36269	Glutathione hydrolase 5 proenzyme	rs573431210	67276383	17	G	T	0.027	0.157	0.023	7.7E-12	trans
938	GGT5	P36269	Glutathione hydrolase 5 proenzyme	rs5760275	24643449	22	G	C	0.207	0.329	0.009	2.7E-270	cis
939	GHR	P10912	Growth hormone receptor	rs1260326	27730940	2	T	C	0.392	0.049	0.007	3.3E-12	trans
939	GHR	P10912	Growth hormone receptor	rs4073476	42625592	5	A	C	0.727	0.685	0.009	0.0E+00	cis
939	GHR	P10912	Growth hormone receptor	rs28601761	126500031	8	C	G	0.581	0.050	0.007	6.8E-13	trans
939	GHR	P10912	Growth hormone receptor	rs174568	61593816	11	T	C	0.344	0.049	0.007	1.0E-11	trans
940	GHRL	Q9UBU3	Appetite-regulating hormone	rs34911341	10331519	3	T	C	0.006	1.419	0.047	9.2E-201	cis
940	GHRL	Q9UBU3	Appetite-regulating hormone	rs7438696	26518865	4	T	G	0.282	0.070	0.008	4.2E-18	trans
940	GHRL	Q9UBU3	Appetite-regulating hormone	rs2087038	71911588	4	A	G	0.710	0.067	0.008	3.2E-17	trans
940	GHRL	Q9UBU3	Appetite-regulating hormone	rs11745870	169622800	5	C	T	0.686	0.076	0.008	1.7E-22	trans
940	GHRL	Q9UBU3	Appetite-regulating hormone	rs11786721	143760179	8	C	T	0.410	0.071	0.007	5.0E-22	trans
940	GHRL	Q9UBU3	Appetite-regulating hormone	rs4500149	113469448	9	A	G	0.425	0.064	0.007	2.6E-18	trans
940	GHRL	Q9UBU3	Appetite-regulating hormone	rs8176746	136131322	9	G	T	0.939	0.158	0.015	3.5E-26	trans
940	GHRL	Q9UBU3	Appetite-regulating hormone	rs17138478	36073320	17	A	C	0.131	0.078	0.011	2.0E-13	trans
940	GHRL	Q9UBU3	Appetite-regulating hormone	rs6129534	38884859	20	T	G	0.296	0.074	0.008	7.1E-21	trans
941	GIGYF2	Q6Y7W6	GRB10-interacting GYF protein 2	rs1354034	56849749	3	C	T	0.604	0.072	0.008	1.2E-21	trans
941	GIGYF2	Q6Y7W6	GRB10-interacting GYF protein 2	rs5021727	32578633	6	G	A	0.461	0.061	0.008	4.5E-16	trans
942	GIMAP7	Q8NHV1	GTPase IMAP family member 7	rs3735080	150217309	7	C	T	0.769	0.607	0.009	0.0E+00	cis
942	GIMAP7	Q8NHV1	GTPase IMAP family member 7	rs56156922	56987369	16	C	T	0.325	0.066	0.007	2.0E-18	trans
942	GIMAP7	Q8NHV1	GTPase IMAP family member 7	rs4632248	54324995	19	T	G	0.209	0.059	0.009	5.6E-12	trans
943	GIP	P09681	Gastric inhibitory polypeptide	rs6879048	95778033	5	A	T	0.834	0.138	0.010	2.0E-45	trans
944	GIPC2	Q8TF65	PDZ domain-containing protein GIPC2	rs7537908	78518939	1	A	G	0.794	0.314	0.009	2.4E-268	cis
945	GIPC3	Q8TF64	PDZ domain-containing protein GIPC3	rs6993770	106581528	8	A	T	0.713	0.065	0.008	3.7E-15	trans
945	GIPC3	Q8TF64	PDZ domain-containing protein GIPC3	rs34722692	3585519	19	G	A	0.888	0.434	0.012	1.1E-290	cis
946	GIT1	Q9Y2X7	ARF GTPase-activating protein GIT1	rs1354034	56849749	3	C	T	0.604	0.061	0.008	1.5E-15	trans
946	GIT1	Q9Y2X7	ARF GTPase-activating protein GIT1	rs342296	106372903	7	G	A	0.544	0.051	0.007	9.6E-12	trans
946	GIT1	Q9Y2X7	ARF GTPase-activating protein GIT1	rs142511067	28056287	17	G	C	0.022	0.245	0.027	3.1E-20	cis
947	GKN1	Q9NS71	Gastrokine-1	rs71469854	1215138	11	T	C	0.175	0.088	0.010	2.5E-18	trans
947	GKN1	Q9NS71	Gastrokine-1	rs601338	49206674	19	G	A	0.491	0.086	0.008	4.5E-29	trans
947	GKN1	Q9NS71	Gastrokine-1	rs4816636	41026510	21	G	T	0.749	0.064	0.009	4.2E-13	trans
948	GLA	P06280	Alpha-galactosidase A	rs1260326	27730940	2	T	C	0.392	0.068	0.008	4.2E-19	trans
948	GLA	P06280	Alpha-galactosidase A	rs11650885	55085265	17	C	T	0.539	0.063	0.008	1.2E-16	trans
948	GLA	P06280	Alpha-galactosidase A	rs3027584	100662921	X	T	C	0.015	1.528	0.025	0.0E+00	cis
949	GLB1	P16278	Beta-galactosidase	rs114165349	27021913	1	C	G	0.023	0.177	0.024	9.3E-14	trans
949	GLB1	P16278	Beta-galactosidase	rs7637133	33138763	3	A	C	0.616	0.228	0.007	1.1E-209	cis
949	GLB1	P16278	Beta-galactosidase	rs687339	135932359	3	T	C	0.771	0.069	0.009	1.4E-15	trans
949	GLB1	P16278	Beta-galactosidase	rs9457800	160411747	6	C	T	0.865	0.071	0.010	1.0E-11	trans
949	GLB1	P16278	Beta-galactosidase	rs1293303	11720227	8	C	G	0.446	0.074	0.007	5.9E-25	trans
949	GLB1	P16278	Beta-galactosidase	rs150641790	41687844	13	TG	T	0.075	0.107	0.014	3.5E-15	trans
949	GLB1	P16278	Beta-galactosidase	rs145078947	93652974	14	T	G	0.003	0.729	0.070	2.2E-25	trans
949	GLB1	P16278	Beta-galactosidase	rs58542926	19379549	19	T	C	0.075	0.198	0.014	1.1E-48	trans
949	GLB1	P16278	Beta-galactosidase	rs142042446	45386467	19	G	GTAA	0.850	0.070	0.010	4.7E-12	trans
950	GLO1	Q04760	Lactoylglutathione lyase	rs4746	38650628	6	T	G	0.536	0.331	0.008	0.0E+00	cis
951	GLOD4	Q9HC38	Glyoxalase domain-containing protein 4	rs1354034	56849749	3	C	T	0.604	0.058	0.008	2.2E-14	trans
952	GLRX	P35754	Glutaredoxin-1	rs6556886	95163449	5	G	A	0.871	0.753	0.011	0.0E+00	cis
952	GLRX	P35754	Glutaredoxin-1	rs11759553	135422296	6	A	T	0.727	0.065	0.007	3.4E-18	trans
953	GLRX5	Q86SX6	Glutaredoxin-related protein 5, mitochondrial	rs342299	106373718	7	C	T	0.545	0.058	0.008	2.8E-14	trans
953	GLRX5	Q86SX6	Glutaredoxin-related protein 5, mitochondrial	rs7080386	65048306	10	A	C	0.412	0.063	0.008	7.6E-16	trans
953	GLRX5	Q86SX6	Glutaredoxin-related protein 5, mitochondrial	rs179133	95995853	14	T	C	0.183	0.156	0.010	1.7E-54	cis
954	GLYR1	Q49A26	Putative oxidoreductase GLYR1	rs7080536	115348046	10	G	A	0.956	0.229	0.019	4.7E-34	trans

955	GM2A	P17900	Ganglioside GM2 activator	rs153478	150639439	5	A	G	0.357	0.695	0.008	0.0E+00	cis
955	GM2A	P17900	Ganglioside GM2 activator	rs77924615	20392332	16	G	A	0.803	0.064	0.008	4.7E-15	trans
956	GMFG	O60234	Glia maturation factor gamma	rs1354034	56849749	3	C	T	0.604	0.054	0.008	1.8E-12	trans
956	GMFG	O60234	Glia maturation factor gamma	rs342298	106373646	7	C	T	0.545	0.063	0.008	8.8E-17	trans
956	GMFG	O60234	Glia maturation factor gamma	rs194016	39817100	19	A	G	0.890	0.104	0.012	5.1E-18	cis
957	GMPR	P36959	GMP reductase 1	rs1042391	16290761	6	A	T	0.380	0.252	0.008	2.8E-245	cis
957	GMPR	P36959	GMP reductase 1	rs342293	106372219	7	C	G	0.541	0.049	0.007	7.6E-12	trans
958	GMPR2	Q9P2T1	GMP reductase 2	rs1354034	56849749	3	C	T	0.604	0.068	0.008	4.1E-19	trans
958	GMPR2	Q9P2T1	GMP reductase 2	rs7765828	16294722	6	C	G	0.619	0.094	0.008	5.8E-34	trans
958	GMPR2	Q9P2T1	GMP reductase 2	rs7080386	65048306	10	A	C	0.412	0.061	0.008	1.5E-15	trans
958	GMPR2	Q9P2T1	GMP reductase 2	rs11502185	180258	11	C	T	0.260	0.065	0.010	1.0E-11	trans
959	GNAS	O95467	Neuroendocrine secretory protein 55	rs188468174	25291697	1	T	C	0.014	0.241	0.032	5.4E-14	trans
959	GNAS	O95467	Neuroendocrine secretory protein 55	rs41263822	32632447	6	C	A	0.657	0.081	0.008	1.0E-22	trans
959	GNAS	O95467	Neuroendocrine secretory protein 55	rs6026450	57272722	20	G	A	0.632	0.075	0.008	1.4E-22	cis
960	GNE	Q9Y223	Bifunctional UDP-N-acetylglucosamine 2-epimerase/N-acetylmannosamine kinase	rs1354034	56849749	3	C	T	0.604	0.128	0.008	6.7E-62	trans
960	GNE	Q9Y223	Bifunctional UDP-N-acetylglucosamine 2-epimerase/N-acetylmannosamine kinase	rs6993770	106581528	8	A	T	0.713	0.061	0.008	3.6E-13	trans
960	GNE	Q9Y223	Bifunctional UDP-N-acetylglucosamine 2-epimerase/N-acetylmannosamine kinase	rs7090758	65335315	10	T	C	0.524	0.053	0.008	3.1E-12	trans
960	GNE	Q9Y223	Bifunctional UDP-N-acetylglucosamine 2-epimerase/N-acetylmannosamine kinase	rs11502185	180258	11	C	T	0.260	0.069	0.010	1.3E-12	trans
961	GNLY	P22749	Granulysin	rs144015106	25074572	1	A	G	0.993	0.503	0.042	5.3E-33	trans
961	GNLY	P22749	Granulysin	rs628745	57406927	1	C	T	0.330	0.057	0.007	6.9E-16	trans
961	GNLY	P22749	Granulysin	rs7545591	185395846	1	G	C	0.322	0.058	0.007	3.7E-16	trans
961	GNLY	P22749	Granulysin	rs7577293	85935282	2	T	C	0.231	0.708	0.009	0.0E+00	cis
961	GNLY	P22749	Granulysin	rs73069104	47280733	3	C	T	0.363	0.067	0.007	4.0E-22	trans
961	GNLY	P22749	Granulysin	rs11711157	194061826	3	C	T	0.722	0.056	0.007	2.6E-14	trans
961	GNLY	P22749	Granulysin	rs2927608	96252432	5	A	G	0.450	0.072	0.007	2.5E-27	trans
961	GNLY	P22749	Granulysin	rs9368661	31156041	6	T	C	0.260	0.126	0.008	7.1E-61	trans
961	GNLY	P22749	Granulysin	rs8177651	6015390	10	A	T	0.323	0.055	0.007	4.2E-14	trans
961	GNLY	P22749	Granulysin	rs4919420	101806252	10	A	G	0.584	0.066	0.007	7.1E-23	trans
961	GNLY	P22749	Granulysin	rs9916257	33797371	17	G	T	0.560	0.056	0.007	4.9E-17	trans
962	GNPDA1	P46926	Glucosamine-6-phosphate isomerase 1	rs2228243	186395113	3	G	A	0.200	0.087	0.009	2.9E-20	trans
962	GNPDA1	P46926	Glucosamine-6-phosphate isomerase 1	rs11949303	141407705	5	C	T	0.149	0.128	0.011	7.1E-33	cis
963	GNPDA2	Q8TDQ7	Glucosamine-6-phosphate isomerase 2	rs1354034	56849749	3	C	T	0.604	0.069	0.008	5.2E-20	trans
963	GNPDA2	Q8TDQ7	Glucosamine-6-phosphate isomerase 2	rs9324861	141396566	5	C	T	0.851	0.248	0.010	1.1E-124	trans
964	GOLGA3	Q08378	Golgin subfamily A member 3	rs3811444	248039451	1	C	T	0.667	0.187	0.008	1.5E-122	trans
964	GOLGA3	Q08378	Golgin subfamily A member 3	rs1354034	56849749	3	C	T	0.604	0.096	0.008	3.8E-37	trans
964	GOLGA3	Q08378	Golgin subfamily A member 3	rs342299	106373718	7	C	T	0.545	0.055	0.007	2.6E-13	trans
965	GOLM2	Q6P4E1	Protein GOLM2	rs10804823	167810566	3	T	A	0.494	0.052	0.007	2.2E-12	trans
965	GOLM2	Q6P4E1	Protein GOLM2	rs13107325	103188709	4	C	T	0.925	0.106	0.014	5.3E-14	trans
965	GOLM2	Q6P4E1	Protein GOLM2	rs1265905	31909941	6	A	G	0.870	0.111	0.011	3.3E-24	trans
965	GOLM2	Q6P4E1	Protein GOLM2	rs41266341	159006373	6	C	A	0.009	0.409	0.039	1.1E-25	trans
965	GOLM2	Q6P4E1	Protein GOLM2	rs488962	117072525	11	T	C	0.226	0.214	0.009	3.2E-129	trans
965	GOLM2	Q6P4E1	Protein GOLM2	rs77542162	67081278	17	G	A	0.023	0.235	0.025	8.8E-22	trans
966	GOPC	Q9HD26	Golgi-associated PDZ and coiled-coil motif-containing protein	rs1354034	56849749	3	C	T	0.604	0.077	0.008	3.2E-24	trans
966	GOPC	Q9HD26	Golgi-associated PDZ and coiled-coil motif-containing protein	rs6993770	106581528	8	A	T	0.713	0.057	0.008	7.8E-12	trans
966	GOPC	Q9HD26	Golgi-associated PDZ and coiled-coil motif-containing protein	rs11502185	180258	11	C	T	0.260	0.077	0.010	8.1E-16	trans
966	GOPC	Q9HD26	Golgi-associated PDZ and coiled-coil motif-containing protein	rs892090	55539072	19	G	T	0.834	0.069	0.010	1.1E-11	trans
967	GORASP2	Q9H8Y8	Golgi reassembly-stacking protein 2	rs1354034	56849749	3	C	T	0.605	0.072	0.008	7.7E-21	trans
967	GORASP2	Q9H8Y8	Golgi reassembly-stacking protein 2	rs6993770	106581528	8	A	T	0.713	0.065	0.008	4.5E-15	trans
968	GOT1	P17174	Aspartate aminotransferase, cytoplasmic	rs56278466	17875857	10	G	T	0.661	0.109	0.008	3.1E-45	trans
968	GOT1	P17174	Aspartate aminotransferase, cytoplasmic	rs3747207	44324855	22	A	G	0.215	0.083	0.009	4.6E-21	trans
969	GP1BA	P07359	Platelet glycoprotein Ib alpha chain	rs3767809	118154831	1	C	T	0.224	0.067	0.009	7.4E-14	trans
969	GP1BA	P07359	Platelet glycoprotein Ib alpha chain	rs3804749	122833003	3	C	T	0.407	0.053	0.008	3.3E-12	trans
969	GP1BA	P07359	Platelet glycoprotein Ib alpha chain	rs78565404	184090242	3	T	C	0.055	0.132	0.017	2.0E-15	trans
969	GP1BA	P07359	Platelet glycoprotein Ib alpha chain	rs2853677	1287194	5	G	A	0.423	0.052	0.008	3.1E-12	trans
969	GP1BA	P07359	Platelet glycoprotein Ib alpha chain	rs35786788	135419042	6	A	G	0.252	0.104	0.009	5.1E-33	trans
969	GP1BA	P07359	Platelet glycoprotein Ib alpha chain	rs6961069	80218961	7	T	C	0.402	0.061	0.008	1.8E-15	trans
969	GP1BA	P07359	Platelet glycoprotein Ib alpha chain	rs6993770	106581528	8	A	T	0.713	0.126	0.008	8.3E-53	trans
969	GP1BA	P07359	Platelet glycoprotein Ib alpha chain	rs385893	4763176	9	C	T	0.524	0.108	0.007	3.2E-47	trans
969	GP1BA	P07359	Platelet glycoprotein Ib alpha chain	rs12762934	104359884	10	T	C	0.325	0.054	0.008	7.2E-12	trans
969	GP1BA	P07359	Platelet glycoprotein Ib alpha chain	rs11217176	119065044	11	A	G	0.268	0.068	0.008	8.4E-16	trans
969	GP1BA	P07359	Platelet glycoprotein Ib alpha chain	rs7944186	126289368	11	A	G	0.474	0.087	0.007	4.4E-31	trans
969	GP1BA	P07359	Platelet glycoprotein Ib alpha chain	rs2127870	65796846	14	C	G	0.795	0.064	0.009	4.8E-12	trans
969	GP1BA	P07359	Platelet glycoprotein Ib alpha chain	rs1555405	101176769	14	G	A	0.749	0.072	0.009	7.8E-17	trans
969	GP1BA	P07359	Platelet glycoprotein Ib alpha chain	rs7176023	65101007	15	C	T	0.066	0.110	0.015	3.6E-13	trans
969	GP1BA	P07359	Platelet glycoprotein Ib alpha chain	rs553749201	4837195	17	C	A	0.947	0.256	0.017	3.1E-51	cis
970	GP1BB	P13224	Platelet glycoprotein Ib beta chain	rs826404	118164722	1	C	T	0.586	0.190	0.008	9.1E-133	trans
970	GP1BB	P13224	Platelet glycoprotein Ib beta chain	rs3811444	248039451	1	C	T	0.668	0.063	0.008	5.4E-15	trans
970	GP1BB	P13224	Platelet glycoprotein Ib beta chain	rs854572	94954696	7	G	C	0.528	0.067	0.008	7.3E-19	trans
970	GP1BB	P13224	Platelet glycoprotein Ib beta chain	rs6993770	106581528	8	A	T	0.714	0.105	0.008	2.2E-36	trans
970	GP1BB	P13224	Platelet glycoprotein Ib beta chain	rs7896518	65104500	10	G	A	0.425	0.111	0.008	3.4E-47	trans
970	GP1BB	P13224	Platelet glycoprotein Ib beta chain	rs77639164	88568054	16	C	A	0.278	0.098	0.009	9.0E-31	trans
970	GP1BB	P13224	Platelet glycoprotein Ib beta chain	rs56337033	4835627	17	T	C	0.004	0.520	0.067	7.5E-15	trans
971	GP2	P55259	Pancreatic secretory granule membrane major glycoprotein GP2	rs6571015	96534478	6	A	G	0.790	0.148	0.008	2.9E-71	trans
971	GP2	P55259	Pancreatic secretory granule membrane major glycoprotein GP2	rs6459924	158908074	7	T	C	0.759	0.062	0.008	2.6E-15	trans
971	GP2	P55259	Pancreatic secretory granule membrane major glycoprotein GP2	rs4430753	20332200	16	A	G	0.512	0.233	0.007	2.8E-255	cis
971	GP2	P55259	Pancreatic secretory granule membrane major glycoprotein GP2	rs200489612	7106378	17	A	G	0.005	0.373	0.051	1.6E-13	trans
971	GP2	P55259	Pancreatic secretory granule membrane major glycoprotein GP2	rs708686	5840619	19	T	C	0.268	0.063	0.008	2.9E-16	trans
971	GP2	P55259	Pancreatic secretory granule membrane major glycoprotein GP2	rs492602	49206417	19	G	A	0.509	0.169	0.007	1.0E-135	trans
972	GP5	P40197	Platelet glycoprotein V	rs56221835	231019655	1	C	G	0.093	0.093	0.013	1.7E-12	trans
972	GP5	P40197	Platelet glycoprotein V	rs3804749	122833003	3	C	T	0.407	0.111	0.008	8.4E-45	trans
972	GP5	P40197	Platelet glycoprotein V	rs6993770	106581528	8	A	T	0.713	0.081	0.008	4.3E-22	trans
972	GP5	P40197	Platelet glycoprotein V	rs12005199	4763491	9	A	G	0.312	0.058	0.008	5.3E-12	trans
972	GP5	P40197	Platelet glycoprotein V	rs8099942	55533478	19	G	C	0.801	0.070	0.010	7.3E-13	trans
973	GP6	Q9HCN6	Platelet glycoprotein VI	rs1354034	56849749	3	C	T	0.604	0.059	0.008	4.1E-15	trans
973	GP6	Q9HCN6	Platelet glycoprotein VI	rs17622656	131820997	5	G	A	0.611	0.053	0.008	2.5E-12	trans
973	GP6	Q9HCN6	Platelet glycoprotein VI	rs6993770	106581528	8	A	T	0.714	0.078	0.008	9.6E-22	trans
973	GP6	Q9HCN6	Platelet glycoprotein VI	rs7896518	65104500	10	G	A	0.424	0.059	0.008	8.3E-15	trans
973	GP6	Q9HCN6	Platelet glycoprotein VI	rs892090	55539072	19	G	T	0.834	0.413	0.010	0.0E+00	cis
974	GPA33	Q99795	Cell surface A33 antigen	rs72689400	167038219	1	C	A	0.984	0.679	0.029	7.3E-124	cis
974	GPA33	Q99795	Cell surface A33 antigen	rs2241764	133174764	2	C	T	0.661	0.094	0.008	8.1E-35	trans
974	GPA33	Q99795	Cell surface A33 antigen	rs35853577	233558879	2	T	C	0.574	0.074	0.007	1.0E-23	trans
974	GPA33	Q99795	Cell surface A33 antigen	rs2077610	70911271	3	A	T	0.552	0.051	0.007	2.6E-12	trans
974	GPA33	Q99795	Cell surface A33 antigen	rs9816553	153957050	3	G	A	0.148	0.090	0.010	2.0E-18	trans
974	GPA33	Q99795	Cell surface A33 antigen	rs939885	195955762	3	A	G	0.485	0.072	0.007	3.5E-23	trans
974	GPA33	Q99795	Cell surface A33 antigen	rs6911827	22130601	6	T	C	0.453	0.051	0.007	1.5E-12	trans
974	GPA33	Q99795	Cell surface A33 antigen	rs9478784	151020330	6	T	C	0.096	0.123	0.012	7.9E-24	trans
974	GPA33	Q99795	Cell surface A33 antigen	rs931493	71145692	9	G	A	0.968	0.176	0.021	5.5E-17	trans
974	GPA33	Q99795	Cell surface A33 antigen	rs2291428	45958856	10	G	C	0.756	0.085	0.008	3.7E-24	trans
974	GPA33	Q99795	Cell surface A33 antigen	rs56398830	103701690	13	G	A	0.989	0.889	0.035	2.8E-146	trans
974	GPA33	Q99795	Cell surface A33 antigen	rs35193388	4420817	16	G	GC	0.265	0.074	0.008	4.9E-19	trans
974	GPA33	Q99795	Cell surface A33 antigen	rs6505173	28646057	17	G	A	0.440	0.162	0.007	4.2E-109	trans
974	GPA33	Q99795	Cell surface A33 antigen	rs681343	49206462	19	C	T	0.491	0.207	0.007	5.1E-177	trans
974	GPA33	Q99795	Cell surface A33 antigen	rs3746778	61341472	20	A	G	0.420	0.072	0.007	7.6E-22	trans
975	GPC1	P35052	Glypican-1	rs1535701	93805201	1	G	T	0.623	0.079	0.007	1.7E-27	trans
975	GPC1	P35052	Glypican-1	rs12758626	118092167	1	A	G	0.651	0.050	0.007	1.5E-11	trans
975	GPC1	P35052	Glypican-1	rs150816167	179571862	1	C	T	0.042	0.125	0.018	1.9E-12	trans
975	GPC1	P35052	Glypican-1	rs2273967	230415293	1	T	C	0.249	0.072	0.008	9.6E-19	trans
975	GPC1	P35052	Glypican-1	rs71414736	127861945	2	C	G	0.978	0.192	0.025	1.5E-14	trans
975	GPC1	P35052	Glypican-1	rs147895889	170959089	2	A	G	0.989	0.272	0.035	1.6E-14	trans
975	GPC1	P35052	Glypican-1	rs77078946	241403417	2	G	T	0.114	0.506	0.011	0.0E+00	cis
975	GPC1	P35052	Glypican-1	rs200409727	129197499	4	A	AT	0.803	0.062	0.009	2.8E-12	trans
975	GPC1	P35052	Glypican-1	rs3965330	177049442	5	A	G	0.469	0.189	0.007	1.1E-155	trans
975	GPC1	P35052	Glypican-1	rs2625445	61540328	8	G	A	0.757	0.074	0.008	2.3E-19	trans
975	GPC1	P35052	Glypican-1	rs7835610	91709053	8	C	T	0.495	0.055	0.007	2.8E-15	trans
975	GPC1	P35052	Glypican-1	rs56278466	17875857	10	G	T	0.661	0.062	0.007	8.0E-17	trans
975	GPC1	P35052	Glypican-1	rs9318186	74106939	13	A	G	0.445	0.052	0.007	2.7E-13	trans
975	GPC1	P35052	Glypican-1	rs7147722	50112712	14	T	G	0.538	0.103	0.007	1.7E-48	trans
975	GPC1	P35052	Glypican-1	rs1801689	64210580	17	C	A	0.030	0.191	0.021	1.0E-20	trans
975	GPC1	P35052	Glypican-1	rs5763646	30359410	22	C	T	0.412	0.058	0.007	6.8E-16	trans
976	GPC5	P78333	Glypican-5	rs1260326	27730940	2	T	C	0.392	0.058	0.006	6.2E-20	trans
976	GPC5	P78333	Glypican-5	rs1354034	56849749	3	T	C	0.396	0.048	0.006	9.9E-15	trans
976	GPC5	P78333	Glypican-5	rs4098923	31193756	6	A	G	0.552	0.044	0.006	1.1E-11	trans
976	GPC5	P78333	Glypican-5	rs6993770	106581528	8	A	T	0.713	0.062	0.007	9.6E-20	trans
976	GPC5	P78333	Glypican-5	rs28601761	126500031	8	C	G	0.581	0.051	0.006	9.8E-16	trans
976	GPC5	P78333	Glypican-5	rs342706	92417058	13	T	C	0.728	0.878	0.009	0.0E+00	cis
976	GPC5	P78333	Glypican-5	rs1654425	55538980	19	C	T	0.834	0.092	0.008	1.4E-28	trans
977	GPHA2	Q96T91	Glycoprotein hormone alpha-2	rs114391421	64730319	11	A	C	0.046	0.499	0.018	4.0E-174	cis
977	GPHA2	Q96T91	Glycoprotein hormone alpha-2	rs1002136	2098006	17	C	A	0.600	0.101	0.008	2.2E-40	trans
978	GPI	P06744	Glucose-6-phosphate isomerase	rs1354034	56849749	3	C	T	0.604	0.061	0.008	2.0E-15	trans
978	GPI	P06744	Glucose-6-phosphate isomerase	rs342290	106367604	7	A	C	0.544	0.055	0.008	2.1E-13	trans
979	GPIHBP1	Q8IV16	Glycosylphosphatidylinositol-anchored high density lipoprotein-binding protein 1	rs12664131	31328582	6	T	A	0.848	0.072	0.010	5.2E-12	trans
979	GPIHBP1	Q8IV16	Glycosylphosphatidylinositol-anchored high density lipoprotein-binding protein 1	rs9657541	10643164	8	C	T	0.795	0.072	0.009	2.4E-14	trans
979	GPIHBP1	Q8IV16	Glycosylphosphatidylinositol-anchored high density lipoprotein-binding protein 1	rs6420185	144303071	8	G	A	0.612	0.272	0.008	1.7E-264	cis
979	GPIHBP1	Q8IV16	Glycosylphosphatidylinositol-anchored high density lipoprotein-binding protein 1	rs8176741	136131461	9	A	G	0.061	0.149	0.016	1.0E-21	trans
980	GPNMB	Q14956	Transmembrane glycoprotein NMB	rs11721064	98408826	3	G	T	0.395	0.063	0.007	3.2E-17	trans
980	GPNMB	Q14956	Transmembrane glycoprotein NMB	rs12203592	396321	6	C	T	0.784	0.076	0.009	2.3E-17	trans
980	GPNMB	Q14956	Transmembrane glycoprotein NMB	rs75801644	23306141	7	G	A	0.977	1.118	0.026	0.0E+00	cis
980	GPNMB	Q14956	Transmembrane glycoprotein NMB	rs74612335	126238633	11	T	C	0.867	0.089	0.011	1.4E-16	trans
980	GPNMB	Q14956	Transmembrane glycoprotein NMB	rs186021206	7069412	17	A	G	0.006	0.738	0.050	1.1E-49	trans
981	GPR158	Q5T848	Probable G-protein coupled receptor 158	rs1931291	25718437	10	A	T	0.305	0.064	0.008	3.3E-15	cis
982	GPR15L	Q6UWK7	Protein GPR15L	rs61816761	152285861	1	A	G	0.023	0.236	0.025	1.8E-21	trans
982	GPR15L	Q6UWK7	Protein GPR15L	rs9326982	115331118	5	C	T	0.394	0.053	0.007	3.7E-13	trans
982	GPR15L	Q6UWK7	Protein GPR15L	rs4399275	85918938	10	G	T	0.421	0.221	0.008	2.6E-187	cis

982	GPR15L	Q6UWK7	Protein GPR15L	rs117268623	51527970	19	T	C	0.033	0.141	0.020	1.8E-12	trans
983	GPR37	O15354	Prosaposin receptor GPR37	rs780094	27741237	2	T	C	0.380	0.083	0.007	2.3E-37	trans
983	GPR37	O15354	Prosaposin receptor GPR37	rs511154	135950921	3	G	A	0.766	0.071	0.007	4.2E-21	trans
983	GPR37	O15354	Prosaposin receptor GPR37	rs60598740	124129046	7	G	A	0.738	0.785	0.009	0.0E+00	cis
983	GPR37	O15354	Prosaposin receptor GPR37	rs28601761	126500031	8	C	G	0.581	0.090	0.006	8.8E-44	trans
983	GPR37	O15354	Prosaposin receptor GPR37	rs11023881	16246700	11	A	T	0.387	0.059	0.006	3.7E-20	trans
983	GPR37	O15354	Prosaposin receptor GPR37	rs964184	116648917	11	G	C	0.133	0.063	0.009	8.0E-12	trans
983	GPR37	O15354	Prosaposin receptor GPR37	rs1292066	57932314	17	A	T	0.215	0.060	0.008	4.3E-15	trans
983	GPR37	O15354	Prosaposin receptor GPR37	rs390082	45416831	19	G	T	0.109	0.083	0.010	1.6E-16	trans
984	GPRC5C	Q9NQ84	G-protein coupled receptor family C group 5 member C	rs4835265	146821410	4	A	C	0.156	0.089	0.010	2.0E-17	trans
984	GPRC5C	Q9NQ84	G-protein coupled receptor family C group 5 member C	rs2670824	72438972	17	C	G	0.076	0.213	0.014	1.9E-49	cis
984	GPRC5C	Q9NQ84	G-protein coupled receptor family C group 5 member C	rs9959832	56086820	18	C	T	0.202	0.073	0.009	8.1E-15	trans
984	GPRC5C	Q9NQ84	G-protein coupled receptor family C group 5 member C	rs189448562	3705115	20	C	G	0.011	0.431	0.037	6.1E-32	trans
985	GRAP2	O75791	GRB2-related adapter protein 2	rs1354034	56849749	3	C	T	0.604	0.053	0.008	3.7E-12	trans
985	GRAP2	O75791	GRB2-related adapter protein 2	rs6993770	106581528	8	A	T	0.713	0.063	0.008	2.5E-14	trans
985	GRAP2	O75791	GRB2-related adapter protein 2	rs148328786	40342993	22	G	A	0.014	0.322	0.034	3.3E-21	cis
986	GRHPR	Q9UBQ7	Glyoxylate reductase/hydroxypyruvate reductase	rs1354034	56849749	3	C	T	0.604	0.057	0.007	3.0E-14	trans
986	GRHPR	Q9UBQ7	Glyoxylate reductase/hydroxypyruvate reductase	rs342298	106373646	7	C	T	0.545	0.051	0.007	5.7E-12	trans
986	GRHPR	Q9UBQ7	Glyoxylate reductase/hydroxypyruvate reductase	rs6993770	106581528	8	A	T	0.713	0.059	0.008	3.8E-13	trans
986	GRHPR	Q9UBQ7	Glyoxylate reductase/hydroxypyruvate reductase	rs4878690	37422656	9	T	C	0.326	0.269	0.008	8.0E-249	cis
987	GRIK2	Q13002	Glutamate receptor ionotropic, kainate 2	rs59321237	101329742	6	T	C	0.029	0.738	0.023	8.4E-236	cis
987	GRIK2	Q13002	Glutamate receptor ionotropic, kainate 2	rs35106244	49203829	19	T	C	0.463	0.061	0.008	3.6E-15	trans
988	GRK5	P34947	G protein-coupled receptor kinase 5	rs10886430	121010256	10	A	G	0.873	0.407	0.012	3.4E-262	cis
989	GRN	P28799	Progranulin	rs12740374	109817590	1	G	T	0.778	0.676	0.009	0.0E+00	trans
989	GRN	P28799	Progranulin	rs75029704	29849506	6	T	C	0.096	0.161	0.012	3.0E-43	trans
989	GRN	P28799	Progranulin	rs1137827	136129715	9	T	C	0.061	0.109	0.014	8.7E-15	trans
989	GRN	P28799	Progranulin	rs885828	73578503	10	G	A	0.709	0.237	0.008	7.9E-216	trans
989	GRN	P28799	Progranulin	rs2298475	126278203	11	T	C	0.925	0.102	0.013	8.7E-16	trans
989	GRN	P28799	Progranulin	rs72802342	75234872	16	C	A	0.924	0.197	0.013	7.6E-52	trans
989	GRN	P28799	Progranulin	rs186021206	7069412	17	A	G	0.006	0.684	0.047	2.4E-48	trans
989	GRN	P28799	Progranulin	rs5848	42430244	17	C	T	0.726	0.250	0.008	1.1E-229	cis
989	GRN	P28799	Progranulin	rs1065853	45413233	19	T	G	0.081	0.097	0.012	6.6E-15	trans
990	GRP	P07492	Gastrin-releasing peptide	rs964184	116648917	11	G	C	0.133	0.085	0.011	3.7E-15	trans
990	GRP	P07492	Gastrin-releasing peptide	rs7216766	46168579	17	C	G	0.185	0.075	0.009	1.5E-15	trans
990	GRP	P07492	Gastrin-releasing peptide	rs17835713	56878091	18	A	G	0.165	0.462	0.010	0.0E+00	cis
990	GRP	P07492	Gastrin-releasing peptide	rs72654473	45414399	19	A	C	0.109	0.114	0.012	2.3E-22	trans
991	GRPEL1	Q9HAV7	GrpE protein homolog 1, mitochondrial	rs4361977	155076079	1	C	T	0.566	0.053	0.008	2.1E-12	trans
991	GRPEL1	Q9HAV7	GrpE protein homolog 1, mitochondrial	rs4835265	146821410	4	A	C	0.156	0.107	0.010	5.1E-25	trans
991	GRPEL1	Q9HAV7	GrpE protein homolog 1, mitochondrial	rs9959832	56086820	18	C	T	0.202	0.081	0.009	7.1E-18	trans
992	GRSF1	Q12849	G-rich sequence factor 1	rs342298	106373646	7	C	T	0.545	0.055	0.008	1.8E-12	trans
993	GSAP	A4D1B5	Gamma-secretase-activating protein	rs73333648	100164116	10	C	G	0.946	0.141	0.017	2.4E-16	trans
994	GSN	P06396	Gelsolin	rs116185403	124083642	9	T	C	0.007	1.026	0.045	1.1E-116	cis
994	GSN	P06396	Gelsolin	rs9936550	75242850	16	C	T	0.923	0.098	0.014	4.1E-12	trans
994	GSN	P06396	Gelsolin	rs12986064	54755133	19	T	C	0.492	0.082	0.008	1.2E-26	trans
995	GSR	P00390	Glutathione reductase, mitochondrial	rs34744948	31562767	6	C	G	0.049	0.128	0.017	6.8E-14	trans
995	GSR	P00390	Glutathione reductase, mitochondrial	rs8190982	30556381	8	T	TCC	0.675	0.125	0.008	1.0E-53	cis
995	GSR	P00390	Glutathione reductase, mitochondrial	rs12342201	95894964	9	G	A	0.524	0.062	0.007	7.4E-17	trans
996	GSTA1	P08263	Glutathione S-transferase A1	rs28383314	32587213	6	C	T	0.626	0.053	0.008	4.5E-12	trans
996	GSTA1	P08263	Glutathione S-transferase A1	rs6458871	52665977	6	T	C	0.574	0.315	0.007	0.0E+00	cis
996	GSTA1	P08263	Glutathione S-transferase A1	rs112875651	126506694	8	G	A	0.609	0.059	0.007	4.4E-15	trans
996	GSTA1	P08263	Glutathione S-transferase A1	rs10733608	117148430	9	G	T	0.512	0.068	0.007	1.3E-20	trans
996	GSTA1	P08263	Glutathione S-transferase A1	rs10883451	101924418	10	T	C	0.498	0.053	0.007	2.2E-13	trans
996	GSTA1	P08263	Glutathione S-transferase A1	rs28929474	94844947	14	T	C	0.021	0.216	0.025	1.3E-17	trans
996	GSTA1	P08263	Glutathione S-transferase A1	rs10405357	54759666	19	T	C	0.565	0.058	0.007	1.6E-15	trans
996	GSTA1	P08263	Glutathione S-transferase A1	rs3747207	44324855	22	A	G	0.215	0.110	0.009	2.1E-36	trans
997	GSTA3	Q16772	Glutathione S-transferase A3	rs2749005	52621433	6	G	T	0.648	0.325	0.008	0.0E+00	cis
997	GSTA3	Q16772	Glutathione S-transferase A3	rs11370710	117158957	9	C	CA	0.506	0.052	0.007	5.2E-12	trans
997	GSTA3	Q16772	Glutathione S-transferase A3	rs28929474	94844947	14	T	C	0.021	0.218	0.026	6.9E-17	trans
997	GSTA3	Q16772	Glutathione S-transferase A3	rs738409	44324727	22	G	C	0.217	0.097	0.009	2.4E-27	trans
998	GSTM4	Q03013	Glutathione S-transferase Mu 4	rs140584594	110232983	1	A	G	0.268	0.430	0.009	0.0E+00	cis
999	GSTP1	P09211	Glutathione S-transferase P	rs762803	67352256	11	A	C	0.449	0.205	0.008	4.0E-152	cis
1000	GSTT2B	P0CG30	Glutathione S-transferase theta-2B	rs5751777	24267047	22	C	T	0.412	1.037	0.008	0.0E+00	cis
1001	GTF2IRD1	Q9UHL9	General transcription factor II-I repeat domain-containing protein 1	rs149375627	106523717	14	TG	T	0.435	0.092	0.009	9.0E-26	trans
1002	GTPBP2	Q9BX10	GTP-binding protein 2	rs342298	106373646	7	C	T	0.545	0.054	0.008	1.0E-12	trans
1002	GTPBP2	Q9BX10	GTP-binding protein 2	rs6993770	106581528	8	A	T	0.713	0.080	0.008	6.0E-22	trans
1003	GUCA2A	Q02747	Guanylin	rs142157365	42628591	1	A	T	0.998	2.109	0.078	4.6E-162	cis
1003	GUCA2A	Q02747	Guanylin	rs10455394	82559099	6	C	G	0.510	0.088	0.007	5.0E-33	trans
1003	GUCA2A	Q02747	Guanylin	rs2511241	72945341	11	T	C	0.930	0.110	0.014	1.8E-14	trans
1003	GUCA2A	Q02747	Guanylin	rs4982408	21570614	14	C	A	0.837	0.086	0.010	3.6E-18	trans
1003	GUCA2A	Q02747	Guanylin	rs1424231	79706751	16	C	T	0.337	0.052	0.008	1.6E-11	trans
1003	GUCA2A	Q02747	Guanylin	rs17743980	9793839	17	A	G	0.315	0.056	0.008	1.5E-12	trans
1004	GUCY2C	P25092	Heat-stable enterotoxin receptor	rs2516979	106348575	14	C	G	0.686	0.112	0.010	8.7E-31	trans
1005	GUSB	P08236	Beta-glucuronidase	rs6779146	135907213	3	T	C	0.769	0.100	0.008	1.8E-33	trans
1005	GUSB	P08236	Beta-glucuronidase	rs115850161	119732447	4	C	T	0.061	0.178	0.015	1.1E-33	trans
1005	GUSB	P08236	Beta-glucuronidase	rs72814178	172486739	5	G	A	0.132	0.074	0.010	7.7E-13	trans
1005	GUSB	P08236	Beta-glucuronidase	rs111331197	7883468	6	C	T	0.989	0.256	0.033	2.1E-14	trans
1005	GUSB	P08236	Beta-glucuronidase	rs28538349	64723071	7	G	A	0.481	0.052	0.007	3.3E-13	trans
1005	GUSB	P08236	Beta-glucuronidase	rs149606212	65444841	7	C	T	0.998	1.405	0.076	1.3E-76	cis
1005	GUSB	P08236	Beta-glucuronidase	rs11003134	54538159	10	A	C	0.144	0.069	0.010	4.3E-12	trans
1005	GUSB	P08236	Beta-glucuronidase	rs99780	61596633	11	T	C	0.350	0.083	0.007	4.7E-30	trans
1005	GUSB	P08236	Beta-glucuronidase	rs150641790	41687844	13	TG	T	0.075	0.095	0.013	1.1E-12	trans
1005	GUSB	P08236	Beta-glucuronidase	rs139974673	44027885	15	C	T	0.024	0.275	0.022	1.8E-34	trans
1005	GUSB	P08236	Beta-glucuronidase	rs1801690	64208285	17	C	G	0.943	0.274	0.015	5.9E-74	trans
1005	GUSB	P08236	Beta-glucuronidase	rs5826313	71958151	18	CA	C	0.321	0.063	0.007	6.8E-17	trans
1005	GUSB	P08236	Beta-glucuronidase	rs2974755	13062663	19	T	C	0.602	0.048	0.007	8.8E-12	trans
1005	GUSB	P08236	Beta-glucuronidase	rs58542926	19379549	19	T	C	0.075	0.193	0.013	1.8E-48	trans
1006	GYS1	P13807	Glycogen [starch] synthase, muscle	rs3748136	9030160	8	G	A	0.787	0.310	0.009	3.9E-247	trans
1006	GYS1	P13807	Glycogen [starch] synthase, muscle	rs59305841	47179476	16	T	A	0.042	0.126	0.019	9.9E-12	trans
1007	GZMA	P12544	Granzyme A	rs76036957	113496453	2	A	G	0.009	0.303	0.038	1.8E-15	trans
1007	GZMA	P12544	Granzyme A	rs13063578	47087837	3	T	A	0.602	0.065	0.007	2.0E-18	trans
1007	GZMA	P12544	Granzyme A	rs17033014	103546186	4	A	G	0.667	0.057	0.008	1.6E-13	trans
1007	GZMA	P12544	Granzyme A	rs2047745	54489541	5	C	T	0.209	0.070	0.009	7.7E-15	cis
1007	GZMA	P12544	Granzyme A	rs3846730	131806780	5	C	T	0.762	0.060	0.009	4.4E-12	trans
1007	GZMA	P12544	Granzyme A	rs9264645	31238524	6	A	G	0.641	0.266	0.008	1.2E-260	trans
1007	GZMA	P12544	Granzyme A	rs2347784	6524843	7	C	G	0.728	0.074	0.008	2.3E-19	trans
1007	GZMA	P12544	Granzyme A	rs6476398	33119241	9	C	T	0.690	0.087	0.008	3.7E-28	trans
1007	GZMA	P12544	Granzyme A	rs41280225	116023979	9	A	C	0.869	0.089	0.011	1.3E-16	trans
1007	GZMA	P12544	Granzyme A	rs113430316	128116241	11	T	TA	0.719	0.056	0.008	5.9E-12	trans
1007	GZMA	P12544	Granzyme A	rs145078947	93652974	14	T	G	0.003	0.656	0.071	3.6E-20	trans
1007	GZMA	P12544	Granzyme A	rs2034892	16491549	19	G	A	0.314	0.054	0.008	5.2E-12	trans
1007	GZMA	P12544	Granzyme A	rs12986064	54755133	19	T	C	0.492	0.079	0.008	1.1E-25	trans
1008	GZMB	P10144	Granzyme B	rs2843153	2245221	1	C	T	0.212	0.066	0.009	3.1E-14	trans
1008	GZMB	P10144	Granzyme B	rs1984520	25237986	1	C	T	0.632	0.059	0.007	1.5E-15	trans
1008	GZMB	P10144	Granzyme B	rs2725124	100145777	5	A	C	0.618	0.070	0.007	9.6E-22	trans
1008	GZMB	P10144	Granzyme B	rs41563615	31324375	6	T	G	0.054	0.413	0.016	2.8E-148	trans
1008	GZMB	P10144	Granzyme B	rs576785	128053274	11	T	C	0.706	0.089	0.008	1.0E-30	trans
1008	GZMB	P10144	Granzyme B	rs8192918	25101878	14	C	CT	0.776	0.354	0.009	0.0E+00	cis
1008	GZMB	P10144	Granzyme B	rs12451746	33806863	17	G	A	0.570	0.133	0.007	6.3E-77	trans
1008	GZMB	P10144	Granzyme B	rs56133626	16441973	19	G	A	0.674	0.060	0.008	1.6E-15	trans
1009	GZMH	P20718	Granzyme H	rs10732976	185409061	1	G	A	0.306	0.087	0.008	3.0E-27	trans
1009	GZMH	P20718	Granzyme H	rs2524080	31242089	6	T	A	0.676	0.123	0.008	4.9E-55	trans
1009	GZMH	P20718	Granzyme H	rs2347784	6524843	7	C	G	0.728	0.063	0.008	4.5E-14	trans
1009	GZMH	P20718	Granzyme H	rs78325861	72378489	10	G	C	0.042	0.159	0.018	3.6E-18	trans
1009	GZMH	P20718	Granzyme H	rs576785	128053274	11	T	C	0.706	0.108	0.008	9.4E-41	trans
1009	GZMH	P20718	Granzyme H	rs190749747	25076303	14	C	G	0.989	0.897	0.037	3.0E-132	cis
1009	GZMH	P20718	Granzyme H	rs56133626	16441973	19	G	A	0.673	0.053	0.008	1.5E-11	trans
1010	H2AP	O75409	Huntingtin-interacting protein M	rs35835517	106471857	14	T	G	0.148	0.145	0.011	9.3E-40	trans
1011	HADH	Q16836	Hydroxyacyl-coenzyme A dehydrogenase, mitochondrial	rs17038246	108899748	4	T	C	0.081	0.106	0.014	4.2E-14	cis
1011	HADH	Q16836	Hydroxyacyl-coenzyme A dehydrogenase, mitochondrial	rs190627972	106361376	14	C	T	0.161	0.147	0.011	1.1E-40	trans
1012	HAGH	Q16775	Hydroxyacylglutathione hydrolase, mitochondrial	rs115389912	1869974	16	G	A	0.995	0.809	0.050	3.4E-59	cis
1013	HAO1	Q9UJM8	Hydroxyacid oxidase 1	rs6656611	16515805	1	C	T	0.602	0.062	0.007	4.9E-17	trans
1013	HAO1	Q9UJM8	Hydroxyacid oxidase 1	rs12904	155106697	1	G	A	0.588	0.058	0.007	2.3E-15	trans
1013	HAO1	Q9UJM8	Hydroxyacid oxidase 1	rs7608141	296381	2	C	A	0.332	0.059	0.008	8.6E-14	trans
1013	HAO1	Q9UJM8	Hydroxyacid oxidase 1	rs4835265	146821410	4	A	C	0.156	0.119	0.010	6.6E-33	trans
1013	HAO1	Q9UJM8	Hydroxyacid oxidase 1	rs112875651	126506694	8	G	A	0.609	0.051	0.007	6.7E-12	trans
1013	HAO1	Q9UJM8	Hydroxyacid oxidase 1	rs151068477	52642516	10	T	G	0.008	0.285	0.042	1.6E-11	trans
1013	HAO1	Q9UJM8	Hydroxyacid oxidase 1	rs12373325	56087648	18	T	C	0.202	0.098	0.009	1.4E-27	trans
1013	HAO1	Q9UJM8	Hydroxyacid oxidase 1	rs738409	44324727	22	G	C	0.217	0.065	0.009	1.1E-13	trans
1014	HARS1	P12081	Histidine--tRNA ligase, cytoplasmic	rs1354034	56849749	3	C	T	0.604	0.073	0.008	1.8E-21	trans
1014	HARS1	P12081	Histidine--tRNA ligase, cytoplasmic	rs342298	106373646	7	C	T	0.546	0.053	0.008	1.4E-12	trans
1015	HAVCR1	Q96D42	Hepatitis A virus cellular receptor 1	rs2070803	155157715	1	G	A	0.426	0.049	0.006	1.1E-15	trans
1015	HAVCR1	Q96D42	Hepatitis A virus cellular receptor 1	rs434215	699046	5	A	G	0.276	0.061	0.008	2.2E-13	trans
1015	HAVCR1	Q96D42	Hepatitis A virus cellular receptor 1	rs6555820	156472149	5	C	A	0.520	0.476	0.007	0.0E+00	cis
1015	HAVCR1	Q96D42	Hepatitis A virus cellular receptor 1	rs112875651	126506694	8	G	A	0.609	0.061	0.006	1.5E-22	trans
1015	HAVCR1	Q96D42	Hepatitis A virus cellular receptor 1	rs2070895	58723939	15	A	G	0.221	0.078	0.007	1.4E-26	trans
1015	HAVCR1	Q96D42	Hepatitis A virus cellular receptor 1	rs77924615	20392332	16	G	A	0.803	0.120	0.008	1.8E-54	trans
1015	HAVCR1	Q96D42	Hepatitis A virus cellular receptor 1	rs112001035	66823805	17	A	G	0.061	0.221	0.013	3.5E-65	trans
1016	HAVCR2	Q8TDQ0	Hepatitis A virus cellular receptor 2	rs61747728	179526214	1	T	C	0.038	0.156	0.019	2.3E-16	trans
1016	HAVCR2	Q8TDQ0	Hepatitis A virus cellular receptor 2	rs7569939	62506055	2	A	G	0.480	0.061	0.007	5.8E-17	trans
1016	HAVCR2	Q8TDQ0	Hepatitis A virus cellular receptor 2	rs7630058	98337517	3	C	T	0.779	0.083	0.009	7.8E-21	trans
1016	HAVCR2	Q8TDQ0	Hepatitis A virus cellular receptor 2	rs116363290	156536137	5	C	T	0.994	1.704	0.052	4.1E-236	cis
1016	HAVCR2	Q8TDQ0	Hepatitis A virus cellular receptor 2	rs509227	10535591	6	C	A	0.523	0.054	0.007	1.5E-13	trans
1016	HAVCR2	Q8TDQ0	Hepatitis A virus cellular receptor 2	rs950802	60152584	11	A	G	0.306	0.114	0.008	3.2E-47	trans

1016	HAVCR2	Q8TDQ0	Hepatitis A virus cellular receptor 2	rs77924615	20392332	16	G	A	0.803	0.065	0.009	2.2E-12	trans
1016	HAVCR2	Q8TDQ0	Hepatitis A virus cellular receptor 2	rs186021206	7069412	17	A	G	0.006	0.479	0.050	8.3E-22	trans
1017	HBEGF	Q99075	Proheparin-binding EGF-like growth factor	rs3811444	248039451	1	C	T	0.668	0.061	0.008	5.6E-15	trans
1017	HBEGF	Q99075	Proheparin-binding EGF-like growth factor	rs78909033	241510903	2	G	A	0.864	0.086	0.011	1.7E-15	trans
1017	HBEGF	Q99075	Proheparin-binding EGF-like growth factor	rs1354034	56849749	3	T	C	0.396	0.158	0.008	1.2E-97	trans
1017	HBEGF	Q99075	Proheparin-binding EGF-like growth factor	rs274555	131722951	5	T	C	0.399	0.054	0.008	1.4E-12	trans
1017	HBEGF	Q99075	Proheparin-binding EGF-like growth factor	rs4098923	31193756	6	A	G	0.552	0.066	0.008	1.9E-17	trans
1017	HBEGF	Q99075	Proheparin-binding EGF-like growth factor	rs62396356	41167763	6	G	A	0.134	0.085	0.011	5.1E-15	trans
1017	HBEGF	Q99075	Proheparin-binding EGF-like growth factor	rs342299	106373718	7	C	T	0.545	0.052	0.007	3.6E-12	trans
1017	HBEGF	Q99075	Proheparin-binding EGF-like growth factor	rs447124	4746539	9	T	C	0.494	0.055	0.007	7.9E-14	trans
1017	HBEGF	Q99075	Proheparin-binding EGF-like growth factor	rs10820606	99192919	9	A	C	0.771	0.115	0.009	1.0E-37	trans
1017	HBEGF	Q99075	Proheparin-binding EGF-like growth factor	rs467369	136905765	9	T	C	0.527	0.063	0.007	2.4E-17	trans
1017	HBEGF	Q99075	Proheparin-binding EGF-like growth factor	rs10761737	65052205	10	C	T	0.413	0.174	0.008	2.9E-117	trans
1017	HBEGF	Q99075	Proheparin-binding EGF-like growth factor	rs17490626	71218646	10	C	G	0.130	0.078	0.011	1.7E-12	trans
1017	HBEGF	Q99075	Proheparin-binding EGF-like growth factor	rs12445050	81870969	16	T	C	0.138	0.075	0.011	2.2E-12	trans
1017	HBEGF	Q99075	Proheparin-binding EGF-like growth factor	rs516051	27882705	17	A	C	0.481	0.068	0.007	2.8E-20	trans
1017	HBEGF	Q99075	Proheparin-binding EGF-like growth factor	rs1654425	55538980	19	C	T	0.834	0.150	0.010	7.9E-51	trans
1017	HBEGF	Q99075	Proheparin-binding EGF-like growth factor	rs3790176	19261922	20	A	G	0.342	0.058	0.008	1.3E-13	trans
1017	HBEGF	Q99075	Proheparin-binding EGF-like growth factor	rs116905185	49221303	20	C	T	0.011	0.302	0.036	3.4E-17	trans
1017	HBEGF	Q99075	Proheparin-binding EGF-like growth factor	rs112219723	56939389	X	T	C	0.025	0.180	0.020	7.8E-20	trans
1017	HBEGF	Q99075	Proheparin-binding EGF-like growth factor	rs181581269	64023914	X	T	C	0.033	0.186	0.018	4.5E-26	trans
1018	HBQ1	P09105	Hemoglobin subunit theta-1	rs11759553	135422296	6	A	T	0.728	0.055	0.008	2.8E-12	trans
1019	HBZ	P02008	Hemoglobin subunit zeta	rs17625587	198990494	1	A	G	0.261	0.044	0.006	1.6E-13	trans
1019	HBZ	P02008	Hemoglobin subunit zeta	rs7606173	60725451	2	G	C	0.568	0.136	0.005	3.8E-143	trans
1019	HBZ	P02008	Hemoglobin subunit zeta	rs9992667	38680186	4	T	C	0.216	0.061	0.006	5.6E-22	trans
1019	HBZ	P02008	Hemoglobin subunit zeta	rs592423	139840693	6	A	C	0.447	0.063	0.005	4.9E-33	trans
1019	HBZ	P02008	Hemoglobin subunit zeta	rs2461286	203254	16	A	G	0.351	1.031	0.008	0.0E+00	cis
1019	HBZ	P02008	Hemoglobin subunit zeta	rs9909979	27140147	17	C	A	0.202	0.060	0.007	2.1E-19	trans
1019	HBZ	P02008	Hemoglobin subunit zeta	rs56397034	13000550	19	G	C	0.609	0.060	0.005	7.3E-29	trans
1020	HCG22	E2RYF7	Protein PBMUCL2	rs2905757	31022828	6	T	C	0.323	0.397	0.008	0.0E+00	cis
1021	HCLS1	P14317	Hematopoietic lineage cell-specific protein	rs1354034	56849749	3	C	T	0.604	0.052	0.008	3.7E-12	trans
1021	HCLS1	P14317	Hematopoietic lineage cell-specific protein	rs3732410	121415720	3	C	T	0.225	0.095	0.009	1.1E-26	cis
1021	HCLS1	P14317	Hematopoietic lineage cell-specific protein	rs34436714	54327313	19	A	C	0.209	0.108	0.009	2.2E-32	trans
1022	HDAC8	Q9BY41	Histone deacetylase 8	rs28672722	32626537	6	T	G	0.222	0.081	0.009	2.7E-18	trans
1023	HDDC2	Q7Z4H3	5'-deoxynucleotidase HDDC2	rs73581685	125623247	6	C	T	0.820	0.874	0.010	0.0E+00	cis
1024	HDGF	P51858	Hepatoma-derived growth factor	rs3806417	156711623	1	A	C	0.335	1.146	0.008	0.0E+00	cis
1024	HDGF	P51858	Hepatoma-derived growth factor	rs6993770	106581528	8	A	T	0.714	0.051	0.005	1.7E-21	trans
1025	HDGFL2	Q7Z4V5	Hepatoma-derived growth factor-related protein 2	rs1354034	56849749	3	C	T	0.604	0.117	0.008	1.4E-53	trans
1025	HDGFL2	Q7Z4V5	Hepatoma-derived growth factor-related protein 2	rs6993770	106581528	8	A	T	0.713	0.059	0.008	7.9E-13	trans
1026	HEG1	Q9ULI3	Protein HEG homolog 1	rs6438868	124728472	3	G	C	0.617	0.109	0.007	6.0E-51	cis
1026	HEG1	Q9ULI3	Protein HEG homolog 1	rs9272779	32610314	6	T	C	0.596	0.062	0.007	5.4E-18	trans
1026	HEG1	Q9ULI3	Protein HEG homolog 1	rs2519093	136141870	9	C	T	0.815	0.429	0.009	0.0E+00	trans
1026	HEG1	Q9ULI3	Protein HEG homolog 1	rs964184	116648917	11	G	C	0.133	0.084	0.010	5.7E-16	trans
1026	HEG1	Q9ULI3	Protein HEG homolog 1	rs34434834	126307796	11	A	G	0.033	0.274	0.020	1.4E-43	trans
1026	HEG1	Q9ULI3	Protein HEG homolog 1	rs6602911	114547372	13	T	C	0.355	0.070	0.007	1.3E-21	trans
1026	HEG1	Q9ULI3	Protein HEG homolog 1	rs186021206	7069412	17	A	G	0.006	0.830	0.049	1.5E-65	trans
1027	HEPACAM2	A8MVW5	HEPACAM family member 2	rs304303	44178070	1	T	G	0.269	0.069	0.007	5.6E-21	trans
1027	HEPACAM2	A8MVW5	HEPACAM family member 2	rs7548219	209936466	1	A	G	0.767	0.074	0.008	8.4E-22	trans
1027	HEPACAM2	A8MVW5	HEPACAM family member 2	rs1260326	27730940	2	C	T	0.608	0.053	0.007	4.4E-15	trans
1027	HEPACAM2	A8MVW5	HEPACAM family member 2	rs4672497	62523565	2	C	G	0.778	0.209	0.008	2.8E-152	trans
1027	HEPACAM2	A8MVW5	HEPACAM family member 2	rs7560701	114026882	2	G	C	0.501	0.075	0.007	1.8E-29	trans
1027	HEPACAM2	A8MVW5	HEPACAM family member 2	rs3864106	186656602	3	G	C	0.416	0.503	0.007	0.0E+00	trans
1027	HEPACAM2	A8MVW5	HEPACAM family member 2	rs13107325	103188709	4	T	C	0.075	0.104	0.012	8.2E-17	trans
1027	HEPACAM2	A8MVW5	HEPACAM family member 2	rs2479016	2245345	6	A	G	0.573	0.055	0.007	4.3E-16	trans
1027	HEPACAM2	A8MVW5	HEPACAM family member 2	rs884366	109574095	6	G	A	0.690	0.058	0.007	2.2E-16	trans
1027	HEPACAM2	A8MVW5	HEPACAM family member 2	rs9402685	135419688	6	C	T	0.261	0.073	0.008	3.2E-22	trans
1027	HEPACAM2	A8MVW5	HEPACAM family member 2	rs592423	139840693	6	C	A	0.553	0.117	0.007	9.9E-70	trans
1027	HEPACAM2	A8MVW5	HEPACAM family member 2	rs6796	6502367	7	C	T	0.279	0.053	0.007	6.5E-13	trans
1027	HEPACAM2	A8MVW5	HEPACAM family member 2	rs6592965	50427982	7	G	A	0.546	0.133	0.007	7.7E-90	trans
1027	HEPACAM2	A8MVW5	HEPACAM family member 2	rs183332045	144698467	8	G	T	0.993	0.318	0.040	8.8E-16	trans
1027	HEPACAM2	A8MVW5	HEPACAM family member 2	rs2519093	136141870	9	C	T	0.816	0.420	0.009	0.0E+00	trans
1027	HEPACAM2	A8MVW5	HEPACAM family member 2	rs17476364	71094504	10	C	T	0.110	0.072	0.011	8.7E-12	trans
1027	HEPACAM2	A8MVW5	HEPACAM family member 2	rs67372909	80805016	10	C	T	0.241	0.081	0.008	3.6E-26	trans
1027	HEPACAM2	A8MVW5	HEPACAM family member 2	rs10891245	111167792	11	T	G	0.704	0.054	0.007	4.0E-14	trans
1027	HEPACAM2	A8MVW5	HEPACAM family member 2	rs3967200	126232385	11	C	T	0.866	0.089	0.010	2.6E-20	trans
1027	HEPACAM2	A8MVW5	HEPACAM family member 2	rs552960	54002939	15	C	T	0.493	0.048	0.007	2.9E-13	trans
1027	HEPACAM2	A8MVW5	HEPACAM family member 2	rs186021206	7069412	17	A	G	0.006	1.238	0.045	7.5E-164	trans
1027	HEPACAM2	A8MVW5	HEPACAM family member 2	rs72835417	47241642	17	A	G	0.110	0.238	0.011	5.3E-112	trans
1027	HEPACAM2	A8MVW5	HEPACAM family member 2	rs8887	4502201	19	T	C	0.426	0.081	0.007	2.7E-33	trans
1027	HEPACAM2	A8MVW5	HEPACAM family member 2	rs485186	49207206	19	G	A	0.538	0.115	0.007	4.1E-68	trans
1027	HEPACAM2	A8MVW5	HEPACAM family member 2	rs746064	42581448	21	A	G	0.380	0.232	0.007	4.3E-253	trans
1028	HEPH	Q9BQS7	Hephaestin	rs13107325	103188709	4	T	C	0.075	0.181	0.014	2.1E-38	trans
1028	HEPH	Q9BQS7	Hephaestin	rs186021206	7069412	17	A	G	0.006	0.667	0.050	7.5E-40	trans
1028	HEPH	Q9BQS7	Hephaestin	rs681343	49206462	19	C	T	0.491	0.180	0.007	9.0E-130	trans
1028	HEPH	Q9BQS7	Hephaestin	rs928873	43293371	21	C	T	0.713	0.079	0.008	3.4E-22	trans
1028	HEPH	Q9BQS7	Hephaestin	rs184082176	57691078	X	G	A	0.999	1.204	0.092	2.3E-39	trans
1028	HEPH	Q9BQS7	Hephaestin	rs2227291	77268502	X	G	C	0.803	0.100	0.008	2.2E-39	trans
1029	HEXIM1	O94992	Protein HEXIM1	rs1354034	56849749	3	C	T	0.604	0.136	0.008	2.6E-72	trans
1029	HEXIM1	O94992	Protein HEXIM1	rs114694170	88180196	5	C	T	0.060	0.124	0.016	2.7E-15	trans
1029	HEXIM1	O94992	Protein HEXIM1	rs7080386	65048306	10	A	C	0.412	0.057	0.008	2.2E-14	trans
1029	HEXIM1	O94992	Protein HEXIM1	rs11502185	180258	11	C	T	0.260	0.082	0.009	3.4E-18	trans
1029	HEXIM1	O94992	Protein HEXIM1	rs36160143	43244389	17	T	C	0.198	0.084	0.009	6.6E-19	cis
1029	HEXIM1	O94992	Protein HEXIM1	rs1671152	55526345	19	G	T	0.841	0.069	0.010	7.4E-12	trans
1030	HGF	P14210	Hepatocyte growth factor	rs59950280	3452345	4	A	G	0.330	0.139	0.008	6.5E-70	trans
1030	HGF	P14210	Hepatocyte growth factor	rs5745687	81359051	7	C	T	0.935	0.185	0.015	2.4E-36	cis
1030	HGF	P14210	Hepatocyte growth factor	rs2511241	72945341	11	T	C	0.929	0.151	0.014	1.1E-26	trans
1030	HGF	P14210	Hepatocyte growth factor	rs704	26694861	17	G	A	0.527	0.059	0.007	3.8E-16	trans
1031	HGFAC	Q04756	Hepatocyte growth factor activator	rs11127048	27752463	2	G	A	0.382	0.048	0.007	5.9E-12	trans
1031	HGFAC	Q04756	Hepatocyte growth factor activator	rs1047891	211540507	2	A	C	0.313	0.096	0.007	3.4E-40	trans
1031	HGFAC	Q04756	Hepatocyte growth factor activator	rs4690097	3445745	4	C	T	0.316	0.483	0.008	0.0E+00	cis
1031	HGFAC	Q04756	Hepatocyte growth factor activator	rs4861708	187157233	4	A	G	0.512	0.062	0.008	2.3E-16	trans
1031	HGFAC	Q04756	Hepatocyte growth factor activator	rs2731672	176842474	5	C	T	0.744	0.061	0.008	6.3E-16	trans
1031	HGFAC	Q04756	Hepatocyte growth factor activator	rs4895864	130364482	6	T	C	0.575	0.047	0.007	3.1E-12	trans
1031	HGFAC	Q04756	Hepatocyte growth factor activator	rs9987289	9183358	8	G	A	0.908	0.150	0.012	3.4E-38	trans
1031	HGFAC	Q04756	Hepatocyte growth factor activator	rs28929474	94844947	14	T	C	0.021	0.214	0.023	5.6E-20	trans
1031	HGFAC	Q04756	Hepatocyte growth factor activator	rs7212366	74593181	17	T	C	0.492	0.062	0.007	1.2E-17	trans
1032	HGS	O14964	Hepatocyte growth factor-regulated tyrosine kinase substrate	rs3811444	248039451	1	C	T	0.667	0.062	0.008	1.2E-14	trans
1032	HGS	O14964	Hepatocyte growth factor-regulated tyrosine kinase substrate	rs1354034	56849749	3	C	T	0.604	0.058	0.008	5.9E-14	trans
1032	HGS	O14964	Hepatocyte growth factor-regulated tyrosine kinase substrate	rs342293	106372219	7	C	G	0.540	0.053	0.008	3.9E-12	trans
1033	HHEX	Q03014	Hematopoietically-expressed homeobox protein HHEX	rs35457250	186338564	3	T	C	0.009	0.271	0.040	1.1E-11	trans
1033	HHEX	Q03014	Hematopoietically-expressed homeobox protein HHEX	rs6993770	106581528	8	A	T	0.713	0.060	0.008	3.9E-13	trans
1033	HHEX	Q03014	Hematopoietically-expressed homeobox protein HHEX	rs200309755	27845774	17	T	C	0.537	0.053	0.008	1.2E-11	trans
1033	HHEX	Q03014	Hematopoietically-expressed homeobox protein HHEX	rs8178824	64224775	17	T	C	0.030	0.204	0.022	2.1E-20	trans
1034	HJV	Q6ZVN8	Hemojuvelin	rs12740374	109817590	1	G	T	0.779	0.189	0.009	5.7E-95	trans
1034	HJV	Q6ZVN8	Hemojuvelin	rs41302089	145475529	1	G	T	0.984	0.611	0.031	4.9E-88	cis
1034	HJV	Q6ZVN8	Hemojuvelin	rs2954021	126482077	8	A	G	0.496	0.073	0.008	9.9E-22	trans
1034	HJV	Q6ZVN8	Hemojuvelin	rs77542162	67081278	17	G	A	0.023	0.201	0.025	1.7E-15	trans
1035	HLA-A	P04439	HLA class I histocompatibility antigen, A alpha chain	rs74439742	57059427	16	C	T	0.828	0.063	0.006	7.7E-28	trans
1036	HLA-DRA	P01903	HLA class II histocompatibility antigen, DR alpha chain	rs36096565	32560025	6	G	A	0.212	0.681	0.010	0.0E+00	cis
1037	HLA-E	P13747	HLA class I histocompatibility antigen, alpha chain E	rs2927608	96252432	5	G	A	0.551	0.188	0.007	2.0E-155	trans
1037	HLA-E	P13747	HLA class I histocompatibility antigen, alpha chain E	rs2523594	31326629	6	C	T	0.378	0.321	0.008	0.0E+00	cis
1037	HLA-E	P13747	HLA class I histocompatibility antigen, alpha chain E	rs74439742	57059427	16	C	T	0.828	0.093	0.009	7.5E-24	trans
1038	HMBS	P08397	Porphobilinogen deaminase	rs72997349	118945502	11	C	T	0.031	0.380	0.021	2.5E-73	cis
1039	HMCN2	Q8NDA2	Hemicentin-2	rs6415851	133053928	9	G	A	0.512	0.624	0.008	0.0E+00	cis
1039	HMCN2	Q8NDA2	Hemicentin-2	rs56278466	17875857	10	G	T	0.661	0.082	0.007	4.7E-32	trans
1039	HMCN2	Q8NDA2	Hemicentin-2	rs35887873	126219396	11	C	T	0.228	0.086	0.008	3.0E-28	trans
1039	HMCN2	Q8NDA2	Hemicentin-2	rs34042070	72101525	16	C	G	0.815	0.108	0.009	1.5E-36	trans
1039	HMCN2	Q8NDA2	Hemicentin-2	rs200489612	7106378	17	A	G	0.005	0.448	0.049	1.5E-19	trans
1040	HMOX1	P09601	Heme oxygenase 1	rs385076	32489851	2	C	T	0.644	0.170	0.008	3.2E-107	trans
1040	HMOX1	P09601	Heme oxygenase 1	rs6786207	69838315	3	G	T	0.350	0.078	0.008	5.1E-24	trans
1040	HMOX1	P09601	Heme oxygenase 1	rs17229943	68682536	5	C	A	0.051	0.171	0.017	1.0E-24	trans
1040	HMOX1	P09601	Heme oxygenase 1	rs13167071	156373637	5	G	C	0.358	0.057	0.008	1.3E-13	trans
1040	HMOX1	P09601	Heme oxygenase 1	rs2394941	31220273	6	T	A	0.731	0.065	0.008	5.9E-15	trans
1040	HMOX1	P09601	Heme oxygenase 1	rs2637678	116787378	6	C	T	0.409	0.073	0.007	1.4E-22	trans
1040	HMOX1	P09601	Heme oxygenase 1	rs17476364	71094504	10	C	T	0.110	0.085	0.012	6.9E-13	trans
1040	HMOX1	P09601	Heme oxygenase 1	rs7080536	115348046	10	A	G	0.044	0.356	0.018	1.8E-84	trans
1040	HMOX1	P09601	Heme oxygenase 1	rs12149545	56993161	16	A	G	0.316	0.069	0.008	1.4E-18	trans
1040	HMOX1	P09601	Heme oxygenase 1	rs34931250	66879927	17	T	C	0.060	0.122	0.015	3.3E-15	trans
1040	HMOX1	P09601	Heme oxygenase 1	rs1065853	45413233	19	T	G	0.082	0.188	0.013	3.1E-44	trans
1040	HMOX1	P09601	Heme oxygenase 1	rs34632751	48715196	19	C	T	0.027	0.324	0.023	1.7E-46	trans
1040	HMOX1	P09601	Heme oxygenase 1	rs112229561	26163326	20	C	T	0.880	0.091	0.011	1.5E-15	trans
1040	HMOX1	P09601	Heme oxygenase 1	rs113460913	30121896	20	T	TGA	0.846	0.200	0.010	2.2E-85	trans
1040	HMOX1	P09601	Heme oxygenase 1	rs2071747	35777185	22	G	C	0.942	0.155	0.016	5.9E-23	cis
1041	HMOX2	P30519	Heme oxygenase 2	rs4835265	146821410	4	A	C	0.156	0.101	0.010	4.3E-22	trans
1041	HMOX2	P30519	Heme oxygenase 2	rs4785965	4514655	16	G	A	0.265	0.121	0.009	8.3E-45	cis
1041	HMOX2	P30519	Heme oxygenase 2	rs4503880	56084054	18	T	C	0.201	0.087	0.009	5.3E-20	trans
1041	HMOX2	P30519	Heme oxygenase 2	rs429358	45411941	19	C	T	0.155	0.107	0.010	2.1E-24	trans
1042	HNMT	P50135	Histamine N-methyltransferase	rs11558538	138759649	2	C	T	0.892	1.117	0.012	0.0E+00	cis
1042	HNMT	P50135	Histamine N-methyltransferase	rs7032525	95882901	9	T	C	0.800	0.060	0.008	7.7E-14	trans
1042	HNMT	P50135	Histamine N-methyltransferase	rs10418046	54327869	19	G	T	0.211	0.060	0.008	1.5E-14	trans
1042	HNMT	P50135	Histamine N-methyltransferase	rs738409	44324727	22	G	C	0.217	0.067	0.008	2.6E-18	trans
1043	HNRNPK	P61978	Heterogeneous nuclear ribonucleoprotein K	rs6993770	106581528	8	A	T	0.713	0.066	0.008	3.5E-15	trans
1044	HNRNPUL1	Q9BUJ2	Heterogeneous nuclear ribonucleoprotein U-like protein 1	rs1354034	56849749	3	C	T	0.604	0.123	0.007	1.5E-60	trans

1044	HNRNPUL1	Q9BUJ2	Heterogeneous nuclear ribonucleoprotein U-like protein 1	rs11604127	196944	11	T	C	0.231	0.075	0.009	1.2E-17	trans
1045	HPCAL1	P37235	Hippocalcin-like protein 1	rs375285744	10476228	2	C	T	0.149	0.219	0.011	9.7E-95	cis
1046	HPGDS	O60760	Hematopoietic prostaglandin D synthase	rs2865353	95270878	4	C	T	0.612	0.463	0.008	0.0E+00	cis
1046	HPGDS	O60760	Hematopoietic prostaglandin D synthase	rs2398827	95907107	9	C	T	0.602	0.066	0.007	1.1E-19	trans
1047	HPSE	Q9Y251	Heparanase	rs7618405	18250509	3	C	A	0.795	0.074	0.009	3.6E-15	trans
1047	HPSE	Q9Y251	Heparanase	rs61751211	84223426	4	T	C	0.989	0.805	0.040	1.8E-88	cis
1047	HPSE	Q9Y251	Heparanase	rs3734208	46097758	6	C	G	0.357	0.073	0.008	3.1E-20	trans
1047	HPSE	Q9Y251	Heparanase	rs6961069	80218961	7	T	C	0.402	0.058	0.008	7.4E-14	trans
1047	HPSE	Q9Y251	Heparanase	rs6993770	106581528	8	A	T	0.713	0.158	0.008	3.0E-79	trans
1047	HPSE	Q9Y251	Heparanase	rs15951	5075248	16	G	A	0.466	0.058	0.008	2.2E-14	trans
1047	HPSE	Q9Y251	Heparanase	rs892090	55539072	19	G	T	0.834	0.124	0.010	3.8E-34	trans
1047	HPSE	Q9Y251	Heparanase	rs3827978	19281291	20	T	C	0.353	0.065	0.008	1.5E-16	trans
1048	HRC	P23327	Sarcoplasmic reticulum histidine-rich calcium-binding protein	rs1260326	27730940	2	C	T	0.608	0.065	0.008	1.1E-16	trans
1048	HRC	P23327	Sarcoplasmic reticulum histidine-rich calcium-binding protein	rs9853056	52555957	3	C	T	0.559	0.086	0.008	5.5E-29	trans
1048	HRC	P23327	Sarcoplasmic reticulum histidine-rich calcium-binding protein	rs163192	68379836	5	A	C	0.464	0.084	0.008	5.5E-28	trans
1048	HRC	P23327	Sarcoplasmic reticulum histidine-rich calcium-binding protein	rs28361032	31973016	6	A	G	0.610	0.057	0.008	8.9E-12	trans
1048	HRC	P23327	Sarcoplasmic reticulum histidine-rich calcium-binding protein	rs71475909	47377283	11	G	A	0.357	0.057	0.008	9.0E-13	trans
1048	HRC	P23327	Sarcoplasmic reticulum histidine-rich calcium-binding protein	rs148966785	49658233	19	G	A	0.992	0.680	0.042	4.7E-59	cis
1049	HRG	P04196	Histidine-rich glycoprotein	rs12406245	93860625	1	A	G	0.632	0.058	0.007	1.6E-15	trans
1049	HRG	P04196	Histidine-rich glycoprotein	rs1260326	27730940	2	T	C	0.392	0.051	0.007	1.7E-12	trans
1049	HRG	P04196	Histidine-rich glycoprotein	rs9898	186390627	3	T	C	0.329	0.501	0.008	0.0E+00	cis
1049	HRG	P04196	Histidine-rich glycoprotein	rs59950280	3452345	4	A	G	0.330	0.052	0.008	4.5E-12	trans
1049	HRG	P04196	Histidine-rich glycoprotein	rs4934470	91067438	10	T	C	0.274	0.119	0.008	1.1E-51	trans
1050	HS1BP3	Q53T59	HCLS1-binding protein 3	rs77551133	20840823	2	G	A	0.993	0.866	0.047	6.5E-77	cis
1050	HS1BP3	Q53T59	HCLS1-binding protein 3	rs11502185	180258	11	C	T	0.260	0.073	0.010	4.5E-14	trans
1051	HS3ST3B1	Q9Y662	Heparan sulfate glucosamine 3-O-sulfotransferase 3B1	rs4970836	109821797	1	A	G	0.773	0.074	0.009	1.7E-17	trans
1051	HS3ST3B1	Q9Y662	Heparan sulfate glucosamine 3-O-sulfotransferase 3B1	rs4846914	230295691	1	A	G	0.606	0.140	0.007	6.6E-80	trans
1051	HS3ST3B1	Q9Y662	Heparan sulfate glucosamine 3-O-sulfotransferase 3B1	rs11096641	20374158	2	G	A	0.529	0.057	0.007	4.8E-15	trans
1051	HS3ST3B1	Q9Y662	Heparan sulfate glucosamine 3-O-sulfotransferase 3B1	rs5030062	186454180	3	A	C	0.627	0.064	0.007	1.2E-17	trans
1051	HS3ST3B1	Q9Y662	Heparan sulfate glucosamine 3-O-sulfotransferase 3B1	rs13107325	103188709	4	C	T	0.925	0.107	0.014	9.5E-15	trans
1051	HS3ST3B1	Q9Y662	Heparan sulfate glucosamine 3-O-sulfotransferase 3B1	rs4241819	187157140	4	C	T	0.491	0.092	0.007	3.3E-37	trans
1051	HS3ST3B1	Q9Y662	Heparan sulfate glucosamine 3-O-sulfotransferase 3B1	rs1801020	176836532	5	A	G	0.255	0.064	0.008	1.6E-14	trans
1051	HS3ST3B1	Q9Y662	Heparan sulfate glucosamine 3-O-sulfotransferase 3B1	rs28929474	94844947	14	T	C	0.021	0.229	0.025	1.8E-19	trans
1051	HS3ST3B1	Q9Y662	Heparan sulfate glucosamine 3-O-sulfotransferase 3B1	rs9302635	72144174	16	C	T	0.182	0.070	0.009	5.7E-14	trans
1051	HS3ST3B1	Q9Y662	Heparan sulfate glucosamine 3-O-sulfotransferase 3B1	rs17669311	13837051	17	A	G	0.392	0.329	0.008	0.0E+00	cis
1052	HS6ST1	O60243	Heparan-sulfate 6-O-sulfotransferase 1	rs12740374	109817590	1	G	T	0.779	0.075	0.009	1.5E-17	trans
1052	HS6ST1	O60243	Heparan-sulfate 6-O-sulfotransferase 1	rs907866	20371380	2	G	A	0.552	0.076	0.007	2.5E-24	trans
1052	HS6ST1	O60243	Heparan-sulfate 6-O-sulfotransferase 1	rs6761320	129050354	2	C	T	0.572	0.210	0.008	4.9E-172	cis
1052	HS6ST1	O60243	Heparan-sulfate 6-O-sulfotransferase 1	rs17282078	124481760	3	T	A	0.870	0.096	0.011	6.7E-19	trans
1052	HS6ST1	O60243	Heparan-sulfate 6-O-sulfotransferase 1	rs13107325	103188709	4	T	C	0.075	0.170	0.014	1.3E-34	trans
1052	HS6ST1	O60243	Heparan-sulfate 6-O-sulfotransferase 1	rs1290147	149919739	5	G	C	0.352	0.052	0.008	8.1E-12	trans
1052	HS6ST1	O60243	Heparan-sulfate 6-O-sulfotransferase 1	rs573098	31841890	6	T	A	0.675	0.064	0.008	3.8E-16	trans
1052	HS6ST1	O60243	Heparan-sulfate 6-O-sulfotransferase 1	rs33951980	73029437	7	C	T	0.871	0.078	0.011	5.1E-13	trans
1052	HS6ST1	O60243	Heparan-sulfate 6-O-sulfotransferase 1	rs6952090	129922498	7	T	C	0.652	0.061	0.008	1.6E-15	trans
1052	HS6ST1	O60243	Heparan-sulfate 6-O-sulfotransferase 1	rs56278466	17875857	10	G	T	0.661	0.068	0.008	9.6E-19	trans
1052	HS6ST1	O60243	Heparan-sulfate 6-O-sulfotransferase 1	rs1149597	76471616	11	C	T	0.831	0.079	0.010	3.4E-16	trans
1052	HS6ST1	O60243	Heparan-sulfate 6-O-sulfotransferase 1	rs12903915	51028748	15	T	C	0.659	0.064	0.008	6.1E-17	trans
1052	HS6ST1	O60243	Heparan-sulfate 6-O-sulfotransferase 1	rs72802342	75234872	16	C	A	0.924	0.190	0.014	4.0E-42	trans
1052	HS6ST1	O60243	Heparan-sulfate 6-O-sulfotransferase 1	rs704	26694861	17	G	A	0.527	0.152	0.007	2.6E-96	trans
1052	HS6ST1	O60243	Heparan-sulfate 6-O-sulfotransferase 1	rs77542162	67081278	17	G	A	0.023	0.296	0.024	7.4E-34	trans
1053	HS6ST2	Q96MM7	Heparan-sulfate 6-O-sulfotransferase 2	rs2187831	131869906	X	T	C	0.501	0.209	0.006	3.4E-255	cis
1054	HSBP1	O75506	Heat shock factor-binding protein 1	rs12038396	156459535	1	T	C	0.653	0.062	0.007	2.6E-17	trans
1054	HSBP1	O75506	Heat shock factor-binding protein 1	rs3811444	248039451	1	C	T	0.667	0.078	0.007	2.7E-26	trans
1054	HSBP1	O75506	Heat shock factor-binding protein 1	rs72799293	54222053	2	A	G	0.024	0.278	0.023	3.4E-34	trans
1054	HSBP1	O75506	Heat shock factor-binding protein 1	rs847803	64982415	2	A	G	0.324	0.056	0.007	3.2E-14	trans
1054	HSBP1	O75506	Heat shock factor-binding protein 1	rs13084580	39188182	3	C	T	0.886	0.148	0.011	1.2E-41	trans
1054	HSBP1	O75506	Heat shock factor-binding protein 1	rs1354034	56849749	3	C	T	0.605	0.056	0.007	1.8E-15	trans
1054	HSBP1	O75506	Heat shock factor-binding protein 1	rs114694170	88180196	5	T	C	0.940	0.127	0.015	9.1E-18	trans
1054	HSBP1	O75506	Heat shock factor-binding protein 1	rs6938130	32831247	6	A	T	0.592	0.048	0.007	1.1E-11	trans
1054	HSBP1	O75506	Heat shock factor-binding protein 1	rs342293	106372219	7	C	G	0.540	0.086	0.007	3.4E-35	trans
1054	HSBP1	O75506	Heat shock factor-binding protein 1	rs7832219	106578977	8	C	T	0.274	0.056	0.008	5.9E-13	trans
1054	HSBP1	O75506	Heat shock factor-binding protein 1	rs10820606	99192919	9	C	A	0.229	0.176	0.008	5.0E-97	trans
1054	HSBP1	O75506	Heat shock factor-binding protein 1	rs707236	83803160	16	T	A	0.655	0.568	0.008	0.0E+00	cis
1054	HSBP1	O75506	Heat shock factor-binding protein 1	rs9951002	73426923	18	A	T	0.233	0.075	0.008	9.5E-20	trans
1055	HSD11B1	P28845	Corticosteroid 11-beta-dehydrogenase isozyme 1	rs78444298	184672098	1	A	G	0.019	0.249	0.027	7.5E-20	trans
1055	HSD11B1	P28845	Corticosteroid 11-beta-dehydrogenase isozyme 1	rs7542235	196823613	1	G	A	0.195	0.156	0.009	5.3E-62	trans
1055	HSD11B1	P28845	Corticosteroid 11-beta-dehydrogenase isozyme 1	rs4240624	9184231	8	A	G	0.908	0.169	0.013	2.5E-39	trans
1055	HSD11B1	P28845	Corticosteroid 11-beta-dehydrogenase isozyme 1	rs174576	61603510	11	A	C	0.352	0.075	0.008	5.8E-22	trans
1055	HSD11B1	P28845	Corticosteroid 11-beta-dehydrogenase isozyme 1	rs695110	75456581	11	C	T	0.154	0.078	0.010	4.7E-14	trans
1055	HSD11B1	P28845	Corticosteroid 11-beta-dehydrogenase isozyme 1	rs1076485	116772441	11	T	C	0.124	0.109	0.011	9.5E-22	trans
1055	HSD11B1	P28845	Corticosteroid 11-beta-dehydrogenase isozyme 1	rs28929474	94844947	14	T	C	0.021	0.656	0.026	2.8E-137	trans
1055	HSD11B1	P28845	Corticosteroid 11-beta-dehydrogenase isozyme 1	rs56156922	56987369	16	C	T	0.325	0.054	0.008	1.4E-11	trans
1055	HSD11B1	P28845	Corticosteroid 11-beta-dehydrogenase isozyme 1	rs1801689	64210580	17	C	A	0.030	0.444	0.022	8.8E-92	trans
1055	HSD11B1	P28845	Corticosteroid 11-beta-dehydrogenase isozyme 1	rs58542926	19379549	19	T	C	0.075	0.228	0.014	5.7E-58	trans
1055	HSD11B1	P28845	Corticosteroid 11-beta-dehydrogenase isozyme 1	rs141887102	59077031	19	C	A	0.183	0.087	0.010	3.0E-19	trans
1056	HSD17B14	Q9BPX1	17-beta-hydroxysteroid dehydrogenase 14	rs55730499	161005610	6	T	C	0.082	0.086	0.011	7.5E-16	trans
1056	HSD17B14	Q9BPX1	17-beta-hydroxysteroid dehydrogenase 14	rs112875651	126506694	8	G	A	0.609	0.061	0.006	8.4E-24	trans
1056	HSD17B14	Q9BPX1	17-beta-hydroxysteroid dehydrogenase 14	rs35299026	49318380	19	G	A	0.953	1.523	0.018	0.0E+00	cis
1057	HSDL2	Q6YN16	Hydroxysteroid dehydrogenase-like protein 2	rs62180180	199849973	2	C	T	0.695	0.074	0.007	1.8E-23	trans
1057	HSDL2	Q6YN16	Hydroxysteroid dehydrogenase-like protein 2	rs10817342	115199507	9	T	C	0.380	0.601	0.008	0.0E+00	cis
1058	HSPA1A	P0DMV8	Heat shock 70 kDa protein 1A	rs1354034	56849749	3	C	T	0.604	0.052	0.008	7.6E-12	trans
1058	HSPA1A	P0DMV8	Heat shock 70 kDa protein 1A	rs181412562	31817577	6	C	T	0.998	1.266	0.089	3.5E-46	cis
1058	HSPA1A	P0DMV8	Heat shock 70 kDa protein 1A	rs342293	106372219	7	C	G	0.541	0.056	0.007	4.0E-14	trans
1058	HSPA1A	P0DMV8	Heat shock 70 kDa protein 1A	rs11218968	122958478	11	A	G	0.958	0.147	0.019	2.4E-15	trans
1059	HSPA2	P54652	Heat shock-related 70 kDa protein 2	rs10498515	65045056	14	T	C	0.799	0.127	0.009	3.6E-42	cis
1060	HSPB1	P04792	Heat shock protein beta-1	rs1354034	56849749	3	C	T	0.604	0.052	0.007	6.6E-13	trans
1060	HSPB1	P04792	Heat shock protein beta-1	rs6926219	122720806	6	G	A	0.454	0.092	0.007	1.0E-37	trans
1060	HSPB1	P04792	Heat shock protein beta-1	rs342298	106373646	7	C	T	0.545	0.077	0.008	3.8E-24	trans
1060	HSPB1	P04792	Heat shock protein beta-1	rs7896518	65104500	10	G	A	0.424	0.078	0.007	2.3E-26	trans
1061	HSPB6	O14558	Heat shock protein beta-6	rs12463674	179432185	2	G	A	0.315	0.058	0.007	4.7E-16	trans
1061	HSPB6	O14558	Heat shock protein beta-6	rs12331051	187139807	4	T	C	0.428	0.059	0.007	4.7E-18	trans
1061	HSPB6	O14558	Heat shock protein beta-6	rs151828	96060262	5	C	T	0.547	0.048	0.007	2.0E-13	trans
1061	HSPB6	O14558	Heat shock protein beta-6	rs13437444	28070998	6	T	C	0.215	0.062	0.008	3.8E-15	trans
1061	HSPB6	O14558	Heat shock protein beta-6	rs2234962	121429633	10	C	T	0.216	0.058	0.008	4.0E-13	trans
1061	HSPB6	O14558	Heat shock protein beta-6	rs1337298	74130675	13	C	T	0.561	0.050	0.007	3.5E-14	trans
1062	HSPG2	P98160	Basement membrane-specific heparan sulfate proteoglycan core protein	rs6684152	22147764	1	A	G	0.706	0.175	0.008	2.1E-103	cis
1062	HSPG2	P98160	Basement membrane-specific heparan sulfate proteoglycan core protein	rs13146355	77412140	4	A	G	0.456	0.050	0.007	6.1E-12	trans
1062	HSPG2	P98160	Basement membrane-specific heparan sulfate proteoglycan core protein	rs77924615	20392332	16	G	A	0.803	0.078	0.009	5.4E-17	trans
1063	HTRA2	O43464	Serine protease HTRA2, mitochondrial	rs6993770	106581528	8	A	T	0.714	0.062	0.008	8.3E-14	trans
1064	HYAL1	Q12794	Hyaluronidase-1	rs12371	180163390	1	A	G	0.912	0.090	0.013	7.0E-12	trans
1064	HYAL1	Q12794	Hyaluronidase-1	rs116482870	50339622	3	C	T	0.938	0.549	0.016	4.0E-274	cis
1065	HYOU1	Q9Y4L1	Hypoxia up-regulated protein 1	rs7532110	47961964	1	A	T	0.803	0.090	0.009	6.8E-22	trans
1065	HYOU1	Q9Y4L1	Hypoxia up-regulated protein 1	rs61747728	179526214	1	T	C	0.038	0.226	0.019	4.5E-32	trans
1065	HYOU1	Q9Y4L1	Hypoxia up-regulated protein 1	rs75166367	162964301	2	G	A	0.940	0.137	0.016	1.3E-18	trans
1065	HYOU1	Q9Y4L1	Hypoxia up-regulated protein 1	rs149547076	57557444	3	T	C	0.989	0.330	0.036	1.4E-19	trans
1065	HYOU1	Q9Y4L1	Hypoxia up-regulated protein 1	rs17715183	127195197	7	G	C	0.933	0.111	0.015	1.1E-13	trans
1065	HYOU1	Q9Y4L1	Hypoxia up-regulated protein 1	rs2509121	118928253	11	T	C	0.401	0.183	0.008	8.4E-129	cis
1065	HYOU1	Q9Y4L1	Hypoxia up-regulated protein 1	rs28929474	94844947	14	T	C	0.021	0.302	0.026	1.6E-31	trans
1065	HYOU1	Q9Y4L1	Hypoxia up-regulated protein 1	rs11636578	101787969	15	C	T	0.324	0.060	0.008	4.0E-14	trans
1065	HYOU1	Q9Y4L1	Hypoxia up-regulated protein 1	rs1801689	64210580	17	C	A	0.030	0.288	0.022	1.2E-40	trans
1065	HYOU1	Q9Y4L1	Hypoxia up-regulated protein 1	rs2659005	79218714	17	T	C	0.441	0.082	0.007	1.7E-28	trans
1065	HYOU1	Q9Y4L1	Hypoxia up-regulated protein 1	rs33950747	36339247	19	T	C	0.075	0.111	0.014	2.1E-15	trans
1065	HYOU1	Q9Y4L1	Hypoxia up-regulated protein 1	rs10419198	50038017	19	T	C	0.250	0.059	0.009	3.4E-12	trans
1065	HYOU1	Q9Y4L1	Hypoxia up-regulated protein 1	rs136205	36754203	22	C	G	0.252	0.061	0.009	1.5E-12	trans
1065	HYOU1	Q9Y4L1	Hypoxia up-regulated protein 1	rs519725	112103635	X	G	A	0.782	0.064	0.007	4.8E-18	trans
1066	ICA1	Q05084	Islet cell autoantigen 1	rs1354034	56849749	3	C	T	0.604	0.053	0.008	3.1E-12	trans
1066	ICA1	Q05084	Islet cell autoantigen 1	rs3952	38456598	22	A	G	0.686	0.218	0.008	2.1E-158	trans
1067	ICAM1	P05362	Intercellular adhesion molecule 1	rs1049728	65421117	11	G	C	0.941	0.167	0.015	2.8E-29	trans
1067	ICAM1	P05362	Intercellular adhesion molecule 1	rs34434834	126307796	11	A	G	0.033	0.148	0.019	2.3E-14	trans
1067	ICAM1	P05362	Intercellular adhesion molecule 1	rs186021206	7069412	17	A	G	0.006	0.636	0.047	6.0E-41	trans
1067	ICAM1	P05362	Intercellular adhesion molecule 1	rs5030377	10393234	19	G	A	0.378	0.415	0.008	0.0E+00	cis
1067	ICAM1	P05362	Intercellular adhesion molecule 1	rs3136642	39398416	19	A	G	0.633	0.056	0.008	5.8E-13	trans
1068	ICAM2	P13598	Intercellular adhesion molecule 2	rs11682865	62617796	2	G	C	0.805	0.051	0.007	4.5E-13	trans
1068	ICAM2	P13598	Intercellular adhesion molecule 2	rs10935473	98416900	3	G	T	0.560	0.717	0.007	0.0E+00	trans
1068	ICAM2	P13598	Intercellular adhesion molecule 2	rs9399137	135419018	6	C	T	0.258	0.057	0.006	7.1E-19	trans
1068	ICAM2	P13598	Intercellular adhesion molecule 2	rs6993770	106581528	8	A	T	0.713	0.064	0.006	5.6E-25	trans
1068	ICAM2	P13598	Intercellular adhesion molecule 2	rs2519093	136141870	9	C	T	0.816	0.859	0.009	0.0E+00	trans
1068	ICAM2	P13598	Intercellular adhesion molecule 2	rs78689694	126234820	11	G	C	0.867	0.083	0.008	9.4E-24	trans
1068	ICAM2	P13598	Intercellular adhesion molecule 2	rs186021206	7069412	17	A	G	0.006	1.269	0.038	1.2E-238	trans
1068	ICAM2	P13598	Intercellular adhesion molecule 2	rs368565	49201217	19	C	T	0.568	0.044	0.006	8.2E-15	trans
1068	ICAM2	P13598	Intercellular adhesion molecule 2	rs2837988	42619544	21	A	C	0.362	0.049	0.006	5.8E-17	trans
1069	ICAM3	P32942	Intercellular adhesion molecule 3	rs188468174	25291697	1	C	T	0.986	0.372	0.030	1.2E-34	trans
1069	ICAM3	P32942	Intercellular adhesion molecule 3	rs2289235	231738168	2	G	A	0.531	0.149	0.007	1.8E-98	trans
1069	ICAM3	P32942	Intercellular adhesion molecule 3	rs6764279	186723975	3	C	A	0.723	0.088	0.008	2.4E-28	trans
1069	ICAM3	P32942	Intercellular adhesion molecule 3	rs2524078	31242649	6	A	G	0.826	0.195	0.010	6.1E-92	trans
1069	ICAM3	P32942	Intercellular adhesion molecule 3	rs7758383	143169723	6	G	A	0.487	0.083	0.007	8.0E-31	trans
1069	ICAM3	P32942	Intercellular adhesion molecule 3	rs2278467	24188844	8	T	C	0.845	0.216	0.010	2.9E-106	trans
1069	ICAM3	P32942	Intercellular adhesion molecule 3	rs12574844	126273386	11	G	A	0.929	0.156	0.014	2.3E-29	trans
1069	ICAM3	P32942	Intercellular adhesion molecule 3	rs76428106	28604007	13	C	T	0.013	0.304	0.033	1.7E-20	trans
1069	ICAM3	P32942	Intercellular adhesion molecule 3	rs9316367	48810789	13	C	T	0.749	0.113	0.008	3.1E-43	trans
1069	ICAM3	P32942	Intercellular adhesion molecule 3	rs186021206	7069412	17	A	G	0.006	1.198	0.049	2.0E-131	trans
1069	ICAM3	P32942	Intercellular adhesion molecule 3	rs34562254	16842991	17	A	G	0.097	0.088	0.012	2.2E-13	trans
1069	ICAM3	P32942	Intercellular adhesion molecule 3	rs2305482	38140927	17	A	C	0.460	0.062	0.007	4.5E-18	trans

1069	ICAM3	P32942	Intercellular adhesion molecule 3	rs56378716	56356502	17	G	A	0.013	0.298	0.031	7.0E-22	trans
1069	ICAM3	P32942	Intercellular adhesion molecule 3	rs2725405	79220224	17	C	G	0.441	0.097	0.007	1.5E-41	trans
1069	ICAM3	P32942	Intercellular adhesion molecule 3	rs28378712	10457472	19	G	T	0.313	0.318	0.008	0.0E+00	cis
1069	ICAM3	P32942	Intercellular adhesion molecule 3	rs602662	49206985	19	A	G	0.536	0.053	0.007	4.4E-13	trans
1069	ICAM3	P32942	Intercellular adhesion molecule 3	rs6517655	42536246	21	C	G	0.273	0.063	0.008	3.6E-15	trans
1070	ICAM4	Q14773	Intercellular adhesion molecule 4	rs11576522	207789269	1	G	A	0.655	0.076	0.008	9.7E-23	trans
1070	ICAM4	Q14773	Intercellular adhesion molecule 4	rs7606173	60725451	2	G	C	0.568	0.065	0.006	3.4E-24	trans
1070	ICAM4	Q14773	Intercellular adhesion molecule 4	rs13103731	145056487	4	T	C	0.449	0.215	0.006	3.0E-240	trans
1070	ICAM4	Q14773	Intercellular adhesion molecule 4	rs2523990	30077229	6	A	G	0.460	0.053	0.006	1.3E-16	trans
1070	ICAM4	Q14773	Intercellular adhesion molecule 4	rs62427982	107437166	6	T	C	0.325	0.057	0.007	1.2E-16	trans
1070	ICAM4	Q14773	Intercellular adhesion molecule 4	rs9376091	135419636	6	C	T	0.741	0.095	0.007	3.2E-38	trans
1070	ICAM4	Q14773	Intercellular adhesion molecule 4	rs6592965	50427982	7	G	A	0.546	0.057	0.006	1.1E-18	trans
1070	ICAM4	Q14773	Intercellular adhesion molecule 4	rs1595608	80801407	10	G	T	0.241	0.054	0.007	7.1E-13	trans
1070	ICAM4	Q14773	Intercellular adhesion molecule 4	rs11627485	65487694	14	C	T	0.449	0.048	0.006	6.5E-14	trans
1070	ICAM4	Q14773	Intercellular adhesion molecule 4	rs17580	94847262	14	A	T	0.050	0.159	0.015	1.7E-27	trans
1070	ICAM4	Q14773	Intercellular adhesion molecule 4	rs186021206	7069412	17	A	G	0.006	0.515	0.044	1.0E-31	trans
1070	ICAM4	Q14773	Intercellular adhesion molecule 4	rs73425119	43753831	18	C	G	0.260	0.072	0.007	6.0E-23	trans
1070	ICAM4	Q14773	Intercellular adhesion molecule 4	rs5030377	10393234	19	A	G	0.622	0.115	0.007	1.3E-67	cis
1070	ICAM4	Q14773	Intercellular adhesion molecule 4	rs746064	42581448	21	A	G	0.380	0.094	0.007	4.3E-46	trans
1071	ICAM5	Q9UMF0	Intercellular adhesion molecule 5	rs3864106	186656602	3	G	C	0.416	0.045	0.006	1.0E-13	trans
1071	ICAM5	Q9UMF0	Intercellular adhesion molecule 5	rs2049865	116588546	8	A	C	0.578	0.048	0.006	2.4E-15	trans
1071	ICAM5	Q9UMF0	Intercellular adhesion molecule 5	rs2519093	136141870	9	C	T	0.816	0.408	0.008	0.0E+00	trans
1071	ICAM5	Q9UMF0	Intercellular adhesion molecule 5	rs186021206	7069412	17	A	G	0.006	0.590	0.041	2.5E-46	trans
1071	ICAM5	Q9UMF0	Intercellular adhesion molecule 5	rs77542162	67081278	17	G	A	0.023	0.523	0.020	5.2E-147	trans
1071	ICAM5	Q9UMF0	Intercellular adhesion molecule 5	rs281439	10400110	19	C	G	0.780	0.844	0.009	0.0E+00	cis
1072	ICOSLG	O75144	ICOS ligand	rs74152937	101805621	10	A	G	0.046	0.122	0.017	1.4E-12	trans
1072	ICOSLG	O75144	ICOS ligand	rs186021206	7069412	17	A	G	0.006	0.782	0.050	2.0E-55	trans
1072	ICOSLG	O75144	ICOS ligand	rs6518352	45646698	21	A	G	0.279	0.402	0.009	0.0E+00	cis
1073	IDI2	Q9BXS1	Isopentenyl-diphosphate delta-isomerase 2	rs1044261	1065710	10	C	T	0.922	0.616	0.014	0.0E+00	cis
1074	IDO1	P14902	Indoleamine 2,3-dioxygenase 1	rs2637681	116769845	6	G	T	0.413	0.073	0.007	1.2E-22	trans
1074	IDO1	P14902	Indoleamine 2,3-dioxygenase 1	rs12342201	95894964	9	G	A	0.523	0.058	0.007	4.6E-15	trans
1074	IDO1	P14902	Indoleamine 2,3-dioxygenase 1	rs13215699	32601153	6	A	G	0.185	0.078	0.010	3.9E-15	trans
1074	IDO1	P14902	Indoleamine 2,3-dioxygenase 1	rs7010461	39781444	8	T	C	0.334	0.276	0.008	7.3E-260	cis
1074	IDO1	P14902	Indoleamine 2,3-dioxygenase 1	rs11993233	145002283	8	A	G	0.574	0.054	0.007	5.3E-13	trans
1074	IDO1	P14902	Indoleamine 2,3-dioxygenase 1	rs12363646	128067342	11	T	C	0.709	0.060	0.008	7.9E-14	trans
1074	IDO1	P14902	Indoleamine 2,3-dioxygenase 1	rs4632248	54324995	19	T	G	0.210	0.082	0.009	4.2E-20	trans
1075	IDS	P22304	Iduronate 2-sulfatase	rs704	26694861	17	G	A	0.527	0.087	0.007	2.4E-31	trans
1075	IDS	P22304	Iduronate 2-sulfatase	rs5936292	148560698	X	G	A	0.850	0.071	0.009	1.7E-16	cis
1076	IDUA	P35475	Alpha-L-iduronidase	rs687339	135932359	3	T	C	0.771	0.058	0.008	4.7E-14	trans
1076	IDUA	P35475	Alpha-L-iduronidase	rs3796622	983060	4	C	T	0.640	0.543	0.008	0.0E+00	cis
1076	IDUA	P35475	Alpha-L-iduronidase	rs114816312	110638824	4	T	C	0.008	0.288	0.042	1.3E-11	trans
1076	IDUA	P35475	Alpha-L-iduronidase	rs2389688	119736796	4	A	C	0.558	0.052	0.008	4.3E-12	trans
1076	IDUA	P35475	Alpha-L-iduronidase	rs28929474	94844947	14	T	C	0.021	0.261	0.023	1.4E-30	trans
1076	IDUA	P35475	Alpha-L-iduronidase	rs147233090	44028047	15	T	C	0.024	0.208	0.021	3.7E-22	trans
1076	IDUA	P35475	Alpha-L-iduronidase	rs58542926	19379549	19	T	C	0.075	0.115	0.012	8.8E-21	trans
1076	IDUA	P35475	Alpha-L-iduronidase	rs429358	45411941	19	T	C	0.844	0.077	0.009	1.3E-17	trans
1076	IDUA	P35475	Alpha-L-iduronidase	rs1883711	39179822	20	C	G	0.031	0.131	0.019	6.6E-12	trans
1077	IFI30	P13284	Gamma-interferon-inducible lysosomal thiol reductase	rs71640036	187161120	4	T	G	0.506	0.050	0.007	1.8E-12	trans
1077	IFI30	P13284	Gamma-interferon-inducible lysosomal thiol reductase	rs2394172	29756800	6	T	C	0.066	0.111	0.014	2.0E-15	trans
1077	IFI30	P13284	Gamma-interferon-inducible lysosomal thiol reductase	rs7259445	18283011	19	A	C	0.782	0.507	0.009	0.0E+00	cis
1078	IFIT3	O14879	Interferon-induced protein with tetratricopeptide repeats 3	rs6753170	58481863	2	C	T	0.628	0.070	0.008	1.2E-18	trans
1078	IFIT3	O14879	Interferon-induced protein with tetratricopeptide repeats 3	rs76036957	113496453	2	A	G	0.009	0.382	0.040	9.1E-22	trans
1078	IFIT3	O14879	Interferon-induced protein with tetratricopeptide repeats 3	rs55792153	138854203	5	C	A	0.744	0.200	0.009	1.7E-114	trans
1078	IFIT3	O14879	Interferon-induced protein with tetratricopeptide repeats 3	rs610913	74155346	6	G	T	0.382	0.084	0.008	6.2E-27	trans
1078	IFIT3	O14879	Interferon-induced protein with tetratricopeptide repeats 3	rs2274878	91102098	10	C	G	0.754	0.108	0.009	5.2E-35	cis
1079	IFNAR1	P17181	Interferon alpha/beta receptor 1	rs186021206	7069412	17	A	G	0.006	0.515	0.049	3.8E-26	trans
1079	IFNAR1	P17181	Interferon alpha/beta receptor 1	rs12720356	10469975	19	A	C	0.901	0.211	0.012	4.7E-70	trans
1079	IFNAR1	P17181	Interferon alpha/beta receptor 1	rs914142	34725807	21	G	A	0.271	0.450	0.008	0.0E+00	cis
1080	IFNG	P01579	Interferon gamma	rs76830965	159637678	3	A	C	0.118	0.083	0.012	3.0E-12	trans
1080	IFNG	P01579	Interferon gamma	rs9264259	31223685	6	C	T	0.713	0.064	0.009	1.9E-12	trans
1081	IFNGR1	P15260	Interferon gamma receptor 1	rs61747728	179526214	1	T	C	0.038	0.195	0.019	1.5E-24	trans
1081	IFNGR1	P15260	Interferon gamma receptor 1	rs1260326	27730940	2	C	T	0.609	0.055	0.007	2.6E-13	trans
1081	IFNGR1	P15260	Interferon gamma receptor 1	rs73065135	46888509	3	C	G	0.099	0.087	0.012	1.7E-12	trans
1081	IFNGR1	P15260	Interferon gamma receptor 1	rs9853056	52555957	3	C	T	0.559	0.050	0.007	9.5E-12	trans
1081	IFNGR1	P15260	Interferon gamma receptor 1	rs2394177	29787369	6	G	A	0.075	0.125	0.014	3.9E-19	trans
1081	IFNGR1	P15260	Interferon gamma receptor 1	rs9376267	137531031	6	C	T	0.763	0.202	0.009	6.3E-122	cis
1081	IFNGR1	P15260	Interferon gamma receptor 1	rs8176672	136142185	9	T	C	0.061	0.360	0.015	4.4E-123	trans
1081	IFNGR1	P15260	Interferon gamma receptor 1	rs186021206	7069412	17	A	G	0.006	0.848	0.050	3.8E-64	trans
1081	IFNGR1	P15260	Interferon gamma receptor 1	rs2659005	79218714	17	T	C	0.441	0.055	0.007	5.8E-14	trans
1082	IFNGR2	P38484	Interferon gamma receptor 2	rs12740374	109817590	1	G	T	0.779	0.075	0.006	7.1E-31	trans
1082	IFNGR2	P38484	Interferon gamma receptor 2	rs10901252	136128000	9	C	G	0.061	0.127	0.011	2.9E-29	trans
1082	IFNGR2	P38484	Interferon gamma receptor 2	rs56278466	17875857	10	G	T	0.661	0.072	0.006	1.4E-36	trans
1082	IFNGR2	P38484	Interferon gamma receptor 2	rs174564	61588305	11	G	A	0.349	0.095	0.006	7.5E-64	trans
1082	IFNGR2	P38484	Interferon gamma receptor 2	rs12149545	56993161	16	A	G	0.316	0.055	0.006	2.7E-21	trans
1082	IFNGR2	P38484	Interferon gamma receptor 2	rs72802342	75234872	16	C	A	0.924	0.071	0.010	1.0E-11	trans
1082	IFNGR2	P38484	Interferon gamma receptor 2	rs1065853	45413233	19	T	G	0.081	0.106	0.010	1.5E-26	trans
1082	IFNGR2	P38484	Interferon gamma receptor 2	rs492602	49206417	19	A	G	0.490	0.054	0.005	1.6E-23	trans
1082	IFNGR2	P38484	Interferon gamma receptor 2	rs9808753	34787312	21	G	A	0.140	1.433	0.011	0.0E+00	cis
1083	IFNL1	Q8IU54	Interferon lambda-1	rs35667974	163124637	2	T	C	0.978	0.335	0.026	2.3E-37	trans
1083	IFNL1	Q8IU54	Interferon lambda-1	rs3775291	187004074	4	C	T	0.703	0.092	0.008	3.5E-28	trans
1083	IFNL1	Q8IU54	Interferon lambda-1	rs9270747	32568292	6	G	A	0.657	0.074	0.008	2.5E-19	trans
1083	IFNL1	Q8IU54	Interferon lambda-1	rs4346838	167403430	6	A	G	0.524	0.056	0.008	3.0E-13	trans
1083	IFNL1	Q8IU54	Interferon lambda-1	rs10424978	4837557	19	C	A	0.400	0.059	0.008	6.2E-14	trans
1084	IFNLR1	Q8IU57	Interferon lambda receptor 1	rs139958347	24513723	1	G	C	0.960	0.942	0.021	0.0E+00	cis
1084	IFNLR1	Q8IU57	Interferon lambda receptor 1	rs10753239	31362224	1	T	C	0.540	0.056	0.008	4.0E-13	trans
1084	IFNLR1	Q8IU57	Interferon lambda receptor 1	rs1801689	64210580	17	C	A	0.030	0.230	0.022	1.3E-26	trans
1085	IGBP1	P78318	Immunoglobulin-binding protein 1	rs6993770	106581528	8	A	T	0.714	0.063	0.008	9.9E-15	trans
1085	IGBP1	P78318	Immunoglobulin-binding protein 1	rs10733789	64948684	10	C	T	0.313	0.055	0.008	1.0E-11	trans
1085	IGBP1	P78318	Immunoglobulin-binding protein 1	rs113690423	69402868	X	C	T	0.858	0.078	0.009	1.0E-18	cis
1086	IGDCC4	Q8TDY8	Immunoglobulin superfamily DCC subclass member 4	rs7147722	50112712	14	T	G	0.539	0.054	0.007	1.0E-14	trans
1086	IGDCC4	Q8TDY8	Immunoglobulin superfamily DCC subclass member 4	rs56362513	65801720	15	C	T	0.020	0.964	0.026	0.0E+00	cis
1086	IGDCC4	Q8TDY8	Immunoglobulin superfamily DCC subclass member 4	rs77542162	67081278	17	G	A	0.023	0.174	0.023	3.9E-14	trans
1087	IGF1R	P08069	Insulin-like growth factor 1 receptor	rs9271421	32587859	6	T	C	0.145	0.120	0.010	4.4E-33	trans
1087	IGF1R	P08069	Insulin-like growth factor 1 receptor	rs2519093	136141870	9	C	T	0.816	0.481	0.009	0.0E+00	trans
1087	IGF1R	P08069	Insulin-like growth factor 1 receptor	rs35115159	99490912	15	G	A	0.203	0.130	0.009	3.4E-49	cis
1087	IGF1R	P08069	Insulin-like growth factor 1 receptor	rs11574938	30485393	16	C	G	0.516	0.093	0.007	4.6E-40	trans
1087	IGF1R	P08069	Insulin-like growth factor 1 receptor	rs200489612	7106378	17	A	G	0.005	0.882	0.053	4.3E-62	trans
1087	IGF1R	P08069	Insulin-like growth factor 1 receptor	rs9916257	33797371	17	G	T	0.561	0.065	0.007	4.6E-20	trans
1087	IGF1R	P08069	Insulin-like growth factor 1 receptor	rs56133626	16441973	19	G	A	0.674	0.068	0.008	1.1E-19	trans
1087	IGF1R	P08069	Insulin-like growth factor 1 receptor	rs760462	46328099	21	C	T	0.818	0.082	0.009	1.7E-19	trans
1088	IGF2R	P11717	Cation-independent mannose-6-phosphate receptor	rs6727484	112815002	2	A	G	0.171	0.073	0.009	2.2E-15	trans
1088	IGF2R	P11717	Cation-independent mannose-6-phosphate receptor	rs3131623	31418810	6	A	T	0.184	0.111	0.010	1.2E-30	trans
1088	IGF2R	P11717	Cation-independent mannose-6-phosphate receptor	rs2282140	160494521	6	T	C	0.131	0.556	0.011	0.0E+00	cis
1088	IGF2R	P11717	Cation-independent mannose-6-phosphate receptor	rs9987289	9183358	8	A	G	0.092	0.087	0.012	3.5E-13	trans
1088	IGF2R	P11717	Cation-independent mannose-6-phosphate receptor	rs72752535	117354865	9	C	A	0.005	0.422	0.054	3.7E-15	trans
1088	IGF2R	P11717	Cation-independent mannose-6-phosphate receptor	rs10144207	88434134	14	C	T	0.646	0.056	0.007	1.1E-14	trans
1088	IGF2R	P11717	Cation-independent mannose-6-phosphate receptor	rs145078947	93652974	14	T	G	0.003	0.989	0.068	2.2E-47	trans
1088	IGF2R	P11717	Cation-independent mannose-6-phosphate receptor	rs186021206	7069412	17	A	G	0.006	0.370	0.048	7.2E-15	trans
1088	IGF2R	P11717	Cation-independent mannose-6-phosphate receptor	rs1838105	45008935	17	A	G	0.351	0.049	0.007	1.3E-11	trans
1088	IGF2R	P11717	Cation-independent mannose-6-phosphate receptor	rs1801689	64210580	17	C	A	0.030	0.149	0.020	2.2E-13	trans
1088	IGF2R	P11717	Cation-independent mannose-6-phosphate receptor	rs200378	18289634	19	C	T	0.505	0.062	0.007	4.8E-19	trans
1089	IGFBP1	P08833	Insulin-like growth factor-binding protein 1	rs12740374	109817590	1	G	T	0.779	0.073	0.009	1.2E-16	trans
1089	IGFBP1	P08833	Insulin-like growth factor-binding protein 1	rs1260326	27730940	2	T	C	0.392	0.081	0.008	5.9E-27	trans
1090	IGFBP2	P18065	Insulin-like growth factor-binding protein 2	rs1260326	27730940	2	C	T	0.608	0.065	0.008	1.2E-17	trans
1090	IGFBP2	P18065	Insulin-like growth factor-binding protein 2	rs4674100	217480424	2	G	A	0.707	0.070	0.009	3.5E-16	cis
1090	IGFBP2	P18065	Insulin-like growth factor-binding protein 2	rs13108218	3443931	4	A	G	0.381	0.057	0.008	6.7E-14	trans
1091	IGFBP3	P17936	Insulin-like growth factor-binding protein 3	rs4234798	7219933	4	G	T	0.616	0.105	0.007	3.8E-51	trans
1091	IGFBP3	P17936	Insulin-like growth factor-binding protein 3	rs2854746	45960645	7	C	G	0.397	0.461	0.007	0.0E+00	cis
1091	IGFBP3	P17936	Insulin-like growth factor-binding protein 3	rs9987289	9183358	8	A	G	0.092	0.080	0.012	1.0E-11	trans
1091	IGFBP3	P17936	Insulin-like growth factor-binding protein 3	rs1065656	1838836	16	G	C	0.694	0.120	0.007	3.7E-58	trans
1091	IGFBP3	P17936	Insulin-like growth factor-binding protein 3	rs738409	44324727	22	C	G	0.783	0.064	0.008	6.4E-15	trans
1092	IGFBP4	P22692	Insulin-like growth factor-binding protein 4	rs4859682	77410318	4	A	C	0.457	0.053	0.007	6.0E-14	trans
1092	IGFBP4	P22692	Insulin-like growth factor-binding protein 4	rs28640218	20359267	16	G	T	0.809	0.069	0.009	4.6E-14	trans
1093	IGFBP6	P24592	Insulin-like growth factor-binding protein 6	rs13146355	77412140	4	A	G	0.456	0.048	0.007	2.2E-13	trans
1093	IGFBP6	P24592	Insulin-like growth factor-binding protein 6	rs10265221	151414329	7	C	T	0.287	0.052	0.007	6.9E-13	trans
1093	IGFBP6	P24592	Insulin-like growth factor-binding protein 6	rs4997081	20365234	16	G	C	0.808	0.075	0.008	1.1E-19	trans
1094	IGFBP7	Q16270	Insulin-like growth factor-binding protein 7	rs1718860	57949517	4	G	A	0.267	0.372	0.008	0.0E+00	cis
1094	IGFBP7	Q16270	Insulin-like growth factor-binding protein 7	rs3104394	32667520	6	G	A	0.140	0.124	0.010	2.9E-35	trans
1094	IGFBP7	Q16270	Insulin-like growth factor-binding protein 7	rs7282172	46446861	21	G	C	0.173	0.065	0.009	5.7E-13	trans
1094	IGFBP7	Q16270	Insulin-like growth factor-binding protein 7	rs738409	44324727	22	G	C	0.217	0.060	0.008	2.7E-13	trans
1095	IGFBPL1	Q8WX77	Insulin-like growth factor-binding protein-like 1	rs1260326	27730940	2	T	C	0.392	0.070	0.007	2.0E-25	trans
1095	IGFBPL1	Q8WX77	Insulin-like growth factor-binding protein-like 1	rs7857243	38409081	9	A	G	0.807	0.495	0.009	0.0E+00	cis
1096	IGHMBP2	P38935	DNA-binding protein SMUBP-2	rs607929	32586998	6	C	G	0.551	0.054	0.008	2.6E-12	trans
1096	IGHMBP2	P38935	DNA-binding protein SMUBP-2	rs11228377	68603346	11	T	C	0.402	0.079	0.008	7.7E-24	cis
1097	IGLC2	P0DOY2	Immunoglobulin lambda constant 2	rs188468174	25291697	1	C	T	0.986	0.284	0.032	3.7E-19	trans
1097	IGLC2	P0DOY2	Immunoglobulin lambda constant 2	rs12741847	118192796	1	C	T	0.610	0.054	0.008	1.3E-12	trans
1097	IGLC2	P0DOY2	Immunoglobulin lambda constant 2	rs115975731	89131386	2	G	A	0.968	0.396	0.021	5.0E-76	trans
1097	IGLC2	P0DOY2	Immunoglobulin lambda constant 2	rs111632177	150931805	7	A	G	0.114	0.080	0.012	7.3E-12	trans
1097	IGLC2	P0DOY2	Immunoglobulin lambda constant 2	rs12880641	103251452	14	T	G	0.339	0.058	0.008	1.3E-13	trans
1097	IGLC2	P0DOY2	Immunoglobulin lambda constant 2	rs1407	106174261	14	C	G	0.716	0.071	0.009	4.3E-16	trans
1097	IGLC2	P0DOY2	Immunoglobulin lambda constant 2	rs34562254	16842991	17	A	G	0.097	0.109	0.012	1.3E-18	trans
1097	IGLC2	P0DOY2	Immunoglobulin lambda constant 2	rs2856876	23249440	22	A	C	0.116	0.229	0.012	7.9E-87	cis
1098	IGSF21	Q96ID5	Immunoglobulin superfamily member 21	rs11261050	18447853	1	C	T	0.681	0.152	0.008	3.2E-82	cis

1098	IGSF21	Q96ID5	Immunoglobulin superfamily member 21	rs2395204	32452005	6	A	G	0.823	0.083	0.010	3.0E-18	trans
1098	IGSF21	Q96ID5	Immunoglobulin superfamily member 21	rs186021206	7069412	17	A	G	0.006	0.683	0.049	2.9E-43	trans
1099	IGSF3	O75054	Immunoglobulin superfamily member 3	rs2284860	117122887	1	C	T	0.217	0.110	0.009	6.7E-34	cis
1099	IGSF3	O75054	Immunoglobulin superfamily member 3	rs8176749	136131188	9	T	C	0.061	0.140	0.015	5.9E-20	trans
1099	IGSF3	O75054	Immunoglobulin superfamily member 3	rs56278466	17875857	10	G	T	0.661	0.153	0.008	1.4E-85	trans
1099	IGSF3	O75054	Immunoglobulin superfamily member 3	rs3747207	44324855	22	A	G	0.216	0.064	0.009	5.7E-13	trans
1100	IGSF8	Q969P0	Immunoglobulin superfamily member 8	rs12408242	160061934	1	C	T	0.215	0.156	0.009	1.3E-65	cis
1100	IGSF8	Q969P0	Immunoglobulin superfamily member 8	rs6967348	129247134	7	G	A	0.088	0.092	0.013	3.5E-12	trans
1100	IGSF8	Q969P0	Immunoglobulin superfamily member 8	rs6993770	106581528	8	A	T	0.713	0.086	0.008	2.0E-25	trans
1100	IGSF8	Q969P0	Immunoglobulin superfamily member 8	rs28365809	130029661	11	T	C	0.854	0.135	0.011	1.5E-35	trans
1100	IGSF8	Q969P0	Immunoglobulin superfamily member 8	rs892090	55539072	19	G	T	0.834	0.073	0.010	4.0E-13	trans
1101	IGSF9	Q9P2J2	Protein turtle homolog A	rs3747617	159912899	1	C	T	0.947	0.479	0.017	2.6E-181	cis
1101	IGSF9	Q9P2J2	Protein turtle homolog A	rs1260326	27730940	2	T	C	0.391	0.136	0.008	8.9E-74	trans
1101	IGSF9	Q9P2J2	Protein turtle homolog A	rs1047891	211540507	2	C	A	0.687	0.067	0.008	2.0E-17	trans
1101	IGSF9	Q9P2J2	Protein turtle homolog A	rs71556736	73034929	7	C	T	0.871	0.125	0.011	1.2E-30	trans
1101	IGSF9	Q9P2J2	Protein turtle homolog A	rs139974673	44027885	15	C	T	0.024	0.165	0.024	2.5E-12	trans
1102	IKZF2	Q9UKS7	Zinc finger protein Helios	rs34136174	32583682	6	C	A	0.425	0.079	0.008	2.4E-23	trans
1102	IKZF2	Q9UKS7	Zinc finger protein Helios	rs762465	22574855	22	T	C	0.635	0.106	0.008	9.0E-40	trans
1103	IL10	P22301	Interleukin-10	rs2476601	114377568	1	A	G	0.100	0.088	0.013	3.9E-12	trans
1103	IL10	P22301	Interleukin-10	rs3024495	206942413	1	C	T	0.846	0.141	0.011	3.6E-40	cis
1103	IL10	P22301	Interleukin-10	rs67619842	32536085	6	T	A	0.124	0.181	0.012	6.4E-54	trans
1103	IL10	P22301	Interleukin-10	rs3135932	117864063	11	G	A	0.165	0.265	0.010	2.5E-147	trans
1103	IL10	P22301	Interleukin-10	rs7203793	11182134	16	C	G	0.643	0.067	0.008	3.6E-17	trans
1104	IL10RA	Q13651	Interleukin-10 receptor subunit alpha	rs3135932	117864063	11	G	A	0.165	0.189	0.010	1.2E-74	cis
1105	IL10RB	Q08334	Interleukin-10 receptor subunit beta	rs7532110	47961964	1	A	T	0.803	0.060	0.009	2.9E-12	trans
1105	IL10RB	Q08334	Interleukin-10 receptor subunit beta	rs61747728	179526214	1	T	C	0.038	0.192	0.018	1.9E-27	trans
1105	IL10RB	Q08334	Interleukin-10 receptor subunit beta	rs56387622	46888198	3	C	T	0.099	0.079	0.011	5.9E-12	trans
1105	IL10RB	Q08334	Interleukin-10 receptor subunit beta	rs73632737	126226775	11	C	G	0.072	0.089	0.013	1.3E-11	trans
1105	IL10RB	Q08334	Interleukin-10 receptor subunit beta	rs186021206	7069412	17	A	G	0.006	0.413	0.047	6.6E-19	trans
1105	IL10RB	Q08334	Interleukin-10 receptor subunit beta	rs2659005	79218714	17	T	C	0.441	0.070	0.007	9.4E-25	trans
1105	IL10RB	Q08334	Interleukin-10 receptor subunit beta	rs2515717	34662282	21	A	G	0.450	0.532	0.007	0.0E+00	cis
1106	IL11	P20809	Interleukin-11	rs188468174	25291697	1	T	C	0.014	0.242	0.033	2.1E-13	trans
1106	IL11	P20809	Interleukin-11	rs28479853	107106328	14	A	G	0.143	0.076	0.011	9.7E-12	trans
1107	IL12A_IL12B	P29459,P29460	Interleukin-12	rs12471768	64928603	2	C	T	0.705	0.050	0.007	1.7E-11	trans
1107	IL12A_IL12B	P29459,P29460	Interleukin-12	rs13025330	111851212	2	C	T	0.783	0.060	0.008	4.1E-13	trans
1107	IL12A_IL12B	P29459,P29460	Interleukin-12	rs72852162	145486323	2	C	A	0.112	0.077	0.011	1.4E-12	trans
1107	IL12A_IL12B	P29459,P29460	Interleukin-12	rs11130215	5026008	3	G	A	0.173	0.098	0.009	9.7E-27	trans
1107	IL12A_IL12B	P29459,P29460	Interleukin-12	rs9815073	188115682	3	A	C	0.342	0.303	0.008	0.0E+00	trans
1107	IL12A_IL12B	P29460	Interleukin-12	rs4244437	158773117	5	A	G	0.677	0.543	0.008	0.0E+00	cis
1107	IL12A_IL12B	P29459,P29460	Interleukin-12	rs9265987	31316288	6	C	T	0.322	0.098	0.007	1.9E-41	trans
1107	IL12A_IL12B	P29459,P29460	Interleukin-12	rs836559	6448763	7	C	G	0.459	0.068	0.007	2.4E-23	trans
1107	IL12A_IL12B	P29459,P29460	Interleukin-12	rs4287251	23670597	10	T	C	0.799	0.064	0.008	3.5E-14	trans
1107	IL12A_IL12B	P29459,P29460	Interleukin-12	rs11039216	47406592	11	T	C	0.530	0.046	0.007	1.6E-11	trans
1107	IL12A_IL12B	P29459,P29460	Interleukin-12	rs561877	96019254	11	G	A	0.682	0.056	0.007	1.7E-14	trans
1107	IL12A_IL12B	P29459,P29460	Interleukin-12	rs76428106	28604007	13	C	T	0.013	0.390	0.031	8.4E-36	trans
1107	IL12A_IL12B	P29459,P29460	Interleukin-12	rs423575	69182120	14	C	G	0.516	0.052	0.007	2.4E-14	trans
1107	IL12A_IL12B	P29459,P29460	Interleukin-12	rs12588969	103230758	14	G	C	0.316	0.107	0.007	1.3E-48	trans
1107	IL12A_IL12B	P29459,P29460	Interleukin-12	rs1987102	80265888	15	T	C	0.740	0.075	0.008	2.1E-22	trans
1108	IL12B	P29460	Interleukin-12 subunit beta	rs58394161	92939959	1	C	T	0.152	0.067	0.009	7.6E-13	trans
1108	IL12B	P29460	Interleukin-12 subunit beta	rs12471768	64928603	2	C	T	0.705	0.050	0.007	1.2E-11	trans
1108	IL12B	P29460	Interleukin-12 subunit beta	rs13025330	111851212	2	C	T	0.783	0.057	0.008	3.6E-12	trans
1108	IL12B	P29460	Interleukin-12 subunit beta	rs72852162	145486323	2	C	A	0.112	0.082	0.011	1.5E-14	trans
1108	IL12B	P29460	Interleukin-12 subunit beta	rs11130215	5026008	3	G	A	0.173	0.090	0.009	8.1E-24	trans
1108	IL12B	P29460	Interleukin-12 subunit beta	rs9815073	188115682	3	A	C	0.343	0.304	0.007	0.0E+00	trans
1108	IL12B	P29460	Interleukin-12 subunit beta	rs4244437	158773117	5	A	G	0.677	0.560	0.008	0.0E+00	cis
1108	IL12B	P29460	Interleukin-12 subunit beta	rs3130459	31154633	6	C	T	0.257	0.108	0.008	1.9E-44	trans
1108	IL12B	P29460	Interleukin-12 subunit beta	rs836558	6447991	7	A	C	0.459	0.067	0.007	1.2E-23	trans
1108	IL12B	P29460	Interleukin-12 subunit beta	rs4287251	23670597	10	T	C	0.799	0.062	0.008	7.4E-14	trans
1108	IL12B	P29460	Interleukin-12 subunit beta	rs11039216	47406592	11	T	C	0.530	0.048	0.007	1.4E-12	trans
1108	IL12B	P29460	Interleukin-12 subunit beta	rs502822	96022489	11	C	T	0.697	0.061	0.007	8.6E-17	trans
1108	IL12B	P29460	Interleukin-12 subunit beta	rs76428106	28604007	13	C	T	0.013	0.398	0.031	2.4E-38	trans
1108	IL12B	P29460	Interleukin-12 subunit beta	rs374173	69182119	14	G	C	0.516	0.052	0.007	1.9E-14	trans
1108	IL12B	P29460	Interleukin-12 subunit beta	rs12588969	103230758	14	G	C	0.316	0.108	0.007	1.3E-50	trans
1108	IL12B	P29460	Interleukin-12 subunit beta	rs1987102	80265888	15	T	C	0.740	0.077	0.008	3.6E-24	trans
1108	IL12B	P29460	Interleukin-12 subunit beta	rs4843855	85902546	16	T	C	0.272	0.051	0.008	1.7E-11	trans
1108	IL12B	P29460	Interleukin-12 subunit beta	rs73068668	55763262	19	A	G	0.075	0.090	0.013	1.4E-12	trans
1109	IL12RB1	P42701	Interleukin-12 receptor subunit beta-1	rs2844619	31242223	6	G	C	0.607	0.084	0.007	6.8E-30	trans
1109	IL12RB1	P42701	Interleukin-12 receptor subunit beta-1	rs950802	60152584	11	A	G	0.307	0.279	0.008	1.7E-270	trans
1109	IL12RB1	P42701	Interleukin-12 receptor subunit beta-1	rs12720356	10469975	19	A	C	0.901	0.133	0.013	2.5E-26	trans
1109	IL12RB1	P42701	Interleukin-12 receptor subunit beta-1	rs369378	18189759	19	G	A	0.787	0.456	0.009	0.0E+00	cis
1110	IL12RB2	Q99665	Interleukin-12 receptor subunit beta-2	rs511874	32293255	6	G	C	0.717	0.074	0.009	5.0E-18	trans
1111	IL13	P35225	Interleukin-13	rs34118068	106434607	14	A	G	0.105	0.148	0.013	9.1E-31	trans
1112	IL13RA1	P78552	Interleukin-13 receptor subunit alpha-1	rs351973	795457	19	G	A	0.641	0.079	0.008	7.6E-23	trans
1112	IL13RA1	P78552	Interleukin-13 receptor subunit alpha-1	rs73201506	33044408	21	A	G	0.955	0.142	0.018	1.0E-14	trans
1112	IL13RA1	P78552	Interleukin-13 receptor subunit alpha-1	rs2495635	117924097	X	G	A	0.162	0.856	0.008	0.0E+00	cis
1113	IL15	P40933	Interleukin-15	rs149968614	65312368	1	T	C	0.002	0.649	0.088	1.5E-13	trans
1113	IL15	P40933	Interleukin-15	rs6693121	101744633	1	A	C	0.408	0.052	0.008	6.4E-12	trans
1113	IL15	P40933	Interleukin-15	rs4848370	111811665	2	T	C	0.272	0.145	0.008	1.8E-67	trans
1113	IL15	P40933	Interleukin-15	rs1058315	232573944	2	G	C	0.773	0.067	0.009	5.6E-14	trans
1113	IL15	P40933	Interleukin-15	rs4683324	47251284	3	T	C	0.423	0.081	0.008	3.8E-27	trans
1113	IL15	P40933	Interleukin-15	rs9844706	128380781	3	G	A	0.110	0.085	0.012	8.8E-13	trans
1113	IL15	P40933	Interleukin-15	rs2523594	31326629	6	C	T	0.378	0.123	0.008	3.9E-57	trans
1113	IL15	P40933	Interleukin-15	rs6924776	109612276	6	G	A	0.487	0.052	0.007	2.5E-12	trans
1113	IL15	P40933	Interleukin-15	rs1378901	128213807	8	C	T	0.437	0.051	0.007	1.3E-11	trans
1113	IL15	P40933	Interleukin-15	rs8177636	6018594	10	T	C	0.568	0.056	0.007	4.9E-14	trans
1113	IL15	P40933	Interleukin-15	rs2646420	8467037	10	C	T	0.621	0.063	0.008	1.5E-16	trans
1113	IL15	P40933	Interleukin-15	rs1926197	90748626	10	A	G	0.545	0.058	0.007	4.8E-15	trans
1113	IL15	P40933	Interleukin-15	rs12363646	128067342	11	T	C	0.709	0.063	0.008	1.4E-14	trans
1113	IL15	P40933	Interleukin-15	rs76428106	28604007	13	T	C	0.987	0.302	0.034	6.8E-19	trans
1113	IL15	P40933	Interleukin-15	rs11632933	70744226	15	A	G	0.618	0.052	0.008	1.1E-11	trans
1113	IL15	P40933	Interleukin-15	rs58745116	33803592	17	A	G	0.390	0.147	0.008	3.0E-82	trans
1113	IL15	P40933	Interleukin-15	rs3212802	17937104	19	T	G	0.184	0.089	0.010	3.6E-20	trans
1113	IL15	P40933	Interleukin-15	rs7268811	35550871	20	A	C	0.167	0.070	0.010	2.5E-12	trans
1113	IL15	P40933	Interleukin-15	rs35477491	15273817	21	T	C	0.224	0.072	0.011	1.1E-11	trans
1113	IL15	P40933	Interleukin-15	rs228951	37529798	22	G	A	0.366	0.074	0.008	2.4E-21	trans
1114	IL15RA	Q13261	Interleukin-15 receptor subunit alpha	rs1050486	31324702	6	T	C	0.616	0.063	0.008	7.5E-15	trans
1114	IL15RA	Q13261	Interleukin-15 receptor subunit alpha	rs3136630	5997820	10	C	T	0.704	0.259	0.008	4.8E-214	cis
1115	IL16	Q14005	Pro-interleukin-16	rs12342201	95894964	9	G	A	0.523	0.061	0.007	1.3E-18	trans
1115	IL16	Q14005	Pro-interleukin-16	rs58754882	81595358	15	G	A	0.910	0.968	0.013	0.0E+00	cis
1116	IL17C	Q9P0M4	Interleukin-17C	rs8177833	150466690	5	G	GC	0.055	0.117	0.016	9.9E-13	trans
1116	IL17C	Q9P0M4	Interleukin-17C	rs17700884	88684495	16	G	T	0.638	0.120	0.008	7.6E-53	cis
1117	IL17D	Q8TAD2	Interleukin-17D	rs4770059	21151047	13	T	C	0.263	0.398	0.008	0.0E+00	cis
1117	IL17D	Q8TAD2	Interleukin-17D	rs77542162	67081278	17	G	A	0.023	0.248	0.022	8.8E-29	trans
1118	IL17F	Q96PD4	Interleukin-17F	rs10152051	106573559	14	A	G	0.553	0.068	0.008	5.0E-17	trans
1119	IL17RA	Q96F46	Interleukin-17 receptor A	rs78689694	126234820	11	G	C	0.867	0.064	0.008	1.3E-15	trans
1119	IL17RA	Q96F46	Interleukin-17 receptor A	rs186021206	7069412	17	A	G	0.006	0.474	0.037	3.1E-37	trans
1119	IL17RA	Q96F46	Interleukin-17 receptor A	rs4819959	17586631	22	A	G	0.495	0.857	0.008	0.0E+00	cis
1120	IL17RB	Q9NRM6	Interleukin-17 receptor B	rs1260326	27730940	2	C	T	0.609	0.056	0.006	3.6E-20	trans
1120	IL17RB	Q9NRM6	Interleukin-17 receptor B	rs6445607	53877149	3	G	T	0.375	0.807	0.008	0.0E+00	cis
1120	IL17RB	Q9NRM6	Interleukin-17 receptor B	rs57413756	170738665	3	C	G	0.889	0.085	0.012	1.5E-12	trans
1120	IL17RB	Q9NRM6	Interleukin-17 receptor B	rs2894204	31237061	6	C	T	0.367	0.065	0.006	1.6E-26	trans
1120	IL17RB	Q9NRM6	Interleukin-17 receptor B	rs34121855	73040814	7	G	T	0.201	0.053	0.007	6.2E-13	trans
1120	IL17RB	Q9NRM6	Interleukin-17 receptor B	rs1870148	82271341	10	A	G	0.789	0.057	0.007	4.7E-15	trans
1120	IL17RB	Q9NRM6	Interleukin-17 receptor B	rs28929474	94844947	14	T	C	0.021	0.229	0.021	2.2E-28	trans
1120	IL17RB	Q9NRM6	Interleukin-17 receptor B	rs77542162	67081278	17	G	A	0.023	0.154	0.020	9.8E-15	trans
1121	IL18	Q14116	Interleukin-18	rs385076	32489851	2	C	T	0.644	0.220	0.008	2.6E-187	trans
1121	IL18	Q14116	Interleukin-18	rs77762937	203467470	2	T	G	0.288	0.060	0.008	4.8E-14	trans
1121	IL18	Q14116	Interleukin-18	rs130066	31122315	6	G	C	0.542	0.053	0.007	1.2E-13	trans
1121	IL18	Q14116	Interleukin-18	rs2290414	144643603	8	G	A	0.559	0.072	0.007	5.0E-24	trans
1121	IL18	Q14116	Interleukin-18	rs117558678	270330	11	T	A	0.240	0.072	0.009	1.3E-16	trans
1121	IL18	Q14116	Interleukin-18	rs17103763	105003872	11	A	T	0.261	0.108	0.008	1.7E-38	trans
1121	IL18	Q14116	Interleukin-18	rs2250417	112085316	11	T	C	0.460	0.295	0.007	0.0E+00	cis
1121	IL18	Q14116	Interleukin-18	rs10418046	54327869	19	G	T	0.211	0.066	0.009	2.9E-14	trans
1122	IL18BP	O95998	Interleukin-18-binding protein	rs61747728	179526214	1	T	C	0.038	0.164	0.019	1.8E-18	trans
1122	IL18BP	O95998	Interleukin-18-binding protein	rs10901252	136128000	9	C	G	0.061	0.155	0.015	8.6E-25	trans
1122	IL18BP	O95998	Interleukin-18-binding protein	rs5743659	71709878	11	G	A	0.994	0.547	0.048	1.2E-30	cis
1122	IL18BP	O95998	Interleukin-18-binding protein	rs112771035	126225876	11	G	C	0.071	0.144	0.014	1.5E-24	trans
1122	IL18BP	O95998	Interleukin-18-binding protein	rs186021206	7069412	17	A	G	0.006	0.865	0.050	3.4E-68	trans
1122	IL18BP	O95998	Interleukin-18-binding protein	rs10445407	79261809	17	C	A	0.521	0.051	0.007	1.7E-12	trans
1123	IL18R1	Q13478	Interleukin-18 receptor 1	rs10190555	102994056	2	A	G	0.226	0.898	0.009	0.0E+00	cis
1123	IL18R1	Q13478	Interleukin-18 receptor 1	rs407258	155560978	3	T	C	0.750	0.067	0.007	6.1E-22	trans
1123	IL18R1	Q13478	Interleukin-18 receptor 1	rs28636815	77197397	4	A	G	0.623	0.044	0.006	9.0E-13	trans
1123	IL18R1	Q13478	Interleukin-18 receptor 1	rs56278466	17875857	10	G	T	0.661	0.111	0.006	3.4E-68	trans
1123	IL18R1	Q13478	Interleukin-18 receptor 1	rs28929474	94844947	14	T	C	0.021	0.264	0.021	8.5E-36	trans
1123	IL18R1	Q13478	Interleukin-18 receptor 1	rs147233090	44028047	15	T	C	0.024	0.149	0.020	7.5E-14	trans
1123	IL18R1	Q13478	Interleukin-18 receptor 1	rs77303550	72079657	16	C	T	0.804	0.067	0.008	2.6E-18	trans
1123	IL18R1	Q13478	Interleukin-18 receptor 1	rs1801689	64210580	17	C	A	0.030	0.429	0.018	1.0E-130	trans
1123	IL18R1	Q13478	Interleukin-18 receptor 1	rs12019136	5835677	19	A	G	0.041	0.113	0.015	8.2E-14	trans
1124	IL18RAP	O95256	Interleukin-18 receptor accessory protein	rs72490060	89298051	2	T	C	0.195	0.118	0.011	6.6E-27	trans
1124	IL18RAP	O95256	Interleukin-18 receptor accessory protein	rs62146110	95431707	2	T	C	0.227	0.074	0.009	6.4E-16	trans
1124	IL18RAP	O95256	Interleukin-18 receptor accessory protein	rs1655901	29916804	6	T	C	0.441	0.072	0.008	1.7E-20	trans
1124	IL18RAP	O95256	Interleukin-18 receptor accessory protein	rs4774021	107218161	14	T	G	0.137	0.157	0.011	8.5E-46	trans
1125	IL19	Q9UHD0	Interleukin-19	rs2243191	207015957	1	C	T	0.779	0.356	0.009	0.0E+00	cis

1125	IL19	Q9UHD0	Interleukin-19	rs80235202	32503831	6	G	A	0.282	0.056	0.008	1.1E-11	trans
1125	IL19	Q9UHD0	Interleukin-19	rs34819998	126220990	11	C	G	0.146	0.076	0.011	1.4E-12	trans
1125	IL19	Q9UHD0	Interleukin-19	rs2608894	5848000	19	T	C	0.235	0.069	0.009	5.0E-15	trans
1125	IL19	Q9UHD0	Interleukin-19	rs492602	49206417	19	G	A	0.509	0.378	0.007	0.0E+00	trans
1126	IL1A	P01583	Interleukin-1 alpha	rs28464365	32587050	6	T	C	0.956	0.267	0.019	1.6E-45	trans
1127	IL1B	P01584	Interleukin-1 beta	rs6993770	106581528	8	A	T	0.713	0.077	0.008	3.5E-20	trans
1127	IL1B	P01584	Interleukin-1 beta	rs34436714	54327313	19	A	C	0.209	0.297	0.009	7.9E-218	trans
1128	IL1R1	P14778	Interleukin-1 receptor type 1	rs61830291	221001142	1	A	C	0.903	0.126	0.012	9.8E-25	trans
1128	IL1R1	P14778	Interleukin-1 receptor type 1	rs6545929	62610179	2	G	A	0.805	0.084	0.009	1.9E-19	trans
1128	IL1R1	P14778	Interleukin-1 receptor type 1	rs3917238	102773083	2	C	T	0.699	0.184	0.008	1.5E-114	cis
1128	IL1R1	P14778	Interleukin-1 receptor type 1	rs55709272	113867288	2	C	T	0.439	0.135	0.007	4.7E-74	trans
1128	IL1R1	P14778	Interleukin-1 receptor type 1	rs10935473	98416900	3	G	T	0.560	0.289	0.007	0.0E+00	trans
1128	IL1R1	P14778	Interleukin-1 receptor type 1	rs12505880	56307155	4	A	C	0.344	0.052	0.008	1.4E-11	trans
1128	IL1R1	P14778	Interleukin-1 receptor type 1	rs1634775	31303236	6	A	G	0.377	0.075	0.007	1.2E-23	trans
1128	IL1R1	P14778	Interleukin-1 receptor type 1	rs4841133	9183664	8	A	G	0.092	0.088	0.013	1.7E-12	trans
1128	IL1R1	P14778	Interleukin-1 receptor type 1	rs8176672	136142185	9	T	C	0.061	0.214	0.015	5.5E-46	trans
1128	IL1R1	P14778	Interleukin-1 receptor type 1	rs56278466	17875857	10	G	T	0.661	0.064	0.008	4.3E-17	trans
1128	IL1R1	P14778	Interleukin-1 receptor type 1	rs28929474	94844947	14	T	C	0.021	0.250	0.025	6.7E-23	trans
1128	IL1R1	P14778	Interleukin-1 receptor type 1	rs186021206	7069412	17	A	G	0.006	0.605	0.050	3.3E-34	trans
1128	IL1R1	P14778	Interleukin-1 receptor type 1	rs2292642	76395430	17	C	T	0.396	0.070	0.007	4.2E-21	trans
1128	IL1R1	P14778	Interleukin-1 receptor type 1	rs2837988	42619544	21	A	C	0.362	0.089	0.008	8.1E-32	trans
1129	IL1R2	P27930	Interleukin-1 receptor type 2	rs165316	91533297	1	A	G	0.802	0.063	0.009	2.1E-13	trans
1129	IL1R2	P27930	Interleukin-1 receptor type 2	rs629301	109818306	1	G	T	0.222	0.070	0.008	2.4E-17	trans
1129	IL1R2	P27930	Interleukin-1 receptor type 2	rs2310170	102616154	2	T	G	0.600	0.517	0.008	0.0E+00	cis
1129	IL1R2	P27930	Interleukin-1 receptor type 2	rs3129853	32398648	6	G	A	0.693	0.071	0.007	1.1E-21	trans
1129	IL1R2	P27930	Interleukin-1 receptor type 2	rs6973520	128740443	7	T	C	0.507	0.067	0.007	2.4E-22	trans
1129	IL1R2	P27930	Interleukin-1 receptor type 2	rs28601761	126500031	8	C	G	0.580	0.125	0.007	4.0E-71	trans
1129	IL1R2	P27930	Interleukin-1 receptor type 2	rs1870138	82269611	10	G	A	0.796	0.084	0.008	4.9E-23	trans
1129	IL1R2	P27930	Interleukin-1 receptor type 2	rs10831245	94319888	11	A	G	0.387	0.066	0.007	1.3E-20	trans
1129	IL1R2	P27930	Interleukin-1 receptor type 2	rs186021206	7069412	17	A	G	0.006	0.497	0.047	6.0E-26	trans
1129	IL1R2	P27930	Interleukin-1 receptor type 2	rs77542162	67081278	17	G	A	0.023	0.179	0.023	7.3E-15	trans
1130	IL1RAP	Q9NPH3	Interleukin-1 receptor accessory protein	rs6444442	190346060	3	A	G	0.154	1.401	0.010	0.0E+00	cis
1130	IL1RAP	Q9NPH3	Interleukin-1 receptor accessory protein	rs4921914	18272438	8	C	T	0.221	0.055	0.006	7.3E-21	trans
1130	IL1RAP	Q9NPH3	Interleukin-1 receptor accessory protein	rs139974673	44027885	15	C	T	0.024	0.196	0.016	3.3E-36	trans
1130	IL1RAP	Q9NPH3	Interleukin-1 receptor accessory protein	rs273510	18223350	19	A	G	0.707	0.044	0.005	2.1E-16	trans
1130	IL1RAP	Q9NPH3	Interleukin-1 receptor accessory protein	rs7060124	109940046	X	C	G	0.602	0.029	0.004	8.2E-13	trans
1131	IL1RL1	Q01638	Interleukin-1 receptor-like 1	rs13028833	61644329	2	G	C	0.517	0.063	0.007	5.8E-20	trans
1131	IL1RL1	Q01638	Interleukin-1 receptor-like 1	rs12470864	102926362	2	G	A	0.609	0.689	0.007	0.0E+00	cis
1131	IL1RL1	Q01638	Interleukin-1 receptor-like 1	rs7616330	71115751	3	C	A	0.825	0.051	0.007	4.6E-12	trans
1131	IL1RL1	Q01638	Interleukin-1 receptor-like 1	rs13107325	103188709	4	T	C	0.075	0.113	0.011	1.3E-26	trans
1131	IL1RL1	Q01638	Interleukin-1 receptor-like 1	rs2519093	136141870	9	C	T	0.816	0.091	0.007	2.2E-36	trans
1131	IL1RL1	Q01638	Interleukin-1 receptor-like 1	rs11603123	126305495	11	A	G	0.034	0.381	0.016	2.2E-132	trans
1131	IL1RL1	Q01638	Interleukin-1 receptor-like 1	rs1378942	75077367	15	A	C	0.681	0.053	0.006	4.2E-19	trans
1131	IL1RL1	Q01638	Interleukin-1 receptor-like 1	rs12445547	88518569	16	G	T	0.688	0.060	0.006	1.2E-23	trans
1131	IL1RL1	Q01638	Interleukin-1 receptor-like 1	rs186021206	7069412	17	A	G	0.006	0.963	0.038	9.8E-139	trans
1131	IL1RL1	Q01638	Interleukin-1 receptor-like 1	rs2899319	39874314	22	C	A	0.617	0.044	0.006	3.9E-14	trans
1132	IL1RL2	Q9HB29	Interleukin-1 receptor-like 2	rs10779835	230299949	1	T	C	0.388	0.049	0.007	1.4E-12	trans
1132	IL1RL2	Q9HB29	Interleukin-1 receptor-like 2	rs3917265	102778461	2	T	C	0.457	0.437	0.007	0.0E+00	cis
1132	IL1RL2	Q9HB29	Interleukin-1 receptor-like 2	rs11928797	33457493	3	A	C	0.116	0.075	0.010	7.3E-13	trans
1132	IL1RL2	Q9HB29	Interleukin-1 receptor-like 2	rs13203302	96007698	6	T	C	0.084	0.106	0.012	1.1E-18	trans
1132	IL1RL2	Q9HB29	Interleukin-1 receptor-like 2	rs10815254	5630344	9	T	C	0.281	0.051	0.007	1.0E-11	trans
1132	IL1RL2	Q9HB29	Interleukin-1 receptor-like 2	rs56278466	17875857	10	G	T	0.661	0.067	0.007	2.5E-21	trans
1132	IL1RL2	Q9HB29	Interleukin-1 receptor-like 2	rs3007036	50106045	14	G	A	0.539	0.058	0.007	4.7E-18	trans
1132	IL1RL2	Q9HB29	Interleukin-1 receptor-like 2	rs28929474	94844947	14	T	C	0.021	0.216	0.024	3.9E-20	trans
1132	IL1RL2	Q9HB29	Interleukin-1 receptor-like 2	rs62011287	63791228	15	A	G	0.658	0.054	0.007	2.5E-14	trans
1132	IL1RL2	Q9HB29	Interleukin-1 receptor-like 2	rs7599	36038390	19	A	G	0.366	0.056	0.007	3.9E-16	trans
1133	IL1RN	P18510	Interleukin-1 receptor antagonist protein	rs55709272	113867288	2	T	C	0.561	0.213	0.008	1.7E-173	cis
1133	IL1RN	P18510	Interleukin-1 receptor antagonist protein	rs12941811	38159335	17	T	C	0.425	0.055	0.007	2.3E-13	trans
1134	IL22	Q9GZX6	Interleukin-22	rs6689	32627700	6	A	G	0.811	0.072	0.009	6.4E-15	trans
1134	IL22	Q9GZX6	Interleukin-22	rs4794067	45808828	17	T	C	0.741	0.060	0.008	3.0E-13	trans
1135	IL22RA1	Q8N6P7	Interleukin-22 receptor subunit alpha-1	rs7355101	24451085	1	A	T	0.805	0.318	0.010	1.2E-239	cis
1135	IL22RA1	Q8N6P7	Interleukin-22 receptor subunit alpha-1	rs681343	49206462	19	C	T	0.491	0.149	0.008	5.3E-86	trans
1136	IL2RA	P01589	Interleukin-2 receptor subunit alpha	rs61747728	179526214	1	T	C	0.038	0.128	0.017	2.6E-13	trans
1136	IL2RA	P01589	Interleukin-2 receptor subunit alpha	rs734866	24475497	3	C	A	0.195	0.087	0.008	6.6E-25	trans
1136	IL2RA	P01589	Interleukin-2 receptor subunit alpha	rs3094005	31465047	6	T	G	0.141	0.120	0.010	1.8E-35	trans
1136	IL2RA	P01589	Interleukin-2 receptor subunit alpha	rs12722497	6095928	10	A	C	0.089	0.956	0.013	0.0E+00	cis
1136	IL2RA	P01589	Interleukin-2 receptor subunit alpha	rs1955512	33175822	14	A	G	0.572	0.063	0.007	1.1E-19	trans
1136	IL2RA	P01589	Interleukin-2 receptor subunit alpha	rs924138	16126764	16	T	C	0.389	0.064	0.007	3.0E-20	trans
1136	IL2RA	P01589	Interleukin-2 receptor subunit alpha	rs2659007	79217478	17	A	G	0.450	0.054	0.007	1.3E-15	trans
1136	IL2RA	P01589	Interleukin-2 receptor subunit alpha	rs5916966	105300358	X	A	G	0.880	0.063	0.009	1.7E-13	trans
1137	IL2RG	P31785	Cytokine receptor common subunit gamma	rs186021206	7069412	17	A	G	0.006	0.413	0.052	2.4E-15	trans
1138	IL31	Q6EBC2	Interleukin-31	rs4520243	137775155	9	C	T	0.347	0.082	0.008	8.8E-24	trans
1139	IL31RA	Q8NI17	Interleukin-31 receptor subunit alpha	rs161651	55205595	5	C	T	0.695	0.631	0.008	0.0E+00	cis
1139	IL31RA	Q8NI17	Interleukin-31 receptor subunit alpha	rs1570350	143592386	6	G	A	0.439	0.047	0.007	2.1E-12	trans
1140	IL32	P24001	Interleukin-32	rs117199990	19820916	8	T	C	0.101	0.108	0.012	2.2E-19	trans
1140	IL32	P24001	Interleukin-32	rs12905732	58683542	15	C	G	0.200	0.096	0.009	2.1E-26	trans
1140	IL32	P24001	Interleukin-32	rs45499297	3115272	16	T	C	0.906	0.395	0.013	6.7E-216	cis
1140	IL32	P24001	Interleukin-32	rs247617	56990716	16	A	C	0.324	0.104	0.008	1.4E-40	trans
1140	IL32	P24001	Interleukin-32	rs12975366	54759361	19	T	C	0.605	0.070	0.007	1.9E-21	trans
1141	IL33	O95760	Interleukin-33	rs185705622	6291948	9	G	C	0.995	0.388	0.057	1.3E-11	cis
1142	IL34	Q6ZMJ4	Interleukin-34	rs4985556	70694000	16	C	A	0.878	1.070	0.012	0.0E+00	cis
1143	IL36A	Q9UHA7	Interleukin-36 alpha	rs895497	113763575	2	A	G	0.297	0.063	0.008	1.5E-13	cis
1144	IL36G	Q9NZH8	Interleukin-36 gamma	rs61816761	152285861	1	A	G	0.024	0.256	0.027	9.8E-22	trans
1144	IL36G	Q9NZH8	Interleukin-36 gamma	rs13410389	113738023	2	G	A	0.838	0.107	0.010	9.2E-25	cis
1145	IL3RA	P26951	Interleukin-3 receptor subunit alpha	rs876038	50308527	7	C	T	0.684	0.055	0.007	1.4E-13	trans
1145	IL3RA	P26951	Interleukin-3 receptor subunit alpha	rs2519093	136141870	9	C	T	0.816	0.592	0.010	0.0E+00	trans
1145	IL3RA	P26951	Interleukin-3 receptor subunit alpha	rs35458154	126296825	11	A	G	0.026	0.245	0.022	1.8E-28	trans
1145	IL3RA	P26951	Interleukin-3 receptor subunit alpha	rs186021206	7069412	17	A	G	0.006	0.748	0.048	2.4E-55	trans
1146	IL4R	P24394	Interleukin-4 receptor subunit alpha	rs73133996	98429219	3	G	C	0.561	0.054	0.007	7.5E-14	trans
1146	IL4R	P24394	Interleukin-4 receptor subunit alpha	rs2519093	136141870	9	C	T	0.816	0.086	0.009	2.1E-20	trans
1146	IL4R	P24394	Interleukin-4 receptor subunit alpha	rs28929474	94844947	14	T	C	0.021	0.482	0.025	2.2E-80	trans
1146	IL4R	P24394	Interleukin-4 receptor subunit alpha	rs8060025	27327214	16	G	T	0.612	0.314	0.008	0.0E+00	cis
1146	IL4R	P24394	Interleukin-4 receptor subunit alpha	rs186021206	7069412	17	A	G	0.006	0.631	0.050	4.3E-37	trans
1147	IL5RA	Q01344	Interleukin-5 receptor subunit alpha	rs188468174	25291697	1	C	T	0.986	0.716	0.029	5.3E-137	trans
1147	IL5RA	Q01344	Interleukin-5 receptor subunit alpha	rs77400868	3150964	3	G	A	0.133	0.517	0.011	0.0E+00	cis
1147	IL5RA	Q01344	Interleukin-5 receptor subunit alpha	rs9819371	141206800	3	C	T	0.935	0.106	0.015	1.0E-12	trans
1147	IL5RA	Q01344	Interleukin-5 receptor subunit alpha	rs9869437	196228360	3	A	C	0.351	0.076	0.008	1.1E-22	trans
1147	IL5RA	Q01344	Interleukin-5 receptor subunit alpha	rs93059	103468518	4	G	A	0.563	0.056	0.007	2.3E-16	trans
1147	IL5RA	Q01344	Interleukin-5 receptor subunit alpha	rs6810926	146968852	4	G	A	0.171	0.075	0.009	3.5E-17	trans
1147	IL5RA	Q01344	Interleukin-5 receptor subunit alpha	rs3134745	31242762	6	C	T	0.673	0.124	0.007	3.1E-66	trans
1147	IL5RA	Q01344	Interleukin-5 receptor subunit alpha	rs66867810	41940886	6	G	A	0.678	0.056	0.007	6.1E-15	trans
1147	IL5RA	Q01344	Interleukin-5 receptor subunit alpha	rs7758383	143169723	6	A	G	0.512	0.053	0.007	3.2E-15	trans
1147	IL5RA	Q01344	Interleukin-5 receptor subunit alpha	rs7797255	50351604	7	A	G	0.600	0.068	0.007	4.0E-23	trans
1147	IL5RA	Q01344	Interleukin-5 receptor subunit alpha	rs78740585	150944302	7	A	G	0.114	0.194	0.011	9.2E-76	trans
1147	IL5RA	Q01344	Interleukin-5 receptor subunit alpha	rs2511713	103577865	8	A	G	0.735	0.065	0.008	7.5E-18	trans
1147	IL5RA	Q01344	Interleukin-5 receptor subunit alpha	rs12342831	33124872	9	T	C	0.746	0.054	0.008	1.4E-12	trans
1147	IL5RA	Q01344	Interleukin-5 receptor subunit alpha	rs10896045	65555524	11	A	G	0.297	0.050	0.007	6.3E-12	trans
1147	IL5RA	Q01344	Interleukin-5 receptor subunit alpha	rs12880641	103251452	14	T	G	0.340	0.060	0.007	2.0E-17	trans
1147	IL5RA	Q01344	Interleukin-5 receptor subunit alpha	rs3803800	7462969	17	A	G	0.210	0.072	0.008	2.0E-18	trans
1147	IL5RA	Q01344	Interleukin-5 receptor subunit alpha	rs34562254	16842991	17	A	G	0.097	0.194	0.011	3.4E-66	trans
1147	IL5RA	Q01344	Interleukin-5 receptor subunit alpha	rs4794067	45808828	17	T	C	0.741	0.052	0.008	1.5E-11	trans
1147	IL5RA	Q01344	Interleukin-5 receptor subunit alpha	rs1005980	19271498	19	C	G	0.538	0.074	0.007	8.1E-28	trans
1148	IL6	P05231	Interleukin-6	rs55709272	113867288	2	C	T	0.439	0.053	0.007	5.8E-13	trans
1148	IL6	P05231	Interleukin-6	rs12133641	154428283	1	G	A	0.406	0.231	0.008	2.6E-206	trans
1148	IL6	P05231	Interleukin-6	rs9977672	40463283	21	G	A	0.741	0.060	0.008	1.9E-12	trans
1149	IL6R	P08887	Interleukin-6 receptor subunit alpha	rs4129267	154426264	1	T	C	0.404	1.105	0.008	0.0E+00	cis
1149	IL6R	P08887	Interleukin-6 receptor subunit alpha	rs9272460	32605583	6	G	A	0.359	0.037	0.005	1.1E-13	trans
1149	IL6R	P08887	Interleukin-6 receptor subunit alpha	rs4055121	126232337	11	C	T	0.865	0.052	0.007	1.0E-13	trans
1149	IL6R	P08887	Interleukin-6 receptor subunit alpha	rs186021206	7069412	17	A	G	0.006	0.426	0.032	1.4E-39	trans
1149	IL6R	P08887	Interleukin-6 receptor subunit alpha	rs123698	807442	19	C	G	0.605	0.034	0.005	1.1E-12	trans
1150	IL6ST	P40189	Interleukin-6 receptor subunit beta	rs3791101	44366250	1	A	G	0.308	0.059	0.008	6.8E-14	trans
1150	IL6ST	P40189	Interleukin-6 receptor subunit beta	rs10935473	98416900	3	G	T	0.560	0.174	0.007	4.5E-122	trans
1150	IL6ST	P40189	Interleukin-6 receptor subunit beta	rs11739016	55263373	5	T	C	0.119	0.261	0.011	3.6E-117	cis
1150	IL6ST	P40189	Interleukin-6 receptor subunit beta	rs4721064	12284378	7	A	G	0.402	0.051	0.007	6.6E-12	trans
1150	IL6ST	P40189	Interleukin-6 receptor subunit beta	rs507666	136149399	9	G	A	0.815	0.215	0.009	2.8E-114	trans
1150	IL6ST	P40189	Interleukin-6 receptor subunit beta	rs11011672	20205065	10	T	G	0.703	0.061	0.008	6.2E-14	trans
1150	IL6ST	P40189	Interleukin-6 receptor subunit beta	rs4055121	126232337	11	C	T	0.866	0.149	0.011	1.7E-43	trans
1150	IL6ST	P40189	Interleukin-6 receptor subunit beta	rs186021206	7069412	17	A	G	0.006	1.210	0.050	3.9E-129	trans
1150	IL6ST	P40189	Interleukin-6 receptor subunit beta	rs11444782	3680463	20	A	AT	0.442	0.055	0.007	1.1E-13	trans
1150	IL6ST	P40189	Interleukin-6 receptor subunit beta	rs2008174	39860130	22	C	T	0.260	0.075	0.009	1.1E-18	trans
1151	IL7	P13232	Interleukin-7	rs3804749	122833003	3	C	T	0.407	0.065	0.008	4.1E-17	trans
1151	IL7	P13232	Interleukin-7	rs3207561	31236685	6	G	A	0.375	0.054	0.008	3.8E-12	trans
1151	IL7	P13232	Interleukin-7	rs146201210	79748057	8	C	T	0.905	0.183	0.013	3.8E-45	cis
1151	IL7	P13232	Interleukin-7	rs6993770	106581528	8	A	T	0.713	0.105	0.008	6.1E-36	trans
1152	IL7R	P16871	Interleukin-7 receptor subunit alpha	rs34556682	27186149	1	A	G	0.919	0.082	0.011	1.0E-14	trans
1152	IL7R	P16871	Interleukin-7 receptor subunit alpha	rs3791101	44366250	1	A	G	0.308	0.054	0.006	1.0E-17	trans
1152	IL7R	P16871	Interleukin-7 receptor subunit alpha	rs9850919	169177924	3	T	C	0.594	0.049	0.006	2.6E-16	trans
1152	IL7R	P16871	Interleukin-7 receptor subunit alpha	rs13071610	186672937	3	G	A	0.883	0.061	0.009	1.6E-11	trans
1152	IL7R	P16871	Interleukin-7 receptor subunit alpha	rs6897932	35874575	5	C	T	0.729	0.889	0.008	0.0E+00	cis
1152	IL7R	P16871	Interleukin-7 receptor subunit alpha	rs12189871	31251924	6	T	C	0.092	0.160	0.010	3.2E-57	trans
1152	IL7R	P16871	Interleukin-7 receptor subunit alpha	rs170622	35032100	7	C	T	0.792	0.053	0.007	8.3E-14	trans
1152	IL7R	P16871	Interleukin-7 receptor subunit alpha	rs2519093	136141870	9	C	T	0.816	0.593	0.008	0.0E+00	trans
1152	IL7R	P16871	Interleukin-7 receptor subunit alpha	rs1059091	309127	11	A	G	0.682	0.042	0.006	1.2E-11	trans

1152	IL7R	P16871	Interleukin-7 receptor subunit alpha	rs35166255	126301756	11	A	G	0.034	0.464	0.016	3.1E-181	trans
1152	IL7R	P16871	Interleukin-7 receptor subunit alpha	rs186021206	7069412	17	A	G	0.006	1.125	0.040	2.4E-175	trans
1153	ILKAP	Q9H0C8	Integrin-linked kinase-associated serine/threonine phosphatase 2C	rs6993770	106581528	8	A	T	0.713	0.062	0.008	1.9E-13	trans
1154	IMMT	Q16891	MICOS complex subunit MIC60	rs35835517	106471857	14	G	T	0.852	0.080	0.010	2.2E-14	trans
1155	IMPA1	P29218	Inositol monophosphatase 1	rs342298	106373646	7	C	T	0.546	0.051	0.008	1.6E-11	trans
1155	IMPA1	P29218	Inositol monophosphatase 1	rs2400589	82575783	8	C	T	0.703	0.212	0.008	8.6E-145	cis
1156	IMPACT	Q9P2X3	Protein IMPACT	rs1354034	56849749	3	C	T	0.604	0.067	0.007	3.9E-19	trans
1157	IMPG1	Q17R60	Interphotoreceptor matrix proteoglycan 1	rs58534292	106483584	14	C	T	0.650	0.057	0.008	3.4E-12	trans
1157	IMPG1	Q17R60	Interphotoreceptor matrix proteoglycan 1	rs112765826	106427068	14	G	A	0.638	0.065	0.008	2.9E-14	trans
1158	ING1	Q9UK53	Inhibitor of growth protein 1	rs6993770	106581528	8	A	T	0.713	0.059	0.008	3.0E-12	trans
1159	INHBB	P09529	Inhibin beta B chain	rs17050272	121306440	2	A	G	0.412	0.365	0.007	0.0E+00	cis
1159	INHBB	P09529	Inhibin beta B chain	rs9375702	130384187	6	C	T	0.307	0.054	0.008	2.1E-12	trans
1159	INHBB	P09529	Inhibin beta B chain	rs7826120	59371725	8	C	T	0.670	0.076	0.008	1.6E-23	trans
1159	INHBB	P09529	Inhibin beta B chain	rs28929474	94844947	14	T	C	0.021	0.269	0.025	9.0E-27	trans
1159	INHBB	P09529	Inhibin beta B chain	rs56325564	45766771	17	A	G	0.483	0.064	0.007	9.4E-19	trans
1160	INHBC	P55103	Inhibin beta C chain	rs1730858	107619244	1	C	T	0.651	0.054	0.007	1.2E-13	trans
1160	INHBC	P55103	Inhibin beta C chain	rs1260326	27730940	2	T	C	0.392	0.062	0.007	9.4E-19	trans
1160	INHBC	P55103	Inhibin beta C chain	rs204893	32094593	6	C	T	0.431	0.050	0.007	6.8E-13	trans
1160	INHBC	P55103	Inhibin beta C chain	rs10822145	64934548	10	C	T	0.528	0.054	0.007	5.7E-15	trans
1160	INHBC	P55103	Inhibin beta C chain	rs28929474	94844947	14	T	C	0.021	0.190	0.024	3.6E-15	trans
1161	INPP1	P49441	Inositol polyphosphate 1-phosphatase	rs342298	106373646	7	C	T	0.546	0.053	0.008	1.6E-12	trans
1162	INPP5J	Q15735	Phosphatidylinositol 4,5-bisphosphate 5-phosphatase A	rs13306780	42329004	17	C	A	0.702	0.068	0.008	1.2E-16	trans
1163	INSL3	P51460	Insulin-like 3	rs2011425	234627608	2	T	G	0.921	0.062	0.009	1.7E-12	trans
1163	INSL3	P51460	Insulin-like 3	rs7763473	131146425	6	T	C	0.691	0.049	0.005	1.4E-21	trans
1164	INSL4	Q14641	Early placenta insulin-like peptide	rs80293268	8207579	1	G	C	0.952	0.133	0.018	4.9E-13	trans
1164	INSL4	Q14641	Early placenta insulin-like peptide	rs7806596	64039465	7	G	A	0.558	0.097	0.008	7.7E-38	trans
1164	INSL4	Q14641	Early placenta insulin-like peptide	rs41313766	5231360	9	C	T	0.983	0.652	0.030	7.1E-107	cis
1165	INSL5	Q9Y5Q6	Insulin-like peptide INSL5	rs11809759	67269905	1	G	A	0.399	0.180	0.007	4.2E-130	cis
1165	INSL5	Q9Y5Q6	Insulin-like peptide INSL5	rs4849328	111755538	2	C	G	0.446	0.074	0.007	6.4E-24	trans
1165	INSL5	Q9Y5Q6	Insulin-like peptide INSL5	rs12365875	4721934	11	T	C	0.856	0.146	0.010	2.5E-45	trans
1165	INSL5	Q9Y5Q6	Insulin-like peptide INSL5	rs10423928	46182304	19	A	T	0.190	0.069	0.009	4.8E-14	trans
1166	INSR	P06213	Insulin receptor	rs61804210	161670827	1	T	C	0.105	0.085	0.013	1.5E-11	trans
1166	INSR	P06213	Insulin receptor	rs112635299	94838142	14	T	G	0.022	0.184	0.026	3.1E-12	trans
1166	INSR	P06213	Insulin receptor	rs7197010	30495657	16	C	T	0.541	0.055	0.008	1.1E-12	trans
1166	INSR	P06213	Insulin receptor	rs704	26694861	17	G	A	0.526	0.060	0.008	8.4E-15	trans
1166	INSR	P06213	Insulin receptor	rs11658693	33800249	17	G	A	0.080	0.098	0.014	3.7E-12	trans
1166	INSR	P06213	Insulin receptor	rs1801689	64210580	17	C	A	0.030	0.195	0.022	2.6E-18	trans
1166	INSR	P06213	Insulin receptor	rs760459	46328835	21	T	A	0.791	0.064	0.009	1.6E-11	trans
1167	IPCEF1	Q8WWN9	Interactor protein for cytohesin exchange factors 1	rs1354034	56849749	3	C	T	0.604	0.064	0.008	4.9E-17	trans
1167	IPCEF1	Q8WWN9	Interactor protein for cytohesin exchange factors 1	rs883273	154630541	6	A	C	0.322	0.084	0.008	1.0E-25	cis
1168	IQGAP2	Q13576	Ras GTPase-activating-like protein IQGAP2	rs10037254	75930640	5	A	G	0.604	0.145	0.008	5.0E-80	cis
1169	IRAG2	Q12912	Inositol 1,4,5-triphosphate receptor associated 2	rs7080536	115348046	10	G	A	0.956	0.128	0.019	5.8E-12	trans
1169	IRAG2	Q12912	Inositol 1,4,5-triphosphate receptor associated 2	rs72932837	64876740	11	C	T	0.759	0.076	0.009	3.8E-18	trans
1170	IRAK1	P51617	Interleukin-1 receptor-associated kinase 1	rs1354034	56849749	3	C	T	0.604	0.058	0.008	1.6E-14	trans
1171	ISLR2	Q6UXK2	Immunoglobulin superfamily containing leucine-rich repeat protein 2	rs13135092	103198082	4	G	A	0.083	0.089	0.013	1.7E-12	trans
1171	ISLR2	Q6UXK2	Immunoglobulin superfamily containing leucine-rich repeat protein 2	rs9263451	31040488	6	C	A	0.895	0.080	0.011	1.8E-12	trans
1171	ISLR2	Q6UXK2	Immunoglobulin superfamily containing leucine-rich repeat protein 2	rs2519093	136141870	9	C	T	0.816	0.649	0.010	0.0E+00	trans
1171	ISLR2	Q6UXK2	Immunoglobulin superfamily containing leucine-rich repeat protein 2	rs60843925	126238832	11	T	C	0.864	0.130	0.010	7.5E-37	trans
1171	ISLR2	Q6UXK2	Immunoglobulin superfamily containing leucine-rich repeat protein 2	rs34868798	74469716	15	C	T	0.224	0.246	0.008	5.4E-185	cis
1171	ISLR2	Q6UXK2	Immunoglobulin superfamily containing leucine-rich repeat protein 2	rs186021206	7069412	17	A	G	0.006	0.953	0.048	1.2E-88	trans
1171	ISLR2	Q6UXK2	Immunoglobulin superfamily containing leucine-rich repeat protein 2	rs4803462	41908936	19	A	T	0.607	0.061	0.007	7.4E-18	trans
1172	ISM1	B1AKI9	Isthmin-1	rs539657009	161628456	1	T	C	0.129	0.077	0.011	5.2E-12	trans
1172	ISM1	B1AKI9	Isthmin-1	rs7641117	194064343	3	G	C	0.669	0.059	0.008	2.9E-14	trans
1172	ISM1	B1AKI9	Isthmin-1	rs1998123	13475884	20	A	G	0.303	0.187	0.008	2.2E-119	cis
1173	IST1	P53990	IST1 homolog	rs1354034	56849749	3	C	T	0.605	0.053	0.008	3.9E-12	trans
1173	IST1	P53990	IST1 homolog	rs342298	106373646	7	C	T	0.545	0.053	0.008	2.5E-12	trans
1173	IST1	P53990	IST1 homolog	rs10733789	64948684	10	C	T	0.313	0.061	0.008	1.1E-13	trans
1173	IST1	P53990	IST1 homolog	rs79258602	71961535	16	A	C	0.977	0.257	0.025	6.2E-25	cis
1174	ITGA11	Q9UKX5	Integrin alpha-11	rs1260326	27730940	2	C	T	0.609	0.062	0.007	1.1E-16	trans
1174	ITGA11	Q9UKX5	Integrin alpha-11	rs7111242	116695780	11	A	G	0.118	0.120	0.011	7.7E-26	trans
1174	ITGA11	Q9UKX5	Integrin alpha-11	rs35887873	126219396	11	C	T	0.228	0.120	0.009	9.6E-44	trans
1174	ITGA11	Q9UKX5	Integrin alpha-11	rs2306022	68628163	15	C	T	0.910	0.359	0.013	2.3E-169	cis
1174	ITGA11	Q9UKX5	Integrin alpha-11	rs186021206	7069412	17	A	G	0.006	0.794	0.050	1.6E-56	trans
1174	ITGA11	Q9UKX5	Integrin alpha-11	rs3092635	44542093	20	AT	A	0.848	0.087	0.010	2.4E-17	trans
1175	ITGA2	P17301	Integrin alpha-2	rs1354034	56849749	3	C	T	0.604	0.047	0.007	7.2E-13	trans
1175	ITGA2	P17301	Integrin alpha-2	rs12245149	65321147	10	C	A	0.512	0.049	0.006	1.5E-14	trans
1175	ITGA2	P17301	Integrin alpha-2	rs6589572	116681563	11	G	A	0.124	0.142	0.010	1.6E-48	trans
1175	ITGA2	P17301	Integrin alpha-2	rs17580	94847262	14	A	T	0.050	0.263	0.015	8.6E-71	trans
1175	ITGA2	P17301	Integrin alpha-2	rs438811	45416741	19	T	C	0.237	0.091	0.008	1.2E-33	trans
1175	ITGA2	P17301	Integrin alpha-2	rs2868346	44547970	20	C	T	0.240	0.093	0.007	1.1E-35	trans
1176	ITGA5	P08648	Integrin alpha-5	rs539657009	161628456	1	T	C	0.130	0.095	0.011	2.4E-17	trans
1176	ITGA5	P08648	Integrin alpha-5	rs2124440	182328214	2	A	G	0.556	0.059	0.007	9.2E-16	trans
1176	ITGA5	P08648	Integrin alpha-5	rs139078629	216251538	2	G	A	0.990	0.693	0.037	4.1E-77	trans
1176	ITGA5	P08648	Integrin alpha-5	rs56185965	47580158	3	T	A	0.839	0.069	0.010	9.8E-12	trans
1176	ITGA5	P08648	Integrin alpha-5	rs78776915	32469380	6	T	C	0.416	0.065	0.008	4.1E-16	trans
1176	ITGA5	P08648	Integrin alpha-5	rs41341748	16012594	8	A	G	0.011	0.240	0.035	5.9E-12	trans
1176	ITGA5	P08648	Integrin alpha-5	rs8176746	136131322	9	T	G	0.061	0.228	0.015	1.9E-51	trans
1176	ITGA5	P08648	Integrin alpha-5	rs10748526	82273079	10	C	T	0.795	0.118	0.009	1.6E-39	trans
1176	ITGA5	P08648	Integrin alpha-5	rs7161799	58770523	15	T	C	0.077	0.092	0.014	1.7E-11	trans
1176	ITGA5	P08648	Integrin alpha-5	rs186021206	7069412	17	A	G	0.006	0.707	0.050	1.2E-45	trans
1176	ITGA5	P08648	Integrin alpha-5	rs7288265	44423396	22	G	A	0.671	0.076	0.008	1.7E-22	trans
1177	ITGA6	P23229	Integrin alpha-6	rs6714597	173338486	2	C	T	0.308	0.231	0.008	1.9E-176	cis
1177	ITGA6	P23229	Integrin alpha-6	rs5030062	186454180	3	C	A	0.373	0.076	0.008	6.1E-23	trans
1177	ITGA6	P23229	Integrin alpha-6	rs4861708	187157233	4	A	G	0.512	0.170	0.007	8.1E-114	trans
1177	ITGA6	P23229	Integrin alpha-6	rs2897457	52352376	5	G	T	0.509	0.122	0.007	8.6E-61	trans
1177	ITGA6	P23229	Integrin alpha-6	rs2731673	176839898	5	C	T	0.745	0.086	0.008	3.7E-24	trans
1177	ITGA6	P23229	Integrin alpha-6	rs579459	136154168	9	T	C	0.793	0.104	0.009	1.0E-29	trans
1177	ITGA6	P23229	Integrin alpha-6	rs7896518	65104500	10	G	A	0.424	0.063	0.008	1.2E-16	trans
1178	ITGAL	P20701	Integrin alpha-L	rs3856240	25892525	1	C	T	0.464	0.057	0.008	5.5E-14	trans
1178	ITGAL	P20701	Integrin alpha-L	rs11574938	30485393	16	G	C	0.484	0.156	0.008	2.9E-94	cis
1178	ITGAL	P20701	Integrin alpha-L	rs760462	46328099	21	C	T	0.818	0.067	0.010	5.1E-12	trans
1179	ITGAM	P11215	Integrin alpha-M	rs10164604	135054366	2	A	T	0.900	0.083	0.012	1.7E-11	trans
1179	ITGAM	P11215	Integrin alpha-M	rs118162691	21767809	8	A	C	0.044	0.143	0.018	3.1E-15	trans
1179	ITGAM	P11215	Integrin alpha-M	rs7865362	33117965	9	T	C	0.363	0.110	0.008	2.5E-45	trans
1179	ITGAM	P11215	Integrin alpha-M	rs4601794	126245145	11	A	G	0.941	0.112	0.016	4.2E-13	trans
1179	ITGAM	P11215	Integrin alpha-M	rs11150613	31357810	16	T	C	0.676	0.164	0.008	2.5E-95	cis
1179	ITGAM	P11215	Integrin alpha-M	rs186021206	7069412	17	A	G	0.006	0.996	0.050	9.1E-87	trans
1179	ITGAM	P11215	Integrin alpha-M	rs4804774	7772173	19	C	G	0.237	0.084	0.009	8.2E-21	trans
1179	ITGAM	P11215	Integrin alpha-M	rs2230531	46308800	21	T	C	0.008	0.487	0.042	6.5E-31	trans
1180	ITGAV	P06756	Integrin alpha-V	rs2595391	187532373	2	G	T	0.108	0.229	0.011	5.4E-88	cis
1180	ITGAV	P06756	Integrin alpha-V	rs60648773	126237281	11	G	A	0.773	0.067	0.009	2.6E-15	trans
1180	ITGAV	P06756	Integrin alpha-V	rs17580	94847262	14	A	T	0.050	0.120	0.016	1.9E-13	trans
1180	ITGAV	P06756	Integrin alpha-V	rs186021206	7069412	17	A	G	0.006	0.863	0.049	3.7E-70	trans
1181	ITGB1	P05556	Integrin beta-1	rs246411	52276458	5	C	T	0.319	0.099	0.008	9.7E-38	trans
1181	ITGB1	P05556	Integrin beta-1	rs7070761	65317056	10	A	T	0.512	0.049	0.007	6.9E-12	trans
1181	ITGB1	P05556	Integrin beta-1	rs11220462	126243952	11	G	A	0.867	0.128	0.011	3.2E-33	trans
1181	ITGB1	P05556	Integrin beta-1	rs186021206	7069412	17	A	G	0.006	1.094	0.050	1.1E-107	trans
1181	ITGB1	P05556	Integrin beta-1	rs12484278	39873937	22	A	G	0.195	0.064	0.009	2.6E-12	trans
1182	ITGB1BP2	Q9UKP3	Integrin beta-1-binding protein 2	rs2631367	131705458	5	G	C	0.519	0.052	0.008	8.9E-12	trans
1182	ITGB1BP2	Q9UKP3	Integrin beta-1-binding protein 2	rs342298	106373646	7	C	T	0.545	0.098	0.008	4.8E-38	trans
1182	ITGB1BP2	Q9UKP3	Integrin beta-1-binding protein 2	rs13338372	9038779	16	A	G	0.810	0.067	0.010	4.7E-12	trans
1183	ITGB2	P05107	Integrin beta-2	rs7433300	47120640	3	G	A	0.385	0.051	0.007	5.7E-12	trans
1183	ITGB2	P05107	Integrin beta-2	rs118162691	21767809	8	A	C	0.043	0.142	0.018	1.7E-15	trans
1183	ITGB2	P05107	Integrin beta-2	rs7865362	33117965	9	T	C	0.363	0.089	0.008	1.5E-31	trans
1183	ITGB2	P05107	Integrin beta-2	rs56278466	17875857	10	G	T	0.662	0.066	0.008	4.6E-18	trans
1183	ITGB2	P05107	Integrin beta-2	rs4601794	126245145	11	A	G	0.941	0.119	0.015	7.7E-15	trans
1183	ITGB2	P05107	Integrin beta-2	rs11574639	31372081	16	A	G	0.684	0.123	0.008	4.0E-56	trans
1183	ITGB2	P05107	Integrin beta-2	rs186021206	7069412	17	A	G	0.006	1.112	0.050	1.7E-109	trans
1183	ITGB2	P05107	Integrin beta-2	rs57543961	33802931	17	C	CAT	0.584	0.051	0.007	9.0E-12	trans
1183	ITGB2	P05107	Integrin beta-2	rs760462	46328099	21	C	T	0.818	0.149	0.009	5.2E-57	cis
1184	ITGB5	P18084	Integrin beta-5	rs35986780	187588880	2	A	G	0.703	0.060	0.008	7.6E-14	trans
1184	ITGB5	P18084	Integrin beta-5	rs1354034	56849749	3	C	T	0.604	0.077	0.007	3.6E-25	trans
1184	ITGB5	P18084	Integrin beta-5	rs185447638	124526716	3	A	G	0.019	0.297	0.027	2.9E-28	cis
1184	ITGB5	P18084	Integrin beta-5	rs56399423	131672657	5	T	C	0.565	0.050	0.007	8.0E-12	trans
1184	ITGB5	P18084	Integrin beta-5	rs7896518	65104500	10	G	A	0.424	0.092	0.007	3.0E-35	trans
1184	ITGB5	P18084	Integrin beta-5	rs186021206	7069412	17	A	G	0.006	0.524	0.050	1.2E-25	trans
1185	ITGB6	P18564	Integrin beta-6	rs6432601	160977943	2	G	A	0.693	0.289	0.008	3.9E-302	cis
1185	ITGB6	P18564	Integrin beta-6	rs13107325	103188709	4	C	T	0.925	0.136	0.013	6.0E-24	trans
1185	ITGB6	P18564	Integrin beta-6	rs6471893	61549610	8	G	C	0.241	0.068	0.008	3.7E-16	trans
1185	ITGB6	P18564	Integrin beta-6	rs4382584	139386138	9	A	G	0.275	0.080	0.008	3.8E-24	trans
1185	ITGB6	P18564	Integrin beta-6	rs56278466	17875857	10	G	T	0.661	0.053	0.007	8.1E-13	trans
1185	ITGB6	P18564	Integrin beta-6	rs75348693	50031930	14	A	G	0.546	0.064	0.007	1.3E-19	trans
1185	ITGB6	P18564	Integrin beta-6	rs77542162	67081278	17	G	A	0.023	0.203	0.024	5.8E-18	trans
1186	ITGB7	P26010	Integrin beta-7	rs37454	44294411	1	A	C	0.353	0.171	0.007	7.8E-121	trans
1186	ITGB7	P26010	Integrin beta-7	rs343808	111330007	1	T	C	0.242	0.056	0.008	1.2E-11	trans
1186	ITGB7	P26010	Integrin beta-7	rs6759003	62559205	2	C	T	0.640	0.077	0.007	9.0E-26	trans
1186	ITGB7	P26010	Integrin beta-7	rs62168051	135083003	2	G	C	0.157	0.210	0.010	3.2E-106	trans
1186	ITGB7	P26010	Integrin beta-7	rs6771051	169724228	3	G	T	0.544	0.108	0.007	1.5E-51	trans
1186	ITGB7	P26010	Integrin beta-7	rs4686445	186627608	3	T	C	0.682	0.129	0.008	1.0E-64	trans
1186	ITGB7	P26010	Integrin beta-7	rs6796	6502367	7	C	T	0.279	0.067	0.008	5.5E-18	trans
1186	ITGB7	P26010	Integrin beta-7	rs6955795	150508625	7	G	C	0.453	0.055	0.007	6.8E-15	trans
1186	ITGB7	P26010	Integrin beta-7	rs7865745	33140976	9	A	G	0.311	0.070	0.008	2.4E-20	trans
1186	ITGB7	P26010	Integrin beta-7	rs635634	136155000	9	T	C	0.184	0.106	0.009	3.5E-31	trans

1186	ITGB7	P26010	Integrin beta-7	rs78689694	126234820	11	G	C	0.867	0.144	0.010	5.2E-44	trans
1186	ITGB7	P26010	Integrin beta-7	rs7161799	58770523	15	T	C	0.077	0.138	0.013	1.2E-25	trans
1186	ITGB7	P26010	Integrin beta-7	rs9896869	3629848	17	A	G	0.453	0.354	0.007	0.0E+00	trans
1186	ITGB7	P26010	Integrin beta-7	rs186021206	7069412	17	A	G	0.006	1.815	0.051	6.5E-281	trans
1186	ITGB7	P26010	Integrin beta-7	rs2659005	79218714	17	T	C	0.442	0.086	0.007	3.3E-31	trans
1187	ITGBL1	O95965	Integrin beta-like protein 1	rs41341748	16012594	8	A	G	0.011	0.351	0.032	9.4E-28	trans
1187	ITGBL1	O95965	Integrin beta-like protein 1	rs12018094	101986692	13	G	T	0.812	0.363	0.009	0.0E+00	cis
1187	ITGBL1	O95965	Integrin beta-like protein 1	rs73045269	41825191	19	T	C	0.171	0.072	0.009	6.1E-16	trans
1187	ITGBL1	O95965	Integrin beta-like protein 1	rs738409	44324727	22	G	C	0.217	0.071	0.008	1.3E-18	trans
1188	ITIH1	P19827	Inter-alpha-trypsin inhibitor heavy chain H1	rs678	52820981	3	A	T	0.643	0.140	0.008	2.0E-69	cis
1189	ITIH3	Q06033	Inter-alpha-trypsin inhibitor heavy chain H3	rs1260326	27730940	2	T	C	0.392	0.055	0.007	3.5E-15	trans
1189	ITIH3	Q06033	Inter-alpha-trypsin inhibitor heavy chain H3	rs9881468	52828628	3	T	C	0.343	0.310	0.007	0.0E+00	cis
1189	ITIH3	Q06033	Inter-alpha-trypsin inhibitor heavy chain H3	rs35656703	126258285	3	C	A	0.749	0.203	0.008	2.2E-138	trans
1189	ITIH3	Q06033	Inter-alpha-trypsin inhibitor heavy chain H3	rs244451	129227377	5	T	C	0.319	0.081	0.007	1.5E-28	trans
1189	ITIH3	Q06033	Inter-alpha-trypsin inhibitor heavy chain H3	rs35246381	18272535	8	T	C	0.780	0.057	0.008	3.4E-12	trans
1189	ITIH3	Q06033	Inter-alpha-trypsin inhibitor heavy chain H3	rs28601761	126500031	8	G	C	0.419	0.055	0.007	2.1E-15	trans
1189	ITIH3	Q06033	Inter-alpha-trypsin inhibitor heavy chain H3	rs2073785	109698759	X	G	T	0.600	0.041	0.006	1.4E-12	trans
1190	ITIH4	Q14624	Inter-alpha-trypsin inhibitor heavy chain H4	rs1260326	27730940	2	T	C	0.392	0.094	0.007	2.1E-38	trans
1190	ITIH4	Q14624	Inter-alpha-trypsin inhibitor heavy chain H4	rs2710331	52837855	3	C	T	0.342	0.248	0.008	1.9E-230	cis
1190	ITIH4	Q14624	Inter-alpha-trypsin inhibitor heavy chain H4	rs2731673	176839898	5	C	T	0.745	0.114	0.008	6.4E-45	trans
1191	ITIH5	Q86UX2	Inter-alpha-trypsin inhibitor heavy chain H5	rs41298373	7622009	10	A	G	0.100	0.169	0.013	6.9E-40	cis
1192	ITM2A	O43736	Integral membrane protein 2A	rs9264683	31240940	6	A	T	0.831	0.100	0.010	1.3E-23	trans
1192	ITM2A	O43736	Integral membrane protein 2A	rs709932	94849201	14	C	T	0.846	0.298	0.010	7.1E-177	trans
1192	ITM2A	O43736	Integral membrane protein 2A	rs1046294	35660546	19	T	C	0.603	0.063	0.008	1.7E-16	trans
1192	ITM2A	O43736	Integral membrane protein 2A	rs1736641	78607948	X	G	A	0.303	0.188	0.007	5.5E-168	cis
1193	ITPA	Q9BY32	Inosine triphosphate pyrophosphatase	rs1354034	56849749	3	C	T	0.604	0.047	0.007	1.6E-12	trans
1193	ITPA	Q9BY32	Inosine triphosphate pyrophosphatase	rs1127354	3193842	20	C	A	0.925	1.185	0.015	0.0E+00	cis
1194	ITPR1	Q14643	Inositol 1,4,5-trisphosphate receptor type 1	rs68066031	224880498	2	T	C	0.770	0.138	0.009	1.6E-51	trans
1194	ITPR1	Q14643	Inositol 1,4,5-trisphosphate receptor type 1	rs76604555	4856285	3	G	A	0.878	0.138	0.012	8.7E-32	cis
1194	ITPR1	Q14643	Inositol 1,4,5-trisphosphate receptor type 1	rs1354034	56849749	3	T	C	0.395	0.059	0.008	1.6E-14	trans
1194	ITPR1	Q14643	Inositol 1,4,5-trisphosphate receptor type 1	rs6993770	106581528	8	A	T	0.713	0.095	0.008	2.6E-29	trans
1194	ITPR1	Q14643	Inositol 1,4,5-trisphosphate receptor type 1	rs7080386	65048306	10	A	C	0.412	0.077	0.008	4.1E-23	trans
1194	ITPR1	Q14643	Inositol 1,4,5-trisphosphate receptor type 1	rs7172696	101991311	15	A	G	0.283	0.124	0.008	3.1E-48	trans
1194	ITPR1	Q14643	Inositol 1,4,5-trisphosphate receptor type 1	rs12445050	81870969	16	T	C	0.139	0.080	0.011	3.2E-13	trans
1194	ITPR1	Q14643	Inositol 1,4,5-trisphosphate receptor type 1	rs1654425	55538980	19	C	T	0.835	0.120	0.010	1.5E-31	trans
1195	JAM2	P57087	Junctional adhesion molecule B	rs3811444	248039451	1	T	C	0.333	0.154	0.008	1.0E-90	trans
1195	JAM2	P57087	Junctional adhesion molecule B	rs1260326	27730940	2	C	T	0.608	0.051	0.007	2.6E-12	trans
1195	JAM2	P57087	Junctional adhesion molecule B	rs8176741	136131461	9	A	G	0.061	0.248	0.015	1.3E-61	trans
1195	JAM2	P57087	Junctional adhesion molecule B	rs12277151	133973454	11	C	T	0.923	0.106	0.014	5.3E-15	trans
1195	JAM2	P57087	Junctional adhesion molecule B	rs36060036	20361950	16	C	T	0.833	0.077	0.010	8.5E-16	trans
1196	JAM3	Q9BX67	Junctional adhesion molecule C	rs3811444	248039451	1	C	T	0.668	0.291	0.008	2.6E-284	trans
1196	JAM3	Q9BX67	Junctional adhesion molecule C	rs1354034	56849749	3	C	T	0.604	0.087	0.008	4.2E-30	trans
1196	JAM3	Q9BX67	Junctional adhesion molecule C	rs3804749	122833003	3	C	T	0.407	0.058	0.008	6.0E-14	trans
1196	JAM3	Q9BX67	Junctional adhesion molecule C	rs6993770	106581528	8	A	T	0.713	0.115	0.008	1.0E-43	trans
1196	JAM3	Q9BX67	Junctional adhesion molecule C	rs7896518	65104500	10	G	A	0.424	0.067	0.008	3.8E-18	trans
1196	JAM3	Q9BX67	Junctional adhesion molecule C	rs94499	134023728	11	G	A	0.219	0.123	0.009	7.9E-42	cis
1196	JAM3	Q9BX67	Junctional adhesion molecule C	rs892090	55539072	19	G	T	0.834	0.086	0.010	1.6E-17	trans
1197	JCHAIN	P01591	Immunoglobulin J chain	rs188468174	25291697	1	T	C	0.014	0.358	0.032	1.3E-28	trans
1197	JCHAIN	P01591	Immunoglobulin J chain	rs139015452	111807596	2	C	CACTG	0.770	0.069	0.009	4.9E-14	trans
1197	JCHAIN	P01591	Immunoglobulin J chain	rs617119	32455767	3	T	C	0.513	0.059	0.008	6.7E-15	trans
1197	JCHAIN	P01591	Immunoglobulin J chain	rs116446171	484453	6	G	C	0.021	0.211	0.026	1.1E-15	trans
1197	JCHAIN	P01591	Immunoglobulin J chain	rs1264457	30458064	6	G	A	0.393	0.082	0.008	8.0E-26	trans
1197	JCHAIN	P01591	Immunoglobulin J chain	rs62445869	50477611	7	G	A	0.725	0.061	0.008	9.2E-13	trans
1197	JCHAIN	P01591	Immunoglobulin J chain	rs2511710	103575949	8	G	T	0.735	0.087	0.009	4.6E-24	trans
1197	JCHAIN	P01591	Immunoglobulin J chain	rs9658720	90760669	10	T	TG	0.446	0.059	0.008	1.6E-14	trans
1197	JCHAIN	P01591	Immunoglobulin J chain	rs36051450	123344959	11	C	T	0.196	0.084	0.010	3.6E-18	trans
1197	JCHAIN	P01591	Immunoglobulin J chain	rs8051050	30687909	16	G	A	0.260	0.084	0.009	1.3E-22	trans
1197	JCHAIN	P01591	Immunoglobulin J chain	rs4815598	3701425	20	C	G	0.544	0.058	0.008	1.1E-14	trans
1198	JPT2	Q9H910	Jupiter microtubule associated homolog 2	rs1354034	56849749	3	C	T	0.605	0.069	0.008	1.9E-19	trans
1198	JPT2	Q9H910	Jupiter microtubule associated homolog 2	rs342298	106373646	7	C	T	0.545	0.056	0.008	1.6E-13	trans
1199	KAZALD1	Q96I82	Kazal-type serine protease inhibitor domain-containing protein 1	rs7639267	52568805	3	T	G	0.560	0.062	0.007	5.6E-19	trans
1199	KAZALD1	Q96I82	Kazal-type serine protease inhibitor domain-containing protein 1	rs2228243	186395113	3	A	G	0.800	0.218	0.009	5.4E-139	trans
1199	KAZALD1	Q96I82	Kazal-type serine protease inhibitor domain-containing protein 1	rs3731211	21986847	9	T	A	0.279	0.062	0.008	1.5E-15	trans
1199	KAZALD1	Q96I82	Kazal-type serine protease inhibitor domain-containing protein 1	rs2519093	136141870	9	C	T	0.816	0.068	0.009	2.5E-14	trans
1199	KAZALD1	Q96I82	Kazal-type serine protease inhibitor domain-containing protein 1	rs11190812	102824292	10	G	A	0.960	1.696	0.019	0.0E+00	cis
1200	KAZN	Q674X7	Kazrin	rs1354034	56849749	3	C	T	0.605	0.101	0.008	3.0E-41	trans
1200	KAZN	Q674X7	Kazrin	rs342293	106372219	7	C	G	0.540	0.062	0.007	7.4E-17	trans
1201	KDM3A	Q9Y4C1	Lysine-specific demethylase 3A	rs4548048	32625022	6	T	C	0.103	0.141	0.013	8.9E-27	trans
1201	KDM3A	Q9Y4C1	Lysine-specific demethylase 3A	rs10957978	81285139	8	T	G	0.648	0.055	0.008	6.1E-12	trans
1201	KDM3A	Q9Y4C1	Lysine-specific demethylase 3A	rs12588969	103230758	14	G	C	0.316	0.059	0.008	6.7E-13	trans
1202	KDR	P35968	Vascular endothelial growth factor receptor 2	rs11682865	62617796	2	G	C	0.805	0.063	0.008	2.8E-14	trans
1202	KDR	P35968	Vascular endothelial growth factor receptor 2	rs10935473	98416900	3	G	T	0.560	0.158	0.007	4.8E-124	trans
1202	KDR	P35968	Vascular endothelial growth factor receptor 2	rs34231037	55972946	4	A	G	0.965	1.325	0.020	0.0E+00	cis
1202	KDR	P35968	Vascular endothelial growth factor receptor 2	rs9267947	32211218	6	A	G	0.522	0.070	0.007	4.2E-26	trans
1202	KDR	P35968	Vascular endothelial growth factor receptor 2	rs932589	137083138	6	C	T	0.596	0.056	0.007	1.5E-16	trans
1202	KDR	P35968	Vascular endothelial growth factor receptor 2	rs7012814	9173358	8	G	A	0.528	0.049	0.007	2.3E-13	trans
1202	KDR	P35968	Vascular endothelial growth factor receptor 2	rs13273990	18934020	8	C	T	0.413	0.046	0.007	5.6E-12	trans
1202	KDR	P35968	Vascular endothelial growth factor receptor 2	rs3110291	105981953	8	C	A	0.201	0.058	0.008	4.3E-12	trans
1202	KDR	P35968	Vascular endothelial growth factor receptor 2	rs2031610	90754487	10	T	C	0.639	0.050	0.007	1.8E-13	trans
1202	KDR	P35968	Vascular endothelial growth factor receptor 2	rs17625540	95184355	11	G	A	0.705	0.050	0.007	1.1E-11	trans
1202	KDR	P35968	Vascular endothelial growth factor receptor 2	rs35166255	126301756	11	A	G	0.034	0.249	0.018	1.7E-42	trans
1202	KDR	P35968	Vascular endothelial growth factor receptor 2	rs186021206	7069412	17	A	G	0.006	0.437	0.045	5.0E-22	trans
1203	KEL	P23276	Kell blood group glycoprotein	rs1427407	60718043	2	T	G	0.152	0.152	0.009	7.4E-62	trans
1203	KEL	P23276	Kell blood group glycoprotein	rs35176351	186654826	3	C	A	0.417	0.314	0.007	0.0E+00	trans
1203	KEL	P23276	Kell blood group glycoprotein	rs11752051	2379497	6	A	G	0.485	0.069	0.007	1.9E-25	trans
1203	KEL	P23276	Kell blood group glycoprotein	rs8176059	142651354	7	G	A	0.989	1.247	0.032	0.0E+00	cis
1203	KEL	P23276	Kell blood group glycoprotein	rs2519093	136141870	9	C	T	0.816	0.419	0.009	0.0E+00	trans
1203	KEL	P23276	Kell blood group glycoprotein	rs17476364	71094504	10	C	T	0.110	0.075	0.011	1.2E-12	trans
1203	KEL	P23276	Kell blood group glycoprotein	rs11023872	16234573	11	A	C	0.084	0.092	0.012	1.2E-14	trans
1203	KEL	P23276	Kell blood group glycoprotein	rs4055121	126232337	11	C	T	0.865	0.096	0.010	4.9E-23	trans
1203	KEL	P23276	Kell blood group glycoprotein	rs11630124	51015913	15	A	T	0.023	0.221	0.022	1.6E-23	trans
1203	KEL	P23276	Kell blood group glycoprotein	rs186021206	7069412	17	A	G	0.006	1.337	0.045	1.4E-191	trans
1203	KEL	P23276	Kell blood group glycoprotein	rs8887	4502201	19	T	C	0.426	0.065	0.007	6.6E-22	trans
1203	KEL	P23276	Kell blood group glycoprotein	rs492602	49206417	19	G	A	0.509	0.062	0.007	1.5E-20	trans
1203	KEL	P23276	Kell blood group glycoprotein	rs6014993	55991637	20	G	A	0.487	0.050	0.007	1.9E-14	trans
1204	KHK	P50053	Ketohexokinase	rs2304681	27315252	2	G	A	0.629	0.428	0.008	0.0E+00	cis
1204	KHK	P50053	Ketohexokinase	rs3747207	44324855	22	A	G	0.216	0.067	0.009	3.1E-14	trans
1205	KIAA0319	Q5VV43	Dyslexia-associated protein KIAA0319	rs3181238	24654443	6	T	C	0.640	0.308	0.008	0.0E+00	cis
1206	KIAA2013	Q8IYS2	Uncharacterized protein KIAA2013	rs71534539	32512754	6	G	A	0.535	0.064	0.008	3.9E-14	trans
1207	KIF1C	O43896	Kinesin-like protein KIF1C	rs148934699	4925475	17	C	T	0.994	0.543	0.049	4.9E-29	cis
1208	KIF22	Q14807	Kinesin-like protein KIF22	rs1354034	56849749	3	C	T	0.604	0.059	0.008	3.7E-14	trans
1208	KIF22	Q14807	Kinesin-like protein KIF22	rs7041446	137776054	9	A	G	0.459	0.062	0.008	6.4E-16	trans
1209	KIFBP	Q96EK5	KIF-binding protein	rs1354034	56849749	3	C	T	0.605	0.107	0.008	2.7E-44	trans
1209	KIFBP	Q96EK5	KIF-binding protein	rs342293	106372219	7	C	G	0.541	0.081	0.008	7.6E-27	trans
1209	KIFBP	Q96EK5	KIF-binding protein	rs11502185	180258	11	C	T	0.260	0.070	0.010	3.6E-13	trans
1209	KIFBP	Q96EK5	KIF-binding protein	rs58603603	64142608	15	A	G	0.923	0.102	0.014	5.3E-13	trans
1210	KIR2DL2	P43627	Killer cell immunoglobulin-like receptor 2DL2	rs30378	96121994	5	A	G	0.333	0.048	0.007	4.8E-13	trans
1210	KIR2DL2	P43627	Killer cell immunoglobulin-like receptor 2DL2	rs9264665	31239279	6	T	C	0.353	0.557	0.007	0.0E+00	trans
1210	KIR2DL2	P43627	Killer cell immunoglobulin-like receptor 2DL2	rs60955532	55253196	19	A	C	0.560	0.620	0.007	0.0E+00	cis
1211	KIR2DL3	P43628	Killer cell immunoglobulin-like receptor 2DL3	rs1050451	31239802	6	C	G	0.352	0.543	0.008	0.0E+00	trans
1211	KIR2DL3	P43628	Killer cell immunoglobulin-like receptor 2DL3	rs16985907	55246812	19	G	C	0.962	0.879	0.018	0.0E+00	cis
1212	KIR2DS4	P43632	Killer cell immunoglobulin-like receptor 2DS4	rs9264261	31223738	6	T	C	0.285	0.054	0.006	1.6E-20	trans
1212	KIR2DS4	P43632	Killer cell immunoglobulin-like receptor 2DS4	rs7255036	55338699	19	C	G	0.226	1.325	0.009	0.0E+00	cis
1213	KIR3DL1	P43629	Killer cell immunoglobulin-like receptor 3DL1	rs9259983	29904403	6	A	C	0.035	0.528	0.014	2.6E-305	trans
1213	KIR3DL1	P43629	Killer cell immunoglobulin-like receptor 3DL1	rs12150998	55343039	19	A	G	0.436	0.926	0.008	0.0E+00	cis
1214	KIR3DL2	P43630	Killer cell immunoglobulin-like receptor 3DL2	rs150240916	55348842	19	A	G	0.034	0.341	0.022	8.1E-55	cis
1215	KIRREL2	Q6UWL6	Kin of IRRE-like protein 2	rs6571015	96534478	6	A	G	0.791	0.152	0.009	1.5E-63	trans
1215	KIRREL2	Q6UWL6	Kin of IRRE-like protein 2	rs8176743	136131415	9	T	C	0.062	0.542	0.015	3.4E-270	trans
1215	KIRREL2	Q6UWL6	Kin of IRRE-like protein 2	rs421195	333146	16	C	T	0.892	0.306	0.012	2.0E-143	trans
1215	KIRREL2	Q6UWL6	Kin of IRRE-like protein 2	rs8045196	75256360	16	T	G	0.269	0.109	0.008	2.4E-38	trans
1215	KIRREL2	Q6UWL6	Kin of IRRE-like protein 2	rs1673931	88976477	16	C	T	0.379	0.067	0.008	8.3E-19	trans
1215	KIRREL2	Q6UWL6	Kin of IRRE-like protein 2	rs186021206	7069412	17	A	G	0.006	0.492	0.050	1.1E-22	trans
1215	KIRREL2	Q6UWL6	Kin of IRRE-like protein 2	rs404299	36349752	19	G	A	0.967	0.459	0.021	1.0E-110	cis
1215	KIRREL2	Q6UWL6	Kin of IRRE-like protein 2	rs2638280	49209318	19	G	A	0.463	0.066	0.007	9.5E-19	trans
1216	KIT	P10721	Mast/stem cell growth factor receptor Kit	rs115340020	92766438	1	G	A	0.972	0.174	0.021	3.6E-16	trans
1216	KIT	P10721	Mast/stem cell growth factor receptor Kit	rs2884425	234694483	1	C	T	0.506	0.070	0.007	1.3E-22	trans
1216	KIT	P10721	Mast/stem cell growth factor receptor Kit	rs74227709	247722588	1	A	G	0.074	0.160	0.014	1.1E-31	trans
1216	KIT	P10721	Mast/stem cell growth factor receptor Kit	rs12470153	223155911	2	G	C	0.340	0.054	0.008	1.0E-12	trans
1216	KIT	P10721	Mast/stem cell growth factor receptor Kit	rs6795463	69846108	3	G	T	0.569	0.060	0.007	4.9E-17	trans
1216	KIT	P10721	Mast/stem cell growth factor receptor Kit	rs6440573	148607919	3	G	C	0.950	0.252	0.016	5.6E-53	trans
1216	KIT	P10721	Mast/stem cell growth factor receptor Kit	rs6762074	197812370	3	G	A	0.667	0.079	0.008	1.4E-25	trans
1216	KIT	P10721	Mast/stem cell growth factor receptor Kit	rs218263	55408104	4	C	T	0.876	0.080	0.011	1.4E-13	cis
1216	KIT	P10721	Mast/stem cell growth factor receptor Kit	rs72704195	175490632	4	A	G	0.623	0.054	0.008	6.7E-13	trans
1216	KIT	P10721	Mast/stem cell growth factor receptor Kit	rs12203592	396321	6	C	T	0.783	0.108	0.009	4.3E-34	trans
1216	KIT	P10721	Mast/stem cell growth factor receptor Kit	rs2844572	31335575	6	A	C	0.657	0.058	0.007	7.0E-15	trans
1216	KIT	P10721	Mast/stem cell growth factor receptor Kit	rs12350739	16885017	9	G	A	0.388	0.137	0.007	1.6E-77	trans
1216	KIT	P10721	Mast/stem cell growth factor receptor Kit	rs10118718	109438975	9	A	G	0.100	0.087	0.012	4.1E-13	trans
1216	KIT	P10721	Mast/stem cell growth factor receptor Kit	rs7098111	119573178	10	T	C	0.167	0.112	0.010	5.2E-31	trans
1216	KIT	P10721	Mast/stem cell growth factor receptor Kit	rs3008336	135099533	10	T	C	0.164	0.072	0.010	5.2E-14	trans
1216	KIT	P10721	Mast/stem cell growth factor receptor Kit	rs35056404	44600733	11	A	G	0.061	0.181	0.015	1.1E-33	trans
1216	KIT	P10721	Mast/stem cell growth factor receptor Kit	rs948055	126292289	11	G	C	0.477	0.073	0.007	1.8E-24	trans
1216	KIT	P10721	Mast/stem cell growth factor receptor Kit	rs1805007	89986117	16	C	T	0.894	0.151	0.012	2.5E-39	trans
1216	KIT	P10721	Mast/stem cell growth factor receptor Kit	rs17758695	60920854	18	C	T	0.971	0.238	0.021	1.2E-29	trans

1216	KIT	P10721	Mast/stem cell growth factor receptor Kit	rs78744187	33754548	19	T	C	0.082	0.324	0.013	5.2E-137	trans
1216	KIT	P10721	Mast/stem cell growth factor receptor Kit	rs2248555	42520337	21	G	A	0.503	0.099	0.007	5.6E-44	trans
1217	KITLG	P21583	Kit ligand	rs590820	230309619	1	G	A	0.417	0.052	0.007	4.0E-12	trans
1217	KITLG	P21583	Kit ligand	rs705379	94953895	7	A	G	0.478	0.081	0.007	9.9E-28	trans
1217	KITLG	P21583	Kit ligand	rs983309	9177732	8	G	T	0.883	0.085	0.011	8.7E-14	trans
1217	KITLG	P21583	Kit ligand	rs112875651	126506694	8	A	G	0.391	0.067	0.008	1.1E-18	trans
1217	KITLG	P21583	Kit ligand	rs2740488	107661742	9	A	C	0.733	0.103	0.008	2.1E-35	trans
1217	KITLG	P21583	Kit ligand	rs507666	136149399	9	G	A	0.815	0.072	0.009	4.8E-14	trans
1217	KITLG	P21583	Kit ligand	rs174564	61588305	11	G	A	0.349	0.068	0.008	7.3E-19	trans
1217	KITLG	P21583	Kit ligand	rs673335	75450576	11	C	T	0.155	0.082	0.010	4.9E-16	trans
1217	KITLG	P21583	Kit ligand	rs150844304	43726625	15	A	C	0.976	0.188	0.024	2.6E-15	trans
1217	KITLG	P21583	Kit ligand	rs247617	56990716	16	A	C	0.324	0.135	0.008	8.4E-66	trans
1217	KITLG	P21583	Kit ligand	rs2292318	67985706	16	T	C	0.124	0.157	0.011	1.9E-44	trans
1217	KITLG	P21583	Kit ligand	rs34931250	66879927	17	C	T	0.940	0.113	0.015	1.9E-13	trans
1217	KITLG	P21583	Kit ligand	rs1645788	54808174	19	G	A	0.232	0.105	0.009	9.5E-34	trans
1217	KITLG	P21583	Kit ligand	rs2868346	44547970	20	C	T	0.241	0.310	0.009	7.4E-282	trans
1218	KLB	Q86Z14	Beta-klotho	rs114165349	27021913	1	G	C	0.977	0.147	0.021	2.6E-12	trans
1218	KLB	Q86Z14	Beta-klotho	rs13108218	3443931	4	G	A	0.618	0.052	0.008	1.5E-11	trans
1218	KLB	Q86Z14	Beta-klotho	rs13103023	39457617	4	G	A	0.672	0.762	0.008	0.0E+00	cis
1218	KLB	Q86Z14	Beta-klotho	rs3135911	176513896	5	C	A	0.709	0.166	0.007	7.4E-122	trans
1218	KLB	Q86Z14	Beta-klotho	rs28929474	94844947	14	T	C	0.021	0.325	0.022	4.1E-48	trans
1218	KLB	Q86Z14	Beta-klotho	rs60134803	9600728	17	G	A	0.235	0.053	0.008	8.2E-12	trans
1218	KLB	Q86Z14	Beta-klotho	rs112001035	66823805	17	G	A	0.939	0.667	0.014	0.0E+00	trans
1218	KLB	Q86Z14	Beta-klotho	rs41292412	56118358	18	C	T	0.987	0.199	0.029	5.8E-12	trans
1218	KLB	Q86Z14	Beta-klotho	rs149131600	35549122	19	T	C	0.175	0.065	0.008	1.1E-14	trans
1219	KLF4	O43474	Krueppel-like factor 4	rs7080536	115348046	10	G	A	0.956	0.186	0.019	8.9E-23	trans
1219	KLF4	O43474	Krueppel-like factor 4	rs10418046	54327869	19	G	T	0.211	0.065	0.009	3.3E-12	trans
1220	KLK1	P06870	Kallikrein-1	rs12758813	16343079	1	A	G	0.399	0.041	0.006	4.4E-13	trans
1220	KLK1	P06870	Kallikrein-1	rs2576570	118147675	1	A	C	0.423	0.055	0.006	4.5E-22	trans
1220	KLK1	P06870	Kallikrein-1	rs423144	155169355	1	T	G	0.426	0.048	0.006	1.4E-17	trans
1220	KLK1	P06870	Kallikrein-1	rs35275076	121970361	2	T	C	0.454	0.047	0.006	3.3E-16	trans
1220	KLK1	P06870	Kallikrein-1	rs11889674	214026818	2	A	G	0.392	0.047	0.006	8.7E-17	trans
1220	KLK1	P06870	Kallikrein-1	rs2170582	44371669	5	A	G	0.329	0.041	0.006	4.2E-12	trans
1220	KLK1	P06870	Kallikrein-1	rs3749751	134509677	5	T	C	0.591	0.039	0.006	9.8E-12	trans
1220	KLK1	P06870	Kallikrein-1	rs8176693	136137657	9	T	C	0.061	0.175	0.012	3.6E-52	trans
1220	KLK1	P06870	Kallikrein-1	rs3862630	126243649	11	T	C	0.123	0.217	0.009	1.1E-134	trans
1220	KLK1	P06870	Kallikrein-1	rs9529913	72345089	13	T	C	0.604	0.061	0.006	1.2E-26	trans
1220	KLK1	P06870	Kallikrein-1	rs55714927	7080316	17	T	C	0.195	0.066	0.007	6.7E-21	trans
1220	KLK1	P06870	Kallikrein-1	rs601338	49206674	19	A	G	0.509	0.889	0.008	0.0E+00	cis
1220	KLK1	P06870	Kallikrein-1	rs66464836	42684084	21	C	G	0.888	0.071	0.009	6.9E-16	trans
1221	KLK10	O43240	Kallikrein-10	rs4576692	120081873	1	C	T	0.324	0.058	0.007	1.5E-15	trans
1221	KLK10	O43240	Kallikrein-10	rs2023472	30075864	6	G	A	0.588	0.047	0.007	7.9E-12	trans
1221	KLK10	O43240	Kallikrein-10	rs375524395	109507030	9	G	A	0.456	0.049	0.007	9.8E-13	trans
1221	KLK10	O43240	Kallikrein-10	rs1065853	45413233	19	T	G	0.082	0.094	0.014	1.3E-11	trans
1221	KLK10	O43240	Kallikrein-10	rs2569454	51523203	19	C	T	0.578	0.593	0.008	0.0E+00	cis
1222	KLK11	Q9UBX7	Kallikrein-11	rs34255800	242731446	2	G	C	0.846	0.230	0.010	1.9E-114	trans
1222	KLK11	Q9UBX7	Kallikrein-11	rs369705328	17848192	10	C	G	0.128	0.078	0.011	6.1E-13	trans
1222	KLK11	Q9UBX7	Kallikrein-11	rs36060036	20361950	16	C	T	0.833	0.069	0.010	1.3E-12	trans
1222	KLK11	Q9UBX7	Kallikrein-11	rs186021206	7069412	17	A	G	0.006	0.475	0.049	5.6E-22	trans
1222	KLK11	Q9UBX7	Kallikrein-11	rs117268623	51527970	19	C	T	0.968	1.147	0.021	0.0E+00	cis
1222	KLK11	Q9UBX7	Kallikrein-11	rs12986064	54755133	19	T	C	0.492	0.051	0.008	1.5E-11	trans
1223	KLK12	Q9UKR0	Kallikrein-12	rs2479016	2245345	6	A	G	0.573	0.068	0.005	1.9E-50	trans
1223	KLK12	Q9UKR0	Kallikrein-12	rs11220477	126275402	11	C	T	0.958	0.133	0.011	3.7E-33	trans
1223	KLK12	Q9UKR0	Kallikrein-12	rs79744308	5827765	19	A	G	0.042	0.141	0.019	3.8E-13	trans
1223	KLK12	Q9UKR0	Kallikrein-12	rs3745540	51535130	19	A	G	0.418	1.167	0.008	0.0E+00	cis
1224	KLK13	Q9UKR3	Kallikrein-13	rs12995525	107559994	2	G	T	0.496	0.061	0.007	3.6E-18	trans
1224	KLK13	Q9UKR3	Kallikrein-13	rs2479016	2245345	6	A	G	0.573	0.058	0.007	7.6E-16	trans
1224	KLK13	Q9UKR3	Kallikrein-13	rs5511	95033595	14	T	A	0.220	0.142	0.008	2.7E-63	trans
1224	KLK13	Q9UKR3	Kallikrein-13	rs55707100	43820717	15	C	T	0.976	0.168	0.023	1.1E-13	trans
1224	KLK13	Q9UKR3	Kallikrein-13	rs186021206	7069412	17	A	G	0.006	0.640	0.048	8.2E-40	trans
1224	KLK13	Q9UKR3	Kallikrein-13	rs75181977	45569886	18	G	A	0.102	0.098	0.012	2.4E-17	trans
1224	KLK13	Q9UKR3	Kallikrein-13	rs78060698	5832773	19	A	G	0.041	0.187	0.019	5.1E-23	trans
1224	KLK13	Q9UKR3	Kallikrein-13	rs681343	49206462	19	C	T	0.491	0.074	0.008	8.0E-23	trans
1224	KLK13	Q9UKR3	Kallikrein-13	rs3760739	51538561	19	G	T	0.650	0.454	0.008	0.0E+00	cis
1225	KLK14	Q9P0G3	Kallikrein-14	rs61816761	152285861	1	A	G	0.024	0.169	0.024	1.4E-12	trans
1225	KLK14	Q9P0G3	Kallikrein-14	rs58741371	119633581	2	C	T	0.197	0.064	0.009	1.9E-13	trans
1225	KLK14	Q9P0G3	Kallikrein-14	rs6880842	2469415	5	G	A	0.385	0.048	0.007	1.6E-11	trans
1225	KLK14	Q9P0G3	Kallikrein-14	rs11249893	8700851	8	T	C	0.477	0.052	0.007	2.2E-14	trans
1225	KLK14	Q9P0G3	Kallikrein-14	rs60978445	110773037	9	T	C	0.813	0.072	0.009	5.3E-16	trans
1225	KLK14	Q9P0G3	Kallikrein-14	rs635634	136155000	9	C	T	0.816	0.076	0.009	1.3E-17	trans
1225	KLK14	Q9P0G3	Kallikrein-14	rs11599750	101805442	10	C	T	0.591	0.062	0.007	2.8E-19	trans
1225	KLK14	Q9P0G3	Kallikrein-14	rs2569491	51584916	19	A	G	0.299	0.416	0.008	0.0E+00	cis
1225	KLK14	Q9P0G3	Kallikrein-14	rs12986064	54755133	19	T	C	0.492	0.068	0.008	5.2E-19	trans
1225	KLK14	Q9P0G3	Kallikrein-14	rs1276365	55594543	20	G	A	0.558	0.047	0.007	1.4E-11	trans
1226	KLK15	Q9H2R5	Kallikrein-15	rs7560701	114026882	2	G	C	0.501	0.063	0.006	2.5E-29	trans
1226	KLK15	Q9H2R5	Kallikrein-15	rs4536908	23897809	4	T	C	0.461	0.039	0.006	2.8E-12	trans
1226	KLK15	Q9H2R5	Kallikrein-15	rs72840032	101889964	10	C	T	0.957	0.104	0.014	5.7E-14	trans
1226	KLK15	Q9H2R5	Kallikrein-15	rs626277	72347696	13	A	C	0.604	0.044	0.006	1.2E-14	trans
1226	KLK15	Q9H2R5	Kallikrein-15	rs73048483	51340543	19	A	G	0.074	1.415	0.015	0.0E+00	cis
1227	KLK3	P07288	Prostate-specific antigen	rs62113212	51360840	19	C	T	0.925	0.105	0.009	1.3E-31	cis
1228	KLK4	Q9Y5K2	Kallikrein-4	rs1572993	205045087	1	G	A	0.585	0.048	0.006	1.9E-15	trans
1228	KLK4	Q9Y5K2	Kallikrein-4	rs28929474	94844947	14	C	T	0.979	0.441	0.021	2.5E-99	trans
1228	KLK4	Q9Y5K2	Kallikrein-4	rs2664153	51415252	19	G	A	0.361	0.418	0.007	0.0E+00	cis
1229	KLK6	Q92876	Kallikrein-6	rs9933330	20366810	16	C	T	0.818	0.066	0.010	5.3E-12	trans
1229	KLK6	Q92876	Kallikrein-6	rs268891	51471983	19	A	C	0.349	0.192	0.008	2.4E-131	cis
1229	KLK6	Q92876	Kallikrein-6	rs12986064	54755133	19	T	C	0.492	0.069	0.008	3.6E-19	trans
1230	KLK7	P49862	Kallikrein-7	rs80293268	8207579	1	G	C	0.952	0.157	0.017	2.0E-19	trans
1230	KLK7	P49862	Kallikrein-7	rs61816761	152285861	1	A	G	0.024	0.244	0.025	3.8E-23	trans
1230	KLK7	P49862	Kallikrein-7	rs1991821	51484905	19	G	A	0.910	0.693	0.013	0.0E+00	cis
1230	KLK7	P49862	Kallikrein-7	rs12986064	54755133	19	T	C	0.493	0.182	0.008	4.7E-120	trans
1231	KLK8	O60259	Kallikrein-8	rs61816761	152285861	1	A	G	0.023	0.324	0.024	3.6E-41	trans
1231	KLK8	O60259	Kallikrein-8	rs121908120	219755011	2	T	A	0.972	0.155	0.021	3.6E-13	trans
1231	KLK8	O60259	Kallikrein-8	rs186021206	7069412	17	A	G	0.006	0.390	0.048	2.5E-16	trans
1231	KLK8	O60259	Kallikrein-8	rs74705037	51504808	19	A	G	0.046	1.051	0.018	0.0E+00	cis
1231	KLK8	O60259	Kallikrein-8	rs12986064	54755133	19	T	C	0.492	0.061	0.008	2.1E-15	trans
1232	KLKB1	P03952	Plasma kallikrein	rs1260326	27730940	2	T	C	0.392	0.102	0.007	1.6E-49	trans
1232	KLKB1	P03952	Plasma kallikrein	rs710446	186459927	3	C	T	0.409	0.354	0.007	0.0E+00	trans
1232	KLKB1	P03952	Plasma kallikrein	rs4253306	187174030	4	G	T	0.900	0.562	0.012	0.0E+00	cis
1232	KLKB1	P03952	Plasma kallikrein	rs2731674	176839890	5	T	G	0.256	0.084	0.008	1.4E-27	trans
1232	KLKB1	P03952	Plasma kallikrein	rs738408	44324730	22	C	T	0.783	0.059	0.008	5.3E-13	trans
1233	KLRB1	Q12918	Killer cell lectin-like receptor subfamily B member 1	rs4149909	242023898	1	G	A	0.032	0.152	0.021	2.5E-13	trans
1233	KLRB1	Q12918	Killer cell lectin-like receptor subfamily B member 1	rs6759003	62559205	2	C	T	0.640	0.081	0.008	2.2E-26	trans
1233	KLRB1	Q12918	Killer cell lectin-like receptor subfamily B member 1	rs2009581	111807677	2	G	A	0.728	0.072	0.008	1.3E-18	trans
1233	KLRB1	Q12918	Killer cell lectin-like receptor subfamily B member 1	rs4449583	1284135	5	T	C	0.327	0.072	0.008	5.0E-20	trans
1233	KLRB1	Q12918	Killer cell lectin-like receptor subfamily B member 1	rs2249932	31327161	6	C	T	0.298	0.085	0.008	2.0E-25	trans
1233	KLRB1	Q12918	Killer cell lectin-like receptor subfamily B member 1	rs900567	79559622	8	C	T	0.248	0.064	0.009	4.1E-14	trans
1233	KLRB1	Q12918	Killer cell lectin-like receptor subfamily B member 1	rs7086174	6016399	10	A	C	0.328	0.063	0.008	7.1E-16	trans
1233	KLRB1	Q12918	Killer cell lectin-like receptor subfamily B member 1	rs12437803	50997266	15	G	A	0.807	0.184	0.009	1.5E-85	trans
1233	KLRB1	Q12918	Killer cell lectin-like receptor subfamily B member 1	rs11071528	60701797	15	C	G	0.805	0.079	0.009	9.5E-18	trans
1234	KLRD1	Q13241	Natural killer cells antigen CD94	rs2009581	111807677	2	G	A	0.728	0.111	0.008	2.1E-47	trans
1234	KLRD1	Q13241	Natural killer cells antigen CD94	rs13063578	47087837	3	T	A	0.602	0.079	0.007	6.4E-30	trans
1234	KLRD1	Q13241	Natural killer cells antigen CD94	rs2706385	131831766	5	G	T	0.662	0.055	0.007	3.0E-14	trans
1234	KLRD1	Q13241	Natural killer cells antigen CD94	rs1655895	29912517	6	T	C	0.147	0.273	0.010	3.5E-173	trans
1234	KLRD1	Q13241	Natural killer cells antigen CD94	rs12684650	139110654	9	C	T	0.681	0.054	0.007	9.0E-14	trans
1234	KLRD1	Q13241	Natural killer cells antigen CD94	rs11869875	33794099	17	G	A	0.674	0.063	0.007	3.2E-18	trans
1234	KLRD1	Q13241	Natural killer cells antigen CD94	rs3744283	62160131	17	G	C	0.840	0.069	0.009	6.4E-14	trans
1234	KLRD1	Q13241	Natural killer cells antigen CD94	rs875622	16467759	19	G	A	0.768	0.055	0.008	1.1E-11	trans
1234	KLRD1	Q13241	Natural killer cells antigen CD94	rs28873836	52314655	19	G	C	0.734	0.240	0.008	8.2E-208	trans
1235	KLRF1	Q9NZS2	Killer cell lectin-like receptor subfamily F member 1	rs2643902	2245439	1	T	C	0.212	0.065	0.009	4.1E-13	trans
1235	KLRF1	Q9NZS2	Killer cell lectin-like receptor subfamily F member 1	rs183053322	25076059	1	C	T	0.988	0.259	0.034	5.3E-14	trans
1235	KLRF1	Q9NZS2	Killer cell lectin-like receptor subfamily F member 1	rs2009581	111807677	2	G	A	0.728	0.057	0.008	1.8E-12	trans
1235	KLRF1	Q9NZS2	Killer cell lectin-like receptor subfamily F member 1	rs13063578	47087837	3	T	A	0.602	0.061	0.007	1.6E-16	trans
1235	KLRF1	Q9NZS2	Killer cell lectin-like receptor subfamily F member 1	rs2522051	131797578	5	T	C	0.548	0.065	0.007	1.2E-18	trans
1235	KLRF1	Q9NZS2	Killer cell lectin-like receptor subfamily F member 1	rs8177653	6015381	10	G	A	0.335	0.064	0.008	8.8E-17	trans
1235	KLRF1	Q9NZS2	Killer cell lectin-like receptor subfamily F member 1	rs513365	128105510	11	A	G	0.712	0.073	0.008	4.6E-20	trans
1235	KLRF1	Q9NZS2	Killer cell lectin-like receptor subfamily F member 1	rs9916257	33797371	17	G	T	0.561	0.114	0.007	3.7E-55	trans
1235	KLRF1	Q9NZS2	Killer cell lectin-like receptor subfamily F member 1	rs4968607	62132330	17	G	A	0.841	0.077	0.010	7.2E-15	trans
1235	KLRF1	Q9NZS2	Killer cell lectin-like receptor subfamily F member 1	rs150358287	3687141	20	A	C	0.012	0.267	0.033	1.3E-15	trans
1235	KLRF1	Q9NZS2	Killer cell lectin-like receptor subfamily F member 1	rs35915710	35515151	20	C	A	0.832	0.071	0.010	1.5E-13	trans
1236	KLRK1	P26718	NKG2-D type II integral membrane protein	rs4672505	62560332	2	G	A	0.639	0.106	0.007	6.0E-47	trans
1236	KLRK1	P26718	NKG2-D type II integral membrane protein	rs2009581	111807677	2	G	A	0.728	0.120	0.008	1.1E-51	trans
1236	KLRK1	P26718	NKG2-D type II integral membrane protein	rs13063578	47087837	3	T	A	0.602	0.094	0.007	4.1E-39	trans
1236	KLRK1	P26718	NKG2-D type II integral membrane protein	rs2844512	31409566	6	G	C	0.604	0.070	0.007	2.3E-22	trans
1236	KLRK1	P26718	NKG2-D type II integral membrane protein	rs186021206	7069412	17	A	G	0.006	0.400	0.048	8.9E-17	trans
1236	KLRK1	P26718	NKG2-D type II integral membrane protein	rs3803813	62117733	17	T	C	0.841	0.074	0.010	2.1E-14	trans
1236	KLRK1	P26718	NKG2-D type II integral membrane protein	rs2659005	79218714	17	T	C	0.441	0.094	0.007	3.7E-40	trans
1237	KRT18	P05783	Keratin, type I cytoskeletal 18	rs142552223	172234017	3	G	A	0.963	0.129	0.019	1.2E-11	trans
1237	KRT18	P05783	Keratin, type I cytoskeletal 18	rs7678352	146814317	4	T	C	0.164	0.072	0.010	8.8E-14	trans
1237	KRT18	P05783	Keratin, type I cytoskeletal 18	rs7043196	117134191	9	C	T	0.503	0.052	0.007	6.5E-13	trans
1237	KRT18	P05783	Keratin, type I cytoskeletal 18	rs35696875	70982136	10	TCA	T	0.698	0.053	0.008	1.7E-11	trans
1237	KRT18	P05783	Keratin, type I cytoskeletal 18	rs10883451	101924418	10	T	C	0.498	0.054	0.007	4.4E-14	trans
1237	KRT18	P05783	Keratin, type I cytoskeletal 18	rs11601507	5701074	11	A	C	0.067	0.196	0.014	1.4E-43	trans
1237	KRT18	P05783	Keratin, type I cytoskeletal 18	rs28929474	94844947	14	T	C	0.021	0.201	0.025	1.5E-15	trans

1237	KRT18	P05783	Keratin, type I cytoskeletal 18	rs132642	36545137	22	T	A	0.830	0.071	0.010	8.2E-14	trans
1237	KRT18	P05783	Keratin, type I cytoskeletal 18	rs738409	44324727	22	G	C	0.217	0.102	0.009	6.9E-32	trans
1238	KRT19	P08727	Keratin, type I cytoskeletal 19	rs7405611	39691412	17	A	G	0.623	0.147	0.008	1.1E-81	cis
1238	KRT19	P08727	Keratin, type I cytoskeletal 19	rs601338	49206674	19	G	A	0.491	0.059	0.007	5.0E-15	trans
1239	KRT5	P13647	Keratin, type II cytoskeletal 5	rs35661734	150551295	1	C	T	0.039	0.278	0.019	3.0E-49	trans
1239	KRT5	P13647	Keratin, type II cytoskeletal 5	rs73015965	161127501	6	A	G	0.994	0.693	0.049	7.2E-45	trans
1239	KRT5	P13647	Keratin, type II cytoskeletal 5	rs7240884	57568693	18	G	A	0.422	0.085	0.007	1.4E-30	trans
1240	KYAT1	Q16773	Kynurenine--oxoglutarate transaminase 1	rs138429240	6688639	1	T	C	0.005	0.671	0.053	3.4E-36	trans
1240	KYAT1	Q16773	Kynurenine--oxoglutarate transaminase 1	rs3811444	248039451	1	C	T	0.668	0.054	0.008	7.9E-12	trans
1240	KYAT1	Q16773	Kynurenine--oxoglutarate transaminase 1	rs1354034	56849749	3	C	T	0.604	0.062	0.008	5.6E-16	trans
1240	KYAT1	Q16773	Kynurenine--oxoglutarate transaminase 1	rs35072117	101541689	3	TG	T	0.461	0.056	0.008	1.1E-12	trans
1240	KYAT1	Q16773	Kynurenine--oxoglutarate transaminase 1	rs6993770	106581528	8	A	T	0.713	0.058	0.008	1.7E-12	trans
1241	KYNU	Q16719	Kynureninase	rs78189771	143635782	2	G	A	0.048	0.570	0.017	3.7E-244	cis
1241	KYNU	Q16719	Kynureninase	rs201117812	32610286	6	G	A	0.829	0.077	0.011	3.2E-13	trans
1241	KYNU	Q16719	Kynureninase	rs113609637	95903099	9	A	AT	0.859	0.071	0.010	8.4E-12	trans
1241	KYNU	Q16719	Kynureninase	rs200210321	19393890	19	AG	A	0.074	0.105	0.014	1.9E-14	trans
1241	KYNU	Q16719	Kynureninase	rs3747207	44324855	22	A	G	0.216	0.104	0.009	5.4E-33	trans
1242	L1CAM	P32004	Neural cell adhesion molecule L1	rs3791101	44366250	1	A	G	0.308	0.067	0.008	3.9E-18	trans
1242	L1CAM	P32004	Neural cell adhesion molecule L1	rs1260326	27730940	2	C	T	0.609	0.060	0.007	1.9E-16	trans
1242	L1CAM	P32004	Neural cell adhesion molecule L1	rs56278466	17875857	10	G	T	0.661	0.054	0.007	4.4E-13	trans
1242	L1CAM	P32004	Neural cell adhesion molecule L1	rs3781781	126280545	11	T	C	0.684	0.104	0.008	1.8E-42	trans
1242	L1CAM	P32004	Neural cell adhesion molecule L1	rs186021206	7069412	17	A	G	0.006	1.298	0.049	1.9E-156	trans
1242	L1CAM	P32004	Neural cell adhesion molecule L1	rs35866622	49218060	19	T	C	0.500	0.049	0.007	9.2E-12	trans
1242	L1CAM	P32004	Neural cell adhesion molecule L1	rs5750830	39840828	22	C	A	0.251	0.085	0.008	2.5E-25	trans
1242	L1CAM	P32004	Neural cell adhesion molecule L1	rs5945364	153148238	X	C	G	0.187	0.140	0.008	6.7E-77	cis
1243	L3HYPDH	Q96EM0	Trans-3-hydroxy-L-proline dehydratase	rs1354034	56849749	3	C	T	0.604	0.057	0.008	9.6E-14	trans
1243	L3HYPDH	Q96EM0	Trans-3-hydroxy-L-proline dehydratase	rs11950562	131652529	5	A	C	0.522	0.053	0.008	3.0E-12	trans
1243	L3HYPDH	Q96EM0	Trans-3-hydroxy-L-proline dehydratase	rs342293	106372219	7	C	G	0.541	0.058	0.008	2.5E-14	trans
1243	L3HYPDH	Q96EM0	Trans-3-hydroxy-L-proline dehydratase	rs6993770	106581528	8	A	T	0.713	0.063	0.008	3.0E-14	trans
1243	L3HYPDH	Q96EM0	Trans-3-hydroxy-L-proline dehydratase	rs35751397	64914016	10	TA	T	0.469	0.054	0.008	8.1E-13	trans
1243	L3HYPDH	Q96EM0	Trans-3-hydroxy-L-proline dehydratase	rs2838493	45563826	21	A	G	0.182	0.116	0.010	1.1E-32	trans
1244	LACRT	Q9GZZ8	Extracellular glycoprotein lacritin	rs10462072	44398277	5	C	T	0.220	0.062	0.009	6.8E-12	trans
1244	LACRT	Q9GZZ8	Extracellular glycoprotein lacritin	rs929542	7364609	7	T	C	0.122	0.189	0.011	8.8E-61	trans
1244	LACRT	Q9GZZ8	Extracellular glycoprotein lacritin	rs11825821	31893669	11	T	G	0.629	0.156	0.008	5.3E-88	trans
1244	LACRT	Q9GZZ8	Extracellular glycoprotein lacritin	rs113293194	74655090	17	C	A	0.070	0.129	0.015	2.4E-18	trans
1244	LACRT	Q9GZZ8	Extracellular glycoprotein lacritin	rs681343	49206462	19	T	C	0.509	0.075	0.007	1.5E-23	trans
1245	LACTB2	Q53H82	Endoribonuclease LACTB2	rs1354034	56849749	3	C	T	0.604	0.070	0.008	6.2E-20	trans
1245	LACTB2	Q53H82	Endoribonuclease LACTB2	rs191588099	71529866	8	G	A	0.993	1.110	0.047	8.8E-126	cis
1246	LAG3	P18627	Lymphocyte activation gene 3 protein	rs12740374	109817590	1	G	T	0.779	0.190	0.009	1.1E-105	trans
1246	LAG3	P18627	Lymphocyte activation gene 3 protein	rs115216147	70665526	5	T	A	0.933	0.158	0.015	1.0E-26	trans
1246	LAG3	P18627	Lymphocyte activation gene 3 protein	rs35829610	131823862	5	C	CTG	0.662	0.074	0.008	2.7E-22	trans
1246	LAG3	P18627	Lymphocyte activation gene 3 protein	rs9264419	31230137	6	A	G	0.110	0.106	0.011	4.4E-20	trans
1246	LAG3	P18627	Lymphocyte activation gene 3 protein	rs950802	60152584	11	A	G	0.306	0.053	0.008	7.2E-12	trans
1246	LAG3	P18627	Lymphocyte activation gene 3 protein	rs139974673	44027885	15	T	C	0.976	0.203	0.023	2.9E-18	trans
1246	LAG3	P18627	Lymphocyte activation gene 3 protein	rs200489612	7106378	17	A	G	0.005	0.586	0.054	4.0E-27	trans
1246	LAG3	P18627	Lymphocyte activation gene 3 protein	rs77542162	67081278	17	G	A	0.023	0.286	0.024	1.1E-32	trans
1247	LAIR1	Q6GTX8	Leukocyte-associated immunoglobulin-like receptor 1	rs333947	110470764	1	A	G	0.148	0.065	0.009	1.7E-12	trans
1247	LAIR1	Q6GTX8	Leukocyte-associated immunoglobulin-like receptor 1	rs77924615	20392332	16	G	A	0.803	0.064	0.008	1.9E-14	trans
1247	LAIR1	Q6GTX8	Leukocyte-associated immunoglobulin-like receptor 1	rs111949142	54870191	19	A	T	0.184	0.723	0.010	0.0E+00	cis
1248	LAIR2	Q6ISS4	Leukocyte-associated immunoglobulin-like receptor 2	rs13063578	47087837	3	T	A	0.602	0.045	0.006	6.7E-15	trans
1248	LAIR2	Q6ISS4	Leukocyte-associated immunoglobulin-like receptor 2	rs35663614	16438428	19	G	GC	0.374	0.064	0.008	3.2E-16	trans
1248	LAIR2	Q6ISS4	Leukocyte-associated immunoglobulin-like receptor 2	rs73070113	55014172	19	A	T	0.865	1.060	0.012	0.0E+00	cis
1249	LAMA4	Q16363	Laminin subunit alpha-4	rs61809091	183118663	1	C	T	0.415	0.083	0.008	1.9E-24	trans
1249	LAMA4	Q16363	Laminin subunit alpha-4	rs1421929	52353613	5	A	G	0.509	0.050	0.007	1.5E-11	trans
1249	LAMA4	Q16363	Laminin subunit alpha-4	rs1917342	71349754	6	A	G	0.696	0.056	0.008	3.5E-12	trans
1249	LAMA4	Q16363	Laminin subunit alpha-4	rs7765769	112459550	6	C	T	0.443	0.167	0.008	1.3E-109	cis
1249	LAMA4	Q16363	Laminin subunit alpha-4	rs41281051	107613207	7	T	C	0.541	0.055	0.007	1.4E-13	trans
1249	LAMA4	Q16363	Laminin subunit alpha-4	rs6993770	106581528	8	A	T	0.713	0.102	0.008	1.2E-35	trans
1249	LAMA4	Q16363	Laminin subunit alpha-4	rs10761731	65027610	10	T	A	0.415	0.088	0.008	9.6E-32	trans
1249	LAMA4	Q16363	Laminin subunit alpha-4	rs13339274	81875559	16	T	C	0.144	0.080	0.010	2.1E-14	trans
1249	LAMA4	Q16363	Laminin subunit alpha-4	rs143040759	34093726	17	A	G	0.021	0.614	0.026	3.2E-125	trans
1249	LAMA4	Q16363	Laminin subunit alpha-4	rs892090	55539072	19	G	T	0.834	0.126	0.010	2.4E-36	trans
1249	LAMA4	Q16363	Laminin subunit alpha-4	rs4813351	19268933	20	T	C	0.371	0.052	0.008	7.6E-12	trans
1250	LAMB1	P07942	Laminin subunit beta-1	rs11683120	204129311	2	A	G	0.451	0.098	0.006	4.6E-53	trans
1250	LAMB1	P07942	Laminin subunit beta-1	rs13107325	103188709	4	T	C	0.075	0.085	0.012	2.4E-12	trans
1250	LAMB1	P07942	Laminin subunit beta-1	rs2910686	96252589	5	C	T	0.450	0.134	0.006	9.6E-97	trans
1250	LAMB1	P07942	Laminin subunit beta-1	rs9268833	32428062	6	T	C	0.317	0.076	0.007	2.3E-28	trans
1250	LAMB1	P07942	Laminin subunit beta-1	rs436573	107739906	7	A	T	0.575	0.637	0.008	0.0E+00	cis
1250	LAMB1	P07942	Laminin subunit beta-1	rs3124776	136284450	9	T	C	0.521	0.047	0.006	1.7E-13	trans
1250	LAMB1	P07942	Laminin subunit beta-1	rs112635299	94838142	14	T	G	0.022	0.148	0.022	1.3E-11	trans
1250	LAMB1	P07942	Laminin subunit beta-1	rs11075921	72132129	16	T	C	0.160	0.081	0.009	7.3E-21	trans
1250	LAMB1	P07942	Laminin subunit beta-1	rs55799523	55999545	19	A	C	0.266	0.090	0.007	6.6E-34	trans
1251	LAMP1	P11279	Lysosome-associated membrane glycoprotein 1	rs9857570	58280690	3	A	G	0.676	0.056	0.008	9.1E-12	trans
1251	LAMP1	P11279	Lysosome-associated membrane glycoprotein 1	rs9270664	32566149	6	G	A	0.364	0.056	0.008	1.4E-12	trans
1251	LAMP1	P11279	Lysosome-associated membrane glycoprotein 1	rs1293303	11720227	8	C	G	0.446	0.100	0.008	4.3E-38	trans
1251	LAMP1	P11279	Lysosome-associated membrane glycoprotein 1	rs74612335	126238633	11	T	C	0.867	0.088	0.011	6.6E-15	trans
1251	LAMP1	P11279	Lysosome-associated membrane glycoprotein 1	rs9549739	113975290	13	A	G	0.292	0.067	0.009	9.0E-15	cis
1251	LAMP1	P11279	Lysosome-associated membrane glycoprotein 1	rs34843303	79234470	15	T	C	0.888	0.110	0.012	1.8E-19	trans
1251	LAMP1	P11279	Lysosome-associated membrane glycoprotein 1	rs186021206	7069412	17	A	G	0.006	0.405	0.052	9.8E-15	trans
1252	LAMP2	P13473	Lysosome-associated membrane glycoprotein 2	rs10908419	154567699	1	G	A	0.508	0.053	0.007	1.6E-12	trans
1252	LAMP2	P13473	Lysosome-associated membrane glycoprotein 2	rs41274050	52573772	10	T	C	0.010	0.267	0.038	1.1E-12	trans
1252	LAMP2	P13473	Lysosome-associated membrane glycoprotein 2	rs2008511	119568858	X	G	A	0.617	0.364	0.006	0.0E+00	cis
1253	LAMP3	Q9UQV4	Lysosome-associated membrane glycoprotein 3	rs11921884	182828245	3	T	G	0.649	0.233	0.008	2.7E-210	cis
1253	LAMP3	Q9UQV4	Lysosome-associated membrane glycoprotein 3	rs13107325	103188709	4	T	C	0.075	0.101	0.013	6.3E-14	trans
1253	LAMP3	Q9UQV4	Lysosome-associated membrane glycoprotein 3	rs9263519	31067184	6	G	A	0.673	0.061	0.008	6.2E-16	trans
1253	LAMP3	Q9UQV4	Lysosome-associated membrane glycoprotein 3	rs507666	136149399	9	G	A	0.815	0.071	0.009	8.2E-15	trans
1253	LAMP3	Q9UQV4	Lysosome-associated membrane glycoprotein 3	rs35705950	1241221	11	T	G	0.110	0.082	0.011	4.3E-13	trans
1253	LAMP3	Q9UQV4	Lysosome-associated membrane glycoprotein 3	rs117169628	89262657	16	G	A	0.857	0.090	0.010	4.6E-19	trans
1253	LAMP3	Q9UQV4	Lysosome-associated membrane glycoprotein 3	rs186021206	7069412	17	A	G	0.006	0.435	0.049	3.2E-19	trans
1253	LAMP3	Q9UQV4	Lysosome-associated membrane glycoprotein 3	rs892225	1152656	19	A	G	0.619	0.066	0.007	1.5E-19	trans
1254	LARP1	Q6PKG0	La-related protein 1	rs1354034	56849749	3	C	T	0.604	0.069	0.008	1.8E-19	trans
1255	LAT	O43561	Linker for activation of T-cells family member 1	rs3804749	122833003	3	C	T	0.407	0.059	0.008	2.8E-14	trans
1255	LAT	O43561	Linker for activation of T-cells family member 1	rs17622656	131820997	5	G	A	0.612	0.054	0.008	2.8E-12	trans
1255	LAT	O43561	Linker for activation of T-cells family member 1	rs6993770	106581528	8	A	T	0.713	0.094	0.008	3.8E-30	trans
1255	LAT	O43561	Linker for activation of T-cells family member 1	rs7080386	65048306	10	A	C	0.412	0.066	0.008	3.2E-18	trans
1255	LAT	O43561	Linker for activation of T-cells family member 1	rs892090	55539072	19	G	T	0.834	0.096	0.010	1.4E-21	trans
1256	LAT2	Q9GZY6	Linker for activation of T-cells family member 2	rs1354034	56849749	3	C	T	0.605	0.061	0.008	9.5E-16	trans
1257	LATS1	O95835	Serine/threonine-protein kinase LATS1	rs1354034	56849749	3	C	T	0.604	0.094	0.008	1.2E-33	trans
1257	LATS1	O95835	Serine/threonine-protein kinase LATS1	rs6993770	106581528	8	A	T	0.713	0.058	0.008	5.1E-12	trans
1258	LAYN	Q6UX15	Layilin	rs61747728	179526214	1	T	C	0.038	0.125	0.018	3.8E-12	trans
1258	LAYN	Q6UX15	Layilin	rs8176719	136132908	9	TC	T	0.340	0.069	0.007	1.9E-21	trans
1258	LAYN	Q6UX15	Layilin	rs542275	111431614	11	T	C	0.616	0.389	0.007	0.0E+00	cis
1258	LAYN	Q6UX15	Layilin	rs7203642	20367130	16	A	G	0.808	0.068	0.009	7.2E-15	trans
1259	LBP	P18428	Lipopolysaccharide-binding protein	rs112001035	66823805	17	A	G	0.061	0.109	0.014	2.7E-14	trans
1259	LBP	P18428	Lipopolysaccharide-binding protein	rs2232613	36997655	20	C	T	0.918	1.188	0.014	0.0E+00	cis
1260	LBR	Q14739	Delta(14)-sterol reductase LBR	rs1354034	56849749	3	C	T	0.604	0.165	0.008	1.1E-105	trans
1260	LBR	Q14739	Delta(14)-sterol reductase LBR	rs342293	106372219	7	C	G	0.540	0.060	0.007	6.9E-16	trans
1260	LBR	Q14739	Delta(14)-sterol reductase LBR	rs10733789	64948684	10	C	T	0.313	0.062	0.008	7.9E-15	trans
1260	LBR	Q14739	Delta(14)-sterol reductase LBR	rs7080536	115348046	10	G	A	0.956	0.223	0.018	5.8E-34	trans
1260	LBR	Q14739	Delta(14)-sterol reductase LBR	rs11604127	196944	11	T	C	0.231	0.087	0.009	6.9E-23	trans
1261	LCAT	P04180	Phosphatidylcholine-sterol acyltransferase	rs1260326	27730940	2	T	C	0.391	0.092	0.008	1.1E-34	trans
1261	LCAT	P04180	Phosphatidylcholine-sterol acyltransferase	rs1047891	211540507	2	C	A	0.687	0.056	0.008	2.6E-12	trans
1261	LCAT	P04180	Phosphatidylcholine-sterol acyltransferase	rs204886	32029415	6	C	T	0.506	0.056	0.007	1.1E-14	trans
1261	LCAT	P04180	Phosphatidylcholine-sterol acyltransferase	rs1513275	28259233	7	C	T	0.192	0.066	0.010	5.1E-12	trans
1261	LCAT	P04180	Phosphatidylcholine-sterol acyltransferase	rs34121855	73040814	7	T	G	0.799	0.088	0.009	5.2E-22	trans
1261	LCAT	P04180	Phosphatidylcholine-sterol acyltransferase	rs9987289	9183358	8	G	A	0.908	0.098	0.013	9.2E-15	trans
1261	LCAT	P04180	Phosphatidylcholine-sterol acyltransferase	rs6589565	116640237	11	A	G	0.067	0.123	0.015	5.7E-17	trans
1261	LCAT	P04180	Phosphatidylcholine-sterol acyltransferase	rs7205167	11791057	16	T	A	0.488	0.053	0.007	5.4E-13	trans
1261	LCAT	P04180	Phosphatidylcholine-sterol acyltransferase	rs4986970	67976320	16	A	T	0.965	0.318	0.020	4.0E-57	cis
1261	LCAT	P04180	Phosphatidylcholine-sterol acyltransferase	rs367070	54800500	19	A	G	0.770	0.154	0.009	3.2E-69	trans
1261	LCAT	P04180	Phosphatidylcholine-sterol acyltransferase	rs1800961	43042364	20	C	T	0.968	0.172	0.021	7.8E-17	trans
1261	LCAT	P04180	Phosphatidylcholine-sterol acyltransferase	rs3747207	44324855	22	G	A	0.784	0.063	0.009	8.7E-13	trans
1262	LCN15	Q6UWW0	Lipocalin-15	rs4880140	139658576	9	C	G	0.779	0.657	0.009	0.0E+00	cis
1262	LCN15	Q6UWW0	Lipocalin-15	rs80158569	99782937	14	G	A	0.921	0.094	0.013	5.0E-13	trans
1262	LCN15	Q6UWW0	Lipocalin-15	rs1291142	35525362	20	A	G	0.327	0.060	0.007	2.6E-16	trans
1263	LCN2	P80188	Neutrophil gelatinase-associated lipocalin	rs3917932	36943916	1	C	G	0.425	0.073	0.008	2.9E-22	trans
1263	LCN2	P80188	Neutrophil gelatinase-associated lipocalin	rs3014874	153337943	1	A	G	0.264	0.075	0.008	5.3E-19	trans
1263	LCN2	P80188	Neutrophil gelatinase-associated lipocalin	rs113542380	43464818	2	A	G	0.074	0.104	0.014	4.6E-13	trans
1263	LCN2	P80188	Neutrophil gelatinase-associated lipocalin	rs2305637	47045846	3	T	C	0.176	0.083	0.010	2.3E-17	trans
1263	LCN2	P80188	Neutrophil gelatinase-associated lipocalin	rs218264	55408875	4	T	A	0.246	0.070	0.009	5.4E-16	trans
1263	LCN2	P80188	Neutrophil gelatinase-associated lipocalin	rs5869700	95253458	5	CT	C	0.328	0.076	0.008	8.2E-22	trans
1263	LCN2	P80188	Neutrophil gelatinase-associated lipocalin	rs12520928	132305891	5	A	C	0.165	0.096	0.010	5.5E-22	trans
1263	LCN2	P80188	Neutrophil gelatinase-associated lipocalin	rs2158799	28277107	7	G	C	0.609	0.062	0.008	6.3E-16	trans
1263	LCN2	P80188	Neutrophil gelatinase-associated lipocalin	rs1870028	50258313	7	T	C	0.073	0.158	0.014	4.0E-28	trans
1263	LCN2	P80188	Neutrophil gelatinase-associated lipocalin	rs7846314	61650831	8	T	A	0.186	0.123	0.010	4.7E-38	trans
1263	LCN2	P80188	Neutrophil gelatinase-associated lipocalin	rs4407843	130609368	8	A	C	0.432	0.066	0.008	2.5E-18	trans
1263	LCN2	P80188	Neutrophil gelatinase-associated lipocalin	rs542243688	130905550	9	C	A	0.971	0.236	0.025	1.3E-20	cis
1263	LCN2	P80188	Neutrophil gelatinase-associated lipocalin	rs3826331	38150492	17	T	C	0.402	0.125	0.008	8.2E-61	trans
1263	LCN2	P80188	Neutrophil gelatinase-associated lipocalin	rs36058989	12521704	19	C	T	0.782	0.067	0.009	1.1E-13	trans
1263	LCN2	P80188	Neutrophil gelatinase-associated lipocalin	rs78030362	18575193	19	A	G	0.927	0.149	0.014	5.2E-25	trans
1263	LCN2	P80188	Neutrophil gelatinase-associated lipocalin	rs243591	19155806	21	A	C	0.521	0.058	0.007	8.5E-15	trans
1263	LCN2	P80188	Neutrophil gelatinase-associated lipocalin	rs5936474	147357492	X	G	A	0.584	0.049	0.006	3.4E-15	trans
1264	LCP1	P13796	Plastin-2	rs47219	196598327	1	A	T	0.768	0.057	0.007	1.3E-15	trans

1264	LCP1	P13796	Plastin-2	rs78506734	32529695	6	T	C	0.175	0.058	0.008	7.8E-13	trans
1264	LCP1	P13796	Plastin-2	rs12342201	95894964	9	G	A	0.524	0.062	0.006	9.3E-25	trans
1264	LCP1	P13796	Plastin-2	rs7323373	46707836	13	T	A	0.093	1.473	0.013	0.0E+00	cis
1265	LDLR	P01130	Low-density lipoprotein receptor	rs1260326	27730940	2	T	C	0.391	0.082	0.007	4.0E-29	trans
1265	LDLR	P01130	Low-density lipoprotein receptor	rs4704834	156443066	5	G	A	0.648	0.082	0.008	1.8E-27	trans
1265	LDLR	P01130	Low-density lipoprotein receptor	rs3812316	73020337	7	C	G	0.873	0.095	0.011	8.8E-19	trans
1265	LDLR	P01130	Low-density lipoprotein receptor	rs13702	19824492	8	T	C	0.713	0.093	0.008	3.1E-31	trans
1265	LDLR	P01130	Low-density lipoprotein receptor	rs2737246	116659578	8	G	C	0.718	0.074	0.008	2.5E-20	trans
1265	LDLR	P01130	Low-density lipoprotein receptor	rs28601761	126500031	8	C	G	0.581	0.095	0.007	2.4E-38	trans
1265	LDLR	P01130	Low-density lipoprotein receptor	rs2068888	94839642	10	G	A	0.550	0.050	0.007	3.1E-12	trans
1265	LDLR	P01130	Low-density lipoprotein receptor	rs174533	61549025	11	A	G	0.346	0.074	0.008	2.1E-22	trans
1265	LDLR	P01130	Low-density lipoprotein receptor	rs964184	116648917	11	G	C	0.134	0.204	0.011	2.8E-82	trans
1265	LDLR	P01130	Low-density lipoprotein receptor	rs261342	58731153	15	G	C	0.223	0.061	0.009	2.2E-12	trans
1265	LDLR	P01130	Low-density lipoprotein receptor	rs247617	56990716	16	C	A	0.676	0.093	0.008	1.7E-33	trans
1265	LDLR	P01130	Low-density lipoprotein receptor	rs77542162	67081278	17	G	A	0.023	0.210	0.024	2.3E-18	trans
1265	LDLR	P01130	Low-density lipoprotein receptor	rs116843064	8429323	19	G	A	0.980	0.210	0.026	9.2E-16	trans
1265	LDLR	P01130	Low-density lipoprotein receptor	rs8107974	19388500	19	A	T	0.923	0.110	0.014	6.7E-16	trans
1265	LDLR	P01130	Low-density lipoprotein receptor	rs1065853	45413233	19	T	G	0.082	0.430	0.013	1.1E-228	trans
1266	LDLRAP1	Q5SW96	Low density lipoprotein receptor adapter protein 1	rs75446219	25877492	1	T	C	0.036	0.179	0.020	9.5E-19	cis
1266	LDLRAP1	Q5SW96	Low density lipoprotein receptor adapter protein 1	rs6993770	106581528	8	A	T	0.714	0.071	0.008	1.0E-17	trans
1267	LECT2	O14960	Leukocyte cell-derived chemotaxin-2	rs11573156	20306146	1	C	G	0.228	0.053	0.006	1.6E-18	trans
1267	LECT2	O14960	Leukocyte cell-derived chemotaxin-2	rs10027182	148981406	4	G	A	0.256	0.039	0.006	6.4E-12	trans
1267	LECT2	O14960	Leukocyte cell-derived chemotaxin-2	rs12331618	187139939	4	G	A	0.513	0.037	0.005	1.4E-13	trans
1267	LECT2	O14960	Leukocyte cell-derived chemotaxin-2	rs31517	135287029	5	T	C	0.371	0.800	0.007	0.0E+00	cis
1267	LECT2	O14960	Leukocyte cell-derived chemotaxin-2	rs4252117	161143376	6	G	A	0.295	0.086	0.005	5.6E-55	trans
1267	LECT2	O14960	Leukocyte cell-derived chemotaxin-2	rs507666	136149399	9	G	A	0.815	0.054	0.006	3.2E-17	trans
1267	LECT2	O14960	Leukocyte cell-derived chemotaxin-2	rs28929474	94844947	14	T	C	0.021	0.121	0.017	4.0E-12	trans
1267	LECT2	O14960	Leukocyte cell-derived chemotaxin-2	rs2569491	51584916	19	G	A	0.701	0.047	0.005	8.4E-18	trans
1268	LEFTY2	O00292	Left-right determination factor 2	rs10027275	148981496	4	C	G	0.741	0.055	0.007	9.1E-16	trans
1268	LEFTY2	O00292	Left-right determination factor 2	rs635634	136155000	9	C	T	0.816	0.082	0.008	2.2E-26	trans
1268	LEFTY2	O00292	Left-right determination factor 2	rs7412	45412079	19	T	C	0.081	0.110	0.011	7.7E-24	trans
1268	LEFTY2	O00292	Left-right determination factor 2	rs738408	44324730	22	T	C	0.217	0.055	0.007	2.6E-14	trans
1269	LEG1	Q6P5S2	Protein LEG1 homolog	rs908702	207105641	1	T	C	0.448	0.100	0.007	1.6E-44	trans
1269	LEG1	Q6P5S2	Protein LEG1 homolog	rs12991732	43645811	2	G	C	0.734	0.055	0.008	8.6E-12	trans
1269	LEG1	Q6P5S2	Protein LEG1 homolog	rs4362858	40424213	4	T	C	0.283	0.065	0.008	2.2E-16	trans
1269	LEG1	Q6P5S2	Protein LEG1 homolog	rs12500456	56427220	4	C	T	0.698	0.053	0.008	5.8E-12	trans
1269	LEG1	Q6P5S2	Protein LEG1 homolog	rs7447651	35268309	5	C	T	0.363	0.054	0.007	2.3E-13	trans
1269	LEG1	Q6P5S2	Protein LEG1 homolog	rs1196393	2259655	6	G	A	0.533	0.111	0.007	7.9E-51	trans
1269	LEG1	Q6P5S2	Protein LEG1 homolog	rs1550942	127887795	6	A	G	0.494	0.372	0.007	0.0E+00	cis
1269	LEG1	Q6P5S2	Protein LEG1 homolog	rs12533016	97849648	7	C	T	0.412	0.078	0.007	7.9E-27	trans
1269	LEG1	Q6P5S2	Protein LEG1 homolog	rs56278466	17875857	10	G	T	0.661	0.074	0.007	3.0E-23	trans
1269	LEG1	Q6P5S2	Protein LEG1 homolog	rs2956074	34864880	11	T	C	0.596	0.058	0.007	1.4E-15	trans
1269	LEG1	Q6P5S2	Protein LEG1 homolog	rs4937126	126281897	11	G	A	0.685	0.079	0.008	3.5E-25	trans
1269	LEG1	Q6P5S2	Protein LEG1 homolog	rs848125	37247812	14	G	T	0.558	0.103	0.007	5.1E-47	trans
1269	LEG1	Q6P5S2	Protein LEG1 homolog	rs2842329	69017595	14	G	T	0.266	0.055	0.008	1.5E-11	trans
1269	LEG1	Q6P5S2	Protein LEG1 homolog	rs17855739	5831840	19	T	C	0.041	0.966	0.019	0.0E+00	trans
1269	LEG1	Q6P5S2	Protein LEG1 homolog	rs34262244	17905907	19	G	A	0.705	0.056	0.008	1.4E-11	trans
1269	LEG1	Q6P5S2	Protein LEG1 homolog	rs601338	49206674	19	A	G	0.509	0.240	0.008	1.1E-223	trans
1269	LEG1	Q6P5S2	Protein LEG1 homolog	rs2801986	69076825	X	C	T	0.245	0.052	0.007	3.2E-14	trans
1270	LEP	P41159	Leptin	rs56094641	53806453	16	G	A	0.402	0.047	0.007	8.5E-13	trans
1271	LEPR	P48357	Leptin receptor	rs10399687	66073982	1	C	T	0.829	0.276	0.010	1.7E-173	cis
1271	LEPR	P48357	Leptin receptor	rs1260326	27730940	2	T	C	0.392	0.151	0.008	8.2E-89	trans
1271	LEPR	P48357	Leptin receptor	rs111269058	72975702	7	C	T	0.878	0.082	0.011	2.3E-13	trans
1271	LEPR	P48357	Leptin receptor	rs2298475	126278203	11	T	C	0.925	0.096	0.014	4.1E-12	trans
1271	LEPR	P48357	Leptin receptor	rs28929474	94844947	14	T	C	0.021	0.299	0.026	2.4E-31	trans
1271	LEPR	P48357	Leptin receptor	rs186021206	7069412	17	A	G	0.006	0.414	0.050	1.6E-16	trans
1271	LEPR	P48357	Leptin receptor	rs1801689	64210580	17	C	A	0.030	0.255	0.021	1.2E-32	trans
1272	LGALS1	P09382	Galectin-1	rs10418046	54327869	19	G	T	0.211	0.066	0.009	1.9E-13	trans
1272	LGALS1	P09382	Galectin-1	rs4820294	38071043	22	G	A	0.666	0.283	0.008	1.4E-277	cis
1273	LGALS3	P17931	Galectin-3	rs6472539	71629810	8	G	C	0.485	0.052	0.006	2.0E-16	trans
1273	LGALS3	P17931	Galectin-3	rs3832943	55606801	14	A	AATT	0.576	0.534	0.007	0.0E+00	cis
1273	LGALS3	P17931	Galectin-3	rs17855739	5831840	19	T	C	0.041	0.181	0.016	8.0E-30	trans
1273	LGALS3	P17931	Galectin-3	rs516246	49206172	19	T	C	0.509	0.077	0.006	1.3E-33	trans
1273	LGALS3	P17931	Galectin-3	rs12986064	54755133	19	T	C	0.493	0.064	0.007	2.2E-22	trans
1274	LGALS3BP	Q08380	Galectin-3-binding protein	rs115216147	70665526	5	A	T	0.066	0.107	0.015	3.4E-13	trans
1274	LGALS3BP	Q08380	Galectin-3-binding protein	rs11242113	131777234	5	A	G	0.186	0.089	0.009	8.5E-22	trans
1274	LGALS3BP	Q08380	Galectin-3-binding protein	rs28753035	31307688	6	C	G	0.166	0.068	0.010	8.1E-12	trans
1274	LGALS3BP	Q08380	Galectin-3-binding protein	rs41341748	16012594	8	A	G	0.011	1.641	0.035	0.0E+00	trans
1274	LGALS3BP	Q08380	Galectin-3-binding protein	rs8176741	136131461	9	A	G	0.061	0.104	0.015	2.7E-12	trans
1274	LGALS3BP	Q08380	Galectin-3-binding protein	rs7232	59940599	11	T	A	0.627	0.061	0.007	1.8E-16	trans
1274	LGALS3BP	Q08380	Galectin-3-binding protein	rs4968402	58051316	17	A	G	0.839	0.099	0.010	4.1E-24	trans
1274	LGALS3BP	Q08380	Galectin-3-binding protein	rs111526614	76962761	17	C	T	0.982	0.480	0.028	2.4E-64	cis
1274	LGALS3BP	Q08380	Galectin-3-binding protein	rs6115999	3699741	20	T	C	0.012	0.766	0.034	3.5E-114	trans
1275	LGALS4	P56470	Galectin-4	rs17655730	270715	11	C	T	0.245	0.058	0.008	2.3E-13	trans
1275	LGALS4	P56470	Galectin-4	rs140695578	39265229	19	T	A	0.032	0.280	0.019	3.7E-47	cis
1276	LGALS7_LGALS7B	P47929	Galectin-7	rs61816761	152285861	1	A	G	0.024	0.316	0.024	3.7E-38	trans
1276	LGALS7_LGALS7B	P47929	Galectin-7	rs833270	181520166	3	C	T	0.617	0.049	0.007	1.2E-11	trans
1276	LGALS7_LGALS7B	P47929	Galectin-7	rs17658212	41145919	15	C	T	0.940	0.106	0.015	2.3E-12	trans
1276	LGALS7_LGALS7B	P47929	Galectin-7	rs1549299	31154146	16	G	A	0.295	0.057	0.008	9.5E-14	trans
1276	LGALS7_LGALS7B	P47929	Galectin-7	rs6508818	39277154	19	G	A	0.578	0.225	0.007	5.1E-214	cis
1276	LGALS7_LGALS7B	P47929	Galectin-7	rs584768	49213284	19	A	G	0.533	0.048	0.007	1.7E-11	trans
1277	LGALS8	O00214	Galectin-8	rs1475133	236716686	1	A	G	0.762	0.306	0.009	8.3E-283	cis
1277	LGALS8	O00214	Galectin-8	rs1354034	56849749	3	C	T	0.604	0.074	0.007	2.3E-24	trans
1277	LGALS8	O00214	Galectin-8	rs342293	106372219	7	C	G	0.540	0.084	0.007	1.1E-31	trans
1277	LGALS8	O00214	Galectin-8	rs55646585	144999621	8	T	C	0.413	0.063	0.007	4.0E-18	trans
1277	LGALS8	O00214	Galectin-8	rs2519093	136141870	9	C	T	0.815	0.482	0.010	0.0E+00	trans
1277	LGALS8	O00214	Galectin-8	rs7080386	65048306	10	A	C	0.412	0.056	0.007	1.7E-14	trans
1278	LGALS9	O00182	Galectin-9	rs79526289	25971979	17	A	G	0.750	0.514	0.008	0.0E+00	cis
1278	LGALS9	O00182	Galectin-9	rs601338	49206674	19	A	G	0.510	0.084	0.007	2.5E-35	trans
1279	LGMN	Q99538	Legumain	rs3811444	248039451	1	C	T	0.668	0.067	0.008	1.7E-18	trans
1279	LGMN	Q99538	Legumain	rs35677470	58183636	3	A	G	0.083	0.129	0.013	8.8E-23	trans
1279	LGMN	Q99538	Legumain	rs6848819	154451678	4	C	A	0.032	0.147	0.020	5.1E-13	trans
1279	LGMN	Q99538	Legumain	rs8191772	160461334	6	G	A	0.336	0.061	0.008	1.4E-15	trans
1279	LGMN	Q99538	Legumain	rs17154130	80229484	7	C	T	0.062	0.120	0.015	1.4E-15	trans
1279	LGMN	Q99538	Legumain	rs7832219	106578977	8	T	C	0.726	0.118	0.008	1.9E-48	trans
1279	LGMN	Q99538	Legumain	rs2737246	116659578	8	G	C	0.718	0.059	0.008	3.9E-13	trans
1279	LGMN	Q99538	Legumain	rs6986384	124832181	8	A	G	0.081	0.093	0.013	2.1E-12	trans
1279	LGMN	Q99538	Legumain	rs600038	136151806	9	T	C	0.793	0.072	0.009	9.2E-16	trans
1279	LGMN	Q99538	Legumain	rs2250645	91008873	10	T	C	0.300	0.072	0.008	6.2E-20	trans
1279	LGMN	Q99538	Legumain	rs12767683	104316581	10	A	C	0.325	0.061	0.008	3.6E-15	trans
1279	LGMN	Q99538	Legumain	rs148659834	93176042	14	G	A	0.988	1.055	0.034	2.7E-213	cis
1279	LGMN	Q99538	Legumain	rs3026096	5275918	17	G	A	0.507	0.056	0.007	1.2E-14	trans
1279	LGMN	Q99538	Legumain	rs55976836	48774683	19	G	A	0.770	0.061	0.009	2.0E-12	trans
1279	LGMN	Q99538	Legumain	rs1654425	55538980	19	C	T	0.834	0.105	0.010	4.3E-27	trans
1280	LHB	P01229	Lutropin subunit beta	rs72481571	106369875	14	A	G	0.106	0.157	0.013	3.4E-33	trans
1280	LHB	P01229	Lutropin subunit beta	rs3795048	49520842	19	A	T	0.939	0.120	0.016	2.9E-14	cis
1281	LIFR	P42702	Leukemia inhibitory factor receptor	rs34579268	58410554	3	T	C	0.371	0.063	0.008	1.3E-16	trans
1281	LIFR	P42702	Leukemia inhibitory factor receptor	rs34211178	98383562	3	G	A	0.560	0.058	0.007	3.1E-15	trans
1281	LIFR	P42702	Leukemia inhibitory factor receptor	rs11923060	186589390	3	T	C	0.157	0.087	0.010	6.1E-18	trans
1281	LIFR	P42702	Leukemia inhibitory factor receptor	rs62353655	38493566	5	G	A	0.954	0.360	0.018	6.9E-93	cis
1281	LIFR	P42702	Leukemia inhibitory factor receptor	rs562289	32577046	6	T	C	0.230	0.066	0.009	3.1E-14	trans
1281	LIFR	P42702	Leukemia inhibitory factor receptor	rs2519093	136141870	9	C	T	0.816	0.421	0.010	0.0E+00	trans
1281	LIFR	P42702	Leukemia inhibitory factor receptor	rs11596680	20208483	10	G	A	0.327	0.057	0.008	4.5E-13	trans
1281	LIFR	P42702	Leukemia inhibitory factor receptor	rs78689694	126234820	11	G	C	0.867	0.099	0.011	3.4E-20	trans
1281	LIFR	P42702	Leukemia inhibitory factor receptor	rs186021206	7069412	17	A	G	0.006	1.010	0.050	8.1E-90	trans
1281	LIFR	P42702	Leukemia inhibitory factor receptor	rs2252576	42615293	21	T	C	0.244	0.061	0.009	9.5E-13	trans
1282	LILRA2	Q8N149	Leukocyte immunoglobulin-like receptor subfamily A member 2	rs2070901	161185058	1	T	G	0.270	0.068	0.007	1.7E-20	trans
1282	LILRA2	Q8N149	Leukocyte immunoglobulin-like receptor subfamily A member 2	rs1257169	134963862	2	C	A	0.501	0.078	0.006	7.1E-33	trans
1282	LILRA2	Q8N149	Leukocyte immunoglobulin-like receptor subfamily A member 2	rs140473879	155479065	4	C	T	0.996	1.007	0.054	1.3E-76	trans
1282	LILRA2	Q8N149	Leukocyte immunoglobulin-like receptor subfamily A member 2	rs2723509	17817037	7	G	A	0.591	0.049	0.007	7.5E-14	trans
1282	LILRA2	Q8N149	Leukocyte immunoglobulin-like receptor subfamily A member 2	rs635634	136155000	9	T	C	0.184	0.061	0.008	3.0E-13	trans
1282	LILRA2	Q8N149	Leukocyte immunoglobulin-like receptor subfamily A member 2	rs4055121	126232337	11	C	T	0.865	0.083	0.009	3.5E-18	trans
1282	LILRA2	Q8N149	Leukocyte immunoglobulin-like receptor subfamily A member 2	rs76428106	28604007	13	C	T	0.013	0.234	0.030	4.4E-15	trans
1282	LILRA2	Q8N149	Leukocyte immunoglobulin-like receptor subfamily A member 2	rs186021206	7069412	17	A	G	0.006	0.892	0.044	3.7E-90	trans
1282	LILRA2	Q8N149	Leukocyte immunoglobulin-like receptor subfamily A member 2	rs77542162	67081278	17	G	A	0.023	0.152	0.022	2.0E-12	trans
1282	LILRA2	Q8N149	Leukocyte immunoglobulin-like receptor subfamily A member 2	rs35866622	49218060	19	T	C	0.500	0.052	0.007	2.1E-12	trans
1282	LILRA2	Q8N149	Leukocyte immunoglobulin-like receptor subfamily A member 2	rs28469702	55105727	19	A	G	0.909	1.036	0.013	0.0E+00	cis
1283	LILRA3	Q8N6C8	Leukocyte immunoglobulin-like receptor subfamily A member 3	rs141094656	92753336	1	C	T	0.015	0.167	0.020	1.5E-16	trans
1283	LILRA3	Q8N6C8	Leukocyte immunoglobulin-like receptor subfamily A member 3	rs186021206	7069412	17	A	G	0.006	0.360	0.032	2.5E-29	trans
1283	LILRA3	Q8N6C8	Leukocyte immunoglobulin-like receptor subfamily A member 3	rs367070	54800500	19	A	G	0.770	1.219	0.009	0.0E+00	cis
1284	LILRA5	A6NI73	Leukocyte immunoglobulin-like receptor subfamily A member 5	rs4233366	161159147	1	T	C	0.268	0.126	0.008	4.4E-54	trans
1284	LILRA5	A6NI73	Leukocyte immunoglobulin-like receptor subfamily A member 5	rs731557	196705015	1	A	C	0.194	0.211	0.009	5.1E-119	trans
1284	LILRA5	A6NI73	Leukocyte immunoglobulin-like receptor subfamily A member 5	rs597697	10538945	6	G	A	0.576	0.053	0.007	2.2E-13	trans
1284	LILRA5	A6NI73	Leukocyte immunoglobulin-like receptor subfamily A member 5	rs10980802	113918856	9	A	G	0.515	0.074	0.007	3.9E-25	trans
1284	LILRA5	A6NI73	Leukocyte immunoglobulin-like receptor subfamily A member 5	rs1892548	41002641	13	T	C	0.342	0.064	0.008	3.3E-17	trans
1284	LILRA5	A6NI73	Leukocyte immunoglobulin-like receptor subfamily A member 5	rs8033923	80257747	15	A	G	0.793	0.069	0.009	3.7E-15	trans
1284	LILRA5	A6NI73	Leukocyte immunoglobulin-like receptor subfamily A member 5	rs186021206	7069412	17	A	G	0.006	0.390	0.049	1.3E-15	trans
1284	LILRA5	A6NI73	Leukocyte immunoglobulin-like receptor subfamily A member 5	rs913672	48906967	20	G	T	0.341	0.052	0.008	4.1E-12	trans
1285	LILRA6	Q6PI73	Leukocyte immunoglobulin-like receptor subfamily A member 6	rs186021206	7069412	17	A	G	0.006	0.340	0.038	2.0E-19	trans
1285	LILRA6	Q6PI73	Leukocyte immunoglobulin-like receptor subfamily A member 6	rs11668526	54749060	19	T	C	0.594	0.796	0.008	0.0E+00	cis
1286	LILRB1	Q8NHL6	Leukocyte immunoglobulin-like receptor subfamily B member 1	rs1257169	134963862	2	C	A	0.501	0.070	0.006	2.6E-29	trans
1286	LILRB1	Q8NHL6	Leukocyte immunoglobulin-like receptor subfamily B member 1	rs27295	96358687	5	C	T	0.447	0.046	0.006	1.3E-13	trans
1286	LILRB1	Q8NHL6	Leukocyte immunoglobulin-like receptor subfamily B member 1	rs9266271	31326082	6	C	T	0.351	0.205	0.007	1.3E-203	trans

1286	LILRB1	Q8NHL6	Leukocyte immunoglobulin-like receptor subfamily B member 1	rs2031902	33117524	9	C	T	0.413	0.047	0.006	1.9E-13	trans
1286	LILRB1	Q8NHL6	Leukocyte immunoglobulin-like receptor subfamily B member 1	rs115478735	136149711	9	T	A	0.184	0.083	0.008	4.7E-25	trans
1286	LILRB1	Q8NHL6	Leukocyte immunoglobulin-like receptor subfamily B member 1	rs1072160	71194006	10	A	G	0.445	0.078	0.006	1.3E-35	trans
1286	LILRB1	Q8NHL6	Leukocyte immunoglobulin-like receptor subfamily B member 1	rs75071241	126232186	11	G	A	0.940	0.090	0.013	5.1E-12	trans
1286	LILRB1	Q8NHL6	Leukocyte immunoglobulin-like receptor subfamily B member 1	rs75974417	57063494	16	T	C	0.211	0.063	0.008	1.8E-16	trans
1286	LILRB1	Q8NHL6	Leukocyte immunoglobulin-like receptor subfamily B member 1	rs186021206	7069412	17	A	G	0.006	0.938	0.043	1.0E-106	trans
1286	LILRB1	Q8NHL6	Leukocyte immunoglobulin-like receptor subfamily B member 1	rs367070	54800500	19	A	G	0.770	0.671	0.009	0.0E+00	cis
1287	LILRB2	Q8N423	Leukocyte immunoglobulin-like receptor subfamily B member 2	rs10801559	196704204	1	G	A	0.597	0.081	0.005	1.4E-50	trans
1287	LILRB2	Q8N423	Leukocyte immunoglobulin-like receptor subfamily B member 2	rs9267084	31409605	6	A	G	0.154	0.137	0.007	1.2E-77	trans
1287	LILRB2	Q8N423	Leukocyte immunoglobulin-like receptor subfamily B member 2	rs2031902	33117524	9	C	T	0.413	0.040	0.005	1.0E-13	trans
1287	LILRB2	Q8N423	Leukocyte immunoglobulin-like receptor subfamily B member 2	rs1072160	71194006	10	A	G	0.445	0.059	0.005	9.3E-29	trans
1287	LILRB2	Q8N423	Leukocyte immunoglobulin-like receptor subfamily B member 2	rs72744945	56931193	15	C	A	0.097	0.061	0.009	6.9E-12	trans
1287	LILRB2	Q8N423	Leukocyte immunoglobulin-like receptor subfamily B member 2	rs186021206	7069412	17	A	G	0.006	0.585	0.036	3.6E-58	trans
1287	LILRB2	Q8N423	Leukocyte immunoglobulin-like receptor subfamily B member 2	rs77542162	67081278	17	G	A	0.023	0.124	0.018	2.5E-12	trans
1287	LILRB2	Q8N423	Leukocyte immunoglobulin-like receptor subfamily B member 2	rs383925	54783521	19	C	T	0.794	1.161	0.009	0.0E+00	cis
1287	LILRB2	Q8N423	Leukocyte immunoglobulin-like receptor subfamily B member 2	rs3747207	44324855	22	A	G	0.215	0.045	0.006	1.6E-12	trans
1288	LILRB4	Q8NHJ6	Leukocyte immunoglobulin-like receptor subfamily B member 4	rs9270270	32557208	6	T	C	0.161	0.732	0.010	0.0E+00	trans
1288	LILRB4	Q8NHJ6	Leukocyte immunoglobulin-like receptor subfamily B member 4	rs1065853	45413233	19	T	G	0.081	0.084	0.012	5.0E-12	trans
1288	LILRB4	Q8NHJ6	Leukocyte immunoglobulin-like receptor subfamily B member 4	rs7247412	52313868	19	T	C	0.734	0.072	0.008	1.9E-21	trans
1288	LILRB4	Q8NHJ6	Leukocyte immunoglobulin-like receptor subfamily B member 4	rs417477	55171903	19	G	A	0.118	0.381	0.010	4.2E-297	cis
1289	LILRB5	O75023	Leukocyte immunoglobulin-like receptor subfamily B member 5	rs2082206	71204623	10	A	G	0.445	0.050	0.004	3.5E-37	trans
1289	LILRB5	O75023	Leukocyte immunoglobulin-like receptor subfamily B member 5	rs186021206	7069412	17	A	G	0.006	0.354	0.027	4.8E-40	trans
1289	LILRB5	O75023	Leukocyte immunoglobulin-like receptor subfamily B member 5	rs12975366	54759361	19	T	C	0.605	1.185	0.008	0.0E+00	cis
1290	LIPF	P07098	Gastric triacylglycerol lipase	rs13195237	32451730	6	A	G	0.766	0.084	0.009	2.6E-22	trans
1290	LIPF	P07098	Gastric triacylglycerol lipase	rs12799829	60970554	11	T	C	0.341	0.141	0.008	2.1E-74	trans
1290	LIPF	P07098	Gastric triacylglycerol lipase	rs392702	36339063	19	A	G	0.034	0.255	0.020	2.9E-36	trans
1290	LIPF	P07098	Gastric triacylglycerol lipase	rs4812975	44545460	20	G	A	0.250	0.064	0.008	2.6E-14	trans
1291	LMNB1	P20700	Lamin-B1	rs2746507	17424516	1	T	A	0.619	0.152	0.008	3.9E-86	trans
1291	LMNB1	P20700	Lamin-B1	rs7080536	115348046	10	A	G	0.044	0.312	0.019	3.4E-63	trans
1292	LMNB2	Q03252	Lamin-B2	rs2706762	70488470	2	C	T	0.849	0.099	0.010	1.5E-22	trans
1292	LMNB2	Q03252	Lamin-B2	rs4348853	93827936	11	C	T	0.557	0.054	0.007	2.2E-13	trans
1292	LMNB2	Q03252	Lamin-B2	rs6045561	1920605	20	T	C	0.724	0.056	0.008	6.7E-12	trans
1293	LMOD1	P29536	Leiomodin-1	rs12342201	95894964	9	G	A	0.523	0.049	0.007	7.1E-13	trans
1293	LMOD1	P29536	Leiomodin-1	rs2341097	46268902	19	C	T	0.640	0.050	0.007	3.7E-12	trans
1294	LONP1	P36776	Lon protease homolog, mitochondrial	rs342293	106372219	7	C	G	0.540	0.055	0.008	5.4E-13	trans
1294	LONP1	P36776	Lon protease homolog, mitochondrial	rs6993770	106581528	8	A	T	0.713	0.061	0.008	3.9E-13	trans
1295	LPA	P08519	Apolipoprotein(a)	rs56393506	161089307	6	T	C	0.172	0.761	0.010	0.0E+00	cis
1295	LPA	P08519	Apolipoprotein(a)	rs1065853	45413233	19	G	T	0.919	0.135	0.011	8.8E-35	trans
1296	LPCAT2	Q7L5N7	Lysophosphatidylcholine acyltransferase 2	rs16955343	55566309	16	C	T	0.954	0.253	0.018	6.3E-45	cis
1297	LPL	P06858	Lipoprotein lipase	rs1260326	27730940	2	C	T	0.608	0.054	0.006	4.1E-17	trans
1297	LPL	P06858	Lipoprotein lipase	rs5835988	165501927	2	TG	T	0.403	0.047	0.006	3.2E-13	trans
1297	LPL	P06858	Lipoprotein lipase	rs1515104	227092405	2	T	A	0.350	0.052	0.007	2.4E-15	trans
1297	LPL	P06858	Lipoprotein lipase	rs72959041	127454893	6	G	A	0.950	0.101	0.015	6.3E-12	trans
1297	LPL	P06858	Lipoprotein lipase	rs149522	130449842	7	C	T	0.601	0.048	0.007	2.2E-13	trans
1297	LPL	P06858	Lipoprotein lipase	rs1801177	19805708	8	G	A	0.982	1.479	0.025	0.0E+00	cis
1297	LPL	P06858	Lipoprotein lipase	rs7201247	995434	16	G	T	0.373	0.047	0.006	3.0E-13	trans
1297	LPL	P06858	Lipoprotein lipase	rs116843064	8429323	19	A	G	0.020	0.234	0.023	3.9E-25	trans
1298	LPO	P22079	Lactoperoxidase	rs61804164	161623025	1	C	G	0.124	0.080	0.011	1.8E-12	trans
1298	LPO	P22079	Lactoperoxidase	rs6703244	202187520	1	G	A	0.413	0.078	0.007	1.1E-25	trans
1298	LPO	P22079	Lactoperoxidase	rs908702	207105641	1	T	C	0.448	0.053	0.007	5.9E-13	trans
1298	LPO	P22079	Lactoperoxidase	rs9274442	32633231	6	G	C	0.717	0.079	0.009	3.3E-18	trans
1298	LPO	P22079	Lactoperoxidase	rs62418808	119518918	6	G	A	0.668	0.175	0.008	1.9E-109	trans
1298	LPO	P22079	Lactoperoxidase	rs7791463	97819047	7	G	A	0.466	0.076	0.007	5.9E-25	trans
1298	LPO	P22079	Lactoperoxidase	rs56278466	17875857	10	G	T	0.661	0.138	0.008	5.7E-70	trans
1298	LPO	P22079	Lactoperoxidase	rs7219860	56321271	17	G	A	0.782	0.307	0.009	1.1E-253	cis
1298	LPO	P22079	Lactoperoxidase	rs12019136	5835677	19	A	G	0.041	0.625	0.019	1.8E-236	trans
1298	LPO	P22079	Lactoperoxidase	rs34262244	17905907	19	G	A	0.706	0.069	0.008	2.3E-16	trans
1298	LPO	P22079	Lactoperoxidase	rs516246	49206172	19	T	C	0.509	0.155	0.008	5.3E-94	trans
1298	LPO	P22079	Lactoperoxidase	rs61739245	31877713	20	A	T	0.981	0.253	0.027	7.7E-21	trans
1298	LPO	P22079	Lactoperoxidase	rs34826118	68776342	X	G	A	0.690	0.052	0.007	8.7E-15	trans
1299	LPP	Q93052	Lipoma-preferred partner	rs342296	106372903	7	G	A	0.543	0.057	0.008	5.0E-14	trans
1300	LRCH4	O75427	Leucine-rich repeat and calponin homology domain-containing protein 4	rs56328569	100202219	7	G	C	0.222	0.077	0.009	1.3E-17	cis
1300	LRCH4	O75427	Leucine-rich repeat and calponin homology domain-containing protein 4	rs34127158	274671	9	A	T	0.661	0.065	0.008	2.8E-16	trans
1300	LRCH4	O75427	Leucine-rich repeat and calponin homology domain-containing protein 4	rs11620465	47250344	13	T	C	0.521	0.065	0.008	3.1E-17	trans
1300	LRCH4	O75427	Leucine-rich repeat and calponin homology domain-containing protein 4	rs10418046	54327869	19	G	T	0.211	0.080	0.009	3.4E-18	trans
1301	LRG1	P02750	Leucine-rich alpha-2-glycoprotein	rs6700896	66089782	1	C	T	0.623	0.047	0.007	8.4E-12	trans
1301	LRG1	P02750	Leucine-rich alpha-2-glycoprotein	rs147233090	44028047	15	C	T	0.976	0.163	0.022	1.7E-13	trans
1301	LRG1	P02750	Leucine-rich alpha-2-glycoprotein	rs8105192	4547371	19	C	G	0.500	0.560	0.007	0.0E+00	cis
1302	LRIG1	Q96JA1	Leucine-rich repeats and immunoglobulin-like domains protein 1	rs4846922	230307182	1	C	T	0.655	0.094	0.007	2.1E-46	trans
1302	LRIG1	Q96JA1	Leucine-rich repeats and immunoglobulin-like domains protein 1	rs59104589	242237902	2	C	T	0.643	0.046	0.006	6.2E-13	trans
1302	LRIG1	Q96JA1	Leucine-rich repeats and immunoglobulin-like domains protein 1	rs2306272	66434643	3	T	C	0.712	0.858	0.008	0.0E+00	cis
1302	LRIG1	Q96JA1	Leucine-rich repeats and immunoglobulin-like domains protein 1	rs9372538	119791212	6	T	C	0.283	0.075	0.007	1.9E-27	trans
1302	LRIG1	Q96JA1	Leucine-rich repeats and immunoglobulin-like domains protein 1	rs56278466	17875857	10	G	T	0.661	0.074	0.007	5.5E-30	trans
1302	LRIG1	Q96JA1	Leucine-rich repeats and immunoglobulin-like domains protein 1	rs28929474	94844947	14	T	C	0.021	0.335	0.022	1.5E-53	trans
1302	LRIG1	Q96JA1	Leucine-rich repeats and immunoglobulin-like domains protein 1	rs339969	60883281	15	A	C	0.617	0.046	0.006	4.0E-13	trans
1303	LRP1	Q07954	Prolow-density lipoprotein receptor-related protein 1	rs1010554	52542919	3	T	C	0.529	0.047	0.007	8.7E-13	trans
1303	LRP1	Q07954	Prolow-density lipoprotein receptor-related protein 1	rs59674561	31403023	6	T	G	0.025	1.844	0.023	0.0E+00	trans
1303	LRP1	Q07954	Prolow-density lipoprotein receptor-related protein 1	rs328	19819724	8	C	G	0.899	0.224	0.011	4.1E-95	trans
1303	LRP1	Q07954	Prolow-density lipoprotein receptor-related protein 1	rs10820966	95055429	9	A	T	0.668	0.115	0.007	1.7E-61	trans
1303	LRP1	Q07954	Prolow-density lipoprotein receptor-related protein 1	rs2229498	70856852	10	G	A	0.165	0.115	0.009	3.9E-39	trans
1303	LRP1	Q07954	Prolow-density lipoprotein receptor-related protein 1	rs11846959	94846316	14	A	G	0.253	0.118	0.007	3.8E-56	trans
1303	LRP1	Q07954	Prolow-density lipoprotein receptor-related protein 1	rs200489612	7106378	17	A	G	0.005	0.614	0.049	7.0E-36	trans
1303	LRP1	Q07954	Prolow-density lipoprotein receptor-related protein 1	rs112467088	42879403	21	T	A	0.309	0.056	0.007	2.9E-15	trans
1304	LRP11	Q86VZ4	Low-density lipoprotein receptor-related protein 11	rs61747728	179526214	1	T	C	0.038	0.170	0.018	2.0E-22	trans
1304	LRP11	Q86VZ4	Low-density lipoprotein receptor-related protein 11	rs2342858	150127103	6	T	C	0.355	0.605	0.008	0.0E+00	cis
1304	LRP11	Q86VZ4	Low-density lipoprotein receptor-related protein 11	rs36060036	20361950	16	C	T	0.832	0.066	0.009	4.0E-13	trans
1304	LRP11	Q86VZ4	Low-density lipoprotein receptor-related protein 11	rs186021206	7069412	17	A	G	0.006	0.428	0.046	1.5E-20	trans
1304	LRP11	Q86VZ4	Low-density lipoprotein receptor-related protein 11	rs1801689	64210580	17	C	A	0.030	0.182	0.020	1.8E-20	trans
1305	LRPAP1	P30533	Alpha-2-macroglobulin receptor-associated protein	rs143486729	3525656	4	G	A	0.966	0.550	0.018	1.1E-208	cis
1305	LRPAP1	P30533	Alpha-2-macroglobulin receptor-associated protein	rs13107325	103188709	4	T	C	0.075	0.101	0.012	1.5E-16	trans
1305	LRPAP1	P30533	Alpha-2-macroglobulin receptor-associated protein	rs115881826	31325273	6	G	A	0.037	1.789	0.019	0.0E+00	trans
1305	LRPAP1	P30533	Alpha-2-macroglobulin receptor-associated protein	rs7923609	65133822	10	G	A	0.471	0.052	0.006	1.0E-16	trans
1305	LRPAP1	P30533	Alpha-2-macroglobulin receptor-associated protein	rs55646096	64918514	11	C	T	0.822	0.067	0.008	7.3E-16	trans
1305	LRPAP1	P30533	Alpha-2-macroglobulin receptor-associated protein	rs11846959	94846316	14	A	G	0.253	0.074	0.007	3.5E-24	trans
1306	LRRC25	Q8N386	Leucine-rich repeat-containing protein 25	rs4672491	62511587	2	T	G	0.479	0.071	0.007	3.1E-24	trans
1306	LRRC25	Q8N386	Leucine-rich repeat-containing protein 25	rs13388024	219097773	2	A	G	0.497	0.048	0.007	3.1E-12	trans
1306	LRRC25	Q8N386	Leucine-rich repeat-containing protein 25	rs2470750	98690592	3	A	T	0.607	0.208	0.007	4.6E-183	trans
1306	LRRC25	Q8N386	Leucine-rich repeat-containing protein 25	rs13107325	103188709	4	C	T	0.925	0.143	0.013	2.9E-27	trans
1306	LRRC25	Q8N386	Leucine-rich repeat-containing protein 25	rs509227	10535591	6	C	A	0.523	0.288	0.007	0.0E+00	trans
1306	LRRC25	Q8N386	Leucine-rich repeat-containing protein 25	rs2858331	32681277	6	A	G	0.632	0.081	0.007	1.1E-28	trans
1306	LRRC25	Q8N386	Leucine-rich repeat-containing protein 25	rs12667978	128777461	7	C	T	0.428	0.142	0.007	3.4E-91	trans
1306	LRRC25	Q8N386	Leucine-rich repeat-containing protein 25	rs950802	60152584	11	A	G	0.306	0.171	0.008	3.0E-113	trans
1306	LRRC25	Q8N386	Leucine-rich repeat-containing protein 25	rs4055121	126232337	11	C	T	0.865	0.084	0.010	2.4E-16	trans
1306	LRRC25	Q8N386	Leucine-rich repeat-containing protein 25	rs186021206	7069412	17	A	G	0.006	1.049	0.048	2.4E-105	trans
1306	LRRC25	Q8N386	Leucine-rich repeat-containing protein 25	rs2659005	79218714	17	T	C	0.441	0.078	0.007	9.1E-29	trans
1306	LRRC25	Q8N386	Leucine-rich repeat-containing protein 25	rs4893	48833608	19	A	G	0.960	0.453	0.018	2.8E-134	trans
1307	LRRC37A2	A6NM11	Leucine-rich repeat-containing protein 37A2	rs13107325	103188709	4	T	C	0.075	0.091	0.009	1.4E-23	trans
1307	LRRC37A2	A6NM11	Leucine-rich repeat-containing protein 37A2	rs2844566	31337703	6	T	A	0.636	0.038	0.005	3.1E-14	trans
1307	LRRC37A2	A6NM11	Leucine-rich repeat-containing protein 37A2	rs62057151	43903842	17	T	C	0.233	1.115	0.009	0.0E+00	cis
1307	LRRC37A2	A6NM11	Leucine-rich repeat-containing protein 37A2	rs41410147	62962184	17	T	C	0.067	0.365	0.017	2.2E-98	trans
1307	LRRC37A2	A6NM11	Leucine-rich repeat-containing protein 37A2	rs1065853	45413233	19	T	G	0.081	0.066	0.009	4.5E-14	trans
1308	LRRC38	Q5VT99	Leucine-rich repeat-containing protein 38	rs35329337	13822608	1	AAT	A	0.314	0.359	0.008	0.0E+00	cis
1309	LRRC59	Q96AG4	Leucine-rich repeat-containing protein 59	rs1354034	56849749	3	C	T	0.604	0.105	0.008	7.3E-43	trans
1309	LRRC59	Q96AG4	Leucine-rich repeat-containing protein 59	rs342293	106372219	7	C	G	0.541	0.064	0.008	2.1E-17	trans
1309	LRRC59	Q96AG4	Leucine-rich repeat-containing protein 59	rs35751397	64914016	10	TA	T	0.469	0.059	0.008	7.4E-15	trans
1309	LRRC59	Q96AG4	Leucine-rich repeat-containing protein 59	rs73396521	267568	11	A	G	0.245	0.067	0.009	2.8E-14	trans
1310	LRRFIP1	Q32MZ4	Leucine-rich repeat flightless-interacting protein 1	rs62183645	238565893	2	A	T	0.218	0.071	0.009	2.5E-15	cis
1311	LRRN1	Q6UXK5	Leucine-rich repeat neuronal protein 1	rs9839475	3807778	3	A	G	0.349	0.811	0.008	0.0E+00	cis
1311	LRRN1	Q6UXK5	Leucine-rich repeat neuronal protein 1	rs702634	53271420	5	A	G	0.691	0.047	0.007	1.1E-11	trans
1311	LRRN1	Q6UXK5	Leucine-rich repeat neuronal protein 1	rs1405212	117490664	6	T	C	0.372	0.064	0.007	1.4E-22	trans
1311	LRRN1	Q6UXK5	Leucine-rich repeat neuronal protein 1	rs55861089	1783757	11	A	G	0.895	0.110	0.010	1.8E-26	trans
1311	LRRN1	Q6UXK5	Leucine-rich repeat neuronal protein 1	rs594144	74726828	11	T	C	0.466	0.049	0.006	1.5E-14	trans
1311	LRRN1	Q6UXK5	Leucine-rich repeat neuronal protein 1	rs6496452	85372645	15	T	A	0.450	0.045	0.006	2.5E-12	trans
1312	LRTM2	Q8N967	Leucine-rich repeat and transmembrane domain-containing protein 2	rs79473248	186474392	3	A	G	0.032	0.322	0.021	2.0E-52	trans
1312	LRTM2	Q8N967	Leucine-rich repeat and transmembrane domain-containing protein 2	rs369705328	17848192	10	C	G	0.128	0.078	0.011	1.8E-12	trans
1312	LRTM2	Q8N967	Leucine-rich repeat and transmembrane domain-containing protein 2	rs1870148	82271341	10	A	G	0.789	0.062	0.009	3.7E-12	trans
1312	LRTM2	Q8N967	Leucine-rich repeat and transmembrane domain-containing protein 2	rs186021206	7069412	17	A	G	0.006	0.643	0.050	1.5E-37	trans
1313	LSM8	O95777	U6 snRNA-associated Sm-like protein LSm8	rs232220	89163993	2	T	C	0.951	0.155	0.021	9.9E-14	trans
1314	LSP1	P33241	Lymphocyte-specific protein 1	rs907612	1874221	11	T	C	0.379	0.149	0.008	1.4E-85	cis
1314	LSP1	P33241	Lymphocyte-specific protein 1	rs12941811	38159335	17	T	C	0.425	0.053	0.007	6.9E-13	trans
1315	LTA	P01374	Lymphotoxin-alpha	rs2009581	111807677	2	G	A	0.728	0.071	0.007	1.6E-24	trans
1315	LTA	P01374	Lymphotoxin-alpha	rs58106596	232579379	2	A	G	0.221	0.060	0.008	1.0E-15	trans
1315	LTA	P01374	Lymphotoxin-alpha	rs2229092	31540757	6	A	C	0.936	1.493	0.015	0.0E+00	cis
1315	LTA	P01374	Lymphotoxin-alpha	rs186021206	7069412	17	A	G	0.006	0.305	0.043	8.8E-13	trans
1315	LTA	P01374	Lymphotoxin-alpha	rs34557412	16852187	17	G	A	0.007	0.438	0.038	1.8E-31	trans
1315	LTA	P01374	Lymphotoxin-alpha	rs8071789	38006333	17	C	T	0.512	0.043	0.006	1.2E-11	trans
1315	LTA	P01374	Lymphotoxin-alpha	rs344560	6665020	19	C	T	0.945	0.166	0.014	5.5E-34	trans
1315	LTA	P01374	Lymphotoxin-alpha	rs738409	44324727	22	G	C	0.217	0.058	0.007	8.8E-15	trans
1316	LTB	Q06643	Lymphotoxin-beta	rs2395470	31235261	6	G	A	0.670	0.167	0.008	7.2E-96	cis
1317	LTBP2	Q14767	Latent-transforming growth factor beta-binding protein 2	rs4920605	17315425	1	A	G	0.568	0.070	0.006	1.3E-28	trans
1317	LTBP2	Q14767	Latent-transforming growth factor beta-binding protein 2	rs333947	110470764	1	G	A	0.852	0.064	0.009	5.3E-13	trans
1317	LTBP2	Q14767	Latent-transforming growth factor beta-binding protein 2	rs2462512	17847838	7	C	A	0.492	0.052	0.006	1.5E-16	trans
1317	LTBP2	Q14767	Latent-transforming growth factor beta-binding protein 2	rs888414	75104905	14	G	A	0.617	0.105	0.006	5.8E-60	cis

1317	LTBP2	Q14767	Latent-transforming growth factor beta-binding protein 2	rs2118181	48915884	15	T	C	0.898	0.081	0.010	4.8E-15	trans
1317	LTBP2	Q14767	Latent-transforming growth factor beta-binding protein 2	rs186021206	7069412	17	A	G	0.006	0.591	0.043	1.0E-42	trans
1317	LTBP2	Q14767	Latent-transforming growth factor beta-binding protein 2	rs146385050	60637258	17	A	C	0.183	0.056	0.008	7.9E-12	trans
1318	LTBP3	Q9NS15	Latent-transforming growth factor beta-binding protein 3	rs140336797	96306314	5	AT	A	0.434	0.059	0.007	2.4E-17	trans
1318	LTBP3	Q9NS15	Latent-transforming growth factor beta-binding protein 3	rs4099470	65319986	11	T	C	0.055	0.896	0.016	0.0E+00	cis
1318	LTBP3	Q9NS15	Latent-transforming growth factor beta-binding protein 3	rs28929474	94844947	14	T	C	0.021	0.260	0.024	1.3E-26	trans
1318	LTBP3	Q9NS15	Latent-transforming growth factor beta-binding protein 3	rs77303550	72079657	16	C	T	0.804	0.068	0.009	8.4E-15	trans
1318	LTBP3	Q9NS15	Latent-transforming growth factor beta-binding protein 3	rs1801689	64210580	17	C	A	0.030	0.726	0.021	4.9E-273	trans
1318	LTBP3	Q9NS15	Latent-transforming growth factor beta-binding protein 3	rs73045269	41825191	19	T	C	0.171	0.146	0.009	3.3E-57	trans
1319	LTBR	P36941	Tumor necrosis factor receptor superfamily member 3	rs61747728	179526214	1	T	C	0.038	0.202	0.019	3.1E-27	trans
1319	LTBR	P36941	Tumor necrosis factor receptor superfamily member 3	rs1260326	27730940	2	C	T	0.609	0.070	0.007	1.6E-21	trans
1319	LTBR	P36941	Tumor necrosis factor receptor superfamily member 3	rs12613853	178997647	2	G	A	0.383	0.053	0.007	1.3E-12	trans
1319	LTBR	P36941	Tumor necrosis factor receptor superfamily member 3	rs1434055	204086859	2	G	A	0.444	0.063	0.007	5.1E-18	trans
1319	LTBR	P36941	Tumor necrosis factor receptor superfamily member 3	rs27295	96358687	5	C	T	0.447	0.050	0.007	2.9E-12	trans
1319	LTBR	P36941	Tumor necrosis factor receptor superfamily member 3	rs28929474	94844947	14	T	C	0.021	0.952	0.025	9.4E-307	trans
1319	LTBR	P36941	Tumor necrosis factor receptor superfamily member 3	rs77924615	20392332	16	G	A	0.803	0.073	0.009	8.2E-16	trans
1319	LTBR	P36941	Tumor necrosis factor receptor superfamily member 3	rs1801689	64210580	17	C	A	0.030	0.152	0.021	4.3E-13	trans
1319	LTBR	P36941	Tumor necrosis factor receptor superfamily member 3	rs123698	807442	19	C	G	0.605	0.068	0.007	3.1E-20	trans
1320	LUZP2	Q86TE4	Leucine zipper protein 2	rs76794772	25049828	11	T	A	0.963	0.591	0.021	4.1E-178	cis
1321	LXN	Q9BS40	Latexin	rs6785524	158337231	3	C	A	0.475	0.167	0.008	2.8E-109	cis
1321	LXN	Q9BS40	Latexin	rs55636108	186381262	3	C	T	0.206	0.075	0.009	1.1E-15	trans
1322	LY6D	Q14210	Lymphocyte antigen 6D	rs61816761	152285861	1	A	G	0.024	0.320	0.025	1.2E-38	trans
1322	LY6D	Q14210	Lymphocyte antigen 6D	rs2870238	77373079	4	T	C	0.503	0.049	0.007	2.1E-12	trans
1322	LY6D	Q14210	Lymphocyte antigen 6D	rs2572925	143867905	8	C	T	0.595	0.443	0.008	0.0E+00	cis
1322	LY6D	Q14210	Lymphocyte antigen 6D	rs77924615	20392332	16	G	A	0.803	0.068	0.009	2.0E-14	trans
1322	LY6D	Q14210	Lymphocyte antigen 6D	rs56035820	51519710	19	T	C	0.234	0.059	0.008	2.9E-12	trans
1323	LY75	O60449	Lymphocyte antigen 75	rs72957586	160762687	2	A	G	0.558	0.934	0.008	0.0E+00	cis
1323	LY75	O60449	Lymphocyte antigen 75	rs34794906	31237858	6	C	T	0.265	0.049	0.006	9.0E-17	trans
1323	LY75	O60449	Lymphocyte antigen 75	rs1137827	136129715	9	T	C	0.061	0.094	0.011	3.2E-18	trans
1323	LY75	O60449	Lymphocyte antigen 75	rs12574844	126273386	11	G	A	0.929	0.101	0.010	3.2E-23	trans
1323	LY75	O60449	Lymphocyte antigen 75	rs186021206	7069412	17	A	G	0.006	0.923	0.036	1.0E-146	trans
1324	LY9	Q9HBG7	T-lymphocyte surface antigen Ly-9	rs188468174	25291697	1	C	T	0.986	0.249	0.032	6.2E-15	trans
1324	LY9	Q9HBG7	T-lymphocyte surface antigen Ly-9	rs37455	44294228	1	G	A	0.352	0.070	0.008	4.0E-19	trans
1324	LY9	Q9HBG7	T-lymphocyte surface antigen Ly-9	rs12128261	160762784	1	G	T	0.217	0.600	0.009	0.0E+00	cis
1324	LY9	Q9HBG7	T-lymphocyte surface antigen Ly-9	rs2009581	111807677	2	G	A	0.727	0.063	0.008	1.3E-15	trans
1324	LY9	Q9HBG7	T-lymphocyte surface antigen Ly-9	rs9824474	169745527	3	A	G	0.633	0.055	0.007	8.3E-14	trans
1324	LY9	Q9HBG7	T-lymphocyte surface antigen Ly-9	rs7634389	186738421	3	T	C	0.610	0.071	0.007	3.7E-23	trans
1324	LY9	Q9HBG7	T-lymphocyte surface antigen Ly-9	rs35829610	131823862	5	C	CTG	0.662	0.063	0.007	1.1E-17	trans
1324	LY9	Q9HBG7	T-lymphocyte surface antigen Ly-9	rs11185602	50299077	7	A	G	0.675	0.051	0.007	5.2E-12	trans
1324	LY9	Q9HBG7	T-lymphocyte surface antigen Ly-9	rs8176719	136132908	9	TC	T	0.340	0.057	0.007	9.0E-15	trans
1324	LY9	Q9HBG7	T-lymphocyte surface antigen Ly-9	rs75071241	126232186	11	G	A	0.940	0.103	0.015	3.0E-12	trans
1324	LY9	Q9HBG7	T-lymphocyte surface antigen Ly-9	rs76428106	28604007	13	C	T	0.013	0.268	0.032	1.0E-16	trans
1324	LY9	Q9HBG7	T-lymphocyte surface antigen Ly-9	rs186021206	7069412	17	A	G	0.006	1.036	0.048	1.0E-103	trans
1324	LY9	Q9HBG7	T-lymphocyte surface antigen Ly-9	rs2725405	79220224	17	C	G	0.442	0.083	0.007	3.6E-32	trans
1325	LY96	Q9Y6Y9	Lymphocyte antigen 96	rs188468174	25291697	1	C	T	0.986	0.487	0.032	2.4E-53	trans
1325	LY96	Q9Y6Y9	Lymphocyte antigen 96	rs35668054	72054837	2	C	T	0.924	0.116	0.014	6.6E-16	trans
1325	LY96	Q9Y6Y9	Lymphocyte antigen 96	rs67612510	32526436	6	G	A	0.464	0.076	0.009	1.2E-18	trans
1325	LY96	Q9Y6Y9	Lymphocyte antigen 96	rs3804333	106727215	6	C	T	0.796	0.062	0.009	1.7E-11	trans
1325	LY96	Q9Y6Y9	Lymphocyte antigen 96	rs6472812	74917084	8	A	G	0.044	0.202	0.018	3.5E-29	cis
1325	LY96	Q9Y6Y9	Lymphocyte antigen 96	rs3181356	117692882	9	T	C	0.229	0.062	0.009	1.7E-11	trans
1325	LY96	Q9Y6Y9	Lymphocyte antigen 96	rs7943728	61547068	11	A	G	0.194	0.078	0.009	9.2E-17	trans
1325	LY96	Q9Y6Y9	Lymphocyte antigen 96	rs117775520	106174221	14	G	A	0.964	0.187	0.020	1.6E-21	trans
1325	LY96	Q9Y6Y9	Lymphocyte antigen 96	rs3803800	7462969	17	A	G	0.211	0.081	0.009	2.7E-19	trans
1325	LY96	Q9Y6Y9	Lymphocyte antigen 96	rs34557412	16852187	17	A	G	0.993	0.463	0.045	7.2E-25	trans
1325	LY96	Q9Y6Y9	Lymphocyte antigen 96	rs5763821	30549071	22	A	C	0.610	0.064	0.008	1.4E-16	trans
1326	LYAR	Q9NX58	Cell growth-regulating nucleolar protein	rs11723339	4271886	4	C	T	0.379	0.055	0.008	4.6E-12	cis
1327	LYN	P07948	Tyrosine-protein kinase Lyn	rs1354034	56849749	3	C	T	0.605	0.056	0.008	2.7E-13	trans
1327	LYN	P07948	Tyrosine-protein kinase Lyn	rs2055663	83560679	4	T	C	0.136	0.091	0.011	1.3E-16	trans
1327	LYN	P07948	Tyrosine-protein kinase Lyn	rs10733789	64948684	10	C	T	0.313	0.055	0.008	1.3E-11	trans
1327	LYN	P07948	Tyrosine-protein kinase Lyn	rs2186882	119168370	11	A	G	0.270	0.072	0.008	3.1E-17	trans
1327	LYN	P07948	Tyrosine-protein kinase Lyn	rs892090	55539072	19	G	T	0.834	0.070	0.010	4.4E-12	trans
1328	LYPD3	O95274	Ly6/PLAUR domain-containing protein 3	rs1260326	27730940	2	C	T	0.609	0.052	0.007	9.5E-14	trans
1328	LYPD3	O95274	Ly6/PLAUR domain-containing protein 3	rs1047891	211540507	2	A	C	0.313	0.055	0.007	4.8E-14	trans
1328	LYPD3	O95274	Ly6/PLAUR domain-containing protein 3	rs2479016	2245345	6	A	G	0.573	0.078	0.007	3.3E-29	trans
1328	LYPD3	O95274	Ly6/PLAUR domain-containing protein 3	rs112155110	32501417	6	C	T	0.598	0.054	0.007	4.0E-14	trans
1328	LYPD3	O95274	Ly6/PLAUR domain-containing protein 3	rs7842080	134599329	8	G	A	0.217	0.179	0.008	1.1E-102	trans
1328	LYPD3	O95274	Ly6/PLAUR domain-containing protein 3	rs2519093	136141870	9	C	T	0.816	0.149	0.009	3.1E-64	trans
1328	LYPD3	O95274	Ly6/PLAUR domain-containing protein 3	rs10893502	126254018	11	T	C	0.409	0.072	0.007	2.9E-24	trans
1328	LYPD3	O95274	Ly6/PLAUR domain-containing protein 3	rs56990244	427999	16	C	G	0.412	0.100	0.007	1.4E-47	trans
1328	LYPD3	O95274	Ly6/PLAUR domain-containing protein 3	rs62034713	3359124	16	G	A	0.249	0.162	0.008	4.7E-93	trans
1328	LYPD3	O95274	Ly6/PLAUR domain-containing protein 3	rs186021206	7069412	17	A	G	0.006	0.959	0.047	6.8E-93	trans
1328	LYPD3	O95274	Ly6/PLAUR domain-containing protein 3	rs276937	28611061	18	T	A	0.320	0.133	0.007	1.5E-73	trans
1328	LYPD3	O95274	Ly6/PLAUR domain-containing protein 3	rs708686	5840619	19	T	C	0.268	0.083	0.008	8.8E-24	trans
1328	LYPD3	O95274	Ly6/PLAUR domain-containing protein 3	rs11671471	43996732	19	G	A	0.887	0.522	0.012	0.0E+00	cis
1328	LYPD3	O95274	Ly6/PLAUR domain-containing protein 3	rs601338	49206674	19	A	G	0.509	0.260	0.007	6.7E-278	trans
1328	LYPD3	O95274	Ly6/PLAUR domain-containing protein 3	rs383510	42858367	21	T	C	0.490	0.080	0.007	1.2E-31	trans
1329	LYPD8	Q6UX82	Ly6/PLAUR domain-containing protein 8	rs12044252	249065691	1	C	T	0.094	0.846	0.013	0.0E+00	cis
1329	LYPD8	Q6UX82	Ly6/PLAUR domain-containing protein 8	rs4556017	100632790	7	C	T	0.146	0.077	0.010	1.4E-14	trans
1329	LYPD8	Q6UX82	Ly6/PLAUR domain-containing protein 8	rs11220520	126357908	11	T	C	0.195	0.106	0.009	1.6E-32	trans
1329	LYPD8	Q6UX82	Ly6/PLAUR domain-containing protein 8	rs186021206	7069412	17	A	G	0.006	0.550	0.049	1.7E-29	trans
1329	LYPD8	Q6UX82	Ly6/PLAUR domain-containing protein 8	rs73338360	47271207	17	C	G	0.101	0.128	0.012	1.1E-27	trans
1329	LYPD8	Q6UX82	Ly6/PLAUR domain-containing protein 8	rs708686	5840619	19	T	C	0.268	0.168	0.008	3.3E-99	trans
1329	LYPD8	Q6UX82	Ly6/PLAUR domain-containing protein 8	rs4760	44153100	19	A	G	0.844	0.117	0.010	2.5E-33	trans
1330	LYSMD3	Q7Z3D4	LysM and putative peptidoglycan-binding domain-containing protein 3	rs68066031	224880498	2	C	T	0.229	0.095	0.009	5.0E-25	trans
1330	LYSMD3	Q7Z3D4	LysM and putative peptidoglycan-binding domain-containing protein 3	rs1354034	56849749	3	C	T	0.604	0.053	0.008	1.2E-11	trans
1330	LYSMD3	Q7Z3D4	LysM and putative peptidoglycan-binding domain-containing protein 3	rs10805870	89963988	5	C	A	0.405	0.055	0.008	4.3E-12	cis
1330	LYSMD3	Q7Z3D4	LysM and putative peptidoglycan-binding domain-containing protein 3	rs6519245	22697490	22	C	A	0.589	0.060	0.008	1.3E-14	trans
1331	LYVE1	Q9Y5Y7	Lymphatic vessel endothelial hyaluronic acid receptor 1	rs7639097	98777329	3	G	T	0.386	0.063	0.008	7.8E-16	trans
1331	LYVE1	Q9Y5Y7	Lymphatic vessel endothelial hyaluronic acid receptor 1	rs7865362	33117965	9	T	C	0.363	0.132	0.008	2.5E-66	trans
1331	LYVE1	Q9Y5Y7	Lymphatic vessel endothelial hyaluronic acid receptor 1	rs147866228	79118400	9	C	T	0.992	0.378	0.041	2.4E-20	trans
1331	LYVE1	Q9Y5Y7	Lymphatic vessel endothelial hyaluronic acid receptor 1	rs7077538	71993094	10	T	G	0.677	0.054	0.008	2.5E-12	trans
1331	LYVE1	Q9Y5Y7	Lymphatic vessel endothelial hyaluronic acid receptor 1	rs7119709	10626691	11	A	G	0.040	0.350	0.019	2.8E-75	cis
1331	LYVE1	Q9Y5Y7	Lymphatic vessel endothelial hyaluronic acid receptor 1	rs10769263	47417183	11	A	C	0.613	0.058	0.008	1.7E-14	trans
1331	LYVE1	Q9Y5Y7	Lymphatic vessel endothelial hyaluronic acid receptor 1	rs6591649	60970849	11	G	A	0.345	0.066	0.008	2.8E-17	trans
1331	LYVE1	Q9Y5Y7	Lymphatic vessel endothelial hyaluronic acid receptor 1	rs112771035	126225876	11	G	C	0.071	0.462	0.014	2.0E-226	trans
1331	LYVE1	Q9Y5Y7	Lymphatic vessel endothelial hyaluronic acid receptor 1	rs1955512	33175822	14	A	G	0.573	0.066	0.007	1.3E-18	trans
1331	LYVE1	Q9Y5Y7	Lymphatic vessel endothelial hyaluronic acid receptor 1	rs186021206	7069412	17	A	G	0.006	1.398	0.050	5.7E-172	trans
1331	LYVE1	Q9Y5Y7	Lymphatic vessel endothelial hyaluronic acid receptor 1	rs6112930	2106355	20	G	A	0.609	0.069	0.007	1.1E-20	trans
1332	LYZL2	Q7Z4W2	Lysozyme-like protein 2	rs138933538	236187372	1	T	C	0.998	0.516	0.074	2.7E-12	trans
1332	LYZL2	Q7Z4W2	Lysozyme-like protein 2	rs116792212	30894447	10	T	C	0.040	0.495	0.018	1.6E-167	cis
1333	LZTFL1	Q9NQ48	Leucine zipper transcription factor-like protein 1	rs1129183	45869972	3	C	T	0.922	0.266	0.014	2.4E-79	cis
1333	LZTFL1	Q9NQ48	Leucine zipper transcription factor-like protein 1	rs1354034	56849749	3	C	T	0.604	0.072	0.008	8.3E-21	trans
1333	LZTFL1	Q9NQ48	Leucine zipper transcription factor-like protein 1	rs61237993	34130435	9	A	G	0.127	0.089	0.011	5.5E-15	trans
1333	LZTFL1	Q9NQ48	Leucine zipper transcription factor-like protein 1	rs11502185	180258	11	C	T	0.260	0.067	0.010	3.9E-12	trans
1334	MAD1L1	Q9Y6D9	Mitotic spindle assembly checkpoint protein MAD1	rs143579216	2265063	7	A	G	0.003	1.275	0.075	3.9E-64	cis
1335	MAGED1	Q9Y5V3	Melanoma-associated antigen D1	rs1354034	56849749	3	C	T	0.604	0.060	0.008	1.8E-14	trans
1335	MAGED1	Q9Y5V3	Melanoma-associated antigen D1	rs342293	106372219	7	C	G	0.540	0.074	0.008	4.3E-22	trans
1335	MAGED1	Q9Y5V3	Melanoma-associated antigen D1	rs12847714	51678066	X	C	T	0.857	0.064	0.009	3.0E-12	cis
1336	MAMDC2	Q7Z304	MAM domain-containing protein 2	rs13412535	224874874	2	A	G	0.231	0.068	0.009	1.7E-15	trans
1336	MAMDC2	Q7Z304	MAM domain-containing protein 2	rs7580831	238219499	2	C	A	0.674	0.109	0.008	5.2E-45	trans
1336	MAMDC2	Q7Z304	MAM domain-containing protein 2	rs12646270	90857479	4	A	G	0.055	0.196	0.016	3.3E-35	trans
1336	MAMDC2	Q7Z304	MAM domain-containing protein 2	rs13107325	103188709	4	T	C	0.075	0.196	0.014	7.6E-46	trans
1336	MAMDC2	Q7Z304	MAM domain-containing protein 2	rs62572585	72592304	9	A	C	0.258	0.224	0.008	1.3E-161	cis
1336	MAMDC2	Q7Z304	MAM domain-containing protein 2	rs147233090	44028047	15	C	T	0.976	0.184	0.024	1.2E-14	trans
1336	MAMDC2	Q7Z304	MAM domain-containing protein 2	rs11854097	75995221	15	A	G	0.744	0.058	0.008	2.6E-12	trans
1336	MAMDC2	Q7Z304	MAM domain-containing protein 2	rs186021206	7069412	17	A	G	0.006	0.476	0.050	7.4E-22	trans
1336	MAMDC2	Q7Z304	MAM domain-containing protein 2	rs11701038	47472736	21	T	C	0.162	0.068	0.010	6.8E-12	trans
1337	MAMDC4	Q6UXC1	Apical endosomal glycoprotein	rs12740374	109817590	1	G	T	0.779	0.162	0.009	2.5E-75	trans
1337	MAMDC4	Q6UXC1	Apical endosomal glycoprotein	rs1260326	27730940	2	T	C	0.392	0.135	0.008	2.7E-72	trans
1337	MAMDC4	Q6UXC1	Apical endosomal glycoprotein	rs13240065	73015369	7	G	A	0.873	0.178	0.011	2.8E-59	trans
1338	MAN1A2	O60476	Mannosyl-oligosaccharide 1,2-alpha-mannosidase IB	rs12740374	109817590	1	G	T	0.779	0.232	0.009	1.5E-160	trans
1338	MAN1A2	O60476	Mannosyl-oligosaccharide 1,2-alpha-mannosidase IB	rs10923344	118096628	1	A	G	0.618	0.193	0.007	2.4E-152	cis
1338	MAN1A2	O60476	Mannosyl-oligosaccharide 1,2-alpha-mannosidase IB	rs4846913	230294715	1	A	C	0.607	0.052	0.007	1.8E-12	trans
1338	MAN1A2	O60476	Mannosyl-oligosaccharide 1,2-alpha-mannosidase IB	rs13153570	115284614	5	A	G	0.556	0.051	0.007	3.2E-13	trans
1338	MAN1A2	O60476	Mannosyl-oligosaccharide 1,2-alpha-mannosidase IB	rs10982154	117087212	9	A	T	0.069	0.124	0.015	2.4E-17	trans
1338	MAN1A2	O60476	Mannosyl-oligosaccharide 1,2-alpha-mannosidase IB	rs56278466	17875857	10	G	T	0.661	0.133	0.007	4.4E-72	trans
1338	MAN1A2	O60476	Mannosyl-oligosaccharide 1,2-alpha-mannosidase IB	rs77542162	67081278	17	G	A	0.023	0.234	0.023	1.4E-23	trans
1338	MAN1A2	O60476	Mannosyl-oligosaccharide 1,2-alpha-mannosidase IB	rs28873836	52314655	19	G	C	0.734	0.198	0.008	2.4E-135	trans
1339	MAN2B2	Q9Y2E5	Epididymis-specific alpha-mannosidase	rs114165349	27021913	1	C	G	0.023	0.141	0.018	1.9E-15	trans
1339	MAN2B2	Q9Y2E5	Epididymis-specific alpha-mannosidase	rs687339	135932359	3	T	C	0.771	0.073	0.006	1.7E-30	trans
1339	MAN2B2	Q9Y2E5	Epididymis-specific alpha-mannosidase	rs2301791	6599684	4	A	T	0.478	0.824	0.007	0.0E+00	cis
1339	MAN2B2	Q9Y2E5	Epididymis-specific alpha-mannosidase	rs77945361	154254534	4	G	A	0.852	0.077	0.010	3.5E-14	trans
1339	MAN2B2	Q9Y2E5	Epididymis-specific alpha-mannosidase	rs4895864	130364482	6	T	C	0.575	0.055	0.005	9.9E-25	trans
1339	MAN2B2	Q9Y2E5	Epididymis-specific alpha-mannosidase	rs9414801	65149089	10	G	A	0.467	0.048	0.005	5.4E-19	trans
1339	MAN2B2	Q9Y2E5	Epididymis-specific alpha-mannosidase	rs41280378	74692646	10	T	G	0.957	0.102	0.013	7.3E-15	trans
1339	MAN2B2	Q9Y2E5	Epididymis-specific alpha-mannosidase	rs145078947	93652974	14	T	G	0.003	0.758	0.053	4.7E-46	trans
1339	MAN2B2	Q9Y2E5	Epididymis-specific alpha-mannosidase	rs58542926	19379549	19	T	C	0.075	0.193	0.010	3.4E-81	trans
1339	MAN2B2	Q9Y2E5	Epididymis-specific alpha-mannosidase	rs429358	45411941	19	T	C	0.845	0.091	0.007	2.3E-34	trans
1339	MAN2B2	Q9Y2E5	Epididymis-specific alpha-mannosidase	rs738409	44324727	22	G	C	0.217	0.066	0.006	9.8E-25	trans
1340	MANEAL	Q5VSG8	Glycoprotein endo-alpha-1,2-mannosidase-like protein	rs11016919	131572206	10	T	C	0.137	0.102	0.011	3.2E-19	trans
1341	MANF	P55145	Mesencephalic astrocyte-derived neurotrophic factor	rs11721253	51547219	3	G	A	0.865	0.098	0.011	4.3E-19	cis
1341	MANF	P55145	Mesencephalic astrocyte-derived neurotrophic factor	rs1354034	56849749	3	C	T	0.604	0.065	0.008	2.0E-17	trans
1341	MANF	P55145	Mesencephalic astrocyte-derived neurotrophic factor	rs3804749	122833003	3	C	T	0.407	0.052	0.008	1.7E-11	trans

1341	MANF	P55145	Mesencephalic astrocyte-derived neurotrophic factor	rs342298	106373646	7	C	T	0.546	0.065	0.008	1.1E-17	trans
1341	MANF	P55145	Mesencephalic astrocyte-derived neurotrophic factor	rs6993770	106581528	8	A	T	0.713	0.059	0.008	1.3E-12	trans
1341	MANF	P55145	Mesencephalic astrocyte-derived neurotrophic factor	rs892090	55539072	19	G	T	0.834	0.073	0.010	7.5E-13	trans
1342	MANSC1	Q9H8J5	MANSC domain-containing protein 1	rs3917932	36943916	1	C	G	0.424	0.057	0.008	6.0E-14	trans
1342	MANSC1	Q9H8J5	MANSC domain-containing protein 1	rs61747728	179526214	1	T	C	0.038	0.182	0.019	1.2E-20	trans
1342	MANSC1	Q9H8J5	MANSC domain-containing protein 1	rs1260326	27730940	2	C	T	0.609	0.082	0.008	8.8E-27	trans
1342	MANSC1	Q9H8J5	MANSC domain-containing protein 1	rs6705658	62539316	2	T	C	0.642	0.053	0.008	7.0E-12	trans
1342	MANSC1	Q9H8J5	MANSC domain-containing protein 1	rs75166367	162964301	2	G	A	0.940	0.122	0.016	6.8E-15	trans
1342	MANSC1	Q9H8J5	MANSC domain-containing protein 1	rs11721168	58408679	3	A	G	0.376	0.056	0.008	3.7E-13	trans
1342	MANSC1	Q9H8J5	MANSC domain-containing protein 1	rs112926042	718590	4	T	C	0.309	0.056	0.008	5.2E-12	trans
1342	MANSC1	Q9H8J5	MANSC domain-containing protein 1	rs4861708	187157233	4	G	A	0.488	0.073	0.007	1.9E-22	trans
1342	MANSC1	Q9H8J5	MANSC domain-containing protein 1	rs2143466	32309323	6	C	T	0.614	0.063	0.008	1.6E-16	trans
1342	MANSC1	Q9H8J5	MANSC domain-containing protein 1	rs11137085	6877468	8	C	G	0.385	0.059	0.008	4.9E-14	trans
1342	MANSC1	Q9H8J5	MANSC domain-containing protein 1	rs495828	136154867	9	G	T	0.792	0.088	0.009	1.1E-21	trans
1342	MANSC1	Q9H8J5	MANSC domain-containing protein 1	rs1059091	309127	11	A	G	0.682	0.060	0.008	9.4E-14	trans
1342	MANSC1	Q9H8J5	MANSC domain-containing protein 1	rs73017394	126181847	11	C	A	0.761	0.139	0.009	6.9E-56	trans
1342	MANSC1	Q9H8J5	MANSC domain-containing protein 1	rs9506687	22035802	13	A	G	0.268	0.061	0.008	4.5E-13	trans
1342	MANSC1	Q9H8J5	MANSC domain-containing protein 1	rs4997081	20365234	16	G	C	0.808	0.068	0.010	8.2E-13	trans
1342	MANSC1	Q9H8J5	MANSC domain-containing protein 1	rs186021206	7069412	17	A	G	0.006	0.735	0.051	3.0E-46	trans
1342	MANSC1	Q9H8J5	MANSC domain-containing protein 1	rs12941811	38159335	17	T	C	0.425	0.063	0.008	5.4E-17	trans
1342	MANSC1	Q9H8J5	MANSC domain-containing protein 1	rs55715868	79274825	17	A	G	0.463	0.059	0.008	3.0E-14	trans
1343	MANSC4	A6NHS7	MANSC domain-containing protein 4	rs17482078	96118866	5	T	C	0.225	0.053	0.007	7.4E-13	trans
1343	MANSC4	A6NHS7	MANSC domain-containing protein 4	rs9275109	32649676	6	G	T	0.382	0.135	0.006	6.0E-96	trans
1343	MANSC4	A6NHS7	MANSC domain-containing protein 4	rs10995299	64504203	10	T	C	0.411	0.076	0.006	7.2E-33	trans
1344	MAP1LC3B2	A6NCE7	Microtubule-associated proteins 1A/1B light chain 3 beta 2	rs58534292	106483584	14	T	C	0.349	0.057	0.008	3.3E-12	trans
1344	MAP1LC3B2	A6NCE7	Microtubule-associated proteins 1A/1B light chain 3 beta 2	rs2857338	106382849	14	G	T	0.498	0.092	0.009	4.3E-25	trans
1345	MAP2	P11137	Microtubule-associated protein 2	rs1342442	156466699	1	A	G	0.653	0.073	0.008	9.8E-21	trans
1345	MAP2	P11137	Microtubule-associated protein 2	rs113770841	210360867	2	C	G	0.015	0.435	0.031	6.1E-46	cis
1345	MAP2	P11137	Microtubule-associated protein 2	rs78909033	241510903	2	A	G	0.136	0.114	0.011	1.7E-25	trans
1345	MAP2	P11137	Microtubule-associated protein 2	rs1354034	56849749	3	C	T	0.604	0.071	0.008	1.2E-20	trans
1345	MAP2	P11137	Microtubule-associated protein 2	rs342293	106372219	7	C	G	0.540	0.059	0.007	3.4E-15	trans
1345	MAP2	P11137	Microtubule-associated protein 2	rs10418205	6100433	19	A	G	0.626	0.059	0.008	3.3E-14	trans
1346	MAP2K1	Q02750	Dual specificity mitogen-activated protein kinase kinase 1	rs1354034	56849749	3	C	T	0.605	0.076	0.008	6.3E-24	trans
1346	MAP2K1	Q02750	Dual specificity mitogen-activated protein kinase kinase 1	rs6993770	106581528	8	A	T	0.713	0.065	0.008	3.8E-15	trans
1346	MAP2K1	Q02750	Dual specificity mitogen-activated protein kinase kinase 1	rs12245367	65321464	10	C	T	0.519	0.054	0.007	3.8E-13	trans
1346	MAP2K1	Q02750	Dual specificity mitogen-activated protein kinase kinase 1	rs77428206	66776480	15	A	C	0.096	0.086	0.013	1.5E-11	cis
1347	MAP2K6	P52564	Dual specificity mitogen-activated protein kinase kinase 6	rs342298	106373646	7	C	T	0.545	0.064	0.008	1.8E-17	trans
1347	MAP2K6	P52564	Dual specificity mitogen-activated protein kinase kinase 6	rs7896518	65104500	10	G	A	0.424	0.060	0.008	6.1E-15	trans
1348	MAP3K5	Q99683	Mitogen-activated protein kinase kinase kinase 5	rs1354034	56849749	3	C	T	0.604	0.055	0.008	2.3E-13	trans
1349	MAP4K5	Q9Y4K4	Mitogen-activated protein kinase kinase kinase kinase 5	rs17780143	50901768	14	G	A	0.943	0.373	0.016	8.1E-119	cis
1350	MAPK13	O15264	Mitogen-activated protein kinase 13	rs12210904	36098191	6	A	C	0.294	0.066	0.009	1.3E-14	cis
1351	MAPK9	P45984	Mitogen-activated protein kinase 9	rs3763131	179734312	5	C	T	0.195	0.143	0.009	2.5E-51	cis
1352	MAPKAPK2	P49137	MAP kinase-activated protein kinase 2	rs11119390	206901382	1	G	C	0.299	0.147	0.008	1.6E-71	cis
1353	MAPRE3	Q9UPY8	Microtubule-associated protein RP/EB family member 3	rs71534539	32512754	6	G	A	0.536	0.125	0.009	3.2E-48	trans
1353	MAPRE3	Q9UPY8	Microtubule-associated protein RP/EB family member 3	rs952558	81288734	8	T	A	0.626	0.061	0.008	1.2E-14	trans
1354	MARCO	Q9UEW3	Macrophage receptor MARCO	rs12740374	109817590	1	G	T	0.779	0.130	0.009	4.1E-51	trans
1354	MARCO	Q9UEW3	Macrophage receptor MARCO	rs4672487	62495245	2	T	G	0.432	0.092	0.007	3.0E-35	trans
1354	MARCO	Q9UEW3	Macrophage receptor MARCO	rs35288880	119728472	2	C	T	0.837	0.340	0.010	3.3E-255	cis
1354	MARCO	Q9UEW3	Macrophage receptor MARCO	rs62165726	134966562	2	C	A	0.963	0.365	0.019	6.8E-79	trans
1354	MARCO	Q9UEW3	Macrophage receptor MARCO	rs59859734	98480932	3	A	T	0.586	0.070	0.007	1.3E-21	trans
1354	MARCO	Q9UEW3	Macrophage receptor MARCO	rs12667978	128777461	7	T	C	0.570	0.055	0.007	4.6E-14	trans
1354	MARCO	Q9UEW3	Macrophage receptor MARCO	rs73338697	94982593	14	G	A	0.993	0.309	0.043	1.2E-12	trans
1354	MARCO	Q9UEW3	Macrophage receptor MARCO	rs11861563	75209790	16	G	A	0.414	0.062	0.007	4.1E-17	trans
1354	MARCO	Q9UEW3	Macrophage receptor MARCO	rs186021206	7069412	17	A	G	0.006	1.090	0.049	2.2E-109	trans
1355	MARS1	P56192	Methionine--tRNA ligase, cytoplasmic	rs1354034	56849749	3	C	T	0.604	0.076	0.008	2.1E-23	trans
1355	MARS1	P56192	Methionine--tRNA ligase, cytoplasmic	rs9328454	8082613	6	C	T	0.710	0.056	0.008	1.0E-11	trans
1355	MARS1	P56192	Methionine--tRNA ligase, cytoplasmic	rs342298	106373646	7	C	T	0.545	0.058	0.008	1.9E-14	trans
1356	MASP1	P48740	Mannan-binding lectin serine protease 1	rs3765901	11104676	1	A	G	0.789	0.123	0.009	1.4E-43	trans
1356	MASP1	P48740	Mannan-binding lectin serine protease 1	rs3820897	3642361	2	C	T	0.821	0.192	0.010	6.9E-90	trans
1356	MASP1	P48740	Mannan-binding lectin serine protease 1	rs1486236	180739450	2	A	C	0.374	0.314	0.008	0.0E+00	trans
1356	MASP1	P48740	Mannan-binding lectin serine protease 1	rs698090	186964300	3	T	C	0.644	0.213	0.008	8.9E-171	cis
1356	MASP1	P48740	Mannan-binding lectin serine protease 1	rs9987289	9183358	8	A	G	0.092	0.091	0.012	1.6E-13	trans
1356	MASP1	P48740	Mannan-binding lectin serine protease 1	rs41341748	16012594	8	A	G	0.011	0.257	0.034	4.1E-14	trans
1356	MASP1	P48740	Mannan-binding lectin serine protease 1	rs10104003	55422440	8	T	C	0.210	0.099	0.009	2.9E-29	trans
1356	MASP1	P48740	Mannan-binding lectin serine protease 1	rs7899547	54536839	10	G	T	0.653	0.133	0.008	3.7E-70	trans
1356	MASP1	P48740	Mannan-binding lectin serine protease 1	rs4926	57381989	11	G	A	0.726	0.088	0.008	5.4E-28	trans
1356	MASP1	P48740	Mannan-binding lectin serine protease 1	rs6118	95053863	14	T	C	0.100	0.100	0.012	3.4E-17	trans
1356	MASP1	P48740	Mannan-binding lectin serine protease 1	rs704	26694861	17	G	A	0.527	0.071	0.007	2.7E-23	trans
1356	MASP1	P48740	Mannan-binding lectin serine protease 1	rs9944724	60184611	18	G	T	0.377	0.060	0.007	2.8E-16	trans
1357	MATN2	O00339	Matrilin-2	rs13159365	135389433	5	T	C	0.510	0.080	0.007	1.4E-30	trans
1357	MATN2	O00339	Matrilin-2	rs2685412	27810603	8	G	C	0.271	0.257	0.008	5.8E-213	trans
1357	MATN2	O00339	Matrilin-2	rs1869609	98991221	8	G	A	0.663	0.396	0.008	0.0E+00	cis
1357	MATN2	O00339	Matrilin-2	rs35157100	117900293	9	G	GA	0.708	0.168	0.008	2.0E-106	trans
1357	MATN2	O00339	Matrilin-2	rs2269434	47360412	11	C	T	0.347	0.052	0.007	1.2E-12	trans
1357	MATN2	O00339	Matrilin-2	rs77542162	67081278	17	G	A	0.023	0.161	0.023	3.8E-12	trans
1358	MATN3	O15232	Matrilin-3	rs11694716	20207619	2	G	A	0.364	0.564	0.008	0.0E+00	cis
1358	MATN3	O15232	Matrilin-3	rs66530140	187161211	4	T	C	0.487	0.058	0.007	6.7E-18	trans
1358	MATN3	O15232	Matrilin-3	rs2519093	136141870	9	C	T	0.816	0.086	0.009	3.0E-23	trans
1358	MATN3	O15232	Matrilin-3	rs11657101	60636826	17	G	A	0.638	0.119	0.007	1.9E-64	trans
1359	MAVS	Q7Z434	Mitochondrial antiviral-signaling protein	rs7080386	65048306	10	A	C	0.412	0.055	0.008	3.8E-13	trans
1359	MAVS	Q7Z434	Mitochondrial antiviral-signaling protein	rs45437096	3844929	20	C	T	0.855	0.135	0.011	7.5E-34	cis
1360	MAX	P61244	Protein max	rs17622656	131820997	5	G	A	0.612	0.059	0.008	8.6E-14	trans
1360	MAX	P61244	Protein max	rs342298	106373646	7	C	T	0.546	0.054	0.008	2.6E-12	trans
1360	MAX	P61244	Protein max	rs13095	64914372	10	C	G	0.469	0.059	0.008	1.7E-14	trans
1360	MAX	P61244	Protein max	rs8005829	65576165	14	T	C	0.583	0.078	0.008	5.2E-24	cis
1360	MAX	P61244	Protein max	rs12588296	107083740	14	A	C	0.749	0.067	0.009	6.4E-14	trans
1361	MB	P02144	Myoglobin	rs12463674	179432185	2	G	A	0.316	0.076	0.007	2.7E-27	trans
1361	MB	P02144	Myoglobin	rs2151597	103343401	9	A	G	0.439	0.050	0.007	1.1E-14	trans
1361	MB	P02144	Myoglobin	rs2234962	121429633	10	C	T	0.216	0.056	0.008	1.1E-12	trans
1361	MB	P02144	Myoglobin	rs1028883	74108587	13	G	T	0.571	0.065	0.007	1.7E-22	trans
1361	MB	P02144	Myoglobin	rs4901541	55235282	14	C	T	0.439	0.054	0.007	2.3E-16	trans
1361	MB	P02144	Myoglobin	rs1799938	65052304	17	A	G	0.114	0.071	0.010	3.1E-12	trans
1362	MBL2	P11226	Mannose-binding protein C	rs1260326	27730940	2	C	T	0.608	0.065	0.005	1.4E-37	trans
1362	MBL2	P11226	Mannose-binding protein C	rs9398803	126683594	6	G	A	0.473	0.049	0.005	1.0E-22	trans
1362	MBL2	P11226	Mannose-binding protein C	rs112875651	126506694	8	A	G	0.391	0.038	0.005	6.8E-14	trans
1362	MBL2	P11226	Mannose-binding protein C	rs7899547	54536839	10	G	T	0.654	0.915	0.008	0.0E+00	cis
1362	MBL2	P11226	Mannose-binding protein C	rs145162217	109933024	X	T	C	0.474	0.040	0.004	3.7E-19	trans
1363	MCAM	P43121	Cell surface glycoprotein MUC18	rs1260326	27730940	2	C	T	0.608	0.069	0.007	2.7E-21	trans
1363	MCAM	P43121	Cell surface glycoprotein MUC18	rs13107325	103188709	4	T	C	0.075	0.105	0.014	9.6E-15	trans
1363	MCAM	P43121	Cell surface glycoprotein MUC18	rs41289902	112460365	6	T	C	0.014	0.484	0.030	8.6E-59	trans
1363	MCAM	P43121	Cell surface glycoprotein MUC18	rs8176693	136137657	9	T	C	0.061	0.229	0.015	1.0E-53	trans
1363	MCAM	P43121	Cell surface glycoprotein MUC18	rs34587557	119185677	11	T	C	0.949	0.324	0.016	6.6E-89	cis
1363	MCAM	P43121	Cell surface glycoprotein MUC18	rs11220462	126243952	11	G	A	0.867	0.099	0.011	5.7E-21	trans
1363	MCAM	P43121	Cell surface glycoprotein MUC18	rs186021206	7069412	17	A	G	0.006	0.967	0.049	9.8E-87	trans
1363	MCAM	P43121	Cell surface glycoprotein MUC18	rs7286917	39860868	22	A	G	0.255	0.075	0.008	9.2E-19	trans
1364	MCEE	Q96PE7	Methylmalonyl-CoA epimerase, mitochondrial	rs6716795	71364623	2	T	C	0.464	0.188	0.008	4.1E-132	cis
1365	MCEMP1	Q8IX19	Mast cell-expressed membrane protein 1	rs1342442	156466699	1	A	G	0.653	0.113	0.008	3.7E-47	trans
1365	MCEMP1	Q8IX19	Mast cell-expressed membrane protein 1	rs78909033	241510903	2	A	G	0.136	0.103	0.011	3.0E-21	trans
1365	MCEMP1	Q8IX19	Mast cell-expressed membrane protein 1	rs1354034	56849749	3	C	T	0.604	0.053	0.008	2.1E-12	trans
1365	MCEMP1	Q8IX19	Mast cell-expressed membrane protein 1	rs114694170	88180196	5	T	C	0.940	0.200	0.016	1.7E-36	trans
1365	MCEMP1	Q8IX19	Mast cell-expressed membrane protein 1	rs6993770	106581528	8	A	T	0.713	0.121	0.008	3.2E-49	trans
1365	MCEMP1	Q8IX19	Mast cell-expressed membrane protein 1	rs10820606	99192919	9	C	A	0.229	0.156	0.009	6.7E-66	trans
1365	MCEMP1	Q8IX19	Mast cell-expressed membrane protein 1	rs8193004	135866154	9	C	T	0.398	0.083	0.008	9.0E-27	trans
1365	MCEMP1	Q8IX19	Mast cell-expressed membrane protein 1	rs10776883	138999156	9	T	C	0.312	0.278	0.008	9.5E-250	trans
1365	MCEMP1	Q8IX19	Mast cell-expressed membrane protein 1	rs73000929	113953622	11	G	A	0.963	0.168	0.020	1.2E-17	trans
1365	MCEMP1	Q8IX19	Mast cell-expressed membrane protein 1	rs4783183	85415465	16	C	G	0.126	0.109	0.011	2.4E-22	trans
1365	MCEMP1	Q8IX19	Mast cell-expressed membrane protein 1	rs3848232	88566147	16	C	G	0.295	0.059	0.008	7.7E-13	trans
1365	MCEMP1	Q8IX19	Mast cell-expressed membrane protein 1	rs141879681	7719439	19	G	A	0.006	1.432	0.049	6.6E-187	cis
1366	MCFD2	Q8NI22	Multiple coagulation factor deficiency protein 2	rs6419615	47152291	2	G	C	0.293	0.146	0.008	3.2E-68	cis
1366	MCFD2	Q8NI22	Multiple coagulation factor deficiency protein 2	rs6993770	106581528	8	A	T	0.713	0.079	0.008	2.9E-21	trans
1366	MCFD2	Q8NI22	Multiple coagulation factor deficiency protein 2	rs112635299	94838142	14	T	G	0.022	0.346	0.026	1.5E-40	trans
1366	MCFD2	Q8NI22	Multiple coagulation factor deficiency protein 2	rs17696641	57015800	18	A	T	0.148	0.085	0.011	1.2E-15	trans
1367	MCTS1	Q9ULC4	Malignant T-cell-amplified sequence 1	rs72508413	32454597	6	A	G	0.657	0.102	0.008	4.0E-36	trans
1368	MDGA1	Q8NFP4	MAM domain-containing glycosylphosphatidylinositol anchor protein 1	rs9349050	37663624	6	T	C	0.486	1.080	0.007	0.0E+00	cis
1368	MDGA1	Q8NFP4	MAM domain-containing glycosylphosphatidylinositol anchor protein 1	rs9987289	9183358	8	A	G	0.092	0.062	0.008	5.8E-14	trans
1368	MDGA1	Q8NFP4	MAM domain-containing glycosylphosphatidylinositol anchor protein 1	rs35887873	126219396	11	C	T	0.228	0.068	0.006	1.8E-33	trans
1368	MDGA1	Q8NFP4	MAM domain-containing glycosylphosphatidylinositol anchor protein 1	rs186021206	7069412	17	A	G	0.006	0.508	0.033	1.1E-54	trans
1368	MDGA1	Q8NFP4	MAM domain-containing glycosylphosphatidylinositol anchor protein 1	rs77542162	67081278	17	G	A	0.023	0.139	0.016	1.5E-18	trans
1368	MDGA1	Q8NFP4	MAM domain-containing glycosylphosphatidylinositol anchor protein 1	rs7287617	39897163	22	A	G	0.645	0.039	0.005	4.8E-15	trans
1369	MDH1	P40925	Malate dehydrogenase, cytoplasmic	rs71422399	64213877	2	T	C	0.265	0.092	0.008	1.2E-27	cis
1370	MDK	P21741	Midkine	rs6427756	199000727	1	A	G	0.265	0.081	0.008	1.2E-21	trans
1370	MDK	P21741	Midkine	rs7618405	18250509	3	C	A	0.795	0.067	0.009	4.5E-13	trans
1370	MDK	P21741	Midkine	rs1354034	56849749	3	T	C	0.396	0.059	0.008	6.9E-15	trans
1370	MDK	P21741	Midkine	rs9358555	22481782	6	A	G	0.861	0.073	0.011	1.2E-11	trans
1370	MDK	P21741	Midkine	rs6961069	80218961	7	T	C	0.402	0.052	0.008	9.9E-12	trans
1370	MDK	P21741	Midkine	rs6993770	106581528	8	A	T	0.714	0.081	0.008	7.7E-23	trans
1370	MDK	P21741	Midkine	rs7080386	65048306	10	A	C	0.412	0.069	0.008	4.1E-20	trans
1370	MDK	P21741	Midkine	rs117125830	56019770	11	C	T	0.994	0.515	0.049	3.9E-26	trans
1370	MDK	P21741	Midkine	rs12445050	81870969	16	T	C	0.138	0.075	0.011	3.6E-12	trans
1370	MDK	P21741	Midkine	rs57839578	5268337	17	A	T	0.521	0.052	0.007	3.4E-12	trans
1370	MDK	P21741	Midkine	rs892090	55539072	19	G	T	0.834	0.123	0.010	1.6E-34	trans
1370	MDK	P21741	Midkine	rs14142	43974067	20	C	T	0.627	0.062	0.008	1.2E-15	trans
1371	MECR	Q9BV79	Enoyl-[acyl-carrier-protein] reductase, mitochondrial	rs144612117	29544575	1	CCCTCTGGAAG	C	0.135	0.107	0.011	2.4E-21	cis

1371	MECR	Q9BV79	Enoyl-[acyl-carrier-protein] reductase, mitochondrial	rs11242109	131677047	5	G	T	0.519	0.057	0.008	1.0E-13	trans
1371	MECR	Q9BV79	Enoyl-[acyl-carrier-protein] reductase, mitochondrial	rs6993770	106581528	8	A	T	0.713	0.063	0.008	5.6E-14	trans
1371	MECR	Q9BV79	Enoyl-[acyl-carrier-protein] reductase, mitochondrial	rs7896518	65104500	10	G	A	0.424	0.054	0.008	4.5E-12	trans
1372	MED18	Q9BUE0	Mediator of RNA polymerase II transcription subunit 18	rs12245149	65321147	10	C	A	0.512	0.057	0.008	7.9E-14	trans
1372	MED18	Q9BUE0	Mediator of RNA polymerase II transcription subunit 18	rs200309755	27845774	17	T	C	0.537	0.055	0.008	3.9E-12	trans
1373	MEGF10	Q96KG7	Multiple epidermal growth factor-like domains protein 10	rs62276885	98696852	3	T	G	0.181	0.060	0.009	8.3E-12	trans
1373	MEGF10	Q96KG7	Multiple epidermal growth factor-like domains protein 10	rs112394041	126628584	5	CT	C	0.458	0.398	0.007	0.0E+00	cis
1373	MEGF10	Q96KG7	Multiple epidermal growth factor-like domains protein 10	rs6938239	34683635	6	G	A	0.139	0.066	0.010	9.8E-12	trans
1373	MEGF10	Q96KG7	Multiple epidermal growth factor-like domains protein 10	rs200889152	117507982	6	A	C	0.625	0.065	0.007	5.3E-21	trans
1373	MEGF10	Q96KG7	Multiple epidermal growth factor-like domains protein 10	rs10813959	33166234	9	C	T	0.422	0.077	0.007	4.2E-30	trans
1373	MEGF10	Q96KG7	Multiple epidermal growth factor-like domains protein 10	rs508196	18263873	10	C	T	0.564	0.053	0.007	7.7E-15	trans
1373	MEGF10	Q96KG7	Multiple epidermal growth factor-like domains protein 10	rs7232	59940599	11	A	T	0.374	0.051	0.007	1.4E-13	trans
1373	MEGF10	Q96KG7	Multiple epidermal growth factor-like domains protein 10	rs2236653	126283785	11	T	C	0.426	0.051	0.007	2.9E-14	trans
1373	MEGF10	Q96KG7	Multiple epidermal growth factor-like domains protein 10	rs3007036	50106045	14	G	A	0.539	0.057	0.007	3.1E-17	trans
1373	MEGF10	Q96KG7	Multiple epidermal growth factor-like domains protein 10	rs186021206	7069412	17	A	G	0.006	0.668	0.047	4.0E-46	trans
1373	MEGF10	Q96KG7	Multiple epidermal growth factor-like domains protein 10	rs77542162	67081278	17	G	A	0.023	0.799	0.023	2.4E-268	trans
1373	MEGF10	Q96KG7	Multiple epidermal growth factor-like domains protein 10	rs6068599	52259618	20	T	C	0.318	0.057	0.007	3.0E-15	trans
1374	MEGF11	A6BM72	Multiple epidermal growth factor-like domains protein 11	rs11630800	66535743	15	T	C	0.211	0.164	0.009	5.0E-68	cis
1374	MEGF11	A6BM72	Multiple epidermal growth factor-like domains protein 11	rs77542162	67081278	17	G	A	0.023	0.411	0.026	8.2E-58	trans
1375	MEGF9	Q9H1U4	Multiple epidermal growth factor-like domains protein 9	rs646776	109818530	1	C	T	0.222	0.084	0.009	6.9E-22	trans
1375	MEGF9	Q9H1U4	Multiple epidermal growth factor-like domains protein 9	rs4652308	178637915	1	C	T	0.475	0.055	0.007	5.0E-14	trans
1375	MEGF9	Q9H1U4	Multiple epidermal growth factor-like domains protein 9	rs13107325	103188709	4	T	C	0.075	0.182	0.014	4.5E-39	trans
1375	MEGF9	Q9H1U4	Multiple epidermal growth factor-like domains protein 9	rs1461729	9187242	8	A	G	0.103	0.089	0.012	1.3E-13	trans
1375	MEGF9	Q9H1U4	Multiple epidermal growth factor-like domains protein 9	rs28601761	126500031	8	C	G	0.581	0.081	0.007	1.4E-27	trans
1375	MEGF9	Q9H1U4	Multiple epidermal growth factor-like domains protein 9	rs10984978	123441375	9	C	T	0.701	0.341	0.008	0.0E+00	cis
1375	MEGF9	Q9H1U4	Multiple epidermal growth factor-like domains protein 9	rs9633740	82265271	10	G	A	0.795	0.079	0.009	2.7E-18	trans
1375	MEGF9	Q9H1U4	Multiple epidermal growth factor-like domains protein 9	rs186021206	7069412	17	A	G	0.006	0.460	0.050	6.3E-20	trans
1375	MEGF9	Q9H1U4	Multiple epidermal growth factor-like domains protein 9	rs77542162	67081278	17	G	A	0.023	0.260	0.024	1.6E-26	trans
1375	MEGF9	Q9H1U4	Multiple epidermal growth factor-like domains protein 9	rs73066226	59008213	19	C	T	0.164	0.083	0.010	3.4E-17	trans
1376	MELTF	P08582	Melanotransferrin	rs8177245	133479230	3	G	A	0.342	0.054	0.008	5.3E-12	trans
1376	MELTF	P08582	Melanotransferrin	rs3773862	196739538	3	C	T	0.547	0.408	0.008	0.0E+00	cis
1376	MELTF	P08582	Melanotransferrin	rs13135092	103198082	4	G	A	0.084	0.101	0.012	4.4E-17	trans
1376	MELTF	P08582	Melanotransferrin	rs55818129	39425092	5	T	G	0.882	0.074	0.010	4.5E-13	trans
1376	MELTF	P08582	Melanotransferrin	rs79220007	26098474	6	T	C	0.923	0.267	0.012	2.1E-103	trans
1376	MELTF	P08582	Melanotransferrin	rs56278466	17875857	10	G	T	0.661	0.107	0.007	1.8E-53	trans
1376	MELTF	P08582	Melanotransferrin	rs174576	61603510	11	A	C	0.352	0.054	0.007	5.5E-15	trans
1376	MELTF	P08582	Melanotransferrin	rs2076085	37470041	22	C	A	0.465	0.057	0.007	2.1E-18	trans
1377	MENT	Q9BUN1	Protein MENT	rs12132412	21820042	1	A	G	0.610	0.081	0.007	8.4E-32	trans
1377	MENT	Q9BUN1	Protein MENT	rs13402561	3639921	2	C	G	0.183	0.092	0.009	3.5E-26	trans
1377	MENT	Q9BUN1	Protein MENT	rs1486236	180739450	2	C	A	0.627	0.072	0.007	9.3E-25	trans
1377	MENT	Q9BUN1	Protein MENT	rs850313	186953226	3	C	G	0.288	0.059	0.007	1.4E-15	trans
1377	MENT	Q9BUN1	Protein MENT	rs1372344	19852939	8	T	A	0.261	0.099	0.008	3.3E-39	trans
1377	MENT	Q9BUN1	Protein MENT	rs964184	116648917	11	C	G	0.867	0.131	0.010	1.2E-40	trans
1377	MENT	Q9BUN1	Protein MENT	rs821840	56993886	16	G	A	0.325	0.122	0.007	2.6E-65	trans
1377	MENT	Q9BUN1	Protein MENT	rs116843064	8429323	19	A	G	0.020	0.201	0.026	5.8E-15	trans
1377	MENT	Q9BUN1	Protein MENT	rs200210321	19393890	19	A	AG	0.926	0.127	0.014	1.5E-20	trans
1377	MENT	Q9BUN1	Protein MENT	rs483082	45416178	19	T	G	0.237	0.567	0.008	0.0E+00	trans
1378	MEP1A	Q16819	Meprin A subunit alpha	rs1047891	211540507	2	A	C	0.313	0.065	0.008	3.5E-18	trans
1378	MEP1A	Q16819	Meprin A subunit alpha	rs299284	162896650	5	T	C	0.106	0.152	0.011	1.3E-41	trans
1378	MEP1A	Q16819	Meprin A subunit alpha	rs2274656	46761495	6	G	A	0.615	0.194	0.007	3.3E-160	cis
1378	MEP1A	Q16819	Meprin A subunit alpha	rs2954021	126482077	8	A	G	0.496	0.047	0.007	1.3E-11	trans
1378	MEP1A	Q16819	Meprin A subunit alpha	rs10733608	117148430	9	G	T	0.511	0.150	0.007	1.8E-98	trans
1378	MEP1A	Q16819	Meprin A subunit alpha	rs10793962	136129115	9	T	A	0.061	0.377	0.015	6.4E-147	trans
1378	MEP1A	Q16819	Meprin A subunit alpha	rs2185756	94430497	10	A	C	0.594	0.077	0.007	4.2E-27	trans
1378	MEP1A	Q16819	Meprin A subunit alpha	rs2862954	101912064	10	T	C	0.498	0.160	0.007	7.5E-117	trans
1378	MEP1A	Q16819	Meprin A subunit alpha	rs339969	60883281	15	A	C	0.617	0.056	0.007	3.0E-15	trans
1378	MEP1A	Q16819	Meprin A subunit alpha	rs12950511	47320938	17	T	C	0.341	0.052	0.007	1.8E-12	trans
1378	MEP1A	Q16819	Meprin A subunit alpha	rs708686	5840619	19	T	C	0.268	0.181	0.008	6.3E-116	trans
1378	MEP1A	Q16819	Meprin A subunit alpha	rs681343	49206462	19	C	T	0.491	0.156	0.007	1.3E-108	trans
1378	MEP1A	Q16819	Meprin A subunit alpha	rs1800961	43042364	20	C	T	0.968	0.244	0.020	8.4E-36	trans
1379	MEP1B	Q16820	Meprin A subunit beta	rs193084249	26987646	1	A	G	0.976	0.153	0.016	2.4E-21	trans
1379	MEP1B	Q16820	Meprin A subunit beta	rs1730862	107614003	1	A	G	0.657	0.041	0.005	1.3E-15	trans
1379	MEP1B	Q16820	Meprin A subunit beta	rs1260326	27730940	2	C	T	0.608	0.058	0.005	7.9E-32	trans
1379	MEP1B	Q16820	Meprin A subunit beta	rs9884482	106081636	4	T	C	0.628	0.036	0.005	3.3E-13	trans
1379	MEP1B	Q16820	Meprin A subunit beta	rs4895864	130364482	6	T	C	0.575	0.039	0.005	7.5E-16	trans
1379	MEP1B	Q16820	Meprin A subunit beta	rs6973520	128740443	7	T	C	0.506	0.036	0.005	7.5E-14	trans
1379	MEP1B	Q16820	Meprin A subunit beta	rs3735165	150068371	7	C	T	0.309	0.072	0.005	2.6E-44	trans
1379	MEP1B	Q16820	Meprin A subunit beta	rs4292651	10572531	8	C	A	0.391	0.033	0.005	1.5E-11	trans
1379	MEP1B	Q16820	Meprin A subunit beta	rs112875651	126506694	8	A	G	0.391	0.042	0.005	6.3E-17	trans
1379	MEP1B	Q16820	Meprin A subunit beta	rs174537	61552680	11	T	G	0.345	0.052	0.005	6.3E-25	trans
1379	MEP1B	Q16820	Meprin A subunit beta	rs17616063	51436882	16	A	G	0.925	0.104	0.009	5.5E-30	trans
1379	MEP1B	Q16820	Meprin A subunit beta	rs16940186	86009740	16	C	T	0.149	0.135	0.007	1.1E-87	trans
1379	MEP1B	Q16820	Meprin A subunit beta	rs7212366	74593181	17	C	T	0.509	0.047	0.005	5.7E-20	trans
1379	MEP1B	Q16820	Meprin A subunit beta	rs680321	29797958	18	T	C	0.544	1.074	0.008	0.0E+00	cis
1379	MEP1B	Q16820	Meprin A subunit beta	rs1800961	43042364	20	T	C	0.032	0.098	0.014	4.0E-13	trans
1380	MEPE	Q9NQ76	Matrix extracellular phosphoglycoprotein	rs6547692	27734972	2	A	G	0.557	0.051	0.007	1.1E-12	trans
1380	MEPE	Q9NQ76	Matrix extracellular phosphoglycoprotein	rs77623237	230114499	2	T	C	0.824	0.144	0.009	1.2E-53	trans
1380	MEPE	Q9NQ76	Matrix extracellular phosphoglycoprotein	rs17013212	88754060	4	C	T	0.863	0.182	0.010	1.1E-69	cis
1380	MEPE	Q9NQ76	Matrix extracellular phosphoglycoprotein	rs4861708	187157233	4	G	A	0.488	0.064	0.007	2.5E-19	trans
1380	MEPE	Q9NQ76	Matrix extracellular phosphoglycoprotein	rs55712380	139610124	6	G	T	0.719	0.053	0.008	1.7E-11	trans
1380	MEPE	Q9NQ76	Matrix extracellular phosphoglycoprotein	rs757980	28725536	7	A	G	0.751	0.068	0.008	1.5E-16	trans
1380	MEPE	Q9NQ76	Matrix extracellular phosphoglycoprotein	rs7842942	120008587	8	C	T	0.566	0.066	0.007	2.3E-20	trans
1380	MEPE	Q9NQ76	Matrix extracellular phosphoglycoprotein	rs544626513	180302	11	C	G	0.229	0.066	0.009	2.9E-14	trans
1380	MEPE	Q9NQ76	Matrix extracellular phosphoglycoprotein	rs3008070	23026451	13	T	G	0.781	0.061	0.009	8.4E-13	trans
1380	MEPE	Q9NQ76	Matrix extracellular phosphoglycoprotein	rs806292	50811031	13	A	G	0.468	0.058	0.007	4.6E-16	trans
1380	MEPE	Q9NQ76	Matrix extracellular phosphoglycoprotein	rs2614763	51142733	15	A	C	0.455	0.100	0.007	2.2E-45	trans
1380	MEPE	Q9NQ76	Matrix extracellular phosphoglycoprotein	rs4776350	67542179	15	G	C	0.231	0.062	0.008	9.6E-14	trans
1380	MEPE	Q9NQ76	Matrix extracellular phosphoglycoprotein	rs10521142	27130155	16	G	A	0.558	0.073	0.007	2.1E-24	trans
1381	MERTK	Q12866	Tyrosine-protein kinase Mer	rs10188640	112741097	2	G	A	0.374	0.266	0.007	9.9E-283	cis
1381	MERTK	Q12866	Tyrosine-protein kinase Mer	rs10935473	98416900	3	G	T	0.560	0.077	0.007	8.6E-28	trans
1381	MERTK	Q12866	Tyrosine-protein kinase Mer	rs139750425	3470174	4	T	TCTGC	0.743	0.055	0.008	7.0E-12	trans
1381	MERTK	Q12866	Tyrosine-protein kinase Mer	rs35371668	32561638	6	C	T	0.819	0.107	0.009	1.9E-30	trans
1381	MERTK	Q12866	Tyrosine-protein kinase Mer	rs11596680	20208483	10	G	A	0.328	0.067	0.008	6.6E-19	trans
1381	MERTK	Q12866	Tyrosine-protein kinase Mer	rs7088799	65016174	10	G	T	0.414	0.072	0.007	1.0E-23	trans
1381	MERTK	Q12866	Tyrosine-protein kinase Mer	rs174546	61569830	11	T	C	0.345	0.073	0.007	6.5E-23	trans
1381	MERTK	Q12866	Tyrosine-protein kinase Mer	rs494144	89198796	11	T	C	0.463	0.082	0.007	2.0E-31	trans
1381	MERTK	Q12866	Tyrosine-protein kinase Mer	rs28929474	94844947	14	T	C	0.021	0.196	0.025	2.0E-15	trans
1381	MERTK	Q12866	Tyrosine-protein kinase Mer	rs423135	89740873	16	A	G	0.518	0.068	0.007	4.4E-22	trans
1381	MERTK	Q12866	Tyrosine-protein kinase Mer	rs186021206	7069412	17	A	G	0.006	0.610	0.048	2.1E-36	trans
1381	MERTK	Q12866	Tyrosine-protein kinase Mer	rs12451766	45621261	17	G	A	0.505	0.059	0.007	6.1E-17	trans
1381	MERTK	Q12866	Tyrosine-protein kinase Mer	rs12609794	35555585	19	A	G	0.749	0.061	0.008	4.7E-14	trans
1381	MERTK	Q12866	Tyrosine-protein kinase Mer	rs111981233	50016479	19	G	T	0.080	0.091	0.013	1.6E-12	trans
1382	MESD	Q14696	LRP chaperone MESD	rs1354034	56849749	3	C	T	0.604	0.069	0.008	2.7E-19	trans
1382	MESD	Q14696	LRP chaperone MESD	rs6993770	106581528	8	A	T	0.714	0.088	0.008	3.1E-26	trans
1383	MET	P08581	Hepatocyte growth factor receptor	rs3768321	40035928	1	G	T	0.806	0.063	0.009	1.8E-12	trans
1383	MET	P08581	Hepatocyte growth factor receptor	rs200287431	49761567	3	TGGCGGATCCAAA	T	0.175	0.068	0.009	3.1E-13	trans
1383	MET	P08581	Hepatocyte growth factor receptor	rs5398	170715830	3	G	A	0.716	0.088	0.008	2.1E-29	trans
1383	MET	P08581	Hepatocyte growth factor receptor	rs1858830	116312439	7	C	G	0.557	0.160	0.008	1.6E-98	cis
1383	MET	P08581	Hepatocyte growth factor receptor	rs7799998	128738950	7	A	G	0.550	0.075	0.007	4.1E-25	trans
1383	MET	P08581	Hepatocyte growth factor receptor	rs507666	136149399	9	G	A	0.815	0.331	0.009	5.1E-278	trans
1383	MET	P08581	Hepatocyte growth factor receptor	rs10740129	65250808	10	A	G	0.470	0.068	0.007	8.4E-22	trans
1383	MET	P08581	Hepatocyte growth factor receptor	rs1878036	82280137	10	G	T	0.795	0.124	0.009	6.7E-46	trans
1383	MET	P08581	Hepatocyte growth factor receptor	rs174547	61570783	11	C	T	0.344	0.104	0.007	2.4E-44	trans
1383	MET	P08581	Hepatocyte growth factor receptor	rs1041316	57099859	14	A	G	0.729	0.082	0.008	8.4E-25	trans
1383	MET	P08581	Hepatocyte growth factor receptor	rs28929474	94844947	14	T	C	0.021	0.315	0.025	6.1E-37	trans
1383	MET	P08581	Hepatocyte growth factor receptor	rs1982784	4666537	16	G	A	0.814	0.068	0.009	6.7E-14	trans
1383	MET	P08581	Hepatocyte growth factor receptor	rs4850	21976762	16	A	G	0.055	0.145	0.015	7.6E-21	trans
1383	MET	P08581	Hepatocyte growth factor receptor	rs186021206	7069412	17	A	G	0.006	0.572	0.049	1.6E-31	trans
1383	MET	P08581	Hepatocyte growth factor receptor	rs77542162	67081278	17	G	A	0.023	0.387	0.024	9.8E-60	trans
1383	MET	P08581	Hepatocyte growth factor receptor	rs10401969	19407718	19	T	C	0.923	0.102	0.013	1.6E-14	trans
1383	MET	P08581	Hepatocyte growth factor receptor	rs112167630	42874901	19	A	G	0.029	0.260	0.021	1.5E-34	trans
1383	MET	P08581	Hepatocyte growth factor receptor	rs914187	42619749	21	T	C	0.400	0.057	0.007	4.1E-15	trans
1384	METAP1D	Q6UB28	Methionine aminopeptidase 1D, mitochondrial	rs138869820	172931008	2	G	A	0.998	0.812	0.093	2.1E-18	cis
1384	METAP1D	Q6UB28	Methionine aminopeptidase 1D, mitochondrial	rs6993770	106581528	8	A	T	0.713	0.065	0.008	4.5E-15	trans
1385	METAP2	P50579	Methionine aminopeptidase 2	rs1354034	56849749	3	C	T	0.604	0.096	0.008	1.8E-36	trans
1385	METAP2	P50579	Methionine aminopeptidase 2	rs6993770	106581528	8	A	T	0.714	0.069	0.008	3.8E-17	trans
1385	METAP2	P50579	Methionine aminopeptidase 2	rs11502185	180258	11	C	T	0.260	0.067	0.010	2.4E-12	trans
1385	METAP2	P50579	Methionine aminopeptidase 2	rs10405535	33072085	19	G	A	0.685	0.096	0.008	3.9E-32	trans
1386	MFAP4	P55083	Microfibril-associated glycoprotein 4	rs12733767	93383672	1	T	C	0.104	0.095	0.012	5.4E-15	trans
1386	MFAP4	P55083	Microfibril-associated glycoprotein 4	rs7636227	52566682	3	A	G	0.553	0.110	0.007	4.0E-50	trans
1386	MFAP4	P55083	Microfibril-associated glycoprotein 4	rs689122	72386729	5	C	T	0.523	0.051	0.007	4.3E-12	trans
1386	MFAP4	P55083	Microfibril-associated glycoprotein 4	rs36189266	32526768	6	G	A	0.336	0.058	0.008	4.9E-13	trans
1386	MFAP4	P55083	Microfibril-associated glycoprotein 4	rs507666	136149399	9	G	A	0.815	0.079	0.010	8.2E-17	trans
1386	MFAP4	P55083	Microfibril-associated glycoprotein 4	rs139356332	19289286	17	G	C	0.977	0.722	0.025	1.8E-180	cis
1386	MFAP4	P55083	Microfibril-associated glycoprotein 4	rs2303729	41111069	19	G	A	0.566	0.053	0.007	1.1E-12	trans
1386	MFAP4	P55083	Microfibril-associated glycoprotein 4	rs738409	44324727	22	G	C	0.217	0.064	0.009	3.9E-13	trans
1387	MFAP5	Q13361	Microfibrillar-associated protein 5	rs4894803	171800256	3	G	A	0.410	0.077	0.007	1.5E-25	trans
1387	MFAP5	Q13361	Microfibrillar-associated protein 5	rs1918352	99763439	7	A	G	0.552	0.051	0.007	4.5E-12	trans
1387	MFAP5	Q13361	Microfibrillar-associated protein 5	rs532436	136149830	9	G	A	0.815	0.083	0.009	1.0E-18	trans
1387	MFAP5	Q13361	Microfibrillar-associated protein 5	rs1126464	89704365	16	G	C	0.756	0.059	0.008	1.8E-12	trans
1387	MFAP5	Q13361	Microfibrillar-associated protein 5	rs7412	45412079	19	T	C	0.081	0.096	0.013	5.2E-13	trans
1388	MFGE8	Q08431	Lactadherin	rs1260326	27730940	2	T	C	0.392	0.065	0.007	9.5E-19	trans
1388	MFGE8	Q08431	Lactadherin	rs11134475	156399950	5	G	A	0.639	0.057	0.008	5.3E-14	trans

1388	MFGE8	Q08431	Lactadherin	rs521977	31836827	6	G	T	0.677	0.064	0.008	8.5E-17	trans
1388	MFGE8	Q08431	Lactadherin	rs17145750	73026378	7	C	T	0.840	0.080	0.010	7.1E-16	trans
1388	MFGE8	Q08431	Lactadherin	rs28601761	126500031	8	C	G	0.581	0.061	0.007	1.1E-16	trans
1388	MFGE8	Q08431	Lactadherin	rs7232	59940599	11	A	T	0.373	0.188	0.008	2.6E-136	trans
1388	MFGE8	Q08431	Lactadherin	rs964184	116648917	11	G	C	0.133	0.163	0.011	2.3E-52	trans
1388	MFGE8	Q08431	Lactadherin	rs6602913	114544125	13	C	A	0.366	0.063	0.007	3.6E-17	trans
1388	MFGE8	Q08431	Lactadherin	rs34239095	89455768	15	G	C	0.282	0.373	0.008	0.0E+00	cis
1389	MGLL	Q99685	Monoglyceride lipase	rs7578	127408239	3	A	G	0.557	0.060	0.008	1.3E-15	cis
1389	MGLL	Q99685	Monoglyceride lipase	rs6993770	106581528	8	A	T	0.713	0.091	0.008	7.9E-28	trans
1389	MGLL	Q99685	Monoglyceride lipase	rs77303550	72079657	16	T	C	0.196	0.066	0.009	3.7E-12	trans
1390	MGMT	P16455	Methylated-DNA--protein-cysteine methyltransferase	rs1354034	56849749	3	C	T	0.604	0.055	0.007	1.7E-14	trans
1390	MGMT	P16455	Methylated-DNA--protein-cysteine methyltransferase	rs342298	106373646	7	C	T	0.545	0.055	0.007	9.7E-15	trans
1390	MGMT	P16455	Methylated-DNA--protein-cysteine methyltransferase	rs1008982	131445731	10	T	C	0.627	0.414	0.008	0.0E+00	cis
1391	MIA	Q16674	Melanoma-derived growth regulatory protein	rs4917	186337713	3	C	T	0.640	0.057	0.007	1.1E-17	trans
1391	MIA	Q16674	Melanoma-derived growth regulatory protein	rs60028714	86393300	4	C	T	0.378	0.055	0.007	2.9E-16	trans
1391	MIA	Q16674	Melanoma-derived growth regulatory protein	rs3132568	31101917	6	A	G	0.744	0.052	0.007	1.2E-12	trans
1391	MIA	Q16674	Melanoma-derived growth regulatory protein	rs77542162	67081278	17	G	A	0.023	0.271	0.022	3.1E-36	trans
1391	MIA	Q16674	Melanoma-derived growth regulatory protein	rs2607421	41277077	19	G	A	0.073	1.383	0.014	0.0E+00	cis
1392	MICALL2	Q8IY33	MICAL-like protein 2	rs2289035	1538614	7	A	G	0.398	0.069	0.008	4.5E-19	cis
1393	MICB_MICA	Q29980,Q29983	MHC class I polypeptide-related sequence A_MHC class I polypeptide-related sequence B	rs2009581	111807677	2	G	A	0.728	0.030	0.004	1.0E-12	trans
1393	MICB_MICA	Q29980,Q29983	MHC class I polypeptide-related sequence A_MHC class I polypeptide-related sequence B	rs10935473	98416900	3	G	T	0.560	0.100	0.004	3.9E-157	trans
1393	MICB_MICA	Q29980,Q29983	MHC class I polypeptide-related sequence A_MHC class I polypeptide-related sequence B	rs3132467	31380943	6	G	A	0.743	1.102	0.009	0.0E+00	cis
1393	MICB_MICA	Q29980,Q29983	MHC class I polypeptide-related sequence A_MHC class I polypeptide-related sequence B	rs35715239	55624602	15	G	C	0.666	0.055	0.004	2.3E-44	trans
1393	MICB_MICA	Q29980,Q29983	MHC class I polypeptide-related sequence A_MHC class I polypeptide-related sequence B	rs186021206	7069412	17	A	G	0.006	0.322	0.026	2.1E-36	trans
1393	MICB_MICA	Q29980,Q29983	MHC class I polypeptide-related sequence A_MHC class I polypeptide-related sequence B	rs4760	44153100	19	G	A	0.156	0.037	0.005	3.1E-13	trans
1394	MIF	P14174	Macrophage migration inhibitory factor	rs1354034	56849749	3	C	T	0.604	0.066	0.008	4.1E-18	trans
1394	MIF	P14174	Macrophage migration inhibitory factor	rs2330634	24250795	22	C	G	0.404	0.110	0.008	2.1E-46	cis
1395	MILR1	Q7Z6M3	Allergin-1	rs2524119	31229404	6	C	T	0.545	0.059	0.007	4.1E-17	trans
1395	MILR1	Q7Z6M3	Allergin-1	rs4055121	126232337	11	C	T	0.866	0.100	0.010	2.1E-22	trans
1395	MILR1	Q7Z6M3	Allergin-1	rs186021206	7069412	17	A	G	0.006	1.250	0.052	5.8E-127	trans
1395	MILR1	Q7Z6M3	Allergin-1	rs704	26694861	17	G	A	0.526	0.059	0.008	6.5E-15	trans
1395	MILR1	Q7Z6M3	Allergin-1	rs138176943	62464621	17	A	G	0.058	0.796	0.016	0.0E+00	cis
1396	MINDY1	Q8N5J2	Ubiquitin carboxyl-terminal hydrolase MINDY-1	rs140386498	150972959	1	A	T	0.988	0.628	0.035	1.6E-73	cis
1396	MINDY1	Q8N5J2	Ubiquitin carboxyl-terminal hydrolase MINDY-1	rs6993770	106581528	8	A	T	0.714	0.064	0.008	4.7E-15	trans
1397	MINK1	Q8N4C8	Misshapen-like kinase 1	rs342298	106373646	7	C	T	0.545	0.059	0.008	4.0E-15	trans
1397	MINK1	Q8N4C8	Misshapen-like kinase 1	rs6993770	106581528	8	A	T	0.713	0.063	0.008	3.2E-14	trans
1397	MINK1	Q8N4C8	Misshapen-like kinase 1	rs200309755	27845774	17	T	C	0.537	0.054	0.008	3.3E-12	trans
1398	MITD1	Q8WV92	MIT domain-containing protein 1	rs13009147	99803998	2	T	G	0.348	0.075	0.008	1.5E-21	cis
1398	MITD1	Q8WV92	MIT domain-containing protein 1	rs1354034	56849749	3	C	T	0.604	0.056	0.008	2.3E-13	trans
1399	MLN	P12872	Promotilin	rs870465	3240199	1	A	G	0.174	0.063	0.009	5.8E-12	trans
1399	MLN	P12872	Promotilin	rs826404	118164722	1	T	C	0.413	0.061	0.007	7.1E-20	trans
1399	MLN	P12872	Promotilin	rs11240492	205461223	1	C	G	0.866	0.100	0.010	2.5E-24	trans
1399	MLN	P12872	Promotilin	rs16862260	222069957	2	G	A	0.792	0.226	0.008	1.3E-158	trans
1399	MLN	P12872	Promotilin	rs147351977	183939658	3	A	G	0.028	0.139	0.020	2.9E-12	trans
1399	MLN	P12872	Promotilin	rs12521989	7331212	5	A	T	0.652	0.066	0.007	1.6E-21	trans
1399	MLN	P12872	Promotilin	rs56092644	95774809	5	T	G	0.244	0.079	0.008	8.3E-25	trans
1399	MLN	P12872	Promotilin	rs67131976	20686878	6	T	C	0.173	0.172	0.009	6.1E-77	trans
1399	MLN	P12872	Promotilin	rs1547671	33777260	6	C	T	0.456	0.414	0.007	0.0E+00	cis
1399	MLN	P12872	Promotilin	rs13217983	41681878	6	T	C	0.138	0.075	0.010	2.5E-13	trans
1399	MLN	P12872	Promotilin	rs12114906	104988784	8	T	C	0.749	0.063	0.008	5.5E-16	trans
1399	MLN	P12872	Promotilin	rs3762055	117090575	9	C	T	0.068	0.175	0.014	4.2E-36	trans
1399	MLN	P12872	Promotilin	rs4309096	47091740	10	C	T	0.646	0.065	0.007	1.1E-20	trans
1399	MLN	P12872	Promotilin	rs5785928	71320654	10	C	CT	0.308	0.058	0.007	9.2E-16	trans
1399	MLN	P12872	Promotilin	rs11213809	111135745	11	A	G	0.344	0.051	0.007	2.6E-13	trans
1399	MLN	P12872	Promotilin	rs2485287	38519281	13	C	A	0.465	0.076	0.007	5.9E-31	trans
1399	MLN	P12872	Promotilin	rs28837202	62295386	15	C	T	0.437	0.061	0.007	3.4E-20	trans
1399	MLN	P12872	Promotilin	rs17681738	9793240	17	T	C	0.329	0.053	0.007	6.5E-14	trans
1399	MLN	P12872	Promotilin	rs633372	49209226	19	G	A	0.465	0.072	0.007	2.7E-27	trans
1399	MLN	P12872	Promotilin	rs2208150	16903467	20	C	T	0.591	0.084	0.007	2.7E-35	trans
1400	MME	P08473	Neprilysin	rs1497406	16505320	1	G	A	0.580	0.076	0.007	9.5E-29	trans
1400	MME	P08473	Neprilysin	rs4687657	52852538	3	T	G	0.260	0.581	0.008	0.0E+00	trans
1400	MME	P08473	Neprilysin	rs79837905	154785591	3	A	G	0.920	0.107	0.013	1.3E-15	cis
1400	MME	P08473	Neprilysin	rs4835265	146821410	4	A	C	0.156	0.118	0.009	2.3E-36	trans
1400	MME	P08473	Neprilysin	rs112875651	126506694	8	G	A	0.609	0.049	0.007	2.7E-12	trans
1400	MME	P08473	Neprilysin	rs151068477	52642516	10	T	G	0.008	0.275	0.040	3.6E-12	trans
1400	MME	P08473	Neprilysin	rs1650146	79688208	10	G	A	0.618	0.049	0.007	2.7E-12	trans
1400	MME	P08473	Neprilysin	rs61007561	103564734	14	AG	A	0.246	0.110	0.008	3.8E-44	trans
1400	MME	P08473	Neprilysin	rs12373325	56087648	18	T	C	0.202	0.096	0.008	1.3E-29	trans
1400	MME	P08473	Neprilysin	rs58659609	3482459	19	A	C	0.185	0.074	0.009	4.1E-17	trans
1400	MME	P08473	Neprilysin	rs189448562	3705115	20	C	G	0.011	0.275	0.033	5.7E-17	trans
1400	MME	P08473	Neprilysin	rs12157484	18455122	22	C	T	0.757	0.056	0.008	1.3E-12	trans
1401	MMP1	P03956	Interstitial collagenase	rs12136856	156473114	1	G	C	0.653	0.092	0.007	2.7E-37	trans
1401	MMP1	P03956	Interstitial collagenase	rs12622659	68646279	2	G	A	0.699	0.059	0.007	2.1E-15	trans
1401	MMP1	P03956	Interstitial collagenase	rs76036957	113496453	2	A	G	0.009	0.316	0.036	5.6E-19	trans
1401	MMP1	P03956	Interstitial collagenase	rs28706788	159575028	5	G	A	0.458	0.055	0.007	1.3E-15	trans
1401	MMP1	P03956	Interstitial collagenase	rs5879021	109635558	6	GC	G	0.520	0.071	0.007	7.0E-25	trans
1401	MMP1	P03956	Interstitial collagenase	rs6993770	106581528	8	A	T	0.713	0.157	0.008	9.0E-96	trans
1401	MMP1	P03956	Interstitial collagenase	rs10820606	99192919	9	C	A	0.229	0.080	0.008	3.2E-22	trans
1401	MMP1	P03956	Interstitial collagenase	rs534191	102671697	11	T	C	0.454	0.448	0.008	0.0E+00	cis
1401	MMP1	P03956	Interstitial collagenase	rs12445050	81870969	16	T	C	0.138	0.073	0.010	1.2E-13	trans
1401	MMP1	P03956	Interstitial collagenase	rs1654425	55538980	19	C	T	0.834	0.120	0.009	1.1E-38	trans
1401	MMP1	P03956	Interstitial collagenase	rs6081569	19303786	20	C	G	0.369	0.058	0.007	1.9E-16	trans
1402	MMP10	P09238	Stromelysin-2	rs187265184	32525769	6	G	C	0.356	0.064	0.009	2.0E-13	trans
1402	MMP10	P09238	Stromelysin-2	rs486055	102650424	11	C	T	0.838	0.396	0.010	0.0E+00	cis
1402	MMP10	P09238	Stromelysin-2	rs681343	49206462	19	T	C	0.510	0.137	0.007	1.3E-81	trans
1403	MMP12	P39900	Macrophage metalloelastase	rs2770	31321807	6	G	A	0.553	0.055	0.007	8.9E-14	trans
1403	MMP12	P39900	Macrophage metalloelastase	rs17368814	102748695	11	A	G	0.875	0.749	0.011	0.0E+00	cis
1403	MMP12	P39900	Macrophage metalloelastase	rs12986064	54755133	19	T	C	0.492	0.080	0.007	1.9E-33	trans
1404	MMP15	P51511	Matrix metalloproteinase-15	rs187793617	106360691	14	G	C	0.160	0.123	0.011	1.2E-29	trans
1405	MMP3	P08254	Stromelysin-1	rs41341748	16012594	8	A	G	0.011	0.245	0.025	3.4E-22	trans
1405	MMP3	P08254	Stromelysin-1	rs678815	102713777	11	C	G	0.483	0.479	0.006	0.0E+00	cis
1405	MMP3	P08254	Stromelysin-1	rs2267372	38598234	22	G	A	0.600	0.050	0.005	8.7E-21	trans
1406	MMP7	P09237	Matrilysin	rs6694034	202003987	1	C	A	0.449	0.109	0.007	2.4E-51	trans
1406	MMP7	P09237	Matrilysin	rs11568819	102401633	11	A	G	0.061	0.567	0.015	4.8E-302	cis
1406	MMP7	P09237	Matrilysin	rs77924615	20392332	16	G	A	0.803	0.092	0.009	1.6E-24	trans
1406	MMP7	P09237	Matrilysin	rs12975366	54759361	19	T	C	0.605	0.063	0.007	4.5E-18	trans
1407	MMP8	P22894	Neutrophil collagenase	rs2511241	72945341	11	T	C	0.929	0.189	0.015	1.7E-38	trans
1407	MMP8	P22894	Neutrophil collagenase	rs35231465	102584135	11	G	A	0.975	1.252	0.024	0.0E+00	cis
1407	MMP8	P22894	Neutrophil collagenase	rs7208815	38168715	17	T	C	0.393	0.087	0.007	1.3E-33	trans
1408	MMP9	P14780	Matrix metalloproteinase-9	rs1886654	236105910	1	T	C	0.106	0.081	0.012	1.3E-11	trans
1408	MMP9	P14780	Matrix metalloproteinase-9	rs28498283	43360065	2	T	A	0.255	0.059	0.009	6.7E-12	trans
1408	MMP9	P14780	Matrix metalloproteinase-9	rs4619875	153701130	4	C	T	0.597	0.059	0.007	3.9E-15	trans
1408	MMP9	P14780	Matrix metalloproteinase-9	rs9267111	31416379	6	G	A	0.348	0.070	0.008	1.5E-19	trans
1408	MMP9	P14780	Matrix metalloproteinase-9	rs2158799	28277107	7	G	C	0.609	0.054	0.008	1.5E-12	trans
1408	MMP9	P14780	Matrix metalloproteinase-9	rs2511241	72945341	11	T	C	0.929	0.384	0.014	5.2E-155	trans
1408	MMP9	P14780	Matrix metalloproteinase-9	rs2289511	88454910	14	G	A	0.419	0.059	0.007	3.8E-15	trans
1408	MMP9	P14780	Matrix metalloproteinase-9	rs17576	44640225	20	G	A	0.351	0.145	0.008	5.2E-79	cis
1408	MMP9	P14780	Matrix metalloproteinase-9	rs184339396	49043608	X	C	G	0.033	0.207	0.018	3.9E-32	trans
1409	MOCS2	O96007	Molybdopterin synthase catalytic subunit	rs1354034	56849749	3	C	T	0.604	0.084	0.007	1.8E-32	trans
1409	MOCS2	O96007	Molybdopterin synthase catalytic subunit	rs6880055	52389428	5	G	A	0.342	0.449	0.008	0.0E+00	cis
1409	MOCS2	O96007	Molybdopterin synthase catalytic subunit	rs6967348	129247134	7	G	A	0.088	0.092	0.012	6.3E-14	trans
1410	MOG	Q16653	Myelin-oligodendrocyte glycoprotein	rs9461541	29625195	6	A	G	0.951	0.451	0.017	1.7E-160	cis
1410	MOG	Q16653	Myelin-oligodendrocyte glycoprotein	rs8021280	75290018	14	G	C	0.463	0.054	0.007	1.4E-14	trans
1411	MORF4L2	Q15014	Mortality factor 4-like protein 2	rs71534539	32512754	6	G	A	0.536	0.076	0.009	7.4E-19	trans
1412	MPHOSPH8	Q99549	M-phase phosphoprotein 8	rs1354034	56849749	3	C	T	0.604	0.068	0.008	2.4E-18	trans
1412	MPHOSPH8	Q99549	M-phase phosphoprotein 8	rs35424555	20221516	13	G	A	0.122	0.088	0.012	5.0E-14	cis
1413	MPI	P34949	Mannose-6-phosphate isomerase	rs1354034	56849749	3	C	T	0.605	0.068	0.008	4.2E-19	trans
1413	MPI	P34949	Mannose-6-phosphate isomerase	rs7080386	65048306	10	A	C	0.412	0.060	0.008	2.3E-15	trans
1413	MPI	P34949	Mannose-6-phosphate isomerase	rs4886632	75173925	15	T	G	0.576	0.200	0.008	5.3E-152	cis
1414	MPIG6B	O95866	Megakaryocyte and platelet inhibitory receptor G6b	rs6961069	80218961	7	T	C	0.402	0.056	0.008	4.3E-13	trans
1414	MPIG6B	O95866	Megakaryocyte and platelet inhibitory receptor G6b	rs6993770	106581528	8	A	T	0.713	0.101	0.008	1.3E-33	trans
1414	MPIG6B	O95866	Megakaryocyte and platelet inhibitory receptor G6b	rs7896518	65104500	10	G	A	0.424	0.077	0.008	2.9E-23	trans
1414	MPIG6B	O95866	Megakaryocyte and platelet inhibitory receptor G6b	rs892090	55539072	19	G	T	0.834	0.102	0.010	9.2E-24	trans
1415	MPO	P05164	Myeloperoxidase	rs2120782	229670389	1	C	G	0.205	0.086	0.009	2.3E-21	trans
1415	MPO	P05164	Myeloperoxidase	rs13277237	130604563	8	G	A	0.432	0.075	0.007	7.6E-24	trans
1415	MPO	P05164	Myeloperoxidase	rs757081	17351683	11	G	C	0.332	0.055	0.008	1.9E-12	trans
1415	MPO	P05164	Myeloperoxidase	rs12941811	38159335	17	T	C	0.426	0.056	0.008	1.8E-13	trans
1415	MPO	P05164	Myeloperoxidase	rs34097845	56358429	17	C	T	0.943	0.507	0.017	6.7E-205	cis
1415	MPO	P05164	Myeloperoxidase	rs6034875	17627727	20	G	A	0.420	0.068	0.007	3.5E-20	trans
1416	MPRIP	Q6WCQ1	Myosin phosphatase Rho-interacting protein	rs3910516	216303053	2	G	A	0.736	0.071	0.009	1.5E-16	trans
1417	MRC1	P22897	Macrophage mannose receptor 1	rs11125916	62609378	2	A	C	0.195	0.062	0.009	1.9E-12	trans
1417	MRC1	P22897	Macrophage mannose receptor 1	rs2579651	120227185	2	G	A	0.872	0.074	0.011	3.1E-12	trans
1417	MRC1	P22897	Macrophage mannose receptor 1	rs1257169	134963862	2	C	A	0.501	0.059	0.007	3.4E-17	trans
1417	MRC1	P22897	Macrophage mannose receptor 1	rs35771982	160885418	2	C	G	0.508	0.133	0.007	6.4E-81	trans
1417	MRC1	P22897	Macrophage mannose receptor 1	rs12515263	74349500	5	A	G	0.043	0.230	0.017	1.1E-40	trans
1417	MRC1	P22897	Macrophage mannose receptor 1	rs35139284	32561370	6	T	C	0.321	0.083	0.008	3.0E-28	trans
1417	MRC1	P22897	Macrophage mannose receptor 1	rs12667978	128777461	7	T	C	0.571	0.052	0.007	2.1E-13	trans
1417	MRC1	P22897	Macrophage mannose receptor 1	rs4876611	116671848	8	G	A	0.718	0.069	0.008	1.2E-18	trans
1417	MRC1	P22897	Macrophage mannose receptor 1	rs5030924	71210908	10	A	T	0.555	0.072	0.007	2.4E-23	trans
1417	MRC1	P22897	Macrophage mannose receptor 1	rs174564	61588305	11	G	A	0.349	0.077	0.007	5.6E-26	trans
1417	MRC1	P22897	Macrophage mannose receptor 1	rs186021206	7069412	17	A	G	0.006	0.582	0.048	8.2E-34	trans
1417	MRC1	P22897	Macrophage mannose receptor 1	rs7246479	55824332	19	G	T	0.510	0.054	0.007	7.0E-15	trans
1417	MRC1	P22897	Macrophage mannose receptor 1	rs4611747	29757090	22	T	C	0.289	0.072	0.008	1.7E-20	trans

1418	MRI1	Q9BV20	Methylthioribose-1-phosphate isomerase	rs1354034	56849749	3	C	T	0.604	0.055	0.007	5.0E-14	trans
1418	MRI1	Q9BV20	Methylthioribose-1-phosphate isomerase	rs452960	13871411	19	A	T	0.578	0.347	0.008	0.0E+00	cis
1419	MRPL58	Q14197	Peptidyl-tRNA hydrolase ICT1, mitochondrial	rs1354034	56849749	3	C	T	0.604	0.057	0.008	1.6E-13	trans
1419	MRPL58	Q14197	Peptidyl-tRNA hydrolase ICT1, mitochondrial	rs342293	106372219	7	C	G	0.540	0.058	0.008	2.7E-14	trans
1419	MRPL58	Q14197	Peptidyl-tRNA hydrolase ICT1, mitochondrial	rs6993770	106581528	8	A	T	0.714	0.066	0.008	3.0E-15	trans
1419	MRPL58	Q14197	Peptidyl-tRNA hydrolase ICT1, mitochondrial	rs11085147	5711930	19	T	C	0.099	0.174	0.013	2.5E-40	trans
1420	MSLN	Q13421	Mesothelin	rs72851462	25723038	2	G	C	0.885	0.101	0.011	4.2E-21	trans
1420	MSLN	Q13421	Mesothelin	rs2517403	31067009	6	T	C	0.668	0.068	0.007	6.6E-21	trans
1420	MSLN	Q13421	Mesothelin	rs1214759	43352980	6	A	G	0.319	0.093	0.007	7.0E-38	trans
1420	MSLN	Q13421	Mesothelin	rs11788059	109480268	9	C	T	0.339	0.071	0.007	4.2E-23	trans
1420	MSLN	Q13421	Mesothelin	rs2235503	810593	16	A	C	0.193	0.754	0.009	0.0E+00	cis
1420	MSLN	Q13421	Mesothelin	rs4807630	1170445	19	T	C	0.309	0.056	0.007	3.8E-14	trans
1420	MSLN	Q13421	Mesothelin	rs34450188	9531446	19	G	A	0.557	0.081	0.007	7.0E-32	trans
1420	MSLN	Q13421	Mesothelin	rs492602	49206417	19	G	A	0.509	0.068	0.007	6.0E-23	trans
1421	MSMB	P08118	Beta-microseminoprotein	rs4687657	52852538	3	T	G	0.259	0.089	0.006	9.5E-53	trans
1421	MSMB	P08118	Beta-microseminoprotein	rs62406524	36925976	6	G	C	0.904	0.110	0.009	4.8E-34	trans
1421	MSMB	P08118	Beta-microseminoprotein	rs73671501	23465965	8	T	C	0.170	0.079	0.007	6.6E-32	trans
1421	MSMB	P08118	Beta-microseminoprotein	rs10993994	51549496	10	C	T	0.608	0.936	0.007	0.0E+00	cis
1421	MSMB	P08118	Beta-microseminoprotein	rs36120341	46224971	19	C	T	0.636	0.049	0.005	2.5E-20	trans
1422	MSR1	P21757	Macrophage scavenger receptor types I and II	rs41271951	150737220	1	A	G	0.915	0.101	0.012	4.1E-17	trans
1422	MSR1	P21757	Macrophage scavenger receptor types I and II	rs2293330	143103842	3	G	T	0.827	0.068	0.009	3.0E-14	trans
1422	MSR1	P21757	Macrophage scavenger receptor types I and II	rs1471401	88774340	4	G	A	0.661	0.050	0.007	1.3E-12	trans
1422	MSR1	P21757	Macrophage scavenger receptor types I and II	rs115216147	70665526	5	A	T	0.067	0.094	0.014	7.9E-12	trans
1422	MSR1	P21757	Macrophage scavenger receptor types I and II	rs509227	10535591	6	C	A	0.523	0.054	0.007	1.4E-15	trans
1422	MSR1	P21757	Macrophage scavenger receptor types I and II	rs139299944	32602665	6	C	CT	0.656	0.097	0.007	5.3E-43	trans
1422	MSR1	P21757	Macrophage scavenger receptor types I and II	rs10863	130145589	7	G	A	0.853	0.071	0.009	6.5E-14	trans
1422	MSR1	P21757	Macrophage scavenger receptor types I and II	rs41341748	16012594	8	G	A	0.989	1.547	0.033	0.0E+00	cis
1422	MSR1	P21757	Macrophage scavenger receptor types I and II	rs2031902	33117524	9	C	T	0.413	0.058	0.007	3.2E-17	trans
1422	MSR1	P21757	Macrophage scavenger receptor types I and II	rs635634	136155000	9	T	C	0.184	0.075	0.009	6.9E-18	trans
1422	MSR1	P21757	Macrophage scavenger receptor types I and II	rs11220505	126334805	11	T	C	0.223	0.143	0.008	2.0E-69	trans
1422	MSR1	P21757	Macrophage scavenger receptor types I and II	rs186021206	7069412	17	A	G	0.006	1.383	0.046	1.0E-198	trans
1423	MSRA	Q9UJ68	Mitochondrial peptide methionine sulfoxide reductase	rs1354034	56849749	3	C	T	0.604	0.060	0.008	4.0E-15	trans
1423	MSRA	Q9UJ68	Mitochondrial peptide methionine sulfoxide reductase	rs12546887	10292876	8	C	T	0.410	0.057	0.008	6.4E-14	cis
1424	MST1	P26927	Hepatocyte growth factor-like protein	rs9435340	107593201	1	T	A	0.345	0.049	0.005	2.1E-25	trans
1424	MST1	P26927	Hepatocyte growth factor-like protein	rs1260326	27730940	2	T	C	0.392	0.068	0.005	4.4E-51	trans
1424	MST1	P26927	Hepatocyte growth factor-like protein	rs11130213	49712297	3	C	T	0.709	1.051	0.008	0.0E+00	cis
1424	MST1	P26927	Hepatocyte growth factor-like protein	rs1799895	24801834	4	C	G	0.988	0.146	0.021	1.5E-12	trans
1424	MST1	P26927	Hepatocyte growth factor-like protein	rs8111	32083175	6	T	C	0.254	0.056	0.005	1.3E-28	trans
1424	MST1	P26927	Hepatocyte growth factor-like protein	rs28929474	94844947	14	T	C	0.021	0.175	0.015	1.0E-29	trans
1424	MST1	P26927	Hepatocyte growth factor-like protein	rs149394327	64228995	17	C	G	0.030	0.180	0.013	8.0E-44	trans
1425	MSTN	O14793	Growth/differentiation factor 8	rs143242500	190924861	2	G	A	0.002	0.786	0.087	1.2E-19	cis
1425	MSTN	O14793	Growth/differentiation factor 8	rs34950020	133800545	6	G	A	0.681	0.079	0.007	3.9E-26	trans
1425	MSTN	O14793	Growth/differentiation factor 8	rs1815739	66328095	11	C	T	0.557	0.061	0.007	2.1E-18	trans
1425	MSTN	O14793	Growth/differentiation factor 8	rs8096658	77156537	18	G	C	0.489	0.054	0.007	1.6E-14	trans
1426	MTDH	Q86UE4	Protein LYRIC	rs1354034	56849749	3	C	T	0.604	0.147	0.008	8.1E-80	trans
1426	MTDH	Q86UE4	Protein LYRIC	rs342293	106372219	7	C	G	0.541	0.056	0.008	1.6E-13	trans
1426	MTDH	Q86UE4	Protein LYRIC	rs6993770	106581528	8	A	T	0.713	0.069	0.008	3.2E-16	trans
1426	MTDH	Q86UE4	Protein LYRIC	rs11502185	180258	11	C	T	0.260	0.088	0.010	2.5E-19	trans
1427	MTHFSD	Q2M296	Methenyltetrahydrofolate synthase domain-containing protein	rs1354034	56849749	3	C	T	0.604	0.060	0.007	7.3E-17	trans
1427	MTHFSD	Q2M296	Methenyltetrahydrofolate synthase domain-containing protein	rs10900809	131826322	5	G	A	0.611	0.050	0.007	1.3E-11	trans
1427	MTHFSD	Q2M296	Methenyltetrahydrofolate synthase domain-containing protein	rs9939348	86560860	16	C	T	0.598	0.464	0.008	0.0E+00	cis
1428	MTIF3	Q9H2K0	Translation initiation factor IF-3, mitochondrial	rs10075801	131677642	5	A	G	0.512	0.058	0.008	3.7E-14	trans
1428	MTIF3	Q9H2K0	Translation initiation factor IF-3, mitochondrial	rs6993770	106581528	8	A	T	0.713	0.064	0.008	1.4E-14	trans
1428	MTIF3	Q9H2K0	Translation initiation factor IF-3, mitochondrial	rs1218825	28009920	13	C	G	0.956	0.218	0.019	7.2E-32	cis
1429	MTSS2	Q765P7	Protein MTSS 2	rs1354034	56849749	3	C	T	0.604	0.057	0.008	5.7E-14	trans
1429	MTSS2	Q765P7	Protein MTSS 2	rs6993770	106581528	8	T	A	0.286	0.064	0.008	1.2E-14	trans
1429	MTSS2	Q765P7	Protein MTSS 2	rs113315674	100720053	9	A	G	0.365	0.076	0.008	8.1E-23	trans
1430	MTUS1	Q9ULD2	Microtubule-associated tumor suppressor 1	rs142323734	17658751	8	A	G	0.041	0.432	0.019	3.5E-111	cis
1431	MUC13	Q9H3R2	Mucin-13	rs1127233	124627024	3	G	T	0.262	0.244	0.009	9.1E-178	cis
1431	MUC13	Q9H3R2	Mucin-13	rs4556017	100632790	7	C	T	0.147	0.104	0.011	9.2E-23	trans
1431	MUC13	Q9H3R2	Mucin-13	rs2519093	136141870	9	C	T	0.816	0.252	0.010	1.3E-147	trans
1432	MUC16	Q8WXI7	Mucin-16	rs62193070	242730588	2	A	G	0.837	0.259	0.010	1.4E-135	trans
1432	MUC16	Q8WXI7	Mucin-16	rs7205754	815413	16	C	A	0.961	0.201	0.020	1.0E-24	trans
1432	MUC16	Q8WXI7	Mucin-16	rs10421318	9120919	19	G	A	0.649	0.072	0.008	1.4E-18	cis
1433	MUC2	Q02817	Mucin-2	rs1933630	230914706	1	A	C	0.157	0.070	0.008	1.0E-16	trans
1433	MUC2	Q02817	Mucin-2	rs12416873	1081577	11	G	A	0.792	0.265	0.008	1.2E-259	cis
1433	MUC2	Q02817	Mucin-2	rs10899345	76747519	11	A	G	0.663	0.082	0.007	4.1E-35	trans
1433	MUC2	Q02817	Mucin-2	rs11215025	114375189	11	C	T	0.843	0.065	0.009	2.7E-14	trans
1433	MUC2	Q02817	Mucin-2	rs2608894	5848000	19	T	C	0.235	0.153	0.008	1.1E-76	trans
1433	MUC2	Q02817	Mucin-2	rs681343	49206462	19	T	C	0.509	0.573	0.007	0.0E+00	trans
1434	MXRA8	Q9BRK3	Matrix remodeling-associated protein 8	rs1260326	27730940	2	C	T	0.608	0.055	0.007	3.3E-14	trans
1434	MXRA8	Q9BRK3	Matrix remodeling-associated protein 8	rs13167730	79370247	5	T	G	0.095	0.093	0.012	6.9E-15	trans
1434	MXRA8	Q9BRK3	Matrix remodeling-associated protein 8	rs331	19820405	8	A	G	0.264	0.056	0.008	2.1E-12	trans
1434	MXRA8	Q9BRK3	Matrix remodeling-associated protein 8	rs56278466	17875857	10	G	T	0.661	0.077	0.007	2.6E-25	trans
1434	MXRA8	Q9BRK3	Matrix remodeling-associated protein 8	rs200839648	116689154	11	T	A	0.132	0.113	0.011	2.6E-23	trans
1434	MXRA8	Q9BRK3	Matrix remodeling-associated protein 8	rs261290	58678720	15	T	C	0.346	0.103	0.007	8.8E-43	trans
1434	MXRA8	Q9BRK3	Matrix remodeling-associated protein 8	rs247617	56990716	16	A	C	0.324	0.099	0.008	4.3E-39	trans
1434	MXRA8	Q9BRK3	Matrix remodeling-associated protein 8	rs58761784	8492259	19	T	C	0.715	0.058	0.008	2.1E-13	trans
1434	MXRA8	Q9BRK3	Matrix remodeling-associated protein 8	rs1065853	45413233	19	T	G	0.081	0.160	0.013	9.0E-35	trans
1435	MYBPC1	Q00872	Myosin-binding protein C, slow-type	rs6431221	127884594	2	A	C	0.515	0.050	0.007	5.1E-13	trans
1435	MYBPC1	Q00872	Myosin-binding protein C, slow-type	rs12463674	179432185	2	G	A	0.316	0.075	0.008	3.8E-23	trans
1435	MYBPC1	Q00872	Myosin-binding protein C, slow-type	rs2234962	121429633	10	C	T	0.216	0.065	0.008	2.1E-14	trans
1435	MYBPC1	Q00872	Myosin-binding protein C, slow-type	rs1034048	74126852	13	G	A	0.559	0.065	0.007	4.7E-20	trans
1435	MYBPC1	Q00872	Myosin-binding protein C, slow-type	rs1801690	64208285	17	G	C	0.056	0.131	0.015	4.8E-18	trans
1436	MYCBP2	O75592	E3 ubiquitin-protein ligase MYCBP2	rs1354034	56849749	3	C	T	0.604	0.063	0.008	8.0E-17	trans
1437	MYDGF	Q969H8	Myeloid-derived growth factor	rs1354034	56849749	3	C	T	0.604	0.063	0.008	2.4E-16	trans
1437	MYDGF	Q969H8	Myeloid-derived growth factor	rs6993770	106581528	8	A	T	0.713	0.074	0.008	7.2E-19	trans
1437	MYDGF	Q969H8	Myeloid-derived growth factor	rs60893137	4656839	19	A	C	0.979	0.307	0.026	5.3E-32	cis
1438	MYH9	P35579	Myosin-9	rs1354034	56849749	3	C	T	0.605	0.075	0.008	7.1E-23	trans
1438	MYH9	P35579	Myosin-9	rs10075801	131677642	5	A	G	0.512	0.058	0.008	2.7E-14	trans
1438	MYH9	P35579	Myosin-9	rs342293	106372219	7	C	G	0.540	0.056	0.008	1.2E-13	trans
1438	MYH9	P35579	Myosin-9	rs6993770	106581528	8	A	T	0.714	0.075	0.008	1.0E-19	trans
1438	MYH9	P35579	Myosin-9	rs7210399	27780216	17	T	C	0.485	0.058	0.008	1.7E-14	trans
1439	MYL3	P08590	Myosin light chain 3	rs6431221	127884594	2	A	C	0.515	0.063	0.007	2.7E-19	trans
1439	MYL3	P08590	Myosin light chain 3	rs12463674	179432185	2	G	A	0.315	0.084	0.008	1.8E-27	trans
1439	MYL3	P08590	Myosin light chain 3	rs200564310	117506042	6	T	G	0.843	0.071	0.010	3.6E-13	trans
1439	MYL3	P08590	Myosin light chain 3	rs73015965	161127501	6	G	A	0.006	0.340	0.048	8.7E-13	trans
1439	MYL3	P08590	Myosin light chain 3	rs2234962	121429633	10	C	T	0.216	0.062	0.009	4.7E-13	trans
1439	MYL3	P08590	Myosin light chain 3	rs1034048	74126852	13	G	A	0.559	0.081	0.007	1.8E-29	trans
1439	MYL3	P08590	Myosin light chain 3	rs76452927	64241706	17	A	G	0.054	0.121	0.016	1.6E-14	trans
1440	MYL4	P12829	Myosin light chain 4	rs111803166	30126225	6	G	A	0.009	0.331	0.039	2.0E-17	trans
1441	MYL6B	P14649	Myosin light chain 6B	rs71534539	32512754	6	G	A	0.535	0.075	0.009	9.2E-19	trans
1442	MYLPF	Q96A32	Myosin regulatory light chain 2, skeletal muscle isoform	rs10833712	22201007	11	G	T	0.579	0.054	0.008	6.5E-13	trans
1443	MYOC	Q99972	Myocilin	rs171003	171616521	1	A	G	0.580	0.267	0.007	3.5E-292	cis
1443	MYOC	Q99972	Myocilin	rs6888037	127406259	5	T	G	0.751	0.084	0.008	2.0E-25	trans
1443	MYOC	Q99972	Myocilin	rs56278466	17875857	10	G	T	0.661	0.102	0.007	7.6E-44	trans
1443	MYOC	Q99972	Myocilin	rs11632195	65493488	15	C	T	0.361	0.087	0.007	5.7E-33	trans
1443	MYOC	Q99972	Myocilin	rs186021206	7069412	17	A	G	0.006	0.511	0.048	7.1E-27	trans
1443	MYOC	Q99972	Myocilin	rs61739916	18897440	19	C	T	0.055	0.140	0.015	4.9E-20	trans
1444	MYOM2	P54296	Myomesin-2	rs117165873	2054077	8	G	A	0.005	1.071	0.056	1.8E-80	cis
1444	MYOM2	P54296	Myomesin-2	rs10833711	22200763	11	A	G	0.528	0.065	0.008	2.2E-17	trans
1445	MYOM3	Q5VTT5	Myomesin-3	rs6700245	24417415	1	T	C	0.853	0.107	0.010	1.4E-26	cis
1445	MYOM3	Q5VTT5	Myomesin-3	rs6431221	127884594	2	A	C	0.515	0.064	0.007	1.2E-19	trans
1445	MYOM3	Q5VTT5	Myomesin-3	rs12464787	179440029	2	A	G	0.311	0.082	0.008	3.9E-26	trans
1445	MYOM3	Q5VTT5	Myomesin-3	rs663494	117629650	6	T	A	0.667	0.057	0.008	2.1E-12	trans
1445	MYOM3	Q5VTT5	Myomesin-3	rs72842207	121433675	10	T	C	0.215	0.078	0.009	1.3E-19	trans
1445	MYOM3	Q5VTT5	Myomesin-3	rs1799938	65052304	17	A	G	0.115	0.099	0.011	3.2E-19	trans
1446	MZB1	Q8WU39	Marginal zone B- and B1-cell-specific protein	rs149547076	57557444	3	T	C	0.989	0.547	0.037	1.4E-50	trans
1446	MZB1	Q8WU39	Marginal zone B- and B1-cell-specific protein	rs11739135	131733397	5	C	G	0.415	0.051	0.008	7.7E-12	trans
1446	MZB1	Q8WU39	Marginal zone B- and B1-cell-specific protein	rs6796	6502367	7	C	T	0.279	0.056	0.008	1.6E-11	trans
1446	MZB1	Q8WU39	Marginal zone B- and B1-cell-specific protein	rs61839660	6094697	10	T	C	0.097	0.099	0.012	1.4E-15	trans
1446	MZB1	Q8WU39	Marginal zone B- and B1-cell-specific protein	rs3803286	103246470	14	A	G	0.334	0.062	0.008	2.1E-15	trans
1446	MZB1	Q8WU39	Marginal zone B- and B1-cell-specific protein	rs10150206	106206700	14	A	G	0.658	0.157	0.008	2.3E-86	trans
1446	MZB1	Q8WU39	Marginal zone B- and B1-cell-specific protein	rs3803800	7462969	17	A	G	0.211	0.092	0.009	1.1E-24	trans
1446	MZB1	Q8WU39	Marginal zone B- and B1-cell-specific protein	rs34562254	16842991	17	A	G	0.097	0.156	0.012	2.8E-36	trans
1446	MZB1	Q8WU39	Marginal zone B- and B1-cell-specific protein	rs9624334	24166256	22	G	C	0.849	0.085	0.010	1.8E-16	trans
1447	MZT1	Q08AG7	Mitotic-spindle organizing protein 1	rs1354034	56849749	3	C	T	0.604	0.058	0.008	9.3E-14	trans
1448	NAA10	P41227	N-alpha-acetyltransferase 10	rs1354034	56849749	3	C	T	0.604	0.055	0.008	3.0E-12	trans
1449	NAA80	Q93015	N-alpha-acetyltransferase 80	rs201512291	50334462	3	G	A	0.994	0.719	0.047	4.6E-52	cis
1449	NAA80	Q93015	N-alpha-acetyltransferase 80	rs1354034	56849749	3	C	T	0.604	0.066	0.008	7.9E-18	trans
1449	NAA80	Q93015	N-alpha-acetyltransferase 80	rs6993770	106581528	8	A	T	0.713	0.070	0.008	4.0E-17	trans
1449	NAA80	Q93015	N-alpha-acetyltransferase 80	rs7896518	65104500	10	G	A	0.424	0.066	0.008	2.3E-17	trans
1449	NAA80	Q93015	N-alpha-acetyltransferase 80	rs892090	55539072	19	G	T	0.834	0.071	0.010	2.9E-12	trans
1450	NAAA	Q02083	N-acylethanolamine-hydrolyzing acid amidase	rs5014539	172485753	1	T	G	0.573	0.048	0.006	7.0E-14	trans
1450	NAAA	Q02083	N-acylethanolamine-hydrolyzing acid amidase	rs687339	135932359	3	T	C	0.771	0.057	0.008	1.3E-13	trans
1450	NAAA	Q02083	N-acylethanolamine-hydrolyzing acid amidase	rs112197434	76854118	4	A	T	0.766	0.669	0.009	0.0E+00	cis
1450	NAAA	Q02083	N-acylethanolamine-hydrolyzing acid amidase	rs77945361	154254534	4	G	A	0.852	0.086	0.010	2.7E-16	trans
1450	NAAA	Q02083	N-acylethanolamine-hydrolyzing acid amidase	rs4471106	5629133	9	G	A	0.713	0.050	0.007	4.2E-12	trans
1450	NAAA	Q02083	N-acylethanolamine-hydrolyzing acid amidase	rs4878583	34634422	9	T	C	0.173	0.072	0.009	3.5E-17	trans
1450	NAAA	Q02083	N-acylethanolamine-hydrolyzing acid amidase	rs7904738	74710484	10	G	C	0.912	0.087	0.011	1.5E-14	trans
1450	NAAA	Q02083	N-acylethanolamine-hydrolyzing acid amidase	rs2862954	101912064	10	T	C	0.498	0.050	0.006	4.4E-15	trans
1450	NAAA	Q02083	N-acylethanolamine-hydrolyzing acid amidase	rs7096937	113950418	10	T	C	0.270	0.063	0.007	5.4E-18	trans
1450	NAAA	Q02083	N-acylethanolamine-hydrolyzing acid amidase	rs150641790	41687844	13	TG	T	0.075	0.105	0.012	2.2E-17	trans

1450	NAAA	Q02083	N-acylethanolamine-hydrolyzing acid amidase	rs145078947	93652974	14	T	G	0.003	0.807	0.063	1.5E-37	trans
1450	NAAA	Q02083	N-acylethanolamine-hydrolyzing acid amidase	rs200210321	19393890	19	AG	A	0.074	0.125	0.012	6.2E-24	trans
1450	NAAA	Q02083	N-acylethanolamine-hydrolyzing acid amidase	rs429358	45411941	19	T	C	0.845	0.118	0.009	1.4E-39	trans
1450	NAAA	Q02083	N-acylethanolamine-hydrolyzing acid amidase	rs626283	54677001	19	C	G	0.442	0.061	0.007	6.9E-21	trans
1450	NAAA	Q02083	N-acylethanolamine-hydrolyzing acid amidase	rs738408	44324730	22	T	C	0.217	0.056	0.008	8.1E-13	trans
1451	NADK	O95544	NAD kinase	rs1014988	1680219	1	A	G	0.510	0.252	0.007	5.0E-255	cis
1451	NADK	O95544	NAD kinase	rs71309976	56728081	3	T	C	0.011	0.293	0.037	4.0E-15	trans
1451	NADK	O95544	NAD kinase	rs389883	31947460	6	T	G	0.676	0.057	0.008	8.1E-14	trans
1451	NADK	O95544	NAD kinase	rs56077246	144983493	8	C	T	0.428	0.083	0.007	4.0E-30	trans
1452	NAGA	P17050	Alpha-N-acetylgalactosaminidase	rs684773	135956305	3	C	A	0.767	0.064	0.009	1.1E-12	trans
1452	NAGA	P17050	Alpha-N-acetylgalactosaminidase	rs28672722	32626537	6	T	G	0.222	0.092	0.009	5.8E-24	trans
1453	NAGK	Q9UJ70	N-acetyl-D-glucosamine kinase	rs11680831	71292522	2	T	C	0.658	0.223	0.008	1.1E-173	cis
1453	NAGK	Q9UJ70	N-acetyl-D-glucosamine kinase	rs60629714	95889160	9	T	C	0.800	0.066	0.009	1.4E-12	trans
1454	NAGPA	Q9UK23	N-acetylglucosamine-1-phosphodiester alpha-N-acetylglucosaminidase	rs1260326	27730940	2	T	C	0.392	0.060	0.007	1.0E-15	trans
1454	NAGPA	Q9UK23	N-acetylglucosamine-1-phosphodiester alpha-N-acetylglucosaminidase	rs535777	32577633	6	G	C	0.857	0.092	0.011	3.1E-18	trans
1454	NAGPA	Q9UK23	N-acetylglucosamine-1-phosphodiester alpha-N-acetylglucosaminidase	rs28601761	126500031	8	C	G	0.581	0.053	0.007	8.2E-13	trans
1454	NAGPA	Q9UK23	N-acetylglucosamine-1-phosphodiester alpha-N-acetylglucosaminidase	rs507666	136149399	9	G	A	0.815	0.120	0.009	8.6E-37	trans
1454	NAGPA	Q9UK23	N-acetylglucosamine-1-phosphodiester alpha-N-acetylglucosaminidase	rs12599777	5079466	16	A	G	0.817	0.410	0.010	0.0E+00	cis
1454	NAGPA	Q9UK23	N-acetylglucosamine-1-phosphodiester alpha-N-acetylglucosaminidase	rs186021206	7069412	17	A	G	0.006	0.632	0.050	1.6E-36	trans
1454	NAGPA	Q9UK23	N-acetylglucosamine-1-phosphodiester alpha-N-acetylglucosaminidase	rs1801689	64210580	17	C	A	0.030	0.180	0.021	3.2E-17	trans
1455	NAP1L4	Q99733	Nucleosome assembly protein 1-like 4	rs3811444	248039451	1	C	T	0.667	0.053	0.008	5.7E-12	trans
1455	NAP1L4	Q99733	Nucleosome assembly protein 1-like 4	rs1354034	56849749	3	C	T	0.605	0.054	0.007	4.9E-13	trans
1455	NAP1L4	Q99733	Nucleosome assembly protein 1-like 4	rs1018846	30137649	6	A	G	0.064	0.104	0.015	2.4E-12	trans
1455	NAP1L4	Q99733	Nucleosome assembly protein 1-like 4	rs6993770	106581528	8	A	T	0.714	0.066	0.008	1.8E-16	trans
1455	NAP1L4	Q99733	Nucleosome assembly protein 1-like 4	rs57530484	2987643	11	TAACAGACCTCTC	T	0.767	0.263	0.009	7.9E-201	cis
1455	NAP1L4	Q99733	Nucleosome assembly protein 1-like 4	rs12449930	74419333	17	T	C	0.466	0.071	0.007	2.2E-22	trans
1456	NAPRT	Q6XQN6	Nicotinate phosphoribosyltransferase	rs10282929	144681777	8	T	G	0.796	0.410	0.009	0.0E+00	cis
1457	NARS1	O43776	Asparagine--tRNA ligase, cytoplasmic	rs1354034	56849749	3	C	T	0.604	0.061	0.008	1.2E-15	trans
1457	NARS1	O43776	Asparagine--tRNA ligase, cytoplasmic	rs13257521	9031995	8	G	C	0.211	0.091	0.009	5.8E-23	trans
1458	NBL1	P41271	Neuroblastoma suppressor of tumorigenicity 1	rs12408663	19968058	1	T	C	0.768	0.123	0.009	1.9E-44	cis
1458	NBL1	P41271	Neuroblastoma suppressor of tumorigenicity 1	rs1260326	27730940	2	C	T	0.609	0.055	0.008	3.0E-13	trans
1458	NBL1	P41271	Neuroblastoma suppressor of tumorigenicity 1	rs36060036	20361950	16	C	T	0.833	0.076	0.010	1.3E-14	trans
1459	NBN	O60934	Nibrin	rs1354034	56849749	3	C	T	0.604	0.061	0.008	6.4E-16	trans
1459	NBN	O60934	Nibrin	rs143331722	131930968	5	G	A	0.988	0.315	0.036	2.8E-18	trans
1459	NBN	O60934	Nibrin	rs74378230	91043364	8	A	G	0.996	0.607	0.058	5.7E-26	cis
1460	NCAM1	P13591	Neural cell adhesion molecule 1	rs333947	110470764	1	G	A	0.852	0.065	0.010	1.0E-11	trans
1460	NCAM1	P13591	Neural cell adhesion molecule 1	rs2301455	172418268	1	T	C	0.559	0.060	0.007	3.2E-18	trans
1460	NCAM1	P13591	Neural cell adhesion molecule 1	rs66824127	52551265	3	C	T	0.557	0.060	0.007	1.8E-18	trans
1460	NCAM1	P13591	Neural cell adhesion molecule 1	rs28383282	32586375	6	T	C	0.211	0.062	0.009	7.0E-13	trans
1460	NCAM1	P13591	Neural cell adhesion molecule 1	rs11214489	112975934	11	C	T	0.815	0.333	0.009	5.7E-284	cis
1460	NCAM1	P13591	Neural cell adhesion molecule 1	rs9318188	74110416	13	A	G	0.455	0.114	0.007	1.1E-60	trans
1460	NCAM1	P13591	Neural cell adhesion molecule 1	rs2035645	92946326	15	G	T	0.562	0.107	0.007	5.6E-54	trans
1461	NCAM2	O15394	Neural cell adhesion molecule 2	rs2071916	426476	16	T	C	0.414	0.194	0.007	1.5E-182	trans
1461	NCAM2	O15394	Neural cell adhesion molecule 2	rs186021206	7069412	17	A	G	0.006	0.427	0.045	3.4E-21	trans
1461	NCAM2	O15394	Neural cell adhesion molecule 2	rs77542162	67081278	17	G	A	0.023	0.188	0.022	1.5E-17	trans
1461	NCAM2	O15394	Neural cell adhesion molecule 2	rs4760	44153100	19	G	A	0.156	0.116	0.009	3.4E-37	trans
1461	NCAM2	O15394	Neural cell adhesion molecule 2	rs760715	39862343	22	C	T	0.247	0.068	0.008	2.8E-17	trans
1462	NCAN	O14594	Neurocan core protein	rs61804160	161620089	1	A	T	0.142	0.067	0.010	9.3E-12	trans
1462	NCAN	O14594	Neurocan core protein	rs6786821	140999276	3	G	C	0.622	0.095	0.007	1.3E-40	trans
1462	NCAN	O14594	Neurocan core protein	rs12521652	83018430	5	A	G	0.670	0.050	0.007	1.6E-11	trans
1462	NCAN	O14594	Neurocan core protein	rs6913309	32339840	6	A	T	0.280	0.053	0.008	1.2E-11	trans
1462	NCAN	O14594	Neurocan core protein	rs12175504	149985215	6	G	T	0.354	0.083	0.007	5.4E-30	trans
1462	NCAN	O14594	Neurocan core protein	rs12801188	47388214	11	A	G	0.361	0.057	0.007	2.7E-15	trans
1462	NCAN	O14594	Neurocan core protein	rs60111444	101629538	15	G	A	0.226	0.068	0.008	1.6E-16	trans
1462	NCAN	O14594	Neurocan core protein	rs7191514	17463790	16	G	A	0.658	0.075	0.007	2.7E-24	trans
1462	NCAN	O14594	Neurocan core protein	rs6504649	48437456	17	C	G	0.612	0.068	0.007	1.6E-21	trans
1462	NCAN	O14594	Neurocan core protein	rs7234321	48532085	18	T	G	0.622	0.062	0.007	5.0E-18	trans
1462	NCAN	O14594	Neurocan core protein	rs3761077	19325963	19	G	T	0.890	0.241	0.011	2.3E-102	cis
1463	NCF2	P19878	Neutrophil cytosol factor 2	rs35012521	183532364	1	T	A	0.990	0.948	0.036	6.8E-154	cis
1463	NCF2	P19878	Neutrophil cytosol factor 2	rs149698681	42651900	15	C	G	0.022	0.209	0.025	3.6E-17	trans
1463	NCF2	P19878	Neutrophil cytosol factor 2	rs12941811	38159335	17	T	C	0.425	0.055	0.007	2.1E-14	trans
1463	NCF2	P19878	Neutrophil cytosol factor 2	rs4632248	54324995	19	T	G	0.209	0.158	0.009	1.6E-71	trans
1464	NCR1	O76036	Natural cytotoxicity triggering receptor 1	rs181807963	154976671	1	G	T	0.992	0.307	0.043	1.2E-12	trans
1464	NCR1	O76036	Natural cytotoxicity triggering receptor 1	rs6693836	185411306	1	A	G	0.638	0.065	0.008	1.2E-17	trans
1464	NCR1	O76036	Natural cytotoxicity triggering receptor 1	rs11586197	208028029	1	C	T	0.248	0.067	0.008	2.8E-15	trans
1464	NCR1	O76036	Natural cytotoxicity triggering receptor 1	rs76529516	60583370	2	G	A	0.028	0.155	0.022	2.6E-12	trans
1464	NCR1	O76036	Natural cytotoxicity triggering receptor 1	rs2009581	111807677	2	G	A	0.728	0.127	0.008	2.2E-54	trans
1464	NCR1	O76036	Natural cytotoxicity triggering receptor 1	rs13104208	142670642	4	C	T	0.159	0.082	0.010	1.2E-16	trans
1464	NCR1	O76036	Natural cytotoxicity triggering receptor 1	rs2248116	131804347	5	C	A	0.429	0.062	0.007	2.4E-17	trans
1464	NCR1	O76036	Natural cytotoxicity triggering receptor 1	rs9260316	29916914	6	G	T	0.435	0.064	0.007	8.0E-18	trans
1464	NCR1	O76036	Natural cytotoxicity triggering receptor 1	rs78565598	60095481	8	G	T	0.168	0.070	0.010	9.4E-13	trans
1464	NCR1	O76036	Natural cytotoxicity triggering receptor 1	rs7086174	6016399	10	A	C	0.329	0.083	0.008	1.6E-26	trans
1464	NCR1	O76036	Natural cytotoxicity triggering receptor 1	rs1250569	81045207	10	C	T	0.445	0.064	0.007	3.6E-18	trans
1464	NCR1	O76036	Natural cytotoxicity triggering receptor 1	rs6586167	90756146	10	G	T	0.499	0.056	0.007	2.6E-14	trans
1464	NCR1	O76036	Natural cytotoxicity triggering receptor 1	rs9916257	33797371	17	G	T	0.561	0.082	0.007	3.3E-29	trans
1464	NCR1	O76036	Natural cytotoxicity triggering receptor 1	rs4065275	38080865	17	G	A	0.501	0.071	0.007	1.7E-22	trans
1464	NCR1	O76036	Natural cytotoxicity triggering receptor 1	rs2659007	79217478	17	A	G	0.450	0.056	0.007	1.8E-14	trans
1464	NCR1	O76036	Natural cytotoxicity triggering receptor 1	rs7255591	55416170	19	G	C	0.911	0.426	0.013	4.4E-236	cis
1464	NCR1	O76036	Natural cytotoxicity triggering receptor 1	rs6103572	42657862	20	C	T	0.726	0.065	0.008	1.0E-15	trans
1465	NCR3LG1	Q68D85	Natural cytotoxicity triggering receptor 3 ligand 1	rs61747728	179526214	1	T	C	0.038	0.135	0.017	7.2E-15	trans
1465	NCR3LG1	Q68D85	Natural cytotoxicity triggering receptor 3 ligand 1	rs1047891	211540507	2	A	C	0.313	0.050	0.007	4.5E-12	trans
1465	NCR3LG1	Q68D85	Natural cytotoxicity triggering receptor 3 ligand 1	rs10935473	98416900	3	G	T	0.560	0.301	0.007	0.0E+00	trans
1465	NCR3LG1	Q68D85	Natural cytotoxicity triggering receptor 3 ligand 1	rs10058350	43141644	5	C	T	0.702	0.062	0.007	1.8E-17	trans
1465	NCR3LG1	Q68D85	Natural cytotoxicity triggering receptor 3 ligand 1	rs507666	136149399	9	G	A	0.816	0.326	0.009	4.4E-304	trans
1465	NCR3LG1	Q68D85	Natural cytotoxicity triggering receptor 3 ligand 1	rs12146443	17384498	11	C	T	0.617	0.361	0.007	0.0E+00	cis
1465	NCR3LG1	Q68D85	Natural cytotoxicity triggering receptor 3 ligand 1	rs36027301	67809268	11	T	C	0.057	0.107	0.015	6.0E-13	trans
1465	NCR3LG1	Q68D85	Natural cytotoxicity triggering receptor 3 ligand 1	rs186021206	7069412	17	A	G	0.006	0.954	0.046	4.9E-97	trans
1465	NCR3LG1	Q68D85	Natural cytotoxicity triggering receptor 3 ligand 1	rs12980852	54346093	19	G	A	0.340	0.057	0.007	1.0E-15	trans
1466	NCS1	P62166	Neuronal calcium sensor 1	rs66927685	169931266	2	T	TGAACCTTAGAGACTGAAGA	0.473	0.049	0.007	1.5E-12	trans
1466	NCS1	P62166	Neuronal calcium sensor 1	rs4704825	156382308	5	G	A	0.637	0.051	0.007	3.6E-13	trans
1466	NCS1	P62166	Neuronal calcium sensor 1	rs662	94937446	7	C	T	0.287	0.061	0.008	3.7E-16	trans
1466	NCS1	P62166	Neuronal calcium sensor 1	rs12342201	95894964	9	G	A	0.524	0.071	0.007	5.0E-25	trans
1466	NCS1	P62166	Neuronal calcium sensor 1	rs1054879	132995768	9	A	G	0.508	0.118	0.007	1.5E-66	cis
1466	NCS1	P62166	Neuronal calcium sensor 1	rs9807819	46239598	19	C	T	0.637	0.056	0.007	2.2E-15	trans
1466	NCS1	P62166	Neuronal calcium sensor 1	rs6065904	44534651	20	G	A	0.785	0.056	0.008	1.1E-11	trans
1467	NDUFB7	P17568	NADH dehydrogenase [ubiquinone] 1 beta subcomplex subunit 7	rs1801316	119005968	X	C	G	0.010	0.293	0.034	1.3E-17	trans
1468	NEB	P20929	Nebulin	rs41341748	16012594	8	A	G	0.011	0.266	0.037	6.0E-13	trans
1469	NECAP2	Q9NVZ3	Adaptin ear-binding coat-associated protein 2	rs2393967	65133156	10	C	A	0.314	0.062	0.008	7.9E-14	trans
1470	NECTIN2	Q92692	Nectin-2	rs61747728	179526214	1	T	C	0.038	0.183	0.019	2.5E-21	trans
1470	NECTIN2	Q92692	Nectin-2	rs1137844	36343079	19	G	C	0.315	0.060	0.008	1.5E-13	trans
1470	NECTIN2	Q92692	Nectin-2	rs440277	45361224	19	G	A	0.690	0.183	0.008	3.1E-112	cis
1470	NECTIN2	Q92692	Nectin-2	rs601338	49206674	19	G	A	0.491	0.158	0.008	3.0E-97	trans
1471	NECTIN4	Q96NY8	Nectin-4	rs35434391	161049509	1	G	T	0.883	0.447	0.012	0.0E+00	cis
1471	NECTIN4	Q96NY8	Nectin-4	rs61747728	179526214	1	T	C	0.038	0.144	0.019	1.1E-13	trans
1471	NECTIN4	Q96NY8	Nectin-4	rs1260326	27730940	2	C	T	0.609	0.068	0.007	1.7E-19	trans
1471	NECTIN4	Q96NY8	Nectin-4	rs79069746	71205473	10	C	T	0.138	0.100	0.011	2.2E-21	trans
1471	NECTIN4	Q96NY8	Nectin-4	rs77924615	20392332	16	G	A	0.803	0.079	0.009	2.1E-17	trans
1472	NEDD4L	Q96PU5	E3 ubiquitin-protein ligase NEDD4-like	rs77100396	2669976	7	A	C	0.016	0.216	0.030	4.5E-13	trans
1472	NEDD4L	Q96PU5	E3 ubiquitin-protein ligase NEDD4-like	rs74930558	56216182	15	A	G	0.218	0.086	0.009	6.8E-22	trans
1473	NEDD9	Q14511	Enhancer of filamentation 1	rs34436714	54327313	19	A	C	0.209	0.110	0.009	1.9E-31	trans
1474	NEFL	P07196	Neurofilament light polypeptide	rs7197999	75278920	16	T	C	0.524	0.046	0.007	2.1E-12	trans
1475	NELL1	Q92832	Protein kinase C-binding protein NELL1	rs10890482	47946433	1	C	T	0.808	0.076	0.009	3.2E-17	trans
1475	NELL1	Q92832	Protein kinase C-binding protein NELL1	rs4344946	120189720	2	A	G	0.136	0.102	0.010	7.0E-23	trans
1475	NELL1	Q92832	Protein kinase C-binding protein NELL1	rs3903119	176992443	2	T	C	0.340	0.059	0.007	2.6E-15	trans
1475	NELL1	Q92832	Protein kinase C-binding protein NELL1	rs13107325	103188709	4	T	C	0.075	0.176	0.013	2.5E-39	trans
1475	NELL1	Q92832	Protein kinase C-binding protein NELL1	rs9501590	31364384	6	G	A	0.340	0.054	0.007	4.5E-13	trans
1475	NELL1	Q92832	Protein kinase C-binding protein NELL1	rs2207136	50809720	6	T	C	0.533	0.053	0.007	6.1E-14	trans
1475	NELL1	Q92832	Protein kinase C-binding protein NELL1	rs11458788	100953047	6	AC	A	0.505	0.051	0.007	1.1E-12	trans
1475	NELL1	Q92832	Protein kinase C-binding protein NELL1	rs2339228	91734482	8	A	G	0.502	0.063	0.007	2.0E-19	trans
1475	NELL1	Q92832	Protein kinase C-binding protein NELL1	rs56278466	17875857	10	G	T	0.661	0.067	0.007	1.2E-19	trans
1475	NELL1	Q92832	Protein kinase C-binding protein NELL1	rs7917848	28994862	10	C	G	0.349	0.099	0.007	8.2E-41	trans
1475	NELL1	Q92832	Protein kinase C-binding protein NELL1	rs61652119	20955270	11	A	G	0.049	0.794	0.017	0.0E+00	cis
1475	NELL1	Q92832	Protein kinase C-binding protein NELL1	rs2298475	126278203	11	T	C	0.925	0.163	0.014	3.3E-32	trans
1475	NELL1	Q92832	Protein kinase C-binding protein NELL1	rs4541022	94166312	15	C	T	0.119	0.085	0.011	7.0E-15	trans
1475	NELL1	Q92832	Protein kinase C-binding protein NELL1	rs186021206	7069412	17	A	G	0.006	0.622	0.049	1.4E-37	trans
1475	NELL1	Q92832	Protein kinase C-binding protein NELL1	rs72835417	47241642	17	A	G	0.110	0.140	0.011	1.9E-35	trans
1475	NELL1	Q92832	Protein kinase C-binding protein NELL1	rs2188264	112145059	X	C	T	0.779	0.058	0.007	1.3E-16	trans
1476	NELL2	Q99435	Protein kinase C-binding protein NELL2	rs17285324	46482980	3	A	T	0.825	0.070	0.009	1.0E-13	trans
1476	NELL2	Q99435	Protein kinase C-binding protein NELL2	rs13303	52558008	3	C	T	0.560	0.063	0.007	4.1E-18	trans
1476	NELL2	Q99435	Protein kinase C-binding protein NELL2	rs1126735	58413408	3	G	A	0.369	0.056	0.007	5.1E-14	trans
1476	NELL2	Q99435	Protein kinase C-binding protein NELL2	rs55818129	39425092	5	T	G	0.882	0.077	0.011	6.8E-12	trans
1476	NELL2	Q99435	Protein kinase C-binding protein NELL2	rs113239947	31461956	6	A	T	0.757	0.068	0.008	2.8E-16	trans
1476	NELL2	Q99435	Protein kinase C-binding protein NELL2	rs2519093	136141870	9	C	T	0.816	0.198	0.009	1.5E-100	trans
1476	NELL2	Q99435	Protein kinase C-binding protein NELL2	rs56278466	17875857	10	G	T	0.661	0.062	0.008	1.3E-16	trans
1476	NELL2	Q99435	Protein kinase C-binding protein NELL2	rs76485191	21192233	11	T	G	0.340	0.056	0.008	5.2E-13	trans
1476	NELL2	Q99435	Protein kinase C-binding protein NELL2	rs36027301	67809268	11	C	T	0.943	0.125	0.015	6.0E-16	trans
1476	NELL2	Q99435	Protein kinase C-binding protein NELL2	rs186021206	7069412	17	A	G	0.006	0.426	0.049	3.7E-18	trans
1476	NELL2	Q99435	Protein kinase C-binding protein NELL2	rs4804790	7797154	19	G	T	0.929	0.132	0.014	4.3E-21	trans
1477	NENF	Q9UMX5	Neudesin	rs607929	32586998	6	C	G	0.551	0.089	0.008	8.8E-31	trans
1477	NENF	Q9UMX5	Neudesin	rs6473226	81285759	8	C	T	0.626	0.060	0.008	4.3E-14	trans
1478	NEO1	Q92859	Neogenin	rs11632433	73467072	15	G	T	0.098	0.114	0.013	2.6E-19	cis
1479	NEXN	Q0ZGT2	Nexilin	rs1354034	56849749	3	C	T	0.604	0.055	0.008	3.4E-13	trans
1479	NEXN	Q0ZGT2	Nexilin	rs342293	106372219	7	C	G	0.540	0.052	0.007	3.4E-12	trans

1479	NEXN	Q0ZGT2	Nexilin	rs7924036	65191645	10	T	G	0.501	0.052	0.007	1.8E-12	trans
1480	NFASC	O94856	Neurofascin	rs2519093	136141870	9	C	T	0.816	0.090	0.009	3.8E-25	trans
1480	NFASC	O94856	Neurofascin	rs56278466	17875857	10	G	T	0.661	0.167	0.007	6.9E-121	trans
1480	NFASC	O94856	Neurofascin	rs58534292	106483584	14	C	T	0.650	0.054	0.007	2.3E-14	trans
1480	NFASC	O94856	Neurofascin	rs12895651	106494269	14	C	T	0.701	0.092	0.008	2.3E-29	trans
1480	NFASC	O94856	Neurofascin	rs3747207	44324855	22	A	G	0.216	0.066	0.008	9.2E-16	trans
1481	NFATC1	O95644	Nuclear factor of activated T-cells, cytoplasmic 1	rs657693	77162462	18	A	G	0.640	0.110	0.008	1.5E-46	cis
1482	NFATC3	Q12968	Nuclear factor of activated T-cells, cytoplasmic 3	rs570919834	32481691	6	T	C	0.255	0.108	0.010	8.6E-30	trans
1482	NFATC3	Q12968	Nuclear factor of activated T-cells, cytoplasmic 3	rs13263709	81287175	8	C	T	0.648	0.060	0.008	8.5E-14	trans
1483	NFE2	Q16621	Transcription factor NF-E2 45 kDa subunit	rs1354034	56849749	3	C	T	0.605	0.053	0.008	3.7E-12	trans
1483	NFE2	Q16621	Transcription factor NF-E2 45 kDa subunit	rs342298	106373646	7	C	T	0.545	0.069	0.008	2.6E-20	trans
1484	NFKB1	P19838	Nuclear factor NF-kappa-B p105 subunit	rs1354034	56849749	3	C	T	0.604	0.063	0.008	2.8E-16	trans
1484	NFKB1	P19838	Nuclear factor NF-kappa-B p105 subunit	rs17032705	103432974	4	A	G	0.398	0.115	0.008	1.1E-50	cis
1485	NFKBIE	O00221	NF-kappa-B inhibitor epsilon	rs1354034	56849749	3	C	T	0.604	0.058	0.008	2.8E-14	trans
1485	NFKBIE	O00221	NF-kappa-B inhibitor epsilon	rs28362856	44234539	6	G	C	0.957	0.523	0.019	4.4E-174	cis
1486	NFU1	Q9UMS0	NFU1 iron-sulfur cluster scaffold homolog, mitochondrial	rs74637005	69650730	2	G	A	0.963	0.328	0.021	5.2E-57	cis
1486	NFU1	Q9UMS0	NFU1 iron-sulfur cluster scaffold homolog, mitochondrial	rs1354034	56849749	3	C	T	0.605	0.058	0.008	5.1E-14	trans
1486	NFU1	Q9UMS0	NFU1 iron-sulfur cluster scaffold homolog, mitochondrial	rs342299	106373718	7	C	T	0.545	0.054	0.008	1.8E-12	trans
1486	NFU1	Q9UMS0	NFU1 iron-sulfur cluster scaffold homolog, mitochondrial	rs6993770	106581528	8	A	T	0.713	0.073	0.008	2.0E-18	trans
1487	NFX1	Q12986	Transcriptional repressor NF-X1	rs1354034	56849749	3	C	T	0.604	0.057	0.008	6.5E-14	trans
1488	NGRN	Q9NPE2	Neugrin	rs3751488	23304094	14	A	G	0.235	0.061	0.009	9.6E-12	trans
1488	NGRN	Q9NPE2	Neugrin	rs11085147	5711930	19	T	C	0.099	0.119	0.013	6.1E-20	trans
1489	NHLRC3	Q5JS37	NHL repeat-containing protein 3	rs9264645	31238524	6	A	G	0.641	0.120	0.008	1.8E-54	trans
1489	NHLRC3	Q5JS37	NHL repeat-containing protein 3	rs56278466	17875857	10	G	T	0.660	0.057	0.008	1.5E-13	trans
1489	NHLRC3	Q5JS37	NHL repeat-containing protein 3	rs149175958	39621965	13	G	T	0.996	2.127	0.057	2.7E-308	cis
1489	NHLRC3	Q5JS37	NHL repeat-containing protein 3	rs1801248	52520435	13	T	C	0.043	0.206	0.018	3.5E-29	trans
1489	NHLRC3	Q5JS37	NHL repeat-containing protein 3	rs2269335	88902471	16	T	G	0.326	0.063	0.008	1.8E-15	trans
1489	NHLRC3	Q5JS37	NHL repeat-containing protein 3	rs112001035	66823805	17	G	A	0.939	0.107	0.016	8.1E-12	trans
1490	NID1	P14543	Nidogen-1	rs6671171	156871318	1	A	G	0.111	0.085	0.012	4.4E-13	trans
1490	NID1	P14543	Nidogen-1	rs76183323	236230317	1	G	A	0.989	0.762	0.038	8.9E-88	cis
1490	NID1	P14543	Nidogen-1	rs13412535	224874874	2	G	A	0.769	0.069	0.009	1.2E-15	trans
1490	NID1	P14543	Nidogen-1	rs11719450	58456811	3	T	C	0.370	0.064	0.008	2.4E-17	trans
1490	NID1	P14543	Nidogen-1	rs34716199	72193315	5	AT	A	0.536	0.055	0.007	1.1E-13	trans
1490	NID1	P14543	Nidogen-1	rs4098923	31193756	6	A	G	0.553	0.069	0.008	1.5E-19	trans
1490	NID1	P14543	Nidogen-1	rs342299	106373718	7	C	T	0.545	0.056	0.007	3.1E-14	trans
1490	NID1	P14543	Nidogen-1	rs6993770	106581528	8	A	T	0.714	0.057	0.008	1.4E-12	trans
1490	NID1	P14543	Nidogen-1	rs927826	20220846	10	T	G	0.706	0.187	0.008	8.7E-120	trans
1490	NID1	P14543	Nidogen-1	rs10761731	65027610	10	T	A	0.415	0.089	0.007	3.1E-33	trans
1490	NID1	P14543	Nidogen-1	rs12445050	81870969	16	T	C	0.138	0.091	0.011	5.4E-18	trans
1490	NID1	P14543	Nidogen-1	rs892090	55539072	19	G	T	0.835	0.129	0.010	5.5E-39	trans
1491	NID2	Q14112	Nidogen-2	rs13412535	224874874	2	G	A	0.769	0.058	0.008	7.3E-13	trans
1491	NID2	Q14112	Nidogen-2	rs7618405	18250509	3	C	A	0.795	0.058	0.009	9.4E-12	trans
1491	NID2	Q14112	Nidogen-2	rs1354034	56849749	3	T	C	0.396	0.056	0.007	4.8E-16	trans
1491	NID2	Q14112	Nidogen-2	rs274555	131722951	5	T	C	0.399	0.052	0.007	2.5E-13	trans
1491	NID2	Q14112	Nidogen-2	rs35173808	31326148	6	C	A	0.904	0.096	0.012	1.2E-16	trans
1491	NID2	Q14112	Nidogen-2	rs1917342	71349754	6	A	G	0.697	0.051	0.007	5.2E-12	trans
1491	NID2	Q14112	Nidogen-2	rs6961069	80218961	7	T	C	0.402	0.058	0.007	1.4E-16	trans
1491	NID2	Q14112	Nidogen-2	rs6993770	106581528	8	A	T	0.714	0.095	0.008	4.7E-36	trans
1491	NID2	Q14112	Nidogen-2	rs10820606	99192919	9	A	C	0.770	0.071	0.008	5.4E-18	trans
1491	NID2	Q14112	Nidogen-2	rs10740118	65101207	10	C	G	0.415	0.088	0.007	2.1E-36	trans
1491	NID2	Q14112	Nidogen-2	rs12767683	104316581	10	A	C	0.325	0.057	0.007	7.3E-15	trans
1491	NID2	Q14112	Nidogen-2	rs2516600	52487766	14	C	T	0.544	0.576	0.008	0.0E+00	cis
1491	NID2	Q14112	Nidogen-2	rs59001897	65160392	15	A	T	0.175	0.065	0.009	6.4E-13	trans
1491	NID2	Q14112	Nidogen-2	rs12445050	81870969	16	T	C	0.138	0.100	0.010	3.0E-24	trans
1491	NID2	Q14112	Nidogen-2	rs892090	55539072	19	G	T	0.834	0.143	0.009	6.2E-54	trans
1491	NID2	Q14112	Nidogen-2	rs3827978	19281291	20	T	C	0.353	0.070	0.007	2.3E-22	trans
1491	NID2	Q14112	Nidogen-2	rs8780	99929532	X	C	G	0.339	0.044	0.006	1.7E-13	trans
1492	NINJ1	Q92982	Ninjurin-1	rs9274442	32633231	6	G	C	0.718	0.084	0.009	8.7E-20	trans
1492	NINJ1	Q92982	Ninjurin-1	rs11379524	95882745	9	AC	A	0.630	0.243	0.008	1.2E-201	cis
1493	NIT1	Q86X76	Deaminated glutathione amidase	rs76502631	161088000	1	G	A	0.972	0.726	0.023	1.0E-224	cis
1494	NIT2	Q9NQR4	Omega-amidase NIT2	rs200416332	100057993	3	G	A	0.998	1.264	0.094	7.2E-41	cis
1495	NME3	Q13232	Nucleoside diphosphate kinase 3	rs188468174	25291697	1	C	T	0.986	0.372	0.031	2.7E-32	trans
1495	NME3	Q13232	Nucleoside diphosphate kinase 3	rs57008601	203975534	2	T	G	0.431	0.055	0.007	1.9E-13	trans
1495	NME3	Q13232	Nucleoside diphosphate kinase 3	rs373385028	96325026	5	A	T	0.449	0.079	0.007	2.2E-26	trans
1495	NME3	Q13232	Nucleoside diphosphate kinase 3	rs1014651	102976644	9	A	G	0.531	0.050	0.007	1.7E-11	trans
1495	NME3	Q13232	Nucleoside diphosphate kinase 3	rs2519093	136141870	9	C	T	0.816	0.113	0.010	2.3E-32	trans
1495	NME3	Q13232	Nucleoside diphosphate kinase 3	rs11825456	125472348	11	A	T	0.067	0.103	0.015	3.8E-12	trans
1495	NME3	Q13232	Nucleoside diphosphate kinase 3	rs55707100	43820717	15	C	T	0.976	0.178	0.024	1.0E-13	trans
1495	NME3	Q13232	Nucleoside diphosphate kinase 3	rs1801689	64210580	17	C	A	0.030	0.156	0.022	4.0E-13	trans
1495	NME3	Q13232	Nucleoside diphosphate kinase 3	rs312068	3207338	19	C	T	0.453	0.093	0.007	2.6E-35	trans
1495	NME3	Q13232	Nucleoside diphosphate kinase 3	rs7599	36038390	19	A	G	0.366	0.152	0.008	1.6E-88	trans
1495	NME3	Q13232	Nucleoside diphosphate kinase 3	rs12975366	54759361	19	T	C	0.605	0.077	0.008	1.5E-24	trans
1495	NME3	Q13232	Nucleoside diphosphate kinase 3	rs5749077	30760818	22	C	A	0.231	0.064	0.009	7.9E-13	trans
1496	NMI	Q13287	N-myc-interactor	rs1048135	152139416	2	G	A	0.528	0.453	0.008	0.0E+00	cis
1496	NMI	Q13287	N-myc-interactor	rs1354034	56849749	3	C	T	0.604	0.051	0.007	5.9E-12	trans
1496	NMI	Q13287	N-myc-interactor	rs528854	41098108	17	A	G	0.908	0.092	0.013	2.0E-13	trans
1497	NMNAT1	Q9HAN9	Nicotinamide/nicotinic acid mononucleotide adenylyltransferase 1	rs180754466	10020795	1	A	C	0.010	0.671	0.039	3.7E-66	cis
1497	NMNAT1	Q9HAN9	Nicotinamide/nicotinic acid mononucleotide adenylyltransferase 1	rs9271573	32590501	6	C	A	0.598	0.054	0.007	2.6E-13	trans
1497	NMNAT1	Q9HAN9	Nicotinamide/nicotinic acid mononucleotide adenylyltransferase 1	rs58534292	106483584	14	T	C	0.350	0.088	0.008	2.4E-30	trans
1497	NMNAT1	Q9HAN9	Nicotinamide/nicotinic acid mononucleotide adenylyltransferase 1	rs34799380	106464822	14	T	C	0.849	0.190	0.010	1.1E-77	trans
1497	NMNAT1	Q9HAN9	Nicotinamide/nicotinic acid mononucleotide adenylyltransferase 1	rs704	26694861	17	G	A	0.527	0.200	0.007	1.1E-165	trans
1498	NMT1	P30419	Glycylpeptide N-tetradecanoyltransferase 1	rs1354034	56849749	3	C	T	0.604	0.055	0.008	8.0E-13	trans
1499	NOMO1	Q15155	Nodal modulator 1	rs174544	61567753	11	A	C	0.309	0.077	0.008	1.0E-24	trans
1499	NOMO1	Q15155	Nodal modulator 1	rs28929474	94844947	14	T	C	0.021	0.396	0.025	1.4E-58	trans
1499	NOMO1	Q15155	Nodal modulator 1	rs1801689	64210580	17	C	A	0.030	0.183	0.020	3.0E-19	trans
1500	NOS1	P29475	Nitric oxide synthase, brain	rs12574283	22255486	11	A	G	0.710	0.068	0.008	1.6E-17	trans
1500	NOS1	P29475	Nitric oxide synthase, brain	rs1800273	31986607	X	G	A	0.955	0.144	0.014	2.6E-24	trans
1501	NOS2	P35228	Nitric oxide synthase, inducible	rs2297518	26096597	17	G	A	0.811	0.091	0.010	1.1E-20	cis
1502	NOS3	P29474	Nitric oxide synthase, endothelial	rs7572209	86090072	2	T	C	0.801	0.085	0.009	2.6E-19	trans
1502	NOS3	P29474	Nitric oxide synthase, endothelial	rs6415128	32330894	6	A	G	0.559	0.051	0.008	1.2E-11	trans
1503	NOTCH1	P46531	Neurogenic locus notch homolog protein 1	rs1260326	27730940	2	C	T	0.608	0.058	0.007	7.9E-15	trans
1503	NOTCH1	P46531	Neurogenic locus notch homolog protein 1	rs4240624	9184231	8	G	A	0.092	0.106	0.013	4.6E-17	trans
1503	NOTCH1	P46531	Neurogenic locus notch homolog protein 1	rs56278466	17875857	10	G	T	0.661	0.079	0.008	3.8E-25	trans
1503	NOTCH1	P46531	Neurogenic locus notch homolog protein 1	rs34434834	126307796	11	A	G	0.033	0.142	0.020	3.5E-12	trans
1503	NOTCH1	P46531	Neurogenic locus notch homolog protein 1	rs186021206	7069412	17	A	G	0.006	0.636	0.050	7.2E-37	trans
1504	NOTCH2	Q04721	Neurogenic locus notch homolog protein 2	rs2641348	120437884	1	A	G	0.889	0.172	0.012	1.9E-47	cis
1504	NOTCH2	Q04721	Neurogenic locus notch homolog protein 2	rs12088164	145383227	1	G	A	0.038	1.611	0.019	0.0E+00	trans
1504	NOTCH2	Q04721	Neurogenic locus notch homolog protein 2	rs17580	94847262	14	A	T	0.050	0.287	0.016	1.4E-74	trans
1504	NOTCH2	Q04721	Neurogenic locus notch homolog protein 2	rs186021206	7069412	17	A	G	0.006	0.331	0.047	2.1E-12	trans
1504	NOTCH2	Q04721	Neurogenic locus notch homolog protein 2	rs77542162	67081278	17	G	A	0.023	0.158	0.023	4.7E-12	trans
1505	NOTCH3	Q9UM47	Neurogenic locus notch homolog protein 3	rs1260326	27730940	2	C	T	0.608	0.068	0.007	1.5E-21	trans
1505	NOTCH3	Q9UM47	Neurogenic locus notch homolog protein 3	rs8192585	32188823	6	G	A	0.953	0.115	0.017	4.0E-12	trans
1505	NOTCH3	Q9UM47	Neurogenic locus notch homolog protein 3	rs34114302	2533790	7	C	T	0.678	0.071	0.007	2.8E-21	trans
1505	NOTCH3	Q9UM47	Neurogenic locus notch homolog protein 3	rs507666	136149399	9	G	A	0.815	0.073	0.009	1.0E-15	trans
1505	NOTCH3	Q9UM47	Neurogenic locus notch homolog protein 3	rs17580	94847262	14	T	A	0.951	0.155	0.016	5.7E-22	trans
1505	NOTCH3	Q9UM47	Neurogenic locus notch homolog protein 3	rs139974673	44027885	15	T	C	0.976	0.198	0.023	2.3E-18	trans
1505	NOTCH3	Q9UM47	Neurogenic locus notch homolog protein 3	rs186021206	7069412	17	A	G	0.006	0.483	0.048	1.4E-23	trans
1505	NOTCH3	Q9UM47	Neurogenic locus notch homolog protein 3	rs77542162	67081278	17	G	A	0.023	0.189	0.023	7.6E-16	trans
1505	NOTCH3	Q9UM47	Neurogenic locus notch homolog protein 3	rs147373451	15302941	19	T	C	0.997	1.324	0.070	8.4E-80	cis
1506	NPC2	P61916	NPC intracellular cholesterol transporter 2	rs28394165	77394018	4	C	T	0.456	0.049	0.007	1.2E-11	trans
1506	NPC2	P61916	NPC intracellular cholesterol transporter 2	rs1050420	31239518	6	C	T	0.632	0.051	0.007	6.7E-12	trans
1506	NPC2	P61916	NPC intracellular cholesterol transporter 2	rs140130028	74947404	14	C	T	0.991	2.022	0.038	0.0E+00	cis
1507	NPDC1	Q9NQX5	Neural proliferation differentiation and control protein 1	rs13146355	77412140	4	A	G	0.456	0.051	0.007	1.7E-12	trans
1507	NPDC1	Q9NQX5	Neural proliferation differentiation and control protein 1	rs10265221	151414329	7	C	T	0.287	0.059	0.008	1.6E-13	trans
1507	NPDC1	Q9NQX5	Neural proliferation differentiation and control protein 1	rs36060036	20361950	16	C	T	0.832	0.083	0.010	1.3E-17	trans
1507	NPDC1	Q9NQX5	Neural proliferation differentiation and control protein 1	rs11672900	49220323	19	G	A	0.544	0.057	0.007	4.2E-15	trans
1508	NPHS1	O60500	Nephrin	rs1337526	47965130	1	G	A	0.804	0.072	0.009	1.8E-15	trans
1508	NPHS1	O60500	Nephrin	rs9383023	15109703	6	T	C	0.743	0.058	0.008	4.5E-12	trans
1508	NPHS1	O60500	Nephrin	rs2298475	126278203	11	T	C	0.925	0.092	0.014	1.2E-11	trans
1509	NPL	Q9BXD5	N-acetylneuraminate lyase	rs116448311	183096425	1	C	T	0.995	1.708	0.045	0.0E+00	cis
1509	NPL	Q9BXD5	N-acetylneuraminate lyase	rs192695654	52538331	3	T	C	0.006	0.412	0.043	2.3E-21	trans
1509	NPL	Q9BXD5	N-acetylneuraminate lyase	rs7675964	9941434	4	C	T	0.726	0.093	0.007	1.2E-36	trans
1509	NPL	Q9BXD5	N-acetylneuraminate lyase	rs72492305	32509333	6	C	T	0.325	0.052	0.007	8.1E-14	trans
1509	NPL	Q9BXD5	N-acetylneuraminate lyase	rs2398827	95907107	9	C	T	0.602	0.050	0.007	1.8E-13	trans
1509	NPL	Q9BXD5	N-acetylneuraminate lyase	rs1065853	45413233	19	T	G	0.081	0.088	0.012	4.0E-13	trans
1510	NPPB	P16860	Natriuretic peptides B	rs198389	11919271	1	G	A	0.419	0.183	0.008	1.2E-127	cis
1510	NPPB	P16860	Natriuretic peptides B	rs2234962	121429633	10	C	T	0.216	0.068	0.009	4.4E-14	trans
1511	NPPC	P23582	C-type natriuretic peptide	rs77806605	49361661	2	G	C	0.266	0.052	0.008	4.0E-12	trans
1511	NPPC	P23582	C-type natriuretic peptide	rs4859682	77410318	4	A	C	0.457	0.047	0.007	1.1E-12	trans
1512	NPR1	P16066	Atrial natriuretic peptide receptor 1	rs9274278	32631711	6	C	T	0.552	0.060	0.008	7.1E-13	trans
1513	NPTX1	Q15818	Neuronal pentraxin-1	rs78265569	2146165	1	A	C	0.088	0.102	0.012	1.6E-18	trans
1513	NPTX1	Q15818	Neuronal pentraxin-1	rs12025349	213940781	1	T	C	0.603	0.097	0.007	1.0E-47	trans
1513	NPTX1	Q15818	Neuronal pentraxin-1	rs13064675	24230288	3	T	C	0.805	0.059	0.008	7.8E-13	trans
1513	NPTX1	Q15818	Neuronal pentraxin-1	rs4693802	87979212	4	A	G	0.754	0.063	0.008	9.4E-17	trans
1513	NPTX1	Q15818	Neuronal pentraxin-1	rs66833621	6923834	6	C	T	0.194	0.118	0.008	2.8E-45	trans
1513	NPTX1	Q15818	Neuronal pentraxin-1	rs16896742	29922740	6	A	G	0.637	0.239	0.007	1.3E-262	trans
1513	NPTX1	Q15818	Neuronal pentraxin-1	rs2519093	136141870	9	C	T	0.816	0.097	0.008	1.8E-30	trans
1513	NPTX1	Q15818	Neuronal pentraxin-1	rs10786548	101256326	10	A	G	0.891	0.074	0.010	1.2E-12	trans
1513	NPTX1	Q15818	Neuronal pentraxin-1	rs7937122	126224448	11	G	C	0.390	0.078	0.007	9.1E-31	trans
1513	NPTX1	Q15818	Neuronal pentraxin-1	rs186021206	7069412	17	A	G	0.006	0.710	0.049	2.2E-47	trans
1513	NPTX1	Q15818	Neuronal pentraxin-1	rs62068268	78539090	17	T	C	0.089	0.818	0.013	0.0E+00	cis
1513	NPTX1	Q15818	Neuronal pentraxin-1	rs4806469	55698245	19	A	G	0.189	0.058	0.008	3.5E-12	trans
1513	NPTX1	Q15818	Neuronal pentraxin-1	rs7280064	16377650	21	T	C	0.154	0.069	0.009	1.6E-14	trans

1513	NPTX1	Q15818	Neuronal pentraxin-1	rs56292043	39663918	X	C	T	0.364	0.051	0.006	8.9E-20	trans
1514	NPTX2	P47972	Neuronal pentraxin-2	rs13303	52558008	3	C	T	0.560	0.055	0.006	6.1E-19	trans
1514	NPTX2	P47972	Neuronal pentraxin-2	rs71309976	56728081	3	T	C	0.011	0.281	0.032	6.8E-19	trans
1514	NPTX2	P47972	Neuronal pentraxin-2	rs147023494	29907978	6	C	T	0.973	0.171	0.019	1.0E-19	trans
1514	NPTX2	P47972	Neuronal pentraxin-2	rs61007246	98283815	7	T	C	0.715	0.344	0.007	0.0E+00	cis
1514	NPTX2	P47972	Neuronal pentraxin-2	rs186021206	7069412	17	A	G	0.006	0.295	0.042	1.7E-12	trans
1514	NPTX2	P47972	Neuronal pentraxin-2	rs192164176	39220697	22	G	C	0.002	0.499	0.072	3.9E-12	trans
1515	NPTXR	O95502	Neuronal pentraxin receptor	rs2240046	20371273	7	G	C	0.063	0.097	0.014	1.7E-11	trans
1515	NPTXR	O95502	Neuronal pentraxin receptor	rs61007246	98283815	7	T	C	0.714	0.098	0.008	3.1E-38	trans
1515	NPTXR	O95502	Neuronal pentraxin receptor	rs369705328	17848192	10	C	G	0.128	0.089	0.010	7.5E-18	trans
1515	NPTXR	O95502	Neuronal pentraxin receptor	rs186021206	7069412	17	A	G	0.006	0.316	0.047	1.4E-11	trans
1515	NPTXR	O95502	Neuronal pentraxin receptor	rs111577133	39241262	22	G	A	0.019	2.022	0.027	0.0E+00	cis
1516	NPY	P01303	Pro-neuropeptide Y	rs5030062	186454180	3	A	C	0.627	0.103	0.008	3.7E-43	trans
1516	NPY	P01303	Pro-neuropeptide Y	rs4861708	187157233	4	G	A	0.488	0.186	0.007	3.7E-143	trans
1516	NPY	P01303	Pro-neuropeptide Y	rs639933	134467751	5	A	C	0.631	0.055	0.008	4.5E-13	trans
1516	NPY	P01303	Pro-neuropeptide Y	rs1801020	176836532	5	A	G	0.255	0.090	0.008	2.0E-27	trans
1516	NPY	P01303	Pro-neuropeptide Y	rs2523675	31436032	6	A	G	0.341	0.054	0.008	1.9E-12	trans
1516	NPY	P01303	Pro-neuropeptide Y	rs56393506	161089307	6	T	C	0.172	0.075	0.010	1.2E-14	trans
1516	NPY	P01303	Pro-neuropeptide Y	rs9718966	545796	7	C	G	0.513	0.055	0.007	1.4E-13	trans
1516	NPY	P01303	Pro-neuropeptide Y	rs4722337	24309098	7	A	T	0.445	0.158	0.007	7.9E-103	cis
1516	NPY	P01303	Pro-neuropeptide Y	rs33951980	73029437	7	C	T	0.871	0.077	0.011	1.3E-12	trans
1516	NPY	P01303	Pro-neuropeptide Y	rs75802599	19843579	8	G	C	0.910	0.089	0.013	2.2E-12	trans
1516	NPY	P01303	Pro-neuropeptide Y	rs16888589	117635602	8	G	A	0.077	0.176	0.014	4.8E-38	trans
1516	NPY	P01303	Pro-neuropeptide Y	rs1809844	86188011	16	C	T	0.503	0.057	0.007	6.4E-15	trans
1516	NPY	P01303	Pro-neuropeptide Y	rs77542162	67081278	17	G	A	0.023	0.301	0.024	1.9E-35	trans
1516	NPY	P01303	Pro-neuropeptide Y	rs72654473	45414399	19	A	C	0.109	0.143	0.012	5.7E-35	trans
1517	NRCAM	Q92823	Neuronal cell adhesion molecule	rs61804210	161670827	1	T	C	0.105	0.104	0.012	2.9E-18	trans
1517	NRCAM	Q92823	Neuronal cell adhesion molecule	rs1260326	27730940	2	C	T	0.608	0.065	0.007	4.3E-18	trans
1517	NRCAM	Q92823	Neuronal cell adhesion molecule	rs1391373	32603660	6	C	T	0.359	0.057	0.008	4.4E-14	trans
1517	NRCAM	Q92823	Neuronal cell adhesion molecule	rs532436	136149830	9	G	A	0.815	0.109	0.009	7.5E-31	trans
1517	NRCAM	Q92823	Neuronal cell adhesion molecule	rs56278466	17875857	10	G	T	0.662	0.138	0.008	3.3E-72	trans
1517	NRCAM	Q92823	Neuronal cell adhesion molecule	rs186021206	7069412	17	A	G	0.006	0.377	0.050	3.9E-14	trans
1518	NRGN	Q92686	Neurogranin	rs11121012	7794289	1	G	A	0.647	0.086	0.008	1.5E-27	trans
1518	NRGN	Q92686	Neurogranin	rs1354034	56849749	3	C	T	0.604	0.053	0.008	4.9E-12	trans
1518	NRGN	Q92686	Neurogranin	rs342293	106372219	7	C	G	0.540	0.054	0.007	4.7E-13	trans
1518	NRGN	Q92686	Neurogranin	rs6993770	106581528	8	A	T	0.713	0.065	0.008	3.3E-15	trans
1519	NRP1	O14786	Neuropilin-1	rs181835401	63135955	1	A	G	0.354	0.092	0.008	8.2E-34	trans
1519	NRP1	O14786	Neuropilin-1	rs2090034	20372409	2	G	A	0.532	0.052	0.007	7.7E-13	trans
1519	NRP1	O14786	Neuropilin-1	rs1260326	27730940	2	C	T	0.608	0.066	0.007	4.6E-19	trans
1519	NRP1	O14786	Neuropilin-1	rs71071936	72869866	7	CT	C	0.122	0.088	0.011	7.8E-16	trans
1519	NRP1	O14786	Neuropilin-1	rs2506150	33483308	10	G	A	0.640	0.305	0.008	0.0E+00	cis
1519	NRP1	O14786	Neuropilin-1	rs186021206	7069412	17	A	G	0.006	0.603	0.049	3.1E-34	trans
1519	NRP1	O14786	Neuropilin-1	rs77542162	67081278	17	G	A	0.023	0.213	0.024	9.0E-19	trans
1520	NRP2	O60462	Neuropilin-2	rs4611630	62613467	2	A	G	0.194	0.062	0.009	1.2E-11	trans
1520	NRP2	O60462	Neuropilin-2	rs2289023	205986321	2	A	G	0.773	0.202	0.009	1.1E-120	cis
1520	NRP2	O60462	Neuropilin-2	rs17285324	46482980	3	A	T	0.825	0.075	0.009	2.1E-15	trans
1520	NRP2	O60462	Neuropilin-2	rs13081028	52555316	3	A	G	0.557	0.059	0.007	1.7E-16	trans
1520	NRP2	O60462	Neuropilin-2	rs55818129	39425092	5	T	G	0.882	0.076	0.011	5.9E-12	trans
1520	NRP2	O60462	Neuropilin-2	rs2844572	31335575	6	A	C	0.657	0.078	0.008	3.0E-25	trans
1520	NRP2	O60462	Neuropilin-2	rs56287412	17978828	7	C	T	0.511	0.062	0.007	6.9E-18	trans
1520	NRP2	O60462	Neuropilin-2	rs2205513	38490844	X	A	G	0.688	0.049	0.006	8.0E-15	trans
1521	NRTN	Q99748	Neurturin	rs36055559	5799433	19	G	A	0.828	0.076	0.011	1.0E-12	cis
1522	NSFL1C	Q9UNZ2	NSFL1 cofactor p47	rs1354034	56849749	3	C	T	0.604	0.063	0.007	3.4E-17	trans
1522	NSFL1C	Q9UNZ2	NSFL1 cofactor p47	rs6042675	1485256	20	G	C	0.611	0.080	0.008	3.1E-26	cis
1523	NT5C	Q8TCD5	5'(3')-deoxyribonucleotidase, cytosolic type	rs1354034	56849749	3	C	T	0.604	0.056	0.007	3.2E-15	trans
1523	NT5C	Q8TCD5	5'(3')-deoxyribonucleotidase, cytosolic type	rs7896518	65104500	10	G	A	0.424	0.059	0.007	8.7E-17	trans
1523	NT5C	Q8TCD5	5'(3')-deoxyribonucleotidase, cytosolic type	rs78625720	73140941	17	G	A	0.968	1.539	0.021	0.0E+00	cis
1524	NT5C3A	Q9H0P0	Cytosolic 5'-nucleotidase 3A	rs1354034	56849749	3	C	T	0.604	0.058	0.008	2.8E-14	trans
1524	NT5C3A	Q9H0P0	Cytosolic 5'-nucleotidase 3A	rs2706396	131796809	5	C	A	0.570	0.051	0.008	1.2E-11	trans
1524	NT5C3A	Q9H0P0	Cytosolic 5'-nucleotidase 3A	rs10252880	33110659	7	G	C	0.675	0.109	0.008	8.7E-42	cis
1524	NT5C3A	Q9H0P0	Cytosolic 5'-nucleotidase 3A	rs6993770	106581528	8	A	T	0.713	0.064	0.008	1.3E-14	trans
1524	NT5C3A	Q9H0P0	Cytosolic 5'-nucleotidase 3A	rs7896518	65104500	10	G	A	0.424	0.068	0.008	1.0E-18	trans
1525	NT5E	P21589	5'-nucleotidase	rs1497406	16505320	1	G	A	0.580	0.060	0.007	1.8E-19	trans
1525	NT5E	P21589	5'-nucleotidase	rs7429191	149113080	3	A	G	0.707	0.054	0.007	5.6E-14	trans
1525	NT5E	P21589	5'-nucleotidase	rs13107325	103188709	4	C	T	0.925	0.094	0.013	7.3E-14	trans
1525	NT5E	P21589	5'-nucleotidase	rs4835265	146821410	4	A	C	0.156	0.118	0.009	9.3E-39	trans
1525	NT5E	P21589	5'-nucleotidase	rs10075805	31021358	5	A	G	0.725	0.055	0.007	8.0E-14	trans
1525	NT5E	P21589	5'-nucleotidase	rs2229523	86199233	6	A	G	0.340	0.587	0.008	0.0E+00	cis
1525	NT5E	P21589	5'-nucleotidase	rs754466	79680434	10	T	A	0.253	0.051	0.008	1.5E-11	trans
1525	NT5E	P21589	5'-nucleotidase	rs12373325	56087648	18	T	C	0.202	0.078	0.008	1.4E-21	trans
1525	NT5E	P21589	5'-nucleotidase	rs601338	49206674	19	A	G	0.509	0.054	0.007	2.3E-16	trans
1525	NT5E	P21589	5'-nucleotidase	rs189448562	3705115	20	C	G	0.011	0.324	0.032	1.5E-24	trans
1525	NT5E	P21589	5'-nucleotidase	rs6076335	25314864	20	G	T	0.481	0.050	0.007	2.5E-14	trans
1526	NTF3	P20783	Neurotrophin-3	rs13402475	3639909	2	C	G	0.183	0.086	0.010	2.3E-18	trans
1526	NTF3	P20783	Neurotrophin-3	rs10868235	87493755	9	C	T	0.474	0.085	0.008	5.7E-30	trans
1526	NTF3	P20783	Neurotrophin-3	rs117126605	88792227	15	T	C	0.982	0.356	0.029	1.5E-35	trans
1526	NTF3	P20783	Neurotrophin-3	rs12454720	72123370	18	T	G	0.529	0.087	0.008	1.5E-30	trans
1527	NTF4	P34130	Neurotrophin-4	rs6562775	74193515	13	A	G	0.504	0.053	0.008	4.9E-12	trans
1528	NTproBNP	P16860	N-terminal prohormone of brain natriuretic peptide	rs198379	11915467	1	C	T	0.413	0.261	0.007	2.5E-294	cis
1528	NTproBNP	P16860	N-terminal prohormone of brain natriuretic peptide	rs3800560	128461094	7	T	C	0.085	0.087	0.012	1.3E-12	trans
1528	NTproBNP	P16860	N-terminal prohormone of brain natriuretic peptide	rs72671655	106347897	8	A	T	0.038	0.144	0.018	7.9E-16	trans
1528	NTproBNP	P16860	N-terminal prohormone of brain natriuretic peptide	rs2234962	121429633	10	C	T	0.216	0.087	0.008	1.7E-25	trans
1528	NTproBNP	P16860	N-terminal prohormone of brain natriuretic peptide	rs17064679	55953604	18	T	C	0.606	0.048	0.007	8.0E-12	trans
1529	NTRK2	Q16620	BDNF/NT-3 growth factors receptor	rs4665972	27598097	2	C	T	0.608	0.058	0.008	1.5E-14	trans
1529	NTRK2	Q16620	BDNF/NT-3 growth factors receptor	rs13107325	103188709	4	T	C	0.075	0.099	0.014	1.8E-12	trans
1529	NTRK2	Q16620	BDNF/NT-3 growth factors receptor	rs77562929	87285367	9	T	G	0.007	0.495	0.046	2.0E-26	cis
1529	NTRK2	Q16620	BDNF/NT-3 growth factors receptor	rs56278466	17875857	10	G	T	0.662	0.084	0.008	3.0E-27	trans
1529	NTRK2	Q16620	BDNF/NT-3 growth factors receptor	rs2298475	126278203	11	T	C	0.925	0.101	0.014	2.4E-13	trans
1529	NTRK2	Q16620	BDNF/NT-3 growth factors receptor	rs186021206	7069412	17	A	G	0.006	0.516	0.050	8.8E-25	trans
1529	NTRK2	Q16620	BDNF/NT-3 growth factors receptor	rs2008174	39860130	22	C	T	0.260	0.068	0.009	1.7E-15	trans
1530	NTRK3	Q16288	NT-3 growth factor receptor	rs34324830	93087855	1	C	T	0.886	0.098	0.011	9.3E-18	trans
1530	NTRK3	Q16288	NT-3 growth factor receptor	rs7340151	9665450	2	T	C	0.672	0.056	0.008	8.3E-13	trans
1530	NTRK3	Q16288	NT-3 growth factor receptor	rs35887873	126219396	11	C	T	0.228	0.059	0.009	5.3E-12	trans
1530	NTRK3	Q16288	NT-3 growth factor receptor	rs79445108	104193024	14	G	A	0.932	0.160	0.015	6.5E-28	trans
1530	NTRK3	Q16288	NT-3 growth factor receptor	rs28735437	88514855	15	C	G	0.140	0.273	0.011	8.9E-146	cis
1530	NTRK3	Q16288	NT-3 growth factor receptor	rs200489612	7106378	17	A	G	0.005	0.524	0.054	5.5E-22	trans
1530	NTRK3	Q16288	NT-3 growth factor receptor	rs914187	42619749	21	T	C	0.400	0.112	0.007	4.6E-51	trans
1530	NTRK3	Q16288	NT-3 growth factor receptor	rs2413593	39872127	22	G	A	0.495	0.054	0.007	2.5E-13	trans
1531	NUB1	Q9Y5A7	NEDD8 ultimate buster 1	rs1354034	56849749	3	C	T	0.604	0.084	0.008	3.4E-28	trans
1531	NUB1	Q9Y5A7	NEDD8 ultimate buster 1	rs342298	106373646	7	C	T	0.545	0.057	0.008	4.2E-14	trans
1531	NUB1	Q9Y5A7	NEDD8 ultimate buster 1	rs113920358	151038887	7	C	T	0.871	0.266	0.012	1.2E-116	cis
1531	NUB1	Q9Y5A7	NEDD8 ultimate buster 1	rs11502185	180258	11	C	T	0.260	0.072	0.010	4.2E-14	trans
1532	NUBP1	P53384	Cytosolic Fe-S cluster assembly factor NUBP1	rs71534539	32512754	6	G	A	0.535	0.084	0.008	1.5E-23	trans
1533	NUCB2	P80303	Nucleobindin-2	rs2524096	31236467	6	T	G	0.420	0.051	0.007	2.8E-12	trans
1533	NUCB2	P80303	Nucleobindin-2	rs6993770	106581528	8	A	T	0.714	0.077	0.008	1.5E-22	trans
1533	NUCB2	P80303	Nucleobindin-2	rs214068	17305650	11	T	C	0.423	0.406	0.008	0.0E+00	cis
1533	NUCB2	P80303	Nucleobindin-2	rs892090	55539072	19	G	T	0.834	0.082	0.010	3.0E-17	trans
1534	NUDC	Q9Y266	Nuclear migration protein nudC	rs1354034	56849749	3	C	T	0.604	0.079	0.008	1.6E-25	trans
1534	NUDC	Q9Y266	Nuclear migration protein nudC	rs342298	106373646	7	C	T	0.545	0.063	0.007	2.5E-17	trans
1535	NUDT16	Q96DE0	U8 snoRNA-decapping enzyme	rs1354034	56849749	3	C	T	0.605	0.065	0.008	2.8E-17	trans
1535	NUDT16	Q96DE0	U8 snoRNA-decapping enzyme	rs150437674	131100956	3	C	G	0.989	1.211	0.036	2.3E-251	cis
1535	NUDT16	Q96DE0	U8 snoRNA-decapping enzyme	rs10733789	64948684	10	C	T	0.313	0.069	0.008	1.8E-17	trans
1536	NUDT2	P50583	Bis(5'-nucleosyl)-tetraphosphatase [asymmetrical]	rs7848667	34340949	9	G	A	0.157	0.332	0.010	1.5E-224	cis
1537	NUDT5	Q9UKK9	ADP-sugar pyrophosphatase	rs1354034	56849749	3	C	T	0.604	0.064	0.008	3.2E-17	trans
1537	NUDT5	Q9UKK9	ADP-sugar pyrophosphatase	rs10508438	12244079	10	A	G	0.336	0.061	0.008	1.1E-14	cis
1538	NUMB	P49757	Protein numb homolog	rs1354034	56849749	3	C	T	0.604	0.081	0.008	2.6E-26	trans
1538	NUMB	P49757	Protein numb homolog	rs6993770	106581528	8	A	T	0.713	0.058	0.008	3.7E-12	trans
1538	NUMB	P49757	Protein numb homolog	rs11502185	180258	11	C	T	0.260	0.070	0.010	2.7E-13	trans
1538	NUMB	P49757	Protein numb homolog	rs7141139	73887890	14	T	G	0.711	0.084	0.008	7.6E-24	cis
1539	NXPH3	O95157	Neurexophilin-3	rs60191984	9307896	1	T	TTGGG	0.599	0.068	0.008	3.6E-19	trans
1539	NXPH3	O95157	Neurexophilin-3	rs39602	96364454	5	C	G	0.445	0.086	0.007	4.8E-31	trans
1539	NXPH3	O95157	Neurexophilin-3	rs1558918	130029507	7	A	T	0.328	0.062	0.008	1.3E-15	trans
1539	NXPH3	O95157	Neurexophilin-3	rs35887873	126219396	11	C	T	0.228	0.078	0.009	4.6E-19	trans
1539	NXPH3	O95157	Neurexophilin-3	rs186021206	7069412	17	A	G	0.006	0.574	0.051	4.1E-29	trans
1540	OBP2B	Q9NPH6	Odorant-binding protein 2b	rs144623173	9262341	1	T	TA	0.588	0.054	0.005	3.7E-23	trans
1540	OBP2B	Q9NPH6	Odorant-binding protein 2b	rs61685000	202181185	1	G	C	0.590	0.037	0.005	7.3E-12	trans
1540	OBP2B	Q9NPH6	Odorant-binding protein 2b	rs12621139	119632724	2	A	G	0.182	0.070	0.007	6.8E-24	trans
1540	OBP2B	Q9NPH6	Odorant-binding protein 2b	rs463725	2497642	5	C	G	0.424	0.036	0.005	1.5E-11	trans
1540	OBP2B	Q9NPH6	Odorant-binding protein 2b	rs34320230	97785500	7	C	T	0.465	0.060	0.005	3.2E-29	trans
1540	OBP2B	Q9NPH6	Odorant-binding protein 2b	rs1825146	32085677	8	A	C	0.427	0.048	0.005	2.5E-19	trans
1540	OBP2B	Q9NPH6	Odorant-binding protein 2b	rs4962104	136087047	9	C	T	0.800	0.704	0.008	0.0E+00	cis
1540	OBP2B	Q9NPH6	Odorant-binding protein 2b	rs11516994	90066236	10	A	C	0.356	0.042	0.006	3.5E-14	trans
1540	OBP2B	Q9NPH6	Odorant-binding protein 2b	rs9543287	73814441	13	C	G	0.677	0.047	0.006	3.3E-17	trans
1540	OBP2B	Q9NPH6	Odorant-binding protein 2b	rs8047587	53798622	16	G	T	0.562	0.040	0.005	4.3E-14	trans
1540	OBP2B	Q9NPH6	Odorant-binding protein 2b	rs12986064	54755133	19	T	C	0.492	0.048	0.005	8.8E-19	trans
1540	OBP2B	Q9NPH6	Odorant-binding protein 2b	rs11911496	41049472	21	A	G	0.514	0.040	0.005	2.0E-14	trans
1540	OBP2B	Q9NPH6	Odorant-binding protein 2b	rs1978060	19749525	22	G	A	0.614	0.040	0.005	1.8E-13	trans
1541	OCLN	Q16625	Occludin	rs2583707	68757774	5	T	C	0.976	0.473	0.024	4.6E-86	cis
1542	ODAM	A1E959	Odontogenic ameloblast-associated protein	rs76153614	71061318	4	T	C	0.948	0.587	0.017	1.3E-249	cis
1543	OGA	O60502	Protein O-GlcNAcase	rs342293	106372219	7	C	G	0.540	0.052	0.007	2.6E-12	trans
1543	OGA	O60502	Protein O-GlcNAcase	rs6993770	106581528	8	A	T	0.713	0.070	0.008	1.1E-17	trans
1543	OGA	O60502	Protein O-GlcNAcase	rs3781299	103590594	10	C	A	0.709	0.134	0.008	1.6E-60	cis
1544	OGFR	Q9NZT2	Opioid growth factor receptor	rs6993770	106581528	8	A	T	0.714	0.055	0.008	9.6E-12	trans

1545	OGN	P20774	Mimecan	rs13146355	77412140	4	A	G	0.457	0.043	0.006	4.2E-12	trans
1545	OGN	P20774	Mimecan	rs7026361	95138564	9	T	G	0.369	0.597	0.007	0.0E+00	cis
1545	OGN	P20774	Mimecan	rs145678014	32927778	11	G	T	0.955	0.112	0.015	2.5E-13	trans
1546	OLFM4	Q6UX06	Olfactomedin-4	rs28498283	43360065	2	T	A	0.255	0.074	0.008	7.9E-21	trans
1546	OLFM4	Q6UX06	Olfactomedin-4	rs72606404	57841213	4	G	C	0.816	0.113	0.009	6.5E-38	trans
1546	OLFM4	Q6UX06	Olfactomedin-4	rs1870026	50258206	7	A	G	0.060	0.096	0.014	1.4E-11	trans
1546	OLFM4	Q6UX06	Olfactomedin-4	rs457420	136909261	9	T	C	0.386	0.063	0.007	1.7E-19	trans
1546	OLFM4	Q6UX06	Olfactomedin-4	rs4917766	99097191	10	A	G	0.597	0.069	0.007	1.4E-23	trans
1546	OLFM4	Q6UX06	Olfactomedin-4	rs17552047	53602462	13	G	A	0.724	0.859	0.009	0.0E+00	cis
1546	OLFM4	Q6UX06	Olfactomedin-4	rs1045929	38175426	17	T	C	0.388	0.057	0.007	3.5E-16	trans
1546	OLFM4	Q6UX06	Olfactomedin-4	rs138284624	56385573	17	T	C	0.013	0.248	0.030	7.9E-17	trans
1546	OLFM4	Q6UX06	Olfactomedin-4	rs10420726	5007681	19	T	C	0.227	0.121	0.008	2.6E-50	trans
1546	OLFM4	Q6UX06	Olfactomedin-4	rs9305635	39582227	21	C	G	0.669	0.054	0.007	3.7E-14	trans
1547	OLR1	P78380	Oxidized low-density lipoprotein receptor 1	rs61740470	46491985	3	T	C	0.010	0.281	0.037	2.5E-14	trans
1547	OLR1	P78380	Oxidized low-density lipoprotein receptor 1	rs2871960	141121814	3	A	C	0.555	0.103	0.007	2.0E-43	trans
1547	OLR1	P78380	Oxidized low-density lipoprotein receptor 1	rs45489595	47291635	11	C	T	0.887	0.162	0.012	4.3E-43	trans
1547	OLR1	P78380	Oxidized low-density lipoprotein receptor 1	rs2511241	72945341	11	T	C	0.930	0.200	0.015	8.4E-43	trans
1547	OLR1	P78380	Oxidized low-density lipoprotein receptor 1	rs587862	128595173	11	G	A	0.270	0.059	0.008	4.3E-12	trans
1547	OLR1	P78380	Oxidized low-density lipoprotein receptor 1	rs11871747	38177282	17	C	T	0.394	0.067	0.008	9.5E-19	trans
1548	OMD	Q99983	Osteomodulin	rs13303	52558008	3	C	T	0.560	0.097	0.007	3.2E-41	trans
1548	OMD	Q99983	Osteomodulin	rs4253281	187164349	4	G	A	0.487	0.061	0.007	3.0E-17	trans
1548	OMD	Q99983	Osteomodulin	rs704	26694861	17	G	A	0.527	0.088	0.007	1.4E-34	trans
1548	OMD	Q99983	Osteomodulin	rs738408	44324730	22	T	C	0.217	0.062	0.009	5.8E-13	trans
1549	OMG	P23515	Oligodendrocyte-myelin glycoprotein	rs112168180	126394937	5	C	T	0.972	0.163	0.022	1.9E-13	trans
1549	OMG	P23515	Oligodendrocyte-myelin glycoprotein	rs6457541	32285348	6	G	C	0.342	0.061	0.008	1.7E-15	trans
1549	OMG	P23515	Oligodendrocyte-myelin glycoprotein	rs3757724	20395067	7	T	C	0.061	0.182	0.015	1.6E-32	trans
1549	OMG	P23515	Oligodendrocyte-myelin glycoprotein	rs35107030	72951095	7	G	C	0.044	0.180	0.018	2.1E-23	trans
1549	OMG	P23515	Oligodendrocyte-myelin glycoprotein	rs56278466	17875857	10	G	T	0.661	0.115	0.008	1.1E-50	trans
1549	OMG	P23515	Oligodendrocyte-myelin glycoprotein	rs200489612	7106378	17	A	G	0.005	0.532	0.055	4.9E-22	trans
1549	OMG	P23515	Oligodendrocyte-myelin glycoprotein	rs72813607	29461525	17	G	A	0.860	0.125	0.011	1.5E-32	cis
1549	OMG	P23515	Oligodendrocyte-myelin glycoprotein	rs17196752	48887268	20	C	T	0.811	0.069	0.009	2.1E-13	trans
1550	OMP	P47874	Olfactory marker protein	rs2233546	76813961	11	C	T	0.947	0.387	0.017	3.1E-110	cis
1551	OPHN1	O60890	Oligophrenin-1	rs1354034	56849749	3	C	T	0.604	0.062	0.008	2.2E-16	trans
1551	OPHN1	O60890	Oligophrenin-1	rs145954821	67663257	X	C	T	0.983	0.252	0.026	1.5E-21	cis
1552	OPLAH	O14841	5-oxoprolinase	rs6993770	106581528	8	A	T	0.713	0.087	0.008	3.1E-25	trans
1552	OPLAH	O14841	5-oxoprolinase	rs55646585	144999621	8	T	C	0.414	0.187	0.008	1.5E-131	cis
1553	OPTC	Q9UBM4	Opticin	rs139024490	203468792	1	T	C	0.996	1.725	0.054	2.6E-220	cis
1553	OPTC	Q9UBM4	Opticin	rs78011579	88698039	2	G	A	0.969	0.243	0.022	3.8E-28	trans
1553	OPTC	Q9UBM4	Opticin	rs67449961	32497550	6	C	G	0.818	0.089	0.010	1.4E-19	trans
1553	OPTC	Q9UBM4	Opticin	rs2108790	7255479	7	T	C	0.607	0.052	0.007	1.2E-12	trans
1553	OPTC	Q9UBM4	Opticin	rs55851518	79277564	9	G	A	0.944	0.158	0.016	2.3E-24	trans
1553	OPTC	Q9UBM4	Opticin	rs10750291	124706625	11	G	A	0.675	0.073	0.008	3.1E-21	trans
1553	OPTC	Q9UBM4	Opticin	rs4436712	60808002	14	T	G	0.385	0.060	0.007	4.1E-16	trans
1553	OPTC	Q9UBM4	Opticin	rs35731193	106506348	14	G	GA	0.555	0.054	0.008	8.1E-13	trans
1553	OPTC	Q9UBM4	Opticin	rs186021206	7069412	17	A	G	0.006	0.657	0.050	5.0E-40	trans
1554	ORM1	P02763	Alpha-1-acid glycoprotein 1	rs2017857	148988399	4	G	C	0.749	0.059	0.009	6.5E-12	trans
1554	ORM1	P02763	Alpha-1-acid glycoprotein 1	rs10982156	117088064	9	A	T	0.069	0.741	0.016	0.0E+00	cis
1554	ORM1	P02763	Alpha-1-acid glycoprotein 1	rs7896518	65104500	10	A	G	0.576	0.059	0.007	3.4E-15	trans
1555	OSCAR	Q8IYS5	Osteoclast-associated immunoglobulin-like receptor	rs13063578	47087837	3	T	A	0.602	0.045	0.007	7.2E-12	trans
1555	OSCAR	Q8IYS5	Osteoclast-associated immunoglobulin-like receptor	rs73401939	29740346	6	G	A	0.006	0.618	0.043	2.5E-46	trans
1555	OSCAR	Q8IYS5	Osteoclast-associated immunoglobulin-like receptor	rs12544501	130605931	8	T	C	0.431	0.060	0.007	5.1E-20	trans
1555	OSCAR	Q8IYS5	Osteoclast-associated immunoglobulin-like receptor	rs76428106	28604007	13	C	T	0.013	0.348	0.030	7.3E-32	trans
1555	OSCAR	Q8IYS5	Osteoclast-associated immunoglobulin-like receptor	rs186021206	7069412	17	A	G	0.006	0.624	0.044	4.8E-45	trans
1555	OSCAR	Q8IYS5	Osteoclast-associated immunoglobulin-like receptor	rs2305482	38140927	17	A	C	0.461	0.044	0.006	6.2E-12	trans
1555	OSCAR	Q8IYS5	Osteoclast-associated immunoglobulin-like receptor	rs2659005	79218714	17	T	C	0.441	0.044	0.007	1.4E-11	trans
1555	OSCAR	Q8IYS5	Osteoclast-associated immunoglobulin-like receptor	rs4442925	54554950	19	T	C	0.449	0.521	0.008	0.0E+00	cis
1556	OSM	P13725	Oncostatin-M	rs28498283	43360065	2	T	A	0.255	0.066	0.009	3.4E-14	trans
1556	OSM	P13725	Oncostatin-M	rs9325122	148202936	5	C	T	0.448	0.051	0.007	7.5E-12	trans
1556	OSM	P13725	Oncostatin-M	rs12264390	959111	10	C	T	0.240	0.060	0.009	3.7E-12	trans
1556	OSM	P13725	Oncostatin-M	rs2511241	72945341	11	T	C	0.929	0.362	0.015	4.3E-136	trans
1556	OSM	P13725	Oncostatin-M	rs10483987	88419242	14	T	C	0.847	0.079	0.010	4.4E-14	trans
1556	OSM	P13725	Oncostatin-M	rs11871747	38177282	17	C	T	0.393	0.127	0.008	1.7E-62	trans
1556	OSM	P13725	Oncostatin-M	rs4823082	30600105	22	G	C	0.977	0.188	0.025	3.0E-14	cis
1556	OSM	P13725	Oncostatin-M	rs139113470	49369120	X	C	G	0.033	0.154	0.018	6.6E-18	trans
1557	OSMR	Q99650	Oncostatin-M-specific receptor subunit beta	rs2228145	154426970	1	A	C	0.594	0.050	0.006	1.7E-14	trans
1557	OSMR	Q99650	Oncostatin-M-specific receptor subunit beta	rs9292723	38852572	5	C	T	0.723	0.596	0.008	0.0E+00	cis
1557	OSMR	Q99650	Oncostatin-M-specific receptor subunit beta	rs8176693	136137657	9	T	C	0.061	0.100	0.013	3.8E-14	trans
1557	OSMR	Q99650	Oncostatin-M-specific receptor subunit beta	rs56187480	63789479	15	A	G	0.343	0.063	0.007	5.2E-21	trans
1557	OSMR	Q99650	Oncostatin-M-specific receptor subunit beta	rs186021206	7069412	17	A	G	0.006	0.595	0.044	3.4E-42	trans
1558	OSTN	P61366	Osteocrin	rs34711532	190849577	3	C	CG	0.271	0.106	0.009	1.1E-33	cis
1559	OTOA	Q7RTW8	Otoancorin	rs8055493	21771016	16	A	G	0.575	0.057	0.008	4.1E-13	cis
1560	OTUD6B	Q8N6M0	Deubiquitinase OTUD6B	rs1354034	56849749	3	C	T	0.605	0.066	0.008	2.1E-18	trans
1560	OTUD6B	Q8N6M0	Deubiquitinase OTUD6B	rs342298	106373646	7	C	T	0.545	0.051	0.007	1.5E-11	trans
1561	OTUD7B	Q6GQQ9	OTU domain-containing protein 7B	rs1354034	56849749	3	C	T	0.604	0.075	0.008	1.2E-22	trans
1561	OTUD7B	Q6GQQ9	OTU domain-containing protein 7B	rs6993770	106581528	8	A	T	0.714	0.065	0.008	3.0E-15	trans
1562	OXCT1	P55809	Succinyl-CoA:3-ketoacid coenzyme A transferase 1, mitochondrial	rs10075801	131677642	5	A	G	0.512	0.052	0.008	1.3E-11	trans
1562	OXCT1	P55809	Succinyl-CoA:3-ketoacid coenzyme A transferase 1, mitochondrial	rs342298	106373646	7	C	T	0.545	0.059	0.008	8.6E-15	trans
1562	OXCT1	P55809	Succinyl-CoA:3-ketoacid coenzyme A transferase 1, mitochondrial	rs6993770	106581528	8	A	T	0.713	0.064	0.008	2.9E-14	trans
1562	OXCT1	P55809	Succinyl-CoA:3-ketoacid coenzyme A transferase 1, mitochondrial	rs7896518	65104500	10	G	A	0.424	0.054	0.008	6.2E-12	trans
1563	OXT	P01178	Oxytocin-neurophysin 1	rs10929757	11702661	2	C	A	0.576	0.056	0.007	2.7E-15	trans
1563	OXT	P01178	Oxytocin-neurophysin 1	rs11127048	27752463	2	G	A	0.382	0.056	0.007	1.3E-14	trans
1563	OXT	P01178	Oxytocin-neurophysin 1	rs4895864	130364482	6	T	C	0.574	0.049	0.007	2.7E-12	trans
1563	OXT	P01178	Oxytocin-neurophysin 1	rs13229619	73030175	7	G	A	0.872	0.098	0.010	4.9E-21	trans
1563	OXT	P01178	Oxytocin-neurophysin 1	rs45446698	99332948	7	T	G	0.958	0.130	0.017	5.5E-14	trans
1563	OXT	P01178	Oxytocin-neurophysin 1	rs112875651	126506694	8	G	A	0.609	0.067	0.007	2.4E-20	trans
1563	OXT	P01178	Oxytocin-neurophysin 1	rs9414801	65149089	10	G	A	0.467	0.047	0.007	1.4E-11	trans
1563	OXT	P01178	Oxytocin-neurophysin 1	rs4782568	83980529	16	G	C	0.451	0.056	0.007	7.0E-16	trans
1563	OXT	P01178	Oxytocin-neurophysin 1	rs877172	3049890	20	G	T	0.312	0.627	0.009	0.0E+00	cis
1563	OXT	P01178	Oxytocin-neurophysin 1	rs1800961	43042364	20	C	T	0.968	0.142	0.021	1.2E-11	trans
1564	P4HB	P07237	Protein disulfide-isomerase	rs3910516	216303053	2	G	A	0.737	0.065	0.009	2.9E-14	trans
1564	P4HB	P07237	Protein disulfide-isomerase	rs1354034	56849749	3	C	T	0.604	0.058	0.008	4.7E-14	trans
1564	P4HB	P07237	Protein disulfide-isomerase	rs6993770	106581528	8	A	T	0.714	0.058	0.008	3.1E-12	trans
1564	P4HB	P07237	Protein disulfide-isomerase	rs112635299	94838142	14	T	G	0.021	0.191	0.026	2.1E-13	trans
1564	P4HB	P07237	Protein disulfide-isomerase	rs113708033	79835930	17	T	G	0.192	0.087	0.010	1.6E-19	cis
1565	PACS2	Q86VP3	Phosphofurin acidic cluster sorting protein 2	rs13412535	224874874	2	A	G	0.231	0.085	0.009	2.1E-21	trans
1565	PACS2	Q86VP3	Phosphofurin acidic cluster sorting protein 2	rs1354034	56849749	3	C	T	0.604	0.076	0.008	5.3E-23	trans
1565	PACS2	Q86VP3	Phosphofurin acidic cluster sorting protein 2	rs7149013	105759840	14	C	T	0.295	0.178	0.008	8.0E-103	cis
1566	PADI2	Q9Y2J8	Protein-arginine deiminase type-2	rs2235913	17425077	1	A	G	0.618	0.122	0.008	9.0E-54	cis
1567	PADI4	Q9UM07	Protein-arginine deiminase type-4	rs2240336	17674402	1	C	T	0.585	0.257	0.008	8.5E-249	cis
1568	PAEP	P09466	Glycodelin	rs9409964	138471928	9	A	G	0.188	0.871	0.009	0.0E+00	cis
1568	PAEP	P09466	Glycodelin	rs4805162	36674305	19	A	G	0.476	0.047	0.006	2.0E-17	trans
1569	PAFAH1B3	Q15102	Platelet-activating factor acetylhydrolase IB subunit alpha1	rs71534539	32512754	6	G	A	0.536	0.137	0.008	7.6E-60	trans
1569	PAFAH1B3	Q15102	Platelet-activating factor acetylhydrolase IB subunit alpha1	rs10957979	81289787	8	G	A	0.648	0.075	0.008	4.2E-21	trans
1570	PAG1	Q9NWQ8	Phosphoprotein associated with glycosphingolipid-enriched microdomains 1	rs1354034	56849749	3	C	T	0.604	0.054	0.008	4.4E-12	trans
1570	PAG1	Q9NWQ8	Phosphoprotein associated with glycosphingolipid-enriched microdomains 1	rs4436084	82038006	8	T	C	0.650	0.078	0.008	1.7E-22	cis
1570	PAG1	Q9NWQ8	Phosphoprotein associated with glycosphingolipid-enriched microdomains 1	rs1532085	58683366	15	G	A	0.617	0.059	0.008	9.7E-14	trans
1570	PAG1	Q9NWQ8	Phosphoprotein associated with glycosphingolipid-enriched microdomains 1	rs247617	56990716	16	C	A	0.675	0.081	0.008	5.1E-23	trans
1570	PAG1	Q9NWQ8	Phosphoprotein associated with glycosphingolipid-enriched microdomains 1	rs7208422	76130575	17	A	T	0.522	0.080	0.008	1.2E-25	trans
1571	PAGR1	Q9BTK6	PAXIP1-associated glutamate-rich protein 1	rs13257521	9031995	8	G	C	0.210	0.093	0.009	2.4E-23	trans
1572	PALM	O75781	Paralemmin-1	rs150394890	223864380	1	T	G	0.009	0.288	0.040	4.8E-13	trans
1572	PALM	O75781	Paralemmin-1	rs12610821	719295	19	G	C	0.057	0.319	0.016	5.9E-88	cis
1572	PALM	O75781	Paralemmin-1	rs429358	45411941	19	C	T	0.156	0.197	0.010	9.9E-84	trans
1573	PALM2	Q8IXS6	Paralemmin-2	rs1023005	112490295	9	T	C	0.231	0.234	0.009	2.0E-161	cis
1574	PAM	P19021	Peptidyl-glycine alpha-amidating monooxygenase	rs149802978	102346866	5	C	G	0.946	1.099	0.017	0.0E+00	cis
1574	PAM	P19021	Peptidyl-glycine alpha-amidating monooxygenase	rs75901433	32499150	6	G	A	0.558	0.051	0.007	6.9E-14	trans
1574	PAM	P19021	Peptidyl-glycine alpha-amidating monooxygenase	rs56278466	17875857	10	G	T	0.662	0.108	0.007	1.2E-53	trans
1574	PAM	P19021	Peptidyl-glycine alpha-amidating monooxygenase	rs12975366	54759361	19	T	C	0.605	0.073	0.007	6.1E-27	trans
1575	PAMR1	Q6UXH9	Inactive serine protease PAMR1	rs1260326	27730940	2	T	C	0.392	0.059	0.007	9.4E-18	trans
1575	PAMR1	Q6UXH9	Inactive serine protease PAMR1	rs1150753	32059867	6	A	G	0.869	0.222	0.010	2.0E-106	trans
1575	PAMR1	Q6UXH9	Inactive serine protease PAMR1	rs61736408	35456061	11	T	C	0.843	0.476	0.010	0.0E+00	cis
1575	PAMR1	Q6UXH9	Inactive serine protease PAMR1	rs4055121	126232337	11	C	T	0.865	0.073	0.011	6.1E-12	trans
1576	PAPPA	Q13219	Pappalysin-1	rs967968	61656056	2	G	C	0.511	0.077	0.007	2.1E-26	trans
1576	PAPPA	Q13219	Pappalysin-1	rs4328821	128316435	3	A	G	0.893	0.119	0.012	1.6E-24	trans
1576	PAPPA	Q13219	Pappalysin-1	rs13107325	103188709	4	T	C	0.075	0.103	0.014	6.9E-14	trans
1576	PAPPA	Q13219	Pappalysin-1	rs7808353	2446680	7	A	T	0.833	0.084	0.010	6.9E-18	trans
1576	PAPPA	Q13219	Pappalysin-1	rs117448041	118342600	9	C	T	0.020	0.187	0.026	1.3E-12	cis
1576	PAPPA	Q13219	Pappalysin-1	rs13448	46695414	11	T	C	0.735	0.058	0.008	2.7E-12	trans
1576	PAPPA	Q13219	Pappalysin-1	rs147496186	49354822	11	G	C	0.958	0.129	0.018	1.1E-12	trans
1576	PAPPA	Q13219	Pappalysin-1	rs550729	57159376	11	C	T	0.729	0.263	0.008	2.6E-224	trans
1576	PAPPA	Q13219	Pappalysin-1	rs74035509	88567333	16	T	C	0.078	0.107	0.014	2.2E-15	trans
1576	PAPPA	Q13219	Pappalysin-1	rs35617692	56272501	17	A	C	0.011	0.299	0.034	4.4E-18	trans
1577	PARD3	Q8TEW0	Partitioning defective 3 homolog	rs9418120	35060501	10	A	G	0.260	0.076	0.009	2.8E-18	cis
1578	PARK7	Q99497	Parkinson disease protein 7	rs3766606	8022197	1	T	G	0.174	0.209	0.010	2.3E-100	cis
1578	PARK7	Q99497	Parkinson disease protein 7	rs1354034	56849749	3	C	T	0.604	0.057	0.008	2.7E-14	trans
1579	PARP1	P09874	Poly [ADP-ribose] polymerase 1	rs1433574	226609339	1	A	C	0.839	0.159	0.010	1.1E-55	cis
1579	PARP1	P09874	Poly [ADP-ribose] polymerase 1	rs4632248	54324995	19	T	G	0.209	0.149	0.009	3.9E-60	trans
1580	PAXX	Q9BUH6	Protein PAXX	rs6993770	106581528	8	A	T	0.713	0.059	0.008	8.9E-13	trans
1581	PBLD	P30039	Phenazine biosynthesis-like domain-containing protein	rs61854835	70038894	10	G	A	0.237	0.576	0.009	0.0E+00	cis
1582	PBXIP1	Q96AQ6	Pre-B-cell leukemia transcription factor-interacting protein 1	rs390082	45416831	19	G	T	0.110	0.081	0.012	8.3E-12	trans
1583	PCARE	A6NGG8	Photoreceptor cilium actin regulator	rs141575023	106370370	14	T	C	0.138	0.234	0.011	3.8E-95	trans
1584	PCBD1	P61457	Pterin-4-alpha-carbinolamine dehydratase	rs7041363	117146043	9	C	G	0.510	0.060	0.007	7.1E-16	trans
1584	PCBD1	P61457	Pterin-4-alpha-carbinolamine dehydratase	rs2630336	72648422	10	G	T	0.640	0.181	0.008	4.1E-121	cis
1584	PCBD1	P61457	Pterin-4-alpha-carbinolamine dehydratase	rs10883451	101924418	10	T	C	0.498	0.053	0.007	6.6E-13	trans

1584	PCBD1	P61457	Pterin-4-alpha-carbinolamine dehydratase	rs217184	72105965	16	T	C	0.804	0.073	0.009	2.7E-15	trans
1584	PCBD1	P61457	Pterin-4-alpha-carbinolamine dehydratase	rs58542926	19379549	19	T	C	0.075	0.098	0.014	2.2E-12	trans
1584	PCBD1	P61457	Pterin-4-alpha-carbinolamine dehydratase	rs3747207	44324855	22	A	G	0.215	0.093	0.009	1.2E-25	trans
1585	PCBP2	Q15366	Poly(rC)-binding protein 2	rs1354034	56849749	3	C	T	0.604	0.057	0.008	6.3E-14	trans
1586	PCDH1	Q08174	Protocadherin-1	rs4645358	141260085	5	T	C	0.575	0.081	0.007	9.9E-28	cis
1586	PCDH1	Q08174	Protocadherin-1	rs4077811	95840344	9	G	A	0.144	0.074	0.010	9.5E-13	trans
1586	PCDH1	Q08174	Protocadherin-1	rs8176741	136131461	9	A	G	0.061	0.231	0.015	4.0E-51	trans
1586	PCDH1	Q08174	Protocadherin-1	rs56278466	17875857	10	G	T	0.661	0.054	0.008	3.2E-12	trans
1586	PCDH1	Q08174	Protocadherin-1	rs58534292	106483584	14	T	C	0.350	0.125	0.008	7.9E-58	trans
1586	PCDH1	Q08174	Protocadherin-1	rs2005652	106447273	14	T	C	0.369	0.131	0.008	3.3E-63	trans
1587	PCDH12	Q9NPG4	Protocadherin-12	rs11927405	98402969	3	C	T	0.559	0.096	0.007	2.9E-39	trans
1587	PCDH12	Q9NPG4	Protocadherin-12	rs73186030	122013465	3	T	C	0.128	0.121	0.011	3.8E-29	trans
1587	PCDH12	Q9NPG4	Protocadherin-12	rs164085	141333319	5	A	G	0.510	0.327	0.008	0.0E+00	cis
1587	PCDH12	Q9NPG4	Protocadherin-12	rs2519093	136141870	9	C	T	0.816	0.271	0.009	2.3E-182	trans
1587	PCDH12	Q9NPG4	Protocadherin-12	rs186021206	7069412	17	A	G	0.006	0.361	0.049	2.7E-13	trans
1587	PCDH12	Q9NPG4	Protocadherin-12	rs1135062	45322744	19	A	G	0.709	0.066	0.008	5.4E-17	trans
1588	PCDH17	O14917	Protocadherin-17	rs6789547	58426047	3	C	T	0.827	0.064	0.009	3.7E-12	trans
1588	PCDH17	O14917	Protocadherin-17	rs35467127	32522148	6	T	C	0.201	0.068	0.009	9.5E-15	trans
1588	PCDH17	O14917	Protocadherin-17	rs8176749	136131188	9	T	C	0.061	0.649	0.015	0.0E+00	trans
1588	PCDH17	O14917	Protocadherin-17	rs56278466	17875857	10	G	T	0.662	0.112	0.007	1.0E-52	trans
1588	PCDH17	O14917	Protocadherin-17	rs11603123	126305495	11	A	G	0.034	0.169	0.019	1.4E-18	trans
1588	PCDH17	O14917	Protocadherin-17	rs11148472	60020189	13	C	A	0.391	0.353	0.008	0.0E+00	cis
1588	PCDH17	O14917	Protocadherin-17	rs186021206	7069412	17	A	G	0.006	0.357	0.048	5.5E-14	trans
1588	PCDH17	O14917	Protocadherin-17	rs5757683	39850174	22	G	A	0.255	0.073	0.008	5.8E-20	trans
1589	PCDH7	O60245	Protocadherin-7	rs646776	109818530	1	T	C	0.778	0.099	0.009	2.4E-28	trans
1589	PCDH7	O60245	Protocadherin-7	rs1374657	30837143	4	C	T	0.717	0.116	0.008	9.9E-45	cis
1589	PCDH7	O60245	Protocadherin-7	rs61000956	187164081	4	C	T	0.482	0.060	0.008	4.8E-15	trans
1589	PCDH7	O60245	Protocadherin-7	rs2731672	176842474	5	C	T	0.744	0.066	0.008	9.4E-15	trans
1589	PCDH7	O60245	Protocadherin-7	rs445925	45415640	19	A	G	0.109	0.090	0.012	3.7E-14	trans
1590	PCDH9	Q9HC56	Protocadherin-9	rs9874262	186669005	3	T	C	0.483	0.073	0.007	2.8E-24	trans
1590	PCDH9	Q9HC56	Protocadherin-9	rs28601761	126500031	8	C	G	0.581	0.051	0.007	2.3E-12	trans
1590	PCDH9	Q9HC56	Protocadherin-9	rs10984506	100716140	9	A	T	0.641	0.054	0.007	2.5E-13	trans
1590	PCDH9	Q9HC56	Protocadherin-9	rs73196184	67808183	13	A	G	0.113	0.341	0.012	1.2E-185	cis
1590	PCDH9	Q9HC56	Protocadherin-9	rs186021206	7069412	17	A	G	0.006	0.655	0.049	3.3E-41	trans
1591	PCDHB15	Q9Y5E8	Protocadherin beta-15	rs1622822	231332294	2	T	C	0.145	0.103	0.010	1.8E-24	trans
1591	PCDHB15	Q9Y5E8	Protocadherin beta-15	rs13088318	101242751	3	A	G	0.662	0.051	0.007	8.7E-12	trans
1591	PCDHB15	Q9Y5E8	Protocadherin beta-15	rs2740584	140561768	5	C	T	0.766	0.409	0.009	0.0E+00	cis
1591	PCDHB15	Q9Y5E8	Protocadherin beta-15	rs28367646	31268332	6	C	A	0.240	0.061	0.009	3.7E-12	trans
1591	PCDHB15	Q9Y5E8	Protocadherin beta-15	rs11599284	135044009	10	A	G	0.127	0.115	0.011	1.2E-25	trans
1591	PCDHB15	Q9Y5E8	Protocadherin beta-15	rs260416	58717025	19	G	A	0.102	0.081	0.012	2.3E-12	trans
1592	PCNA	P12004	Proliferating cell nuclear antigen	rs371337007	32493146	6	G	A	0.354	0.062	0.009	1.6E-11	trans
1593	PCOLCE	Q15113	Procollagen C-endopeptidase enhancer 1	rs9801017	100236202	7	A	G	0.624	0.172	0.012	6.7E-47	cis
1594	PCSK7	Q16549	Proprotein convertase subtilisin/kexin type 7	rs4406273	31266090	6	A	G	0.092	0.128	0.013	4.4E-23	trans
1594	PCSK7	Q16549	Proprotein convertase subtilisin/kexin type 7	rs171052	117081763	11	C	G	0.774	0.379	0.009	0.0E+00	cis
1595	PCSK9	Q8NBP7	Proprotein convertase subtilisin/kexin type 9	rs11591147	55505647	1	G	T	0.982	1.153	0.028	0.0E+00	cis
1595	PCSK9	Q8NBP7	Proprotein convertase subtilisin/kexin type 9	rs547186	21269241	2	A	T	0.496	0.072	0.007	1.6E-23	trans
1595	PCSK9	Q8NBP7	Proprotein convertase subtilisin/kexin type 9	rs1260326	27730940	2	T	C	0.392	0.076	0.007	2.6E-25	trans
1595	PCSK9	Q8NBP7	Proprotein convertase subtilisin/kexin type 9	rs2001945	126477978	8	G	C	0.481	0.057	0.007	1.9E-15	trans
1595	PCSK9	Q8NBP7	Proprotein convertase subtilisin/kexin type 9	rs174568	61593816	11	T	C	0.344	0.079	0.007	5.3E-26	trans
1595	PCSK9	Q8NBP7	Proprotein convertase subtilisin/kexin type 9	rs673335	75450576	11	T	C	0.845	0.066	0.010	1.6E-11	trans
1595	PCSK9	Q8NBP7	Proprotein convertase subtilisin/kexin type 9	rs261342	58731153	15	G	C	0.223	0.066	0.009	3.5E-14	trans
1595	PCSK9	Q8NBP7	Proprotein convertase subtilisin/kexin type 9	rs8106503	11196886	19	T	C	0.881	0.094	0.011	1.4E-17	trans
1596	PCYT2	Q99447	Ethanolamine-phosphate cytidylyltransferase	rs1354034	56849749	3	C	T	0.605	0.072	0.008	7.4E-21	trans
1596	PCYT2	Q99447	Ethanolamine-phosphate cytidylyltransferase	rs6993770	106581528	8	A	T	0.713	0.094	0.008	7.1E-30	trans
1597	PDAP1	Q13442	28 kDa heat- and acid-stable phosphoprotein	rs1354034	56849749	3	C	T	0.604	0.073	0.008	2.6E-21	trans
1597	PDAP1	Q13442	28 kDa heat- and acid-stable phosphoprotein	rs342298	106373646	7	C	T	0.545	0.054	0.008	1.1E-12	trans
1598	PDCD1	Q15116	Programmed cell death protein 1	rs37471	44277548	1	T	C	0.352	0.071	0.008	7.5E-21	trans
1598	PDCD1	Q15116	Programmed cell death protein 1	rs2476601	114377568	1	A	G	0.101	0.086	0.012	1.3E-12	trans
1598	PDCD1	Q15116	Programmed cell death protein 1	rs376812565	243182484	2	C	T	0.361	0.062	0.009	1.8E-12	trans
1598	PDCD1	Q15116	Programmed cell death protein 1	rs11888817	62530980	2	C	T	0.642	0.054	0.008	3.7E-12	trans
1598	PDCD1	Q15116	Programmed cell death protein 1	rs11571291	204721132	2	T	C	0.576	0.058	0.008	1.5E-14	trans
1598	PDCD1	Q15116	Programmed cell death protein 1	rs9838308	169751292	3	T	C	0.634	0.054	0.008	2.1E-12	trans
1598	PDCD1	Q15116	Programmed cell death protein 1	rs3131622	31420500	6	G	T	0.450	0.054	0.007	9.1E-14	trans
1598	PDCD1	Q15116	Programmed cell death protein 1	rs12772559	71184356	10	C	G	0.256	0.084	0.008	9.9E-24	trans
1598	PDCD1	Q15116	Programmed cell death protein 1	rs7161799	58770523	15	T	C	0.077	0.129	0.014	5.8E-21	trans
1598	PDCD1	Q15116	Programmed cell death protein 1	rs186021206	7069412	17	A	G	0.006	0.616	0.050	1.2E-34	trans
1598	PDCD1	Q15116	Programmed cell death protein 1	rs2659005	79218714	17	T	C	0.442	0.065	0.007	1.4E-18	trans
1599	PDCD1LG2	Q9BQ51	Programmed cell death 1 ligand 2	rs812490	44271656	1	T	C	0.548	0.058	0.006	1.1E-20	trans
1599	PDCD1LG2	Q9BQ51	Programmed cell death 1 ligand 2	rs10935473	98416900	3	G	T	0.560	0.459	0.007	0.0E+00	trans
1599	PDCD1LG2	Q9BQ51	Programmed cell death 1 ligand 2	rs2381282	5514839	9	C	T	0.439	0.601	0.007	0.0E+00	cis
1599	PDCD1LG2	Q9BQ51	Programmed cell death 1 ligand 2	rs507666	136149399	9	G	A	0.815	0.199	0.009	2.2E-101	trans
1599	PDCD1LG2	Q9BQ51	Programmed cell death 1 ligand 2	rs4055121	126232337	11	C	T	0.865	0.081	0.009	3.9E-19	trans
1599	PDCD1LG2	Q9BQ51	Programmed cell death 1 ligand 2	rs186021206	7069412	17	A	G	0.006	0.973	0.043	5.2E-115	trans
1600	PDCD5	O14737	Programmed cell death protein 5	rs1354034	56849749	3	C	T	0.604	0.065	0.007	4.1E-19	trans
1600	PDCD5	O14737	Programmed cell death protein 5	rs10402931	33072069	19	A	G	0.684	0.384	0.008	0.0E+00	cis
1601	PDCD6	O75340	Programmed cell death protein 6	rs676705	57384297	1	G	A	0.649	0.055	0.006	6.7E-22	trans
1601	PDCD6	O75340	Programmed cell death protein 6	rs9654451	299543	5	C	T	0.872	1.497	0.011	0.0E+00	cis
1601	PDCD6	O75340	Programmed cell death protein 6	rs9495061	138701918	6	C	A	0.941	0.086	0.012	5.7E-14	trans
1601	PDCD6	O75340	Programmed cell death protein 6	rs11438680	65174698	10	GA	G	0.461	0.042	0.005	1.4E-14	trans
1601	PDCD6	O75340	Programmed cell death protein 6	rs112635299	94838142	14	T	G	0.022	0.161	0.019	4.2E-18	trans
1602	PDE5A	O76074	cGMP-specific 3',5'-cyclic phosphodiesterase	rs1354034	56849749	3	C	T	0.604	0.053	0.008	2.9E-12	trans
1602	PDE5A	O76074	cGMP-specific 3',5'-cyclic phosphodiesterase	rs58583086	120556362	4	A	G	0.375	0.158	0.008	4.6E-91	cis
1602	PDE5A	O76074	cGMP-specific 3',5'-cyclic phosphodiesterase	rs7080386	65048306	10	A	C	0.412	0.064	0.008	6.0E-17	trans
1603	PDGFA	P04085	Platelet-derived growth factor subunit A	rs6671171	156871318	1	A	G	0.111	0.087	0.012	1.5E-13	trans
1603	PDGFA	P04085	Platelet-derived growth factor subunit A	rs3811444	248039451	1	C	T	0.668	0.072	0.008	8.5E-20	trans
1603	PDGFA	P04085	Platelet-derived growth factor subunit A	rs7618405	18250509	3	C	A	0.795	0.066	0.009	7.2E-13	trans
1603	PDGFA	P04085	Platelet-derived growth factor subunit A	rs1354034	56849749	3	T	C	0.396	0.121	0.008	5.8E-58	trans
1603	PDGFA	P04085	Platelet-derived growth factor subunit A	rs2278668	122835232	3	T	C	0.407	0.058	0.008	2.2E-14	trans
1603	PDGFA	P04085	Platelet-derived growth factor subunit A	rs4572884	102783351	4	C	T	0.602	0.069	0.008	8.7E-20	trans
1603	PDGFA	P04085	Platelet-derived growth factor subunit A	rs10058074	131686146	5	G	A	0.524	0.053	0.007	7.5E-13	trans
1603	PDGFA	P04085	Platelet-derived growth factor subunit A	rs2516471	31400292	6	G	A	0.936	0.124	0.015	5.6E-16	trans
1603	PDGFA	P04085	Platelet-derived growth factor subunit A	rs146842698	71328273	6	T	TAAATA	0.695	0.059	0.008	7.4E-13	trans
1603	PDGFA	P04085	Platelet-derived growth factor subunit A	rs2759391	163864226	6	A	C	0.657	0.056	0.008	9.1E-13	trans
1603	PDGFA	P04085	Platelet-derived growth factor subunit A	rs144168379	641183	7	C	T	0.997	0.446	0.066	1.5E-11	cis
1603	PDGFA	P04085	Platelet-derived growth factor subunit A	rs6961069	80218961	7	T	C	0.403	0.069	0.008	9.5E-20	trans
1603	PDGFA	P04085	Platelet-derived growth factor subunit A	rs6993770	106581528	8	A	T	0.713	0.180	0.008	5.7E-106	trans
1603	PDGFA	P04085	Platelet-derived growth factor subunit A	rs7080386	65048306	10	A	C	0.412	0.118	0.008	3.0E-55	trans
1603	PDGFA	P04085	Platelet-derived growth factor subunit A	rs34377578	104336426	10	C	A	0.253	0.082	0.009	1.5E-21	trans
1603	PDGFA	P04085	Platelet-derived growth factor subunit A	rs59001897	65160392	15	A	T	0.175	0.079	0.010	7.3E-16	trans
1603	PDGFA	P04085	Platelet-derived growth factor subunit A	rs12445050	81870969	16	T	C	0.138	0.113	0.011	6.9E-26	trans
1603	PDGFA	P04085	Platelet-derived growth factor subunit A	rs1654425	55538980	19	C	T	0.834	0.169	0.010	1.7E-63	trans
1603	PDGFA	P04085	Platelet-derived growth factor subunit A	rs6081565	19287904	20	A	G	0.352	0.073	0.008	4.0E-21	trans
1603	PDGFA	P04085	Platelet-derived growth factor subunit A	rs5967171	99957136	X	C	T	0.333	0.049	0.006	4.8E-14	trans
1604	PDGFB	P01127	Platelet-derived growth factor subunit B	rs75699653	156871995	1	T	C	0.024	0.173	0.025	2.0E-12	trans
1604	PDGFB	P01127	Platelet-derived growth factor subunit B	rs68066031	224880498	2	C	T	0.230	0.229	0.009	2.6E-145	trans
1604	PDGFB	P01127	Platelet-derived growth factor subunit B	rs1354034	56849749	3	T	C	0.396	0.075	0.008	2.6E-23	trans
1604	PDGFB	P01127	Platelet-derived growth factor subunit B	rs4572884	102783351	4	C	T	0.602	0.065	0.008	6.5E-18	trans
1604	PDGFB	P01127	Platelet-derived growth factor subunit B	rs274555	131722951	5	T	C	0.399	0.053	0.008	6.6E-12	trans
1604	PDGFB	P01127	Platelet-derived growth factor subunit B	rs1917342	71349754	6	A	G	0.696	0.060	0.008	1.4E-13	trans
1604	PDGFB	P01127	Platelet-derived growth factor subunit B	rs6961069	80218961	7	T	C	0.403	0.066	0.008	3.2E-18	trans
1604	PDGFB	P01127	Platelet-derived growth factor subunit B	rs6993770	106581528	8	A	T	0.713	0.140	0.008	4.9E-65	trans
1604	PDGFB	P01127	Platelet-derived growth factor subunit B	rs10820606	99192919	9	A	C	0.770	0.077	0.009	1.0E-17	trans
1604	PDGFB	P01127	Platelet-derived growth factor subunit B	rs10761737	65052205	10	C	T	0.413	0.105	0.008	2.0E-43	trans
1604	PDGFB	P01127	Platelet-derived growth factor subunit B	rs34377578	104336426	10	C	A	0.253	0.081	0.009	3.5E-21	trans
1604	PDGFB	P01127	Platelet-derived growth factor subunit B	rs60128101	65160389	15	A	T	0.175	0.071	0.010	8.5E-13	trans
1604	PDGFB	P01127	Platelet-derived growth factor subunit B	rs11639051	101992998	15	A	C	0.295	0.091	0.008	5.5E-29	trans
1604	PDGFB	P01127	Platelet-derived growth factor subunit B	rs12445050	81870969	16	T	C	0.138	0.105	0.011	2.5E-22	trans
1604	PDGFB	P01127	Platelet-derived growth factor subunit B	rs1654425	55538980	19	C	T	0.834	0.165	0.010	5.2E-60	trans
1604	PDGFB	P01127	Platelet-derived growth factor subunit B	rs3790176	19261922	20	A	G	0.343	0.078	0.008	2.1E-23	trans
1604	PDGFB	P01127	Platelet-derived growth factor subunit B	rs5967171	99957136	X	C	T	0.333	0.049	0.007	3.4E-14	trans
1605	PDGFC	Q9NRA1	Platelet-derived growth factor C	rs12499706	157095850	4	C	G	0.725	0.091	0.009	2.7E-26	cis
1605	PDGFC	Q9NRA1	Platelet-derived growth factor C	rs3775298	187150478	4	A	G	0.487	0.086	0.008	4.2E-29	trans
1605	PDGFC	Q9NRA1	Platelet-derived growth factor C	rs75077631	176840084	5	G	GC	0.258	0.061	0.009	3.7E-12	trans
1605	PDGFC	Q9NRA1	Platelet-derived growth factor C	rs150611042	117083803	9	C	A	0.928	0.472	0.015	5.8E-207	trans
1605	PDGFC	Q9NRA1	Platelet-derived growth factor C	rs28929474	94844947	14	T	C	0.021	0.352	0.027	5.7E-39	trans
1605	PDGFC	Q9NRA1	Platelet-derived growth factor C	rs149394327	64228995	17	C	G	0.030	0.359	0.023	1.9E-56	trans
1605	PDGFC	Q9NRA1	Platelet-derived growth factor C	rs1065853	45413233	19	T	G	0.081	0.183	0.014	2.7E-38	trans
1606	PDGFRA	P16234	Platelet-derived growth factor receptor alpha	rs56033718	58446398	3	G	A	0.371	0.082	0.007	1.1E-27	trans
1606	PDGFRA	P16234	Platelet-derived growth factor receptor alpha	rs4558798	98308915	3	A	G	0.327	0.053	0.008	3.9E-12	trans
1606	PDGFRA	P16234	Platelet-derived growth factor receptor alpha	rs35597368	55139771	4	T	C	0.890	0.425	0.012	1.6E-286	cis
1606	PDGFRA	P16234	Platelet-derived growth factor receptor alpha	rs6913664	32295874	6	T	C	0.473	0.056	0.007	7.1E-15	trans
1606	PDGFRA	P16234	Platelet-derived growth factor receptor alpha	rs12148472	79231478	15	C	T	0.130	0.076	0.011	1.0E-12	trans
1606	PDGFRA	P16234	Platelet-derived growth factor receptor alpha	rs180915454	7192025	17	T	C	0.004	0.406	0.059	4.6E-12	trans
1606	PDGFRA	P16234	Platelet-derived growth factor receptor alpha	rs2294915	44340904	22	T	C	0.232	0.063	0.009	1.9E-13	trans
1607	PDGFRB	P09619	Platelet-derived growth factor receptor beta	rs2304058	149508544	5	G	C	0.558	1.179	0.008	0.0E+00	cis
1607	PDGFRB	P09619	Platelet-derived growth factor receptor beta	rs186021206	7069412	17	A	G	0.006	0.418	0.033	3.5E-37	trans
1608	PDIA4	P13667	Protein disulfide-isomerase A4	rs1354034	56849749	3	C	T	0.605	0.067	0.008	3.4E-18	trans
1608	PDIA4	P13667	Protein disulfide-isomerase A4	rs3804749	122833003	3	C	T	0.407	0.053	0.008	5.4E-12	trans
1608	PDIA4	P13667	Protein disulfide-isomerase A4	rs6953222	148727620	7	G	T	0.826	0.117	0.010	2.8E-32	cis
1608	PDIA4	P13667	Protein disulfide-isomerase A4	rs6993770	106581528	8	A	T	0.714	0.057	0.008	6.9E-12	trans
1609	PDIA5	Q14554	Protein disulfide-isomerase A5	rs3804749	122833003	3	C	T	0.407	0.249	0.008	4.7E-218	cis
1609	PDIA5	Q14554	Protein disulfide-isomerase A5	rs2844530	31353435	6	G	C	0.144	0.291	0.011	5.4E-156	trans
1609	PDIA5	Q14554	Protein disulfide-isomerase A5	rs1646724	134001875	7	G	T	0.482	0.073	0.008	1.1E-21	trans

1609	PDIA5	Q14554	Protein disulfide-isomerase A5	rs6081565	19287904	20	A	G	0.354	0.080	0.008	4.9E-24	trans
1610	PDLIM5	Q96HC4	PDZ and LIM domain protein 5	rs1354034	56849749	3	C	T	0.604	0.055	0.008	2.8E-13	trans
1610	PDLIM5	Q96HC4	PDZ and LIM domain protein 5	rs342298	106373646	7	C	T	0.545	0.078	0.007	2.4E-25	trans
1610	PDLIM5	Q96HC4	PDZ and LIM domain protein 5	rs6993770	106581528	8	A	T	0.714	0.075	0.008	1.5E-19	trans
1611	PDLIM7	Q9NR12	PDZ and LIM domain protein 7	rs1354034	56849749	3	C	T	0.604	0.055	0.008	3.4E-13	trans
1611	PDLIM7	Q9NR12	PDZ and LIM domain protein 7	rs342296	106372903	7	G	A	0.544	0.080	0.007	7.8E-27	trans
1612	PDZD2	O15018	PDZ domain-containing protein 2	rs183711	31638475	5	G	T	0.920	0.394	0.014	8.7E-173	cis
1612	PDZD2	O15018	PDZ domain-containing protein 2	rs342293	106372219	7	C	G	0.540	0.066	0.008	2.5E-18	trans
1613	PDZK1	Q5T2W1	Na(+)/H(+) exchange regulatory cofactor NHE-RF3	rs1471633	145723739	1	A	C	0.466	0.111	0.008	6.0E-49	cis
1613	PDZK1	Q5T2W1	Na(+)/H(+) exchange regulatory cofactor NHE-RF3	rs4917	186337713	3	C	T	0.640	0.132	0.008	1.8E-62	trans
1613	PDZK1	Q5T2W1	Na(+)/H(+) exchange regulatory cofactor NHE-RF3	rs7990	32609965	6	C	A	0.865	0.139	0.012	1.6E-33	trans
1614	PEAR1	Q5VY43	Platelet endothelial aggregation receptor 1	rs4661012	156885491	1	T	G	0.638	0.213	0.008	1.6E-165	cis
1614	PEAR1	Q5VY43	Platelet endothelial aggregation receptor 1	rs1354034	56849749	3	T	C	0.396	0.064	0.007	1.4E-17	trans
1614	PEAR1	Q5VY43	Platelet endothelial aggregation receptor 1	rs13107325	103188709	4	T	C	0.075	0.170	0.014	4.2E-34	trans
1614	PEAR1	Q5VY43	Platelet endothelial aggregation receptor 1	rs9389269	135427159	6	C	T	0.272	0.061	0.008	1.6E-13	trans
1614	PEAR1	Q5VY43	Platelet endothelial aggregation receptor 1	rs10758659	4858019	9	G	A	0.564	0.057	0.007	2.7E-14	trans
1614	PEAR1	Q5VY43	Platelet endothelial aggregation receptor 1	rs8176741	136131461	9	A	G	0.061	0.438	0.015	5.8E-176	trans
1614	PEAR1	Q5VY43	Platelet endothelial aggregation receptor 1	rs3967200	126232385	11	C	T	0.865	0.078	0.011	4.3E-13	trans
1614	PEAR1	Q5VY43	Platelet endothelial aggregation receptor 1	rs186021206	7069412	17	A	G	0.006	0.758	0.050	4.0E-51	trans
1615	PEBP1	P30086	Phosphatidylethanolamine-binding protein 1	rs1354034	56849749	3	C	T	0.604	0.059	0.007	4.7E-15	trans
1616	PECAM1	P16284	Platelet endothelial cell adhesion molecule	rs1354034	56849749	3	C	T	0.604	0.073	0.007	8.3E-24	trans
1616	PECAM1	P16284	Platelet endothelial cell adhesion molecule	rs56083751	131664638	5	T	A	0.522	0.051	0.007	7.4E-13	trans
1616	PECAM1	P16284	Platelet endothelial cell adhesion molecule	rs2519093	136141870	9	C	T	0.816	0.587	0.010	0.0E+00	trans
1616	PECAM1	P16284	Platelet endothelial cell adhesion molecule	rs35166255	126301756	11	A	G	0.034	0.324	0.020	3.8E-60	trans
1616	PECAM1	P16284	Platelet endothelial cell adhesion molecule	rs186021206	7069412	17	A	G	0.006	0.611	0.049	6.3E-36	trans
1617	PECR	Q9BY49	Peroxisomal trans-2-enoyl-CoA reductase	rs10197542	216903894	2	A	C	0.896	0.454	0.013	2.7E-286	cis
1617	PECR	Q9BY49	Peroxisomal trans-2-enoyl-CoA reductase	rs6993770	106581528	8	A	T	0.713	0.059	0.008	2.6E-12	trans
1618	PENK	P01210	Proenkephalin-A	rs781656	57778645	4	A	G	0.183	0.090	0.009	1.1E-21	trans
1618	PENK	P01210	Proenkephalin-A	rs2870238	77373079	4	T	C	0.503	0.049	0.007	8.3E-12	trans
1618	PENK	P01210	Proenkephalin-A	rs11154902	137301298	6	C	T	0.538	0.057	0.007	5.7E-15	trans
1618	PENK	P01210	Proenkephalin-A	rs2576575	57361293	8	C	T	0.534	0.301	0.007	0.0E+00	cis
1619	PEPD	P12955	Xaa-Pro dipeptidase	rs1047891	211540507	2	C	A	0.687	0.072	0.008	5.4E-21	trans
1619	PEPD	P12955	Xaa-Pro dipeptidase	rs7032525	95882901	9	T	C	0.800	0.132	0.009	7.0E-50	trans
1619	PEPD	P12955	Xaa-Pro dipeptidase	rs35032070	47408592	11	G	A	0.636	0.078	0.007	2.4E-26	trans
1619	PEPD	P12955	Xaa-Pro dipeptidase	rs72731930	57408432	15	C	T	0.928	0.210	0.014	1.2E-52	trans
1619	PEPD	P12955	Xaa-Pro dipeptidase	rs17569	33882222	19	G	A	0.843	0.493	0.010	0.0E+00	cis
1619	PEPD	P12955	Xaa-Pro dipeptidase	rs6125961	48884124	20	C	A	0.799	0.063	0.009	1.0E-12	trans
1619	PEPD	P12955	Xaa-Pro dipeptidase	rs2904552	18905964	22	T	C	0.104	0.135	0.012	1.0E-29	trans
1620	PER3	P56645	Period circadian protein homolog 3	rs228647	7914177	1	A	G	0.563	0.370	0.008	0.0E+00	cis
1620	PER3	P56645	Period circadian protein homolog 3	rs1354034	56849749	3	C	T	0.604	0.052	0.007	9.8E-13	trans
1620	PER3	P56645	Period circadian protein homolog 3	rs6440003	141094209	3	G	A	0.557	0.050	0.007	2.1E-12	trans
1620	PER3	P56645	Period circadian protein homolog 3	rs6993770	106581528	8	A	T	0.713	0.133	0.008	1.8E-63	trans
1621	PF4	P02776	Platelet factor 4	rs13412535	224874874	2	G	A	0.769	0.061	0.009	6.0E-12	trans
1621	PF4	P02776	Platelet factor 4	rs1354034	56849749	3	T	C	0.395	0.072	0.008	5.7E-21	trans
1621	PF4	P02776	Platelet factor 4	rs11574452	74846661	4	C	A	0.955	0.201	0.018	9.4E-28	cis
1621	PF4	P02776	Platelet factor 4	rs4698839	102801941	4	C	T	0.602	0.058	0.008	4.1E-14	trans
1621	PF4	P02776	Platelet factor 4	rs274555	131722951	5	T	C	0.399	0.060	0.008	7.6E-15	trans
1621	PF4	P02776	Platelet factor 4	rs2516471	31400292	6	G	A	0.937	0.114	0.016	2.3E-13	trans
1621	PF4	P02776	Platelet factor 4	rs6961069	80218961	7	T	C	0.403	0.063	0.008	1.3E-16	trans
1621	PF4	P02776	Platelet factor 4	rs6993770	106581528	8	A	T	0.713	0.115	0.008	6.9E-44	trans
1621	PF4	P02776	Platelet factor 4	rs10820606	99192919	9	A	C	0.770	0.066	0.009	2.4E-13	trans
1621	PF4	P02776	Platelet factor 4	rs10761737	65052205	10	C	T	0.413	0.121	0.008	2.2E-56	trans
1621	PF4	P02776	Platelet factor 4	rs12445050	81870969	16	T	C	0.138	0.087	0.011	9.2E-16	trans
1621	PF4	P02776	Platelet factor 4	rs1654425	55538980	19	C	T	0.834	0.149	0.010	1.1E-48	trans
1621	PF4	P02776	Platelet factor 4	rs6081569	19303786	20	C	G	0.369	0.072	0.008	3.2E-20	trans
1621	PF4	P02776	Platelet factor 4	rs2096256	99924813	X	G	A	0.342	0.046	0.007	2.2E-12	trans
1622	PFKFB2	O60825	6-phosphofructo-2-kinase/fructose-2,6-bisphosphatase 2	rs1560	207250631	1	A	G	0.518	0.188	0.007	4.1E-142	cis
1622	PFKFB2	O60825	6-phosphofructo-2-kinase/fructose-2,6-bisphosphatase 2	rs1354034	56849749	3	C	T	0.605	0.058	0.007	9.3E-15	trans
1622	PFKFB2	O60825	6-phosphofructo-2-kinase/fructose-2,6-bisphosphatase 2	rs6993770	106581528	8	A	T	0.714	0.060	0.008	2.2E-13	trans
1622	PFKFB2	O60825	6-phosphofructo-2-kinase/fructose-2,6-bisphosphatase 2	rs7080386	65048306	10	A	C	0.411	0.054	0.007	7.4E-13	trans
1623	PGA4	P0DJD7	Pepsin A-4	rs1639109	31194583	6	G	A	0.846	0.093	0.010	6.0E-20	trans
1623	PGA4	P0DJD7	Pepsin A-4	rs117739035	408174	11	T	G	0.037	0.148	0.020	4.0E-14	trans
1623	PGA4	P0DJD7	Pepsin A-4	rs4414241	60972512	11	T	C	0.243	0.078	0.010	5.3E-16	cis
1623	PGA4	P0DJD7	Pepsin A-4	rs112585477	37891654	17	A	G	0.041	0.129	0.019	4.1E-12	trans
1624	PGD	P52209	6-phosphogluconate dehydrogenase, decarboxylating	rs1354034	56849749	3	C	T	0.604	0.056	0.008	1.2E-13	trans
1624	PGD	P52209	6-phosphogluconate dehydrogenase, decarboxylating	rs6993770	106581528	8	A	T	0.713	0.059	0.008	4.1E-13	trans
1624	PGD	P52209	6-phosphogluconate dehydrogenase, decarboxylating	rs3847326	65331859	10	A	G	0.518	0.060	0.007	3.7E-16	trans
1625	PGF	P49763	Placenta growth factor	rs9943922	28994655	13	C	T	0.533	0.084	0.007	6.3E-36	trans
1625	PGF	P49763	Placenta growth factor	rs4902651	69272125	14	C	T	0.498	0.047	0.007	3.3E-12	trans
1625	PGF	P49763	Placenta growth factor	rs704	26694861	17	G	A	0.527	0.048	0.007	5.1E-13	trans
1626	PGLYRP1	O75594	Peptidoglycan recognition protein 1	rs3917932	36943916	1	C	G	0.424	0.067	0.007	1.7E-19	trans
1626	PGLYRP1	O75594	Peptidoglycan recognition protein 1	rs113542380	43464818	2	A	G	0.074	0.138	0.014	8.9E-23	trans
1626	PGLYRP1	O75594	Peptidoglycan recognition protein 1	rs6436124	220041433	2	A	C	0.607	0.084	0.008	4.6E-29	trans
1626	PGLYRP1	O75594	Peptidoglycan recognition protein 1	rs2290547	47061183	3	A	G	0.176	0.065	0.010	8.2E-12	trans
1626	PGLYRP1	O75594	Peptidoglycan recognition protein 1	rs41266485	127390525	3	T	C	0.204	0.078	0.009	5.9E-18	trans
1626	PGLYRP1	O75594	Peptidoglycan recognition protein 1	rs218264	55408875	4	T	A	0.247	0.066	0.009	1.3E-14	trans
1626	PGLYRP1	O75594	Peptidoglycan recognition protein 1	rs141484466	31324552	6	C	G	0.042	0.315	0.019	4.2E-60	trans
1626	PGLYRP1	O75594	Peptidoglycan recognition protein 1	rs11775560	61660163	8	G	A	0.186	0.130	0.009	4.1E-44	trans
1626	PGLYRP1	O75594	Peptidoglycan recognition protein 1	rs4407843	130609368	8	A	C	0.432	0.054	0.007	3.6E-13	trans
1626	PGLYRP1	O75594	Peptidoglycan recognition protein 1	rs2074038	44087989	11	T	G	0.100	0.117	0.012	9.6E-22	trans
1626	PGLYRP1	O75594	Peptidoglycan recognition protein 1	rs2511241	72945341	11	T	C	0.929	0.101	0.014	1.1E-12	trans
1626	PGLYRP1	O75594	Peptidoglycan recognition protein 1	rs2072563	46526648	19	G	A	0.675	0.297	0.008	1.6E-306	cis
1626	PGLYRP1	O75594	Peptidoglycan recognition protein 1	rs5936474	147357492	X	G	A	0.584	0.045	0.006	1.6E-13	trans
1627	PGLYRP2	Q96PD5	N-acetylmuramoyl-L-alanine amidase	rs59950280	3452345	4	A	G	0.330	0.064	0.007	9.7E-29	trans
1627	PGLYRP2	Q96PD5	N-acetylmuramoyl-L-alanine amidase	rs33951980	73029437	7	C	T	0.871	0.072	0.008	1.1E-19	trans
1627	PGLYRP2	Q96PD5	N-acetylmuramoyl-L-alanine amidase	rs34440547	15582863	19	C	T	0.781	1.144	0.009	0.0E+00	cis
1627	PGLYRP2	Q96PD5	N-acetylmuramoyl-L-alanine amidase	rs2229742	16339172	21	C	G	0.104	0.065	0.009	8.7E-14	trans
1628	PGLYRP4	Q96LB8	Peptidoglycan recognition protein 4	rs111586430	153324310	1	T	C	0.057	0.922	0.017	0.0E+00	cis
1629	PGR	P06401	Progesterone receptor	rs139299944	32602665	6	CT	C	0.344	0.098	0.008	4.8E-34	trans
1629	PGR	P06401	Progesterone receptor	rs4813331	1897442	20	C	T	0.619	0.149	0.008	2.8E-78	trans
1630	PHACTR2	O75167	Phosphatase and actin regulator 2	rs1354034	56849749	3	C	T	0.604	0.070	0.008	2.3E-20	trans
1630	PHACTR2	O75167	Phosphatase and actin regulator 2	rs9484759	143858683	6	G	A	0.204	0.248	0.009	5.8E-157	cis
1631	PHLDB1	Q86UU1	Pleckstrin homology-like domain family B member 1	rs1354034	56849749	3	C	T	0.605	0.061	0.008	1.3E-15	trans
1631	PHLDB1	Q86UU1	Pleckstrin homology-like domain family B member 1	rs342293	106372219	7	C	G	0.540	0.075	0.007	1.2E-23	trans
1632	PHYKPL	Q8IUZ5	5-phosphohydroxy-L-lysine phospho-lyase	rs142181517	177638965	5	T	A	0.993	2.038	0.044	0.0E+00	cis
1633	PI16	Q6UXB8	Peptidase inhibitor 16	rs61821645	219275920	1	G	A	0.027	0.173	0.023	4.5E-14	trans
1633	PI16	Q6UXB8	Peptidase inhibitor 16	rs35004449	52852897	3	T	G	0.259	0.343	0.008	0.0E+00	trans
1633	PI16	Q6UXB8	Peptidase inhibitor 16	rs62406524	36925976	6	G	C	0.904	0.430	0.013	5.2E-252	cis
1633	PI16	Q6UXB8	Peptidase inhibitor 16	rs34931250	66879927	17	C	T	0.939	0.166	0.015	3.1E-29	trans
1633	PI16	Q6UXB8	Peptidase inhibitor 16	rs12975366	54759361	19	C	T	0.396	0.073	0.007	4.4E-24	trans
1634	PI3	P19957	Elafin	rs34155263	72263225	2	C	T	0.562	0.059	0.007	1.1E-16	trans
1634	PI3	P19957	Elafin	rs28550378	126608642	8	T	C	0.465	0.050	0.007	6.7E-13	trans
1634	PI3	P19957	Elafin	rs7088444	80769705	10	T	C	0.478	0.060	0.007	1.4E-17	trans
1634	PI3	P19957	Elafin	rs35770435	43788804	20	T	C	0.180	0.353	0.009	0.0E+00	cis
1635	PIBF1	Q8WXW3	Progesterone-induced-blocking factor 1, Isoform 4	rs1354034	56849749	3	C	T	0.604	0.094	0.008	1.1E-34	trans
1635	PIBF1	Q8WXW3	Progesterone-induced-blocking factor 1, Isoform 4	rs342296	106372903	7	G	A	0.543	0.058	0.007	7.5E-15	trans
1635	PIBF1	Q8WXW3	Progesterone-induced-blocking factor 1, Isoform 4	rs6993770	106581528	8	A	T	0.714	0.061	0.008	2.3E-13	trans
1635	PIBF1	Q8WXW3	Progesterone-induced-blocking factor 1, Isoform 4	rs61966211	73562649	13	G	T	0.616	0.062	0.008	7.6E-16	cis
1636	PIGR	P01833	Polymeric immunoglobulin receptor	rs291102	207106478	1	G	A	0.976	0.508	0.025	4.9E-95	cis
1636	PIGR	P01833	Polymeric immunoglobulin receptor	rs1200427	45526470	6	T	A	0.245	0.085	0.009	2.1E-22	trans
1636	PIGR	P01833	Polymeric immunoglobulin receptor	rs113810201	150926254	7	G	A	0.115	0.101	0.012	7.1E-18	trans
1636	PIGR	P01833	Polymeric immunoglobulin receptor	rs61882275	34504292	11	G	A	0.623	0.057	0.008	1.9E-13	trans
1636	PIGR	P01833	Polymeric immunoglobulin receptor	rs708686	5840619	19	T	C	0.268	0.110	0.009	1.7E-38	trans
1637	PIK3AP1	Q6ZUJ8	Phosphoinositide 3-kinase adapter protein 1	rs11204523	248020556	1	G	C	0.241	0.079	0.009	5.0E-20	trans
1637	PIK3AP1	Q6ZUJ8	Phosphoinositide 3-kinase adapter protein 1	rs1354034	56849749	3	C	T	0.604	0.103	0.007	3.5E-44	trans
1637	PIK3AP1	Q6ZUJ8	Phosphoinositide 3-kinase adapter protein 1	rs7695151	2772530	4	C	T	0.536	0.054	0.007	6.7E-14	trans
1637	PIK3AP1	Q6ZUJ8	Phosphoinositide 3-kinase adapter protein 1	rs78842280	47481628	6	T	G	0.726	0.063	0.008	2.1E-14	trans
1637	PIK3AP1	Q6ZUJ8	Phosphoinositide 3-kinase adapter protein 1	rs342293	106372219	7	C	G	0.541	0.052	0.007	4.5E-13	trans
1637	PIK3AP1	Q6ZUJ8	Phosphoinositide 3-kinase adapter protein 1	rs41317268	98469114	10	A	G	0.805	0.230	0.009	6.5E-136	cis
1638	PIK3IP1	Q96FE7	Phosphoinositide-3-kinase-interacting protein 1	rs28640218	20359267	16	G	T	0.809	0.074	0.009	1.3E-16	trans
1638	PIK3IP1	Q96FE7	Phosphoinositide-3-kinase-interacting protein 1	rs2413028	31541907	22	T	C	0.829	0.368	0.009	0.0E+00	cis
1639	PIKFYVE	Q9Y2I7	1-phosphatidylinositol 3-phosphate 5-kinase	rs1354034	56849749	3	C	T	0.604	0.054	0.008	1.4E-12	trans
1639	PIKFYVE	Q9Y2I7	1-phosphatidylinositol 3-phosphate 5-kinase	rs34436714	54327313	19	A	C	0.209	0.066	0.009	8.7E-13	trans
1640	PILRA	Q9UKJ1	Paired immunoglobulin-like type 2 receptor alpha	rs61747728	179526214	1	T	C	0.038	0.100	0.015	1.4E-11	trans
1640	PILRA	Q9UKJ1	Paired immunoglobulin-like type 2 receptor alpha	rs10085857	100614226	7	T	C	0.480	0.189	0.008	6.7E-129	trans
1640	PILRA	Q9UKJ1	Paired immunoglobulin-like type 2 receptor alpha	rs1859788	99971834	7	G	A	0.681	1.000	0.008	0.0E+00	cis
1640	PILRA	Q9UKJ1	Paired immunoglobulin-like type 2 receptor alpha	rs186021206	7069412	17	A	G	0.006	0.556	0.039	6.4E-46	trans
1640	PILRA	Q9UKJ1	Paired immunoglobulin-like type 2 receptor alpha	rs140528980	74604670	17	T	C	0.605	0.042	0.006	6.0E-13	trans
1640	PILRA	Q9UKJ1	Paired immunoglobulin-like type 2 receptor alpha	rs61698755	79257880	17	T	C	0.433	0.050	0.006	2.6E-18	trans
1641	PILRB	Q9UKJ0	Paired immunoglobulin-like type 2 receptor beta	rs10085857	100614226	7	T	C	0.481	0.219	0.008	9.4E-169	trans
1641	PILRB	Q9UKJ0	Paired immunoglobulin-like type 2 receptor beta	rs1859788	99971834	7	G	A	0.681	1.147	0.008	0.0E+00	cis
1641	PILRB	Q9UKJ0	Paired immunoglobulin-like type 2 receptor beta	rs186021206	7069412	17	A	G	0.006	0.471	0.035	1.7E-41	trans
1641	PILRB	Q9UKJ0	Paired immunoglobulin-like type 2 receptor beta	rs140528980	74604670	17	T	C	0.605	0.036	0.005	8.0E-12	trans
1641	PILRB	Q9UKJ0	Paired immunoglobulin-like type 2 receptor beta	rs8077394	79258787	17	A	G	0.446	0.046	0.005	2.6E-19	trans
1642	PINLYP	A6NC86	phospholipase A2 inhibitor and Ly6/PLAUR domain-containing protein	rs80293268	8207579	1	G	C	0.952	0.120	0.016	1.2E-14	trans
1642	PINLYP	A6NC86	phospholipase A2 inhibitor and Ly6/PLAUR domain-containing protein	rs36010924	152088844	1	A	G	0.816	0.089	0.008	1.5E-27	trans
1642	PINLYP	A6NC86	phospholipase A2 inhibitor and Ly6/PLAUR domain-containing protein	rs4681529	149336634	3	T	A	0.323	0.059	0.007	2.5E-18	trans
1642	PINLYP	A6NC86	phospholipase A2 inhibitor and Ly6/PLAUR domain-containing protein	rs35457250	186338564	3	T	C	0.009	0.634	0.034	9.8E-79	trans
1642	PINLYP	A6NC86	phospholipase A2 inhibitor and Ly6/PLAUR domain-containing protein	rs2517801	29877773	6	C	T	0.612	0.046	0.007	1.6E-11	trans
1642	PINLYP	A6NC86	phospholipase A2 inhibitor and Ly6/PLAUR domain-containing protein	rs16888292	117316979	8	G	A	0.806	0.059	0.008	1.4E-13	trans
1642	PINLYP	A6NC86	phospholipase A2 inhibitor and Ly6/PLAUR domain-containing protein	rs7905367	54334653	10	C	G	0.797	0.053	0.008	1.0E-11	trans
1642	PINLYP	A6NC86	phospholipase A2 inhibitor and Ly6/PLAUR domain-containing protein	rs58589035	60042787	11	T	C	0.880	0.067	0.010	6.2E-12	trans
1642	PINLYP	A6NC86	phospholipase A2 inhibitor and Ly6/PLAUR domain-containing protein	rs3803856	45942176	17	T	C	0.352	0.047	0.007	3.1E-12	trans

1642	PINLYP	A6NC86	phospholipase A2 inhibitor and Ly6/PLAUR domain-containing protein	rs112001035	66823805	17	A	G	0.061	0.354	0.014	2.0E-150	trans
1642	PINLYP	A6NC86	phospholipase A2 inhibitor and Ly6/PLAUR domain-containing protein	rs12457893	60926161	18	A	C	0.542	0.047	0.006	2.4E-13	trans
1642	PINLYP	A6NC86	phospholipase A2 inhibitor and Ly6/PLAUR domain-containing protein	rs3213276	44073794	19	T	C	0.588	0.767	0.008	0.0E+00	cis
1643	PKD1	P98161	Polycystin-1	rs2728104	88973006	4	T	C	0.889	0.098	0.011	7.0E-20	trans
1643	PKD1	P98161	Polycystin-1	rs8176749	136131188	9	T	C	0.061	0.143	0.014	3.5E-24	trans
1643	PKD1	P98161	Polycystin-1	rs56278466	17875857	10	G	T	0.661	0.109	0.007	4.3E-52	trans
1643	PKD1	P98161	Polycystin-1	rs186021206	7069412	17	A	G	0.006	0.669	0.047	1.3E-46	trans
1643	PKD1	P98161	Polycystin-1	rs868875	7831166	19	A	G	0.699	0.052	0.007	2.5E-12	trans
1643	PKD1	P98161	Polycystin-1	rs189448562	3705115	20	C	G	0.011	0.545	0.033	3.1E-62	trans
1644	PKD2	Q13563	Polycystin-2	rs1354034	56849749	3	C	T	0.604	0.100	0.008	4.1E-38	trans
1644	PKD2	Q13563	Polycystin-2	rs2728104	88973006	4	T	C	0.889	0.137	0.012	6.6E-30	cis
1644	PKD2	Q13563	Polycystin-2	rs7080536	115348046	10	G	A	0.956	0.227	0.019	2.5E-33	trans
1644	PKD2	Q13563	Polycystin-2	rs11604127	196944	11	T	C	0.230	0.064	0.009	2.2E-12	trans
1644	PKD2	Q13563	Polycystin-2	rs12292693	64936719	11	A	C	0.740	0.060	0.009	4.5E-12	trans
1645	PKLR	P30613	Pyruvate kinase PKLR	rs77350683	155484430	1	T	C	0.932	0.364	0.015	2.4E-132	cis
1645	PKLR	P30613	Pyruvate kinase PKLR	rs6592965	50427982	7	A	G	0.454	0.060	0.007	2.0E-16	trans
1645	PKLR	P30613	Pyruvate kinase PKLR	rs2361710	78111877	17	T	C	0.530	0.052	0.007	1.6E-12	trans
1646	PKN3	Q6P5Z2	Serine/threonine-protein kinase N3	rs1354034	56849749	3	C	T	0.604	0.079	0.008	2.5E-24	trans
1646	PKN3	Q6P5Z2	Serine/threonine-protein kinase N3	rs13283282	131465481	9	G	C	0.146	0.215	0.011	8.8E-89	cis
1647	PLA2G10	O15496	Group 10 secretory phospholipase A2	rs10889356	63155349	1	A	G	0.348	0.058	0.007	6.7E-15	trans
1647	PLA2G10	O15496	Group 10 secretory phospholipase A2	rs6547409	21190209	2	T	C	0.052	0.111	0.016	7.3E-12	trans
1647	PLA2G10	O15496	Group 10 secretory phospholipase A2	rs1260326	27730940	2	C	T	0.608	0.050	0.007	8.2E-12	trans
1647	PLA2G10	O15496	Group 10 secretory phospholipase A2	rs149401529	52547411	3	G	C	0.994	0.464	0.047	5.2E-23	trans
1647	PLA2G10	O15496	Group 10 secretory phospholipase A2	rs28367569	31236102	6	C	T	0.045	0.571	0.018	2.2E-231	trans
1647	PLA2G10	O15496	Group 10 secretory phospholipase A2	rs78963197	19836646	8	C	T	0.097	0.107	0.012	1.0E-18	trans
1647	PLA2G10	O15496	Group 10 secretory phospholipase A2	rs2976388	143760256	8	G	A	0.577	0.257	0.007	5.3E-272	trans
1647	PLA2G10	O15496	Group 10 secretory phospholipase A2	rs2519093	136141870	9	C	T	0.816	0.112	0.009	5.1E-34	trans
1647	PLA2G10	O15496	Group 10 secretory phospholipase A2	rs34481144	320836	11	C	T	0.498	0.111	0.007	4.9E-52	trans
1647	PLA2G10	O15496	Group 10 secretory phospholipase A2	rs7232	59940599	11	A	T	0.373	0.070	0.007	8.3E-21	trans
1647	PLA2G10	O15496	Group 10 secretory phospholipase A2	rs964184	116648917	11	C	G	0.867	0.113	0.011	1.1E-26	trans
1647	PLA2G10	O15496	Group 10 secretory phospholipase A2	rs186021206	7069412	17	A	G	0.006	0.379	0.049	1.0E-14	trans
1647	PLA2G10	O15496	Group 10 secretory phospholipase A2	rs112001035	66823805	17	A	G	0.061	0.156	0.015	1.8E-24	trans
1647	PLA2G10	O15496	Group 10 secretory phospholipase A2	rs1065853	45413233	19	T	G	0.081	0.212	0.013	3.4E-58	trans
1647	PLA2G10	O15496	Group 10 secretory phospholipase A2	rs492602	49206417	19	A	G	0.490	0.075	0.007	2.0E-25	trans
1648	PLA2G15	Q8NCC3	Phospholipase A2 group XV	rs2187668	32605884	6	T	C	0.148	0.136	0.010	1.7E-41	trans
1648	PLA2G15	Q8NCC3	Phospholipase A2 group XV	rs9346804	160401028	6	G	A	0.863	0.081	0.011	1.1E-14	trans
1648	PLA2G15	Q8NCC3	Phospholipase A2 group XV	rs145078947	93652974	14	T	G	0.003	1.260	0.072	9.9E-69	trans
1648	PLA2G15	Q8NCC3	Phospholipase A2 group XV	rs3743588	11836508	16	A	G	0.286	0.062	0.008	5.7E-14	trans
1648	PLA2G15	Q8NCC3	Phospholipase A2 group XV	rs7672	68294800	16	C	G	0.285	0.245	0.008	2.8E-196	cis
1648	PLA2G15	Q8NCC3	Phospholipase A2 group XV	rs186021206	7069412	17	A	G	0.006	0.460	0.050	1.7E-20	trans
1648	PLA2G15	Q8NCC3	Phospholipase A2 group XV	rs58542926	19379549	19	T	C	0.075	0.123	0.014	2.6E-19	trans
1649	PLA2G1B	P04054	Phospholipase A2	rs1260326	27730940	2	C	T	0.608	0.053	0.007	1.6E-12	trans
1649	PLA2G1B	P04054	Phospholipase A2	rs17032925	67266483	2	T	C	0.917	0.094	0.013	2.2E-12	trans
1649	PLA2G1B	P04054	Phospholipase A2	rs1361108	126767600	6	C	T	0.544	0.079	0.007	5.2E-27	trans
1649	PLA2G1B	P04054	Phospholipase A2	rs7003060	18729818	8	T	G	0.301	0.062	0.008	1.1E-14	trans
1649	PLA2G1B	P04054	Phospholipase A2	rs1372993	129565662	8	G	A	0.884	0.164	0.012	6.5E-46	trans
1649	PLA2G1B	P04054	Phospholipase A2	rs116842520	135954065	9	A	G	0.036	0.149	0.020	9.7E-14	trans
1649	PLA2G1B	P04054	Phospholipase A2	rs2438988	49370090	10	A	G	0.365	0.074	0.008	1.2E-20	trans
1649	PLA2G1B	P04054	Phospholipase A2	rs4751995	118397884	10	A	G	0.481	0.079	0.007	4.9E-27	trans
1649	PLA2G1B	P04054	Phospholipase A2	rs56994090	101306447	14	T	C	0.583	0.051	0.007	8.5E-12	trans
1649	PLA2G1B	P04054	Phospholipase A2	rs72802342	75234872	16	C	A	0.924	0.203	0.014	1.2E-46	trans
1649	PLA2G1B	P04054	Phospholipase A2	rs1673931	88976477	16	T	C	0.622	0.135	0.008	1.7E-71	trans
1649	PLA2G1B	P04054	Phospholipase A2	rs1468481	69139979	17	T	C	0.768	0.062	0.009	9.8E-13	trans
1649	PLA2G1B	P04054	Phospholipase A2	rs62090604	42416240	18	T	G	0.077	0.094	0.014	6.2E-12	trans
1649	PLA2G1B	P04054	Phospholipase A2	rs681343	49206462	19	T	C	0.509	0.104	0.007	1.0E-45	trans
1649	PLA2G1B	P04054	Phospholipase A2	rs71327329	29327347	22	A	G	0.871	0.075	0.011	8.1E-12	trans
1650	PLA2G2A	P14555	Phospholipase A2, membrane associated	rs11573156	20306146	1	C	G	0.228	0.731	0.009	0.0E+00	cis
1650	PLA2G2A	P14555	Phospholipase A2, membrane associated	rs6698842	66164732	1	T	C	0.633	0.081	0.008	1.2E-26	trans
1650	PLA2G2A	P14555	Phospholipase A2, membrane associated	rs34931250	66879927	17	T	C	0.060	0.189	0.014	2.6E-42	trans
1650	PLA2G2A	P14555	Phospholipase A2, membrane associated	rs112896133	12429782	19	G	A	0.987	0.201	0.029	2.5E-12	trans
1650	PLA2G2A	P14555	Phospholipase A2, membrane associated	rs10405357	54759666	19	T	C	0.565	0.047	0.007	2.8E-12	trans
1651	PLA2G4A	P47712	Cytosolic phospholipase A2	rs1354034	56849749	3	C	T	0.604	0.066	0.008	4.9E-18	trans
1651	PLA2G4A	P47712	Cytosolic phospholipase A2	rs1149577	76545414	11	C	T	0.213	0.067	0.009	2.2E-13	trans
1652	PLA2G7	Q13093	Platelet-activating factor acetylhydrolase	rs11591147	55505647	1	G	T	0.982	0.219	0.027	5.2E-16	trans
1652	PLA2G7	Q13093	Platelet-activating factor acetylhydrolase	rs7528419	109817192	1	A	G	0.777	0.099	0.009	4.9E-31	trans
1652	PLA2G7	Q13093	Platelet-activating factor acetylhydrolase	rs6871667	74604742	5	A	G	0.404	0.052	0.007	9.1E-13	trans
1652	PLA2G7	Q13093	Platelet-activating factor acetylhydrolase	rs2119690	19859539	8	G	A	0.712	0.055	0.008	2.4E-12	trans
1652	PLA2G7	Q13093	Platelet-activating factor acetylhydrolase	rs28601761	126500031	8	C	G	0.581	0.063	0.007	4.5E-18	trans
1652	PLA2G7	Q13093	Platelet-activating factor acetylhydrolase	rs562028	59877850	11	C	A	0.629	0.074	0.007	1.3E-23	trans
1652	PLA2G7	Q13093	Platelet-activating factor acetylhydrolase	rs1065853	45413233	19	G	T	0.919	0.481	0.013	1.4E-284	trans
1653	PLAT	P00750	Tissue-type plasminogen activator	rs7765526	147713764	6	G	A	0.541	0.050	0.007	3.3E-12	trans
1653	PLAT	P00750	Tissue-type plasminogen activator	rs2020921	42044965	8	G	A	0.975	0.280	0.023	2.0E-35	cis
1653	PLAT	P00750	Tissue-type plasminogen activator	rs8112983	49229525	19	C	T	0.536	0.069	0.007	2.2E-22	trans
1654	PLAU	P00749	Urokinase-type plasminogen activator	rs10194846	62541752	2	C	T	0.614	0.056	0.007	1.9E-14	trans
1654	PLAU	P00749	Urokinase-type plasminogen activator	rs17231342	160726544	2	T	G	0.541	0.085	0.007	1.4E-32	trans
1654	PLAU	P00749	Urokinase-type plasminogen activator	rs2470750	98690592	3	A	T	0.607	0.066	0.007	3.6E-19	trans
1654	PLAU	P00749	Urokinase-type plasminogen activator	rs6858148	100065917	4	C	T	0.356	0.051	0.007	8.5E-12	trans
1654	PLAU	P00749	Urokinase-type plasminogen activator	rs8176741	136131461	9	A	G	0.061	0.172	0.015	1.0E-30	trans
1654	PLAU	P00749	Urokinase-type plasminogen activator	rs369705328	17848192	10	C	G	0.128	0.084	0.011	7.1E-15	trans
1654	PLAU	P00749	Urokinase-type plasminogen activator	rs55744193	75672059	10	G	A	0.993	0.815	0.042	9.4E-84	cis
1654	PLAU	P00749	Urokinase-type plasminogen activator	rs4055121	126232337	11	C	T	0.865	0.146	0.011	7.7E-44	trans
1654	PLAU	P00749	Urokinase-type plasminogen activator	rs186021206	7069412	17	A	G	0.006	1.269	0.049	2.5E-147	trans
1654	PLAU	P00749	Urokinase-type plasminogen activator	rs9948409	24761928	18	G	A	0.703	0.111	0.008	4.0E-45	trans
1654	PLAU	P00749	Urokinase-type plasminogen activator	rs36229204	44175982	19	C	T	0.968	0.528	0.021	8.4E-145	trans
1655	PLAUR	Q03405	Urokinase plasminogen activator surface receptor	rs9378247	31321425	6	G	A	0.817	0.066	0.009	2.8E-12	trans
1655	PLAUR	Q03405	Urokinase plasminogen activator surface receptor	rs8176741	136131461	9	A	G	0.061	0.105	0.015	2.8E-12	trans
1655	PLAUR	Q03405	Urokinase plasminogen activator surface receptor	rs2688623	75689615	10	T	C	0.559	0.108	0.007	1.3E-49	trans
1655	PLAUR	Q03405	Urokinase plasminogen activator surface receptor	rs2511241	72945341	11	T	C	0.929	0.139	0.014	4.9E-23	trans
1655	PLAUR	Q03405	Urokinase plasminogen activator surface receptor	rs7952602	126233669	11	G	C	0.866	0.099	0.011	1.1E-20	trans
1655	PLAUR	Q03405	Urokinase plasminogen activator surface receptor	rs186021206	7069412	17	A	G	0.006	0.908	0.050	7.8E-75	trans
1655	PLAUR	Q03405	Urokinase plasminogen activator surface receptor	rs2302524	44156472	19	T	C	0.834	0.177	0.010	1.5E-74	cis
1656	PLB1	Q6P1J6	Phospholipase B1, membrane-associated	rs80293268	8207579	1	G	C	0.952	0.151	0.013	2.9E-30	trans
1656	PLB1	Q6P1J6	Phospholipase B1, membrane-associated	rs34119886	153197742	1	A	G	0.799	0.049	0.007	3.8E-13	trans
1656	PLB1	Q6P1J6	Phospholipase B1, membrane-associated	rs6753929	28761981	2	G	C	0.747	0.987	0.008	0.0E+00	cis
1656	PLB1	Q6P1J6	Phospholipase B1, membrane-associated	rs833270	181520166	3	C	T	0.617	0.053	0.006	2.2E-21	trans
1656	PLB1	Q6P1J6	Phospholipase B1, membrane-associated	rs25914	108993021	5	C	T	0.850	0.093	0.008	2.5E-34	trans
1656	PLB1	Q6P1J6	Phospholipase B1, membrane-associated	rs2436396	139533563	5	A	G	0.299	0.049	0.006	2.1E-16	trans
1656	PLB1	Q6P1J6	Phospholipase B1, membrane-associated	rs7905367	54334653	10	C	G	0.797	0.063	0.007	3.3E-21	trans
1656	PLB1	Q6P1J6	Phospholipase B1, membrane-associated	rs11220477	126275402	11	C	T	0.958	0.113	0.013	2.7E-17	trans
1656	PLB1	Q6P1J6	Phospholipase B1, membrane-associated	rs12957090	34127020	18	C	T	0.486	0.036	0.005	1.2E-11	trans
1656	PLB1	Q6P1J6	Phospholipase B1, membrane-associated	rs12457893	60926161	18	A	C	0.543	0.048	0.005	2.1E-18	trans
1656	PLB1	Q6P1J6	Phospholipase B1, membrane-associated	rs35633871	10444166	19	G	A	0.952	0.161	0.012	4.9E-38	trans
1657	PLCB2	Q00722	1-phosphatidylinositol 4,5-bisphosphate phosphodiesterase beta-2	rs1354034	56849749	3	C	T	0.604	0.092	0.008	2.2E-33	trans
1657	PLCB2	Q00722	1-phosphatidylinositol 4,5-bisphosphate phosphodiesterase beta-2	rs11242109	131677047	5	G	T	0.519	0.052	0.007	2.8E-12	trans
1657	PLCB2	Q00722	1-phosphatidylinositol 4,5-bisphosphate phosphodiesterase beta-2	rs342298	106373646	7	C	T	0.545	0.069	0.007	5.7E-20	trans
1657	PLCB2	Q00722	1-phosphatidylinositol 4,5-bisphosphate phosphodiesterase beta-2	rs6993770	106581528	8	A	T	0.713	0.119	0.008	3.2E-47	trans
1657	PLCB2	Q00722	1-phosphatidylinositol 4,5-bisphosphate phosphodiesterase beta-2	rs4924450	40597229	15	G	A	0.294	0.153	0.008	6.4E-77	cis
1658	PLEKHO1	Q53GL0	Pleckstrin homology domain-containing family O member 1	rs72692857	150001632	1	C	T	0.165	0.078	0.010	4.3E-15	cis
1658	PLEKHO1	Q53GL0	Pleckstrin homology domain-containing family O member 1	rs1354034	56849749	3	C	T	0.604	0.078	0.008	6.7E-25	trans
1659	PLG	P00747	Plasminogen	rs1260326	27730940	2	T	C	0.392	0.086	0.007	5.2E-32	trans
1659	PLG	P00747	Plasminogen	rs9898	186390627	3	T	C	0.329	0.074	0.008	1.9E-22	trans
1659	PLG	P00747	Plasminogen	rs9274442	32633231	6	G	C	0.717	0.067	0.009	6.6E-13	trans
1659	PLG	P00747	Plasminogen	rs73015965	161127501	6	A	G	0.994	2.183	0.051	0.0E+00	cis
1659	PLG	P00747	Plasminogen	rs17145738	72982874	7	C	T	0.879	0.084	0.011	1.3E-14	trans
1660	PLIN1	O60240	Perilipin-1	rs150004289	90216446	15	G	A	0.997	0.760	0.070	4.5E-27	cis
1661	PLIN3	O60664	Perilipin-3	rs6993770	106581528	8	A	T	0.713	0.063	0.008	4.6E-14	trans
1661	PLIN3	O60664	Perilipin-3	rs262557	4872095	19	T	C	0.757	0.074	0.009	1.2E-16	cis
1662	PLPBP	O94903	Pyridoxal phosphate homeostasis protein	rs1354034	56849749	3	C	T	0.604	0.067	0.008	7.8E-19	trans
1662	PLPBP	O94903	Pyridoxal phosphate homeostasis protein	rs6993770	106581528	8	A	T	0.713	0.062	0.008	7.4E-14	trans
1663	PLSCR3	Q9NRY6	Phospholipid scramblase 3	rs7924036	65191645	10	T	G	0.501	0.056	0.007	6.1E-14	trans
1663	PLSCR3	Q9NRY6	Phospholipid scramblase 3	rs2277641	7297121	17	G	A	0.978	0.224	0.026	9.4E-18	cis
1664	PLTP	P55058	Phospholipid transfer protein	rs34211178	98383562	3	G	A	0.560	0.078	0.007	3.0E-28	trans
1664	PLTP	P55058	Phospholipid transfer protein	rs3916027	19824868	8	A	G	0.264	0.057	0.008	5.8E-13	trans
1664	PLTP	P55058	Phospholipid transfer protein	rs13284054	107669073	9	T	C	0.876	0.075	0.011	4.1E-12	trans
1664	PLTP	P55058	Phospholipid transfer protein	rs174533	61549025	11	G	A	0.654	0.058	0.007	1.9E-15	trans
1664	PLTP	P55058	Phospholipid transfer protein	rs78689694	126234820	11	G	C	0.867	0.090	0.010	3.3E-18	trans
1664	PLTP	P55058	Phospholipid transfer protein	rs147233090	44028047	15	C	T	0.977	0.174	0.023	5.4E-14	trans
1664	PLTP	P55058	Phospholipid transfer protein	rs35853021	58680643	15	T	G	0.356	0.096	0.007	1.2E-39	trans
1664	PLTP	P55058	Phospholipid transfer protein	rs247617	56990716	16	A	C	0.324	0.125	0.007	1.1E-62	trans
1664	PLTP	P55058	Phospholipid transfer protein	rs4986970	67976320	16	A	T	0.965	0.132	0.019	3.0E-12	trans
1664	PLTP	P55058	Phospholipid transfer protein	rs186021206	7069412	17	A	G	0.006	0.995	0.048	3.4E-95	trans
1664	PLTP	P55058	Phospholipid transfer protein	rs6073958	44551855	20	T	C	0.801	0.502	0.009	0.0E+00	cis
1665	PLXDC1	Q8IUK5	Plexin domain-containing protein 1	rs3775298	187150478	4	A	G	0.487	0.073	0.007	8.0E-23	trans
1665	PLXDC1	Q8IUK5	Plexin domain-containing protein 1	rs11386832	96121370	5	TA	T	0.737	0.128	0.008	4.6E-53	trans
1665	PLXDC1	Q8IUK5	Plexin domain-containing protein 1	rs885122	115331335	5	A	G	0.605	0.053	0.008	3.1E-12	trans
1665	PLXDC1	Q8IUK5	Plexin domain-containing protein 1	rs79428469	32602034	6	C	T	0.224	0.063	0.009	2.5E-12	trans
1665	PLXDC1	Q8IUK5	Plexin domain-containing protein 1	rs142988850	99764240	7	G	A	0.005	0.488	0.052	1.3E-20	trans
1665	PLXDC1	Q8IUK5	Plexin domain-containing protein 1	rs62076601	37223409	17	C	T	0.951	0.535	0.018	4.5E-198	cis
1666	PLXDC2	Q6UX71	Plexin domain-containing protein 2	rs9332635	169495433	1	C	T	0.271	0.093	0.008	3.9E-29	trans
1666	PLXDC2	Q6UX71	Plexin domain-containing protein 2	rs10185914	3690436	2	T	C	0.969	0.475	0.022	5.0E-108	trans
1666	PLXDC2	Q6UX71	Plexin domain-containing protein 2	rs1486236	180739450	2	A	C	0.373	0.069	0.008	3.3E-19	trans
1666	PLXDC2	Q6UX71	Plexin domain-containing protein 2	rs34928543	32560695	6	G	C	0.807	0.273	0.009	3.6E-182	trans
1666	PLXDC2	Q6UX71	Plexin domain-containing protein 2	rs2519093	136141870	9	C	T	0.816	0.190	0.010	3.6E-88	trans

1666	PLXDC2	Q6UX71	Plexin domain-containing protein 2	rs7919659	20584551	10	C	G	0.201	0.086	0.009	1.6E-20	cis
1666	PLXDC2	Q6UX71	Plexin domain-containing protein 2	rs186021206	7069412	17	A	G	0.006	0.962	0.050	2.7E-81	trans
1666	PLXDC2	Q6UX71	Plexin domain-containing protein 2	rs112001035	66823805	17	G	A	0.939	0.147	0.016	1.2E-20	trans
1667	PLXNA4	Q9HCM2	Plexin-A4	rs1354034	56849749	3	C	T	0.604	0.099	0.008	9.0E-38	trans
1667	PLXNA4	Q9HCM2	Plexin-A4	rs11242109	131677047	5	G	T	0.518	0.063	0.008	1.1E-16	trans
1667	PLXNA4	Q9HCM2	Plexin-A4	rs6467443	132238011	7	T	G	0.217	0.104	0.009	3.3E-29	cis
1667	PLXNA4	Q9HCM2	Plexin-A4	rs6993770	106581528	8	A	T	0.713	0.107	0.008	7.5E-38	trans
1667	PLXNA4	Q9HCM2	Plexin-A4	rs892090	55539072	19	G	T	0.834	0.081	0.010	1.5E-15	trans
1668	PLXNB2	O15031	Plexin-B2	rs7523508	177937755	1	A	G	0.723	0.051	0.007	1.1E-13	trans
1668	PLXNB2	O15031	Plexin-B2	rs1260326	27730940	2	C	T	0.609	0.050	0.006	2.8E-15	trans
1668	PLXNB2	O15031	Plexin-B2	rs1126735	58413408	3	G	A	0.369	0.053	0.006	1.4E-16	trans
1668	PLXNB2	O15031	Plexin-B2	rs9269965	32552219	6	A	G	0.606	0.055	0.007	3.5E-15	trans
1668	PLXNB2	O15031	Plexin-B2	rs2519093	136141870	9	C	T	0.815	0.118	0.008	7.6E-49	trans
1668	PLXNB2	O15031	Plexin-B2	rs56278466	17875857	10	G	T	0.661	0.189	0.007	6.0E-181	trans
1668	PLXNB2	O15031	Plexin-B2	rs112635299	94838142	14	T	G	0.022	0.146	0.021	6.2E-12	trans
1668	PLXNB2	O15031	Plexin-B2	rs186021206	7069412	17	A	G	0.006	0.315	0.043	1.5E-13	trans
1668	PLXNB2	O15031	Plexin-B2	rs1801689	64210580	17	C	A	0.030	0.145	0.018	9.8E-16	trans
1668	PLXNB2	O15031	Plexin-B2	rs868876	7831226	19	A	T	0.699	0.059	0.007	2.7E-18	trans
1668	PLXNB2	O15031	Plexin-B2	rs11547731	50722134	22	T	C	0.347	0.713	0.008	0.0E+00	cis
1669	PLXNB3	Q9ULL4	Plexin-B3	rs9402686	135427817	6	A	G	0.271	0.061	0.008	6.0E-13	trans
1669	PLXNB3	Q9ULL4	Plexin-B3	rs6993770	106581528	8	A	T	0.713	0.058	0.008	2.7E-12	trans
1669	PLXNB3	Q9ULL4	Plexin-B3	rs409801	4744743	9	C	T	0.507	0.059	0.008	4.0E-15	trans
1669	PLXNB3	Q9ULL4	Plexin-B3	rs4502008	126287754	11	G	T	0.478	0.061	0.008	7.5E-16	trans
1669	PLXNB3	Q9ULL4	Plexin-B3	rs892090	55539072	19	G	T	0.834	0.077	0.010	5.0E-14	trans
1669	PLXNB3	Q9ULL4	Plexin-B3	rs731416	153031839	X	C	T	0.146	0.154	0.009	7.0E-66	cis
1670	PM20D1	Q6GTS8	N-fatty-acyl-amino acid synthase/hydrolase PM20D1	rs760077	155178782	1	A	T	0.395	0.074	0.008	1.8E-21	trans
1670	PM20D1	Q6GTS8	N-fatty-acyl-amino acid synthase/hydrolase PM20D1	rs112268616	205816893	1	T	C	0.660	0.840	0.008	0.0E+00	cis
1670	PM20D1	Q6GTS8	N-fatty-acyl-amino acid synthase/hydrolase PM20D1	rs11680123	60514748	2	G	A	0.593	0.124	0.006	4.9E-97	trans
1670	PM20D1	Q6GTS8	N-fatty-acyl-amino acid synthase/hydrolase PM20D1	rs121908120	219755011	2	T	A	0.972	0.121	0.018	1.6E-11	trans
1670	PM20D1	Q6GTS8	N-fatty-acyl-amino acid synthase/hydrolase PM20D1	rs11707641	12155888	3	C	T	0.162	0.057	0.008	2.7E-13	trans
1670	PM20D1	Q6GTS8	N-fatty-acyl-amino acid synthase/hydrolase PM20D1	rs2519093	136141870	9	C	T	0.816	0.057	0.007	3.2E-14	trans
1670	PM20D1	Q6GTS8	N-fatty-acyl-amino acid synthase/hydrolase PM20D1	rs12764992	96997175	10	G	A	0.418	0.046	0.006	9.9E-15	trans
1670	PM20D1	Q6GTS8	N-fatty-acyl-amino acid synthase/hydrolase PM20D1	rs34560261	90734426	15	C	T	0.830	0.062	0.008	2.2E-15	trans
1671	PMM2	O15305	Phosphomannomutase 2	rs34258285	8906914	16	A	C	0.975	0.680	0.024	5.6E-172	cis
1672	PMVK	Q15126	Phosphomevalonate kinase	rs114951074	154903917	1	C	T	0.993	0.658	0.047	2.3E-45	cis
1672	PMVK	Q15126	Phosphomevalonate kinase	rs1354034	56849749	3	C	T	0.603	0.056	0.008	6.5E-13	trans
1672	PMVK	Q15126	Phosphomevalonate kinase	rs342298	106373646	7	C	T	0.546	0.070	0.008	6.0E-20	trans
1672	PMVK	Q15126	Phosphomevalonate kinase	rs6993770	106581528	8	A	T	0.713	0.066	0.008	4.9E-15	trans
1672	PMVK	Q15126	Phosphomevalonate kinase	rs7896518	65104500	10	G	A	0.424	0.054	0.008	4.2E-12	trans
1673	PNLIP	P16233	Pancreatic triacylglycerol lipase	rs333947	110470764	1	G	A	0.851	0.078	0.010	3.6E-14	trans
1673	PNLIP	P16233	Pancreatic triacylglycerol lipase	rs2487569	120442257	1	A	T	0.887	0.082	0.012	1.8E-12	trans
1673	PNLIP	P16233	Pancreatic triacylglycerol lipase	rs17032925	67266483	2	T	C	0.917	0.178	0.013	2.2E-40	trans
1673	PNLIP	P16233	Pancreatic triacylglycerol lipase	rs66473811	64000464	3	C	T	0.166	0.083	0.010	6.0E-17	trans
1673	PNLIP	P16233	Pancreatic triacylglycerol lipase	rs10213531	110855049	4	A	G	0.656	0.061	0.008	2.9E-15	trans
1673	PNLIP	P16233	Pancreatic triacylglycerol lipase	rs2854008	31312538	6	G	A	0.696	0.060	0.008	1.0E-13	trans
1673	PNLIP	P16233	Pancreatic triacylglycerol lipase	rs6571015	96534478	6	A	G	0.791	0.146	0.009	1.6E-58	trans
1673	PNLIP	P16233	Pancreatic triacylglycerol lipase	rs9482772	127446790	6	C	T	0.450	0.084	0.007	9.9E-30	trans
1673	PNLIP	P16233	Pancreatic triacylglycerol lipase	rs4921967	18719629	8	G	T	0.313	0.079	0.008	1.4E-23	trans
1673	PNLIP	P16233	Pancreatic triacylglycerol lipase	rs4733612	129569999	8	A	G	0.733	0.060	0.008	3.4E-13	trans
1673	PNLIP	P16233	Pancreatic triacylglycerol lipase	rs8176749	136131188	9	T	C	0.061	0.503	0.015	2.2E-234	trans
1673	PNLIP	P16233	Pancreatic triacylglycerol lipase	rs174548	61571348	11	G	C	0.315	0.066	0.008	3.8E-17	trans
1673	PNLIP	P16233	Pancreatic triacylglycerol lipase	rs61910478	100592308	11	A	T	0.731	0.075	0.008	6.1E-20	trans
1673	PNLIP	P16233	Pancreatic triacylglycerol lipase	rs72802342	75234872	16	C	A	0.924	0.283	0.014	8.4E-89	trans
1673	PNLIP	P16233	Pancreatic triacylglycerol lipase	rs1673931	88976477	16	T	C	0.622	0.123	0.008	3.1E-59	trans
1673	PNLIP	P16233	Pancreatic triacylglycerol lipase	rs601338	49206674	19	A	G	0.509	0.168	0.007	3.6E-114	trans
1674	PNLIPRP1	P54315	Inactive pancreatic lipase-related protein 1	rs35004807	51108411	1	TGTAA	T	0.345	0.061	0.008	1.6E-15	trans
1674	PNLIPRP1	P54315	Inactive pancreatic lipase-related protein 1	rs2027447	120440029	1	T	C	0.889	0.095	0.012	2.0E-16	trans
1674	PNLIPRP1	P54315	Inactive pancreatic lipase-related protein 1	rs11724862	186572503	4	T	C	0.764	0.068	0.009	4.5E-15	trans
1674	PNLIPRP1	P54315	Inactive pancreatic lipase-related protein 1	rs35536837	126688588	6	C	CT	0.548	0.079	0.007	1.1E-27	trans
1674	PNLIPRP1	P54315	Inactive pancreatic lipase-related protein 1	rs7800611	51019662	7	G	A	0.954	0.126	0.017	2.0E-13	trans
1674	PNLIPRP1	P54315	Inactive pancreatic lipase-related protein 1	rs6966838	117083580	7	C	T	0.368	0.059	0.007	1.9E-15	trans
1674	PNLIPRP1	P54315	Inactive pancreatic lipase-related protein 1	rs3812304	158871744	7	G	A	0.768	0.063	0.008	1.5E-13	trans
1674	PNLIPRP1	P54315	Inactive pancreatic lipase-related protein 1	rs2131181	18720624	8	C	T	0.318	0.080	0.008	3.8E-25	trans
1674	PNLIPRP1	P54315	Inactive pancreatic lipase-related protein 1	rs28456	61589481	11	G	A	0.315	0.059	0.008	1.4E-14	trans
1674	PNLIPRP1	P54315	Inactive pancreatic lipase-related protein 1	rs686056	100646689	11	G	A	0.677	0.089	0.008	3.2E-31	trans
1674	PNLIPRP1	P54315	Inactive pancreatic lipase-related protein 1	rs9579128	28481938	13	T	C	0.589	0.050	0.007	8.6E-12	trans
1674	PNLIPRP1	P54315	Inactive pancreatic lipase-related protein 1	rs72802342	75234872	16	C	A	0.924	0.663	0.014	0.0E+00	trans
1674	PNLIPRP1	P54315	Inactive pancreatic lipase-related protein 1	rs1126464	89704365	16	G	C	0.756	0.099	0.009	9.1E-31	trans
1674	PNLIPRP1	P54315	Inactive pancreatic lipase-related protein 1	rs17138478	36073320	17	A	C	0.131	0.096	0.011	2.0E-19	trans
1674	PNLIPRP1	P54315	Inactive pancreatic lipase-related protein 1	rs62090602	42413766	18	T	A	0.077	0.097	0.013	5.4E-13	trans
1674	PNLIPRP1	P54315	Inactive pancreatic lipase-related protein 1	rs2836255	39634196	21	G	A	0.516	0.053	0.007	1.8E-13	trans
1674	PNLIPRP1	P54315	Inactive pancreatic lipase-related protein 1	rs80184311	37908610	22	C	G	0.106	0.080	0.012	4.0E-12	trans
1675	PNLIPRP2	P54317	Pancreatic lipase-related protein 2	rs8176741	136131461	9	A	G	0.061	0.205	0.010	3.1E-95	trans
1675	PNLIPRP2	P54317	Pancreatic lipase-related protein 2	rs56278466	17875857	10	G	T	0.662	0.057	0.008	1.1E-12	trans
1675	PNLIPRP2	P54317	Pancreatic lipase-related protein 2	rs4751995	118397884	10	G	A	0.519	1.136	0.008	0.0E+00	cis
1675	PNLIPRP2	P54317	Pancreatic lipase-related protein 2	rs72802342	75234872	16	C	A	0.924	0.119	0.009	4.9E-39	trans
1675	PNLIPRP2	P54317	Pancreatic lipase-related protein 2	rs601338	49206674	19	A	G	0.510	0.160	0.005	2.9E-242	trans
1676	PNMA1	Q8ND90	Paraneoplastic antigen Ma1	rs342293	106372219	7	C	G	0.540	0.055	0.008	5.5E-13	trans
1676	PNMA1	Q8ND90	Paraneoplastic antigen Ma1	rs113315674	100720053	9	A	G	0.365	0.057	0.008	6.0E-13	trans
1676	PNMA1	Q8ND90	Paraneoplastic antigen Ma1	rs140960424	35034359	10	GGAAAGAGAGAGA	G	0.735	0.173	0.009	3.3E-88	trans
1676	PNMA1	Q8ND90	Paraneoplastic antigen Ma1	rs35564315	2007315	19	A	C	0.167	0.071	0.010	5.1E-12	trans
1677	PNPT1	Q8TCS8	Polyribonucleotide nucleotidyltransferase 1, mitochondrial	rs9394070	31366295	6	C	A	0.935	0.143	0.016	3.2E-20	trans
1677	PNPT1	Q8TCS8	Polyribonucleotide nucleotidyltransferase 1, mitochondrial	rs6001857	22689698	22	T	C	0.833	0.084	0.010	1.8E-16	trans
1678	PODXL	O00592	Podocalyxin	rs767298	7274584	7	G	T	0.366	0.161	0.008	1.0E-97	trans
1678	PODXL	O00592	Podocalyxin	rs3212298	131193739	7	C	T	0.956	0.549	0.018	6.3E-207	cis
1678	PODXL	O00592	Podocalyxin	rs590666	79103366	9	T	C	0.481	0.098	0.008	9.1E-39	trans
1678	PODXL	O00592	Podocalyxin	rs2519093	136141870	9	C	T	0.816	0.549	0.010	0.0E+00	trans
1678	PODXL	O00592	Podocalyxin	rs34434834	126307796	11	A	G	0.034	0.332	0.020	2.1E-61	trans
1678	PODXL	O00592	Podocalyxin	rs186021206	7069412	17	A	G	0.006	0.863	0.050	1.1E-66	trans
1679	PODXL2	Q9NZ53	Podocalyxin-like protein 2	rs35196180	161590903	1	CG	C	0.137	0.078	0.011	8.6E-13	trans
1679	PODXL2	Q9NZ53	Podocalyxin-like protein 2	rs61747728	179526214	1	T	C	0.038	0.129	0.019	1.5E-11	trans
1679	PODXL2	Q9NZ53	Podocalyxin-like protein 2	rs1047891	211540507	2	A	C	0.313	0.055	0.008	2.8E-12	trans
1679	PODXL2	Q9NZ53	Podocalyxin-like protein 2	rs781656	57778645	4	A	G	0.184	0.169	0.009	4.0E-71	trans
1679	PODXL2	Q9NZ53	Podocalyxin-like protein 2	rs1129187	42932200	6	G	T	0.547	0.051	0.007	7.3E-12	trans
1679	PODXL2	Q9NZ53	Podocalyxin-like protein 2	rs635634	136155000	9	T	C	0.184	0.073	0.010	2.3E-14	trans
1679	PODXL2	Q9NZ53	Podocalyxin-like protein 2	rs74841302	42084329	15	G	A	0.983	0.198	0.028	1.7E-12	trans
1679	PODXL2	Q9NZ53	Podocalyxin-like protein 2	rs4781978	17562883	16	G	C	0.212	0.117	0.009	3.7E-38	trans
1679	PODXL2	Q9NZ53	Podocalyxin-like protein 2	rs11869940	45578435	17	A	T	0.495	0.051	0.007	6.5E-12	trans
1679	PODXL2	Q9NZ53	Podocalyxin-like protein 2	rs6504649	48437456	17	C	G	0.612	0.117	0.008	2.7E-55	trans
1679	PODXL2	Q9NZ53	Podocalyxin-like protein 2	rs55639531	394341	18	G	C	0.286	0.058	0.008	7.1E-13	trans
1679	PODXL2	Q9NZ53	Podocalyxin-like protein 2	rs45512696	35550878	19	C	T	0.824	0.073	0.010	3.4E-14	trans
1680	POF1B	Q8WVV4	Protein POF1B	rs62142192	30975961	2	T	G	0.202	0.083	0.010	2.3E-18	trans
1680	POF1B	Q8WVV4	Protein POF1B	rs11898628	101751685	2	G	A	0.150	0.077	0.011	5.8E-13	trans
1680	POF1B	Q8WVV4	Protein POF1B	rs13208948	32646454	6	C	T	0.736	0.065	0.009	1.3E-12	trans
1680	POF1B	Q8WVV4	Protein POF1B	rs363774	84563135	X	T	A	0.228	0.122	0.008	1.3E-59	cis
1681	POMC	P01189	Pro-opiomelanocortin	rs6680345	101735513	1	T	C	0.508	0.123	0.007	1.7E-63	trans
1681	POMC	P01189	Pro-opiomelanocortin	rs3754861	25393722	2	C	A	0.071	0.145	0.014	9.6E-24	cis
1681	POMC	P01189	Pro-opiomelanocortin	rs2594950	177508622	2	C	G	0.344	0.078	0.008	7.0E-24	trans
1681	POMC	P01189	Pro-opiomelanocortin	rs9472040	43348512	6	A	G	0.682	0.053	0.008	9.5E-12	trans
1681	POMC	P01189	Pro-opiomelanocortin	rs13199674	127185489	6	G	A	0.557	0.051	0.007	2.7E-12	trans
1681	POMC	P01189	Pro-opiomelanocortin	rs8045100	81603400	16	C	T	0.457	0.180	0.007	2.2E-133	trans
1681	POMC	P01189	Pro-opiomelanocortin	rs877127	13911655	18	T	A	0.678	0.065	0.008	6.0E-17	trans
1681	POMC	P01189	Pro-opiomelanocortin	rs145530718	10568883	19	C	G	0.020	0.346	0.026	3.2E-39	trans
1681	POMC	P01189	Pro-opiomelanocortin	rs4408777	62706105	20	A	G	0.562	0.087	0.007	9.1E-33	trans
1682	PON1	P27169	Serum paraoxonase/arylesterase 1	rs6671730	2339139	1	A	G	0.437	0.064	0.006	2.2E-27	trans
1682	PON1	P27169	Serum paraoxonase/arylesterase 1	rs705379	94953895	7	G	A	0.523	0.757	0.008	0.0E+00	cis
1682	PON1	P27169	Serum paraoxonase/arylesterase 1	rs305	19817401	8	G	A	0.234	0.052	0.007	6.1E-14	trans
1682	PON1	P27169	Serum paraoxonase/arylesterase 1	rs173539	56988044	16	T	C	0.332	0.060	0.006	4.4E-22	trans
1683	PON2	Q15165	Serum paraoxonase/arylesterase 2	rs79654026	196725939	1	G	A	0.181	0.081	0.008	8.8E-24	trans
1683	PON2	Q15165	Serum paraoxonase/arylesterase 2	rs6961624	95037661	7	T	C	0.762	0.903	0.009	0.0E+00	cis
1683	PON2	Q15165	Serum paraoxonase/arylesterase 2	rs28929474	94844947	14	T	C	0.021	0.206	0.021	1.4E-22	trans
1683	PON2	Q15165	Serum paraoxonase/arylesterase 2	rs58542926	19379549	19	T	C	0.076	0.154	0.011	5.1E-42	trans
1684	PON3	Q15166	Serum paraoxonase/lactonase 3	rs16840639	196824773	1	C	T	0.195	0.085	0.009	2.2E-22	trans
1684	PON3	Q15166	Serum paraoxonase/lactonase 3	rs149867961	95025744	7	T	C	0.974	1.051	0.023	0.0E+00	cis
1684	PON3	Q15166	Serum paraoxonase/lactonase 3	rs4240624	9184231	8	A	G	0.908	0.094	0.012	6.1E-15	trans
1684	PON3	Q15166	Serum paraoxonase/lactonase 3	rs3208305	19823648	8	T	A	0.286	0.058	0.008	5.9E-14	trans
1684	PON3	Q15166	Serum paraoxonase/lactonase 3	rs1077835	58723426	15	G	A	0.222	0.057	0.008	1.2E-11	trans
1684	PON3	Q15166	Serum paraoxonase/lactonase 3	rs247617	56990716	16	A	C	0.324	0.071	0.007	3.7E-21	trans
1684	PON3	Q15166	Serum paraoxonase/lactonase 3	rs149394327	64228995	17	C	G	0.030	0.189	0.021	3.5E-20	trans
1684	PON3	Q15166	Serum paraoxonase/lactonase 3	rs34415062	59023166	19	A	G	0.179	0.160	0.009	1.3E-68	trans
1685	POSTN	Q15063	Periostin	rs59410274	52561441	3	G	C	0.994	0.317	0.045	2.6E-12	trans
1685	POSTN	Q15063	Periostin	rs74735511	132269004	3	A	G	0.911	0.094	0.013	1.7E-13	trans
1685	POSTN	Q15063	Periostin	rs4705926	131561758	5	C	T	0.536	0.058	0.007	1.4E-15	trans
1685	POSTN	Q15063	Periostin	rs2389858	17908130	7	T	A	0.510	0.079	0.007	1.3E-27	trans
1686	PPBP	P02775	Platelet basic protein	rs12086222	156869630	1	C	G	0.111	0.085	0.012	1.2E-12	trans
1686	PPBP	P02775	Platelet basic protein	rs13412535	224874874	2	G	A	0.769	0.072	0.009	4.9E-16	trans
1686	PPBP	P02775	Platelet basic protein	rs7618405	18250509	3	C	A	0.795	0.065	0.009	3.6E-12	trans
1686	PPBP	P02775	Platelet basic protein	rs1354034	56849749	3	T	C	0.395	0.076	0.008	2.9E-23	trans
1686	PPBP	P02775	Platelet basic protein	rs10016018	102794621	4	A	T	0.603	0.069	0.008	1.8E-19	trans
1686	PPBP	P02775	Platelet basic protein	rs2516471	31400292	6	G	A	0.937	0.120	0.016	1.1E-14	trans
1686	PPBP	P02775	Platelet basic protein	rs6961069	80218961	7	T	C	0.403	0.071	0.008	2.0E-20	trans
1686	PPBP	P02775	Platelet basic protein	rs6993770	106581528	8	A	T	0.713	0.128	0.008	4.6E-54	trans
1686	PPBP	P02775	Platelet basic protein	rs10820606	99192919	9	A	C	0.770	0.063	0.009	3.2E-12	trans

1686	PPBP	P02775	Platelet basic protein	rs7080386	65048306	10	A	C	0.412	0.127	0.008	4.8E-62	trans
1686	PPBP	P02775	Platelet basic protein	rs12445050	81870969	16	T	C	0.138	0.102	0.011	3.7E-21	trans
1686	PPBP	P02775	Platelet basic protein	rs892090	55539072	19	G	T	0.834	0.163	0.010	4.9E-58	trans
1686	PPBP	P02775	Platelet basic protein	rs6081569	19303786	20	C	G	0.369	0.072	0.008	1.2E-20	trans
1686	PPBP	P02775	Platelet basic protein	rs2096256	99924813	X	G	A	0.342	0.044	0.007	1.2E-11	trans
1687	PPCDC	Q96CD2	Phosphopantothenoylcysteine decarboxylase	rs1354034	56849749	3	C	T	0.604	0.051	0.007	3.5E-12	trans
1687	PPCDC	Q96CD2	Phosphopantothenoylcysteine decarboxylase	rs12591994	75320306	15	G	A	0.065	0.670	0.015	0.0E+00	cis
1688	PPIB	P23284	Peptidyl-prolyl cis-trans isomerase B	rs1354034	56849749	3	C	T	0.604	0.054	0.008	3.0E-12	trans
1688	PPIB	P23284	Peptidyl-prolyl cis-trans isomerase B	rs6993770	106581528	8	A	T	0.713	0.076	0.008	5.8E-20	trans
1688	PPIB	P23284	Peptidyl-prolyl cis-trans isomerase B	rs892090	55539072	19	G	T	0.834	0.074	0.010	3.2E-13	trans
1689	PPIF	P30405	Peptidyl-prolyl cis-trans isomerase F, mitochondrial	rs6993770	106581528	8	A	T	0.714	0.064	0.008	1.8E-14	trans
1690	PPL	O60437	Periplakin	rs151830	96057882	5	A	G	0.606	0.056	0.008	8.6E-13	trans
1690	PPL	O60437	Periplakin	rs61499243	5000212	16	T	C	0.111	0.137	0.012	1.3E-28	cis
1691	PPM1B	O75688	Protein phosphatase 1B	rs4548048	32625022	6	T	C	0.103	0.120	0.013	1.3E-19	trans
1692	PPM1F	P49593	Protein phosphatase 1F	rs1354034	56849749	3	C	T	0.604	0.059	0.008	1.2E-14	trans
1692	PPM1F	P49593	Protein phosphatase 1F	rs342298	106373646	7	C	T	0.545	0.056	0.008	1.4E-13	trans
1692	PPM1F	P49593	Protein phosphatase 1F	rs6993770	106581528	8	A	T	0.713	0.070	0.008	3.2E-17	trans
1692	PPM1F	P49593	Protein phosphatase 1F	rs5750281	22327787	22	C	T	0.957	0.126	0.018	8.6E-12	cis
1693	PPME1	Q9Y570	Protein phosphatase methylesterase 1	rs3782123	205198	11	A	C	0.734	0.058	0.008	3.8E-12	trans
1694	PPP1R12A	O14974	Protein phosphatase 1 regulatory subunit 12A	rs68066031	224880498	2	C	T	0.230	0.109	0.009	6.6E-34	trans
1694	PPP1R12A	O14974	Protein phosphatase 1 regulatory subunit 12A	rs1354034	56849749	3	C	T	0.604	0.055	0.008	7.3E-13	trans
1694	PPP1R12A	O14974	Protein phosphatase 1 regulatory subunit 12A	rs17622656	131820997	5	G	A	0.612	0.052	0.008	1.6E-11	trans
1694	PPP1R12A	O14974	Protein phosphatase 1 regulatory subunit 12A	rs342298	106373646	7	C	T	0.545	0.067	0.008	6.6E-19	trans
1694	PPP1R12A	O14974	Protein phosphatase 1 regulatory subunit 12A	rs6993770	106581528	8	A	T	0.714	0.057	0.008	3.8E-12	trans
1695	PPP1R12B	O60237	Protein phosphatase 1 regulatory subunit 12B	rs68066031	224880498	2	C	T	0.230	0.073	0.009	2.1E-15	trans
1696	PPP1R14A	Q96A00	Protein phosphatase 1 regulatory subunit 14A	rs342298	106373646	7	C	T	0.545	0.064	0.008	1.5E-17	trans
1696	PPP1R14A	Q96A00	Protein phosphatase 1 regulatory subunit 14A	rs6993770	106581528	8	A	T	0.714	0.056	0.008	1.1E-11	trans
1696	PPP1R14A	Q96A00	Protein phosphatase 1 regulatory subunit 14A	rs71354995	38791841	19	A	G	0.744	0.226	0.009	1.5E-151	cis
1697	PPP1R14D	Q9NXH3	Protein phosphatase 1 regulatory subunit 14D	rs9271657	32592582	6	C	T	0.214	0.075	0.010	8.2E-15	trans
1697	PPP1R14D	Q9NXH3	Protein phosphatase 1 regulatory subunit 14D	rs11855641	41088017	15	G	A	0.340	0.061	0.008	1.6E-14	cis
1698	PPP1R2	P41236	Protein phosphatase inhibitor 2	rs1354034	56849749	3	C	T	0.604	0.079	0.008	3.3E-25	trans
1698	PPP1R2	P41236	Protein phosphatase inhibitor 2	rs7080386	65048306	10	A	C	0.412	0.053	0.008	2.3E-12	trans
1698	PPP1R2	P41236	Protein phosphatase inhibitor 2	rs11502185	180258	11	C	T	0.259	0.074	0.010	1.8E-14	trans
1698	PPP1R2	P41236	Protein phosphatase inhibitor 2	rs892090	55539072	19	G	T	0.834	0.069	0.010	6.9E-12	trans
1699	PPP1R9B	Q96SB3	Neurabin-2	rs1354034	56849749	3	C	T	0.604	0.068	0.007	2.1E-19	trans
1699	PPP1R9B	Q96SB3	Neurabin-2	rs342296	106372903	7	G	A	0.544	0.051	0.007	4.0E-12	trans
1700	PPP2R5A	Q15172	Serine/threonine-protein phosphatase 2A 56 kDa regulatory subunit alpha isoform	rs351409	212459257	1	A	G	0.979	0.301	0.026	2.8E-30	cis
1700	PPP2R5A	Q15172	Serine/threonine-protein phosphatase 2A 56 kDa regulatory subunit alpha isoform	rs6993770	106581528	8	A	T	0.713	0.064	0.008	8.8E-15	trans
1701	PPP3R1	P63098	Calcineurin subunit B type 1	rs188468174	25291697	1	C	T	0.986	0.315	0.032	6.8E-23	trans
1701	PPP3R1	P63098	Calcineurin subunit B type 1	rs1354034	56849749	3	C	T	0.604	0.054	0.008	1.3E-12	trans
1701	PPP3R1	P63098	Calcineurin subunit B type 1	rs55768089	102743698	4	A	T	0.310	0.105	0.008	4.5E-38	trans
1701	PPP3R1	P63098	Calcineurin subunit B type 1	rs915651	31749184	6	G	A	0.870	0.086	0.011	1.4E-14	trans
1701	PPP3R1	P63098	Calcineurin subunit B type 1	rs9414801	65149089	10	G	A	0.467	0.064	0.008	2.0E-17	trans
1702	PPY	P01298	Pancreatic prohormone	rs1493691	120531973	1	G	A	0.892	0.106	0.012	2.6E-19	trans
1702	PPY	P01298	Pancreatic prohormone	rs73174306	169194244	3	T	A	0.042	0.415	0.018	1.2E-113	trans
1702	PPY	P01298	Pancreatic prohormone	rs6451770	44691395	5	T	G	0.621	0.063	0.007	3.3E-17	trans
1702	PPY	P01298	Pancreatic prohormone	rs9348441	20680678	6	T	A	0.739	0.066	0.008	1.8E-15	trans
1702	PPY	P01298	Pancreatic prohormone	rs9375435	126661858	6	C	T	0.547	0.067	0.007	4.2E-20	trans
1702	PPY	P01298	Pancreatic prohormone	rs184163936	156781472	7	T	C	0.993	0.366	0.047	5.4E-15	trans
1702	PPY	P01298	Pancreatic prohormone	rs2438988	49370090	10	A	G	0.365	0.217	0.008	3.2E-163	trans
1702	PPY	P01298	Pancreatic prohormone	rs9320010	53053897	18	G	A	0.399	0.052	0.007	3.1E-12	trans
1703	PRAP1	Q96NZ9	Proline-rich acidic protein 1	rs687339	135932359	3	T	C	0.771	0.084	0.008	1.1E-23	trans
1703	PRAP1	Q96NZ9	Proline-rich acidic protein 1	rs143674922	135161903	10	G	A	0.982	1.298	0.028	0.0E+00	cis
1703	PRAP1	Q96NZ9	Proline-rich acidic protein 1	rs681343	49206462	19	C	T	0.491	0.119	0.007	9.6E-64	trans
1704	PRCP	P42785	Lysosomal Pro-X carboxypeptidase	rs3010061	183824869	1	T	C	0.200	0.061	0.009	7.7E-12	trans
1704	PRCP	P42785	Lysosomal Pro-X carboxypeptidase	rs11125916	62609378	2	A	C	0.195	0.065	0.009	2.4E-13	trans
1704	PRCP	P42785	Lysosomal Pro-X carboxypeptidase	rs13111888	77131294	4	A	G	0.178	0.064	0.009	9.6E-12	trans
1704	PRCP	P42785	Lysosomal Pro-X carboxypeptidase	rs7834466	10634805	8	G	T	0.390	0.067	0.007	4.9E-20	trans
1704	PRCP	P42785	Lysosomal Pro-X carboxypeptidase	rs10104003	55422440	8	T	C	0.210	0.166	0.009	3.7E-81	trans
1704	PRCP	P42785	Lysosomal Pro-X carboxypeptidase	rs10116966	33122370	9	T	A	0.690	0.081	0.008	5.8E-26	trans
1704	PRCP	P42785	Lysosomal Pro-X carboxypeptidase	rs56278466	17875857	10	G	T	0.661	0.141	0.007	1.1E-79	trans
1704	PRCP	P42785	Lysosomal Pro-X carboxypeptidase	rs145078947	93652974	14	T	G	0.003	1.052	0.070	2.9E-51	trans
1705	PRDX1	Q06830	Peroxiredoxin-1	rs2356552	46009316	1	C	T	0.898	0.109	0.012	3.0E-18	cis
1705	PRDX1	Q06830	Peroxiredoxin-1	rs6993770	106581528	8	A	T	0.714	0.066	0.008	1.8E-15	trans
1706	PRDX2	P32119	Peroxiredoxin-2	rs11759553	135422296	6	A	T	0.727	0.063	0.008	5.3E-15	trans
1707	PRDX3	P30048	Thioredoxin-dependent peroxide reductase, mitochondrial	rs7358070	120956728	10	G	C	0.675	0.065	0.008	2.7E-15	cis
1708	PRDX5	P30044	Peroxiredoxin-5, mitochondrial	rs77269065	64087269	11	A	G	0.005	0.980	0.053	3.5E-77	cis
1709	PRDX6	P30041	Peroxiredoxin-6	rs33951697	173458326	1	T	C	0.185	0.174	0.010	6.6E-74	cis
1710	PRELP	P51888	Prolargin	rs41313926	203452409	1	G	A	0.990	2.271	0.035	0.0E+00	cis
1711	PRG2	P13727	Bone marrow proteoglycan	rs1260326	27730940	2	C	T	0.608	0.052	0.008	7.1E-12	trans
1711	PRG2	P13727	Bone marrow proteoglycan	rs1186706	61673394	2	G	T	0.509	0.135	0.007	4.1E-75	trans
1711	PRG2	P13727	Bone marrow proteoglycan	rs10208293	102966310	2	G	A	0.720	0.057	0.008	3.5E-12	trans
1711	PRG2	P13727	Bone marrow proteoglycan	rs11689257	162958353	2	G	A	0.931	0.102	0.015	5.6E-12	trans
1711	PRG2	P13727	Bone marrow proteoglycan	rs1047891	211540507	2	A	C	0.313	0.058	0.008	2.8E-13	trans
1711	PRG2	P13727	Bone marrow proteoglycan	rs12619285	213824045	2	A	G	0.734	0.063	0.008	2.6E-14	trans
1711	PRG2	P13727	Bone marrow proteoglycan	rs900175	14280232	3	T	G	0.656	0.065	0.008	7.4E-17	trans
1711	PRG2	P13727	Bone marrow proteoglycan	rs4328821	128316435	3	A	G	0.893	0.244	0.012	2.0E-94	trans
1711	PRG2	P13727	Bone marrow proteoglycan	rs11405616	141510261	5	CA	C	0.606	0.052	0.008	5.4E-12	trans
1711	PRG2	P13727	Bone marrow proteoglycan	rs76641566	50284557	7	G	A	0.927	0.095	0.014	8.8E-12	trans
1711	PRG2	P13727	Bone marrow proteoglycan	rs13256023	61648551	8	C	T	0.815	0.108	0.009	7.8E-31	trans
1711	PRG2	P13727	Bone marrow proteoglycan	rs7840212	130599247	8	C	T	0.662	0.069	0.008	5.8E-19	trans
1711	PRG2	P13727	Bone marrow proteoglycan	rs1888909	6197392	9	T	C	0.251	0.057	0.008	1.0E-11	trans
1711	PRG2	P13727	Bone marrow proteoglycan	rs10823305	70906916	10	A	G	0.830	0.068	0.010	1.5E-12	trans
1711	PRG2	P13727	Bone marrow proteoglycan	rs734095	2323198	11	C	G	0.888	0.080	0.012	4.7E-12	trans
1711	PRG2	P13727	Bone marrow proteoglycan	rs10896615	57164280	11	C	T	0.124	0.146	0.011	1.6E-39	cis
1711	PRG2	P13727	Bone marrow proteoglycan	rs12431998	21432455	14	G	A	0.813	0.085	0.009	9.3E-20	trans
1711	PRG2	P13727	Bone marrow proteoglycan	rs423135	89740873	16	A	G	0.517	0.053	0.007	6.2E-13	trans
1711	PRG2	P13727	Bone marrow proteoglycan	rs35617692	56272501	17	A	C	0.011	0.416	0.035	3.1E-33	trans
1711	PRG2	P13727	Bone marrow proteoglycan	rs4807601	4547917	19	A	G	0.499	0.153	0.007	1.9E-93	trans
1711	PRG2	P13727	Bone marrow proteoglycan	rs146776010	40225031	19	G	C	0.994	0.530	0.048	1.5E-28	trans
1711	PRG2	P13727	Bone marrow proteoglycan	rs2242892	36395974	21	G	A	0.929	0.105	0.014	1.8E-13	trans
1712	PRG3	Q9Y2Y8	Proteoglycan 3	rs1434282	199010721	1	T	C	0.726	0.077	0.008	1.5E-20	trans
1712	PRG3	Q9Y2Y8	Proteoglycan 3	rs7587389	61649791	2	G	A	0.518	0.198	0.007	1.8E-158	trans
1712	PRG3	Q9Y2Y8	Proteoglycan 3	rs1420101	102957716	2	T	C	0.386	0.058	0.008	3.2E-14	trans
1712	PRG3	Q9Y2Y8	Proteoglycan 3	rs6740627	202654841	2	G	A	0.158	0.077	0.010	3.0E-14	trans
1712	PRG3	Q9Y2Y8	Proteoglycan 3	rs12619285	213824045	2	A	G	0.734	0.094	0.008	1.9E-29	trans
1712	PRG3	Q9Y2Y8	Proteoglycan 3	rs2596896	13013763	3	T	G	0.325	0.067	0.008	2.0E-17	trans
1712	PRG3	Q9Y2Y8	Proteoglycan 3	rs13089722	128306757	3	G	A	0.894	0.250	0.012	9.8E-99	trans
1712	PRG3	Q9Y2Y8	Proteoglycan 3	rs7449456	131953427	5	T	C	0.189	0.069	0.009	1.2E-13	trans
1712	PRG3	Q9Y2Y8	Proteoglycan 3	rs11405616	141510261	5	CA	C	0.606	0.054	0.008	7.0E-13	trans
1712	PRG3	Q9Y2Y8	Proteoglycan 3	rs1136688	29910761	6	G	A	0.570	0.057	0.007	2.3E-14	trans
1712	PRG3	Q9Y2Y8	Proteoglycan 3	rs76641566	50284557	7	G	A	0.927	0.148	0.014	3.1E-26	trans
1712	PRG3	Q9Y2Y8	Proteoglycan 3	rs513973	105314326	7	T	A	0.229	0.072	0.009	2.2E-15	trans
1712	PRG3	Q9Y2Y8	Proteoglycan 3	rs12539299	148888534	7	C	T	0.384	0.055	0.008	2.3E-13	trans
1712	PRG3	Q9Y2Y8	Proteoglycan 3	rs13256023	61648551	8	C	T	0.815	0.119	0.009	4.4E-37	trans
1712	PRG3	Q9Y2Y8	Proteoglycan 3	rs7840212	130599247	8	C	T	0.662	0.090	0.008	1.7E-31	trans
1712	PRG3	Q9Y2Y8	Proteoglycan 3	rs6558372	144630070	8	G	C	0.521	0.087	0.007	2.0E-32	trans
1712	PRG3	Q9Y2Y8	Proteoglycan 3	rs1888909	6197392	9	T	C	0.251	0.076	0.008	1.1E-19	trans
1712	PRG3	Q9Y2Y8	Proteoglycan 3	rs150813342	135864513	9	C	T	0.992	0.279	0.041	9.9E-12	trans
1712	PRG3	Q9Y2Y8	Proteoglycan 3	rs34108746	57146225	11	G	A	0.079	0.226	0.014	1.9E-62	cis
1712	PRG3	Q9Y2Y8	Proteoglycan 3	rs9566645	41563026	13	T	C	0.812	0.121	0.009	2.4E-38	trans
1712	PRG3	Q9Y2Y8	Proteoglycan 3	rs725613	11169683	16	T	G	0.642	0.059	0.008	7.5E-15	trans
1712	PRG3	Q9Y2Y8	Proteoglycan 3	rs371029929	4562665	17	A	C	0.754	0.100	0.009	8.2E-32	trans
1712	PRG3	Q9Y2Y8	Proteoglycan 3	rs35617692	56272501	17	A	C	0.011	0.520	0.035	9.4E-51	trans
1712	PRG3	Q9Y2Y8	Proteoglycan 3	rs146776010	40225031	19	G	C	0.994	0.453	0.048	2.3E-21	trans
1712	PRG3	Q9Y2Y8	Proteoglycan 3	rs56117721	36398586	21	T	A	0.928	0.100	0.014	2.2E-12	trans
1713	PRKAB1	Q9Y478	5'-AMP-activated protein kinase subunit beta-1	rs1354034	56849749	3	C	T	0.604	0.055	0.008	5.4E-13	trans
1714	PRKAR1A	P10644	cAMP-dependent protein kinase type I-alpha regulatory subunit	rs68066031	224880498	2	C	T	0.230	0.221	0.009	2.8E-132	trans
1714	PRKAR1A	P10644	cAMP-dependent protein kinase type I-alpha regulatory subunit	rs1354034	56849749	3	C	T	0.604	0.067	0.008	1.4E-18	trans
1714	PRKAR1A	P10644	cAMP-dependent protein kinase type I-alpha regulatory subunit	rs342293	106372219	7	C	G	0.540	0.056	0.008	1.1E-13	trans
1714	PRKAR1A	P10644	cAMP-dependent protein kinase type I-alpha regulatory subunit	rs7896518	65104500	10	G	A	0.424	0.064	0.008	1.3E-16	trans
1714	PRKAR1A	P10644	cAMP-dependent protein kinase type I-alpha regulatory subunit	rs1552950	101994233	15	G	C	0.293	0.073	0.008	9.3E-19	trans
1715	PRKAR2A	P13861	cAMP-dependent protein kinase type II-alpha regulatory subunit	rs151304617	49348189	3	A	G	0.999	1.667	0.115	1.3E-47	cis
1715	PRKAR2A	P13861	cAMP-dependent protein kinase type II-alpha regulatory subunit	rs1354034	56849749	3	C	T	0.604	0.053	0.008	2.0E-12	trans
1715	PRKAR2A	P13861	cAMP-dependent protein kinase type II-alpha regulatory subunit	rs7896518	65104500	10	G	A	0.424	0.055	0.008	3.2E-13	trans
1716	PRKG1	Q13976	cGMP-dependent protein kinase 1	rs1354034	56849749	3	C	T	0.604	0.060	0.008	5.3E-15	trans
1716	PRKG1	Q13976	cGMP-dependent protein kinase 1	rs6993770	106581528	8	A	T	0.713	0.070	0.008	3.0E-17	trans
1716	PRKG1	Q13976	cGMP-dependent protein kinase 1	rs1937682	53678982	10	A	T	0.762	0.218	0.009	2.6E-133	cis
1716	PRKG1	Q13976	cGMP-dependent protein kinase 1	rs1863243	10677373	11	A	G	0.668	0.060	0.008	3.8E-14	trans
1717	PRKRA	O75569	Interferon-inducible double-stranded RNA-dependent protein kinase activator A	rs1354034	56849749	3	C	T	0.604	0.102	0.008	4.8E-40	trans
1717	PRKRA	O75569	Interferon-inducible double-stranded RNA-dependent protein kinase activator A	rs11502185	180258	11	C	T	0.260	0.077	0.010	1.7E-15	trans
1718	PRL	P01236	Prolactin	rs3055719	1457093	5	A	ATTTC	0.273	0.056	0.008	1.1E-11	trans
1718	PRL	P01236	Prolactin	rs7861599	5067298	9	C	T	0.507	0.078	0.007	4.5E-26	trans
1718	PRL	P01236	Prolactin	rs11042976	2198259	11	G	A	0.518	0.051	0.007	3.1E-12	trans
1719	PRND	Q9UKY0	Prion-like protein doppel	rs9657541	10643164	8	C	T	0.794	0.121	0.009	4.9E-41	trans
1719	PRND	Q9UKY0	Prion-like protein doppel	rs507666	136149399	9	G	A	0.815	0.268	0.010	1.7E-174	trans
1719	PRND	Q9UKY0	Prion-like protein doppel	rs1015605	130834783	10	A	G	0.364	0.068	0.008	6.8E-19	trans
1719	PRND	Q9UKY0	Prion-like protein doppel	rs4760	44153100	19	G	A	0.155	0.074	0.010	1.5E-13	trans
1719	PRND	Q9UKY0	Prion-like protein doppel	rs741233	46894786	19	C	T	0.688	0.077	0.008	5.0E-23	trans
1719	PRND	Q9UKY0	Prion-like protein doppel	rs36029876	4723988	20	C	A	0.896	0.381	0.012	9.7E-214	cis
1720	PROC	P04070	Vitamin K-dependent protein C	rs599839	109822166	1	A	G	0.772	0.086	0.008	1.2E-25	trans
1720	PROC	P04070	Vitamin K-dependent protein C	rs1260326	27730940	2	T	C	0.392	0.136	0.007	1.9E-80	trans
1720	PROC	P04070	Vitamin K-dependent protein C	rs1799810	128176040	2	A	T	0.573	0.199	0.007	3.1E-172	cis

1720	PROC	P04070	Vitamin K-dependent protein C	rs13108218	3443931	4	A	G	0.381	0.053	0.007	1.9E-13	trans
1720	PROC	P04070	Vitamin K-dependent protein C	rs9275239	32660717	6	A	G	0.479	0.054	0.007	1.2E-14	trans
1720	PROC	P04070	Vitamin K-dependent protein C	rs13226650	73017005	7	A	G	0.803	0.076	0.009	1.5E-18	trans
1720	PROC	P04070	Vitamin K-dependent protein C	rs2393969	65140440	10	A	C	0.531	0.050	0.007	5.1E-13	trans
1720	PROC	P04070	Vitamin K-dependent protein C	rs149394327	64228995	17	C	G	0.030	0.142	0.020	2.2E-12	trans
1721	PROCR	Q9UNN8	Endothelial protein C receptor	rs867186	33764554	20	G	A	0.086	0.730	0.014	0.0E+00	cis
1722	PROK1	P58294	Prokineticin-1	rs1857513	110993457	1	A	G	0.159	0.479	0.008	0.0E+00	cis
1722	PROK1	P58294	Prokineticin-1	rs7451690	7109433	6	C	T	0.482	0.035	0.005	9.5E-12	trans
1722	PROK1	P58294	Prokineticin-1	rs2856694	32652281	6	T	C	0.814	0.060	0.007	1.7E-17	trans
1722	PROK1	P58294	Prokineticin-1	rs7497289	40333657	15	A	G	0.283	0.040	0.006	1.1E-12	trans
1723	PROS1	P07225	Vitamin K-dependent protein S	rs1260326	27730940	2	T	C	0.392	0.067	0.008	4.1E-19	trans
1723	PROS1	P07225	Vitamin K-dependent protein S	rs10982156	117088064	9	T	A	0.931	0.421	0.015	7.0E-170	trans
1723	PROS1	P07225	Vitamin K-dependent protein S	rs924135	16123459	16	T	A	0.610	0.055	0.008	4.1E-13	trans
1724	PRR4	Q16378	Proline-rich protein 4	rs12032424	202191871	1	A	G	0.413	0.067	0.007	1.4E-24	trans
1724	PRR4	Q16378	Proline-rich protein 4	rs10182233	16207569	2	T	C	0.603	0.053	0.007	1.1E-15	trans
1724	PRR4	Q16378	Proline-rich protein 4	rs17513198	232259376	2	A	G	0.093	0.119	0.011	3.2E-27	trans
1724	PRR4	Q16378	Proline-rich protein 4	rs56030777	33163059	9	G	A	0.772	0.054	0.008	1.5E-12	trans
1724	PRR4	Q16378	Proline-rich protein 4	rs2981737	124317260	10	C	G	0.307	0.052	0.007	1.6E-13	trans
1724	PRR4	Q16378	Proline-rich protein 4	rs34239025	17911052	19	C	T	0.814	0.152	0.008	2.8E-75	trans
1725	PRRT3	Q5FWE3	Proline-rich transmembrane protein 3	rs61747728	179526214	1	T	C	0.038	0.170	0.019	1.7E-18	trans
1725	PRRT3	Q5FWE3	Proline-rich transmembrane protein 3	rs75166367	162964301	2	G	A	0.940	0.141	0.016	2.3E-19	trans
1725	PRRT3	Q5FWE3	Proline-rich transmembrane protein 3	rs55847233	9991101	3	C	G	0.751	0.213	0.009	1.1E-132	cis
1726	PRSS2	P07478	Trypsin-2	rs1003582	29538403	6	G	C	0.506	0.053	0.007	8.1E-13	trans
1726	PRSS2	P07478	Trypsin-2	rs9482772	127446790	6	C	T	0.451	0.078	0.007	1.2E-25	trans
1726	PRSS2	P07478	Trypsin-2	rs3752404	142470574	7	A	G	0.584	0.139	0.008	6.2E-76	cis
1726	PRSS2	P07478	Trypsin-2	rs748208	18712513	8	T	C	0.288	0.070	0.008	3.1E-17	trans
1726	PRSS2	P07478	Trypsin-2	rs4733612	129569999	8	A	G	0.733	0.068	0.008	8.3E-16	trans
1726	PRSS2	P07478	Trypsin-2	rs174578	61605499	11	A	T	0.353	0.064	0.008	1.4E-16	trans
1726	PRSS2	P07478	Trypsin-2	rs686056	100646689	11	G	A	0.677	0.078	0.008	5.7E-23	trans
1726	PRSS2	P07478	Trypsin-2	rs72802342	75234872	16	C	A	0.925	0.264	0.014	1.4E-75	trans
1726	PRSS2	P07478	Trypsin-2	rs533406	88974860	16	A	G	0.619	0.107	0.008	3.7E-44	trans
1726	PRSS2	P07478	Trypsin-2	rs17138478	36073320	17	A	C	0.131	0.076	0.011	5.5E-12	trans
1726	PRSS2	P07478	Trypsin-2	rs7234846	42403140	18	A	T	0.077	0.109	0.014	5.1E-15	trans
1727	PRSS22	Q9GZN4	Brain-specific serine protease 4	rs6917212	32583005	6	C	G	0.573	0.054	0.008	1.3E-12	trans
1727	PRSS22	Q9GZN4	Brain-specific serine protease 4	rs56278466	17875857	10	G	T	0.661	0.062	0.008	7.6E-15	trans
1727	PRSS22	Q9GZN4	Brain-specific serine protease 4	rs148815576	93685488	14	G	T	0.002	0.639	0.094	1.1E-11	trans
1727	PRSS22	Q9GZN4	Brain-specific serine protease 4	rs11634947	49997216	15	C	G	0.631	0.054	0.008	3.4E-12	trans
1727	PRSS22	Q9GZN4	Brain-specific serine protease 4	rs8046218	2908703	16	T	C	0.325	0.260	0.008	1.3E-220	cis
1728	PRSS27	Q9BQR3	Serine protease 27	rs2360962	199004709	1	A	T	0.262	0.056	0.008	3.9E-13	trans
1728	PRSS27	Q9BQR3	Serine protease 27	rs1354034	56849749	3	T	C	0.396	0.049	0.007	1.1E-12	trans
1728	PRSS27	Q9BQR3	Serine protease 27	rs35225290	141130835	3	C	T	0.656	0.091	0.007	4.6E-37	trans
1728	PRSS27	Q9BQR3	Serine protease 27	rs541115	187638092	3	A	T	0.338	0.052	0.007	2.6E-13	trans
1728	PRSS27	Q9BQR3	Serine protease 27	rs968042	106016717	4	A	G	0.682	0.054	0.007	1.2E-13	trans
1728	PRSS27	Q9BQR3	Serine protease 27	rs9501828	2339918	6	T	C	0.533	0.157	0.007	4.6E-116	trans
1728	PRSS27	Q9BQR3	Serine protease 27	rs10758189	33125804	9	T	C	0.689	0.054	0.007	1.6E-13	trans
1728	PRSS27	Q9BQR3	Serine protease 27	rs141129381	100691900	9	TG	T	0.363	0.079	0.007	7.1E-29	trans
1728	PRSS27	Q9BQR3	Serine protease 27	rs635634	136155000	9	C	T	0.816	0.060	0.009	1.2E-11	trans
1728	PRSS27	Q9BQR3	Serine protease 27	rs112972631	104358178	10	G	T	0.747	0.058	0.008	9.3E-14	trans
1728	PRSS27	Q9BQR3	Serine protease 27	rs546202	65510024	11	C	T	0.550	0.065	0.007	9.1E-21	trans
1728	PRSS27	Q9BQR3	Serine protease 27	rs11220477	126275402	11	C	T	0.958	0.527	0.017	1.4E-206	trans
1728	PRSS27	Q9BQR3	Serine protease 27	rs71386687	2767894	16	T	G	0.082	0.387	0.013	5.2E-193	cis
1728	PRSS27	Q9BQR3	Serine protease 27	rs708686	5840619	19	T	C	0.268	0.160	0.008	1.8E-82	trans
1728	PRSS27	Q9BQR3	Serine protease 27	rs681343	49206462	19	T	C	0.509	0.553	0.007	0.0E+00	trans
1728	PRSS27	Q9BQR3	Serine protease 27	rs61658003	55544203	19	A	C	0.843	0.077	0.010	3.8E-14	trans
1728	PRSS27	Q9BQR3	Serine protease 27	rs587935	40910740	21	T	C	0.666	0.053	0.007	1.9E-13	trans
1729	PRSS53	Q2L4Q9	Serine protease 53	rs903323	154572228	1	C	T	0.520	0.060	0.006	2.5E-26	trans
1729	PRSS53	Q2L4Q9	Serine protease 53	rs35487539	53932006	2	T	C	0.205	0.051	0.007	3.3E-13	trans
1729	PRSS53	Q2L4Q9	Serine protease 53	rs142160737	39569149	4	A	G	0.076	0.082	0.011	3.4E-14	trans
1729	PRSS53	Q2L4Q9	Serine protease 53	rs28929474	94844947	14	T	C	0.021	0.235	0.020	1.7E-32	trans
1729	PRSS53	Q2L4Q9	Serine protease 53	rs4468641	31096876	16	C	A	0.385	0.885	0.008	0.0E+00	cis
1729	PRSS53	Q2L4Q9	Serine protease 53	rs1801689	64210580	17	C	A	0.030	0.480	0.017	2.4E-183	trans
1730	PRSS8	Q16651	Prostasin	rs1260326	27730940	2	T	C	0.391	0.065	0.007	5.2E-20	trans
1730	PRSS8	Q16651	Prostasin	rs13229619	73030175	7	G	A	0.872	0.106	0.010	1.9E-24	trans
1730	PRSS8	Q16651	Prostasin	rs62032958	415317	16	G	A	0.932	0.197	0.014	1.3E-45	trans
1730	PRSS8	Q16651	Prostasin	rs889555	31122571	16	T	C	0.282	0.115	0.008	5.7E-49	cis
1730	PRSS8	Q16651	Prostasin	rs9912592	45556945	17	G	A	0.442	0.060	0.007	2.2E-17	trans
1730	PRSS8	Q16651	Prostasin	rs57822461	38732583	19	C	T	0.818	0.094	0.009	4.5E-25	trans
1731	PRTFDC1	Q9NRG1	Phosphoribosyltransferase domain-containing protein 1	rs143356584	25241502	10	C	T	0.986	0.858	0.033	1.1E-150	cis
1732	PRTG	Q2VWP7	Protogenin	rs13107325	103188709	4	T	C	0.075	0.096	0.013	4.7E-13	trans
1732	PRTG	Q2VWP7	Protogenin	rs78506734	32529695	6	T	C	0.175	0.072	0.009	1.6E-14	trans
1732	PRTG	Q2VWP7	Protogenin	rs8176746	136131322	9	T	G	0.061	0.299	0.015	2.7E-94	trans
1732	PRTG	Q2VWP7	Protogenin	rs10851591	56003705	15	G	A	0.693	0.449	0.008	0.0E+00	cis
1732	PRTG	Q2VWP7	Protogenin	rs7498431	88081904	16	G	C	0.612	0.092	0.007	6.0E-38	trans
1732	PRTG	Q2VWP7	Protogenin	rs186021206	7069412	17	A	G	0.006	0.559	0.048	1.8E-31	trans
1732	PRTG	Q2VWP7	Protogenin	rs704	26694861	17	G	A	0.527	0.057	0.007	2.4E-16	trans
1732	PRTG	Q2VWP7	Protogenin	rs77542162	67081278	17	G	A	0.023	0.190	0.023	3.1E-16	trans
1732	PRTG	Q2VWP7	Protogenin	rs6565921	74557753	18	A	G	0.366	0.064	0.007	4.2E-19	trans
1733	PRTN3	P24158	Myeloblastin	rs3917932	36943916	1	C	G	0.424	0.070	0.007	2.2E-22	trans
1733	PRTN3	P24158	Myeloblastin	rs6782228	128323424	3	G	C	0.731	0.054	0.008	1.5E-11	trans
1733	PRTN3	P24158	Myeloblastin	rs41559422	31323667	6	A	G	0.037	0.248	0.019	2.0E-40	trans
1733	PRTN3	P24158	Myeloblastin	rs915125	82463376	6	T	C	0.278	0.088	0.008	2.1E-28	trans
1733	PRTN3	P24158	Myeloblastin	rs11155297	143825104	6	G	T	0.736	0.055	0.008	6.5E-12	trans
1733	PRTN3	P24158	Myeloblastin	rs2977810	6815457	8	C	T	0.334	0.068	0.008	7.9E-19	trans
1733	PRTN3	P24158	Myeloblastin	rs13277237	130604563	8	G	A	0.433	0.089	0.007	2.3E-35	trans
1733	PRTN3	P24158	Myeloblastin	rs118119317	82226685	9	G	A	0.968	0.138	0.020	4.3E-12	trans
1733	PRTN3	P24158	Myeloblastin	rs73000965	113982321	11	A	T	0.316	0.060	0.008	3.5E-15	trans
1733	PRTN3	P24158	Myeloblastin	rs16942887	67928042	16	A	G	0.117	0.075	0.011	1.4E-11	trans
1733	PRTN3	P24158	Myeloblastin	rs8078723	38166879	17	C	T	0.393	0.091	0.007	1.1E-36	trans
1733	PRTN3	P24158	Myeloblastin	rs56378716	56356502	17	G	A	0.013	0.242	0.031	4.8E-15	trans
1733	PRTN3	P24158	Myeloblastin	rs10425544	836043	19	C	T	0.713	0.499	0.009	0.0E+00	cis
1734	PRUNE2	Q8WUY3	Protein prune homolog 2	rs11819066	135174202	10	C	T	0.032	0.248	0.022	3.1E-30	trans
1735	PSAP	P07602	Prosaposin	rs1736085	11703659	8	C	T	0.260	0.094	0.008	2.8E-30	trans
1735	PSAP	P07602	Prosaposin	rs6993770	106581528	8	A	T	0.714	0.093	0.008	3.0E-31	trans
1735	PSAP	P07602	Prosaposin	rs57681041	73643962	10	C	T	0.779	0.249	0.009	6.8E-176	cis
1735	PSAP	P07602	Prosaposin	rs145078947	93652974	14	T	G	0.003	1.032	0.072	6.9E-47	trans
1735	PSAP	P07602	Prosaposin	rs2289702	79237293	15	T	C	0.107	0.133	0.012	1.3E-29	trans
1735	PSAP	P07602	Prosaposin	rs58542926	19379549	19	T	C	0.076	0.097	0.014	1.4E-12	trans
1736	PSAPL1	Q6NUJ1	Proactivator polypeptide-like 1	rs7537101	16150384	1	C	T	0.465	0.047	0.007	9.6E-13	trans
1736	PSAPL1	Q6NUJ1	Proactivator polypeptide-like 1	rs1062964	59042388	1	C	G	0.945	0.125	0.014	5.7E-18	trans
1736	PSAPL1	Q6NUJ1	Proactivator polypeptide-like 1	rs61816761	152285861	1	A	G	0.023	0.529	0.023	1.1E-115	trans
1736	PSAPL1	Q6NUJ1	Proactivator polypeptide-like 1	rs6757005	10910851	2	C	T	0.486	0.050	0.007	2.1E-14	trans
1736	PSAPL1	Q6NUJ1	Proactivator polypeptide-like 1	rs1436662	190021928	3	G	A	0.593	0.093	0.007	3.5E-44	trans
1736	PSAPL1	Q6NUJ1	Proactivator polypeptide-like 1	rs10014338	7434607	4	A	C	0.184	0.630	0.009	0.0E+00	cis
1736	PSAPL1	Q6NUJ1	Proactivator polypeptide-like 1	rs9368728	11471043	6	A	G	0.289	0.050	0.007	6.0E-12	trans
1736	PSAPL1	Q6NUJ1	Proactivator polypeptide-like 1	rs3132568	31101917	6	A	G	0.744	0.063	0.008	5.9E-17	trans
1736	PSAPL1	Q6NUJ1	Proactivator polypeptide-like 1	rs376534130	73190227	7	T	C	0.414	0.058	0.007	1.5E-17	trans
1736	PSAPL1	Q6NUJ1	Proactivator polypeptide-like 1	rs11249885	8562008	8	T	C	0.242	0.092	0.008	3.0E-33	trans
1736	PSAPL1	Q6NUJ1	Proactivator polypeptide-like 1	rs3779660	11692569	8	T	G	0.339	0.049	0.007	1.3E-12	trans
1736	PSAPL1	Q6NUJ1	Proactivator polypeptide-like 1	rs3758294	107664815	9	T	C	0.796	0.088	0.008	8.9E-28	trans
1736	PSAPL1	Q6NUJ1	Proactivator polypeptide-like 1	rs17658212	41145919	15	C	T	0.940	0.281	0.014	3.3E-87	trans
1736	PSAPL1	Q6NUJ1	Proactivator polypeptide-like 1	rs889591	58283141	16	G	C	0.739	0.051	0.007	7.7E-12	trans
1736	PSAPL1	Q6NUJ1	Proactivator polypeptide-like 1	rs181040167	68840204	16	C	T	0.994	0.336	0.047	8.2E-13	trans
1736	PSAPL1	Q6NUJ1	Proactivator polypeptide-like 1	rs10686390	38995397	17	A	ATC	0.391	0.058	0.007	9.5E-18	trans
1736	PSAPL1	Q6NUJ1	Proactivator polypeptide-like 1	rs4078130	45664615	18	T	C	0.552	0.086	0.007	3.8E-39	trans
1736	PSAPL1	Q6NUJ1	Proactivator polypeptide-like 1	rs6508781	38785318	19	A	C	0.832	0.195	0.009	5.5E-109	trans
1736	PSAPL1	Q6NUJ1	Proactivator polypeptide-like 1	rs181163991	51508860	19	T	C	0.983	0.229	0.028	1.7E-16	trans
1736	PSAPL1	Q6NUJ1	Proactivator polypeptide-like 1	rs10521460	49759745	X	T	A	0.077	0.143	0.010	1.2E-44	trans
1737	PSCA	O43653	Prostate stem cell antigen	rs2976388	143760256	8	A	G	0.422	1.161	0.008	0.0E+00	cis
1737	PSCA	O43653	Prostate stem cell antigen	rs2519093	136141870	9	C	T	0.816	0.146	0.006	1.2E-146	trans
1737	PSCA	O43653	Prostate stem cell antigen	rs186021206	7069412	17	A	G	0.006	0.228	0.030	2.0E-14	trans
1737	PSCA	O43653	Prostate stem cell antigen	rs2608894	5848000	19	T	C	0.235	0.036	0.005	1.2E-11	trans
1737	PSCA	O43653	Prostate stem cell antigen	rs4760	44153100	19	G	A	0.155	0.116	0.006	6.9E-82	trans
1737	PSCA	O43653	Prostate stem cell antigen	rs516246	49206172	19	C	T	0.491	0.120	0.004	4.5E-164	trans
1738	PSG1	P11464	Pregnancy-specific beta-1-glycoprotein 1	rs7526027	59683994	1	C	T	0.292	0.048	0.007	4.7E-13	trans
1738	PSG1	P11464	Pregnancy-specific beta-1-glycoprotein 1	rs78689694	126234820	11	G	C	0.867	0.097	0.009	6.8E-28	trans
1738	PSG1	P11464	Pregnancy-specific beta-1-glycoprotein 1	rs909222	75909727	14	C	A	0.456	0.045	0.006	4.8E-14	trans
1738	PSG1	P11464	Pregnancy-specific beta-1-glycoprotein 1	rs186021206	7069412	17	A	G	0.006	0.318	0.041	8.4E-15	trans
1738	PSG1	P11464	Pregnancy-specific beta-1-glycoprotein 1	rs12986075	43373034	19	G	A	0.801	0.962	0.009	0.0E+00	cis
1738	PSG1	P11464	Pregnancy-specific beta-1-glycoprotein 1	rs61740142	2464112	20	T	C	0.962	0.290	0.016	3.6E-77	trans
1738	PSG1	P11464	Pregnancy-specific beta-1-glycoprotein 1	rs6068155	50991061	20	T	C	0.632	0.044	0.006	8.4E-13	trans
1739	PSIP1	O75475	PC4 and SFRS1-interacting protein	rs1354034	56849749	3	C	T	0.604	0.126	0.008	2.1E-62	trans
1739	PSIP1	O75475	PC4 and SFRS1-interacting protein	rs7080536	115348046	10	G	A	0.956	0.160	0.018	3.1E-18	trans
1739	PSIP1	O75475	PC4 and SFRS1-interacting protein	rs11604127	196944	11	T	C	0.231	0.060	0.009	9.4E-12	trans
1740	PSME1	Q06323	Proteasome activator complex subunit 1	rs1354034	56849749	3	C	T	0.604	0.054	0.007	4.9E-13	trans
1740	PSME1	Q06323	Proteasome activator complex subunit 1	rs2236352	24610733	14	C	T	0.256	0.094	0.008	1.3E-28	cis
1741	PSME2	Q9UL46	Proteasome activator complex subunit 2	rs2236352	24610733	14	C	T	0.256	0.104	0.008	4.6E-35	cis
1742	PSMG3	Q9BT73	Proteasome assembly chaperone 3	rs9503502	3260555	6	T	A	0.168	0.196	0.010	3.3E-91	trans
1742	PSMG3	Q9BT73	Proteasome assembly chaperone 3	rs34407549	1608812	7	C	G	0.992	0.290	0.042	8.4E-12	cis
1743	PSPN	O60542	Persephin	rs4381790	18671795	2	C	A	0.455	0.046	0.006	1.3E-16	trans
1743	PSPN	O60542	Persephin	rs6542472	119329438	2	C	A	0.357	0.041	0.006	1.2E-12	trans
1743	PSPN	O60542	Persephin	rs2102843	233168620	2	T	C	0.940	0.095	0.012	6.1E-16	trans
1743	PSPN	O60542	Persephin	rs13111751	120050762	4	C	G	0.663	0.046	0.006	8.0E-15	trans
1743	PSPN	O60542	Persephin	rs9543374	74044117	13	C	T	0.736	0.071	0.006	2.6E-29	trans

1743	PSPN	O60542	Persephin	rs138903858	105634487	14	G	T	0.972	0.145	0.017	7.8E-17	trans
1743	PSPN	O60542	Persephin	rs58634535	3641542	20	G	A	0.844	0.056	0.008	4.5E-13	trans
1743	PSPN	O60542	Persephin	rs6517463	39752673	21	T	C	0.245	0.050	0.006	8.4E-15	trans
1743	PSPN	O60542	Persephin	rs5930556	131336429	X	G	A	0.307	0.042	0.005	6.1E-17	trans
1744	PSRC1	Q6PGN9	Proline/serine-rich coiled-coil protein 1	rs34863121	109823458	1	T	C	0.013	0.725	0.034	5.9E-103	cis
1744	PSRC1	Q6PGN9	Proline/serine-rich coiled-coil protein 1	rs10733789	64948684	10	C	T	0.313	0.056	0.008	1.5E-11	trans
1744	PSRC1	Q6PGN9	Proline/serine-rich coiled-coil protein 1	rs6070696	57597645	20	G	A	0.185	0.094	0.010	1.2E-21	trans
1745	PSTPIP2	Q9H939	Proline-serine-threonine phosphatase-interacting protein 2	rs10822145	64934548	10	T	C	0.472	0.059	0.008	2.4E-14	trans
1745	PSTPIP2	Q9H939	Proline-serine-threonine phosphatase-interacting protein 2	rs72642432	43571278	18	G	A	0.868	0.226	0.011	1.9E-87	cis
1746	PTGDS	P41222	Prostaglandin-H2 D-isomerase	rs12934455	20357281	16	C	T	0.834	0.067	0.010	2.5E-12	trans
1746	PTGDS	P41222	Prostaglandin-H2 D-isomerase	rs704	26694861	17	A	G	0.473	0.048	0.007	1.6E-11	trans
1747	PTGES2	Q9H7Z7	Prostaglandin E synthase 2	rs13283456	130884753	9	C	T	0.790	0.097	0.009	3.1E-25	cis
1748	PTGR1	Q14914	Prostaglandin reductase 1	rs2169387	9181395	8	A	G	0.102	0.085	0.012	1.4E-13	trans
1748	PTGR1	Q14914	Prostaglandin reductase 1	rs112875651	126506694	8	G	A	0.609	0.055	0.007	2.1E-14	trans
1748	PTGR1	Q14914	Prostaglandin reductase 1	rs7036102	114326547	9	G	A	0.050	0.758	0.017	0.0E+00	cis
1748	PTGR1	Q14914	Prostaglandin reductase 1	rs1788783	21161134	18	C	T	0.498	0.052	0.007	6.2E-14	trans
1748	PTGR1	Q14914	Prostaglandin reductase 1	rs12975366	54759361	19	T	C	0.604	0.049	0.007	6.8E-12	trans
1748	PTGR1	Q14914	Prostaglandin reductase 1	rs738409	44324727	22	G	C	0.217	0.061	0.008	3.5E-13	trans
1749	PTH	P01270	Parathyroid hormone	rs10051765	176799992	5	T	C	0.669	0.074	0.008	1.4E-20	trans
1749	PTH	P01270	Parathyroid hormone	rs7800944	73035857	7	T	C	0.714	0.070	0.008	3.2E-17	trans
1749	PTH	P01270	Parathyroid hormone	rs286	19815256	8	A	T	0.914	0.096	0.013	7.7E-13	trans
1749	PTH	P01270	Parathyroid hormone	rs35870583	52735238	20	GT	G	0.187	0.122	0.010	6.7E-35	trans
1750	PTK7	Q13308	Inactive tyrosine-protein kinase 7	rs116450546	43057265	6	T	A	0.036	0.156	0.021	6.9E-14	cis
1751	PTN	P21246	Pleiotrophin	rs4252165	161161559	6	A	C	0.294	0.071	0.008	2.5E-19	trans
1751	PTN	P21246	Pleiotrophin	rs1431092	137016309	7	A	T	0.425	0.151	0.007	1.2E-93	cis
1751	PTN	P21246	Pleiotrophin	rs4541868	106590705	8	C	A	0.726	0.058	0.008	1.1E-12	trans
1751	PTN	P21246	Pleiotrophin	rs1654425	55538980	19	C	T	0.834	0.071	0.010	1.9E-13	trans
1752	PTP4A3	O75365	Protein tyrosine phosphatase type IVA 3	rs2242197	99118622	8	A	C	0.680	0.236	0.008	1.4E-183	trans
1753	PTPN1	P18031	Tyrosine-protein phosphatase non-receptor type 1	rs1354034	56849749	3	C	T	0.604	0.084	0.008	1.4E-28	trans
1753	PTPN1	P18031	Tyrosine-protein phosphatase non-receptor type 1	rs342293	106372219	7	C	G	0.541	0.052	0.007	5.4E-12	trans
1753	PTPN1	P18031	Tyrosine-protein phosphatase non-receptor type 1	rs7080536	115348046	10	G	A	0.956	0.194	0.019	2.6E-25	trans
1754	PTPN6	P29350	Tyrosine-protein phosphatase non-receptor type 6	rs1354034	56849749	3	C	T	0.604	0.061	0.008	3.2E-15	trans
1754	PTPN6	P29350	Tyrosine-protein phosphatase non-receptor type 6	rs342298	106373646	7	C	T	0.546	0.073	0.008	5.1E-22	trans
1755	PTPRB	P23467	Receptor-type tyrosine-protein phosphatase beta	rs4543739	25049680	1	A	C	0.751	0.054	0.008	6.7E-12	trans
1755	PTPRB	P23467	Receptor-type tyrosine-protein phosphatase beta	rs6825454	155501188	4	C	T	0.257	0.085	0.008	2.5E-27	trans
1755	PTPRB	P23467	Receptor-type tyrosine-protein phosphatase beta	rs189209601	32524561	6	A	G	0.057	0.116	0.016	8.6E-13	trans
1755	PTPRB	P23467	Receptor-type tyrosine-protein phosphatase beta	rs8176747	136131315	9	G	C	0.061	0.541	0.014	1.2E-306	trans
1755	PTPRB	P23467	Receptor-type tyrosine-protein phosphatase beta	rs34434834	126307796	11	A	G	0.033	0.256	0.019	1.2E-40	trans
1755	PTPRB	P23467	Receptor-type tyrosine-protein phosphatase beta	rs6602909	114551993	13	C	T	0.324	0.084	0.007	5.7E-30	trans
1755	PTPRB	P23467	Receptor-type tyrosine-protein phosphatase beta	rs653152	7840343	19	G	A	0.322	0.081	0.007	3.0E-28	trans
1756	PTPRC	P08575	Receptor-type tyrosine-protein phosphatase C	rs139888769	93435677	1	GT	G	0.042	0.171	0.019	1.1E-19	trans
1756	PTPRC	P08575	Receptor-type tyrosine-protein phosphatase C	rs552839429	198695989	1	T	C	0.983	0.806	0.029	8.6E-173	cis
1756	PTPRC	P08575	Receptor-type tyrosine-protein phosphatase C	rs75071241	126232186	11	G	A	0.940	0.154	0.016	4.0E-23	trans
1756	PTPRC	P08575	Receptor-type tyrosine-protein phosphatase C	rs186021206	7069412	17	A	G	0.006	1.325	0.051	5.7E-150	trans
1757	PTPRF	P10586	Receptor-type tyrosine-protein phosphatase F	rs2842194	44028962	1	G	A	0.775	0.089	0.009	2.9E-25	cis
1757	PTPRF	P10586	Receptor-type tyrosine-protein phosphatase F	rs6531216	20374286	2	G	A	0.470	0.062	0.007	6.5E-18	trans
1757	PTPRF	P10586	Receptor-type tyrosine-protein phosphatase F	rs9873618	170733076	3	G	A	0.711	0.064	0.008	4.1E-16	trans
1757	PTPRF	P10586	Receptor-type tyrosine-protein phosphatase F	rs17039766	110006432	4	A	C	0.056	0.292	0.016	1.4E-77	trans
1757	PTPRF	P10586	Receptor-type tyrosine-protein phosphatase F	rs2308527	31239821	6	G	T	0.414	0.059	0.007	5.0E-16	trans
1757	PTPRF	P10586	Receptor-type tyrosine-protein phosphatase F	rs581080	15305378	9	C	G	0.821	0.064	0.009	9.9E-12	trans
1757	PTPRF	P10586	Receptor-type tyrosine-protein phosphatase F	rs507666	136149399	9	G	A	0.815	0.083	0.009	3.9E-19	trans
1757	PTPRF	P10586	Receptor-type tyrosine-protein phosphatase F	rs10761741	65066186	10	T	G	0.413	0.069	0.007	4.7E-21	trans
1757	PTPRF	P10586	Receptor-type tyrosine-protein phosphatase F	rs9633740	82265271	10	G	A	0.795	0.138	0.009	1.2E-54	trans
1757	PTPRF	P10586	Receptor-type tyrosine-protein phosphatase F	rs1535	61597972	11	G	A	0.347	0.055	0.007	2.3E-13	trans
1757	PTPRF	P10586	Receptor-type tyrosine-protein phosphatase F	rs28929474	94844947	14	T	C	0.021	0.334	0.025	3.0E-40	trans
1757	PTPRF	P10586	Receptor-type tyrosine-protein phosphatase F	rs1801689	64210580	17	A	C	0.970	0.217	0.021	3.7E-25	trans
1757	PTPRF	P10586	Receptor-type tyrosine-protein phosphatase F	rs516246	49206172	19	C	T	0.491	0.101	0.007	1.3E-44	trans
1758	PTPRH	Q9HD43	Receptor-type tyrosine-protein phosphatase H	rs35939242	12301893	3	C	A	0.341	0.048	0.007	1.5E-12	trans
1758	PTPRH	Q9HD43	Receptor-type tyrosine-protein phosphatase H	rs13067380	24229058	3	A	G	0.805	0.096	0.008	2.2E-31	trans
1758	PTPRH	Q9HD43	Receptor-type tyrosine-protein phosphatase H	rs3731211	21986847	9	T	A	0.278	0.057	0.007	4.4E-15	trans
1758	PTPRH	Q9HD43	Receptor-type tyrosine-protein phosphatase H	rs2519093	136141870	9	C	T	0.816	0.207	0.008	8.9E-132	trans
1758	PTPRH	Q9HD43	Receptor-type tyrosine-protein phosphatase H	rs59889308	50280792	10	T	C	0.237	0.061	0.008	1.5E-15	trans
1758	PTPRH	Q9HD43	Receptor-type tyrosine-protein phosphatase H	rs3862630	126243649	11	C	T	0.878	0.145	0.010	8.3E-45	trans
1758	PTPRH	Q9HD43	Receptor-type tyrosine-protein phosphatase H	rs753728	93523060	14	G	A	0.885	0.089	0.010	2.7E-18	trans
1758	PTPRH	Q9HD43	Receptor-type tyrosine-protein phosphatase H	rs186021206	7069412	17	A	G	0.006	0.442	0.045	3.0E-23	trans
1758	PTPRH	Q9HD43	Receptor-type tyrosine-protein phosphatase H	rs2288523	55702910	19	A	C	0.131	0.907	0.011	0.0E+00	cis
1759	PTPRK	Q15262	Receptor-type tyrosine-protein phosphatase kappa	rs9321109	128377497	6	G	A	0.171	0.136	0.010	1.4E-41	cis
1759	PTPRK	Q15262	Receptor-type tyrosine-protein phosphatase kappa	rs2519093	136141870	9	C	T	0.816	0.167	0.010	4.5E-65	trans
1759	PTPRK	Q15262	Receptor-type tyrosine-protein phosphatase kappa	rs56278466	17875857	10	G	T	0.661	0.089	0.008	4.0E-29	trans
1760	PTPRM	P28827	Receptor-type tyrosine-protein phosphatase mu	rs61804211	161671306	1	T	G	0.105	0.096	0.011	7.5E-20	trans
1760	PTPRM	P28827	Receptor-type tyrosine-protein phosphatase mu	rs34579268	58410554	3	T	C	0.371	0.046	0.007	9.2E-12	trans
1760	PTPRM	P28827	Receptor-type tyrosine-protein phosphatase mu	rs13107325	103188709	4	T	C	0.075	0.117	0.012	2.3E-21	trans
1760	PTPRM	P28827	Receptor-type tyrosine-protein phosphatase mu	rs7768875	32560752	6	C	T	0.672	0.051	0.007	3.9E-13	trans
1760	PTPRM	P28827	Receptor-type tyrosine-protein phosphatase mu	rs2519093	136141870	9	C	T	0.816	0.699	0.010	0.0E+00	trans
1760	PTPRM	P28827	Receptor-type tyrosine-protein phosphatase mu	rs174537	61552680	11	T	G	0.345	0.047	0.007	2.1E-12	trans
1760	PTPRM	P28827	Receptor-type tyrosine-protein phosphatase mu	rs11603123	126305495	11	A	G	0.034	0.178	0.018	1.3E-23	trans
1760	PTPRM	P28827	Receptor-type tyrosine-protein phosphatase mu	rs186021206	7069412	17	A	G	0.006	0.638	0.044	4.1E-47	trans
1760	PTPRM	P28827	Receptor-type tyrosine-protein phosphatase mu	rs72911294	8253602	18	C	T	0.729	0.080	0.008	6.6E-25	cis
1760	PTPRM	P28827	Receptor-type tyrosine-protein phosphatase mu	rs4429561	39799789	22	A	G	0.312	0.058	0.007	4.0E-17	trans
1761	PTPRN2	Q92932	Receptor-type tyrosine-protein phosphatase N2	rs3800860	157903504	7	C	T	0.642	0.155	0.008	1.9E-88	cis
1761	PTPRN2	Q92932	Receptor-type tyrosine-protein phosphatase N2	rs7203642	20367130	16	A	G	0.808	0.064	0.009	7.9E-12	trans
1762	PTPRR	Q15256	Receptor-type tyrosine-protein phosphatase R	rs77924615	20392332	16	G	A	0.803	0.064	0.009	6.2E-12	trans
1763	PTPRS	Q13332	Receptor-type tyrosine-protein phosphatase S	rs1260326	27730940	2	C	T	0.608	0.091	0.007	1.7E-34	trans
1763	PTPRS	Q13332	Receptor-type tyrosine-protein phosphatase S	rs186021206	7069412	17	A	G	0.006	0.433	0.050	5.4E-18	trans
1763	PTPRS	Q13332	Receptor-type tyrosine-protein phosphatase S	rs55763631	5293583	19	A	C	0.048	0.659	0.018	6.1E-278	cis
1763	PTPRS	Q13332	Receptor-type tyrosine-protein phosphatase S	rs180956060	129114784	X	A	G	0.101	0.090	0.011	2.5E-16	trans
1764	PTPRZ1	P23471	Receptor-type tyrosine-protein phosphatase zeta	rs17422797	32265528	6	T	C	0.074	0.095	0.013	5.9E-14	trans
1764	PTPRZ1	P23471	Receptor-type tyrosine-protein phosphatase zeta	rs7797305	121488137	7	C	T	0.513	0.169	0.007	6.4E-139	cis
1764	PTPRZ1	P23471	Receptor-type tyrosine-protein phosphatase zeta	rs35281152	120041968	8	AG	A	0.532	0.057	0.007	2.3E-17	trans
1764	PTPRZ1	P23471	Receptor-type tyrosine-protein phosphatase zeta	rs10956755	135302956	8	A	G	0.700	0.099	0.007	3.4E-42	trans
1764	PTPRZ1	P23471	Receptor-type tyrosine-protein phosphatase zeta	rs56278466	17875857	10	G	T	0.661	0.086	0.007	1.9E-34	trans
1764	PTPRZ1	P23471	Receptor-type tyrosine-protein phosphatase zeta	rs373909380	17405684	16	T	A	0.228	0.112	0.008	2.4E-40	trans
1764	PTPRZ1	P23471	Receptor-type tyrosine-protein phosphatase zeta	rs200489612	7106378	17	A	G	0.005	0.811	0.050	6.2E-59	trans
1764	PTPRZ1	P23471	Receptor-type tyrosine-protein phosphatase zeta	rs12451299	48432324	17	C	G	0.242	0.059	0.008	4.4E-14	trans
1764	PTPRZ1	P23471	Receptor-type tyrosine-protein phosphatase zeta	rs601338	49206674	19	A	G	0.509	0.099	0.007	3.6E-49	trans
1765	PTRHD1	Q6GMV3	Putative peptidyl-tRNA hydrolase PTRHD1	rs1354034	56849749	3	C	T	0.604	0.071	0.008	1.7E-20	trans
1765	PTRHD1	Q6GMV3	Putative peptidyl-tRNA hydrolase PTRHD1	rs7080386	65048306	10	A	C	0.412	0.062	0.008	7.8E-16	trans
1766	PTS	Q03393	6-pyruvoyl tetrahydrobiopterin synthase	rs183616935	52538367	3	C	A	0.006	0.480	0.049	1.6E-22	trans
1766	PTS	Q03393	6-pyruvoyl tetrahydrobiopterin synthase	rs112875651	126506694	8	G	A	0.609	0.054	0.008	2.1E-12	trans
1766	PTS	Q03393	6-pyruvoyl tetrahydrobiopterin synthase	rs12796145	112076587	11	G	C	0.767	0.236	0.009	3.7E-154	cis
1766	PTS	Q03393	6-pyruvoyl tetrahydrobiopterin synthase	rs738408	44324730	22	T	C	0.217	0.086	0.009	1.7E-21	trans
1767	PTX3	P26022	Pentraxin-related protein PTX3	rs678	52820981	3	A	T	0.643	0.079	0.008	9.0E-24	trans
1767	PTX3	P26022	Pentraxin-related protein PTX3	rs56025932	157155858	3	A	C	0.957	0.150	0.019	1.5E-15	cis
1767	PTX3	P26022	Pentraxin-related protein PTX3	rs41341748	16012594	8	A	G	0.011	0.337	0.035	1.7E-21	trans
1767	PTX3	P26022	Pentraxin-related protein PTX3	rs7084817	7774358	10	C	G	0.948	0.636	0.017	0.0E+00	trans
1768	PVALB	P20472	Parvalbumin alpha	rs3811444	248039451	1	C	T	0.668	0.067	0.007	8.6E-24	trans
1768	PVALB	P20472	Parvalbumin alpha	rs10820606	99192919	9	C	A	0.229	0.052	0.008	7.5E-12	trans
1768	PVALB	P20472	Parvalbumin alpha	rs7896518	65104500	10	G	A	0.424	0.059	0.006	1.6E-20	trans
1768	PVALB	P20472	Parvalbumin alpha	rs4821544	37258503	22	T	C	0.697	0.813	0.008	0.0E+00	cis
1768	PVALB	P20472	Parvalbumin alpha	rs5963141	39697946	X	A	C	0.494	0.043	0.005	7.8E-16	trans
1769	PVR	P15151	Poliovirus receptor	rs13234131	73025975	7	A	G	0.873	0.074	0.009	5.6E-16	trans
1769	PVR	P15151	Poliovirus receptor	rs200489612	7106378	17	A	G	0.005	0.314	0.046	8.4E-12	trans
1769	PVR	P15151	Poliovirus receptor	rs2301274	45146402	19	T	C	0.759	0.786	0.009	0.0E+00	cis
1770	PXDNL	A1KZ92	Peroxidasin-like protein	rs7833909	52366300	8	A	G	0.876	0.345	0.012	3.1E-196	cis
1771	PXN	P49023	Paxillin	rs3129754	32583046	6	G	A	0.420	0.056	0.007	7.4E-16	trans
1771	PXN	P49023	Paxillin	rs4632248	54324995	19	T	G	0.210	0.068	0.008	4.2E-16	trans
1772	PYDC1	Q8WXC3	Pyrin domain-containing protein 1	rs61816761	152285861	1	A	G	0.024	0.401	0.023	9.5E-69	trans
1772	PYDC1	Q8WXC3	Pyrin domain-containing protein 1	rs1352846	72617775	4	A	G	0.707	0.706	0.008	0.0E+00	trans
1772	PYDC1	Q8WXC3	Pyrin domain-containing protein 1	rs6557841	21988515	8	A	C	0.385	0.048	0.007	8.5E-13	trans
1772	PYDC1	Q8WXC3	Pyrin domain-containing protein 1	rs10995328	64602608	10	A	G	0.400	0.054	0.007	3.7E-16	trans
1772	PYDC1	Q8WXC3	Pyrin domain-containing protein 1	rs17658212	41145919	15	C	T	0.940	0.175	0.014	2.5E-35	trans
1772	PYDC1	Q8WXC3	Pyrin domain-containing protein 1	rs4561481	31259553	16	A	G	0.674	0.306	0.007	0.0E+00	cis
1772	PYDC1	Q8WXC3	Pyrin domain-containing protein 1	rs112710702	58907700	19	G	A	0.957	0.138	0.016	1.6E-17	trans
1773	PYY	P10082	Peptide YY	rs56092644	95774809	5	T	G	0.245	0.166	0.009	5.4E-79	trans
1773	PYY	P10082	Peptide YY	rs2341378	42040807	17	T	C	0.680	0.245	0.008	9.8E-200	cis
1773	PYY	P10082	Peptide YY	rs72943036	60845776	18	T	C	0.085	0.103	0.013	2.7E-14	trans
1773	PYY	P10082	Peptide YY	rs516246	49206172	19	T	C	0.509	0.069	0.008	4.1E-20	trans
1774	PZP	P20742	Pregnancy zone protein	rs7082470	65277026	10	G	A	0.532	0.058	0.005	2.0E-29	trans
1774	PZP	P20742	Pregnancy zone protein	rs9905583	45741016	17	T	C	0.486	0.037	0.005	3.2E-13	trans
1774	PZP	P20742	Pregnancy zone protein	rs11666424	18218899	19	A	G	0.726	0.039	0.006	8.1E-12	trans
1775	QDPR	P09417	Dihydropteridine reductase	rs1354034	56849749	3	C	T	0.604	0.061	0.007	3.0E-16	trans
1776	QPCT	Q16769	Glutaminyl-peptide cyclotransferase	rs188468174	25291697	1	C	T	0.986	0.398	0.031	2.2E-37	trans
1776	QPCT	Q16769	Glutaminyl-peptide cyclotransferase	rs4670696	37599963	2	A	C	0.976	0.884	0.025	6.7E-281	cis
1776	QPCT	Q16769	Glutaminyl-peptide cyclotransferase	rs4917	186337713	3	C	T	0.640	0.072	0.008	5.1E-21	trans
1776	QPCT	Q16769	Glutaminyl-peptide cyclotransferase	rs73169678	150953205	7	A	C	0.112	0.095	0.012	2.7E-16	trans
1776	QPCT	Q16769	Glutaminyl-peptide cyclotransferase	rs13277237	130604563	8	G	A	0.433	0.070	0.007	2.6E-21	trans
1776	QPCT	Q16769	Glutaminyl-peptide cyclotransferase	rs56278466	17875857	10	G	T	0.662	0.089	0.008	6.0E-31	trans
1776	QPCT	Q16769	Glutaminyl-peptide cyclotransferase	rs10896045	65555524	11	A	G	0.298	0.150	0.008	4.3E-78	trans
1776	QPCT	Q16769	Glutaminyl-peptide cyclotransferase	rs3803800	7462969	17	A	G	0.211	0.063	0.009	1.4E-12	trans
1776	QPCT	Q16769	Glutaminyl-peptide cyclotransferase	rs34562254	16842991	17	A	G	0.097	0.118	0.012	4.6E-22	trans
1776	QPCT	Q16769	Glutaminyl-peptide cyclotransferase	rs34003767	38194296	17	A	G	0.631	0.055	0.008	3.2E-13	trans
1776	QPCT	Q16769	Glutaminyl-peptide cyclotransferase	rs2725405	79220224	17	C	G	0.441	0.051	0.007	5.8E-12	trans
1777	QSOX1	O00391	Sulfhydryl oxidase 1	rs12371	180163390	1	A	G	0.912	1.086	0.013	0.0E+00	cis

1777	QSOX1	O00391	Sulfhydryl oxidase 1	rs10817200	114395586	9	A	G	0.735	0.056	0.008	3.9E-13	trans
1777	QSOX1	O00391	Sulfhydryl oxidase 1	rs186021206	7069412	17	A	G	0.006	0.346	0.046	9.7E-14	trans
1778	RAB10	P61026	Ras-related protein Rab-10	rs1354034	56849749	3	C	T	0.604	0.058	0.008	5.7E-14	trans
1778	RAB10	P61026	Ras-related protein Rab-10	rs7916868	64988931	10	T	A	0.502	0.055	0.008	1.8E-13	trans
1779	RAB11FIP3	O75154	Rab11 family-interacting protein 3	rs7916868	64988931	10	T	A	0.502	0.058	0.007	6.8E-15	trans
1779	RAB11FIP3	O75154	Rab11 family-interacting protein 3	rs74847787	546321	16	T	C	0.969	0.165	0.022	2.8E-14	cis
1780	RAB27B	O00194	Ras-related protein Rab-27B	rs2631367	131705458	5	G	C	0.519	0.057	0.008	2.6E-14	trans
1780	RAB27B	O00194	Ras-related protein Rab-27B	rs342299	106373718	7	C	T	0.545	0.051	0.008	9.4E-12	trans
1780	RAB27B	O00194	Ras-related protein Rab-27B	rs6993770	106581528	8	A	T	0.713	0.099	0.008	1.1E-32	trans
1780	RAB27B	O00194	Ras-related protein Rab-27B	rs7896518	65104500	10	G	A	0.424	0.067	0.008	4.9E-18	trans
1780	RAB27B	O00194	Ras-related protein Rab-27B	rs55656227	55535326	19	A	G	0.812	0.069	0.010	1.2E-12	trans
1781	RAB2B	Q8WUD1	Ras-related protein Rab-2B	rs1354034	56849749	3	C	T	0.604	0.065	0.008	2.6E-17	trans
1781	RAB2B	Q8WUD1	Ras-related protein Rab-2B	rs7916868	64988931	10	T	A	0.501	0.056	0.007	5.1E-14	trans
1781	RAB2B	Q8WUD1	Ras-related protein Rab-2B	rs34855848	21929718	14	C	CA	0.863	0.233	0.011	4.9E-100	cis
1782	RAB44	Q7Z6P3	Ras-related protein Rab-44	rs10424405	54321933	19	G	A	0.212	0.077	0.009	7.1E-17	trans
1783	RAB6A	P20340	Ras-related protein Rab-6A	rs5030062	186454180	3	C	A	0.373	0.196	0.007	1.5E-155	trans
1783	RAB6A	P20340	Ras-related protein Rab-6A	rs4861708	187157233	4	A	G	0.512	0.390	0.007	0.0E+00	trans
1783	RAB6A	P20340	Ras-related protein Rab-6A	rs1801020	176836532	5	G	A	0.745	0.209	0.008	1.0E-145	trans
1783	RAB6A	P20340	Ras-related protein Rab-6A	rs821840	56993886	16	G	A	0.325	0.068	0.008	1.4E-19	trans
1784	RABEP1	Q15276	Rab GTPase-binding effector protein 1	rs1354034	56849749	3	C	T	0.604	0.061	0.008	5.1E-16	trans
1784	RABEP1	Q15276	Rab GTPase-binding effector protein 1	rs342293	106372219	7	C	G	0.540	0.051	0.007	9.2E-12	trans
1784	RABEP1	Q15276	Rab GTPase-binding effector protein 1	rs6993770	106581528	8	A	T	0.713	0.059	0.008	6.5E-13	trans
1785	RABEPK	Q7Z6M1	Rab9 effector protein with kelch motifs	rs7858935	127986904	9	T	A	0.147	0.207	0.011	5.2E-80	cis
1786	RABGAP1L	Q5R372	Rab GTPase-activating protein 1-like	rs6661868	174076767	1	G	A	0.201	0.092	0.009	1.4E-22	cis
1786	RABGAP1L	Q5R372	Rab GTPase-activating protein 1-like	rs1354034	56849749	3	C	T	0.604	0.057	0.008	5.9E-14	trans
1787	RAD23B	P54727	UV excision repair protein RAD23 homolog B	rs6993770	106581528	8	A	T	0.713	0.061	0.008	5.9E-13	trans
1787	RAD23B	P54727	UV excision repair protein RAD23 homolog B	rs7035725	110049914	9	C	G	0.450	0.064	0.008	4.5E-17	cis
1788	RALB	P11234	Ras-related protein Ral-B	rs148102406	121035039	2	T	TGTGA	0.317	0.117	0.008	1.7E-44	cis
1789	RALY	Q9UKM9	RNA-binding protein Raly	rs7080536	115348046	10	G	A	0.956	0.162	0.019	6.0E-18	trans
1790	RANBP1	P43487	Ran-specific GTPase-activating protein	rs1354034	56849749	3	C	T	0.605	0.060	0.008	3.6E-15	trans
1790	RANBP1	P43487	Ran-specific GTPase-activating protein	rs76889809	20070956	22	A	C	0.055	0.123	0.017	1.2E-13	cis
1791	RAP1A	P62834	Ras-related protein Rap-1A	rs11571302	204742934	2	G	T	0.525	0.061	0.008	2.4E-15	trans
1791	RAP1A	P62834	Ras-related protein Rap-1A	rs13263709	81287175	8	C	T	0.649	0.055	0.008	6.7E-12	trans
1792	RARRES1	P49788	Retinoic acid receptor responder protein 1	rs6441224	158450417	3	C	T	0.525	0.457	0.008	0.0E+00	cis
1793	RARRES2	Q99969	Retinoic acid receptor responder protein 2	rs1260326	27730940	2	T	C	0.392	0.054	0.008	7.1E-13	trans
1793	RARRES2	Q99969	Retinoic acid receptor responder protein 2	rs1265905	31909941	6	A	G	0.870	0.094	0.011	6.0E-18	trans
1793	RARRES2	Q99969	Retinoic acid receptor responder protein 2	rs3735167	150039555	7	T	C	0.256	0.216	0.008	3.3E-144	cis
1794	RASSF2	P50749	Ras association domain-containing protein 2	rs2246735	4803494	20	T	C	0.573	0.095	0.008	8.1E-36	cis
1795	RBFOX3	A6NFN3	RNA binding protein fox-1 homolog 3	rs7080536	115348046	10	G	A	0.956	0.122	0.017	1.2E-12	trans
1796	RBKS	Q9H477	Ribokinase	rs140948699	28081439	2	C	G	0.991	1.812	0.039	0.0E+00	cis
1797	RBM17	Q96I25	Splicing factor 45	rs342293	106372219	7	C	G	0.540	0.075	0.008	3.9E-23	trans
1797	RBM17	Q96I25	Splicing factor 45	rs6993770	106581528	8	A	T	0.714	0.079	0.008	1.5E-21	trans
1797	RBM17	Q96I25	Splicing factor 45	rs11256771	6146262	10	C	T	0.963	0.378	0.020	7.4E-81	cis
1798	RBP1	P09455	Retinol-binding protein 1	rs2071387	139257603	3	G	A	0.182	0.137	0.010	1.0E-42	cis
1798	RBP1	P09455	Retinol-binding protein 1	rs607929	32586998	6	C	G	0.551	0.062	0.008	1.1E-15	trans
1799	RBP2	P50120	Retinol-binding protein 2	rs1375131	135954797	2	C	T	0.265	0.059	0.009	1.3E-11	trans
1799	RBP2	P50120	Retinol-binding protein 2	rs295469	139212900	3	G	A	0.622	0.068	0.008	1.4E-18	cis
1799	RBP2	P50120	Retinol-binding protein 2	rs6472539	71629810	8	G	C	0.485	0.062	0.008	1.6E-16	trans
1799	RBP2	P50120	Retinol-binding protein 2	rs17655730	270715	11	C	T	0.246	0.112	0.009	2.4E-37	trans
1800	RBP5	P82980	Retinol-binding protein 5	rs112875651	126506694	8	G	A	0.609	0.051	0.007	4.0E-12	trans
1800	RBP5	P82980	Retinol-binding protein 5	rs1110236	95902595	9	G	A	0.865	0.090	0.010	5.0E-18	trans
1800	RBP5	P82980	Retinol-binding protein 5	rs11370710	117158957	9	C	CA	0.506	0.053	0.007	8.7E-14	trans
1801	RBP7	Q96R05	Retinoid-binding protein 7	rs12990177	43682659	2	A	T	0.481	0.049	0.007	1.1E-11	trans
1801	RBP7	Q96R05	Retinoid-binding protein 7	rs9657541	10643164	8	C	T	0.795	0.060	0.009	6.9E-12	trans
1801	RBP7	Q96R05	Retinoid-binding protein 7	rs34436714	54327313	19	A	C	0.209	0.325	0.009	2.3E-295	trans
1802	RBPMS2	Q6ZRY4	RNA-binding protein with multiple splicing 2	rs1354034	56849749	3	C	T	0.605	0.100	0.008	3.2E-39	trans
1802	RBPMS2	Q6ZRY4	RNA-binding protein with multiple splicing 2	rs342299	106373718	7	C	T	0.545	0.055	0.008	2.7E-13	trans
1802	RBPMS2	Q6ZRY4	RNA-binding protein with multiple splicing 2	rs6993770	106581528	8	A	T	0.713	0.056	0.008	1.2E-11	trans
1802	RBPMS2	Q6ZRY4	RNA-binding protein with multiple splicing 2	rs73396521	267568	11	A	G	0.245	0.061	0.009	3.8E-12	trans
1802	RBPMS2	Q6ZRY4	RNA-binding protein with multiple splicing 2	rs28711906	65062278	15	A	T	0.048	0.181	0.018	6.7E-25	cis
1803	RCC1	P18754	Regulator of chromosome condensation	rs12881562	106461353	14	G	C	0.146	0.077	0.011	7.5E-12	trans
1804	RCOR1	Q9UKL0	REST corepressor 1	rs7214731	64212366	17	T	C	0.277	0.062	0.009	5.4E-13	trans
1805	RECK	O95980	Reversion-inducing cysteine-rich protein with Kazal motifs	rs13135092	103198082	4	G	A	0.084	0.087	0.013	1.3E-11	trans
1805	RECK	O95980	Reversion-inducing cysteine-rich protein with Kazal motifs	rs71521299	36058816	9	T	G	0.984	0.853	0.029	1.8E-190	cis
1805	RECK	O95980	Reversion-inducing cysteine-rich protein with Kazal motifs	rs10793962	136129115	9	T	A	0.061	0.134	0.015	3.1E-19	trans
1805	RECK	O95980	Reversion-inducing cysteine-rich protein with Kazal motifs	rs56278466	17875857	10	G	T	0.661	0.091	0.007	2.6E-34	trans
1805	RECK	O95980	Reversion-inducing cysteine-rich protein with Kazal motifs	rs603424	102075479	10	G	A	0.834	0.069	0.010	5.2E-13	trans
1805	RECK	O95980	Reversion-inducing cysteine-rich protein with Kazal motifs	rs186021206	7069412	17	A	G	0.006	0.354	0.048	2.3E-13	trans
1805	RECK	O95980	Reversion-inducing cysteine-rich protein with Kazal motifs	rs4760	44153100	19	A	G	0.844	0.070	0.010	6.9E-13	trans
1806	REEP4	Q9H6H4	Receptor expression-enhancing protein 4	rs1354034	56849749	3	C	T	0.604	0.063	0.008	1.5E-16	trans
1806	REEP4	Q9H6H4	Receptor expression-enhancing protein 4	rs117291678	21993410	8	G	C	0.982	0.597	0.028	2.0E-100	cis
1807	REG1A	P05451	Lithostathine-1-alpha	rs11126696	79323888	2	G	A	0.618	0.251	0.008	3.2E-242	cis
1807	REG1A	P05451	Lithostathine-1-alpha	rs4421693	124306428	10	T	G	0.576	0.189	0.007	1.2E-146	trans
1807	REG1A	P05451	Lithostathine-1-alpha	rs708686	5840619	19	T	C	0.268	0.106	0.008	2.6E-39	trans
1807	REG1A	P05451	Lithostathine-1-alpha	rs679574	49206108	19	C	G	0.491	0.186	0.007	7.5E-147	trans
1808	REG1B	P48304	Lithostathine-1-beta	rs11126696	79323888	2	G	A	0.619	0.345	0.008	0.0E+00	cis
1808	REG1B	P48304	Lithostathine-1-beta	rs4421693	124306428	10	T	G	0.576	0.119	0.007	6.1E-61	trans
1808	REG1B	P48304	Lithostathine-1-beta	rs72802342	75234872	16	C	A	0.924	0.189	0.014	8.4E-44	trans
1808	REG1B	P48304	Lithostathine-1-beta	rs708686	5840619	19	T	C	0.268	0.090	0.008	1.7E-29	trans
1808	REG1B	P48304	Lithostathine-1-beta	rs492602	49206417	19	A	G	0.491	0.183	0.007	1.9E-144	trans
1809	REG3A	Q06141	Regenerating islet-derived protein 3-alpha	rs147616162	79395511	2	A	T	0.059	0.407	0.015	9.8E-156	cis
1809	REG3A	Q06141	Regenerating islet-derived protein 3-alpha	rs11707109	40914588	3	A	G	0.856	0.079	0.010	9.9E-15	trans
1809	REG3A	Q06141	Regenerating islet-derived protein 3-alpha	rs35846682	41682486	6	T	C	0.092	0.127	0.012	7.4E-25	trans
1809	REG3A	Q06141	Regenerating islet-derived protein 3-alpha	rs12201703	106402308	6	G	A	0.921	0.165	0.013	3.0E-36	trans
1809	REG3A	Q06141	Regenerating islet-derived protein 3-alpha	rs11761603	1286912	7	C	T	0.700	0.056	0.008	2.2E-12	trans
1809	REG3A	Q06141	Regenerating islet-derived protein 3-alpha	rs13253974	23377910	8	G	A	0.676	0.061	0.008	7.9E-16	trans
1809	REG3A	Q06141	Regenerating islet-derived protein 3-alpha	rs17338413	75474936	15	C	T	0.922	0.353	0.013	7.4E-155	trans
1810	REG3G	Q6UW15	Regenerating islet-derived protein 3-gamma	rs2861742	79307817	2	A	G	0.253	0.287	0.009	3.8E-239	cis
1811	REG4	Q9BYZ8	Regenerating islet-derived protein 4	rs79795228	120359286	1	A	C	0.017	0.633	0.028	2.8E-115	cis
1811	REG4	Q9BYZ8	Regenerating islet-derived protein 4	rs137872268	144879054	1	C	T	0.018	0.260	0.028	7.5E-21	trans
1812	RELB	Q01201	Transcription factor RelB	rs228682	7856346	1	C	T	0.395	0.090	0.008	2.4E-30	trans
1813	RELT	Q969Z4	Tumor necrosis factor receptor superfamily member 19L	rs13146355	77412140	4	A	G	0.457	0.047	0.007	8.3E-13	trans
1813	RELT	Q969Z4	Tumor necrosis factor receptor superfamily member 19L	rs11235729	73098326	11	A	G	0.207	0.399	0.008	0.0E+00	cis
1813	RELT	Q969Z4	Tumor necrosis factor receptor superfamily member 19L	rs77924615	20392332	16	G	A	0.803	0.064	0.008	1.7E-14	trans
1814	REN	P00797	Renin	rs1288923	163607767	1	G	A	0.443	0.058	0.007	1.4E-15	trans
1814	REN	P00797	Renin	rs193280350	204148649	1	A	G	0.008	0.838	0.043	1.1E-85	cis
1814	REN	P00797	Renin	rs5051	230849872	1	C	T	0.601	0.050	0.007	7.4E-12	trans
1814	REN	P00797	Renin	rs116219813	204081675	2	T	C	0.128	0.098	0.011	5.2E-20	trans
1815	RET	P07949	Proto-oncogene tyrosine-protein kinase receptor Ret	rs6747755	101578458	2	A	G	0.221	0.060	0.008	1.1E-12	trans
1815	RET	P07949	Proto-oncogene tyrosine-protein kinase receptor Ret	rs6762552	98431207	3	C	T	0.310	0.083	0.008	7.1E-28	trans
1815	RET	P07949	Proto-oncogene tyrosine-protein kinase receptor Ret	rs10819413	101639209	9	T	C	0.392	0.049	0.007	8.3E-12	trans
1815	RET	P07949	Proto-oncogene tyrosine-protein kinase receptor Ret	rs189906184	38988693	10	T	A	0.024	0.393	0.025	1.5E-56	trans
1815	RET	P07949	Proto-oncogene tyrosine-protein kinase receptor Ret	rs2744077	43364506	10	T	C	0.175	0.471	0.010	0.0E+00	cis
1815	RET	P07949	Proto-oncogene tyrosine-protein kinase receptor Ret	rs35887873	126219396	11	C	T	0.229	0.082	0.008	9.4E-23	trans
1815	RET	P07949	Proto-oncogene tyrosine-protein kinase receptor Ret	rs186021206	7069412	17	A	G	0.006	0.465	0.049	1.0E-21	trans
1815	RET	P07949	Proto-oncogene tyrosine-protein kinase receptor Ret	rs9952412	46465540	18	C	A	0.517	0.048	0.007	8.6E-12	trans
1816	RETN	Q9HD89	Resistin	rs3917932	36943916	1	C	G	0.424	0.062	0.007	4.3E-17	trans
1816	RETN	Q9HD89	Resistin	rs17405635	43355763	2	A	G	0.256	0.098	0.008	2.1E-31	trans
1816	RETN	Q9HD89	Resistin	rs2305637	47045846	3	T	C	0.176	0.076	0.010	2.1E-15	trans
1816	RETN	Q9HD89	Resistin	rs9880192	128297569	3	G	C	0.586	0.052	0.007	3.0E-12	trans
1816	RETN	Q9HD89	Resistin	rs218264	55408875	4	T	A	0.247	0.057	0.008	1.4E-11	trans
1816	RETN	Q9HD89	Resistin	rs13107325	103188709	4	T	C	0.075	0.109	0.014	3.9E-15	trans
1816	RETN	Q9HD89	Resistin	rs149110519	144385777	6	T	C	0.036	0.384	0.020	5.5E-86	trans
1816	RETN	Q9HD89	Resistin	rs445	92408370	7	C	T	0.905	0.110	0.012	4.6E-19	trans
1816	RETN	Q9HD89	Resistin	rs1865223	130606476	8	G	T	0.432	0.086	0.007	8.1E-32	trans
1816	RETN	Q9HD89	Resistin	rs2074038	44087989	11	T	G	0.100	0.115	0.012	1.5E-21	trans
1816	RETN	Q9HD89	Resistin	rs11642657	86016328	16	T	C	0.008	0.313	0.044	8.8E-13	trans
1816	RETN	Q9HD89	Resistin	rs6503533	38184580	17	C	T	0.384	0.109	0.007	1.3E-48	trans
1816	RETN	Q9HD89	Resistin	rs3219175	7733855	19	A	G	0.008	1.543	0.046	2.4E-242	cis
1816	RETN	Q9HD89	Resistin	rs6069597	36922727	20	T	C	0.469	0.060	0.007	8.6E-17	trans
1816	RETN	Q9HD89	Resistin	rs243591	19155806	21	A	C	0.521	0.060	0.007	2.6E-16	trans
1817	RFC4	P35249	Replication factor C subunit 4	rs200303599	140932849	5	TA	T	0.005	0.800	0.053	3.2E-51	trans
1818	RGCC	Q9H4X1	Regulator of cell cycle RGCC	rs3811444	248039451	1	C	T	0.667	0.062	0.008	6.4E-15	trans
1818	RGCC	Q9H4X1	Regulator of cell cycle RGCC	rs12767683	104316581	10	A	C	0.325	0.059	0.008	1.0E-13	trans
1819	RGMA	Q96B86	Repulsive guidance molecule A	rs147440039	145395364	1	C	G	0.969	0.151	0.021	3.3E-13	trans
1819	RGMA	Q96B86	Repulsive guidance molecule A	rs1260326	27730940	2	C	T	0.608	0.080	0.007	1.3E-28	trans
1819	RGMA	Q96B86	Repulsive guidance molecule A	rs6804368	30753573	3	A	G	0.556	0.066	0.007	2.4E-20	trans
1819	RGMA	Q96B86	Repulsive guidance molecule A	rs13147041	83872675	4	C	T	0.639	0.055	0.008	2.4E-13	trans
1819	RGMA	Q96B86	Repulsive guidance molecule A	rs11956061	126245898	5	T	C	0.481	0.069	0.007	3.2E-22	trans
1819	RGMA	Q96B86	Repulsive guidance molecule A	rs4841132	9183596	8	A	G	0.092	0.085	0.012	4.9E-12	trans
1819	RGMA	Q96B86	Repulsive guidance molecule A	rs1885372	35838030	9	A	G	0.593	0.283	0.007	0.0E+00	trans
1819	RGMA	Q96B86	Repulsive guidance molecule A	rs4775282	60937924	15	A	T	0.646	0.058	0.008	1.7E-14	trans
1819	RGMA	Q96B86	Repulsive guidance molecule A	rs56058910	73126038	15	C	T	0.138	0.081	0.011	1.5E-14	trans
1819	RGMA	Q96B86	Repulsive guidance molecule A	rs3752102	93616014	15	C	A	0.531	0.227	0.007	3.0E-215	cis
1819	RGMA	Q96B86	Repulsive guidance molecule A	rs117353460	89306703	16	T	C	0.033	0.159	0.020	8.3E-16	trans
1819	RGMA	Q96B86	Repulsive guidance molecule A	rs2904766	37848677	17	G	A	0.670	0.060	0.008	1.9E-15	trans
1820	RGMB	Q6NW40	RGM domain family member B	rs61747728	179526214	1	T	C	0.038	0.138	0.019	2.4E-13	trans
1820	RGMB	Q6NW40	RGM domain family member B	rs56870134	97773523	5	T	C	0.548	0.171	0.007	1.1E-116	cis
1820	RGMB	Q6NW40	RGM domain family member B	rs34262842	20355811	16	A	G	0.834	0.084	0.010	5.8E-18	trans
1821	RGS10	O43665	Regulator of G-protein signaling 10	rs17622656	131820997	5	G	A	0.612	0.055	0.008	9.8E-13	trans
1821	RGS10	O43665	Regulator of G-protein signaling 10	rs6993770	106581528	8	A	T	0.714	0.059	0.008	1.0E-12	trans
1822	RGS8	P57771	Regulator of G-protein signaling 8	rs34917868	107074334	14	A	G	0.142	0.239	0.011	1.2E-101	trans
1823	RHOC	P08134	Rho-related GTP-binding protein RhoC	rs1006298	113061959	1	C	G	0.482	0.084	0.008	3.9E-28	cis

1823	RHOC	P08134	Rho-related GTP-binding protein RhoC	rs1354034	56849749	3	C	T	0.604	0.063	0.008	3.7E-16	trans
1823	RHOC	P08134	Rho-related GTP-binding protein RhoC	rs342293	106372219	7	C	G	0.541	0.054	0.008	1.5E-12	trans
1824	RIDA	P52758	2-iminobutanoate/2-iminopropanoate deaminase	rs77539382	99120794	8	T	TC	0.681	0.318	0.008	0.0E+00	cis
1824	RIDA	P52758	2-iminobutanoate/2-iminopropanoate deaminase	rs7041363	117146043	9	C	G	0.510	0.060	0.007	9.5E-17	trans
1824	RIDA	P52758	2-iminobutanoate/2-iminopropanoate deaminase	rs3747207	44324855	22	A	G	0.215	0.072	0.009	2.0E-16	trans
1825	RILP	Q96NA2	Rab-interacting lysosomal protein	rs1354034	56849749	3	C	T	0.604	0.079	0.007	3.6E-26	trans
1825	RILP	Q96NA2	Rab-interacting lysosomal protein	rs6993770	106581528	8	A	T	0.714	0.070	0.008	9.8E-18	trans
1825	RILP	Q96NA2	Rab-interacting lysosomal protein	rs11604127	196944	11	T	C	0.231	0.060	0.009	7.5E-12	trans
1825	RILP	Q96NA2	Rab-interacting lysosomal protein	rs183827902	1552519	17	T	C	0.017	0.268	0.028	3.9E-21	cis
1826	RILPL2	Q969X0	RILP-like protein 2	rs736801	131833599	5	C	T	0.612	0.053	0.008	4.7E-12	trans
1826	RILPL2	Q969X0	RILP-like protein 2	rs6993770	106581528	8	A	T	0.713	0.070	0.008	1.8E-17	trans
1827	RIPK4	P57078	Receptor-interacting serine/threonine-protein kinase 4	rs3829431	106361807	14	A	G	0.161	0.115	0.011	1.1E-25	trans
1828	RLN2	P04090	Prorelaxin H2	rs41264945	155640115	1	T	C	0.056	0.192	0.016	5.4E-33	trans
1828	RLN2	P04090	Prorelaxin H2	rs68066031	224880498	2	T	C	0.770	0.070	0.009	5.1E-15	trans
1828	RLN2	P04090	Prorelaxin H2	rs2273782	5335470	9	T	C	0.226	0.158	0.009	3.7E-70	cis
1829	RNASE1	P07998	Ribonuclease pancreatic	rs4859682	77410318	4	A	C	0.456	0.050	0.007	2.5E-12	trans
1829	RNASE1	P07998	Ribonuclease pancreatic	rs17254387	21280678	14	A	G	0.694	0.217	0.008	1.1E-163	cis
1830	RNASE10	Q5GAN6	Inactive ribonuclease-like protein 10	rs7144945	20971442	14	C	T	0.493	0.803	0.008	0.0E+00	cis
1831	RNASE3	P12724	Eosinophil cationic protein	rs28498283	43360065	2	T	A	0.255	0.064	0.009	4.7E-14	trans
1831	RNASE3	P12724	Eosinophil cationic protein	rs75004383	65086229	2	A	G	0.042	0.129	0.018	1.7E-12	trans
1831	RNASE3	P12724	Eosinophil cationic protein	rs147307766	21385991	14	T	C	0.054	0.569	0.017	1.8E-254	cis
1832	RNASE4	P34096	Ribonuclease 4	rs780094	27741237	2	T	C	0.380	0.056	0.007	3.0E-14	trans
1832	RNASE4	P34096	Ribonuclease 4	rs1047891	211540507	2	C	A	0.687	0.057	0.008	1.5E-13	trans
1832	RNASE4	P34096	Ribonuclease 4	rs12588573	21146584	14	C	T	0.782	0.365	0.009	0.0E+00	cis
1833	RNASE6	Q93091	Ribonuclease K6	rs9272278	32603656	6	G	C	0.156	0.101	0.009	2.2E-26	trans
1833	RNASE6	Q93091	Ribonuclease K6	rs1045922	21250124	14	G	A	0.766	0.445	0.009	0.0E+00	cis
1833	RNASE6	Q93091	Ribonuclease K6	rs145078947	93652974	14	T	G	0.003	0.647	0.072	3.8E-19	trans
1834	RNASEH2A	O75792	Ribonuclease H2 subunit A	rs7247284	12921186	19	T	C	0.967	0.286	0.021	6.3E-42	cis
1835	RNASET2	O00584	Ribonuclease T2	rs61747728	179526214	1	T	C	0.038	0.133	0.018	8.8E-14	trans
1835	RNASET2	O00584	Ribonuclease T2	rs56193304	203644537	2	G	T	0.136	0.073	0.010	6.5E-13	trans
1835	RNASET2	O00584	Ribonuclease T2	rs13107325	103188709	4	T	C	0.075	0.122	0.013	9.8E-21	trans
1835	RNASET2	O00584	Ribonuclease T2	rs3756838	167371251	6	A	G	0.215	0.464	0.009	0.0E+00	cis
1835	RNASET2	O00584	Ribonuclease T2	rs145078947	93652974	14	T	G	0.003	1.331	0.068	6.1E-86	trans
1836	RNF149	Q8NC42	E3 ubiquitin-protein ligase RNF149	rs61747728	179526214	1	T	C	0.038	0.137	0.019	7.3E-13	trans
1836	RNF149	Q8NC42	E3 ubiquitin-protein ligase RNF149	rs12621632	101897325	2	T	C	0.406	0.281	0.008	1.2E-297	cis
1836	RNF149	Q8NC42	E3 ubiquitin-protein ligase RNF149	rs10131857	106510674	14	C	T	0.442	0.111	0.008	6.4E-49	trans
1837	RNF168	Q8IYW5	E3 ubiquitin-protein ligase RNF168	rs74750549	32511488	6	C	T	0.707	0.062	0.009	1.2E-11	trans
1838	RNF43	Q68DV7	E3 ubiquitin-protein ligase RNF43	rs28929474	94844947	14	T	C	0.021	0.202	0.027	5.6E-14	trans
1838	RNF43	Q68DV7	E3 ubiquitin-protein ligase RNF43	rs34523089	56436109	17	T	C	0.157	0.139	0.011	3.4E-40	cis
1839	RNF5	Q99942	E3 ubiquitin-protein ligase RNF5	rs1354034	56849749	3	C	T	0.605	0.098	0.008	1.2E-36	trans
1839	RNF5	Q99942	E3 ubiquitin-protein ligase RNF5	rs11794772	99118445	9	G	A	0.742	0.080	0.009	2.9E-20	trans
1839	RNF5	Q99942	E3 ubiquitin-protein ligase RNF5	rs1976848	201584	11	A	T	0.214	0.083	0.010	5.8E-18	trans
1839	RNF5	Q99942	E3 ubiquitin-protein ligase RNF5	rs11231074	62161714	11	A	G	0.715	0.106	0.008	3.4E-36	trans
1839	RNF5	Q99942	E3 ubiquitin-protein ligase RNF5	rs11671654	12014355	19	G	C	0.495	0.090	0.008	2.5E-32	trans
1840	ROBO1	Q9Y6N7	Roundabout homolog 1	rs12752838	8913656	1	G	A	0.506	0.060	0.007	1.7E-16	trans
1840	ROBO1	Q9Y6N7	Roundabout homolog 1	rs3773244	78784770	3	A	G	0.197	0.236	0.009	3.6E-143	cis
1840	ROBO1	Q9Y6N7	Roundabout homolog 1	rs10935478	98428155	3	C	T	0.555	0.060	0.007	7.5E-16	trans
1840	ROBO1	Q9Y6N7	Roundabout homolog 1	rs3849768	39418887	5	A	C	0.209	0.064	0.009	8.6E-13	trans
1840	ROBO1	Q9Y6N7	Roundabout homolog 1	rs116017457	32521909	6	G	T	0.651	0.089	0.008	2.1E-29	trans
1840	ROBO1	Q9Y6N7	Roundabout homolog 1	rs9987289	9183358	8	A	G	0.092	0.085	0.013	1.5E-11	trans
1840	ROBO1	Q9Y6N7	Roundabout homolog 1	rs56278466	17875857	10	G	T	0.662	0.120	0.008	6.4E-55	trans
1840	ROBO1	Q9Y6N7	Roundabout homolog 1	rs186021206	7069412	17	A	G	0.006	0.674	0.050	2.5E-41	trans
1840	ROBO1	Q9Y6N7	Roundabout homolog 1	rs77542162	67081278	17	G	A	0.023	0.186	0.024	2.0E-14	trans
1840	ROBO1	Q9Y6N7	Roundabout homolog 1	rs5964488	65253769	X	C	T	0.152	0.089	0.008	5.4E-26	trans
1841	ROBO2	Q9HCK4	Roundabout homolog 2	rs775722	77643616	3	T	C	0.554	0.103	0.007	2.6E-47	cis
1841	ROBO2	Q9HCK4	Roundabout homolog 2	rs67370636	32523963	6	T	C	0.334	0.078	0.008	4.0E-22	trans
1841	ROBO2	Q9HCK4	Roundabout homolog 2	rs9987289	9183358	8	A	G	0.092	0.112	0.012	2.3E-20	trans
1841	ROBO2	Q9HCK4	Roundabout homolog 2	rs56278466	17875857	10	G	T	0.661	0.060	0.007	4.9E-16	trans
1841	ROBO2	Q9HCK4	Roundabout homolog 2	rs186021206	7069412	17	A	G	0.006	0.487	0.048	3.0E-24	trans
1842	ROBO4	Q8WZ75	Roundabout homolog 4	rs2519093	136141870	9	C	T	0.816	0.541	0.010	0.0E+00	trans
1842	ROBO4	Q8WZ75	Roundabout homolog 4	rs59648931	124767067	11	T	C	0.769	0.104	0.009	8.7E-33	cis
1842	ROBO4	Q8WZ75	Roundabout homolog 4	rs200489612	7106378	17	A	G	0.005	0.383	0.055	2.2E-12	trans
1843	ROR1	Q01973	Inactive tyrosine-protein kinase transmembrane receptor ROR1	rs7532110	47961964	1	A	T	0.803	0.067	0.009	3.5E-13	trans
1843	ROR1	Q01973	Inactive tyrosine-protein kinase transmembrane receptor ROR1	rs6588083	64614011	1	T	C	0.830	0.394	0.010	0.0E+00	cis
1843	ROR1	Q01973	Inactive tyrosine-protein kinase transmembrane receptor ROR1	rs150816167	179571862	1	C	T	0.042	0.207	0.019	6.8E-29	trans
1843	ROR1	Q01973	Inactive tyrosine-protein kinase transmembrane receptor ROR1	rs1260326	27730940	2	C	T	0.608	0.063	0.007	9.6E-18	trans
1843	ROR1	Q01973	Inactive tyrosine-protein kinase transmembrane receptor ROR1	rs75166367	162964301	2	G	A	0.940	0.107	0.015	9.0E-13	trans
1843	ROR1	Q01973	Inactive tyrosine-protein kinase transmembrane receptor ROR1	rs13107325	103188709	4	T	C	0.075	0.103	0.014	3.3E-14	trans
1843	ROR1	Q01973	Inactive tyrosine-protein kinase transmembrane receptor ROR1	rs2298475	126278203	11	T	C	0.925	0.102	0.013	2.2E-14	trans
1843	ROR1	Q01973	Inactive tyrosine-protein kinase transmembrane receptor ROR1	rs77924615	20392332	16	G	A	0.803	0.077	0.009	1.3E-17	trans
1843	ROR1	Q01973	Inactive tyrosine-protein kinase transmembrane receptor ROR1	rs186021206	7069412	17	A	G	0.006	0.465	0.049	1.5E-21	trans
1843	ROR1	Q01973	Inactive tyrosine-protein kinase transmembrane receptor ROR1	rs2659005	79218714	17	T	C	0.441	0.066	0.007	2.1E-20	trans
1843	ROR1	Q01973	Inactive tyrosine-protein kinase transmembrane receptor ROR1	rs33950747	36339247	19	T	C	0.075	0.133	0.013	7.2E-23	trans
1843	ROR1	Q01973	Inactive tyrosine-protein kinase transmembrane receptor ROR1	rs519725	112103635	X	G	A	0.782	0.057	0.007	1.5E-15	trans
1844	RP2	O75695	Protein XRP2	rs429358	45411941	19	C	T	0.155	0.145	0.011	5.2E-43	trans
1844	RP2	O75695	Protein XRP2	rs1805147	46719498	X	C	T	0.968	0.663	0.018	3.0E-301	cis
1845	RPA2	P15927	Replication protein A 32 kDa subunit	rs17185038	28219658	1	G	C	0.067	0.204	0.015	6.2E-40	cis
1846	RPE	Q96AT9	Ribulose-phosphate 3-epimerase	rs2723211	210880492	2	A	G	0.447	0.057	0.008	7.2E-14	cis
1846	RPE	Q96AT9	Ribulose-phosphate 3-epimerase	rs9271696	32593085	6	T	C	0.620	0.057	0.008	5.4E-13	trans
1847	RPL14	P50914	60S ribosomal protein L14	rs148850234	40486865	3	C	T	0.948	0.118	0.017	8.3E-12	cis
1847	RPL14	P50914	60S ribosomal protein L14	rs1354034	56849749	3	C	T	0.605	0.064	0.008	2.0E-16	trans
1848	RRAS	P10301	Ras-related protein R-Ras	rs342298	106373646	7	C	T	0.545	0.058	0.008	4.4E-14	trans
1849	RRM2	P31350	Ribonucleoside-diphosphate reductase subunit M2	rs7247412	52313868	19	T	C	0.733	0.067	0.009	1.6E-14	trans
1850	RRM2B	Q7LG56	Ribonucleoside-diphosphate reductase subunit M2 B	rs1354034	56849749	3	C	T	0.604	0.089	0.008	1.1E-30	trans
1850	RRM2B	Q7LG56	Ribonucleoside-diphosphate reductase subunit M2 B	rs5893603	103250839	8	CG	C	0.078	0.194	0.014	3.2E-42	cis
1850	RRM2B	Q7LG56	Ribonucleoside-diphosphate reductase subunit M2 B	rs11604127	196944	11	T	C	0.231	0.062	0.009	1.4E-11	trans
1851	RSPO1	Q2MKA7	R-spondin-1	rs36043533	38079517	1	G	T	0.056	0.478	0.016	3.9E-194	cis
1851	RSPO1	Q2MKA7	R-spondin-1	rs13412535	224874874	2	G	A	0.769	0.060	0.009	3.3E-12	trans
1851	RSPO1	Q2MKA7	R-spondin-1	rs1354034	56849749	3	T	C	0.396	0.055	0.007	1.4E-13	trans
1851	RSPO1	Q2MKA7	R-spondin-1	rs6993770	106581528	8	A	T	0.713	0.089	0.008	3.4E-28	trans
1851	RSPO1	Q2MKA7	R-spondin-1	rs61469632	135861990	9	T	C	0.937	0.109	0.015	3.8E-13	trans
1851	RSPO1	Q2MKA7	R-spondin-1	rs12445050	81870969	16	T	C	0.138	0.078	0.011	1.3E-13	trans
1851	RSPO1	Q2MKA7	R-spondin-1	rs892090	55539072	19	G	T	0.834	0.106	0.010	3.2E-27	trans
1851	RSPO1	Q2MKA7	R-spondin-1	rs13651	43974079	20	T	C	0.488	0.085	0.007	1.1E-31	trans
1852	RSPO3	Q9BXY4	R-spondin-3	rs1936800	127436064	6	T	C	0.527	0.157	0.007	9.8E-112	cis
1852	RSPO3	Q9BXY4	R-spondin-3	rs11751347	161092438	6	C	T	0.896	0.119	0.012	4.3E-25	trans
1852	RSPO3	Q9BXY4	R-spondin-3	rs6993770	106581528	8	A	T	0.714	0.063	0.008	2.5E-16	trans
1852	RSPO3	Q9BXY4	R-spondin-3	rs8050794	3743046	16	C	T	0.704	0.063	0.008	1.1E-16	trans
1852	RSPO3	Q9BXY4	R-spondin-3	rs77924615	20392332	16	G	A	0.803	0.061	0.009	4.7E-12	trans
1852	RSPO3	Q9BXY4	R-spondin-3	rs892090	55539072	19	G	T	0.834	0.076	0.009	4.0E-16	trans
1852	RSPO3	Q9BXY4	R-spondin-3	rs2743388	43973201	20	A	C	0.487	0.058	0.007	6.5E-17	trans
1853	RTBDN	Q9BSG5	Retbindin	rs7687767	57824932	4	G	A	0.184	0.070	0.010	2.8E-13	trans
1853	RTBDN	Q9BSG5	Retbindin	rs7255045	12932269	19	G	A	0.738	0.171	0.008	1.2E-91	cis
1854	RTN4IP1	Q8WWV3	Reticulon-4-interacting protein 1, mitochondrial	rs2054365	107077502	6	A	G	0.410	0.093	0.008	2.9E-34	cis
1855	RTN4R	Q9BZR6	Reticulon-4 receptor	rs6658843	172399082	1	G	C	0.389	0.062	0.007	9.2E-18	trans
1855	RTN4R	Q9BZR6	Reticulon-4 receptor	rs1260326	27730940	2	T	C	0.392	0.058	0.007	1.6E-15	trans
1855	RTN4R	Q9BZR6	Reticulon-4 receptor	rs1047891	211540507	2	A	C	0.313	0.062	0.008	3.3E-16	trans
1855	RTN4R	Q9BZR6	Reticulon-4 receptor	rs6779146	135907213	3	T	C	0.769	0.058	0.008	4.0E-12	trans
1855	RTN4R	Q9BZR6	Reticulon-4 receptor	rs2126259	9185146	8	T	C	0.103	0.082	0.012	1.8E-12	trans
1855	RTN4R	Q9BZR6	Reticulon-4 receptor	rs28929474	94844947	14	T	C	0.021	0.311	0.025	4.0E-36	trans
1855	RTN4R	Q9BZR6	Reticulon-4 receptor	rs56332871	96714816	15	A	C	0.270	0.054	0.008	9.3E-12	trans
1855	RTN4R	Q9BZR6	Reticulon-4 receptor	rs1065853	45413233	19	T	G	0.081	0.116	0.013	3.6E-19	trans
1855	RTN4R	Q9BZR6	Reticulon-4 receptor	rs663353	20174712	22	G	A	0.252	0.298	0.009	2.7E-266	cis
1856	RUVBL1	Q9Y265	RuvB-like 1	rs1312	23040462	22	T	C	0.589	0.239	0.008	2.2E-182	trans
1857	RWDD1	Q9H446	RWD domain-containing protein 1	rs1354034	56849749	3	C	T	0.604	0.074	0.008	8.7E-23	trans
1857	RWDD1	Q9H446	RWD domain-containing protein 1	rs147899431	116892681	6	C	G	0.043	0.307	0.018	5.6E-63	cis
1858	S100A11	P31949	Protein S100-A11	rs2999553	152012534	1	C	G	0.042	0.326	0.018	3.1E-72	cis
1858	S100A11	P31949	Protein S100-A11	rs34436714	54327313	19	A	C	0.209	0.229	0.009	2.6E-141	trans
1859	S100A12	P80511	Protein S100-A12	rs3014874	153337943	1	G	A	0.735	0.237	0.008	4.5E-176	cis
1859	S100A12	P80511	Protein S100-A12	rs6984820	144993324	8	T	C	0.419	0.051	0.007	4.9E-12	trans
1859	S100A12	P80511	Protein S100-A12	rs10982156	117088064	9	A	T	0.069	0.137	0.015	8.2E-20	trans
1859	S100A12	P80511	Protein S100-A12	rs34436714	54327313	19	A	C	0.209	0.168	0.009	4.3E-77	trans
1860	S100A13	Q99584	Protein S100-A13	rs114678888	153595448	1	C	A	0.959	0.504	0.019	3.8E-158	cis
1860	S100A13	Q99584	Protein S100-A13	rs10405357	54759666	19	T	C	0.565	0.066	0.007	4.3E-19	trans
1861	S100A14	Q9HCY8	Protein S100-A14	rs11548102	153587256	1	A	T	0.511	0.175	0.007	6.2E-121	cis
1861	S100A14	Q9HCY8	Protein S100-A14	rs35853577	233558879	2	T	C	0.574	0.068	0.008	2.8E-19	trans
1861	S100A14	Q9HCY8	Protein S100-A14	rs2971446	154073445	3	G	C	0.159	0.070	0.010	8.6E-12	trans
1861	S100A14	Q9HCY8	Protein S100-A14	rs2291428	45958856	10	G	C	0.757	0.072	0.009	8.4E-17	trans
1861	S100A14	Q9HCY8	Protein S100-A14	rs56398830	103701690	13	G	A	0.989	0.608	0.035	8.2E-66	trans
1861	S100A14	Q9HCY8	Protein S100-A14	rs35193388	4420817	16	G	GC	0.264	0.065	0.009	1.6E-14	trans
1861	S100A14	Q9HCY8	Protein S100-A14	rs12938714	28645110	17	A	G	0.330	0.126	0.008	7.9E-57	trans
1861	S100A14	Q9HCY8	Protein S100-A14	rs2318997	55529230	18	T	G	0.566	0.062	0.008	1.6E-16	trans
1861	S100A14	Q9HCY8	Protein S100-A14	rs601338	49206674	19	G	A	0.491	0.109	0.007	4.9E-48	trans
1861	S100A14	Q9HCY8	Protein S100-A14	rs3746778	61341472	20	A	G	0.420	0.052	0.008	1.2E-11	trans
1862	S100A16	Q96FQ6	Protein S100-A16	rs9700928	153584986	1	A	C	0.514	0.104	0.008	2.5E-40	cis
1862	S100A16	Q96FQ6	Protein S100-A16	rs56398830	103701690	13	G	A	0.989	0.288	0.037	6.1E-15	trans
1862	S100A16	Q96FQ6	Protein S100-A16	rs601338	49206674	19	G	A	0.491	0.075	0.008	3.8E-22	trans
1863	S100A4	P26447	Protein S100-A4	rs56350425	153520520	1	C	T	0.051	0.361	0.017	3.4E-101	cis
1863	S100A4	P26447	Protein S100-A4	rs6993770	106581528	8	A	T	0.714	0.058	0.008	1.2E-12	trans
1863	S100A4	P26447	Protein S100-A4	rs150813342	135864513	9	C	T	0.991	0.351	0.042	3.3E-17	trans
1863	S100A4	P26447	Protein S100-A4	rs3817621	12998205	19	G	C	0.760	0.061	0.009	3.1E-12	trans
1864	S100G	P29377	Protein S100-G	rs784504	39195260	3	C	G	0.811	0.070	0.010	5.4E-13	trans
1864	S100G	P29377	Protein S100-G	rs59921059	16497738	X	C	T	0.721	0.152	0.007	3.7E-106	cis

1865	S100P	P25815	Protein S100-P	rs12613605	43358910	2	T	G	0.213	0.074	0.009	6.9E-16	trans
1865	S100P	P25815	Protein S100-P	rs7846314	61650831	8	T	A	0.185	0.080	0.010	5.9E-17	trans
1865	S100P	P25815	Protein S100-P	rs6503533	38184580	17	C	T	0.383	0.055	0.008	5.6E-13	trans
1865	S100P	P25815	Protein S100-P	rs7412	45412079	19	T	C	0.081	0.190	0.014	3.2E-43	trans
1865	S100P	P25815	Protein S100-P	rs4632248	54324995	19	T	G	0.209	0.069	0.009	7.8E-14	trans
1866	SAA4	P35542	Serum amyloid A-4 protein	rs7117890	18248503	11	G	A	0.476	0.364	0.008	0.0E+00	cis
1867	SAMD9L	Q8IVG5	Sterile alpha motif domain-containing protein 9-like	rs1354034	56849749	3	C	T	0.604	0.052	0.007	2.5E-12	trans
1867	SAMD9L	Q8IVG5	Sterile alpha motif domain-containing protein 9-like	rs17165120	92777091	7	A	C	0.931	0.567	0.015	0.0E+00	cis
1868	SARG	Q9BW04	Specifically androgen-regulated gene protein	rs74441312	207205404	1	G	T	0.019	0.263	0.028	1.1E-20	cis
1868	SARG	Q9BW04	Specifically androgen-regulated gene protein	rs2631367	131705458	5	G	C	0.519	0.061	0.007	3.3E-16	trans
1868	SARG	Q9BW04	Specifically androgen-regulated gene protein	rs139141690	101499930	7	G	A	0.995	0.416	0.055	2.8E-14	trans
1868	SARG	Q9BW04	Specifically androgen-regulated gene protein	rs10820606	99192919	9	A	C	0.771	0.095	0.009	2.8E-26	trans
1868	SARG	Q9BW04	Specifically androgen-regulated gene protein	rs57171122	77830707	18	C	T	0.478	0.074	0.007	5.5E-23	trans
1869	SAT2	Q96F10	Thialysine N-epsilon-acetyltransferase	rs13894	7529902	17	G	A	0.927	1.014	0.015	0.0E+00	cis
1870	SBSN	Q6UWP8	Suprabasin	rs61816761	152285861	1	A	G	0.023	0.224	0.025	7.9E-20	trans
1870	SBSN	Q6UWP8	Suprabasin	rs1260326	27730940	2	C	T	0.608	0.054	0.007	8.5E-14	trans
1870	SBSN	Q6UWP8	Suprabasin	rs141134546	49070468	4	T	C	0.020	0.184	0.025	2.6E-13	trans
1870	SBSN	Q6UWP8	Suprabasin	rs17082071	52780188	4	G	T	0.020	0.193	0.025	7.7E-15	trans
1871	SCAMP3	O14828	Secretory carrier-associated membrane protein 3	rs1142287	155230131	1	C	T	0.743	0.085	0.009	3.6E-23	cis
1871	SCAMP3	O14828	Secretory carrier-associated membrane protein 3	rs1354034	56849749	3	C	T	0.605	0.082	0.008	3.2E-27	trans
1871	SCAMP3	O14828	Secretory carrier-associated membrane protein 3	rs342293	106372219	7	C	G	0.541	0.058	0.007	9.5E-15	trans
1871	SCAMP3	O14828	Secretory carrier-associated membrane protein 3	rs6993770	106581528	8	A	T	0.714	0.061	0.008	1.7E-13	trans
1872	SCARA5	Q6ZMJ2	Scavenger receptor class A member 5	rs149340229	18008324	6	TA	T	0.037	0.133	0.018	1.3E-13	trans
1872	SCARA5	Q6ZMJ2	Scavenger receptor class A member 5	rs2726951	27805783	8	C	T	0.731	0.337	0.008	0.0E+00	cis
1872	SCARA5	Q6ZMJ2	Scavenger receptor class A member 5	rs8176719	136132908	9	TC	T	0.340	0.103	0.007	6.3E-47	trans
1872	SCARA5	Q6ZMJ2	Scavenger receptor class A member 5	rs4055121	126232337	11	C	T	0.865	0.082	0.010	1.5E-16	trans
1872	SCARA5	Q6ZMJ2	Scavenger receptor class A member 5	rs186021206	7069412	17	A	G	0.006	0.374	0.046	7.1E-16	trans
1873	SCARB1	Q8WTV0	Scavenger receptor class B member 1	rs113075502	106357446	14	A	G	0.226	0.070	0.010	1.3E-11	trans
1874	SCARB2	Q14108	Lysosome membrane protein 2	rs10935473	98416900	3	G	T	0.559	0.053	0.007	5.0E-14	trans
1874	SCARB2	Q14108	Lysosome membrane protein 2	rs28563976	77097373	4	A	C	0.854	0.315	0.010	2.2E-218	cis
1874	SCARB2	Q14108	Lysosome membrane protein 2	rs1042133	33048606	6	C	G	0.157	0.081	0.010	4.8E-17	trans
1874	SCARB2	Q14108	Lysosome membrane protein 2	rs13192569	123127597	6	G	A	0.861	0.070	0.010	5.1E-12	trans
1874	SCARB2	Q14108	Lysosome membrane protein 2	rs62018815	51058854	15	G	A	0.973	0.221	0.022	2.8E-24	trans
1875	SCARF1	Q14162	Scavenger receptor class F member 1	rs3811444	248039451	1	C	T	0.668	0.053	0.008	3.5E-12	trans
1875	SCARF1	Q14162	Scavenger receptor class F member 1	rs342298	106373646	7	C	T	0.545	0.053	0.007	3.1E-13	trans
1875	SCARF1	Q14162	Scavenger receptor class F member 1	rs6993770	106581528	8	A	T	0.713	0.123	0.008	2.1E-53	trans
1875	SCARF1	Q14162	Scavenger receptor class F member 1	rs579459	136154168	9	T	C	0.792	0.064	0.009	1.0E-12	trans
1875	SCARF1	Q14162	Scavenger receptor class F member 1	rs2272011	1542190	17	G	A	0.409	0.379	0.008	0.0E+00	cis
1875	SCARF1	Q14162	Scavenger receptor class F member 1	rs892090	55539072	19	G	T	0.834	0.086	0.010	1.5E-18	trans
1876	SCARF2	Q96GP6	Scavenger receptor class F member 2	rs61747728	179526214	1	T	C	0.038	0.125	0.017	5.9E-14	trans
1876	SCARF2	Q96GP6	Scavenger receptor class F member 2	rs1260326	27730940	2	C	T	0.608	0.044	0.007	1.5E-11	trans
1876	SCARF2	Q96GP6	Scavenger receptor class F member 2	rs507666	136149399	9	G	A	0.815	0.121	0.008	1.9E-48	trans
1876	SCARF2	Q96GP6	Scavenger receptor class F member 2	rs4055121	126232337	11	C	T	0.865	0.073	0.009	5.8E-15	trans
1876	SCARF2	Q96GP6	Scavenger receptor class F member 2	rs77924615	20392332	16	G	A	0.803	0.058	0.008	9.9E-13	trans
1876	SCARF2	Q96GP6	Scavenger receptor class F member 2	rs186021206	7069412	17	A	G	0.006	0.610	0.044	2.4E-44	trans
1876	SCARF2	Q96GP6	Scavenger receptor class F member 2	rs5763025	20786488	22	C	A	0.802	0.374	0.008	0.0E+00	cis
1877	SCG2	P13521	Secretogranin-2	rs1761879	117260230	6	G	A	0.678	0.066	0.008	1.8E-16	trans
1877	SCG2	P13521	Secretogranin-2	rs635634	136155000	9	C	T	0.816	0.069	0.010	1.0E-12	trans
1877	SCG2	P13521	Secretogranin-2	rs77924615	20392332	16	G	A	0.803	0.071	0.009	5.3E-14	trans
1877	SCG2	P13521	Secretogranin-2	rs681343	49206462	19	T	C	0.509	0.073	0.007	2.0E-22	trans
1878	SCG3	Q8WXD2	Secretogranin-3	rs6798507	71547039	3	A	G	0.645	0.052	0.007	2.5E-14	trans
1878	SCG3	Q8WXD2	Secretogranin-3	rs781657	57778685	4	G	A	0.184	0.108	0.008	1.4E-37	trans
1878	SCG3	Q8WXD2	Secretogranin-3	rs806798	26214473	6	C	T	0.468	0.052	0.007	1.6E-15	trans
1878	SCG3	Q8WXD2	Secretogranin-3	rs2606134	51973650	15	C	G	0.757	0.708	0.009	0.0E+00	cis
1878	SCG3	Q8WXD2	Secretogranin-3	rs186021206	7069412	17	A	G	0.006	0.441	0.045	8.2E-23	trans
1879	SCGB1A1	P11684	Uteroglobin	rs3856521	61405723	2	G	A	0.849	0.069	0.010	1.7E-12	trans
1879	SCGB1A1	P11684	Uteroglobin	rs1515498	189508302	3	A	G	0.638	0.106	0.007	2.1E-47	trans
1879	SCGB1A1	P11684	Uteroglobin	rs10051184	126372809	5	T	C	0.503	0.054	0.007	1.0E-14	trans
1879	SCGB1A1	P11684	Uteroglobin	rs3210176	32627850	6	T	C	0.628	0.057	0.007	1.2E-14	trans
1879	SCGB1A1	P11684	Uteroglobin	rs7040029	117619214	9	T	C	0.292	0.054	0.008	3.9E-12	trans
1879	SCGB1A1	P11684	Uteroglobin	rs7072547	111982398	10	G	A	0.821	0.070	0.009	3.0E-14	trans
1879	SCGB1A1	P11684	Uteroglobin	rs10501135	34811180	11	T	C	0.586	0.142	0.007	3.4E-83	trans
1879	SCGB1A1	P11684	Uteroglobin	rs3741240	62186542	11	G	A	0.649	0.339	0.008	0.0E+00	cis
1879	SCGB1A1	P11684	Uteroglobin	rs1766147	36708179	14	C	T	0.702	0.060	0.008	1.3E-14	trans
1879	SCGB1A1	P11684	Uteroglobin	rs7182041	50306583	15	C	A	0.440	0.083	0.007	3.2E-31	trans
1879	SCGB1A1	P11684	Uteroglobin	rs1997814	10646671	20	T	G	0.318	0.059	0.008	1.9E-14	trans
1880	SCGB3A1	Q96QR1	Secretoglobin family 3A member 1	rs4971100	155155731	1	A	G	0.426	0.090	0.007	1.0E-34	trans
1880	SCGB3A1	Q96QR1	Secretoglobin family 3A member 1	rs58235267	63277843	2	C	G	0.511	0.067	0.007	4.6E-20	trans
1880	SCGB3A1	Q96QR1	Secretoglobin family 3A member 1	rs62279940	189506188	3	G	A	0.735	0.057	0.008	6.3E-12	trans
1880	SCGB3A1	Q96QR1	Secretoglobin family 3A member 1	rs2432162	102118633	5	T	C	0.632	0.171	0.008	1.9E-111	trans
1880	SCGB3A1	Q96QR1	Secretoglobin family 3A member 1	rs307802	180019237	5	C	T	0.406	0.118	0.007	3.6E-56	cis
1880	SCGB3A1	Q96QR1	Secretoglobin family 3A member 1	rs35795116	31298123	6	G	A	0.874	0.086	0.012	5.0E-12	trans
1880	SCGB3A1	Q96QR1	Secretoglobin family 3A member 1	rs141020575	97754097	7	T	TA	0.461	0.082	0.007	2.6E-29	trans
1880	SCGB3A1	Q96QR1	Secretoglobin family 3A member 1	rs502857	34854662	11	T	C	0.291	0.203	0.008	1.6E-137	trans
1880	SCGB3A1	Q96QR1	Secretoglobin family 3A member 1	rs3741240	62186542	11	A	G	0.351	0.165	0.008	1.8E-100	trans
1880	SCGB3A1	Q96QR1	Secretoglobin family 3A member 1	rs1927191	73768029	13	A	G	0.428	0.056	0.007	2.7E-14	trans
1880	SCGB3A1	Q96QR1	Secretoglobin family 3A member 1	rs56156922	56987369	16	C	T	0.325	0.087	0.008	8.1E-29	trans
1880	SCGB3A1	Q96QR1	Secretoglobin family 3A member 1	rs371181880	20115552	18	A	G	0.949	0.118	0.017	9.2E-13	trans
1880	SCGB3A1	Q96QR1	Secretoglobin family 3A member 1	rs139063750	46312068	19	T	C	0.098	0.104	0.012	2.1E-17	trans
1880	SCGB3A1	Q96QR1	Secretoglobin family 3A member 1	rs4240082	150017550	X	T	C	0.591	0.141	0.006	1.8E-117	trans
1881	SCGB3A2	Q96PL1	Secretoglobin family 3A member 2	rs72691756	118117179	1	A	G	0.124	0.184	0.011	3.9E-60	trans
1881	SCGB3A2	Q96PL1	Secretoglobin family 3A member 2	rs34153848	10072750	2	A	G	0.365	0.057	0.008	8.2E-14	trans
1881	SCGB3A2	Q96PL1	Secretoglobin family 3A member 2	rs58235267	63277843	2	C	G	0.512	0.074	0.007	1.7E-23	trans
1881	SCGB3A2	Q96PL1	Secretoglobin family 3A member 2	rs1515498	189508302	3	A	G	0.637	0.132	0.008	5.0E-67	trans
1881	SCGB3A2	Q96PL1	Secretoglobin family 3A member 2	rs17717320	147205914	5	C	G	0.802	0.248	0.009	2.5E-154	cis
1881	SCGB3A2	Q96PL1	Secretoglobin family 3A member 2	rs6905736	19843767	6	C	A	0.151	0.086	0.010	1.3E-16	trans
1881	SCGB3A2	Q96PL1	Secretoglobin family 3A member 2	rs6927418	24827912	6	C	T	0.443	0.052	0.007	2.9E-12	trans
1881	SCGB3A2	Q96PL1	Secretoglobin family 3A member 2	rs62405419	50787459	6	G	T	0.883	0.077	0.011	1.7E-11	trans
1881	SCGB3A2	Q96PL1	Secretoglobin family 3A member 2	rs12666406	97790991	7	G	A	0.465	0.063	0.007	5.7E-18	trans
1881	SCGB3A2	Q96PL1	Secretoglobin family 3A member 2	rs4881548	1025666	10	C	T	0.500	0.058	0.007	2.4E-15	trans
1881	SCGB3A2	Q96PL1	Secretoglobin family 3A member 2	rs7942701	34798883	11	T	C	0.415	0.064	0.007	6.9E-18	trans
1881	SCGB3A2	Q96PL1	Secretoglobin family 3A member 2	rs3741240	62186542	11	A	G	0.351	0.138	0.008	5.7E-72	trans
1881	SCGB3A2	Q96PL1	Secretoglobin family 3A member 2	rs2414000	50312468	15	G	T	0.432	0.059	0.007	1.7E-15	trans
1881	SCGB3A2	Q96PL1	Secretoglobin family 3A member 2	rs173539	56988044	16	T	C	0.332	0.067	0.008	4.6E-18	trans
1881	SCGB3A2	Q96PL1	Secretoglobin family 3A member 2	rs756719	72967363	16	C	T	0.725	0.058	0.008	2.6E-12	trans
1881	SCGB3A2	Q96PL1	Secretoglobin family 3A member 2	rs12167717	19826640	22	G	A	0.761	0.071	0.009	8.7E-17	trans
1882	SCGN	O76038	Secretagogin	rs6859236	150535644	5	G	A	0.485	0.104	0.008	1.4E-43	trans
1882	SCGN	O76038	Secretagogin	rs10946788	25660510	6	G	A	0.725	0.092	0.008	9.6E-28	cis
1882	SCGN	O76038	Secretagogin	rs9297994	59392324	8	A	G	0.666	0.059	0.008	2.0E-13	trans
1882	SCGN	O76038	Secretagogin	rs11043076	2223474	11	T	C	0.134	0.090	0.011	7.1E-16	trans
1882	SCGN	O76038	Secretagogin	rs601338	49206674	19	G	A	0.491	0.136	0.008	8.5E-72	trans
1883	SCLY	Q96I15	Selenocysteine lyase	rs62196005	239007130	2	T	A	0.275	0.194	0.008	1.3E-127	cis
1883	SCLY	Q96I15	Selenocysteine lyase	rs4835265	146821410	4	A	C	0.156	0.073	0.010	1.1E-13	trans
1883	SCLY	Q96I15	Selenocysteine lyase	rs10733608	117148430	9	G	T	0.512	0.051	0.007	2.7E-12	trans
1883	SCLY	Q96I15	Selenocysteine lyase	rs10883451	101924418	10	T	C	0.498	0.052	0.007	4.5E-13	trans
1883	SCLY	Q96I15	Selenocysteine lyase	rs58964858	93816083	11	C	T	0.893	0.086	0.012	2.0E-13	trans
1883	SCLY	Q96I15	Selenocysteine lyase	rs112635299	94838142	14	T	G	0.022	0.171	0.025	4.4E-12	trans
1883	SCLY	Q96I15	Selenocysteine lyase	rs3747207	44324855	22	A	G	0.215	0.074	0.009	2.6E-17	trans
1884	SCN4B	Q8IWT1	Sodium channel subunit beta-4	rs1260326	27730940	2	C	T	0.608	0.050	0.007	1.0E-14	trans
1884	SCN4B	Q8IWT1	Sodium channel subunit beta-4	rs9273757	32629417	6	C	G	0.233	0.053	0.008	1.0E-11	trans
1885	SCPEP1	Q9HB40	Retinoid-inducible serine carboxypeptidase	rs6993770	106581528	8	A	T	0.714	0.085	0.008	2.9E-25	trans
1885	SCPEP1	Q9HB40	Retinoid-inducible serine carboxypeptidase	rs1031101	54533360	10	G	A	0.142	0.081	0.011	2.6E-14	trans
1885	SCPEP1	Q9HB40	Retinoid-inducible serine carboxypeptidase	rs10761756	65172328	10	T	C	0.470	0.053	0.007	4.7E-13	trans
1885	SCPEP1	Q9HB40	Retinoid-inducible serine carboxypeptidase	rs34449028	55085363	17	A	T	0.699	0.365	0.009	0.0E+00	cis
1885	SCPEP1	Q9HB40	Retinoid-inducible serine carboxypeptidase	rs2071225	100662901	X	G	A	0.939	0.129	0.013	8.4E-24	trans
1886	SCRIB	Q14160	Protein scribble homolog	rs2621380	32761353	6	C	T	0.446	0.053	0.008	4.1E-12	trans
1887	SCRN1	Q12765	Secernin-1	rs1434282	199010721	1	T	C	0.726	0.095	0.008	5.1E-29	trans
1887	SCRN1	Q12765	Secernin-1	rs17324153	29966141	7	C	T	0.043	0.267	0.019	7.4E-47	cis
1888	SCT	P09683	Secretin	rs13264644	143753546	8	G	A	0.432	0.058	0.008	7.7E-14	trans
1889	SDC1	P18827	Syndecan-1	rs907866	20371380	2	A	G	0.447	0.131	0.007	8.4E-71	cis
1889	SDC1	P18827	Syndecan-1	rs1260326	27730940	2	C	T	0.608	0.073	0.007	8.2E-23	trans
1889	SDC1	P18827	Syndecan-1	rs13107325	103188709	4	T	C	0.075	0.335	0.014	1.4E-127	trans
1889	SDC1	P18827	Syndecan-1	rs28522260	1183964	7	C	A	0.832	0.072	0.010	1.3E-13	trans
1889	SDC1	P18827	Syndecan-1	rs9987289	9183358	8	A	G	0.092	0.110	0.013	1.3E-18	trans
1889	SDC1	P18827	Syndecan-1	rs532436	136149830	9	G	A	0.815	0.105	0.009	9.1E-29	trans
1889	SDC1	P18827	Syndecan-1	rs11438680	65174698	10	GA	G	0.461	0.061	0.007	6.2E-17	trans
1889	SDC1	P18827	Syndecan-1	rs10881600	82256258	10	C	T	0.553	0.049	0.007	1.4E-11	trans
1889	SDC1	P18827	Syndecan-1	rs186021206	7069412	17	A	G	0.006	0.668	0.050	5.1E-41	trans
1889	SDC1	P18827	Syndecan-1	rs2204211	47273967	17	A	G	0.102	0.088	0.012	2.6E-13	trans
1889	SDC1	P18827	Syndecan-1	rs620982	29803271	18	T	C	0.544	0.075	0.007	5.9E-25	trans
1889	SDC1	P18827	Syndecan-1	rs601338	49206674	19	A	G	0.509	0.076	0.007	2.2E-25	trans
1889	SDC1	P18827	Syndecan-1	rs2294915	44340904	22	T	C	0.232	0.058	0.009	1.2E-11	trans
1890	SDC4	P31431	Syndecan-4	rs6662263	171944242	1	G	A	0.804	0.072	0.009	9.9E-15	trans
1890	SDC4	P31431	Syndecan-4	rs1354034	56849749	3	T	C	0.396	0.083	0.007	9.9E-29	trans
1890	SDC4	P31431	Syndecan-4	rs61177759	139847293	6	C	T	0.799	0.062	0.009	1.7E-11	trans
1890	SDC4	P31431	Syndecan-4	rs11763765	1060510	7	C	T	0.852	0.173	0.010	1.2E-62	trans
1890	SDC4	P31431	Syndecan-4	rs139141690	101499930	7	G	A	0.995	0.448	0.054	9.2E-17	trans
1890	SDC4	P31431	Syndecan-4	rs6993770	106581528	8	A	T	0.713	0.121	0.008	1.1E-50	trans
1890	SDC4	P31431	Syndecan-4	rs385893	4763176	9	C	T	0.524	0.087	0.007	2.1E-32	trans
1890	SDC4	P31431	Syndecan-4	rs61750929	91495135	9	C	T	0.945	0.112	0.016	4.0E-12	trans
1890	SDC4	P31431	Syndecan-4	rs8193003	135865496	9	T	C	0.393	0.056	0.008	1.9E-13	trans
1890	SDC4	P31431	Syndecan-4	rs58641111	82244956	10	C	CGT	0.534	0.083	0.007	5.7E-29	trans
1890	SDC4	P31431	Syndecan-4	rs12762934	104359884	10	T	C	0.325	0.102	0.008	1.0E-38	trans

1890	SDC4	P31431	Syndecan-4	rs33972805	126288872	11	C	CCT	0.473	0.055	0.007	1.2E-13	trans
1890	SDC4	P31431	Syndecan-4	rs12881545	101176212	14	C	G	0.668	0.057	0.008	2.4E-13	trans
1890	SDC4	P31431	Syndecan-4	rs34890846	9041493	16	A	ATTG	0.811	0.076	0.009	4.7E-16	trans
1890	SDC4	P31431	Syndecan-4	rs4783186	85415734	16	T	C	0.126	0.076	0.011	3.6E-12	trans
1890	SDC4	P31431	Syndecan-4	rs1654425	55538980	19	C	T	0.834	0.076	0.010	1.7E-14	trans
1890	SDC4	P31431	Syndecan-4	rs4411786	1930897	20	T	C	0.737	0.064	0.008	3.4E-14	trans
1890	SDC4	P31431	Syndecan-4	rs2251577	43974571	20	G	C	0.487	0.223	0.007	8.2E-198	cis
1890	SDC4	P31431	Syndecan-4	rs2229742	16339172	21	C	G	0.103	0.087	0.012	3.8E-13	trans
1891	SDCCAG8	Q86SQ7	Serologically defined colon cancer antigen 8	rs953492	243471192	1	G	A	0.534	0.203	0.007	6.0E-165	cis
1891	SDCCAG8	Q86SQ7	Serologically defined colon cancer antigen 8	rs1354034	56849749	3	C	T	0.604	0.086	0.007	1.3E-30	trans
1892	SDHB	P21912	Succinate dehydrogenase	rs4361977	155076079	1	C	T	0.566	0.055	0.008	7.0E-13	trans
1892	SDHB	P21912	Succinate dehydrogenase	rs4835265	146821410	4	A	C	0.156	0.140	0.010	1.3E-41	trans
1892	SDHB	P21912	Succinate dehydrogenase	rs10075805	31021358	5	A	G	0.724	0.060	0.008	1.1E-12	trans
1892	SDHB	P21912	Succinate dehydrogenase	rs4581712	80497601	16	A	C	0.272	0.058	0.008	6.1E-12	trans
1892	SDHB	P21912	Succinate dehydrogenase	rs4245267	56096214	18	C	A	0.211	0.096	0.009	1.7E-25	trans
1893	SDK2	Q58EX2	Protein sidekick-2	rs116522341	32367697	6	G	C	0.033	0.209	0.021	5.3E-24	trans
1893	SDK2	Q58EX2	Protein sidekick-2	rs186021206	7069412	17	A	G	0.006	0.574	0.052	8.7E-29	trans
1893	SDK2	Q58EX2	Protein sidekick-2	rs11655934	71587577	17	G	A	0.851	0.674	0.011	0.0E+00	cis
1894	SEC31A	O94979	Protein transport protein Sec31A	rs1354034	56849749	3	C	T	0.605	0.083	0.008	4.6E-27	trans
1894	SEC31A	O94979	Protein transport protein Sec31A	rs11541628	83765605	4	C	A	0.975	0.310	0.024	2.5E-37	cis
1894	SEC31A	O94979	Protein transport protein Sec31A	rs342293	106372219	7	C	G	0.540	0.058	0.008	2.4E-14	trans
1894	SEC31A	O94979	Protein transport protein Sec31A	rs6993770	106581528	8	A	T	0.713	0.063	0.008	6.2E-14	trans
1895	SEL1L	Q9UBV2	Protein sel-1 homolog 1	rs11499034	81972441	14	T	C	0.985	1.255	0.032	0.0E+00	cis
1895	SEL1L	Q9UBV2	Protein sel-1 homolog 1	rs28929474	94844947	14	T	C	0.021	0.557	0.027	2.2E-97	trans
1896	SELE	P16581	E-selectin	rs599839	109822166	1	A	G	0.772	0.070	0.007	5.0E-21	trans
1896	SELE	P16581	E-selectin	rs7538317	169707576	1	C	G	0.344	0.095	0.007	6.0E-47	cis
1896	SELE	P16581	E-selectin	rs10935473	98416900	3	G	T	0.560	0.088	0.006	4.5E-45	trans
1896	SELE	P16581	E-selectin	rs13135092	103198082	4	G	A	0.083	0.080	0.011	1.2E-12	trans
1896	SELE	P16581	E-selectin	rs2519093	136141870	9	C	T	0.816	0.978	0.010	0.0E+00	trans
1896	SELE	P16581	E-selectin	rs35166255	126301756	11	A	G	0.034	0.324	0.017	1.5E-78	trans
1896	SELE	P16581	E-selectin	rs186021206	7069412	17	A	G	0.006	0.587	0.043	7.6E-43	trans
1896	SELE	P16581	E-selectin	rs17855739	5831840	19	T	C	0.041	0.179	0.016	2.3E-30	trans
1897	SELENOP	P49908	Selenoprotein P	rs10038285	42689540	5	A	G	0.733	0.103	0.009	7.0E-34	cis
1898	SELL	P14151	L-selectin	rs2223286	169665632	1	T	C	0.689	0.407	0.008	0.0E+00	cis
1898	SELL	P14151	L-selectin	rs139015452	111807596	2	CACTG	C	0.229	0.058	0.008	2.3E-12	trans
1898	SELL	P14151	L-selectin	rs9824474	169745527	3	A	G	0.633	0.051	0.007	1.0E-12	trans
1898	SELL	P14151	L-selectin	rs218265	55408999	4	C	T	0.153	0.073	0.010	3.0E-14	trans
1898	SELL	P14151	L-selectin	rs6796	6502367	7	C	T	0.279	0.077	0.008	9.0E-24	trans
1898	SELL	P14151	L-selectin	rs1794089	126196807	11	A	G	0.234	0.084	0.008	2.9E-24	trans
1898	SELL	P14151	L-selectin	rs76428106	28604007	13	C	T	0.013	0.326	0.032	5.0E-25	trans
1898	SELL	P14151	L-selectin	rs186021206	7069412	17	A	G	0.006	0.726	0.047	1.5E-53	trans
1898	SELL	P14151	L-selectin	rs12941811	38159335	17	T	C	0.426	0.069	0.007	1.6E-23	trans
1898	SELL	P14151	L-selectin	rs7224668	79235788	17	T	C	0.443	0.049	0.007	2.3E-12	trans
1899	SELP	P16109	P-selectin	rs6136	169563951	1	T	G	0.886	0.403	0.012	1.1E-257	cis
1899	SELP	P16109	P-selectin	rs56043070	247719769	1	A	G	0.073	0.114	0.014	2.1E-15	trans
1899	SELP	P16109	P-selectin	rs11242109	131677047	5	G	T	0.519	0.062	0.007	3.7E-17	trans
1899	SELP	P16109	P-selectin	rs6961069	80218961	7	T	C	0.402	0.056	0.007	1.1E-13	trans
1899	SELP	P16109	P-selectin	rs6993770	106581528	8	A	T	0.714	0.116	0.008	3.4E-46	trans
1899	SELP	P16109	P-selectin	rs2519093	136141870	9	C	T	0.816	0.219	0.010	2.2E-116	trans
1899	SELP	P16109	P-selectin	rs7896518	65104500	10	G	A	0.424	0.074	0.007	3.4E-23	trans
1899	SELP	P16109	P-selectin	rs12445050	81870969	16	T	C	0.138	0.099	0.011	7.8E-21	trans
1899	SELP	P16109	P-selectin	rs892090	55539072	19	G	T	0.834	0.136	0.010	5.0E-43	trans
1900	SELPLG	Q14242	P-selectin glycoprotein ligand 1	rs343808	111330007	1	C	T	0.758	0.074	0.008	6.0E-19	trans
1900	SELPLG	Q14242	P-selectin glycoprotein ligand 1	rs10214273	35883986	5	T	G	0.727	0.068	0.008	7.7E-18	trans
1900	SELPLG	Q14242	P-selectin glycoprotein ligand 1	rs4248814	31346755	6	A	G	0.202	0.071	0.009	6.3E-16	trans
1900	SELPLG	Q14242	P-selectin glycoprotein ligand 1	rs10093797	79572713	8	C	A	0.248	0.058	0.008	1.3E-12	trans
1900	SELPLG	Q14242	P-selectin glycoprotein ligand 1	rs2142306	134470631	8	C	T	0.412	0.049	0.007	1.3E-11	trans
1900	SELPLG	Q14242	P-selectin glycoprotein ligand 1	rs536644	79145788	9	A	G	0.546	0.099	0.007	1.7E-43	trans
1900	SELPLG	Q14242	P-selectin glycoprotein ligand 1	rs10819317	130668957	9	G	A	0.166	0.096	0.010	7.5E-24	trans
1900	SELPLG	Q14242	P-selectin glycoprotein ligand 1	rs60005225	38168128	17	G	GT	0.618	0.052	0.007	1.4E-12	trans
1901	SEMA3F	Q13275	Semaphorin-3F	rs4679317	126688271	3	G	A	0.314	0.075	0.008	3.6E-21	trans
1901	SEMA3F	Q13275	Semaphorin-3F	rs564699	89200873	11	C	T	0.464	0.065	0.007	8.1E-19	trans
1901	SEMA3F	Q13275	Semaphorin-3F	rs1037117	102068658	15	A	G	0.252	0.065	0.008	2.1E-14	trans
1901	SEMA3F	Q13275	Semaphorin-3F	rs8055162	89715282	16	G	A	0.433	0.067	0.007	2.1E-19	trans
1902	SEMA3G	Q9NS98	Semaphorin-3G	rs12740374	109817590	1	G	T	0.779	0.302	0.008	9.7E-287	trans
1902	SEMA3G	Q9NS98	Semaphorin-3G	rs2766588	35753227	6	C	T	0.607	0.458	0.008	0.0E+00	trans
1902	SEMA3G	Q9NS98	Semaphorin-3G	rs4947530	51026911	7	T	C	0.954	0.111	0.016	1.5E-11	trans
1902	SEMA3G	Q9NS98	Semaphorin-3G	rs2653414	17726069	8	A	C	0.013	0.305	0.030	9.9E-24	trans
1902	SEMA3G	Q9NS98	Semaphorin-3G	rs117119759	136212168	9	A	G	0.029	0.142	0.021	6.1E-12	trans
1902	SEMA3G	Q9NS98	Semaphorin-3G	rs4747199	73569318	10	C	T	0.712	0.092	0.008	5.0E-34	trans
1902	SEMA3G	Q9NS98	Semaphorin-3G	rs72802342	75234872	16	C	A	0.924	0.215	0.013	3.9E-60	trans
1902	SEMA3G	Q9NS98	Semaphorin-3G	rs5848	42430244	17	C	T	0.725	0.101	0.008	1.4E-38	trans
1903	SEMA4C	Q9C0C4	Semaphorin-4C	rs34794906	31237858	6	C	T	0.264	0.062	0.008	1.4E-13	trans
1903	SEMA4C	Q9C0C4	Semaphorin-4C	rs564699	89200873	11	C	T	0.464	0.056	0.007	2.9E-14	trans
1903	SEMA4C	Q9C0C4	Semaphorin-4C	rs186021206	7069412	17	A	G	0.006	0.505	0.051	4.8E-23	trans
1903	SEMA4C	Q9C0C4	Semaphorin-4C	rs6010224	50714491	22	C	A	0.443	0.050	0.007	1.2E-11	trans
1904	SEMA4D	Q92854	Semaphorin-4D	rs1354034	56849749	3	C	T	0.604	0.101	0.008	7.0E-40	trans
1904	SEMA4D	Q92854	Semaphorin-4D	rs6993770	106581528	8	A	T	0.714	0.056	0.008	1.5E-11	trans
1904	SEMA4D	Q92854	Semaphorin-4D	rs36020742	92051455	9	T	C	0.183	0.107	0.010	5.1E-28	cis
1904	SEMA4D	Q92854	Semaphorin-4D	rs7080386	65048306	10	A	C	0.412	0.054	0.008	2.2E-12	trans
1904	SEMA4D	Q92854	Semaphorin-4D	rs7949566	126285301	11	A	G	0.422	0.067	0.008	1.9E-18	trans
1905	SEMA7A	O75326	Semaphorin-7A	rs7606173	60725451	2	G	C	0.568	0.063	0.007	6.1E-18	trans
1905	SEMA7A	O75326	Semaphorin-7A	rs2009581	111807677	2	G	A	0.727	0.057	0.008	3.9E-12	trans
1905	SEMA7A	O75326	Semaphorin-7A	rs3864106	186656602	3	G	C	0.416	0.187	0.007	3.1E-139	trans
1905	SEMA7A	O75326	Semaphorin-7A	rs28716466	201074	4	C	T	0.297	0.070	0.008	2.8E-18	trans
1905	SEMA7A	O75326	Semaphorin-7A	rs13135092	103198082	4	G	A	0.083	0.094	0.013	1.3E-12	trans
1905	SEMA7A	O75326	Semaphorin-7A	rs9263850	31156188	6	G	A	0.280	0.060	0.008	1.6E-13	trans
1905	SEMA7A	O75326	Semaphorin-7A	rs58217236	119612485	6	T	C	0.447	0.059	0.007	1.4E-15	trans
1905	SEMA7A	O75326	Semaphorin-7A	rs2347784	6524843	7	C	G	0.729	0.083	0.008	1.8E-24	trans
1905	SEMA7A	O75326	Semaphorin-7A	rs12554596	37002142	9	A	G	0.796	0.067	0.009	9.8E-14	trans
1905	SEMA7A	O75326	Semaphorin-7A	rs10793962	136129115	9	T	A	0.061	0.225	0.015	5.6E-50	trans
1905	SEMA7A	O75326	Semaphorin-7A	rs56278466	17875857	10	G	T	0.661	0.162	0.008	3.6E-98	trans
1905	SEMA7A	O75326	Semaphorin-7A	rs78994380	74692412	15	C	A	0.888	0.231	0.012	3.9E-84	cis
1905	SEMA7A	O75326	Semaphorin-7A	rs200489612	7106378	17	A	G	0.005	0.705	0.055	4.2E-38	trans
1905	SEMA7A	O75326	Semaphorin-7A	rs34557412	16852187	17	G	A	0.007	0.554	0.044	3.6E-36	trans
1905	SEMA7A	O75326	Semaphorin-7A	rs485186	49207206	19	G	A	0.537	0.050	0.007	8.5E-12	trans
1906	SEPTIN3	Q9UH03	Neuronal-specific septin-3	rs115126145	230286220	1	T	C	0.022	0.181	0.025	6.4E-13	trans
1906	SEPTIN3	Q9UH03	Neuronal-specific septin-3	rs3777182	95263752	5	A	T	0.267	0.098	0.008	8.3E-32	trans
1906	SEPTIN3	Q9UH03	Neuronal-specific septin-3	rs686250	32585055	6	A	G	0.432	0.084	0.008	3.1E-28	trans
1906	SEPTIN3	Q9UH03	Neuronal-specific septin-3	rs75430132	137784552	9	C	T	0.838	0.069	0.010	8.6E-12	trans
1906	SEPTIN3	Q9UH03	Neuronal-specific septin-3	rs61896141	61556039	11	C	A	0.191	0.067	0.009	1.5E-12	trans
1906	SEPTIN3	Q9UH03	Neuronal-specific septin-3	rs550897	114442103	11	G	A	0.462	0.233	0.007	2.3E-215	trans
1906	SEPTIN3	Q9UH03	Neuronal-specific septin-3	rs117775520	106174221	14	G	A	0.964	0.196	0.020	1.1E-23	trans
1906	SEPTIN3	Q9UH03	Neuronal-specific septin-3	rs34562254	16842991	17	A	G	0.097	0.120	0.012	2.2E-22	trans
1906	SEPTIN3	Q9UH03	Neuronal-specific septin-3	rs9624334	24166256	22	C	G	0.151	0.090	0.010	3.6E-18	trans
1907	SEPTIN8	Q92599	Septin-8	rs11204523	248020556	1	G	C	0.242	0.070	0.009	3.4E-16	trans
1907	SEPTIN8	Q92599	Septin-8	rs7665521	77908323	4	T	C	0.423	0.071	0.007	6.5E-22	trans
1907	SEPTIN8	Q92599	Septin-8	rs30512	132119157	5	C	T	0.888	0.145	0.011	7.0E-37	cis
1908	SEPTIN9	Q9UHD8	Septin-9	rs55645755	75452693	17	A	G	0.985	0.355	0.031	1.2E-30	cis
1908	SEPTIN9	Q9UHD8	Septin-9	rs10432210	22861794	18	T	C	0.209	0.065	0.009	2.4E-12	trans
1909	SERPINA1	P01009	Alpha-1-antitrypsin	rs17580	94847262	14	T	A	0.950	0.374	0.018	3.7E-100	cis
1910	SERPINA11	Q86U17	Serpin A11	rs1260326	27730940	2	T	C	0.392	0.066	0.006	1.8E-29	trans
1910	SERPINA11	Q86U17	Serpin A11	rs4634107	186341393	3	T	G	0.523	0.056	0.006	3.6E-23	trans
1910	SERPINA11	Q86U17	Serpin A11	rs13108218	3443931	4	A	G	0.381	0.044	0.006	1.6E-13	trans
1910	SERPINA11	Q86U17	Serpin A11	rs144861591	26072992	6	C	T	0.923	0.076	0.011	1.9E-12	trans
1910	SERPINA11	Q86U17	Serpin A11	rs10740131	65271488	10	T	A	0.470	0.057	0.006	3.9E-23	trans
1910	SERPINA11	Q86U17	Serpin A11	rs12898137	94910582	14	T	A	0.589	0.621	0.007	0.0E+00	cis
1910	SERPINA11	Q86U17	Serpin A11	rs7188155	88526422	16	G	C	0.310	0.043	0.006	3.8E-12	trans
1910	SERPINA11	Q86U17	Serpin A11	rs12976652	46396610	19	T	C	0.881	0.097	0.009	1.5E-28	trans
1911	SERPINA12	Q8IW75	Serpin A12	rs1495743	18273300	8	G	C	0.222	0.051	0.007	7.5E-12	trans
1911	SERPINA12	Q8IW75	Serpin A12	rs174533	61549025	11	G	A	0.654	0.046	0.006	1.3E-12	trans
1911	SERPINA12	Q8IW75	Serpin A12	rs17091005	94988407	14	C	T	0.218	0.778	0.009	0.0E+00	cis
1911	SERPINA12	Q8IW75	Serpin A12	rs339969	60883281	15	C	A	0.383	0.095	0.006	4.4E-51	trans
1911	SERPINA12	Q8IW75	Serpin A12	rs738408	44324730	22	T	C	0.216	0.055	0.007	1.2E-13	trans
1912	SERPINA3	P01011	Alpha-1-antichymotrypsin	rs11208702	66135917	1	A	C	0.805	0.073	0.009	5.6E-15	trans
1912	SERPINA3	P01011	Alpha-1-antichymotrypsin	rs28601761	126500031	8	G	C	0.419	0.053	0.007	9.2E-13	trans
1912	SERPINA3	P01011	Alpha-1-antichymotrypsin	rs61976127	95097666	14	A	G	0.815	0.447	0.010	0.0E+00	cis
1913	SERPINA4	P29622	Kallistatin	rs1260326	27730940	2	T	C	0.392	0.076	0.007	7.6E-28	trans
1913	SERPINA4	P29622	Kallistatin	rs1230167	99987328	4	T	A	0.672	0.065	0.007	6.5E-19	trans
1913	SERPINA4	P29622	Kallistatin	rs1051921	73007943	7	G	A	0.804	0.067	0.009	8.1E-15	trans
1913	SERPINA4	P29622	Kallistatin	rs5511	95033595	14	T	A	0.220	0.617	0.009	0.0E+00	cis
1914	SERPINA5	P05154	Plasma serine protease inhibitor	rs1260326	27730940	2	T	C	0.392	0.092	0.008	1.3E-34	trans
1914	SERPINA5	P05154	Plasma serine protease inhibitor	rs710446	186459927	3	C	T	0.409	0.060	0.007	1.1E-15	trans
1914	SERPINA5	P05154	Plasma serine protease inhibitor	rs3733402	187158034	4	A	G	0.512	0.180	0.007	5.3E-132	trans
1914	SERPINA5	P05154	Plasma serine protease inhibitor	rs1801020	176836532	5	G	A	0.745	0.193	0.008	6.8E-116	trans
1914	SERPINA5	P05154	Plasma serine protease inhibitor	rs1130267	95063107	14	A	G	0.599	0.201	0.008	3.3E-153	cis
1914	SERPINA5	P05154	Plasma serine protease inhibitor	rs7212366	74593181	17	T	C	0.492	0.054	0.008	7.0E-12	trans
1915	SERPINA6	P08185	Corticosteroid-binding globulin	rs5785581	65318502	10	CA	C	0.476	0.057	0.007	2.6E-17	trans
1915	SERPINA6	P08185	Corticosteroid-binding globulin	rs139097404	43933941	15	C	T	0.023	0.177	0.023	8.7E-15	trans
1916	SERPINA7	P05543	Thyroxine-binding globulin	rs60935030	136049625	3	T	TAATTA	0.252	0.060	0.009	8.1E-12	trans
1916	SERPINA7	P05543	Thyroxine-binding globulin	rs10095930	116974302	8	T	C	0.584	0.060	0.008	4.0E-15	trans
1916	SERPINA7	P05543	Thyroxine-binding globulin	rs1804495	105278361	X	C	A	0.881	0.522	0.010	0.0E+00	cis
1917	SERPINA9	Q86WD7	Serpin A9	rs2038499	94949291	14	C	T	0.718	0.498	0.008	0.0E+00	cis

1918	SERPINB1	P30740	Leukocyte elastase inhibitor	rs6925835	2815588	6	T	C	0.176	0.102	0.010	3.0E-25	cis
1918	SERPINB1	P30740	Leukocyte elastase inhibitor	rs6993770	106581528	8	A	T	0.714	0.082	0.008	2.5E-23	trans
1919	SERPINB6	P35237	Serpin B6	rs9405601	2972897	6	A	G	0.438	0.161	0.008	7.5E-101	cis
1919	SERPINB6	P35237	Serpin B6	rs6993770	106581528	8	A	T	0.713	0.064	0.008	7.4E-15	trans
1920	SERPINB8	P50452	Serpin B8	rs3826616	61654463	18	A	G	0.419	0.727	0.008	0.0E+00	cis
1920	SERPINB8	P50452	Serpin B8	rs4632248	54324995	19	T	G	0.210	0.099	0.007	4.0E-40	trans
1921	SERPINB9	P50453	Serpin B9	rs7751676	2931879	6	T	C	0.064	0.223	0.015	1.1E-47	cis
1921	SERPINB9	P50453	Serpin B9	rs6993770	106581528	8	A	T	0.713	0.070	0.008	3.7E-17	trans
1922	SERPINC1	P01008	Antithrombin-III	rs2227624	173884010	1	A	T	0.997	0.871	0.063	1.4E-43	cis
1922	SERPINC1	P01008	Antithrombin-III	rs1260326	27730940	2	T	C	0.392	0.089	0.008	1.2E-32	trans
1922	SERPINC1	P01008	Antithrombin-III	rs7920036	65293860	10	T	C	0.525	0.067	0.007	5.5E-20	trans
1922	SERPINC1	P01008	Antithrombin-III	rs123698	807442	19	C	G	0.605	0.058	0.007	7.5E-15	trans
1923	SERPIND1	P05546	Heparin cofactor 2	rs1260326	27730940	2	T	C	0.392	0.111	0.007	6.4E-51	trans
1923	SERPIND1	P05546	Heparin cofactor 2	rs2856451	32011358	6	G	A	0.514	0.055	0.007	3.3E-14	trans
1923	SERPIND1	P05546	Heparin cofactor 2	rs36019311	73051990	7	G	GTA	0.871	0.100	0.011	1.5E-20	trans
1923	SERPIND1	P05546	Heparin cofactor 2	rs217184	72105965	16	C	T	0.196	0.319	0.009	2.1E-253	trans
1923	SERPIND1	P05546	Heparin cofactor 2	rs393665	54755860	19	T	A	0.703	0.066	0.008	5.8E-17	trans
1923	SERPIND1	P05546	Heparin cofactor 2	rs117254553	21029718	22	G	T	0.997	0.872	0.076	1.7E-30	cis
1923	SERPIND1	P05546	Heparin cofactor 2	rs738408	44324730	22	C	T	0.783	0.064	0.009	1.7E-13	trans
1924	SERPINE1	P05121	Plasminogen activator inhibitor 1	rs2274319	156450873	1	T	C	0.347	0.058	0.008	8.1E-14	trans
1924	SERPINE1	P05121	Plasminogen activator inhibitor 1	rs13412535	224874874	2	G	A	0.769	0.061	0.009	4.3E-12	trans
1924	SERPINE1	P05121	Plasminogen activator inhibitor 1	rs78909033	241510903	2	G	A	0.864	0.085	0.011	5.4E-15	trans
1924	SERPINE1	P05121	Plasminogen activator inhibitor 1	rs7618405	18250509	3	C	A	0.795	0.064	0.009	2.8E-12	trans
1924	SERPINE1	P05121	Plasminogen activator inhibitor 1	rs1354034	56849749	3	T	C	0.396	0.121	0.008	9.8E-58	trans
1924	SERPINE1	P05121	Plasminogen activator inhibitor 1	rs3792376	122844951	3	T	G	0.406	0.052	0.008	5.2E-12	trans
1924	SERPINE1	P05121	Plasminogen activator inhibitor 1	rs4572884	102783351	4	C	T	0.602	0.058	0.008	2.1E-14	trans
1924	SERPINE1	P05121	Plasminogen activator inhibitor 1	rs114694170	88180196	5	C	T	0.060	0.150	0.016	1.3E-21	trans
1924	SERPINE1	P05121	Plasminogen activator inhibitor 1	rs2631367	131705458	5	G	C	0.519	0.057	0.007	2.1E-14	trans
1924	SERPINE1	P05121	Plasminogen activator inhibitor 1	rs35173808	31326148	6	C	A	0.903	0.085	0.013	1.5E-11	trans
1924	SERPINE1	P05121	Plasminogen activator inhibitor 1	rs6961069	80218961	7	T	C	0.403	0.066	0.008	3.7E-18	trans
1924	SERPINE1	P05121	Plasminogen activator inhibitor 1	rs2227674	100776208	7	A	G	0.800	0.119	0.009	5.9E-37	cis
1924	SERPINE1	P05121	Plasminogen activator inhibitor 1	rs6993770	106581528	8	A	T	0.713	0.160	0.008	8.1E-85	trans
1924	SERPINE1	P05121	Plasminogen activator inhibitor 1	rs61469632	135861990	9	T	C	0.937	0.138	0.015	1.5E-19	trans
1924	SERPINE1	P05121	Plasminogen activator inhibitor 1	rs34377578	104336426	10	C	A	0.253	0.071	0.009	1.1E-16	trans
1924	SERPINE1	P05121	Plasminogen activator inhibitor 1	rs61978213	70653758	14	A	G	0.042	0.127	0.018	7.6E-12	trans
1924	SERPINE1	P05121	Plasminogen activator inhibitor 1	rs60128101	65160389	15	A	T	0.175	0.070	0.010	8.7E-13	trans
1924	SERPINE1	P05121	Plasminogen activator inhibitor 1	rs12445050	81870969	16	T	C	0.138	0.099	0.011	2.1E-20	trans
1924	SERPINE1	P05121	Plasminogen activator inhibitor 1	rs1654425	55538980	19	C	T	0.834	0.159	0.010	5.9E-57	trans
1924	SERPINE1	P05121	Plasminogen activator inhibitor 1	rs3790176	19261922	20	A	G	0.342	0.070	0.008	4.3E-19	trans
1924	SERPINE1	P05121	Plasminogen activator inhibitor 1	rs3002416	39710195	X	C	T	0.592	0.047	0.006	4.2E-13	trans
1924	SERPINE1	P05121	Plasminogen activator inhibitor 1	rs6616172	99932520	X	T	G	0.346	0.049	0.006	1.9E-14	trans
1925	SERPINE2	P07093	Glia-derived nexin	rs12086222	156869630	1	C	G	0.111	0.078	0.011	1.1E-11	trans
1925	SERPINE2	P07093	Glia-derived nexin	rs68066031	224880498	2	T	C	0.770	0.632	0.009	0.0E+00	cis
1925	SERPINE2	P07093	Glia-derived nexin	rs7618405	18250509	3	C	A	0.795	0.069	0.009	9.6E-15	trans
1925	SERPINE2	P07093	Glia-derived nexin	rs1354034	56849749	3	T	C	0.396	0.062	0.007	3.2E-17	trans
1925	SERPINE2	P07093	Glia-derived nexin	rs10016018	102794621	4	A	T	0.603	0.054	0.007	2.7E-13	trans
1925	SERPINE2	P07093	Glia-derived nexin	rs2516471	31400292	6	G	A	0.936	0.101	0.015	1.1E-11	trans
1925	SERPINE2	P07093	Glia-derived nexin	rs6961069	80218961	7	T	C	0.402	0.052	0.007	2.2E-12	trans
1925	SERPINE2	P07093	Glia-derived nexin	rs6993770	106581528	8	A	T	0.713	0.122	0.008	3.3E-53	trans
1925	SERPINE2	P07093	Glia-derived nexin	rs61469632	135861990	9	T	C	0.937	0.119	0.015	1.2E-15	trans
1925	SERPINE2	P07093	Glia-derived nexin	rs7080386	65048306	10	A	C	0.412	0.085	0.007	8.8E-31	trans
1925	SERPINE2	P07093	Glia-derived nexin	rs12445050	81870969	16	T	C	0.138	0.099	0.010	2.1E-21	trans
1925	SERPINE2	P07093	Glia-derived nexin	rs1654425	55538980	19	C	T	0.834	0.142	0.010	4.3E-48	trans
1925	SERPINE2	P07093	Glia-derived nexin	rs6081569	19303786	20	C	G	0.368	0.061	0.007	4.0E-16	trans
1926	SERPINF1	P36955	Pigment epithelium-derived factor	rs61747728	179526214	1	T	C	0.038	0.166	0.019	4.4E-19	trans
1926	SERPINF1	P36955	Pigment epithelium-derived factor	rs62088172	1666253	17	C	T	0.647	0.283	0.008	6.1E-296	cis
1927	SERPINF2	P08697	Alpha-2-antiplasmin	rs140584594	110232983	1	G	A	0.732	0.064	0.008	5.6E-15	trans
1927	SERPINF2	P08697	Alpha-2-antiplasmin	rs1260326	27730940	2	T	C	0.392	0.090	0.007	3.0E-33	trans
1927	SERPINF2	P08697	Alpha-2-antiplasmin	rs11078597	1618363	17	T	C	0.812	0.207	0.009	9.9E-108	cis
1928	SERPING1	P05155	Plasma protease C1 inhibitor	rs140584594	110232983	1	G	A	0.732	0.063	0.008	4.6E-15	trans
1928	SERPING1	P05155	Plasma protease C1 inhibitor	rs9427402	161492667	1	C	G	0.115	0.094	0.011	5.1E-17	trans
1928	SERPING1	P05155	Plasma protease C1 inhibitor	rs1260326	27730940	2	T	C	0.392	0.105	0.007	4.1E-46	trans
1928	SERPING1	P05155	Plasma protease C1 inhibitor	rs35332062	73012042	7	G	A	0.873	0.084	0.011	8.3E-15	trans
1928	SERPING1	P05155	Plasma protease C1 inhibitor	rs9987289	9183358	8	G	A	0.908	0.091	0.012	2.5E-13	trans
1928	SERPING1	P05155	Plasma protease C1 inhibitor	rs112055915	16044767	8	A	G	0.931	0.096	0.014	9.9E-12	trans
1928	SERPING1	P05155	Plasma protease C1 inhibitor	rs150336831	49669257	11	T	A	0.042	0.184	0.019	9.9E-23	trans
1928	SERPING1	P05155	Plasma protease C1 inhibitor	rs28362944	57365723	11	T	C	0.951	0.425	0.017	1.5E-140	cis
1928	SERPING1	P05155	Plasma protease C1 inhibitor	rs28929474	94844947	14	C	T	0.979	0.268	0.025	1.0E-26	trans
1929	SERPINH1	P50454	Serpin H1	rs1354034	56849749	3	C	T	0.604	0.061	0.008	2.1E-15	trans
1929	SERPINH1	P50454	Serpin H1	rs9886239	50336551	7	C	A	0.331	0.064	0.008	9.8E-16	trans
1929	SERPINH1	P50454	Serpin H1	rs7080386	65048306	10	A	C	0.412	0.080	0.008	7.3E-26	trans
1929	SERPINH1	P50454	Serpin H1	rs636418	75292742	11	T	A	0.579	0.137	0.008	6.0E-70	cis
1929	SERPINH1	P50454	Serpin H1	rs2006166	106384722	14	T	C	0.882	0.120	0.013	4.8E-20	trans
1930	SERPINI1	Q99574	Neuroserpin	rs11717419	167464512	3	A	G	0.221	0.462	0.009	0.0E+00	cis
1930	SERPINI1	Q99574	Neuroserpin	rs1801020	176836532	5	G	A	0.745	0.138	0.008	3.7E-65	trans
1930	SERPINI1	Q99574	Neuroserpin	rs56278466	17875857	10	G	T	0.661	0.053	0.007	1.1E-12	trans
1930	SERPINI1	Q99574	Neuroserpin	rs10740118	65101207	10	C	G	0.414	0.050	0.007	3.3E-12	trans
1930	SERPINI1	Q99574	Neuroserpin	rs79629788	44562148	20	T	C	0.023	0.163	0.023	2.7E-12	trans
1931	SERPINI2	O75830	Serpin I2	rs145849315	3194320	1	T	C	0.903	0.088	0.012	3.0E-14	trans
1931	SERPINI2	O75830	Serpin I2	rs35004807	51108411	1	TGTAA	T	0.344	0.067	0.007	3.3E-23	trans
1931	SERPINI2	O75830	Serpin I2	rs17032925	67266483	2	T	C	0.917	0.142	0.012	1.7E-34	trans
1931	SERPINI2	O75830	Serpin I2	rs6444344	167178334	3	A	G	0.124	0.509	0.010	0.0E+00	cis
1931	SERPINI2	O75830	Serpin I2	rs9398804	126703390	6	T	A	0.561	0.071	0.006	3.4E-28	trans
1931	SERPINI2	O75830	Serpin I2	rs13258924	18714874	8	A	G	0.287	0.058	0.007	4.0E-16	trans
1931	SERPINI2	O75830	Serpin I2	rs4733612	129569999	8	A	G	0.733	0.054	0.007	5.7E-14	trans
1931	SERPINI2	O75830	Serpin I2	rs8176743	136131415	9	T	C	0.061	1.146	0.014	0.0E+00	trans
1931	SERPINI2	O75830	Serpin I2	rs174564	61588305	11	G	A	0.349	0.059	0.007	3.6E-19	trans
1931	SERPINI2	O75830	Serpin I2	rs74997273	100588748	11	A	T	0.732	0.060	0.007	7.2E-17	trans
1931	SERPINI2	O75830	Serpin I2	rs9579128	28481938	13	T	C	0.590	0.065	0.006	8.6E-24	trans
1931	SERPINI2	O75830	Serpin I2	rs28365941	333138	16	T	C	0.864	0.138	0.010	2.1E-45	trans
1931	SERPINI2	O75830	Serpin I2	rs72802342	75234872	16	C	A	0.924	0.631	0.013	0.0E+00	trans
1931	SERPINI2	O75830	Serpin I2	rs1126464	89704365	16	G	C	0.756	0.074	0.008	3.0E-22	trans
1931	SERPINI2	O75830	Serpin I2	rs186021206	7069412	17	A	G	0.006	0.405	0.043	1.0E-20	trans
1931	SERPINI2	O75830	Serpin I2	rs17138478	36073320	17	A	C	0.131	0.071	0.009	6.4E-14	trans
1931	SERPINI2	O75830	Serpin I2	rs62090602	42413766	18	T	A	0.077	0.098	0.012	1.8E-16	trans
1931	SERPINI2	O75830	Serpin I2	rs601338	49206674	19	A	G	0.509	0.332	0.007	0.0E+00	trans
1931	SERPINI2	O75830	Serpin I2	rs2410017	39690219	21	T	C	0.615	0.054	0.007	1.3E-16	trans
1932	SESTD1	Q86VW0	SEC14 domain and spectrin repeat-containing protein 1	rs10170520	180094613	2	A	T	0.671	0.104	0.008	1.2E-37	cis
1932	SESTD1	Q86VW0	SEC14 domain and spectrin repeat-containing protein 1	rs1354034	56849749	3	C	T	0.604	0.055	0.008	1.1E-12	trans
1932	SESTD1	Q86VW0	SEC14 domain and spectrin repeat-containing protein 1	rs10733789	64948684	10	C	T	0.313	0.064	0.008	5.0E-15	trans
1933	SETMAR	Q53H47	Histone-lysine N-methyltransferase SETMAR	rs35083095	4403818	3	T	C	0.507	0.216	0.007	1.3E-194	cis
1934	SEZ6	Q53EL9	Seizure protein 6 homolog	rs186021206	7069412	17	A	G	0.006	0.435	0.052	9.2E-17	trans
1935	SEZ6L	Q9BYH1	Seizure 6-like protein	rs781657	57778685	4	G	A	0.184	0.064	0.009	4.2E-12	trans
1935	SEZ6L	Q9BYH1	Seizure 6-like protein	rs9270747	32568292	6	G	A	0.657	0.055	0.008	3.6E-13	trans
1935	SEZ6L	Q9BYH1	Seizure 6-like protein	rs10838702	47410888	11	T	G	0.386	0.056	0.007	3.9E-14	trans
1935	SEZ6L	Q9BYH1	Seizure 6-like protein	rs60194243	126247299	11	C	T	0.941	0.114	0.015	4.0E-14	trans
1935	SEZ6L	Q9BYH1	Seizure 6-like protein	rs186021206	7069412	17	A	G	0.006	0.488	0.049	3.1E-23	trans
1935	SEZ6L	Q9BYH1	Seizure 6-like protein	rs137203	26688831	22	G	T	0.981	0.571	0.026	4.4E-105	cis
1936	SEZ6L2	Q6UXD5	Seizure 6-like protein 2	rs1260326	27730940	2	C	T	0.608	0.047	0.007	1.5E-11	trans
1936	SEZ6L2	Q6UXD5	Seizure 6-like protein 2	rs1961950	57820817	4	C	T	0.915	0.182	0.012	2.2E-50	trans
1936	SEZ6L2	Q6UXD5	Seizure 6-like protein 2	rs2523567	31330015	6	C	G	0.768	0.075	0.008	1.8E-20	trans
1936	SEZ6L2	Q6UXD5	Seizure 6-like protein 2	rs56278466	17875857	10	G	T	0.661	0.057	0.007	9.4E-16	trans
1936	SEZ6L2	Q6UXD5	Seizure 6-like protein 2	rs113443718	29892184	16	A	G	0.308	0.162	0.007	1.5E-106	cis
1936	SEZ6L2	Q6UXD5	Seizure 6-like protein 2	rs186021206	7069412	17	A	G	0.006	0.663	0.047	3.7E-45	trans
1937	SF3B4	Q15427	Splicing factor 3B subunit 4	rs12078573	149903609	1	G	A	0.921	0.097	0.014	4.7E-12	cis
1937	SF3B4	Q15427	Splicing factor 3B subunit 4	rs10067881	95162475	5	G	A	0.871	0.109	0.011	3.7E-22	trans
1937	SF3B4	Q15427	Splicing factor 3B subunit 4	rs6993770	106581528	8	A	T	0.714	0.063	0.008	2.8E-14	trans
1938	SFRP1	Q8N474	Secreted frizzled-related protein 1	rs2318761	150257714	1	G	A	0.468	0.055	0.007	1.8E-14	trans
1938	SFRP1	Q8N474	Secreted frizzled-related protein 1	rs72643819	41167193	8	G	T	0.613	0.189	0.007	2.8E-140	cis
1938	SFRP1	Q8N474	Secreted frizzled-related protein 1	rs6420185	144303071	8	G	A	0.612	0.062	0.007	1.1E-16	trans
1938	SFRP1	Q8N474	Secreted frizzled-related protein 1	rs13651	43974079	20	T	C	0.488	0.061	0.007	6.6E-18	trans
1939	SFRP4	Q6FHJ7	Secreted frizzled-related protein 4	rs7526027	59683994	1	T	C	0.708	0.062	0.008	4.1E-14	trans
1939	SFRP4	Q6FHJ7	Secreted frizzled-related protein 4	rs75207237	37972390	7	A	T	0.128	0.296	0.011	2.1E-155	cis
1939	SFRP4	Q6FHJ7	Secreted frizzled-related protein 4	rs35887873	126219396	11	C	T	0.228	0.138	0.009	1.8E-55	trans
1940	SFTPA1	Q8IWL2	Pulmonary surfactant-associated protein A1	rs1965708	81317045	10	G	T	0.813	0.284	0.010	2.9E-181	cis
1941	SFTPA2	Q8IWL1	Pulmonary surfactant-associated protein A2	rs11675149	203941042	2	C	A	0.429	0.047	0.007	1.6E-12	trans
1941	SFTPA2	Q8IWL1	Pulmonary surfactant-associated protein A2	rs6883627	126465924	5	C	T	0.459	0.054	0.007	3.1E-16	trans
1941	SFTPA2	Q8IWL1	Pulmonary surfactant-associated protein A2	rs17886395	81318663	10	G	C	0.132	0.940	0.011	0.0E+00	cis
1941	SFTPA2	Q8IWL1	Pulmonary surfactant-associated protein A2	rs2753532	106359076	14	T	C	0.118	0.099	0.012	8.9E-18	trans
1941	SFTPA2	Q8IWL1	Pulmonary surfactant-associated protein A2	rs117981338	71088843	17	T	C	0.014	0.250	0.028	1.0E-18	trans
1942	SFTPD	P35247	Pulmonary surfactant-associated protein D	rs2075571	155174106	1	T	C	0.424	0.042	0.006	1.8E-13	trans
1942	SFTPD	P35247	Pulmonary surfactant-associated protein D	rs56218657	207269449	1	G	A	0.962	0.115	0.015	7.5E-15	trans
1942	SFTPD	P35247	Pulmonary surfactant-associated protein D	rs10031518	89892798	4	A	T	0.341	0.056	0.006	4.5E-21	trans
1942	SFTPD	P35247	Pulmonary surfactant-associated protein D	rs4262138	129322675	5	T	A	0.143	0.065	0.008	1.3E-15	trans
1942	SFTPD	P35247	Pulmonary surfactant-associated protein D	rs12660817	31336757	6	G	A	0.055	0.694	0.013	0.0E+00	trans
1942	SFTPD	P35247	Pulmonary surfactant-associated protein D	rs2451951	109496630	9	T	C	0.505	0.113	0.006	1.1E-87	trans
1942	SFTPD	P35247	Pulmonary surfactant-associated protein D	rs721917	81706324	10	A	G	0.582	0.758	0.007	0.0E+00	cis
1942	SFTPD	P35247	Pulmonary surfactant-associated protein D	rs35705950	1241221	11	T	G	0.110	0.066	0.009	3.2E-13	trans
1942	SFTPD	P35247	Pulmonary surfactant-associated protein D	rs635804	109903108	13	C	G	0.285	0.043	0.006	6.1E-12	trans
1942	SFTPD	P35247	Pulmonary surfactant-associated protein D	rs58462329	84403753	16	G	C	0.855	0.214	0.008	1.5E-152	trans
1942	SFTPD	P35247	Pulmonary surfactant-associated protein D	rs369234167	44611782	17	T	C	0.800	0.064	0.008	1.7E-17	trans
1943	SGSH	P51688	N-sulphoglucosamine sulphohydrolase	rs174557	61581368	11	G	A	0.309	0.040	0.006	1.3E-12	trans
1943	SGSH	P51688	N-sulphoglucosamine sulphohydrolase	rs145078947	93652974	14	T	G	0.003	0.840	0.051	4.9E-60	trans

1943	SGSH	P51688	N-sulphoglucosamine sulphohydrolase	rs7503034	78184393	17	C	T	0.669	0.886	0.007	0.0E+00	cis
1943	SGSH	P51688	N-sulphoglucosamine sulphohydrolase	rs58542926	19379549	19	T	C	0.075	0.096	0.010	1.4E-22	trans
1943	SGSH	P51688	N-sulphoglucosamine sulphohydrolase	rs429358	45411941	19	T	C	0.845	0.051	0.007	1.8E-12	trans
1943	SGSH	P51688	N-sulphoglucosamine sulphohydrolase	rs738409	44324727	22	G	C	0.217	0.055	0.006	1.8E-18	trans
1944	SH2B3	Q9UQQ2	SH2B adapter protein 3	rs11950562	131652529	5	A	C	0.522	0.055	0.008	4.6E-13	trans
1944	SH2B3	Q9UQQ2	SH2B adapter protein 3	rs342293	106372219	7	C	G	0.540	0.061	0.008	7.6E-16	trans
1944	SH2B3	Q9UQQ2	SH2B adapter protein 3	rs6993770	106581528	8	A	T	0.713	0.089	0.008	2.4E-26	trans
1944	SH2B3	Q9UQQ2	SH2B adapter protein 3	rs409801	4744743	9	C	T	0.507	0.053	0.008	2.7E-12	trans
1945	SH2D1A	O60880	SH2 domain-containing protein 1A	rs75763843	42062380	18	C	A	0.126	0.128	0.011	2.2E-29	trans
1945	SH2D1A	O60880	SH2 domain-containing protein 1A	rs12164382	123480147	X	T	C	0.417	0.258	0.006	0.0E+00	cis
1946	SH3BP1	Q9Y3L3	SH3 domain-binding protein 1	rs12710562	38029723	22	G	A	0.183	0.268	0.010	4.0E-161	cis
1947	SH3GLB2	Q9NR46	Endophilin-B2	rs9866126	186391067	3	A	G	0.660	0.094	0.008	1.6E-34	trans
1947	SH3GLB2	Q9NR46	Endophilin-B2	rs62585602	131782964	9	G	A	0.525	0.273	0.007	4.9E-301	cis
1948	SHBG	P04278	Sex hormone-binding globulin	rs114165349	27021913	1	G	C	0.977	0.167	0.022	5.2E-14	trans
1948	SHBG	P04278	Sex hormone-binding globulin	rs1730850	107597988	1	C	T	0.343	0.053	0.007	1.1E-13	trans
1948	SHBG	P04278	Sex hormone-binding globulin	rs1260326	27730940	2	C	T	0.609	0.064	0.007	7.9E-21	trans
1948	SHBG	P04278	Sex hormone-binding globulin	rs13108218	3443931	4	A	G	0.381	0.051	0.007	2.4E-13	trans
1948	SHBG	P04278	Sex hormone-binding globulin	rs7015	97920623	7	G	A	0.815	0.068	0.009	2.9E-15	trans
1948	SHBG	P04278	Sex hormone-binding globulin	rs7047907	86368660	9	G	A	0.254	0.053	0.008	5.9E-12	trans
1948	SHBG	P04278	Sex hormone-binding globulin	rs28929474	94844947	14	T	C	0.021	0.184	0.023	3.8E-15	trans
1948	SHBG	P04278	Sex hormone-binding globulin	rs139974673	44027885	15	T	C	0.976	0.147	0.022	1.3E-11	trans
1948	SHBG	P04278	Sex hormone-binding globulin	rs56332871	96714816	15	A	C	0.270	0.058	0.008	9.5E-15	trans
1948	SHBG	P04278	Sex hormone-binding globulin	rs858519	7531965	17	C	T	0.558	0.274	0.007	0.0E+00	cis
1948	SHBG	P04278	Sex hormone-binding globulin	rs149751685	17347142	19	A	G	0.025	0.180	0.024	4.8E-14	trans
1948	SHBG	P04278	Sex hormone-binding globulin	rs5942965	109800345	X	A	G	0.403	0.061	0.006	2.1E-27	trans
1949	SHD	Q96IW2	SH2 domain-containing adapter protein D	rs58534292	106483584	14	C	T	0.651	0.068	0.008	5.2E-17	trans
1949	SHD	Q96IW2	SH2 domain-containing adapter protein D	rs72481571	106369875	14	A	G	0.106	0.214	0.014	1.8E-52	trans
1950	SHH	Q15465	Sonic hedgehog protein	rs10225434	156173333	7	T	C	0.581	0.057	0.008	5.6E-13	cis
1951	SHISA5	Q8N114	Protein shisa-5	rs11706087	48539536	3	T	C	0.975	0.392	0.023	1.4E-63	cis
1951	SHISA5	Q8N114	Protein shisa-5	rs13146355	77412140	4	A	G	0.456	0.048	0.007	7.1E-12	trans
1951	SHISA5	Q8N114	Protein shisa-5	rs7203642	20367130	16	A	G	0.808	0.067	0.009	1.5E-13	trans
1952	SHMT1	P34896	Serine hydroxymethyltransferase, cytosolic	rs1354034	56849749	3	C	T	0.604	0.057	0.007	4.8E-18	trans
1952	SHMT1	P34896	Serine hydroxymethyltransferase, cytosolic	rs342296	106372903	7	G	A	0.544	0.047	0.006	4.4E-13	trans
1952	SHMT1	P34896	Serine hydroxymethyltransferase, cytosolic	rs6993770	106581528	8	A	T	0.713	0.074	0.007	2.0E-25	trans
1952	SHMT1	P34896	Serine hydroxymethyltransferase, cytosolic	rs669340	18263957	17	C	G	0.634	0.719	0.008	0.0E+00	cis
1953	SHPK	Q9UHJ6	Sedoheptulokinase	rs224495	3513127	17	G	A	0.899	0.132	0.013	5.9E-25	cis
1954	SIAE	Q9HAT2	Sialate O-acetylesterase	rs114269697	65314212	1	C	A	0.987	0.239	0.033	5.7E-13	trans
1954	SIAE	Q9HAT2	Sialate O-acetylesterase	rs6993770	106581528	8	A	T	0.714	0.067	0.008	2.3E-16	trans
1954	SIAE	Q9HAT2	Sialate O-acetylesterase	rs56278466	17875857	10	G	T	0.661	0.074	0.008	2.6E-21	trans
1954	SIAE	Q9HAT2	Sialate O-acetylesterase	rs7896518	65104500	10	G	A	0.424	0.094	0.008	4.2E-35	trans
1954	SIAE	Q9HAT2	Sialate O-acetylesterase	rs78778622	124530664	11	T	C	0.940	0.444	0.016	1.0E-174	cis
1954	SIAE	Q9HAT2	Sialate O-acetylesterase	rs72701845	93217023	14	G	A	0.962	0.155	0.020	9.6E-15	trans
1954	SIAE	Q9HAT2	Sialate O-acetylesterase	rs892090	55539072	19	G	T	0.834	0.095	0.010	2.0E-21	trans
1955	SIGLEC1	Q9BZZ2	Sialoadhesin	rs17849502	183532580	1	T	G	0.052	0.111	0.015	7.8E-13	trans
1955	SIGLEC1	Q9BZZ2	Sialoadhesin	rs62165726	134966562	2	C	A	0.963	0.408	0.018	2.7E-108	trans
1955	SIGLEC1	Q9BZZ2	Sialoadhesin	rs115216147	70665526	5	A	T	0.067	0.107	0.014	5.3E-14	trans
1955	SIGLEC1	Q9BZZ2	Sialoadhesin	rs11185602	50299077	7	A	G	0.675	0.064	0.007	4.3E-18	trans
1955	SIGLEC1	Q9BZZ2	Sialoadhesin	rs2031902	33117524	9	C	T	0.413	0.072	0.007	2.9E-24	trans
1955	SIGLEC1	Q9BZZ2	Sialoadhesin	rs11220505	126334805	11	T	C	0.222	0.099	0.008	5.1E-32	trans
1955	SIGLEC1	Q9BZZ2	Sialoadhesin	rs186021206	7069412	17	A	G	0.006	1.325	0.048	2.7E-170	trans
1955	SIGLEC1	Q9BZZ2	Sialoadhesin	rs11668950	18282940	19	A	G	0.269	0.090	0.008	6.3E-31	trans
1955	SIGLEC1	Q9BZZ2	Sialoadhesin	rs189448562	3705115	20	G	C	0.989	1.842	0.035	0.0E+00	cis
1956	SIGLEC10	Q96LC7	Sialic acid-binding Ig-like lectin 10	rs1257169	134963862	2	C	A	0.502	0.052	0.007	1.2E-13	trans
1956	SIGLEC10	Q96LC7	Sialic acid-binding Ig-like lectin 10	rs4857410	98353913	3	C	T	0.851	0.081	0.010	2.3E-16	trans
1956	SIGLEC10	Q96LC7	Sialic acid-binding Ig-like lectin 10	rs634501	180218668	5	A	G	0.264	0.060	0.008	5.2E-14	trans
1956	SIGLEC10	Q96LC7	Sialic acid-binding Ig-like lectin 10	rs562289	32577046	6	T	C	0.230	0.068	0.008	3.8E-16	trans
1956	SIGLEC10	Q96LC7	Sialic acid-binding Ig-like lectin 10	rs186021206	7069412	17	A	G	0.006	0.840	0.048	1.5E-67	trans
1956	SIGLEC10	Q96LC7	Sialic acid-binding Ig-like lectin 10	rs34557412	16852187	17	G	A	0.007	0.440	0.043	6.5E-25	trans
1956	SIGLEC10	Q96LC7	Sialic acid-binding Ig-like lectin 10	rs148783636	51910979	19	T	C	0.980	1.577	0.029	0.0E+00	cis
1956	SIGLEC10	Q96LC7	Sialic acid-binding Ig-like lectin 10	rs738409	44324727	22	G	C	0.217	0.061	0.008	7.1E-13	trans
1957	SIGLEC5	O15389	Sialic acid-binding Ig-like lectin 5	rs186021206	7069412	17	A	G	0.006	0.445	0.040	2.3E-29	trans
1957	SIGLEC5	O15389	Sialic acid-binding Ig-like lectin 5	rs1106476	52130637	19	T	A	0.887	1.219	0.012	0.0E+00	cis
1958	SIGLEC6	O43699	Sialic acid-binding Ig-like lectin 6	rs115427247	92878407	1	C	T	0.970	0.156	0.020	3.2E-15	trans
1958	SIGLEC6	O43699	Sialic acid-binding Ig-like lectin 6	rs4601639	205656583	1	T	C	0.734	0.065	0.008	1.2E-17	trans
1958	SIGLEC6	O43699	Sialic acid-binding Ig-like lectin 6	rs74227709	247722588	1	A	G	0.074	0.141	0.013	2.4E-28	trans
1958	SIGLEC6	O43699	Sialic acid-binding Ig-like lectin 6	rs3111414	8443859	2	C	G	0.202	0.119	0.008	1.6E-45	trans
1958	SIGLEC6	O43699	Sialic acid-binding Ig-like lectin 6	rs4384720	145425401	2	G	C	0.920	0.116	0.012	2.6E-21	trans
1958	SIGLEC6	O43699	Sialic acid-binding Ig-like lectin 6	rs1516527	148609704	3	C	T	0.950	0.223	0.015	6.6E-47	trans
1958	SIGLEC6	O43699	Sialic acid-binding Ig-like lectin 6	rs13107325	103188709	4	T	C	0.075	0.116	0.013	1.2E-19	trans
1958	SIGLEC6	O43699	Sialic acid-binding Ig-like lectin 6	rs4863714	140907337	4	G	A	0.641	0.055	0.007	2.5E-15	trans
1958	SIGLEC6	O43699	Sialic acid-binding Ig-like lectin 6	rs3131618	31434621	6	G	A	0.142	0.124	0.010	4.4E-38	trans
1958	SIGLEC6	O43699	Sialic acid-binding Ig-like lectin 6	rs56278466	17875857	10	G	T	0.661	0.049	0.007	2.5E-12	trans
1958	SIGLEC6	O43699	Sialic acid-binding Ig-like lectin 6	rs4937127	126290510	11	A	G	0.478	0.103	0.007	8.2E-53	trans
1958	SIGLEC6	O43699	Sialic acid-binding Ig-like lectin 6	rs76428106	28604007	13	C	T	0.013	0.491	0.031	3.1E-57	trans
1958	SIGLEC6	O43699	Sialic acid-binding Ig-like lectin 6	rs111164440	89046625	16	A	G	0.632	0.054	0.007	2.4E-14	trans
1958	SIGLEC6	O43699	Sialic acid-binding Ig-like lectin 6	rs146261845	7012254	17	T	C	0.007	0.434	0.043	1.1E-23	trans
1958	SIGLEC6	O43699	Sialic acid-binding Ig-like lectin 6	rs2659005	79218714	17	T	C	0.442	0.046	0.007	7.8E-12	trans
1958	SIGLEC6	O43699	Sialic acid-binding Ig-like lectin 6	rs78744187	33754548	19	T	C	0.082	0.150	0.013	5.0E-29	trans
1958	SIGLEC6	O43699	Sialic acid-binding Ig-like lectin 6	rs62617068	52034549	19	C	A	0.988	1.758	0.034	0.0E+00	cis
1959	SIGLEC7	Q9Y286	Sialic acid-binding Ig-like lectin 7	rs1257169	134963862	2	C	A	0.501	0.059	0.007	1.2E-16	trans
1959	SIGLEC7	Q9Y286	Sialic acid-binding Ig-like lectin 7	rs11721064	98408826	3	G	T	0.395	0.056	0.007	1.5E-14	trans
1959	SIGLEC7	Q9Y286	Sialic acid-binding Ig-like lectin 7	rs4393849	194478411	3	G	A	0.308	0.056	0.008	3.5E-13	trans
1959	SIGLEC7	Q9Y286	Sialic acid-binding Ig-like lectin 7	rs11220462	126243952	11	G	A	0.868	0.084	0.010	1.7E-15	trans
1959	SIGLEC7	Q9Y286	Sialic acid-binding Ig-like lectin 7	rs186021206	7069412	17	A	G	0.006	0.962	0.049	2.2E-86	trans
1959	SIGLEC7	Q9Y286	Sialic acid-binding Ig-like lectin 7	rs2160725	66395726	17	A	C	0.515	0.083	0.007	1.1E-30	trans
1959	SIGLEC7	Q9Y286	Sialic acid-binding Ig-like lectin 7	rs60202083	47772184	19	T	A	0.578	0.066	0.007	9.4E-19	trans
1959	SIGLEC7	Q9Y286	Sialic acid-binding Ig-like lectin 7	rs140185670	51646140	19	G	C	0.932	0.635	0.015	0.0E+00	cis
1960	SIGLEC8	Q9NYZ4	Sialic acid-binding Ig-like lectin 8	rs77762937	203467470	2	T	G	0.288	0.053	0.007	9.5E-13	trans
1960	SIGLEC8	Q9NYZ4	Sialic acid-binding Ig-like lectin 8	rs55921103	69810294	3	T	G	0.653	0.100	0.007	5.2E-45	trans
1960	SIGLEC8	Q9NYZ4	Sialic acid-binding Ig-like lectin 8	rs10032035	99785789	4	C	T	0.257	0.057	0.008	1.9E-13	trans
1960	SIGLEC8	Q9NYZ4	Sialic acid-binding Ig-like lectin 8	rs2279587	52214581	5	A	G	0.059	0.132	0.014	2.8E-20	trans
1960	SIGLEC8	Q9NYZ4	Sialic acid-binding Ig-like lectin 8	rs2256974	31555392	6	A	C	0.174	0.065	0.009	2.0E-13	trans
1960	SIGLEC8	Q9NYZ4	Sialic acid-binding Ig-like lectin 8	rs6458951	53508627	6	C	T	0.263	0.068	0.008	8.8E-19	trans
1960	SIGLEC8	Q9NYZ4	Sialic acid-binding Ig-like lectin 8	rs4876611	116671848	8	G	A	0.718	0.170	0.008	6.5E-112	trans
1960	SIGLEC8	Q9NYZ4	Sialic acid-binding Ig-like lectin 8	rs10796294	15523359	10	G	A	0.569	0.078	0.007	5.4E-30	trans
1960	SIGLEC8	Q9NYZ4	Sialic acid-binding Ig-like lectin 8	rs200489612	7106378	17	A	G	0.005	0.455	0.050	1.3E-19	trans
1960	SIGLEC8	Q9NYZ4	Sialic acid-binding Ig-like lectin 8	rs151278524	21075814	18	T	C	0.007	0.280	0.041	1.2E-11	trans
1960	SIGLEC8	Q9NYZ4	Sialic acid-binding Ig-like lectin 8	rs36518	51942903	19	A	G	0.137	0.684	0.011	0.0E+00	cis
1961	SIGLEC9	Q9Y336	Sialic acid-binding Ig-like lectin 9	rs61830291	221001142	1	A	C	0.903	0.070	0.010	1.7E-12	trans
1961	SIGLEC9	Q9Y336	Sialic acid-binding Ig-like lectin 9	rs2593704	135005277	2	G	C	0.218	0.061	0.007	4.1E-18	trans
1961	SIGLEC9	Q9Y336	Sialic acid-binding Ig-like lectin 9	rs10935473	98416900	3	G	T	0.560	0.631	0.007	0.0E+00	trans
1961	SIGLEC9	Q9Y336	Sialic acid-binding Ig-like lectin 9	rs1468906	186648117	3	A	G	0.656	0.049	0.007	3.9E-12	trans
1961	SIGLEC9	Q9Y336	Sialic acid-binding Ig-like lectin 9	rs9260	32610461	6	A	G	0.193	0.111	0.007	9.4E-50	trans
1961	SIGLEC9	Q9Y336	Sialic acid-binding Ig-like lectin 9	rs4841132	9183596	8	A	G	0.092	0.096	0.010	3.7E-21	trans
1961	SIGLEC9	Q9Y336	Sialic acid-binding Ig-like lectin 9	rs3967200	126232385	11	C	T	0.865	0.196	0.009	7.8E-115	trans
1961	SIGLEC9	Q9Y336	Sialic acid-binding Ig-like lectin 9	rs186021206	7069412	17	A	G	0.006	1.557	0.040	0.0E+00	trans
1961	SIGLEC9	Q9Y336	Sialic acid-binding Ig-like lectin 9	rs2075803	51628529	19	A	G	0.447	0.573	0.007	0.0E+00	cis
1962	SIL1	Q9H173	Nucleotide exchange factor SIL1	rs35457250	186338564	3	T	C	0.009	0.444	0.041	2.7E-27	trans
1962	SIL1	Q9H173	Nucleotide exchange factor SIL1	rs2351905	138471330	5	T	C	0.440	0.059	0.008	3.6E-14	cis
1962	SIL1	Q9H173	Nucleotide exchange factor SIL1	rs28929474	94844947	14	T	C	0.021	0.500	0.027	1.6E-76	trans
1962	SIL1	Q9H173	Nucleotide exchange factor SIL1	rs1801689	64210580	17	C	A	0.030	0.165	0.022	2.3E-13	trans
1963	SIRPA	P78324	Tyrosine-protein phosphatase non-receptor type substrate 1	rs61747728	179526214	1	T	C	0.038	0.071	0.010	4.4E-13	trans
1963	SIRPA	P78324	Tyrosine-protein phosphatase non-receptor type substrate 1	rs1260326	27730940	2	C	T	0.609	0.031	0.004	3.5E-16	trans
1963	SIRPA	P78324	Tyrosine-protein phosphatase non-receptor type substrate 1	rs34458031	98752035	3	A	G	0.086	0.072	0.007	7.2E-27	trans
1963	SIRPA	P78324	Tyrosine-protein phosphatase non-receptor type substrate 1	rs507666	136149399	9	G	A	0.815	0.076	0.005	6.7E-54	trans
1963	SIRPA	P78324	Tyrosine-protein phosphatase non-receptor type substrate 1	rs186021206	7069412	17	A	G	0.006	0.431	0.026	1.2E-61	trans
1963	SIRPA	P78324	Tyrosine-protein phosphatase non-receptor type substrate 1	rs2659005	79218714	17	T	C	0.441	0.029	0.004	6.3E-14	trans
1963	SIRPA	P78324	Tyrosine-protein phosphatase non-receptor type substrate 1	rs6136377	1896288	20	A	G	0.618	1.234	0.008	0.0E+00	cis
1964	SIRPB1	O00241	Signal-regulatory protein beta-1	rs61747728	179526214	1	T	C	0.038	0.137	0.016	2.5E-18	trans
1964	SIRPB1	O00241	Signal-regulatory protein beta-1	rs1260326	27730940	2	C	T	0.609	0.044	0.006	1.2E-12	trans
1964	SIRPB1	O00241	Signal-regulatory protein beta-1	rs10053046	131821558	5	A	G	0.675	0.050	0.006	1.3E-14	trans
1964	SIRPB1	O00241	Signal-regulatory protein beta-1	rs13277237	130604563	8	G	A	0.432	0.051	0.006	6.1E-17	trans
1964	SIRPB1	O00241	Signal-regulatory protein beta-1	rs76428106	28604007	13	C	T	0.013	0.279	0.028	6.6E-24	trans
1964	SIRPB1	O00241	Signal-regulatory protein beta-1	rs186021206	7069412	17	A	G	0.006	0.360	0.041	3.6E-18	trans
1964	SIRPB1	O00241	Signal-regulatory protein beta-1	rs1045929	38175426	17	T	C	0.388	0.052	0.006	2.8E-17	trans
1964	SIRPB1	O00241	Signal-regulatory protein beta-1	rs2777895	57879610	17	G	A	0.549	0.079	0.006	1.9E-39	trans
1964	SIRPB1	O00241	Signal-regulatory protein beta-1	rs7224668	79235788	17	T	C	0.442	0.050	0.006	2.4E-16	trans
1964	SIRPB1	O00241	Signal-regulatory protein beta-1	rs79462675	1597175	20	C	A	0.868	0.950	0.011	0.0E+00	cis
1965	SIRT1	Q96EB6	NAD-dependent protein deacetylase sirtuin-1	rs28672722	32626537	6	T	G	0.222	0.093	0.009	1.3E-23	trans
1966	SIRT2	Q8IXJ6	NAD-dependent protein deacetylase sirtuin-2	rs1354034	56849749	3	C	T	0.605	0.063	0.008	2.3E-16	trans
1966	SIRT2	Q8IXJ6	NAD-dependent protein deacetylase sirtuin-2	rs342298	106373646	7	C	T	0.546	0.065	0.008	3.7E-18	trans
1966	SIRT2	Q8IXJ6	NAD-dependent protein deacetylase sirtuin-2	rs7896518	65104500	10	G	A	0.424	0.063	0.008	3.1E-16	trans
1966	SIRT2	Q8IXJ6	NAD-dependent protein deacetylase sirtuin-2	rs11502185	180258	11	C	T	0.260	0.066	0.010	4.9E-12	trans
1966	SIRT2	Q8IXJ6	NAD-dependent protein deacetylase sirtuin-2	rs144373891	39379770	19	C	T	0.991	0.716	0.039	5.9E-74	cis
1967	SIT1	Q9Y3P8	Signaling threshold-regulating transmembrane adapter 1	rs10468017	58678512	15	C	T	0.703	0.066	0.008	1.5E-15	trans
1967	SIT1	Q9Y3P8	Signaling threshold-regulating transmembrane adapter 1	rs12446515	56987015	16	C	T	0.676	0.084	0.008	1.7E-25	trans
1967	SIT1	Q9Y3P8	Signaling threshold-regulating transmembrane adapter 1	rs4613118	7297452	17	A	G	0.141	0.081	0.011	7.0E-14	trans
1967	SIT1	Q9Y3P8	Signaling threshold-regulating transmembrane adapter 1	rs7208422	76130575	17	A	T	0.523	0.084	0.008	9.9E-29	trans
1967	SIT1	Q9Y3P8	Signaling threshold-regulating transmembrane adapter 1	rs10469472	17486926	19	G	T	0.119	0.099	0.012	2.4E-17	trans
1968	SKAP1	Q86WV1	Src kinase-associated phosphoprotein 1	rs28414666	32626451	6	A	G	0.210	0.068	0.009	1.2E-13	trans
1968	SKAP1	Q86WV1	Src kinase-associated phosphoprotein 1	rs76438067	26911904	7	C	T	0.767	0.092	0.009	2.3E-25	trans
1968	SKAP1	Q86WV1	Src kinase-associated phosphoprotein 1	rs5817082	56997349	16	CA	C	0.266	0.060	0.009	1.6E-12	trans
1968	SKAP1	Q86WV1	Src kinase-associated phosphoprotein 1	rs7208422	76130575	17	A	T	0.522	0.067	0.007	2.1E-19	trans

1968	SKAP1	Q86WV1	Src kinase-associated phosphoprotein 1	rs74460666	17521101	19	G	T	0.121	0.093	0.011	3.8E-16	trans
1969	SKAP2	O75563	Src kinase-associated phosphoprotein 2	rs1354034	56849749	3	C	T	0.604	0.069	0.008	2.2E-19	trans
1969	SKAP2	O75563	Src kinase-associated phosphoprotein 2	rs17622656	131820997	5	G	A	0.611	0.055	0.008	6.3E-13	trans
1969	SKAP2	O75563	Src kinase-associated phosphoprotein 2	rs6993770	106581528	8	A	T	0.713	0.068	0.008	1.3E-16	trans
1970	SLA2	Q9H6Q3	Src-like-adapter 2	rs113102870	35242796	20	G	A	0.993	0.764	0.047	2.3E-60	cis
1971	SLAMF1	Q13291	Signaling lymphocytic activation molecule	rs37456	44292989	1	T	C	0.352	0.061	0.008	2.1E-14	trans
1971	SLAMF1	Q13291	Signaling lymphocytic activation molecule	rs7535367	160638250	1	G	T	0.861	0.145	0.011	2.1E-39	cis
1971	SLAMF1	Q13291	Signaling lymphocytic activation molecule	rs3821819	186732725	3	G	A	0.613	0.058	0.008	1.1E-13	trans
1971	SLAMF1	Q13291	Signaling lymphocytic activation molecule	rs9264669	31239681	6	T	A	0.573	0.068	0.008	4.3E-18	trans
1971	SLAMF1	Q13291	Signaling lymphocytic activation molecule	rs186021206	7069412	17	A	G	0.006	0.777	0.052	9.4E-50	trans
1972	SLAMF6	Q96DU3	SLAM family member 6	rs11265413	160460099	1	T	A	0.797	0.215	0.009	4.9E-114	cis
1972	SLAMF6	Q96DU3	SLAM family member 6	rs186021206	7069412	17	A	G	0.006	0.441	0.053	6.9E-17	trans
1973	SLAMF7	Q9NQ25	SLAM family member 7	rs188468174	25291697	1	C	T	0.986	0.545	0.032	6.8E-65	trans
1973	SLAMF7	Q9NQ25	SLAM family member 7	rs11581248	160720074	1	C	T	0.864	0.943	0.011	0.0E+00	cis
1973	SLAMF7	Q9NQ25	SLAM family member 7	rs4972973	231737798	2	G	A	0.531	0.056	0.007	9.4E-17	trans
1973	SLAMF7	Q9NQ25	SLAM family member 7	rs7621161	186727170	3	C	A	0.720	0.135	0.008	4.8E-71	trans
1973	SLAMF7	Q9NQ25	SLAM family member 7	rs3094087	31061561	6	T	C	0.829	0.096	0.009	5.9E-27	trans
1973	SLAMF7	Q9NQ25	SLAM family member 7	rs3780480	33163486	9	G	T	0.746	0.054	0.008	2.2E-12	trans
1973	SLAMF7	Q9NQ25	SLAM family member 7	rs186021206	7069412	17	A	G	0.006	0.943	0.046	6.2E-92	trans
1973	SLAMF7	Q9NQ25	SLAM family member 7	rs34562254	16842991	17	A	G	0.097	0.096	0.011	1.7E-17	trans
1973	SLAMF7	Q9NQ25	SLAM family member 7	rs8077394	79258787	17	A	G	0.446	0.054	0.007	1.5E-15	trans
1974	SLAMF8	Q9P0V8	SLAM family member 8	rs34687326	159799910	1	G	A	0.878	1.343	0.012	0.0E+00	cis
1974	SLAMF8	Q9P0V8	SLAM family member 8	rs2190504	30507380	7	C	T	0.443	0.052	0.007	1.0E-13	trans
1974	SLAMF8	Q9P0V8	SLAM family member 8	rs12667978	128777461	7	T	C	0.572	0.049	0.007	5.6E-12	trans
1974	SLAMF8	Q9P0V8	SLAM family member 8	rs186021206	7069412	17	A	G	0.006	0.469	0.049	5.5E-22	trans
1975	SLC16A1	P53985	Monocarboxylate transporter 1	rs6670866	113428827	1	A	G	0.439	0.062	0.008	1.5E-15	cis
1975	SLC16A1	P53985	Monocarboxylate transporter 1	rs3811444	248039451	1	C	T	0.667	0.104	0.008	1.4E-37	trans
1975	SLC16A1	P53985	Monocarboxylate transporter 1	rs4835097	144881840	4	G	A	0.709	0.069	0.008	3.6E-16	trans
1975	SLC16A1	P53985	Monocarboxylate transporter 1	rs633382	139827741	6	C	T	0.964	0.256	0.021	5.7E-35	trans
1976	SLC27A4	Q6P1M0	Long-chain fatty acid transport protein 4	rs1354034	56849749	3	C	T	0.604	0.093	0.008	3.3E-33	trans
1976	SLC27A4	Q6P1M0	Long-chain fatty acid transport protein 4	rs137948800	131069717	9	TA	T	0.123	0.081	0.012	2.4E-12	cis
1976	SLC27A4	Q6P1M0	Long-chain fatty acid transport protein 4	rs7080536	115348046	10	G	A	0.957	0.190	0.019	7.8E-24	trans
1977	SLC39A14	Q15043	Zinc transporter ZIP14	rs79624003	73012785	7	A	G	0.873	0.081	0.011	1.4E-12	trans
1977	SLC39A14	Q15043	Zinc transporter ZIP14	rs35486529	22251868	8	C	T	0.745	0.107	0.009	4.2E-34	cis
1977	SLC39A14	Q15043	Zinc transporter ZIP14	rs28929474	94844947	14	T	C	0.021	0.309	0.027	1.2E-30	trans
1978	SLC39A5	Q6ZMH5	Zinc transporter ZIP5	rs12065546	230295245	1	T	C	0.852	0.078	0.010	3.4E-14	trans
1978	SLC39A5	Q6ZMH5	Zinc transporter ZIP5	rs9263789	31128329	6	G	A	0.835	0.092	0.010	5.5E-21	trans
1978	SLC39A5	Q6ZMH5	Zinc transporter ZIP5	rs7786376	73042614	7	A	G	0.723	0.067	0.008	7.6E-17	trans
1978	SLC39A5	Q6ZMH5	Zinc transporter ZIP5	rs2272662	145639726	8	T	C	0.422	0.058	0.007	3.4E-15	trans
1978	SLC39A5	Q6ZMH5	Zinc transporter ZIP5	rs28929474	94844947	14	T	C	0.021	0.338	0.025	3.4E-40	trans
1978	SLC39A5	Q6ZMH5	Zinc transporter ZIP5	rs11078597	1618363	17	C	T	0.188	0.064	0.009	6.2E-12	trans
1978	SLC39A5	Q6ZMH5	Zinc transporter ZIP5	rs12609794	35555585	19	A	G	0.748	0.056	0.008	1.2E-11	trans
1978	SLC39A5	Q6ZMH5	Zinc transporter ZIP5	rs55953905	42882671	21	T	C	0.247	0.095	0.008	1.7E-29	trans
1979	SLC4A1	P02730	Band 3 anion transport protein	rs12734030	207793964	1	C	T	0.852	0.071	0.010	1.0E-11	trans
1979	SLC4A1	P02730	Band 3 anion transport protein	rs145919155	42307386	17	G	A	0.950	0.138	0.017	6.6E-16	cis
1980	SLC51B	Q86UW2	Organic solute transporter subunit beta	rs4414734	133177981	2	T	A	0.665	0.108	0.008	2.0E-42	trans
1980	SLC51B	Q86UW2	Organic solute transporter subunit beta	rs939885	195955762	3	G	A	0.514	0.168	0.007	1.5E-110	trans
1980	SLC51B	Q86UW2	Organic solute transporter subunit beta	rs11239528	45955036	10	A	G	0.757	0.096	0.009	2.3E-28	trans
1980	SLC51B	Q86UW2	Organic solute transporter subunit beta	rs56398830	103701690	13	G	A	0.989	0.687	0.035	1.9E-83	trans
1980	SLC51B	Q86UW2	Organic solute transporter subunit beta	rs3813582	79749353	16	C	T	0.314	0.055	0.008	6.2E-12	trans
1980	SLC51B	Q86UW2	Organic solute transporter subunit beta	rs12938714	28645110	17	A	G	0.330	0.144	0.008	6.7E-74	trans
1980	SLC51B	Q86UW2	Organic solute transporter subunit beta	rs11078916	37746307	17	T	C	0.279	0.072	0.008	8.8E-18	trans
1980	SLC51B	Q86UW2	Organic solute transporter subunit beta	rs681343	49206462	19	C	T	0.491	0.220	0.008	5.6E-185	trans
1981	SLC9A3R1	O14745	Na(+)/H(+) exchange regulatory cofactor NHE-RF1	rs342299	106373718	7	C	T	0.545	0.058	0.008	2.2E-14	trans
1981	SLC9A3R1	O14745	Na(+)/H(+) exchange regulatory cofactor NHE-RF1	rs6993770	106581528	8	A	T	0.713	0.057	0.008	6.8E-12	trans
1981	SLC9A3R1	O14745	Na(+)/H(+) exchange regulatory cofactor NHE-RF1	rs745666	72744798	17	C	G	0.439	0.092	0.008	6.0E-31	cis
1982	SLC9A3R2	Q15599	Na(+)/H(+) exchange regulatory cofactor NHE-RF2	rs3211995	2089006	16	G	A	0.841	0.147	0.010	1.9E-46	cis
1982	SLC9A3R2	Q15599	Na(+)/H(+) exchange regulatory cofactor NHE-RF2	rs3764261	56993324	16	C	A	0.675	0.059	0.008	1.6E-13	trans
1983	SLIRP	Q9GZT3	SRA stem-loop-interacting RNA-binding protein, mitochondrial	rs77333309	106369865	14	A	G	0.106	0.101	0.014	1.2E-12	trans
1984	SLIT2	O94813	Slit homolog 2 protein	rs1185160	20200351	4	G	T	0.299	0.061	0.008	3.1E-13	cis
1985	SLITRK1	Q96PX8	SLIT and NTRK-like protein 1	rs13107325	103188709	4	T	C	0.075	0.108	0.014	9.8E-15	trans
1985	SLITRK1	Q96PX8	SLIT and NTRK-like protein 1	rs2876803	84368785	13	A	T	0.170	0.157	0.010	4.2E-58	cis
1985	SLITRK1	Q96PX8	SLIT and NTRK-like protein 1	rs186021206	7069412	17	A	G	0.006	0.503	0.051	2.5E-23	trans
1985	SLITRK1	Q96PX8	SLIT and NTRK-like protein 1	rs33950747	36339247	19	T	C	0.075	0.101	0.014	3.2E-13	trans
1986	SLITRK2	Q9H156	SLIT and NTRK-like protein 2	rs61830291	221001142	1	A	C	0.903	0.088	0.012	9.4E-13	trans
1986	SLITRK2	Q9H156	SLIT and NTRK-like protein 2	rs62262391	123105119	3	T	C	0.225	0.074	0.009	1.5E-17	trans
1986	SLITRK2	Q9H156	SLIT and NTRK-like protein 2	rs68119427	57758030	4	G	C	0.349	0.052	0.008	1.1E-11	trans
1986	SLITRK2	Q9H156	SLIT and NTRK-like protein 2	rs13107325	103188709	4	T	C	0.075	0.263	0.014	2.8E-79	trans
1986	SLITRK2	Q9H156	SLIT and NTRK-like protein 2	rs78334147	109017236	5	T	C	0.101	0.105	0.012	5.4E-18	trans
1986	SLITRK2	Q9H156	SLIT and NTRK-like protein 2	rs2811708	21973422	9	T	G	0.265	0.068	0.008	1.7E-16	trans
1986	SLITRK2	Q9H156	SLIT and NTRK-like protein 2	rs1131773	95840256	9	G	A	0.144	0.071	0.010	8.5E-12	trans
1986	SLITRK2	Q9H156	SLIT and NTRK-like protein 2	rs56278466	17875857	10	G	T	0.661	0.073	0.008	2.3E-21	trans
1986	SLITRK2	Q9H156	SLIT and NTRK-like protein 2	rs10823396	71243323	10	G	C	0.465	0.051	0.007	2.2E-12	trans
1986	SLITRK2	Q9H156	SLIT and NTRK-like protein 2	rs12805381	11276088	11	T	G	0.451	0.083	0.007	3.1E-29	trans
1986	SLITRK2	Q9H156	SLIT and NTRK-like protein 2	rs186021206	7069412	17	A	G	0.006	0.438	0.050	2.3E-18	trans
1986	SLITRK2	Q9H156	SLIT and NTRK-like protein 2	rs6626211	146064128	X	A	G	0.672	0.102	0.006	1.3E-56	cis
1987	SLITRK6	Q9H5Y7	SLIT and NTRK-like protein 6	rs61747728	179526214	1	T	C	0.038	0.143	0.019	5.7E-14	trans
1987	SLITRK6	Q9H5Y7	SLIT and NTRK-like protein 6	rs1260326	27730940	2	C	T	0.609	0.064	0.007	1.9E-17	trans
1987	SLITRK6	Q9H5Y7	SLIT and NTRK-like protein 6	rs13107325	103188709	4	T	C	0.075	0.104	0.014	1.0E-13	trans
1987	SLITRK6	Q9H5Y7	SLIT and NTRK-like protein 6	rs7712631	153609602	5	A	G	0.420	0.087	0.007	6.2E-32	trans
1987	SLITRK6	Q9H5Y7	SLIT and NTRK-like protein 6	rs587600964	136172323	9	C	G	0.880	0.085	0.011	7.5E-14	trans
1987	SLITRK6	Q9H5Y7	SLIT and NTRK-like protein 6	rs36115375	7777799	10	C	A	0.937	0.151	0.015	2.5E-23	trans
1987	SLITRK6	Q9H5Y7	SLIT and NTRK-like protein 6	rs56278466	17875857	10	G	T	0.661	0.066	0.008	1.6E-17	trans
1987	SLITRK6	Q9H5Y7	SLIT and NTRK-like protein 6	rs12863734	86370571	13	G	A	0.983	1.411	0.029	0.0E+00	cis
1988	SLMAP	Q14BN4	Sarcolemmal membrane-associated protein	rs1354034	56849749	3	C	T	0.605	0.071	0.008	9.6E-21	cis
1989	SLURP1	P55000	Secreted Ly-6/uPAR-related protein 1	rs61816761	152285861	1	A	G	0.024	0.239	0.025	1.1E-21	trans
1989	SLURP1	P55000	Secreted Ly-6/uPAR-related protein 1	rs10624245	143815697	8	CTG	C	0.533	0.409	0.007	0.0E+00	cis
1989	SLURP1	P55000	Secreted Ly-6/uPAR-related protein 1	rs2847501	120115709	11	T	C	0.080	0.092	0.013	1.7E-12	trans
1989	SLURP1	P55000	Secreted Ly-6/uPAR-related protein 1	rs10426	51517798	19	A	G	0.215	0.064	0.009	2.7E-13	trans
1990	SMAD1	Q15797	Mothers against decapentaplegic homolog 1	rs1354034	56849749	3	C	T	0.604	0.053	0.008	5.5E-12	trans
1990	SMAD1	Q15797	Mothers against decapentaplegic homolog 1	rs450269	106338463	7	G	C	0.550	0.062	0.008	3.6E-16	trans
1990	SMAD1	Q15797	Mothers against decapentaplegic homolog 1	rs6993770	106581528	8	A	T	0.713	0.074	0.008	1.1E-18	trans
1990	SMAD1	Q15797	Mothers against decapentaplegic homolog 1	rs964184	116648917	11	G	C	0.133	0.080	0.011	7.2E-13	trans
1991	SMAD3	P84022	Mothers against decapentaplegic homolog 3	rs3811444	248039451	1	C	T	0.668	0.070	0.008	3.6E-19	trans
1991	SMAD3	P84022	Mothers against decapentaplegic homolog 3	rs342293	106372219	7	C	G	0.540	0.102	0.007	2.1E-43	trans
1991	SMAD3	P84022	Mothers against decapentaplegic homolog 3	rs34189958	67356481	15	A	C	0.233	0.245	0.009	7.0E-169	cis
1991	SMAD3	P84022	Mothers against decapentaplegic homolog 3	rs3002416	39710195	X	T	C	0.407	0.069	0.006	1.4E-26	trans
1992	SMAD5	Q99717	Mothers against decapentaplegic homolog 5	rs4643790	154709854	4	G	A	0.803	0.214	0.009	6.9E-117	trans
1992	SMAD5	Q99717	Mothers against decapentaplegic homolog 5	rs3129777	32646184	6	C	A	0.785	0.081	0.010	4.7E-17	trans
1993	SMNDC1	O75940	Survival of motor neuron-related-splicing factor 30	rs4632248	54324995	19	T	G	0.210	0.158	0.009	5.6E-70	trans
1994	SMOC1	Q9H4F8	SPARC-related modular calcium-binding protein 1	rs2090034	20372409	2	G	A	0.531	0.057	0.007	6.5E-17	trans
1994	SMOC1	Q9H4F8	SPARC-related modular calcium-binding protein 1	rs35457250	186338564	3	T	C	0.009	0.298	0.036	2.4E-16	trans
1994	SMOC1	Q9H4F8	SPARC-related modular calcium-binding protein 1	rs11751024	32586236	6	A	C	0.398	0.056	0.007	1.2E-15	trans
1994	SMOC1	Q9H4F8	SPARC-related modular calcium-binding protein 1	rs2960834	1202956	7	C	G	0.446	0.053	0.007	2.1E-14	trans
1994	SMOC1	Q9H4F8	SPARC-related modular calcium-binding protein 1	rs705379	94953895	7	A	G	0.477	0.077	0.007	1.0E-28	trans
1994	SMOC1	Q9H4F8	SPARC-related modular calcium-binding protein 1	rs1958078	70354858	14	C	A	0.847	0.367	0.010	5.0E-304	cis
1994	SMOC1	Q9H4F8	SPARC-related modular calcium-binding protein 1	rs28929474	94844947	14	T	C	0.021	0.188	0.025	2.5E-14	trans
1994	SMOC1	Q9H4F8	SPARC-related modular calcium-binding protein 1	rs77542162	67081278	17	G	A	0.023	0.463	0.023	5.0E-91	trans
1994	SMOC1	Q9H4F8	SPARC-related modular calcium-binding protein 1	rs584007	45416478	19	G	A	0.642	0.058	0.007	2.5E-16	trans
1994	SMOC1	Q9H4F8	SPARC-related modular calcium-binding protein 1	rs2267867	43965903	20	A	G	0.757	0.061	0.008	2.4E-14	trans
1994	SMOC1	Q9H4F8	SPARC-related modular calcium-binding protein 1	rs4239651	46340596	20	T	C	0.208	0.071	0.008	4.1E-17	trans
1995	SMOC2	Q9H3U7	SPARC-related modular calcium-binding protein 2	rs56296467	168863807	6	T	C	0.722	0.395	0.008	0.0E+00	cis
1995	SMOC2	Q9H3U7	SPARC-related modular calcium-binding protein 2	rs6984210	22033615	8	C	G	0.946	0.111	0.015	5.0E-14	trans
1995	SMOC2	Q9H3U7	SPARC-related modular calcium-binding protein 2	rs77924615	20392332	16	G	A	0.803	0.057	0.008	8.4E-12	trans
1995	SMOC2	Q9H3U7	SPARC-related modular calcium-binding protein 2	rs7247412	52313868	19	T	C	0.733	0.119	0.008	4.3E-57	trans
1996	SMPD1	P17405	Sphingomyelin phosphodiesterase	rs78444298	184672098	1	G	A	0.981	0.205	0.025	2.3E-16	trans
1996	SMPD1	P17405	Sphingomyelin phosphodiesterase	rs11735092	88226231	4	T	C	0.562	0.049	0.007	1.2E-12	trans
1996	SMPD1	P17405	Sphingomyelin phosphodiesterase	rs114816312	110638824	4	T	C	0.008	0.345	0.039	1.7E-18	trans
1996	SMPD1	P17405	Sphingomyelin phosphodiesterase	rs2389688	119736796	4	A	C	0.558	0.050	0.007	6.9E-13	trans
1996	SMPD1	P17405	Sphingomyelin phosphodiesterase	rs77945361	154254534	4	G	A	0.852	0.080	0.010	2.0E-16	trans
1996	SMPD1	P17405	Sphingomyelin phosphodiesterase	rs11791806	34633529	9	T	C	0.173	0.065	0.009	9.1E-13	trans
1996	SMPD1	P17405	Sphingomyelin phosphodiesterase	rs2862954	101912064	10	T	C	0.498	0.056	0.007	1.8E-16	trans
1996	SMPD1	P17405	Sphingomyelin phosphodiesterase	rs1050239	6415463	11	G	A	0.757	0.438	0.008	0.0E+00	cis
1996	SMPD1	P17405	Sphingomyelin phosphodiesterase	rs150641790	41687844	13	TG	T	0.075	0.120	0.013	8.1E-20	trans
1996	SMPD1	P17405	Sphingomyelin phosphodiesterase	rs28929474	94844947	14	T	C	0.021	0.350	0.024	4.3E-48	trans
1996	SMPD1	P17405	Sphingomyelin phosphodiesterase	rs139974673	44027885	15	C	T	0.024	0.168	0.022	4.1E-14	trans
1996	SMPD1	P17405	Sphingomyelin phosphodiesterase	rs58542926	19379549	19	T	C	0.075	0.157	0.013	2.1E-33	trans
1996	SMPD1	P17405	Sphingomyelin phosphodiesterase	rs429358	45411941	19	T	C	0.845	0.098	0.010	6.1E-25	trans
1996	SMPD1	P17405	Sphingomyelin phosphodiesterase	rs11668882	54675097	19	C	T	0.441	0.059	0.007	2.6E-17	trans
1996	SMPD1	P17405	Sphingomyelin phosphodiesterase	rs738409	44324727	22	G	C	0.217	0.070	0.008	2.2E-17	trans
1997	SMPD3	Q9NY59	Sphingomyelin phosphodiesterase 3	rs139299944	32602665	6	CT	C	0.344	0.079	0.008	1.2E-22	trans
1998	SMPDL3A	Q92484	Acid sphingomyelinase-like phosphodiesterase 3a	rs114165349	27021913	1	C	G	0.023	0.177	0.020	1.2E-18	trans
1998	SMPDL3A	Q92484	Acid sphingomyelinase-like phosphodiesterase 3a	rs687339	135932359	3	T	C	0.771	0.090	0.007	1.5E-35	trans
1998	SMPDL3A	Q92484	Acid sphingomyelinase-like phosphodiesterase 3a	rs114816312	110638824	4	T	C	0.008	0.266	0.035	1.2E-14	trans
1998	SMPDL3A	Q92484	Acid sphingomyelinase-like phosphodiesterase 3a	rs17259257	119778889	4	C	T	0.944	0.100	0.013	3.8E-14	trans
1998	SMPDL3A	Q92484	Acid sphingomyelinase-like phosphodiesterase 3a	rs41280463	154191226	4	G	A	0.832	0.076	0.008	3.6E-21	trans
1998	SMPDL3A	Q92484	Acid sphingomyelinase-like phosphodiesterase 3a	rs2522062	131805416	5	G	A	0.184	0.054	0.008	3.3E-12	trans
1998	SMPDL3A	Q92484	Acid sphingomyelinase-like phosphodiesterase 3a	rs28385609	123122464	6	C	T	0.862	1.017	0.011	0.0E+00	cis
1998	SMPDL3A	Q92484	Acid sphingomyelinase-like phosphodiesterase 3a	rs9414801	65149089	10	G	A	0.467	0.057	0.006	1.0E-20	trans
1998	SMPDL3A	Q92484	Acid sphingomyelinase-like phosphodiesterase 3a	rs2862954	101912064	10	T	C	0.498	0.063	0.006	7.5E-26	trans
1998	SMPDL3A	Q92484	Acid sphingomyelinase-like phosphodiesterase 3a	rs10787429	113949664	10	T	C	0.273	0.058	0.007	1.3E-17	trans
1998	SMPDL3A	Q92484	Acid sphingomyelinase-like phosphodiesterase 3a	rs150641790	41687844	13	TG	T	0.075	0.124	0.012	6.6E-27	trans
1998	SMPDL3A	Q92484	Acid sphingomyelinase-like phosphodiesterase 3a	rs6571912	39733360	14	C	G	0.460	0.063	0.006	2.9E-25	trans

1998	SMPDL3A	Q92484	Acid sphingomyelinase-like phosphodiesterase 3a	rs145078947	93652974	14	T	G	0.003	0.805	0.060	2.7E-41	trans
1998	SMPDL3A	Q92484	Acid sphingomyelinase-like phosphodiesterase 3a	rs147233090	44028047	15	T	C	0.024	0.171	0.020	9.0E-18	trans
1998	SMPDL3A	Q92484	Acid sphingomyelinase-like phosphodiesterase 3a	rs339969	60883281	15	A	C	0.617	0.050	0.006	1.3E-15	trans
1998	SMPDL3A	Q92484	Acid sphingomyelinase-like phosphodiesterase 3a	rs58542926	19379549	19	T	C	0.076	0.189	0.011	3.0E-61	trans
1998	SMPDL3A	Q92484	Acid sphingomyelinase-like phosphodiesterase 3a	rs429358	45411941	19	T	C	0.845	0.103	0.008	6.5E-35	trans
1998	SMPDL3A	Q92484	Acid sphingomyelinase-like phosphodiesterase 3a	rs626283	54677001	19	C	G	0.442	0.057	0.006	9.2E-21	trans
1998	SMPDL3A	Q92484	Acid sphingomyelinase-like phosphodiesterase 3a	rs738408	44324730	22	T	C	0.217	0.070	0.007	7.5E-22	trans
1999	SMTN	P53814	Smoothelin	rs1354034	56849749	3	C	T	0.604	0.072	0.007	8.1E-22	trans
1999	SMTN	P53814	Smoothelin	rs114694170	88180196	5	C	T	0.060	0.151	0.016	5.3E-22	trans
1999	SMTN	P53814	Smoothelin	rs342293	106372219	7	C	G	0.540	0.050	0.007	7.6E-12	trans
1999	SMTN	P53814	Smoothelin	rs12292693	64936719	11	A	C	0.740	0.075	0.008	8.6E-19	trans
2000	SNAP23	O00161	Synaptosomal-associated protein 23	rs1354034	56849749	3	C	T	0.604	0.111	0.008	9.3E-48	trans
2000	SNAP23	O00161	Synaptosomal-associated protein 23	rs17622656	131820997	5	G	A	0.612	0.052	0.008	1.7E-11	trans
2000	SNAP23	O00161	Synaptosomal-associated protein 23	rs6993770	106581528	8	A	T	0.713	0.059	0.008	1.1E-12	trans
2000	SNAP23	O00161	Synaptosomal-associated protein 23	rs10820606	99192919	9	A	C	0.770	0.084	0.009	2.2E-20	trans
2000	SNAP23	O00161	Synaptosomal-associated protein 23	rs11502185	180258	11	C	T	0.259	0.084	0.010	1.8E-18	trans
2001	SNAP25	P60880	Synaptosomal-associated protein 25	rs10908505	156468243	1	A	T	0.652	0.056	0.008	1.3E-13	trans
2001	SNAP25	P60880	Synaptosomal-associated protein 25	rs342293	106372219	7	C	G	0.541	0.190	0.007	1.1E-151	trans
2001	SNAP25	P60880	Synaptosomal-associated protein 25	rs3780420	100677166	9	C	T	0.364	0.059	0.008	5.7E-15	trans
2001	SNAP25	P60880	Synaptosomal-associated protein 25	rs60757417	135864436	9	G	C	0.061	0.111	0.015	3.2E-13	trans
2001	SNAP25	P60880	Synaptosomal-associated protein 25	rs7896518	65104500	10	G	A	0.424	0.059	0.007	2.0E-15	trans
2001	SNAP25	P60880	Synaptosomal-associated protein 25	rs1477123	4560627	16	T	A	0.274	0.069	0.008	1.8E-17	trans
2001	SNAP25	P60880	Synaptosomal-associated protein 25	rs429358	45411941	19	C	T	0.156	0.625	0.010	0.0E+00	trans
2001	SNAP25	P60880	Synaptosomal-associated protein 25	rs362562	10243186	20	G	A	0.586	0.103	0.008	1.6E-42	cis
2002	SNAP29	O95721	Synaptosomal-associated protein 29	rs1354034	56849749	3	C	T	0.604	0.078	0.008	3.5E-24	trans
2002	SNAP29	O95721	Synaptosomal-associated protein 29	rs11242109	131677047	5	G	T	0.519	0.058	0.008	1.4E-14	trans
2002	SNAP29	O95721	Synaptosomal-associated protein 29	rs6993770	106581528	8	A	T	0.714	0.078	0.008	7.6E-21	trans
2002	SNAP29	O95721	Synaptosomal-associated protein 29	rs892090	55539072	19	G	T	0.834	0.078	0.010	2.4E-14	trans
2002	SNAP29	O95721	Synaptosomal-associated protein 29	rs5759874	21017148	22	A	G	0.914	0.130	0.014	2.0E-21	cis
2003	SNCA	P37840	Alpha-synuclein	rs3811444	248039451	1	C	T	0.668	0.070	0.008	4.1E-18	trans
2003	SNCA	P37840	Alpha-synuclein	rs1354034	56849749	3	C	T	0.604	0.078	0.008	2.2E-24	trans
2003	SNCA	P37840	Alpha-synuclein	rs55730499	161005610	6	T	C	0.082	0.103	0.014	6.7E-14	trans
2003	SNCA	P37840	Alpha-synuclein	rs342296	106372903	7	G	A	0.543	0.064	0.008	1.6E-17	trans
2003	SNCA	P37840	Alpha-synuclein	rs2393967	65133156	10	C	A	0.314	0.071	0.008	5.0E-18	trans
2004	SNCG	O76070	Gamma-synuclein	rs873110	88724619	10	C	T	0.755	1.061	0.009	0.0E+00	cis
2005	SNED1	Q8TER0	Sushi, nidogen and EGF-like domain-containing protein 1	rs2074822	241974289	2	G	A	0.507	0.125	0.008	7.8E-60	cis
2006	SNX15	Q9NRS6	Sorting nexin-15	rs1354034	56849749	3	C	T	0.604	0.056	0.007	1.0E-14	trans
2006	SNX15	Q9NRS6	Sorting nexin-15	rs6993770	106581528	8	A	T	0.713	0.060	0.008	1.5E-14	trans
2006	SNX15	Q9NRS6	Sorting nexin-15	rs2393967	65133156	10	C	A	0.314	0.052	0.008	1.3E-11	trans
2006	SNX15	Q9NRS6	Sorting nexin-15	rs2670910	64807136	11	G	A	0.914	0.781	0.013	0.0E+00	cis
2007	SNX18	Q96RF0	Sorting nexin-18	rs2548611	53818895	5	T	C	0.188	0.096	0.010	1.8E-23	cis
2008	SNX2	O60749	Sorting nexin-2	rs27740	122204772	5	T	A	0.553	0.096	0.008	2.4E-36	cis
2008	SNX2	O60749	Sorting nexin-2	rs28891437	32641342	6	G	A	0.788	0.080	0.010	1.3E-15	trans
2008	SNX2	O60749	Sorting nexin-2	rs55648536	107097210	14	T	G	0.705	0.081	0.009	2.1E-20	trans
2009	SNX5	Q9Y5X3	Sorting nexin-5	rs1354034	56849749	3	C	T	0.605	0.055	0.008	1.8E-12	trans
2009	SNX5	Q9Y5X3	Sorting nexin-5	rs13172043	122184759	5	A	G	0.556	0.141	0.008	2.1E-74	trans
2010	SNX9	Q9Y5X1	Sorting nexin-9	rs688181	158092638	6	T	C	0.283	0.089	0.008	4.4E-27	cis
2010	SNX9	Q9Y5X1	Sorting nexin-9	rs342298	106373646	7	C	T	0.545	0.073	0.007	2.2E-22	trans
2011	SOD1	P00441	Superoxide dismutase [Cu-Zn]	rs6993770	106581528	8	A	T	0.714	0.109	0.008	4.2E-40	trans
2011	SOD1	P00441	Superoxide dismutase [Cu-Zn]	rs7282332	33126239	21	A	G	0.103	0.098	0.012	2.0E-15	cis
2012	SOD2	P04179	Superoxide dismutase [Mn], mitochondrial	rs1570805	156264648	1	T	C	0.112	0.083	0.012	5.0E-13	trans
2012	SOD2	P04179	Superoxide dismutase [Mn], mitochondrial	rs915651	31749184	6	G	A	0.870	0.073	0.011	1.4E-11	trans
2012	SOD2	P04179	Superoxide dismutase [Mn], mitochondrial	rs5746105	160112638	6	G	A	0.299	0.158	0.008	2.2E-87	cis
2012	SOD2	P04179	Superoxide dismutase [Mn], mitochondrial	rs4541868	106590705	8	C	A	0.726	0.057	0.008	4.7E-12	trans
2012	SOD2	P04179	Superoxide dismutase [Mn], mitochondrial	rs2072694	1505429	16	G	C	0.518	0.056	0.007	1.4E-14	trans
2012	SOD2	P04179	Superoxide dismutase [Mn], mitochondrial	rs12975366	54759361	19	T	C	0.605	0.158	0.007	2.4E-99	trans
2013	SOD3	P08294	Extracellular superoxide dismutase	rs3796735	8599432	4	C	T	0.334	0.114	0.007	3.7E-54	trans
2013	SOD3	P08294	Extracellular superoxide dismutase	rs1799895	24801834	4	G	C	0.012	2.301	0.032	0.0E+00	cis
2013	SOD3	P08294	Extracellular superoxide dismutase	rs13107325	103188709	4	T	C	0.075	0.159	0.013	1.7E-33	trans
2013	SOD3	P08294	Extracellular superoxide dismutase	rs56278466	17875857	10	G	T	0.661	0.061	0.007	7.9E-18	trans
2013	SOD3	P08294	Extracellular superoxide dismutase	rs184458383	75249552	16	C	T	0.924	0.126	0.013	2.2E-23	trans
2013	SOD3	P08294	Extracellular superoxide dismutase	rs77542162	67081278	17	G	A	0.023	0.167	0.022	8.7E-14	trans
2014	SORCS2	Q96PQ0	VPS10 domain-containing receptor SorCS2	rs4234798	7219933	4	T	G	0.383	0.457	0.008	0.0E+00	cis
2014	SORCS2	Q96PQ0	VPS10 domain-containing receptor SorCS2	rs4253238	187148387	4	C	T	0.487	0.074	0.007	2.8E-24	trans
2014	SORCS2	Q96PQ0	VPS10 domain-containing receptor SorCS2	rs7203642	20367130	16	A	G	0.808	0.065	0.009	5.6E-14	trans
2015	SORD	Q00796	Sorbitol dehydrogenase	rs56060952	45328981	15	C	T	0.128	0.205	0.011	1.1E-76	cis
2015	SORD	Q00796	Sorbitol dehydrogenase	rs3747207	44324855	22	A	G	0.216	0.072	0.009	8.9E-16	trans
2016	SORT1	Q99523	Sortilin	rs61394658	109873290	1	G	A	0.773	0.187	0.009	1.8E-97	cis
2016	SORT1	Q99523	Sortilin	rs13303	52558008	3	C	T	0.560	0.066	0.007	7.4E-19	trans
2016	SORT1	Q99523	Sortilin	rs61244449	58359023	3	TCTC	T	0.371	0.064	0.008	8.0E-17	trans
2016	SORT1	Q99523	Sortilin	rs139596721	154461313	4	CT	C	0.032	0.160	0.021	2.6E-14	trans
2016	SORT1	Q99523	Sortilin	rs4703589	72097351	5	C	T	0.530	0.050	0.007	1.1E-11	trans
2016	SORT1	Q99523	Sortilin	rs4098923	31193756	6	A	G	0.552	0.055	0.008	1.5E-12	trans
2016	SORT1	Q99523	Sortilin	rs139141690	101499930	7	A	G	0.005	0.650	0.054	4.2E-33	trans
2016	SORT1	Q99523	Sortilin	rs6993770	106581528	8	A	T	0.713	0.179	0.008	4.1E-106	trans
2016	SORT1	Q99523	Sortilin	rs385893	4763176	9	C	T	0.524	0.057	0.007	7.6E-15	trans
2016	SORT1	Q99523	Sortilin	rs141129381	100691900	9	TG	T	0.363	0.077	0.008	1.3E-23	trans
2016	SORT1	Q99523	Sortilin	rs2519093	136141870	9	C	T	0.816	0.089	0.010	1.6E-20	trans
2016	SORT1	Q99523	Sortilin	rs17490626	71218646	10	C	G	0.130	0.166	0.011	3.3E-51	trans
2016	SORT1	Q99523	Sortilin	rs892090	55539072	19	G	T	0.834	0.127	0.010	1.6E-37	trans
2017	SOST	Q9BQB4	Sclerostin	rs4973180	230101512	2	T	C	0.822	0.080	0.009	1.6E-18	trans
2017	SOST	Q9BQB4	Sclerostin	rs66642874	44761054	6	T	C	0.776	0.066	0.008	1.3E-15	trans
2017	SOST	Q9BQB4	Sclerostin	rs34115816	133369102	6	A	G	0.321	0.082	0.007	8.3E-29	trans
2017	SOST	Q9BQB4	Sclerostin	rs61591132	150952770	7	A	G	0.113	0.089	0.011	3.4E-16	trans
2017	SOST	Q9BQB4	Sclerostin	rs11995824	120012700	8	C	G	0.456	0.104	0.007	4.2E-51	trans
2017	SOST	Q9BQB4	Sclerostin	rs505429	86886650	11	G	A	0.314	0.055	0.007	7.3E-14	trans
2017	SOST	Q9BQB4	Sclerostin	rs34001253	43083293	13	G	A	0.033	0.189	0.020	4.5E-22	trans
2017	SOST	Q9BQB4	Sclerostin	rs1513671	41807310	17	G	C	0.361	0.061	0.007	2.1E-17	cis
2017	SOST	Q9BQB4	Sclerostin	rs884205	60054857	18	C	A	0.747	0.072	0.008	2.9E-19	trans
2017	SOST	Q9BQB4	Sclerostin	rs34952318	11177055	20	G	A	0.950	0.135	0.016	1.9E-17	trans
2017	SOST	Q9BQB4	Sclerostin	rs6127099	52731402	20	A	T	0.722	0.056	0.008	1.5E-12	trans
2018	SPACA5_SPACA5B	Q96QH8	Sperm acrosome-associated protein 5	rs2152823	47927449	X	A	G	0.533	0.120	0.006	3.2E-83	cis
2019	SPAG1	Q07617	Sperm-associated antigen 1	rs2439461	101223778	8	G	A	0.432	0.715	0.008	0.0E+00	cis
2020	SPARC	P09486	SPARC	rs13412535	224874874	2	G	A	0.769	0.065	0.009	2.3E-13	trans
2020	SPARC	P09486	SPARC	rs7618405	18250509	3	C	A	0.795	0.063	0.009	1.1E-11	trans
2020	SPARC	P09486	SPARC	rs1354034	56849749	3	T	C	0.396	0.103	0.008	1.3E-41	trans
2020	SPARC	P09486	SPARC	rs6961069	80218961	7	T	C	0.402	0.066	0.008	9.4E-18	trans
2020	SPARC	P09486	SPARC	rs6993770	106581528	8	A	T	0.714	0.116	0.008	1.4E-44	trans
2020	SPARC	P09486	SPARC	rs12767683	104316581	10	A	C	0.325	0.096	0.008	2.9E-33	trans
2020	SPARC	P09486	SPARC	rs59001897	65160392	15	A	T	0.175	0.072	0.010	5.9E-13	trans
2020	SPARC	P09486	SPARC	rs28734133	81874035	16	G	A	0.180	0.083	0.010	1.1E-17	trans
2020	SPARC	P09486	SPARC	rs892090	55539072	19	G	T	0.834	0.162	0.010	2.6E-57	trans
2020	SPARC	P09486	SPARC	rs3790176	19261922	20	A	G	0.343	0.054	0.008	5.4E-12	trans
2021	SPARCL1	Q14515	SPARC-like protein 1	rs56273319	88413024	4	G	A	0.353	0.714	0.008	0.0E+00	cis
2021	SPARCL1	Q14515	SPARC-like protein 1	rs12291877	6451455	11	T	C	0.247	0.050	0.007	8.4E-12	trans
2021	SPARCL1	Q14515	SPARC-like protein 1	rs217184	72105965	16	T	C	0.804	0.150	0.008	2.1E-78	trans
2021	SPARCL1	Q14515	SPARC-like protein 1	rs77542162	67081278	17	G	A	0.023	0.216	0.021	2.0E-24	trans
2021	SPARCL1	Q14515	SPARC-like protein 1	rs55639531	394341	18	G	C	0.286	0.047	0.007	1.6E-11	trans
2022	SPART	Q8N0X7	Spartin	rs342299	106373718	7	C	T	0.545	0.053	0.008	2.7E-12	trans
2022	SPART	Q8N0X7	Spartin	rs6993770	106581528	8	A	T	0.713	0.078	0.008	2.7E-21	trans
2023	SPESP1	Q6UW49	Sperm equatorial segment protein 1	rs6433469	152366084	2	G	A	0.375	0.089	0.007	5.9E-36	trans
2023	SPESP1	Q6UW49	Sperm equatorial segment protein 1	rs3750228	70980738	8	C	T	0.124	0.090	0.010	5.3E-18	trans
2023	SPESP1	Q6UW49	Sperm equatorial segment protein 1	rs8038764	41907905	15	A	C	0.533	0.105	0.007	2.0E-51	trans
2023	SPESP1	Q6UW49	Sperm equatorial segment protein 1	rs72752337	69198385	15	C	T	0.007	1.354	0.043	2.0E-219	cis
2023	SPESP1	Q6UW49	Sperm equatorial segment protein 1	rs12609754	21155558	19	C	T	0.573	0.061	0.007	1.5E-18	trans
2024	SPINK1	P00995	Serine protease inhibitor Kazal-type 1	rs7725017	147217823	5	C	A	0.396	0.405	0.008	0.0E+00	cis
2024	SPINK1	P00995	Serine protease inhibitor Kazal-type 1	rs77924615	20392332	16	G	A	0.803	0.065	0.009	6.9E-13	trans
2025	SPINK2	P20155	Serine protease inhibitor Kazal-type 2	rs188468174	25291697	1	T	C	0.014	0.370	0.026	8.9E-45	trans
2025	SPINK2	P20155	Serine protease inhibitor Kazal-type 2	rs28656303	57689945	4	G	A	0.020	1.460	0.024	0.0E+00	cis
2025	SPINK2	P20155	Serine protease inhibitor Kazal-type 2	rs2522050	131796609	5	T	C	0.190	0.070	0.008	3.1E-19	trans
2025	SPINK2	P20155	Serine protease inhibitor Kazal-type 2	rs56353819	91012867	6	C	T	0.649	0.047	0.006	3.1E-13	trans
2025	SPINK2	P20155	Serine protease inhibitor Kazal-type 2	rs78740585	150944302	7	A	G	0.114	0.168	0.010	5.7E-68	trans
2025	SPINK2	P20155	Serine protease inhibitor Kazal-type 2	rs10982164	117091067	9	A	G	0.067	0.328	0.013	7.2E-137	trans
2025	SPINK2	P20155	Serine protease inhibitor Kazal-type 2	rs28449958	28528781	16	A	G	0.344	0.048	0.006	8.5E-14	trans
2025	SPINK2	P20155	Serine protease inhibitor Kazal-type 2	rs77542162	67081278	17	G	A	0.023	0.247	0.021	2.1E-33	trans
2025	SPINK2	P20155	Serine protease inhibitor Kazal-type 2	rs7247412	52313868	19	T	C	0.734	0.048	0.007	7.8E-12	trans
2026	SPINK4	O60575	Serine protease inhibitor Kazal-type 4	rs808096	42498607	3	G	T	0.474	0.056	0.006	7.2E-21	trans
2026	SPINK4	O60575	Serine protease inhibitor Kazal-type 4	rs369940	40726247	5	A	T	0.321	0.068	0.006	1.3E-25	trans
2026	SPINK4	O60575	Serine protease inhibitor Kazal-type 4	rs4879679	33234019	9	C	T	0.157	1.114	0.010	0.0E+00	cis
2026	SPINK4	O60575	Serine protease inhibitor Kazal-type 4	rs12804877	1085189	11	G	A	0.224	0.052	0.007	8.8E-13	trans
2026	SPINK4	O60575	Serine protease inhibitor Kazal-type 4	rs28529068	19673005	18	G	A	0.243	0.115	0.007	2.8E-60	trans
2026	SPINK4	O60575	Serine protease inhibitor Kazal-type 4	rs2834350	35397128	21	C	T	0.623	0.045	0.006	4.1E-13	trans
2027	SPINK5	Q9NQ38	Serine protease inhibitor Kazal-type 5	rs1260326	27730940	2	C	T	0.608	0.063	0.007	1.1E-18	trans
2027	SPINK5	Q9NQ38	Serine protease inhibitor Kazal-type 5	rs11384422	60499861	2	AG	A	0.583	0.051	0.007	9.5E-13	trans
2027	SPINK5	Q9NQ38	Serine protease inhibitor Kazal-type 5	rs6883868	147500883	5	T	C	0.624	0.410	0.008	0.0E+00	cis
2027	SPINK5	Q9NQ38	Serine protease inhibitor Kazal-type 5	rs587600964	136172323	9	C	G	0.881	0.094	0.011	9.2E-18	trans
2027	SPINK5	Q9NQ38	Serine protease inhibitor Kazal-type 5	rs11220477	126275402	11	C	T	0.958	0.133	0.017	2.8E-14	trans
2027	SPINK5	Q9NQ38	Serine protease inhibitor Kazal-type 5	rs186021206	7069412	17	A	G	0.006	0.865	0.048	1.2E-72	trans
2027	SPINK5	Q9NQ38	Serine protease inhibitor Kazal-type 5	rs681343	49206462	19	T	C	0.510	0.198	0.007	1.3E-171	trans
2028	SPINK6	Q6UWN8	Serine protease inhibitor Kazal-type 6	rs12717962	147566124	5	A	G	0.446	0.359	0.007	0.0E+00	cis
2029	SPINK8	P0C7L1	Serine protease inhibitor Kazal-type 8	rs78159520	48349023	3	T	C	0.686	0.461	0.008	0.0E+00	cis
2030	SPINT1	O43278	Kunitz-type protease inhibitor 1	rs4671378	60494312	2	C	T	0.559	0.063	0.007	1.9E-17	trans
2030	SPINT1	O43278	Kunitz-type protease inhibitor 1	rs13080057	98502553	3	A	G	0.086	0.130	0.013	1.1E-22	trans

2030	SPINT1	O43278	Kunitz-type protease inhibitor 1	rs538174489	32508201	6	C	T	0.692	0.079	0.008	5.5E-23	trans
2030	SPINT1	O43278	Kunitz-type protease inhibitor 1	rs56278466	17875857	10	G	T	0.661	0.089	0.008	2.7E-30	trans
2030	SPINT1	O43278	Kunitz-type protease inhibitor 1	rs17658212	41145919	15	C	T	0.940	0.676	0.016	0.0E+00	cis
2030	SPINT1	O43278	Kunitz-type protease inhibitor 1	rs186021206	7069412	17	A	G	0.006	0.840	0.051	1.1E-61	trans
2030	SPINT1	O43278	Kunitz-type protease inhibitor 1	rs708686	5840619	19	T	C	0.268	0.104	0.008	2.4E-35	trans
2030	SPINT1	O43278	Kunitz-type protease inhibitor 1	rs584768	49213284	19	A	G	0.534	0.052	0.007	2.4E-12	trans
2031	SPINT2	O43291	Kunitz-type protease inhibitor 2	rs13412535	224874874	2	G	A	0.769	0.069	0.009	2.1E-15	trans
2031	SPINT2	O43291	Kunitz-type protease inhibitor 2	rs1354034	56849749	3	T	C	0.396	0.095	0.007	5.1E-37	trans
2031	SPINT2	O43291	Kunitz-type protease inhibitor 2	rs5030062	186454180	3	A	C	0.627	0.068	0.008	7.8E-19	trans
2031	SPINT2	O43291	Kunitz-type protease inhibitor 2	rs10016018	102794621	4	A	T	0.603	0.060	0.008	2.0E-15	trans
2031	SPINT2	O43291	Kunitz-type protease inhibitor 2	rs139596721	154461313	4	CT	C	0.032	0.145	0.021	6.7E-12	trans
2031	SPINT2	O43291	Kunitz-type protease inhibitor 2	rs66530140	187161211	4	T	C	0.486	0.153	0.007	1.7E-94	trans
2031	SPINT2	O43291	Kunitz-type protease inhibitor 2	rs10058074	131686146	5	G	A	0.524	0.056	0.007	1.9E-14	trans
2031	SPINT2	O43291	Kunitz-type protease inhibitor 2	rs2545801	176841339	5	T	C	0.256	0.072	0.008	6.8E-18	trans
2031	SPINT2	O43291	Kunitz-type protease inhibitor 2	rs35173808	31326148	6	C	A	0.903	0.098	0.012	3.6E-15	trans
2031	SPINT2	O43291	Kunitz-type protease inhibitor 2	rs6961069	80218961	7	T	C	0.403	0.071	0.008	3.0E-21	trans
2031	SPINT2	O43291	Kunitz-type protease inhibitor 2	rs139141690	101499930	7	A	G	0.005	0.439	0.054	4.2E-16	trans
2031	SPINT2	O43291	Kunitz-type protease inhibitor 2	rs6993770	106581528	8	A	T	0.713	0.127	0.008	2.5E-55	trans
2031	SPINT2	O43291	Kunitz-type protease inhibitor 2	rs10761737	65052205	10	C	T	0.413	0.091	0.007	3.4E-34	trans
2031	SPINT2	O43291	Kunitz-type protease inhibitor 2	rs11591571	104342804	10	A	G	0.341	0.053	0.008	7.8E-12	trans
2031	SPINT2	O43291	Kunitz-type protease inhibitor 2	rs12445050	81870969	16	T	C	0.138	0.090	0.011	2.3E-17	trans
2031	SPINT2	O43291	Kunitz-type protease inhibitor 2	rs45437199	38795250	19	G	A	0.817	0.191	0.009	5.7E-90	cis
2031	SPINT2	O43291	Kunitz-type protease inhibitor 2	rs892090	55539072	19	G	T	0.834	0.133	0.010	9.3E-41	trans
2032	SPOCK1	Q08629	Testican-1	rs34546909	47963691	1	GT	G	0.804	0.149	0.009	2.1E-58	trans
2032	SPOCK1	Q08629	Testican-1	rs4655581	68720435	1	C	T	0.906	0.094	0.013	8.1E-14	trans
2032	SPOCK1	Q08629	Testican-1	rs77683480	18680741	2	G	A	0.689	0.055	0.008	6.3E-12	trans
2032	SPOCK1	Q08629	Testican-1	rs62155324	67768340	2	C	A	0.965	0.186	0.020	4.6E-20	trans
2032	SPOCK1	Q08629	Testican-1	rs4835737	136895064	5	A	G	0.635	0.131	0.008	4.5E-66	cis
2032	SPOCK1	Q08629	Testican-1	rs7756288	15110712	6	T	C	0.744	0.098	0.008	5.4E-31	trans
2032	SPOCK1	Q08629	Testican-1	rs3129777	32646184	6	C	A	0.785	0.090	0.010	7.8E-21	trans
2032	SPOCK1	Q08629	Testican-1	rs2505507	43644824	10	T	C	0.275	0.061	0.008	1.6E-13	trans
2032	SPOCK1	Q08629	Testican-1	rs703987	80939219	10	G	C	0.389	0.056	0.008	6.7E-14	trans
2032	SPOCK1	Q08629	Testican-1	rs149440923	71708649	11	G	A	0.015	0.227	0.030	4.0E-14	trans
2032	SPOCK1	Q08629	Testican-1	rs3916033	44889703	17	T	C	0.555	0.052	0.007	1.7E-12	trans
2032	SPOCK1	Q08629	Testican-1	rs5968946	85826601	X	A	G	0.514	0.047	0.006	4.7E-15	trans
2032	SPOCK1	Q08629	Testican-1	rs4829460	112144627	X	C	T	0.776	0.055	0.007	2.8E-14	trans
2033	SPON1	Q9HCB6	Spondin-1	rs1270942	31918860	6	A	G	0.868	0.097	0.010	1.6E-21	trans
2033	SPON1	Q9HCB6	Spondin-1	rs8176741	136131461	9	A	G	0.061	0.159	0.014	2.5E-28	trans
2033	SPON1	Q9HCB6	Spondin-1	rs10832164	14048480	11	C	T	0.480	0.291	0.007	0.0E+00	cis
2033	SPON1	Q9HCB6	Spondin-1	rs4939113	56986252	11	G	A	0.197	0.076	0.009	2.2E-17	trans
2033	SPON1	Q9HCB6	Spondin-1	rs77542162	67081278	17	G	A	0.023	0.276	0.023	5.4E-33	trans
2033	SPON1	Q9HCB6	Spondin-1	rs516316	49206145	19	G	C	0.490	0.074	0.007	6.1E-27	trans
2034	SPON2	Q9BUD6	Spondin-2	rs28855974	1114909	4	G	A	0.118	0.315	0.012	4.6E-163	cis
2035	SPP1	P10451	Osteopontin	rs35085476	159550104	1	T	C	0.977	0.167	0.024	3.6E-12	trans
2035	SPP1	P10451	Osteopontin	rs13303	52558008	3	C	T	0.560	0.050	0.007	4.7E-12	trans
2035	SPP1	P10451	Osteopontin	rs56254643	88858885	4	T	C	0.808	0.114	0.009	7.3E-36	cis
2035	SPP1	P10451	Osteopontin	rs11995824	120012700	8	G	C	0.543	0.063	0.007	2.7E-18	trans
2035	SPP1	P10451	Osteopontin	rs117068593	93118229	14	C	T	0.813	0.086	0.009	1.2E-20	trans
2035	SPP1	P10451	Osteopontin	rs738408	44324730	22	T	C	0.217	0.063	0.009	5.8E-13	trans
2036	SPRED2	Q7Z698	Sprouty-related, EVH1 domain-containing protein 2	rs7569084	65656969	2	C	T	0.414	0.072	0.008	7.9E-21	cis
2037	SPRING1	Q9H741	SREBP regulating gene protein	rs71534539	32512754	6	G	A	0.536	0.071	0.009	1.2E-16	trans
2038	SPRR1B	P22528	Cornifin-B	rs9272544	32606878	6	G	A	0.505	0.055	0.008	8.4E-13	trans
2039	SPRR3	Q9UBC9	Small proline-rich protein 3	rs61811427	152968419	1	T	C	0.551	0.240	0.007	3.6E-254	cis
2039	SPRR3	Q9UBC9	Small proline-rich protein 3	rs35887622	20763620	13	G	A	0.015	0.302	0.028	1.5E-26	trans
2039	SPRR3	Q9UBC9	Small proline-rich protein 3	rs12970138	55680818	18	T	G	0.408	0.052	0.007	1.1E-13	trans
2040	SPRY2	O43597	Protein sprouty homolog 2	rs1354034	56849749	3	C	T	0.604	0.100	0.008	2.6E-38	trans
2040	SPRY2	O43597	Protein sprouty homolog 2	rs6866614	131787137	5	G	A	0.574	0.052	0.008	1.7E-11	trans
2040	SPRY2	O43597	Protein sprouty homolog 2	rs342298	106373646	7	C	T	0.546	0.067	0.008	1.5E-18	trans
2040	SPRY2	O43597	Protein sprouty homolog 2	rs6993770	106581528	8	A	T	0.713	0.080	0.008	6.8E-22	trans
2040	SPRY2	O43597	Protein sprouty homolog 2	rs11502185	180258	11	C	T	0.259	0.072	0.010	1.5E-13	trans
2040	SPRY2	O43597	Protein sprouty homolog 2	rs504122	80911525	13	G	A	0.630	0.082	0.008	1.4E-25	cis
2041	SPTLC1	O15269	Serine palmitoyltransferase 1	rs45461899	94830356	9	C	A	0.976	0.550	0.025	7.9E-108	cis
2041	SPTLC1	O15269	Serine palmitoyltransferase 1	rs2272587	78023519	14	C	G	0.422	0.078	0.008	3.3E-24	trans
2042	SRC	P12931	Proto-oncogene tyrosine-protein kinase Src	rs17622656	131820997	5	G	A	0.612	0.054	0.008	2.7E-12	trans
2043	SRP14	P37108	Signal recognition particle 14 kDa protein	rs1354034	56849749	3	C	T	0.605	0.108	0.008	5.0E-47	trans
2044	SRPK2	P78362	SRSF protein kinase 2	rs1354034	56849749	3	C	T	0.604	0.108	0.008	8.6E-46	trans
2044	SRPK2	P78362	SRSF protein kinase 2	rs342298	106373646	7	C	T	0.545	0.053	0.007	7.5E-13	trans
2044	SRPK2	P78362	SRSF protein kinase 2	rs7080386	65048306	10	A	C	0.412	0.064	0.008	3.6E-17	trans
2044	SRPK2	P78362	SRSF protein kinase 2	rs11502185	180258	11	C	T	0.260	0.065	0.010	7.2E-12	trans
2045	SRPX	P78539	Sushi repeat-containing protein SRPX	rs28929474	94844947	14	T	C	0.021	0.209	0.024	9.9E-18	trans
2045	SRPX	P78539	Sushi repeat-containing protein SRPX	rs77542162	67081278	17	G	A	0.023	0.434	0.023	2.4E-77	trans
2045	SRPX	P78539	Sushi repeat-containing protein SRPX	rs35318931	38009121	X	G	A	0.912	0.988	0.010	0.0E+00	cis
2046	SSC4D	Q8WTU2	Scavenger receptor cysteine-rich domain-containing group B protein	rs12140070	28335581	1	A	G	0.692	0.154	0.007	5.2E-95	trans
2046	SSC4D	Q8WTU2	Scavenger receptor cysteine-rich domain-containing group B protein	rs469773	91530259	1	C	T	0.803	0.070	0.009	4.8E-16	trans
2046	SSC4D	Q8WTU2	Scavenger receptor cysteine-rich domain-containing group B protein	rs12540573	76039013	7	C	A	0.062	0.687	0.015	0.0E+00	cis
2046	SSC4D	Q8WTU2	Scavenger receptor cysteine-rich domain-containing group B protein	rs112635299	94838142	14	T	G	0.022	0.184	0.024	5.0E-15	trans
2046	SSC4D	Q8WTU2	Scavenger receptor cysteine-rich domain-containing group B protein	rs2498786	105262368	14	C	G	0.381	0.103	0.007	8.2E-48	trans
2046	SSC4D	Q8WTU2	Scavenger receptor cysteine-rich domain-containing group B protein	rs339969	60883281	15	A	C	0.617	0.058	0.007	1.4E-16	trans
2046	SSC4D	Q8WTU2	Scavenger receptor cysteine-rich domain-containing group B protein	rs4803852	46238926	19	T	G	0.823	0.065	0.009	3.8E-13	trans
2046	SSC4D	Q8WTU2	Scavenger receptor cysteine-rich domain-containing group B protein	rs1800961	43042364	20	C	T	0.968	0.157	0.019	3.4E-16	trans
2046	SSC4D	Q8WTU2	Scavenger receptor cysteine-rich domain-containing group B protein	rs2739344	24298293	22	A	C	0.420	0.055	0.007	1.8E-15	trans
2047	SSC5D	A1L4H1	Soluble scavenger receptor cysteine-rich domain-containing protein SSC5D	rs13107325	103188709	4	T	C	0.075	0.150	0.013	1.8E-30	trans
2047	SSC5D	A1L4H1	Soluble scavenger receptor cysteine-rich domain-containing protein SSC5D	rs2142306	134470631	8	C	T	0.412	0.090	0.007	7.1E-38	trans
2047	SSC5D	A1L4H1	Soluble scavenger receptor cysteine-rich domain-containing protein SSC5D	rs7358384	11641024	11	A	T	0.383	0.107	0.007	2.4E-51	trans
2047	SSC5D	A1L4H1	Soluble scavenger receptor cysteine-rich domain-containing protein SSC5D	rs964184	116648917	11	G	C	0.133	0.075	0.010	1.6E-13	trans
2047	SSC5D	A1L4H1	Soluble scavenger receptor cysteine-rich domain-containing protein SSC5D	rs200489612	7106378	17	A	G	0.005	0.685	0.052	2.5E-40	trans
2047	SSC5D	A1L4H1	Soluble scavenger receptor cysteine-rich domain-containing protein SSC5D	rs55799523	55999545	19	A	C	0.265	0.539	0.008	0.0E+00	cis
2047	SSC5D	A1L4H1	Soluble scavenger receptor cysteine-rich domain-containing protein SSC5D	rs181210490	3695977	20	A	G	0.012	0.850	0.033	3.0E-150	trans
2048	SSH3	Q8TE77	Protein phosphatase Slingshot homolog 3	rs9275576	32679326	6	C	T	0.856	0.101	0.011	1.0E-19	trans
2049	SSNA1	O43805	Sjoegren syndrome nuclear autoantigen 1	rs1354034	56849749	3	C	T	0.605	0.101	0.008	8.0E-40	trans
2049	SSNA1	O43805	Sjoegren syndrome nuclear autoantigen 1	rs342298	106373646	7	C	T	0.545	0.055	0.008	1.8E-13	trans
2050	ST13	P50502	Hsc70-interacting protein	rs1354034	56849749	3	C	T	0.604	0.068	0.008	1.2E-19	trans
2050	ST13	P50502	Hsc70-interacting protein	rs1005402	41291730	22	T	C	0.467	0.054	0.007	3.1E-13	cis
2051	ST3GAL1	Q11201	CMP-N-acetylneuraminate-beta-galactosamide-alpha-2,3-sialyltransferase 1	rs12740374	109817590	1	G	T	0.779	0.104	0.009	3.6E-33	trans
2051	ST3GAL1	Q11201	CMP-N-acetylneuraminate-beta-galactosamide-alpha-2,3-sialyltransferase 1	rs539657009	161628456	1	T	C	0.129	0.080	0.011	5.2E-13	trans
2051	ST3GAL1	Q11201	CMP-N-acetylneuraminate-beta-galactosamide-alpha-2,3-sialyltransferase 1	rs4672497	62523565	2	G	C	0.222	0.180	0.009	9.5E-95	trans
2051	ST3GAL1	Q11201	CMP-N-acetylneuraminate-beta-galactosamide-alpha-2,3-sialyltransferase 1	rs9643300	134503148	8	C	T	0.435	0.237	0.007	6.6E-225	cis
2051	ST3GAL1	Q11201	CMP-N-acetylneuraminate-beta-galactosamide-alpha-2,3-sialyltransferase 1	rs56278466	17875857	10	G	T	0.661	0.053	0.008	1.9E-12	trans
2051	ST3GAL1	Q11201	CMP-N-acetylneuraminate-beta-galactosamide-alpha-2,3-sialyltransferase 1	rs28929474	94844947	14	T	C	0.021	0.244	0.025	4.7E-22	trans
2051	ST3GAL1	Q11201	CMP-N-acetylneuraminate-beta-galactosamide-alpha-2,3-sialyltransferase 1	rs28873836	52314655	19	G	C	0.734	0.101	0.008	5.2E-35	trans
2052	ST6GAL1	P15907	Beta-galactoside alpha-2,6-sialyltransferase 1	rs4686837	186739677	3	G	A	0.687	0.180	0.008	2.9E-113	cis
2052	ST6GAL1	P15907	Beta-galactoside alpha-2,6-sialyltransferase 1	rs6993770	106581528	8	A	T	0.713	0.057	0.008	1.5E-12	trans
2052	ST6GAL1	P15907	Beta-galactoside alpha-2,6-sialyltransferase 1	rs142744740	116688791	11	A	C	0.189	0.076	0.010	5.3E-13	trans
2052	ST6GAL1	P15907	Beta-galactoside alpha-2,6-sialyltransferase 1	rs28929474	94844947	14	T	C	0.021	0.209	0.026	3.3E-16	trans
2052	ST6GAL1	P15907	Beta-galactoside alpha-2,6-sialyltransferase 1	rs61658003	55544203	19	A	C	0.843	0.072	0.010	1.9E-12	trans
2053	ST8SIA1	Q92185	Alpha-N-acetylneuraminide alpha-2,8-sialyltransferase	rs28636516	32606701	6	G	A	0.128	0.088	0.012	3.2E-14	trans
2054	STAB2	Q8WWQ8	Stabilin-2	rs10935473	98416900	3	G	T	0.560	0.097	0.007	5.1E-46	trans
2054	STAB2	Q8WWQ8	Stabilin-2	rs200663261	32244080	6	C	CTACA	0.739	0.079	0.008	1.4E-24	trans
2054	STAB2	Q8WWQ8	Stabilin-2	rs4896219	137079542	6	C	A	0.483	0.053	0.007	7.0E-15	trans
2054	STAB2	Q8WWQ8	Stabilin-2	rs1969976	33120204	9	G	A	0.306	0.074	0.008	5.8E-22	trans
2054	STAB2	Q8WWQ8	Stabilin-2	rs4055121	126232337	11	C	T	0.865	0.082	0.010	2.0E-16	trans
2054	STAB2	Q8WWQ8	Stabilin-2	rs12920182	54417304	16	A	G	0.668	0.059	0.007	1.4E-16	trans
2054	STAB2	Q8WWQ8	Stabilin-2	rs186021206	7069412	17	A	G	0.006	0.673	0.047	3.1E-47	trans
2054	STAB2	Q8WWQ8	Stabilin-2	rs77542162	67081278	17	G	A	0.023	0.192	0.023	3.0E-17	trans
2054	STAB2	Q8WWQ8	Stabilin-2	rs12979429	7829180	19	A	G	0.284	0.063	0.008	1.2E-16	trans
2054	STAB2	Q8WWQ8	Stabilin-2	rs11672900	49220323	19	G	A	0.545	0.052	0.007	2.2E-14	trans
2055	STAMBP	O95630	STAM-binding protein	rs1354034	56849749	3	C	T	0.604	0.058	0.008	3.9E-14	trans
2055	STAMBP	O95630	STAM-binding protein	rs10733789	64948684	10	C	T	0.313	0.055	0.008	7.5E-12	trans
2056	STAT2	P52630	Signal transducer and activator of transcription 2	rs1354034	56849749	3	C	T	0.604	0.076	0.008	4.1E-23	trans
2057	STAT5B	P51692	Signal transducer and activator of transcription 5B	rs342298	106373646	7	C	T	0.546	0.062	0.008	1.9E-16	trans
2057	STAT5B	P51692	Signal transducer and activator of transcription 5B	rs6993770	106581528	8	A	T	0.714	0.068	0.008	2.7E-16	trans
2058	STAU1	O95793	Double-stranded RNA-binding protein Staufen homolog 1	rs13412535	224874874	2	G	A	0.769	0.116	0.009	1.5E-36	trans
2058	STAU1	O95793	Double-stranded RNA-binding protein Staufen homolog 1	rs71534539	32512754	6	G	A	0.536	0.079	0.009	2.6E-20	trans
2058	STAU1	O95793	Double-stranded RNA-binding protein Staufen homolog 1	rs34200987	107152294	14	G	T	0.233	0.106	0.010	5.9E-25	trans
2058	STAU1	O95793	Double-stranded RNA-binding protein Staufen homolog 1	rs11631612	101994285	15	C	T	0.708	0.059	0.009	5.9E-12	trans
2059	STC1	P52823	Stanniocalcin-1	rs57247992	23732894	8	A	G	0.077	0.121	0.014	1.3E-18	cis
2059	STC1	P52823	Stanniocalcin-1	rs2519093	136141870	9	C	T	0.815	0.080	0.009	3.1E-17	trans
2059	STC1	P52823	Stanniocalcin-1	rs113185690	1003848	10	T	C	0.077	0.113	0.014	5.5E-16	trans
2059	STC1	P52823	Stanniocalcin-1	rs9529913	72345089	13	T	C	0.604	0.079	0.007	7.3E-26	trans
2059	STC1	P52823	Stanniocalcin-1	rs12460997	7758793	19	C	A	0.515	0.114	0.007	2.3E-55	trans
2060	STC2	O76061	Stanniocalcin-2	rs2894602	227249802	2	G	A	0.766	0.061	0.009	1.4E-12	trans
2060	STC2	O76061	Stanniocalcin-2	rs11103374	139087521	9	T	C	0.132	0.075	0.011	3.4E-12	trans
2060	STC2	O76061	Stanniocalcin-2	rs1126464	89704365	16	G	C	0.756	0.068	0.008	6.9E-16	trans
2060	STC2	O76061	Stanniocalcin-2	rs12986064	54755133	19	T	C	0.492	0.069	0.007	2.7E-20	trans
2061	STIP1	P31948	Stress-induced-phosphoprotein 1	rs1354034	56849749	3	C	T	0.604	0.064	0.008	2.3E-17	trans
2061	STIP1	P31948	Stress-induced-phosphoprotein 1	rs10739067	4684770	9	G	A	0.343	0.077	0.008	7.2E-23	trans
2062	STK11	Q15831	Serine/threonine-protein kinase STK11	rs1354034	56849749	3	C	T	0.604	0.073	0.008	1.2E-21	trans
2062	STK11	Q15831	Serine/threonine-protein kinase STK11	rs342293	106372219	7	C	G	0.540	0.052	0.008	5.7E-12	trans
2062	STK11	Q15831	Serine/threonine-protein kinase STK11	rs6993770	106581528	8	A	T	0.713	0.072	0.008	5.9E-18	trans
2062	STK11	Q15831	Serine/threonine-protein kinase STK11	rs7924036	65191645	10	T	G	0.501	0.052	0.007	3.6E-12	trans
2062	STK11	Q15831	Serine/threonine-protein kinase STK11	rs892090	55539072	19	G	T	0.834	0.068	0.010	1.7E-11	trans
2063	STK24	Q9Y6E0	Serine/threonine-protein kinase 24	rs6993770	106581528	8	A	T	0.713	0.059	0.008	1.9E-12	trans
2064	STK4	Q13043	Serine/threonine-protein kinase 4	rs1354034	56849749	3	C	T	0.604	0.073	0.008	1.4E-21	trans

2064	STK4	Q13043	Serine/threonine-protein kinase 4	rs6993770	106581528	8	A	T	0.713	0.077	0.008	2.4E-20	trans
2064	STK4	Q13043	Serine/threonine-protein kinase 4	rs2299978	43687706	20	C	T	0.474	0.053	0.008	1.8E-12	cis
2065	STX16	O14662	Syntaxin-16	rs218476	57237670	20	A	G	0.380	0.062	0.008	3.9E-15	cis
2066	STX3	Q13277	Syntaxin-3	rs10191964	24287598	2	G	A	0.840	0.253	0.010	2.8E-132	trans
2066	STX3	Q13277	Syntaxin-3	rs9271377	32587165	6	T	G	0.634	0.059	0.008	6.4E-14	trans
2067	STX4	Q12846	Syntaxin-4	rs138182020	64938076	11	C	CT	0.738	0.074	0.009	1.4E-17	trans
2068	STX7	O15400	Syntaxin-7	rs3811444	248039451	1	C	T	0.668	0.081	0.007	1.4E-30	trans
2068	STX7	O15400	Syntaxin-7	rs1318638	30127873	6	T	C	0.063	0.105	0.015	3.7E-12	trans
2068	STX7	O15400	Syntaxin-7	rs3813356	132834518	6	C	T	0.549	0.590	0.008	0.0E+00	cis
2069	STX8	Q9UNK0	Syntaxin-8	rs1354034	56849749	3	C	T	0.604	0.054	0.008	1.4E-12	trans
2069	STX8	Q9UNK0	Syntaxin-8	rs6993770	106581528	8	A	T	0.713	0.060	0.008	2.7E-13	trans
2069	STX8	Q9UNK0	Syntaxin-8	rs8077500	9343771	17	A	G	0.244	0.102	0.009	2.4E-31	cis
2070	STXBP1	P61764	Syntaxin-binding protein 1	rs1354034	56849749	3	C	T	0.604	0.058	0.008	3.5E-14	trans
2070	STXBP1	P61764	Syntaxin-binding protein 1	rs7896518	65104500	10	G	A	0.424	0.083	0.008	8.9E-27	trans
2071	SUGP1	Q8IWZ8	SURP and G-patch domain-containing protein 1	rs17751061	19413092	19	T	C	0.160	0.325	0.010	5.0E-222	cis
2072	SUGT1	Q9Y2Z0	Protein SGT1 homolog	rs1354034	56849749	3	C	T	0.604	0.063	0.008	4.0E-16	trans
2073	SULT1A1	P50225	Sulfotransferase 1A1	rs148788997	30323168	16	C	G	0.126	0.150	0.012	1.1E-38	cis
2074	SULT2A1	Q06520	Bile salt sulfotransferase	rs4861708	187157233	4	A	G	0.512	0.077	0.007	1.5E-25	trans
2074	SULT2A1	Q06520	Bile salt sulfotransferase	rs6894249	131797547	5	A	G	0.613	0.052	0.008	6.7E-12	trans
2074	SULT2A1	Q06520	Bile salt sulfotransferase	rs62129966	48374950	19	C	A	0.836	0.378	0.010	1.6E-307	cis
2074	SULT2A1	Q06520	Bile salt sulfotransferase	rs3747207	44324855	22	A	G	0.216	0.073	0.009	3.2E-16	trans
2075	SUMF2	Q8NBJ7	Inactive C-alpha-formylglycine-generating enzyme 2	rs1289395	227042462	1	T	C	0.537	0.048	0.007	1.0E-11	trans
2075	SUMF2	Q8NBJ7	Inactive C-alpha-formylglycine-generating enzyme 2	rs9852409	57623541	3	T	A	0.178	0.189	0.009	3.5E-91	trans
2075	SUMF2	Q8NBJ7	Inactive C-alpha-formylglycine-generating enzyme 2	rs114082534	119734184	4	A	G	0.056	0.115	0.016	1.2E-13	trans
2075	SUMF2	Q8NBJ7	Inactive C-alpha-formylglycine-generating enzyme 2	rs62396264	31320662	6	G	C	0.036	0.255	0.019	3.9E-41	trans
2075	SUMF2	Q8NBJ7	Inactive C-alpha-formylglycine-generating enzyme 2	rs6796	6502367	7	C	T	0.279	0.072	0.008	4.1E-18	trans
2075	SUMF2	Q8NBJ7	Inactive C-alpha-formylglycine-generating enzyme 2	rs35384521	56145825	7	G	A	0.981	1.533	0.027	0.0E+00	cis
2075	SUMF2	Q8NBJ7	Inactive C-alpha-formylglycine-generating enzyme 2	rs79492875	127215059	7	C	A	0.931	0.192	0.015	5.0E-38	trans
2075	SUMF2	Q8NBJ7	Inactive C-alpha-formylglycine-generating enzyme 2	rs11999525	139371342	9	G	A	0.741	0.061	0.008	7.8E-14	trans
2075	SUMF2	Q8NBJ7	Inactive C-alpha-formylglycine-generating enzyme 2	rs477838	32101281	11	A	G	0.713	0.061	0.008	5.4E-15	trans
2075	SUMF2	Q8NBJ7	Inactive C-alpha-formylglycine-generating enzyme 2	rs438811	45416741	19	T	C	0.238	0.063	0.008	5.9E-14	trans
2075	SUMF2	Q8NBJ7	Inactive C-alpha-formylglycine-generating enzyme 2	rs4806498	54674742	19	C	T	0.569	0.071	0.007	3.3E-23	trans
2076	SUOX	P51687	Sulfite oxidase, mitochondrial	rs6674486	35263315	1	T	A	0.347	0.053	0.008	8.3E-12	trans
2076	SUOX	P51687	Sulfite oxidase, mitochondrial	rs13874	66419956	3	T	C	0.437	0.055	0.007	1.1E-13	trans
2076	SUOX	P51687	Sulfite oxidase, mitochondrial	rs9272212	32602033	6	G	A	0.612	0.055	0.008	8.2E-13	trans
2076	SUOX	P51687	Sulfite oxidase, mitochondrial	rs75773116	39904491	6	G	A	0.014	1.179	0.032	2.3E-289	trans
2076	SUOX	P51687	Sulfite oxidase, mitochondrial	rs112635299	94838142	14	T	G	0.022	0.173	0.025	6.7E-12	trans
2076	SUOX	P51687	Sulfite oxidase, mitochondrial	rs3747207	44324855	22	A	G	0.216	0.075	0.009	4.3E-17	trans
2077	SUSD1	Q6UWL2	Sushi domain-containing protein 1	rs9820435	122846881	3	C	T	0.407	0.055	0.008	1.8E-12	trans
2077	SUSD1	Q6UWL2	Sushi domain-containing protein 1	rs2631360	131707429	5	A	G	0.517	0.060	0.008	2.7E-15	trans
2077	SUSD1	Q6UWL2	Sushi domain-containing protein 1	rs61744929	2325427	11	T	C	0.981	0.441	0.028	1.4E-56	trans
2078	SUSD2	Q9UGT4	Sushi domain-containing protein 2	rs62292583	186659794	3	C	T	0.871	0.084	0.010	9.9E-17	trans
2078	SUSD2	Q9UGT4	Sushi domain-containing protein 2	rs9269502	32543121	6	A	G	0.641	0.062	0.008	1.1E-13	trans
2078	SUSD2	Q9UGT4	Sushi domain-containing protein 2	rs200489612	7106378	17	A	G	0.005	0.578	0.050	2.1E-30	trans
2078	SUSD2	Q9UGT4	Sushi domain-containing protein 2	rs117587385	24577204	22	A	G	0.038	1.205	0.019	0.0E+00	cis
2079	SUSD4	Q5VX71	Sushi domain-containing protein 4	rs1418152	223447975	1	T	G	0.599	0.380	0.008	0.0E+00	cis
2079	SUSD4	Q5VX71	Sushi domain-containing protein 4	rs28929474	94844947	14	T	C	0.021	0.314	0.025	1.6E-35	trans
2080	SUSD5	O60279	Sushi domain-containing protein 5	rs150816167	179571862	1	C	T	0.042	0.120	0.016	1.2E-14	trans
2080	SUSD5	O60279	Sushi domain-containing protein 5	rs1260326	27730940	2	C	T	0.608	0.056	0.006	6.1E-19	trans
2080	SUSD5	O60279	Sushi domain-containing protein 5	rs75166367	162964301	2	G	A	0.940	0.098	0.013	4.0E-14	trans
2080	SUSD5	O60279	Sushi domain-containing protein 5	rs62621812	127015083	7	A	G	0.021	0.224	0.022	2.3E-24	trans
2080	SUSD5	O60279	Sushi domain-containing protein 5	rs4752550	123151488	10	T	C	0.416	0.043	0.006	5.4E-12	trans
2080	SUSD5	O60279	Sushi domain-containing protein 5	rs36060036	20361950	16	C	T	0.832	0.061	0.008	1.4E-13	trans
2080	SUSD5	O60279	Sushi domain-containing protein 5	rs6643106	112128231	X	G	T	0.775	0.045	0.006	1.1E-13	trans
2081	SV2A	Q7L0J3	Synaptic vesicle glycoprotein 2A	rs68144650	149894445	1	A	C	0.920	0.296	0.014	3.9E-98	cis
2082	SWAP70	Q9UH65	Switch-associated protein 70	rs7123629	9678767	11	C	G	0.061	0.290	0.016	1.6E-75	cis
2083	SYAP1	Q96A49	Synapse-associated protein 1	rs1354034	56849749	3	C	T	0.604	0.079	0.008	6.7E-25	trans
2083	SYAP1	Q96A49	Synapse-associated protein 1	rs342299	106373718	7	C	T	0.545	0.055	0.008	4.5E-13	trans
2083	SYAP1	Q96A49	Synapse-associated protein 1	rs6993770	106581528	8	A	T	0.713	0.061	0.008	3.4E-13	trans
2083	SYAP1	Q96A49	Synapse-associated protein 1	rs7080386	65048306	10	A	C	0.412	0.060	0.008	4.2E-15	trans
2083	SYAP1	Q96A49	Synapse-associated protein 1	rs4830563	16762158	X	G	A	0.710	0.055	0.007	7.6E-16	cis
2084	SYTL4	Q96C24	Synaptotagmin-like protein 4	rs11242109	131677047	5	G	T	0.519	0.051	0.008	9.8E-12	trans
2084	SYTL4	Q96C24	Synaptotagmin-like protein 4	rs5921624	99932816	X	G	C	0.655	0.140	0.007	2.2E-102	cis
2085	TAB2	Q9NYJ8	TGF-beta-activated kinase 1 and MAP3K7-binding protein 2	rs1354034	56849749	3	C	T	0.604	0.053	0.008	3.7E-12	trans
2086	TACC3	Q9Y6A5	Transforming acidic coiled-coil-containing protein 3	rs6993770	106581528	8	A	T	0.714	0.067	0.008	5.2E-16	trans
2086	TACC3	Q9Y6A5	Transforming acidic coiled-coil-containing protein 3	rs7080386	65048306	10	A	C	0.412	0.084	0.008	3.2E-28	trans
2086	TACC3	Q9Y6A5	Transforming acidic coiled-coil-containing protein 3	rs11865642	9050637	16	C	A	0.189	0.071	0.010	1.9E-13	trans
2086	TACC3	Q9Y6A5	Transforming acidic coiled-coil-containing protein 3	rs13381663	22860759	18	C	T	0.209	0.088	0.009	4.1E-22	trans
2087	TACSTD2	P09758	Tumor-associated calcium signal transducer 2	rs7333	59041220	1	C	T	0.836	0.907	0.011	0.0E+00	cis
2087	TACSTD2	P09758	Tumor-associated calcium signal transducer 2	rs12740374	109817590	1	G	T	0.779	0.147	0.009	2.8E-54	trans
2087	TACSTD2	P09758	Tumor-associated calcium signal transducer 2	rs34070949	98354191	3	C	A	0.528	0.053	0.007	1.9E-15	trans
2087	TACSTD2	P09758	Tumor-associated calcium signal transducer 2	rs73745607	29824108	6	C	T	0.082	0.102	0.013	1.7E-15	trans
2087	TACSTD2	P09758	Tumor-associated calcium signal transducer 2	rs7824174	98812758	8	C	T	0.479	0.259	0.007	0.0E+00	trans
2087	TACSTD2	P09758	Tumor-associated calcium signal transducer 2	rs8176693	136137657	9	T	C	0.061	0.277	0.014	1.2E-88	trans
2087	TACSTD2	P09758	Tumor-associated calcium signal transducer 2	rs261342	58731153	15	G	C	0.223	0.070	0.008	4.5E-18	trans
2087	TACSTD2	P09758	Tumor-associated calcium signal transducer 2	rs56228609	56987765	16	T	C	0.315	0.073	0.007	4.2E-24	trans
2087	TACSTD2	P09758	Tumor-associated calcium signal transducer 2	rs72802342	75234872	16	C	A	0.924	0.100	0.013	3.6E-15	trans
2087	TACSTD2	P09758	Tumor-associated calcium signal transducer 2	rs186021206	7069412	17	A	G	0.007	0.659	0.045	8.4E-48	trans
2087	TACSTD2	P09758	Tumor-associated calcium signal transducer 2	rs72835428	47260650	17	T	C	0.123	0.116	0.010	4.2E-30	trans
2087	TACSTD2	P09758	Tumor-associated calcium signal transducer 2	rs601338	49206674	19	A	G	0.510	0.066	0.007	8.5E-23	trans
2087	TACSTD2	P09758	Tumor-associated calcium signal transducer 2	rs2868346	44547970	20	T	C	0.760	0.060	0.008	5.3E-15	trans
2088	TADA3	O75528	Transcriptional adapter 3	rs1354034	56849749	3	C	T	0.604	0.057	0.008	9.5E-14	trans
2089	TAFA5	Q7Z5A7	Chemokine-like protein TAFA-5	rs2870238	77373079	4	T	C	0.503	0.061	0.007	8.4E-17	trans
2089	TAFA5	Q7Z5A7	Chemokine-like protein TAFA-5	rs79072	48767186	22	T	G	0.715	0.182	0.009	3.0E-98	cis
2090	TALDO1	P37837	Transaldolase	rs1354034	56849749	3	C	T	0.605	0.062	0.007	1.6E-16	trans
2090	TALDO1	P37837	Transaldolase	rs6993770	106581528	8	A	T	0.714	0.085	0.008	1.5E-25	trans
2090	TALDO1	P37837	Transaldolase	rs150770244	824410	11	A	G	0.007	0.506	0.047	1.5E-26	cis
2091	TARBP2	Q15633	RISC-loading complex subunit TARBP2	rs1354034	56849749	3	C	T	0.604	0.073	0.008	2.4E-21	trans
2091	TARBP2	Q15633	RISC-loading complex subunit TARBP2	rs342293	106372219	7	C	G	0.541	0.055	0.008	3.4E-13	trans
2092	TAX1BP1	Q86VP1	Tax1-binding protein 1	rs1354034	56849749	3	C	T	0.604	0.099	0.008	4.1E-39	trans
2092	TAX1BP1	Q86VP1	Tax1-binding protein 1	rs6993770	106581528	8	A	T	0.713	0.057	0.008	1.8E-12	trans
2092	TAX1BP1	Q86VP1	Tax1-binding protein 1	rs11604127	196944	11	T	C	0.231	0.064	0.009	4.6E-13	trans
2093	TBC1D17	Q9HA65	TBC1 domain family member 17	rs7248744	50385031	19	G	A	0.425	0.414	0.007	0.0E+00	cis
2094	TBC1D23	Q9NUY8	TBC1 domain family member 23	rs1036770	100018715	3	C	T	0.415	0.154	0.008	2.7E-90	cis
2094	TBC1D23	Q9NUY8	TBC1 domain family member 23	rs342298	106373646	7	C	T	0.546	0.051	0.007	1.0E-11	trans
2094	TBC1D23	Q9NUY8	TBC1 domain family member 23	rs6993770	106581528	8	A	T	0.714	0.066	0.008	1.8E-15	trans
2094	TBC1D23	Q9NUY8	TBC1 domain family member 23	rs7090758	65335315	10	T	C	0.523	0.051	0.007	5.8E-12	trans
2095	TBC1D5	Q92609	TBC1 domain family member 5	rs1354034	56849749	3	C	T	0.604	0.059	0.008	5.1E-15	trans
2095	TBC1D5	Q92609	TBC1 domain family member 5	rs9971407	64898226	11	C	T	0.758	0.067	0.009	1.0E-14	trans
2096	TBCA	O75347	Tubulin-specific chaperone A	rs1354034	56849749	3	C	T	0.604	0.070	0.008	4.8E-20	trans
2096	TBCA	O75347	Tubulin-specific chaperone A	rs254415	77071703	5	T	G	0.305	0.140	0.008	5.6E-65	cis
2097	TBCB	Q99426	Tubulin-folding cofactor B	rs1354034	56849749	3	C	T	0.604	0.063	0.008	1.7E-16	trans
2097	TBCB	Q99426	Tubulin-folding cofactor B	rs342298	106373646	7	C	T	0.546	0.054	0.008	5.8E-13	trans
2097	TBCB	Q99426	Tubulin-folding cofactor B	rs6993770	106581528	8	A	T	0.713	0.063	0.008	4.0E-14	trans
2097	TBCB	Q99426	Tubulin-folding cofactor B	rs7896518	65104500	10	G	A	0.424	0.060	0.008	8.5E-15	trans
2098	TBCC	Q15814	Tubulin-specific chaperone C	rs1354034	56849749	3	C	T	0.604	0.061	0.008	1.9E-15	trans
2098	TBCC	Q15814	Tubulin-specific chaperone C	rs2234026	42713618	6	G	A	0.987	0.577	0.033	7.1E-69	cis
2098	TBCC	Q15814	Tubulin-specific chaperone C	rs342298	106373646	7	C	T	0.546	0.057	0.008	6.1E-14	trans
2098	TBCC	Q15814	Tubulin-specific chaperone C	rs10822145	64934548	10	T	C	0.472	0.052	0.008	6.2E-12	trans
2098	TBCC	Q15814	Tubulin-specific chaperone C	rs892090	55539072	19	G	T	0.834	0.069	0.010	1.5E-11	trans
2099	TBL1X	O60907	F-box-like/WD repeat-containing protein TBL1X	rs1354034	56849749	3	T	C	0.396	0.124	0.008	1.4E-57	trans
2099	TBL1X	O60907	F-box-like/WD repeat-containing protein TBL1X	rs11950562	131652529	5	A	C	0.522	0.052	0.008	8.0E-12	trans
2099	TBL1X	O60907	F-box-like/WD repeat-containing protein TBL1X	rs139141690	101499930	7	G	A	0.995	0.441	0.056	2.8E-15	trans
2099	TBL1X	O60907	F-box-like/WD repeat-containing protein TBL1X	rs6993770	106581528	8	A	T	0.713	0.072	0.008	9.0E-18	trans
2099	TBL1X	O60907	F-box-like/WD repeat-containing protein TBL1X	rs12767683	104316581	10	A	C	0.325	0.081	0.008	8.8E-24	trans
2100	TCL1A	P56279	T-cell leukemia/lymphoma protein 1A	rs301816	8505058	1	G	A	0.410	0.052	0.007	2.9E-12	trans
2100	TCL1A	P56279	T-cell leukemia/lymphoma protein 1A	rs17361097	153001219	4	A	G	0.438	0.052	0.007	8.3E-13	trans
2100	TCL1A	P56279	T-cell leukemia/lymphoma protein 1A	rs10689049	88120079	5	CCT	C	0.309	0.064	0.008	2.6E-16	trans
2100	TCL1A	P56279	T-cell leukemia/lymphoma protein 1A	rs77018303	143192064	5	G	T	0.982	0.215	0.027	1.7E-15	trans
2100	TCL1A	P56279	T-cell leukemia/lymphoma protein 1A	rs17056278	158252438	5	C	G	0.924	0.200	0.014	1.6E-48	trans
2100	TCL1A	P56279	T-cell leukemia/lymphoma protein 1A	rs34907473	31311938	6	A	G	0.723	0.078	0.008	1.6E-21	trans
2100	TCL1A	P56279	T-cell leukemia/lymphoma protein 1A	rs652951	44564008	6	C	A	0.508	0.051	0.007	2.5E-12	trans
2100	TCL1A	P56279	T-cell leukemia/lymphoma protein 1A	rs9483788	135435501	6	T	C	0.743	0.074	0.008	5.7E-19	trans
2100	TCL1A	P56279	T-cell leukemia/lymphoma protein 1A	rs116968179	50189183	7	G	A	0.967	0.146	0.021	1.2E-12	trans
2100	TCL1A	P56279	T-cell leukemia/lymphoma protein 1A	rs1433577	130595881	8	G	A	0.726	0.084	0.008	4.5E-25	trans
2100	TCL1A	P56279	T-cell leukemia/lymphoma protein 1A	rs4948492	63719739	10	C	T	0.341	0.074	0.008	2.9E-22	trans
2100	TCL1A	P56279	T-cell leukemia/lymphoma protein 1A	rs12784975	98380137	10	T	C	0.808	0.078	0.009	2.3E-17	trans
2100	TCL1A	P56279	T-cell leukemia/lymphoma protein 1A	rs76428106	28604007	13	C	T	0.013	0.229	0.033	5.0E-12	trans
2100	TCL1A	P56279	T-cell leukemia/lymphoma protein 1A	rs78986913	96162418	14	G	A	0.957	0.714	0.018	0.0E+00	cis
2100	TCL1A	P56279	T-cell leukemia/lymphoma protein 1A	rs11853271	91025830	15	A	G	0.322	0.060	0.008	5.5E-15	trans
2100	TCL1A	P56279	T-cell leukemia/lymphoma protein 1A	rs13330176	86019087	16	A	T	0.239	0.088	0.009	4.6E-25	trans
2100	TCL1A	P56279	T-cell leukemia/lymphoma protein 1A	rs12947321	2719872	17	A	G	0.507	0.062	0.007	1.2E-17	trans
2100	TCL1A	P56279	T-cell leukemia/lymphoma protein 1A	rs35222145	38007321	17	T	G	0.508	0.102	0.007	6.8E-44	trans
2100	TCL1A	P56279	T-cell leukemia/lymphoma protein 1A	rs6065926	44735854	20	A	G	0.247	0.057	0.008	1.5E-11	trans
2100	TCL1A	P56279	T-cell leukemia/lymphoma protein 1A	rs75743846	42325017	22	G	A	0.982	0.189	0.028	7.2E-12	trans
2101	TCN1	P20061	Transcobalamin-1	rs10208011	242723070	2	C	T	0.132	0.082	0.010	4.9E-17	trans
2101	TCN1	P20061	Transcobalamin-1	rs4857909	128300468	3	G	A	0.882	0.102	0.010	7.2E-23	trans
2101	TCN1	P20061	Transcobalamin-1	rs72667759	87952199	4	C	G	0.807	0.058	0.008	6.1E-12	trans
2101	TCN1	P20061	Transcobalamin-1	rs849867	22337342	6	A	G	0.482	0.055	0.007	1.5E-16	trans
2101	TCN1	P20061	Transcobalamin-1	rs9294380	88152667	6	G	A	0.430	0.051	0.007	5.0E-14	trans
2101	TCN1	P20061	Transcobalamin-1	rs6796	6502367	7	C	T	0.279	0.085	0.007	1.2E-30	trans
2101	TCN1	P20061	Transcobalamin-1	rs34324219	59623378	11	C	A	0.893	0.754	0.012	0.0E+00	cis
2101	TCN1	P20061	Transcobalamin-1	rs3967200	126232385	11	C	T	0.865	0.108	0.011	1.7E-23	trans

2101	TCN1	P20061	Transcobalamin-1	rs8015478	23586018	14	C	A	0.736	0.062	0.008	3.3E-16	trans
2101	TCN1	P20061	Transcobalamin-1	rs186021206	7069412	17	A	G	0.006	0.659	0.046	5.4E-47	trans
2101	TCN1	P20061	Transcobalamin-1	rs12941811	38159335	17	T	C	0.426	0.061	0.007	7.1E-20	trans
2101	TCN1	P20061	Transcobalamin-1	rs17855739	5831840	19	T	C	0.041	0.233	0.017	1.3E-40	trans
2101	TCN1	P20061	Transcobalamin-1	rs679574	49206108	19	G	C	0.509	0.395	0.007	0.0E+00	trans
2102	TCN2	P20062	Transcobalamin-2	rs150384171	8369918	19	A	ACTC	0.014	1.239	0.027	0.0E+00	trans
2102	TCN2	P20062	Transcobalamin-2	rs12986064	54755133	19	T	C	0.493	0.071	0.006	9.9E-28	trans
2102	TCN2	P20062	Transcobalamin-2	rs740234	31008745	22	G	A	0.204	0.633	0.009	0.0E+00	cis
2103	TCOF1	Q13428	Treacle protein	rs56180593	149740732	5	C	T	0.995	0.549	0.052	8.9E-26	cis
2103	TCOF1	Q13428	Treacle protein	rs7080536	115348046	10	G	A	0.956	0.160	0.019	4.7E-18	trans
2104	TCTN3	Q6NUS6	Tectonic-3	rs78444298	184672098	1	A	G	0.019	0.193	0.023	1.8E-17	trans
2104	TCTN3	Q6NUS6	Tectonic-3	rs7646106	5257971	3	T	C	0.175	0.058	0.008	1.8E-12	trans
2104	TCTN3	Q6NUS6	Tectonic-3	rs35457250	186338564	3	T	C	0.009	0.486	0.033	7.2E-49	trans
2104	TCTN3	Q6NUS6	Tectonic-3	rs28601761	126500031	8	C	G	0.580	0.052	0.006	2.5E-16	trans
2104	TCTN3	Q6NUS6	Tectonic-3	rs61871796	97425330	10	C	A	0.349	0.631	0.008	0.0E+00	cis
2104	TCTN3	Q6NUS6	Tectonic-3	rs28929474	94844947	14	T	C	0.021	0.272	0.022	3.8E-36	trans
2104	TCTN3	Q6NUS6	Tectonic-3	rs56990244	427999	16	C	G	0.412	0.077	0.006	3.6E-34	trans
2104	TCTN3	Q6NUS6	Tectonic-3	rs12599863	75593932	16	G	A	0.309	0.061	0.007	1.9E-19	trans
2104	TCTN3	Q6NUS6	Tectonic-3	rs186021206	7069412	17	A	G	0.006	0.524	0.043	1.8E-34	trans
2104	TCTN3	Q6NUS6	Tectonic-3	rs1801689	64210580	17	C	A	0.030	0.278	0.018	5.6E-53	trans
2104	TCTN3	Q6NUS6	Tectonic-3	rs112001035	66823805	17	A	G	0.061	0.169	0.013	3.6E-37	trans
2104	TCTN3	Q6NUS6	Tectonic-3	rs429358	45411941	19	T	C	0.845	0.090	0.009	7.9E-26	trans
2105	TDGF1	P13385	Teratocarcinoma-derived growth factor 1	rs112481213	46619238	3	A	T	0.240	1.319	0.009	0.0E+00	cis
2105	TDGF1	P13385	Teratocarcinoma-derived growth factor 1	rs113354603	117086241	9	G	A	0.930	0.070	0.010	5.7E-12	trans
2105	TDGF1	P13385	Teratocarcinoma-derived growth factor 1	rs507666	136149399	9	G	A	0.816	0.052	0.006	4.0E-16	trans
2106	TDP1	Q9NUW8	Tyrosyl-DNA phosphodiesterase 1	rs9268833	32428062	6	T	C	0.317	0.068	0.008	7.4E-17	trans
2106	TDP1	Q9NUW8	Tyrosyl-DNA phosphodiesterase 1	rs79027981	90428434	14	G	A	0.017	0.307	0.029	2.1E-26	cis
2106	TDP1	Q9NUW8	Tyrosyl-DNA phosphodiesterase 1	rs10147250	106588222	14	T	C	0.553	0.056	0.008	1.8E-12	trans
2107	TDRKH	Q9Y2W6	Tudor and KH domain-containing protein	rs4845556	151744858	1	A	G	0.481	0.314	0.008	0.0E+00	cis
2107	TDRKH	Q9Y2W6	Tudor and KH domain-containing protein	rs1354034	56849749	3	C	T	0.604	0.057	0.008	6.3E-14	trans
2107	TDRKH	Q9Y2W6	Tudor and KH domain-containing protein	rs6993770	106581528	8	A	T	0.713	0.069	0.008	4.1E-17	trans
2107	TDRKH	Q9Y2W6	Tudor and KH domain-containing protein	rs7896518	65104500	10	G	A	0.424	0.055	0.008	2.5E-13	trans
2108	TEF	Q10587	Thyrotroph embryonic factor	rs4822027	41786227	22	A	G	0.210	0.084	0.010	6.5E-19	cis
2109	TEK	Q02763	Angiopoietin-1 receptor	rs10935473	98416900	3	G	T	0.560	0.072	0.007	6.5E-28	trans
2109	TEK	Q02763	Angiopoietin-1 receptor	rs13107325	103188709	4	T	C	0.075	0.117	0.012	2.9E-21	trans
2109	TEK	Q02763	Angiopoietin-1 receptor	rs1050518	31324641	6	T	A	0.702	0.223	0.007	7.4E-197	trans
2109	TEK	Q02763	Angiopoietin-1 receptor	rs2134964	89397637	8	A	G	0.300	0.105	0.007	2.1E-49	trans
2109	TEK	Q02763	Angiopoietin-1 receptor	rs682632	27183463	9	A	C	0.040	1.045	0.019	0.0E+00	cis
2109	TEK	Q02763	Angiopoietin-1 receptor	rs8176759	136129647	9	A	G	0.061	0.811	0.015	0.0E+00	trans
2109	TEK	Q02763	Angiopoietin-1 receptor	rs174530	61546592	11	G	A	0.373	0.045	0.007	1.5E-11	trans
2109	TEK	Q02763	Angiopoietin-1 receptor	rs35166255	126301756	11	A	G	0.034	0.286	0.018	5.1E-57	trans
2109	TEK	Q02763	Angiopoietin-1 receptor	rs1042704	23312594	14	G	A	0.785	0.097	0.008	1.3E-34	trans
2109	TEK	Q02763	Angiopoietin-1 receptor	rs186021206	7069412	17	A	G	0.006	0.479	0.045	6.7E-27	trans
2110	TET2	Q6N021	Methylcytosine dioxygenase TET2	rs2516886	107105677	14	C	T	0.142	0.078	0.011	2.0E-12	trans
2111	TEX101	Q9BY14	Testis-expressed protein 101	rs35033974	43920612	19	G	T	0.871	0.332	0.008	0.0E+00	cis
2112	TF	P02787	Serotransferrin	rs13008704	190387487	2	T	C	0.506	0.054	0.007	8.7E-14	trans
2112	TF	P02787	Serotransferrin	rs8177247	133479499	3	G	T	0.342	0.329	0.008	0.0E+00	cis
2112	TF	P02787	Serotransferrin	rs79220007	26098474	6	T	C	0.923	0.435	0.014	1.6E-219	trans
2112	TF	P02787	Serotransferrin	rs174576	61603510	11	A	C	0.352	0.051	0.008	1.3E-11	trans
2113	TFF1	P04155	Trefoil factor 1	rs760077	155178782	1	A	T	0.395	0.095	0.007	5.1E-39	trans
2113	TFF1	P04155	Trefoil factor 1	rs62133344	69177269	2	A	C	0.056	0.409	0.015	2.5E-154	trans
2113	TFF1	P04155	Trefoil factor 1	rs2075842	1215056	11	C	G	0.416	0.072	0.007	5.3E-24	trans
2113	TFF1	P04155	Trefoil factor 1	rs536255	41010801	21	C	T	0.809	0.081	0.009	1.1E-18	trans
2113	TFF1	P04155	Trefoil factor 1	rs3761376	43787038	21	G	A	0.765	0.304	0.009	1.6E-280	cis
2114	TFF2	Q03403	Trefoil factor 2	rs760077	155178782	1	A	T	0.395	0.099	0.007	2.8E-41	trans
2114	TFF2	Q03403	Trefoil factor 2	rs2978981	143759137	8	C	T	0.559	0.052	0.007	3.4E-13	trans
2114	TFF2	Q03403	Trefoil factor 2	rs2075842	1215056	11	C	G	0.416	0.114	0.007	2.1E-54	trans
2114	TFF2	Q03403	Trefoil factor 2	rs2217142	49434635	13	C	T	0.418	0.075	0.007	1.4E-24	trans
2114	TFF2	Q03403	Trefoil factor 2	rs186021206	7069412	17	A	G	0.006	0.715	0.049	8.7E-48	trans
2114	TFF2	Q03403	Trefoil factor 2	rs601338	49206674	19	G	A	0.490	0.050	0.007	4.1E-12	trans
2114	TFF2	Q03403	Trefoil factor 2	rs1534081	41023611	21	G	A	0.748	0.070	0.008	4.7E-17	trans
2114	TFF2	Q03403	Trefoil factor 2	rs225344	43775884	21	A	G	0.726	0.125	0.008	3.1E-54	cis
2115	TFF3	Q07654	Trefoil factor 3	rs10023335	77358987	4	C	T	0.411	0.050	0.007	4.9E-12	trans
2115	TFF3	Q07654	Trefoil factor 3	rs2524277	31407579	6	A	G	0.059	0.614	0.016	0.0E+00	trans
2115	TFF3	Q07654	Trefoil factor 3	rs118095917	43733628	21	C	T	0.994	1.277	0.046	1.3E-172	cis
2116	TFPI	P10646	Tissue factor pathway inhibitor	rs12127364	169469142	1	A	C	0.271	0.145	0.008	1.6E-74	trans
2116	TFPI	P10646	Tissue factor pathway inhibitor	rs1260326	27730940	2	T	C	0.392	0.054	0.007	2.3E-13	trans
2116	TFPI	P10646	Tissue factor pathway inhibitor	rs7576066	188343781	2	A	G	0.294	0.347	0.008	0.0E+00	cis
2116	TFPI	P10646	Tissue factor pathway inhibitor	rs28601761	126500031	8	C	G	0.581	0.060	0.007	5.4E-17	trans
2116	TFPI	P10646	Tissue factor pathway inhibitor	rs1111796	117086226	9	A	T	0.924	0.172	0.014	1.4E-35	trans
2116	TFPI	P10646	Tissue factor pathway inhibitor	rs7412	45412079	19	C	T	0.918	0.153	0.013	1.1E-32	trans
2117	TFPI2	P48307	Tissue factor pathway inhibitor 2	rs61804208	161661411	1	T	G	0.105	0.137	0.012	4.9E-32	trans
2117	TFPI2	P48307	Tissue factor pathway inhibitor 2	rs68066031	224880498	2	C	T	0.230	0.070	0.008	2.1E-16	trans
2117	TFPI2	P48307	Tissue factor pathway inhibitor 2	rs6768289	126256216	3	G	A	0.747	0.141	0.008	4.8E-67	trans
2117	TFPI2	P48307	Tissue factor pathway inhibitor 2	rs6924387	137082948	6	A	G	0.594	0.060	0.007	1.6E-16	trans
2117	TFPI2	P48307	Tissue factor pathway inhibitor 2	rs62466699	93579292	7	C	T	0.058	0.242	0.015	4.8E-55	cis
2117	TFPI2	P48307	Tissue factor pathway inhibitor 2	rs10112582	55433152	8	G	A	0.201	0.094	0.009	1.8E-26	trans
2117	TFPI2	P48307	Tissue factor pathway inhibitor 2	rs35719208	105966314	8	T	C	0.188	0.096	0.009	4.1E-26	trans
2117	TFPI2	P48307	Tissue factor pathway inhibitor 2	rs5791751	47034557	11	T	TA	0.327	0.085	0.008	7.0E-29	trans
2117	TFPI2	P48307	Tissue factor pathway inhibitor 2	rs2160725	66395726	17	A	C	0.515	0.058	0.007	3.0E-16	trans
2118	TFRC	P02786	Transferrin receptor protein 1	rs3811647	133484029	3	G	A	0.658	0.055	0.008	2.6E-12	trans
2118	TFRC	P02786	Transferrin receptor protein 1	rs3817672	195800811	3	C	T	0.437	0.524	0.008	0.0E+00	cis
2118	TFRC	P02786	Transferrin receptor protein 1	rs252152	141445774	5	G	A	0.662	0.081	0.007	1.5E-28	trans
2118	TFRC	P02786	Transferrin receptor protein 1	rs1800562	26093141	6	G	A	0.922	0.277	0.013	9.0E-101	trans
2118	TFRC	P02786	Transferrin receptor protein 1	rs6592965	50427982	7	G	A	0.546	0.063	0.007	8.2E-20	trans
2118	TFRC	P02786	Transferrin receptor protein 1	rs3211931	80298173	7	C	T	0.567	0.050	0.007	6.9E-13	trans
2118	TFRC	P02786	Transferrin receptor protein 1	rs2075672	100240296	7	A	G	0.377	0.067	0.007	3.4E-21	trans
2118	TFRC	P02786	Transferrin receptor protein 1	rs652455	117071449	11	C	T	0.149	0.193	0.010	6.4E-86	trans
2118	TFRC	P02786	Transferrin receptor protein 1	rs74035509	88567333	16	T	C	0.078	0.093	0.013	9.1E-13	trans
2118	TFRC	P02786	Transferrin receptor protein 1	rs2072860	37470604	22	G	A	0.466	0.139	0.007	4.5E-89	trans
2119	TG	P01266	Thyroglobulin	rs334721	61638521	1	A	C	0.040	0.171	0.019	1.8E-19	trans
2119	TG	P01266	Thyroglobulin	rs17020146	108374702	1	C	A	0.088	0.157	0.013	1.5E-33	trans
2119	TG	P01266	Thyroglobulin	rs1690789	218698027	1	T	C	0.486	0.084	0.007	5.0E-30	trans
2119	TG	P01266	Thyroglobulin	rs3748916	113984033	2	A	G	0.472	0.070	0.007	2.4E-21	trans
2119	TG	P01266	Thyroglobulin	rs13015993	217625523	2	G	A	0.268	0.070	0.008	7.5E-17	trans
2119	TG	P01266	Thyroglobulin	rs4678938	37181015	3	G	C	0.531	0.058	0.007	3.4E-15	trans
2119	TG	P01266	Thyroglobulin	rs10519980	149635025	4	C	T	0.796	0.064	0.009	1.3E-12	trans
2119	TG	P01266	Thyroglobulin	rs66760320	43906255	6	T	C	0.250	0.063	0.008	1.7E-13	trans
2119	TG	P01266	Thyroglobulin	rs9497965	148521292	6	T	C	0.409	0.085	0.007	6.3E-30	trans
2119	TG	P01266	Thyroglobulin	rs17719705	32414332	8	T	A	0.454	0.055	0.007	6.3E-14	trans
2119	TG	P01266	Thyroglobulin	rs75143612	133849655	8	C	G	0.028	0.246	0.022	1.5E-28	cis
2119	TG	P01266	Thyroglobulin	rs10814915	4290544	9	C	T	0.586	0.052	0.007	4.5E-12	trans
2119	TG	P01266	Thyroglobulin	rs976575	109649778	9	A	G	0.640	0.080	0.008	3.6E-25	trans
2119	TG	P01266	Thyroglobulin	rs7128207	45230061	11	G	T	0.440	0.067	0.007	2.4E-19	trans
2119	TG	P01266	Thyroglobulin	rs9507285	24784888	13	C	G	0.652	0.054	0.008	3.7E-12	trans
2119	TG	P01266	Thyroglobulin	rs4338740	49735297	15	C	T	0.259	0.095	0.008	7.1E-30	trans
2119	TG	P01266	Thyroglobulin	rs12148331	89110081	15	C	T	0.680	0.054	0.008	9.0E-12	trans
2119	TG	P01266	Thyroglobulin	rs17689024	79739978	16	C	G	0.313	0.095	0.008	6.8E-33	trans
2119	TG	P01266	Thyroglobulin	rs1203948	22604772	20	G	A	0.215	0.121	0.009	1.3E-41	trans
2120	TGFA	P01135	Protransforming growth factor alpha	rs17639251	70776281	2	C	T	0.119	0.175	0.012	2.9E-51	cis
2121	TGFB1	P01137	Transforming growth factor beta-1 proprotein	rs3804749	122833003	3	C	T	0.407	0.072	0.008	2.8E-21	trans
2121	TGFB1	P01137	Transforming growth factor beta-1 proprotein	rs373385028	96325026	5	A	T	0.448	0.087	0.007	4.0E-32	trans
2121	TGFB1	P01137	Transforming growth factor beta-1 proprotein	rs6796	6502367	7	C	T	0.279	0.060	0.008	2.7E-13	trans
2121	TGFB1	P01137	Transforming growth factor beta-1 proprotein	rs2544221	133993014	7	C	G	0.462	0.066	0.007	5.9E-19	trans
2121	TGFB1	P01137	Transforming growth factor beta-1 proprotein	rs6993770	106581528	8	A	T	0.713	0.139	0.008	2.3E-65	trans
2121	TGFB1	P01137	Transforming growth factor beta-1 proprotein	rs2519093	136141870	9	C	T	0.816	0.110	0.010	1.1E-30	trans
2121	TGFB1	P01137	Transforming growth factor beta-1 proprotein	rs73045269	41825191	19	T	C	0.171	0.288	0.010	2.9E-187	cis
2121	TGFB1	P01137	Transforming growth factor beta-1 proprotein	rs892090	55539072	19	G	T	0.834	0.117	0.010	1.2E-31	trans
2122	TGFB2	P61812	Transforming growth factor beta-2 proprotein	rs7538934	218694349	1	T	C	0.119	0.197	0.012	3.8E-62	cis
2123	TGFBI	Q15582	Transforming growth factor-beta-induced protein ig-h3	rs2493134	230849359	1	T	C	0.601	0.257	0.007	1.1E-300	trans
2123	TGFBI	Q15582	Transforming growth factor-beta-induced protein ig-h3	rs11127048	27752463	2	G	A	0.382	0.064	0.007	6.7E-20	trans
2123	TGFBI	Q15582	Transforming growth factor-beta-induced protein ig-h3	rs13159365	135389433	5	C	T	0.490	0.546	0.007	0.0E+00	cis
2123	TGFBI	Q15582	Transforming growth factor-beta-induced protein ig-h3	rs9268812	32424568	6	G	A	0.658	0.069	0.007	3.5E-22	trans
2123	TGFBI	Q15582	Transforming growth factor-beta-induced protein ig-h3	rs28601761	126500031	8	C	G	0.581	0.061	0.007	4.5E-19	trans
2123	TGFBI	Q15582	Transforming growth factor-beta-induced protein ig-h3	rs10982156	117088064	9	T	A	0.930	0.445	0.014	7.1E-229	trans
2123	TGFBI	Q15582	Transforming growth factor-beta-induced protein ig-h3	rs56278466	17875857	10	G	T	0.661	0.056	0.007	9.3E-16	trans
2123	TGFBI	Q15582	Transforming growth factor-beta-induced protein ig-h3	rs28929474	94844947	14	T	C	0.021	0.192	0.023	2.2E-16	trans
2123	TGFBI	Q15582	Transforming growth factor-beta-induced protein ig-h3	rs116683462	57851781	17	T	A	0.543	0.074	0.007	9.0E-29	trans
2123	TGFBI	Q15582	Transforming growth factor-beta-induced protein ig-h3	rs12975366	54759361	19	T	C	0.605	0.064	0.007	5.6E-21	trans
2124	TGFBR2	P37173	TGF-beta receptor type-2	rs61747728	179526214	1	T	C	0.038	0.192	0.019	4.8E-25	trans
2124	TGFBR2	P37173	TGF-beta receptor type-2	rs1260326	27730940	2	C	T	0.609	0.056	0.007	3.1E-14	trans
2124	TGFBR2	P37173	TGF-beta receptor type-2	rs75166367	162964301	2	G	A	0.940	0.103	0.015	7.0E-12	trans
2124	TGFBR2	P37173	TGF-beta receptor type-2	rs56388355	30181399	3	AG	A	0.402	0.051	0.008	1.4E-11	cis
2124	TGFBR2	P37173	TGF-beta receptor type-2	rs3129887	32410691	6	A	G	0.188	0.089	0.009	1.7E-22	trans
2124	TGFBR2	P37173	TGF-beta receptor type-2	rs10265221	151414329	7	C	T	0.287	0.053	0.008	1.0E-11	trans
2124	TGFBR2	P37173	TGF-beta receptor type-2	rs507666	136149399	9	G	A	0.816	0.170	0.009	3.6E-75	trans
2124	TGFBR2	P37173	TGF-beta receptor type-2	rs2837988	42619544	21	A	C	0.362	0.051	0.007	8.0E-12	trans
2125	TGFBR3	Q03167	Transforming growth factor beta receptor type 3	rs11207423	59650829	1	C	G	0.277	0.100	0.008	2.4E-33	trans
2125	TGFBR3	Q03167	Transforming growth factor beta receptor type 3	rs2634023	92324150	1	C	T	0.920	0.231	0.014	2.0E-64	cis
2125	TGFBR3	Q03167	Transforming growth factor beta receptor type 3	rs150816167	179571862	1	C	T	0.042	0.144	0.019	1.3E-14	trans
2125	TGFBR3	Q03167	Transforming growth factor beta receptor type 3	rs71640034	187161048	4	G	A	0.486	0.076	0.007	6.1E-25	trans
2125	TGFBR3	Q03167	Transforming growth factor beta receptor type 3	rs181242111	17865664	10	A	G	0.118	0.098	0.012	1.2E-15	trans
2125	TGFBR3	Q03167	Transforming growth factor beta receptor type 3	rs77924615	20392332	16	G	A	0.803	0.072	0.009	1.7E-14	trans

2125	TGFBR3	Q03167	Transforming growth factor beta receptor type 3	rs1236213	28477742	21	G	T	0.407	0.159	0.008	3.2E-97	trans
2126	TGM2	P21980	Protein-glutamine gamma-glutamyltransferase 2	rs9272445	32605420	6	C	T	0.869	0.116	0.011	3.5E-25	trans
2126	TGM2	P21980	Protein-glutamine gamma-glutamyltransferase 2	rs6592965	50427982	7	A	G	0.454	0.062	0.007	1.3E-17	trans
2126	TGM2	P21980	Protein-glutamine gamma-glutamyltransferase 2	rs2076382	36793966	20	T	C	0.629	0.169	0.007	3.2E-113	cis
2127	TGOLN2	O43493	Trans-Golgi network integral membrane protein 2	rs629301	109818306	1	G	T	0.222	0.061	0.008	4.5E-15	trans
2127	TGOLN2	O43493	Trans-Golgi network integral membrane protein 2	rs61747728	179526214	1	T	C	0.038	0.182	0.017	1.1E-27	trans
2127	TGOLN2	O43493	Trans-Golgi network integral membrane protein 2	rs4247303	85554080	2	G	A	0.518	0.715	0.007	0.0E+00	cis
2127	TGOLN2	O43493	Trans-Golgi network integral membrane protein 2	rs169504	32153406	6	A	C	0.186	0.062	0.008	1.1E-13	trans
2127	TGOLN2	O43493	Trans-Golgi network integral membrane protein 2	rs4997081	20365234	16	G	C	0.808	0.066	0.008	8.5E-16	trans
2127	TGOLN2	O43493	Trans-Golgi network integral membrane protein 2	rs3814995	36342212	19	T	C	0.315	0.057	0.007	2.4E-16	trans
2128	THAP12	O43422	52 kDa repressor of the inhibitor of the protein kinase	rs2516980	106348477	14	C	A	0.780	0.125	0.011	3.2E-32	trans
2129	THBD	P07204	Thrombomodulin	rs1337526	47965130	1	G	A	0.803	0.067	0.009	7.4E-14	trans
2129	THBD	P07204	Thrombomodulin	rs61747728	179526214	1	T	C	0.038	0.173	0.019	1.0E-20	trans
2129	THBD	P07204	Thrombomodulin	rs1260326	27730940	2	C	T	0.608	0.052	0.007	1.1E-12	trans
2129	THBD	P07204	Thrombomodulin	rs75166367	162964301	2	G	A	0.940	0.113	0.015	3.3E-14	trans
2129	THBD	P07204	Thrombomodulin	rs638333	72419267	5	C	T	0.290	0.071	0.008	7.5E-19	trans
2129	THBD	P07204	Thrombomodulin	rs9264666	31239296	6	G	C	0.445	0.061	0.007	1.3E-17	trans
2129	THBD	P07204	Thrombomodulin	rs8176743	136131415	9	T	C	0.061	0.329	0.015	6.2E-108	trans
2129	THBD	P07204	Thrombomodulin	rs34434834	126307796	11	A	G	0.033	0.253	0.020	4.3E-37	trans
2129	THBD	P07204	Thrombomodulin	rs8038694	101774279	15	A	G	0.219	0.063	0.009	3.5E-13	trans
2129	THBD	P07204	Thrombomodulin	rs77924615	20392332	16	G	A	0.803	0.077	0.009	2.5E-17	trans
2129	THBD	P07204	Thrombomodulin	rs186021206	7069412	17	A	G	0.006	0.398	0.049	3.2E-16	trans
2129	THBD	P07204	Thrombomodulin	rs2659005	79218714	17	T	C	0.441	0.068	0.007	4.4E-21	trans
2129	THBD	P07204	Thrombomodulin	rs33950747	36339247	19	T	C	0.075	0.144	0.014	1.7E-26	trans
2129	THBD	P07204	Thrombomodulin	rs1042579	23028724	20	A	G	0.192	0.201	0.009	4.3E-107	cis
2130	THBS2	P35442	Thrombospondin-2	rs2271708	40936541	5	T	C	0.994	0.343	0.046	8.6E-14	trans
2130	THBS2	P35442	Thrombospondin-2	rs78109196	169624701	6	A	C	0.097	0.661	0.013	0.0E+00	cis
2130	THBS2	P35442	Thrombospondin-2	rs3967200	126232385	11	C	T	0.865	0.118	0.010	1.5E-29	trans
2130	THBS2	P35442	Thrombospondin-2	rs186021206	7069412	17	A	G	0.006	0.560	0.049	1.4E-30	trans
2130	THBS2	P35442	Thrombospondin-2	rs146385050	60637258	17	A	C	0.183	0.233	0.009	1.2E-139	trans
2130	THBS2	P35442	Thrombospondin-2	rs2111504	32917455	19	A	T	0.172	0.144	0.009	2.0E-53	trans
2130	THBS2	P35442	Thrombospondin-2	rs738409	44324727	22	G	C	0.217	0.070	0.009	3.7E-16	trans
2131	THBS4	P35443	Thrombospondin-4	rs2438632	79392193	5	T	G	0.398	0.244	0.008	3.3E-227	cis
2131	THBS4	P35443	Thrombospondin-4	rs2881756	7256440	7	T	C	0.411	0.074	0.007	5.9E-23	trans
2131	THBS4	P35443	Thrombospondin-4	rs8176719	136132908	9	TC	T	0.340	0.053	0.008	3.0E-12	trans
2131	THBS4	P35443	Thrombospondin-4	rs56278466	17875857	10	G	T	0.661	0.093	0.008	1.2E-33	trans
2131	THBS4	P35443	Thrombospondin-4	rs112771035	126225876	11	C	G	0.929	0.133	0.014	6.4E-21	trans
2131	THBS4	P35443	Thrombospondin-4	rs186021206	7069412	17	A	G	0.006	1.326	0.050	4.0E-154	trans
2132	THOP1	P52888	Thimet oligopeptidase	rs2741990	2796644	19	T	C	0.293	0.268	0.008	5.2E-247	cis
2133	THPO	P40225	Thrombopoietin	rs113926195	58223405	3	G	GCC	0.770	0.067	0.009	1.0E-13	trans
2133	THPO	P40225	Thrombopoietin	rs3792366	122839876	3	G	A	0.407	0.052	0.008	1.2E-11	trans
2133	THPO	P40225	Thrombopoietin	rs6141	184090266	3	T	C	0.532	0.062	0.008	2.3E-16	cis
2133	THPO	P40225	Thrombopoietin	rs11242109	131677047	5	G	T	0.518	0.060	0.008	1.7E-15	trans
2133	THPO	P40225	Thrombopoietin	rs56255857	38241328	7	T	C	0.755	0.123	0.009	5.4E-45	trans
2133	THPO	P40225	Thrombopoietin	rs6993770	106581528	8	A	T	0.713	0.128	0.008	3.2E-53	trans
2133	THPO	P40225	Thrombopoietin	rs10762489	73651458	10	G	A	0.223	0.068	0.009	3.7E-14	trans
2133	THPO	P40225	Thrombopoietin	rs56385468	1794676	11	T	G	0.913	0.212	0.013	1.0E-56	trans
2133	THPO	P40225	Thrombopoietin	rs28929474	94844947	14	T	C	0.021	0.360	0.026	2.1E-42	trans
2133	THPO	P40225	Thrombopoietin	rs151234	28505660	16	C	G	0.129	0.083	0.011	1.7E-13	trans
2133	THPO	P40225	Thrombopoietin	rs12445050	81870969	16	T	C	0.138	0.076	0.011	3.6E-12	trans
2133	THPO	P40225	Thrombopoietin	rs149394327	64228995	17	C	G	0.030	0.157	0.022	1.1E-12	trans
2133	THPO	P40225	Thrombopoietin	rs1654425	55538980	19	C	T	0.834	0.102	0.010	1.1E-23	trans
2133	THPO	P40225	Thrombopoietin	rs5921631	99953191	X	C	G	0.360	0.050	0.006	1.3E-14	trans
2134	THSD1	Q9NS62	Thrombospondin type-1 domain-containing protein 1	rs2519093	136141870	9	C	T	0.815	0.202	0.010	1.5E-89	trans
2134	THSD1	Q9NS62	Thrombospondin type-1 domain-containing protein 1	rs149590732	52971687	13	G	A	0.004	1.111	0.063	1.2E-70	cis
2135	THTPA	Q9BU02	Thiamine-triphosphatase	rs1354034	56849749	3	C	T	0.604	0.063	0.007	6.9E-17	trans
2135	THTPA	Q9BU02	Thiamine-triphosphatase	rs342298	106373646	7	C	T	0.545	0.057	0.007	1.6E-14	trans
2135	THTPA	Q9BU02	Thiamine-triphosphatase	rs28403575	24025340	14	C	T	0.912	0.750	0.013	0.0E+00	cis
2136	THY1	P04216	Thy-1 membrane glycoprotein	rs603424	102075479	10	G	A	0.834	0.081	0.010	8.1E-17	trans
2136	THY1	P04216	Thy-1 membrane glycoprotein	rs55933700	119293913	11	G	T	0.975	1.094	0.025	0.0E+00	cis
2136	THY1	P04216	Thy-1 membrane glycoprotein	rs186021206	7069412	17	A	G	0.006	0.350	0.049	1.6E-12	trans
2137	TIA1	P31483	Nucleolysin TIA-1 isoform p40	rs1354034	56849749	3	C	T	0.604	0.085	0.008	4.6E-28	trans
2137	TIA1	P31483	Nucleolysin TIA-1 isoform p40	rs11502185	180258	11	C	T	0.260	0.076	0.010	8.4E-15	trans
2138	TIE1	P35590	Tyrosine-protein kinase receptor Tie-1	rs4660729	43767900	1	G	C	0.609	0.191	0.007	5.0E-154	cis
2138	TIE1	P35590	Tyrosine-protein kinase receptor Tie-1	rs10935473	98416900	3	G	T	0.560	0.222	0.007	1.8E-211	trans
2138	TIE1	P35590	Tyrosine-protein kinase receptor Tie-1	rs2962006	57448076	5	G	C	0.613	0.051	0.007	2.2E-12	trans
2138	TIE1	P35590	Tyrosine-protein kinase receptor Tie-1	rs9271325	32582513	6	C	G	0.528	0.054	0.007	2.8E-14	trans
2138	TIE1	P35590	Tyrosine-protein kinase receptor Tie-1	rs4841133	9183664	8	A	G	0.092	0.096	0.012	2.8E-15	trans
2138	TIE1	P35590	Tyrosine-protein kinase receptor Tie-1	rs8176743	136131415	9	T	C	0.061	0.897	0.015	0.0E+00	trans
2138	TIE1	P35590	Tyrosine-protein kinase receptor Tie-1	rs56278466	17875857	10	G	T	0.661	0.065	0.007	1.2E-18	trans
2138	TIE1	P35590	Tyrosine-protein kinase receptor Tie-1	rs174564	61588305	11	G	A	0.349	0.059	0.007	7.1E-16	trans
2138	TIE1	P35590	Tyrosine-protein kinase receptor Tie-1	rs34434834	126307796	11	A	G	0.033	0.164	0.020	6.6E-17	trans
2138	TIE1	P35590	Tyrosine-protein kinase receptor Tie-1	rs878381	138032	16	C	T	0.142	0.143	0.010	1.1E-45	trans
2138	TIE1	P35590	Tyrosine-protein kinase receptor Tie-1	rs186021206	7069412	17	A	G	0.006	0.520	0.048	4.5E-27	trans
2138	TIE1	P35590	Tyrosine-protein kinase receptor Tie-1	rs4015	49253389	19	C	T	0.805	0.067	0.009	1.8E-14	trans
2139	TIGAR	Q9NQ88	Fructose-2,6-bisphosphatase TIGAR	rs4632248	54324995	19	T	G	0.209	0.075	0.010	3.6E-15	trans
2140	TIGIT	Q495A1	T-cell immunoreceptor with Ig and ITIM domains	rs6792290	114013194	3	G	C	0.290	0.095	0.008	2.7E-29	cis
2141	TIMD4	Q96H15	T-cell immunoglobulin and mucin domain-containing protein 4	rs1060622	93620393	1	A	G	0.621	0.070	0.007	5.9E-22	trans
2141	TIMD4	Q96H15	T-cell immunoglobulin and mucin domain-containing protein 4	rs1878051	235094643	1	T	A	0.669	0.055	0.008	3.7E-13	trans
2141	TIMD4	Q96H15	T-cell immunoglobulin and mucin domain-containing protein 4	rs10189685	203488449	2	A	G	0.296	0.123	0.008	1.5E-56	trans
2141	TIMD4	Q96H15	T-cell immunoglobulin and mucin domain-containing protein 4	rs11395592	69842341	3	A	AT	0.573	0.050	0.007	2.8E-12	trans
2141	TIMD4	Q96H15	T-cell immunoglobulin and mucin domain-containing protein 4	rs115216147	70665526	5	A	T	0.066	0.110	0.015	8.5E-14	trans
2141	TIMD4	Q96H15	T-cell immunoglobulin and mucin domain-containing protein 4	rs38029	96353223	5	C	G	0.439	0.065	0.007	1.7E-19	trans
2141	TIMD4	Q96H15	T-cell immunoglobulin and mucin domain-containing protein 4	rs4704826	156392082	5	C	A	0.362	0.232	0.007	5.1E-211	cis
2141	TIMD4	Q96H15	T-cell immunoglobulin and mucin domain-containing protein 4	rs9270270	32557208	6	T	C	0.160	0.149	0.010	1.3E-49	trans
2141	TIMD4	Q96H15	T-cell immunoglobulin and mucin domain-containing protein 4	rs10456852	106356046	6	T	C	0.131	0.105	0.011	5.2E-23	trans
2141	TIMD4	Q96H15	T-cell immunoglobulin and mucin domain-containing protein 4	rs2737245	116658583	8	T	G	0.282	0.056	0.008	1.8E-12	trans
2141	TIMD4	Q96H15	T-cell immunoglobulin and mucin domain-containing protein 4	rs2142306	134470631	8	C	T	0.412	0.063	0.007	2.5E-18	trans
2141	TIMD4	Q96H15	T-cell immunoglobulin and mucin domain-containing protein 4	rs2068888	94839642	10	A	G	0.450	0.061	0.007	1.1E-17	trans
2141	TIMD4	Q96H15	T-cell immunoglobulin and mucin domain-containing protein 4	rs72823014	115786236	10	A	G	0.127	0.083	0.011	4.9E-15	trans
2141	TIMD4	Q96H15	T-cell immunoglobulin and mucin domain-containing protein 4	rs10769256	47378396	11	C	T	0.600	0.063	0.007	1.5E-17	trans
2141	TIMD4	Q96H15	T-cell immunoglobulin and mucin domain-containing protein 4	rs112771035	126225876	11	G	C	0.071	0.495	0.014	2.6E-273	trans
2141	TIMD4	Q96H15	T-cell immunoglobulin and mucin domain-containing protein 4	rs3764261	56993324	16	A	C	0.325	0.055	0.008	3.7E-13	trans
2141	TIMD4	Q96H15	T-cell immunoglobulin and mucin domain-containing protein 4	rs34417180	72219756	16	C	A	0.832	0.065	0.009	5.6E-12	trans
2141	TIMD4	Q96H15	T-cell immunoglobulin and mucin domain-containing protein 4	rs4843861	85950976	16	A	T	0.731	0.074	0.008	4.1E-20	trans
2141	TIMD4	Q96H15	T-cell immunoglobulin and mucin domain-containing protein 4	rs186021206	7069412	17	A	G	0.006	1.131	0.049	3.0E-119	trans
2141	TIMD4	Q96H15	T-cell immunoglobulin and mucin domain-containing protein 4	rs601338	49206674	19	A	G	0.509	0.049	0.007	3.4E-12	trans
2141	TIMD4	Q96H15	T-cell immunoglobulin and mucin domain-containing protein 4	rs2143876	39119074	20	C	T	0.136	0.090	0.010	4.3E-18	trans
2141	TIMD4	Q96H15	T-cell immunoglobulin and mucin domain-containing protein 4	rs66817580	42689637	21	T	G	0.153	0.158	0.010	5.8E-58	trans
2141	TIMD4	Q96H15	T-cell immunoglobulin and mucin domain-containing protein 4	rs3848900	65238011	X	G	A	0.152	0.058	0.008	1.1E-12	trans
2142	TIMM10	P62072	Mitochondrial import inner membrane translocase subunit Tim10	rs1354034	56849749	3	C	T	0.604	0.063	0.008	2.4E-16	trans
2142	TIMM10	P62072	Mitochondrial import inner membrane translocase subunit Tim10	rs2848626	57283988	11	C	T	0.233	0.215	0.009	8.2E-128	cis
2143	TIMM8A	O60220	Mitochondrial import inner membrane translocase subunit Tim8 A	rs6993770	106581528	8	A	T	0.713	0.059	0.008	9.9E-13	trans
2144	TIMP1	P01033	Metalloproteinase inhibitor 1	rs1775815	118148830	1	T	G	0.571	0.066	0.008	1.7E-18	trans
2144	TIMP1	P01033	Metalloproteinase inhibitor 1	rs12407843	156873727	1	A	G	0.110	0.098	0.012	1.4E-16	trans
2144	TIMP1	P01033	Metalloproteinase inhibitor 1	rs9380143	29802045	6	C	T	0.716	0.096	0.008	1.5E-31	trans
2144	TIMP1	P01033	Metalloproteinase inhibitor 1	rs6993770	106581528	8	A	T	0.713	0.115	0.008	5.1E-45	trans
2144	TIMP1	P01033	Metalloproteinase inhibitor 1	rs1654425	55538980	19	C	T	0.834	0.113	0.010	1.5E-29	trans
2145	TIMP2	P16035	Metalloproteinase inhibitor 2	rs76685449	52570330	3	G	T	0.023	0.180	0.025	9.5E-13	trans
2145	TIMP2	P16035	Metalloproteinase inhibitor 2	rs75314265	32560132	6	C	T	0.013	0.508	0.033	2.1E-52	trans
2145	TIMP2	P16035	Metalloproteinase inhibitor 2	rs8066695	76913843	17	C	T	0.455	0.093	0.008	1.1E-34	cis
2146	TIMP3	P35625	Metalloproteinase inhibitor 3	rs2274319	156450873	1	T	C	0.347	0.063	0.007	7.3E-19	trans
2146	TIMP3	P35625	Metalloproteinase inhibitor 3	rs13412535	224874874	2	G	A	0.769	0.057	0.008	8.7E-13	trans
2146	TIMP3	P35625	Metalloproteinase inhibitor 3	rs1354034	56849749	3	T	C	0.396	0.047	0.007	6.2E-12	trans
2146	TIMP3	P35625	Metalloproteinase inhibitor 3	rs11734099	6891435	4	A	G	0.174	0.077	0.009	2.8E-18	trans
2146	TIMP3	P35625	Metalloproteinase inhibitor 3	rs114694170	88180196	5	C	T	0.060	0.173	0.014	8.3E-34	trans
2146	TIMP3	P35625	Metalloproteinase inhibitor 3	rs11953411	150798334	5	G	A	0.394	0.061	0.007	1.6E-18	trans
2146	TIMP3	P35625	Metalloproteinase inhibitor 3	rs6961069	80218961	7	T	C	0.403	0.049	0.007	1.5E-12	trans
2146	TIMP3	P35625	Metalloproteinase inhibitor 3	rs61469632	135861990	9	T	C	0.937	0.123	0.014	6.0E-19	trans
2146	TIMP3	P35625	Metalloproteinase inhibitor 3	rs7896518	65104500	10	G	A	0.424	0.082	0.007	1.3E-32	trans
2146	TIMP3	P35625	Metalloproteinase inhibitor 3	rs17655730	270715	11	C	T	0.245	0.056	0.008	7.5E-13	trans
2146	TIMP3	P35625	Metalloproteinase inhibitor 3	rs12445050	81870969	16	T	C	0.138	0.086	0.010	6.5E-19	trans
2146	TIMP3	P35625	Metalloproteinase inhibitor 3	rs74035509	88567333	16	T	C	0.078	0.090	0.013	1.3E-12	trans
2146	TIMP3	P35625	Metalloproteinase inhibitor 3	rs892090	55539072	19	G	T	0.834	0.136	0.009	7.9E-51	trans
2146	TIMP3	P35625	Metalloproteinase inhibitor 3	rs6081569	19303786	20	C	G	0.368	0.050	0.007	8.9E-13	trans
2146	TIMP3	P35625	Metalloproteinase inhibitor 3	rs5749504	33162831	22	C	T	0.274	0.652	0.008	0.0E+00	cis
2147	TIMP4	Q99727	Metalloproteinase inhibitor 4	rs78058190	219699999	2	G	A	0.949	0.119	0.018	1.4E-11	trans
2147	TIMP4	Q99727	Metalloproteinase inhibitor 4	rs2943649	227105691	2	C	G	0.351	0.060	0.007	8.7E-17	trans
2147	TIMP4	Q99727	Metalloproteinase inhibitor 4	rs184262	12134740	3	G	A	0.828	0.447	0.010	0.0E+00	cis
2147	TIMP4	Q99727	Metalloproteinase inhibitor 4	rs34579624	32630119	6	A	G	0.146	0.088	0.011	6.2E-17	trans
2147	TIMP4	Q99727	Metalloproteinase inhibitor 4	rs1358980	43764551	6	C	T	0.520	0.058	0.007	5.0E-17	trans
2147	TIMP4	Q99727	Metalloproteinase inhibitor 4	rs10405357	54759666	19	T	C	0.564	0.048	0.007	9.2E-12	trans
2148	TINAGL1	Q9GZM7	Tubulointerstitial nephritis antigen-like	rs78265569	2146165	1	C	A	0.911	0.089	0.013	7.2E-12	trans
2148	TINAGL1	Q9GZM7	Tubulointerstitial nephritis antigen-like	rs2229474	22182115	1	A	G	0.014	0.618	0.031	1.0E-87	trans
2148	TINAGL1	Q9GZM7	Tubulointerstitial nephritis antigen-like	rs945214	32047305	1	A	G	0.386	0.085	0.008	2.7E-28	cis
2148	TINAGL1	Q9GZM7	Tubulointerstitial nephritis antigen-like	rs7648959	159562497	3	G	A	0.297	0.088	0.008	2.7E-28	trans
2148	TINAGL1	Q9GZM7	Tubulointerstitial nephritis antigen-like	rs56278466	17875857	10	G	T	0.661	0.062	0.008	5.9E-16	trans
2148	TINAGL1	Q9GZM7	Tubulointerstitial nephritis antigen-like	rs9583474	110895400	13	A	C	0.789	0.077	0.009	6.7E-18	trans
2148	TINAGL1	Q9GZM7	Tubulointerstitial nephritis antigen-like	rs8124907	60913127	20	A	G	0.759	0.066	0.009	1.1E-14	trans
2149	TJAP1	Q5JTD0	Tight junction-associated protein 1	rs1354034	56849749	3	C	T	0.604	0.062	0.007	1.2E-16	trans
2150	TK1	P04183	Thymidine kinase, cytosolic	rs9697210	131468740	9	A	G	0.146	0.091	0.011	8.0E-17	trans
2150	TK1	P04183	Thymidine kinase, cytosolic	rs144204502	76183233	17	C	T	0.987	0.350	0.035	4.0E-24	cis
2151	TLR1	Q15399	Toll-like receptor 1	rs5743618	38798648	4	A	C	0.224	0.503	0.009	0.0E+00	cis

2151	TLR1	Q15399	Toll-like receptor 1	rs56278466	17875857	10	G	T	0.661	0.068	0.008	9.6E-19	trans
2152	TLR3	O15455	Toll-like receptor 3	rs61804206	161658821	1	G	A	0.105	0.041	0.006	1.7E-11	trans
2152	TLR3	O15455	Toll-like receptor 3	rs3775291	187004074	4	C	T	0.703	1.151	0.008	0.0E+00	cis
2152	TLR3	O15455	Toll-like receptor 3	rs635634	136155000	9	C	T	0.816	0.042	0.005	3.1E-18	trans
2152	TLR3	O15455	Toll-like receptor 3	rs56278466	17875857	10	G	T	0.661	0.042	0.004	1.1E-26	trans
2152	TLR3	O15455	Toll-like receptor 3	rs16940186	86009740	16	T	C	0.851	0.036	0.005	7.0E-12	trans
2152	TLR3	O15455	Toll-like receptor 3	rs4760	44153100	19	G	A	0.156	0.086	0.005	2.2E-63	trans
2152	TLR3	O15455	Toll-like receptor 3	rs738409	44324727	22	G	C	0.217	0.056	0.005	2.1E-35	trans
2153	TLR4	O00206	Toll-like receptor 4	rs4986790	120475302	9	A	G	0.940	0.327	0.016	7.2E-90	cis
2154	TMCO5A	Q8N6Q1	Transmembrane and coiled-coil domain-containing protein 5A	rs516246	49206172	19	T	C	0.509	0.055	0.008	9.4E-13	trans
2155	TMED8	Q6PL24	Protein TMED8	rs1354034	56849749	3	C	T	0.605	0.052	0.008	1.1E-11	trans
2155	TMED8	Q6PL24	Protein TMED8	rs6993770	106581528	8	A	T	0.713	0.057	0.008	5.5E-12	trans
2155	TMED8	Q6PL24	Protein TMED8	rs11620921	77939965	14	A	G	0.397	0.066	0.008	5.3E-18	cis
2156	TMEM106A	Q96A25	Transmembrane protein 106A	rs4950771	202111779	1	T	G	0.066	0.198	0.015	3.3E-39	trans
2156	TMEM106A	Q96A25	Transmembrane protein 106A	rs9857570	58280690	3	A	G	0.676	0.099	0.008	4.4E-35	trans
2156	TMEM106A	Q96A25	Transmembrane protein 106A	rs62443342	38265748	7	A	T	0.752	0.083	0.009	5.2E-22	trans
2156	TMEM106A	Q96A25	Transmembrane protein 106A	rs1293303	11720227	8	G	C	0.554	0.113	0.008	5.2E-50	trans
2156	TMEM106A	Q96A25	Transmembrane protein 106A	rs6993770	106581528	8	A	T	0.714	0.075	0.008	1.1E-19	trans
2156	TMEM106A	Q96A25	Transmembrane protein 106A	rs10812628	27631390	9	G	A	0.736	0.080	0.009	9.1E-21	trans
2156	TMEM106A	Q96A25	Transmembrane protein 106A	rs7080386	65048306	10	A	C	0.412	0.123	0.008	2.7E-58	trans
2156	TMEM106A	Q96A25	Transmembrane protein 106A	rs72701845	93217023	14	G	A	0.962	0.384	0.020	1.7E-79	trans
2156	TMEM106A	Q96A25	Transmembrane protein 106A	rs34687590	41301286	17	T	TG	0.615	0.107	0.008	9.5E-41	cis
2156	TMEM106A	Q96A25	Transmembrane protein 106A	rs892090	55539072	19	G	T	0.834	0.091	0.010	3.3E-19	trans
2156	TMEM106A	Q96A25	Transmembrane protein 106A	rs142598052	24912633	20	T	C	0.994	0.367	0.048	2.2E-14	trans
2157	TMEM132A	Q24JP5	Transmembrane protein 132A	rs11379371	60702412	11	AG	A	0.401	0.613	0.008	0.0E+00	cis
2158	TMEM25	Q86YD3	Transmembrane protein 25	rs45558732	118402962	11	G	T	0.966	1.020	0.021	0.0E+00	cis
2158	TMEM25	Q86YD3	Transmembrane protein 25	rs186021206	7069412	17	A	G	0.006	0.382	0.051	4.8E-14	trans
2159	TMPRSS11D	O60235	Transmembrane protease serine 11D	rs148060334	68705712	4	A	G	0.964	0.342	0.020	3.4E-65	cis
2160	TMPRSS15	P98073	Enteropeptidase	rs11134475	156399950	5	G	A	0.639	0.076	0.008	1.3E-22	trans
2160	TMPRSS15	P98073	Enteropeptidase	rs113981974	41377702	8	G	C	0.111	0.097	0.012	4.5E-16	trans
2160	TMPRSS15	P98073	Enteropeptidase	rs708686	5840619	19	T	C	0.267	0.114	0.008	2.7E-41	trans
2160	TMPRSS15	P98073	Enteropeptidase	rs438811	45416741	19	T	C	0.237	0.059	0.009	1.5E-11	trans
2160	TMPRSS15	P98073	Enteropeptidase	rs492602	49206417	19	A	G	0.491	0.182	0.008	6.8E-129	trans
2160	TMPRSS15	P98073	Enteropeptidase	rs2824805	19770734	21	C	G	0.173	0.126	0.010	1.0E-37	cis
2161	TMPRSS5	Q9H3S3	Transmembrane protease serine 5	rs35383942	201437832	1	T	C	0.062	0.132	0.013	2.2E-25	trans
2161	TMPRSS5	Q9H3S3	Transmembrane protease serine 5	rs12024555	222147845	1	A	G	0.195	0.054	0.008	1.9E-12	trans
2161	TMPRSS5	Q9H3S3	Transmembrane protease serine 5	rs199927946	112750714	2	T	TGTG	0.621	0.044	0.006	7.3E-12	trans
2161	TMPRSS5	Q9H3S3	Transmembrane protease serine 5	rs1082409	98757074	3	G	T	0.662	0.051	0.006	2.5E-15	trans
2161	TMPRSS5	Q9H3S3	Transmembrane protease serine 5	rs67670470	152330739	3	A	C	0.336	0.049	0.006	3.5E-14	trans
2161	TMPRSS5	Q9H3S3	Transmembrane protease serine 5	rs4686658	193722967	3	C	T	0.224	0.052	0.007	1.7E-12	trans
2161	TMPRSS5	Q9H3S3	Transmembrane protease serine 5	rs60028714	86393300	4	C	T	0.378	0.060	0.006	3.7E-21	trans
2161	TMPRSS5	Q9H3S3	Transmembrane protease serine 5	rs9386298	96487569	6	T	A	0.497	0.048	0.006	8.4E-15	trans
2161	TMPRSS5	Q9H3S3	Transmembrane protease serine 5	rs116923669	33109581	9	G	A	0.934	0.148	0.013	4.5E-32	trans
2161	TMPRSS5	Q9H3S3	Transmembrane protease serine 5	rs56278466	17875857	10	G	T	0.661	0.067	0.006	2.4E-25	trans
2161	TMPRSS5	Q9H3S3	Transmembrane protease serine 5	rs10887793	89990905	10	T	G	0.449	0.052	0.006	2.2E-17	trans
2161	TMPRSS5	Q9H3S3	Transmembrane protease serine 5	rs7114195	113561421	11	C	A	0.639	0.785	0.008	0.0E+00	cis
2161	TMPRSS5	Q9H3S3	Transmembrane protease serine 5	rs28404455	50815358	13	C	T	0.093	0.084	0.010	1.4E-15	trans
2161	TMPRSS5	Q9H3S3	Transmembrane protease serine 5	rs72681869	50655357	14	C	G	0.011	0.209	0.029	7.2E-13	trans
2161	TMPRSS5	Q9H3S3	Transmembrane protease serine 5	rs3742780	75505008	14	G	C	0.524	0.052	0.006	1.9E-17	trans
2161	TMPRSS5	Q9H3S3	Transmembrane protease serine 5	rs186021206	7069412	17	A	G	0.006	0.555	0.042	4.1E-40	trans
2161	TMPRSS5	Q9H3S3	Transmembrane protease serine 5	rs504549	37437201	19	C	T	0.621	0.046	0.006	2.4E-13	trans
2161	TMPRSS5	Q9H3S3	Transmembrane protease serine 5	rs2836683	40197438	21	C	T	0.338	0.045	0.006	2.2E-12	trans
2162	TMSB10	P63313	Thymosin beta-10	rs1354034	56849749	3	C	T	0.604	0.055	0.008	4.8E-13	trans
2162	TMSB10	P63313	Thymosin beta-10	rs342293	106372219	7	C	G	0.541	0.057	0.008	2.6E-14	trans
2162	TMSB10	P63313	Thymosin beta-10	rs6993770	106581528	8	A	T	0.714	0.060	0.008	4.6E-13	trans
2162	TMSB10	P63313	Thymosin beta-10	rs11502185	180258	11	C	T	0.259	0.073	0.010	2.8E-14	trans
2162	TMSB10	P63313	Thymosin beta-10	rs4632248	54324995	19	T	G	0.209	0.130	0.009	2.9E-44	trans
2163	TNC	P24821	Tenascin	rs1892534	66105944	1	C	T	0.626	0.051	0.007	9.1E-13	trans
2163	TNC	P24821	Tenascin	rs2646260	238277795	2	A	G	0.756	0.060	0.008	2.0E-13	trans
2163	TNC	P24821	Tenascin	rs4686846	186772411	3	T	C	0.233	0.067	0.008	5.4E-16	trans
2163	TNC	P24821	Tenascin	rs13159365	135389433	5	T	C	0.510	0.194	0.007	2.7E-167	trans
2163	TNC	P24821	Tenascin	rs113760175	22343592	6	A	G	0.071	0.104	0.014	3.6E-14	trans
2163	TNC	P24821	Tenascin	rs1990790	130033630	7	C	T	0.487	0.052	0.007	1.8E-13	trans
2163	TNC	P24821	Tenascin	rs2685412	27810603	8	G	C	0.270	0.380	0.008	0.0E+00	trans
2163	TNC	P24821	Tenascin	rs4263799	72394991	8	T	C	0.480	0.082	0.007	5.8E-29	trans
2163	TNC	P24821	Tenascin	rs112286501	100027593	8	T	C	0.044	0.149	0.018	6.0E-17	trans
2163	TNC	P24821	Tenascin	rs7021057	117897993	9	T	C	0.706	0.244	0.008	4.1E-213	cis
2163	TNC	P24821	Tenascin	rs7949566	126285301	11	A	G	0.421	0.080	0.007	1.5E-29	trans
2163	TNC	P24821	Tenascin	rs760715	39862343	22	C	T	0.247	0.104	0.009	1.7E-34	trans
2164	TNF	P01375	Tumor necrosis factor	rs4645843	31544562	6	C	T	0.999	1.446	0.112	4.4E-38	cis
2164	TNF	P01375	Tumor necrosis factor	rs7161799	58770523	15	T	C	0.076	0.237	0.014	5.1E-63	trans
2165	TNFAIP2	Q03169	Tumor necrosis factor alpha-induced protein 2	rs342293	106372219	7	C	G	0.540	0.086	0.007	7.2E-31	trans
2165	TNFAIP2	Q03169	Tumor necrosis factor alpha-induced protein 2	rs12342201	95894964	9	G	A	0.524	0.055	0.007	1.4E-13	trans
2166	TNFAIP8	O95379	Tumor necrosis factor alpha-induced protein 8	rs1035376	118648250	5	A	G	0.882	0.123	0.012	6.5E-25	cis
2167	TNFAIP8L2	Q6P589	Tumor necrosis factor alpha-induced protein 8-like protein 2	rs4970991	151004003	1	C	T	0.793	0.066	0.009	1.3E-12	cis
2167	TNFAIP8L2	Q6P589	Tumor necrosis factor alpha-induced protein 8-like protein 2	rs1354034	56849749	3	C	T	0.604	0.065	0.008	2.6E-17	trans
2167	TNFAIP8L2	Q6P589	Tumor necrosis factor alpha-induced protein 8-like protein 2	rs342299	106373718	7	C	T	0.545	0.062	0.008	6.0E-16	trans
2168	TNFRSF10A	O00220	Tumor necrosis factor receptor superfamily member 10A	rs79287178	172294500	3	A	G	0.030	0.249	0.020	6.5E-35	trans
2168	TNFRSF10A	O00220	Tumor necrosis factor receptor superfamily member 10A	rs1632984	29773392	6	A	T	0.854	0.069	0.009	1.5E-13	trans
2168	TNFRSF10A	O00220	Tumor necrosis factor receptor superfamily member 10A	rs13278062	23082971	8	G	T	0.498	0.462	0.007	0.0E+00	cis
2168	TNFRSF10A	O00220	Tumor necrosis factor receptor superfamily member 10A	rs1801689	64210580	17	C	A	0.030	0.129	0.019	1.7E-11	trans
2169	TNFRSF10B	O14763	Tumor necrosis factor receptor superfamily member 10B	rs231996	172273735	3	C	A	0.240	0.060	0.008	1.4E-14	trans
2169	TNFRSF10B	O14763	Tumor necrosis factor receptor superfamily member 10B	rs1105944	22885109	8	G	A	0.896	0.538	0.011	0.0E+00	cis
2170	TNFRSF10C	O14798	Tumor necrosis factor receptor superfamily member 10C	rs3917932	36943916	1	C	G	0.424	0.045	0.006	3.2E-14	trans
2170	TNFRSF10C	O14798	Tumor necrosis factor receptor superfamily member 10C	rs3014874	153337943	1	G	A	0.736	0.045	0.007	1.3E-11	trans
2170	TNFRSF10C	O14798	Tumor necrosis factor receptor superfamily member 10C	rs5030738	161599654	1	T	G	0.017	0.200	0.023	1.1E-18	trans
2170	TNFRSF10C	O14798	Tumor necrosis factor receptor superfamily member 10C	rs6723921	62531732	2	G	A	0.613	0.047	0.006	5.0E-15	trans
2170	TNFRSF10C	O14798	Tumor necrosis factor receptor superfamily member 10C	rs10182318	197793304	2	T	C	0.102	0.088	0.010	8.2E-20	trans
2170	TNFRSF10C	O14798	Tumor necrosis factor receptor superfamily member 10C	rs12497115	47220341	3	T	C	0.175	0.053	0.008	5.2E-12	trans
2170	TNFRSF10C	O14798	Tumor necrosis factor receptor superfamily member 10C	rs3774315	172231986	3	G	A	0.273	0.080	0.007	4.5E-34	trans
2170	TNFRSF10C	O14798	Tumor necrosis factor receptor superfamily member 10C	rs2249741	31240712	6	C	A	0.504	0.056	0.006	5.1E-22	trans
2170	TNFRSF10C	O14798	Tumor necrosis factor receptor superfamily member 10C	rs149110519	144385777	6	T	C	0.036	0.134	0.016	1.5E-17	trans
2170	TNFRSF10C	O14798	Tumor necrosis factor receptor superfamily member 10C	rs1010366	7229826	7	C	T	0.317	0.063	0.006	5.3E-24	trans
2170	TNFRSF10C	O14798	Tumor necrosis factor receptor superfamily member 10C	rs445	92408370	7	C	T	0.905	0.091	0.010	4.8E-20	trans
2170	TNFRSF10C	O14798	Tumor necrosis factor receptor superfamily member 10C	rs7846314	61650831	8	T	A	0.186	0.059	0.008	1.9E-14	trans
2170	TNFRSF10C	O14798	Tumor necrosis factor receptor superfamily member 10C	rs3124753	136249929	9	G	A	0.528	0.068	0.006	1.7E-31	trans
2170	TNFRSF10C	O14798	Tumor necrosis factor receptor superfamily member 10C	rs2241244	38153554	17	G	C	0.463	0.091	0.006	5.7E-55	trans
2170	TNFRSF10C	O14798	Tumor necrosis factor receptor superfamily member 10C	rs4760	44153100	19	A	G	0.844	1.185	0.010	0.0E+00	trans
2171	TNFRSF11A	Q9Y6Q6	Tumor necrosis factor receptor superfamily member 11A	rs9275587	32680379	6	T	C	0.527	0.046	0.007	5.9E-12	trans
2171	TNFRSF11A	Q9Y6Q6	Tumor necrosis factor receptor superfamily member 11A	rs342293	106372219	7	G	C	0.459	0.053	0.007	6.8E-16	trans
2171	TNFRSF11A	Q9Y6Q6	Tumor necrosis factor receptor superfamily member 11A	rs141129381	100691900	9	TG	T	0.363	0.047	0.007	6.3E-12	trans
2171	TNFRSF11A	Q9Y6Q6	Tumor necrosis factor receptor superfamily member 11A	rs7080386	65048306	10	A	C	0.412	0.053	0.007	3.1E-15	trans
2171	TNFRSF11A	Q9Y6Q6	Tumor necrosis factor receptor superfamily member 11A	rs12946745	74483109	17	G	C	0.335	0.049	0.007	4.0E-12	trans
2171	TNFRSF11A	Q9Y6Q6	Tumor necrosis factor receptor superfamily member 11A	rs113339733	60018329	18	G	A	0.807	0.598	0.009	0.0E+00	cis
2171	TNFRSF11A	Q9Y6Q6	Tumor necrosis factor receptor superfamily member 11A	rs601338	49206674	19	G	A	0.491	0.122	0.007	2.4E-74	trans
2172	TNFRSF11B	O00300	Tumor necrosis factor receptor superfamily member 11B	rs114165349	27021913	1	G	C	0.977	0.234	0.023	2.8E-25	trans
2172	TNFRSF11B	O00300	Tumor necrosis factor receptor superfamily member 11B	rs79287178	172294500	3	A	G	0.030	0.205	0.021	6.8E-23	trans
2172	TNFRSF11B	O00300	Tumor necrosis factor receptor superfamily member 11B	rs1013495	81140253	10	T	C	0.455	0.056	0.007	2.8E-16	trans
2172	TNFRSF11B	O00300	Tumor necrosis factor receptor superfamily member 11B	rs7927191	16252982	11	T	C	0.734	0.060	0.008	3.3E-15	trans
2172	TNFRSF11B	O00300	Tumor necrosis factor receptor superfamily member 11B	rs3825568	69260588	14	C	T	0.540	0.047	0.007	2.3E-12	trans
2172	TNFRSF11B	O00300	Tumor necrosis factor receptor superfamily member 11B	rs28929474	94844947	14	T	C	0.021	0.161	0.024	9.5E-12	trans
2172	TNFRSF11B	O00300	Tumor necrosis factor receptor superfamily member 11B	rs200489612	7106378	17	A	G	0.005	0.358	0.051	2.3E-12	trans
2172	TNFRSF11B	O00300	Tumor necrosis factor receptor superfamily member 11B	rs704	26694861	17	G	A	0.527	0.198	0.007	3.7E-186	trans
2172	TNFRSF11B	O00300	Tumor necrosis factor receptor superfamily member 11B	rs77542162	67081278	17	G	A	0.023	0.157	0.023	6.8E-12	trans
2172	TNFRSF11B	O00300	Tumor necrosis factor receptor superfamily member 11B	rs3747207	44324855	22	A	G	0.215	0.075	0.008	6.7E-20	trans
2173	TNFRSF12A	Q9NP84	Tumor necrosis factor receptor superfamily member 12A	rs964184	116648917	11	G	C	0.133	0.078	0.010	6.0E-14	trans
2173	TNFRSF12A	Q9NP84	Tumor necrosis factor receptor superfamily member 12A	rs74758164	3070628	16	T	C	0.039	0.159	0.018	2.4E-18	cis
2173	TNFRSF12A	Q9NP84	Tumor necrosis factor receptor superfamily member 12A	rs77542162	67081278	17	G	A	0.023	0.221	0.024	6.3E-21	trans
2174	TNFRSF13B	O14836	Tumor necrosis factor receptor superfamily member 13B	rs1793891	31221698	6	C	T	0.732	0.059	0.008	1.4E-12	trans
2174	TNFRSF13B	O14836	Tumor necrosis factor receptor superfamily member 13B	rs10806425	90926612	6	C	A	0.586	0.053	0.007	1.9E-12	trans
2174	TNFRSF13B	O14836	Tumor necrosis factor receptor superfamily member 13B	rs3804329	106686427	6	G	A	0.204	0.089	0.009	3.2E-22	trans
2174	TNFRSF13B	O14836	Tumor necrosis factor receptor superfamily member 13B	rs73169672	150948078	7	T	A	0.114	0.089	0.012	1.1E-14	trans
2174	TNFRSF13B	O14836	Tumor necrosis factor receptor superfamily member 13B	rs1483572	79635243	8	A	C	0.311	0.054	0.008	8.3E-12	trans
2174	TNFRSF13B	O14836	Tumor necrosis factor receptor superfamily member 13B	rs374039502	108960385	13	A	T	0.021	0.714	0.028	2.4E-145	trans
2174	TNFRSF13B	O14836	Tumor necrosis factor receptor superfamily member 13B	rs9989163	103235012	14	G	A	0.525	0.054	0.007	2.1E-13	trans
2174	TNFRSF13B	O14836	Tumor necrosis factor receptor superfamily member 13B	rs34562254	16842991	17	G	A	0.903	0.174	0.012	1.3E-44	cis
2174	TNFRSF13B	O14836	Tumor necrosis factor receptor superfamily member 13B	rs12975366	54759361	19	T	C	0.605	0.061	0.008	5.2E-16	trans
2174	TNFRSF13B	O14836	Tumor necrosis factor receptor superfamily member 13B	rs6053024	4975805	20	T	G	0.390	0.056	0.008	1.4E-13	trans
2174	TNFRSF13B	O14836	Tumor necrosis factor receptor superfamily member 13B	rs73165110	42291856	22	G	A	0.921	0.189	0.014	3.6E-43	trans
2175	TNFRSF13C	Q96RJ3	Tumor necrosis factor receptor superfamily member 13C	rs17056298	158268679	5	C	G	0.925	0.130	0.014	9.7E-22	trans
2175	TNFRSF13C	Q96RJ3	Tumor necrosis factor receptor superfamily member 13C	rs17198888	31266015	6	G	A	0.191	0.072	0.009	1.8E-15	trans
2175	TNFRSF13C	Q96RJ3	Tumor necrosis factor receptor superfamily member 13C	rs2395904	130617819	8	T	G	0.725	0.054	0.008	1.3E-11	trans
2175	TNFRSF13C	Q96RJ3	Tumor necrosis factor receptor superfamily member 13C	rs12784975	98380137	10	T	C	0.809	0.073	0.009	6.6E-16	trans
2175	TNFRSF13C	Q96RJ3	Tumor necrosis factor receptor superfamily member 13C	rs374039502	108960385	13	A	T	0.021	0.217	0.027	3.7E-16	trans
2175	TNFRSF13C	Q96RJ3	Tumor necrosis factor receptor superfamily member 13C	rs13330176	86019087	16	A	T	0.239	0.059	0.008	2.2E-12	trans
2175	TNFRSF13C	Q96RJ3	Tumor necrosis factor receptor superfamily member 13C	rs34557412	16852187	17	G	A	0.007	0.667	0.043	4.0E-53	trans
2175	TNFRSF13C	Q96RJ3	Tumor necrosis factor receptor superfamily member 13C	rs35222145	38007321	17	T	G	0.508	0.073	0.007	4.4E-24	trans
2175	TNFRSF13C	Q96RJ3	Tumor necrosis factor receptor superfamily member 13C	rs17676919	60783729	18	A	G	0.903	0.082	0.012	7.0E-12	trans
2175	TNFRSF13C	Q96RJ3	Tumor necrosis factor receptor superfamily member 13C	rs1967330	17659683	19	T	C	0.567	0.050	0.007	2.5E-12	trans
2175	TNFRSF13C	Q96RJ3	Tumor necrosis factor receptor superfamily member 13C	rs28823595	3705133	20	A	G	0.087	0.088	0.013	3.2E-12	trans
2176	TNFRSF14	Q92956	Tumor necrosis factor receptor superfamily member 14	rs2495366	2488930	1	A	G	0.494	0.115	0.007	3.9E-54	cis
2176	TNFRSF14	Q92956	Tumor necrosis factor receptor superfamily member 14	rs61747728	179526214	1	T	C	0.038	0.134	0.019	3.8E-12	trans
2176	TNFRSF14	Q92956	Tumor necrosis factor receptor superfamily member 14	rs11926707	46925539	3	T	C	0.378	0.053	0.008	7.1E-12	trans

2176	TNFRSF14	Q92956	Tumor necrosis factor receptor superfamily member 14	rs342293	106372219	7	C	G	0.541	0.054	0.007	4.3E-13	trans
2176	TNFRSF14	Q92956	Tumor necrosis factor receptor superfamily member 14	rs6993770	106581528	8	A	T	0.713	0.063	0.008	1.2E-14	trans
2177	TNFRSF17	Q02223	Tumor necrosis factor receptor superfamily member 17	rs6759	227069737	1	T	C	0.515	0.059	0.007	4.8E-15	trans
2177	TNFRSF17	Q02223	Tumor necrosis factor receptor superfamily member 17	rs9799792	969322	4	T	C	0.443	0.065	0.008	2.7E-17	trans
2177	TNFRSF17	Q02223	Tumor necrosis factor receptor superfamily member 17	rs2647074	32574360	6	C	T	0.673	0.080	0.008	2.1E-23	trans
2177	TNFRSF17	Q02223	Tumor necrosis factor receptor superfamily member 17	rs61839660	6094697	10	T	C	0.097	0.085	0.013	1.3E-11	trans
2177	TNFRSF17	Q02223	Tumor necrosis factor receptor superfamily member 17	rs1265847	28929686	10	G	A	0.738	0.058	0.009	1.4E-11	trans
2177	TNFRSF17	Q02223	Tumor necrosis factor receptor superfamily member 17	rs3803286	103246470	14	A	G	0.334	0.087	0.008	8.6E-28	trans
2177	TNFRSF17	Q02223	Tumor necrosis factor receptor superfamily member 17	rs34562254	16842991	17	A	G	0.097	0.232	0.013	8.3E-76	trans
2177	TNFRSF17	Q02223	Tumor necrosis factor receptor superfamily member 17	rs9957535	60763298	18	G	A	0.916	0.091	0.013	1.3E-11	trans
2177	TNFRSF17	Q02223	Tumor necrosis factor receptor superfamily member 17	rs5754102	21916272	22	A	C	0.181	0.070	0.010	8.1E-13	trans
2178	TNFRSF19	Q9NS68	Tumor necrosis factor receptor superfamily member 19	rs6605317	197756208	3	A	T	0.227	0.064	0.008	1.3E-14	trans
2178	TNFRSF19	Q9NS68	Tumor necrosis factor receptor superfamily member 19	rs13146355	77412140	4	A	G	0.457	0.049	0.007	1.5E-12	trans
2178	TNFRSF19	Q9NS68	Tumor necrosis factor receptor superfamily member 19	rs61947047	24153909	13	T	C	0.309	0.259	0.008	1.3E-246	cis
2178	TNFRSF19	Q9NS68	Tumor necrosis factor receptor superfamily member 19	rs12434499	106352172	14	T	C	0.161	0.089	0.010	2.4E-19	trans
2178	TNFRSF19	Q9NS68	Tumor necrosis factor receptor superfamily member 19	rs35830321	20353027	16	C	CA	0.833	0.078	0.009	8.6E-17	trans
2178	TNFRSF19	Q9NS68	Tumor necrosis factor receptor superfamily member 19	rs2252576	42615293	21	T	C	0.244	0.147	0.008	1.4E-71	trans
2179	TNFRSF1A	P19438	Tumor necrosis factor receptor superfamily member 1A	rs61747728	179526214	1	T	C	0.038	0.165	0.019	1.7E-18	trans
2179	TNFRSF1A	P19438	Tumor necrosis factor receptor superfamily member 1A	rs13019008	9693736	2	A	G	0.514	0.073	0.007	1.1E-23	trans
2179	TNFRSF1A	P19438	Tumor necrosis factor receptor superfamily member 1A	rs10982156	117088064	9	A	T	0.069	0.176	0.015	2.3E-32	trans
2179	TNFRSF1A	P19438	Tumor necrosis factor receptor superfamily member 1A	rs8176672	136142185	9	T	C	0.061	0.123	0.015	2.3E-16	trans
2179	TNFRSF1A	P19438	Tumor necrosis factor receptor superfamily member 1A	rs28640218	20359267	16	G	T	0.809	0.075	0.009	3.0E-16	trans
2179	TNFRSF1A	P19438	Tumor necrosis factor receptor superfamily member 1A	rs516316	49206145	19	G	C	0.490	0.055	0.007	5.7E-14	trans
2180	TNFRSF1B	P20333	Tumor necrosis factor receptor superfamily member 1B	rs5746026	12253062	1	G	A	0.960	0.392	0.019	1.2E-96	cis
2180	TNFRSF1B	P20333	Tumor necrosis factor receptor superfamily member 1B	rs17162190	26872832	1	A	G	0.199	0.062	0.009	1.5E-11	trans
2180	TNFRSF1B	P20333	Tumor necrosis factor receptor superfamily member 1B	rs61747728	179526214	1	T	C	0.038	0.183	0.019	8.9E-22	trans
2180	TNFRSF1B	P20333	Tumor necrosis factor receptor superfamily member 1B	rs2239527	31509779	6	G	C	0.360	0.086	0.008	4.5E-30	trans
2180	TNFRSF1B	P20333	Tumor necrosis factor receptor superfamily member 1B	rs77924615	20392332	16	G	A	0.803	0.065	0.009	2.0E-12	trans
2180	TNFRSF1B	P20333	Tumor necrosis factor receptor superfamily member 1B	rs3814995	36342212	19	T	C	0.315	0.056	0.008	6.6E-13	trans
2181	TNFRSF21	O75509	Tumor necrosis factor receptor superfamily member 21	rs7528419	109817192	1	A	G	0.777	0.073	0.009	1.1E-16	trans
2181	TNFRSF21	O75509	Tumor necrosis factor receptor superfamily member 21	rs150816167	179571862	1	C	T	0.042	0.184	0.019	3.1E-23	trans
2181	TNFRSF21	O75509	Tumor necrosis factor receptor superfamily member 21	rs1260326	27730940	2	C	T	0.608	0.058	0.007	1.2E-14	trans
2181	TNFRSF21	O75509	Tumor necrosis factor receptor superfamily member 21	rs75166367	162964301	2	G	A	0.940	0.107	0.015	2.9E-12	trans
2181	TNFRSF21	O75509	Tumor necrosis factor receptor superfamily member 21	rs13107325	103188709	4	T	C	0.075	0.114	0.014	2.6E-16	trans
2181	TNFRSF21	O75509	Tumor necrosis factor receptor superfamily member 21	rs148598211	47254244	6	C	T	0.996	1.874	0.057	1.9E-240	cis
2181	TNFRSF21	O75509	Tumor necrosis factor receptor superfamily member 21	rs2298475	126278203	11	T	C	0.925	0.183	0.014	5.9E-40	trans
2181	TNFRSF21	O75509	Tumor necrosis factor receptor superfamily member 21	rs77924615	20392332	16	G	A	0.803	0.070	0.009	3.7E-14	trans
2181	TNFRSF21	O75509	Tumor necrosis factor receptor superfamily member 21	rs186021206	7069412	17	A	G	0.006	1.139	0.050	5.6E-114	trans
2181	TNFRSF21	O75509	Tumor necrosis factor receptor superfamily member 21	rs10445407	79261809	17	C	A	0.521	0.053	0.007	5.3E-13	trans
2182	TNFRSF4	P43489	Tumor necrosis factor receptor superfamily member 4	rs2524137	31264582	6	C	T	0.308	0.125	0.008	1.7E-55	trans
2182	TNFRSF4	P43489	Tumor necrosis factor receptor superfamily member 4	rs4946811	107420516	6	C	A	0.361	0.054	0.008	1.8E-12	trans
2182	TNFRSF4	P43489	Tumor necrosis factor receptor superfamily member 4	rs72778736	57301035	16	T	C	0.256	0.057	0.008	1.3E-11	trans
2183	TNFRSF6B	O95407	Tumor necrosis factor receptor superfamily member 6B	rs200607887	32386869	6	GT	G	0.017	0.219	0.028	1.2E-14	trans
2183	TNFRSF6B	O95407	Tumor necrosis factor receptor superfamily member 6B	rs77542162	67081278	17	G	A	0.023	0.204	0.024	1.2E-17	trans
2183	TNFRSF6B	O95407	Tumor necrosis factor receptor superfamily member 6B	rs6062497	62336258	20	C	T	0.334	0.309	0.008	0.0E+00	cis
2184	TNFRSF8	P28908	Tumor necrosis factor receptor superfamily member 8	rs35249183	12099345	1	G	A	0.100	0.348	0.013	3.5E-168	cis
2184	TNFRSF8	P28908	Tumor necrosis factor receptor superfamily member 8	rs3094005	31465047	6	T	G	0.141	0.137	0.011	4.6E-38	trans
2184	TNFRSF8	P28908	Tumor necrosis factor receptor superfamily member 8	rs10817685	117689305	9	C	T	0.571	0.114	0.007	4.8E-53	trans
2184	TNFRSF8	P28908	Tumor necrosis factor receptor superfamily member 8	rs76428106	28604007	13	C	T	0.013	0.231	0.034	8.2E-12	trans
2184	TNFRSF8	P28908	Tumor necrosis factor receptor superfamily member 8	rs2695167	42028820	15	G	A	0.017	0.369	0.028	4.4E-39	trans
2184	TNFRSF8	P28908	Tumor necrosis factor receptor superfamily member 8	rs186021206	7069412	17	A	G	0.006	0.388	0.050	1.5E-14	trans
2184	TNFRSF8	P28908	Tumor necrosis factor receptor superfamily member 8	rs3814995	36342212	19	T	C	0.315	0.054	0.008	1.2E-11	trans
2185	TNFRSF9	Q07011	Tumor necrosis factor receptor superfamily member 9	rs2493214	7994420	1	A	G	0.810	0.194	0.009	5.1E-97	cis
2185	TNFRSF9	Q07011	Tumor necrosis factor receptor superfamily member 9	rs3117574	31725230	6	A	G	0.133	0.124	0.011	6.0E-31	trans
2185	TNFRSF9	Q07011	Tumor necrosis factor receptor superfamily member 9	rs374039502	108960385	13	A	T	0.021	0.198	0.027	2.3E-13	trans
2185	TNFRSF9	Q07011	Tumor necrosis factor receptor superfamily member 9	rs34557412	16852187	17	G	A	0.007	0.495	0.044	1.9E-29	trans
2185	TNFRSF9	Q07011	Tumor necrosis factor receptor superfamily member 9	rs61750000	6534728	19	G	C	0.995	0.395	0.053	8.3E-14	trans
2186	TNFSF10	P50591	Tumor necrosis factor ligand superfamily member 10	rs7519758	196825287	1	T	C	0.194	0.133	0.009	3.4E-51	trans
2186	TNFSF10	P50591	Tumor necrosis factor ligand superfamily member 10	rs79287178	172294500	3	G	A	0.970	0.537	0.022	6.3E-135	cis
2186	TNFSF10	P50591	Tumor necrosis factor ligand superfamily member 10	rs10502	90039670	6	C	G	0.381	0.050	0.007	3.2E-12	trans
2186	TNFSF10	P50591	Tumor necrosis factor ligand superfamily member 10	rs174547	61570783	11	C	T	0.345	0.069	0.007	6.3E-21	trans
2186	TNFSF10	P50591	Tumor necrosis factor ligand superfamily member 10	rs28929474	94844947	14	T	C	0.021	0.843	0.025	1.6E-250	trans
2186	TNFSF10	P50591	Tumor necrosis factor ligand superfamily member 10	rs4788460	72154509	16	T	C	0.294	0.093	0.008	3.4E-33	trans
2186	TNFSF10	P50591	Tumor necrosis factor ligand superfamily member 10	rs8178824	64224775	17	T	C	0.030	0.513	0.021	1.9E-134	trans
2186	TNFSF10	P50591	Tumor necrosis factor ligand superfamily member 10	rs680321	29797958	18	T	C	0.544	0.109	0.007	2.0E-53	trans
2186	TNFSF10	P50591	Tumor necrosis factor ligand superfamily member 10	rs4760	44153100	19	A	G	0.844	0.480	0.010	0.0E+00	trans
2187	TNFSF11	O14788	Tumor necrosis factor ligand superfamily member 11	rs79287178	172294500	3	G	A	0.970	0.661	0.023	4.4E-187	trans
2187	TNFSF11	O14788	Tumor necrosis factor ligand superfamily member 11	rs7011570	23085269	8	C	T	0.747	0.177	0.009	1.7E-94	trans
2187	TNFSF11	O14788	Tumor necrosis factor ligand superfamily member 11	rs2737212	116621214	8	C	T	0.450	0.054	0.007	4.4E-13	trans
2187	TNFSF11	O14788	Tumor necrosis factor ligand superfamily member 11	rs1156545	120080692	8	G	A	0.542	0.222	0.008	8.0E-192	trans
2187	TNFSF11	O14788	Tumor necrosis factor ligand superfamily member 11	rs2062305	43052880	13	G	A	0.468	0.112	0.007	7.8E-52	cis
2187	TNFSF11	O14788	Tumor necrosis factor ligand superfamily member 11	rs7146217	69250915	14	C	T	0.531	0.051	0.007	5.0E-12	trans
2187	TNFSF11	O14788	Tumor necrosis factor ligand superfamily member 11	rs2207132	39142516	20	G	A	0.967	0.149	0.021	4.9E-13	trans
2188	TNFSF12	O43508	Tumor necrosis factor ligand superfamily member 12	rs2228099	150808889	1	C	G	0.633	0.063	0.007	1.7E-17	trans
2188	TNFSF12	O43508	Tumor necrosis factor ligand superfamily member 12	rs1354034	56849749	3	T	C	0.396	0.052	0.007	1.5E-12	trans
2188	TNFSF12	O43508	Tumor necrosis factor ligand superfamily member 12	rs828596	98465313	3	C	G	0.586	0.066	0.007	1.4E-19	trans
2188	TNFSF12	O43508	Tumor necrosis factor ligand superfamily member 12	rs9842051	143021856	3	C	G	0.731	0.071	0.008	2.6E-18	trans
2188	TNFSF12	O43508	Tumor necrosis factor ligand superfamily member 12	rs13135092	103198082	4	G	A	0.084	0.136	0.013	1.3E-25	trans
2188	TNFSF12	O43508	Tumor necrosis factor ligand superfamily member 12	rs4098923	31193756	6	A	G	0.552	0.057	0.008	5.8E-14	trans
2188	TNFSF12	O43508	Tumor necrosis factor ligand superfamily member 12	rs139141690	101499930	7	A	G	0.005	0.358	0.053	1.1E-11	trans
2188	TNFSF12	O43508	Tumor necrosis factor ligand superfamily member 12	rs6993770	106581528	8	A	T	0.713	0.071	0.008	4.5E-19	trans
2188	TNFSF12	O43508	Tumor necrosis factor ligand superfamily member 12	rs10107388	145004944	8	C	T	0.366	0.076	0.007	3.5E-24	trans
2188	TNFSF12	O43508	Tumor necrosis factor ligand superfamily member 12	rs10761741	65066186	10	T	G	0.414	0.098	0.007	2.0E-41	trans
2188	TNFSF12	O43508	Tumor necrosis factor ligand superfamily member 12	rs11158588	65799876	14	G	A	0.207	0.074	0.009	8.6E-17	trans
2188	TNFSF12	O43508	Tumor necrosis factor ligand superfamily member 12	rs151233	28506428	16	T	C	0.130	0.074	0.011	3.2E-12	trans
2188	TNFSF12	O43508	Tumor necrosis factor ligand superfamily member 12	rs80067372	7452752	17	A	G	0.288	0.372	0.008	0.0E+00	cis
2188	TNFSF12	O43508	Tumor necrosis factor ligand superfamily member 12	rs77542162	67081278	17	G	A	0.023	0.181	0.025	2.7E-13	trans
2188	TNFSF12	O43508	Tumor necrosis factor ligand superfamily member 12	rs892090	55539072	19	G	T	0.834	0.104	0.010	6.2E-27	trans
2188	TNFSF12	O43508	Tumor necrosis factor ligand superfamily member 12	rs5906245	46467354	X	T	C	0.321	0.056	0.006	1.0E-18	trans
2189	TNFSF13	O75888	Tumor necrosis factor ligand superfamily member 13	rs12740374	109817590	1	G	T	0.779	0.237	0.008	2.0E-175	trans
2189	TNFSF13	O75888	Tumor necrosis factor ligand superfamily member 13	rs1354034	56849749	3	T	C	0.396	0.053	0.007	9.3E-14	trans
2189	TNFSF13	O75888	Tumor necrosis factor ligand superfamily member 13	rs35173808	31326148	6	C	A	0.903	0.099	0.012	4.2E-17	trans
2189	TNFSF13	O75888	Tumor necrosis factor ligand superfamily member 13	rs11570136	12058832	16	A	T	0.327	0.067	0.007	1.8E-19	trans
2189	TNFSF13	O75888	Tumor necrosis factor ligand superfamily member 13	rs3803800	7462969	17	A	G	0.211	0.325	0.009	4.1E-307	cis
2189	TNFSF13	O75888	Tumor necrosis factor ligand superfamily member 13	rs16961828	16820224	17	G	C	0.898	0.099	0.012	4.5E-17	trans
2189	TNFSF13	O75888	Tumor necrosis factor ligand superfamily member 13	rs77542162	67081278	17	G	A	0.023	0.177	0.024	1.0E-13	trans
2189	TNFSF13	O75888	Tumor necrosis factor ligand superfamily member 13	rs892090	55539072	19	G	T	0.834	0.076	0.009	3.1E-16	trans
2190	TNFSF13B	Q9Y275	Tumor necrosis factor ligand superfamily member 13B	rs35675148	31656213	3	C	CAT	0.225	0.077	0.008	3.7E-20	trans
2190	TNFSF13B	Q9Y275	Tumor necrosis factor ligand superfamily member 13B	rs3129755	32583057	6	G	A	0.568	0.096	0.007	2.0E-40	trans
2190	TNFSF13B	Q9Y275	Tumor necrosis factor ligand superfamily member 13B	rs374039502	108960385	13	A	T	0.021	0.746	0.027	2.4E-174	cis
2190	TNFSF13B	Q9Y275	Tumor necrosis factor ligand superfamily member 13B	rs79874727	106370437	14	A	G	0.106	0.089	0.013	4.1E-12	trans
2190	TNFSF13B	Q9Y275	Tumor necrosis factor ligand superfamily member 13B	rs34557412	16852187	17	G	A	0.007	0.598	0.043	5.9E-45	trans
2190	TNFSF13B	Q9Y275	Tumor necrosis factor ligand superfamily member 13B	rs12986064	54755133	19	T	C	0.493	0.118	0.007	1.6E-59	trans
2190	TNFSF13B	Q9Y275	Tumor necrosis factor ligand superfamily member 13B	rs74510325	50315382	22	G	C	0.035	0.203	0.019	5.9E-26	trans
2191	TNFSF14	O43557	Tumor necrosis factor ligand superfamily member 14	rs2631367	131705458	5	G	C	0.518	0.060	0.007	7.6E-16	trans
2191	TNFSF14	O43557	Tumor necrosis factor ligand superfamily member 14	rs6993770	106581528	8	A	T	0.713	0.055	0.008	1.5E-11	trans
2191	TNFSF14	O43557	Tumor necrosis factor ligand superfamily member 14	rs12411442	104349531	10	A	G	0.571	0.051	0.007	6.7E-12	trans
2191	TNFSF14	O43557	Tumor necrosis factor ligand superfamily member 14	rs344560	6665020	19	C	T	0.945	0.554	0.017	1.6E-239	cis
2191	TNFSF14	O43557	Tumor necrosis factor ligand superfamily member 14	rs892090	55539072	19	G	T	0.834	0.107	0.010	1.8E-25	trans
2192	TNFSF8	P32971	Tumor necrosis factor ligand superfamily member 8	rs37453	44295047	1	G	A	0.352	0.150	0.008	1.8E-85	trans
2192	TNFSF8	P32971	Tumor necrosis factor ligand superfamily member 8	rs34479374	98728717	3	A	G	0.084	0.154	0.013	2.5E-31	trans
2192	TNFSF8	P32971	Tumor necrosis factor ligand superfamily member 8	rs9824474	169745527	3	A	G	0.633	0.108	0.008	5.7E-45	trans
2192	TNFSF8	P32971	Tumor necrosis factor ligand superfamily member 8	rs2523996	29905621	6	A	G	0.443	0.062	0.008	1.1E-15	trans
2192	TNFSF8	P32971	Tumor necrosis factor ligand superfamily member 8	rs2126259	9185146	8	T	C	0.103	0.097	0.012	9.6E-16	trans
2192	TNFSF8	P32971	Tumor necrosis factor ligand superfamily member 8	rs10081728	117695685	9	T	C	0.593	0.236	0.008	5.9E-215	cis
2192	TNFSF8	P32971	Tumor necrosis factor ligand superfamily member 8	rs7937564	60218504	11	G	A	0.680	0.055	0.008	2.3E-12	trans
2192	TNFSF8	P32971	Tumor necrosis factor ligand superfamily member 8	rs78689694	126234820	11	G	C	0.867	0.146	0.011	2.0E-41	trans
2192	TNFSF8	P32971	Tumor necrosis factor ligand superfamily member 8	rs186021206	7069412	17	A	G	0.006	1.816	0.051	2.3E-281	trans
2192	TNFSF8	P32971	Tumor necrosis factor ligand superfamily member 8	rs2659005	79218714	17	T	C	0.441	0.065	0.007	3.4E-18	trans
2193	TNIP1	Q15025	TNFAIP3-interacting protein 1	rs1354034	56849749	3	C	T	0.604	0.074	0.007	3.7E-23	trans
2193	TNIP1	Q15025	TNFAIP3-interacting protein 1	rs1585213	103444698	4	T	C	0.399	0.051	0.007	1.3E-11	trans
2193	TNIP1	Q15025	TNFAIP3-interacting protein 1	rs342293	106372219	7	C	G	0.540	0.053	0.007	8.0E-13	trans
2194	TNN	Q9UQP3	Tenascin-N	rs535777	32577633	6	G	C	0.857	0.078	0.008	1.5E-24	trans
2194	TNN	Q9UQP3	Tenascin-N	rs4841132	9183596	8	A	G	0.092	0.073	0.009	2.7E-15	trans
2194	TNN	Q9UQP3	Tenascin-N	rs2685412	27810603	8	G	C	0.270	0.146	0.006	2.9E-132	trans
2194	TNN	Q9UQP3	Tenascin-N	rs2068888	94839642	10	G	A	0.549	0.038	0.005	4.6E-13	trans
2194	TNN	Q9UQP3	Tenascin-N	rs174561	61582708	11	C	T	0.310	0.046	0.006	4.9E-16	trans
2195	TNR	Q92752	Tenascin-R	rs2235256	175569047	1	A	G	0.554	0.375	0.007	0.0E+00	cis
2195	TNR	Q92752	Tenascin-R	rs2169305	159509380	3	C	T	0.361	0.078	0.007	5.6E-27	trans
2195	TNR	Q92752	Tenascin-R	rs2507997	31314781	6	A	G	0.694	0.071	0.008	8.0E-21	trans
2195	TNR	Q92752	Tenascin-R	rs1111796	117086226	9	G	A	0.604	0.138	0.007	1.6E-79	trans
2195	TNR	Q92752	Tenascin-R	rs11594905	77659733	10	A	G	0.136	0.080	0.010	3.6E-15	trans
2195	TNR	Q92752	Tenascin-R	rs1727	91066769	10	C	A	0.276	0.057	0.008	4.1E-13	trans
2195	TNR	Q92752	Tenascin-R	rs7120175	30490453	11	G	C	0.548	0.052	0.007	1.4E-13	trans
2195	TNR	Q92752	Tenascin-R	rs11603123	126305495	11	A	G	0.034	0.342	0.019	4.7E-70	trans
2195	TNR	Q92752	Tenascin-R	rs12879626	34721134	14	G	T	0.610	0.133	0.007	3.7E-75	trans
2195	TNR	Q92752	Tenascin-R	rs10658607	57857146	15	A	AAAAT	0.677	0.055	0.008	2.6E-13	trans
2195	TNR	Q92752	Tenascin-R	rs2439430	66917844	15	T	G	0.499	0.067	0.007	2.1E-21	trans
2195	TNR	Q92752	Tenascin-R	rs4886410	75065644	15	C	G	0.682	0.056	0.008	9.3E-14	trans
2195	TNR	Q92752	Tenascin-R	rs3817428	89415247	15	G	C	0.266	0.152	0.008	5.0E-82	trans
2195	TNR	Q92752	Tenascin-R	rs186021206	7069412	17	A	G	0.006	0.590	0.048	1.6E-34	trans
2196	TNXB	P22105	Tenascin-X	rs147650812	135398870	5	T	C	0.992	0.272	0.040	6.5E-12	trans
2196	TNXB	P22105	Tenascin-X	rs9267797	32030700	6	T	C	0.015	1.661	0.031	0.0E+00	cis

2196	TNXB	P22105	Tenascin-X	rs181242111	17865664	10	A	G	0.118	0.089	0.011	4.4E-15	trans
2196	TNXB	P22105	Tenascin-X	rs78689694	126234820	11	G	C	0.867	0.150	0.010	4.0E-50	trans
2196	TNXB	P22105	Tenascin-X	rs186021206	7069412	17	A	G	0.006	0.808	0.047	5.8E-67	trans
2196	TNXB	P22105	Tenascin-X	rs1236213	28477742	21	G	T	0.407	0.050	0.007	8.0E-13	trans
2197	TOMM20	Q15388	Mitochondrial import receptor subunit TOM20 homolog	rs1354034	56849749	3	C	T	0.604	0.073	0.008	2.0E-21	trans
2197	TOMM20	Q15388	Mitochondrial import receptor subunit TOM20 homolog	rs342293	106372219	7	C	G	0.541	0.060	0.008	1.4E-15	trans
2197	TOMM20	Q15388	Mitochondrial import receptor subunit TOM20 homolog	rs6993770	106581528	8	A	T	0.713	0.065	0.008	6.9E-15	trans
2197	TOMM20	Q15388	Mitochondrial import receptor subunit TOM20 homolog	rs7896518	65104500	10	G	A	0.424	0.063	0.008	3.1E-16	trans
2197	TOMM20	Q15388	Mitochondrial import receptor subunit TOM20 homolog	rs73396521	267568	11	A	G	0.245	0.060	0.009	8.2E-12	trans
2198	TOP1	P11387	DNA topoisomerase 1	rs760077	155178782	1	A	T	0.394	0.074	0.008	7.3E-22	trans
2198	TOP1	P11387	DNA topoisomerase 1	rs112268616	205816893	1	T	C	0.660	0.745	0.008	0.0E+00	trans
2198	TOP1	P11387	DNA topoisomerase 1	rs11680123	60514748	2	G	A	0.593	0.115	0.006	2.1E-79	trans
2198	TOP1	P11387	DNA topoisomerase 1	rs121908120	219755011	2	T	A	0.972	0.134	0.019	5.0E-13	trans
2198	TOP1	P11387	DNA topoisomerase 1	rs11707641	12155888	3	C	T	0.162	0.059	0.008	2.4E-13	trans
2198	TOP1	P11387	DNA topoisomerase 1	rs34211178	98383562	3	G	A	0.559	0.044	0.006	2.2E-13	trans
2198	TOP1	P11387	DNA topoisomerase 1	rs36034702	90734627	15	C	T	0.831	0.070	0.008	5.8E-18	trans
2199	TOP2B	Q02880	DNA topoisomerase 2-beta	rs3806631	25706846	3	T	A	0.200	0.098	0.009	5.4E-25	cis
2199	TOP2B	Q02880	DNA topoisomerase 2-beta	rs1354034	56849749	3	C	T	0.604	0.194	0.008	9.6E-140	trans
2199	TOP2B	Q02880	DNA topoisomerase 2-beta	rs6993770	106581528	8	A	T	0.713	0.057	0.008	9.0E-12	trans
2200	TOR1AIP1	Q5JTV8	Torsin-1A-interacting protein 1	rs486906	179863237	1	T	G	0.616	0.709	0.008	0.0E+00	cis
2200	TOR1AIP1	Q5JTV8	Torsin-1A-interacting protein 1	rs61000956	187164081	4	T	C	0.518	0.056	0.007	2.4E-16	trans
2200	TOR1AIP1	Q5JTV8	Torsin-1A-interacting protein 1	rs1801020	176836532	5	A	G	0.255	0.055	0.008	7.3E-13	trans
2200	TOR1AIP1	Q5JTV8	Torsin-1A-interacting protein 1	rs34436714	54327313	19	A	C	0.209	0.081	0.008	9.5E-23	trans
2201	TP53	P04637	Cellular tumor antigen p53	rs1641549	7574775	17	T	C	0.255	0.066	0.009	2.0E-13	cis
2202	TP53BP1	Q12888	TP53-binding protein 1	rs78037977	172715702	1	A	G	0.876	0.081	0.012	7.4E-12	trans
2202	TP53BP1	Q12888	TP53-binding protein 1	rs3087243	204738919	2	G	A	0.548	0.054	0.008	2.6E-12	trans
2202	TP53BP1	Q12888	TP53-binding protein 1	rs148451580	32498626	6	G	A	0.608	0.122	0.008	2.2E-49	trans
2202	TP53BP1	Q12888	TP53-binding protein 1	rs10957978	81285139	8	T	G	0.648	0.063	0.008	2.8E-15	trans
2203	TP53I3	Q53FA7	Quinone oxidoreductase PIG3	rs1134516	24342532	2	G	A	0.841	0.596	0.010	0.0E+00	cis
2203	TP53I3	Q53FA7	Quinone oxidoreductase PIG3	rs1354034	56849749	3	C	T	0.604	0.069	0.007	9.9E-21	trans
2203	TP53I3	Q53FA7	Quinone oxidoreductase PIG3	rs6993770	106581528	8	A	T	0.713	0.079	0.008	8.9E-23	trans
2204	TP53INP1	Q96A56	Tumor protein p53-inducible nuclear protein 1	rs58534292	106483584	14	T	C	0.350	0.057	0.008	2.3E-12	trans
2204	TP53INP1	Q96A56	Tumor protein p53-inducible nuclear protein 1	rs56366203	106474284	14	C	G	0.502	0.064	0.008	3.4E-16	trans
2204	TP53INP1	Q96A56	Tumor protein p53-inducible nuclear protein 1	rs8192297	90344352	15	T	C	0.886	0.153	0.012	2.3E-36	trans
2205	TPBGL	P0DKB5	Trophoblast glycoprotein-like	rs58175074	196820080	1	G	A	0.197	0.074	0.010	2.3E-14	trans
2206	TPD52L2	O43399	Tumor protein D54	rs1354034	56849749	3	C	T	0.604	0.077	0.008	5.1E-23	trans
2206	TPD52L2	O43399	Tumor protein D54	rs342298	106373646	7	C	T	0.545	0.070	0.008	7.2E-20	trans
2206	TPD52L2	O43399	Tumor protein D54	rs6993770	106581528	8	A	T	0.713	0.067	0.008	1.3E-15	trans
2207	TPK1	Q9H3S4	Thiamin pyrophosphokinase 1	rs61747728	179526214	1	T	C	0.038	0.140	0.019	4.6E-14	trans
2207	TPK1	Q9H3S4	Thiamin pyrophosphokinase 1	rs10261806	144532104	7	G	A	0.597	0.334	0.008	0.0E+00	cis
2207	TPK1	Q9H3S4	Thiamin pyrophosphokinase 1	rs4879666	33125279	9	A	G	0.510	0.062	0.007	6.2E-18	trans
2207	TPK1	Q9H3S4	Thiamin pyrophosphokinase 1	rs12342201	95894964	9	G	A	0.524	0.061	0.007	1.0E-17	trans
2207	TPK1	Q9H3S4	Thiamin pyrophosphokinase 1	rs56278466	17875857	10	G	T	0.661	0.102	0.008	6.5E-42	trans
2207	TPK1	Q9H3S4	Thiamin pyrophosphokinase 1	rs7213540	79255912	17	C	T	0.461	0.049	0.007	6.3E-12	trans
2207	TPK1	Q9H3S4	Thiamin pyrophosphokinase 1	rs33950747	36339247	19	T	C	0.075	0.112	0.013	7.3E-17	trans
2208	TPM3	P06753	Tropomyosin alpha-3 chain	rs4478991	22199265	11	G	A	0.572	0.058	0.008	1.1E-13	trans
2209	TPMT	P51580	Thiopurine S-methyltransferase	rs115306927	18126938	6	A	T	0.950	1.095	0.017	0.0E+00	cis
2210	TPP1	O14773	Tripeptidyl-peptidase 1	rs77074394	154456852	4	G	A	0.032	0.156	0.021	1.5E-13	trans
2210	TPP1	O14773	Tripeptidyl-peptidase 1	rs10455861	160401662	6	G	A	0.863	0.099	0.011	3.9E-20	trans
2210	TPP1	O14773	Tripeptidyl-peptidase 1	rs17154155	80234243	7	T	G	0.407	0.058	0.007	5.2E-15	trans
2210	TPP1	O14773	Tripeptidyl-peptidase 1	rs6993770	106581528	8	A	T	0.713	0.113	0.008	4.6E-44	trans
2210	TPP1	O14773	Tripeptidyl-peptidase 1	rs9414801	65149089	10	G	A	0.467	0.077	0.007	3.5E-25	trans
2210	TPP1	O14773	Tripeptidyl-peptidase 1	rs140726254	6638944	11	A	G	0.002	2.890	0.079	8.1E-294	cis
2210	TPP1	O14773	Tripeptidyl-peptidase 1	rs145078947	93652974	14	T	C	0.003	0.876	0.072	7.5E-34	trans
2210	TPP1	O14773	Tripeptidyl-peptidase 1	rs1045693	5075226	16	G	A	0.468	0.081	0.007	2.6E-27	trans
2210	TPP1	O14773	Tripeptidyl-peptidase 1	rs8064959	5277729	17	A	T	0.690	0.054	0.008	7.8E-12	trans
2210	TPP1	O14773	Tripeptidyl-peptidase 1	rs200210321	19393890	19	AG	A	0.074	0.124	0.014	1.3E-18	trans
2210	TPP1	O14773	Tripeptidyl-peptidase 1	rs1654425	55538980	19	C	T	0.834	0.094	0.010	2.7E-21	trans
2211	TPPP3	Q9BW30	Tubulin polymerization-promoting protein family member 3	rs13334364	67332365	16	T	C	0.925	0.117	0.015	1.3E-15	cis
2212	TPR	P12270	Nucleoprotein TPR	rs115482159	186301436	1	T	C	0.006	0.420	0.051	3.4E-16	cis
2213	TPSAB1	Q15661	Tryptase alpha/beta-1	rs115427247	92878407	1	C	T	0.970	0.155	0.015	2.6E-25	trans
2213	TPSAB1	Q15661	Tryptase alpha/beta-1	rs16856110	205631767	1	A	G	0.770	0.057	0.006	4.1E-21	trans
2213	TPSAB1	Q15661	Tryptase alpha/beta-1	rs2884425	234694483	1	C	T	0.507	0.038	0.005	7.9E-14	trans
2213	TPSAB1	Q15661	Tryptase alpha/beta-1	rs56043070	247719769	1	A	G	0.073	0.094	0.010	2.8E-22	trans
2213	TPSAB1	Q15661	Tryptase alpha/beta-1	rs62252239	69893971	3	T	G	0.212	0.045	0.006	2.2E-13	trans
2213	TPSAB1	Q15661	Tryptase alpha/beta-1	rs586320	114604017	3	A	G	0.412	0.048	0.005	9.9E-21	trans
2213	TPSAB1	Q15661	Tryptase alpha/beta-1	rs28455789	159963534	4	G	A	0.827	0.048	0.007	1.1E-12	trans
2213	TPSAB1	Q15661	Tryptase alpha/beta-1	rs876606	77801083	5	A	G	0.256	0.048	0.006	2.9E-16	trans
2213	TPSAB1	Q15661	Tryptase alpha/beta-1	rs56278466	17875857	10	G	T	0.661	0.039	0.005	3.2E-13	trans
2213	TPSAB1	Q15661	Tryptase alpha/beta-1	rs7127546	61689731	11	A	G	0.374	0.052	0.005	4.4E-23	trans
2213	TPSAB1	Q15661	Tryptase alpha/beta-1	rs8051930	1295818	16	A	T	0.478	0.832	0.008	0.0E+00	cis
2213	TPSAB1	Q15661	Tryptase alpha/beta-1	rs17758695	60920854	18	C	T	0.971	0.124	0.015	1.0E-16	trans
2213	TPSAB1	Q15661	Tryptase alpha/beta-1	rs78744187	33754548	19	T	C	0.082	0.186	0.009	5.0E-91	trans
2213	TPSAB1	Q15661	Tryptase alpha/beta-1	rs111402121	44281824	19	CTCTT	C	0.377	0.070	0.005	1.2E-40	trans
2214	TPSD1	Q9BZJ3	Tryptase delta	rs34072109	61687921	11	CTCTA	C	0.377	0.054	0.008	7.9E-13	trans
2214	TPSD1	Q9BZJ3	Tryptase delta	rs78744187	33754548	19	T	C	0.082	0.140	0.013	1.7E-25	trans
2214	TPSD1	Q9BZJ3	Tryptase delta	rs111402121	44281824	19	CTCTT	C	0.377	0.059	0.008	8.8E-15	trans
2215	TRAF2	Q12933	TNF receptor-associated factor 2	rs1354034	56849749	3	C	T	0.604	0.070	0.007	4.7E-21	trans
2215	TRAF2	Q12933	TNF receptor-associated factor 2	rs532086	31881309	6	T	C	0.838	0.069	0.010	4.1E-12	trans
2215	TRAF2	Q12933	TNF receptor-associated factor 2	rs4239702	44749251	20	T	C	0.278	0.061	0.008	9.2E-14	trans
2215	TRAF2	Q12933	TNF receptor-associated factor 2	rs36206512	135730217	X	C	A	0.035	0.147	0.017	3.7E-18	trans
2216	TRAF3	Q13114	TNF receptor-associated factor 3, Isoform 2	rs6929057	32597005	6	C	A	0.249	0.096	0.009	1.3E-27	trans
2216	TRAF3	Q13114	TNF receptor-associated factor 3, Isoform 2	rs13263709	81287175	8	C	T	0.649	0.056	0.008	2.4E-12	trans
2217	TRDMT1	O14717	tRNA (cytosine(38)-C(5))-methyltransferase	rs1354034	56849749	3	C	T	0.604	0.078	0.008	4.3E-24	trans
2217	TRDMT1	O14717	tRNA (cytosine(38)-C(5))-methyltransferase	rs6602180	17192362	10	T	C	0.537	0.147	0.008	3.0E-82	cis
2218	TREH	O43280	Trehalase	rs114165349	27021913	1	C	G	0.023	0.122	0.018	3.6E-12	trans
2218	TREH	O43280	Trehalase	rs2484697	221094718	1	A	G	0.476	0.037	0.005	5.7E-12	trans
2218	TREH	O43280	Trehalase	rs9492442	130396651	6	G	A	0.681	0.039	0.006	8.4E-12	trans
2218	TREH	O43280	Trehalase	rs13240065	73015369	7	A	G	0.127	0.064	0.008	1.0E-15	trans
2218	TREH	O43280	Trehalase	rs11455968	118563703	11	GA	G	0.325	0.884	0.008	0.0E+00	cis
2218	TREH	O43280	Trehalase	rs8000528	74751477	13	G	T	0.545	0.039	0.005	1.7E-13	trans
2218	TREH	O43280	Trehalase	rs55707100	43820717	15	T	C	0.024	0.166	0.017	4.0E-22	trans
2218	TREH	O43280	Trehalase	rs339969	60883281	15	A	C	0.616	0.061	0.005	7.2E-29	trans
2219	TREM2	Q9NZC2	Triggering receptor expressed on myeloid cells 2	rs10919543	161508617	1	G	A	0.316	0.050	0.007	7.6E-12	trans
2219	TREM2	Q9NZC2	Triggering receptor expressed on myeloid cells 2	rs2274700	196682947	1	G	A	0.597	0.057	0.007	5.0E-17	trans
2219	TREM2	Q9NZC2	Triggering receptor expressed on myeloid cells 2	rs531950386	32486540	6	G	T	0.518	0.066	0.008	2.9E-18	trans
2219	TREM2	Q9NZC2	Triggering receptor expressed on myeloid cells 2	rs143332484	41129207	6	C	T	0.990	1.483	0.034	0.0E+00	cis
2219	TREM2	Q9NZC2	Triggering receptor expressed on myeloid cells 2	rs913914	102258250	9	C	G	0.632	0.052	0.007	7.5E-14	trans
2219	TREM2	Q9NZC2	Triggering receptor expressed on myeloid cells 2	rs7232	59940599	11	A	T	0.374	0.401	0.007	0.0E+00	trans
2219	TREM2	Q9NZC2	Triggering receptor expressed on myeloid cells 2	rs261290	58678720	15	T	C	0.345	0.048	0.007	9.1E-12	trans
2219	TREM2	Q9NZC2	Triggering receptor expressed on myeloid cells 2	rs738409	44324727	22	G	C	0.217	0.065	0.008	6.1E-16	trans
2220	TREML1	Q86YW5	Trem-like transcript 1 protein	rs4698839	102801941	4	C	T	0.602	0.065	0.008	3.1E-17	trans
2220	TREML1	Q86YW5	Trem-like transcript 1 protein	rs10058074	131686146	5	G	A	0.525	0.057	0.008	3.2E-14	trans
2220	TREML1	Q86YW5	Trem-like transcript 1 protein	rs6961069	80218961	7	T	C	0.403	0.058	0.008	3.1E-14	trans
2220	TREML1	Q86YW5	Trem-like transcript 1 protein	rs6993770	106581528	8	A	T	0.713	0.105	0.008	1.2E-36	trans
2220	TREML1	Q86YW5	Trem-like transcript 1 protein	rs7896518	65104500	10	G	A	0.424	0.094	0.008	2.3E-34	trans
2220	TREML1	Q86YW5	Trem-like transcript 1 protein	rs12445050	81870969	16	T	C	0.138	0.088	0.011	7.3E-16	trans
2220	TREML1	Q86YW5	Trem-like transcript 1 protein	rs1654425	55538980	19	C	T	0.834	0.138	0.010	1.0E-41	trans
2220	TREML1	Q86YW5	Trem-like transcript 1 protein	rs6112308	19291868	20	T	C	0.354	0.061	0.008	6.8E-15	trans
2221	TREML2	Q5T2D2	Trem-like transcript 2 protein	rs142421172	179682087	1	A	G	0.040	0.135	0.019	2.1E-12	trans
2221	TREML2	Q5T2D2	Trem-like transcript 2 protein	rs11759347	41167097	6	A	C	0.238	0.307	0.009	7.7E-266	cis
2221	TREML2	Q5T2D2	Trem-like transcript 2 protein	rs56293029	135419039	6	A	C	0.255	0.083	0.009	1.3E-21	trans
2221	TREML2	Q5T2D2	Trem-like transcript 2 protein	rs409801	4744743	9	C	T	0.507	0.061	0.007	5.0E-17	trans
2221	TREML2	Q5T2D2	Trem-like transcript 2 protein	rs7080386	65048306	10	A	C	0.412	0.060	0.007	4.2E-16	trans
2221	TREML2	Q5T2D2	Trem-like transcript 2 protein	rs12576289	126265841	11	A	G	0.781	0.065	0.009	4.3E-13	trans
2221	TREML2	Q5T2D2	Trem-like transcript 2 protein	rs186021206	7069412	17	A	G	0.006	0.465	0.050	1.3E-20	trans
2222	TRIAP1	O43715	TP53-regulated inhibitor of apoptosis 1	rs148212596	193074511	1	A	G	0.979	0.434	0.026	4.7E-62	trans
2222	TRIAP1	O43715	TP53-regulated inhibitor of apoptosis 1	rs3755115	53994160	2	A	C	0.024	0.163	0.024	1.5E-11	trans
2222	TRIAP1	O43715	TP53-regulated inhibitor of apoptosis 1	rs9856296	14155453	3	T	C	0.704	0.079	0.008	1.3E-21	trans
2222	TRIAP1	O43715	TP53-regulated inhibitor of apoptosis 1	rs1354034	56849749	3	C	T	0.604	0.085	0.008	2.7E-29	trans
2222	TRIAP1	O43715	TP53-regulated inhibitor of apoptosis 1	rs7896518	65104500	10	G	A	0.424	0.055	0.008	5.5E-13	trans
2222	TRIAP1	O43715	TP53-regulated inhibitor of apoptosis 1	rs10418046	54327869	19	G	T	0.212	0.094	0.009	1.4E-24	trans
2223	TRIM21	P19474	E3 ubiquitin-protein ligase TRIM21	rs915652	31749142	6	G	A	0.869	0.090	0.011	1.8E-16	trans
2224	TRIM24	O15164	Transcription intermediary factor 1-alpha	rs1354034	56849749	3	C	T	0.604	0.091	0.008	1.3E-32	trans
2224	TRIM24	O15164	Transcription intermediary factor 1-alpha	rs11502185	180258	11	C	T	0.260	0.069	0.010	1.1E-12	trans
2225	TRIM25	Q14258	E3 ubiquitin/ISG15 ligase TRIM25	rs2525992	54988182	17	C	T	0.268	0.100	0.008	6.0E-33	cis
2226	TRIM40	Q6P9F5	E3 ubiquitin ligase TRIM40	rs3815079	30114365	6	A	T	0.767	0.275	0.009	9.5E-203	cis
2227	TRIM5	Q9C035	Tripartite motif-containing protein 5	rs11821656	5689144	11	G	C	0.860	0.408	0.011	0.0E+00	cis
2228	TRIM58	Q8NG06	E3 ubiquitin-protein ligase TRIM58	rs11204523	248020556	1	C	G	0.759	0.226	0.009	1.4E-142	cis
2228	TRIM58	Q8NG06	E3 ubiquitin-protein ligase TRIM58	rs342293	106372219	7	C	G	0.540	0.051	0.007	6.5E-12	trans
2228	TRIM58	Q8NG06	E3 ubiquitin-protein ligase TRIM58	rs7896518	65104500	10	G	A	0.424	0.054	0.008	1.1E-12	trans
2229	TSC1	Q92574	Hamartin	rs4548048	32625022	6	T	C	0.103	0.123	0.013	6.9E-21	trans
2230	TSC22D1	Q15714	TSC22 domain family protein 1	rs1354034	56849749	3	C	T	0.604	0.053	0.008	4.3E-12	trans
2230	TSC22D1	Q15714	TSC22 domain family protein 1	rs6866614	131787137	5	G	A	0.574	0.052	0.008	8.6E-12	trans
2230	TSC22D1	Q15714	TSC22 domain family protein 1	rs11502185	180258	11	C	T	0.260	0.065	0.010	1.2E-11	trans
2230	TSC22D1	Q15714	TSC22 domain family protein 1	rs963569	45242379	13	C	T	0.219	0.069	0.009	4.8E-14	cis
2231	TSHB	P01222	Thyrotropin subunit beta	rs6426808	19835415	1	A	G	0.512	0.088	0.008	3.2E-31	trans
2231	TSHB	P01222	Thyrotropin subunit beta	rs334713	61622287	1	C	A	0.956	0.141	0.018	1.9E-14	trans
2231	TSHB	P01222	Thyrotropin subunit beta	rs2712172	217620733	2	G	A	0.740	0.076	0.009	1.3E-18	trans
2231	TSHB	P01222	Thyrotropin subunit beta	rs10028213	149652610	4	C	G	0.796	0.109	0.009	7.0E-32	trans
2231	TSHB	P01222	Thyrotropin subunit beta	rs2046045	76535811	5	G	T	0.395	0.150	0.008	7.4E-84	trans
2231	TSHB	P01222	Thyrotropin subunit beta	rs11751601	32559520	6	T	C	0.392	0.061	0.008	2.8E-14	trans
2231	TSHB	P01222	Thyrotropin subunit beta	rs2396084	43804825	6	G	A	0.689	0.077	0.008	5.4E-21	trans

2231	TSHB	P01222	Thyrotropin subunit beta	rs2983511	166043959	6	G	C	0.693	0.120	0.008	7.0E-49	trans
2231	TSHB	P01222	Thyrotropin subunit beta	rs7032019	100548144	9	A	G	0.670	0.056	0.008	3.4E-12	trans
2231	TSHB	P01222	Thyrotropin subunit beta	rs7128207	45230061	11	T	G	0.560	0.074	0.008	1.8E-22	trans
2231	TSHB	P01222	Thyrotropin subunit beta	rs116909374	36738361	14	C	T	0.961	0.183	0.019	4.6E-21	trans
2231	TSHB	P01222	Thyrotropin subunit beta	rs10519226	49738233	15	T	C	0.755	0.071	0.009	2.7E-16	trans
2231	TSHB	P01222	Thyrotropin subunit beta	rs200293726	79754440	16	A	T	0.694	0.081	0.008	3.5E-23	trans
2232	TSPAN1	O60635	Tetraspanin-1	rs34463133	46651169	1	G	A	0.997	0.449	0.061	1.2E-13	cis
2232	TSPAN1	O60635	Tetraspanin-1	rs2268535	186757083	3	A	G	0.237	0.061	0.008	1.1E-13	trans
2232	TSPAN1	O60635	Tetraspanin-1	rs708686	5840619	19	T	C	0.268	0.060	0.008	3.9E-14	trans
2233	TSPAN15	O95858	Tetraspanin-15	rs1227967	71267834	10	T	C	0.499	0.056	0.008	6.7E-13	cis
2234	TSPAN7	P41732	Tetraspanin-7	rs9271176	32578127	6	A	G	0.327	0.064	0.008	8.5E-15	trans
2235	TSPAN8	P19075	Tetraspanin-8	rs4263162	133195387	2	C	G	0.666	0.125	0.007	1.1E-66	trans
2235	TSPAN8	P19075	Tetraspanin-8	rs4144796	233562210	2	T	C	0.582	0.048	0.007	6.0E-12	trans
2235	TSPAN8	P19075	Tetraspanin-8	rs1727952	153871565	3	C	T	0.148	0.073	0.010	4.6E-14	trans
2235	TSPAN8	P19075	Tetraspanin-8	rs939885	195955762	3	A	G	0.486	0.067	0.007	1.8E-22	trans
2235	TSPAN8	P19075	Tetraspanin-8	rs9478784	151020330	6	T	C	0.096	0.085	0.012	2.9E-13	trans
2235	TSPAN8	P19075	Tetraspanin-8	rs8176759	136129647	9	A	G	0.062	0.130	0.014	5.8E-20	trans
2235	TSPAN8	P19075	Tetraspanin-8	rs56398830	103701690	13	G	A	0.989	0.479	0.033	9.2E-49	trans
2235	TSPAN8	P19075	Tetraspanin-8	rs2297066	103566835	14	G	C	0.242	0.056	0.008	3.6E-12	trans
2235	TSPAN8	P19075	Tetraspanin-8	rs1968109	89854829	16	G	C	0.315	0.090	0.007	2.0E-34	trans
2235	TSPAN8	P19075	Tetraspanin-8	rs34353378	28639246	17	C	A	0.330	0.120	0.007	3.8E-61	trans
2235	TSPAN8	P19075	Tetraspanin-8	rs708686	5840619	19	T	C	0.268	0.080	0.008	4.8E-25	trans
2235	TSPAN8	P19075	Tetraspanin-8	rs681343	49206462	19	C	T	0.491	0.180	0.007	7.4E-149	trans
2236	TSPYL1	Q9H0U9	Testis-specific Y-encoded-like protein 1	rs61746508	116600616	6	C	A	0.983	0.412	0.030	1.7E-43	cis
2236	TSPYL1	Q9H0U9	Testis-specific Y-encoded-like protein 1	rs7080386	65048306	10	A	C	0.412	0.056	0.008	5.0E-13	trans
2237	TST	Q16762	Thiosulfate sulfurtransferase	rs4821544	37258503	22	T	C	0.697	0.108	0.008	2.8E-38	cis
2238	TTF2	Q9UNY4	Transcription termination factor 2	rs75261221	117604855	1	G	A	0.052	0.784	0.017	0.0E+00	cis
2239	TTN	Q8WZ42	Titin	rs534819727	32481692	6	T	C	0.256	0.079	0.009	1.1E-16	trans
2240	TTR	P02766	Transthyretin	rs140584594	110232983	1	G	A	0.732	0.069	0.008	6.7E-18	trans
2240	TTR	P02766	Transthyretin	rs1260326	27730940	2	T	C	0.392	0.053	0.007	2.1E-13	trans
2240	TTR	P02766	Transthyretin	rs4841133	9183664	8	G	A	0.908	0.145	0.012	3.1E-32	trans
2240	TTR	P02766	Transthyretin	rs3999089	65203808	10	A	G	0.531	0.059	0.007	7.3E-17	trans
2240	TTR	P02766	Transthyretin	rs4575545	79755446	16	G	A	0.695	0.106	0.008	7.6E-43	trans
2240	TTR	P02766	Transthyretin	rs1667229	29142206	18	C	T	0.540	0.118	0.007	1.0E-60	cis
2240	TTR	P02766	Transthyretin	rs1883711	39179822	20	G	C	0.969	0.270	0.021	2.1E-38	trans
2240	TTR	P02766	Transthyretin	rs3747207	44324855	22	G	A	0.785	0.060	0.009	2.2E-12	trans
2241	TWF2	Q6IBS0	Twinfilin-2	rs7622851	52333671	3	G	C	0.498	0.057	0.007	3.8E-14	cis
2241	TWF2	Q6IBS0	Twinfilin-2	rs1354034	56849749	3	C	T	0.605	0.058	0.008	3.8E-14	trans
2241	TWF2	Q6IBS0	Twinfilin-2	rs6993770	106581528	8	A	T	0.714	0.062	0.008	8.7E-14	trans
2242	TXLNA	P40222	Alpha-taxilin	rs1354034	56849749	3	C	T	0.604	0.102	0.008	6.1E-41	trans
2242	TXLNA	P40222	Alpha-taxilin	rs342293	106372219	7	C	G	0.541	0.058	0.007	5.3E-15	trans
2242	TXLNA	P40222	Alpha-taxilin	rs11502185	180258	11	C	T	0.260	0.067	0.010	2.2E-12	trans
2243	TXN	P10599	Thioredoxin	rs146018216	113013717	9	G	A	0.998	1.152	0.095	6.2E-34	cis
2244	TXNDC15	Q96J42	Thioredoxin domain-containing protein 15	rs188468174	25291697	1	C	T	0.986	0.517	0.027	3.9E-82	trans
2244	TXNDC15	Q96J42	Thioredoxin domain-containing protein 15	rs115276619	184865132	1	A	T	0.017	0.208	0.025	3.3E-16	trans
2244	TXNDC15	Q96J42	Thioredoxin domain-containing protein 15	rs6054	155489608	4	T	C	0.006	0.311	0.042	2.0E-13	trans
2244	TXNDC15	Q96J42	Thioredoxin domain-containing protein 15	rs6876611	96243598	5	A	T	0.518	0.053	0.008	1.7E-12	trans
2244	TXNDC15	Q96J42	Thioredoxin domain-containing protein 15	rs3733897	134223593	5	G	A	0.135	1.170	0.011	0.0E+00	cis
2244	TXNDC15	Q96J42	Thioredoxin domain-containing protein 15	rs2524096	31236467	6	G	T	0.580	0.171	0.006	2.2E-154	trans
2244	TXNDC15	Q96J42	Thioredoxin domain-containing protein 15	rs73015965	161127501	6	G	A	0.006	0.337	0.043	2.9E-15	trans
2244	TXNDC15	Q96J42	Thioredoxin domain-containing protein 15	rs28929474	94844947	14	T	C	0.021	0.233	0.022	4.2E-26	trans
2244	TXNDC15	Q96J42	Thioredoxin domain-containing protein 15	rs4781137	11774950	16	T	C	0.510	0.044	0.006	2.8E-12	trans
2244	TXNDC15	Q96J42	Thioredoxin domain-containing protein 15	rs9302635	72144174	16	T	C	0.818	0.086	0.008	5.9E-26	trans
2244	TXNDC15	Q96J42	Thioredoxin domain-containing protein 15	rs1801689	64210580	17	C	A	0.030	0.509	0.018	3.0E-167	trans
2245	TXNDC5	Q8NBS9	Thioredoxin domain-containing protein 5	rs1354034	56849749	3	C	T	0.604	0.072	0.008	5.2E-21	trans
2245	TXNDC5	Q8NBS9	Thioredoxin domain-containing protein 5	rs7764128	7882205	6	G	A	0.892	0.158	0.012	9.5E-40	cis
2245	TXNDC5	Q8NBS9	Thioredoxin domain-containing protein 5	rs6993770	106581528	8	A	T	0.714	0.068	0.008	1.0E-16	trans
2246	TXNDC9	O14530	Thioredoxin domain-containing protein 9	rs147732775	100115083	2	A	G	0.011	0.251	0.037	8.9E-12	cis
2246	TXNDC9	O14530	Thioredoxin domain-containing protein 9	rs1354034	56849749	3	C	T	0.604	0.074	0.008	5.8E-22	trans
2247	TXNL1	O43396	Thioredoxin-like protein 1	rs71534539	32512754	6	G	A	0.536	0.107	0.009	6.6E-36	trans
2248	TXNRD1	Q16881	Thioredoxin reductase 1, cytoplasmic	rs1354034	56849749	3	C	T	0.604	0.054	0.008	9.4E-13	trans
2248	TXNRD1	Q16881	Thioredoxin reductase 1, cytoplasmic	rs6993770	106581528	8	A	T	0.714	0.068	0.008	1.4E-16	trans
2248	TXNRD1	Q16881	Thioredoxin reductase 1, cytoplasmic	rs12975366	54759361	19	T	C	0.605	0.061	0.008	1.2E-15	trans
2249	TYMP	P19971	Thymidine phosphorylase	rs131805	50964153	22	C	T	0.783	0.225	0.009	2.9E-132	cis
2250	TYRO3	Q06418	Tyrosine-protein kinase receptor TYRO3	rs10935473	98416900	3	G	T	0.560	0.230	0.007	7.2E-269	trans
2250	TYRO3	Q06418	Tyrosine-protein kinase receptor TYRO3	rs2519093	136141870	9	C	T	0.816	0.225	0.008	3.2E-158	trans
2250	TYRO3	Q06418	Tyrosine-protein kinase receptor TYRO3	rs174530	61546592	11	G	A	0.373	0.049	0.007	1.9E-13	trans
2250	TYRO3	Q06418	Tyrosine-protein kinase receptor TYRO3	rs3967200	126232385	11	C	T	0.866	0.074	0.009	5.3E-15	trans
2250	TYRO3	Q06418	Tyrosine-protein kinase receptor TYRO3	rs8024626	41863181	15	G	A	0.698	0.646	0.008	0.0E+00	cis
2250	TYRO3	Q06418	Tyrosine-protein kinase receptor TYRO3	rs186021206	7069412	17	A	G	0.006	0.960	0.044	2.1E-105	trans
2250	TYRO3	Q06418	Tyrosine-protein kinase receptor TYRO3	rs2008174	39860130	22	C	T	0.260	0.088	0.007	7.0E-32	trans
2251	TYRP1	P17643	5,6-dihydroxyindole-2-carboxylic acid oxidase	rs1801274	161479745	1	A	G	0.465	0.065	0.008	3.7E-17	trans
2251	TYRP1	P17643	5,6-dihydroxyindole-2-carboxylic acid oxidase	rs28746784	32632261	6	T	C	0.200	0.101	0.011	1.3E-20	trans
2251	TYRP1	P17643	5,6-dihydroxyindole-2-carboxylic acid oxidase	rs10960751	12675264	9	T	C	0.370	0.081	0.008	3.4E-24	cis
2251	TYRP1	P17643	5,6-dihydroxyindole-2-carboxylic acid oxidase	rs7153844	107073863	14	T	G	0.748	0.078	0.009	2.3E-17	trans
2252	UBE2L6	O14933	Ubiquitin/ISG15-conjugating enzyme E2 L6	rs1354034	56849749	3	C	T	0.604	0.081	0.007	3.7E-27	trans
2252	UBE2L6	O14933	Ubiquitin/ISG15-conjugating enzyme E2 L6	rs6993770	106581528	8	A	T	0.713	0.062	0.008	2.3E-14	trans
2252	UBE2L6	O14933	Ubiquitin/ISG15-conjugating enzyme E2 L6	rs28362950	57371918	11	A	G	0.267	0.254	0.008	2.2E-199	cis
2253	UBE2Z	Q9H832	Ubiquitin-conjugating enzyme E2 Z	rs62404112	32611122	6	C	T	0.056	0.119	0.017	1.3E-12	trans
2254	UBXN1	Q04323	UBX domain-containing protein 1	rs1354034	56849749	3	C	T	0.604	0.071	0.008	9.7E-21	trans
2254	UBXN1	Q04323	UBX domain-containing protein 1	rs342298	106373646	7	C	T	0.545	0.056	0.008	9.5E-14	trans
2254	UBXN1	Q04323	UBX domain-containing protein 1	rs142782985	62446179	11	C	G	0.002	0.556	0.080	4.4E-12	cis
2255	UFD1	Q92890	Ubiquitin recognition factor in ER-associated degradation protein 1	rs342298	106373646	7	C	T	0.545	0.055	0.008	2.5E-13	trans
2256	ULBP2	Q9BZM5	UL16-binding protein 2	rs4703854	71693463	5	T	C	0.763	0.069	0.008	7.0E-18	trans
2256	ULBP2	Q9BZM5	UL16-binding protein 2	rs6924387	137082948	6	A	G	0.594	0.057	0.008	5.6E-14	trans
2256	ULBP2	Q9BZM5	UL16-binding protein 2	rs60340208	150301195	6	A	AG	0.851	0.676	0.011	0.0E+00	cis
2256	ULBP2	Q9BZM5	UL16-binding protein 2	rs10100209	10584212	8	C	T	0.391	0.070	0.007	3.1E-23	trans
2256	ULBP2	Q9BZM5	UL16-binding protein 2	rs10104997	55440068	8	T	C	0.210	0.116	0.008	4.6E-44	trans
2256	ULBP2	Q9BZM5	UL16-binding protein 2	rs4804181	12509536	19	C	A	0.223	0.115	0.008	4.6E-44	trans
2257	UMOD	P07911	Uromodulin	rs4846826	217418118	1	C	T	0.371	0.041	0.006	5.1E-12	trans
2257	UMOD	P07911	Uromodulin	rs12465018	9236443	2	G	A	0.829	0.057	0.008	8.7E-14	trans
2257	UMOD	P07911	Uromodulin	rs72819488	95947099	2	G	A	0.844	0.062	0.008	5.1E-15	trans
2257	UMOD	P07911	Uromodulin	rs6542126	114022569	2	A	C	0.163	0.054	0.008	2.5E-12	trans
2257	UMOD	P07911	Uromodulin	rs6793835	135819934	3	A	G	0.266	0.046	0.006	9.4E-13	trans
2257	UMOD	P07911	Uromodulin	rs56012466	151406788	7	A	G	0.272	0.068	0.006	3.3E-26	trans
2257	UMOD	P07911	Uromodulin	rs13288823	6386584	9	C	T	0.947	0.094	0.013	1.5E-13	trans
2257	UMOD	P07911	Uromodulin	rs77713116	65531109	11	C	G	0.658	0.084	0.006	1.8E-43	trans
2257	UMOD	P07911	Uromodulin	rs10851885	76304503	15	A	G	0.743	0.050	0.007	3.2E-14	trans
2257	UMOD	P07911	Uromodulin	rs12922822	20367645	16	C	T	0.818	0.969	0.009	0.0E+00	cis
2257	UMOD	P07911	Uromodulin	rs186021206	7069412	17	A	G	0.006	0.295	0.040	1.5E-13	trans
2257	UMOD	P07911	Uromodulin	rs2934951	37833328	17	A	G	0.317	0.043	0.006	7.0E-12	trans
2257	UMOD	P07911	Uromodulin	rs72835417	47241642	17	A	G	0.110	0.247	0.009	2.9E-156	trans
2257	UMOD	P07911	Uromodulin	rs896642	59379074	18	T	C	0.222	0.047	0.007	8.2E-12	trans
2257	UMOD	P07911	Uromodulin	rs1997596	16578448	21	T	C	0.342	0.046	0.006	7.0E-14	trans
2258	UNC5D	Q6UXZ4	Netrin receptor UNC5D	rs13260977	35105155	8	A	G	0.582	0.064	0.008	2.7E-16	cis
2259	UNC79	Q9P2D8	Protein unc-79 homolog	rs953492	243471192	1	G	A	0.534	0.126	0.008	1.0E-59	trans
2260	UPB1	Q9UBR1	Beta-ureidopropionase	rs1260326	27730940	2	T	C	0.391	0.054	0.008	6.4E-13	trans
2260	UPB1	Q9UBR1	Beta-ureidopropionase	rs10119644	117129592	9	T	A	0.503	0.061	0.007	3.0E-16	trans
2260	UPB1	Q9UBR1	Beta-ureidopropionase	rs143493067	24919586	22	G	A	0.998	0.859	0.090	1.4E-21	cis
2260	UPB1	Q9UBR1	Beta-ureidopropionase	rs3747207	44324855	22	A	G	0.216	0.089	0.009	4.7E-23	trans
2261	UROD	P06132	Uroporphyrinogen decarboxylase	rs12749939	45478433	1	C	T	0.742	0.477	0.008	0.0E+00	cis
2261	UROD	P06132	Uroporphyrinogen decarboxylase	rs75214378	200785658	2	A	G	0.042	0.155	0.018	7.4E-18	trans
2261	UROD	P06132	Uroporphyrinogen decarboxylase	rs78564552	3216933	3	T	C	0.004	0.470	0.059	1.2E-15	trans
2261	UROD	P06132	Uroporphyrinogen decarboxylase	rs1354034	56849749	3	C	T	0.604	0.078	0.007	9.8E-27	trans
2261	UROD	P06132	Uroporphyrinogen decarboxylase	rs2393967	65133156	10	C	A	0.314	0.055	0.008	1.1E-12	trans
2262	UROS	P10746	Uroporphyrinogen-III synthase	rs57467915	220081416	2	A	G	0.015	0.275	0.031	1.3E-18	trans
2262	UROS	P10746	Uroporphyrinogen-III synthase	rs10794023	127493414	10	A	C	0.206	0.138	0.009	3.4E-48	cis
2263	USO1	O60763	General vesicular transport factor p115	rs1354034	56849749	3	C	T	0.605	0.062	0.008	3.3E-16	trans
2263	USO1	O60763	General vesicular transport factor p115	rs342298	106373646	7	C	T	0.546	0.055	0.008	1.9E-13	trans
2263	USO1	O60763	General vesicular transport factor p115	rs6993770	106581528	8	A	T	0.714	0.069	0.008	9.7E-17	trans
2264	USP25	Q9UHP3	Ubiquitin carboxyl-terminal hydrolase 25	rs1354034	56849749	3	C	T	0.604	0.080	0.008	2.1E-26	trans
2264	USP25	Q9UHP3	Ubiquitin carboxyl-terminal hydrolase 25	rs342298	106373646	7	C	T	0.545	0.050	0.007	1.4E-11	trans
2265	USP28	Q96RU2	Ubiquitin carboxyl-terminal hydrolase 28	rs4288784	113745608	11	C	G	0.923	0.153	0.014	1.7E-26	cis
2266	USP47	Q96K76	Ubiquitin carboxyl-terminal hydrolase 47	rs5751547	23104069	22	C	T	0.592	0.093	0.008	1.3E-31	trans
2267	USP8	P40818	Ubiquitin carboxyl-terminal hydrolase 8	rs78909033	241510903	2	G	A	0.864	0.187	0.011	2.9E-65	trans
2267	USP8	P40818	Ubiquitin carboxyl-terminal hydrolase 8	rs1354034	56849749	3	C	T	0.604	0.068	0.008	6.3E-19	trans
2267	USP8	P40818	Ubiquitin carboxyl-terminal hydrolase 8	rs342298	106373646	7	C	T	0.545	0.057	0.008	2.3E-14	trans
2267	USP8	P40818	Ubiquitin carboxyl-terminal hydrolase 8	rs4380013	50759428	15	A	G	0.196	0.091	0.009	6.3E-22	cis
2268	UXS1	Q8NBZ7	UDP-glucuronic acid decarboxylase 1	rs660240	109817838	1	C	T	0.785	0.062	0.009	1.3E-11	trans
2268	UXS1	Q8NBZ7	UDP-glucuronic acid decarboxylase 1	rs138588539	106804592	2	CT	C	0.078	0.175	0.014	1.6E-35	cis
2268	UXS1	Q8NBZ7	UDP-glucuronic acid decarboxylase 1	rs68066031	224880498	2	T	C	0.771	0.153	0.009	4.0E-64	trans
2268	UXS1	Q8NBZ7	UDP-glucuronic acid decarboxylase 1	rs710446	186459927	3	T	C	0.591	0.055	0.008	5.7E-13	trans
2268	UXS1	Q8NBZ7	UDP-glucuronic acid decarboxylase 1	rs71640036	187161120	4	G	T	0.494	0.127	0.008	9.6E-62	trans
2268	UXS1	Q8NBZ7	UDP-glucuronic acid decarboxylase 1	rs2731673	176839898	5	T	C	0.256	0.068	0.009	3.4E-15	trans
2268	UXS1	Q8NBZ7	UDP-glucuronic acid decarboxylase 1	rs1149607	76492446	11	C	A	0.818	0.238	0.010	3.5E-129	trans
2269	VAMP5	O95183	Vesicle-associated membrane protein 5	rs6547623	85801964	2	A	T	0.300	0.084	0.008	1.0E-23	cis
2270	VAMP8	Q9BV40	Vesicle-associated membrane protein 8	rs3731828	85806266	2	C	T	0.717	0.173	0.008	9.1E-94	cis
2271	VASH1	Q7L8A9	Tubulinyl-Tyr carboxypeptidase 1	rs1354034	56849749	3	C	T	0.604	0.103	0.008	6.3E-41	trans
2271	VASH1	Q7L8A9	Tubulinyl-Tyr carboxypeptidase 1	rs114694170	88180196	5	C	T	0.060	0.130	0.016	5.0E-16	trans
2271	VASH1	Q7L8A9	Tubulinyl-Tyr carboxypeptidase 1	rs11502185	180258	11	C	T	0.260	0.071	0.010	2.4E-13	trans
2271	VASH1	Q7L8A9	Tubulinyl-Tyr carboxypeptidase 1	rs11159226	77181432	14	G	T	0.060	0.210	0.016	1.2E-39	cis
2272	VASN	Q6EMK4	Vasorin	rs150816167	179571862	1	C	T	0.042	0.129	0.018	9.5E-13	trans
2272	VASN	Q6EMK4	Vasorin	rs1260326	27730940	2	C	T	0.609	0.064	0.007	2.2E-18	trans

2272	VASN	Q6EMK4	Vasorin	rs4841132	9183596	8	A	G	0.092	0.097	0.012	5.2E-15	trans
2272	VASN	Q6EMK4	Vasorin	rs7896518	65104500	10	G	A	0.424	0.053	0.007	3.1E-13	trans
2272	VASN	Q6EMK4	Vasorin	rs35713275	4431360	16	G	A	0.937	0.904	0.016	0.0E+00	cis
2272	VASN	Q6EMK4	Vasorin	rs77542162	67081278	17	G	A	0.023	0.187	0.024	5.1E-15	trans
2273	VASP	P50552	Vasodilator-stimulated phosphoprotein	rs1354034	56849749	3	C	T	0.604	0.057	0.008	6.8E-14	trans
2273	VASP	P50552	Vasodilator-stimulated phosphoprotein	rs342299	106373718	7	C	T	0.545	0.064	0.008	2.2E-17	trans
2274	VAT1	Q99536	Synaptic vesicle membrane protein VAT-1 homolog	rs1047891	211540507	2	C	A	0.688	0.065	0.008	8.8E-16	trans
2274	VAT1	Q99536	Synaptic vesicle membrane protein VAT-1 homolog	rs11787335	145044104	8	T	C	0.357	0.054	0.008	6.3E-12	trans
2274	VAT1	Q99536	Synaptic vesicle membrane protein VAT-1 homolog	rs11379524	95882745	9	AC	A	0.630	0.076	0.008	2.6E-22	trans
2274	VAT1	Q99536	Synaptic vesicle membrane protein VAT-1 homolog	rs4149307	107589744	9	T	C	0.153	0.095	0.010	5.1E-20	trans
2274	VAT1	Q99536	Synaptic vesicle membrane protein VAT-1 homolog	rs190543502	43757184	15	T	C	0.977	0.220	0.025	1.4E-18	trans
2274	VAT1	Q99536	Synaptic vesicle membrane protein VAT-1 homolog	rs173539	56988044	16	T	C	0.332	0.057	0.008	1.2E-12	trans
2274	VAT1	Q99536	Synaptic vesicle membrane protein VAT-1 homolog	rs200561116	67972194	16	AT	A	0.116	0.103	0.012	5.5E-18	trans
2274	VAT1	Q99536	Synaptic vesicle membrane protein VAT-1 homolog	rs7210098	41293613	17	C	T	0.665	0.141	0.008	7.8E-70	cis
2274	VAT1	Q99536	Synaptic vesicle membrane protein VAT-1 homolog	rs2868346	44547970	20	C	T	0.241	0.152	0.009	1.9E-68	trans
2275	VAV3	Q9UKW4	Guanine nucleotide exchange factor VAV3	rs345308	108459607	1	G	A	0.209	0.065	0.009	3.3E-12	cis
2275	VAV3	Q9UKW4	Guanine nucleotide exchange factor VAV3	rs1354034	56849749	3	C	T	0.604	0.053	0.008	5.8E-12	trans
2276	VCAM1	P19320	Vascular cell adhesion protein 1	rs139561173	101145190	1	A	C	0.789	0.105	0.009	6.4E-31	cis
2276	VCAM1	P19320	Vascular cell adhesion protein 1	rs2009581	111807677	2	G	A	0.728	0.059	0.008	9.4E-13	trans
2276	VCAM1	P19320	Vascular cell adhesion protein 1	rs1257169	134963862	2	C	A	0.501	0.110	0.007	6.0E-50	trans
2276	VCAM1	P19320	Vascular cell adhesion protein 1	rs35723031	182324042	2	G	GT	0.442	0.057	0.007	1.2E-14	trans
2276	VCAM1	P19320	Vascular cell adhesion protein 1	rs28418426	32619654	6	C	T	0.534	0.063	0.008	1.0E-14	trans
2276	VCAM1	P19320	Vascular cell adhesion protein 1	rs8176693	136137657	9	T	C	0.061	0.146	0.015	8.5E-22	trans
2276	VCAM1	P19320	Vascular cell adhesion protein 1	rs186021206	7069412	17	A	G	0.006	0.858	0.050	8.1E-65	trans
2276	VCAM1	P19320	Vascular cell adhesion protein 1	rs34557412	16852187	17	G	A	0.007	0.328	0.045	1.9E-13	trans
2276	VCAM1	P19320	Vascular cell adhesion protein 1	rs2092501	44347251	22	A	G	0.152	0.070	0.010	9.5E-12	trans
2277	VCAN	P13611	Versican core protein	rs3754053	161575844	1	G	C	0.576	0.052	0.007	9.2E-15	trans
2277	VCAN	P13611	Versican core protein	rs34552760	56189503	2	A	G	0.376	0.094	0.007	3.7E-44	trans
2277	VCAN	P13611	Versican core protein	rs12491835	16241579	3	T	C	0.764	0.053	0.008	4.8E-12	trans
2277	VCAN	P13611	Versican core protein	rs1229984	100239319	4	T	C	0.023	0.268	0.022	3.4E-34	trans
2277	VCAN	P13611	Versican core protein	rs13107325	103188709	4	T	C	0.075	0.153	0.012	1.0E-34	trans
2277	VCAN	P13611	Versican core protein	rs309559	82833369	5	A	G	0.519	0.649	0.007	0.0E+00	cis
2277	VCAN	P13611	Versican core protein	rs1917368	17911752	7	G	T	0.608	0.051	0.007	1.1E-14	trans
2277	VCAN	P13611	Versican core protein	rs11369732	99634456	7	GT	G	0.517	0.062	0.007	4.8E-21	trans
2277	VCAN	P13611	Versican core protein	rs12676683	19613524	8	C	A	0.907	0.210	0.011	3.8E-78	trans
2277	VCAN	P13611	Versican core protein	rs2945779	134541542	8	T	C	0.370	0.050	0.007	2.1E-13	trans
2277	VCAN	P13611	Versican core protein	rs2435381	43678796	10	T	C	0.275	0.088	0.007	6.4E-34	trans
2277	VCAN	P13611	Versican core protein	rs112680434	101768144	15	A	AGAATGT	0.635	0.058	0.007	6.8E-18	trans
2277	VCAN	P13611	Versican core protein	rs200489612	7106378	17	A	G	0.005	0.388	0.049	1.9E-15	trans
2277	VCAN	P13611	Versican core protein	rs2665397	57826731	17	A	C	0.544	0.056	0.006	3.6E-18	trans
2277	VCAN	P13611	Versican core protein	rs55639531	394341	18	G	C	0.285	0.059	0.007	2.7E-16	trans
2278	VCPKMT	Q9H867	Protein-lysine methyltransferase METTL21D	rs142528336	50581011	14	A	C	0.990	0.395	0.040	9.5E-23	cis
2278	VCPKMT	Q9H867	Protein-lysine methyltransferase METTL21D	rs3814952	106355582	14	G	A	0.774	0.069	0.010	1.5E-11	trans
2279	VEGFA	P15692	Vascular endothelial growth factor A	rs2274319	156450873	1	T	C	0.347	0.055	0.007	4.4E-16	trans
2279	VEGFA	P15692	Vascular endothelial growth factor A	rs114694170	88180196	5	C	T	0.060	0.167	0.014	6.7E-35	trans
2279	VEGFA	P15692	Vascular endothelial growth factor A	rs6921438	43925607	6	G	A	0.510	0.688	0.007	0.0E+00	cis
2279	VEGFA	P15692	Vascular endothelial growth factor A	rs13236689	80236014	7	G	T	0.407	0.050	0.006	1.2E-14	trans
2279	VEGFA	P15692	Vascular endothelial growth factor A	rs6993770	106581528	8	A	T	0.713	0.224	0.007	2.3E-216	trans
2279	VEGFA	P15692	Vascular endothelial growth factor A	rs2375981	2692583	9	C	G	0.556	0.190	0.007	6.7E-186	trans
2279	VEGFA	P15692	Vascular endothelial growth factor A	rs10761731	65027610	10	T	A	0.415	0.123	0.006	1.7E-79	trans
2279	VEGFA	P15692	Vascular endothelial growth factor A	rs12445050	81870969	16	T	C	0.138	0.071	0.009	1.1E-14	trans
2279	VEGFA	P15692	Vascular endothelial growth factor A	rs34416903	88559245	16	C	T	0.280	0.084	0.007	6.5E-31	trans
2279	VEGFA	P15692	Vascular endothelial growth factor A	rs892090	55539072	19	G	T	0.834	0.104	0.009	2.3E-33	trans
2279	VEGFA	P15692	Vascular endothelial growth factor A	rs3790176	19261922	20	A	G	0.342	0.054	0.007	7.4E-16	trans
2280	VEGFB	P49765	Vascular endothelial growth factor B	rs143543800	22161394	1	T	C	0.003	0.959	0.068	1.5E-44	trans
2280	VEGFB	P49765	Vascular endothelial growth factor B	rs188468174	25291697	1	C	T	0.986	0.538	0.032	3.4E-62	trans
2280	VEGFB	P49765	Vascular endothelial growth factor B	rs2305669	25915648	4	G	A	0.492	0.072	0.008	1.9E-21	trans
2280	VEGFB	P49765	Vascular endothelial growth factor B	rs7040440	117091074	9	C	T	0.919	0.146	0.015	1.3E-23	trans
2280	VEGFB	P49765	Vascular endothelial growth factor B	rs757081	17351683	11	C	G	0.668	0.068	0.008	2.5E-17	trans
2280	VEGFB	P49765	Vascular endothelial growth factor B	rs660442	64042997	11	A	G	0.200	0.115	0.009	3.6E-34	cis
2281	VEGFC	P49767	Vascular endothelial growth factor C	rs7618405	18250509	3	C	A	0.795	0.067	0.009	6.7E-13	trans
2281	VEGFC	P49767	Vascular endothelial growth factor C	rs1354034	56849749	3	T	C	0.396	0.071	0.008	6.4E-21	trans
2281	VEGFC	P49767	Vascular endothelial growth factor C	rs10016018	102794621	4	A	T	0.603	0.064	0.008	3.3E-17	trans
2281	VEGFC	P49767	Vascular endothelial growth factor C	rs35029317	177753427	4	A	G	0.994	0.797	0.049	7.6E-59	cis
2281	VEGFC	P49767	Vascular endothelial growth factor C	rs11242109	131677047	5	G	T	0.519	0.052	0.007	3.2E-12	trans
2281	VEGFC	P49767	Vascular endothelial growth factor C	rs35173808	31326148	6	C	A	0.904	0.098	0.013	1.4E-14	trans
2281	VEGFC	P49767	Vascular endothelial growth factor C	rs6961069	80218961	7	T	C	0.402	0.066	0.008	5.6E-18	trans
2281	VEGFC	P49767	Vascular endothelial growth factor C	rs6993770	106581528	8	A	T	0.714	0.088	0.008	1.8E-26	trans
2281	VEGFC	P49767	Vascular endothelial growth factor C	rs73000929	113953622	11	G	A	0.963	0.138	0.020	2.6E-12	trans
2281	VEGFC	P49767	Vascular endothelial growth factor C	rs59001897	65160392	15	A	T	0.175	0.071	0.010	1.0E-12	trans
2281	VEGFC	P49767	Vascular endothelial growth factor C	rs12445050	81870969	16	T	C	0.138	0.105	0.011	1.9E-22	trans
2281	VEGFC	P49767	Vascular endothelial growth factor C	rs1654425	55538980	19	C	T	0.835	0.163	0.010	1.0E-58	trans
2281	VEGFC	P49767	Vascular endothelial growth factor C	rs6081555	19245723	20	T	G	0.343	0.070	0.008	8.1E-19	trans
2282	VEGFD	O43915	Vascular endothelial growth factor D	rs710446	186459927	3	C	T	0.409	0.060	0.008	1.3E-15	trans
2282	VEGFD	O43915	Vascular endothelial growth factor D	rs12331618	187139939	4	G	A	0.512	0.142	0.007	4.8E-80	trans
2282	VEGFD	O43915	Vascular endothelial growth factor D	rs3217225	109190935	5	TCTC	T	0.834	0.080	0.010	9.0E-16	trans
2282	VEGFD	O43915	Vascular endothelial growth factor D	rs75077631	176840084	5	GC	G	0.742	0.083	0.008	7.6E-23	trans
2282	VEGFD	O43915	Vascular endothelial growth factor D	rs10982164	117091067	9	G	A	0.933	0.123	0.016	1.3E-14	trans
2282	VEGFD	O43915	Vascular endothelial growth factor D	rs56278466	17875857	10	G	T	0.661	0.069	0.008	1.2E-18	trans
2282	VEGFD	O43915	Vascular endothelial growth factor D	rs1037117	102068658	15	G	A	0.748	0.081	0.009	2.6E-21	trans
2282	VEGFD	O43915	Vascular endothelial growth factor D	rs192812042	15436623	X	A	G	0.129	0.760	0.009	0.0E+00	cis
2283	VIT	Q6UXI7	Vitrin	rs4233367	161163037	1	T	C	0.391	0.136	0.006	2.9E-115	trans
2283	VIT	Q6UXI7	Vitrin	rs1468810	36993940	2	C	A	0.414	0.432	0.007	0.0E+00	cis
2283	VIT	Q6UXI7	Vitrin	rs2577831	52628056	3	C	A	0.521	0.045	0.006	7.3E-15	trans
2283	VIT	Q6UXI7	Vitrin	rs13107325	103188709	4	T	C	0.075	0.113	0.011	1.8E-24	trans
2283	VIT	Q6UXI7	Vitrin	rs2142306	134470631	8	C	T	0.412	0.105	0.006	5.9E-70	trans
2283	VIT	Q6UXI7	Vitrin	rs186021206	7069412	17	A	G	0.006	0.612	0.040	5.2E-53	trans
2283	VIT	Q6UXI7	Vitrin	rs704	26694861	17	G	A	0.527	0.050	0.006	5.8E-18	trans
2283	VIT	Q6UXI7	Vitrin	rs2267372	38598234	22	G	A	0.600	0.044	0.006	4.9E-14	trans
2284	VMO1	Q7Z5L0	Vitelline membrane outer layer protein 1 homolog	rs117779442	19839709	8	C	T	0.926	0.073	0.011	1.7E-11	trans
2284	VMO1	Q7Z5L0	Vitelline membrane outer layer protein 1 homolog	rs112875651	126506694	8	G	A	0.609	0.043	0.006	2.1E-13	trans
2284	VMO1	Q7Z5L0	Vitelline membrane outer layer protein 1 homolog	rs964184	116648917	11	G	C	0.133	0.086	0.008	1.7E-24	trans
2284	VMO1	Q7Z5L0	Vitelline membrane outer layer protein 1 homolog	rs112597211	106369433	14	T	C	0.864	0.110	0.008	1.1E-39	trans
2284	VMO1	Q7Z5L0	Vitelline membrane outer layer protein 1 homolog	rs261290	58678720	15	T	C	0.344	0.127	0.006	6.9E-97	trans
2284	VMO1	Q7Z5L0	Vitelline membrane outer layer protein 1 homolog	rs8078118	4690038	17	A	C	0.417	0.755	0.008	0.0E+00	cis
2284	VMO1	Q7Z5L0	Vitelline membrane outer layer protein 1 homolog	rs3826688	45418961	19	C	T	0.641	0.047	0.006	3.8E-15	trans
2285	VNN1	O95497	Pantetheinase	rs1260326	27730940	2	T	C	0.391	0.054	0.005	8.6E-24	trans
2285	VNN1	O95497	Pantetheinase	rs13240065	73015369	7	A	G	0.127	0.054	0.008	5.1E-12	trans
2285	VNN1	O95497	Pantetheinase	rs4240624	9184231	8	G	A	0.092	0.082	0.009	2.6E-19	trans
2285	VNN1	O95497	Pantetheinase	rs9414801	65149089	10	G	A	0.467	0.038	0.005	3.6E-13	trans
2285	VNN1	O95497	Pantetheinase	rs174544	61567753	11	A	C	0.309	0.048	0.006	3.8E-17	trans
2285	VNN1	O95497	Pantetheinase	rs147233090	44028047	15	T	C	0.024	0.140	0.017	5.8E-16	trans
2285	VNN1	O95497	Pantetheinase	rs174418	58687603	15	C	T	0.598	0.059	0.005	4.2E-28	trans
2285	VNN1	O95497	Pantetheinase	rs429358	45411941	19	T	C	0.844	0.081	0.007	1.1E-28	trans
2285	VNN1	O95497	Pantetheinase	rs11668882	54675097	19	C	T	0.441	0.049	0.005	1.5E-20	trans
2286	VNN2	O95498	Vascular non-inflammatory molecule 2	rs7583027	62544391	2	C	A	0.643	0.046	0.007	4.9E-12	trans
2286	VNN2	O95498	Vascular non-inflammatory molecule 2	rs9493425	133074645	6	A	G	0.677	0.697	0.008	0.0E+00	cis
2286	VNN2	O95498	Vascular non-inflammatory molecule 2	rs56278466	17875857	10	G	T	0.661	0.067	0.007	4.0E-23	trans
2286	VNN2	O95498	Vascular non-inflammatory molecule 2	rs603424	102075479	10	G	A	0.834	0.063	0.009	3.7E-13	trans
2286	VNN2	O95498	Vascular non-inflammatory molecule 2	rs2511241	72945341	11	T	C	0.930	0.177	0.013	1.1E-44	trans
2286	VNN2	O95498	Vascular non-inflammatory molecule 2	rs73017399	126186312	11	C	G	0.239	0.074	0.008	1.2E-22	trans
2286	VNN2	O95498	Vascular non-inflammatory molecule 2	rs146261845	7012254	17	T	C	0.007	0.296	0.042	2.1E-12	trans
2286	VNN2	O95498	Vascular non-inflammatory molecule 2	rs7208815	38168715	17	T	C	0.392	0.101	0.007	2.0E-52	trans
2286	VNN2	O95498	Vascular non-inflammatory molecule 2	rs4760	44153100	19	A	G	0.844	0.184	0.009	1.7E-95	trans
2287	VPS28	Q9UK41	Vacuolar protein sorting-associated protein 28 homolog	rs72797306	131800750	5	G	C	0.570	0.055	0.008	5.7E-13	trans
2287	VPS28	Q9UK41	Vacuolar protein sorting-associated protein 28 homolog	rs4756952	18589236	11	T	C	0.335	0.085	0.008	1.7E-26	trans
2288	VPS37A	Q8NEZ2	Vacuolar protein sorting-associated protein 37A	rs1354034	56849749	3	C	T	0.605	0.053	0.008	4.2E-12	trans
2288	VPS37A	Q8NEZ2	Vacuolar protein sorting-associated protein 37A	rs964184	116648917	11	G	C	0.133	0.080	0.011	3.0E-13	trans
2289	VPS4B	O75351	Vacuolar protein sorting-associated protein 4B	rs1354034	56849749	3	C	T	0.604	0.059	0.008	2.2E-14	trans
2289	VPS4B	O75351	Vacuolar protein sorting-associated protein 4B	rs7916868	64988931	10	T	A	0.501	0.055	0.008	1.8E-13	trans
2290	VPS53	Q5VIR6	Vacuolar protein sorting-associated protein 53 homolog	rs1354034	56849749	3	C	T	0.605	0.055	0.008	3.8E-13	trans
2290	VPS53	Q5VIR6	Vacuolar protein sorting-associated protein 53 homolog	rs342296	106372903	7	G	A	0.544	0.053	0.007	2.0E-12	trans
2291	VSIG10	Q8N0Z9	V-set and immunoglobulin domain-containing protein 10	rs2519093	136141870	9	C	T	0.816	0.129	0.008	4.2E-54	trans
2291	VSIG10	Q8N0Z9	V-set and immunoglobulin domain-containing protein 10	rs200489612	7106378	17	A	G	0.005	0.394	0.048	2.0E-16	trans
2292	VSIG10L	Q86VR7	V-set and immunoglobulin domain-containing protein 10-like	rs1035454	51845556	19	C	T	0.576	0.208	0.008	9.5E-155	cis
2293	VSIG2	Q96IQ7	V-set and immunoglobulin domain-containing protein 2	rs4556017	100632790	7	C	T	0.147	0.088	0.010	7.5E-18	trans
2293	VSIG2	Q96IQ7	V-set and immunoglobulin domain-containing protein 2	rs8176741	136131461	9	A	G	0.061	0.210	0.015	2.5E-44	trans
2293	VSIG2	Q96IQ7	V-set and immunoglobulin domain-containing protein 2	rs6591649	60970849	11	G	A	0.345	0.137	0.008	5.5E-71	trans
2293	VSIG2	Q96IQ7	V-set and immunoglobulin domain-containing protein 2	rs11219769	124620147	11	T	G	0.256	0.213	0.008	1.2E-142	cis
2293	VSIG2	Q96IQ7	V-set and immunoglobulin domain-containing protein 2	rs708686	5840619	19	T	C	0.268	0.126	0.008	1.7E-54	trans
2293	VSIG2	Q96IQ7	V-set and immunoglobulin domain-containing protein 2	rs516246	49206172	19	C	T	0.491	0.083	0.007	3.8E-30	trans
2294	VSIG4	Q9Y279	V-set and immunoglobulin domain-containing protein 4	rs551397	196642072	1	C	T	0.770	0.066	0.009	1.7E-14	trans
2294	VSIG4	Q9Y279	V-set and immunoglobulin domain-containing protein 4	rs11130306	52541253	3	G	A	0.481	0.049	0.007	1.4E-11	trans
2294	VSIG4	Q9Y279	V-set and immunoglobulin domain-containing protein 4	rs12667978	128777461	7	T	C	0.571	0.049	0.007	1.2E-11	trans
2294	VSIG4	Q9Y279	V-set and immunoglobulin domain-containing protein 4	rs10769256	47378396	11	C	T	0.600	0.102	0.007	1.7E-43	trans
2294	VSIG4	Q9Y279	V-set and immunoglobulin domain-containing protein 4	rs28640218	20359267	16	G	T	0.809	0.064	0.009	4.0E-12	trans
2294	VSIG4	Q9Y279	V-set and immunoglobulin domain-containing protein 4	rs2230199	6718387	19	C	G	0.217	0.067	0.009	4.7E-14	trans
2294	VSIG4	Q9Y279	V-set and immunoglobulin domain-containing protein 4	rs190243615	58429702	X	C	T	0.991	0.963	0.033	1.1E-187	trans
2294	VSIG4	Q9Y279	V-set and immunoglobulin domain-containing protein 4	rs41306131	65253454	X	C	A	0.990	1.323	0.030	0.0E+00	cis
2295	VSIR	Q9H7M9	V-type immunoglobulin domain-containing suppressor of T-cell activation	rs13108400	102794613	4	A	G	0.603	0.054	0.008	1.0E-12	trans
2295	VSIR	Q9H7M9	V-type immunoglobulin domain-containing suppressor of T-cell activation	rs11242109	131677047	5	G	T	0.518	0.060	0.007	3.6E-16	trans
2295	VSIR	Q9H7M9	V-type immunoglobulin domain-containing suppressor of T-cell activation	rs6961069	80218961	7	T	C	0.402	0.057	0.008	5.2E-14	trans
2295	VSIR	Q9H7M9	V-type immunoglobulin domain-containing suppressor of T-cell activation	rs342299	106373718	7	C	T	0.545	0.058	0.007	3.2E-15	trans

2295	VSIR	Q9H7M9	V-type immunoglobulin domain-containing suppressor of T-cell activation	rs6993770	106581528	8	A	T	0.713	0.122	0.008	1.1E-50	trans
2295	VSIR	Q9H7M9	V-type immunoglobulin domain-containing suppressor of T-cell activation	rs10999992	73528898	10	C	T	0.147	0.440	0.011	0.0E+00	cis
2295	VSIR	Q9H7M9	V-type immunoglobulin domain-containing suppressor of T-cell activation	rs1654425	55538980	19	C	T	0.834	0.104	0.010	1.6E-25	trans
2296	VSNL1	P62760	Visinin-like protein 1	rs446947	17712696	2	T	A	0.276	0.313	0.008	0.0E+00	cis
2296	VSNL1	P62760	Visinin-like protein 1	rs5952	195306289	3	G	A	0.005	1.357	0.048	3.3E-176	trans
2296	VSNL1	P62760	Visinin-like protein 1	rs112875651	126506694	8	G	A	0.609	0.047	0.007	6.1E-12	trans
2296	VSNL1	P62760	Visinin-like protein 1	rs113609637	95903099	9	A	AT	0.858	0.069	0.010	4.4E-13	trans
2296	VSNL1	P62760	Visinin-like protein 1	rs4926	57381989	11	G	A	0.726	0.060	0.007	4.1E-16	trans
2296	VSNL1	P62760	Visinin-like protein 1	rs2289125	89224453	11	C	A	0.789	0.061	0.008	5.0E-14	trans
2296	VSNL1	P62760	Visinin-like protein 1	rs10405357	54759666	19	T	C	0.564	0.050	0.007	7.8E-14	trans
2297	VSTM1	Q6UX27	V-set and transmembrane domain-containing protein 1	rs60816814	29888602	6	A	C	0.045	0.183	0.016	1.6E-31	trans
2297	VSTM1	Q6UX27	V-set and transmembrane domain-containing protein 1	rs2433724	54545531	19	T	C	0.571	0.768	0.008	0.0E+00	cis
2297	VSTM1	Q6UX27	V-set and transmembrane domain-containing protein 1	rs189448562	3705115	20	C	G	0.011	0.225	0.029	6.4E-15	trans
2298	VSTM2L	Q96N03	V-set and transmembrane domain-containing protein 2-like protein	rs646776	109818530	1	T	C	0.778	0.209	0.009	1.7E-113	trans
2298	VSTM2L	Q96N03	V-set and transmembrane domain-containing protein 2-like protein	rs2273349	36624756	20	C	A	0.816	0.146	0.010	2.1E-48	cis
2299	VTA1	Q9NP79	Vacuolar protein sorting-associated protein VTA1 homolog	rs1354034	56849749	3	C	T	0.604	0.075	0.008	1.0E-22	trans
2299	VTA1	Q9NP79	Vacuolar protein sorting-associated protein VTA1 homolog	rs10900809	131826322	5	G	A	0.611	0.058	0.008	3.7E-14	trans
2299	VTA1	Q9NP79	Vacuolar protein sorting-associated protein VTA1 homolog	rs10733789	64948684	10	C	T	0.313	0.058	0.008	8.2E-13	trans
2300	VTCN1	Q7Z7D3	V-set domain-containing T-cell activation inhibitor 1	rs56174814	117723307	1	A	T	0.157	0.108	0.011	6.5E-24	cis
2300	VTCN1	Q7Z7D3	V-set domain-containing T-cell activation inhibitor 1	rs516316	49206145	19	C	G	0.509	0.078	0.008	1.1E-23	trans
2301	VTI1A	Q96AJ9	Vesicle transport through interaction with t-SNAREs homolog 1A	rs1354034	56849749	3	C	T	0.604	0.082	0.008	5.4E-27	trans
2301	VTI1A	Q96AJ9	Vesicle transport through interaction with t-SNAREs homolog 1A	rs342299	106373718	7	C	T	0.545	0.053	0.008	1.4E-12	trans
2301	VTI1A	Q96AJ9	Vesicle transport through interaction with t-SNAREs homolog 1A	rs6993770	106581528	8	A	T	0.713	0.073	0.008	1.7E-18	trans
2301	VTI1A	Q96AJ9	Vesicle transport through interaction with t-SNAREs homolog 1A	rs7896518	65104500	10	G	A	0.424	0.056	0.008	2.9E-13	trans
2301	VTI1A	Q96AJ9	Vesicle transport through interaction with t-SNAREs homolog 1A	rs79671623	114141103	10	G	A	0.882	0.086	0.012	1.2E-13	cis
2302	VWA1	Q6PCB0	von Willebrand factor A domain-containing protein 1	rs115503338	1369623	1	G	A	0.986	1.494	0.033	0.0E+00	cis
2302	VWA1	Q6PCB0	von Willebrand factor A domain-containing protein 1	rs35457250	186338564	3	T	C	0.009	0.525	0.039	2.3E-41	trans
2302	VWA1	Q6PCB0	von Willebrand factor A domain-containing protein 1	rs6054	155489608	4	T	C	0.006	0.398	0.049	5.0E-16	trans
2302	VWA1	Q6PCB0	von Willebrand factor A domain-containing protein 1	rs28929474	94844947	14	T	C	0.021	0.240	0.026	7.4E-21	trans
2302	VWA1	Q6PCB0	von Willebrand factor A domain-containing protein 1	rs1801689	64210580	17	C	A	0.030	0.265	0.021	3.9E-35	trans
2302	VWA1	Q6PCB0	von Willebrand factor A domain-containing protein 1	rs7256200	45415935	19	G	T	0.872	0.085	0.011	1.3E-14	trans
2303	VWC2	Q2TAL6	Brorin	rs61747728	179526214	1	T	C	0.038	0.129	0.017	1.9E-13	trans
2303	VWC2	Q2TAL6	Brorin	rs769604	49815390	7	C	G	0.541	0.458	0.007	0.0E+00	cis
2304	VWC2L	B2RUY7	von Willebrand factor C domain-containing protein 2-like	rs10932542	215390981	2	A	G	0.241	0.099	0.009	2.1E-30	cis
2305	VWF	P04275	von Willebrand factor	rs9386182	147691069	6	T	A	0.558	0.065	0.007	4.1E-18	trans
2305	VWF	P04275	von Willebrand factor	rs2726953	27801305	8	A	G	0.295	0.058	0.008	7.2E-13	trans
2306	WARS	P23381	Tryptophan--tRNA ligase, cytoplasmic	rs2273804	100842637	14	C	G	0.740	0.258	0.009	1.9E-199	cis
2307	WAS	P42768	Wiskott-Aldrich syndrome protein	rs5030082	186458949	3	G	A	0.405	0.062	0.008	1.4E-15	trans
2307	WAS	P42768	Wiskott-Aldrich syndrome protein	rs4861708	187157233	4	A	G	0.512	0.113	0.008	2.6E-49	trans
2307	WAS	P42768	Wiskott-Aldrich syndrome protein	rs1801020	176836532	5	G	A	0.745	0.087	0.009	2.2E-23	trans
2308	WASF1	Q92558	Wiskott-Aldrich syndrome protein family member 1	rs1980532	110720209	6	A	G	0.555	0.287	0.008	0.0E+00	cis
2308	WASF1	Q92558	Wiskott-Aldrich syndrome protein family member 1	rs7916868	64988931	10	T	A	0.502	0.050	0.007	1.7E-11	trans
2309	WASHC3	Q9Y3C0	WASH complex subunit 3	rs1354034	56849749	3	C	T	0.604	0.055	0.008	4.6E-13	trans
2309	WASHC3	Q9Y3C0	WASH complex subunit 3	rs342293	106372219	7	C	G	0.540	0.058	0.007	8.1E-15	trans
2309	WASHC3	Q9Y3C0	WASH complex subunit 3	rs707234	83804256	16	T	A	0.656	0.083	0.008	1.1E-26	trans
2310	WASL	O00401	Neural Wiskott-Aldrich syndrome protein	rs3087243	204738919	2	G	A	0.548	0.064	0.008	1.4E-16	trans
2311	WFDC1	Q9HC57	WAP four-disulfide core domain protein 1	rs68066031	224880498	2	T	C	0.770	0.202	0.009	2.7E-110	trans
2311	WFDC1	Q9HC57	WAP four-disulfide core domain protein 1	rs400345	84328494	16	C	T	0.816	0.357	0.010	2.2E-281	cis
2312	WFDC12	Q8WWY7	WAP four-disulfide core domain protein 12	rs61816761	152285861	1	A	G	0.024	0.730	0.021	4.6E-261	trans
2312	WFDC12	Q8WWY7	WAP four-disulfide core domain protein 12	rs11264533	156718448	1	C	G	0.335	0.054	0.006	2.5E-17	trans
2312	WFDC12	Q8WWY7	WAP four-disulfide core domain protein 12	rs17350445	187632339	3	C	T	0.936	0.120	0.012	2.6E-22	trans
2312	WFDC12	Q8WWY7	WAP four-disulfide core domain protein 12	rs9845881	190029282	3	C	T	0.590	0.073	0.006	6.1E-33	trans
2312	WFDC12	Q8WWY7	WAP four-disulfide core domain protein 12	rs10979132	110720207	9	T	A	0.832	0.054	0.008	1.1E-11	trans
2312	WFDC12	Q8WWY7	WAP four-disulfide core domain protein 12	rs10483947	80775115	14	T	C	0.093	0.079	0.010	2.2E-14	trans
2312	WFDC12	Q8WWY7	WAP four-disulfide core domain protein 12	rs8038032	35302869	15	A	G	0.677	0.084	0.006	4.1E-39	trans
2312	WFDC12	Q8WWY7	WAP four-disulfide core domain protein 12	rs35673728	41047777	15	T	C	0.937	0.177	0.012	1.0E-47	trans
2312	WFDC12	Q8WWY7	WAP four-disulfide core domain protein 12	rs6508781	38785318	19	A	C	0.831	0.134	0.008	4.8E-63	trans
2312	WFDC12	Q8WWY7	WAP four-disulfide core domain protein 12	rs75002084	43755307	20	C	G	0.569	0.425	0.007	0.0E+00	cis
2312	WFDC12	Q8WWY7	WAP four-disulfide core domain protein 12	rs2283638	40176111	21	C	A	0.673	0.061	0.006	4.5E-22	trans
2313	WFDC2	Q14508	WAP four-disulfide core domain protein 2	rs77924615	20392332	16	G	A	0.803	0.074	0.009	1.0E-16	trans
2313	WFDC2	Q14508	WAP four-disulfide core domain protein 2	rs28894750	49213531	19	T	A	0.534	0.062	0.007	2.1E-18	trans
2313	WFDC2	Q14508	WAP four-disulfide core domain protein 2	rs973446	44094375	20	C	G	0.922	0.139	0.013	1.9E-26	cis
2314	WFIKKN1	Q96NZ8	WAP, Kazal, immunoglobulin, Kunitz and NTR domain-containing protein 1	rs72654647	25022314	1	A	G	0.245	0.071	0.008	4.3E-18	trans
2314	WFIKKN1	Q96NZ8	WAP, Kazal, immunoglobulin, Kunitz and NTR domain-containing protein 1	rs11676298	227291731	2	C	G	0.808	0.082	0.009	2.7E-20	trans
2314	WFIKKN1	Q96NZ8	WAP, Kazal, immunoglobulin, Kunitz and NTR domain-containing protein 1	rs79431137	123099518	3	G	A	0.220	0.071	0.008	5.3E-17	trans
2314	WFIKKN1	Q96NZ8	WAP, Kazal, immunoglobulin, Kunitz and NTR domain-containing protein 1	rs34250475	154430042	4	T	C	0.454	0.048	0.007	1.2E-11	trans
2314	WFIKKN1	Q96NZ8	WAP, Kazal, immunoglobulin, Kunitz and NTR domain-containing protein 1	rs27659	96343552	5	A	G	0.449	0.048	0.007	9.1E-12	trans
2314	WFIKKN1	Q96NZ8	WAP, Kazal, immunoglobulin, Kunitz and NTR domain-containing protein 1	rs2647074	32574360	6	T	C	0.327	0.091	0.007	5.1E-34	trans
2314	WFIKKN1	Q96NZ8	WAP, Kazal, immunoglobulin, Kunitz and NTR domain-containing protein 1	rs3731211	21986847	9	T	A	0.278	0.082	0.008	7.6E-26	trans
2314	WFIKKN1	Q96NZ8	WAP, Kazal, immunoglobulin, Kunitz and NTR domain-containing protein 1	rs183805019	102347007	9	T	C	0.996	0.457	0.060	1.8E-14	trans
2314	WFIKKN1	Q96NZ8	WAP, Kazal, immunoglobulin, Kunitz and NTR domain-containing protein 1	rs4937333	128330520	11	C	T	0.526	0.052	0.007	3.0E-13	trans
2314	WFIKKN1	Q96NZ8	WAP, Kazal, immunoglobulin, Kunitz and NTR domain-containing protein 1	rs55798945	669708	16	G	T	0.304	0.278	0.008	2.6E-259	cis
2314	WFIKKN1	Q96NZ8	WAP, Kazal, immunoglobulin, Kunitz and NTR domain-containing protein 1	rs34557412	16852187	17	G	A	0.007	0.392	0.042	2.4E-20	trans
2314	WFIKKN1	Q96NZ8	WAP, Kazal, immunoglobulin, Kunitz and NTR domain-containing protein 1	rs1883932	8609588	20	T	A	0.509	0.112	0.007	2.7E-58	trans
2315	WFIKKN2	Q8TEU8	WAP, Kazal, immunoglobulin, Kunitz and NTR domain-containing protein 2	rs6664906	31354813	1	T	C	0.536	0.047	0.007	2.2E-12	trans
2315	WFIKKN2	Q8TEU8	WAP, Kazal, immunoglobulin, Kunitz and NTR domain-containing protein 2	rs4546329	60589739	5	C	T	0.503	0.050	0.007	5.4E-14	trans
2315	WFIKKN2	Q8TEU8	WAP, Kazal, immunoglobulin, Kunitz and NTR domain-containing protein 2	rs4591185	48909434	17	T	A	0.342	0.680	0.008	0.0E+00	cis
2315	WFIKKN2	Q8TEU8	WAP, Kazal, immunoglobulin, Kunitz and NTR domain-containing protein 2	rs33950747	36339247	19	T	C	0.075	0.140	0.013	9.8E-29	trans
2316	WIF1	Q9Y5W5	Wnt inhibitory factor 1	rs1414660	240586695	1	T	C	0.194	0.084	0.009	2.6E-20	trans
2316	WIF1	Q9Y5W5	Wnt inhibitory factor 1	rs6768977	12508169	3	G	A	0.534	0.053	0.007	1.4E-13	trans
2316	WIF1	Q9Y5W5	Wnt inhibitory factor 1	rs10104997	55440068	8	C	T	0.790	0.102	0.009	4.6E-31	trans
2316	WIF1	Q9Y5W5	Wnt inhibitory factor 1	rs10963680	18629283	9	A	G	0.243	0.057	0.008	1.5E-11	trans
2316	WIF1	Q9Y5W5	Wnt inhibitory factor 1	rs7896518	65104500	10	G	A	0.424	0.061	0.007	7.3E-17	trans
2316	WIF1	Q9Y5W5	Wnt inhibitory factor 1	rs10741178	130903004	10	C	T	0.778	0.062	0.009	1.2E-12	trans
2316	WIF1	Q9Y5W5	Wnt inhibitory factor 1	rs7949566	126285301	11	A	G	0.422	0.069	0.007	1.3E-21	trans
2316	WIF1	Q9Y5W5	Wnt inhibitory factor 1	rs12894709	75441734	14	G	A	0.526	0.055	0.007	2.1E-14	trans
2316	WIF1	Q9Y5W5	Wnt inhibitory factor 1	rs117068593	93118229	14	C	T	0.813	0.078	0.009	4.1E-17	trans
2316	WIF1	Q9Y5W5	Wnt inhibitory factor 1	rs186021206	7069412	17	A	G	0.006	0.488	0.049	6.4E-23	trans
2316	WIF1	Q9Y5W5	Wnt inhibitory factor 1	rs2145943	38563245	20	G	A	0.407	0.098	0.007	1.4E-40	trans
2317	WNT9A	O14904	Protein Wnt-9a	rs4653881	228079939	1	C	T	0.622	0.129	0.007	6.6E-72	cis
2317	WNT9A	O14904	Protein Wnt-9a	rs9274045	32630055	6	T	C	0.833	0.069	0.009	1.6E-13	trans
2318	WWP2	O00308	NEDD4-like E3 ubiquitin-protein ligase WWP2	rs1354034	56849749	3	C	T	0.604	0.082	0.008	8.5E-28	trans
2318	WWP2	O00308	NEDD4-like E3 ubiquitin-protein ligase WWP2	rs342293	106372219	7	C	G	0.541	0.058	0.007	6.1E-15	trans
2318	WWP2	O00308	NEDD4-like E3 ubiquitin-protein ligase WWP2	rs72783174	69809678	16	C	T	0.922	0.304	0.014	1.8E-107	cis
2319	XCL1	P47992	Lymphotactin	rs4656599	168503386	1	T	C	0.190	0.833	0.010	0.0E+00	cis
2319	XCL1	P47992	Lymphotactin	rs2009581	111807677	2	G	A	0.727	0.148	0.007	3.4E-94	trans
2319	XCL1	P47992	Lymphotactin	rs13063578	47087837	3	T	A	0.602	0.089	0.007	3.0E-42	trans
2319	XCL1	P47992	Lymphotactin	rs2548993	131808869	5	A	G	0.722	0.067	0.007	1.1E-20	trans
2319	XCL1	P47992	Lymphotactin	rs9265813	31310540	6	T	A	0.170	0.074	0.009	1.4E-17	trans
2319	XCL1	P47992	Lymphotactin	rs705379	94953895	7	A	G	0.477	0.063	0.006	9.6E-23	trans
2319	XCL1	P47992	Lymphotactin	rs8177651	6015390	10	A	T	0.324	0.051	0.007	2.0E-13	trans
2319	XCL1	P47992	Lymphotactin	rs77542162	67081278	17	G	A	0.023	0.268	0.021	4.5E-36	trans
2319	XCL1	P47992	Lymphotactin	rs6031304	42657548	20	C	T	0.726	0.049	0.007	7.8E-12	trans
2320	XG	P55808	Glycoprotein Xg	rs28579419	2699645	X	T	G	0.147	0.148	0.007	1.1E-108	cis
2321	XIAP	P98170	E3 ubiquitin-protein ligase XIAP	rs1354034	56849749	3	C	T	0.604	0.080	0.008	1.6E-25	trans
2322	XPNPEP2	O43895	Xaa-Pro aminopeptidase 2	rs114165349	27021913	1	C	G	0.023	0.420	0.025	7.2E-65	trans
2322	XPNPEP2	O43895	Xaa-Pro aminopeptidase 2	rs1260326	27730940	2	T	C	0.391	0.070	0.008	2.4E-20	trans
2322	XPNPEP2	O43895	Xaa-Pro aminopeptidase 2	rs1584300	61586001	2	C	A	0.149	0.072	0.010	3.1E-12	trans
2322	XPNPEP2	O43895	Xaa-Pro aminopeptidase 2	rs687339	135932359	3	T	C	0.771	0.061	0.009	5.3E-12	trans
2322	XPNPEP2	O43895	Xaa-Pro aminopeptidase 2	rs2851270	100027351	4	G	A	0.337	0.080	0.008	1.8E-24	trans
2322	XPNPEP2	O43895	Xaa-Pro aminopeptidase 2	rs10822143	64887856	10	C	T	0.499	0.061	0.007	2.5E-16	trans
2322	XPNPEP2	O43895	Xaa-Pro aminopeptidase 2	rs11838867	91981823	13	G	A	0.975	0.164	0.024	7.2E-12	trans
2322	XPNPEP2	O43895	Xaa-Pro aminopeptidase 2	rs11621792	24871926	14	T	C	0.453	0.076	0.007	2.1E-24	trans
2322	XPNPEP2	O43895	Xaa-Pro aminopeptidase 2	rs2498786	105262368	14	C	G	0.381	0.083	0.008	1.5E-27	trans
2322	XPNPEP2	O43895	Xaa-Pro aminopeptidase 2	rs339969	60883281	15	A	C	0.616	0.094	0.008	6.0E-35	trans
2322	XPNPEP2	O43895	Xaa-Pro aminopeptidase 2	rs60018147	3375572	19	A	G	0.881	0.092	0.012	1.7E-14	trans
2322	XPNPEP2	O43895	Xaa-Pro aminopeptidase 2	rs2207132	39142516	20	G	A	0.967	0.219	0.021	1.1E-25	trans
2322	XPNPEP2	O43895	Xaa-Pro aminopeptidase 2	rs1800961	43042364	20	C	T	0.968	0.227	0.021	2.0E-27	trans
2322	XPNPEP2	O43895	Xaa-Pro aminopeptidase 2	rs11096255	128865061	X	G	A	0.788	0.830	0.008	0.0E+00	cis
2322	XPNPEP2	O43895	Xaa-Pro aminopeptidase 2	rs187650585	132923525	X	C	T	0.997	0.536	0.063	1.8E-17	trans
2323	XRCC4	Q13426	DNA repair protein XRCC4	rs1805377	82648943	5	A	G	0.107	0.104	0.012	6.6E-17	cis
2324	YAP1	P46937	Transcriptional coactivator YAP1	rs542212699	223900610	1	G	A	0.991	0.622	0.037	3.8E-63	trans
2324	YAP1	P46937	Transcriptional coactivator YAP1	rs26515	96054837	5	T	C	0.548	0.142	0.007	1.7E-93	trans
2324	YAP1	P46937	Transcriptional coactivator YAP1	rs10791554	101923553	11	A	G	0.453	0.062	0.007	1.8E-19	cis
2324	YAP1	P46937	Transcriptional coactivator YAP1	rs77924615	20392332	16	G	A	0.803	0.061	0.009	2.6E-12	trans
2325	YARS1	P54577	Tyrosine--tRNA ligase, cytoplasmic	rs1354034	56849749	3	C	T	0.605	0.061	0.008	1.1E-15	trans
2325	YARS1	P54577	Tyrosine--tRNA ligase, cytoplasmic	rs342298	106373646	7	C	T	0.545	0.059	0.008	5.8E-15	trans
2325	YARS1	P54577	Tyrosine--tRNA ligase, cytoplasmic	rs6993770	106581528	8	A	T	0.713	0.067	0.008	6.2E-16	trans
2326	YES1	P07947	Tyrosine-protein kinase Yes	rs1354034	56849749	3	C	T	0.605	0.059	0.008	7.3E-15	trans
2326	YES1	P07947	Tyrosine-protein kinase Yes	rs2741189	707511	18	C	G	0.229	0.085	0.009	8.4E-22	cis
2327	YOD1	Q5VVQ6	Ubiquitin thioesterase OTU1	rs1044145	207217359	1	C	T	0.537	0.106	0.007	1.4E-46	cis
2327	YOD1	Q5VVQ6	Ubiquitin thioesterase OTU1	rs3181215	27077331	17	A	G	0.797	0.076	0.009	6.5E-17	trans
2328	YTHDF3	Q7Z739	YTH domain-containing family protein 3	rs1354034	56849749	3	C	T	0.604	0.086	0.008	7.1E-29	trans
2328	YTHDF3	Q7Z739	YTH domain-containing family protein 3	rs11502185	180258	11	C	T	0.259	0.073	0.010	8.2E-14	trans
2329	YWHAQ	P27348	14-3-3 protein theta	rs1354034	56849749	3	C	T	0.604	0.067	0.008	1.9E-18	trans
2329	YWHAQ	P27348	14-3-3 protein theta	rs7080386	65048306	10	A	C	0.412	0.060	0.008	4.8E-15	trans
2330	ZBP1	Q9H171	Z-DNA-binding protein 1	rs45521631	56195349	20	C	T	0.973	0.909	0.024	0.0E+00	cis
2331	ZBTB16	Q05516	Zinc finger and BTB domain-containing protein 16	rs1354034	56849749	3	C	T	0.604	0.064	0.007	2.0E-18	trans
2331	ZBTB16	Q05516	Zinc finger and BTB domain-containing protein 16	rs1265905	31909941	6	A	G	0.870	0.077	0.011	6.7E-13	trans
2331	ZBTB16	Q05516	Zinc finger and BTB domain-containing protein 16	rs342296	106372903	7	G	A	0.543	0.059	0.007	6.3E-16	trans
2331	ZBTB16	Q05516	Zinc finger and BTB domain-containing protein 16	rs6993770	106581528	8	A	T	0.713	0.060	0.008	5.9E-14	trans
2331	ZBTB16	Q05516	Zinc finger and BTB domain-containing protein 16	rs12767683	104316581	10	A	C	0.325	0.079	0.008	2.0E-24	trans
2331	ZBTB16	Q05516	Zinc finger and BTB domain-containing protein 16	rs73000929	113953622	11	A	G	0.037	0.852	0.020	0.0E+00	cis

2331	ZBTB16	Q05516	Zinc finger and BTB domain-containing protein 16	rs3002416	39710195	X	T	C	0.408	0.050	0.006	1.5E-15	trans
2332	ZBTB17	Q13105	Zinc finger and BTB domain-containing protein 17	rs7080536	115348046	10	G	A	0.956	0.161	0.019	7.5E-18	trans
2332	ZBTB17	Q13105	Zinc finger and BTB domain-containing protein 17	rs7260293	53229394	19	T	C	0.621	0.073	0.008	1.3E-20	trans
2333	ZFYVE19	Q96K21	Abscission/NoCut checkpoint regulator	rs1354034	56849749	3	C	T	0.605	0.056	0.007	8.2E-14	trans
2333	ZFYVE19	Q96K21	Abscission/NoCut checkpoint regulator	rs342298	106373646	7	C	T	0.545	0.064	0.007	4.7E-18	trans
2333	ZFYVE19	Q96K21	Abscission/NoCut checkpoint regulator	rs7896518	65104500	10	G	A	0.424	0.084	0.007	5.8E-29	trans
2333	ZFYVE19	Q96K21	Abscission/NoCut checkpoint regulator	rs11858010	41099163	15	T	C	0.645	0.374	0.008	0.0E+00	cis
2334	ZNF830	Q96NB3	Zinc finger protein 830	rs617119	32455767	3	T	C	0.513	0.060	0.008	2.7E-15	trans
2334	ZNF830	Q96NB3	Zinc finger protein 830	rs3018449	103579609	8	T	C	0.736	0.060	0.009	2.1E-12	trans
2335	ZNRD2	O60232	Protein ZNRD2	rs1354034	56849749	3	C	T	0.605	0.077	0.008	3.4E-24	trans
2335	ZNRD2	O60232	Protein ZNRD2	rs35523137	196220979	3	C	CA	0.640	0.053	0.008	5.5E-12	trans
2335	ZNRD2	O60232	Protein ZNRD2	rs204893	32094593	6	C	T	0.431	0.089	0.007	2.1E-32	trans
2335	ZNRD2	O60232	Protein ZNRD2	rs342293	106372219	7	C	G	0.540	0.062	0.007	3.9E-17	trans
2335	ZNRD2	O60232	Protein ZNRD2	rs35923643	123355391	11	A	G	0.804	0.081	0.009	6.1E-18	trans
2335	ZNRD2	O60232	Protein ZNRD2	rs6497185	94802545	15	C	A	0.274	0.072	0.008	5.3E-18	trans
2336	ZNRF4	Q8WWF5	E3 ubiquitin-protein ligase ZNRF4	rs10914144	171949750	1	C	T	0.803	0.222	0.010	8.7E-119	trans
2336	ZNRF4	Q8WWF5	E3 ubiquitin-protein ligase ZNRF4	rs10822145	64934548	10	T	C	0.472	0.056	0.008	2.3E-13	trans
2337	ZP3	P21754	Zona pellucida sperm-binding protein 3	rs17718444	71499401	3	T	C	0.319	0.053	0.005	4.8E-27	trans
2337	ZP3	P21754	Zona pellucida sperm-binding protein 3	rs9275559	32677534	6	A	C	0.441	0.034	0.005	3.4E-13	trans
2337	ZP3	P21754	Zona pellucida sperm-binding protein 3	rs10281089	76017997	7	T	C	0.431	1.105	0.008	0.0E+00	cis
2337	ZP3	P21754	Zona pellucida sperm-binding protein 3	rs1265831	28912850	10	C	G	0.737	0.106	0.005	7.5E-91	trans
2337	ZP3	P21754	Zona pellucida sperm-binding protein 3	rs3747481	30666367	16	C	T	0.742	0.036	0.005	9.1E-12	trans
2337	ZP3	P21754	Zona pellucida sperm-binding protein 3	rs8073937	7435040	17	G	A	0.348	0.051	0.005	7.7E-26	trans
2337	ZP3	P21754	Zona pellucida sperm-binding protein 3	rs4807125	1646712	19	C	T	0.849	0.069	0.006	9.7E-27	trans

Supplementary Table 2. Association between blood plasma protein and ovarian cancer or its subtypes, with estimates from mendelian randomisation analysis (p-pleoptropy in red are those with MR-egger intercept test p-value below 0.05. Cells in light blue are those that passed the MR-Egger intercept and leave-one-out tests. nsnp is number of quantitative traite loci used as instruments)

				Ovarian cancer (OC)					Serous OC					Endometrioid OC					Clear cell OC					Mucinous OC				
				25509 cases and 40941 controls					14049 cases and 40941 controls					2810 cases and 40941 controls					1366 cases and 40941 controls					1417 cases and 40941 controls				
seqid	protein	nsnp	method	or	lci	uci	pval	p-pleotropy	or.1	lci.1	uci.1	pval.1	p-pleotropy	or.2	lci.2	uci.2	pval.2	p-pleotropy	or.3	lci.3	uci.3	pval.3	p-pleotropy	or.4	lci.4	uci.4	pval.4	p-pleotropy
1	LRRC37A2	5	Inverse variance weighted	1.1	1.07	1.14	1.2E-09	8.0E-01	1.11	1.07	1.14	1.3E-10	8.1E-01	1.09	1.01	1.17	2.4E-02	6.6E-01	1.02	0.94	1.11	5.7E-01	0.36	1.1	1.01	1.19	2.2E-02	2.1E-01
1	LRRC37A2	5	Weighted median	1.1	1.07	1.13	1.0E-12	8.0E-01	1.11	1.07	1.14	2.3E-10	8.1E-01	1.09	1.03	1.16	3.8E-03	6.6E-01	1.03	0.95	1.12	5.1E-01	0.36	1.1	1.02	1.2	1.8E-02	2.1E-01
1	LRRC37A2	5	Weighted mode	1.1	1.06	1.15	2.7E-03	8.0E-01	1.11	1.06	1.16	2.8E-03	8.1E-01	1.09	1	1.19	4.5E-02	6.6E-01	1.03	0.91	1.16	5.8E-01	0.36	1.1	0.98	1.24	8.8E-02	2.1E-01
1	LRRC37A2	5	MR Egger	1.1	1.02	1.18	3.0E-02	8.0E-01	1.1	1.03	1.18	1.7E-02	8.1E-01	1.07	0.9	1.27	3.0E-01	6.6E-01	1.06	0.89	1.27	3.4E-01	3.6E-01	1.16	0.98	1.38	7.0E-02	2.1E-01
2	PTPRM	10	Inverse variance weighted	0.89	0.83	0.95	8.0E-04	3.3E-01	0.85	0.79	0.92	4.1E-05	5.7E-01	0.91	0.81	1.02	9.8E-02	3.8E-02	0.99	0.87	1.14	9.3E-01	8.5E-01	0.86	0.75	0.98	2.2E-02	2.6E-01
2	PTPRM	10	Weighted median	0.87	0.83	0.91	9.5E-09	3.3E-01	0.85	0.8	0.89	2.0E-10	5.7E-01	0.89	0.81	0.98	2.0E-02	3.8E-02	1	0.87	1.16	9.8E-01	8.5E-01	0.83	0.73	0.95	6.2E-03	2.6E-01
2	PTPRM	10	Weighted mode	0.88	0.83	0.93	4.7E-04	3.3E-01	0.84	0.8	0.9	1.3E-04	5.7E-01	0.89	0.79	1	4.7E-02	3.8E-02	1	0.85	1.17	1.0E+00	8.5E-01	0.83	0.71	0.97	2.7E-02	2.6E-01
2	PTPRM	10	MR Egger	0.87	0.78	0.96	9.7E-03	3.3E-01	0.84	0.75	0.94	6.9E-03	5.7E-01	0.84	0.73	0.96	1.7E-02	3.8E-02	0.99	0.81	1.2	8.6E-01	8.5E-01	0.81	0.68	0.98	3.3E-02	2.6E-01
3	PECAM1	4	Inverse variance weighted	0.86	0.81	0.9	1.8E-08	9.7E-01	0.83	0.78	0.88	8.7E-10	8.8E-01	0.86	0.77	0.97	1.3E-02	9.9E-01	0.99	0.85	1.17	9.5E-01	5.1E-01	0.82	0.7	0.97	1.9E-02	8.3E-01
3	PECAM1	4	Weighted median	0.85	0.81	0.9	1.7E-08	9.7E-01	0.82	0.77	0.87	4.0E-10	8.8E-01	0.87	0.77	0.98	1.8E-02	9.9E-01	1	0.85	1.18	9.9E-01	5.1E-01	0.82	0.7	0.95	1.1E-02	8.3E-01
3	PECAM1	4	Weighted mode	0.85	0.78	0.93	9.1E-03	9.7E-01	0.82	0.74	0.9	7.3E-03	8.8E-01	0.87	0.72	1.05	1.1E-01	9.9E-01	1	0.76	1.32	9.7E-01	5.1E-01	0.8	0.62	1.03	6.6E-02	8.3E-01
3	PECAM1	4	MR Egger	0.86	0.69	1.06	8.9E-02	9.7E-01	0.82	0.64	1.05	7.5E-02	8.8E-01	0.87	0.59	1.27	2.5E-01	9.9E-01	1.07	0.62	1.85	6.3E-01	5.1E-01	0.85	0.43	1.66	4.0E-01	8.3E-01
4	ROBO4	3	Inverse variance weighted	0.85	0.78	0.92	4.9E-05	5.9E-01	0.81	0.75	0.88	1.3E-06	4.4E-01	0.85	0.75	0.97	1.4E-02	6.0E-01	1.02	0.77	1.35	8.7E-01	1.0E+00	0.78	0.66	0.93	4.5E-03	8.2E-01
4	ROBO4	3	Weighted median	0.84	0.79	0.9	5.2E-08	5.9E-01	0.81	0.76	0.87	7.1E-10	4.4E-01	0.86	0.76	0.98	2.0E-02	6.0E-01	1	0.84	1.2	9.9E-01	1.0E+00	0.78	0.66	0.93	4.9E-03	8.2E-01
4	ROBO4	3	Weighted mode	0.84	0.73	0.96	3.2E-02	5.9E-01	0.8	0.7	0.92	2.1E-02	4.4E-01	0.86	0.65	1.15	1.6E-01	6.0E-01	1	0.66	1.51	9.8E-01	1.0E+00	0.78	0.54	1.13	1.0E-01	8.2E-01
4	ROBO4	3	MR Egger	0.81	0.29	2.27	2.3E-01	5.9E-01	0.76	0.32	1.82	1.5E-01	4.4E-01	0.91	0.22	3.83	5.7E-01	6.0E-01	1.02	0.01	89.81	9.6E-01	1.0E+00	0.81	0.11	5.8	4.0E-01	8.2E-01
5	IL3RA	4	Inverse variance weighted	0.85	0.81	0.9	1.7E-08	4.3E-01	0.82	0.77	0.87	1.8E-09	3.4E-01	0.86	0.77	0.97	1.2E-02	4.4E-01	1.01	0.86	1.19	8.9E-01	4.7E-01	0.83	0.71	0.97	1.7E-02	6.8E-01
5	IL3RA	4	Weighted median	0.86	0.81	0.91	7.2E-08	4.3E-01	0.82	0.77	0.88	5.0E-09	3.4E-01	0.87	0.77	0.98	1.7E-02	4.4E-01	1.01	0.86	1.18	9.3E-01	4.7E-01	0.82	0.7	0.96	1.2E-02	6.8E-01
5	IL3RA	4	Weighted mode	0.85	0.78	0.93	1.0E-02	4.3E-01	0.82	0.74	0.91	8.2E-03	3.4E-01	0.87	0.72	1.06	1.1E-01	4.4E-01	1.01	0.76	1.33	9.5E-01	4.7E-01	0.8	0.62	1.03	6.5E-02	6.8E-01
5	IL3RA	4	MR Egger	0.88	0.74	1.06	9.5E-02	4.3E-01	0.86	0.7	1.05	8.0E-02	3.4E-01	0.92	0.63	1.34	4.3E-01	4.4E-01	0.93	0.55	1.59	6.3E-01	4.7E-01	0.79	0.44	1.42	2.2E-01	6.8E-01
6	HEG1	7	Inverse variance weighted	0.84	0.77	0.91	5.7E-05	4.1E-01	0.8	0.7	0.92	9.9E-04	6.6E-01	0.88	0.75	1.03	1.1E-01	7.5E-02	0.96	0.79	1.18	7.1E-01	6.1E-01	0.83	0.67	1.03	9.5E-02	2.8E-01
6	HEG1	7	Weighted median	0.83	0.76	0.89	7.7E-07	4.1E-01	0.78	0.72	0.85	3.6E-09	6.6E-01	0.83	0.71	0.98	2.8E-02	7.5E-02	1	0.81	1.25	9.8E-01	6.1E-01	0.8	0.65	0.99	4.1E-02	2.8E-01
6	HEG1	7	Weighted mode	0.8	0.72	0.88	1.4E-03	4.1E-01	0.76	0.68	0.85	1.1E-03	6.6E-01	0.83	0.67	1.02	6.7E-02	7.5E-02	1.01	0.76	1.34	9.5E-01	6.1E-01	0.74	0.56	0.97	3.4E-02	2.8E-01
6	HEG1	7	MR Egger	0.81	0.69	0.95	2.0E-02	4.1E-01	0.78	0.59	1.01	5.8E-02	6.6E-01	0.74	0.56	0.97	3.5E-02	7.5E-02	1.02	0.68	1.53	8.9E-01	6.1E-01	0.73	0.49	1.08	9.4E-02	2.8E-01
7	SELE	8	Inverse variance weighted	0.92	0.87	0.96	6.9E-04	1.3E-02	0.9	0.85	0.94	4.8E-05	2.4E-02	0.92	0.86	0.99	2.1E-02	4.9E-01	1.01	0.92	1.12	8.2E-01	3.6E-01	0.9	0.81	1	5.2E-02	7.5E-02
7	SELE	8	Weighted median	0.91	0.88	0.94	4.0E-08	1.3E-02	0.89	0.85	0.92	4.6E-09	2.4E-02	0.92	0.86	0.99	2.5E-02	4.9E-01	1	0.91	1.11	9.4E-01	3.6E-01	0.88	0.8	0.97	9.5E-03	7.5E-02
7	SELE	8	Weighted mode	0.91	0.87	0.94	4.9E-04	1.3E-02	0.89	0.85	0.93	3.4E-04	2.4E-02	0.92	0.84	1.01	6.6E-02	4.9E-01	1	0.88	1.13	9.9E-01	3.6E-01	0.87	0.78	0.98	2.7E-02	7.5E-02
7	SELE	8	MR Egger	0.88	0.84	0.93	8.0E-04	1.3E-02	0.86	0.81	0.91	6.6E-04	2.4E-02	0.9	0.81	1.01	6.2E-02	4.9E-01	0.97	0.84	1.14	7.0E-01	3.6E-01	0.84	0.72	0.97	2.4E-02	7.5E-02
8	CD34	4	Inverse variance weighted	0.83	0.76	0.91	1.0E-04	3.4E-01	0.79	0.71	0.89	4.2E-05	2.6E-01	0.84	0.72	0.97	1.5E-02	9.1E-01	1.03	0.84	1.26	8.0E-01	5.1E-01	0.79	0.64	0.98	3.4E-02	7.5E-01
8	CD34	4	Weighted median	0.83	0.78	0.89	1.2E-07	3.4E-01	0.79	0.73	0.86	1.2E-08	2.6E-01	0.84	0.72	0.98	2.3E-02	9.1E-01	1.01	0.82	1.25	9.2E-01	5.1E-01	0.77	0.63	0.94	8.9E-03	7.5E-01
8	CD34	4	Weighted mode	0.81	0.72	0.91	1.1E-02	3.4E-01	0.77	0.68	0.88	7.2E-03	2.6E-01	0.84	0.65	1.08	1.2E-01	9.1E-01	1	0.7	1.43	9.7E-01	5.1E-01	0.75	0.54	1.06	7.5E-02	7.5E-01
8	CD34	4	MR Egger	0.73	0.46	1.18	1.1E-01	3.4E-01	0.67	0.4	1.12	7.8E-02	2.6E-01	0.82	0.36	1.84	4.0E-01	9.1E-01	0.85	0.28	2.6	5.9E-01	5.1E-01	0.71	0.17	2.97	4.1E-01	7.5E-01
9	EPHA4	5	Inverse variance weighted	0.87	0.83	0.92	3.7E-07	2.0E-01	0.85	0.77	0.95	2.4E-03	8.8E-02	0.85	0.75	0.96	7.8E-03	4.9E-01	1	0.86	1.17	9.8E-01	8.4E-01	0.84	0.73	0.98	2.3E-02	3.1E-01
9	EPHA4	5	Weighted median	0.87	0.82	0.91	1.5E-07	2.0E-01	0.84	0.79	0.89	4.9E-08	8.8E-02	0.87	0.77	0.97	1.5E-02	4.9E-01	1	0.85	1.17	9.6E-01	8.4E-01	0.84	0.72	0.97	2.1E-02	3.1E-01
9	EPHA4	5	Weighted mode	0.85	0.79	0.92	4.6E-03	2.0E-01	0.82	0.76	0.9	3.2E-03	8.8E-02	0.88	0.75	1.02	7.3E-02	4.9E-01	1	0.79	1.25	9.9E-01	8.4E-01	0.81	0.65	1	5.2E-02	3.1E-01
9	EPHA4	5	MR Egger	0.84	0.75	0.93	1.4E-02	2.0E-01	0.79	0.68	0.91	1.5E-02	8.8E-02	0.89	0.67	1.17	2.6E-01	4.9E-01	0.99	0.71	1.37	9.1E-01	8.4E-01	0.78	0.57	1.07	8.8E-02	3.1E-01
10	LIFR	10	Inverse variance weighted	0.85	0.78	0.92	7.4E-05	2.2E-01	0.81	0.74	0.89	9.2E-06	3.1E-01	0.85	0.74	0.97	1.8E-02	4.2E-01	1.01	0.83	1.22	9.3E-01	6.8E-01	0.89	0.7	1.14	3.6E-01	2.1E-01
10	LIFR	10	Weighted median	0.81	0.75	0.88	3.5E-07	2.2E-01	0.8	0.73	0.88	2.1E-06	3.1E-01	0.83	0.71	0.97	2.3E-02	4.2E-01	1	0.81	1.24	9.8E-01	6.8E-01	0.74	0.59	0.94	1.3E-02	2.1E-01
10	LIFR	10	Weighted mode	0.8	0.73	0.86	1.6E-04	2.2E-01	0.75	0.67	0.84	2.2E-04	3.1E-01	0.84	0.69	1.01	5.8E-02	4.2E-01	1	0.77	1.29	9.7E-01	6.8E-01	0.76	0.6	0.97	3.0E-02	2.1E-01
10	LIFR	10	MR Egger	0.81	0.71	0.92	4.3E-03	2.2E-01	0.78	0.67	0.9	3.7E-03	3.1E-01	0.8	0.64	1	4.9E-02	4.2E-01	0.97	0.71	1.32	8.3E-01	6.8E-01	0.77	0.53	1.12	1.4E-01	2.1E-01
11	EPHB4	10	Inverse variance weighted	0.84	0.78	0.9	4.2E-07	1.9E-01	0.82	0.76	0.89	9.8E-07	9.9E-02	0.84	0.72	0.97	1.8E-02	4.9E-01	1.02	0.83	1.26	8.4E-01	9.1E-01	0.78	0.64	0.95	1.3E-02	7.1E-01
11	EPHB4	10	Weighted median	0.83	0.77	0.9	1.1E-05	1.9E-01	0.77	0.7	0.85	3.8E-07	9.9E-02	0.84	0.7	1	4.5E-02	4.9E-01	1.01	0.8	1.29	9.2E-01	9.1E-01	0.76	0.6	0.96	2.0E-02	7.1E-01
11	EPHB4	10	Weighted mode	0.8	0.73	0.88	5.9E-04	1.9E-01	0.76	0.67	0.85	5.6E-04	9.9E-02	0.83	0.68	1.02	7.3E-02	4.9E-01	1.01	0.77	1.33	9.2E-01	9.1E-01	0.74	0.57	0.98	3.6E-02	7.1E-01
11	EPHB4	10	MR Egger	0.78	0.68	0.9	3.2E-03	1.9E-01	0.74	0.63	0.86	2.1E-03	9.9E-02	0.9	0.67	1.21	4.3E-01	4.9E-01	1	0.67	1.51	9.9E-01	9.1E-01	0.74	0.49	1.09	1.1E-01	7.1E-01
12	FSHB	4	Inverse variance weighted	1.34	1.1	1.63	3.9E-03	7.9E-01	1.26	1	1.58	4.8E-02	5.0E-01	**2.41**	**1.56**	**3.71**	**7.0E-05**	6.0E-01	1.89	1.03	3.45	3.9E-02	7.9E-01	0.84	0.47	1.51	5.7E-01	9.6E-01
12	FSHB	4	Weighted median	1.27	1.02	1.59	3.2E-02	7.9E-01	1.24	0.97	1.59	9.1E-02	5.0E-01	**2.61**	**1.63**	**4.2**	**7.3E-05**	6.0E-01	1.88	0.98	3.58	5.6E-02	7.9E-01	0.8	0.42	1.52	5.0E-01	9.6E-01
12	FSHB	4	Weighted mode	1.27	0.86	1.9	1.5E-01	7.9E-01	1.21	0.79	1.85	2.5E-01	5.0E-01	**2.64**	**1.1**	**6.33**	**3.9E-02**	6.0E-01	1.67	0.5	5.55	2.7E-01	7.9E-01	0.78	0.23	2.67	5.7E-01	9.6E-01
12	FSHB	4	MR Egger	1.26	0.51	3.14	3.8E-01	7.9E-01	1.06	0.37	3.01	8.5E-01	5.0E-01	3.1	0.42	22.97	1.4E-01	6.0E-01	1.59	0.1	25.32	5.5E-01	7.9E-01	0.82	0.06	12	7.8E-01	9.6E-01
13	F3	6	Inverse variance weighted	0.78	0.67	0.92	2.5E-03	9.6E-02	0.74	0.62	0.89	1.6E-03	7.0E-02	0.9	0.71	1.14	4.0E-01	9.0E-02	1.03	0.75	1.41	8.4E-01	9.1E-01	**0.61**	**0.47**	**0.79**	**1.5E-04**	3.2E-01
13	F3	6	Weighted median	0.76	0.68	0.83	2.3E-08	9.6E-02	0.71	0.62	0.8	1.6E-07	7.0E-02	0.85	0.67	1.09	2.0E-01	9.0E-02	0.95	0.71	1.29	7.6E-01	9.1E-01	**0.58**	**0.43**	**0.78**	**3.2E-04**	3.2E-01
13	F3	6	Weighted mode	0.75	0.67	0.85	1.6E-03	9.6E-02	0.7	0.61	0.81	1.6E-03	7.0E-02	0.77	0.57	1.04	7.3E-02	9.0E-02	0.95	0.64	1.4	7.3E-01	9.1E-01	**0.57**	**0.39**	**0.83**	**1.2E-02**	3.2E-01
13	F3	6	MR Egger	0.65	0.48	0.87	1.5E-02	9.6E-02	0.59	0.43	0.81	1.0E-02	7.0E-02	0.67	0.43	1.06	7.1E-02	9.0E-02	1	0.43	2.35	1.0E+00	9.1E-01	**0.5**	**0.27**	**0.93**	**3.5E-02**	3.2E-01
14	IGF1R	7	Inverse variance weighted	0.86	0.78	0.95	3.3E-03	2.5E-01	0.83	0.74	0.93	2.2E-03	1.2E-01	0.87	0.76	0.99	3.3E-02	9.3E-01	1.11	0.85	1.45	4.5E-01	8.5E-01	0.79	0.65	0.95	1.3E-02	6.2E-01
14	IGF1R	7	Weighted median	0.83	0.77	0.88	3.0E-08	2.5E-01	0.8	0.74	0.87	5.6E-08	1.2E-01	0.86	0.75	0.98	2.7E-02	9.3E-01	1.09	0.89	1.33	4.2E-01	8.5E-01	0.77	0.64	0.94	1.0E-02	6.2E-01
14	IGF1R	7	Weighted mode	0.82	0.75	0.89	1.3E-03	2.5E-01	0.79	0.71	0.86	8.5E-04	1.2E-01	0.85	0.71	1.03	8.8E-02	9.3E-01	1.04	0.8	1.35	7.5E-01	8.5E-01	0.77	0.59	0.99	4.3E-02	6.2E-01
14	IGF1R	7	MR Egger	0.81	0.68	0.96	2.7E-02	2.5E-01	0.75	0.62	0.91	1.2E-02	1.2E-01	0.87	0.68	1.12	2.2E-01	9.3E-01	1.07	0.62	1.85	7.5E-01	8.5E-01	0.75	0.51	1.09	1.0E-01	6.2E-01
15	CLSTN3	6	Inverse variance weighted	0.74	0.64	0.86	1.2E-04	3.1E-01	0.7	0.57	0.88	1.6E-03	3.0E-01	0.78	0.59	1.03	7.7E-02	4.1E-01	0.93	0.66	1.32	6.9E-01	9.5E-01	0.63	0.45	0.88	7.5E-03	2.9E-01
15	CLSTN3	6	Weighted median	0.72	0.63	0.82	1.1E-06	3.1E-01	0.66	0.56	0.78	5.7E-07	3.0E-01	0.75	0.57	0.99	3.9E-02	4.1E-01	0.94	0.64	1.4	7.7E-01	9.5E-01	0.58	0.4	0.84	4.1E-03	2.9E-01
15	CLSTN3	6	Weighted mode	0.67	0.56	0.8	2.3E-03	3.1E-01	0.6	0.49	0.75	1.7E-03	3.0E-01	0.72	0.49	1.07	8.9E-02	4.1E-01	1	0.57	1.74	9.9E-01	9.5E-01	0.57	0.34	0.98	4.3E-02	2.9E-01
15	CLSTN3	6	MR Egger	0.65	0.44	0.96	3.6E-02	3.1E-01	0.58	0.33	1.01	5.1E-02	3.0E-01	0.96	0.45	2.06	9.0E-01	4.1E-01	0.95	0.37	2.41	8.9E-01	9.5E-01	0.45	0.18	1.12	7.1E-02	2.9E-01
16	ISLR2	7	Inverse variance weighted	0.9	0.83	0.98	1.3E-02	7.0E-02	0.87	0.8	0.95	1.3E-03	5.2E-02	0.93	0.8	1.08	3.3E-01	4.8E-01	1.02	0.87	1.19	8.3E-01	5.2E-01	0.9	0.76	1.07	2.4E-01	2.4E-01
16	ISLR2	7	Weighted median	0.89	0.85	0.94	8.5E-06	7.0E-02	0.87	0.82	0.92	7.7E-07	5.2E-02	0.9	0.81	1	4.7E-02	4.8E-01	1.01	0.87	1.17	9.1E-01	5.2E-01	0.87	0.75	1	5.7E-02	2.4E-01
16	ISLR2	7	Weighted mode	0.86	0.81	0.92	1.6E-03	7.0E-02	0.83	0.77	0.9	8.9E-04	5.2E-02	0.89	0.77	1.02	7.4E-02	4.8E-01	1.01	0.84	1.21	8.9E-01	5.2E-01	0.82	0.68	0.99	4.0E-02	2.4E-01
16	ISLR2	7	MR Egger	0.84	0.74	0.94	1.1E-02	7.0E-02	0.8	0.72	0.9	4.4E-03	5.2E-02	0.87	0.65	1.16	2.8E-01	4.8E-01	0.96	0.7	1.31	7.4E-01	5.2E-01	0.81	0.59	1.09	1.3E-01	2.4E-01
17	LGALS8	5	Inverse variance weighted	0.88	0.8	0.96	4.9E-03	4.8E-01	0.85	0.75	0.96	6.9E-03	5.0E-01	0.89	0.76	1.04	1.3E-01	1.8E-01	0.99	0.84	1.17	9.4E-01	9.3E-01	0.85	0.72	1	5.5E-02	5.4E-01
17	LGALS8	5	Weighted median	0.83	0.77	0.89	8.3E-07	4.8E-01	0.81	0.73	0.88	3.9E-06	5.0E-01	0.87	0.77	0.98	2.6E-02	1.8E-01	0.99	0.84	1.18	9.5E-01	9.3E-01	0.84	0.71	1	5.7E-02	5.4E-01
17	LGALS8	5	Weighted mode	0.83	0.75	0.93	1.1E-02	4.8E-01	0.79	0.69	0.9	8.1E-03	5.0E-01	0.87	0.72	1.05	1.0E-01	1.8E-01	1	0.78	1.28	9.7E-01	9.3E-01	0.81	0.61	1.06	9.7E-02	5.4E-01
17	LGALS8	5	MR Egger	0.84	0.67	1.06	9.4E-02	4.8E-01	0.81	0.6	1.08	1.0E-01	5.0E-01	0.79	0.59	1.07	8.8E-02	1.8E-01	0.99	0.67	1.46	9.1E-01	9.3E-01	0.79	0.51	1.22	1.8E-01	5.4E-01
18	PODXL	6	Inverse variance weighted	0.9	0.82	0.99	3.8E-02	5.5E-01	0.89	0.78	1	5.4E-02	3.9E-01	0.89	0.8	1	5.2E-02	5.5E-01	0.99	0.85	1.14	8.7E-01	5.7E-01	0.83	0.69	1.01	6.1E-02	5.9E-01
18	PODXL	6	Weighted median	0.86	0.8	0.92	1.9E-05	5.5E-01	0.86	0.79	0.93	4.4E-04	3.9E-01	0.87	0.77	0.98	1.8E-02	5.5E-01	1	0.85	1.17	9.9E-01	5.7E-01	0.82	0.7	0.95	1.0E-02	5.9E-01
18	PODXL	6	Weighted mode	0.84	0.77	0.92	3.3E-03	5.5E-01	0.81	0.73	0.89	2.2E-03	3.9E-01	0.86	0.72	1.02	7.8E-02	5.5E-01	1	0.8	1.24	9.8E-01	5.7E-01	0.82	0.67	1.01	5.7E-02	5.9E-01
18	PODXL	6	MR Egger	0.87	0.68	1.1	1.6E-01	5.5E-01	0.82	0.61	1.09	1.3E-01	3.9E-01	0.94	0.71	1.25	5.9E-01	5.5E-01	1.05	0.75	1.48	7.1E-01	5.7E-01	0.9	0.56	1.46	5.9E-01	5.9E-01
19	MET	17	Inverse variance weighted	0.9	0.82	1	4.9E-02	6.4E-03	0.87	0.77	0.97	1.1E-02	1.8E-02	1.05	0.86	1.28	6.6E-01	1.1E-01	1.05	0.85	1.29	6.7E-01	3.3E-01	0.91	0.75	1.1	3.3E-01	9.8E-03
19	MET	17	Weighted median	0.85	0.77	0.95	3.1E-03	6.4E-03	0.81	0.72	0.91	3.0E-04	1.8E-02	0.82	0.67	1	5.2E-02	1.1E-01	1.03	0.78	1.36	8.3E-01	3.3E-01	0.82	0.64	1.07	1.4E-01	9.8E-03
19	MET	17	Weighted mode	0.77	0.7	0.86	9.8E-05	6.4E-03	0.73	0.65	0.82	4.7E-05	1.8E-02	0.85	0.68	1.04	1.1E-01	1.1E-01	1.04	0.78	1.4	7.6E-01	3.3E-01	0.73	0.55	0.98	3.9E-02	9.8E-03
19	MET	17	MR Egger	0.76	0.65	0.88	1.1E-03	6.4E-03	0.73	0.61	0.86	1.3E-03	1.8E-02	0.84	0.59	1.19	3.0E-01	1.1E-01	0.91	0.62	1.32	5.9E-01	3.3E-01	0.61	0.43	0.87	9.7E-03	9.8E-03
20	IL22RA1	2	Inverse variance weighted	0.95	0.84	1.07	4.1E-01	NA	1.04	0.94	1.15	4.6E-01	NA	0.69	0.57	0.84	1.4E-04	NA	0.87	0.46	1.65	6.7E-01	NA	1	0.64	1.57	9.9E-01	NA
21	NELL2	11	Inverse variance weighted	0.82	0.68	0.99	3.5E-02	6.1E-02	0.8	0.62	1.02	7.3E-02	2.6E-02	0.83	0.65	1.06	1.3E-01	1.5E-01	0.94	0.66	1.34	7.4E-01	3.2E-01	0.83	0.55	1.25	3.7E-01	2.6E-01
21	NELL2	11	Weighted median	0.73	0.6	0.9	2.8E-03	6.1E-02	0.75	0.58	0.96	2.5E-02	2.6E-02	0.69	0.5	0.94	1.9E-02	1.5E-01	0.97	0.62	1.52	9.1E-01	3.2E-01	0.52	0.33	0.82	5.5E-03	2.6E-01
21	NELL2	11	Weighted mode	0.62	0.5	0.76	3.5E-04	6.1E-02	0.55	0.44	0.69	1.6E-04	2.6E-02	0.68	0.45	1.01	5.5E-02	1.5E-01	0.97	0.57	1.65	9.0E-01	3.2E-01	0.55	0.32	0.94	3.3E-02	2.6E-01
21	NELL2	11	MR Egger	0.6	0.41	0.87	1.3E-02	6.1E-02	0.5	0.32	0.79	7.4E-03	2.6E-02	0.59	0.33	1.04	6.4E-02	1.5E-01	1.31	0.57	3	4.8E-01	3.2E-01	0.54	0.21	1.37	1.7E-01	2.6E-01
22	CDSN	16	Inverse variance weighted	0.99	0.95	1.04	7.3E-01	5.6E-01	1.02	0.96	1.09	5.1E-01	7.4E-01	0.98	0.9	1.06	6.4E-01	8.8E-01	**0.8**	**0.72**	**0.9**	**1.6E-04**	4.8E-01	1.08	0.97	1.21	1.8E-01	3.7E-01
22	CDSN	16	Weighted median	0.99	0.95	1.03	6.4E-01	5.6E-01	1.04	0.99	1.08	1.1E-01	7.4E-01	0.98	0.9	1.07	6.1E-01	8.8E-01	**0.82**	**0.73**	**0.93**	**1.4E-03**	4.8E-01	1.06	0.94	1.2	3.1E-01	3.7E-01
22	CDSN	16	Weighted mode	0.99	0.95	1.04	7.5E-01	5.6E-01	1.03	0.98	1.08	2.2E-01	7.4E-01	0.98	0.89	1.07	6.4E-01	8.8E-01	**0.82**	**0.72**	**0.93**	**4.8E-03**	4.8E-01	1.06	0.93	1.22	3.3E-01	3.7E-01
22	CDSN	16	MR Egger	0.98	0.92	1.05	5.4E-01	5.6E-01	1.01	0.92	1.11	7.7E-01	7.4E-01	0.99	0.88	1.1	7.8E-01	8.8E-01	**0.83**	**0.71**	**0.97**	**2.3E-02**	4.8E-01	1.04	0.89	1.21	6.1E-01	3.7E-01
23	FAM171B	3	Inverse variance weighted	1.06	0.95	1.18	3.1E-01	3.1E-01	1.03	0.93	1.15	5.3E-01	3.4E-01	1.01	0.81	1.26	9.1E-01	4.3E-01	1.57	1.24	1.99	2.1E-04	8.4E-01	0.93	0.63	1.36	7.0E-01	4.0E-01
23	FAM171B	3	Weighted median	1.05	0.97	1.13	2.7E-01	3.1E-01	1.03	0.94	1.12	5.8E-01	3.4E-01	0.99	0.84	1.17	9.3E-01	4.3E-01	1.59	1.25	2.02	1.7E-04	8.4E-01	0.94	0.74	1.19	6.2E-01	4.0E-01
23	FAM171B	3	Weighted mode	1.05	0.87	1.25	4.0E-01	3.1E-01	1.02	0.83	1.26	6.9E-01	3.4E-01	0.99	0.69	1.43	9.4E-01	4.3E-01	1.59	0.91	2.78	7.0E-02	8.4E-01	0.98	0.57	1.68	9.0E-01	4.0E-01
23	FAM171B	3	MR Egger	0.97	0.43	2.15	6.8E-01	3.1E-01	0.94	0.37	2.37	5.5E-01	3.4E-01	0.86	0.11	6.53	5.3E-01	4.3E-01	1.63	0.14	19.03	2.4E-01	8.4E-01	1.23	0.04	34.02	5.8E-01	4.0E-01
24	LY9	12	Inverse variance weighted	0.99	0.88	1.11	8.1E-01	6.5E-01	1.02	0.88	1.18	7.9E-01	6.5E-01	0.92	0.8	1.05	2.1E-01	2.8E-01	0.76	0.65	0.89	6.4E-04	3.1E-01	1.07	0.91	1.24	4.2E-01	2.0E-01
24	LY9	12	Weighted median	1	0.94	1.06	9.6E-01	6.5E-01	1.03	0.97	1.1	2.9E-01	6.5E-01	0.94	0.84	1.06	3.4E-01	2.8E-01	0.73	0.62	0.87	2.6E-04	3.1E-01	1.03	0.88	1.21	7.2E-01	2.0E-01
24	LY9	12	Weighted mode	1	0.94	1.06	8.7E-01	6.5E-01	1.04	0.97	1.11	2.8E-01	6.5E-01	0.95	0.84	1.08	4.1E-01	2.8E-01	0.73	0.61	0.88	3.6E-03	3.1E-01	1.02	0.85	1.23	7.8E-01	2.0E-01
24	LY9	12	MR Egger	1.01	0.85	1.19	9.4E-01	6.5E-01	1.05	0.85	1.29	6.4E-01	6.5E-01	0.97	0.81	1.17	7.2E-01	2.8E-01	0.72	0.58	0.89	6.8E-03	3.1E-01	0.99	0.8	1.22	9.1E-01	2.0E-01
25	PSRC1	3	Inverse variance weighted	0.77	0.66	0.88	2.7E-04	7.0E-01	0.74	0.63	0.87	3.4E-04	7.1E-01	0.85	0.56	1.29	4.4E-01	6.7E-01	1.02	0.67	1.56	9.3E-01	9.2E-01	1.01	0.67	1.53	9.6E-01	9.8E-01
25	PSRC1	3	Weighted median	0.77	0.66	0.9	8.4E-04	7.0E-01	0.74	0.62	0.88	8.0E-04	7.1E-01	0.8	0.57	1.12	1.9E-01	6.7E-01	1.01	0.67	1.53	9.6E-01	9.2E-01	1.01	0.65	1.57	9.5E-01	9.8E-01
25	PSRC1	3	Weighted mode	0.78	0.56	1.09	8.7E-02	7.0E-01	0.76	0.51	1.13	9.6E-02	7.1E-01	0.79	0.35	1.81	3.5E-01	6.7E-01	1.01	0.36	2.88	9.6E-01	9.2E-01	1.01	0.35	2.88	9.8E-01	9.8E-01
25	PSRC1	3	MR Egger	0.79	0.24	2.56	2.4E-01	7.0E-01	0.76	0.2	2.98	2.4E-01	7.1E-01	0.75	0.01	51.05	5.5E-01	6.7E-01	1	0.03	31.79	1.0E+00	9.2E-01	1.02	0.03	30.91	9.6E-01	9.8E-01
26	SUMF2	11	Inverse variance weighted	0.97	0.91	1.03	2.9E-01	6.8E-01	1	0.93	1.07	9.9E-01	9.5E-01	0.95	0.83	1.08	4.0E-01	9.8E-01	0.75	0.64	0.89	6.5E-04	1.3E-01	1.01	0.8	1.27	9.4E-01	6.3E-01
26	SUMF2	11	Weighted median	0.95	0.89	1.02	1.4E-01	6.8E-01	0.99	0.91	1.07	7.2E-01	9.5E-01	0.95	0.82	1.1	4.7E-01	9.8E-01	0.72	0.6	0.86	3.4E-04	1.3E-01	1.04	0.84	1.29	7.1E-01	6.3E-01
26	SUMF2	11	Weighted mode	0.95	0.88	1.03	1.8E-01	6.8E-01	0.98	0.9	1.08	6.8E-01	9.5E-01	0.95	0.81	1.11	4.7E-01	9.8E-01	0.72	0.58	0.89	6.6E-03	1.3E-01	1.04	0.83	1.31	6.8E-01	6.3E-01
26	SUMF2	11	MR Egger	0.96	0.89	1.04	2.9E-01	6.8E-01	1	0.91	1.1	9.6E-01	9.5E-01	0.95	0.79	1.13	5.0E-01	9.8E-01	0.69	0.55	0.86	4.4E-03	1.3E-01	1.05	0.76	1.46	7.4E-01	6.3E-01
27	DLK1	7	Inverse variance weighted	1.09	1.02	1.17	1.4E-02	9.5E-01	1.07	0.97	1.17	1.6E-01	8.4E-01	1.16	1	1.35	4.3E-02	3.5E-01	1.2	0.96	1.51	1.2E-01	7.5E-02	0.93	0.77	1.12	4.5E-01	2.9E-01
27	DLK1	7	Weighted median	1.11	1.05	1.17	3.9E-04	9.5E-01	1.09	1.02	1.16	1.3E-02	8.4E-01	1.16	1.02	1.3	1.8E-02	3.5E-01	1.15	0.97	1.36	1.1E-01	7.5E-02	0.96	0.81	1.13	6.2E-01	2.9E-01
27	DLK1	7	Weighted mode	1.11	1.04	1.19	1.0E-02	9.5E-01	1.09	1.01	1.18	3.7E-02	8.4E-01	1.15	0.99	1.33	6.2E-02	3.5E-01	1.15	0.92	1.44	1.9E-01	7.5E-02	0.98	0.79	1.22	8.5E-01	2.9E-01
27	DLK1	7	MR Egger	1.1	0.96	1.25	1.3E-01	9.5E-01	1.08	0.91	1.28	3.1E-01	8.4E-01	1.09	0.85	1.4	3.9E-01	3.5E-01	1.02	0.76	1.37	8.6E-01	7.5E-02	1.02	0.75	1.38	8.9E-01	2.9E-01
28	DNAJB6	3	Inverse variance weighted	0.91	0.75	1.11	3.5E-01	3.0E-01	0.97	0.78	1.2	7.5E-01	3.0E-01	0.63	0.42	0.95	2.9E-02	5.0E-01	0.8	0.54	1.19	2.7E-01	6.7E-01	0.85	0.57	1.26	4.2E-01	6.7E-01
28	DNAJB6	3	Weighted median	0.89	0.78	1.03	1.1E-01	3.0E-01	0.96	0.81	1.13	6.2E-01	3.0E-01	0.59	0.43	0.79	5.0E-04	5.0E-01	0.81	0.54	1.21	3.1E-01	6.7E-01	0.9	0.59	1.37	6.2E-01	6.7E-01
28	DNAJB6	3	Weighted mode	0.87	0.64	1.19	2.0E-01	3.0E-01	0.91	0.62	1.32	3.8E-01	3.0E-01	0.59	0.29	1.2	8.5E-02	5.0E-01	0.83	0.31	2.26	5.2E-01	6.7E-01	0.9	0.34	2.36	6.8E-01	6.7E-01
28	DNAJB6	3	MR Egger	0.74	0.15	3.72	2.5E-01	3.0E-01	0.76	0.12	4.94	3.1E-01	3.0E-01	0.45	0	66.75	2.9E-01	5.0E-01	0.97	0.01	128.19	9.4E-01	6.7E-01	1.02	0.01	128.49	9.6E-01	6.7E-01
29	HADH	2	Inverse variance weighted	0.74	0.43	1.3	3.0E-01	NA	0.84	0.39	1.79	6.5E-01	NA	0.9	0.45	1.79	7.6E-01	NA	0.19	0.07	0.48	5.8E-04	NA	0.99	0.29	3.39	9.9E-01	NA
30	CHMP6	4	Inverse variance weighted	1.18	1	1.39	4.6E-02	4.3E-01	1.24	0.91	1.7	1.8E-01	1.7E-01	1.21	0.89	1.65	2.2E-01	9.5E-01	0.86	0.55	1.33	4.9E-01	7.5E-01	0.92	0.6	1.42	7.2E-01	7.4E-01
30	CHMP6	4	Weighted median	1.2	1.03	1.4	2.2E-02	4.3E-01	1.38	1.15	1.66	5.8E-04	1.7E-01	1.21	0.87	1.67	2.6E-01	9.5E-01	0.84	0.53	1.36	4.9E-01	7.5E-01	0.94	0.6	1.49	8.1E-01	7.4E-01
30	CHMP6	4	Weighted mode	1.25	0.95	1.63	8.1E-02	4.3E-01	1.41	1.02	1.94	4.3E-02	1.7E-01	1.2	0.67	2.16	3.9E-01	9.5E-01	0.82	0.37	1.78	4.7E-01	7.5E-01	0.97	0.44	2.1	8.9E-01	7.4E-01
30	CHMP6	4	MR Egger	1.34	0.7	2.53	1.9E-01	4.3E-01	1.74	0.76	3.97	1.0E-01	1.7E-01	1.19	0.36	3.95	5.9E-01	9.5E-01	0.76	0.14	4.16	5.6E-01	7.5E-01	1.04	0.2	5.51	9.2E-01	7.4E-01
31	LPO	13	Inverse variance weighted	1.05	0.99	1.12	1.1E-01	7.7E-01	0.99	0.92	1.07	8.6E-01	3.8E-01	1.11	0.98	1.25	1.0E-01	9.5E-01	1.22	1.03	1.45	2.2E-02	5.6E-01	**1.33**	**1.13**	**1.57**	**6.3E-04**	6.5E-01
31	LPO	13	Weighted median	1.08	0.99	1.17	7.2E-02	7.7E-01	0.99	0.91	1.07	7.4E-01	3.8E-01	1.01	0.84	1.21	9.2E-01	9.5E-01	1.31	1.05	1.64	1.5E-02	5.6E-01	**1.3**	**1.04**	**1.62**	**2.1E-02**	6.5E-01
31	LPO	13	Weighted mode	1.09	0.99	1.2	7.3E-02	7.7E-01	1.01	0.93	1.1	8.1E-01	3.8E-01	0.97	0.75	1.24	7.7E-01	9.5E-01	1.25	0.98	1.61	7.2E-02	5.6E-01	**1.32**	**1.03**	**1.69**	**3.0E-02**	6.5E-01
31	LPO	13	MR Egger	1.07	0.95	1.2	2.5E-01	7.7E-01	1.04	0.9	1.19	5.6E-01	3.8E-01	1.1	0.88	1.38	3.7E-01	9.5E-01	1.3	0.95	1.78	8.8E-02	5.6E-01	1.27	0.95	1.7	9.9E-02	6.5E-01
32	LAIR2	3	Inverse variance weighted	1.07	1.03	1.11	6.9E-04	7.1E-01	1.06	1.01	1.11	1.2E-02	4.8E-01	1.03	0.95	1.12	5.0E-01	5.9E-01	1.22	1.08	1.38	1.2E-03	6.7E-01	1.12	0.99	1.26	6.3E-02	5.9E-01
32	LAIR2	3	Weighted median	1.07	1.03	1.11	8.6E-04	7.1E-01	1.06	1.01	1.11	1.5E-02	4.8E-01	1.03	0.95	1.12	4.8E-01	5.9E-01	1.23	1.09	1.39	7.1E-04	6.7E-01	1.12	1	1.26	5.4E-02	5.9E-01
32	LAIR2	3	Weighted mode	1.07	0.98	1.17	7.8E-02	7.1E-01	1.06	0.96	1.17	1.3E-01	4.8E-01	1.03	0.85	1.25	5.7E-01	5.9E-01	1.23	0.94	1.61	7.9E-02	6.7E-01	1.12	0.86	1.46	2.0E-01	5.9E-01
32	LAIR2	3	MR Egger	1.06	0.79	1.44	2.3E-01	7.1E-01	1.04	0.74	1.48	3.5E-01	4.8E-01	1.05	0.55	2	5.1E-01	5.9E-01	1.26	0.42	3.8	2.3E-01	6.7E-01	1.15	0.47	2.84	3.0E-01	5.9E-01
33	AOC3	11	Inverse variance weighted	1.09	1.03	1.15	3.3E-03	4.7E-01	1.11	1.04	1.18	9.7E-04	4.8E-01	1.04	0.92	1.17	5.6E-01	1.8E-01	1.01	0.83	1.23	9.1E-01	5.2E-01	1.15	0.95	1.38	1.6E-01	5.2E-01
33	AOC3	11	Weighted median	1.08	1.02	1.14	8.2E-03	4.7E-01	1.1	1.03	1.17	3.4E-03	4.8E-01	1.07	0.95	1.21	2.5E-01	1.8E-01	0.98	0.83	1.17	8.4E-01	5.2E-01	1.13	0.96	1.32	1.4E-01	5.2E-01
33	AOC3	11	Weighted mode	1.07	1.01	1.14	3.2E-02	4.7E-01	1.1	1.02	1.19	2.2E-02	4.8E-01	1.07	0.93	1.23	3.3E-01	1.8E-01	0.98	0.81	1.18	8.3E-01	5.2E-01	1.12	0.93	1.36	2.0E-01	5.2E-01
33	AOC3	11	MR Egger	1.07	0.99	1.16	8.1E-02	4.7E-01	1.09	1	1.19	4.5E-02	4.8E-01	1.1	0.94	1.28	2.2E-01	1.8E-01	0.97	0.73	1.28	7.9E-01	5.2E-01	1.1	0.84	1.43	4.6E-01	5.2E-01
34	CLEC14A	8	Inverse variance weighted	0.87	0.76	1	5.4E-02	2.5E-02	0.86	0.72	1.02	7.8E-02	7.6E-03	0.85	0.72	1	5.5E-02	4.6E-01	0.96	0.76	1.21	7.1E-01	7.3E-01	0.82	0.66	1.03	9.3E-02	1.5E-01
34	CLEC14A	8	Weighted median	0.86	0.78	0.96	4.5E-03	2.5E-02	0.83	0.74	0.94	2.6E-03	7.6E-03	0.84	0.7	1	5.3E-02	4.6E-01	1.02	0.78	1.33	8.9E-01	7.3E-01	0.77	0.6	0.98	3.6E-02	1.5E-01
34	CLEC14A	8	Weighted mode	0.78	0.7	0.87	1.1E-03	2.5E-02	0.74	0.65	0.84	9.8E-04	7.6E-03	0.83	0.65	1.06	1.2E-01	4.6E-01	1.02	0.75	1.4	8.8E-01	7.3E-01	0.7	0.51	0.96	3.3E-02	1.5E-01
34	CLEC14A	8	MR Egger	0.7	0.56	0.87	7.0E-03	2.5E-02	0.62	0.5	0.79	2.5E-03	7.6E-03	0.77	0.52	1.13	1.4E-01	4.6E-01	1.03	0.59	1.77	9.1E-01	7.3E-01	0.61	0.36	1.03	6.1E-02	1.5E-01
35	PTPRK	3	Inverse variance weighted	0.74	0.47	1.16	1.9E-01	5.1E-01	0.7	0.38	1.28	2.5E-01	4.8E-01	0.83	0.5	1.38	4.7E-01	6.7E-01	1.02	0.65	1.61	9.3E-01	7.2E-01	0.48	0.31	0.74	1.0E-03	7.4E-01
35	PTPRK	3	Weighted median	0.84	0.68	1.04	1.1E-01	5.1E-01	0.86	0.69	1.09	2.1E-01	4.8E-01	0.79	0.54	1.17	2.4E-01	6.7E-01	1.05	0.65	1.72	8.3E-01	7.2E-01	0.46	0.29	0.74	1.2E-03	7.4E-01
35	PTPRK	3	Weighted mode	0.56	0.34	0.92	3.8E-02	5.1E-01	0.49	0.28	0.86	3.1E-02	4.8E-01	0.65	0.26	1.63	1.8E-01	6.7E-01	1.06	0.31	3.65	8.5E-01	7.2E-01	0.46	0.14	1.47	1.0E-01	7.4E-01
35	PTPRK	3	MR Egger	0.27	0	144994.61	4.3E-01	5.1E-01	0.18	0	4532849.84	4.2E-01	4.8E-01	0.38	0	28167735.32	6.2E-01	6.7E-01	1.65	0	851346.24	7.1E-01	7.2E-01	0.31	0	110571.09	4.5E-01	7.4E-01
36	PGA4	4	Inverse variance weighted	0.85	0.66	1.09	1.9E-01	5.4E-01	0.62	0.47	0.83	1.1E-03	9.8E-01	1.29	0.76	2.2	3.5E-01	9.4E-01	1.76	0.61	5.1	3.0E-01	2.5E-01	1.86	0.89	3.86	9.7E-02	9.5E-01
36	PGA4	4	Weighted median	0.83	0.62	1.11	2.1E-01	5.4E-01	0.63	0.45	0.9	9.9E-03	9.8E-01	1.28	0.68	2.39	4.4E-01	9.4E-01	1.49	0.53	4.18	4.4E-01	2.5E-01	2.07	0.87	4.92	1.0E-01	9.5E-01
36	PGA4	4	Weighted mode	0.82	0.45	1.5	3.8E-01	5.4E-01	0.65	0.34	1.25	1.3E-01	9.8E-01	1.21	0.37	3.95	6.4E-01	9.4E-01	1.18	0.16	8.54	8.1E-01	2.5E-01	2.2	0.33	14.59	2.8E-01	9.5E-01
36	PGA4	4	MR Egger	1.27	0.11	14.71	7.1E-01	5.4E-01	0.61	0.03	10.64	5.3E-01	9.8E-01	1.16	0.01	228.15	9.2E-01	9.4E-01	37.99	0.01	180493.8	2.1E-01	2.5E-01	1.61	0	8934.14	8.3E-01	9.5E-01
37	BST2	9	Inverse variance weighted	0.86	0.72	1.04	1.2E-01	6.7E-01	0.93	0.8	1.08	3.3E-01	8.5E-01	0.64	0.43	0.95	2.9E-02	2.6E-01	0.94	0.55	1.59	8.2E-01	1.7E-01	0.73	0.45	1.18	2.0E-01	2.3E-01
37	BST2	9	Weighted median	0.92	0.78	1.08	3.2E-01	6.7E-01	0.92	0.77	1.1	3.4E-01	8.5E-01	0.54	0.38	0.78	1.1E-03	2.6E-01	1.16	0.71	1.9	5.5E-01	1.7E-01	0.82	0.49	1.37	4.5E-01	2.3E-01
37	BST2	9	Weighted mode	0.89	0.72	1.1	2.5E-01	6.7E-01	0.93	0.75	1.16	4.8E-01	8.5E-01	0.53	0.34	0.83	1.1E-02	2.6E-01	1.36	0.76	2.44	2.6E-01	1.7E-01	1.14	0.62	2.1	6.2E-01	2.3E-01
37	BST2	9	MR Egger	0.93	0.6	1.43	6.9E-01	6.7E-01	0.95	0.67	1.36	7.5E-01	8.5E-01	0.43	0.17	1.06	6.2E-02	2.6E-01	1.67	0.58	4.86	2.9E-01	1.7E-01	1.17	0.43	3.19	7.2E-01	2.3E-01
38	ENG	8	Inverse variance weighted	0.91	0.82	1	5.4E-02	1.6E-01	0.88	0.79	0.97	1.4E-02	1.3E-01	0.94	0.83	1.07	3.4E-01	9.1E-02	1.01	0.86	1.18	9.2E-01	6.0E-01	0.9	0.76	1.06	2.0E-01	2.2E-01
38	ENG	8	Weighted median	0.9	0.82	0.98	1.1E-02	1.6E-01	0.86	0.79	0.94	1.3E-03	1.3E-01	0.92	0.8	1.05	2.1E-01	9.1E-02	0.99	0.83	1.18	9.2E-01	6.0E-01	0.78	0.65	0.93	5.0E-03	2.2E-01
38	ENG	8	Weighted mode	0.83	0.76	0.91	1.8E-03	1.6E-01	0.79	0.71	0.88	1.2E-03	1.3E-01	0.87	0.73	1.02	8.2E-02	9.1E-02	1	0.81	1.22	9.6E-01	6.0E-01	0.78	0.63	0.98	3.6E-02	2.2E-01
38	ENG	8	MR Egger	0.82	0.67	1	4.6E-02	1.6E-01	0.77	0.63	0.95	2.3E-02	1.3E-01	0.79	0.62	1.02	6.1E-02	9.1E-02	0.94	0.66	1.34	7.0E-01	6.0E-01	0.76	0.54	1.08	1.0E-01	2.2E-01
39	SEMA3G	8	Inverse variance weighted	1.02	0.97	1.08	3.9E-01	4.8E-01	1	0.95	1.06	9.2E-01	5.1E-01	1.04	0.94	1.16	4.4E-01	7.4E-01	1.27	1.1	1.47	1.2E-03	9.9E-01	1.01	0.88	1.17	8.7E-01	8.8E-01
39	SEMA3G	8	Weighted median	1.02	0.97	1.07	4.5E-01	4.8E-01	1	0.94	1.07	9.3E-01	5.1E-01	1.03	0.92	1.15	5.9E-01	7.4E-01	1.27	1.08	1.49	3.4E-03	9.9E-01	0.99	0.85	1.16	9.5E-01	8.8E-01
39	SEMA3G	8	Weighted mode	1.02	0.96	1.08	4.5E-01	4.8E-01	1	0.93	1.08	9.1E-01	5.1E-01	1.03	0.9	1.18	6.4E-01	7.4E-01	1.27	1.05	1.54	2.2E-02	9.9E-01	0.99	0.82	1.2	9.5E-01	8.8E-01
39	SEMA3G	8	MR Egger	0.99	0.87	1.13	8.5E-01	4.8E-01	0.97	0.85	1.11	6.1E-01	5.1E-01	1.01	0.77	1.33	9.4E-01	7.4E-01	1.27	0.9	1.79	1.4E-01	9.9E-01	1.03	0.72	1.48	8.4E-01	8.8E-01
40	ATXN3	2	Inverse variance weighted	0.96	0.87	1.05	3.7E-01	NA	1	0.89	1.12	9.9E-01	NA	0.71	0.58	0.87	1.2E-03	NA	1	0.75	1.34	9.9E-01	NA	1.1	0.82	1.47	5.1E-01	NA
41	KRT18	9	Inverse variance weighted	**0.79**	**0.69**	**0.91**	**1.2E-03**	6.2E-01	0.83	0.7	0.98	2.4E-02	1.9E-01	0.8	0.59	1.08	1.5E-01	3.0E-01	0.65	0.42	0.99	4.6E-02	7.5E-01	0.94	0.62	1.43	7.8E-01	3.2E-01
41	KRT18	9	Weighted median	**0.82**	**0.68**	**1**	**4.6E-02**	6.2E-01	0.87	0.69	1.08	2.0E-01	1.9E-01	0.88	0.6	1.3	5.2E-01	3.0E-01	0.61	0.36	1.04	6.9E-02	7.5E-01	0.92	0.54	1.57	7.6E-01	3.2E-01
41	KRT18	9	Weighted mode	0.85	0.62	1.16	2.6E-01	6.2E-01	0.88	0.56	1.37	5.2E-01	1.9E-01	0.91	0.52	1.58	7.0E-01	3.0E-01	0.57	0.24	1.35	1.7E-01	7.5E-01	0.99	0.44	2.23	9.7E-01	3.2E-01
41	KRT18	9	MR Egger	0.73	0.5	1.08	1.0E-01	6.2E-01	0.65	0.41	1.01	5.6E-02	1.9E-01	1.14	0.49	2.64	7.2E-01	3.0E-01	0.75	0.23	2.44	5.8E-01	7.5E-01	1.5	0.48	4.71	4.3E-01	3.2E-01
42	MUC16	3	Inverse variance weighted	1.2	1.04	1.38	1.1E-02	4.8E-01	1.19	1	1.41	4.8E-02	4.2E-01	1.37	1.01	1.86	4.0E-02	6.2E-01	2.01	1.31	3.09	1.4E-03	7.8E-01	1.26	0.83	1.92	2.7E-01	7.4E-01
42	MUC16	3	Weighted median	1.22	1.06	1.41	6.5E-03	4.8E-01	1.22	1.03	1.44	2.5E-02	4.2E-01	1.41	1.03	1.93	3.2E-02	6.2E-01	1.99	1.29	3.07	2.0E-03	7.8E-01	1.25	0.81	1.93	3.1E-01	7.4E-01
42	MUC16	3	Weighted mode	1.23	0.88	1.72	1.2E-01	4.8E-01	1.22	0.82	1.82	1.6E-01	4.2E-01	1.42	0.67	3	1.8E-01	6.2E-01	1.98	0.67	5.84	1.1E-01	7.8E-01	1.4	0.5	3.95	3.0E-01	7.4E-01

42	MUC16	3	MR Egger	1.36	0.22	8.33	2.7E-01	4.8E-01	1.43	0.18	11.54	2.8E-01	4.2E-01	1.64	0.03	83.4	3.5E-01	6.2E-01	1.76	0.01	445.87	4.2E-01	7.8E-01	1.49	0.01	339.1	5.2E-01	7.4E-01
43	B4GAT1	9	Inverse variance weighted	0.93	0.87	1	6.6E-02	2.7E-01	0.97	0.89	1.05	4.6E-01	1.1E-01	0.93	0.79	1.09	3.6E-01	4.1E-01	0.73	0.59	0.91	4.9E-03	5.6E-01	1.05	0.81	1.37	6.8E-01	8.4E-01
43	B4GAT1	9	Weighted median	0.95	0.87	1.04	2.4E-01	2.7E-01	1.03	0.93	1.13	6.2E-01	1.1E-01	0.97	0.81	1.16	7.5E-01	4.1E-01	0.66	0.51	0.85	1.4E-03	5.6E-01	1.07	0.81	1.42	6.4E-01	8.4E-01
43	B4GAT1	9	Weighted mode	0.96	0.87	1.06	3.4E-01	2.7E-01	1.01	0.9	1.13	9.0E-01	1.1E-01	0.94	0.75	1.17	5.4E-01	4.1E-01	0.68	0.51	0.92	1.9E-02	5.6E-01	1.22	0.87	1.7	2.1E-01	8.4E-01
43	B4GAT1	9	MR Egger	0.99	0.85	1.15	9.0E-01	2.7E-01	1.08	0.91	1.28	3.4E-01	1.1E-01	0.84	0.61	1.16	2.5E-01	4.1E-01	0.67	0.43	1.04	6.7E-02	5.6E-01	1.1	0.63	1.92	7.1E-01	8.4E-01
44	VAT1	9	Inverse variance weighted	**1.19**	**1.04**	**1.36**	**1.0E-02**	8.0E-01	**1.21**	**1.08**	**1.37**	**1.7E-03**	6.9E-01	1.08	0.84	1.39	5.5E-01	4.7E-01	1.08	0.78	1.49	6.5E-01	8.9E-01	1.44	1.05	1.97	2.4E-02	6.9E-01
44	VAT1	9	Weighted median	**1.14**	**0.99**	**1.33**	**7.6E-02**	8.0E-01	**1.24**	**1.06**	**1.46**	**8.9E-03**	6.9E-01	0.9	0.66	1.21	4.7E-01	4.7E-01	1.06	0.68	1.67	7.8E-01	8.9E-01	1.49	0.99	2.23	5.4E-02	6.9E-01
44	VAT1	9	Weighted mode	1.11	0.92	1.34	2.4E-01	8.0E-01	1.21	0.97	1.51	7.9E-02	6.9E-01	0.89	0.62	1.3	5.1E-01	4.7E-01	1.06	0.55	2.03	8.4E-01	8.9E-01	1.44	0.84	2.48	1.6E-01	6.9E-01
44	VAT1	9	MR Egger	1.14	0.73	1.78	5.1E-01	8.0E-01	1.29	0.88	1.88	1.6E-01	6.9E-01	0.85	0.38	1.92	6.5E-01	4.7E-01	1.01	0.34	2.99	9.8E-01	8.9E-01	1.21	0.43	3.43	6.8E-01	6.9E-01
45	TGFBR2	8	Inverse variance weighted	0.84	0.65	1.08	1.7E-01	3.8E-02	0.79	0.6	1.03	8.6E-02	5.3E-02	0.89	0.67	1.18	4.2E-01	1.3E-01	1	0.68	1.48	9.9E-01	9.7E-01	0.77	0.53	1.12	1.7E-01	1.2E-01
45	TGFBR2	8	Weighted median	0.95	0.73	1.23	6.8E-01	3.8E-02	0.94	0.7	1.24	6.5E-01	5.3E-02	0.77	0.55	1.1	1.5E-01	1.3E-01	0.98	0.62	1.55	9.4E-01	9.7E-01	0.83	0.51	1.38	4.8E-01	1.2E-01
45	TGFBR2	8	Weighted mode	0.6	0.46	0.79	3.3E-03	3.8E-02	0.54	0.4	0.73	1.8E-03	5.3E-02	0.74	0.47	1.16	1.6E-01	1.3E-01	1.01	0.55	1.87	9.7E-01	9.7E-01	0.48	0.25	0.93	3.3E-02	1.2E-01
45	TGFBR2	8	MR Egger	0.54	0.34	0.86	1.8E-02	3.8E-02	0.5	0.29	0.85	1.9E-02	5.3E-02	0.57	0.29	1.16	1.0E-01	1.3E-01	0.99	0.37	2.65	9.7E-01	9.7E-01	0.41	0.16	1.08	6.5E-02	1.2E-01
46	CDH15	3	Inverse variance weighted	1.04	0.94	1.16	4.5E-01	3.5E-01	1.02	0.93	1.12	6.8E-01	5.5E-01	1.33	1.08	1.62	5.9E-03	3.8E-01	1.15	0.86	1.54	3.4E-01	3.4E-01	0.97	0.76	1.25	8.2E-01	6.0E-01
46	CDH15	3	Weighted median	1.05	0.96	1.14	2.8E-01	3.5E-01	1.03	0.94	1.14	5.2E-01	5.5E-01	1.34	1.12	1.61	1.8E-03	3.8E-01	1.16	0.91	1.48	2.4E-01	3.4E-01	0.96	0.75	1.22	7.2E-01	6.0E-01
46	CDH15	3	Weighted mode	1.06	0.89	1.26	3.1E-01	3.5E-01	1.03	0.83	1.28	5.9E-01	5.5E-01	1.36	0.9	2.05	8.3E-02	3.8E-01	1.21	0.68	2.14	2.9E-01	3.4E-01	0.95	0.53	1.69	7.3E-01	6.0E-01
46	CDH15	3	MR Egger	1.1	0.54	2.25	3.3E-01	3.5E-01	1.06	0.46	2.42	5.4E-01	5.5E-01	1.49	0.33	6.76	1.9E-01	3.8E-01	1.39	0.17	11.58	3.0E-01	3.4E-01	0.9	0.11	7.56	6.4E-01	6.0E-01
47	ATRN	4	Inverse variance weighted	0.94	0.9	0.98	5.0E-03	3.8E-01	0.95	0.9	1	3.6E-02	9.3E-01	0.95	0.84	1.08	4.5E-01	6.7E-01	0.91	0.74	1.11	3.5E-01	2.9E-01	0.91	0.8	1.03	1.3E-01	6.2E-01
47	ATRN	4	Weighted median	0.94	0.9	0.98	2.1E-03	3.8E-01	0.95	0.9	1	3.4E-02	9.3E-01	0.95	0.87	1.04	3.0E-01	6.7E-01	0.9	0.78	1.02	9.9E-02	2.9E-01	0.9	0.79	1.02	1.1E-01	6.2E-01
47	ATRN	4	Weighted mode	0.93	0.87	1	5.7E-02	3.8E-01	0.95	0.87	1.03	1.3E-01	9.3E-01	0.95	0.81	1.12	4.2E-01	6.7E-01	0.9	0.72	1.12	2.1E-01	2.9E-01	0.9	0.73	1.11	2.1E-01	6.2E-01
47	ATRN	4	MR Egger	0.93	0.83	1.03	9.2E-02	3.8E-01	0.95	0.81	1.1	2.5E-01	9.3E-01	0.94	0.66	1.32	5.0E-01	6.7E-01	0.85	0.55	1.32	2.5E-01	2.9E-01	0.89	0.65	1.21	2.5E-01	6.2E-01
48	TREM2	8	Inverse variance weighted	0.98	0.91	1.05	5.4E-01	6.4E-01	1	0.92	1.09	9.5E-01	8.2E-01	1.02	0.91	1.15	7.3E-01	6.0E-01	0.87	0.74	1.02	7.9E-02	9.9E-01	0.83	0.69	0.99	4.1E-02	3.2E-01
48	TREM2	8	Weighted median	0.99	0.92	1.06	8.0E-01	6.4E-01	1	0.92	1.09	9.9E-01	8.2E-01	1.03	0.9	1.18	6.4E-01	6.0E-01	0.87	0.73	1.04	1.3E-01	9.9E-01	0.76	0.63	0.9	2.2E-03	3.2E-01
48	TREM2	8	Weighted mode	1.01	0.94	1.1	6.9E-01	6.4E-01	1.03	0.93	1.13	5.5E-01	8.2E-01	1.03	0.88	1.2	6.5E-01	6.0E-01	0.88	0.71	1.08	1.8E-01	9.9E-01	0.8	0.65	0.99	4.5E-02	3.2E-01
48	TREM2	8	MR Egger	0.99	0.88	1.11	8.6E-01	6.4E-01	1.01	0.87	1.16	9.3E-01	8.2E-01	1.05	0.87	1.25	5.7E-01	6.0E-01	0.87	0.68	1.1	2.0E-01	9.9E-01	0.77	0.59	1.02	6.1E-02	3.2E-01
49	NCR1	17	Inverse variance weighted	0.95	0.85	1.05	3.3E-01	5.0E-01	0.99	0.87	1.13	8.9E-01	4.8E-01	0.9	0.73	1.09	2.8E-01	3.2E-01	0.78	0.63	0.97	2.6E-02	4.8E-02	1.03	0.83	1.29	7.8E-01	8.2E-01
49	NCR1	17	Weighted median	0.99	0.89	1.1	8.5E-01	5.0E-01	1.04	0.93	1.17	4.7E-01	4.8E-01	0.92	0.74	1.14	4.3E-01	3.2E-01	0.65	0.49	0.85	2.3E-03	4.8E-02	1.05	0.78	1.4	7.6E-01	8.2E-01
49	NCR1	17	Weighted mode	0.98	0.88	1.1	7.6E-01	5.0E-01	1.04	0.91	1.2	5.2E-01	4.8E-01	0.92	0.71	1.19	4.9E-01	3.2E-01	0.66	0.48	0.92	1.8E-02	4.8E-02	1.04	0.74	1.45	8.3E-01	8.2E-01
49	NCR1	17	MR Egger	0.9	0.74	1.1	2.8E-01	5.0E-01	0.93	0.73	1.18	5.2E-01	4.8E-01	0.78	0.54	1.12	1.6E-01	3.2E-01	0.56	0.38	0.84	8.0E-03	4.8E-02	1.07	0.71	1.62	7.3E-01	8.2E-01
50	SFTPA2	5	Inverse variance weighted	1.06	1.02	1.1	6.7E-03	9.8E-01	1.08	1.03	1.13	2.4E-03	6.5E-01	1.03	0.94	1.13	5.0E-01	5.7E-01	1.09	0.96	1.23	1.8E-01	9.2E-01	0.97	0.85	1.09	5.8E-01	8.2E-01
50	SFTPA2	5	Weighted median	1.06	1.02	1.1	6.0E-03	9.8E-01	1.07	1.03	1.13	2.8E-03	6.5E-01	1.03	0.94	1.12	5.5E-01	5.7E-01	1.09	0.97	1.23	1.6E-01	9.2E-01	0.96	0.85	1.09	5.0E-01	8.2E-01
50	SFTPA2	5	Weighted mode	1.06	1	1.12	5.0E-02	9.8E-01	1.07	1	1.15	4.8E-02	6.5E-01	1.03	0.9	1.17	6.2E-01	5.7E-01	1.09	0.91	1.3	2.5E-01	9.2E-01	0.96	0.8	1.16	5.7E-01	8.2E-01
50	SFTPA2	5	MR Egger	1.06	0.98	1.14	1.1E-01	9.8E-01	1.07	0.98	1.17	1.0E-01	6.5E-01	1.01	0.85	1.2	8.2E-01	5.7E-01	1.08	0.86	1.37	3.6E-01	9.2E-01	0.96	0.75	1.21	5.9E-01	8.2E-01
51	LACRT	5	Inverse variance weighted	1.06	0.94	1.19	3.3E-01	2.3E-01	1	0.87	1.14	9.6E-01	9.2E-01	**1.48**	**1.15**	**1.9**	**2.5E-03**	3.1E-01	1.11	0.67	1.82	6.9E-01	1.1E-01	0.74	0.45	1.2	2.2E-01	4.9E-01
51	LACRT	5	Weighted median	1.04	0.9	1.2	6.3E-01	2.3E-01	0.99	0.85	1.15	8.6E-01	9.2E-01	**1.44**	**1.07**	**1.95**	**1.6E-02**	3.1E-01	0.98	0.64	1.52	9.4E-01	1.1E-01	0.68	0.43	1.08	1.0E-01	4.9E-01
51	LACRT	5	Weighted mode	1.02	0.81	1.28	8.6E-01	2.3E-01	0.98	0.76	1.27	8.6E-01	9.2E-01	1.46	0.87	2.47	1.1E-01	3.1E-01	0.93	0.48	1.84	7.9E-01	1.1E-01	0.72	0.32	1.63	3.3E-01	4.9E-01
51	LACRT	5	MR Egger	0.86	0.52	1.4	3.9E-01	2.3E-01	0.98	0.56	1.72	9.1E-01	9.2E-01	1.01	0.35	2.93	9.7E-01	3.1E-01	0.41	0.09	1.82	1.5E-01	1.1E-01	0.45	0.05	3.97	3.3E-01	4.9E-01
52	AGXT	10	Inverse variance weighted	0.88	0.82	0.96	2.5E-03	5.5E-01	0.87	0.8	0.96	3.8E-03	4.7E-01	0.91	0.76	1.08	2.7E-01	6.1E-01	0.95	0.74	1.21	6.6E-01	7.5E-01	1.01	0.8	1.29	9.1E-01	7.6E-01
52	AGXT	10	Weighted median	0.88	0.8	0.97	7.4E-03	5.5E-01	0.86	0.77	0.95	3.2E-03	4.7E-01	0.92	0.76	1.12	4.3E-01	6.1E-01	0.99	0.75	1.29	9.2E-01	7.5E-01	1.05	0.79	1.39	7.5E-01	7.6E-01
52	AGXT	10	Weighted mode	0.88	0.78	0.98	2.4E-02	5.5E-01	0.85	0.74	0.97	1.9E-02	4.7E-01	0.93	0.71	1.21	5.4E-01	6.1E-01	0.97	0.69	1.36	8.4E-01	7.5E-01	1.08	0.75	1.53	6.5E-01	7.6E-01
52	AGXT	10	MR Egger	0.85	0.73	1	4.9E-02	5.5E-01	0.83	0.7	1	4.7E-02	4.7E-01	0.85	0.61	1.19	3.1E-01	6.1E-01	1	0.62	1.6	1.0E+00	7.5E-01	1.07	0.67	1.7	7.5E-01	7.6E-01
53	GFRA2	6	Inverse variance weighted	1.07	0.99	1.16	1.1E-01	3.3E-01	1.08	0.96	1.21	2.2E-01	1.5E-01	1.02	0.87	1.21	7.9E-01	6.0E-01	1.32	0.89	1.94	1.6E-01	3.2E-02	0.91	0.73	1.14	4.2E-01	8.3E-01
53	GFRA2	6	Weighted median	1.05	0.97	1.13	2.7E-01	3.3E-01	1.04	0.94	1.14	4.4E-01	1.5E-01	1.05	0.88	1.25	5.7E-01	6.0E-01	1.46	1.14	1.87	2.6E-03	3.2E-02	0.88	0.69	1.13	3.2E-01	8.3E-01
53	GFRA2	6	Weighted mode	1.04	0.94	1.16	3.5E-01	3.3E-01	1.03	0.9	1.18	5.5E-01	1.5E-01	1.05	0.83	1.33	6.2E-01	6.0E-01	1.49	1.07	2.08	2.7E-02	3.2E-02	0.87	0.65	1.18	3.0E-01	8.3E-01
53	GFRA2	6	MR Egger	1.01	0.84	1.21	9.0E-01	3.3E-01	0.96	0.77	1.2	6.4E-01	1.5E-01	1.09	0.74	1.59	5.7E-01	6.0E-01	2.14	1.26	3.62	1.6E-02	3.2E-02	0.88	0.52	1.48	5.4E-01	8.3E-01
54	PSME2	1	Wald ratio	0.81	0.6	1.08	1.5E-01	NA	0.87	0.61	1.22	4.1E-01	NA	0.94	0.49	1.78	8.4E-01	NA	0.85	0.35	2.08	7.3E-01	NA	0.25	0.1	0.62	2.7E-03	NA
55	CDH1	7	Inverse variance weighted	0.9	0.82	0.98	1.6E-02	5.9E-01	0.92	0.8	1.05	2.0E-01	9.3E-01	0.85	0.77	0.95	2.8E-03	3.0E-01	0.88	0.72	1.06	1.7E-01	1.0E-01	0.86	0.71	1.04	1.2E-01	1.3E-01
55	CDH1	7	Weighted median	0.94	0.87	1	6.7E-02	5.9E-01	0.99	0.9	1.09	8.6E-01	9.3E-01	0.84	0.75	0.94	2.9E-03	3.0E-01	0.87	0.74	1.02	8.3E-02	1.0E-01	0.83	0.7	0.97	2.0E-02	1.3E-01
55	CDH1	7	Weighted mode	0.95	0.86	1.04	2.2E-01	5.9E-01	1.01	0.9	1.13	8.1E-01	9.3E-01	0.84	0.74	0.96	1.9E-02	3.0E-01	0.84	0.69	1.02	7.5E-02	1.0E-01	0.83	0.68	1.01	5.7E-02	1.3E-01
55	CDH1	7	MR Egger	0.87	0.71	1.06	1.2E-01	5.9E-01	0.92	0.68	1.26	5.4E-01	9.3E-01	0.79	0.63	0.98	4.0E-02	3.0E-01	0.72	0.52	1	4.9E-02	1.0E-01	0.71	0.5	1	5.2E-02	1.3E-01
56	CRYGD	9	Inverse variance weighted	0.94	0.91	0.98	2.8E-03	5.8E-01	0.94	0.9	0.98	3.6E-03	6.7E-01	0.98	0.89	1.07	6.7E-01	1.8E-01	0.9	0.8	1.01	6.6E-02	1.1E-01	0.93	0.83	1.04	2.1E-01	7.7E-01
56	CRYGD	9	Weighted median	0.95	0.91	0.98	5.5E-03	5.8E-01	0.94	0.9	0.98	4.0E-03	6.7E-01	1	0.92	1.09	9.8E-01	1.8E-01	0.88	0.78	0.98	2.6E-02	1.1E-01	0.93	0.83	1.05	2.6E-01	7.7E-01
56	CRYGD	9	Weighted mode	0.95	0.9	0.99	2.5E-02	5.8E-01	0.94	0.89	0.99	2.3E-02	6.7E-01	1	0.9	1.11	9.5E-01	1.8E-01	0.87	0.77	0.99	4.2E-02	1.1E-01	0.93	0.81	1.07	2.9E-01	7.7E-01
56	CRYGD	9	MR Egger	0.95	0.9	1.01	8.1E-02	5.8E-01	0.94	0.88	1.01	7.8E-02	6.7E-01	1.03	0.9	1.17	6.2E-01	1.8E-01	0.83	0.69	0.98	3.6E-02	1.1E-01	0.94	0.79	1.12	4.3E-01	7.7E-01
57	HEPH	6	Inverse variance weighted	0.99	0.85	1.15	9.0E-01	8.6E-01	1.08	0.96	1.22	1.8E-01	8.0E-01	0.85	0.65	1.09	2.0E-01	6.5E-01	0.69	0.5	0.95	2.3E-02	5.5E-01	0.99	0.74	1.32	9.3E-01	7.4E-01
57	HEPH	6	Weighted median	0.97	0.85	1.1	6.2E-01	8.6E-01	1.07	0.93	1.23	3.3E-01	8.0E-01	0.77	0.58	1.01	5.7E-02	6.5E-01	0.6	0.43	0.84	2.8E-03	5.5E-01	0.91	0.63	1.32	6.3E-01	7.4E-01
57	HEPH	6	Weighted mode	0.93	0.77	1.11	3.3E-01	8.6E-01	1.07	0.89	1.29	4.1E-01	8.0E-01	0.73	0.49	1.09	1.0E-01	6.5E-01	0.59	0.38	0.92	2.8E-02	5.5E-01	0.82	0.49	1.36	3.5E-01	7.4E-01
57	HEPH	6	MR Egger	1.02	0.66	1.56	9.3E-01	8.6E-01	1.11	0.8	1.56	4.2E-01	8.0E-01	0.94	0.46	1.94	8.3E-01	6.5E-01	0.59	0.26	1.33	1.5E-01	5.5E-01	1.08	0.48	2.42	8.1E-01	7.4E-01
58	TPPP3	1	Wald ratio	0.53	0.35	0.81	2.9E-03	NA	0.53	0.33	0.86	1.1E-02	NA	0.5	0.2	1.24	1.3E-01	NA	0.53	0.15	1.89	3.3E-01	NA	0.21	0.06	0.72	1.2E-02	NA
59	FIS1	5	Inverse variance weighted	0.84	0.7	1.01	6.8E-02	3.4E-01	0.85	0.66	1.1	2.2E-01	3.1E-01	0.91	0.67	1.22	5.2E-01	5.9E-01	1.01	0.67	1.54	9.5E-01	7.9E-01	0.74	0.49	1.1	1.4E-01	8.7E-01
59	FIS1	5	Weighted median	0.79	0.68	0.92	2.9E-03	3.4E-01	0.78	0.65	0.93	6.9E-03	3.1E-01	0.85	0.62	1.17	3.2E-01	5.9E-01	1.04	0.65	1.68	8.6E-01	7.9E-01	0.71	0.45	1.13	1.5E-01	8.7E-01
59	FIS1	5	Weighted mode	0.79	0.64	0.98	3.8E-02	3.4E-01	0.77	0.61	0.98	3.7E-02	3.1E-01	0.86	0.53	1.37	4.1E-01	5.9E-01	1.06	0.51	2.17	8.4E-01	7.9E-01	0.71	0.37	1.37	2.2E-01	8.7E-01
59	FIS1	5	MR Egger	0.75	0.49	1.16	1.3E-01	3.4E-01	0.72	0.4	1.29	1.7E-01	3.1E-01	0.81	0.38	1.77	4.6E-01	5.9E-01	1.09	0.38	3.09	8.1E-01	7.9E-01	0.7	0.26	1.9	3.4E-01	8.7E-01
60	ITIH5	1	Wald ratio	1.09	0.77	1.54	6.2E-01	NA	1.31	0.88	1.94	1.8E-01	NA	0.87	0.42	1.84	7.2E-01	NA	1.2	0.43	3.34	7.3E-01	NA	0.2	0.07	0.58	3.0E-03	NA
61	LEFTY2	4	Inverse variance weighted	0.59	0.33	1.06	7.6E-02	9.4E-01	0.53	0.25	1.14	1.1E-01	9.8E-01	0.61	0.37	1.02	6.0E-02	6.5E-01	0.91	0.45	1.88	8.1E-01	9.6E-01	0.34	0.17	0.7	3.0E-03	8.9E-01
61	LEFTY2	4	Weighted median	0.72	0.49	1.04	8.3E-02	9.4E-01	0.73	0.48	1.12	1.5E-01	9.8E-01	0.75	0.4	1.4	3.7E-01	6.5E-01	0.92	0.39	2.19	8.6E-01	9.6E-01	0.33	0.14	0.8	1.4E-02	8.9E-01
61	LEFTY2	4	Weighted mode	0.84	0.43	1.66	4.8E-01	9.4E-01	0.89	0.46	1.72	6.2E-01	9.8E-01	0.81	0.18	3.73	6.9E-01	6.5E-01	0.94	0.16	5.49	9.1E-01	9.6E-01	0.25	0.04	1.74	1.1E-01	8.9E-01
61	LEFTY2	4	MR Egger	0.66	0	223.02	7.9E-01	9.4E-01	0.55	0	1067.92	7.7E-01	9.8E-01	0.38	0.01	24.01	4.2E-01	6.5E-01	0.85	0	284.27	9.1E-01	9.6E-01	0.28	0	89.2	4.4E-01	8.9E-01
62	PSME1	2	Inverse variance weighted	0.79	0.6	1.03	8.6E-02	NA	0.83	0.6	1.14	2.4E-01	NA	0.91	0.5	1.64	7.4E-01	NA	0.74	0.32	1.68	4.7E-01	NA	0.29	0.13	0.66	3.1E-03	NA
63	PRCP	8	Inverse variance weighted	1.01	0.9	1.13	8.6E-01	9.5E-01	1.08	0.95	1.23	2.5E-01	8.9E-01	0.85	0.67	1.09	2.0E-01	4.7E-01	1.34	0.96	1.86	8.3E-02	2.3E-01	0.58	0.41	0.83	3.2E-03	1.8E-01
63	PRCP	8	Weighted median	1.03	0.89	1.18	7.1E-01	9.5E-01	1.1	0.92	1.3	2.9E-01	8.9E-01	0.82	0.61	1.1	1.8E-01	4.7E-01	1.55	0.98	2.45	6.3E-02	2.3E-01	0.56	0.36	0.89	1.3E-02	1.8E-01
63	PRCP	8	Weighted mode	1.02	0.84	1.26	7.8E-01	9.5E-01	1.14	0.9	1.46	2.4E-01	8.9E-01	0.88	0.58	1.33	4.8E-01	4.7E-01	1.76	0.93	3.32	7.5E-02	2.3E-01	0.49	0.26	0.93	3.3E-02	1.8E-01
63	PRCP	8	MR Egger	1.02	0.79	1.3	8.9E-01	9.5E-01	1.09	0.8	1.49	5.0E-01	8.9E-01	0.98	0.57	1.68	9.3E-01	4.7E-01	1.82	0.9	3.68	8.2E-02	2.3E-01	0.37	0.15	0.87	3.0E-02	1.8E-01
64	THSD1	2	Inverse variance weighted	0.68	0.51	0.92	1.3E-02	NA	0.66	0.39	1.12	1.2E-01	NA	0.64	0.47	0.86	3.4E-03	NA	0.86	0.51	1.44	5.7E-01	NA	0.58	0.39	0.87	7.8E-03	NA
65	PAEP	2	Inverse variance weighted	1.04	0.99	1.1	1.6E-01	NA	1.01	0.97	1.06	5.5E-01	NA	1.05	0.96	1.14	2.6E-01	NA	1.19	1.06	1.34	3.5E-03	NA	1.03	0.92	1.16	5.7E-01	NA
66	CGA	1	Wald ratio	3.41	1.5	7.79	3.6E-03	NA	3.03	1.17	7.82	2.2E-02	NA	5.88	0.99	34.93	5.1E-02	NA	27.32	2.43	307.67	7.4E-03	NA	2.5	0.21	29.57	4.7E-01	NA
67	FABP5	2	Inverse variance weighted	1	0.79	1.27	9.8E-01	NA	1.2	0.91	1.57	2.0E-01	NA	0.48	0.3	0.79	3.7E-03	NA	1.56	0.46	5.26	4.7E-01	NA	0.8	0.4	1.59	5.2E-01	NA
68	GNPDA2	2	Inverse variance weighted	0.81	0.71	0.93	3.7E-03	NA	0.79	0.68	0.93	5.1E-03	NA	0.8	0.59	1.09	1.6E-01	NA	0.6	0.4	0.91	1.7E-02	NA	0.97	0.63	1.47	8.7E-01	NA
69	SPARCL1	5	Inverse variance weighted	0.98	0.94	1.02	2.8E-01	4.9E-01	0.98	0.94	1.02	3.7E-01	3.5E-01	1.03	0.95	1.11	5.0E-01	8.2E-01	0.85	0.75	0.95	3.9E-03	4.5E-01	0.97	0.86	1.08	5.3E-01	9.0E-01
69	SPARCL1	5	Weighted median	0.98	0.95	1.02	3.8E-01	4.9E-01	0.98	0.94	1.03	4.1E-01	3.5E-01	1.03	0.95	1.11	5.3E-01	8.2E-01	0.85	0.76	0.96	5.9E-03	4.5E-01	0.97	0.86	1.09	5.8E-01	9.0E-01
69	SPARCL1	5	Weighted mode	0.98	0.93	1.04	4.7E-01	4.9E-01	0.99	0.93	1.05	6.2E-01	3.5E-01	1.02	0.92	1.14	5.9E-01	8.2E-01	0.85	0.73	1.01	5.6E-02	4.5E-01	0.97	0.82	1.15	6.4E-01	9.0E-01
69	SPARCL1	5	MR Egger	0.99	0.91	1.08	8.1E-01	4.9E-01	1	0.91	1.1	9.3E-01	3.5E-01	1.04	0.86	1.25	5.7E-01	8.2E-01	0.89	0.69	1.14	2.2E-01	4.5E-01	0.97	0.76	1.24	7.4E-01	9.0E-01
70	PGF	3	Inverse variance weighted	1.06	0.83	1.36	6.2E-01	6.1E-01	0.94	0.71	1.25	6.8E-01	5.1E-01	1.16	0.68	1.96	5.9E-01	6.5E-01	2.98	1.42	6.24	3.9E-03	7.2E-01	0.95	0.46	1.97	8.8E-01	7.9E-01
70	PGF	3	Weighted median	1.07	0.82	1.39	6.4E-01	6.1E-01	0.9	0.66	1.22	4.9E-01	5.1E-01	1.16	0.65	2.07	6.3E-01	6.5E-01	3.05	1.38	6.78	6.0E-03	7.2E-01	0.85	0.37	1.94	7.0E-01	7.9E-01
70	PGF	3	Weighted mode	0.99	0.52	1.89	9.7E-01	6.1E-01	0.87	0.42	1.8	4.9E-01	5.1E-01	1.3	0.32	5.22	5.0E-01	6.5E-01	3.31	0.45	24.19	1.2E-01	7.2E-01	0.81	0.11	5.81	6.9E-01	7.9E-01
70	PGF	3	MR Egger	0.79	0	235.43	6.9E-01	6.1E-01	0.58	0	416.77	4.8E-01	5.1E-01	2.06	0	486786.05	5.9E-01	6.5E-01	5.55	0	188155730.8	4.3E-01	7.2E-01	0.6	0	16153981.75	7.7E-01	7.9E-01
71	INPP5J	1	Wald ratio	0.65	0.43	0.99	4.7E-02	NA	0.49	0.3	0.8	3.9E-03	NA	1.19	0.47	2.99	7.1E-01	NA	1.77	0.49	6.47	3.9E-01	NA	1	0.28	3.59	1.0E+00	NA
72	TPSD1	3	Inverse variance weighted	1.18	0.93	1.51	1.8E-01	7.1E-01	1.21	0.91	1.6	1.9E-01	9.4E-01	1.03	0.61	1.74	9.2E-01	4.7E-01	0.83	0.39	1.73	6.1E-01	6.0E-01	2.35	0.59	9.42	2.3E-01	6.5E-01
72	TPSD1	3	Weighted median	1.23	0.94	1.6	1.3E-01	7.1E-01	1.19	0.86	1.63	2.9E-01	9.4E-01	1.02	0.56	1.85	9.5E-01	4.7E-01	0.8	0.35	1.83	6.0E-01	6.0E-01	3.64	1.51	8.76	4.0E-03	6.5E-01
72	TPSD1	3	Weighted mode	1.25	0.57	2.75	3.5E-01	7.1E-01	1.17	0.5	2.75	5.0E-01	9.4E-01	1.28	0.28	5.95	5.6E-01	4.7E-01	1.07	0.14	8.28	9.0E-01	6.0E-01	3.85	0.55	27.05	9.7E-02	6.5E-01
72	TPSD1	3	MR Egger	1.37	0.02	77.72	5.0E-01	7.1E-01	1.17	0.01	259.82	7.8E-01	9.4E-01	2.06	0	12660.24	4.8E-01	4.7E-01	1.58	0	332873.23	7.2E-01	6.0E-01	7.88	0	5.75149E+12	5.1E-01	6.5E-01
73	ADIPOQ	6	Inverse variance weighted	0.97	0.87	1.08	6.0E-01	1.5E-01	1.01	0.88	1.16	9.0E-01	1.3E-01	1	0.76	1.33	9.9E-01	7.4E-01	1.2	0.85	1.7	3.1E-01	8.7E-01	0.66	0.46	0.95	2.4E-02	9.6E-02
73	ADIPOQ	6	Weighted median	0.93	0.82	1.06	2.7E-01	1.5E-01	0.96	0.84	1.11	6.1E-01	1.3E-01	0.94	0.72	1.21	6.2E-01	7.4E-01	1.19	0.82	1.74	3.5E-01	8.7E-01	0.62	0.44	0.86	4.2E-03	9.6E-02
73	ADIPOQ	6	Weighted mode	0.93	0.78	1.11	3.3E-01	1.5E-01	0.95	0.79	1.14	5.2E-01	1.3E-01	0.93	0.66	1.31	6.1E-01	7.4E-01	1.15	0.7	1.87	5.1E-01	8.7E-01	0.58	0.37	0.9	2.5E-02	9.6E-02
73	ADIPOQ	6	MR Egger	0.89	0.72	1.09	1.9E-01	1.5E-01	0.9	0.71	1.15	3.1E-01	1.3E-01	0.95	0.52	1.73	8.3E-01	7.4E-01	1.16	0.55	2.47	6.1E-01	8.7E-01	0.49	0.28	0.87	2.5E-02	9.6E-02
74	GPIHBP1	4	Inverse variance weighted	0.99	0.9	1.09	8.6E-01	9.6E-01	0.99	0.89	1.1	8.3E-01	8.3E-01	1.03	0.84	1.26	7.5E-01	5.4E-01	1.06	0.8	1.41	6.8E-01	5.5E-01	0.67	0.51	0.88	4.3E-03	9.3E-01
74	GPIHBP1	4	Weighted median	0.99	0.9	1.09	9.0E-01	9.6E-01	1	0.89	1.12	9.6E-01	8.3E-01	1.01	0.82	1.25	9.1E-01	5.4E-01	1.03	0.76	1.38	8.7E-01	5.5E-01	0.68	0.51	0.91	1.0E-02	9.3E-01
74	GPIHBP1	4	Weighted mode	0.99	0.85	1.17	9.0E-01	9.6E-01	0.99	0.82	1.2	8.8E-01	8.3E-01	1.02	0.71	1.45	8.9E-01	5.4E-01	1.02	0.62	1.69	8.9E-01	5.5E-01	0.69	0.42	1.13	9.6E-02	9.3E-01
74	GPIHBP1	4	MR Egger	1	0.67	1.49	9.7E-01	9.6E-01	1.01	0.59	1.74	9.2E-01	8.3E-01	0.91	0.38	2.16	6.8E-01	5.4E-01	0.88	0.23	3.35	7.2E-01	5.5E-01	0.65	0.17	2.55	3.1E-01	9.3E-01
75	CLPS	6	Inverse variance weighted	1.02	0.97	1.08	4.4E-01	8.6E-01	1	0.95	1.06	9.6E-01	3.4E-01	1.04	0.9	1.21	6.1E-01	5.4E-01	1.23	1.05	1.44	9.7E-03	8.1E-01	1.02	0.82	1.27	8.3E-01	5.6E-01
75	CLPS	6	Weighted median	1.02	0.97	1.07	4.9E-01	8.6E-01	1	0.95	1.06	9.1E-01	3.4E-01	1.02	0.92	1.14	7.0E-01	5.4E-01	1.23	1.07	1.42	4.4E-03	8.1E-01	1.01	0.87	1.16	9.4E-01	5.6E-01
75	CLPS	6	Weighted mode	1.02	0.96	1.08	5.2E-01	8.6E-01	1.01	0.93	1.08	8.4E-01	3.4E-01	1.02	0.89	1.18	6.7E-01	5.4E-01	1.24	1.03	1.5	3.1E-02	8.1E-01	0.99	0.82	1.2	9.2E-01	5.6E-01
75	CLPS	6	MR Egger	1.02	0.91	1.14	7.2E-01	8.6E-01	1.03	0.93	1.14	5.0E-01	3.4E-01	0.99	0.74	1.34	9.4E-01	5.4E-01	1.21	0.87	1.68	1.9E-01	8.1E-01	0.96	0.62	1.48	8.0E-01	5.6E-01
76	TMPRSS15	6	Inverse variance weighted	0.91	0.82	1.01	6.6E-02	3.7E-01	0.97	0.85	1.1	5.9E-01	7.1E-01	0.87	0.69	1.08	2.1E-01	2.0E-01	0.64	0.47	0.88	5.8E-03	3.9E-01	0.88	0.52	1.49	6.4E-01	6.2E-01
76	TMPRSS15	6	Weighted median	0.89	0.79	1	5.0E-02	3.7E-01	0.97	0.84	1.12	6.4E-01	7.1E-01	0.77	0.58	1.03	7.9E-02	2.0E-01	0.58	0.4	0.84	4.4E-03	3.9E-01	0.87	0.58	1.3	5.0E-01	6.2E-01
76	TMPRSS15	6	Weighted mode	0.88	0.74	1.06	1.4E-01	3.7E-01	1.01	0.83	1.24	8.7E-01	7.1E-01	0.7	0.47	1.02	6.0E-02	2.0E-01	0.55	0.31	0.97	4.2E-02	3.9E-01	0.76	0.41	1.4	3.0E-01	6.2E-01
76	TMPRSS15	6	MR Egger	0.79	0.52	1.19	1.8E-01	3.7E-01	1.04	0.6	1.79	8.5E-01	7.1E-01	0.55	0.23	1.34	1.4E-01	2.0E-01	0.43	0.12	1.49	1.3E-01	3.9E-01	1.32	0.14	12.59	7.5E-01	6.2E-01
77	CEACAM5	12	Inverse variance weighted	1.05	0.99	1.11	1.4E-01	4.8E-01	0.99	0.93	1.05	6.9E-01	1.1E-01	**1.22**	**1.06**	**1.39**	**4.4E-03**	4.3E-01	1.14	0.97	1.34	1.1E-01	5.6E-01	1.24	1.04	1.47	1.6E-02	3.9E-02
77	CEACAM5	12	Weighted median	1.06	1	1.13	4.5E-02	4.8E-01	1	0.93	1.08	9.5E-01	1.1E-01	**1.2**	**1.06**	**1.36**	**5.3E-03**	4.3E-01	1.21	0.98	1.48	7.0E-02	5.6E-01	1.16	0.97	1.39	1.0E-01	3.9E-02
77	CEACAM5	12	Weighted mode	1.06	1	1.13	5.5E-02	4.8E-01	1.01	0.94	1.08	8.4E-01	1.1E-01	**1.2**	**1.06**	**1.36**	**7.6E-03**	4.3E-01	1.18	0.97	1.44	9.0E-02	5.6E-01	1.16	0.96	1.41	1.1E-01	3.9E-02
77	CEACAM5	12	MR Egger	1.07	0.97	1.18	1.5E-01	4.8E-01	1.04	0.95	1.13	4.0E-01	1.1E-01	1.16	0.94	1.43	1.5E-01	4.3E-01	1.2	0.93	1.54	1.5E-01	5.6E-01	1.05	0.84	1.32	6.3E-01	3.9E-02
78	CALCB	1	Wald ratio	1.27	1	1.62	5.4E-02	NA	1.18	0.89	1.56	2.4E-01	NA	2.12	1.26	3.56	4.5E-03	NA	0.82	0.39	1.7	5.9E-01	NA	1.67	0.81	3.44	1.7E-01	NA
79	SCGB1A1	11	Inverse variance weighted	0.92	0.84	1.01	7.4E-02	7.4E-01	0.91	0.82	1	5.4E-02	9.3E-01	1.19	0.99	1.43	5.7E-02	3.7E-01	0.81	0.66	0.99	3.7E-02	8.8E-02	0.79	0.65	0.96	1.6E-02	4.3E-01
79	SCGB1A1	11	Weighted median	0.9	0.83	0.97	4.6E-03	7.4E-01	0.88	0.81	0.96	4.6E-03	9.3E-01	1.16	0.98	1.36	7.9E-02	3.7E-01	0.75	0.6	0.94	1.3E-02	8.8E-02	0.83	0.66	1.03	9.2E-02	4.3E-01
79	SCGB1A1	11	Weighted mode	0.9	0.82	0.98	1.8E-02	7.4E-01	0.88	0.8	0.98	1.9E-02	9.3E-01	1.14	0.94	1.38	1.6E-01	3.7E-01	0.74	0.56	0.97	3.1E-02	8.8E-02	0.83	0.64	1.08	1.5E-01	4.3E-01
79	SCGB1A1	11	MR Egger	0.9	0.76	1.07	2.2E-01	7.4E-01	0.9	0.74	1.09	2.6E-01	9.3E-01	1.07	0.76	1.5	6.6E-01	3.7E-01	0.64	0.44	0.92	2.1E-02	8.8E-02	0.87	0.61	1.25	4.2E-01	4.3E-01
80	COL24A1	2	Inverse variance weighted	1.26	1.07	1.49	4.7E-03	NA	1.26	1.04	1.52	1.7E-02	NA	1.42	0.99	2.03	5.4E-02	NA	1.04	0.64	1.68	8.9E-01	NA	1.37	0.78	2.42	2.7E-01	NA
81	KLRB1	9	Inverse variance weighted	0.98	0.7	1.37	9.0E-01	9.0E-01	1.01	0.67	1.51	9.7E-01	8.3E-01	0.85	0.62	1.18	3.3E-01	9.9E-01	0.8	0.56	1.15	2.2E-01	1.7E-01	0.78	0.54	1.12	1.8E-01	8.8E-02
81	KLRB1	9	Weighted median	0.98	0.85	1.14	8.1E-01	9.0E-01	0.94	0.79	1.12	4.8E-01	8.3E-01	0.83	0.6	1.13	2.4E-01	9.9E-01	1.04	0.67	1.61	8.5E-01	1.7E-01	0.53	0.34	0.82	5.0E-03	8.8E-02
81	KLRB1	9	Weighted mode	0.97	0.81	1.17	7.5E-01	9.0E-01	0.97	0.8	1.18	7.3E-01	8.3E-01	0.84	0.54	1.31	3.9E-01	9.9E-01	1.07	0.6	1.88	8.0E-01	1.7E-01	0.58	0.32	1.05	6.5E-02	8.8E-02
81	KLRB1	9	MR Egger	0.92	0.3	2.88	8.7E-01	9.0E-01	0.89	0.23	3.5	8.5E-01	8.3E-01	0.86	0.29	2.58	7.5E-01	9.9E-01	1.52	0.52	4.45	3.9E-01	1.7E-01	0.35	0.12	0.98	4.7E-02	8.8E-02
82	DEFB103A_	1	Wald ratio	1.77	1.1	2.84	1.8E-02	NA	2.17	1.26	3.74	5.1E-03	NA	1.47	0.53	4.1	4.6E-01	NA	0.47	0.11	2.09	3.2E-01	NA	4.17	1.08	16.07	3.8E-02	NA
83	FGR	1	Wald ratio	1.03	0.76	1.4	8.5E-01	NA	0.94	0.66	1.34	7.4E-01	NA	1.09	0.56	2.14	8.0E-01	NA	1.8	0.69	4.7	2.3E-01	NA	4.06	1.52	10.85	5.1E-03	NA
84	LILRB4	4	Inverse variance weighted	0.97	0.93	1.02	2.8E-01	3.4E-01	0.98	0.93	1.04	5.6E-01	7.3E-01	0.96	0.86	1.08	5.0E-01	2.3E-01	1.09	0.92	1.3	3.2E-01	2.5E-01	0.8	0.65	1	5.2E-02	2.3E-01
84	LILRB4	4	Weighted median	0.98	0.93	1.02	3.1E-01	3.4E-01	0.99	0.93	1.04	6.0E-01	7.3E-01	0.97	0.87	1.07	5.3E-01	2.3E-01	1.1	0.95	1.26	2.0E-01	2.5E-01	0.81	0.7	0.94	5.3E-03	2.3E-01
84	LILRB4	4	Weighted mode	0.98	0.91	1.06	5.2E-01	3.4E-01	0.99	0.9	1.08	6.6E-01	7.3E-01	0.98	0.83	1.15	7.1E-01	2.3E-01	1.15	0.9	1.48	1.7E-01	2.5E-01	0.84	0.65	1.08	1.2E-01	2.3E-01
84	LILRB4	4	MR Egger	1.01	0.87	1.17	8.8E-01	3.4E-01	1	0.84	1.18	9.3E-01	7.3E-01	1.06	0.77	1.46	5.3E-01	2.3E-01	1.24	0.78	1.95	1.8E-01	2.5E-01	0.94	0.54	1.64	7.0E-01	2.3E-01
85	IL15	20	Inverse variance weighted	1.15	1.04	1.27	5.3E-03	3.6E-01	1.13	1.01	1.27	3.5E-02	3.0E-01	1.28	1.07	1.52	7.5E-03	3.4E-01	1.23	0.89	1.69	2.0E-01	8.4E-01	1	0.78	1.27	9.7E-01	2.5E-01
85	IL15	20	Weighted median	1.11	0.98	1.25	9.3E-02	3.6E-01	1.12	0.96	1.31	1.4E-01	3.0E-01	1.3	1.02	1.66	3.6E-02	3.4E-01	1.22	0.81	1.84	3.5E-01	8.4E-01	0.97	0.69	1.35	8.4E-01	2.5E-01
85	IL15	20	Weighted mode	1.07	0.89	1.29	4.6E-01	3.6E-01	1.15	0.92	1.44	2.0E-01	3.0E-01	1.21	0.87	1.67	2.4E-01	3.4E-01	0.89	0.44	1.8	7.4E-01	8.4E-01	0.95	0.63	1.43	8.0E-01	2.5E-01
85	IL15	20	MR Egger	1.28	0.99	1.66	6.3E-02	3.6E-01	1.31	0.96	1.78	8.4E-02	3.0E-01	1.56	0.97	2.49	6.3E-02	3.4E-01	1.14	0.48	2.7	7.5E-01	8.4E-01	0.71	0.37	1.36	2.8E-01	2.5E-01
86	LYSMD3	4	Inverse variance weighted	0.73	0.59	0.91	5.5E-03	3.8E-01	0.7	0.54	0.91	6.8E-03	8.0E-01	0.62	0.31	1.24	1.8E-01	3.4E-01	0.74	0.38	1.44	3.8E-01	8.5E-01	0.97	0.47	2.02	9.4E-01	2.3E-01
86	LYSMD3	4	Weighted median	0.76	0.58	1	5.1E-02	3.8E-01	0.71	0.53	0.95	1.9E-02	8.0E-01	0.74	0.39	1.39	3.4E-01	3.4E-01	0.74	0.35	1.59	4.4E-01	8.5E-01	1.15	0.47	2.78	7.6E-01	2.3E-01
86	LYSMD3	4	Weighted mode	0.8	0.44	1.46	3.3E-01	3.8E-01	0.74	0.4	1.37	2.2E-01	8.0E-01	1.02	0.19	5.33	9.8E-01	3.4E-01	0.78	0.16	3.78	6.5E-01	8.5E-01	1.5	0.24	9.21	5.3E-01	2.3E-01
86	LYSMD3	4	MR Egger	1.25	0.15	10.61	7.0E-01	3.8E-01	0.82	0.07	9.77	7.7E-01	8.0E-01	3.46	0.01	1581.53	4.7E-01	3.4E-01	1.01	0	656.34	9.9E-01	8.5E-01	11.21	0.02	6462.44	2.4E-01	2.3E-01
87	CXADR	5	Inverse variance weighted	0.9	0.81	1	4.6E-02	4.6E-01	0.96	0.83	1.11	6.0E-01	9.9E-01	0.84	0.67	1.06	1.4E-01	5.5E-01	0.78	0.45	1.35	3.7E-01	4.0E-01	0.87	0.62	1.22	4.1E-01	8.4E-01
87	CXADR	5	Weighted median	0.88	0.78	1	4.2E-02	4.6E-01	0.99	0.85	1.14	8.4E-01	9.9E-01	0.84	0.64	1.1	2.1E-01	5.5E-01	0.58	0.39	0.85	5.6E-03	4.0E-01	0.74	0.52	1.07	1.1E-01	8.4E-01
87	CXADR	5	Weighted mode	0.88	0.72	1.07	1.4E-01	4.6E-01	1.02	0.8	1.29	8.5E-01	9.9E-01	0.72	0.48	1.09	9.5E-02	5.5E-01	0.59	0.34	1.03	5.7E-02	4.0E-01	0.75	0.45	1.26	2.0E-01	8.4E-01
87	CXADR	5	MR Egger	0.81	0.54	1.22	2.1E-01	4.6E-01	0.96	0.51	1.82	8.5E-01	9.9E-01	0.7	0.27	1.82	3.2E-01	5.5E-01	0.42	0.05	3.71	3.0E-01	4.0E-01	0.78	0.17	3.64	6.5E-01	8.4E-01
88	NRTN	1	Wald ratio	1.74	1	3.04	5.1E-02	NA	1.5	0.79	2.85	2.2E-01	NA	2.01	0.6	6.73	2.6E-01	NA	0.4	0.08	2.04	2.7E-01	NA	10.98	2.01	59.83	5.6E-03	NA
89	TNFRSF8	7	Inverse variance weighted	1.03	0.93	1.14	5.5E-01	9.6E-01	1.05	0.94	1.19	3.9E-01	5.1E-01	1.09	0.87	1.36	4.5E-01	4.5E-01	1.17	0.76	1.79	4.8E-01	2.9E-02	0.72	0.52	0.98	3.8E-02	3.7E-01
89	TNFRSF8	7	Weighted median	1.02	0.9	1.15	7.8E-01	9.6E-01	1.06	0.91	1.22	4.6E-01	5.1E-01	1.25	0.96	1.63	1.0E-01	4.5E-01	1	0.66	1.52	9.9E-01	2.9E-02	0.56	0.38	0.85	5.7E-03	3.7E-01
89	TNFRSF8	7	Weighted mode	1.01	0.85	1.21	8.6E-01	9.6E-01	1.04	0.87	1.25	6.2E-01	5.1E-01	1.2	0.87	1.67	2.2E-01	4.5E-01	0.92	0.53	1.59	7.2E-01	2.9E-02	0.53	0.31	0.92	3.1E-02	3.7E-01
89	TNFRSF8	7	MR Egger	1.03	0.8	1.31	7.9E-01	9.6E-01	1.12	0.85	1.49	3.3E-01	5.1E-01	1.25	0.74	2.11	3.2E-01	4.5E-01	0.57	0.27	1.18	1.0E-01	2.9E-02	0.56	0.27	1.19	1.1E-01	3.7E-01
90	GALNT10	7	Inverse variance weighted	1.1	0.96	1.26	1.7E-01	9.9E-01	1.14	0.98	1.33	8.7E-02	7.7E-01	0.98	0.8	1.21	8.7E-01	4.5E-01	1.09	0.86	1.39	4.6E-01	9.5E-01	1.26	0.98	1.62	6.7E-02	7.6E-01
90	GALNT10	7	Weighted median	1.04	0.96	1.12	3.7E-01	9.9E-01	1.09	1	1.2	5.8E-02	7.7E-01	0.89	0.76	1.04	1.4E-01	4.5E-01	1.09	0.9	1.32	4.0E-01	9.5E-01	1.31	1.08	1.58	5.7E-03	7.6E-01
90	GALNT10	7	Weighted mode	1.02	0.92	1.13	6.7E-01	9.9E-01	1.05	0.94	1.17	3.3E-01	7.7E-01	0.88	0.72	1.07	1.6E-01	4.5E-01	1.08	0.86	1.38	4.3E-01	9.5E-01	1.3	1.01	1.68	4.4E-02	7.6E-01
90	GALNT10	7	MR Egger	1.1	0.81	1.49	4.7E-01	9.9E-01	1.18	0.84	1.65	2.7E-01	7.7E-01	0.89	0.57	1.36	5.0E-01	4.5E-01	1.11	0.65	1.89	6.5E-01	9.5E-01	1.33	0.77	2.32	2.4E-01	7.6E-01
91	ACOT13	4	Inverse variance weighted	0.99	0.78	1.24	9.1E-01	2.5E-01	0.98	0.67	1.45	9.3E-01	3.4E-01	0.93	0.54	1.61	8.0E-01	3.4E-01	0.83	0.31	2.26	7.2E-01	4.2E-01	0.42	0.23	0.78	5.7E-03	2.2E-01
91	ACOT13	4	Weighted median	0.92	0.7	1.21	5.5E-01	2.5E-01	0.97	0.7	1.35	8.8E-01	3.4E-01	0.88	0.49	1.61	6.9E-01	3.4E-01	0.62	0.26	1.47	2.8E-01	4.2E-01	0.53	0.26	1.11	9.2E-02	2.2E-01
91	ACOT13	4	Weighted mode	0.89	0.51	1.54	5.4E-01	2.5E-01	0.8	0.34	1.84	4.5E-01	3.4E-01	0.84	0.29	2.46	6.5E-01	3.4E-01	0.63	0.17	2.29	3.4E-01	4.2E-01	0.59	0.09	3.73	4.3E-01	2.2E-01
91	ACOT13	4	MR Egger	1.57	0.4	6.1	2.9E-01	2.5E-01	1.79	0.19	16.55	3.8E-01	3.4E-01	2.68	0.06	123.41	3.8E-01	3.4E-01	12.32	0	1607632.65	4.6E-01	4.2E-01	0.17	0.01	2.08	9.4E-02	2.2E-01
92	TCL1A	20	Inverse variance weighted	0.93	0.86	1.01	9.3E-02	8.7E-02	0.97	0.86	1.09	6.4E-01	1.2E-01	0.88	0.75	1.02	8.8E-02	6.1E-01	0.94	0.76	1.16	5.6E-01	7.9E-01	0.8	0.61	1.05	1.1E-01	4.0E-01
92	TCL1A	20	Weighted median	0.88	0.8	0.96	5.7E-03	8.7E-02	0.87	0.79	0.97	1.3E-02	1.2E-01	0.87	0.71	1.06	1.6E-01	6.1E-01	0.96	0.72	1.29	8.0E-01	7.9E-01	0.81	0.62	1.07	1.4E-01	4.0E-01
92	TCL1A	20	Weighted mode	0.88	0.79	0.96	9.8E-03	8.7E-02	0.89	0.8	1	4.5E-02	1.2E-01	0.85	0.69	1.05	1.3E-01	6.1E-01	0.93	0.68	1.28	6.4E-01	7.9E-01	0.82	0.6	1.13	2.1E-01	4.0E-01
92	TCL1A	20	MR Egger	0.86	0.75	0.97	2.1E-02	8.7E-02	0.87	0.73	1.05	1.4E-01	1.2E-01	0.84	0.65	1.07	1.5E-01	6.1E-01	0.91	0.65	1.28	5.7E-01	7.9E-01	0.7	0.45	1.08	1.0E-01	4.0E-01
93	CD160	12	Inverse variance weighted	0.96	0.87	1.05	3.7E-01	7.2E-01	0.97	0.88	1.07	5.4E-01	8.7E-01	0.83	0.73	0.95	5.7E-03	6.2E-01	0.96	0.76	1.21	7.3E-01	5.4E-01	1.09	0.89	1.34	3.9E-01	7.2E-01
93	CD160	12	Weighted median	0.99	0.93	1.05	7.2E-01	7.2E-01	1	0.93	1.08	9.9E-01	8.7E-01	0.88	0.77	1.01	7.0E-02	6.2E-01	0.96	0.8	1.16	6.9E-01	5.4E-01	1.04	0.87	1.26	6.6E-01	7.2E-01
93	CD160	12	Weighted mode	1	0.93	1.07	8.9E-01	7.2E-01	1.01	0.93	1.09	8.5E-01	8.7E-01	0.87	0.74	1.01	7.1E-02	6.2E-01	0.97	0.78	1.21	7.8E-01	5.4E-01	1.04	0.84	1.29	7.0E-01	7.2E-01
93	CD160	12	MR Egger	0.98	0.82	1.17	8.0E-01	7.2E-01	0.98	0.81	1.18	8.2E-01	8.7E-01	0.87	0.68	1.11	2.3E-01	6.2E-01	1.06	0.69	1.62	7.8E-01	5.4E-01	1.04	0.71	1.53	8.2E-01	7.2E-01
94	CCL26	6	Inverse variance weighted	0.94	0.86	1.02	1.3E-01	2.4E-01	0.99	0.87	1.12	8.5E-01	5.2E-01	0.78	0.65	0.93	5.8E-03	4.8E-01	0.97	0.73	1.28	8.3E-01	2.6E-01	0.91	0.64	1.3	6.1E-01	8.7E-01
94	CCL26	6	Weighted median	0.92	0.82	1.02	1.2E-01	2.4E-01	0.97	0.87	1.09	6.5E-01	5.2E-01	0.77	0.62	0.97	2.4E-02	4.8E-01	0.93	0.68	1.26	6.3E-01	2.6E-01	0.91	0.66	1.24	5.5E-01	8.7E-01
94	CCL26	6	Weighted mode	0.91	0.8	1.03	1.1E-01	2.4E-01	0.98	0.85	1.13	6.8E-01	5.2E-01	0.75	0.54	1.03	6.9E-02	4.8E-01	0.9	0.61	1.32	5.2E-01	2.6E-01	0.89	0.61	1.32	4.9E-01	8.7E-01
94	CCL26	6	MR Egger	0.88	0.74	1.05	1.1E-01	2.4E-01	0.94	0.72	1.24	5.7E-01	5.2E-01	0.72	0.51	1.03	6.4E-02	4.8E-01	0.82	0.49	1.37	3.4E-01	2.6E-01	0.94	0.43	2.05	8.5E-01	8.7E-01
95	SDC4	18	Inverse variance weighted	1.13	1	1.27	4.3E-02	9.9E-01	1.15	1.01	1.31	2.9E-02	7.0E-01	1.06	0.86	1.31	5.9E-01	6.1E-01	1.19	0.91	1.55	2.0E-01	8.2E-02	1.02	0.74	1.41	8.9E-01	5.1E-01
95	SDC4	18	Weighted median	1.15	1.01	1.32	3.7E-02	9.9E-01	1.23	1.06	1.43	6.4E-03	7.0E-01	0.93	0.7	1.22	5.9E-01	6.1E-01	1.13	0.78	1.64	5.1E-01	8.2E-02	0.95	0.64	1.4	7.9E-01	5.1E-01

95	SDC4	18	Weighted mode	1.19	0.95	1.49	1.2E-01	9.9E-01	1.3	1.06	1.59	1.6E-02	7.0E-01	0.83	0.51	1.37	4.5E-01	6.1E-01	0.9	0.51	1.59	7.1E-01	8.2E-02	1.3	0.61	2.75	4.7E-01	5.1E-01
95	SDC4	18	MR Egger	1.13	0.76	1.67	5.3E-01	9.9E-01	1.24	0.81	1.89	3.0E-01	7.0E-01	0.9	0.44	1.82	7.5E-01	6.1E-01	0.59	0.25	1.38	2.1E-01	8.2E-02	1.42	0.48	4.15	5.0E-01	5.1E-01
96	NECTIN2	4	Inverse variance weighted	0.94	0.85	1.04	2.4E-01	6.7E-01	1.02	0.91	1.15	7.2E-01	4.1E-01	0.73	0.58	0.92	6.4E-03	5.9E-01	0.95	0.49	1.86	8.9E-01	5.6E-01	0.9	0.66	1.23	5.0E-01	7.6E-01
96	NECTIN2	4	Weighted median	0.99	0.87	1.12	8.9E-01	6.7E-01	1.04	0.91	1.18	6.1E-01	4.1E-01	0.79	0.6	1.03	7.6E-02	5.9E-01	0.93	0.58	1.5	7.6E-01	5.6E-01	0.9	0.64	1.27	5.5E-01	7.6E-01
96	NECTIN2	4	Weighted mode	1	0.79	1.26	1.0E+00	6.7E-01	1.04	0.83	1.29	6.5E-01	4.1E-01	0.77	0.5	1.19	1.6E-01	5.9E-01	1.03	0.5	2.13	9.1E-01	5.6E-01	0.96	0.5	1.85	8.5E-01	7.6E-01
96	NECTIN2	4	MR Egger	1.02	0.48	2.18	9.2E-01	6.7E-01	1.25	0.52	3.01	3.9E-01	4.1E-01	0.92	0.18	4.73	8.5E-01	5.9E-01	0.43	0	79	5.6E-01	5.6E-01	0.75	0.08	7.08	6.4E-01	7.6E-01
97	SMAD5	2	Inverse variance weighted	0.84	0.72	0.97	1.9E-02	NA	0.79	0.66	0.93	6.4E-03	NA	1.03	0.74	1.43	8.6E-01	NA	0.96	0.61	1.52	8.7E-01	NA	1.02	0.36	2.95	9.6E-01	NA
98	GIPC2	1	Wald ratio	1.09	0.98	1.2	1.2E-01	NA	1.11	0.99	1.26	7.7E-02	NA	1.04	0.83	1.3	7.2E-01	NA	0.78	0.57	1.06	1.1E-01	NA	1.55	1.13	2.12	6.5E-03	NA
99	CTSL	12	Inverse variance weighted	0.88	0.79	0.99	3.2E-02	9.8E-01	**0.83**	**0.73**	**0.95**	**6.5E-03**	7.1E-01	0.91	0.72	1.16	4.6E-01	2.3E-01	1.24	0.88	1.74	2.2E-01	8.6E-01	0.94	0.65	1.34	7.2E-01	4.7E-02
99	CTSL	12	Weighted median	0.91	0.79	1.05	2.1E-01	9.8E-01	**0.81**	**0.67**	**0.97**	**2.5E-02**	7.1E-01	0.85	0.62	1.18	3.4E-01	2.3E-01	1.13	0.74	1.74	5.7E-01	8.6E-01	1.14	0.72	1.82	5.7E-01	4.7E-02
99	CTSL	12	Weighted mode	0.86	0.67	1.12	2.4E-01	9.8E-01	0.78	0.57	1.06	1.1E-01	7.1E-01	0.88	0.52	1.5	6.1E-01	2.3E-01	1.02	0.49	2.13	9.5E-01	8.6E-01	1.29	0.54	3.05	5.3E-01	4.7E-02
99	CTSL	12	MR Egger	0.88	0.59	1.32	5.0E-01	9.8E-01	0.77	0.49	1.23	2.5E-01	7.1E-01	0.57	0.24	1.35	1.8E-01	2.3E-01	1.13	0.34	3.73	8.2E-01	8.6E-01	2.82	0.89	8.91	7.3E-02	4.7E-02
100	EFNB2	1	Wald ratio	0.79	0.66	0.94	6.7E-03	NA	0.76	0.62	0.93	7.1E-03	NA	1.13	0.77	1.65	5.4E-01	NA	0.81	0.48	1.37	4.2E-01	NA	0.69	0.41	1.16	1.7E-01	NA
101	SEPTIN3	9	Inverse variance weighted	1.08	0.97	1.2	1.6E-01	8.5E-01	1.1	0.94	1.29	2.4E-01	8.6E-01	1.17	0.97	1.41	1.1E-01	5.9E-01	1.04	0.8	1.36	7.6E-01	9.9E-01	1.07	0.8	1.43	6.7E-01	1.0E+00
101	SEPTIN3	9	Weighted median	1.13	1.02	1.25	2.2E-02	8.5E-01	1.18	1.05	1.32	6.7E-03	8.6E-01	1.15	0.92	1.43	2.1E-01	5.9E-01	1.13	0.83	1.54	4.3E-01	9.9E-01	1.06	0.77	1.47	7.0E-01	1.0E+00
101	SEPTIN3	9	Weighted mode	1.11	0.98	1.25	1.0E-01	8.5E-01	1.17	1.02	1.36	3.4E-02	8.6E-01	1.14	0.87	1.5	3.0E-01	5.9E-01	1.13	0.77	1.68	4.8E-01	9.9E-01	1.06	0.71	1.57	7.5E-01	1.0E+00
101	SEPTIN3	9	MR Egger	1.05	0.78	1.42	6.9E-01	8.5E-01	1.07	0.68	1.67	7.5E-01	8.6E-01	1.05	0.64	1.73	8.2E-01	5.9E-01	1.04	0.5	2.16	9.0E-01	9.9E-01	1.07	0.47	2.44	8.6E-01	1.0E+00
102	KLK1	13	Inverse variance weighted	1.03	1	1.06	5.6E-02	4.6E-01	1	0.97	1.03	9.2E-01	1.1E-01	1.06	0.98	1.16	1.6E-01	2.1E-01	1.13	1.03	1.23	6.9E-03	3.2E-01	1.04	0.92	1.18	5.0E-01	4.2E-01
102	KLK1	13	Weighted median	1.03	1	1.06	7.4E-02	4.6E-01	0.99	0.96	1.03	7.0E-01	1.1E-01	1.07	1.01	1.14	3.1E-02	2.1E-01	1.12	1.03	1.22	1.1E-02	3.2E-01	1.06	0.97	1.16	1.8E-01	4.2E-01
102	KLK1	13	Weighted mode	1.02	0.99	1.06	1.2E-01	4.6E-01	0.99	0.96	1.03	7.2E-01	1.1E-01	1.07	1.01	1.15	3.3E-02	2.1E-01	1.11	1.01	1.23	3.5E-02	3.2E-01	1.06	0.96	1.17	2.3E-01	4.2E-01
102	KLK1	13	MR Egger	1.02	0.98	1.06	2.2E-01	4.6E-01	0.98	0.94	1.02	3.7E-01	1.1E-01	1.1	0.99	1.22	8.2E-02	2.1E-01	1.1	0.98	1.23	9.3E-02	3.2E-01	1.07	0.91	1.26	3.5E-01	4.2E-01
103	FURIN	3	Inverse variance weighted	0.84	0.75	0.95	6.9E-03	7.7E-01	0.86	0.75	0.99	3.6E-02	6.5E-01	0.86	0.66	1.12	2.6E-01	7.6E-01	0.69	0.47	1	5.2E-02	4.4E-01	0.96	0.66	1.38	8.2E-01	6.6E-01
103	FURIN	3	Weighted median	0.84	0.74	0.96	7.8E-03	7.7E-01	0.87	0.75	1	5.0E-02	6.5E-01	0.86	0.65	1.14	2.9E-01	7.6E-01	0.69	0.46	1.02	6.4E-02	4.4E-01	0.91	0.62	1.33	6.3E-01	6.6E-01
103	FURIN	3	Weighted mode	0.83	0.6	1.16	1.4E-01	7.7E-01	0.87	0.62	1.23	2.3E-01	6.5E-01	0.83	0.43	1.59	3.4E-01	7.6E-01	0.59	0.22	1.55	1.4E-01	4.4E-01	0.91	0.39	2.13	6.8E-01	6.6E-01
103	FURIN	3	MR Egger	0.79	0.08	8.11	4.2E-01	7.7E-01	0.97	0.07	14.16	9.0E-01	6.5E-01	0.74	0	114.96	5.9E-01	7.6E-01	0.36	0	430.19	3.2E-01	4.4E-01	0.7	0	770.8	6.4E-01	6.6E-01
104	MUC2	6	Inverse variance weighted	1.05	1	1.09	3.5E-02	9.4E-01	1	0.95	1.05	9.4E-01	3.0E-01	1.09	1	1.19	6.0E-02	1.5E-01	1.15	0.96	1.37	1.4E-01	8.0E-02	1.11	0.98	1.26	8.5E-02	3.9E-01
104	MUC2	6	Weighted median	1.05	1	1.09	4.7E-02	9.4E-01	1	0.95	1.05	9.2E-01	3.0E-01	1.1	1.01	1.21	3.3E-02	1.5E-01	1.19	1.05	1.35	7.0E-03	8.0E-02	1.09	0.96	1.24	1.7E-01	3.9E-01
104	MUC2	6	Weighted mode	1.04	0.99	1.1	9.7E-02	9.4E-01	0.99	0.93	1.06	8.0E-01	3.0E-01	1.11	0.99	1.25	7.2E-02	1.5E-01	1.19	1	1.41	4.8E-02	8.0E-02	1.09	0.92	1.28	2.5E-01	3.9E-01
104	MUC2	6	MR Egger	1.05	0.96	1.15	2.3E-01	9.4E-01	0.97	0.87	1.08	4.4E-01	3.0E-01	1.2	0.99	1.46	6.1E-02	1.5E-01	1.37	1.03	1.83	3.7E-02	8.0E-02	1.04	0.79	1.36	7.2E-01	3.9E-01
105	UROD	5	Inverse variance weighted	0.95	0.89	1	7.1E-02	5.1E-01	0.91	0.85	0.97	7.1E-03	5.7E-01	0.98	0.86	1.12	7.7E-01	3.6E-01	0.93	0.78	1.11	4.2E-01	9.2E-01	1.13	0.95	1.36	1.7E-01	4.9E-01
105	UROD	5	Weighted median	0.95	0.89	1.01	8.2E-02	5.1E-01	0.91	0.85	0.98	9.6E-03	5.7E-01	0.96	0.85	1.1	5.8E-01	3.6E-01	0.92	0.76	1.12	4.1E-01	9.2E-01	1.12	0.93	1.35	2.2E-01	4.9E-01
105	UROD	5	Weighted mode	0.95	0.87	1.03	1.5E-01	5.1E-01	0.91	0.82	1.02	7.9E-02	5.7E-01	0.96	0.79	1.17	6.0E-01	3.6E-01	0.92	0.71	1.2	4.3E-01	9.2E-01	1.12	0.84	1.49	3.3E-01	4.9E-01
105	UROD	5	MR Egger	0.97	0.84	1.11	5.2E-01	5.1E-01	0.93	0.79	1.09	2.5E-01	5.7E-01	0.91	0.67	1.23	4.0E-01	3.6E-01	0.92	0.6	1.4	5.7E-01	9.2E-01	1.22	0.8	1.88	2.3E-01	4.9E-01
106	SUGP1	1	Wald ratio	1.15	1.03	1.28	1.1E-02	NA	1.17	1.03	1.32	1.6E-02	NA	0.98	0.77	1.25	8.8E-01	NA	1.16	0.84	1.62	3.7E-01	NA	1.56	1.13	2.15	7.1E-03	NA
107	DSC2	6	Inverse variance weighted	0.97	0.88	1.06	4.7E-01	5.4E-01	0.98	0.88	1.09	7.0E-01	7.6E-01	0.95	0.77	1.16	6.1E-01	2.7E-01	0.73	0.47	1.13	1.6E-01	3.2E-01	0.86	0.64	1.15	3.0E-01	8.3E-01
107	DSC2	6	Weighted median	0.98	0.88	1.09	6.7E-01	5.4E-01	0.98	0.87	1.11	7.3E-01	7.6E-01	0.94	0.75	1.19	6.1E-01	2.7E-01	0.63	0.45	0.88	7.4E-03	3.2E-01	0.87	0.63	1.2	4.0E-01	8.3E-01
107	DSC2	6	Weighted mode	0.98	0.85	1.14	7.4E-01	5.4E-01	0.98	0.83	1.16	7.5E-01	7.6E-01	1.06	0.77	1.46	6.5E-01	2.7E-01	0.62	0.4	0.96	3.9E-02	3.2E-01	0.87	0.57	1.35	4.6E-01	8.3E-01
107	DSC2	6	MR Egger	1.02	0.79	1.32	8.5E-01	5.4E-01	1.01	0.75	1.35	9.4E-01	7.6E-01	1.18	0.68	2.06	4.6E-01	2.7E-01	0.49	0.16	1.55	1.6E-01	3.2E-01	0.91	0.39	2.14	7.8E-01	8.3E-01
108	GZMA	13	Inverse variance weighted	0.94	0.86	1.01	1.0E-01	7.7E-01	1	0.9	1.1	9.4E-01	7.5E-01	0.81	0.68	0.97	1.9E-02	4.0E-01	0.71	0.54	0.93	1.2E-02	2.1E-01	0.92	0.72	1.18	5.2E-01	3.2E-01
108	GZMA	13	Weighted median	0.92	0.83	1.02	1.1E-01	7.7E-01	0.99	0.89	1.1	7.9E-01	7.5E-01	0.76	0.62	0.93	7.4E-03	4.0E-01	0.68	0.51	0.91	1.0E-02	2.1E-01	1.05	0.78	1.4	7.6E-01	3.2E-01
108	GZMA	13	Weighted mode	0.92	0.82	1.03	1.2E-01	7.7E-01	0.98	0.87	1.1	7.0E-01	7.5E-01	0.77	0.6	0.98	3.4E-02	4.0E-01	0.69	0.5	0.96	2.9E-02	2.1E-01	1.06	0.76	1.47	7.2E-01	3.2E-01
108	GZMA	13	MR Egger	0.95	0.82	1.11	4.9E-01	7.7E-01	1.02	0.83	1.25	8.3E-01	7.5E-01	0.73	0.53	1.02	6.1E-02	4.0E-01	0.9	0.55	1.48	6.6E-01	2.1E-01	1.11	0.68	1.8	6.5E-01	3.2E-01
109	CXCL12	8	Inverse variance weighted	1.03	0.92	1.14	6.5E-01	3.4E-01	1.02	0.9	1.16	7.6E-01	6.3E-01	0.96	0.74	1.24	7.5E-01	7.7E-01	1.17	0.75	1.83	4.9E-01	9.8E-01	1.43	1.01	2.01	4.4E-02	3.2E-01
109	CXCL12	8	Weighted median	1.07	0.94	1.22	3.2E-01	3.4E-01	1.02	0.89	1.18	7.5E-01	6.3E-01	0.93	0.7	1.22	5.8E-01	7.7E-01	1.11	0.74	1.67	6.1E-01	9.8E-01	1.67	1.15	2.44	7.5E-03	3.2E-01
109	CXCL12	8	Weighted mode	1.06	0.9	1.25	4.2E-01	3.4E-01	1.03	0.85	1.25	7.5E-01	6.3E-01	0.92	0.64	1.32	5.9E-01	7.7E-01	1.1	0.67	1.81	6.7E-01	9.8E-01	1.63	1.01	2.64	4.7E-02	3.2E-01
109	CXCL12	8	MR Egger	1.13	0.86	1.49	3.0E-01	3.4E-01	1.08	0.79	1.48	5.8E-01	6.3E-01	0.89	0.45	1.77	6.9E-01	7.7E-01	1.18	0.35	3.95	7.5E-01	9.8E-01	1.98	0.85	4.62	9.7E-02	3.2E-01
110	CD200R1	9	Inverse variance weighted	1.01	0.97	1.04	7.0E-01	2.1E-01	1	0.96	1.04	9.4E-01	2.9E-01	1.01	0.93	1.08	9.0E-01	3.3E-01	0.98	0.89	1.08	7.2E-01	9.5E-01	1.13	1.03	1.24	7.5E-03	7.3E-01
110	CD200R1	9	Weighted median	1	0.96	1.03	8.5E-01	2.1E-01	1	0.96	1.03	8.1E-01	2.9E-01	1	0.94	1.08	9.2E-01	3.3E-01	0.99	0.89	1.1	8.0E-01	9.5E-01	1.13	1.02	1.25	1.5E-02	7.3E-01
110	CD200R1	9	Weighted mode	1	0.96	1.04	9.3E-01	2.1E-01	0.99	0.95	1.04	7.3E-01	2.9E-01	1	0.92	1.08	9.9E-01	3.3E-01	0.97	0.86	1.1	6.1E-01	9.5E-01	1.13	1	1.27	5.1E-02	7.3E-01
110	CD200R1	9	MR Egger	0.99	0.95	1.04	7.7E-01	2.1E-01	0.99	0.94	1.04	5.6E-01	2.9E-01	0.98	0.89	1.09	7.2E-01	3.3E-01	0.98	0.86	1.13	7.9E-01	9.5E-01	1.14	1.01	1.3	3.9E-02	7.3E-01
111	CNTN2	5	Inverse variance weighted	0.96	0.92	1	7.4E-02	1.3E-01	0.96	0.92	1.01	1.4E-01	1.2E-01	0.97	0.9	1.04	3.3E-01	9.1E-01	0.93	0.84	1.02	1.3E-01	8.4E-01	0.99	0.9	1.09	8.1E-01	9.7E-01
111	CNTN2	5	Weighted median	0.96	0.93	0.99	7.6E-03	1.3E-01	0.96	0.92	1	2.7E-02	1.2E-01	0.97	0.9	1.04	3.4E-01	9.1E-01	0.93	0.84	1.02	1.2E-01	8.4E-01	0.98	0.89	1.09	7.6E-01	9.7E-01
111	CNTN2	5	Weighted mode	0.96	0.91	1	6.3E-02	1.3E-01	0.96	0.91	1.01	8.7E-02	1.2E-01	0.96	0.87	1.07	3.7E-01	9.1E-01	0.93	0.8	1.07	2.1E-01	8.4E-01	0.98	0.86	1.13	7.5E-01	9.7E-01
111	CNTN2	5	MR Egger	0.94	0.88	1	5.4E-02	1.3E-01	0.94	0.87	1.01	6.7E-02	1.2E-01	0.96	0.82	1.13	5.0E-01	9.1E-01	0.93	0.77	1.14	3.4E-01	8.4E-01	0.99	0.81	1.21	8.5E-01	9.7E-01
112	JAM2	5	Inverse variance weighted	1.02	0.9	1.15	8.1E-01	7.4E-01	1.04	0.9	1.19	6.3E-01	7.0E-01	0.83	0.63	1.08	1.6E-01	6.4E-01	1	0.69	1.44	9.9E-01	8.3E-01	0.6	0.42	0.88	7.8E-03	4.9E-01
112	JAM2	5	Weighted median	1.02	0.89	1.16	8.3E-01	7.4E-01	1.03	0.88	1.2	7.2E-01	7.0E-01	0.78	0.57	1.07	1.2E-01	6.4E-01	1.01	0.68	1.52	9.4E-01	8.3E-01	0.62	0.4	0.95	2.9E-02	4.9E-01
112	JAM2	5	Weighted mode	1.02	0.82	1.26	8.4E-01	7.4E-01	1.03	0.82	1.28	7.4E-01	7.0E-01	0.78	0.46	1.31	2.5E-01	6.4E-01	1.03	0.55	1.93	9.0E-01	8.3E-01	0.63	0.33	1.22	1.2E-01	4.9E-01
112	JAM2	5	MR Egger	0.98	0.65	1.46	8.6E-01	7.4E-01	0.98	0.62	1.56	9.0E-01	7.0E-01	0.73	0.3	1.76	3.4E-01	6.4E-01	1.08	0.32	3.68	8.5E-01	8.3E-01	0.46	0.13	1.61	1.4E-01	4.9E-01
113	SIGLEC6	17	Inverse variance weighted	0.97	0.9	1.05	4.6E-01	6.4E-02	0.94	0.86	1.03	1.7E-01	3.1E-02	0.96	0.82	1.12	6.0E-01	5.0E-01	1.15	0.92	1.43	2.1E-01	7.2E-01	1.04	0.83	1.3	7.6E-01	7.3E-01
113	SIGLEC6	17	Weighted median	0.94	0.86	1.03	1.7E-01	6.4E-02	0.9	0.82	1	5.4E-02	3.1E-02	0.93	0.77	1.11	4.1E-01	5.0E-01	1.32	0.99	1.77	6.1E-02	7.2E-01	0.97	0.74	1.27	8.3E-01	7.3E-01
113	SIGLEC6	17	Weighted mode	0.92	0.84	1.01	6.9E-02	6.4E-02	0.85	0.77	0.95	7.9E-03	3.1E-02	0.93	0.76	1.13	4.3E-01	5.0E-01	1.34	0.95	1.89	9.2E-02	7.2E-01	0.97	0.72	1.31	8.6E-01	7.3E-01
113	SIGLEC6	17	MR Egger	0.92	0.83	1.01	7.4E-02	6.4E-02	0.87	0.77	0.97	1.5E-02	3.1E-02	0.91	0.72	1.15	4.1E-01	5.0E-01	1.2	0.86	1.67	2.7E-01	7.2E-01	1.07	0.77	1.5	6.6E-01	7.3E-01
114	AXL	6	Inverse variance weighted	1.04	0.94	1.16	4.1E-01	2.3E-01	1.01	0.88	1.15	9.0E-01	2.7E-01	1.25	0.89	1.75	2.0E-01	6.2E-01	1.04	0.77	1.41	8.0E-01	2.6E-01	1	0.74	1.36	9.8E-01	9.5E-01
114	AXL	6	Weighted median	1.02	0.9	1.15	7.6E-01	2.3E-01	0.98	0.85	1.12	7.6E-01	2.7E-01	1.51	1.11	2.04	8.2E-03	6.2E-01	0.92	0.63	1.34	6.5E-01	2.6E-01	0.99	0.68	1.46	9.8E-01	9.5E-01
114	AXL	6	Weighted mode	1.01	0.86	1.2	8.4E-01	2.3E-01	0.96	0.79	1.16	5.9E-01	2.7E-01	1.41	0.93	2.14	8.5E-02	6.2E-01	0.96	0.59	1.54	8.2E-01	2.6E-01	0.98	0.58	1.64	9.2E-01	9.5E-01
114	AXL	6	MR Egger	0.94	0.73	1.21	5.2E-01	2.3E-01	0.9	0.65	1.23	3.8E-01	2.7E-01	1.07	0.43	2.71	8.4E-01	6.2E-01	0.78	0.37	1.65	4.1E-01	2.6E-01	0.99	0.46	2.11	9.7E-01	9.5E-01
115	ACADSB	5	Inverse variance weighted	1	0.9	1.11	9.8E-01	4.3E-01	0.99	0.86	1.13	8.7E-01	5.9E-01	1.07	0.81	1.42	6.2E-01	8.0E-01	0.69	0.5	0.95	2.4E-02	2.7E-01	0.99	0.73	1.34	9.3E-01	2.5E-01
115	ACADSB	5	Weighted median	1.01	0.91	1.13	8.0E-01	4.3E-01	1	0.87	1.15	9.9E-01	5.9E-01	1.08	0.85	1.38	5.3E-01	8.0E-01	0.61	0.42	0.88	8.2E-03	2.7E-01	1.12	0.8	1.58	5.1E-01	2.5E-01
115	ACADSB	5	Weighted mode	1.02	0.87	1.21	7.1E-01	4.3E-01	1.01	0.82	1.24	9.4E-01	5.9E-01	1.07	0.75	1.53	6.2E-01	8.0E-01	0.59	0.35	1.01	5.4E-02	2.7E-01	1.16	0.7	1.92	4.7E-01	2.5E-01
115	ACADSB	5	MR Egger	1.06	0.82	1.37	5.3E-01	4.3E-01	1.04	0.72	1.52	7.4E-01	5.9E-01	1.02	0.46	2.26	9.5E-01	8.0E-01	0.53	0.23	1.2	9.0E-02	2.7E-01	1.28	0.59	2.76	3.8E-01	2.5E-01
116	ELN	7	Inverse variance weighted	1.17	1	1.37	4.8E-02	7.1E-01	1.27	1.06	1.53	8.3E-03	7.5E-01	1.16	0.83	1.63	3.8E-01	5.6E-01	0.98	0.61	1.57	9.3E-01	9.5E-01	0.99	0.62	1.57	9.5E-01	4.9E-01
116	ELN	7	Weighted median	1.23	1.01	1.49	3.7E-02	7.1E-01	1.31	1.05	1.64	1.7E-02	7.5E-01	1.18	0.77	1.81	4.5E-01	5.6E-01	0.99	0.55	1.78	9.8E-01	9.5E-01	0.91	0.52	1.61	7.5E-01	4.9E-01
116	ELN	7	Weighted mode	1.24	0.9	1.71	1.6E-01	7.1E-01	1.36	0.94	1.97	8.7E-02	7.5E-01	1.27	0.54	3.01	5.1E-01	5.6E-01	0.78	0.28	2.18	5.8E-01	9.5E-01	0.87	0.34	2.25	7.4E-01	4.9E-01
116	ELN	7	MR Egger	1.1	0.7	1.72	6.0E-01	7.1E-01	1.2	0.72	2.01	4.1E-01	7.5E-01	1.43	0.54	3.78	3.9E-01	5.6E-01	0.95	0.25	3.65	9.2E-01	9.5E-01	0.7	0.18	2.67	5.3E-01	4.9E-01
117	EXTL1	1	Wald ratio	1.05	0.98	1.13	1.4E-01	NA	1.03	0.95	1.11	5.4E-01	NA	1.12	0.96	1.3	1.4E-01	NA	1.32	1.07	1.63	8.4E-03	NA	1.07	0.87	1.32	5.2E-01	NA
118	C1QTNF9	9	Inverse variance weighted	1.01	0.98	1.04	6.4E-01	7.8E-01	0.98	0.95	1.02	3.0E-01	2.2E-01	1.07	1.01	1.14	2.7E-02	7.9E-02	1.13	1.03	1.23	8.5E-03	3.4E-01	1.04	0.96	1.14	3.4E-01	3.1E-01
118	C1QTNF9	9	Weighted median	1.01	0.98	1.04	7.0E-01	7.8E-01	0.98	0.95	1.02	3.0E-01	2.2E-01	1.06	1	1.13	5.8E-02	7.9E-02	1.12	1.03	1.22	1.1E-02	3.4E-01	1.05	0.96	1.15	2.8E-01	3.1E-01
118	C1QTNF9	9	Weighted mode	1.01	0.97	1.04	7.4E-01	7.8E-01	0.98	0.94	1.02	3.6E-01	2.2E-01	1.07	0.99	1.15	9.0E-02	7.9E-02	1.11	1	1.24	4.5E-02	3.4E-01	1.05	0.94	1.17	3.2E-01	3.1E-01
118	C1QTNF9	9	MR Egger	1.01	0.97	1.05	6.0E-01	7.8E-01	0.99	0.95	1.04	7.5E-01	2.2E-01	1.04	0.95	1.13	3.5E-01	7.9E-02	1.1	0.97	1.24	1.1E-01	3.4E-01	1.07	0.95	1.21	2.2E-01	3.1E-01
119	GRK5	1	Wald ratio	1.02	0.92	1.13	7.6E-01	NA	1.01	0.9	1.14	8.7E-01	NA	0.94	0.75	1.17	5.7E-01	NA	1.55	1.12	2.14	8.5E-03	NA	0.99	0.73	1.35	9.7E-01	NA
120	LRCH4	4	Inverse variance weighted	0.76	0.61	0.93	8.7E-03	5.0E-01	0.78	0.61	1.01	5.5E-02	5.1E-01	0.88	0.56	1.39	5.9E-01	2.5E-01	0.47	0.25	0.89	2.0E-02	9.5E-01	0.52	0.28	0.97	4.1E-02	4.5E-01
120	LRCH4	4	Weighted median	0.76	0.59	0.97	2.5E-02	5.0E-01	0.8	0.6	1.06	1.2E-01	5.1E-01	0.86	0.51	1.47	5.8E-01	2.5E-01	0.49	0.23	1.04	6.3E-02	9.5E-01	0.65	0.31	1.38	2.7E-01	4.5E-01
120	LRCH4	4	Weighted mode	0.75	0.42	1.35	2.1E-01	5.0E-01	0.84	0.4	1.78	5.2E-01	5.1E-01	0.66	0.18	2.41	3.9E-01	2.5E-01	0.52	0.09	2.92	3.2E-01	9.5E-01	0.67	0.12	3.8	5.1E-01	4.5E-01
120	LRCH4	4	MR Egger	1.89	0.02	234.08	6.3E-01	5.0E-01	2.46	0	1267.18	6.0E-01	5.1E-01	42.98	0	1454770.06	2.6E-01	2.5E-01	0.6	0	1276409.6	8.9E-01	9.5E-01	0.02	0	45164.89	3.8E-01	4.5E-01
121	VSIG10	2	Inverse variance weighted	0.55	0.26	1.14	1.1E-01	NA	0.46	0.21	0.99	4.8E-02	NA	0.57	0.34	0.94	2.8E-02	NA	1.42	0.36	5.64	6.2E-01	NA	0.4	0.2	0.79	8.9E-03	NA
122	CD8A	4	Inverse variance weighted	1.02	0.93	1.13	6.4E-01	2.1E-01	1	0.88	1.14	9.6E-01	3.7E-01	1.01	0.87	1.17	9.3E-01	9.0E-01	0.93	0.75	1.14	4.9E-01	2.9E-01	1.35	1.03	1.78	3.2E-02	7.3E-01
122	CD8A	4	Weighted median	1.01	0.94	1.09	7.7E-01	2.1E-01	1.02	0.93	1.11	7.3E-01	3.7E-01	1.01	0.86	1.17	9.5E-01	9.0E-01	0.91	0.72	1.14	4.0E-01	2.9E-01	1.34	1.08	1.66	8.9E-03	7.3E-01
122	CD8A	4	Weighted mode	1.01	0.9	1.13	7.9E-01	2.1E-01	1.01	0.88	1.16	8.5E-01	3.7E-01	1	0.77	1.31	9.7E-01	9.0E-01	0.91	0.64	1.28	4.2E-01	2.9E-01	1.33	0.96	1.85	6.9E-02	7.3E-01
122	CD8A	4	MR Egger	0.95	0.75	1.21	4.5E-01	2.1E-01	0.93	0.63	1.37	5.0E-01	3.7E-01	1	0.61	1.61	9.7E-01	9.0E-01	0.79	0.4	1.55	2.7E-01	2.9E-01	1.25	0.43	3.62	4.6E-01	7.3E-01
123	CD207	12	Inverse variance weighted	1	0.94	1.08	9.2E-01	2.9E-01	0.95	0.89	1.02	1.8E-01	5.6E-01	1	0.86	1.16	9.5E-01	9.2E-01	1.16	0.94	1.44	1.7E-01	8.4E-01	**1.25**	**1.06**	**1.48**	**8.9E-03**	6.1E-01
123	CD207	12	Weighted median	0.98	0.92	1.04	5.2E-01	2.9E-01	0.95	0.88	1.01	1.1E-01	5.6E-01	1	0.88	1.14	9.7E-01	9.2E-01	1.14	0.96	1.36	1.4E-01	8.4E-01	**1.22**	**1.03**	**1.45**	**2.0E-02**	6.1E-01
123	CD207	12	Weighted mode	0.98	0.92	1.05	5.5E-01	2.9E-01	0.94	0.87	1.02	1.4E-01	5.6E-01	1	0.87	1.15	9.6E-01	9.2E-01	1.14	0.93	1.4	1.8E-01	8.4E-01	1.2	0.98	1.48	7.4E-02	6.1E-01
123	CD207	12	MR Egger	0.96	0.86	1.08	4.8E-01	2.9E-01	0.93	0.83	1.05	2.1E-01	5.6E-01	1	0.78	1.29	9.7E-01	9.2E-01	1.19	0.83	1.72	3.1E-01	8.4E-01	1.19	0.9	1.58	1.9E-01	6.1E-01
124	PRND	6	Inverse variance weighted	0.86	0.74	1	4.7E-02	3.2E-01	0.84	0.71	1	4.9E-02	4.5E-01	0.96	0.78	1.18	6.7E-01	1.9E-01	1	0.8	1.25	9.8E-01	3.8E-01	0.74	0.57	0.96	2.3E-02	1.1E-01
124	PRND	6	Weighted median	0.86	0.77	0.97	1.2E-02	3.2E-01	0.92	0.8	1.06	2.5E-01	4.5E-01	0.92	0.76	1.12	4.2E-01	1.9E-01	0.97	0.76	1.24	8.2E-01	3.8E-01	0.71	0.55	0.92	8.9E-03	1.1E-01
124	PRND	6	Weighted mode	0.93	0.76	1.13	3.7E-01	3.2E-01	0.94	0.71	1.25	6.2E-01	4.5E-01	0.9	0.7	1.15	3.1E-01	1.9E-01	0.96	0.69	1.34	7.8E-01	3.8E-01	0.68	0.49	0.93	2.6E-02	1.1E-01
124	PRND	6	MR Egger	0.77	0.54	1.08	1.0E-01	3.2E-01	0.76	0.5	1.16	1.5E-01	4.5E-01	0.78	0.5	1.21	1.9E-01	1.9E-01	0.86	0.5	1.48	4.8E-01	3.8E-01	0.54	0.32	0.91	3.1E-02	1.1E-01
125	LY75	5	Inverse variance weighted	1	0.97	1.02	7.7E-01	3.4E-01	1	0.97	1.03	9.0E-01	3.6E-01	0.92	0.87	0.98	9.1E-03	6.3E-01	0.99	0.91	1.08	7.9E-01	8.0E-01	0.97	0.85	1.09	5.8E-01	4.6E-01
125	LY75	5	Weighted median	1	0.97	1.03	8.2E-01	3.4E-01	1	0.97	1.03	8.5E-01	3.6E-01	0.92	0.87	0.98	1.1E-02	6.3E-01	0.99	0.91	1.08	8.2E-01	8.0E-01	0.97	0.89	1.05	4.5E-01	4.6E-01
125	LY75	5	Weighted mode	1	0.96	1.04	8.7E-01	3.4E-01	1	0.96	1.05	8.3E-01	3.6E-01	0.92	0.85	1.01	6.9E-02	6.3E-01	0.99	0.87	1.13	8.4E-01	8.0E-01	0.98	0.87	1.09	5.7E-01	4.6E-01
125	LY75	5	MR Egger	1.01	0.95	1.08	6.0E-01	3.4E-01	1.02	0.95	1.1	4.6E-01	3.6E-01	0.94	0.82	1.08	2.4E-01	6.3E-01	1	0.82	1.22	9.9E-01	8.0E-01	1.02	0.76	1.38	8.3E-01	4.6E-01
126	GBP2	1	Wald ratio	1.4	1.09	1.81	9.1E-03	NA	1.47	1.1	1.97	1.0E-02	NA	1.71	0.98	2.97	5.7E-02	NA	1.03	0.48	2.21	9.4E-01	NA	0.98	0.46	2.07	9.5E-01	NA
127	SIGLEC7	7	Inverse variance weighted	1.06	0.98	1.16	1.3E-01	1.6E-01	1.14	1.03	1.25	9.1E-03	4.0E-01	0.86	0.7	1.05	1.5E-01	1.7E-01	1.05	0.82	1.34	6.9E-01	2.3E-01	1.05	0.8	1.37	7.3E-01	8.6E-01
127	SIGLEC7	7	Weighted median	1.04	0.95	1.13	4.4E-01	1.6E-01	1.14	1.02	1.27	1.7E-02	4.0E-01	0.81	0.67	0.99	4.1E-02	1.7E-01	0.99	0.75	1.3	9.3E-01	2.3E-01	1.02	0.78	1.33	9.0E-01	8.6E-01
127	SIGLEC7	7	Weighted mode	1.04	0.92	1.17	4.9E-01	1.6E-01	1.14	0.97	1.33	9.6E-02	4.0E-01	0.81	0.65	1.03	7.2E-02	1.7E-01	0.98	0.7	1.37	8.8E-01	2.3E-01	1.01	0.73	1.4	9.4E-01	8.6E-01
127	SIGLEC7	7	MR Egger	1.01	0.88	1.16	8.5E-01	1.6E-01	1.1	0.94	1.29	1.9E-01	4.0E-01	0.77	0.57	1.04	7.5E-02	1.7E-01	0.93	0.62	1.38	6.5E-01	2.3E-01	1.03	0.64	1.66	8.9E-01	8.6E-01
128	DMP1	10	Inverse variance weighted	0.77	0.59	1	5.2E-02	8.6E-01	0.74	0.54	1.03	7.1E-02	8.2E-01	0.81	0.51	1.29	3.8E-01	8.5E-01	0.82	0.51	1.3	4.0E-01	6.8E-02	0.81	0.51	1.3	3.9E-01	7.6E-01
128	DMP1	10	Weighted median	0.81	0.66	0.98	3.3E-02	8.6E-01	0.75	0.61	0.93	9.2E-03	8.2E-01	0.88	0.58	1.35	5.7E-01	8.5E-01	0.76	0.44	1.32	3.4E-01	6.8E-02	0.95	0.56	1.61	8.4E-01	7.6E-01
128	DMP1	10	Weighted mode	0.81	0.64	1.02	7.3E-02	8.6E-01	0.73	0.55	0.97	3.5E-02	8.2E-01	0.94	0.51	1.74	8.2E-01	8.5E-01	0.64	0.27	1.52	2.7E-01	6.8E-02	1.07	0.51	2.24	8.4E-01	7.6E-01
128	DMP1	10	MR Egger	0.68	0.15	3.11	5.8E-01	8.6E-01	0.62	0.1	3.96	5.7E-01	8.2E-01	1.02	0.07	14.37	9.9E-01	8.5E-01	0.12	0.01	1.03	5.3E-02	6.8E-02	1.17	0.08	17.11	8.9E-01	7.6E-01
129	CA1	4	Inverse variance weighted	0.99	0.85	1.15	8.8E-01	6.0E-01	0.98	0.82	1.18	8.5E-01	8.2E-01	0.81	0.53	1.25	3.5E-01	8.6E-01	1.05	0.65	1.68	8.4E-01	4.0E-01	0.54	0.34	0.86	9.4E-03	9.5E-01
129	CA1	4	Weighted median	1.01	0.84	1.2	9.3E-01	6.0E-01	0.98	0.79	1.2	8.3E-01	8.2E-01	0.74	0.48	1.13	1.6E-01	8.6E-01	1.14	0.66	1.96	6.5E-01	4.0E-01	0.53	0.31	0.9	2.0E-02	9.5E-01
129	CA1	4	Weighted mode	1.03	0.7	1.51	8.2E-01	6.0E-01	0.97	0.65	1.45	8.3E-01	8.2E-01	0.68	0.27	1.72	2.8E-01	8.6E-01	1.22	0.41	3.64	6.0E-01	4.0E-01	0.52	0.18	1.51	1.5E-01	9.5E-01
129	CA1	4	MR Egger	0.79	0.17	3.72	5.9E-01	6.0E-01	0.88	0.15	5.27	8.0E-01	8.2E-01	0.64	0	116.5	7.5E-01	8.6E-01	3.28	0.03	375.25	3.9E-01	4.0E-01	0.58	0.01	59.21	6.6E-01	9.5E-01
130	KIR2DL2	3	Inverse variance weighted	1.02	0.97	1.07	3.8E-01	3.3E-01	0.99	0.93	1.05	7.8E-01	3.0E-01	1.11	1.01	1.21	2.9E-02	5.0E-01	1.16	1.04	1.3	9.4E-03	7.6E-01	1.01	0.9	1.13	8.9E-01	9.6E-01
130	KIR2DL2	3	Weighted median	1.02	0.98	1.06	3.0E-01	3.3E-01	0.99	0.95	1.03	6.0E-01	3.0E-01	1.1	1.02	1.19	1.7E-02	5.0E-01	1.16	1.03	1.3	1.1E-02	7.6E-01	1.01	0.9	1.13	8.9E-01	9.6E-01
130	KIR2DL2	3	Weighted mode	1.02	0.94	1.1	3.8E-01	3.3E-01	0.99	0.9	1.09	6.6E-01	3.0E-01	1.1	0.92	1.32	1.4E-01	5.0E-01	1.16	0.89	1.52	1.4E-01	7.6E-01	0.99	0.76	1.29	9.1E-01	9.6E-01
130	KIR2DL2	3	MR Egger	0.98	0.64	1.49	5.9E-01	3.3E-01	0.93	0.57	1.52	3.2E-01	3.0E-01	1.04	0.37	2.88	7.4E-01	5.0E-01	1.12	0.29	4.34	4.7E-01	7.6E-01	1	0.28	3.57	9.8E-01	9.6E-01
131	DTX3	3	Inverse variance weighted	0.88	0.77	1	4.3E-02	9.6E-01	0.9	0.78	1.04	1.5E-01	8.8E-01	0.7	0.53	0.92	9.5E-03	8.5E-01	0.84	0.58	1.23	3.8E-01	5.5E-01	0.88	0.56	1.38	5.8E-01	9.4E-01
131	DTX3	3	Weighted median	0.88	0.78	1	5.8E-02	9.6E-01	0.91	0.78	1.06	2.3E-01	8.8E-01	0.7	0.52	0.93	1.3E-02	8.5E-01	0.83	0.55	1.23	3.5E-01	5.5E-01	0.92	0.64	1.34	6.8E-01	9.4E-01
131	DTX3	3	Weighted mode	0.88	0.66	1.19	2.1E-01	9.6E-01	0.91	0.64	1.29	3.6E-01	8.8E-01	0.69	0.35	1.39	1.5E-01	8.5E-01	0.82	0.3	2.28	5.0E-01	5.5E-01	0.93	0.39	2.23	7.6E-01	9.4E-01
131	DTX3	3	MR Egger	0.88	0.16	5.03	5.3E-01	9.6E-01	0.92	0.12	6.97	7.0E-01	8.8E-01	0.74	0.02	28.75	4.9E-01	8.5E-01	0.63	0	102.52	4.5E-01	5.5E-01	0.83	0	4462.42	8.3E-01	9.4E-01
132	EDIL3	2	Inverse variance weighted	1.27	1.02	1.59	3.3E-02	NA	1.4	1.09	1.81	9.5E-03	NA	1.08	0.64	1.85	7.7E-01	NA	0.67	0.34	1.34	2.6E-01	NA	2.11	1.12	3.99	2.1E-02	NA
133	GOLM2	5	Inverse variance weighted	0.86	0.77	0.96	1.0E-02	3.1E-01	0.87	0.75	1.01	7.7E-02	1.9E-01	0.88	0.68	1.13	3.1E-01	6.7E-01	0.92	0.61	1.39	7.0E-01	4.9E-01	0.99	0.7	1.39	9.3E-01	5.5E-01
133	GOLM2	5	Weighted median	0.85	0.75	0.96	9.5E-03	3.1E-01	0.85	0.73	0.99	3.9E-02	1.9E-01	0.8	0.6	1.06	1.2E-01	6.7E-01	0.92	0.62	1.38	7.0E-01	4.9E-01	0.93	0.63	1.36	7.1E-01	5.5E-01
133	GOLM2	5	Weighted mode	0.86	0.71	1.05	9.9E-02	3.1E-01	0.83	0.66	1.06	1.0E-01	1.9E-01	0.79	0.52	1.2	1.9E-01	6.7E-01	0.99	0.53	1.83	9.6E-01	4.9E-01	0.94	0.52	1.69	7.7E-01	5.5E-01
133	GOLM2	5	MR Egger	1.05	0.61	1.81	8.1E-01	3.1E-01	1.2	0.64	2.24	4.3E-01	1.9E-01	0.73	0.19	2.73	5.0E-01	6.7E-01	0.57	0.07	4.52	4.5E-01	4.9E-01	0.71	0.13	3.72	5.6E-01	5.5E-01
134	CXCL16	10	Inverse variance weighted	1.09	0.83	1.43	5.4E-01	5.6E-02	1.11	0.8	1.53	5.5E-01	6.0E-02	1.18	0.88	1.58	2.8E-01	4.4E-02	1.02	0.77	1.36	8.7E-01	3.5E-01	0.97	0.73	1.28	8.2E-01	8.6E-01
134	CXCL16	10	Weighted median	1.11	0.95	1.31	2.0E-01	5.6E-02	1.14	0.94	1.39	1.8E-01	6.0E-02	1.41	1.06	1.88	1.8E-02	4.4E-02	0.98	0.66	1.44	9.0E-01	3.5E-01	0.96	0.68	1.37	8.4E-01	8.6E-01
134	CXCL16	10	Weighted mode	1.02	0.78	1.33	8.9E-01	5.6E-02	1.53	1.14	2.06	9.6E-03	6.0E-02	1.46	1.03	2.08	3.7E-02	4.4E-02	0.96	0.59	1.57	8.7E-01	3.5E-01	1.04	0.62	1.74	8.8E-01	8.6E-01
134	CXCL16	10	MR Egger	1.73	1	2.98	4.9E-02	5.6E-02	1.92	0.99	3.73	5.4E-02	6.0E-02	1.98	1.11	3.51	2.6E-02	4.4E-02	0.79	0.4	1.57	4.5E-01	3.5E-01	1.02	0.51	2.02	9.6E-01	8.6E-01
135	PRSS27	17	Inverse variance weighted	1.04	0.97	1.12	2.9E-01	9.9E-01	1	0.92	1.09	1.0E+00	8.0E-01	1.12	1.03	1.23	9.7E-03	5.8E-01	1.11	0.99	1.25	6.7E-02	4.4E-01	1.1	0.95	1.28	2.0E-01	9.2E-01
135	PRSS27	17	Weighted median	1.04	0.99	1.09	8.8E-02	9.9E-01	0.99	0.94	1.05	7.6E-01	8.0E-01	1.13	1.02	1.24	1.4E-02	5.8E-01	1.13	0.99	1.3	7.0E-02	4.4E-01	1.1	0.96	1.25	1.6E-01	9.2E-01
135	PRSS27	17	Weighted mode	1.04	0.99	1.09	1.1E-01	9.9E-01	1	0.95	1.05	8.5E-01	8.0E-01	1.12	1.02	1.24	2.4E-02	5.8E-01	1.17	1.01	1.36	4.3E-02	4.4E-01	1.09	0.96	1.24	1.7E-01	9.2E-01
135	PRSS27	17	MR Egger	1.04	0.93	1.16	4.6E-01	9.9E-01	0.99	0.87	1.13	8.6E-01	8.0E-01	1.15	1.01	1.31	3.7E-02	5.8E-01	1.16	0.98	1.37	7.5E-02	4.4E-01	1.09	0.88	1.36	4.0E-01	9.2E-01
136	NOS3	2	Inverse variance weighted	1.25	0.59	2.63	5.7E-01	NA	1.11	0.4	3.07	8.4E-01	NA	1.47	0.75	2.87	2.6E-01	NA	1.42	0.56	3.62	4.6E-01	NA	3.42	1.34	8.7	9.8E-03	NA
137	CD101	6	Inverse variance weighted	1.06	1.01	1.1	1.4E-02	6.9E-01	1.06	1.01	1.11	3.0E-02	8.5E-01	1.02	0.92	1.13	7.4E-01	5.0E-01	1.09	0.88	1.35	4.2E-01	7.2E-01	1.07	0.91	1.25	4.1E-01	5.6E-01
137	CD101	6	Weighted median	1.06	1.01	1.11	1.0E-02	6.9E-01	1.06	1.01	1.12	3.1E-02	8.5E-01	1.02	0.92	1.12	7.5E-01	5.0E-01	1.1	0.96	1.27	1.7E-01	7.2E-01	1.08	0.95	1.24	2.4E-01	5.6E-01
137	CD101	6	Weighted mode	1.06	1	1.13	5.5E-02	6.9E-01	1.06	0.99	1.13	8.1E-02	8.5E-01	1.01	0.88	1.15	9.2E-01	5.0E-01	1.11	0.92	1.32	2.1E-01	7.2E-01	1.1	0.93	1.31	2.0E-01	5.6E-01
137	CD101	6	MR Egger	1.06	0.99	1.14	7.6E-02	6.9E-01	1.06	0.98	1.15	1.1E-01	8.5E-01	1	0.84	1.18	9.6E-01	5.0E-01	1.12	0.77	1.62	4.6E-01	7.2E-01	1.1	0.84	1.44	3.8E-01	5.6E-01
138	ADAM23	5	Inverse variance weighted	1	0.95	1.05	9.2E-01	1.7E-01	1.01	0.96	1.07	6.4E-01	2.3E-01	0.99	0.89	1.1	8.6E-01	6.5E-01	0.89	0.77	1.04	1.6E-01	8.1E-01	0.81	0.7	0.95	1.0E-02	6.8E-01
138	ADAM23	5	Weighted median	0.99	0.94	1.05	7.8E-01	1.7E-01	1.01	0.95	1.07	8.3E-01	2.3E-01	0.99	0.89	1.11	8.6E-01	6.5E-01	0.89	0.76	1.04	1.5E-01	8.1E-01	0.82	0.7	0.96	1.4E-02	6.8E-01
138	ADAM23	5	Weighted mode	0.99	0.92	1.07	7.6E-01	1.7E-01	1.01	0.93	1.09	8.1E-01	2.3E-01	0.99	0.85	1.16	8.7E-01	6.5E-01	0.89	0.71	1.11	2.1E-01	8.1E-01	0.82	0.65	1.03	7.3E-02	6.8E-01
138	ADAM23	5	MR Egger	0.96	0.87	1.07	3.2E-01	1.7E-01	0.98	0.87	1.1	6.1E-01	2.3E-01	0.97	0.77	1.21	6.8E-01	6.5E-01	0.88	0.64	1.21	2.9E-01	8.1E-01	0.84	0.59	1.2	2.2E-01	6.8E-01
139	CASP8	3	Inverse variance weighted	0.86	0.77	0.97	1.3E-02	7.8E-01	0.85	0.75	0.98	2.0E-02	7.9E-01	1.02	0.67	1.55	9.4E-01	5.4E-01	0.89	0.63	1.26	5.3E-01	7.2E-01	0.89	0.62	1.28	5.3E-01	7.5E-01
139	CASP8	3	Weighted median	0.86	0.76	0.96	1.0E-02	7.8E-01	0.85	0.74	0.98	2.2E-02	7.9E-01	0.95	0.74	1.22	7.0E-01	5.4E-01	0.92	0.64	1.33	6.5E-01	7.2E-01	0.86	0.61	1.22	4.0E-01	7.5E-01
139	CASP8	3	Weighted mode	0.86	0.64	1.14	1.4E-01	7.8E-01	0.85	0.62	1.15	1.4E-01	7.9E-01	0.95	0.51	1.75	7.5E-01	5.4E-01	0.93	0.4	2.13	7.3E-01	7.2E-01	0.85	0.39	1.85	4.7E-01	7.5E-01
139	CASP8	3	MR Egger	0.84	0.28	2.52	3.0E-01	7.8E-01	0.83	0.23	2.96	3.2E-01	7.9E-01	0.82	0.01	55.41	6.5E-01	5.4E-01	0.98	0.04	26.36	9.5E-01	7.2E-01	0.8	0.01	69.44	6.4E-01	7.5E-01
140	C1QTNF5	10	Inverse variance weighted	0.99	0.93	1.06	7.7E-01	7.0E-01	1.01	0.92	1.12	7.7E-01	7.2E-02	0.87	0.75	1.02	8.7E-02	8.9E-02	1.09	0.9	1.31	3.9E-01	9.0E-01	1.09	0.86	1.38	4.6E-01	8.4E-02
140	C1QTNF5	10	Weighted median	0.99	0.92	1.07	8.4E-01	7.0E-01	1.04	0.96	1.13	3.1E-01	7.2E-02	0.79	0.67	0.95	1.0E-02	8.9E-02	1.06	0.86	1.29	6.0E-01	9.0E-01	1.01	0.82	1.25	9.0E-01	8.4E-02
140	C1QTNF5	10	Weighted mode	0.99	0.91	1.08	8.8E-01	7.0E-01	1.05	0.95	1.15	3.0E-01	7.2E-02	0.8	0.66	0.97	3.0E-02	8.9E-02	1.05	0.82	1.35	6.6E-01	9.0E-01	1	0.78	1.28	9.9E-01	8.4E-02
140	C1QTNF5	10	MR Egger	1	0.91	1.09	9.6E-01	7.0E-01	1.07	0.95	1.19	2.2E-01	7.2E-02	0.79	0.64	0.98	3.8E-02	8.9E-02	1.08	0.83	1.39	5.2E-01	9.0E-01	0.97	0.73	1.28	7.8E-01	8.4E-02
141	USP8	4	Inverse variance weighted	1.19	0.96	1.48	1.1E-01	8.8E-01	1.25	0.99	1.58	5.6E-02	4.8E-01	1.12	0.78	1.6	5.4E-01	6.8E-01	0.86	0.52	1.43	5.7E-01	5.3E-01	0.88	0.54	1.45	6.2E-01	9.6E-01
141	USP8	4	Weighted median	1.25	1.02	1.52	2.8E-02	8.8E-01	1.35	1.07	1.7	1.0E-02	4.8E-01	1.08	0.72	1.62	7.0E-01	6.8E-01	0.9	0.51	1.6	7.3E-01	5.3E-01	0.85	0.47	1.52	5.8E-01	9.6E-01
141	USP8	4	Weighted mode	1.24	0.81	1.89	2.0E-01	8.8E-01	1.37	0.89	2.11	1.0E-01	4.8E-01	1.05	0.49	2.28	8.5E-01	6.8E-01	0.99	0.32	3.07	9.7E-01	5.3E-01	0.83	0.26	2.58	6.3E-01	9.6E-01
141	USP8	4	MR Egger	1.25	0.33	4.78	5.5E-01	8.8E-01	1.57	0.46	5.42	2.6E-01	4.8E-01	0.93	0.15	5.84	8.8E-01	6.8E-01	1.3	0.1	17.03	7.0E-01	5.3E-01	0.91	0.07	11.2	8.9E-01	9.6E-01
142	PLAUR	7	Inverse variance weighted	0.95	0.84	1.06	3.5E-01	9.7E-01	0.97	0.85	1.11	7.1E-01	7.4E-01	0.71	0.55	0.92	1.0E-02	7.1E-01	0.92	0.65	1.29	6.2E-01	4.0E-01	1.03	0.66	1.6	9.1E-01	9.1E-01
142	PLAUR	7	Weighted median	0.96	0.82	1.11	5.6E-01	9.7E-01	0.98	0.83	1.16	8.4E-01	7.4E-01	0.8	0.56	1.13	2.0E-01	7.1E-01	1.02	0.66	1.57	9.4E-01	4.0E-01	0.94	0.6	1.45	7.7E-01	9.1E-01
142	PLAUR	7	Weighted mode	0.97	0.77	1.21	7.2E-01	9.7E-01	0.96	0.76	1.22	7.0E-01	7.4E-01	0.82	0.46	1.45	4.2E-01	7.1E-01	1.13	0.58	2.2	6.7E-01	4.0E-01	0.95	0.53	1.72	8.4E-01	9.1E-01
142	PLAUR	7	MR Egger	0.94	0.69	1.29	6.5E-01	9.7E-01	0.93	0.65	1.34	6.5E-01	7.4E-01	0.79	0.36	1.72	4.7E-01	7.1E-01	1.22	0.49	3.03	6.0E-01	4.0E-01	0.97	0.27	3.5	9.6E-01	9.1E-01
143	ALPI	4	Inverse variance weighted	0.97	0.92	1.02	2.1E-01	2.8E-01	1.01	0.95	1.07	6.8E-01	5.3E-01	0.9	0.81	1	5.0E-02	2.3E-01	0.85	0.75	0.96	1.0E-02	4.6E-01	0.93	0.81	1.07	3.2E-01	2.8E-01
143	ALPI	4	Weighted median	0.96	0.92	1.01	9.1E-02	2.8E-01	1.01	0.96	1.06	6.5E-01	5.3E-01	0.89	0.81	0.98	2.2E-02	2.3E-01	0.85	0.75	0.96	1.2E-02	4.6E-01	0.93	0.82	1.05	2.2E-01	2.8E-01
143	ALPI	4	Weighted mode	0.96	0.9	1.03	1.8E-01	2.8E-01	1.01	0.93	1.09	7.3E-01	5.3E-01	0.89	0.77	1.04	1.0E-01	2.3E-01	0.85	0.7	1.03	7.2E-02	4.6E-01	0.92	0.75	1.13	2.7E-01	2.8E-01
143	ALPI	4	MR Egger	0.92	0.78	1.09	1.8E-01	2.8E-01	0.98	0.78	1.24	7.5E-01	5.3E-01	0.81	0.58	1.13	1.1E-01	2.3E-01	0.92	0.58	1.45	5.0E-01	4.6E-01	0.82	0.52	1.3	2.1E-01	2.8E-01
144	PSAPL1	20	Inverse variance weighted	0.98	0.93	1.03	4.9E-01	4.8E-01	0.98	0.91	1.06	6.4E-01	3.4E-01	0.99	0.89	1.1	7.9E-01	7.9E-01	**0.84**	**0.73**	**0.96**	**1.0E-02**	3.3E-01	1.04	0.89	1.22	6.0E-01	6.2E-01
144	PSAPL1	20	Weighted median	0.99	0.94	1.05	8.0E-01	4.8E-01	1	0.94	1.07	9.8E-01	3.4E-01	0.98	0.87	1.09	6.6E-01	7.9E-01	0.9	0.77	1.06	2.1E-01	3.3E-01	1.04	0.89	1.21	6.4E-01	6.2E-01

144	PSAPL1	20	Weighted mode	0.99	0.94	1.05	7.5E-01	4.8E-01	1.01	0.94	1.08	8.0E-01	3.4E-01	0.98	0.87	1.1	7.2E-01	7.9E-01	0.88	0.75	1.04	1.4E-01	3.3E-01	1.06	0.89	1.24	5.0E-01	6.2E-01
144	PSAPL1	20	MR Egger	1	0.93	1.07	9.4E-01	4.8E-01	1.01	0.92	1.12	7.9E-01	3.4E-01	0.97	0.84	1.13	7.2E-01	7.9E-01	0.89	0.73	1.07	2.0E-01	3.3E-01	1.08	0.86	1.35	4.8E-01	6.2E-01
145	EREG	7	Inverse variance weighted	1.07	0.92	1.26	3.8E-01	1.8E-01	1.09	0.92	1.29	3.1E-01	3.4E-01	1.02	0.71	1.45	9.2E-01	1.1E-01	1.68	1.03	2.74	3.9E-02	3.1E-01	1.59	0.99	2.57	5.7E-02	9.5E-01
145	EREG	7	Weighted median	1.04	0.88	1.24	6.3E-01	1.8E-01	1.03	0.84	1.25	7.9E-01	3.4E-01	0.98	0.66	1.45	9.2E-01	1.1E-01	2	1.17	3.42	1.1E-02	3.1E-01	1.97	1.12	3.46	1.9E-02	9.5E-01
145	EREG	7	Weighted mode	1.06	0.81	1.38	6.4E-01	1.8E-01	0.96	0.69	1.34	7.7E-01	3.4E-01	0.8	0.4	1.57	4.4E-01	1.1E-01	2.07	0.84	5.11	9.6E-02	3.1E-01	2.08	0.73	5.9	1.4E-01	9.5E-01
145	EREG	7	MR Egger	0.69	0.33	1.45	2.6E-01	1.8E-01	0.77	0.33	1.83	4.7E-01	3.4E-01	0.33	0.07	1.56	1.3E-01	1.1E-01	0.57	0.05	7.18	5.9E-01	3.1E-01	1.7	0.11	26.96	6.4E-01	9.5E-01
146	PXDNL	1	Wald ratio	1.08	0.97	1.21	1.7E-01	NA	1.05	0.92	1.2	4.5E-01	NA	1.16	0.91	1.47	2.4E-01	NA	1.1	0.78	1.54	5.9E-01	NA	1.56	1.11	2.2	1.1E-02	NA
147	IL1RL2	10	Inverse variance weighted	0.93	0.88	0.98	1.1E-02	1.9E-01	0.94	0.88	1	4.4E-02	5.7E-01	0.99	0.86	1.14	9.0E-01	2.0E-01	0.91	0.75	1.11	3.6E-01	7.5E-02	0.81	0.69	0.96	1.5E-02	6.4E-01
147	IL1RL2	10	Weighted median	0.94	0.89	1	3.6E-02	1.9E-01	0.94	0.88	1.01	7.8E-02	5.7E-01	1.01	0.89	1.15	8.9E-01	2.0E-01	0.95	0.8	1.13	5.6E-01	7.5E-02	0.82	0.69	0.98	2.7E-02	6.4E-01
147	IL1RL2	10	Weighted mode	0.94	0.88	1	6.0E-02	1.9E-01	0.94	0.87	1.02	1.1E-01	5.7E-01	1.01	0.88	1.16	8.7E-01	2.0E-01	0.95	0.77	1.18	6.1E-01	7.5E-02	0.82	0.67	1.01	6.2E-02	6.4E-01
147	IL1RL2	10	MR Egger	0.96	0.88	1.05	3.6E-01	1.9E-01	0.95	0.86	1.05	2.9E-01	5.7E-01	1.08	0.88	1.32	4.4E-01	2.0E-01	1.06	0.82	1.39	6.0E-01	7.5E-02	0.84	0.65	1.09	1.7E-01	6.4E-01
148	NEB	1	Wald ratio	0.93	0.57	1.53	7.9E-01	NA	1.39	0.79	2.42	2.5E-01	NA	0.21	0.06	0.7	1.1E-02	NA	1.38	0.34	5.69	6.5E-01	NA	0.9	0.2	3.93	8.8E-01	NA
149	TMCO5A	1	Wald ratio	1.52	0.95	2.44	8.0E-02	NA	0.88	0.51	1.52	6.5E-01	NA	3.29	1.19	9.13	2.2E-02	NA	6.36	1.53	26.49	1.1E-02	NA	2.79	0.68	11.44	1.5E-01	NA
150	PCDH9	5	Inverse variance weighted	0.99	0.89	1.11	9.1E-01	9.4E-01	0.96	0.84	1.09	5.3E-01	6.9E-01	0.96	0.75	1.22	7.4E-01	3.1E-01	1.05	0.75	1.46	7.9E-01	6.4E-01	1.32	0.91	1.91	1.4E-01	1.1E-01
150	PCDH9	5	Weighted median	0.99	0.88	1.12	9.3E-01	9.4E-01	0.95	0.82	1.1	4.8E-01	6.9E-01	0.93	0.7	1.22	5.8E-01	3.1E-01	0.99	0.69	1.43	9.6E-01	6.4E-01	1.54	1.1	2.16	1.1E-02	1.1E-01
150	PCDH9	5	Weighted mode	0.99	0.83	1.19	9.3E-01	9.4E-01	0.94	0.78	1.15	4.6E-01	6.9E-01	0.91	0.62	1.36	5.6E-01	3.1E-01	0.98	0.58	1.68	9.3E-01	6.4E-01	1.54	0.93	2.53	7.4E-02	1.1E-01
150	PCDH9	5	MR Egger	0.99	0.77	1.28	9.0E-01	9.4E-01	0.93	0.7	1.25	5.1E-01	6.9E-01	0.83	0.48	1.44	3.6E-01	3.1E-01	0.96	0.45	2.04	8.7E-01	6.4E-01	1.89	0.92	3.91	6.7E-02	1.1E-01
151	HCG22	1	Wald ratio	0.96	0.9	1.03	2.7E-01	NA	0.97	0.9	1.05	5.0E-01	NA	1.01	0.87	1.17	9.1E-01	NA	0.76	0.62	0.94	1.2E-02	NA	1.12	0.91	1.38	2.7E-01	NA
152	SCARB2	5	Inverse variance weighted	1.02	0.88	1.19	7.8E-01	4.9E-01	0.99	0.81	1.2	9.0E-01	7.5E-01	1.34	1.07	1.67	1.2E-02	8.6E-01	1.14	0.84	1.56	4.0E-01	4.8E-01	0.99	0.64	1.54	9.7E-01	7.0E-01
152	SCARB2	5	Weighted median	1.01	0.9	1.13	8.6E-01	4.9E-01	0.99	0.87	1.12	8.3E-01	7.5E-01	1.34	1.06	1.7	1.5E-02	8.6E-01	1.13	0.82	1.57	4.6E-01	4.8E-01	1.1	0.79	1.52	5.8E-01	7.0E-01
152	SCARB2	5	Weighted mode	1.01	0.85	1.2	8.4E-01	4.9E-01	1.01	0.82	1.23	9.3E-01	7.5E-01	1.34	0.96	1.89	7.2E-02	8.6E-01	1.09	0.7	1.72	6.1E-01	4.8E-01	1.1	0.68	1.78	6.2E-01	7.0E-01
152	SCARB2	5	MR Egger	0.95	0.64	1.41	7.0E-01	4.9E-01	0.94	0.53	1.66	7.6E-01	7.5E-01	1.37	0.78	2.41	1.7E-01	8.6E-01	0.98	0.45	2.15	9.5E-01	4.8E-01	0.87	0.25	3.07	7.5E-01	7.0E-01
153	CRYBB2	11	Inverse variance weighted	0.99	0.91	1.07	7.6E-01	2.0E-01	1.04	0.95	1.13	4.5E-01	4.4E-01	0.96	0.81	1.14	6.5E-01	4.6E-01	0.72	0.56	0.93	1.2E-02	9.8E-01	0.96	0.76	1.22	7.6E-01	8.0E-01
153	CRYBB2	11	Weighted median	0.96	0.88	1.05	3.8E-01	2.0E-01	1.01	0.9	1.12	8.9E-01	4.4E-01	0.94	0.77	1.14	5.1E-01	4.6E-01	0.75	0.57	0.99	4.1E-02	9.8E-01	0.92	0.71	1.21	5.7E-01	8.0E-01
153	CRYBB2	11	Weighted mode	0.95	0.86	1.06	3.3E-01	2.0E-01	1	0.89	1.13	1.0E+00	4.4E-01	0.92	0.72	1.17	4.6E-01	4.6E-01	0.74	0.54	1.02	6.3E-02	9.8E-01	0.91	0.66	1.26	5.4E-01	8.0E-01
153	CRYBB2	11	MR Egger	0.93	0.81	1.07	2.5E-01	2.0E-01	0.99	0.84	1.16	9.0E-01	4.4E-01	0.89	0.66	1.2	4.0E-01	4.6E-01	0.72	0.45	1.15	1.5E-01	9.8E-01	0.93	0.61	1.41	7.0E-01	8.0E-01
154	KDM3A	3	Inverse variance weighted	1.01	0.81	1.25	9.5E-01	5.0E-01	0.98	0.76	1.26	8.6E-01	6.0E-01	1.14	0.64	2	6.6E-01	6.3E-01	0.42	0.21	0.82	1.2E-02	4.5E-01	1.26	0.53	3.02	6.0E-01	7.0E-01
154	KDM3A	3	Weighted median	0.99	0.78	1.25	9.1E-01	5.0E-01	0.98	0.75	1.29	8.8E-01	6.0E-01	1.38	0.79	2.4	2.5E-01	6.3E-01	0.41	0.19	0.85	1.7E-02	4.5E-01	1.56	0.73	3.33	2.5E-01	7.0E-01
154	KDM3A	3	Weighted mode	1.11	0.61	2.02	5.5E-01	5.0E-01	1.04	0.56	1.94	8.2E-01	6.0E-01	1.41	0.35	5.64	4.0E-01	6.3E-01	0.59	0.09	3.95	3.6E-01	4.5E-01	1.68	0.27	10.48	3.5E-01	7.0E-01
154	KDM3A	3	MR Egger	1.29	0.04	41.06	5.2E-01	5.0E-01	1.2	0.02	66.19	6.6E-01	6.0E-01	1.87	0	76802.18	5.9E-01	6.3E-01	1.04	0	48778.17	9.7E-01	4.5E-01	2.38	0	86282772.88	6.4E-01	7.0E-01
155	C1QA	12	Inverse variance weighted	0.98	0.92	1.05	5.9E-01	8.3E-01	0.99	0.92	1.06	6.9E-01	7.4E-01	1.07	0.91	1.27	4.2E-01	5.7E-01	0.79	0.65	0.95	1.2E-02	7.8E-01	1.01	0.83	1.22	9.3E-01	4.7E-01
155	C1QA	12	Weighted median	0.99	0.92	1.06	6.8E-01	8.3E-01	0.99	0.92	1.08	8.7E-01	7.4E-01	1.06	0.9	1.25	4.8E-01	5.7E-01	0.8	0.65	0.99	4.4E-02	7.8E-01	1.03	0.82	1.28	8.2E-01	4.7E-01
155	C1QA	12	Weighted mode	0.99	0.91	1.07	7.1E-01	8.3E-01	0.99	0.9	1.1	8.9E-01	7.4E-01	1.05	0.87	1.26	5.9E-01	5.7E-01	0.8	0.63	1.01	6.3E-02	7.8E-01	1.03	0.8	1.32	8.0E-01	4.7E-01
155	C1QA	12	MR Egger	0.99	0.89	1.11	8.3E-01	8.3E-01	1	0.89	1.12	9.6E-01	7.4E-01	1.02	0.78	1.34	8.8E-01	5.7E-01	0.81	0.6	1.08	1.3E-01	7.8E-01	1.08	0.8	1.46	5.7E-01	4.7E-01
156	DNAJB8	2	Inverse variance weighted	0.84	0.51	1.37	4.8E-01	NA	0.94	0.56	1.59	8.3E-01	NA	0.37	0.17	0.8	1.2E-02	NA	1.07	0.27	4.24	9.3E-01	NA	0.95	0.41	2.18	9.0E-01	NA
157	DKKL1	5	Inverse variance weighted	1	0.98	1.03	7.2E-01	4.0E-01	0.99	0.96	1.02	3.9E-01	9.6E-01	1	0.95	1.06	9.2E-01	2.3E-01	1.02	0.95	1.1	5.4E-01	9.8E-01	1.1	0.98	1.23	9.1E-02	2.7E-01
157	DKKL1	5	Weighted median	1	0.98	1.03	7.7E-01	4.0E-01	0.99	0.96	1.01	3.8E-01	9.6E-01	1	0.95	1.05	9.3E-01	2.3E-01	1.02	0.95	1.1	5.2E-01	9.8E-01	1.1	1.02	1.18	1.2E-02	2.7E-01
157	DKKL1	5	Weighted mode	1	0.97	1.04	7.5E-01	4.0E-01	0.99	0.95	1.02	4.1E-01	9.6E-01	0.99	0.92	1.07	8.5E-01	2.3E-01	1.03	0.93	1.13	5.1E-01	9.8E-01	1.09	0.98	1.21	9.2E-02	2.7E-01
157	DKKL1	5	MR Egger	1	0.95	1.04	7.9E-01	4.0E-01	0.99	0.93	1.04	5.3E-01	9.6E-01	0.97	0.88	1.08	4.7E-01	2.3E-01	1.02	0.88	1.18	6.7E-01	9.8E-01	1.04	0.85	1.28	5.5E-01	2.7E-01
158	ACY3	6	Inverse variance weighted	1.01	0.92	1.11	8.1E-01	2.7E-01	0.98	0.88	1.1	7.7E-01	6.4E-01	1.26	0.99	1.6	5.8E-02	3.5E-01	0.93	0.7	1.24	6.2E-01	4.9E-01	0.77	0.58	1.02	6.4E-02	6.8E-01
158	ACY3	6	Weighted median	1.03	0.93	1.14	5.8E-01	2.7E-01	1	0.89	1.12	9.4E-01	6.4E-01	1.31	1.06	1.62	1.2E-02	3.5E-01	0.95	0.71	1.27	7.2E-01	4.9E-01	0.79	0.58	1.07	1.3E-01	6.8E-01
158	ACY3	6	Weighted mode	1.04	0.91	1.2	4.8E-01	2.7E-01	1	0.85	1.17	9.8E-01	6.4E-01	1.33	1.01	1.77	4.6E-02	3.5E-01	1	0.68	1.48	9.8E-01	4.9E-01	0.79	0.52	1.21	2.2E-01	6.8E-01
158	ACY3	6	MR Egger	1.09	0.88	1.35	3.2E-01	2.7E-01	1.02	0.8	1.31	8.4E-01	6.4E-01	1.48	0.86	2.54	1.1E-01	3.5E-01	1.07	0.56	2.05	7.8E-01	4.9E-01	0.83	0.43	1.59	4.7E-01	6.8E-01
159	ACSL1	1	Wald ratio	0.59	0.39	0.89	1.2E-02	NA	0.6	0.37	0.97	3.6E-02	NA	0.52	0.21	1.3	1.6E-01	NA	0.85	0.24	3.02	8.1E-01	NA	0.29	0.08	1.02	5.4E-02	NA
160	LDLR	15	Inverse variance weighted	1.01	0.92	1.1	9.0E-01	1.3E-01	1.02	0.93	1.11	6.8E-01	2.5E-01	1.01	0.81	1.27	9.0E-01	1.3E-01	0.92	0.74	1.13	4.2E-01	6.4E-01	0.76	0.61	0.94	1.2E-02	2.6E-01
160	LDLR	15	Weighted median	0.98	0.89	1.07	6.5E-01	1.3E-01	1	0.9	1.11	9.8E-01	2.5E-01	0.92	0.75	1.13	4.4E-01	1.3E-01	0.9	0.68	1.2	4.9E-01	6.4E-01	0.77	0.58	1.02	6.9E-02	2.6E-01
160	LDLR	15	Weighted mode	0.98	0.89	1.08	6.7E-01	1.3E-01	0.99	0.87	1.13	8.7E-01	2.5E-01	0.91	0.73	1.14	4.0E-01	1.3E-01	0.9	0.65	1.26	5.2E-01	6.4E-01	0.76	0.57	1.02	6.4E-02	2.6E-01
160	LDLR	15	MR Egger	0.91	0.77	1.08	2.4E-01	1.3E-01	0.94	0.8	1.12	4.7E-01	2.5E-01	0.78	0.52	1.19	2.3E-01	1.3E-01	0.85	0.56	1.29	4.1E-01	6.4E-01	0.63	0.42	0.96	3.3E-02	2.6E-01
161	PPP1R14D	2	Inverse variance weighted	1.4	1.05	1.86	2.2E-02	NA	1.52	1.09	2.12	1.3E-02	NA	1.52	0.81	2.82	1.9E-01	NA	0.54	0.23	1.29	1.7E-01	NA	0.85	0.36	2.01	7.1E-01	NA
162	TLR1	2	Inverse variance weighted	1.06	1	1.13	3.6E-02	NA	1.08	1.01	1.16	2.2E-02	NA	1.1	0.97	1.25	1.4E-01	NA	1.07	0.89	1.27	4.8E-01	NA	0.8	0.66	0.95	1.3E-02	NA
163	TFPI	5	Inverse variance weighted	1.06	0.99	1.14	8.6E-02	4.1E-01	1.07	0.98	1.16	1.2E-01	2.4E-01	1.02	0.87	1.19	8.1E-01	7.7E-01	1.31	1.06	1.62	1.3E-02	9.1E-01	0.97	0.76	1.25	8.4E-01	3.5E-01
163	TFPI	5	Weighted median	1.09	1.01	1.17	3.2E-02	4.1E-01	1.09	1	1.18	4.9E-02	2.4E-01	1.01	0.86	1.19	9.0E-01	7.7E-01	1.3	1.03	1.63	2.5E-02	9.1E-01	1.01	0.8	1.27	9.5E-01	3.5E-01
163	TFPI	5	Weighted mode	1.08	0.97	1.2	1.2E-01	4.1E-01	1.08	0.94	1.24	1.8E-01	2.4E-01	1.01	0.79	1.29	9.1E-01	7.7E-01	1.33	0.94	1.88	8.2E-02	9.1E-01	1.05	0.75	1.45	7.3E-01	3.5E-01
163	TFPI	5	MR Egger	1.11	0.92	1.34	1.7E-01	4.1E-01	1.15	0.93	1.44	1.3E-01	2.4E-01	0.99	0.66	1.49	9.2E-01	7.7E-01	1.29	0.73	2.27	2.5E-01	9.1E-01	1.16	0.61	2.22	5.1E-01	3.5E-01
164	WFDC1	2	Inverse variance weighted	0.99	0.91	1.08	7.8E-01	NA	1.04	0.93	1.16	5.4E-01	NA	0.99	0.83	1.2	9.5E-01	NA	0.97	0.75	1.26	8.3E-01	NA	0.73	0.57	0.94	1.3E-02	NA
165	ERBIN	2	Inverse variance weighted	1.26	0.89	1.77	1.9E-01	NA	1.16	0.78	1.72	4.7E-01	NA	3.12	1.27	7.67	1.3E-02	NA	1.11	0.39	3.12	8.5E-01	NA	1.33	0.48	3.7	5.8E-01	NA
166	IGFBP3	5	Inverse variance weighted	0.94	0.89	0.99	2.6E-02	9.5E-01	0.92	0.87	0.98	1.3E-02	7.2E-01	0.92	0.82	1.04	1.8E-01	9.8E-01	0.99	0.83	1.16	8.6E-01	4.6E-01	1.03	0.88	1.21	7.0E-01	6.1E-01
166	IGFBP3	5	Weighted median	0.94	0.89	1	4.7E-02	9.5E-01	0.93	0.87	1	3.5E-02	7.2E-01	0.92	0.82	1.04	2.1E-01	9.8E-01	0.98	0.83	1.16	8.4E-01	4.6E-01	1.04	0.88	1.23	6.2E-01	6.1E-01
166	IGFBP3	5	Weighted mode	0.94	0.87	1.02	1.1E-01	9.5E-01	0.93	0.85	1.03	1.1E-01	7.2E-01	0.92	0.77	1.11	3.0E-01	9.8E-01	0.97	0.77	1.23	7.4E-01	4.6E-01	1.05	0.82	1.35	6.1E-01	6.1E-01
166	IGFBP3	5	MR Egger	0.94	0.81	1.1	3.1E-01	9.5E-01	0.94	0.8	1.1	2.9E-01	7.2E-01	0.92	0.69	1.24	4.6E-01	9.8E-01	0.91	0.6	1.37	5.1E-01	4.6E-01	1.09	0.72	1.64	5.4E-01	6.1E-01
167	RP2	2	Inverse variance weighted	0.93	0.8	1.09	3.9E-01	NA	0.97	0.81	1.16	7.2E-01	NA	1.2	0.85	1.7	3.0E-01	NA	0.57	0.36	0.89	1.3E-02	NA	0.9	0.56	1.43	6.5E-01	NA
168	IL19	5	Inverse variance weighted	1.07	1.01	1.13	1.3E-02	7.3E-01	1.01	0.95	1.08	6.4E-01	9.0E-01	1.15	1.03	1.3	1.6E-02	7.5E-01	1.19	1.01	1.4	3.7E-02	8.4E-01	1.14	0.89	1.45	2.9E-01	5.9E-01
168	IL19	5	Weighted median	1.07	1.01	1.13	1.4E-02	7.3E-01	1.01	0.95	1.08	6.9E-01	9.0E-01	1.16	1.02	1.3	2.0E-02	7.5E-01	1.18	0.98	1.42	7.2E-02	8.4E-01	1.13	0.96	1.33	1.5E-01	5.9E-01
168	IL19	5	Weighted mode	1.07	0.99	1.16	7.4E-02	7.3E-01	1	0.91	1.1	9.8E-01	9.0E-01	1.16	0.97	1.38	8.2E-02	7.5E-01	1.2	0.91	1.57	1.4E-01	8.4E-01	1.15	0.91	1.45	1.8E-01	5.9E-01
168	IL19	5	MR Egger	1.09	0.93	1.26	1.8E-01	7.3E-01	1.02	0.86	1.22	7.3E-01	9.0E-01	1.19	0.85	1.65	1.9E-01	7.5E-01	1.16	0.72	1.87	4.1E-01	8.4E-01	1.01	0.48	2.14	9.6E-01	5.9E-01
169	GAST	4	Inverse variance weighted	0.89	0.71	1.13	3.6E-01	7.4E-01	0.79	0.66	0.95	1.4E-02	8.9E-01	0.96	0.67	1.36	8.1E-01	9.1E-01	1.27	0.78	2.06	3.3E-01	4.5E-01	1.54	0.95	2.48	7.7E-02	5.6E-01
169	GAST	4	Weighted median	0.86	0.71	1.04	1.1E-01	7.4E-01	0.78	0.63	0.97	2.9E-02	8.9E-01	0.94	0.62	1.44	7.9E-01	9.1E-01	1.09	0.61	1.94	7.7E-01	4.5E-01	1.57	0.91	2.73	1.1E-01	5.6E-01
169	GAST	4	Weighted mode	0.84	0.58	1.23	2.5E-01	7.4E-01	0.77	0.49	1.23	1.8E-01	8.9E-01	0.92	0.39	2.18	7.8E-01	9.1E-01	1.09	0.37	3.23	8.1E-01	4.5E-01	1.8	0.58	5.6	2.0E-01	5.6E-01
169	GAST	4	MR Egger	0.74	0.08	6.58	6.2E-01	7.4E-01	0.75	0.14	3.92	5.3E-01	8.9E-01	0.87	0.03	24.76	8.8E-01	9.1E-01	0.59	0.01	25	6.0E-01	4.5E-01	0.86	0.02	35.49	8.8E-01	5.6E-01
170	AMN	4	Inverse variance weighted	0.95	0.92	0.99	1.4E-02	9.5E-01	0.96	0.92	1	5.7E-02	7.0E-01	0.98	0.91	1.07	6.7E-01	7.3E-01	0.95	0.82	1.1	4.9E-01	2.6E-01	0.93	0.84	1.04	2.3E-01	8.1E-01
170	AMN	4	Weighted median	0.95	0.92	0.99	1.4E-02	9.5E-01	0.96	0.92	1	5.5E-02	7.0E-01	0.98	0.91	1.06	6.9E-01	7.3E-01	0.96	0.86	1.08	5.4E-01	2.6E-01	0.93	0.83	1.03	1.7E-01	8.1E-01
170	AMN	4	Weighted mode	0.95	0.9	1.01	8.6E-02	9.5E-01	0.96	0.88	1.03	1.6E-01	7.0E-01	0.98	0.86	1.13	7.3E-01	7.3E-01	0.97	0.8	1.17	6.1E-01	2.6E-01	0.92	0.77	1.11	2.7E-01	8.1E-01
170	AMN	4	MR Egger	0.96	0.84	1.09	2.8E-01	9.5E-01	0.95	0.81	1.1	2.7E-01	7.0E-01	1	0.75	1.34	9.6E-01	7.3E-01	1.08	0.7	1.66	5.5E-01	2.6E-01	0.92	0.62	1.36	4.4E-01	8.1E-01
171	IL1R1	14	Inverse variance weighted	1.08	1.02	1.15	1.4E-02	8.5E-01	1.06	0.99	1.14	1.1E-01	9.1E-01	1.06	0.93	1.21	3.9E-01	6.4E-02	1.15	0.95	1.38	1.5E-01	5.3E-01	1.07	0.89	1.29	4.4E-01	1.9E-01
171	IL1R1	14	Weighted median	1.09	1	1.17	4.0E-02	8.5E-01	1.05	0.96	1.15	3.0E-01	9.1E-01	1.03	0.88	1.22	7.0E-01	6.4E-02	1.18	0.93	1.5	1.8E-01	5.3E-01	1.17	0.91	1.49	2.2E-01	1.9E-01
171	IL1R1	14	Weighted mode	1.09	0.99	1.19	6.8E-02	8.5E-01	1.06	0.95	1.17	2.6E-01	9.1E-01	1.02	0.85	1.24	7.9E-01	6.4E-02	1.18	0.91	1.53	1.8E-01	5.3E-01	1.21	0.91	1.61	1.8E-01	1.9E-01
171	IL1R1	14	MR Egger	1.07	0.94	1.22	2.9E-01	8.5E-01	1.07	0.92	1.24	3.7E-01	9.1E-01	0.84	0.64	1.12	2.2E-01	6.4E-02	1.27	0.85	1.88	2.2E-01	5.3E-01	1.33	0.9	1.96	1.4E-01	1.9E-01
172	SYTL4	2	Inverse variance weighted	0.88	0.72	1.08	2.2E-01	NA	0.89	0.66	1.21	4.7E-01	NA	0.87	0.56	1.35	5.4E-01	NA	0.73	0.4	1.33	3.0E-01	NA	0.47	0.26	0.86	1.4E-02	NA
173	LMNB2	3	Inverse variance weighted	1.03	0.79	1.35	8.3E-01	5.0E-01	1.25	0.9	1.74	1.8E-01	3.8E-01	0.95	0.53	1.69	8.6E-01	5.3E-01	0.36	0.16	0.81	1.4E-02	4.8E-01	0.58	0.26	1.29	1.8E-01	6.9E-01
173	LMNB2	3	Weighted median	1.08	0.8	1.46	6.1E-01	5.0E-01	1.39	0.96	2.03	8.2E-02	3.8E-01	0.97	0.5	1.89	9.3E-01	5.3E-01	0.35	0.14	0.91	3.0E-02	4.8E-01	0.49	0.2	1.2	1.2E-01	6.9E-01
173	LMNB2	3	Weighted mode	1.16	0.5	2.73	5.2E-01	5.0E-01	1.54	0.43	5.5	2.8E-01	3.8E-01	1.09	0.16	7.53	8.7E-01	5.3E-01	0.49	0.03	7.72	3.8E-01	4.8E-01	0.47	0.04	5.66	3.2E-01	6.9E-01
173	LMNB2	3	MR Egger	0.62	0	455.32	5.3E-01	5.0E-01	0.53	0	1087.21	4.8E-01	3.8E-01	0.35	0	504631.73	5.2E-01	5.3E-01	1.74	0	577303029.5	7.8E-01	4.8E-01	0.27	0	68278716.05	5.5E-01	6.9E-01
174	FAM3D	4	Inverse variance weighted	0.97	0.93	1.02	2.2E-01	7.2E-01	1.01	0.96	1.06	8.0E-01	3.9E-01	0.94	0.82	1.08	4.0E-01	8.2E-01	0.85	0.73	0.99	3.3E-02	2.9E-01	1.02	0.79	1.31	9.0E-01	5.2E-01
174	FAM3D	4	Weighted median	0.96	0.91	1.01	9.7E-02	7.2E-01	1.01	0.96	1.07	6.6E-01	3.9E-01	0.94	0.84	1.06	3.2E-01	8.2E-01	0.84	0.73	0.96	1.4E-02	2.9E-01	0.95	0.82	1.11	5.5E-01	5.2E-01
174	FAM3D	4	Weighted mode	0.96	0.88	1.05	2.2E-01	7.2E-01	1.01	0.93	1.11	7.1E-01	3.9E-01	0.88	0.72	1.07	1.3E-01	8.2E-01	0.83	0.65	1.06	9.4E-02	2.9E-01	0.91	0.69	1.21	3.7E-01	5.2E-01
174	FAM3D	4	MR Egger	0.99	0.81	1.2	8.1E-01	7.2E-01	1.06	0.85	1.31	4.0E-01	3.9E-01	0.91	0.45	1.84	6.2E-01	8.2E-01	0.72	0.41	1.28	1.3E-01	2.9E-01	0.85	0.28	2.65	6.1E-01	5.2E-01
175	LYPD8	7	Inverse variance weighted	0.94	0.9	0.99	2.1E-02	2.2E-01	0.93	0.88	0.99	1.4E-02	6.2E-01	0.92	0.82	1.02	1.2E-01	5.6E-01	1.06	0.91	1.24	4.4E-01	4.1E-01	1.03	0.89	1.21	6.8E-01	9.7E-01
175	LYPD8	7	Weighted median	0.95	0.91	1	7.4E-02	2.2E-01	0.93	0.88	0.99	2.6E-02	6.2E-01	0.93	0.83	1.04	1.7E-01	5.6E-01	1.08	0.93	1.27	3.2E-01	4.1E-01	1.01	0.86	1.19	9.0E-01	9.7E-01
175	LYPD8	7	Weighted mode	0.95	0.89	1.02	1.4E-01	2.2E-01	0.93	0.87	1.01	6.5E-02	6.2E-01	0.93	0.8	1.07	2.5E-01	5.6E-01	1.08	0.88	1.33	3.7E-01	4.1E-01	1.01	0.82	1.24	9.2E-01	9.7E-01
175	LYPD8	7	MR Egger	0.97	0.89	1.06	4.8E-01	2.2E-01	0.94	0.85	1.04	2.0E-01	6.2E-01	0.95	0.78	1.15	4.9E-01	5.6E-01	1.13	0.86	1.48	3.0E-01	4.1E-01	1.04	0.77	1.4	7.7E-01	9.7E-01
176	CDH6	4	Inverse variance weighted	1.05	0.99	1.1	7.7E-02	7.0E-01	1.04	0.98	1.1	2.4E-01	2.4E-01	1.16	0.99	1.36	5.9E-02	3.9E-01	0.98	0.84	1.15	8.3E-01	5.6E-01	0.98	0.85	1.15	8.3E-01	3.0E-01
176	CDH6	4	Weighted median	1.05	1	1.1	6.1E-02	7.0E-01	1.04	0.98	1.11	1.9E-01	2.4E-01	1.16	1.03	1.3	1.4E-02	3.9E-01	0.98	0.84	1.14	7.9E-01	5.6E-01	0.98	0.85	1.13	7.7E-01	3.0E-01
176	CDH6	4	Weighted mode	1.05	0.96	1.15	1.8E-01	7.0E-01	1.04	0.94	1.15	2.7E-01	2.4E-01	1.15	0.97	1.38	8.4E-02	3.9E-01	0.98	0.75	1.28	7.9E-01	5.6E-01	0.96	0.75	1.22	6.2E-01	3.0E-01
176	CDH6	4	MR Egger	1.06	0.88	1.29	3.0E-01	7.0E-01	1.11	0.89	1.38	1.8E-01	2.4E-01	1.03	0.59	1.82	8.2E-01	3.9E-01	0.91	0.51	1.62	5.6E-01	5.6E-01	0.85	0.48	1.49	3.4E-01	3.0E-01
177	KEL	13	Inverse variance weighted	0.92	0.85	0.99	2.7E-02	4.4E-01	0.89	0.81	0.98	1.4E-02	8.4E-01	0.91	0.81	1.02	1.0E-01	2.2E-01	0.92	0.79	1.08	3.2E-01	2.6E-01	0.99	0.8	1.21	8.9E-01	1.8E-01
177	KEL	13	Weighted median	0.94	0.87	1.02	1.2E-01	4.4E-01	0.92	0.84	1	6.1E-02	8.4E-01	0.86	0.75	0.98	2.1E-02	2.2E-01	0.96	0.8	1.15	6.5E-01	2.6E-01	0.96	0.78	1.17	6.7E-01	1.8E-01
177	KEL	13	Weighted mode	0.97	0.87	1.09	6.0E-01	4.4E-01	0.95	0.82	1.11	5.0E-01	8.4E-01	0.86	0.76	0.98	2.6E-02	2.2E-01	0.96	0.8	1.16	6.5E-01	2.6E-01	0.93	0.77	1.12	4.1E-01	1.8E-01
177	KEL	13	MR Egger	0.89	0.79	1	5.8E-02	4.4E-01	0.88	0.76	1.03	9.7E-02	8.4E-01	0.84	0.7	1.01	5.8E-02	2.2E-01	0.85	0.67	1.07	1.5E-01	2.6E-01	0.86	0.63	1.17	2.9E-01	1.8E-01
178	KLRF1	11	Inverse variance weighted	0.95	0.78	1.16	6.2E-01	6.1E-01	0.92	0.72	1.17	4.9E-01	9.4E-01	0.98	0.72	1.35	9.2E-01	5.4E-01	1.04	0.71	1.54	8.3E-01	1.2E-02	1.17	0.78	1.74	4.5E-01	2.4E-01
178	KLRF1	11	Weighted median	0.99	0.84	1.17	8.9E-01	6.1E-01	0.95	0.78	1.15	5.9E-01	9.4E-01	0.94	0.66	1.33	7.3E-01	5.4E-01	0.75	0.44	1.27	2.8E-01	1.2E-02	1.16	0.68	1.95	5.9E-01	2.4E-01
178	KLRF1	11	Weighted mode	0.94	0.75	1.18	5.6E-01	6.1E-01	0.88	0.67	1.15	3.1E-01	9.4E-01	0.9	0.55	1.48	6.6E-01	5.4E-01	0.62	0.21	1.85	3.5E-01	1.2E-02	1.17	0.5	2.74	6.9E-01	2.4E-01
178	KLRF1	11	MR Egger	1.11	0.55	2.24	7.5E-01	6.1E-01	0.95	0.39	2.28	8.9E-01	9.4E-01	1.32	0.43	4.02	5.8E-01	5.4E-01	5.42	1.52	19.27	1.5E-02	1.2E-02	0.58	0.15	2.22	3.8E-01	2.4E-01
179	PDCD6	5	Inverse variance weighted	0.97	0.95	1	2.5E-02	6.3E-01	0.96	0.94	0.99	1.9E-02	4.9E-01	0.97	0.86	1.09	6.1E-01	6.9E-01	1.02	0.94	1.11	6.1E-01	3.0E-01	0.99	0.91	1.07	7.1E-01	7.3E-01
179	PDCD6	5	Weighted median	0.97	0.95	1	2.0E-02	6.3E-01	0.96	0.94	0.99	1.5E-02	4.9E-01	0.97	0.92	1.03	2.8E-01	6.9E-01	1.02	0.94	1.1	6.7E-01	3.0E-01	0.99	0.91	1.07	7.4E-01	7.3E-01
179	PDCD6	5	Weighted mode	0.97	0.93	1.01	9.7E-02	6.3E-01	0.96	0.92	1	6.9E-02	4.9E-01	0.97	0.9	1.05	3.6E-01	6.9E-01	1.02	0.91	1.13	7.0E-01	3.0E-01	0.98	0.88	1.09	6.7E-01	7.3E-01
179	PDCD6	5	MR Egger	0.97	0.92	1.01	1.1E-01	6.3E-01	0.96	0.91	1.01	9.3E-02	4.9E-01	0.95	0.75	1.22	5.8E-01	6.9E-01	0.99	0.86	1.15	8.9E-01	3.0E-01	0.99	0.86	1.15	8.9E-01	7.3E-01
180	ADGRF5	12	Inverse variance weighted	1.07	0.97	1.19	1.6E-01	1.4E-01	1.06	0.95	1.17	3.2E-01	8.1E-02	1.15	0.95	1.39	1.6E-01	2.9E-01	1.42	1.07	1.88	1.5E-02	8.7E-01	1.1	0.8	1.52	5.6E-01	4.7E-01
180	ADGRF5	12	Weighted median	1.08	0.96	1.23	2.0E-01	1.4E-01	1.05	0.91	1.21	5.2E-01	8.1E-02	0.98	0.76	1.26	8.6E-01	2.9E-01	1.55	1.08	2.24	1.9E-02	8.7E-01	1	0.71	1.41	9.8E-01	4.7E-01
180	ADGRF5	12	Weighted mode	1.07	0.91	1.24	3.8E-01	1.4E-01	1.04	0.88	1.22	6.5E-01	8.1E-02	0.98	0.73	1.32	8.9E-01	2.9E-01	1.41	0.9	2.19	1.2E-01	8.7E-01	0.97	0.67	1.43	8.9E-01	4.7E-01
180	ADGRF5	12	MR Egger	0.96	0.79	1.16	6.2E-01	1.4E-01	0.9	0.73	1.12	3.2E-01	8.1E-02	0.97	0.65	1.44	8.6E-01	2.9E-01	1.36	0.74	2.51	2.9E-01	8.7E-01	0.91	0.46	1.8	7.6E-01	4.7E-01
181	TPSAB1	13	Inverse variance weighted	1.01	0.87	1.16	9.4E-01	3.2E-01	1.01	0.85	1.21	8.8E-01	7.4E-01	0.87	0.63	1.19	3.8E-01	2.0E-01	0.8	0.51	1.24	3.1E-01	5.5E-01	1.29	0.75	2.22	3.6E-01	1.2E-01
181	TPSAB1	13	Weighted median	1.14	0.94	1.38	1.9E-01	3.2E-01	1.15	0.91	1.46	2.5E-01	7.4E-01	0.85	0.55	1.3	4.5E-01	2.0E-01	0.91	0.5	1.65	7.5E-01	5.5E-01	1.79	0.95	3.39	7.3E-02	1.2E-01
181	TPSAB1	13	Weighted mode	1.15	0.91	1.46	2.2E-01	3.2E-01	1.13	0.84	1.53	3.8E-01	7.4E-01	1.22	0.67	2.21	4.8E-01	2.0E-01	0.94	0.46	1.9	8.5E-01	5.5E-01	2.76	1.27	6.01	1.5E-02	1.2E-01
181	TPSAB1	13	MR Egger	1.16	0.82	1.65	3.6E-01	3.2E-01	1.08	0.69	1.68	7.2E-01	7.4E-01	1.31	0.62	2.77	4.4E-01	2.0E-01	1.04	0.35	3.07	9.4E-01	5.5E-01	2.85	0.87	9.35	7.9E-02	1.2E-01
182	BAG3	5	Inverse variance weighted	0.89	0.75	1.06	1.9E-01	6.1E-01	0.9	0.74	1.1	3.0E-01	3.8E-01	1.23	0.62	2.46	5.5E-01	4.2E-01	0.9	0.53	1.52	6.8E-01	6.5E-01	0.52	0.31	0.88	1.5E-02	2.7E-01
182	BAG3	5	Weighted median	0.86	0.7	1.06	1.6E-01	6.1E-01	0.85	0.67	1.08	1.8E-01	3.8E-01	1.33	0.82	2.17	2.5E-01	4.2E-01	0.92	0.48	1.79	8.2E-01	6.5E-01	0.53	0.28	1.01	5.3E-02	2.7E-01
182	BAG3	5	Weighted mode	0.79	0.52	1.21	2.0E-01	6.1E-01	0.82	0.53	1.26	2.7E-01	3.8E-01	2.52	0.76	8.4	1.0E-01	4.2E-01	1.11	0.35	3.48	8.2E-01	6.5E-01	0.51	0.18	1.5	1.6E-01	2.7E-01
182	BAG3	5	MR Egger	1.07	0.38	3	8.6E-01	6.1E-01	1.3	0.39	4.33	5.3E-01	3.8E-01	3.97	0.06	255.57	3.7E-01	4.2E-01	0.55	0.02	14.54	6.0E-01	6.5E-01	0.14	0.01	3.54	1.5E-01	2.7E-01
183	AREG	3	Inverse variance weighted	0.9	0.78	1.04	1.5E-01	5.7E-01	0.96	0.81	1.12	5.8E-01	5.5E-01	0.76	0.51	1.13	1.7E-01	5.6E-01	0.54	0.33	0.89	1.5E-02	4.4E-01	1.13	0.61	2.08	7.1E-01	2.9E-01
183	AREG	3	Weighted median	0.92	0.79	1.07	3.0E-01	5.7E-01	0.95	0.8	1.13	5.8E-01	5.5E-01	0.82	0.58	1.14	2.3E-01	5.6E-01	0.62	0.39	0.98	4.2E-02	4.4E-01	1.15	0.74	1.77	5.4E-01	2.9E-01
183	AREG	3	Weighted mode	0.94	0.66	1.33	5.0E-01	5.7E-01	0.92	0.62	1.37	4.7E-01	5.5E-01	0.85	0.4	1.81	4.5E-01	5.6E-01	0.63	0.22	1.77	1.9E-01	4.4E-01	1.42	0.5	4.05	2.9E-01	2.9E-01
183	AREG	3	MR Egger	1.02	0.12	8.77	9.2E-01	5.7E-01	0.83	0.07	9.21	5.0E-01	5.5E-01	1.11	0	619.63	8.7E-01	5.6E-01	0.95	0	742.08	9.4E-01	4.4E-01	2.8	0.01	1396.5	2.8E-01	2.9E-01
184	TAFA5	2	Inverse variance weighted	0.93	0.8	1.09	3.9E-01	NA	0.93	0.78	1.11	4.3E-01	NA	0.93	0.66	1.31	6.8E-01	NA	0.99	0.62	1.58	9.5E-01	NA	0.57	0.36	0.9	1.5E-02	NA
185	TNFRSF4	3	Inverse variance weighted	1.14	0.8	1.61	4.7E-01	9.0E-01	1	0.71	1.41	9.9E-01	9.1E-01	1.44	0.94	2.19	9.0E-02	9.1E-01	2.76	1.08	7.05	3.4E-02	7.6E-01	1.06	0.46	2.46	8.9E-01	3.1E-01
185	TNFRSF4	3	Weighted median	1.09	0.88	1.35	4.3E-01	9.0E-01	0.97	0.76	1.24	8.0E-01	9.1E-01	1.45	0.93	2.26	9.9E-02	9.1E-01	2.28	1.17	4.44	1.5E-02	7.6E-01	0.98	0.54	1.8	9.6E-01	3.1E-01
185	TNFRSF4	3	Weighted mode	1.08	0.65	1.79	5.9E-01	9.0E-01	0.95	0.52	1.76	7.8E-01	9.1E-01	1.47	0.5	4.27	2.6E-01	9.1E-01	2.2	0.54	8.9	1.4E-01	7.6E-01	0.81	0.19	3.37	5.9E-01	3.1E-01
185	TNFRSF4	3	MR Egger	1.03	0	3329.54	9.7E-01	9.0E-01	0.93	0	2613.28	9.2E-01	9.1E-01	1.55	0	1738.55	5.7E-01	9.1E-01	1.54	0	1426587165	8.4E-01	7.6E-01	0.28	0	4622.11	3.5E-01	3.1E-01
186	IL17F	1	Wald ratio	0.69	0.4	1.19	1.8E-01	NA	0.71	0.38	1.33	2.8E-01	NA	0.67	0.21	2.14	5.0E-01	NA	1.33	0.26	6.7	7.3E-01	NA	0.14	0.03	0.68	1.5E-02	NA
187	MRC1	11	Inverse variance weighted	1.14	0.97	1.34	1.0E-01	5.8E-01	1.14	0.95	1.36	1.5E-01	7.5E-01	1.12	0.75	1.67	5.9E-01	8.2E-01	1.2	0.83	1.72	3.3E-01	2.9E-01	1.14	0.8	1.64	4.7E-01	1.5E-01
187	MRC1	11	Weighted median	1.13	0.95	1.34	1.6E-01	5.8E-01	1.27	1.05	1.54	1.5E-02	7.5E-01	0.96	0.65	1.41	8.4E-01	8.2E-01	1.24	0.76	2.03	3.9E-01	2.9E-01	1.4	0.88	2.23	1.5E-01	1.5E-01
187	MRC1	11	Weighted mode	1.24	0.88	1.75	1.9E-01	5.8E-01	1.34	0.99	1.84	6.0E-02	7.5E-01	0.92	0.52	1.62	7.4E-01	8.2E-01	1.66	0.71	3.9	2.1E-01	2.9E-01	1.49	0.75	2.95	2.3E-01	1.5E-01
187	MRC1	11	MR Egger	1.27	0.81	1.97	2.6E-01	5.8E-01	1.21	0.74	2	4.0E-01	7.5E-01	1	0.32	3.12	9.9E-01	8.2E-01	1.82	0.71	4.7	1.8E-01	2.9E-01	2.06	0.8	5.33	1.2E-01	1.5E-01
188	IGFBP7	4	Inverse variance weighted	0.94	0.87	1.02	1.3E-01	5.5E-01	0.93	0.86	1.02	1.2E-01	3.5E-01	0.93	0.78	1.09	3.6E-01	8.1E-01	0.76	0.57	1.03	7.5E-02	5.6E-01	0.98	0.76	1.27	8.9E-01	7.8E-01
188	IGFBP7	4	Weighted median	0.95	0.88	1.02	1.5E-01	5.5E-01	0.94	0.86	1.03	2.0E-01	3.5E-01	0.92	0.78	1.09	3.5E-01	8.1E-01	0.73	0.57	0.94	1.5E-02	5.6E-01	1	0.8	1.27	9.7E-01	7.8E-01
188	IGFBP7	4	Weighted mode	0.95	0.84	1.08	2.8E-01	5.5E-01	0.94	0.82	1.09	2.8E-01	3.5E-01	0.92	0.69	1.21	3.9E-01	8.1E-01	0.71	0.49	1.04	6.7E-02	5.6E-01	1.02	0.68	1.52	9.0E-01	7.8E-01
188	IGFBP7	4	MR Egger	0.98	0.75	1.27	7.2E-01	5.5E-01	1	0.74	1.35	9.7E-01	3.5E-01	0.9	0.51	1.6	5.1E-01	8.1E-01	0.66	0.21	2.07	2.6E-01	5.6E-01	1.04	0.36	3	8.8E-01	7.8E-01
189	PIK3AP1	5	Inverse variance weighted	0.98	0.88	1.1	7.6E-01	2.9E-01	0.98	0.84	1.13	7.4E-01	2.7E-01	0.89	0.69	1.14	3.5E-01	9.0E-01	1.34	0.91	1.97	1.4E-01	2.2E-01	0.8	0.57	1.12	1.9E-01	2.8E-01
189	PIK3AP1	5	Weighted median	1.01	0.88	1.15	9.3E-01	2.9E-01	1.05	0.9	1.21	5.4E-01	2.7E-01	0.91	0.7	1.19	5.0E-01	9.0E-01	1.65	1.1	2.48	1.5E-02	2.2E-01	0.7	0.48	1.02	6.4E-02	2.8E-01
189	PIK3AP1	5	Weighted mode	1.05	0.85	1.29	5.8E-01	2.9E-01	1.05	0.83	1.32	6.0E-01	2.7E-01	0.91	0.62	1.35	5.6E-01	9.0E-01	1.73	0.87	3.42	9.1E-02	2.2E-01	0.7	0.4	1.22	1.5E-01	2.8E-01
189	PIK3AP1	5	MR Egger	1.12	0.77	1.61	4.1E-01	2.9E-01	1.14	0.74	1.74	4.1E-01	2.7E-01	0.91	0.42	2.01	7.4E-01	9.0E-01	2.14	0.69	6.59	1.2E-01	2.2E-01	0.54	0.19	1.59	1.7E-01	2.8E-01
190	SPINK5	7	Inverse variance weighted	0.99	0.89	1.09	7.8E-01	9.7E-01	0.92	0.82	1.03	1.3E-01	9.3E-01	1.05	0.89	1.24	5.3E-01	9.9E-01	1.23	1.04	1.46	1.5E-02	6.1E-01	1.17	0.99	1.4	7.1E-02	1.8E-01
190	SPINK5	7	Weighted median	0.98	0.92	1.04	5.0E-01	9.7E-01	0.92	0.86	0.99	2.4E-02	9.3E-01	1.02	0.89	1.18	7.3E-01	9.9E-01	1.2	0.99	1.46	6.3E-02	6.1E-01	1.2	1.01	1.43	4.2E-02	1.8E-01
190	SPINK5	7	Weighted mode	0.98	0.9	1.06	5.4E-01	9.7E-01	0.92	0.85	1	5.0E-02	9.3E-01	1.04	0.87	1.24	6.3E-01	9.9E-01	1.15	0.88	1.51	2.5E-01	6.1E-01	1.21	0.97	1.51	8.4E-02	1.8E-01
190	SPINK5	7	MR Egger	0.99	0.79	1.24	9.0E-01	9.7E-01	0.91	0.71	1.17	3.9E-01	9.3E-01	1.06	0.73	1.53	7.2E-01	9.9E-01	1.16	0.82	1.65	3.1E-01	6.1E-01	1.38	0.98	1.95	6.3E-02	1.8E-01
191	SAT2	1	Wald ratio	0.94	0.9	0.99	1.5E-02	NA	0.94	0.89	1	3.7E-02	NA	0.9	0.81	0.99	4.0E-02	NA	1.02	0.88	1.19	7.8E-01	NA	1.04	0.89	1.21	6.0E-01	NA
192	GIP	1	Wald ratio	0.77	0.6	1	4.7E-02	NA	0.7	0.52	0.93	1.6E-02	NA	0.73	0.42	1.26	2.6E-01	NA	1.43	0.65	3.13	3.7E-01	NA	1.01	0.47	2.15	9.9E-01	NA
193	SETMAR	1	Wald ratio	1.07	0.95	1.21	2.6E-01	NA	0.99	0.87	1.14	9.3E-01	NA	1.22	0.94	1.58	1.3E-01	NA	1.27	0.89	1.83	1.9E-01	NA	1.55	1.09	2.22	1.6E-02	NA
194	NRGN	3	Inverse variance weighted	1.02	0.81	1.28	8.9E-01	4.9E-01	1.29	0.89	1.88	1.7E-01	3.4E-01	0.73	0.44	1.2	2.1E-01	9.9E-01	0.52	0.26	1.05	7.0E-02	9.4E-01	0.69	0.34	1.38	2.9E-01	5.8E-01
194	NRGN	3	Weighted median	1.08	0.83	1.41	5.6E-01	4.9E-01	1.48	1.08	2.03	1.6E-02	3.4E-01	0.75	0.44	1.29	3.0E-01	9.9E-01	0.53	0.24	1.14	1.0E-01	9.4E-01	0.64	0.3	1.36	2.4E-01	5.8E-01
194	NRGN	3	Weighted mode	1.09	0.57	2.11	6.2E-01	4.9E-01	1.49	0.73	3.04	1.4E-01	3.4E-01	0.79	0.18	3.35	5.5E-01	9.9E-01	0.5	0.06	4.17	3.0E-01	9.4E-01	0.95	0.13	7.06	9.2E-01	5.8E-01
194	NRGN	3	MR Egger	1.84	0	3520.23	4.9E-01	4.9E-01	4.04	0	25301.06	2.9E-01	3.4E-01	0.71	0	8564526.41	8.3E-01	9.9E-01	0.44	0	3325042817	7.3E-01	9.4E-01	2.71	0	17612865627	6.7E-01	5.8E-01
195	MICALL2	1	Wald ratio	1.62	1.09	2.4	1.6E-02	NA	1.68	1.07	2.64	2.5E-02	NA	2.35	1.01	5.48	4.7E-02	NA	0.94	0.29	3.07	9.2E-01	NA	1.31	0.41	4.2	6.5E-01	NA
196	EPHA1	5	Inverse variance weighted	1.07	0.95	1.2	2.8E-01	5.1E-01	1.03	0.91	1.18	6.2E-01	4.8E-01	1.07	0.83	1.37	6.1E-01	2.4E-01	1.59	1.09	2.32	1.6E-02	2.4E-01	0.93	0.66	1.31	6.8E-01	9.0E-01
196	EPHA1	5	Weighted median	1.06	0.94	1.2	3.5E-01	5.1E-01	1.05	0.91	1.21	5.4E-01	4.8E-01	1.14	0.87	1.48	3.4E-01	2.4E-01	1.52	1.06	2.19	2.2E-02	2.4E-01	0.91	0.63	1.31	6.2E-01	9.0E-01
196	EPHA1	5	Weighted mode	1.11	0.91	1.34	2.2E-01	5.1E-01	1.06	0.86	1.3	5.1E-01	4.8E-01	1.15	0.77	1.71	4.0E-01	2.4E-01	1.53	0.91	2.55	8.5E-02	2.4E-01	0.91	0.52	1.57	6.5E-01	9.0E-01
196	EPHA1	5	MR Egger	1.13	0.82	1.55	3.0E-01	5.1E-01	1.12	0.77	1.61	4.1E-01	4.8E-01	1.37	0.7	2.7	2.4E-01	2.4E-01	1.12	0.44	2.86	7.1E-01	2.4E-01	0.9	0.35	2.3	7.4E-01	9.0E-01
197	APLP1	5	Inverse variance weighted	0.89	0.71	1.12	3.1E-01	9.4E-02	0.79	0.65	0.96	1.6E-02	1.7E-01	1.3	0.91	1.86	1.5E-01	4.0E-01	0.94	0.41	2.19	8.9E-01	1.5E-01	0.9	0.4	2.04	8.1E-01	4.7E-01
197	APLP1	5	Weighted median	0.88	0.71	1.1	2.7E-01	9.4E-02	0.82	0.65	1.04	1.0E-01	1.7E-01	1.49	0.93	2.39	9.3E-02	4.0E-01	1.24	0.63	2.41	5.3E-01	1.5E-01	1.35	0.69	2.62	3.8E-01	4.7E-01
197	APLP1	5	Weighted mode	0.88	0.59	1.3	4.1E-01	9.4E-02	0.85	0.55	1.33	3.8E-01	1.7E-01	1.6	0.72	3.53	1.8E-01	4.0E-01	1.55	0.51	4.71	3.3E-01	1.5E-01	1.52	0.45	5.11	4.0E-01	4.7E-01
197	APLP1	5	MR Egger	2.01	0.67	6.04	1.4E-01	9.4E-02	1.61	0.45	5.75	3.2E-01	1.7E-01	2.66	0.24	28.87	2.8E-01	4.0E-01	11.93	0.15	942.47	1.7E-01	1.5E-01	3.73	0.01	1119.55	5.2E-01	4.7E-01
198	CDNF	1	Wald ratio	0.94	0.87	1.02	1.5E-01	NA	0.98	0.9	1.08	7.3E-01	NA	0.86	0.73	1.02	7.9E-02	NA	0.76	0.61	0.95	1.6E-02	NA	0.76	0.61	0.95	1.7E-02	NA
199	TNFRSF9	5	Inverse variance weighted	1.07	0.93	1.23	3.3E-01	8.1E-01	1.04	0.87	1.23	6.7E-01	9.8E-01	1.02	0.77	1.36	8.7E-01	5.3E-01	1.36	0.92	2.02	1.2E-01	8.6E-01	1.4	0.88	2.25	1.6E-01	3.2E-01
199	TNFRSF9	5	Weighted median	1.08	0.93	1.26	3.1E-01	8.1E-01	1.05	0.88	1.25	5.8E-01	9.8E-01	1.03	0.74	1.43	8.7E-01	5.3E-01	1.38	0.91	2.1	1.2E-01	8.6E-01	1.77	1.11	2.82	1.6E-02	3.2E-01
199	TNFRSF9	5	Weighted mode	1.08	0.86	1.37	3.9E-01	8.1E-01	1.01	0.76	1.34	9.2E-01	9.8E-01	1.03	0.64	1.66	8.7E-01	5.3E-01	1.34	0.67	2.67	3.1E-01	8.6E-01	1.85	0.94	3.64	6.6E-02	3.2E-01
199	TNFRSF9	5	MR Egger	1.14	0.52	2.5	6.3E-01	8.1E-01	1.04	0.39	2.82	9.0E-01	9.8E-01	1.38	0.34	5.64	5.2E-01	5.3E-01	1.22	0.18	8.24	7.6E-01	8.6E-01	3.05	0.34	27.01	2.0E-01	3.2E-01
200	CDHR5	8	Inverse variance weighted	1	0.95	1.06	9.4E-01	8.5E-02	0.99	0.94	1.04	6.2E-01	2.6E-01	0.98	0.85	1.13	7.4E-01	1.4E-02	1.17	1.02	1.35	3.0E-02	1.0E+00	1	0.88	1.15	9.5E-01	3.7E-01

200	CDHR5	8	Weighted median	0.99	0.94	1.05	8.3E-01	8.5E-02	0.97	0.92	1.03	3.5E-01	2.6E-01	0.94	0.84	1.05	2.5E-01	1.4E-02	1.2	1.03	1.38	1.7E-02	1.0E+00	0.99	0.86	1.14	8.9E-01	3.7E-01
200	CDHR5	8	Weighted mode	0.99	0.93	1.06	6.8E-01	8.5E-02	0.98	0.92	1.05	4.8E-01	2.6E-01	0.94	0.82	1.07	2.7E-01	1.4E-02	1.2	1.02	1.41	3.3E-02	1.0E+00	0.99	0.84	1.17	8.9E-01	3.7E-01
200	CDHR5	8	MR Egger	0.96	0.89	1.04	2.3E-01	8.5E-02	0.96	0.87	1.05	2.7E-01	2.6E-01	0.83	0.7	0.98	3.6E-02	1.4E-02	1.17	0.9	1.52	1.8E-01	1.0E+00	0.94	0.74	1.19	5.6E-01	3.7E-01
201	KLK11	6	Inverse variance weighted	1.09	1.01	1.18	3.3E-02	8.1E-01	1.1	1.01	1.19	2.7E-02	9.1E-01	1.04	0.87	1.26	6.6E-01	1.1E-01	1.12	0.89	1.41	3.5E-01	9.8E-01	1.29	1.04	1.61	2.2E-02	4.9E-01
201	KLK11	6	Weighted median	1.07	0.99	1.15	8.0E-02	8.1E-01	1.1	1.01	1.19	3.0E-02	9.1E-01	1	0.85	1.17	9.7E-01	1.1E-01	1.06	0.84	1.33	6.5E-01	9.8E-01	1.31	1.05	1.63	1.7E-02	4.9E-01
201	KLK11	6	Weighted mode	1.07	0.97	1.19	1.4E-01	8.1E-01	1.09	0.97	1.22	1.1E-01	9.1E-01	0.98	0.78	1.22	8.1E-01	1.1E-01	1.03	0.75	1.42	8.2E-01	9.8E-01	1.31	0.96	1.8	7.5E-02	4.9E-01
201	KLK11	6	MR Egger	1.08	0.93	1.26	2.1E-01	8.1E-01	1.1	0.96	1.26	1.2E-01	9.1E-01	0.95	0.74	1.21	5.8E-01	1.1E-01	1.12	0.73	1.72	5.1E-01	9.8E-01	1.36	0.94	1.96	7.9E-02	4.9E-01
202	CFHR5	5	Inverse variance weighted	0.95	0.88	1.02	1.4E-01	6.2E-01	0.95	0.87	1.04	2.6E-01	5.1E-01	0.85	0.7	1.04	1.1E-01	3.0E-01	0.8	0.64	0.98	3.4E-02	2.6E-01	0.82	0.66	1.01	6.1E-02	4.1E-01
202	CFHR5	5	Weighted median	0.95	0.88	1.02	1.6E-01	6.2E-01	0.96	0.88	1.05	3.6E-01	5.1E-01	0.83	0.71	0.97	1.7E-02	3.0E-01	0.77	0.62	0.96	1.8E-02	2.6E-01	0.83	0.66	1.03	8.3E-02	4.1E-01
202	CFHR5	5	Weighted mode	0.94	0.85	1.05	2.1E-01	6.2E-01	0.96	0.85	1.09	4.4E-01	5.1E-01	0.82	0.65	1.05	1.6E-01	3.0E-01	0.77	0.57	1.04	7.3E-02	2.6E-01	0.83	0.61	1.13	1.7E-01	4.1E-01
202	CFHR5	5	MR Egger	0.94	0.82	1.07	2.1E-01	6.2E-01	0.97	0.83	1.13	5.3E-01	5.1E-01	0.81	0.59	1.12	1.3E-01	3.0E-01	0.75	0.51	1.08	8.8E-02	2.6E-01	0.86	0.59	1.24	2.8E-01	4.1E-01
203	HEPACAM2	23	Inverse variance weighted	0.95	0.89	1.02	1.5E-01	5.0E-02	0.92	0.85	1	4.9E-02	1.1E-01	0.93	0.83	1.03	1.6E-01	2.7E-01	1.04	0.91	1.2	5.5E-01	8.2E-01	0.99	0.85	1.16	9.1E-01	9.4E-03
203	HEPACAM2	23	Weighted median	1.01	0.92	1.1	8.6E-01	5.0E-02	0.95	0.85	1.06	3.4E-01	1.1E-01	0.84	0.73	0.97	1.7E-02	2.7E-01	1.01	0.83	1.22	9.4E-01	8.2E-01	0.8	0.65	0.98	2.9E-02	9.4E-03
203	HEPACAM2	23	Weighted mode	1.01	0.8	1.27	9.2E-01	5.0E-02	1	0.78	1.29	9.8E-01	1.1E-01	0.84	0.72	0.98	2.9E-02	2.7E-01	1.02	0.85	1.24	7.9E-01	8.2E-01	0.77	0.62	0.96	2.1E-02	9.4E-03
203	HEPACAM2	23	MR Egger	0.87	0.78	0.97	1.7E-02	5.0E-02	0.85	0.74	0.97	1.7E-02	1.1E-01	0.86	0.72	1.03	9.2E-02	2.7E-01	1.06	0.84	1.34	5.8E-01	8.2E-01	0.77	0.61	0.98	3.2E-02	9.4E-03
204	SIRT2	5	Inverse variance weighted	1.19	0.98	1.45	7.9E-02	3.9E-01	1.13	0.94	1.36	2.1E-01	5.6E-01	1.47	1.03	2.1	3.6E-02	8.4E-01	1.28	0.77	2.13	3.5E-01	4.5E-01	1.48	0.77	2.82	2.4E-01	2.0E-01
204	SIRT2	5	Weighted median	1.29	1.05	1.59	1.7E-02	3.9E-01	1.18	0.93	1.5	1.8E-01	5.6E-01	1.43	0.92	2.22	1.2E-01	8.4E-01	1.52	0.82	2.83	1.9E-01	4.5E-01	1.23	0.6	2.52	5.6E-01	2.0E-01
204	SIRT2	5	Weighted mode	1.34	0.96	1.87	7.3E-02	3.9E-01	1.19	0.83	1.69	2.5E-01	5.6E-01	1.43	0.69	2.93	2.4E-01	8.4E-01	1.77	0.58	5.37	2.3E-01	4.5E-01	0.96	0.16	5.87	9.6E-01	2.0E-01
204	SIRT2	5	MR Egger	1.35	0.81	2.26	1.6E-01	3.9E-01	1.23	0.73	2.06	3.0E-01	5.6E-01	1.39	0.49	3.92	3.9E-01	8.4E-01	1.77	0.42	7.5	3.0E-01	4.5E-01	3.06	0.59	15.91	1.2E-01	2.0E-01
205	GFRA3	17	Inverse variance weighted	1.07	0.94	1.22	3.1E-01	5.0E-01	1.07	0.9	1.27	4.2E-01	5.5E-01	1.05	0.83	1.32	6.9E-01	2.6E-01	1.05	0.76	1.46	7.5E-01	3.1E-01	**0.68**	**0.49**	**0.93**	**1.7E-02**	1.7E-01
205	GFRA3	17	Weighted median	1.04	0.88	1.22	6.7E-01	5.0E-01	1.04	0.86	1.26	6.6E-01	5.5E-01	0.95	0.69	1.33	7.8E-01	2.6E-01	0.8	0.52	1.25	3.3E-01	3.1E-01	**0.63**	**0.41**	**0.97**	**3.5E-02**	1.7E-01
205	GFRA3	17	Weighted mode	1.02	0.75	1.38	9.1E-01	5.0E-01	0.95	0.66	1.38	7.8E-01	5.5E-01	0.76	0.38	1.55	4.3E-01	2.6E-01	0.75	0.34	1.66	4.5E-01	3.1E-01	0.62	0.28	1.38	2.2E-01	1.7E-01
205	GFRA3	17	MR Egger	1.22	0.79	1.88	3.4E-01	5.0E-01	1.26	0.7	2.24	4.1E-01	5.5E-01	0.7	0.32	1.52	3.5E-01	2.6E-01	0.65	0.23	1.85	3.9E-01	3.1E-01	1.3	0.46	3.67	5.9E-01	1.7E-01
206	POF1B	4	Inverse variance weighted	1.24	1	1.53	5.0E-02	3.4E-01	1.32	1.05	1.65	1.7E-02	3.8E-01	1.15	0.59	2.24	6.8E-01	3.6E-01	1.52	0.85	2.72	1.6E-01	9.5E-01	1.16	0.65	2.08	6.2E-01	7.0E-01
206	POF1B	4	Weighted median	1.19	0.93	1.51	1.6E-01	3.4E-01	1.28	0.98	1.68	7.5E-02	3.8E-01	1	0.54	1.84	9.9E-01	3.6E-01	1.47	0.75	2.87	2.6E-01	9.5E-01	1.04	0.52	2.05	9.2E-01	7.0E-01
206	POF1B	4	Weighted mode	1.09	0.7	1.68	5.9E-01	3.4E-01	1.21	0.76	1.94	2.8E-01	3.8E-01	0.74	0.24	2.21	5.9E-01	3.6E-01	1.43	0.39	5.2	4.5E-01	9.5E-01	1	0.29	3.42	9.9E-01	7.0E-01
206	POF1B	4	MR Egger	0.75	0.12	4.49	5.6E-01	3.4E-01	0.78	0.1	6.13	6.5E-01	3.8E-01	0.25	0	78.29	4.1E-01	3.6E-01	1.65	0.01	392.03	7.3E-01	9.5E-01	0.67	0	138.75	7.8E-01	7.0E-01
207	ANGPTL2	3	Inverse variance weighted	1.02	0.89	1.16	7.9E-01	3.2E-01	0.99	0.82	1.19	9.2E-01	2.6E-01	1.29	1.05	1.59	1.7E-02	5.4E-01	0.98	0.66	1.46	9.1E-01	7.4E-01	0.92	0.66	1.3	6.4E-01	7.0E-01
207	ANGPTL2	3	Weighted median	1.04	0.94	1.15	4.4E-01	3.2E-01	1.02	0.91	1.15	7.3E-01	2.6E-01	1.26	1.03	1.56	2.8E-02	5.4E-01	0.84	0.61	1.17	3.1E-01	7.4E-01	0.93	0.68	1.28	6.7E-01	7.0E-01
207	ANGPTL2	3	Weighted mode	1.04	0.82	1.32	5.4E-01	3.2E-01	1.02	0.78	1.33	7.6E-01	2.6E-01	1.25	0.76	2.08	1.9E-01	5.4E-01	0.83	0.38	1.85	4.3E-01	7.4E-01	1.01	0.49	2.09	9.4E-01	7.0E-01
207	ANGPTL2	3	MR Egger	1.16	0.38	3.48	3.4E-01	3.2E-01	1.19	0.34	4.25	3.3E-01	2.6E-01	1.13	0.11	11.82	6.2E-01	5.4E-01	1.14	0	347.59	8.2E-01	7.4E-01	1.08	0.01	136.61	8.8E-01	7.0E-01
208	LGALS9	2	Inverse variance weighted	1.01	0.91	1.12	8.5E-01	NA	0.99	0.93	1.06	7.6E-01	NA	1.02	0.76	1.36	9.0E-01	NA	1.03	0.66	1.6	9.0E-01	NA	1.24	1.04	1.48	1.7E-02	NA
209	HDGF	2	Inverse variance weighted	0.98	0.96	1	8.4E-02	NA	0.99	0.95	1.03	6.0E-01	NA	0.99	0.94	1.05	8.1E-01	NA	0.93	0.86	1	4.9E-02	NA	0.92	0.85	0.98	1.7E-02	NA
210	SOST	10	Inverse variance weighted	1.13	0.98	1.32	1.0E-01	7.1E-02	1.1	0.94	1.29	2.3E-01	4.2E-01	1.38	1.03	1.86	3.0E-02	6.2E-01	0.94	0.55	1.62	8.3E-01	1.2E-02	1.21	0.74	1.97	4.4E-01	2.7E-01
210	SOST	10	Weighted median	1.14	0.94	1.38	1.7E-01	7.1E-02	1.07	0.88	1.29	4.9E-01	4.2E-01	1.56	1.08	2.26	1.9E-02	6.2E-01	1.16	0.65	2.07	6.1E-01	1.2E-02	1	0.57	1.74	1.0E+00	2.7E-01
210	SOST	10	Weighted mode	1.15	0.84	1.57	3.3E-01	7.1E-02	1.05	0.76	1.46	7.3E-01	4.2E-01	1.52	0.79	2.92	1.8E-01	6.2E-01	1.42	0.51	3.93	4.5E-01	1.2E-02	0.92	0.39	2.16	8.4E-01	2.7E-01
210	SOST	10	MR Egger	1.76	1.05	2.94	3.5E-02	7.1E-02	1.36	0.75	2.46	2.7E-01	4.2E-01	1.75	0.58	5.25	2.8E-01	6.2E-01	7.26	1.57	33.57	1.8E-02	1.2E-02	2.93	0.48	17.99	2.1E-01	2.7E-01
211	GUCY2C	1	Wald ratio	0.69	0.48	0.99	4.2E-02	NA	0.61	0.4	0.92	1.8E-02	NA	0.57	0.26	1.23	1.5E-01	NA	0.56	0.19	1.63	2.9E-01	NA	1.09	0.38	3.14	8.8E-01	NA
212	CD300LG	11	Inverse variance weighted	1.08	1	1.16	3.6E-02	9.4E-01	1.09	1.01	1.18	1.9E-02	1.0E+00	1.09	0.92	1.3	3.0E-01	8.1E-01	0.97	0.8	1.17	7.6E-01	3.4E-01	0.98	0.81	1.18	8.0E-01	8.8E-01
212	CD300LG	11	Weighted median	1.09	1.01	1.17	2.4E-02	9.4E-01	1.1	1.02	1.2	1.8E-02	1.0E+00	1.12	0.96	1.31	1.5E-01	8.1E-01	1.02	0.83	1.26	8.6E-01	3.4E-01	1	0.82	1.22	1.0E+00	8.8E-01
212	CD300LG	11	Weighted mode	1.08	1	1.17	4.6E-02	9.4E-01	1.1	1.01	1.21	3.9E-02	1.0E+00	1.13	0.95	1.35	1.6E-01	8.1E-01	1.02	0.8	1.29	8.8E-01	3.4E-01	1.01	0.8	1.28	9.3E-01	8.8E-01
212	CD300LG	11	MR Egger	1.08	0.97	1.2	1.3E-01	9.4E-01	1.09	0.98	1.21	9.0E-02	1.0E+00	1.11	0.86	1.44	3.7E-01	8.1E-01	1.04	0.79	1.36	7.6E-01	3.4E-01	0.96	0.73	1.28	7.8E-01	8.8E-01
213	QSOX1	3	Inverse variance weighted	1.01	0.96	1.05	8.2E-01	4.3E-01	1.02	0.96	1.07	5.9E-01	4.1E-01	0.92	0.83	1.02	1.0E-01	5.2E-01	0.97	0.84	1.11	6.3E-01	8.5E-01	1.2	1.03	1.39	1.8E-02	9.5E-01
213	QSOX1	3	Weighted median	1	0.96	1.05	9.0E-01	4.3E-01	1.01	0.96	1.07	6.4E-01	4.1E-01	0.92	0.83	1.01	9.5E-02	5.2E-01	0.97	0.84	1.1	6.1E-01	8.5E-01	1.19	1.02	1.4	3.1E-02	9.5E-01
213	QSOX1	3	Weighted mode	1	0.91	1.11	9.0E-01	4.3E-01	1.01	0.9	1.14	7.2E-01	4.1E-01	0.92	0.73	1.14	2.3E-01	5.2E-01	0.96	0.71	1.31	6.5E-01	8.5E-01	1.19	0.85	1.67	1.6E-01	9.5E-01
213	QSOX1	3	MR Egger	0.98	0.68	1.43	6.9E-01	4.3E-01	0.99	0.64	1.52	8.0E-01	4.1E-01	0.89	0.4	1.97	3.1E-01	5.2E-01	0.95	0.31	2.92	6.9E-01	8.5E-01	1.19	0.38	3.78	3.1E-01	9.5E-01
214	SHMT1	4	Inverse variance weighted	1.04	1.01	1.08	2.5E-02	8.9E-01	1.05	1.01	1.1	2.0E-02	7.2E-01	1.01	0.93	1.1	7.6E-01	9.4E-01	1.05	0.94	1.18	3.9E-01	3.1E-01	0.96	0.86	1.07	4.2E-01	3.9E-01
214	SHMT1	4	Weighted median	1.04	1	1.08	2.7E-02	8.9E-01	1.05	1.01	1.1	1.8E-02	7.2E-01	1.01	0.93	1.1	7.5E-01	9.4E-01	1.05	0.93	1.17	3.7E-01	3.1E-01	0.96	0.86	1.08	5.1E-01	3.9E-01
214	SHMT1	4	Weighted mode	1.04	0.98	1.11	1.2E-01	8.9E-01	1.05	0.98	1.13	1.0E-01	7.2E-01	1.01	0.88	1.17	7.8E-01	9.4E-01	1.06	0.88	1.28	3.7E-01	3.1E-01	0.96	0.8	1.16	5.7E-01	3.9E-01
214	SHMT1	4	MR Egger	1.04	0.92	1.18	3.1E-01	8.9E-01	1.05	0.9	1.21	3.2E-01	7.2E-01	1.02	0.78	1.32	8.2E-01	9.4E-01	1.12	0.82	1.53	2.7E-01	3.1E-01	1	0.74	1.37	9.7E-01	3.9E-01
215	TRAF3	2	Inverse variance weighted	0.85	0.65	1.09	2.0E-01	NA	0.7	0.52	0.94	1.8E-02	NA	1.27	0.73	2.21	4.0E-01	NA	0.73	0.18	2.97	6.7E-01	NA	1.12	0.28	4.38	8.8E-01	NA
216	SOD2	5	Inverse variance weighted	0.95	0.85	1.07	4.0E-01	8.0E-01	1.02	0.86	1.21	8.3E-01	9.3E-01	0.92	0.68	1.25	5.9E-01	3.4E-01	0.65	0.46	0.93	1.8E-02	3.2E-01	0.81	0.57	1.14	2.3E-01	9.2E-01
216	SOD2	5	Weighted median	0.97	0.85	1.1	6.3E-01	8.0E-01	1.03	0.89	1.21	6.8E-01	9.3E-01	0.97	0.71	1.32	8.3E-01	3.4E-01	0.68	0.44	1.04	7.5E-02	3.2E-01	0.82	0.55	1.21	3.1E-01	9.2E-01
216	SOD2	5	Weighted mode	0.97	0.81	1.16	6.8E-01	8.0E-01	1.03	0.83	1.27	7.3E-01	9.3E-01	1	0.64	1.56	9.8E-01	3.4E-01	0.72	0.39	1.36	2.3E-01	3.2E-01	0.81	0.46	1.42	3.6E-01	9.2E-01
216	SOD2	5	MR Egger	0.99	0.6	1.65	9.6E-01	8.0E-01	1	0.42	2.37	9.9E-01	9.3E-01	1.42	0.39	5.21	4.6E-01	3.4E-01	1.12	0.24	5.19	8.3E-01	3.2E-01	0.85	0.17	4.31	7.7E-01	9.2E-01
217	TNFSF10	9	Inverse variance weighted	0.97	0.89	1.05	4.1E-01	3.5E-02	0.97	0.9	1.05	4.5E-01	7.5E-02	1.01	0.85	1.2	9.1E-01	6.2E-02	0.88	0.74	1.04	1.3E-01	4.6E-01	1	0.86	1.17	9.6E-01	1.0E-01
217	TNFSF10	9	Weighted median	0.92	0.86	0.99	1.8E-02	3.5E-02	0.97	0.89	1.04	3.9E-01	7.5E-02	0.94	0.81	1.08	3.8E-01	6.2E-02	0.9	0.73	1.11	3.1E-01	4.6E-01	0.95	0.8	1.15	5.8E-01	1.0E-01
217	TNFSF10	9	Weighted mode	0.92	0.85	1	5.9E-02	3.5E-02	0.95	0.87	1.05	2.8E-01	7.5E-02	0.95	0.81	1.13	5.8E-01	6.2E-02	0.91	0.71	1.16	3.8E-01	4.6E-01	0.93	0.74	1.18	5.5E-01	1.0E-01
217	TNFSF10	9	MR Egger	0.89	0.8	0.99	3.7E-02	3.5E-02	0.91	0.81	1.01	7.5E-02	7.5E-02	0.87	0.69	1.1	2.0E-01	6.2E-02	0.82	0.62	1.09	1.5E-01	4.6E-01	0.87	0.67	1.13	2.6E-01	1.0E-01
218	LGALS3BP	9	Inverse variance weighted	1	0.93	1.08	9.7E-01	9.7E-01	1.05	0.95	1.15	3.6E-01	6.5E-01	0.91	0.74	1.13	4.0E-01	8.1E-01	1.1	0.91	1.32	3.1E-01	9.0E-01	0.98	0.71	1.36	9.2E-01	6.1E-01
218	LGALS3BP	9	Weighted median	1	0.93	1.08	9.5E-01	9.7E-01	1.05	0.97	1.14	2.0E-01	6.5E-01	0.79	0.66	0.96	1.8E-02	8.1E-01	1.05	0.85	1.31	6.3E-01	9.0E-01	0.96	0.77	1.19	6.9E-01	6.1E-01
218	LGALS3BP	9	Weighted mode	1	0.92	1.09	9.3E-01	9.7E-01	1.06	0.96	1.16	2.0E-01	6.5E-01	0.83	0.66	1.04	8.6E-02	8.1E-01	1.07	0.84	1.37	5.2E-01	9.0E-01	0.96	0.74	1.24	7.2E-01	6.1E-01
218	LGALS3BP	9	MR Egger	1	0.9	1.12	9.2E-01	9.7E-01	1.06	0.92	1.22	3.5E-01	6.5E-01	0.9	0.66	1.23	4.4E-01	8.1E-01	1.11	0.86	1.44	3.8E-01	9.0E-01	0.93	0.58	1.5	7.3E-01	6.1E-01
219	VEGFC	13	Inverse variance weighted	1.1	0.97	1.26	1.5E-01	4.2E-01	1.08	0.92	1.26	3.5E-01	6.0E-01	1.06	0.86	1.31	5.8E-01	9.7E-01	1.45	1.07	1.97	1.8E-02	2.1E-01	1.18	0.8	1.75	3.9E-01	6.0E-01
219	VEGFC	13	Weighted median	1.14	1	1.3	5.2E-02	4.2E-01	1.09	0.94	1.27	2.5E-01	6.0E-01	1.03	0.78	1.35	8.5E-01	9.7E-01	1.57	1.04	2.37	3.3E-02	2.1E-01	1.23	0.8	1.89	3.4E-01	6.0E-01
219	VEGFC	13	Weighted mode	1.13	0.97	1.33	1.1E-01	4.2E-01	1.12	0.93	1.34	2.1E-01	6.0E-01	1.04	0.72	1.52	8.4E-01	9.7E-01	1.65	0.83	3.26	1.4E-01	2.1E-01	1.43	0.71	2.89	2.9E-01	6.0E-01
219	VEGFC	13	MR Egger	1.21	0.9	1.62	1.8E-01	4.2E-01	1.16	0.82	1.63	3.7E-01	6.0E-01	1.06	0.67	1.68	7.7E-01	9.7E-01	2.16	1.02	4.57	3.9E-02	2.1E-01	1.45	0.57	3.64	4.0E-01	6.0E-01
220	LPCAT2	1	Wald ratio	0.82	0.64	1.04	1.0E-01	NA	0.92	0.7	1.22	5.8E-01	NA	0.54	0.33	0.9	1.8E-02	NA	0.56	0.27	1.14	1.1E-01	NA	0.49	0.24	0.98	4.5E-02	NA
221	ASAH2	10	Inverse variance weighted	0.95	0.91	1	3.1E-02	2.3E-01	0.96	0.91	1.01	1.1E-01	5.8E-01	1	0.89	1.12	1.0E+00	5.9E-02	0.99	0.86	1.14	9.0E-01	6.5E-01	0.92	0.8	1.05	2.2E-01	9.4E-01
221	ASAH2	10	Weighted median	0.95	0.9	0.99	1.8E-02	2.3E-01	0.95	0.9	1.01	8.0E-02	5.8E-01	0.97	0.88	1.08	5.9E-01	5.9E-02	0.99	0.87	1.14	9.3E-01	6.5E-01	0.92	0.8	1.06	2.5E-01	9.4E-01
221	ASAH2	10	Weighted mode	0.95	0.9	1	4.6E-02	2.3E-01	0.96	0.9	1.01	1.2E-01	5.8E-01	0.97	0.87	1.09	5.8E-01	5.9E-02	0.99	0.85	1.16	9.4E-01	6.5E-01	0.92	0.78	1.08	2.6E-01	9.4E-01
221	ASAH2	10	MR Egger	0.93	0.88	0.99	3.7E-02	2.3E-01	0.95	0.87	1.02	1.5E-01	5.8E-01	0.93	0.81	1.07	2.6E-01	5.9E-02	0.97	0.8	1.18	7.3E-01	6.5E-01	0.92	0.76	1.12	3.5E-01	9.4E-01
222	RIPK4	1	Wald ratio	0.74	0.43	1.26	2.7E-01	NA	0.9	0.49	1.67	7.4E-01	NA	1.5	0.48	4.62	4.8E-01	NA	0.14	0.03	0.71	1.8E-02	NA	0.26	0.05	1.28	9.7E-02	NA
223	PLIN1	1	Wald ratio	0.54	0.3	0.97	3.8E-02	NA	0.79	0.39	1.62	5.3E-01	NA	0.32	0.1	0.98	4.6E-02	NA	0.56	0.09	3.55	5.4E-01	NA	0.16	0.04	0.74	1.9E-02	NA
224	MMP1	11	Inverse variance weighted	1.03	0.97	1.1	3.4E-01	4.1E-01	1.03	0.96	1.11	4.0E-01	4.6E-01	1.02	0.91	1.14	7.0E-01	3.5E-01	1.22	0.98	1.53	7.7E-02	6.7E-01	1.13	0.9	1.41	2.9E-01	4.2E-01
224	MMP1	11	Weighted median	1.01	0.96	1.07	6.3E-01	4.1E-01	1.02	0.95	1.08	6.2E-01	4.6E-01	1.02	0.9	1.15	8.0E-01	3.5E-01	1.22	1.03	1.43	1.9E-02	6.7E-01	1.18	1.01	1.39	4.2E-02	4.2E-01
224	MMP1	11	Weighted mode	1.02	0.95	1.08	5.9E-01	4.1E-01	1.01	0.94	1.09	7.6E-01	4.6E-01	1	0.87	1.16	9.5E-01	3.5E-01	1.21	1.01	1.45	4.3E-02	6.7E-01	1.18	0.98	1.42	7.3E-02	4.2E-01
224	MMP1	11	MR Egger	1	0.9	1.12	9.7E-01	4.1E-01	1	0.88	1.14	9.8E-01	4.6E-01	0.96	0.8	1.16	6.7E-01	3.5E-01	1.16	0.79	1.7	4.1E-01	6.7E-01	1.25	0.85	1.82	2.2E-01	4.2E-01
225	ANGPTL4	1	Wald ratio	1.11	1.01	1.22	3.0E-02	NA	1.14	1.02	1.28	1.9E-02	NA	1.04	0.84	1.28	7.2E-01	NA	0.98	0.73	1.31	8.9E-01	NA	1.04	0.78	1.39	7.8E-01	NA
226	IL22	2	Inverse variance weighted	1.48	1.07	2.06	1.9E-02	NA	1.69	0.77	3.71	1.9E-01	NA	0.88	0.43	1.8	7.3E-01	NA	1.84	0.29	11.69	5.2E-01	NA	1.62	0.61	4.33	3.4E-01	NA
227	GPR37	8	Inverse variance weighted	1.02	0.98	1.06	3.9E-01	9.8E-01	1.03	0.99	1.08	1.8E-01	4.8E-01	0.99	0.91	1.07	8.3E-01	3.7E-01	0.88	0.77	1.01	7.5E-02	3.5E-01	1.04	0.93	1.17	4.7E-01	2.5E-01
227	GPR37	8	Weighted median	1.02	0.98	1.06	3.8E-01	9.8E-01	1.03	0.98	1.08	1.3E-01	4.8E-01	0.98	0.9	1.07	6.8E-01	3.7E-01	0.87	0.78	0.98	1.9E-02	3.5E-01	1.06	0.94	1.19	3.3E-01	2.5E-01
227	GPR37	8	Weighted mode	1.02	0.97	1.06	3.8E-01	9.8E-01	1.03	0.98	1.09	1.9E-01	4.8E-01	0.98	0.89	1.08	6.6E-01	3.7E-01	0.87	0.76	0.99	4.1E-02	3.5E-01	1.06	0.92	1.2	3.7E-01	2.5E-01
227	GPR37	8	MR Egger	1.02	0.96	1.08	5.1E-01	9.8E-01	1.04	0.98	1.11	1.7E-01	4.8E-01	0.96	0.85	1.09	5.0E-01	3.7E-01	0.84	0.68	1.03	8.7E-02	3.5E-01	1.1	0.93	1.3	2.3E-01	2.5E-01
228	CASP7	3	Inverse variance weighted	0.85	0.72	1	5.1E-02	6.2E-01	0.84	0.63	1.11	2.1E-01	4.7E-01	0.93	0.65	1.33	7.0E-01	5.3E-01	0.62	0.4	0.95	2.9E-02	9.2E-01	1.02	0.52	2.03	9.5E-01	3.2E-01
228	CASP7	3	Weighted median	0.83	0.7	0.99	3.4E-02	6.2E-01	0.79	0.65	0.96	1.9E-02	4.7E-01	0.98	0.67	1.44	9.1E-01	5.3E-01	0.62	0.4	0.97	3.7E-02	9.2E-01	1.03	0.56	1.89	9.3E-01	3.2E-01
228	CASP7	3	Weighted mode	0.83	0.56	1.23	1.8E-01	6.2E-01	0.78	0.48	1.26	1.5E-01	4.7E-01	1	0.41	2.44	9.9E-01	5.3E-01	0.62	0.22	1.77	1.9E-01	9.2E-01	1.4	0.34	5.74	4.1E-01	3.2E-01
228	CASP7	3	MR Egger	0.81	0.21	3.1	3.0E-01	6.2E-01	0.75	0.08	6.65	3.4E-01	4.7E-01	1.07	0.05	22.19	8.3E-01	5.3E-01	0.63	0.02	19.74	3.4E-01	9.2E-01	1.59	0.01	171.44	4.3E-01	3.2E-01
229	CTRC	12	Inverse variance weighted	1	0.95	1.05	9.7E-01	5.1E-01	0.99	0.94	1.05	7.9E-01	7.1E-01	0.99	0.91	1.08	8.4E-01	6.5E-01	1.06	0.93	1.21	3.9E-01	3.2E-01	0.88	0.78	1	5.0E-02	1.2E-01
229	CTRC	12	Weighted median	1	0.95	1.04	9.1E-01	5.1E-01	0.98	0.93	1.04	5.9E-01	7.1E-01	0.98	0.89	1.09	7.6E-01	6.5E-01	1.02	0.89	1.18	7.4E-01	3.2E-01	0.84	0.73	0.97	1.9E-02	1.2E-01
229	CTRC	12	Weighted mode	1	0.95	1.05	8.8E-01	5.1E-01	0.99	0.94	1.05	8.1E-01	7.1E-01	0.99	0.89	1.11	8.3E-01	6.5E-01	1.02	0.88	1.17	8.0E-01	3.2E-01	0.84	0.72	0.97	2.5E-02	1.2E-01
229	CTRC	12	MR Egger	0.99	0.92	1.06	6.6E-01	5.1E-01	0.98	0.91	1.07	6.7E-01	7.1E-01	1.01	0.89	1.14	9.0E-01	6.5E-01	1	0.83	1.21	9.8E-01	3.2E-01	0.81	0.68	0.97	2.7E-02	1.2E-01
230	BCL2L11	10	Inverse variance weighted	0.96	0.91	1.02	1.9E-01	4.3E-02	0.97	0.89	1.05	3.9E-01	2.4E-02	1.03	0.87	1.22	7.0E-01	7.0E-01	1.01	0.85	1.21	8.8E-01	6.5E-01	0.89	0.73	1.1	2.8E-01	3.0E-01
230	BCL2L11	10	Weighted median	0.94	0.87	1.01	8.0E-02	4.3E-02	0.93	0.85	1.01	8.4E-02	2.4E-02	0.98	0.82	1.17	8.5E-01	7.0E-01	1.03	0.82	1.3	7.9E-01	6.5E-01	0.85	0.68	1.06	1.4E-01	3.0E-01
230	BCL2L11	10	Weighted mode	0.94	0.86	1.02	1.2E-01	4.3E-02	0.93	0.85	1.02	1.3E-01	2.4E-02	0.95	0.81	1.13	5.8E-01	7.0E-01	1.01	0.77	1.34	9.1E-01	6.5E-01	0.84	0.65	1.08	1.8E-01	3.0E-01
230	BCL2L11	10	MR Egger	0.85	0.74	0.97	2.6E-02	4.3E-02	0.82	0.69	0.96	1.9E-02	2.4E-02	0.97	0.64	1.47	8.8E-01	7.0E-01	0.94	0.62	1.43	7.5E-01	6.5E-01	0.74	0.46	1.18	1.7E-01	3.0E-01
231	RAP1A	2	Inverse variance weighted	0.86	0.62	1.19	3.7E-01	NA	0.85	0.59	1.24	4.0E-01	NA	0.96	0.48	1.94	9.2E-01	NA	0.31	0.12	0.83	1.9E-02	NA	1.04	0.16	6.66	9.6E-01	NA
232	HAVCR1	7	Inverse variance weighted	1.02	0.97	1.07	4.9E-01	5.1E-01	1.02	0.94	1.11	6.2E-01	3.0E-01	0.88	0.78	1.01	6.3E-02	8.5E-01	1.1	0.94	1.29	2.4E-01	8.2E-01	0.92	0.78	1.08	2.9E-01	9.7E-01
232	HAVCR1	7	Weighted median	1.01	0.95	1.06	8.0E-01	5.1E-01	1	0.94	1.07	9.4E-01	3.0E-01	0.87	0.77	0.98	1.9E-02	8.5E-01	1.13	0.96	1.33	1.5E-01	8.2E-01	0.89	0.75	1.06	1.8E-01	9.7E-01
232	HAVCR1	7	Weighted mode	1.01	0.94	1.08	8.3E-01	5.1E-01	1	0.92	1.08	9.5E-01	3.0E-01	0.87	0.75	1.02	7.4E-02	8.5E-01	1.13	0.91	1.39	2.2E-01	8.2E-01	0.89	0.72	1.1	2.3E-01	9.7E-01
232	HAVCR1	7	MR Egger	1	0.91	1.1	1.0E+00	5.1E-01	0.98	0.84	1.13	6.8E-01	3.0E-01	0.87	0.67	1.13	2.3E-01	8.5E-01	1.13	0.82	1.55	3.9E-01	8.2E-01	0.92	0.67	1.27	5.4E-01	9.7E-01
233	PON2	4	Inverse variance weighted	1.02	0.98	1.05	3.5E-01	8.6E-01	1.01	0.97	1.05	7.1E-01	4.8E-01	0.99	0.92	1.06	7.5E-01	4.5E-01	1.05	0.95	1.16	3.2E-01	6.8E-01	1.13	1.02	1.25	1.9E-02	2.9E-01
233	PON2	4	Weighted median	1.02	0.98	1.05	3.7E-01	8.6E-01	1.01	0.97	1.05	6.6E-01	4.8E-01	0.99	0.92	1.06	6.9E-01	4.5E-01	1.05	0.95	1.17	3.1E-01	6.8E-01	1.12	1.02	1.24	2.4E-02	2.9E-01
233	PON2	4	Weighted mode	1.02	0.96	1.08	5.6E-01	8.6E-01	1.01	0.95	1.07	7.6E-01	4.8E-01	0.99	0.88	1.11	9.3E-01	4.5E-01	1.05	0.89	1.25	6.1E-01	6.8E-01	1.12	0.94	1.33	2.9E-01	2.9E-01
233	PON2	4	MR Egger	1.02	0.91	1.14	5.2E-01	8.6E-01	1.03	0.9	1.18	4.7E-01	4.8E-01	0.95	0.75	1.21	4.6E-01	4.5E-01	1.08	0.76	1.54	4.2E-01	6.8E-01	1.04	0.74	1.45	6.8E-01	2.9E-01
234	PCDH7	5	Inverse variance weighted	1.14	0.96	1.35	1.2E-01	5.7E-01	1.09	0.91	1.31	3.3E-01	3.2E-01	0.97	0.67	1.4	8.6E-01	9.4E-01	1.55	0.96	2.5	7.5E-02	4.2E-01	1.3	0.67	2.5	4.3E-01	2.5E-01
234	PCDH7	5	Weighted median	1.13	0.93	1.37	2.1E-01	5.7E-01	1.07	0.86	1.33	5.2E-01	3.2E-01	1.02	0.67	1.54	9.3E-01	9.4E-01	1.99	1.12	3.54	2.0E-02	4.2E-01	1.42	0.78	2.6	2.5E-01	2.5E-01
234	PCDH7	5	Weighted mode	1.15	0.84	1.57	3.0E-01	5.7E-01	1.07	0.74	1.53	6.6E-01	3.2E-01	1.02	0.51	2.04	9.4E-01	9.4E-01	1.95	0.75	5.08	1.2E-01	4.2E-01	1.66	0.58	4.74	2.5E-01	2.5E-01
234	PCDH7	5	MR Egger	0.91	0.29	2.93	8.2E-01	5.7E-01	0.72	0.23	2.27	4.3E-01	3.2E-01	1.04	0.07	15.66	9.7E-01	9.4E-01	3.66	0.18	76.55	2.7E-01	4.2E-01	1.53	0.01	182.97	7.7E-01	2.5E-01
235	PLA2G1B	15	Inverse variance weighted	0.92	0.8	1.06	2.6E-01	4.1E-01	0.9	0.77	1.06	2.0E-01	3.2E-01	0.9	0.73	1.11	3.2E-01	8.5E-01	1.04	0.79	1.38	7.7E-01	3.7E-01	1.1	0.84	1.44	4.8E-01	5.1E-01
235	PLA2G1B	15	Weighted median	0.84	0.73	0.97	2.0E-02	4.1E-01	0.85	0.73	1	4.5E-02	3.2E-01	0.86	0.65	1.13	2.8E-01	8.5E-01	1.14	0.78	1.67	5.1E-01	3.7E-01	1.03	0.72	1.46	8.8E-01	5.1E-01
235	PLA2G1B	15	Weighted mode	0.82	0.66	1.01	5.6E-02	4.1E-01	0.82	0.68	0.98	3.5E-02	3.2E-01	0.81	0.56	1.2	2.7E-01	8.5E-01	1.18	0.66	2.12	5.4E-01	3.7E-01	1	0.6	1.67	1.0E+00	5.1E-01
235	PLA2G1B	15	MR Egger	0.79	0.52	1.2	2.5E-01	4.1E-01	0.75	0.47	1.2	2.1E-01	3.2E-01	0.95	0.5	1.8	8.6E-01	8.5E-01	1.44	0.63	3.29	3.6E-01	3.7E-01	0.78	0.36	1.7	5.1E-01	5.1E-01
236	WIF1	11	Inverse variance weighted	0.89	0.77	1.04	1.3E-01	4.6E-01	0.86	0.75	1	4.3E-02	1.8E-01	0.88	0.57	1.35	5.6E-01	5.7E-01	1.44	0.87	2.39	1.6E-01	8.1E-01	1.39	0.85	2.25	1.9E-01	5.3E-01
236	WIF1	11	Weighted median	0.86	0.73	1.02	8.7E-02	4.6E-01	0.81	0.67	0.97	2.0E-02	1.8E-01	0.84	0.56	1.26	4.0E-01	5.7E-01	1.44	0.82	2.53	2.1E-01	8.1E-01	1.24	0.72	2.12	4.4E-01	5.3E-01
236	WIF1	11	Weighted mode	0.85	0.68	1.07	1.5E-01	4.6E-01	0.79	0.59	1.04	8.6E-02	1.8E-01	0.93	0.49	1.76	8.0E-01	5.7E-01	1.83	0.64	5.29	2.3E-01	8.1E-01	1.17	0.51	2.68	7.1E-01	5.3E-01
236	WIF1	11	MR Egger	0.74	0.42	1.31	2.6E-01	4.6E-01	0.63	0.37	1.06	7.4E-02	1.8E-01	0.59	0.11	3.03	4.8E-01	5.7E-01	0.76	0.09	6.03	7.7E-01	8.1E-01	2.26	0.38	13.49	3.3E-01	5.3E-01
237	MAP2K6	2	Inverse variance weighted	1.15	0.82	1.62	4.2E-01	NA	1.09	0.77	1.53	6.4E-01	NA	2.16	1.13	4.13	2.0E-02	NA	1.13	0.44	2.93	8.0E-01	NA	0.97	0.4	2.38	9.5E-01	NA
238	TYMP	1	Wald ratio	1.17	1.02	1.35	2.8E-02	NA	1.21	1.03	1.43	2.0E-02	NA	0.98	0.72	1.33	8.9E-01	NA	1.08	0.71	1.66	7.1E-01	NA	1.09	0.71	1.66	6.9E-01	NA
239	DUSP3	2	Inverse variance weighted	0.86	0.6	1.24	4.1E-01	NA	0.91	0.61	1.35	6.5E-01	NA	2.39	1.15	4.98	2.0E-02	NA	1.06	0.1	11.04	9.6E-01	NA	0.61	0.08	4.61	6.3E-01	NA
240	PRR4	6	Inverse variance weighted	1.01	0.86	1.18	9.5E-01	2.0E-01	0.95	0.79	1.14	5.6E-01	2.4E-01	0.85	0.61	1.2	3.6E-01	8.3E-01	1.17	0.69	1.99	5.7E-01	1.7E-01	1.75	1.09	2.81	2.0E-02	9.3E-01
240	PRR4	6	Weighted median	1.05	0.85	1.29	6.7E-01	2.0E-01	0.89	0.71	1.12	3.2E-01	2.4E-01	0.77	0.5	1.18	2.2E-01	8.3E-01	0.97	0.55	1.72	9.1E-01	1.7E-01	1.79	1	3.21	4.9E-02	9.3E-01
240	PRR4	6	Weighted mode	0.86	0.65	1.14	2.3E-01	2.0E-01	0.81	0.61	1.07	1.6E-01	2.4E-01	0.74	0.39	1.41	2.8E-01	8.3E-01	0.93	0.41	2.12	8.3E-01	1.7E-01	1.85	0.77	4.47	1.9E-01	9.3E-01
240	PRR4	6	MR Egger	0.78	0.46	1.31	2.5E-01	2.0E-01	0.73	0.4	1.32	2.1E-01	2.4E-01	0.93	0.3	2.9	8.6E-01	8.3E-01	0.5	0.1	2.38	2.8E-01	1.7E-01	1.83	0.38	8.74	3.4E-01	9.3E-01
241	TP53INP1	2	Inverse variance weighted	1.23	0.94	1.6	1.3E-01	NA	1.49	1.06	2.1	2.0E-02	NA	0.96	0.54	1.69	8.8E-01	NA	0.76	0.35	1.67	5.0E-01	NA	0.75	0.32	1.77	5.1E-01	NA
242	UROS	2	Inverse variance weighted	0.92	0.75	1.13	4.2E-01	NA	0.91	0.71	1.15	4.2E-01	NA	0.59	0.37	0.92	2.0E-02	NA	0.81	0.43	1.51	5.1E-01	NA	1.29	0.59	2.82	5.3E-01	NA
243	PDGFRA	7	Inverse variance weighted	1.04	0.91	1.19	5.5E-01	1.5E-01	1.09	0.97	1.22	1.7E-01	1.4E-01	1.04	0.86	1.27	6.6E-01	6.0E-01	0.81	0.6	1.09	1.6E-01	9.0E-01	1.1	0.84	1.44	5.0E-01	8.0E-01
243	PDGFRA	7	Weighted median	1.08	0.98	1.2	1.1E-01	1.5E-01	1.15	1.02	1.29	2.0E-02	1.4E-01	1.08	0.87	1.32	4.9E-01	6.0E-01	0.79	0.58	1.06	1.1E-01	9.0E-01	1.13	0.85	1.51	4.0E-01	8.0E-01
243	PDGFRA	7	Weighted mode	1.09	0.96	1.24	1.5E-01	1.5E-01	1.15	0.99	1.35	6.8E-02	1.4E-01	1.08	0.82	1.41	5.3E-01	6.0E-01	0.79	0.55	1.12	1.5E-01	9.0E-01	1.13	0.77	1.66	4.6E-01	8.0E-01
243	PDGFRA	7	MR Egger	1.14	0.93	1.41	1.6E-01	1.5E-01	1.19	0.98	1.43	6.6E-02	1.4E-01	1.1	0.77	1.56	5.3E-01	6.0E-01	0.82	0.45	1.49	4.4E-01	9.0E-01	1.14	0.7	1.85	5.3E-01	8.0E-01
244	SDCCAG8	2	Inverse variance weighted	0.93	0.83	1.05	2.4E-01	NA	0.91	0.79	1.04	1.6E-01	NA	1.15	0.89	1.49	2.8E-01	NA	1.06	0.74	1.52	7.7E-01	NA	0.66	0.46	0.94	2.0E-02	NA
245	GP5	5	Inverse variance weighted	1.18	1	1.39	5.3E-02	8.5E-01	1.25	1.04	1.52	2.0E-02	8.8E-01	1.05	0.68	1.61	8.3E-01	9.1E-01	1.23	0.69	2.23	4.6E-01	2.7E-01	1.45	0.8	2.63	2.2E-01	9.9E-01
245	GP5	5	Weighted median	1.19	0.97	1.45	9.9E-02	8.5E-01	1.25	1	1.57	5.4E-02	8.8E-01	1.09	0.72	1.65	6.9E-01	9.1E-01	1.12	0.59	2.12	7.4E-01	2.7E-01	1.64	0.86	3.14	1.4E-01	9.9E-01
245	GP5	5	Weighted mode	1.21	0.89	1.66	1.6E-01	8.5E-01	1.23	0.84	1.81	2.1E-01	8.8E-01	1.1	0.55	2.18	7.4E-01	9.1E-01	1.05	0.35	3.14	9.3E-01	2.7E-01	1.66	0.64	4.33	2.2E-01	9.9E-01
245	GP5	5	MR Egger	1.27	0.39	4.16	5.7E-01	8.5E-01	1.17	0.3	4.63	7.4E-01	8.8E-01	1.07	0.08	14.06	9.4E-01	9.1E-01	0.26	0.01	11.72	3.5E-01	2.7E-01	1.49	0.01	199.69	8.1E-01	9.9E-01
246	MATN3	4	Inverse variance weighted	1.02	0.86	1.21	8.0E-01	3.5E-01	1.04	0.84	1.28	7.3E-01	3.4E-01	0.98	0.86	1.12	7.8E-01	4.0E-01	1.11	0.96	1.27	1.6E-01	6.8E-01	0.93	0.73	1.18	5.4E-01	1.4E-01
246	MATN3	4	Weighted median	1.04	0.99	1.1	9.0E-02	3.5E-01	1.07	1.01	1.13	2.0E-02	3.4E-01	1	0.9	1.1	9.5E-01	4.0E-01	1.11	0.96	1.27	1.6E-01	6.8E-01	0.95	0.82	1.09	4.7E-01	1.4E-01
246	MATN3	4	Weighted mode	1.04	0.96	1.13	3.9E-01	3.5E-01	1.07	0.98	1.16	1.9E-01	3.4E-01	1	0.85	1.18	9.7E-01	4.0E-01	1.1	0.88	1.38	2.8E-01	6.8E-01	0.96	0.76	1.22	6.3E-01	1.4E-01
246	MATN3	4	MR Egger	1.12	0.69	1.83	4.1E-01	3.5E-01	1.17	0.64	2.14	3.7E-01	3.4E-01	1.05	0.7	1.59	6.4E-01	4.0E-01	1.07	0.69	1.66	5.9E-01	6.8E-01	1.12	0.7	1.77	4.1E-01	1.4E-01
247	SARG	4	Inverse variance weighted	1.03	0.84	1.25	8.0E-01	5.7E-01	1.1	0.85	1.42	4.7E-01	6.7E-01	0.75	0.48	1.16	1.9E-01	5.2E-01	1.4	0.76	2.57	2.8E-01	7.6E-01	1.55	0.48	5	4.6E-01	2.1E-01
247	SARG	4	Weighted median	1.03	0.8	1.31	8.3E-01	5.7E-01	1.12	0.84	1.49	4.3E-01	6.7E-01	0.71	0.42	1.19	1.9E-01	5.2E-01	1.41	0.68	2.9	3.5E-01	7.6E-01	2.7	1.16	6.28	2.1E-02	2.1E-01
247	SARG	4	Weighted mode	0.98	0.54	1.75	9.0E-01	5.7E-01	0.92	0.43	1.94	7.4E-01	6.7E-01	0.67	0.26	1.74	4.6E-01	5.2E-01	1.5	0.32	6.97	4.6E-01	7.6E-01	2.83	0.58	13.83	1.3E-01	2.1E-01
247	SARG	4	MR Egger	1.19	0.42	3.36	5.5E-01	5.7E-01	0.93	0.2	4.37	8.7E-01	6.7E-01	1.09	0.11	10.92	8.9E-01	5.2E-01	1.11	0.05	25.57	9.0E-01	7.6E-01	8.95	0.08	1026.65	1.9E-01	2.1E-01
248	CELA2A	17	Inverse variance weighted	0.92	0.85	1.01	7.0E-02	5.5E-01	0.94	0.83	1.06	2.9E-01	5.3E-01	0.9	0.76	1.06	2.0E-01	9.1E-01	0.88	0.71	1.1	2.7E-01	8.1E-01	0.82	0.66	1.01	6.5E-02	6.8E-01
248	CELA2A	17	Weighted median	0.89	0.8	0.99	3.1E-02	5.5E-01	0.88	0.78	1	5.0E-02	5.3E-01	0.83	0.67	1.03	8.8E-02	9.1E-01	0.92	0.66	1.28	6.3E-01	8.1E-01	0.79	0.59	1.05	1.0E-01	6.8E-01
248	CELA2A	17	Weighted mode	0.83	0.71	0.97	2.1E-02	5.5E-01	0.84	0.72	0.98	2.8E-02	5.3E-01	0.84	0.64	1.11	2.0E-01	9.1E-01	0.98	0.66	1.45	9.0E-01	8.1E-01	0.97	0.67	1.4	8.6E-01	6.8E-01
248	CELA2A	17	MR Egger	0.89	0.76	1.04	1.4E-01	5.5E-01	0.89	0.71	1.11	2.7E-01	5.3E-01	0.89	0.66	1.2	4.1E-01	9.1E-01	0.85	0.58	1.25	3.9E-01	8.1E-01	0.87	0.59	1.27	4.3E-01	6.8E-01
249	TBCC	5	Inverse variance weighted	1.09	0.92	1.28	3.2E-01	7.5E-01	1.03	0.86	1.25	7.3E-01	8.9E-01	1.7	1.04	2.78	3.4E-02	9.0E-01	1.13	0.68	1.87	6.4E-01	9.9E-01	1.1	0.62	1.93	7.5E-01	6.7E-01
249	TBCC	5	Weighted median	1.07	0.88	1.31	4.9E-01	7.5E-01	1.02	0.82	1.28	8.4E-01	8.9E-01	1.71	1.08	2.69	2.1E-02	9.0E-01	1.05	0.58	1.92	8.7E-01	9.9E-01	0.93	0.52	1.66	7.9E-01	6.7E-01
249	TBCC	5	Weighted mode	1.08	0.78	1.49	5.5E-01	7.5E-01	1.04	0.74	1.46	8.3E-01	8.9E-01	1.57	0.77	3.21	1.6E-01	9.0E-01	1.02	0.39	2.64	9.6E-01	9.9E-01	0.9	0.35	2.33	7.8E-01	6.7E-01
249	TBCC	5	MR Egger	1.05	0.67	1.64	7.6E-01	7.5E-01	1.02	0.64	1.63	9.2E-01	8.9E-01	1.62	0.37	7.07	3.7E-01	9.0E-01	1.13	0.27	4.78	8.1E-01	9.9E-01	0.92	0.19	4.4	8.7E-01	6.7E-01
250	FKBP5	3	Inverse variance weighted	0.9	0.79	1.03	1.2E-01	6.5E-01	0.84	0.72	0.97	2.1E-02	4.7E-01	0.91	0.69	1.2	4.9E-01	8.4E-01	1.07	0.73	1.58	7.2E-01	2.8E-01	0.96	0.63	1.46	8.4E-01	3.7E-01
250	FKBP5	3	Weighted median	0.91	0.8	1.03	1.4E-01	6.5E-01	0.85	0.73	0.99	3.9E-02	4.7E-01	0.89	0.67	1.19	4.4E-01	8.4E-01	1.07	0.71	1.61	7.5E-01	2.8E-01	0.97	0.65	1.45	8.9E-01	3.7E-01
250	FKBP5	3	Weighted mode	0.91	0.69	1.22	3.1E-01	6.5E-01	0.8	0.6	1.23	2.1E-01	4.7E-01	0.89	0.47	1.7	5.3E-01	8.4E-01	1.07	0.44	2.63	7.7E-01	2.8E-01	1.06	0.44	2.56	8.0E-01	3.7E-01
250	FKBP5	3	MR Egger	0.96	0.23	3.97	7.5E-01	6.5E-01	0.94	0.18	4.89	7.3E-01	4.7E-01	0.86	0.04	18.92	6.5E-01	8.4E-01	1.06	0	257.6	9.1E-01	2.8E-01	1.44	0.02	100.44	4.7E-01	3.7E-01
251	RSPO1	8	Inverse variance weighted	0.95	0.84	1.07	4.1E-01	1.9E-02	0.98	0.83	1.15	8.2E-01	1.1E-02	1	0.76	1.31	9.9E-01	5.7E-01	0.96	0.72	1.28	7.6E-01	3.5E-01	0.97	0.66	1.42	8.6E-01	5.8E-01
251	RSPO1	8	Weighted median	0.92	0.81	1.04	1.7E-01	1.9E-02	0.92	0.79	1.07	2.9E-01	1.1E-02	1.08	0.85	1.36	5.4E-01	5.7E-01	0.86	0.62	1.19	3.5E-01	3.5E-01	0.89	0.63	1.26	5.3E-01	5.8E-01
251	RSPO1	8	Weighted mode	0.84	0.72	0.98	2.9E-02	1.9E-02	0.84	0.71	0.99	4.4E-02	1.1E-02	1.06	0.8	1.42	6.3E-01	5.7E-01	0.85	0.55	1.29	3.8E-01	3.5E-01	0.82	0.54	1.26	3.2E-01	5.8E-01
251	RSPO1	8	MR Egger	0.8	0.66	0.95	2.1E-02	1.9E-02	0.78	0.63	0.96	2.6E-02	1.1E-02	1.1	0.65	1.88	6.7E-01	5.7E-01	0.81	0.47	1.4	3.8E-01	3.5E-01	0.84	0.39	1.8	6.1E-01	5.8E-01
252	NAAA	14	Inverse variance weighted	1.02	0.97	1.06	4.2E-01	9.9E-01	1	0.96	1.06	7.2E-01	6.8E-01	1.11	1.02	1.22	2.3E-02	3.6E-01	1.04	0.89	1.21	6.1E-01	8.8E-01	1.09	0.94	1.23	2.6E-01	6.0E-01
252	NAAA	14	Weighted median	1.02	0.97	1.06	4.5E-01	9.9E-01	1	0.95	1.06	9.0E-01	6.8E-01	1.12	1.02	1.23	2.1E-02	3.6E-01	1.02	0.89	1.17	7.7E-01	8.8E-01	1.08	0.95	1.24	2.3E-01	6.0E-01
252	NAAA	14	Weighted mode	1.02	0.97	1.07	4.4E-01	9.9E-01	1	0.95	1.06	8.8E-01	6.8E-01	1.13	1.01	1.26	3.1E-02	3.6E-01	1.02	0.89	1.18	7.5E-01	8.8E-01	1.09	0.94	1.26	2.5E-01	6.0E-01
252	NAAA	14	MR Egger	1.02	0.96	1.08	5.3E-01	9.9E-01	1	0.93	1.08	9.7E-01	6.8E-01	1.12	0.99	1.27	6.7E-02	3.6E-01	1.03	0.83	1.28	7.8E-01	8.8E-01	1.05	0.87	1.27	5.7E-01	6.0E-01
253	TACSTD2	13	Inverse variance weighted	1.03	1	1.06	9.1E-02	4.0E-01	1.04	1	1.09	2.9E-02	4.4E-01	0.98	0.91	1.05	5.6E-01	4.1E-02	1.03	0.91	1.16	6.4E-01	3.3E-01	1.01	0.91	1.12	9.0E-01	7.0E-01
253	TACSTD2	13	Weighted median	1.03	0.99	1.07	1.6E-01	4.0E-01	1.05	1.01	1.09	2.1E-02	4.4E-01	0.95	0.87	1.02	1.7E-01	4.1E-02	1.02	0.91	1.14	7.9E-01	3.3E-01	1.01	0.91	1.12	8.9E-01	7.0E-01
253	TACSTD2	13	Weighted mode	1.03	0.99	1.07	1.7E-01	4.0E-01	1.05	1	1.1	4.4E-02	4.4E-01	0.95	0.87	1.04	2.8E-01	4.1E-02	1.02	0.9	1.15	7.7E-01	3.3E-01	1.02	0.89	1.16	7.8E-01	7.0E-01

253	TACSTD2	13	MR Egger	1.02	0.97	1.07	4.5E-01	4.0E-01	1.06	1	1.12	4.9E-02	4.4E-01	0.91	0.82	1.01	8.4E-02	4.1E-02	0.98	0.83	1.16	7.9E-01	3.3E-01	0.99	0.85	1.16	8.8E-01	7.0E-01
254	CCN3	2	Inverse variance weighted	0.99	0.89	1.1	8.6E-01	NA	1	0.89	1.13	9.9E-01	NA	0.95	0.76	1.19	6.6E-01	NA	1.46	1.06	2.01	2.2E-02	NA	1.09	0.79	1.49	6.1E-01	NA
255	ITGB7	14	Inverse variance weighted	1.04	0.94	1.15	4.2E-01	6.2E-01	1.03	0.91	1.18	6.1E-01	7.9E-01	1.01	0.89	1.14	9.2E-01	7.5E-01	1.04	0.9	1.21	5.9E-01	2.6E-01	1.18	1.02	1.37	2.2E-02	8.6E-01
255	ITGB7	14	Weighted median	1.04	0.97	1.11	2.7E-01	6.2E-01	1.02	0.94	1.1	6.0E-01	7.9E-01	1.05	0.92	1.21	4.6E-01	7.5E-01	1.02	0.84	1.24	8.5E-01	2.6E-01	1.16	0.97	1.39	1.1E-01	8.6E-01
255	ITGB7	14	Weighted mode	1.04	0.96	1.13	2.8E-01	6.2E-01	1.04	0.96	1.14	3.0E-01	7.9E-01	1.02	0.88	1.17	8.3E-01	7.5E-01	1.02	0.82	1.26	8.7E-01	2.6E-01	1.16	0.94	1.44	1.5E-01	8.6E-01
255	ITGB7	14	MR Egger	1.01	0.83	1.22	9.4E-01	6.2E-01	1.01	0.79	1.29	9.3E-01	7.9E-01	0.98	0.77	1.25	8.5E-01	7.5E-01	0.93	0.71	1.21	5.6E-01	2.6E-01	1.16	0.9	1.51	2.3E-01	8.6E-01
256	PCSK7	2	Inverse variance weighted	1.09	1.01	1.18	2.2E-02	NA	1.1	1.01	1.21	2.9E-02	NA	1.14	0.96	1.35	1.3E-01	NA	0.96	0.74	1.23	7.3E-01	NA	1.1	0.84	1.43	4.9E-01	NA
257	SFTPD	11	Inverse variance weighted	0.96	0.9	1.03	3.0E-01	5.0E-01	0.95	0.88	1.03	2.0E-01	6.7E-01	0.97	0.87	1.08	5.9E-01	5.8E-01	1.04	0.95	1.14	4.1E-01	7.9E-01	1.01	0.89	1.15	8.8E-01	3.7E-01
257	SFTPD	11	Weighted median	0.97	0.94	1	5.8E-02	5.0E-01	0.96	0.92	0.99	2.2E-02	6.7E-01	0.96	0.9	1.03	2.5E-01	5.8E-01	1.05	0.95	1.16	3.2E-01	7.9E-01	1.03	0.94	1.14	4.9E-01	3.7E-01
257	SFTPD	11	Weighted mode	0.97	0.93	1.01	8.8E-02	5.0E-01	0.96	0.92	1	4.1E-02	6.7E-01	0.97	0.9	1.05	4.4E-01	5.8E-01	1.05	0.94	1.17	3.3E-01	7.9E-01	1.03	0.92	1.14	6.0E-01	3.7E-01
257	SFTPD	11	MR Egger	0.98	0.89	1.09	7.1E-01	5.0E-01	0.97	0.86	1.08	5.0E-01	6.7E-01	0.99	0.84	1.18	9.4E-01	5.8E-01	1.05	0.92	1.2	4.3E-01	7.9E-01	1.06	0.88	1.28	5.1E-01	3.7E-01
258	DCXR	5	Inverse variance weighted	0.93	0.79	1.09	3.6E-01	9.9E-01	1.02	0.85	1.23	8.1E-01	9.6E-01	0.9	0.63	1.28	5.6E-01	2.9E-01	0.57	0.36	0.92	2.3E-02	8.1E-01	1.04	0.63	1.71	8.9E-01	7.1E-01
258	DCXR	5	Weighted median	0.91	0.75	1.1	3.4E-01	9.9E-01	0.98	0.77	1.24	8.4E-01	9.6E-01	0.88	0.57	1.36	5.7E-01	2.9E-01	0.56	0.32	0.98	4.4E-02	8.1E-01	1.04	0.58	1.88	8.9E-01	7.1E-01
258	DCXR	5	Weighted mode	0.9	0.65	1.24	4.0E-01	9.9E-01	0.95	0.66	1.39	7.4E-01	9.6E-01	0.75	0.27	2.05	4.7E-01	2.9E-01	0.56	0.21	1.51	1.8E-01	8.1E-01	1.18	0.4	3.48	6.9E-01	7.1E-01
258	DCXR	5	MR Egger	0.93	0.58	1.47	6.3E-01	9.9E-01	1.01	0.54	1.89	9.5E-01	9.6E-01	1.3	0.43	3.87	5.0E-01	2.9E-01	0.52	0.14	1.99	2.2E-01	8.1E-01	1.22	0.27	5.54	7.1E-01	7.1E-01
259	CCL25	9	Inverse variance weighted	0.96	0.93	1	6.3E-02	5.5E-01	0.99	0.94	1.05	8.2E-01	6.7E-01	0.91	0.85	0.99	2.3E-02	8.7E-01	0.94	0.84	1.04	2.4E-01	4.1E-01	0.95	0.84	1.07	4.0E-01	1.1E-01
259	CCL25	9	Weighted median	0.97	0.93	1	6.9E-02	5.5E-01	1	0.96	1.04	9.9E-01	6.7E-01	0.94	0.86	1.03	1.9E-01	8.7E-01	1	0.88	1.14	9.9E-01	4.1E-01	0.96	0.86	1.07	4.8E-01	1.1E-01
259	CCL25	9	Weighted mode	0.97	0.92	1.01	1.2E-01	5.5E-01	1	0.95	1.05	9.5E-01	6.7E-01	0.92	0.83	1.02	9.5E-02	8.7E-01	0.96	0.84	1.09	4.6E-01	4.1E-01	0.96	0.86	1.09	4.9E-01	1.1E-01
259	CCL25	9	MR Egger	0.98	0.91	1.05	4.4E-01	5.5E-01	1.01	0.91	1.12	8.7E-01	6.7E-01	0.92	0.81	1.05	1.8E-01	8.7E-01	0.98	0.82	1.18	8.4E-01	4.1E-01	1.05	0.87	1.25	5.6E-01	1.1E-01
260	CD79B	16	Inverse variance weighted	0.93	0.81	1.06	2.6E-01	1.8E-01	0.93	0.8	1.08	3.2E-01	1.1E-01	0.95	0.78	1.16	6.4E-01	2.7E-01	1.12	0.81	1.53	5.0E-01	3.8E-01	0.76	0.6	0.96	2.3E-02	2.7E-01
260	CD79B	16	Weighted median	0.97	0.83	1.13	6.9E-01	1.8E-01	0.97	0.81	1.15	7.0E-01	1.1E-01	0.81	0.64	1.03	8.3E-02	2.7E-01	1.07	0.78	1.48	6.6E-01	3.8E-01	0.74	0.54	1.01	6.2E-02	2.7E-01
260	CD79B	16	Weighted mode	0.71	0.5	1.02	6.1E-02	1.8E-01	1.17	0.57	2.39	6.4E-01	1.1E-01	0.81	0.62	1.07	1.2E-01	2.7E-01	1.07	0.78	1.48	6.4E-01	3.8E-01	0.72	0.5	1.04	7.6E-02	2.7E-01
260	CD79B	16	MR Egger	0.81	0.64	1.04	8.7E-02	1.8E-01	0.78	0.59	1.02	6.7E-02	1.1E-01	0.81	0.55	1.18	2.4E-01	2.7E-01	0.9	0.49	1.66	7.2E-01	3.8E-01	0.92	0.59	1.46	7.1E-01	2.7E-01
261	NCR3LG1	9	Inverse variance weighted	0.99	0.89	1.1	8.1E-01	9.0E-01	0.97	0.86	1.11	6.9E-01	9.0E-01	0.98	0.89	1.09	7.2E-01	9.0E-01	1.14	0.98	1.31	8.0E-02	4.5E-01	0.89	0.76	1.06	1.9E-01	7.6E-01
261	NCR3LG1	9	Weighted median	1.07	1.01	1.14	2.3E-02	9.0E-01	1.07	0.99	1.15	9.5E-02	9.0E-01	1.04	0.91	1.18	6.1E-01	9.0E-01	1.18	1	1.4	5.2E-02	4.5E-01	0.88	0.72	1.07	2.1E-01	7.6E-01
261	NCR3LG1	9	Weighted mode	1.08	1	1.16	5.7E-02	9.0E-01	1.07	0.97	1.18	1.3E-01	9.0E-01	1.05	0.9	1.23	4.8E-01	9.0E-01	1.19	0.99	1.43	6.3E-02	4.5E-01	0.83	0.66	1.03	8.2E-02	7.6E-01
261	NCR3LG1	9	MR Egger	1	0.79	1.26	9.8E-01	9.0E-01	0.96	0.73	1.27	7.6E-01	9.0E-01	0.97	0.79	1.2	7.6E-01	9.0E-01	1.23	0.92	1.64	1.4E-01	4.5E-01	0.93	0.65	1.34	6.5E-01	7.6E-01
262	CKMT1A_C	6	Inverse variance weighted	1.04	0.87	1.24	6.9E-01	3.6E-01	1.14	0.97	1.34	1.1E-01	3.4E-01	0.99	0.66	1.48	9.5E-01	2.4E-01	0.69	0.41	1.17	1.7E-01	4.3E-01	1.06	0.71	1.58	7.7E-01	7.5E-01
262	CKMT1A_C	6	Weighted median	1.15	0.98	1.34	8.0E-02	3.6E-01	1.22	1.03	1.46	2.3E-02	3.4E-01	1.32	0.89	1.96	1.6E-01	2.4E-01	0.63	0.4	0.99	4.4E-02	4.3E-01	1.16	0.74	1.83	5.2E-01	7.5E-01
262	CKMT1A_C	6	Weighted mode	1.12	0.91	1.38	2.1E-01	3.6E-01	1.21	0.95	1.53	9.5E-02	3.4E-01	1.29	0.77	2.18	2.6E-01	2.4E-01	0.6	0.32	1.12	8.8E-02	4.3E-01	1.18	0.57	2.44	5.9E-01	7.5E-01
262	CKMT1A_C	6	MR Egger	1.22	0.74	2.01	3.3E-01	3.6E-01	1.33	0.84	2.1	1.6E-01	3.4E-01	1.56	0.53	4.58	3.1E-01	2.4E-01	0.46	0.1	2.05	2.2E-01	4.3E-01	1.22	0.35	4.28	6.9E-01	7.5E-01
263	NUCB2	4	Inverse variance weighted	1.06	1	1.13	6.2E-02	6.9E-01	1.07	0.97	1.18	1.8E-01	1.6E-01	1.13	0.91	1.42	2.7E-01	1.2E-01	1.11	0.81	1.51	5.2E-01	4.8E-01	1.05	0.8	1.38	7.4E-01	6.1E-01
263	NUCB2	4	Weighted median	1.06	0.99	1.13	9.1E-02	6.9E-01	1.06	0.98	1.14	1.3E-01	1.6E-01	1.17	1.02	1.35	2.3E-02	1.2E-01	1.12	0.91	1.37	2.9E-01	4.8E-01	1.03	0.84	1.26	7.7E-01	6.1E-01
263	NUCB2	4	Weighted mode	1.06	0.94	1.19	2.2E-01	6.9E-01	1.05	0.93	1.19	2.7E-01	1.6E-01	1.18	0.92	1.5	1.2E-01	1.2E-01	1.13	0.81	1.57	3.2E-01	4.8E-01	1.02	0.74	1.42	8.4E-01	6.1E-01
263	NUCB2	4	MR Egger	1.05	0.85	1.28	4.5E-01	6.9E-01	0.98	0.77	1.24	7.4E-01	1.6E-01	1.38	0.88	2.15	9.0E-02	1.2E-01	1.29	0.45	3.66	4.1E-01	4.8E-01	0.95	0.35	2.57	8.4E-01	6.1E-01
264	IGDCC4	3	Inverse variance weighted	0.96	0.85	1.08	4.6E-01	4.4E-01	1	0.86	1.15	9.7E-01	4.1E-01	0.85	0.62	1.16	2.9E-01	8.6E-01	0.64	0.44	0.94	2.3E-02	5.9E-01	0.78	0.54	1.12	1.8E-01	8.5E-01
264	IGDCC4	3	Weighted median	0.97	0.86	1.09	5.7E-01	4.4E-01	1.01	0.88	1.16	9.0E-01	4.1E-01	0.81	0.61	1.06	1.3E-01	8.6E-01	0.64	0.44	0.95	2.6E-02	5.9E-01	0.8	0.55	1.16	2.4E-01	8.5E-01
264	IGDCC4	3	Weighted mode	0.98	0.74	1.28	7.3E-01	4.4E-01	1.02	0.74	1.4	8.0E-01	4.1E-01	0.8	0.43	1.51	2.8E-01	8.6E-01	0.68	0.28	1.68	2.1E-01	5.9E-01	0.8	0.34	1.9	3.9E-01	8.5E-01
264	IGDCC4	3	MR Egger	1	0.42	2.36	9.5E-01	4.4E-01	1.05	0.39	2.81	6.6E-01	4.1E-01	0.82	0.03	19.7	8.5E-01	8.6E-01	0.7	0.04	11.86	3.5E-01	5.9E-01	0.8	0.05	11.87	4.8E-01	8.5E-01
265	PRKG1	4	Inverse variance weighted	1	0.89	1.13	9.6E-01	5.6E-01	1.08	0.91	1.29	3.7E-01	5.2E-01	0.75	0.57	0.97	2.8E-02	4.9E-01	1.14	0.79	1.65	4.9E-01	3.2E-01	0.96	0.63	1.46	8.5E-01	9.0E-01
265	PRKG1	4	Weighted median	1.02	0.9	1.16	7.9E-01	5.6E-01	1.09	0.93	1.28	2.7E-01	5.2E-01	0.72	0.54	0.96	2.3E-02	4.9E-01	1.27	0.85	1.88	2.4E-01	3.2E-01	0.93	0.63	1.38	7.2E-01	9.0E-01
265	PRKG1	4	Weighted mode	1.02	0.82	1.28	7.5E-01	5.6E-01	1.12	0.86	1.46	2.8E-01	5.2E-01	0.71	0.44	1.15	1.1E-01	4.9E-01	1.29	0.64	2.64	3.3E-01	3.2E-01	0.97	0.5	1.9	8.9E-01	9.0E-01
265	PRKG1	4	MR Egger	1.07	0.66	1.74	6.1E-01	5.6E-01	1.21	0.58	2.54	3.8E-01	5.2E-01	0.63	0.22	1.78	2.0E-01	4.9E-01	1.66	0.38	7.27	2.8E-01	3.2E-01	1.02	0.13	7.74	9.8E-01	9.0E-01
266	CTSF	11	Inverse variance weighted	0.97	0.86	1.08	5.5E-01	9.9E-01	0.95	0.85	1.07	4.3E-01	8.0E-01	0.8	0.63	1.03	8.0E-02	5.1E-01	1.35	1.01	1.81	4.5E-02	9.1E-01	0.85	0.59	1.24	4.1E-01	1.9E-01
266	CTSF	11	Weighted median	1.01	0.9	1.15	8.2E-01	9.9E-01	0.98	0.85	1.12	7.2E-01	8.0E-01	0.9	0.69	1.16	4.1E-01	5.1E-01	1.54	1.06	2.23	2.3E-02	9.1E-01	0.78	0.55	1.11	1.7E-01	1.9E-01
266	CTSF	11	Weighted mode	1	0.87	1.14	9.9E-01	9.9E-01	0.98	0.82	1.16	7.8E-01	8.0E-01	0.89	0.67	1.19	4.0E-01	5.1E-01	1.51	0.97	2.33	6.3E-02	9.1E-01	0.78	0.52	1.18	2.1E-01	1.9E-01
266	CTSF	11	MR Egger	0.97	0.76	1.23	7.5E-01	9.9E-01	0.93	0.72	1.2	5.4E-01	8.0E-01	0.92	0.54	1.55	7.2E-01	5.1E-01	1.39	0.73	2.62	2.7E-01	9.1E-01	0.57	0.27	1.23	1.3E-01	1.9E-01
267	HMOX1	15	Inverse variance weighted	1.05	0.95	1.12	4.2E-01	9.6E-01	1.01	0.9	1.1	9.2E-01	4.9E-01	1.05	0.89	1.24	5.4E-01	5.2E-01	1.09	0.87	1.36	4.7E-01	9.0E-01	1.32	1.03	1.68	2.9E-02	7.9E-01
267	HMOX1	15	Weighted median	1.02	0.93	1.13	6.4E-01	9.6E-01	1.02	0.91	1.14	7.6E-01	4.9E-01	1.07	0.86	1.34	5.3E-01	5.2E-01	0.98	0.72	1.33	8.9E-01	9.0E-01	1.48	1.05	2.08	2.4E-02	7.9E-01
267	HMOX1	15	Weighted mode	1.01	0.89	1.15	8.4E-01	9.6E-01	1	0.88	1.15	9.5E-01	4.9E-01	1.08	0.83	1.41	5.3E-01	5.2E-01	0.97	0.67	1.4	8.6E-01	9.0E-01	1.64	0.91	2.94	9.1E-02	7.9E-01
267	HMOX1	15	MR Egger	1.03	0.85	1.24	7.4E-01	9.6E-01	1.07	0.84	1.35	5.8E-01	4.9E-01	0.95	0.66	1.37	7.9E-01	5.2E-01	1.12	0.68	1.84	6.4E-01	9.0E-01	1.24	0.7	2.17	4.3E-01	7.9E-01
268	ARHGAP25	2	Inverse variance weighted	0.92	0.69	1.23	5.9E-01	NA	1.05	0.63	1.75	8.5E-01	NA	0.61	0.36	1.04	6.9E-02	NA	0.43	0.2	0.89	2.4E-02	NA	0.65	0.31	1.35	2.4E-01	NA
269	CNPY4	2	Inverse variance weighted	1.13	0.97	1.31	1.2E-01	NA	1.02	0.86	1.2	8.6E-01	NA	1.39	1.01	1.91	4.6E-02	NA	1.41	0.89	2.23	1.5E-01	NA	1.69	1.07	2.67	2.4E-02	NA
270	MAPKAPK2	1	Wald ratio	0.82	0.68	0.99	3.9E-02	NA	0.78	0.63	0.97	2.4E-02	NA	0.79	0.53	1.19	2.7E-01	NA	0.76	0.43	1.35	3.5E-01	NA	1.27	0.72	2.22	4.1E-01	NA
271	AMBP	8	Inverse variance weighted	1.16	1.02	1.31	2.4E-02	7.9E-01	1.11	0.96	1.29	1.5E-01	3.2E-01	0.96	0.73	1.25	7.4E-01	2.2E-01	1.07	0.73	1.56	7.3E-01	5.6E-01	1.37	0.93	2.01	1.1E-01	4.9E-01
271	AMBP	8	Weighted median	1.12	0.96	1.31	1.5E-01	7.9E-01	1.03	0.85	1.25	7.3E-01	3.2E-01	0.89	0.64	1.24	5.0E-01	2.2E-01	1.17	0.72	1.93	5.3E-01	5.6E-01	1.34	0.84	2.13	2.2E-01	4.9E-01
271	AMBP	8	Weighted mode	1.13	0.91	1.4	2.3E-01	7.9E-01	0.99	0.73	1.33	9.1E-01	3.2E-01	0.86	0.57	1.31	4.3E-01	2.2E-01	1.24	0.56	2.77	5.4E-01	5.6E-01	1.31	0.66	2.63	3.8E-01	4.9E-01
271	AMBP	8	MR Egger	1.13	0.84	1.5	3.5E-01	7.9E-01	1.26	0.9	1.77	1.4E-01	3.2E-01	0.73	0.41	1.31	2.6E-01	2.2E-01	0.89	0.38	2.11	7.6E-01	5.6E-01	1.74	0.69	4.38	1.9E-01	4.9E-01
272	IL12RB1	4	Inverse variance weighted	1.05	0.96	1.15	3.1E-01	1.2E-01	1.07	1.01	1.15	2.9E-02	4.4E-01	0.96	0.77	1.2	7.3E-01	1.5E-01	1.04	0.79	1.37	7.8E-01	1.2E-01	0.96	0.75	1.22	7.2E-01	6.1E-01
272	IL12RB1	4	Weighted median	1.07	1.01	1.13	2.8E-02	1.2E-01	1.08	1.01	1.16	2.4E-02	4.4E-01	1	0.88	1.14	9.9E-01	1.5E-01	1.09	0.91	1.3	3.7E-01	1.2E-01	0.99	0.83	1.18	8.8E-01	6.1E-01
272	IL12RB1	4	Weighted mode	1.07	0.97	1.18	1.3E-01	1.2E-01	1.08	0.96	1.22	1.2E-01	4.4E-01	1	0.82	1.23	9.4E-01	1.5E-01	1.09	0.81	1.46	4.2E-01	1.2E-01	1.01	0.75	1.34	9.6E-01	6.1E-01
272	IL12RB1	4	MR Egger	1.18	0.93	1.5	9.3E-02	1.2E-01	1.13	0.86	1.49	1.9E-01	4.4E-01	1.26	0.7	2.3	2.3E-01	1.5E-01	1.51	0.74	3.1	1.3E-01	1.2E-01	1.1	0.35	3.5	7.6E-01	6.1E-01
273	SSC4D	9	Inverse variance weighted	0.99	0.92	1.06	7.1E-01	1.1E-01	0.99	0.91	1.07	7.8E-01	2.2E-01	0.9	0.77	1.04	1.6E-01	7.0E-02	1.25	1.03	1.52	2.7E-02	4.4E-01	1.03	0.84	1.26	8.0E-01	9.5E-01
273	SSC4D	9	Weighted median	1.01	0.94	1.09	7.8E-01	1.1E-01	1.01	0.93	1.1	8.5E-01	2.2E-01	0.95	0.8	1.12	5.3E-01	7.0E-02	1.29	1.03	1.62	2.4E-02	4.4E-01	1.02	0.81	1.29	8.5E-01	9.5E-01
273	SSC4D	9	Weighted mode	1.01	0.92	1.1	8.3E-01	1.1E-01	1.01	0.91	1.13	7.9E-01	2.2E-01	0.95	0.77	1.17	5.9E-01	7.0E-02	1.3	1.01	1.69	4.4E-02	4.4E-01	1.02	0.76	1.36	8.9E-01	9.5E-01
273	SSC4D	9	MR Egger	1.05	0.94	1.17	3.7E-01	1.1E-01	1.04	0.91	1.18	5.1E-01	2.2E-01	1.04	0.82	1.32	7.1E-01	7.0E-02	1.35	0.98	1.85	6.4E-02	4.4E-01	1.03	0.74	1.44	8.2E-01	9.5E-01
274	MSR1	12	Inverse variance weighted	1.02	0.89	1.17	7.4E-01	6.2E-01	0.99	0.84	1.17	9.4E-01	3.5E-01	1.12	0.88	1.43	3.6E-01	7.0E-01	0.97	0.8	1.17	7.3E-01	9.1E-01	1.05	0.83	1.34	6.6E-01	8.9E-01
274	MSR1	12	Weighted median	1.01	0.94	1.09	7.6E-01	6.2E-01	0.95	0.87	1.04	2.7E-01	3.5E-01	1.26	1.03	1.54	2.4E-02	7.0E-01	0.96	0.76	1.2	7.0E-01	9.1E-01	1.03	0.82	1.3	7.8E-01	8.9E-01
274	MSR1	12	Weighted mode	1	0.92	1.09	9.8E-01	6.2E-01	0.95	0.87	1.04	2.7E-01	3.5E-01	1.18	0.95	1.47	1.2E-01	7.0E-01	0.97	0.75	1.24	7.6E-01	9.1E-01	1.01	0.79	1.3	9.1E-01	8.9E-01
274	MSR1	12	MR Egger	1	0.82	1.21	9.8E-01	6.2E-01	0.94	0.75	1.18	5.5E-01	3.5E-01	1.16	0.82	1.66	3.7E-01	7.0E-01	0.96	0.74	1.24	7.3E-01	9.1E-01	1.04	0.74	1.47	8.0E-01	8.9E-01
275	NMI	3	Inverse variance weighted	1.06	0.98	1.15	1.2E-01	8.8E-01	1.09	0.95	1.25	2.2E-01	9.6E-01	0.99	0.87	1.12	8.2E-01	6.3E-01	1.13	1	1.28	4.6E-02	4.5E-01	1.12	0.94	1.33	1.9E-01	5.9E-01
275	NMI	3	Weighted median	1.06	1	1.13	4.1E-02	8.8E-01	1.08	1.01	1.16	2.4E-02	9.6E-01	0.99	0.87	1.13	8.7E-01	6.3E-01	1.13	0.94	1.35	1.9E-01	4.5E-01	1.13	0.95	1.34	1.8E-01	5.9E-01
275	NMI	3	Weighted mode	1.07	0.94	1.21	1.7E-01	8.8E-01	1.08	0.93	1.26	1.6E-01	9.6E-01	0.99	0.75	1.32	9.1E-01	6.3E-01	1.14	0.8	1.63	2.5E-01	4.5E-01	1.13	0.78	1.64	3.0E-01	5.9E-01
275	NMI	3	MR Egger	1.08	0.36	3.24	5.5E-01	8.8E-01	1.08	0.15	7.78	7.0E-01	9.6E-01	1.03	0.3	3.61	7.9E-01	6.3E-01	1.27	0.22	7.29	3.3E-01	4.5E-01	1.21	0.21	6.87	3.9E-01	5.9E-01
276	S100P	5	Inverse variance weighted	1	0.85	1.18	9.8E-01	9.8E-01	1.08	0.89	1.31	4.1E-01	6.6E-01	1.05	0.71	1.58	8.0E-01	7.6E-01	0.84	0.51	1.39	4.9E-01	9.3E-01	0.56	0.34	0.93	2.5E-02	8.4E-01
276	S100P	5	Weighted median	1	0.82	1.23	9.8E-01	9.8E-01	1.05	0.84	1.33	6.5E-01	6.6E-01	1.16	0.73	1.85	5.2E-01	7.6E-01	0.86	0.49	1.53	6.0E-01	9.3E-01	0.62	0.34	1.16	1.4E-01	8.4E-01
276	S100P	5	Weighted mode	1.01	0.72	1.42	9.4E-01	9.8E-01	1.05	0.73	1.51	7.2E-01	6.6E-01	0.85	0.36	1.99	6.2E-01	7.6E-01	0.88	0.33	2.36	7.3E-01	9.3E-01	0.64	0.24	1.72	2.8E-01	8.4E-01
276	S100P	5	MR Egger	0.99	0.52	1.89	9.8E-01	9.8E-01	0.98	0.47	2.06	9.3E-01	6.6E-01	0.89	0.15	5.24	8.5E-01	7.6E-01	0.89	0.13	6.24	8.6E-01	9.3E-01	0.63	0.09	4.47	5.1E-01	8.4E-01
277	CSF1R	16	Inverse variance weighted	1.09	0.97	1.22	1.3E-01	7.5E-01	1.14	1	1.3	5.1E-02	9.8E-01	1	0.81	1.23	1.0E+00	8.3E-01	1.1	0.89	1.35	3.8E-01	2.8E-01	1.08	0.84	1.37	5.6E-01	5.1E-01
277	CSF1R	16	Weighted median	1.07	0.98	1.18	1.3E-01	7.5E-01	1.13	1.02	1.25	2.5E-02	9.8E-01	0.92	0.73	1.16	4.7E-01	8.3E-01	1.13	0.85	1.5	4.1E-01	2.8E-01	1.15	0.87	1.51	3.2E-01	5.1E-01
277	CSF1R	16	Weighted mode	1.06	0.97	1.17	1.8E-01	7.5E-01	1.1	0.98	1.24	9.3E-02	9.8E-01	0.94	0.71	1.24	6.5E-01	8.3E-01	1.15	0.83	1.58	3.7E-01	2.8E-01	1.07	0.79	1.45	6.3E-01	5.1E-01
277	CSF1R	16	MR Egger	1.11	0.93	1.32	2.2E-01	7.5E-01	1.14	0.93	1.4	1.9E-01	9.8E-01	1.02	0.74	1.41	8.9E-01	8.3E-01	1.23	0.9	1.67	1.8E-01	2.8E-01	1.16	0.8	1.68	3.9E-01	5.1E-01
278	SERPINA4	4	Inverse variance weighted	1.04	0.99	1.09	1.4E-01	7.9E-01	1.04	0.99	1.1	1.5E-01	5.6E-01	1.13	1.02	1.25	2.5E-02	8.0E-01	0.98	0.83	1.17	8.6E-01	7.7E-01	0.96	0.79	1.18	7.2E-01	5.5E-01
278	SERPINA4	4	Weighted median	1.04	0.99	1.09	1.2E-01	7.9E-01	1.05	0.99	1.11	1.3E-01	5.6E-01	1.13	1.01	1.26	3.2E-02	8.0E-01	0.98	0.84	1.14	8.0E-01	7.7E-01	0.95	0.81	1.11	5.0E-01	5.5E-01
278	SERPINA4	4	Weighted mode	1.04	0.96	1.13	2.4E-01	7.9E-01	1.05	0.96	1.15	1.9E-01	5.6E-01	1.13	0.95	1.34	1.2E-01	8.0E-01	0.98	0.77	1.27	8.6E-01	7.7E-01	0.95	0.75	1.2	5.2E-01	5.5E-01
278	SERPINA4	4	MR Egger	1.04	0.91	1.2	3.1E-01	7.9E-01	1.06	0.9	1.25	2.6E-01	5.6E-01	1.11	0.82	1.51	2.7E-01	8.0E-01	0.96	0.53	1.72	7.8E-01	7.7E-01	0.9	0.47	1.71	5.5E-01	5.5E-01
279	IL10RB	7	Inverse variance weighted	1.02	0.97	1.07	5.2E-01	5.2E-01	1.02	0.97	1.08	4.7E-01	9.2E-01	0.89	0.81	0.99	3.4E-02	3.1E-01	1	0.86	1.15	9.8E-01	4.6E-01	1.08	0.94	1.25	2.9E-01	5.9E-01
279	IL10RB	7	Weighted median	1.01	0.96	1.06	6.2E-01	5.2E-01	1.02	0.96	1.08	4.9E-01	9.2E-01	0.88	0.79	0.98	2.5E-02	3.1E-01	0.99	0.85	1.15	8.8E-01	4.6E-01	1.09	0.94	1.25	2.7E-01	5.9E-01
279	IL10RB	7	Weighted mode	1.01	0.95	1.08	6.5E-01	5.2E-01	1.02	0.95	1.1	5.1E-01	9.2E-01	0.88	0.77	1.02	7.3E-02	3.1E-01	0.98	0.82	1.18	8.4E-01	4.6E-01	1.08	0.9	1.3	3.2E-01	5.9E-01
279	IL10RB	7	MR Egger	1	0.91	1.09	9.6E-01	5.2E-01	1.02	0.92	1.13	6.8E-01	9.2E-01	0.84	0.69	1.02	7.0E-02	3.1E-01	0.94	0.72	1.23	5.8E-01	4.6E-01	1.13	0.86	1.47	3.0E-01	5.9E-01
280	SIGLEC9	9	Inverse variance weighted	1	0.97	1.03	9.1E-01	6.3E-01	0.98	0.95	1.02	4.2E-01	6.9E-01	0.96	0.9	1.03	2.7E-01	6.3E-01	1.02	0.94	1.12	5.9E-01	9.5E-01	1.11	1.01	1.21	2.5E-02	8.9E-01
280	SIGLEC9	9	Weighted median	1.03	0.98	1.07	3.1E-01	6.3E-01	0.99	0.94	1.05	7.7E-01	6.9E-01	0.96	0.89	1.05	3.8E-01	6.3E-01	1.07	0.96	1.19	2.2E-01	9.5E-01	1.1	1	1.22	5.3E-02	8.9E-01
280	SIGLEC9	9	Weighted mode	1.03	0.99	1.08	1.3E-01	6.3E-01	1.01	0.97	1.06	5.2E-01	6.9E-01	0.97	0.89	1.06	5.5E-01	6.3E-01	1.03	0.92	1.14	5.8E-01	9.5E-01	1.11	0.99	1.24	6.4E-02	8.9E-01
280	SIGLEC9	9	MR Egger	1.01	0.95	1.07	7.8E-01	6.3E-01	0.99	0.92	1.07	8.4E-01	6.9E-01	0.95	0.84	1.06	3.0E-01	6.3E-01	1.02	0.87	1.2	7.6E-01	9.5E-01	1.1	0.94	1.29	2.0E-01	8.9E-01
281	FES	1	Wald ratio	1.25	1.03	1.53	2.5E-02	NA	1.17	0.93	1.47	1.8E-01	NA	1.16	0.75	1.77	5.1E-01	NA	1.43	0.78	2.61	2.5E-01	NA	1.21	0.67	2.18	5.3E-01	NA
282	NPR1	1	Wald ratio	0.76	0.47	1.21	2.4E-01	NA	0.54	0.32	0.93	2.5E-02	NA	2.43	0.88	6.7	8.6E-02	NA	1.45	0.35	5.95	6.1E-01	NA	0.27	0.07	1.09	6.5E-02	NA
283	FGF19	5	Inverse variance weighted	0.95	0.86	1.05	3.5E-01	3.8E-01	1.04	0.94	1.16	4.3E-01	8.2E-01	0.77	0.59	0.99	4.5E-02	8.2E-01	0.84	0.57	1.24	3.7E-01	1.1E-01	0.86	0.56	1.32	4.9E-01	1.8E-01
283	FGF19	5	Weighted median	0.91	0.82	1.01	8.9E-02	3.8E-01	1.03	0.92	1.16	5.8E-01	8.2E-01	0.77	0.61	0.97	2.5E-02	8.2E-01	0.79	0.57	1.1	1.7E-01	1.1E-01	0.83	0.6	1.13	2.4E-01	1.8E-01
283	FGF19	5	Weighted mode	0.91	0.77	1.07	1.7E-01	3.8E-01	1.05	0.87	1.26	5.4E-01	8.2E-01	0.77	0.55	1.08	9.9E-02	8.2E-01	0.65	0.41	1.04	6.3E-02	1.1E-01	0.81	0.52	1.27	2.7E-01	1.8E-01
283	FGF19	5	MR Egger	0.85	0.57	1.26	2.8E-01	3.8E-01	1.08	0.7	1.65	6.2E-01	8.2E-01	0.7	0.21	2.36	4.2E-01	8.2E-01	0.41	0.13	1.26	8.5E-02	1.1E-01	0.43	0.1	1.73	1.5E-01	1.8E-01
284	EGF	8	Inverse variance weighted	1.13	0.99	1.28	6.0E-02	8.9E-01	1.15	1.01	1.31	3.6E-02	6.3E-01	1.18	0.86	1.61	3.1E-01	6.9E-01	1.5	1.05	2.14	2.6E-02	3.1E-01	1.11	0.69	1.8	6.6E-01	6.6E-01
284	EGF	8	Weighted median	1.13	0.99	1.29	7.1E-02	8.9E-01	1.15	0.99	1.33	6.3E-02	6.3E-01	1.21	0.88	1.67	2.3E-01	6.9E-01	1.46	0.97	2.2	7.2E-02	3.1E-01	0.91	0.58	1.42	6.7E-01	6.6E-01
284	EGF	8	Weighted mode	1.15	0.95	1.4	1.2E-01	8.9E-01	1.15	0.9	1.47	2.1E-01	6.3E-01	1.26	0.75	2.1	3.2E-01	6.9E-01	1.44	0.77	2.67	2.1E-01	3.1E-01	0.8	0.4	1.59	4.7E-01	6.6E-01
284	EGF	8	MR Egger	1.09	0.62	1.93	7.7E-01	8.9E-01	1.03	0.58	1.82	9.2E-01	6.3E-01	1.47	0.36	6	5.2E-01	6.9E-01	0.8	0.19	3.45	7.8E-01	3.1E-01	0.76	0.09	6.38	7.6E-01	6.6E-01
285	BAIAP2	4	Inverse variance weighted	1.26	0.98	1.63	7.5E-02	4.7E-01	1.43	1.04	1.96	2.6E-02	5.0E-01	1.67	1.03	2.69	3.7E-02	5.2E-01	1.07	0.52	2.17	8.6E-01	5.1E-01	1.04	0.53	2.02	9.2E-01	2.8E-01
285	BAIAP2	4	Weighted median	1.19	0.93	1.51	1.6E-01	4.7E-01	1.24	0.92	1.67	1.5E-01	5.0E-01	1.53	0.94	2.48	8.9E-02	5.2E-01	1.05	0.51	2.18	8.9E-01	5.1E-01	0.95	0.46	1.96	8.8E-01	2.8E-01
285	BAIAP2	4	Weighted mode	1.08	0.65	1.8	7.7E-01	4.7E-01	1.17	0.66	2.09	4.4E-01	5.0E-01	1.5	0.66	3.45	2.2E-01	5.2E-01	1.78	0.3	10.67	3.8E-01	5.1E-01	0.78	0.14	4.49	6.9E-01	2.8E-01
285	BAIAP2	4	MR Egger	1.04	0.33	3.22	9.1E-01	4.7E-01	1.14	0.27	4.73	7.4E-01	5.0E-01	1.2	0.14	10.29	7.5E-01	5.2E-01	1.73	0.08	37.66	5.2E-01	5.1E-01	0.47	0.03	6.53	3.4E-01	2.8E-01
286	COL6A3	3	Inverse variance weighted	1.26	1.01	1.59	4.1E-02	6.2E-01	1.29	0.99	1.67	5.8E-02	5.3E-01	1.14	0.7	1.85	6.0E-01	4.8E-01	1.06	0.54	2.1	8.6E-01	7.3E-01	1.13	0.58	2.19	7.2E-01	8.2E-01
286	COL6A3	3	Weighted median	1.3	1.03	1.64	2.6E-02	6.2E-01	1.34	1.02	1.77	3.7E-02	5.3E-01	1.15	0.67	1.98	6.0E-01	4.8E-01	1.09	0.54	2.21	8.0E-01	7.3E-01	1.19	0.57	2.48	6.4E-01	8.2E-01
286	COL6A3	3	Weighted mode	1.31	0.72	2.38	1.9E-01	6.2E-01	1.35	0.65	2.81	2.2E-01	5.3E-01	1.31	0.35	4.93	4.7E-01	4.8E-01	0.86	0.13	5.82	7.6E-01	7.3E-01	1.38	0.19	9.93	5.5E-01	8.2E-01
286	COL6A3	3	MR Egger	0.98	0.01	135.08	9.7E-01	6.2E-01	0.87	0	254.59	8.1E-01	5.3E-01	0.47	0	23809.96	5.4E-01	4.8E-01	1.7	0	2131257.18	7.1E-01	7.3E-01	1.64	0	49810921.65	7.8E-01	8.2E-01
287	SEMA3F	4	Inverse variance weighted	0.87	0.69	1.1	2.4E-01	2.1E-01	0.76	0.57	1	4.9E-02	1.9E-01	1.02	0.66	1.59	9.3E-01	6.6E-01	1	0.41	2.4	1.0E+00	1.7E-01	1.2	0.65	2.2	5.7E-01	8.0E-01
287	SEMA3F	4	Weighted median	0.87	0.68	1.13	3.0E-01	2.1E-01	0.72	0.54	0.96	2.6E-02	1.9E-01	1	0.58	1.71	9.9E-01	6.6E-01	0.9	0.44	2.1	9.1E-01	1.7E-01	1.38	0.67	2.85	3.8E-01	8.0E-01
287	SEMA3F	4	Weighted mode	0.9	0.48	1.71	6.5E-01	2.1E-01	0.69	0.31	1.55	2.4E-01	1.9E-01	0.96	0.27	3.45	9.3E-01	6.6E-01	0.85	0.08	8.79	8.4E-01	1.7E-01	1.48	0.32	6.9	4.8E-01	8.0E-01
287	SEMA3F	4	MR Egger	17.54	0.01	22324.58	2.3E-01	2.1E-01	31.89	0.01	125309.28	2.1E-01	1.9E-01	8.49	0	486166587	6.6E-01	6.6E-01	33583.93	0	7.48317E+13	1.7E-01	1.7E-01	0	0	8758501.37	3.9E-01	8.0E-01
288	CD109	5	Inverse variance weighted	1.02	0.91	1.14	7.4E-01	9.3E-01	1.05	0.92	1.19	5.1E-01	9.1E-01	0.99	0.74	1.31	9.3E-01	2.0E-01	0.94	0.66	1.33	7.2E-01	3.7E-01	0.76	0.53	1.1	1.4E-01	5.6E-01
288	CD109	5	Weighted median	1.02	0.91	1.15	7.3E-01	9.3E-01	1.05	0.91	1.21	4.8E-01	9.1E-01	0.97	0.72	1.23	6.6E-01	2.0E-01	0.98	0.67	1.45	9.4E-01	3.7E-01	0.64	0.44	0.95	2.6E-02	5.6E-01
288	CD109	5	Weighted mode	1.02	0.85	1.22	7.6E-01	9.3E-01	1.05	0.87	1.27	5.2E-01	9.1E-01	0.94	0.64	1.38	6.8E-01	2.0E-01	0.99	0.57	1.73	9.8E-01	3.7E-01	0.65	0.37	1.13	9.5E-02	5.6E-01
288	CD109	5	MR Egger	1.02	0.8	1.32	7.8E-01	9.3E-01	1.05	0.76	1.47	6.4E-01	9.1E-01	0.82	0.47	1.42	3.8E-01	2.0E-01	1.11	0.52	2.37	7.0E-01	3.7E-01	0.63	0.29	1.4	1.7E-01	5.6E-01
289	KIT	20	Inverse variance weighted	0.86	0.72	1.01	7.0E-02	5.9E-01	0.8	0.65	0.98	2.9E-02	6.6E-01	0.94	0.79	1.12	4.6E-01	5.3E-01	0.95	0.77	1.19	6.6E-01	7.1E-01	1.23	0.99	1.53	5.6E-02	2.1E-01
289	KIT	20	Weighted median	1.02	0.91	1.14	7.8E-01	5.9E-01	0.93	0.82	1.06	2.7E-01	6.6E-01	1.1	0.87	1.4	4.2E-01	5.3E-01	1.03	0.75	1.42	8.6E-01	7.1E-01	1.23	0.88	1.71	2.2E-01	2.1E-01
289	KIT	20	Weighted mode	1.06	0.94	1.2	3.3E-01	5.9E-01	1	0.86	1.17	9.6E-01	6.6E-01	1.16	0.82	1.65	3.8E-01	5.3E-01	1.09	0.69	1.68	7.4E-01	7.1E-01	1.55	0.99	2.44	5.5E-02	2.1E-01
289	KIT	20	MR Egger	0.93	0.64	1.35	6.9E-01	5.9E-01	0.87	0.56	1.35	5.1E-01	6.6E-01	1.04	0.7	1.53	8.5E-01	5.3E-01	0.88	0.55	1.42	5.9E-01	7.1E-01	1.7	1.07	2.69	2.6E-02	2.1E-01
290	KLK15	4	Inverse variance weighted	1.03	1	1.07	7.7E-02	9.5E-01	1.05	1	1.09	2.9E-02	8.5E-01	1.03	0.93	1.13	6.1E-01	1.6E-01	1	0.9	1.12	9.3E-01	9.4E-01	1	0.88	1.14	1.0E+00	4.0E-01
290	KLK15	4	Weighted median	1.03	1	1.07	7.3E-02	9.5E-01	1.04	1.01	1.09	2.6E-02	8.5E-01	1.02	0.95	1.1	5.4E-01	1.6E-01	1	0.9	1.11	9.4E-01	9.4E-01	1.01	0.91	1.12	8.8E-01	4.0E-01
290	KLK15	4	Weighted mode	1.03	0.97	1.09	2.1E-01	9.5E-01	1.04	0.98	1.12	1.3E-01	8.5E-01	1.02	0.9	1.15	7.8E-01	1.6E-01	1.01	0.84	1.2	9.3E-01	9.4E-01	1.01	0.86	1.19	8.6E-01	4.0E-01
290	KLK15	4	MR Egger	1.03	0.95	1.12	2.6E-01	9.5E-01	1.05	0.95	1.15	1.8E-01	8.5E-01	0.99	0.82	1.18	8.9E-01	1.6E-01	1	0.78	1.29	9.7E-01	9.4E-01	1.03	0.76	1.41	6.9E-01	4.0E-01
291	VAMP5	1	Wald ratio	0.68	0.49	0.96	2.6E-02	NA	0.77	0.52	1.13	1.8E-01	NA	0.45	0.22	0.94	3.4E-02	NA	0.64	0.23	1.8	4.0E-01	NA	0.51	0.19	1.41	1.9E-01	NA
292	CLEC1A	5	Inverse variance weighted	1.01	0.95	1.07	8.4E-01	9.7E-01	1.02	0.95	1.09	5.3E-01	8.4E-01	0.97	0.85	1.1	6.4E-01	7.9E-01	0.98	0.82	1.17	8.2E-01	4.4E-01	0.83	0.68	1.01	6.6E-02	3.8E-01
292	CLEC1A	5	Weighted median	1.01	0.95	1.07	8.5E-01	9.7E-01	1.02	0.96	1.09	4.9E-01	8.4E-01	0.97	0.85	1.11	6.6E-01	7.9E-01	0.99	0.83	1.19	9.2E-01	4.4E-01	0.81	0.67	0.97	2.6E-02	3.8E-01
292	CLEC1A	5	Weighted mode	1.01	0.92	1.1	8.4E-01	9.7E-01	1.02	0.94	1.12	6.2E-01	8.4E-01	0.97	0.8	1.17	7.4E-01	7.9E-01	1	0.75	1.32	9.9E-01	4.4E-01	0.8	0.61	1.06	9.6E-02	3.8E-01
292	CLEC1A	5	MR Egger	1.01	0.89	1.13	9.0E-01	9.7E-01	1.03	0.89	1.18	5.7E-01	8.4E-01	0.98	0.76	1.28	8.6E-01	7.9E-01	1.04	0.72	1.5	7.5E-01	4.4E-01	0.77	0.51	1.14	1.3E-01	3.8E-01
293	EPHB6	3	Inverse variance weighted	0.94	0.86	1.02	1.6E-01	6.6E-01	0.95	0.86	1.05	3.5E-01	8.5E-01	0.81	0.67	0.98	2.9E-02	7.1E-01	0.85	0.65	1.12	2.5E-01	8.0E-01	0.92	0.7	1.19	5.2E-01	7.1E-01
293	EPHB6	3	Weighted median	0.94	0.86	1.02	1.4E-01	6.6E-01	0.95	0.86	1.06	3.6E-01	8.5E-01	0.8	0.66	0.97	2.6E-02	7.1E-01	0.85	0.65	1.13	2.9E-01	8.0E-01	0.92	0.71	1.2	5.4E-01	7.1E-01
293	EPHB6	3	Weighted mode	0.93	0.75	1.16	5.8E-01	6.6E-01	0.95	0.75	1.19	4.1E-01	8.5E-01	0.8	0.51	1.25	1.7E-01	7.1E-01	0.86	0.44	1.68	4.4E-01	8.0E-01	0.92	0.5	1.72	6.3E-01	7.1E-01
293	EPHB6	3	MR Egger	0.89	0.26	3.08	4.5E-01	6.6E-01	0.93	0.22	3.91	6.3E-01	8.5E-01	0.74	0.05	10.89	3.9E-01	7.1E-01	0.93	0.02	39.88	8.5E-01	8.0E-01	1.04	0.03	42.24	9.2E-01	7.1E-01
294	CALCA	5	Inverse variance weighted	1.16	0.89	1.51	2.6E-01	6.5E-01	1.29	0.96	1.73	9.1E-02	3.3E-01	1.49	0.58	3.78	4.1E-01	7.1E-01	0.48	0.23	0.98	4.4E-02	2.7E-01	0.69	0.12	4.07	6.8E-01	8.8E-01
294	CALCA	5	Weighted median	1.16	0.86	1.56	3.4E-01	6.5E-01	1.33	0.94	1.88	1.1E-01	3.3E-01	2.07	1.09	3.93	2.7E-02	7.1E-01	0.46	0.19	1.11	8.5E-02	2.7E-01	1.03	0.34	3.09	9.6E-01	8.8E-01
294	CALCA	5	Weighted mode	1.17	0.69	1.99	4.6E-01	6.5E-01	1.6	0.8	3.19	1.3E-01	3.3E-01	2.08	0.77	5.61	1.7E-01	7.1E-01	0.46	0.09	2.42	2.6E-01	2.7E-01	1.54	0.09	25.45	6.9E-01	8.8E-01
294	CALCA	5	MR Egger	0.89	0.15	5.3	8.4E-01	6.5E-01	0.7	0.12	3.94	5.6E-01	3.3E-01	3.41	0	2408.25	5.9E-01	7.1E-01	2.68	0.04	190.61	5.1E-01	2.7E-01	0.37	0	111268.52	8.2E-01	8.8E-01
295	BLMH	3	Inverse variance weighted	1.02	0.95	1.09	6.4E-01	3.0E-01	1.01	0.93	1.1	7.8E-01	2.7E-01	1.01	0.91	1.11	9.1E-01	9.6E-01	1.16	0.89	1.51	2.7E-01	2.3E-01	0.99	0.86	1.14	8.7E-01	4.3E-01
295	BLMH	3	Weighted median	1.02	0.97	1.07	4.8E-01	3.0E-01	1.01	0.96	1.07	6.5E-01	2.7E-01	1.01	0.91	1.11	9.1E-01	9.6E-01	1.17	1.02	1.34	2.7E-02	2.3E-01	0.99	0.86	1.14	8.8E-01	4.3E-01
295	BLMH	3	Weighted mode	1.02	0.93	1.13	4.4E-01	3.0E-01	1.02	0.91	1.15	5.5E-01	2.7E-01	1.01	0.8	1.26	9.3E-01	9.6E-01	1.19	0.88	1.6	1.3E-01	2.3E-01	0.98	0.72	1.34	7.9E-01	4.3E-01
295	BLMH	3	MR Egger	1.07	0.67	1.71	3.0E-01	3.0E-01	1.08	0.63	1.86	3.1E-01	2.7E-01	1.01	0.37	2.76	9.2E-01	9.6E-01	1.44	0.36	5.75	1.9E-01	2.3E-01	0.89	0.22	3.6	4.9E-01	4.3E-01
296	TNR	13	Inverse variance weighted	0.98	0.93	1.04	4.8E-01	7.9E-01	0.99	0.93	1.05	7.1E-01	9.5E-01	0.9	0.78	1.03	1.3E-01	1.1E-01	1.09	0.88	1.35	4.2E-01	2.0E-01	0.93	0.77	1.11	4.1E-01	4.3E-01
296	TNR	13	Weighted median	0.97	0.91	1.04	4.2E-01	7.9E-01	0.99	0.92	1.07	8.5E-01	9.5E-01	0.85	0.74	0.98	2.7E-02	1.1E-01	1.19	0.97	1.45	9.3E-02	2.0E-01	0.9	0.74	1.11	3.2E-01	4.3E-01
296	TNR	13	Weighted mode	0.97	0.89	1.05	4.3E-01	7.9E-01	0.99	0.9	1.09	8.8E-01	9.5E-01	0.84	0.71	0.98	3.4E-02	1.1E-01	1.16	0.93	1.46	1.7E-01	2.0E-01	0.83	0.65	1.05	1.1E-01	4.3E-01
296	TNR	13	MR Egger	0.97	0.88	1.07	5.2E-01	7.9E-01	0.99	0.88	1.11	7.8E-01	9.5E-01	0.78	0.63	0.98	3.8E-02	1.1E-01	1.3	0.9	1.88	1.4E-01	2.0E-01	0.84	0.6	1.17	2.8E-01	4.3E-01
297	FOLH1	3	Inverse variance weighted	0.89	0.76	1.04	1.5E-01	5.9E-01	0.81	0.67	0.98	3.3E-02	9.5E-01	0.72	0.4	1.3	2.8E-01	2.6E-01	1.03	0.63	1.68	9.1E-01	5.0E-01	1.24	0.76	2	3.9E-01	8.6E-01
297	FOLH1	3	Weighted median	0.88	0.73	1.05	1.5E-01	5.9E-01	0.83	0.67	1.02	7.8E-02	9.5E-01	0.63	0.42	0.95	2.7E-02	2.6E-01	1.1	0.63	1.92	7.4E-01	5.0E-01	1.21	0.72	2.07	5.6E-01	8.6E-01
297	FOLH1	3	Weighted mode	0.95	0.59	1.52	6.9E-01	5.9E-01	0.83	0.47	1.45	2.9E-01	9.5E-01	1.11	0.37	3.36	7.2E-01	2.6E-01	1.06	0.33	3.42	9.2E-01	5.0E-01	1.18	0.27	5.07	6.7E-01	8.6E-01
297	FOLH1	3	MR Egger	0.68	0.01	73.14	4.8E-01	5.9E-01	0.79	0	178.28	6.7E-01	9.5E-01	0.11	0	3732.73	2.3E-01	2.6E-01	3.03	0	3714232.32	5.0E-01	5.0E-01	1.56	0	1636984.64	7.5E-01	8.6E-01
298	CTSV	13	Inverse variance weighted	0.95	0.79	1.14	5.8E-01	7.1E-01	0.93	0.76	1.14	4.9E-01	8.0E-01	1.08	0.82	1.41	5.8E-01	2.4E-01	0.8	0.62	1.04	9.1E-02	9.9E-01	0.9	0.55	1.46	6.7E-01	5.7E-01
298	CTSV	13	Weighted median	1.04	0.92	1.16	5.3E-01	7.1E-01	0.97	0.85	1.11	6.8E-01	8.0E-01	1.34	1.03	1.74	2.8E-02	2.4E-01	0.78	0.56	1.09	1.5E-01	9.9E-01	0.81	0.58	1.14	2.2E-01	5.7E-01
298	CTSV	13	Weighted mode	1.07	0.94	1.22	3.2E-01	7.1E-01	1	0.86	1.16	9.9E-01	8.0E-01	1.32	1.01	1.73	5.9E-02	2.4E-01	0.78	0.53	1.15	2.3E-01	9.9E-01	0.85	0.54	1.32	4.4E-01	5.7E-01
298	CTSV	13	MR Egger	1.01	0.66	1.56	9.5E-01	7.1E-01	0.88	0.54	1.44	5.9E-01	8.0E-01	1.45	0.8	2.65	2.0E-01	2.4E-01	0.8	0.44	1.45	4.6E-01	9.9E-01	0.69	0.22	2.19	4.9E-01	5.7E-01
299	GSTA1	8	Inverse variance weighted	0.92	0.86	0.99	2.8E-02	9.5E-01	0.93	0.85	1.01	8.6E-02	9.6E-01	0.95	0.81	1.11	5.1E-01	6.7E-01	1.02	0.82	1.28	8.4E-01	4.4E-01	0.88	0.69	1.11	2.8E-01	3.4E-01
299	GSTA1	8	Weighted median	0.92	0.85	0.99	3.4E-02	9.5E-01	0.93	0.84	1.01	9.6E-02	9.6E-01	0.93	0.78	1.11	4.3E-01	6.7E-01	1.06	0.84	1.34	6.3E-01	4.4E-01	0.84	0.66	1.07	1.6E-01	3.4E-01
299	GSTA1	8	Weighted mode	0.92	0.83	1.01	7.7E-02	9.5E-01	0.93	0.82	1.04	1.7E-01	9.6E-01	0.93	0.75	1.16	4.7E-01	6.7E-01	1.06	0.78	1.44	6.9E-01	4.4E-01	0.84	0.64	1.11	1.8E-01	3.4E-01
299	GSTA1	8	MR Egger	0.92	0.8	1.05	1.8E-01	9.5E-01	0.93	0.79	1.09	3.2E-01	9.6E-01	0.91	0.67	1.23	5.2E-01	6.7E-01	1.14	0.75	1.74	4.8E-01	4.4E-01	0.76	0.49	1.19	2.0E-01	3.4E-01
300	MEP1A	13	Inverse variance weighted	0.96	0.9	1.01	1.4E-01	5.9E-01	1	0.93	1.07	9.4E-01	4.4E-01	0.87	0.77	0.98	2.8E-02	8.3E-01	0.92	0.73	1.16	4.7E-01	7.2E-01	0.89	0.73	1.07	2.1E-01	5.5E-01
300	MEP1A	13	Weighted median	0.98	0.91	1.05	5.6E-01	5.9E-01	1.05	0.96	1.14	2.9E-01	4.4E-01	0.88	0.75	1.04	1.3E-01	8.3E-01	0.98	0.76	1.26	8.5E-01	7.2E-01	0.85	0.66	1.08	1.8E-01	5.5E-01
300	MEP1A	13	Weighted mode	0.97	0.89	1.07	5.5E-01	5.9E-01	1.02	0.92	1.14	6.3E-01	4.4E-01	0.89	0.71	1.1	2.4E-01	8.3E-01	0.93	0.69	1.25	6.1E-01	7.2E-01	0.79	0.56	1.1	1.5E-01	5.5E-01
300	MEP1A	13	MR Egger	0.99	0.87	1.12	8.6E-01	5.9E-01	1.05	0.89	1.24	5.2E-01	4.4E-01	0.89	0.67	1.18	3.9E-01	8.3E-01	0.85	0.49	1.47	5.3E-01	7.2E-01	0.99	0.63	1.56	9.6E-01	5.5E-01
301	GSTA3	4	Inverse variance weighted	0.91	0.84	0.99	2.8E-02	8.3E-01	0.91	0.83	1	5.0E-02	8.2E-01	0.94	0.79	1.11	4.5E-01	9.1E-01	1	0.79	1.27	1.0E+00	3.3E-01	0.89	0.7	1.13	3.3E-01	6.0E-01

301	GSTA3	4	Weighted median	0.91	0.84	0.99	3.4E-02	8.3E-01	0.91	0.82	1	5.0E-02	8.2E-01	0.93	0.78	1.11	4.3E-01	9.1E-01	1.02	0.79	1.3	9.0E-01	3.3E-01	0.87	0.69	1.11	2.8E-01	6.0E-01
301	GSTA3	4	Weighted mode	0.91	0.79	1.05	1.3E-01	8.3E-01	0.91	0.77	1.07	1.6E-01	8.2E-01	0.93	0.7	1.23	4.7E-01	9.1E-01	1.03	0.68	1.57	8.1E-01	3.3E-01	0.87	0.59	1.29	3.5E-01	6.0E-01
301	GSTA3	4	MR Egger	0.9	0.68	1.21	2.7E-01	8.3E-01	0.9	0.64	1.25	3.0E-01	8.2E-01	0.92	0.49	1.73	6.3E-01	9.1E-01	1.23	0.51	2.96	4.2E-01	3.3E-01	0.8	0.34	1.91	3.9E-01	6.0E-01
302	BECN1	3	Inverse variance weighted	0.98	0.78	1.22	8.3E-01	6.8E-01	1.11	0.83	1.49	4.9E-01	6.6E-01	0.94	0.5	1.75	8.4E-01	3.4E-01	0.76	0.39	1.5	4.3E-01	7.2E-01	0.47	0.24	0.92	2.8E-02	4.8E-01
302	BECN1	3	Weighted median	1.05	0.82	1.35	6.8E-01	6.8E-01	1.19	0.88	1.61	2.6E-01	6.6E-01	1.03	0.59	1.79	9.2E-01	3.4E-01	0.66	0.31	1.4	2.8E-01	7.2E-01	0.52	0.23	1.17	1.1E-01	4.8E-01
302	BECN1	3	Weighted mode	1.06	0.52	2.15	7.7E-01	6.8E-01	1.29	0.49	3.37	3.8E-01	6.6E-01	1.14	0.26	4.95	7.5E-01	3.4E-01	0.65	0.09	4.55	4.4E-01	7.2E-01	0.63	0.07	5.98	4.7E-01	4.8E-01
302	BECN1	3	MR Egger	1.1	0.05	25.85	7.6E-01	6.8E-01	1.37	0.01	228.32	5.8E-01	6.6E-01	2.05	0	1681.18	4.0E-01	3.4E-01	0.55	0	10118.5	5.8E-01	7.2E-01	0.22	0	5654.96	3.1E-01	4.8E-01
303	BCHE	13	Inverse variance weighted	1.04	0.98	1.1	2.2E-01	6.9E-01	1.02	0.96	1.09	5.7E-01	9.3E-01	1.12	0.99	1.27	8.4E-02	2.5E-01	1.1	0.95	1.28	2.0E-01	1.6E-01	1.1	0.95	1.28	2.1E-01	8.9E-01
303	BCHE	13	Weighted median	1.02	0.97	1.08	3.9E-01	6.9E-01	1	0.94	1.07	9.1E-01	9.3E-01	1.14	1.01	1.29	2.8E-02	2.5E-01	1.14	0.97	1.35	1.1E-01	1.6E-01	1.09	0.92	1.28	3.2E-01	8.9E-01
303	BCHE	13	Weighted mode	1.02	0.96	1.09	5.0E-01	6.9E-01	1	0.93	1.07	9.3E-01	9.3E-01	1.14	1	1.31	5.0E-02	2.5E-01	1.15	0.95	1.39	1.3E-01	1.6E-01	1.09	0.91	1.31	3.2E-01	8.9E-01
303	BCHE	13	MR Egger	1.05	0.96	1.14	2.6E-01	6.9E-01	1.02	0.92	1.12	7.2E-01	9.3E-01	1.19	1	1.42	5.5E-02	2.5E-01	1.21	0.98	1.5	7.8E-02	1.6E-01	1.11	0.9	1.37	3.1E-01	8.9E-01
304	TXNDC15	10	Inverse variance weighted	1.01	0.98	1.04	5.1E-01	8.8E-01	1	0.96	1.05	8.6E-01	8.4E-01	1.08	1.01	1.15	2.8E-02	5.4E-01	0.98	0.87	1.1	7.2E-01	7.8E-01	1.03	0.95	1.13	4.6E-01	7.2E-01
304	TXNDC15	10	Weighted median	1.01	0.98	1.05	4.5E-01	8.8E-01	1.01	0.98	1.05	4.6E-01	8.4E-01	1.07	1	1.15	5.6E-02	5.4E-01	0.98	0.89	1.08	6.9E-01	7.8E-01	1.05	0.95	1.15	3.5E-01	7.2E-01
304	TXNDC15	10	Weighted mode	1.01	0.98	1.05	4.4E-01	8.8E-01	1.01	0.96	1.06	6.6E-01	8.4E-01	1.07	0.99	1.15	8.7E-02	5.4E-01	0.98	0.87	1.09	6.5E-01	7.8E-01	1.05	0.94	1.16	3.6E-01	7.2E-01
304	TXNDC15	10	MR Egger	1.01	0.96	1.05	6.8E-01	8.8E-01	1.01	0.95	1.07	8.1E-01	8.4E-01	1.06	0.96	1.17	2.2E-01	5.4E-01	0.99	0.82	1.2	9.2E-01	7.8E-01	1.05	0.92	1.2	4.4E-01	7.2E-01
305	CD274	6	Inverse variance weighted	1.07	0.99	1.16	1.1E-01	5.6E-01	1.11	1.01	1.22	2.8E-02	7.9E-01	1.05	0.88	1.25	6.1E-01	2.8E-01	1.02	0.77	1.35	9.0E-01	7.8E-01	1.04	0.82	1.33	7.3E-01	1.7E-01
305	CD274	6	Weighted median	1.07	0.99	1.17	9.0E-02	5.6E-01	1.11	1	1.22	3.9E-02	7.9E-01	1.05	0.88	1.26	5.7E-01	2.8E-01	1.04	0.81	1.34	7.5E-01	7.8E-01	1.07	0.83	1.37	5.9E-01	1.7E-01
305	CD274	6	Weighted mode	1.07	0.96	1.2	1.7E-01	5.6E-01	1.11	0.98	1.26	9.2E-02	7.9E-01	1.06	0.82	1.37	5.9E-01	2.8E-01	1.04	0.74	1.47	7.7E-01	7.8E-01	1.08	0.77	1.5	5.9E-01	1.7E-01
305	CD274	6	MR Egger	1.1	0.92	1.32	2.0E-01	5.6E-01	1.09	0.89	1.34	3.0E-01	7.9E-01	1.2	0.81	1.76	2.7E-01	2.8E-01	1.08	0.54	2.16	7.8E-01	7.8E-01	1.34	0.78	2.29	2.0E-01	1.7E-01
306	CA14	2	Inverse variance weighted	0.83	0.62	1.11	2.1E-01	NA	0.86	0.62	1.2	3.7E-01	NA	0.87	0.35	2.15	7.7E-01	NA	0.38	0.16	0.9	2.8E-02	NA	1.2	0.43	3.34	7.3E-01	NA
307	CDH17	10	Inverse variance weighted	0.95	0.89	1	5.4E-02	3.8E-01	0.98	0.92	1.05	6.2E-01	5.1E-02	0.9	0.8	1.03	1.2E-01	3.5E-01	0.84	0.7	1.01	5.7E-02	7.0E-01	0.98	0.82	1.16	7.7E-01	2.7E-01
307	CDH17	10	Weighted median	0.94	0.89	1.01	8.1E-02	3.8E-01	1.02	0.95	1.09	6.1E-01	5.1E-02	0.86	0.75	0.98	2.9E-02	3.5E-01	0.82	0.68	1	4.5E-02	7.0E-01	0.91	0.75	1.11	3.5E-01	2.7E-01
307	CDH17	10	Weighted mode	0.95	0.87	1.03	1.6E-01	3.8E-01	1.01	0.93	1.1	8.0E-01	5.1E-02	0.85	0.72	1	5.5E-02	3.5E-01	0.78	0.62	0.99	4.3E-02	7.0E-01	0.89	0.69	1.13	2.9E-01	2.7E-01
307	CDH17	10	MR Egger	0.98	0.88	1.09	6.6E-01	3.8E-01	1.09	0.96	1.23	1.7E-01	5.1E-02	0.83	0.65	1.06	1.2E-01	3.5E-01	0.8	0.56	1.15	1.9E-01	7.0E-01	0.85	0.62	1.18	3.0E-01	2.7E-01
308	CLNS1A	1	Wald ratio	1.21	0.78	1.89	3.9E-01	NA	1.19	0.71	1.99	5.0E-01	NA	1.03	0.39	2.7	9.5E-01	NA	4.49	1.17	17.2	2.9E-02	NA	0.75	0.2	2.86	6.8E-01	NA
309	COL3A1	1	Wald ratio	1.19	1.02	1.4	2.9E-02	NA	1.16	0.97	1.4	1.0E-01	NA	1.11	0.79	1.56	5.6E-01	NA	1.36	0.85	2.18	2.0E-01	NA	1.36	0.85	2.18	2.0E-01	NA
310	SCARB1	1	Wald ratio	0.98	0.47	2.04	9.7E-01	NA	1.59	0.68	3.67	2.8E-01	NA	0.87	0.18	4.23	8.6E-01	NA	0.08	0.01	0.77	2.9E-02	NA	0.13	0.01	1.18	7.0E-02	NA
311	CSF3R	8	Inverse variance weighted	0.95	0.88	1.03	2.1E-01	6.5E-01	0.98	0.9	1.07	6.7E-01	5.9E-01	0.83	0.7	0.98	2.9E-02	9.7E-01	0.93	0.7	1.23	6.0E-01	7.0E-01	0.93	0.71	1.23	6.1E-01	3.4E-01
311	CSF3R	8	Weighted median	0.93	0.85	1.02	1.3E-01	6.5E-01	0.95	0.86	1.06	3.7E-01	5.9E-01	0.83	0.68	1.01	5.9E-02	9.7E-01	0.9	0.67	1.22	5.1E-01	7.0E-01	0.83	0.61	1.13	2.4E-01	3.4E-01
311	CSF3R	8	Weighted mode	0.91	0.8	1.03	1.0E-01	6.5E-01	0.94	0.82	1.08	3.1E-01	5.9E-01	0.83	0.64	1.07	1.2E-01	9.7E-01	0.93	0.66	1.33	6.6E-01	7.0E-01	0.81	0.57	1.16	2.1E-01	3.4E-01
311	CSF3R	8	MR Egger	0.93	0.82	1.07	2.6E-01	6.5E-01	0.96	0.82	1.11	5.1E-01	5.9E-01	0.83	0.62	1.11	1.6E-01	9.7E-01	0.98	0.59	1.64	9.4E-01	7.0E-01	0.81	0.51	1.3	3.2E-01	3.4E-01
312	CA3	6	Inverse variance weighted	1	0.9	1.12	9.5E-01	6.7E-01	1.04	0.92	1.18	5.1E-01	9.3E-01	0.76	0.6	0.97	2.9E-02	2.6E-01	1.17	0.84	1.63	3.6E-01	9.6E-01	0.79	0.57	1.11	1.8E-01	1.4E-01
312	CA3	6	Weighted median	1.01	0.89	1.15	8.3E-01	6.7E-01	1.04	0.89	1.21	6.2E-01	9.3E-01	0.87	0.65	1.17	3.5E-01	2.6E-01	1.23	0.81	1.88	3.3E-01	9.6E-01	0.72	0.46	1.13	1.5E-01	1.4E-01
312	CA3	6	Weighted mode	1.01	0.83	1.24	8.8E-01	6.7E-01	1.04	0.8	1.35	7.3E-01	9.3E-01	0.87	0.55	1.37	4.7E-01	2.6E-01	1.3	0.67	2.52	3.5E-01	9.6E-01	0.63	0.26	1.55	2.5E-01	1.4E-01
312	CA3	6	MR Egger	1.09	0.65	1.81	6.7E-01	6.7E-01	1.02	0.57	1.85	9.1E-01	9.3E-01	1.26	0.42	3.78	5.9E-01	2.6E-01	1.2	0.26	5.62	7.6E-01	9.6E-01	2.12	0.46	9.78	2.5E-01	1.4E-01
313	HAO1	8	Inverse variance weighted	0.86	0.75	1	5.4E-02	3.0E-01	0.89	0.75	1.06	1.8E-01	9.2E-01	0.84	0.59	1.2	3.4E-01	6.2E-02	0.98	0.63	1.55	9.5E-01	3.6E-01	0.88	0.56	1.37	5.7E-01	3.9E-01
313	HAO1	8	Weighted median	0.81	0.67	0.98	3.0E-02	3.0E-01	0.91	0.75	1.12	3.9E-01	9.2E-01	0.8	0.5	1.26	3.3E-01	6.2E-02	0.84	0.48	1.48	5.4E-01	3.6E-01	0.73	0.41	1.31	2.9E-01	3.9E-01
313	HAO1	8	Weighted mode	0.79	0.57	1.07	1.1E-01	3.0E-01	0.91	0.65	1.27	5.3E-01	9.2E-01	0.65	0.33	1.29	1.8E-01	6.2E-02	0.71	0.28	1.79	4.1E-01	3.6E-01	0.7	0.29	1.68	3.7E-01	3.9E-01
313	HAO1	8	MR Egger	0.68	0.39	1.19	1.4E-01	3.0E-01	0.91	0.48	1.74	7.4E-01	9.2E-01	0.29	0.08	0.97	4.6E-02	6.2E-02	0.51	0.09	2.8	3.7E-01	3.6E-01	0.48	0.09	2.58	3.3E-01	3.9E-01
314	IGSF8	5	Inverse variance weighted	1.05	0.92	1.21	4.8E-01	6.8E-01	1.12	0.91	1.4	2.9E-01	7.8E-01	0.98	0.73	1.32	8.8E-01	5.2E-01	1.17	0.59	2.29	6.6E-01	6.2E-01	0.91	0.53	1.58	7.5E-01	6.1E-01
314	IGSF8	5	Weighted median	1.09	0.92	1.29	3.1E-01	6.8E-01	1.25	1.02	1.52	3.0E-02	7.8E-01	1.01	0.7	1.45	9.7E-01	5.2E-01	1.08	0.61	1.91	8.0E-01	6.2E-01	0.83	0.51	1.38	4.8E-01	6.1E-01
314	IGSF8	5	Weighted mode	1.11	0.84	1.46	3.6E-01	6.8E-01	1.27	0.94	1.71	9.3E-02	7.8E-01	1.01	0.57	1.8	9.7E-01	5.2E-01	0.7	0.3	1.64	3.1E-01	6.2E-01	0.84	0.36	1.93	5.8E-01	6.1E-01
314	IGSF8	5	MR Egger	0.94	0.41	2.17	8.2E-01	6.8E-01	0.99	0.25	3.94	9.8E-01	7.8E-01	1.4	0.26	7.49	5.6E-01	5.2E-01	0.58	0.01	38.03	7.1E-01	6.2E-01	0.51	0.02	15.29	5.8E-01	6.1E-01
315	SERPINA7	3	Inverse variance weighted	0.96	0.88	1.04	3.4E-01	4.4E-01	0.96	0.87	1.06	4.3E-01	9.0E-01	0.97	0.76	1.24	8.2E-01	3.4E-01	0.77	0.55	1.09	1.4E-01	3.0E-01	1.11	0.86	1.43	4.2E-01	9.7E-01
315	SERPINA7	3	Weighted median	0.96	0.88	1.04	3.1E-01	4.4E-01	0.96	0.87	1.06	4.4E-01	9.0E-01	0.95	0.79	1.14	6.0E-01	3.4E-01	0.76	0.59	0.97	3.0E-02	3.0E-01	1.11	0.86	1.43	4.4E-01	9.7E-01
315	SERPINA7	3	Weighted mode	0.95	0.78	1.15	3.6E-01	4.4E-01	0.96	0.77	1.2	5.2E-01	9.0E-01	0.94	0.62	1.42	5.7E-01	3.4E-01	0.73	0.43	1.25	1.3E-01	3.0E-01	1.1	0.62	1.98	5.4E-01	9.7E-01
315	SERPINA7	3	MR Egger	0.92	0.46	1.85	3.7E-01	4.4E-01	0.96	0.43	2.13	6.0E-01	9.0E-01	0.86	0.19	3.85	4.2E-01	3.4E-01	0.64	0.09	4.74	2.1E-01	3.0E-01	1.1	0.14	8.92	6.5E-01	9.7E-01
316	ADAMTSL4	3	Inverse variance weighted	0.83	0.58	1.19	3.2E-01	4.7E-01	0.86	0.65	1.12	2.6E-01	6.7E-01	0.97	0.56	1.68	9.2E-01	3.7E-01	2.19	1.05	4.59	3.7E-02	4.1E-01	0.55	0.28	1.09	8.5E-02	6.1E-01
316	ADAMTSL4	3	Weighted median	0.8	0.58	1.08	1.5E-01	4.7E-01	0.87	0.63	1.21	4.0E-01	6.7E-01	0.92	0.5	1.7	8.0E-01	3.7E-01	2.68	1.1	6.55	3.0E-02	4.1E-01	0.54	0.24	1.18	1.2E-01	6.1E-01
316	ADAMTSL4	3	Weighted mode	0.71	0.28	1.81	2.6E-01	4.7E-01	0.89	0.36	2.19	6.2E-01	6.7E-01	0.77	0.1	6.11	6.5E-01	3.7E-01	3.33	0.19	58.49	2.1E-01	4.1E-01	0.47	0.05	4.68	2.9E-01	6.1E-01
316	ADAMTSL4	3	MR Egger	0.61	0.01	44.25	3.8E-01	4.7E-01	0.73	0.01	42.57	5.1E-01	6.7E-01	0.51	0	281.85	4.0E-01	3.7E-01	0.95	0	11999.07	9.5E-01	4.1E-01	0.37	0	1413.63	3.7E-01	6.1E-01
317	AHSG	2	Inverse variance weighted	1.05	1	1.09	3.0E-02	NA	1.04	0.99	1.09	1.1E-01	NA	1.08	0.99	1.18	8.3E-02	NA	1.05	0.93	1.19	4.0E-01	NA	0.93	0.83	1.05	2.5E-01	NA
318	PAG1	4	Inverse variance weighted	0.87	0.71	1.06	1.6E-01	9.7E-01	0.89	0.71	1.12	3.3E-01	7.6E-01	0.76	0.49	1.17	2.1E-01	7.5E-01	0.82	0.45	1.51	5.3E-01	9.2E-01	0.52	0.28	0.94	3.0E-02	5.6E-01
318	PAG1	4	Weighted median	0.85	0.67	1.07	1.7E-01	9.7E-01	0.89	0.68	1.18	4.3E-01	7.6E-01	0.81	0.47	1.4	4.5E-01	7.5E-01	0.82	0.4	1.68	5.9E-01	9.2E-01	0.62	0.3	1.26	1.9E-01	5.6E-01
318	PAG1	4	Weighted mode	0.77	0.4	1.48	2.9E-01	9.7E-01	0.9	0.53	1.53	5.6E-01	7.6E-01	0.92	0.24	3.49	8.6E-01	7.5E-01	0.82	0.2	3.39	6.9E-01	9.2E-01	0.62	0.17	2.29	3.3E-01	5.6E-01
318	PAG1	4	MR Egger	0.89	0.07	11.74	8.6E-01	9.7E-01	1.13	0.06	21.99	8.8E-01	7.6E-01	0.47	0	121.54	6.2E-01	7.5E-01	1.02	0	2345.25	9.9E-01	9.2E-01	1.76	0	3651.38	7.8E-01	5.6E-01
319	ATP5IF1	3	Inverse variance weighted	0.84	0.69	1.02	8.5E-02	6.6E-01	0.91	0.73	1.14	4.1E-01	4.3E-01	1.03	0.67	1.59	8.9E-01	6.0E-01	0.72	0.4	1.29	2.7E-01	7.0E-01	0.53	0.3	0.95	3.2E-02	6.3E-01
319	ATP5IF1	3	Weighted median	0.85	0.69	1.05	1.2E-01	6.6E-01	0.91	0.72	1.16	4.6E-01	4.3E-01	0.9	0.56	1.44	6.6E-01	6.0E-01	0.64	0.32	1.26	2.0E-01	7.0E-01	0.51	0.27	0.94	3.1E-02	6.3E-01
319	ATP5IF1	3	Weighted mode	0.87	0.52	1.47	3.8E-01	6.6E-01	1	0.55	1.83	1.0E+00	4.3E-01	0.9	0.3	2.65	7.0E-01	6.0E-01	0.63	0.13	2.95	3.3E-01	7.0E-01	0.48	0.1	2.37	1.9E-01	6.3E-01
319	ATP5IF1	3	MR Egger	1.02	0.01	71.83	9.6E-01	6.6E-01	1.44	0.01	196.64	5.2E-01	4.3E-01	0.57	0	30166.74	6.3E-01	6.0E-01	0.44	0	167774.36	5.6E-01	7.0E-01	0.28	0	99422.1	4.3E-01	6.3E-01
320	SCARA5	5	Inverse variance weighted	1.11	0.93	1.32	2.4E-01	2.6E-01	1.17	0.95	1.43	1.5E-01	2.6E-01	1.06	0.86	1.3	6.0E-01	9.8E-01	0.98	0.77	1.26	8.9E-01	7.4E-01	0.96	0.72	1.26	7.5E-01	1.4E-01
320	SCARA5	5	Weighted median	1.07	0.98	1.17	1.4E-01	2.6E-01	1.12	1.01	1.24	3.1E-02	2.6E-01	1.08	0.89	1.29	4.4E-01	9.8E-01	0.99	0.77	1.27	9.5E-01	7.4E-01	0.86	0.67	1.09	2.1E-01	1.4E-01
320	SCARA5	5	Weighted mode	1.05	0.91	1.21	3.7E-01	2.6E-01	1.1	0.94	1.28	1.6E-01	2.6E-01	1.07	0.81	1.42	5.3E-01	9.8E-01	0.99	0.68	1.45	9.6E-01	7.4E-01	0.86	0.61	1.23	3.1E-01	1.4E-01
320	SCARA5	5	MR Egger	0.93	0.58	1.49	6.6E-01	2.6E-01	0.95	0.54	1.67	7.8E-01	2.6E-01	1.06	0.51	2.2	8.1E-01	9.8E-01	1.05	0.5	2.22	8.4E-01	7.4E-01	0.65	0.31	1.35	1.6E-01	1.4E-01
321	UNC79	1	Wald ratio	0.91	0.74	1.12	3.7E-01	NA	0.87	0.68	1.1	2.5E-01	NA	1.35	0.86	2.11	2.0E-01	NA	1.23	0.66	2.31	5.2E-01	NA	0.5	0.27	0.94	3.1E-02	NA
322	PTN	3	Inverse variance weighted	1.17	0.89	1.53	2.5E-01	9.8E-01	1.41	1.03	1.92	3.1E-02	6.2E-01	0.94	0.51	1.74	8.5E-01	4.0E-01	1.02	0.32	3.23	9.8E-01	5.1E-01	1.1	0.33	3.66	8.7E-01	7.1E-01
322	PTN	3	Weighted median	1.15	0.85	1.54	3.7E-01	9.8E-01	1.36	0.96	1.94	8.5E-02	6.2E-01	1.07	0.53	2.15	8.6E-01	4.0E-01	0.82	0.27	2.43	7.1E-01	5.1E-01	0.67	0.24	1.84	4.3E-01	7.1E-01
322	PTN	3	Weighted mode	1.12	0.49	2.56	6.3E-01	9.8E-01	1.33	0.51	3.51	3.3E-01	6.2E-01	1.2	0.12	11.84	7.7E-01	4.0E-01	0.7	0.04	13.12	6.5E-01	5.1E-01	0.65	0.06	7.66	5.3E-01	7.1E-01
322	PTN	3	MR Egger	1.22	0	69970408.95	9.1E-01	9.8E-01	0.48	0	440561764.2	7.3E-01	6.2E-01	62.7	0	3.22446E+18	4.0E-01	4.0E-01	369.07	0	8.50694E+35	5.1E-01	5.1E-01	49.03	0	3.16107E+45	7.1E-01	7.1E-01
323	LTA	8	Inverse variance weighted	1.01	0.95	1.09	7.0E-01	1.6E-01	1.04	0.96	1.12	3.2E-01	1.6E-01	0.93	0.84	1.04	2.0E-01	2.3E-01	0.95	0.86	1.06	3.7E-01	3.3E-01	1.05	0.94	1.17	3.9E-01	5.3E-01
323	LTA	8	Weighted median	1.02	0.98	1.06	3.0E-01	1.6E-01	1.05	1	1.09	3.1E-02	1.6E-01	0.94	0.87	1.01	8.3E-02	2.3E-01	0.96	0.86	1.06	4.0E-01	3.3E-01	1.05	0.94	1.17	4.0E-01	5.3E-01
323	LTA	8	Weighted mode	1.02	0.98	1.07	3.0E-01	1.6E-01	1.05	1	1.1	6.7E-02	1.6E-01	0.94	0.86	1.03	1.3E-01	2.3E-01	0.96	0.85	1.08	4.2E-01	3.3E-01	1.05	0.92	1.19	4.3E-01	5.3E-01
323	LTA	8	MR Egger	1.04	0.95	1.13	3.3E-01	1.6E-01	1.07	0.97	1.18	1.5E-01	1.6E-01	0.96	0.84	1.11	5.3E-01	2.3E-01	0.98	0.85	1.13	7.2E-01	3.3E-01	1.06	0.92	1.23	3.3E-01	5.3E-01
324	NELL1	16	Inverse variance weighted	1	0.94	1.07	9.6E-01	7.0E-01	1.01	0.94	1.09	7.8E-01	6.1E-01	0.91	0.8	1.05	2.0E-01	3.9E-01	1.01	0.79	1.29	9.5E-01	2.8E-03	1.03	0.77	1.38	8.5E-01	1.3E-01
324	NELL1	16	Weighted median	0.98	0.91	1.06	6.0E-01	7.0E-01	0.99	0.91	1.08	8.7E-01	6.1E-01	0.89	0.76	1.05	1.7E-01	3.9E-01	1.2	0.96	1.5	1.0E-01	2.8E-03	0.89	0.71	1.12	3.2E-01	1.3E-01
324	NELL1	16	Weighted mode	0.98	0.89	1.07	5.8E-01	7.0E-01	0.99	0.9	1.09	8.3E-01	6.1E-01	0.89	0.74	1.07	1.8E-01	3.9E-01	1.2	0.94	1.53	1.4E-01	2.8E-03	0.88	0.68	1.14	3.2E-01	1.3E-01
324	NELL1	16	MR Egger	0.99	0.9	1.09	8.3E-01	7.0E-01	0.99	0.89	1.1	9.0E-01	6.1E-01	0.87	0.71	1.06	1.4E-01	3.9E-01	1.34	1.03	1.74	3.1E-02	2.8E-03	0.84	0.56	1.26	3.8E-01	1.3E-01
325	MMP15	1	Wald ratio	0.89	0.54	1.45	6.4E-01	NA	1.14	0.64	2	6.6E-01	NA	1.28	0.45	3.66	6.4E-01	NA	0.19	0.04	0.86	3.1E-02	NA	0.39	0.09	1.7	2.1E-01	NA
326	PAM	4	Inverse variance weighted	1.03	0.97	1.09	3.1E-01	5.0E-01	1.01	0.95	1.07	8.1E-01	8.0E-01	1.15	1.01	1.3	3.1E-02	7.8E-01	0.97	0.82	1.14	7.0E-01	5.5E-01	1.07	0.83	1.39	6.0E-01	7.9E-01
326	PAM	4	Weighted median	1.03	0.98	1.09	2.7E-01	5.0E-01	1.01	0.95	1.07	8.1E-01	8.0E-01	1.15	1.01	1.3	3.1E-02	7.8E-01	0.97	0.82	1.14	7.2E-01	5.5E-01	1.06	0.9	1.25	5.1E-01	7.9E-01
326	PAM	4	Weighted mode	1.03	0.94	1.13	3.4E-01	5.0E-01	1.01	0.9	1.13	8.2E-01	8.0E-01	1.15	0.95	1.39	1.0E-01	7.8E-01	0.98	0.75	1.3	8.6E-01	5.5E-01	1.07	0.81	1.41	4.8E-01	7.9E-01
326	PAM	4	MR Egger	1.04	0.91	1.2	3.3E-01	5.0E-01	1.01	0.86	1.19	7.6E-01	8.0E-01	1.16	0.85	1.58	1.8E-01	7.8E-01	1	0.66	1.51	9.9E-01	5.5E-01	1.04	0.48	2.27	8.3E-01	7.9E-01
327	S100A4	4	Inverse variance weighted	0.97	0.84	1.13	7.2E-01	2.7E-01	0.98	0.8	1.19	8.3E-01	1.9E-01	1.05	0.61	1.81	8.6E-01	6.9E-01	1.11	0.71	1.74	6.3E-01	4.2E-01	0.6	0.38	0.96	3.1E-02	6.3E-01
327	S100A4	4	Weighted median	0.95	0.81	1.12	5.4E-01	2.7E-01	0.97	0.8	1.16	7.1E-01	1.9E-01	0.96	0.68	1.38	8.4E-01	6.9E-01	1.14	0.72	1.8	5.7E-01	4.2E-01	0.58	0.35	0.96	3.3E-02	6.3E-01
327	S100A4	4	Weighted mode	0.93	0.72	1.21	4.6E-01	2.7E-01	0.91	0.67	1.24	4.0E-01	1.9E-01	0.98	0.54	1.78	9.1E-01	6.9E-01	1.16	0.55	2.46	5.8E-01	4.2E-01	0.58	0.23	1.42	1.5E-01	6.3E-01
327	S100A4	4	MR Egger	0.87	0.55	1.37	3.2E-01	2.7E-01	0.83	0.49	1.4	2.6E-01	1.9E-01	0.91	0.13	6.37	8.5E-01	6.9E-01	1.39	0.36	5.4	4.1E-01	4.2E-01	0.53	0.12	2.32	2.0E-01	6.3E-01
328	HJV	4	Inverse variance weighted	1.04	0.93	1.16	4.7E-01	7.4E-01	0.99	0.87	1.12	8.7E-01	7.2E-01	1.31	1.02	1.68	3.1E-02	9.4E-01	1.35	0.9	2.01	1.4E-01	3.8E-01	0.79	0.57	1.1	1.6E-01	9.6E-01
328	HJV	4	Weighted median	1.03	0.91	1.17	6.0E-01	7.4E-01	0.98	0.84	1.13	7.4E-01	7.2E-01	1.32	0.99	1.77	5.8E-02	9.4E-01	1.33	0.92	1.92	1.3E-01	3.8E-01	0.8	0.54	1.17	2.5E-01	9.6E-01
328	HJV	4	Weighted mode	1.03	0.82	1.29	6.9E-01	7.4E-01	0.98	0.76	1.26	7.7E-01	7.2E-01	1.14	0.69	1.9	4.6E-01	9.4E-01	1.34	0.72	2.51	2.3E-01	3.8E-01	0.79	0.4	1.55	3.5E-01	9.6E-01
328	HJV	4	MR Egger	1.01	0.67	1.52	9.1E-01	7.4E-01	0.96	0.6	1.53	7.2E-01	7.2E-01	1.33	0.47	3.8	3.6E-01	9.4E-01	1.01	0.24	4.17	9.9E-01	3.8E-01	0.8	0.24	2.66	5.2E-01	9.6E-01
329	CXCL3	3	Inverse variance weighted	1.07	0.87	1.32	5.3E-01	5.6E-01	1.03	0.81	1.32	8.0E-01	5.9E-01	1.65	1.05	2.62	3.2E-02	4.7E-01	1.98	1.04	3.75	3.7E-02	8.0E-01	0.95	0.5	1.8	8.8E-01	7.6E-01
329	CXCL3	3	Weighted median	1.04	0.82	1.32	7.2E-01	5.6E-01	1.02	0.78	1.33	9.0E-01	5.9E-01	1.54	0.92	2.58	9.9E-02	4.7E-01	1.95	0.96	4	6.6E-02	8.0E-01	1.05	0.53	2.07	8.8E-01	7.6E-01
329	CXCL3	3	Weighted mode	1.17	0.64	2.14	3.8E-01	5.6E-01	1.13	0.58	2.2	5.1E-01	5.9E-01	1.35	0.35	5.29	4.4E-01	4.7E-01	2.15	0.31	14.84	2.3E-01	8.0E-01	1.05	0.21	5.32	9.0E-01	7.6E-01
329	CXCL3	3	MR Egger	1.73	0	2821.95	5.2E-01	5.6E-01	1.7	0	8920.37	5.8E-01	5.9E-01	0.42	0	4004644.19	6.2E-01	4.7E-01	3.43	0	18507517043	6.1E-01	8.0E-01	1.9	0	8928557810	7.8E-01	7.6E-01
330	MPRIP	1	Wald ratio	0.71	0.47	1.07	1.0E-01	NA	0.65	0.4	1.05	8.0E-02	NA	0.87	0.35	2.15	7.6E-01	NA	0.25	0.07	0.89	3.2E-02	NA	2.01	0.56	7.2	2.8E-01	NA
331	MELTF	8	Inverse variance weighted	1.08	1.01	1.17	3.2E-02	8.3E-01	1.07	0.99	1.16	6.9E-02	4.6E-01	1.16	0.96	1.39	1.2E-01	9.1E-01	0.96	0.74	1.24	7.3E-01	9.3E-01	1.05	0.86	1.27	6.5E-01	9.3E-01
331	MELTF	8	Weighted median	1.08	1	1.15	4.5E-02	8.3E-01	1.06	0.98	1.15	1.5E-01	4.6E-01	1.14	0.97	1.33	1.0E-01	9.1E-01	0.98	0.78	1.23	8.8E-01	9.3E-01	1.05	0.85	1.29	6.4E-01	9.3E-01
331	MELTF	8	Weighted mode	1.07	0.99	1.17	8.8E-02	8.3E-01	1.06	0.96	1.17	2.0E-01	4.6E-01	1.14	0.94	1.38	1.6E-01	9.1E-01	1.01	0.77	1.33	9.1E-01	9.3E-01	1.04	0.79	1.36	7.6E-01	9.3E-01
331	MELTF	8	MR Egger	1.07	0.93	1.23	2.6E-01	8.3E-01	1.04	0.91	1.19	4.9E-01	4.6E-01	1.14	0.81	1.62	3.8E-01	9.1E-01	0.97	0.59	1.58	8.7E-01	9.3E-01	1.04	0.73	1.47	8.1E-01	9.3E-01
332	CPA2	4	Inverse variance weighted	0.93	0.74	1.17	5.2E-01	3.0E-01	0.94	0.69	1.27	6.8E-01	3.2E-01	0.84	0.71	0.98	3.2E-02	3.9E-01	0.92	0.74	1.14	4.4E-01	9.6E-01	0.96	0.68	1.36	8.1E-01	1.5E-01
332	CPA2	4	Weighted median	0.97	0.9	1.04	4.1E-01	3.0E-01	0.99	0.91	1.08	8.8E-01	3.2E-01	0.87	0.74	1.02	7.8E-02	3.9E-01	0.91	0.73	1.13	3.9E-01	9.6E-01	1.05	0.84	1.31	6.9E-01	1.5E-01
332	CPA2	4	Weighted mode	0.98	0.87	1.11	6.4E-01	3.0E-01	1.01	0.87	1.17	8.7E-01	3.2E-01	0.86	0.65	1.14	2.0E-01	3.9E-01	0.9	0.62	1.3	4.3E-01	9.6E-01	1.05	0.72	1.51	7.2E-01	1.5E-01
332	CPA2	4	MR Egger	1.19	0.49	2.89	4.9E-01	3.0E-01	1.29	0.39	4.27	4.6E-01	3.2E-01	0.97	0.49	1.94	8.9E-01	3.9E-01	0.91	0.35	2.37	7.1E-01	9.6E-01	1.51	0.56	4.03	2.1E-01	1.5E-01
333	LCN2	16	Inverse variance weighted	0.94	0.86	1.03	1.9E-01	6.4E-01	0.99	0.89	1.1	8.2E-01	6.1E-01	0.97	0.8	1.17	7.3E-01	8.7E-01	0.74	0.57	0.97	3.2E-02	8.0E-01	0.79	0.59	1.06	1.2E-01	7.5E-01
333	LCN2	16	Weighted median	0.98	0.87	1.11	7.4E-01	6.4E-01	1.01	0.87	1.18	8.6E-01	6.1E-01	0.93	0.72	1.21	5.9E-01	8.7E-01	0.88	0.6	1.27	4.8E-01	8.0E-01	0.87	0.59	1.28	4.8E-01	7.5E-01
333	LCN2	16	Weighted mode	1	0.83	1.19	9.8E-01	6.4E-01	1.05	0.85	1.31	6.1E-01	6.1E-01	0.88	0.58	1.33	5.1E-01	8.7E-01	0.89	0.53	1.5	6.4E-01	8.0E-01	0.88	0.48	1.62	6.5E-01	7.5E-01
333	LCN2	16	MR Egger	1	0.76	1.3	9.7E-01	6.4E-01	1.06	0.78	1.44	6.9E-01	6.1E-01	0.93	0.52	1.66	7.9E-01	8.7E-01	0.81	0.36	1.82	5.9E-01	8.0E-01	0.7	0.29	1.7	4.0E-01	7.5E-01
334	CD63	10	Inverse variance weighted	1.11	1.01	1.23	3.2E-02	1.4E-01	1.13	1.01	1.27	3.4E-02	5.5E-01	1.16	0.88	1.55	2.9E-01	9.5E-02	1.17	0.87	1.57	3.0E-01	7.7E-01	1.03	0.69	1.56	8.7E-01	1.5E-01
334	CD63	10	Weighted median	1.03	0.91	1.16	6.6E-01	1.4E-01	1.12	0.97	1.29	1.2E-01	5.5E-01	1.03	0.76	1.4	8.6E-01	9.5E-02	0.94	0.62	1.41	7.6E-01	7.7E-01	0.77	0.51	1.17	2.3E-01	1.5E-01
334	CD63	10	Weighted mode	1.05	0.9	1.22	5.0E-01	1.4E-01	1.1	0.91	1.34	2.8E-01	5.5E-01	0.96	0.68	1.35	7.7E-01	9.5E-02	0.82	0.49	1.38	4.1E-01	7.7E-01	0.75	0.45	1.28	2.5E-01	1.5E-01
334	CD63	10	MR Egger	0.95	0.74	1.22	6.6E-01	1.4E-01	1.06	0.79	1.4	6.7E-01	5.5E-01	0.74	0.4	1.38	3.0E-01	9.5E-02	1.07	0.49	2.34	8.5E-01	7.7E-01	0.57	0.22	1.51	2.2E-01	1.5E-01
335	VTA1	3	Inverse variance weighted	0.82	0.64	1.05	1.1E-01	8.8E-01	0.73	0.55	0.97	3.2E-02	5.8E-01	1.18	0.7	2	5.3E-01	5.0E-01	1.08	0.52	2.26	8.3E-01	4.2E-01	0.59	0.29	1.23	1.6E-01	9.1E-01
335	VTA1	3	Weighted median	0.84	0.64	1.1	2.0E-01	8.8E-01	0.74	0.53	1.02	6.4E-02	5.8E-01	1.05	0.57	1.93	8.7E-01	5.0E-01	1.12	0.47	2.69	7.9E-01	4.2E-01	0.62	0.27	1.46	2.8E-01	9.1E-01
335	VTA1	3	Weighted mode	0.84	0.39	1.85	4.5E-01	8.8E-01	0.79	0.32	1.93	3.7E-01	5.8E-01	0.97	0.18	5.16	9.5E-01	5.0E-01	0.79	0.06	10.2	7.2E-01	4.2E-01	0.66	0.07	6.19	5.0E-01	9.1E-01
335	VTA1	3	MR Egger	1	0	437335.69	1.0E+00	8.8E-01	1.83	0	6078466.71	7.0E-01	5.8E-01	0.13	0	1.97338E+11	5.3E-01	5.0E-01	0.02	0	2.14796E+15	4.3E-01	4.2E-01	0.9	0	5.68204E+16	9.8E-01	9.1E-01
336	KLK8	5	Inverse variance weighted	0.93	0.81	1.07	3.3E-01	9.3E-01	0.95	0.82	1.1	4.8E-01	9.2E-01	1.06	0.9	1.26	4.8E-01	3.0E-01	0.87	0.64	1.18	3.7E-01	7.8E-01	0.9	0.72	1.12	3.5E-01	7.1E-01
336	KLK8	5	Weighted median	0.92	0.86	0.99	3.2E-02	9.3E-01	0.94	0.86	1.02	1.4E-01	9.2E-01	1.04	0.89	1.21	6.2E-01	3.0E-01	0.84	0.66	1.06	1.4E-01	7.8E-01	0.89	0.72	1.12	3.2E-01	7.1E-01
336	KLK8	5	Weighted mode	0.93	0.83	1.03	1.2E-01	9.3E-01	0.94	0.83	1.06	2.2E-01	9.2E-01	1.04	0.83	1.3	6.5E-01	3.0E-01	0.84	0.59	1.18	2.3E-01	7.8E-01	0.89	0.65	1.22	3.7E-01	7.1E-01
336	KLK8	5	MR Egger	0.93	0.69	1.26	5.0E-01	9.3E-01	0.94	0.68	1.3	6.0E-01	9.2E-01	1	0.74	1.34	9.9E-01	3.0E-01	0.84	0.44	1.62	4.7E-01	7.8E-01	0.93	0.61	1.4	6.0E-01	7.1E-01
337	NEFL	1	Wald ratio	0.95	0.54	1.67	8.5E-01	NA	0.81	0.42	1.56	5.3E-01	NA	0.61	0.18	2.11	4.4E-01	NA	0.69	0.12	3.86	6.7E-01	NA	6.42	1.17	35.26	3.3E-02	NA
338	FCRL2	14	Inverse variance weighted	1.04	0.99	1.08	1.2E-01	7.3E-01	1.04	0.99	1.1	1.1E-01	7.2E-01	1.01	0.93	1.1	8.0E-01	2.4E-02	0.96	0.85	1.08	4.8E-01	5.1E-01	1.13	1.01	1.27	3.3E-02	8.0E-01
338	FCRL2	14	Weighted median	1.03	0.99	1.08	1.7E-01	7.3E-01	1.03	0.98	1.09	2.0E-01	7.2E-01	0.98	0.89	1.08	7.0E-01	2.4E-02	0.96	0.84	1.09	5.1E-01	5.1E-01	1.1	0.96	1.25	1.8E-01	8.0E-01
338	FCRL2	14	Weighted mode	1.03	0.98	1.08	2.1E-01	7.3E-01	1.03	0.97	1.1	2.6E-01	7.2E-01	0.98	0.88	1.08	6.5E-01	2.4E-02	0.96	0.84	1.1	5.0E-01	5.1E-01	1.11	0.96	1.29	1.6E-01	8.0E-01
338	FCRL2	14	MR Egger	1.03	0.97	1.1	3.4E-01	7.3E-01	1.03	0.96	1.11	3.2E-01	7.2E-01	0.93	0.83	1.05	2.1E-01	2.4E-02	0.99	0.84	1.16	8.7E-01	5.1E-01	1.15	0.98	1.34	8.6E-02	8.0E-01
339	LY96	11	Inverse variance weighted	0.96	0.85	1.09	5.6E-01	2.8E-02	0.95	0.81	1.12	5.6E-01	3.0E-02	0.93	0.7	1.24	6.2E-01	8.7E-01	1.02	0.69	1.49	9.3E-01	9.5E-02	0.89	0.62	1.29	5.4E-01	4.1E-01
339	LY96	11	Weighted median	0.91	0.76	1.08	2.6E-01	2.8E-02	0.88	0.72	1.07	2.0E-01	3.0E-02	0.92	0.63	1.34	6.6E-01	8.7E-01	0.95	0.57	1.57	8.3E-01	9.5E-02	0.94	0.59	1.49	7.8E-01	4.1E-01
339	LY96	11	Weighted mode	0.88	0.64	1.2	3.7E-01	2.8E-02	0.84	0.61	1.16	2.5E-01	3.0E-02	0.99	0.52	1.89	9.8E-01	8.7E-01	0.82	0.38	1.79	5.9E-01	9.5E-02	1.06	0.49	2.31	8.7E-01	4.1E-01
339	LY96	11	MR Egger	0.74	0.57	0.97	3.3E-02	2.8E-02	0.71	0.52	0.96	3.3E-02	3.0E-02	0.89	0.47	1.71	7.0E-01	8.7E-01	0.59	0.27	1.3	1.6E-01	9.5E-02	0.69	0.32	1.52	3.2E-01	4.1E-01
340	MCEMP1	12	Inverse variance weighted	1.05	0.98	1.12	1.4E-01	7.5E-02	1.05	0.96	1.15	2.7E-01	6.2E-02	1.05	0.91	1.21	5.0E-01	9.6E-01	1.1	0.9	1.35	3.5E-01	7.7E-01	0.85	0.7	1.04	1.2E-01	3.9E-02
340	MCEMP1	12	Weighted median	1.03	0.95	1.12	4.5E-01	7.5E-02	1.06	0.97	1.17	2.1E-01	6.2E-02	1.03	0.86	1.23	7.3E-01	9.6E-01	1.07	0.83	1.39	5.9E-01	7.7E-01	0.97	0.75	1.26	8.4E-01	3.9E-02
340	MCEMP1	12	Weighted mode	1.05	0.95	1.17	3.0E-01	7.5E-02	1.07	0.97	1.19	1.5E-01	6.2E-02	1.02	0.81	1.27	8.7E-01	9.6E-01	1.12	0.83	1.51	4.3E-01	7.7E-01	1	0.75	1.33	1.0E+00	3.9E-02
340	MCEMP1	12	MR Egger	1.15	1.01	1.3	3.3E-02	7.5E-02	1.18	1.01	1.36	3.5E-02	6.2E-02	1.04	0.79	1.37	7.3E-01	9.6E-01	1.15	0.77	1.73	4.6E-01	7.7E-01	1.17	0.81	1.7	3.6E-01	3.9E-02
341	FAM3B	6	Inverse variance weighted	0.98	0.88	1.11	8.0E-01	6.0E-01	0.95	0.83	1.1	4.9E-01	3.0E-01	1.06	0.93	1.21	3.8E-01	8.6E-01	1	0.85	1.17	1.0E+00	4.9E-01	1.16	0.91	1.47	2.3E-01	8.7E-01
341	FAM3B	6	Weighted median	0.99	0.94	1.04	6.2E-01	6.0E-01	0.98	0.93	1.03	4.6E-01	3.0E-01	1.04	0.94	1.16	4.5E-01	8.6E-01	0.94	0.82	1.09	4.2E-01	4.9E-01	1.18	1.01	1.37	3.3E-02	8.7E-01
341	FAM3B	6	Weighted mode	1	0.93	1.07	9.4E-01	6.0E-01	0.98	0.91	1.05	4.8E-01	3.0E-01	1.04	0.9	1.2	5.4E-01	8.6E-01	0.94	0.76	1.16	4.7E-01	4.9E-01	1.13	0.92	1.37	1.9E-01	8.7E-01
341	FAM3B	6	MR Egger	1.02	0.8	1.31	8.3E-01	6.0E-01	1.03	0.79	1.35	7.7E-01	3.0E-01	1.05	0.78	1.41	6.9E-01	8.6E-01	0.94	0.68	1.3	6.3E-01	4.9E-01	1.13	0.67	1.92	5.5E-01	8.7E-01
342	PARK7	2	Inverse variance weighted	0.94	0.8	1.1	4.1E-01	NA	0.98	0.82	1.18	8.4E-01	NA	0.89	0.63	1.25	5.0E-01	NA	0.69	0.43	1.11	1.3E-01	NA	0.6	0.37	0.96	3.3E-02	NA
343	NT5C	3	Inverse variance weighted	0.99	0.94	1.04	6.1E-01	5.9E-01	0.97	0.92	1.03	3.4E-01	6.2E-01	1.14	0.97	1.35	1.1E-01	6.6E-01	0.96	0.83	1.12	6.1E-01	9.0E-01	0.92	0.8	1.06	2.7E-01	7.7E-01
343	NT5C	3	Weighted median	0.99	0.94	1.04	6.6E-01	5.9E-01	0.97	0.92	1.03	3.7E-01	6.2E-01	1.13	1.01	1.27	3.3E-02	6.6E-01	0.96	0.82	1.12	6.0E-01	9.0E-01	0.92	0.8	1.07	2.8E-01	7.7E-01
343	NT5C	3	Weighted mode	0.99	0.89	1.1	7.0E-01	5.9E-01	0.97	0.86	1.1	4.5E-01	6.2E-01	1.13	0.87	1.47	1.8E-01	6.6E-01	0.95	0.69	1.32	6.0E-01	9.0E-01	0.92	0.68	1.26	3.9E-01	7.7E-01
343	NT5C	3	MR Egger	0.99	0.7	1.42	8.8E-01	5.9E-01	0.98	0.65	1.48	6.5E-01	6.2E-01	1.12	0.27	4.62	5.1E-01	6.6E-01	0.96	0.26	3.49	7.3E-01	9.0E-01	0.93	0.34	2.59	5.4E-01	7.7E-01
344	TRIM25	1	Wald ratio	1.4	1.03	1.91	3.3E-02	NA	1.42	1	2.04	5.3E-02	NA	1.37	0.7	2.69	3.6E-01	NA	1.44	0.56	3.68	4.5E-01	NA	1.45	0.57	3.65	4.3E-01	NA
345	LYPD3	14	Inverse variance weighted	1.01	0.92	1.1	8.8E-01	4.8E-01	0.98	0.88	1.08	6.4E-01	6.0E-01	0.99	0.88	1.12	9.3E-01	6.9E-01	1.18	1.01	1.37	3.3E-02	5.1E-01	0.99	0.82	1.2	9.5E-01	2.0E-01
345	LYPD3	14	Weighted median	1.05	0.98	1.12	1.5E-01	4.8E-01	1.04	0.96	1.12	3.6E-01	6.0E-01	0.89	0.76	1.05	1.7E-01	6.9E-01	1.19	0.97	1.46	1.0E-01	5.1E-01	1.11	0.9	1.36	3.4E-01	2.0E-01
345	LYPD3	14	Weighted mode	1.06	0.99	1.13	9.7E-02	4.8E-01	1.03	0.95	1.12	4.6E-01	6.0E-01	0.88	0.7	1.11	2.6E-01	6.9E-01	1.21	0.97	1.51	9.2E-02	5.1E-01	1.11	0.89	1.38	3.4E-01	2.0E-01
345	LYPD3	14	MR Egger	1.05	0.89	1.23	5.3E-01	4.8E-01	1.01	0.84	1.23	9.0E-01	6.0E-01	0.96	0.78	1.2	7.2E-01	6.9E-01	1.25	0.96	1.63	8.8E-02	5.1E-01	1.16	0.84	1.61	3.3E-01	2.0E-01
346	DECR1	3	Inverse variance weighted	1.06	0.86	1.31	6.0E-01	6.4E-01	1.25	0.97	1.61	8.1E-02	7.8E-01	1.03	0.63	1.67	9.1E-01	6.6E-01	0.65	0.29	1.47	3.0E-01	6.2E-01	0.99	0.44	2.22	9.7E-01	9.7E-01
346	DECR1	3	Weighted median	1.03	0.82	1.3	7.8E-01	6.4E-01	1.22	0.92	1.62	1.7E-01	7.8E-01	1.13	0.64	2.02	6.7E-01	6.6E-01	0.53	0.29	0.95	3.3E-02	6.2E-01	0.89	0.44	1.8	7.5E-01	9.7E-01
346	DECR1	3	Weighted mode	1.01	0.58	1.77	9.3E-01	6.4E-01	1.19	0.59	2.41	3.9E-01	7.8E-01	1.24	0.3	5.1	5.9E-01	6.6E-01	0.52	0.13	2.02	1.7E-01	6.2E-01	0.85	0.15	4.92	7.3E-01	9.7E-01
346	DECR1	3	MR Egger	0.99	0.14	6.81	9.6E-01	6.4E-01	1.19	0.12	12.35	5.1E-01	7.8E-01	1.24	0.01	307.91	7.0E-01	6.6E-01	0.5	0	1339.76	4.6E-01	6.2E-01	0.96	0	27019.42	9.7E-01	9.7E-01
347	EGFL7	15	Inverse variance weighted	1.04	0.95	1.13	3.9E-01	5.6E-01	1.11	1.01	1.23	3.3E-02	2.4E-01	0.88	0.73	1.05	1.6E-01	6.8E-01	0.89	0.68	1.16	3.9E-01	2.8E-01	0.99	0.72	1.38	9.8E-01	9.1E-01
347	EGFL7	15	Weighted median	1.04	0.92	1.17	5.3E-01	5.6E-01	1.02	0.88	1.18	7.5E-01	2.4E-01	0.89	0.69	1.15	3.8E-01	6.8E-01	0.81	0.57	1.15	2.4E-01	2.8E-01	0.96	0.68	1.36	8.4E-01	9.1E-01
347	EGFL7	15	Weighted mode	1.05	0.89	1.22	5.5E-01	5.6E-01	1	0.82	1.23	9.7E-01	2.4E-01	0.94	0.67	1.32	7.0E-01	6.8E-01	0.77	0.51	1.18	2.1E-01	2.8E-01	0.87	0.59	1.3	4.7E-01	9.1E-01
347	EGFL7	15	MR Egger	1.09	0.89	1.33	3.7E-01	5.6E-01	1.25	0.99	1.58	5.7E-02	2.4E-01	0.94	0.61	1.45	7.8E-01	6.8E-01	0.67	0.36	1.24	1.8E-01	2.8E-01	0.96	0.43	2.13	9.1E-01	9.1E-01
348	ICAM5	5	Inverse variance weighted	0.95	0.86	1.04	2.7E-01	2.0E-01	0.94	0.83	1.05	2.5E-01	4.1E-01	0.98	0.88	1.09	6.7E-01	5.1E-01	0.98	0.88	1.09	6.9E-01	2.1E-01	0.89	0.76	1.04	1.5E-01	9.0E-02
348	ICAM5	5	Weighted median	0.96	0.91	1	6.3E-02	2.0E-01	0.99	0.93	1.05	6.6E-01	4.1E-01	0.97	0.89	1.06	5.5E-01	5.1E-01	0.99	0.89	1.11	9.0E-01	2.1E-01	0.88	0.78	0.99	3.3E-02	9.0E-02
348	ICAM5	5	Weighted mode	0.99	0.93	1.07	8.3E-01	2.0E-01	0.99	0.92	1.07	8.2E-01	4.1E-01	1.01	0.9	1.15	7.7E-01	5.1E-01	0.99	0.84	1.18	8.9E-01	2.1E-01	0.9	0.76	1.06	1.6E-01	9.0E-02
348	ICAM5	5	MR Egger	1.02	0.84	1.26	7.3E-01	2.0E-01	1	0.75	1.33	9.9E-01	4.1E-01	1.03	0.78	1.35	7.9E-01	5.1E-01	1.08	0.83	1.42	4.3E-01	2.1E-01	1.04	0.8	1.35	6.8E-01	9.0E-02
349	CCL15	2	Inverse variance weighted	0.89	0.78	1.02	8.9E-02	NA	1.04	0.89	1.21	6.6E-01	NA	0.73	0.55	0.98	3.3E-02	NA	0.63	0.38	1.05	7.4E-02	NA	0.76	0.51	1.13	1.7E-01	NA
350	PTPRS	4	Inverse variance weighted	1.09	0.98	1.21	1.2E-01	9.7E-01	1.14	1.01	1.29	3.8E-02	9.3E-01	1.02	0.79	1.32	8.6E-01	6.1E-01	0.96	0.69	1.34	8.3E-01	9.4E-01	0.77	0.55	1.09	1.4E-01	3.6E-01
350	PTPRS	4	Weighted median	1.09	0.97	1.23	1.3E-01	9.7E-01	1.15	1.01	1.32	3.3E-02	9.3E-01	1.01	0.79	1.3	9.3E-01	6.1E-01	0.95	0.67	1.35	7.9E-01	9.4E-01	0.76	0.53	1.08	1.3E-01	3.6E-01
350	PTPRS	4	Weighted mode	1.09	0.9	1.32	2.3E-01	9.7E-01	1.15	0.92	1.44	1.3E-01	9.3E-01	1.01	0.66	1.54	9.4E-01	6.1E-01	0.95	0.54	1.69	8.0E-01	9.4E-01	0.66	0.35	1.27	1.4E-01	3.6E-01
350	PTPRS	4	MR Egger	1.09	0.79	1.5	3.6E-01	9.7E-01	1.14	0.79	1.64	2.7E-01	9.3E-01	0.95	0.41	2.2	8.1E-01	6.1E-01	0.98	0.37	2.59	9.3E-01	9.4E-01	0.64	0.23	1.77	2.0E-01	3.6E-01
351	FRZB	3	Inverse variance weighted	1	0.94	1.06	9.0E-01	7.3E-01	0.97	0.9	1.05	4.5E-01	3.6E-01	1.03	0.91	1.16	6.7E-01	9.3E-01	0.94	0.78	1.14	5.2E-01	4.0E-01	1.21	1.02	1.45	3.4E-02	4.7E-01
351	FRZB	3	Weighted median	1	0.94	1.06	9.3E-01	7.3E-01	0.97	0.91	1.04	4.1E-01	3.6E-01	1.03	0.91	1.17	6.6E-01	9.3E-01	0.93	0.78	1.1	3.8E-01	4.0E-01	1.2	1.01	1.44	3.8E-02	4.7E-01
351	FRZB	3	Weighted mode	1	0.87	1.15	9.6E-01	7.3E-01	0.98	0.84	1.14	6.6E-01	3.6E-01	1.03	0.77	1.37	7.2E-01	9.3E-01	0.93	0.62	1.37	4.9E-01	4.0E-01	1.18	0.81	1.71	2.0E-01	4.7E-01

351	FRZB	3	MR Egger	1.01	0.62	1.65	8.7E-01	7.3E-01	1.02	0.58	1.79	7.8E-01	3.6E-01	1.03	0.35	3.02	7.6E-01	9.3E-01	0.85	0.19	3.69	3.9E-01	4.0E-01	1.12	0.24	5.1	5.3E-01	4.7E-01
352	PPY	8	Inverse variance weighted	1	0.88	1.13	9.9E-01	2.6E-02	1.01	0.86	1.19	8.7E-01	1.0E-02	0.89	0.73	1.08	2.3E-01	6.6E-01	1.02	0.78	1.33	9.1E-01	9.0E-01	0.97	0.75	1.27	8.4E-01	4.6E-01
352	PPY	8	Weighted median	0.97	0.87	1.08	5.8E-01	2.6E-02	0.95	0.83	1.09	4.6E-01	1.0E-02	0.92	0.73	1.16	4.9E-01	6.6E-01	0.96	0.69	1.32	7.8E-01	9.0E-01	0.97	0.7	1.34	8.4E-01	4.6E-01
352	PPY	8	Weighted mode	0.96	0.85	1.1	5.1E-01	2.6E-02	0.95	0.82	1.1	4.1E-01	1.0E-02	0.93	0.7	1.23	5.6E-01	6.6E-01	0.94	0.64	1.38	7.2E-01	9.0E-01	0.97	0.64	1.46	8.6E-01	4.6E-01
352	PPY	8	MR Egger	0.84	0.71	1.01	6.1E-02	2.6E-02	0.79	0.65	0.98	3.4E-02	1.0E-02	0.84	0.57	1.24	3.1E-01	6.6E-01	0.99	0.58	1.69	9.7E-01	9.0E-01	0.85	0.5	1.45	4.9E-01	4.6E-01
353	ADGRG2	5	Inverse variance weighted	1.33	0.84	2.09	2.3E-01	1.1E-01	1.32	0.74	2.35	3.5E-01	8.7E-02	1.04	0.58	1.85	9.0E-01	4.8E-01	0.76	0.41	1.42	3.9E-01	8.3E-01	1.97	1.05	3.69	3.4E-02	7.2E-01
353	ADGRG2	5	Weighted median	1.02	0.78	1.34	9.0E-01	1.1E-01	0.97	0.72	1.31	8.5E-01	8.7E-02	0.93	0.5	1.74	8.3E-01	4.8E-01	0.74	0.34	1.61	4.5E-01	8.3E-01	2.32	1.06	5.06	3.5E-02	7.2E-01
353	ADGRG2	5	Weighted mode	0.99	0.65	1.52	9.7E-01	1.1E-01	0.94	0.58	1.51	7.4E-01	8.7E-02	0.74	0.18	2.96	5.8E-01	4.8E-01	0.87	0.2	3.67	8.0E-01	8.3E-01	2.55	0.62	10.5	1.4E-01	7.2E-01
353	ADGRG2	5	MR Egger	0.71	0.25	2	3.8E-01	1.1E-01	0.58	0.17	1.95	2.5E-01	8.7E-02	0.68	0.1	4.77	5.7E-01	4.8E-01	0.86	0.12	5.87	8.1E-01	8.3E-01	1.61	0.24	11.01	4.9E-01	7.2E-01
354	LDLRAP1	2	Inverse variance weighted	1	0.73	1.37	9.9E-01	NA	1.22	0.85	1.75	2.9E-01	NA	0.48	0.24	0.95	3.4E-02	NA	0.79	0.31	2.01	6.2E-01	NA	0.62	0.25	1.58	3.2E-01	NA
355	CRIM1	3	Inverse variance weighted	0.81	0.55	1.21	3.1E-01	6.3E-01	0.74	0.45	1.2	2.2E-01	6.0E-01	0.84	0.53	1.33	4.6E-01	8.3E-01	0.59	0.36	0.96	3.4E-02	6.0E-01	0.97	0.53	1.79	9.3E-01	7.6E-01
355	CRIM1	3	Weighted median	0.91	0.76	1.08	2.8E-01	6.3E-01	0.84	0.69	1.02	8.3E-02	6.0E-01	0.85	0.57	1.25	4.1E-01	8.3E-01	0.63	0.36	1.08	9.3E-02	6.0E-01	1.12	0.67	1.88	6.6E-01	7.6E-01
355	CRIM1	3	Weighted mode	0.91	0.62	1.32	3.8E-01	6.3E-01	0.85	0.54	1.32	2.4E-01	6.0E-01	0.81	0.25	2.67	5.3E-01	8.3E-01	0.75	0.17	3.38	5.0E-01	6.0E-01	1.15	0.36	3.72	6.6E-01	7.6E-01
355	CRIM1	3	MR Egger	1.66	0	2767787.74	7.3E-01	6.3E-01	1.93	0	50930533.37	7.1E-01	6.0E-01	0.56	0	96825502.28	7.6E-01	8.3E-01	1.55	0	41946097.73	8.0E-01	6.0E-01	2.03	0	88808250442	7.8E-01	7.6E-01
356	MLN	20	Inverse variance weighted	0.95	0.91	1	5.0E-02	7.8E-01	0.94	0.89	1	3.4E-02	9.7E-01	0.93	0.83	1.04	1.9E-01	1.0E+00	0.94	0.81	1.1	4.5E-01	7.6E-01	1.02	0.87	1.19	8.0E-01	9.9E-01
356	MLN	20	Weighted median	0.96	0.9	1.02	1.5E-01	7.8E-01	0.94	0.88	1	6.3E-02	9.7E-01	0.93	0.82	1.05	2.6E-01	1.0E+00	0.95	0.8	1.13	5.6E-01	7.6E-01	1.06	0.88	1.26	5.5E-01	9.9E-01
356	MLN	20	Weighted mode	0.96	0.9	1.02	1.7E-01	7.8E-01	0.94	0.88	1.01	8.3E-02	9.7E-01	0.93	0.82	1.06	2.7E-01	1.0E+00	0.95	0.79	1.13	5.4E-01	7.6E-01	1.06	0.88	1.29	5.2E-01	9.9E-01
356	MLN	20	MR Egger	0.96	0.89	1.04	2.9E-01	7.8E-01	0.94	0.86	1.03	1.8E-01	9.7E-01	0.93	0.78	1.11	4.0E-01	1.0E+00	0.92	0.73	1.17	4.7E-01	7.6E-01	1.02	0.79	1.31	8.7E-01	9.9E-01
357	NPTX1	14	Inverse variance weighted	1.02	0.93	1.12	6.3E-01	2.5E-01	1.02	0.91	1.14	7.9E-01	6.3E-01	1.03	0.9	1.18	6.4E-01	4.3E-02	1.14	0.99	1.32	5.9E-02	3.9E-01	0.93	0.8	1.09	3.8E-01	3.6E-01
357	NPTX1	14	Weighted median	1.05	0.99	1.11	7.7E-02	2.5E-01	1.04	0.98	1.11	2.2E-01	6.3E-01	1.1	0.98	1.24	1.0E-01	4.3E-02	1.18	1	1.38	5.3E-02	3.9E-01	0.95	0.81	1.13	5.8E-01	3.6E-01
357	NPTX1	14	Weighted mode	1.06	1	1.11	3.4E-02	2.5E-01	1.04	0.97	1.11	2.4E-01	6.3E-01	1.1	0.97	1.24	1.3E-01	4.3E-02	1.16	0.97	1.37	8.9E-02	3.9E-01	0.96	0.8	1.16	6.4E-01	3.6E-01
357	NPTX1	14	MR Egger	1.07	0.94	1.23	2.7E-01	2.5E-01	1.04	0.88	1.23	6.2E-01	6.3E-01	1.15	0.98	1.36	8.7E-02	4.3E-02	1.21	0.99	1.48	6.6E-02	3.9E-01	0.99	0.79	1.25	9.6E-01	3.6E-01
358	HBZ	6	Inverse variance weighted	1	0.97	1.03	9.2E-01	6.7E-01	0.99	0.96	1.02	5.5E-01	6.1E-01	1.06	0.99	1.13	9.0E-02	8.7E-01	1.09	1.01	1.19	3.4E-02	8.9E-01	0.98	0.9	1.07	6.4E-01	9.8E-01
358	HBZ	6	Weighted median	1	0.97	1.03	9.3E-01	6.7E-01	0.99	0.96	1.02	5.4E-01	6.1E-01	1.06	1	1.13	5.5E-02	8.7E-01	1.1	1.01	1.19	3.6E-02	8.9E-01	0.98	0.9	1.07	6.5E-01	9.8E-01
358	HBZ	6	Weighted mode	1	0.97	1.04	9.7E-01	6.7E-01	0.99	0.95	1.03	5.5E-01	6.1E-01	1.06	0.98	1.15	1.0E-01	8.7E-01	1.09	0.98	1.23	1.0E-01	8.9E-01	0.98	0.88	1.1	6.9E-01	9.8E-01
358	HBZ	6	MR Egger	1	0.95	1.04	8.9E-01	6.7E-01	0.98	0.93	1.04	4.7E-01	6.1E-01	1.06	0.94	1.2	2.4E-01	8.7E-01	1.1	0.96	1.26	1.3E-01	8.9E-01	0.98	0.85	1.13	7.0E-01	9.8E-01
359	LECT2	8	Inverse variance weighted	1.01	0.94	1.09	7.1E-01	8.4E-01	1	0.92	1.1	9.6E-01	9.7E-01	1.08	1	1.16	4.2E-02	3.8E-01	1.03	0.93	1.14	5.8E-01	5.4E-01	1.02	0.89	1.17	7.8E-01	9.9E-01
359	LECT2	8	Weighted median	1.02	0.98	1.05	3.5E-01	8.4E-01	1	0.97	1.04	8.8E-01	9.7E-01	1.08	1.01	1.16	3.5E-02	3.8E-01	1.03	0.93	1.14	5.6E-01	5.4E-01	1.02	0.93	1.13	6.6E-01	9.9E-01
359	LECT2	8	Weighted mode	1.02	0.98	1.06	3.6E-01	8.4E-01	1	0.96	1.05	8.9E-01	9.7E-01	1.09	0.99	1.19	6.2E-02	3.8E-01	1.03	0.91	1.16	6.2E-01	5.4E-01	1.02	0.91	1.16	6.5E-01	9.9E-01
359	LECT2	8	MR Egger	1.02	0.91	1.14	7.0E-01	8.4E-01	1	0.87	1.16	9.6E-01	9.7E-01	1.1	0.99	1.22	6.7E-02	3.8E-01	1.05	0.91	1.22	4.5E-01	5.4E-01	1.02	0.82	1.27	8.4E-01	9.9E-01
360	CX3CL1	6	Inverse variance weighted	0.82	0.56	1.2	3.1E-01	4.9E-02	0.71	0.48	1.03	7.3E-02	1.2E-01	1.13	0.58	2.21	7.3E-01	5.8E-02	1.36	0.79	2.36	2.7E-01	2.1E-01	1.18	0.45	3.07	7.4E-01	2.8E-02
360	CX3CL1	6	Weighted median	0.94	0.71	1.24	6.6E-01	4.9E-02	0.88	0.64	1.2	4.1E-01	1.2E-01	0.78	0.47	1.28	3.3E-01	5.8E-02	1.25	0.67	2.31	4.8E-01	2.1E-01	1.41	0.58	3.43	4.5E-01	2.8E-02
360	CX3CL1	6	Weighted mode	1.11	0.32	3.82	8.3E-01	4.9E-02	0.89	0.25	3.17	8.2E-01	1.2E-01	0.71	0.36	1.42	2.6E-01	5.8E-02	1.17	0.46	3.02	6.8E-01	2.1E-01	0.36	0.11	1.15	7.3E-02	2.8E-02
360	CX3CL1	6	MR Egger	0.29	0.1	0.87	3.5E-02	4.9E-02	0.3	0.08	1.11	6.3E-02	1.2E-01	0.19	0.03	1.38	8.1E-02	5.8E-02	0.46	0.05	3.95	3.8E-01	2.1E-01	0.08	0.01	0.84	4.1E-02	2.8E-02
361	CXCL11	8	Inverse variance weighted	1.08	0.97	1.2	1.8E-01	1.8E-01	1.05	0.93	1.19	4.0E-01	4.0E-01	1.16	0.96	1.39	1.2E-01	9.5E-02	1.32	1.02	1.7	3.5E-02	3.5E-01	1.19	0.87	1.61	2.7E-01	3.6E-01
361	CXCL11	8	Weighted median	1.01	0.92	1.12	7.8E-01	1.8E-01	1.01	0.9	1.13	9.1E-01	4.0E-01	1.06	0.85	1.32	6.3E-01	9.5E-02	1.19	0.88	1.62	2.6E-01	3.5E-01	1.19	0.86	1.64	2.9E-01	3.6E-01
361	CXCL11	8	Weighted mode	1.02	0.9	1.15	7.3E-01	1.8E-01	1	0.87	1.15	9.8E-01	4.0E-01	1.03	0.78	1.35	8.2E-01	9.5E-02	1.2	0.82	1.75	3.0E-01	3.5E-01	1.2	0.78	1.86	3.5E-01	3.6E-01
361	CXCL11	8	MR Egger	0.96	0.76	1.2	6.4E-01	1.8E-01	0.97	0.73	1.28	7.7E-01	4.0E-01	0.86	0.56	1.32	4.3E-01	9.5E-02	1.07	0.59	1.94	7.8E-01	3.5E-01	0.92	0.44	1.92	7.8E-01	3.6E-01
362	NUB1	4	Inverse variance weighted	1.15	0.97	1.37	1.1E-01	9.8E-01	1.17	0.98	1.4	9.1E-02	8.0E-01	1.1	0.82	1.47	5.4E-01	7.0E-01	0.95	0.63	1.42	7.9E-01	8.1E-01	1.15	0.74	1.78	5.3E-01	8.7E-01
362	NUB1	4	Weighted median	1.17	1.01	1.37	4.2E-02	9.8E-01	1.22	1.01	1.46	3.5E-02	8.0E-01	1.07	0.77	1.49	6.7E-01	7.0E-01	0.9	0.58	1.41	6.6E-01	8.1E-01	1.12	0.72	1.74	6.0E-01	8.7E-01
362	NUB1	4	Weighted mode	1.19	0.89	1.6	1.6E-01	9.8E-01	1.24	0.91	1.68	1.2E-01	8.0E-01	1.07	0.59	1.91	7.5E-01	7.0E-01	0.96	0.43	2.17	8.9E-01	8.1E-01	1.15	0.52	2.56	6.2E-01	8.7E-01
362	NUB1	4	MR Egger	1.15	0.51	2.62	5.3E-01	9.8E-01	1.23	0.53	2.86	4.1E-01	8.0E-01	0.99	0.32	3.1	9.8E-01	7.0E-01	1.03	0.21	5	9.5E-01	8.1E-01	1.24	0.16	9.67	7.0E-01	8.7E-01
363	SLIT2	1	Wald ratio	0.62	0.39	0.99	4.6E-02	NA	0.56	0.33	0.96	3.5E-02	NA	0.71	0.26	1.94	5.1E-01	NA	1.1	0.27	4.45	8.9E-01	NA	0.58	0.15	2.33	4.5E-01	NA
364	HBEGF	19	Inverse variance weighted	1.06	0.96	1.17	2.8E-01	1.5E-01	1.06	0.95	1.18	3.0E-01	2.4E-01	1.21	1.01	1.45	3.5E-02	8.7E-01	1.22	0.92	1.63	1.7E-01	6.0E-01	1.19	0.92	1.54	1.8E-01	9.2E-01
364	HBEGF	19	Weighted median	1.04	0.93	1.15	5.1E-01	1.5E-01	1	0.88	1.12	9.4E-01	2.4E-01	1.12	0.88	1.43	3.6E-01	8.7E-01	1.24	0.9	1.7	1.8E-01	6.0E-01	1.19	0.86	1.65	3.0E-01	9.2E-01
364	HBEGF	19	Weighted mode	1.03	0.91	1.16	6.5E-01	1.5E-01	1.01	0.87	1.17	8.8E-01	2.4E-01	1.2	0.89	1.61	2.1E-01	8.7E-01	1.29	0.9	1.85	1.5E-01	6.0E-01	1.21	0.83	1.77	3.0E-01	9.2E-01
364	HBEGF	19	MR Egger	0.91	0.72	1.15	4.1E-01	1.5E-01	0.93	0.72	1.19	5.4E-01	2.4E-01	1.17	0.75	1.82	4.6E-01	8.7E-01	1.04	0.51	2.12	9.1E-01	6.0E-01	1.16	0.61	2.2	6.3E-01	9.2E-01
365	PTPRB	7	Inverse variance weighted	1	0.92	1.08	9.9E-01	4.4E-01	1.01	0.93	1.11	7.9E-01	3.1E-01	0.95	0.8	1.13	5.6E-01	9.8E-01	1.01	0.79	1.27	9.6E-01	5.7E-01	0.79	0.62	1.01	6.5E-02	7.8E-01
365	PTPRB	7	Weighted median	1.01	0.92	1.1	8.7E-01	4.4E-01	1.03	0.93	1.14	5.8E-01	3.1E-01	0.95	0.79	1.14	5.5E-01	9.8E-01	0.99	0.77	1.29	9.7E-01	5.7E-01	0.75	0.57	0.98	3.6E-02	7.8E-01
365	PTPRB	7	Weighted mode	1.01	0.91	1.13	8.1E-01	4.4E-01	1.03	0.91	1.18	5.6E-01	3.1E-01	0.94	0.74	1.21	5.9E-01	9.8E-01	0.98	0.7	1.37	9.0E-01	5.7E-01	0.74	0.52	1.05	8.1E-02	7.8E-01
365	PTPRB	7	MR Egger	1.03	0.9	1.18	6.0E-01	4.4E-01	1.06	0.9	1.25	3.8E-01	3.1E-01	0.95	0.7	1.29	6.7E-01	9.8E-01	0.94	0.6	1.46	7.2E-01	5.7E-01	0.77	0.49	1.2	1.8E-01	7.8E-01
366	TSPAN8	12	Inverse variance weighted	0.92	0.84	0.99	3.7E-02	4.3E-01	0.96	0.87	1.05	3.8E-01	6.2E-01	0.87	0.7	1.08	2.1E-01	8.8E-01	0.88	0.64	1.2	4.1E-01	9.3E-01	0.85	0.57	1.27	4.3E-01	4.5E-01
366	TSPAN8	12	Weighted median	0.89	0.8	1	5.3E-02	4.3E-01	0.98	0.85	1.11	7.2E-01	6.2E-01	0.83	0.64	1.08	1.7E-01	8.8E-01	0.93	0.63	1.39	7.4E-01	9.3E-01	0.8	0.56	1.15	2.2E-01	4.5E-01
366	TSPAN8	12	Weighted mode	0.89	0.77	1.04	1.2E-01	4.3E-01	1.03	0.87	1.21	7.1E-01	6.2E-01	0.78	0.56	1.08	1.2E-01	8.8E-01	0.58	0.35	0.96	3.6E-02	9.3E-01	0.82	0.54	1.26	3.3E-01	4.5E-01
366	TSPAN8	12	MR Egger	0.98	0.79	1.22	8.5E-01	4.3E-01	1.01	0.79	1.29	9.4E-01	6.2E-01	0.9	0.5	1.63	7.1E-01	8.8E-01	0.85	0.37	1.97	6.8E-01	9.3E-01	1.19	0.41	3.43	7.2E-01	4.5E-01
367	CLEC4D	7	Inverse variance weighted	1.03	0.93	1.14	5.8E-01	6.5E-01	1.04	0.92	1.16	5.6E-01	7.8E-01	0.99	0.79	1.23	9.2E-01	2.7E-01	0.87	0.64	1.18	3.6E-01	6.8E-01	1.4	1.02	1.93	3.6E-02	6.2E-01
367	CLEC4D	7	Weighted median	1.09	0.96	1.23	1.9E-01	6.5E-01	1.11	0.96	1.28	1.6E-01	7.8E-01	1.02	0.78	1.33	9.0E-01	2.7E-01	0.98	0.68	1.41	9.2E-01	6.8E-01	1.52	1.02	2.25	3.8E-02	6.2E-01
367	CLEC4D	7	Weighted mode	1.09	0.93	1.29	2.3E-01	6.5E-01	1.12	0.92	1.35	2.1E-01	7.8E-01	1.09	0.75	1.6	5.8E-01	2.7E-01	0.93	0.59	1.46	7.1E-01	6.8E-01	1.57	0.9	2.72	9.2E-02	6.2E-01
367	CLEC4D	7	MR Egger	1.11	0.72	1.73	5.6E-01	6.5E-01	1.09	0.66	1.81	6.7E-01	7.8E-01	1.53	0.59	3.99	3.0E-01	2.7E-01	1.08	0.28	4.18	8.9E-01	6.8E-01	1.89	0.43	8.35	3.2E-01	6.2E-01
368	C4BPB	11	Inverse variance weighted	1.01	0.93	1.09	8.1E-01	3.6E-01	1.01	0.93	1.1	8.1E-01	8.0E-01	1.04	0.88	1.24	6.5E-01	2.8E-01	0.93	0.72	1.21	6.0E-01	1.6E-02	1.08	0.86	1.34	5.2E-01	9.2E-01
368	C4BPB	11	Weighted median	0.99	0.91	1.08	8.3E-01	3.6E-01	1	0.91	1.1	9.9E-01	8.0E-01	0.98	0.81	1.19	8.6E-01	2.8E-01	0.74	0.56	0.98	3.6E-02	1.6E-02	1.09	0.84	1.4	5.3E-01	9.2E-01
368	C4BPB	11	Weighted mode	0.99	0.9	1.09	8.9E-01	3.6E-01	1	0.89	1.13	9.7E-01	8.0E-01	0.99	0.8	1.22	8.8E-01	2.8E-01	0.77	0.56	1.05	8.7E-02	1.6E-02	1.09	0.82	1.45	5.1E-01	9.2E-01
368	C4BPB	11	MR Egger	0.98	0.87	1.1	7.1E-01	3.6E-01	1	0.88	1.14	9.7E-01	8.0E-01	0.96	0.75	1.23	7.4E-01	2.8E-01	0.7	0.49	1	4.8E-02	1.6E-02	1.07	0.76	1.5	6.8E-01	9.2E-01
369	NRP1	7	Inverse variance weighted	0.96	0.89	1.04	3.2E-01	9.4E-01	0.97	0.89	1.06	5.6E-01	6.2E-01	0.89	0.76	1.06	1.9E-01	5.5E-01	0.78	0.62	0.98	3.6E-02	6.7E-01	0.83	0.66	1.05	1.3E-01	9.0E-01
369	NRP1	7	Weighted median	0.97	0.89	1.05	4.2E-01	9.4E-01	0.97	0.88	1.07	5.1E-01	6.2E-01	0.87	0.73	1.04	1.3E-01	5.5E-01	0.8	0.62	1.03	8.7E-02	6.7E-01	0.82	0.63	1.06	1.3E-01	9.0E-01
369	NRP1	7	Weighted mode	0.97	0.86	1.08	4.8E-01	9.4E-01	0.97	0.86	1.1	5.8E-01	6.2E-01	0.86	0.68	1.09	1.8E-01	5.5E-01	0.81	0.58	1.13	1.7E-01	6.7E-01	0.81	0.58	1.13	1.7E-01	9.0E-01
369	NRP1	7	MR Egger	0.96	0.81	1.13	5.2E-01	9.4E-01	0.95	0.78	1.14	4.7E-01	6.2E-01	0.84	0.59	1.19	2.4E-01	5.5E-01	0.83	0.51	1.36	3.8E-01	6.7E-01	0.82	0.5	1.33	3.4E-01	9.0E-01
370	TGFA	1	Wald ratio	0.87	0.69	1.09	2.2E-01	NA	0.88	0.68	1.15	3.6E-01	NA	1.14	0.7	1.86	6.0E-01	NA	0.47	0.23	0.95	3.6E-02	NA	0.98	0.49	1.94	9.4E-01	NA
371	ATRAID	3	Inverse variance weighted	1.03	0.94	1.13	5.4E-01	6.5E-01	0.99	0.89	1.1	8.6E-01	8.9E-01	1.24	1.01	1.51	3.8E-02	4.4E-01	1.11	0.84	1.47	4.7E-01	4.9E-01	1.12	0.85	1.48	4.2E-01	8.0E-01
371	ATRAID	3	Weighted median	1.03	0.93	1.13	6.1E-01	6.5E-01	0.99	0.88	1.1	8.0E-01	8.9E-01	1.25	1.01	1.55	3.6E-02	4.4E-01	1.1	0.82	1.48	5.4E-01	4.9E-01	1.1	0.81	1.49	5.4E-01	8.0E-01
371	ATRAID	3	Weighted mode	1.01	0.81	1.28	8.1E-01	6.5E-01	0.98	0.75	1.28	7.9E-01	8.9E-01	1.25	0.79	1.99	1.7E-01	4.4E-01	1.06	0.55	2.02	7.4E-01	4.9E-01	1.08	0.55	2.11	6.9E-01	8.0E-01
371	ATRAID	3	MR Egger	1.18	0.06	21.96	6.0E-01	6.5E-01	1.04	0.03	31.08	9.1E-01	8.9E-01	2.22	0	1223.29	3.6E-01	4.4E-01	2.22	0	15033.68	4.6E-01	4.9E-01	1.39	0	7743.69	7.1E-01	8.0E-01
372	ANPEP	5	Inverse variance weighted	1.01	0.91	1.11	9.2E-01	9.5E-01	0.95	0.86	1.05	3.3E-01	2.1E-01	1.14	0.95	1.37	1.7E-01	3.0E-01	1.03	0.83	1.29	7.7E-01	1.6E-01	1.07	0.85	1.36	5.6E-01	4.9E-01
372	ANPEP	5	Weighted median	1	0.93	1.08	9.4E-01	9.5E-01	0.94	0.86	1.02	1.4E-01	2.1E-01	1.18	1.01	1.38	3.6E-02	3.0E-01	1.08	0.86	1.34	5.1E-01	1.6E-01	1.05	0.84	1.31	6.7E-01	4.9E-01
372	ANPEP	5	Weighted mode	1	0.91	1.11	9.2E-01	9.5E-01	0.94	0.83	1.06	2.1E-01	2.1E-01	1.19	0.94	1.5	1.1E-01	3.0E-01	1.08	0.78	1.5	5.3E-01	1.6E-01	1.05	0.77	1.44	6.9E-01	4.9E-01
372	ANPEP	5	MR Egger	1	0.81	1.25	9.7E-01	9.5E-01	0.91	0.78	1.07	1.7E-01	2.1E-01	1.22	0.87	1.71	1.5E-01	3.0E-01	1.17	0.78	1.76	3.1E-01	1.6E-01	1.01	0.62	1.63	9.6E-01	4.9E-01
373	VEGFA	9	Inverse variance weighted	0.99	0.94	1.04	7.1E-01	1.3E-01	1.01	0.95	1.07	7.6E-01	3.9E-01	0.93	0.85	1.01	7.2E-02	1.9E-01	0.95	0.81	1.11	5.3E-01	2.7E-01	1.01	0.86	1.18	9.0E-01	2.8E-01
373	VEGFA	9	Weighted median	0.99	0.95	1.03	4.7E-01	1.3E-01	1.01	0.96	1.05	7.5E-01	3.9E-01	0.91	0.84	0.99	3.6E-02	1.9E-01	0.93	0.83	1.05	2.3E-01	2.7E-01	0.99	0.89	1.12	9.3E-01	2.8E-01
373	VEGFA	9	Weighted mode	0.99	0.94	1.03	5.0E-01	1.3E-01	1.01	0.95	1.07	8.2E-01	3.9E-01	0.92	0.83	1.02	8.8E-02	1.9E-01	0.93	0.81	1.07	2.7E-01	2.7E-01	0.98	0.85	1.13	7.3E-01	2.8E-01
373	VEGFA	9	MR Egger	0.96	0.9	1.03	2.1E-01	1.3E-01	0.99	0.9	1.08	7.4E-01	3.9E-01	0.88	0.78	1	5.0E-02	1.9E-01	0.88	0.7	1.12	2.5E-01	2.7E-01	0.94	0.73	1.19	5.4E-01	2.8E-01
374	FSTL1	7	Inverse variance weighted	0.87	0.63	1.19	3.7E-01	5.2E-01	0.88	0.58	1.32	5.4E-01	7.0E-01	0.73	0.55	0.98	3.6E-02	3.5E-01	1.22	0.81	1.82	3.4E-01	1.6E-01	0.65	0.43	0.98	4.0E-02	7.6E-01
374	FSTL1	7	Weighted median	0.97	0.81	1.15	7.0E-01	5.2E-01	1.01	0.83	1.23	9.1E-01	7.0E-01	0.88	0.61	1.28	5.1E-01	3.5E-01	1.12	0.68	1.84	6.7E-01	1.6E-01	0.68	0.41	1.15	1.5E-01	7.6E-01
374	FSTL1	7	Weighted mode	0.97	0.79	1.2	7.4E-01	5.2E-01	1.03	0.81	1.31	7.7E-01	7.0E-01	0.89	0.54	1.46	5.8E-01	3.5E-01	1.11	0.55	2.24	7.3E-01	1.6E-01	0.67	0.33	1.37	2.2E-01	7.6E-01
374	FSTL1	7	MR Egger	1.12	0.4	3.15	7.9E-01	5.2E-01	1.08	0.27	4.3	9.0E-01	7.0E-01	1.03	0.4	2.6	9.5E-01	3.5E-01	2.44	0.73	8.21	1.2E-01	1.6E-01	0.76	0.18	3.16	6.4E-01	7.6E-01
375	CDH5	9	Inverse variance weighted	1.01	0.97	1.05	6.4E-01	7.3E-01	1.02	0.98	1.06	3.7E-01	5.2E-01	0.97	0.89	1.05	4.5E-01	2.0E-01	1.01	0.88	1.16	9.1E-01	9.4E-01	0.88	0.78	1	4.4E-02	9.4E-01
375	CDH5	9	Weighted median	1.01	0.97	1.05	7.0E-01	7.3E-01	1.02	0.97	1.06	5.1E-01	5.2E-01	0.98	0.9	1.07	6.9E-01	2.0E-01	1.01	0.89	1.13	9.1E-01	9.4E-01	0.87	0.76	0.99	3.7E-02	9.4E-01
375	CDH5	9	Weighted mode	1.01	0.96	1.06	7.0E-01	7.3E-01	1.02	0.96	1.07	4.9E-01	5.2E-01	0.98	0.88	1.09	6.9E-01	2.0E-01	1.01	0.89	1.15	9.0E-01	9.4E-01	0.87	0.76	1	4.5E-02	9.4E-01
375	CDH5	9	MR Egger	1.01	0.96	1.07	5.7E-01	7.3E-01	1.01	0.95	1.07	6.6E-01	5.2E-01	1	0.89	1.12	9.8E-01	2.0E-01	1.01	0.82	1.24	9.0E-01	9.4E-01	0.88	0.75	1.04	1.2E-01	9.4E-01
376	FGFBP2	10	Inverse variance weighted	1.02	0.97	1.07	4.3E-01	7.4E-01	1.05	0.99	1.12	1.3E-01	5.4E-01	0.98	0.9	1.06	6.2E-01	1.8E-01	1	0.89	1.13	1.0E+00	2.0E-01	1.07	0.93	1.23	3.6E-01	9.9E-01
376	FGFBP2	10	Weighted median	1.01	0.96	1.06	7.7E-01	7.4E-01	1.05	1	1.11	4.1E-02	5.4E-01	0.97	0.87	1.08	5.6E-01	1.8E-01	1.02	0.89	1.17	7.8E-01	2.0E-01	1.1	0.96	1.26	1.6E-01	9.9E-01
376	FGFBP2	10	Weighted mode	1.02	0.97	1.07	3.7E-01	7.4E-01	1.06	1	1.11	3.7E-02	5.4E-01	0.97	0.88	1.06	4.4E-01	1.8E-01	1.05	0.92	1.19	4.5E-01	2.0E-01	1.07	0.95	1.21	2.5E-01	9.9E-01
376	FGFBP2	10	MR Egger	1.03	0.96	1.1	4.4E-01	7.4E-01	1.06	0.97	1.16	1.5E-01	5.4E-01	0.94	0.85	1.05	2.6E-01	1.8E-01	1.05	0.9	1.23	4.7E-01	2.0E-01	1.07	0.87	1.32	4.8E-01	9.9E-01
377	GIMAP7	3	Inverse variance weighted	0.95	0.89	1.02	1.8E-01	4.5E-01	0.98	0.92	1.04	4.5E-01	5.1E-01	0.89	0.75	1.07	2.1E-01	2.7E-01	0.92	0.79	1.07	2.9E-01	8.2E-01	0.91	0.78	1.06	2.2E-01	8.2E-01
377	GIMAP7	3	Weighted median	0.95	0.9	1	3.7E-02	4.5E-01	0.98	0.92	1.03	4.0E-01	5.1E-01	0.89	0.8	0.99	3.7E-02	2.7E-01	0.92	0.78	1.08	2.9E-01	8.2E-01	0.91	0.78	1.06	2.2E-01	8.2E-01
377	GIMAP7	3	Weighted mode	0.95	0.85	1.06	1.6E-01	4.5E-01	0.97	0.85	1.12	5.0E-01	5.1E-01	0.88	0.69	1.12	1.5E-01	2.7E-01	0.92	0.66	1.28	3.8E-01	8.2E-01	0.9	0.65	1.25	3.1E-01	8.2E-01
377	GIMAP7	3	MR Egger	0.92	0.51	1.64	3.1E-01	4.5E-01	0.95	0.57	1.59	4.4E-01	5.1E-01	0.8	0.3	2.09	2.0E-01	2.7E-01	0.9	0.23	3.49	5.1E-01	8.2E-01	0.89	0.18	4.4	5.2E-01	8.2E-01
378	IL7	4	Inverse variance weighted	1.05	0.9	1.23	5.4E-01	4.1E-01	1.22	1.01	1.46	3.7E-02	2.6E-01	0.66	0.44	1	5.0E-02	7.8E-01	0.8	0.38	1.71	5.7E-01	3.6E-01	0.98	0.47	2.01	9.5E-01	1.5E-01
378	IL7	4	Weighted median	1.04	0.86	1.25	7.1E-01	4.1E-01	1.21	0.97	1.51	9.8E-02	2.6E-01	0.68	0.46	1.02	6.1E-02	7.8E-01	0.98	0.54	1.76	9.4E-01	3.6E-01	0.71	0.4	1.28	2.6E-01	1.5E-01
378	IL7	4	Weighted mode	1	0.71	1.43	9.7E-01	4.1E-01	1.14	0.75	1.74	3.9E-01	2.6E-01	0.69	0.34	1.42	2.0E-01	7.8E-01	1.11	0.41	2.98	7.6E-01	3.6E-01	0.71	0.26	1.93	3.5E-01	1.5E-01
378	IL7	4	MR Egger	0.88	0.38	2.01	5.6E-01	4.1E-01	0.88	0.34	2.31	6.4E-01	2.6E-01	0.79	0.06	10.02	7.3E-01	7.8E-01	2.04	0.05	83.74	5.0E-01	3.6E-01	0.29	0.03	3.47	1.7E-01	1.5E-01
379	CCL27	8	Inverse variance weighted	1	0.9	1.11	9.6E-01	5.8E-01	1.04	0.92	1.17	5.4E-01	6.3E-01	1.01	0.81	1.26	9.4E-01	4.3E-01	0.72	0.53	0.98	3.7E-02	7.8E-01	0.85	0.59	1.22	3.7E-01	8.7E-01
379	CCL27	8	Weighted median	1.02	0.9	1.15	7.8E-01	5.8E-01	1.05	0.91	1.2	5.3E-01	6.3E-01	1.01	0.77	1.32	9.6E-01	4.3E-01	0.71	0.5	1.03	6.9E-02	7.8E-01	0.83	0.57	1.2	3.2E-01	8.7E-01
379	CCL27	8	Weighted mode	1.02	0.88	1.18	7.6E-01	5.8E-01	1.05	0.87	1.27	5.7E-01	6.3E-01	1.01	0.72	1.43	9.3E-01	4.3E-01	0.72	0.44	1.16	1.5E-01	7.8E-01	0.87	0.53	1.42	5.1E-01	8.7E-01
379	CCL27	8	MR Egger	1.05	0.84	1.3	6.4E-01	5.8E-01	1.08	0.84	1.39	4.7E-01	6.3E-01	1.15	0.72	1.85	4.9E-01	4.3E-01	0.68	0.35	1.3	1.9E-01	7.8E-01	0.89	0.39	2.02	7.3E-01	8.7E-01
380	PF4	14	Inverse variance weighted	1.04	0.94	1.15	4.5E-01	5.5E-01	1.05	0.93	1.19	4.5E-01	5.7E-01	1.04	0.85	1.28	7.0E-01	5.1E-01	1.36	1.02	1.81	3.7E-02	3.6E-01	1.19	0.8	1.78	3.8E-01	4.2E-01
380	PF4	14	Weighted median	1.03	0.9	1.18	6.4E-01	5.5E-01	1.06	0.91	1.24	4.3E-01	5.7E-01	0.99	0.73	1.33	9.3E-01	5.1E-01	1.33	0.88	2.01	1.7E-01	3.6E-01	1.07	0.68	1.68	7.6E-01	4.2E-01
380	PF4	14	Weighted mode	1.06	0.88	1.28	5.2E-01	5.5E-01	1.12	0.89	1.41	3.2E-01	5.7E-01	0.93	0.52	1.66	7.8E-01	5.1E-01	1.39	0.71	2.71	3.1E-01	3.6E-01	0.89	0.46	1.72	7.1E-01	4.2E-01
380	PF4	14	MR Egger	0.96	0.69	1.31	7.6E-01	5.5E-01	0.95	0.64	1.4	7.8E-01	5.7E-01	1.26	0.66	2.41	4.6E-01	5.1E-01	0.94	0.39	2.28	8.9E-01	3.6E-01	0.77	0.22	2.66	6.5E-01	4.2E-01
381	DDAH1	3	Inverse variance weighted	1	0.87	1.15	9.7E-01	3.9E-01	0.96	0.82	1.12	6.0E-01	8.5E-01	1.28	0.86	1.91	2.3E-01	3.1E-01	0.84	0.55	1.28	4.2E-01	9.0E-01	0.9	0.59	1.36	6.0E-01	9.4E-01
381	DDAH1	3	Weighted median	1.04	0.89	1.2	6.4E-01	3.9E-01	0.96	0.81	1.14	6.5E-01	8.5E-01	1.44	1.02	2.02	3.7E-02	3.1E-01	0.92	0.58	1.46	7.2E-01	9.0E-01	0.92	0.59	1.43	7.0E-01	9.4E-01
381	DDAH1	3	Weighted mode	1.05	0.72	1.51	6.5E-01	3.9E-01	0.96	0.62	1.49	7.4E-01	8.5E-01	1.46	0.65	3.29	1.8E-01	3.1E-01	0.93	0.3	2.84	8.0E-01	9.0E-01	0.94	0.32	2.76	8.2E-01	9.4E-01
381	DDAH1	3	MR Egger	1.34	0.08	21.29	4.1E-01	3.9E-01	1.01	0.04	24.65	9.6E-01	8.5E-01	2.96	0.01	1187.09	2.6E-01	3.1E-01	0.77	0	3209.65	7.5E-01	9.0E-01	0.95	0	3586.27	9.5E-01	9.4E-01
382	MANEAL	1	Wald ratio	1.43	0.99	2.09	6.0E-02	NA	1.58	1.03	2.44	3.8E-02	NA	1.43	0.64	3.22	3.9E-01	NA	0.6	0.19	1.9	3.8E-01	NA	1.73	0.57	5.24	3.3E-01	NA
383	CCN2	17	Inverse variance weighted	1.03	0.94	1.12	5.6E-01	4.5E-01	1.02	0.93	1.11	7.2E-01	3.9E-01	1	0.86	1.17	9.9E-01	3.2E-01	1.24	1.01	1.52	3.8E-02	4.6E-01	1.2	0.95	1.53	1.3E-01	2.6E-01
383	CCN2	17	Weighted median	0.99	0.91	1.08	8.0E-01	4.5E-01	0.98	0.89	1.08	7.0E-01	3.9E-01	0.93	0.77	1.11	4.1E-01	3.2E-01	1.28	1	1.65	5.3E-02	4.6E-01	1.1	0.86	1.41	4.4E-01	2.6E-01
383	CCN2	17	Weighted mode	1	0.91	1.1	1.0E+00	4.5E-01	0.99	0.9	1.1	8.8E-01	3.9E-01	0.93	0.77	1.13	4.3E-01	3.2E-01	1.28	0.99	1.67	6.0E-02	4.6E-01	1.13	0.86	1.48	3.5E-01	2.6E-01
383	CCN2	17	MR Egger	0.98	0.84	1.14	8.0E-01	4.5E-01	0.97	0.82	1.13	6.4E-01	3.9E-01	0.9	0.69	1.18	4.4E-01	3.2E-01	1.38	0.96	1.98	8.1E-02	4.6E-01	1	0.66	1.53	1.0E+00	2.6E-01
384	CHMP1A	3	Inverse variance weighted	1.16	0.94	1.44	1.7E-01	5.9E-01	1.24	0.97	1.59	9.1E-02	4.1E-01	1.18	0.54	2.58	6.8E-01	2.5E-01	2	1.04	3.84	3.8E-02	8.5E-01	1.22	0.64	2.34	5.4E-01	7.8E-01
384	CHMP1A	3	Weighted median	1.2	0.94	1.52	1.4E-01	5.9E-01	1.27	0.97	1.65	7.7E-02	4.1E-01	1.05	0.61	1.8	8.7E-01	2.5E-01	1.88	0.93	3.84	8.0E-02	8.5E-01	1.04	0.5	2.15	9.2E-01	7.8E-01
384	CHMP1A	3	Weighted mode	1.22	0.67	2.22	3.0E-01	5.9E-01	1.35	0.73	2.49	1.7E-01	4.1E-01	0.88	0.27	2.83	6.8E-01	2.5E-01	1.82	0.29	11.43	3.0E-01	8.5E-01	0.98	0.18	5.38	9.7E-01	7.8E-01
384	CHMP1A	3	MR Egger	1.63	0	583.72	4.8E-01	5.9E-01	2.45	0	2204.53	3.4E-01	4.1E-01	0.11	0	40127.33	2.8E-01	2.5E-01	1.42	0	84505557.36	8.4E-01	8.5E-01	0.66	0	6343135681	8.5E-01	7.8E-01
385	TFF1	5	Inverse variance weighted	0.93	0.86	1.01	7.6E-02	8.1E-01	0.9	0.82	0.99	3.8E-02	5.5E-01	0.95	0.73	1.23	6.8E-01	6.7E-01	0.97	0.74	1.25	7.9E-01	2.0E-01	1.15	0.91	1.45	2.5E-01	9.0E-01
385	TFF1	5	Weighted median	0.93	0.85	1.02	1.3E-01	8.1E-01	0.92	0.83	1.01	9.1E-02	5.5E-01	0.9	0.75	1.09	2.9E-01	6.7E-01	0.9	0.69	1.17	4.4E-01	2.0E-01	1.12	0.86	1.45	4.0E-01	9.0E-01
385	TFF1	5	Weighted mode	0.93	0.82	1.06	2.2E-01	8.1E-01	0.92	0.79	1.05	1.6E-01	5.5E-01	0.9	0.68	1.18	3.3E-01	6.7E-01	0.9	0.62	1.31	4.7E-01	2.0E-01	1.1	0.76	1.59	5.2E-01	9.0E-01
385	TFF1	5	MR Egger	0.94	0.76	1.17	4.6E-01	8.1E-01	0.95	0.72	1.25	5.9E-01	5.5E-01	0.86	0.39	1.89	5.9E-01	6.7E-01	0.74	0.38	1.42	2.3E-01	2.0E-01	1.12	0.57	2.2	6.2E-01	9.0E-01
386	TSPAN15	1	Wald ratio	1.3	0.8	2.09	2.9E-01	NA	1.23	0.71	2.13	4.6E-01	NA	0.84	0.3	2.36	7.4E-01	NA	4.59	1.09	19.4	3.8E-02	NA	1.29	0.31	5.38	7.3E-01	NA
387	LPL	8	Inverse variance weighted	0.98	0.91	1.06	6.2E-01	7.1E-01	0.97	0.89	1.06	5.2E-01	6.7E-01	0.88	0.73	1.06	1.8E-01	7.8E-01	1.07	0.87	1.33	5.3E-01	7.2E-01	0.99	0.74	1.34	9.7E-01	1.8E-01
387	LPL	8	Weighted median	0.97	0.9	1.03	3.1E-01	7.1E-01	0.96	0.88	1.04	2.8E-01	6.7E-01	0.86	0.75	0.99	3.8E-02	7.8E-01	1.07	0.85	1.34	5.9E-01	7.2E-01	0.91	0.74	1.12	4.0E-01	1.8E-01
387	LPL	8	Weighted mode	0.97	0.89	1.05	3.3E-01	7.1E-01	0.96	0.86	1.06	3.6E-01	6.7E-01	0.86	0.71	1.04	9.6E-02	7.8E-01	1.06	0.81	1.39	6.1E-01	7.2E-01	0.88	0.69	1.14	2.9E-01	1.8E-01
387	LPL	8	MR Egger	0.97	0.87	1.09	5.8E-01	7.1E-01	0.96	0.85	1.09	4.8E-01	6.7E-01	0.89	0.68	1.17	3.5E-01	7.8E-01	1.05	0.78	1.41	6.9E-01	7.2E-01	0.9	0.62	1.31	5.3E-01	1.8E-01
388	LEG1	17	Inverse variance weighted	1	0.95	1.06	9.6E-01	2.2E-01	0.96	0.91	1.02	1.9E-01	1.7E-01	1.03	0.95	1.12	4.8E-01	2.2E-01	1.08	0.94	1.24	2.8E-01	3.4E-01	1.13	1.01	1.27	3.8E-02	9.9E-01
388	LEG1	17	Weighted median	1.02	0.97	1.07	5.1E-01	2.2E-01	0.97	0.92	1.03	3.0E-01	1.7E-01	1.05	0.93	1.19	3.9E-01	2.2E-01	1.08	0.93	1.27	3.2E-01	3.4E-01	1.12	0.98	1.3	1.1E-01	9.9E-01
388	LEG1	17	Weighted mode	1.01	0.97	1.06	5.9E-01	2.2E-01	0.98	0.93	1.03	3.5E-01	1.7E-01	1.03	0.91	1.17	5.9E-01	2.2E-01	1.1	0.94	1.28	2.1E-01	3.4E-01	1.14	0.99	1.3	5.9E-02	9.9E-01
388	LEG1	17	MR Egger	1.03	0.96	1.11	3.8E-01	2.2E-01	1	0.92	1.08	9.8E-01	1.7E-01	1.09	0.96	1.23	1.9E-01	2.2E-01	1.15	0.94	1.41	1.7E-01	3.4E-01	1.13	0.95	1.34	1.5E-01	9.9E-01
389	TNFRSF10A	4	Inverse variance weighted	1.06	1	1.12	3.8E-02	8.2E-01	1.06	1	1.13	6.6E-02	4.9E-01	1.04	0.92	1.17	5.4E-01	4.6E-01	1	0.8	1.26	9.7E-01	6.9E-01	1.17	1	1.38	5.6E-02	4.8E-01
389	TNFRSF10A	4	Weighted median	1.06	1	1.12	4.3E-02	8.2E-01	1.06	0.99	1.13	7.4E-02	4.9E-01	1.05	0.93	1.18	4.7E-01	4.6E-01	1.01	0.85	1.2	9.1E-01	6.9E-01	1.17	0.99	1.38	6.7E-02	4.8E-01
389	TNFRSF10A	4	Weighted mode	1.06	0.97	1.16	1.4E-01	8.2E-01	1.05	0.95	1.17	2.2E-01	4.9E-01	1.06	0.85	1.3	4.7E-01	4.6E-01	1.01	0.77	1.34	8.9E-01	6.9E-01	1.15	0.89	1.5	1.8E-01	4.8E-01
389	TNFRSF10A	4	MR Egger	1.05	0.82	1.33	5.0E-01	8.2E-01	1.01	0.77	1.34	8.7E-01	4.9E-01	1.14	0.68	1.91	3.9E-01	4.6E-01	1.12	0.36	3.5	7.2E-01	6.9E-01	1.04	0.51	2.12	8.5E-01	4.8E-01
390	CD5	6	Inverse variance weighted	1.08	0.93	1.25	3.2E-01	6.5E-01	1.06	0.9	1.24	5.1E-01	3.2E-01	1.38	1.02	1.87	3.8E-02	8.5E-01	1.1	0.6	2.02	7.5E-01	6.4E-02	0.92	0.6	1.39	6.9E-01	2.4E-01
390	CD5	6	Weighted median	1.09	0.91	1.29	3.5E-01	6.5E-01	1.05	0.87	1.28	6.1E-01	3.2E-01	1.42	0.99	2.04	5.9E-02	8.5E-01	0.96	0.58	1.59	8.7E-01	6.4E-02	1.05	0.62	1.78	8.5E-01	2.4E-01
390	CD5	6	Weighted mode	1.11	0.87	1.43	3.3E-01	6.5E-01	1.05	0.76	1.45	7.1E-01	3.2E-01	1.4	0.8	2.45	1.8E-01	8.5E-01	0.93	0.47	1.85	8.0E-01	6.4E-02	1.22	0.61	2.44	5.0E-01	2.4E-01
390	CD5	6	MR Egger	1.23	0.57	2.65	5.1E-01	6.5E-01	1.44	0.66	3.16	2.7E-01	3.2E-01	1.23	0.24	6.4	7.5E-01	8.5E-01	0.19	0.02	1.43	8.4E-02	6.4E-02	2.42	0.32	18.53	2.9E-01	2.4E-01
391	CSF2RA	2	Inverse variance weighted	1.33	0.98	1.8	6.8E-02	NA	1.54	1.02	2.31	3.9E-02	NA	0.71	0.37	1.39	3.2E-01	NA	1.66	0.33	8.44	5.4E-01	NA	1.61	0.63	4.13	3.2E-01	NA
392	RRM2B	3	Inverse variance weighted	0.95	0.71	1.26	7.0E-01	5.4E-01	1.01	0.74	1.37	9.5E-01	7.1E-01	0.9	0.61	1.32	5.9E-01	6.0E-01	0.92	0.47	1.83	8.2E-01	6.1E-01	0.6	0.24	1.46	2.6E-01	2.7E-01
392	RRM2B	3	Weighted median	0.88	0.73	1.07	2.0E-01	5.4E-01	0.95	0.76	1.19	6.8E-01	7.1E-01	0.86	0.57	1.31	4.9E-01	6.0E-01	0.79	0.44	1.44	4.5E-01	6.1E-01	0.52	0.28	0.97	3.9E-02	2.7E-01
392	RRM2B	3	Weighted mode	0.88	0.55	1.4	3.6E-01	5.4E-01	0.94	0.54	1.63	6.8E-01	7.1E-01	0.85	0.31	2.31	5.5E-01	6.0E-01	0.78	0.2	3.01	5.1E-01	6.1E-01	0.48	0.12	1.91	1.5E-01	2.7E-01
392	RRM2B	3	MR Egger	0.7	0.01	80.79	5.2E-01	5.4E-01	0.82	0	315.95	7.4E-01	7.1E-01	0.66	0	268.34	5.4E-01	6.0E-01	0.5	0	106159.37	6.0E-01	6.1E-01	0.16	0	782.45	2.2E-01	2.7E-01
393	TREML2	7	Inverse variance weighted	0.99	0.91	1.08	8.4E-01	5.2E-01	0.98	0.89	1.09	7.3E-01	2.3E-01	1.13	0.93	1.37	2.2E-01	5.3E-01	1.32	1.01	1.72	3.9E-02	4.1E-01	0.97	0.74	1.26	8.1E-01	4.5E-01
393	TREML2	7	Weighted median	0.98	0.89	1.08	6.5E-01	5.2E-01	0.95	0.85	1.07	4.1E-01	2.3E-01	1.12	0.92	1.38	2.7E-01	5.3E-01	1.27	0.95	1.69	1.1E-01	4.1E-01	0.91	0.68	1.22	5.4E-01	4.5E-01
393	TREML2	7	Weighted mode	0.98	0.86	1.11	6.6E-01	5.2E-01	0.95	0.82	1.1	4.5E-01	2.3E-01	1.12	0.87	1.44	3.2E-01	5.3E-01	1.26	0.87	1.84	1.8E-01	4.1E-01	0.89	0.61	1.3	4.9E-01	4.5E-01
393	TREML2	7	MR Egger	0.95	0.8	1.15	5.4E-01	5.2E-01	0.9	0.73	1.11	2.6E-01	2.3E-01	1.04	0.69	1.57	8.2E-01	5.3E-01	1.14	0.66	1.97	5.5E-01	4.1E-01	0.85	0.49	1.46	4.7E-01	4.5E-01
394	ENPP6	5	Inverse variance weighted	1	0.93	1.07	9.3E-01	2.9E-01	1.03	0.95	1.12	4.2E-01	4.2E-01	0.86	0.74	0.99	3.9E-02	5.0E-01	0.8	0.56	1.15	2.3E-01	6.2E-01	1.12	0.92	1.37	2.7E-01	9.1E-01
394	ENPP6	5	Weighted median	1.03	0.94	1.13	4.8E-01	2.9E-01	1.04	0.96	1.14	3.4E-01	4.2E-01	0.92	0.76	1.11	3.6E-01	5.0E-01	0.98	0.72	1.34	9.0E-01	6.2E-01	1.12	0.89	1.41	3.5E-01	9.1E-01
394	ENPP6	5	Weighted mode	1.06	0.92	1.23	3.1E-01	2.9E-01	1.05	0.93	1.19	3.5E-01	4.2E-01	0.9	0.68	1.19	3.4E-01	5.0E-01	1.06	0.66	1.68	7.6E-01	6.2E-01	1.12	0.81	1.53	3.9E-01	9.1E-01
394	ENPP6	5	MR Egger	1.05	0.88	1.25	4.3E-01	2.9E-01	1.08	0.89	1.32	3.0E-01	4.2E-01	0.92	0.63	1.34	5.3E-01	5.0E-01	0.92	0.33	2.6	8.2E-01	6.2E-01	1.14	0.69	1.88	4.8E-01	9.1E-01
395	COL2A1	9	Inverse variance weighted	1.09	0.95	1.26	2.3E-01	8.1E-01	1.08	0.91	1.3	3.8E-01	7.2E-01	1.04	0.8	1.37	7.6E-01	3.8E-01	1.49	1.02	2.18	3.9E-02	4.2E-01	0.97	0.56	1.68	9.1E-01	8.2E-01
395	COL2A1	9	Weighted median	1.06	0.89	1.25	5.3E-01	8.1E-01	1.12	0.91	1.37	2.8E-01	7.2E-01	1.04	0.72	1.52	8.3E-01	3.8E-01	1.37	0.81	2.32	2.4E-01	4.2E-01	0.83	0.48	1.42	4.9E-01	8.2E-01
395	COL2A1	9	Weighted mode	1.02	0.74	1.39	9.0E-01	8.1E-01	1.17	0.83	1.66	3.1E-01	7.2E-01	0.95	0.52	1.75	8.6E-01	3.8E-01	1.29	0.45	3.66	5.9E-01	4.2E-01	0.8	0.37	1.72	5.2E-01	8.2E-01
395	COL2A1	9	MR Egger	1.02	0.52	2.01	9.5E-01	8.1E-01	1.23	0.53	2.85	5.8E-01	7.2E-01	0.66	0.2	2.18	4.4E-01	3.8E-01	0.83	0.16	4.43	8.0E-01	4.2E-01	0.76	0.06	10.09	8.1E-01	8.2E-01
396	SCGN	5	Inverse variance weighted	0.93	0.82	1.05	2.5E-01	7.7E-01	1.05	0.91	1.21	5.2E-01	8.9E-01	0.71	0.48	1.04	8.1E-02	5.4E-01	0.87	0.51	1.47	6.0E-01	1.8E-01	0.74	0.51	1.08	1.1E-01	6.0E-01
396	SCGN	5	Weighted median	0.84	0.72	0.99	3.9E-02	7.7E-01	1.06	0.9	1.25	4.9E-01	8.9E-01	0.83	0.58	1.17	2.8E-01	5.4E-01	1.14	0.64	2.02	6.6E-01	1.8E-01	0.72	0.46	1.11	1.4E-01	6.0E-01
396	SCGN	5	Weighted mode	0.84	0.63	1.13	1.8E-01	7.7E-01	1.06	0.81	1.39	5.9E-01	8.9E-01	0.91	0.54	1.54	6.6E-01	5.4E-01	0.47	0.14	1.54	1.5E-01	1.8E-01	0.67	0.31	1.44	2.2E-01	6.0E-01
396	SCGN	5	MR Egger	0.86	0.38	1.93	5.9E-01	7.7E-01	1.09	0.45	2.68	7.7E-01	8.9E-01	1.21	0.09	15.41	8.3E-01	5.4E-01	0.21	0.02	3.02	1.6E-01	1.8E-01	0.49	0.05	5.03	4.0E-01	6.0E-01
397	LRRN1	5	Inverse variance weighted	0.98	0.95	1.02	4.3E-01	6.3E-01	0.97	0.93	1	7.7E-02	5.6E-01	0.96	0.88	1.05	4.1E-01	5.6E-01	1	0.9	1.1	9.8E-01	8.0E-01	1.11	1.01	1.22	3.9E-02	6.2E-01
397	LRRN1	5	Weighted median	0.99	0.95	1.02	4.1E-01	6.3E-01	0.97	0.93	1	8.7E-02	5.6E-01	0.97	0.9	1.04	3.5E-01	5.6E-01	1	0.9	1.11	9.7E-01	8.0E-01	1.11	1	1.22	4.7E-02	6.2E-01
397	LRRN1	5	Weighted mode	0.98	0.94	1.03	3.9E-01	6.3E-01	0.97	0.92	1.02	1.9E-01	5.6E-01	0.97	0.87	1.08	5.0E-01	5.6E-01	1	0.86	1.15	9.8E-01	8.0E-01	1.11	0.95	1.29	1.3E-01	6.2E-01

397	LRRN1	5	MR Egger	0.99	0.91	1.08	8.1E-01	6.3E-01	0.98	0.9	1.05	3.8E-01	5.6E-01	0.99	0.82	1.19	8.3E-01	5.6E-01	1.01	0.82	1.24	8.9E-01	8.0E-01	1.09	0.89	1.33	2.7E-01	6.2E-01
398	AARSD1	2	Inverse variance weighted	0.86	0.71	1.04	1.2E-01	NA	0.79	0.64	0.99	3.9E-02	NA	1.1	0.73	1.66	6.5E-01	NA	0.98	0.55	1.74	9.4E-01	NA	0.71	0.4	1.26	2.4E-01	NA
399	SMOC2	4	Inverse variance weighted	0.95	0.88	1.02	1.7E-01	3.2E-01	1	0.92	1.09	9.2E-01	3.0E-01	0.94	0.8	1.09	3.9E-01	3.2E-01	0.98	0.79	1.21	8.4E-01	5.7E-01	0.8	0.65	0.99	3.9E-02	6.0E-01
399	SMOC2	4	Weighted median	0.95	0.89	1.02	1.8E-01	3.2E-01	1.01	0.94	1.09	7.4E-01	3.0E-01	0.94	0.8	1.1	4.2E-01	3.2E-01	0.98	0.79	1.22	8.9E-01	5.7E-01	0.81	0.66	1	5.4E-02	6.0E-01
399	SMOC2	4	Weighted mode	0.96	0.86	1.08	3.8E-01	3.2E-01	1.02	0.89	1.16	6.9E-01	3.0E-01	0.97	0.74	1.27	7.7E-01	3.2E-01	1.01	0.71	1.44	9.4E-01	5.7E-01	0.83	0.59	1.16	1.7E-01	6.0E-01
399	SMOC2	4	MR Egger	1.01	0.78	1.31	8.9E-01	3.2E-01	1.08	0.8	1.47	3.6E-01	3.0E-01	1.07	0.61	1.88	6.4E-01	3.2E-01	1.08	0.49	2.38	7.1E-01	5.7E-01	0.88	0.41	1.89	5.5E-01	6.0E-01
400	LCAT	11	Inverse variance weighted	1.02	0.91	1.13	7.7E-01	9.1E-01	0.96	0.85	1.08	5.0E-01	6.1E-01	0.99	0.78	1.24	9.1E-01	5.5E-01	1.09	0.77	1.52	6.4E-01	3.7E-02	1.34	0.97	1.84	7.4E-02	3.3E-01
400	LCAT	11	Weighted median	1.04	0.91	1.19	5.8E-01	9.1E-01	0.97	0.83	1.13	7.1E-01	6.1E-01	1.05	0.77	1.43	7.6E-01	5.5E-01	1.29	0.83	2	2.5E-01	3.7E-02	1.3	0.86	1.98	2.1E-01	3.3E-01
400	LCAT	11	Weighted mode	1.04	0.86	1.25	6.7E-01	9.1E-01	0.97	0.77	1.22	7.4E-01	6.1E-01	0.97	0.62	1.51	8.7E-01	5.5E-01	1.38	0.77	2.48	2.5E-01	3.7E-02	1.25	0.67	2.32	4.5E-01	3.3E-01
400	LCAT	11	MR Egger	1	0.76	1.32	9.7E-01	9.1E-01	0.9	0.66	1.23	4.5E-01	6.1E-01	0.85	0.47	1.55	5.6E-01	5.5E-01	2.48	1.06	5.79	3.9E-02	3.7E-02	0.97	0.43	2.18	9.3E-01	3.3E-01
401	F9	2	Inverse variance weighted	0.91	0.84	1	3.9E-02	NA	0.91	0.83	1.01	7.9E-02	NA	0.89	0.73	1.07	2.1E-01	NA	0.81	0.62	1.05	1.1E-01	NA	0.9	0.7	1.17	4.4E-01	NA
402	IL17C	2	Inverse variance weighted	0.92	0.69	1.22	5.5E-01	NA	0.86	0.55	1.36	5.2E-01	NA	0.64	0.41	1.01	5.7E-02	NA	1.96	1.03	3.71	3.9E-02	NA	1.02	0.55	1.92	9.4E-01	NA
403	LILRA5	8	Inverse variance weighted	0.93	0.84	1.04	2.1E-01	7.3E-01	0.88	0.78	1	5.0E-02	4.8E-01	1.02	0.81	1.29	8.4E-01	1.0E+00	1.01	0.73	1.4	9.4E-01	1.8E-01	1.09	0.73	1.63	6.8E-01	7.2E-01
403	LILRA5	8	Weighted median	0.94	0.82	1.07	3.6E-01	7.3E-01	0.86	0.74	0.99	3.9E-02	4.8E-01	1.05	0.79	1.4	7.4E-01	1.0E+00	0.92	0.61	1.37	6.8E-01	1.8E-01	1	0.65	1.55	9.9E-01	7.2E-01
403	LILRA5	8	Weighted mode	0.93	0.78	1.11	3.7E-01	7.3E-01	0.85	0.71	1.03	8.6E-02	4.8E-01	1.06	0.74	1.53	7.2E-01	1.0E+00	0.84	0.5	1.43	4.7E-01	1.8E-01	1.32	0.74	2.36	3.0E-01	7.2E-01
403	LILRA5	8	MR Egger	0.9	0.7	1.18	3.8E-01	7.3E-01	0.82	0.6	1.1	1.5E-01	4.8E-01	1.02	0.58	1.81	9.2E-01	1.0E+00	0.66	0.3	1.47	2.5E-01	1.8E-01	1.25	0.44	3.57	6.2E-01	7.2E-01
404	SMAD3	3	Inverse variance weighted	1.02	0.91	1.14	7.8E-01	5.5E-01	1.06	0.85	1.32	6.0E-01	5.6E-01	0.8	0.52	1.23	3.1E-01	7.1E-01	0.86	0.61	1.22	4.1E-01	6.1E-01	1.43	1.02	2.01	3.9E-02	7.4E-01
404	SMAD3	3	Weighted median	0.99	0.88	1.12	9.3E-01	5.5E-01	1.02	0.89	1.17	8.0E-01	5.6E-01	0.77	0.59	1	4.8E-02	7.1E-01	0.82	0.58	1.18	2.9E-01	6.1E-01	1.39	0.97	2.01	7.5E-02	7.4E-01
404	SMAD3	3	Weighted mode	0.99	0.76	1.3	9.4E-01	5.5E-01	1.02	0.75	1.37	8.3E-01	5.6E-01	0.74	0.4	1.39	1.8E-01	7.1E-01	0.82	0.34	1.99	4.4E-01	6.1E-01	1.38	0.62	3.09	2.3E-01	7.4E-01
404	SMAD3	3	MR Egger	0.94	0.22	4.02	6.7E-01	5.5E-01	0.91	0.05	15.33	7.5E-01	5.6E-01	0.65	0	441.31	5.5E-01	7.1E-01	0.71	0.01	45.12	4.9E-01	6.1E-01	1.27	0.02	73.81	5.9E-01	7.4E-01
405	SORT1	13	Inverse variance weighted	0.96	0.81	1.12	5.9E-01	9.0E-01	1.01	0.82	1.24	9.4E-01	9.0E-01	0.94	0.79	1.12	5.1E-01	9.6E-01	0.87	0.65	1.15	3.2E-01	3.5E-01	0.91	0.65	1.28	6.0E-01	9.9E-02
405	SORT1	13	Weighted median	1.02	0.92	1.14	6.6E-01	9.0E-01	1.15	1.01	1.3	3.9E-02	9.0E-01	0.99	0.78	1.24	9.0E-01	9.6E-01	0.77	0.55	1.08	1.3E-01	3.5E-01	0.79	0.57	1.1	1.6E-01	9.9E-02
405	SORT1	13	Weighted mode	1.03	0.91	1.16	6.6E-01	9.0E-01	1.14	0.99	1.32	7.3E-02	9.0E-01	0.98	0.74	1.31	9.1E-01	9.6E-01	0.73	0.5	1.08	1.1E-01	3.5E-01	0.79	0.53	1.16	2.1E-01	9.9E-02
405	SORT1	13	MR Egger	0.94	0.64	1.38	7.2E-01	9.0E-01	1.04	0.63	1.71	8.8E-01	9.0E-01	0.93	0.63	1.4	7.2E-01	9.6E-01	0.67	0.35	1.29	2.1E-01	3.5E-01	0.54	0.26	1.12	9.1E-02	9.9E-02
406	ANXA2	3	Inverse variance weighted	1.03	0.98	1.09	2.5E-01	5.6E-01	1.02	0.95	1.09	5.9E-01	5.8E-01	1.14	1.01	1.28	3.9E-02	5.0E-01	1.1	0.92	1.3	2.9E-01	5.7E-01	1.03	0.87	1.22	7.1E-01	6.2E-01
406	ANXA2	3	Weighted median	1.03	0.97	1.09	3.2E-01	5.6E-01	1.01	0.95	1.08	6.6E-01	5.8E-01	1.13	1	1.28	4.9E-02	5.0E-01	1.1	0.93	1.31	2.8E-01	5.7E-01	1.04	0.88	1.24	6.4E-01	6.2E-01
406	ANXA2	3	Weighted mode	1.03	0.91	1.16	4.1E-01	5.6E-01	1.01	0.88	1.17	7.1E-01	5.8E-01	1.13	0.86	1.48	1.9E-01	5.0E-01	1.11	0.75	1.64	3.7E-01	5.7E-01	1.05	0.72	1.54	6.4E-01	6.2E-01
406	ANXA2	3	MR Egger	1	0.55	1.83	9.6E-01	5.6E-01	0.99	0.51	1.91	8.4E-01	5.8E-01	1.05	0.31	3.62	6.8E-01	5.0E-01	1.19	0.21	6.66	4.2E-01	5.7E-01	1.11	0.2	6.08	5.9E-01	6.2E-01
407	TGFBI	9	Inverse variance weighted	0.96	0.93	1	7.3E-02	9.1E-01	0.95	0.91	1	4.0E-02	8.1E-01	0.95	0.87	1.04	2.4E-01	2.4E-01	1	0.88	1.13	9.7E-01	6.5E-01	0.96	0.85	1.09	5.5E-01	6.4E-01
407	TGFBI	9	Weighted median	0.96	0.92	1	4.2E-02	9.1E-01	0.95	0.91	1	5.9E-02	8.1E-01	0.91	0.82	1.01	6.7E-02	2.4E-01	1	0.88	1.15	9.8E-01	6.5E-01	0.97	0.85	1.11	6.2E-01	6.4E-01
407	TGFBI	9	Weighted mode	0.96	0.91	1.01	1.1E-01	9.1E-01	0.95	0.9	1.01	8.2E-02	8.1E-01	0.92	0.82	1.04	1.4E-01	2.4E-01	1	0.86	1.18	9.5E-01	6.5E-01	0.96	0.83	1.11	5.7E-01	6.4E-01
407	TGFBI	9	MR Egger	0.96	0.9	1.03	2.2E-01	9.1E-01	0.96	0.88	1.04	2.4E-01	8.1E-01	0.9	0.77	1.04	1.2E-01	2.4E-01	1.03	0.83	1.27	7.7E-01	6.5E-01	0.99	0.81	1.22	9.4E-01	6.4E-01
408	PRL	3	Inverse variance weighted	0.87	0.67	1.13	2.9E-01	8.5E-01	0.99	0.75	1.31	9.3E-01	9.2E-01	1.01	0.58	1.76	9.7E-01	7.5E-01	1.06	0.5	2.21	8.9E-01	4.3E-01	0.46	0.22	0.97	4.0E-02	9.9E-01
408	PRL	3	Weighted median	0.81	0.62	1.06	1.3E-01	8.5E-01	0.98	0.71	1.35	8.8E-01	9.2E-01	0.82	0.44	1.52	5.2E-01	7.5E-01	1.08	0.46	2.52	8.6E-01	4.3E-01	0.48	0.22	1.06	7.0E-02	9.9E-01
408	PRL	3	Weighted mode	0.79	0.38	1.65	3.0E-01	8.5E-01	0.97	0.42	2.24	8.7E-01	9.2E-01	0.81	0.19	3.49	6.0E-01	7.5E-01	0.71	0.08	6.5	5.8E-01	4.3E-01	0.49	0.06	3.66	2.6E-01	9.9E-01
408	PRL	3	MR Egger	0.7	0	136134.04	7.7E-01	8.5E-01	1.11	0	103179.28	9.3E-01	9.2E-01	0.45	0	28054965119	7.5E-01	7.5E-01	0.09	0	9235368183	4.4E-01	4.3E-01	0.48	0	34583652171	7.7E-01	9.9E-01
409	TNN	5	Inverse variance weighted	0.97	0.76	1.23	8.0E-01	1.1E-01	0.87	0.7	1.09	2.3E-01	1.9E-01	1.12	0.64	1.97	6.9E-01	1.1E-01	1.17	0.7	1.97	5.5E-01	3.2E-01	1.7	1.02	2.81	4.0E-02	5.0E-01
409	TNN	5	Weighted median	0.9	0.74	1.09	2.7E-01	1.1E-01	0.83	0.66	1.03	9.6E-02	1.9E-01	0.94	0.62	1.43	7.8E-01	1.1E-01	1.17	0.65	2.09	6.0E-01	3.2E-01	1.54	0.88	2.69	1.3E-01	5.0E-01
409	TNN	5	Weighted mode	0.89	0.67	1.16	2.9E-01	1.1E-01	0.82	0.58	1.14	1.7E-01	1.9E-01	0.92	0.47	1.77	7.3E-01	1.1E-01	1.07	0.47	2.43	8.3E-01	3.2E-01	1.54	0.64	3.72	2.5E-01	5.0E-01
409	TNN	5	MR Egger	0.7	0.4	1.21	1.3E-01	1.1E-01	0.65	0.35	1.23	1.2E-01	1.9E-01	0.52	0.14	1.88	2.0E-01	1.1E-01	0.69	0.13	3.62	5.3E-01	3.2E-01	1.21	0.24	6.12	7.3E-01	5.0E-01
410	KIAA0319	1	Wald ratio	0.92	0.84	1.01	6.7E-02	NA	0.9	0.81	1	4.0E-02	NA	0.96	0.79	1.17	7.1E-01	NA	0.93	0.71	1.22	6.2E-01	NA	1.18	0.9	1.54	2.3E-01	NA
411	TTN	1	Wald ratio	1.06	0.64	1.77	8.1E-01	NA	1.21	0.67	2.18	5.2E-01	NA	0.48	0.16	1.45	1.9E-01	NA	1.44	0.31	6.57	6.4E-01	NA	4.74	1.07	20.97	4.0E-02	NA
412	ANGPTL1	5	Inverse variance weighted	1.08	0.99	1.18	6.6E-02	8.8E-01	1.09	0.99	1.2	7.7E-02	7.6E-01	1.04	0.87	1.25	6.8E-01	1.9E-01	1	0.67	1.48	1.0E+00	2.6E-01	1.27	0.99	1.63	6.1E-02	8.6E-01
412	ANGPTL1	5	Weighted median	1.09	1	1.18	5.4E-02	8.8E-01	1.11	1	1.22	4.1E-02	7.6E-01	1.02	0.84	1.23	8.7E-01	1.9E-01	1.05	0.79	1.39	7.6E-01	2.6E-01	1.29	0.99	1.68	5.7E-02	8.6E-01
412	ANGPTL1	5	Weighted mode	1.1	0.97	1.24	1.1E-01	8.8E-01	1.11	0.96	1.27	1.2E-01	7.6E-01	1.01	0.78	1.32	9.0E-01	1.9E-01	1.14	0.76	1.7	4.1E-01	2.6E-01	1.29	0.88	1.9	1.4E-01	8.6E-01
412	ANGPTL1	5	MR Egger	1.1	0.83	1.44	3.7E-01	8.8E-01	1.12	0.82	1.54	3.3E-01	7.6E-01	0.79	0.44	1.43	3.0E-01	1.9E-01	1.54	0.49	4.89	3.2E-01	2.6E-01	1.33	0.59	3	3.5E-01	8.6E-01
413	PIGR	5	Inverse variance weighted	1.03	0.86	1.23	7.8E-01	9.7E-01	0.97	0.84	1.13	7.3E-01	7.8E-01	1.35	1.01	1.8	4.1E-02	8.7E-01	1.07	0.69	1.67	7.5E-01	1.4E-01	1.39	0.77	2.54	2.8E-01	9.0E-01
413	PIGR	5	Weighted median	0.98	0.84	1.14	7.9E-01	9.7E-01	0.94	0.79	1.13	5.0E-01	7.8E-01	1.28	0.91	1.82	1.6E-01	8.7E-01	1.35	0.82	2.24	2.4E-01	1.4E-01	1.39	0.85	2.28	1.8E-01	9.0E-01
413	PIGR	5	Weighted mode	0.98	0.78	1.25	8.7E-01	9.7E-01	0.93	0.69	1.26	5.6E-01	7.8E-01	1.27	0.75	2.14	2.8E-01	8.7E-01	1.31	0.59	2.94	4.0E-01	1.4E-01	1.27	0.57	2.85	4.6E-01	9.0E-01
413	PIGR	5	MR Egger	1.02	0.58	1.81	9.2E-01	9.7E-01	0.94	0.62	1.43	6.8E-01	7.8E-01	1.3	0.58	2.92	3.7E-01	8.7E-01	1.92	0.61	6.03	1.7E-01	1.4E-01	1.31	0.19	8.95	6.9E-01	9.0E-01
414	FCAR	9	Inverse variance weighted	0.96	0.93	1	4.4E-02	7.2E-01	0.96	0.92	1	6.9E-02	3.8E-01	0.93	0.86	1.01	6.9E-02	1.2E-01	0.99	0.89	1.11	8.8E-01	7.5E-01	1.04	0.93	1.16	4.9E-01	8.3E-01
414	FCAR	9	Weighted median	0.96	0.93	1	5.7E-02	7.2E-01	0.96	0.92	1	4.1E-02	3.8E-01	0.94	0.86	1.03	1.7E-01	1.2E-01	0.98	0.87	1.11	7.8E-01	7.5E-01	1.04	0.92	1.16	5.6E-01	8.3E-01
414	FCAR	9	Weighted mode	0.96	0.92	1	7.4E-02	7.2E-01	0.96	0.91	1.01	1.3E-01	3.8E-01	0.94	0.85	1.05	2.3E-01	1.2E-01	0.98	0.85	1.13	7.4E-01	7.5E-01	1.03	0.91	1.18	5.8E-01	8.3E-01
414	FCAR	9	MR Egger	0.96	0.91	1.01	1.0E-01	7.2E-01	0.95	0.89	1.01	8.1E-02	3.8E-01	0.97	0.87	1.09	6.0E-01	1.2E-01	0.98	0.83	1.15	7.7E-01	7.5E-01	1.05	0.9	1.22	5.0E-01	8.3E-01
415	MARCO	8	Inverse variance weighted	1.03	0.96	1.11	4.5E-01	3.3E-01	1	0.92	1.09	9.8E-01	4.7E-01	1.05	0.9	1.23	5.4E-01	2.2E-01	1.26	1.01	1.57	4.1E-02	4.7E-01	0.93	0.75	1.15	5.0E-01	2.2E-01
415	MARCO	8	Weighted median	1	0.92	1.09	9.9E-01	3.3E-01	0.96	0.87	1.07	4.6E-01	4.7E-01	1.04	0.85	1.27	7.0E-01	2.2E-01	1.24	0.95	1.62	1.2E-01	4.7E-01	0.97	0.74	1.25	7.9E-01	2.2E-01
415	MARCO	8	Weighted mode	1	0.89	1.11	9.4E-01	3.3E-01	0.96	0.85	1.1	5.3E-01	4.7E-01	1.02	0.79	1.31	8.9E-01	2.2E-01	1.25	0.87	1.77	1.9E-01	4.7E-01	0.96	0.67	1.36	7.7E-01	2.2E-01
415	MARCO	8	MR Egger	0.98	0.85	1.13	7.7E-01	3.3E-01	0.96	0.82	1.13	5.7E-01	4.7E-01	0.92	0.68	1.25	5.5E-01	2.2E-01	1.14	0.75	1.73	4.7E-01	4.7E-01	1.1	0.73	1.66	5.8E-01	2.2E-01
416	SPAG1	1	Wald ratio	0.96	0.93	1	4.1E-02	NA	0.99	0.95	1.03	5.1E-01	NA	0.93	0.86	1.01	6.9E-02	NA	0.95	0.85	1.06	3.7E-01	NA	0.89	0.8	1	4.4E-02	NA
417	AIF1L	2	Inverse variance weighted	1.08	0.98	1.18	1.1E-01	NA	1.11	1	1.24	4.1E-02	NA	0.99	0.81	1.2	9.2E-01	NA	0.95	0.72	1.24	6.9E-01	NA	0.82	0.63	1.08	1.5E-01	NA
418	DDR1	9	Inverse variance weighted	1.06	0.94	1.2	3.1E-01	9.6E-01	0.97	0.85	1.11	6.9E-01	8.8E-01	1.08	0.79	1.47	6.5E-01	9.7E-01	1.24	0.87	1.77	2.3E-01	7.4E-01	1.43	1.01	2	4.1E-02	3.6E-01
418	DDR1	9	Weighted median	1.03	0.87	1.2	7.6E-01	9.6E-01	0.94	0.81	1.11	4.7E-01	8.8E-01	0.86	0.6	1.23	4.2E-01	9.7E-01	1.09	0.67	1.78	7.2E-01	7.4E-01	1.41	0.91	2.18	1.2E-01	3.6E-01
418	DDR1	9	Weighted mode	0.96	0.77	1.21	7.1E-01	9.6E-01	0.95	0.77	1.17	5.7E-01	8.8E-01	0.83	0.41	1.68	5.6E-01	9.7E-01	0.87	0.35	2.2	7.5E-01	7.4E-01	1.35	0.77	2.35	2.5E-01	3.6E-01
418	DDR1	9	MR Egger	1.07	0.77	1.49	6.4E-01	9.6E-01	0.99	0.7	1.4	9.6E-01	8.8E-01	1.06	0.44	2.55	8.7E-01	9.7E-01	1.4	0.55	3.6	4.2E-01	7.4E-01	1.04	0.44	2.45	9.1E-01	3.6E-01
419	NPY	12	Inverse variance weighted	0.95	0.87	1.03	2.1E-01	8.6E-01	0.95	0.86	1.05	3.2E-01	7.3E-01	0.94	0.78	1.13	5.0E-01	7.5E-01	0.93	0.72	1.21	6.0E-01	6.2E-01	0.76	0.59	0.99	4.1E-02	6.2E-01
419	NPY	12	Weighted median	0.9	0.8	1	6.0E-02	8.6E-01	0.89	0.78	1.02	9.0E-02	7.3E-01	0.92	0.72	1.19	5.3E-01	7.5E-01	0.97	0.7	1.35	8.6E-01	6.2E-01	0.74	0.52	1.05	8.9E-02	6.2E-01
419	NPY	12	Weighted mode	0.9	0.78	1.02	9.8E-02	8.6E-01	0.89	0.76	1.06	1.6E-01	7.3E-01	0.9	0.65	1.23	4.7E-01	7.5E-01	1.13	0.75	1.7	5.4E-01	6.2E-01	0.71	0.45	1.12	1.3E-01	6.2E-01
419	NPY	12	MR Egger	0.96	0.77	1.19	7.0E-01	8.6E-01	0.99	0.77	1.26	9.0E-01	7.3E-01	1	0.63	1.59	9.9E-01	7.5E-01	1.07	0.55	2.07	8.3E-01	6.2E-01	0.67	0.35	1.28	2.0E-01	6.2E-01
420	SOD1	2	Inverse variance weighted	1.1	0.87	1.39	4.2E-01	NA	1.32	1.01	1.73	4.1E-02	NA	0.85	0.49	1.49	5.7E-01	NA	0.57	0.28	1.16	1.2E-01	NA	0.79	0.38	1.65	5.3E-01	NA
421	PLTP	11	Inverse variance weighted	1.01	0.95	1.07	8.0E-01	8.1E-01	0.98	0.92	1.04	5.1E-01	7.9E-01	1.02	0.87	1.2	7.7E-01	5.8E-01	1.2	1.01	1.42	4.1E-02	8.8E-01	1.06	0.9	1.26	4.7E-01	1.1E-01
421	PLTP	11	Weighted median	0.99	0.93	1.06	8.7E-01	8.1E-01	0.97	0.91	1.04	4.3E-01	7.9E-01	1.03	0.9	1.18	6.5E-01	5.8E-01	1.19	0.98	1.44	7.5E-02	8.8E-01	0.96	0.8	1.17	7.1E-01	1.1E-01
421	PLTP	11	Weighted mode	0.99	0.93	1.06	8.6E-01	8.1E-01	0.98	0.91	1.06	5.3E-01	7.9E-01	1.03	0.89	1.2	6.5E-01	5.8E-01	1.2	0.97	1.47	7.9E-02	8.8E-01	0.98	0.79	1.22	8.4E-01	1.1E-01
421	PLTP	11	MR Egger	1	0.9	1.11	1.0E+00	8.1E-01	0.99	0.89	1.09	7.9E-01	7.9E-01	1.07	0.83	1.39	5.7E-01	5.8E-01	1.21	0.92	1.59	1.5E-01	8.8E-01	0.92	0.71	1.2	5.0E-01	1.1E-01
422	MINK1	3	Inverse variance weighted	1.3	1	1.71	5.4E-02	5.2E-01	1.38	1.01	1.89	4.1E-02	9.6E-01	1.08	0.56	2.11	8.1E-01	5.8E-01	0.84	0.37	1.9	6.8E-01	4.5E-01	0.79	0.35	1.77	5.7E-01	5.1E-01
422	MINK1	3	Weighted median	1.36	0.97	1.91	7.6E-02	5.2E-01	1.42	1	2.02	5.1E-02	9.6E-01	1.3	0.61	2.79	5.0E-01	5.8E-01	0.69	0.26	1.84	4.6E-01	4.5E-01	0.86	0.33	2.27	7.6E-01	5.1E-01
422	MINK1	3	Weighted mode	1.43	0.59	3.5	2.2E-01	5.2E-01	1.46	0.56	3.79	2.3E-01	9.6E-01	1.51	0.17	13.11	5.0E-01	5.8E-01	0.62	0.04	8.97	5.2E-01	4.5E-01	0.97	0.07	14.07	9.6E-01	5.1E-01
422	MINK1	3	MR Egger	0.15	0	1.23727E+12	5.7E-01	5.2E-01	1.61	0	1.41343E+15	8.9E-01	9.6E-01	0.01	0	2.23448E+33	5.8E-01	5.8E-01	0	0	2.18158E+35	4.5E-01	4.5E-01	0	0	3.52755E+35	5.0E-01	5.1E-01
423	CD99L2	10	Inverse variance weighted	1.02	0.93	1.12	6.5E-01	2.3E-01	1.02	0.91	1.14	7.6E-01	5.7E-01	1.03	0.73	1.44	8.8E-01	2.7E-01	0.95	0.65	1.37	7.8E-01	2.2E-02	1	0.75	1.33	9.8E-01	7.0E-01
423	CD99L2	10	Weighted median	0.99	0.88	1.12	9.1E-01	2.3E-01	1	0.86	1.16	9.9E-01	5.7E-01	0.93	0.7	1.24	6.3E-01	2.7E-01	0.8	0.52	1.21	2.8E-01	2.2E-02	1.11	0.78	1.6	5.6E-01	7.0E-01
423	CD99L2	10	Weighted mode	0.98	0.83	1.15	7.6E-01	2.3E-01	0.99	0.83	1.19	9.4E-01	5.7E-01	0.89	0.59	1.36	5.6E-01	2.7E-01	0.72	0.38	1.34	2.6E-01	2.2E-02	1.13	0.73	1.74	5.5E-01	7.0E-01
423	CD99L2	10	MR Egger	0.95	0.79	1.13	4.9E-01	2.3E-01	0.98	0.8	1.2	8.0E-01	5.7E-01	0.8	0.43	1.49	4.4E-01	2.7E-01	0.56	0.32	0.97	4.1E-02	2.2E-02	0.94	0.55	1.58	7.8E-01	7.0E-01
424	SCN4B	2	Inverse variance weighted	1.2	0.8	1.81	3.8E-01	NA	1.64	1.02	2.63	4.1E-02	NA	0.93	0.38	2.27	8.8E-01	NA	0.45	0.09	2.34	3.4E-01	NA	1.1	0.32	3.76	8.8E-01	NA
425	F13B	3	Inverse variance weighted	0.94	0.89	1	4.1E-02	9.0E-01	0.95	0.89	1.01	1.0E-01	8.8E-01	0.88	0.78	1	4.1E-02	1.0E+00	0.92	0.76	1.1	3.5E-01	9.0E-01	1.01	0.85	1.2	8.9E-01	7.5E-01
425	F13B	3	Weighted median	0.95	0.89	1	5.5E-02	9.0E-01	0.95	0.89	1.01	1.2E-01	8.8E-01	0.88	0.78	1	4.5E-02	1.0E+00	0.92	0.78	1.1	3.6E-01	9.0E-01	1	0.85	1.18	9.9E-01	7.5E-01
425	F13B	3	Weighted mode	0.94	0.82	1.08	2.1E-01	9.0E-01	0.95	0.82	1.1	2.7E-01	8.8E-01	0.88	0.66	1.18	2.1E-01	1.0E+00	0.92	0.63	1.34	4.3E-01	9.0E-01	1	0.68	1.49	9.8E-01	7.5E-01
425	F13B	3	MR Egger	0.95	0.52	1.73	4.7E-01	9.0E-01	0.96	0.48	1.91	5.6E-01	8.8E-01	0.88	0.24	3.22	4.4E-01	1.0E+00	0.94	0.06	15.06	8.3E-01	9.0E-01	0.95	0.08	11.07	8.3E-01	7.5E-01
426	SELL	10	Inverse variance weighted	1.05	0.99	1.12	1.2E-01	3.4E-01	1.08	1	1.16	4.1E-02	2.8E-01	0.96	0.84	1.1	5.5E-01	9.1E-01	1.17	0.97	1.42	9.5E-02	5.5E-01	0.96	0.8	1.15	6.5E-01	6.1E-01
426	SELL	10	Weighted median	1.04	0.97	1.12	2.2E-01	3.4E-01	1.07	0.98	1.16	1.1E-01	2.8E-01	0.97	0.84	1.12	6.4E-01	9.1E-01	1.17	0.96	1.43	1.3E-01	5.5E-01	0.91	0.75	1.12	3.7E-01	6.1E-01
426	SELL	10	Weighted mode	1.05	0.97	1.13	2.1E-01	3.4E-01	1.07	0.98	1.16	1.3E-01	2.8E-01	0.98	0.83	1.15	7.5E-01	9.1E-01	1.16	0.93	1.46	1.7E-01	5.5E-01	0.92	0.73	1.16	4.3E-01	6.1E-01
426	SELL	10	MR Egger	1.02	0.92	1.13	6.6E-01	3.4E-01	1.04	0.92	1.16	5.0E-01	2.8E-01	0.97	0.78	1.2	7.3E-01	9.1E-01	1.11	0.82	1.5	4.5E-01	5.5E-01	0.91	0.68	1.23	5.0E-01	6.1E-01
427	GASK1A	1	Wald ratio	1.02	0.98	1.06	3.0E-01	NA	1.02	0.98	1.06	4.1E-01	NA	1.04	0.96	1.12	3.7E-01	NA	1.11	1	1.25	5.7E-02	NA	1.12	1	1.25	4.1E-02	NA
428	MMP12	3	Inverse variance weighted	1.01	0.96	1.06	7.8E-01	9.4E-01	1.01	0.95	1.07	8.6E-01	8.5E-01	0.95	0.85	1.06	3.2E-01	5.0E-01	1.2	0.96	1.5	1.1E-01	7.4E-01	0.95	0.82	1.1	4.8E-01	5.4E-01
428	MMP12	3	Weighted median	1.01	0.96	1.06	7.8E-01	9.4E-01	1.01	0.95	1.07	8.5E-01	8.5E-01	0.94	0.84	1.05	2.5E-01	5.0E-01	1.19	1.01	1.4	4.1E-02	7.4E-01	0.95	0.81	1.1	4.9E-01	5.4E-01
428	MMP12	3	Weighted mode	1.01	0.9	1.13	7.9E-01	9.4E-01	1.01	0.89	1.14	8.1E-01	8.5E-01	0.94	0.74	1.19	3.5E-01	5.0E-01	1.19	0.83	1.7	1.7E-01	7.4E-01	0.96	0.68	1.35	6.5E-01	5.4E-01
428	MMP12	3	MR Egger	1.01	0.62	1.64	8.4E-01	9.4E-01	1.01	0.63	1.63	8.2E-01	8.5E-01	0.91	0.37	2.21	4.0E-01	5.0E-01	1.15	0.11	11.92	6.0E-01	7.4E-01	1	0.29	3.4	9.8E-01	5.4E-01
429	MYDGF	3	Inverse variance weighted	0.99	0.81	1.21	9.0E-01	6.6E-01	1.02	0.77	1.34	9.1E-01	9.0E-01	0.82	0.53	1.27	3.8E-01	7.1E-01	0.54	0.3	0.98	4.1E-02	8.3E-01	1.03	0.41	2.56	9.5E-01	2.9E-01
429	MYDGF	3	Weighted median	1.03	0.82	1.3	7.8E-01	6.6E-01	0.98	0.74	1.28	8.7E-01	9.0E-01	0.89	0.55	1.43	6.3E-01	7.1E-01	0.55	0.29	1.07	7.7E-02	8.3E-01	0.83	0.41	1.69	6.1E-01	2.9E-01
429	MYDGF	3	Weighted mode	1.04	0.57	1.89	8.0E-01	6.6E-01	0.94	0.46	1.9	7.4E-01	9.0E-01	0.91	0.25	3.33	7.9E-01	7.1E-01	0.5	0.1	2.64	2.2E-01	8.3E-01	2.01	0.3	13.54	2.5E-01	2.9E-01
429	MYDGF	3	MR Egger	1.08	0.1	11.31	7.5E-01	6.6E-01	0.97	0.01	88.69	9.5E-01	9.0E-01	0.97	0.01	155.55	9.4E-01	7.1E-01	0.48	0	414.17	4.0E-01	8.3E-01	2.84	0	5156.78	3.3E-01	2.9E-01
430	RNASET2	5	Inverse variance weighted	1.01	0.91	1.11	8.7E-01	6.7E-01	0.99	0.89	1.09	7.9E-01	6.1E-01	0.93	0.81	1.06	2.9E-01	8.0E-01	1.21	1.01	1.45	4.2E-02	3.3E-01	1.09	0.82	1.44	5.5E-01	6.3E-01
430	RNASET2	5	Weighted median	1.01	0.95	1.08	7.5E-01	6.7E-01	0.99	0.92	1.06	7.3E-01	6.1E-01	0.93	0.81	1.07	3.3E-01	8.0E-01	1.22	1	1.49	4.5E-02	3.3E-01	1.16	0.96	1.41	1.2E-01	6.3E-01
430	RNASET2	5	Weighted mode	1.01	0.93	1.1	7.0E-01	6.7E-01	0.99	0.89	1.1	7.7E-01	6.1E-01	0.93	0.77	1.13	3.6E-01	8.0E-01	1.24	0.93	1.64	1.1E-01	3.3E-01	1.18	0.89	1.57	1.8E-01	6.3E-01
430	RNASET2	5	MR Egger	1.04	0.78	1.4	6.7E-01	6.7E-01	1.03	0.76	1.39	7.8E-01	6.1E-01	0.95	0.67	1.37	7.1E-01	8.0E-01	1.39	0.85	2.27	1.2E-01	3.3E-01	1.22	0.52	2.88	5.1E-01	6.3E-01
431	DKK4	4	Inverse variance weighted	1.08	0.98	1.18	1.2E-01	7.6E-01	1.12	1	1.25	4.2E-02	4.8E-01	1.12	0.92	1.37	2.7E-01	8.7E-01	0.95	0.7	1.27	7.1E-01	3.7E-01	0.88	0.56	1.38	5.7E-01	2.3E-01
431	DKK4	4	Weighted median	1.07	0.97	1.18	1.5E-01	7.6E-01	1.11	0.98	1.24	9.4E-02	4.8E-01	1.09	0.87	1.37	4.4E-01	8.7E-01	0.95	0.7	1.3	7.6E-01	3.7E-01	0.97	0.72	1.32	8.6E-01	2.3E-01
431	DKK4	4	Weighted mode	1.07	0.91	1.27	2.6E-01	7.6E-01	1.1	0.9	1.36	2.3E-01	4.8E-01	1.04	0.71	1.53	7.5E-01	8.7E-01	1.08	0.62	1.89	7.0E-01	3.7E-01	1.03	0.6	1.76	8.6E-01	2.3E-01
431	DKK4	4	MR Egger	1.06	0.81	1.4	4.4E-01	7.6E-01	1.07	0.78	1.47	4.4E-01	4.8E-01	1.1	0.62	1.97	5.5E-01	8.7E-01	1.09	0.48	2.5	6.9E-01	3.7E-01	1.14	0.41	3.18	6.3E-01	2.3E-01
432	TNFRSF10C	13	Inverse variance weighted	0.97	0.93	1	6.0E-02	8.7E-01	0.98	0.93	1.02	2.6E-01	5.0E-01	0.94	0.88	1.01	1.1E-01	5.3E-01	0.95	0.83	1.08	4.5E-01	4.5E-01	0.95	0.84	1.08	4.5E-01	5.7E-01
432	TNFRSF10C	13	Weighted median	0.97	0.93	1	4.2E-02	8.7E-01	0.97	0.93	1.01	1.7E-01	5.0E-01	0.95	0.88	1.01	1.2E-01	5.3E-01	0.96	0.87	1.06	4.3E-01	4.5E-01	0.95	0.86	1.05	3.3E-01	5.7E-01
432	TNFRSF10C	13	Weighted mode	0.96	0.93	1	5.3E-02	8.7E-01	0.97	0.93	1.02	1.9E-01	5.0E-01	0.95	0.87	1.03	1.7E-01	5.3E-01	0.96	0.86	1.08	4.8E-01	4.5E-01	0.95	0.85	1.06	3.1E-01	5.7E-01
432	TNFRSF10C	13	MR Egger	0.97	0.92	1.01	1.5E-01	8.7E-01	0.97	0.92	1.02	2.2E-01	5.0E-01	0.95	0.87	1.05	2.9E-01	5.3E-01	0.98	0.83	1.15	7.5E-01	4.5E-01	0.97	0.82	1.14	6.9E-01	5.7E-01
433	RSPO3	7	Inverse variance weighted	1.13	0.99	1.28	7.2E-02	4.6E-01	1.11	0.93	1.31	2.5E-01	6.4E-01	1.09	0.82	1.44	5.5E-01	7.6E-02	1.34	0.82	2.21	2.5E-01	1.1E-01	1.3	0.85	1.98	2.3E-01	9.6E-01
433	RSPO3	7	Weighted median	1.17	1.01	1.35	4.2E-02	4.6E-01	1.08	0.91	1.29	3.8E-01	6.4E-01	1.15	0.82	1.61	4.1E-01	7.6E-02	1.44	0.9	2.29	1.2E-01	1.1E-01	1.27	0.81	1.99	3.0E-01	9.6E-01
433	RSPO3	7	Weighted mode	1.18	0.96	1.44	9.8E-02	4.6E-01	1.05	0.84	1.33	6.0E-01	6.4E-01	1.46	0.94	2.28	8.2E-02	7.6E-02	1.96	1.02	3.76	4.5E-02	1.1E-01	1.22	0.67	2.24	4.4E-01	9.6E-01
433	RSPO3	7	MR Egger	1.27	0.83	1.96	2.1E-01	4.6E-01	1	0.55	1.79	9.9E-01	6.4E-01	2.15	0.91	5.09	7.1E-02	7.6E-02	3.3	0.88	12.46	6.9E-02	1.1E-01	1.33	0.3	5.86	6.4E-01	9.6E-01
434	SBSN	4	Inverse variance weighted	0.82	0.63	1.06	1.2E-01	2.4E-01	1.02	0.76	1.37	9.0E-01	3.2E-01	0.86	0.49	1.49	5.8E-01	6.2E-01	0.78	0.36	1.68	5.2E-01	8.6E-01	0.51	0.16	1.62	2.6E-01	4.6E-01
434	SBSN	4	Weighted median	0.73	0.54	0.99	4.3E-02	2.4E-01	1.01	0.72	1.42	9.6E-01	3.2E-01	0.91	0.49	1.69	7.7E-01	6.2E-01	0.79	0.33	1.9	6.0E-01	8.6E-01	0.77	0.29	2.02	5.9E-01	4.6E-01
434	SBSN	4	Weighted mode	0.72	0.34	1.55	2.7E-01	2.4E-01	0.94	0.41	2.18	8.3E-01	3.2E-01	1.05	0.27	4.09	9.2E-01	6.2E-01	0.8	0.14	4.7	7.2E-01	8.6E-01	0.95	0.12	7.3	9.4E-01	4.6E-01
434	SBSN	4	MR Egger	0.6	0.22	1.61	1.6E-01	2.4E-01	0.77	0.25	2.38	4.2E-01	3.2E-01	0.68	0.08	5.67	5.1E-01	6.2E-01	0.69	0.04	13.44	6.5E-01	8.6E-01	0.23	0	21.67	3.0E-01	4.6E-01
435	GRPEL1	3	Inverse variance weighted	0.82	0.66	1.03	9.2E-02	4.2E-01	0.9	0.69	1.16	4.2E-01	9.7E-01	0.64	0.37	1.09	1.0E-01	3.7E-01	0.6	0.3	1.18	1.4E-01	6.5E-01	0.95	0.3	2.95	9.3E-01	3.0E-01
435	GRPEL1	3	Weighted median	0.79	0.61	1.02	7.4E-02	4.2E-01	0.9	0.68	1.2	4.8E-01	9.7E-01	0.55	0.31	0.98	4.3E-02	3.7E-01	0.63	0.28	1.39	2.5E-01	6.5E-01	0.65	0.29	1.46	3.0E-01	3.0E-01
435	GRPEL1	3	Weighted mode	0.76	0.38	1.54	2.4E-01	4.2E-01	0.91	0.45	1.84	6.2E-01	9.7E-01	0.52	0.11	2.45	2.1E-01	3.7E-01	0.65	0.08	5.02	4.6E-01	6.5E-01	0.62	0.1	3.95	3.9E-01	3.0E-01
435	GRPEL1	3	MR Egger	0.5	0	86.3	3.4E-01	4.2E-01	0.92	0	352.85	8.8E-01	9.7E-01	0.17	0	13900.9	3.0E-01	3.7E-01	1.24	0	8354479.26	8.9E-01	6.5E-01	0.08	0	1988762.5	3.1E-01	3.0E-01
436	RALB	1	Wald ratio	0.94	0.73	1.19	5.9E-01	NA	1.03	0.78	1.37	8.2E-01	NA	0.63	0.37	1.07	9.0E-02	NA	2.11	1.02	4.36	4.3E-02	NA	0.64	0.31	1.32	2.2E-01	NA
437	CR1	13	Inverse variance weighted	1.03	0.97	1.09	3.7E-01	9.5E-01	1.01	0.94	1.09	7.2E-01	7.4E-01	1.07	0.94	1.22	3.2E-01	5.7E-01	1.16	0.98	1.36	7.7E-02	5.3E-01	1	0.83	1.21	1.0E+00	6.5E-01
437	CR1	13	Weighted median	1.03	0.97	1.1	3.0E-01	9.5E-01	1.02	0.95	1.09	5.9E-01	7.4E-01	1.08	0.95	1.23	2.2E-01	5.7E-01	1.2	1.01	1.42	4.3E-02	5.3E-01	1	0.84	1.2	9.9E-01	6.5E-01
437	CR1	13	Weighted mode	1.03	0.96	1.1	3.8E-01	9.5E-01	1.02	0.95	1.1	5.7E-01	7.4E-01	1.08	0.94	1.25	2.6E-01	5.7E-01	1.18	0.97	1.42	8.5E-02	5.3E-01	0.99	0.82	1.21	9.6E-01	6.5E-01
437	CR1	13	MR Egger	1.03	0.94	1.13	4.9E-01	9.5E-01	1.02	0.92	1.14	6.4E-01	7.4E-01	1.11	0.91	1.35	2.9E-01	5.7E-01	1.21	0.95	1.53	1.1E-01	5.3E-01	1.04	0.79	1.37	7.8E-01	6.5E-01
438	CD164	8	Inverse variance weighted	1.01	0.9	1.13	8.6E-01	5.9E-01	1.07	0.94	1.22	3.3E-01	2.9E-01	0.81	0.63	1.03	9.2E-02	5.6E-01	1.14	0.76	1.69	5.3E-01	7.6E-01	1.42	1.01	1.99	4.3E-02	9.7E-01
438	CD164	8	Weighted median	1	0.87	1.16	9.7E-01	5.9E-01	1.02	0.86	1.2	8.1E-01	2.9E-01	0.85	0.62	1.17	3.2E-01	5.6E-01	0.94	0.6	1.46	7.7E-01	7.6E-01	1.38	0.89	2.13	1.5E-01	9.7E-01
438	CD164	8	Weighted mode	1	0.81	1.23	1.0E+00	5.9E-01	1.02	0.82	1.28	8.1E-01	2.9E-01	0.89	0.57	1.37	5.4E-01	5.6E-01	0.91	0.48	1.7	7.2E-01	7.6E-01	1.31	0.69	2.49	3.5E-01	9.7E-01
438	CD164	8	MR Egger	0.93	0.62	1.38	6.6E-01	5.9E-01	0.87	0.55	1.38	4.8E-01	2.9E-01	0.99	0.42	2.35	9.9E-01	5.6E-01	0.94	0.21	4.22	9.3E-01	7.6E-01	1.45	0.41	5.13	5.0E-01	9.7E-01
439	KLK12	4	Inverse variance weighted	0.98	0.96	1	1.0E-01	7.1E-01	0.97	0.95	1	4.3E-02	8.5E-01	1	0.95	1.05	1.0E+00	7.1E-01	0.99	0.93	1.06	8.6E-01	5.2E-01	0.99	0.92	1.06	7.4E-01	9.6E-01
439	KLK12	4	Weighted median	0.98	0.96	1	8.5E-02	7.1E-01	0.97	0.95	1	4.6E-02	8.5E-01	1	0.95	1.05	9.6E-01	7.1E-01	1	0.93	1.07	9.0E-01	5.2E-01	0.99	0.92	1.05	7.1E-01	9.6E-01
439	KLK12	4	Weighted mode	0.98	0.95	1.02	1.8E-01	7.1E-01	0.97	0.94	1.01	1.2E-01	8.5E-01	1	0.92	1.09	9.9E-01	7.1E-01	1	0.9	1.11	8.9E-01	5.2E-01	0.99	0.89	1.1	7.5E-01	9.6E-01
439	KLK12	4	MR Egger	0.98	0.9	1.06	3.2E-01	7.1E-01	0.97	0.89	1.06	2.9E-01	8.5E-01	0.99	0.81	1.2	7.9E-01	7.1E-01	1.02	0.83	1.27	7.0E-01	5.2E-01	0.99	0.8	1.22	8.1E-01	9.6E-01
440	TXN	1	Wald ratio	1.03	0.63	1.68	9.0E-01	NA	1.32	0.72	2.44	3.7E-01	NA	0.68	0.26	1.75	4.2E-01	NA	0.64	0.18	2.35	5.1E-01	NA	0.3	0.1	0.96	4.3E-02	NA
441	RARRES2	3	Inverse variance weighted	0.88	0.77	1	5.0E-02	8.6E-01	0.86	0.74	1	4.3E-02	5.5E-01	0.98	0.74	1.29	8.7E-01	9.9E-01	0.69	0.47	1.02	6.6E-02	7.0E-01	0.99	0.67	1.45	9.5E-01	8.9E-01
441	RARRES2	3	Weighted median	0.88	0.77	1.01	6.8E-02	8.6E-01	0.86	0.74	1	4.9E-02	5.5E-01	0.97	0.72	1.3	8.5E-01	9.9E-01	0.68	0.46	1	4.8E-02	7.0E-01	0.95	0.64	1.39	7.8E-01	8.9E-01
441	RARRES2	3	Weighted mode	0.89	0.65	1.21	2.4E-01	8.6E-01	0.88	0.63	1.24	2.6E-01	5.5E-01	0.97	0.49	1.91	8.5E-01	9.9E-01	0.67	0.26	1.75	2.2E-01	7.0E-01	0.92	0.36	2.39	7.5E-01	8.9E-01
441	RARRES2	3	MR Egger	0.9	0.18	4.39	5.5E-01	8.6E-01	0.95	0.15	5.95	8.0E-01	5.5E-01	0.98	0.03	30.07	9.6E-01	9.9E-01	0.59	0	72.51	3.9E-01	7.0E-01	0.92	0	312.12	8.9E-01	8.9E-01
442	CR2	19	Inverse variance weighted	1.03	0.94	1.12	5.4E-01	3.9E-01	1.02	0.94	1.11	6.8E-01	8.0E-01	1.04	0.88	1.23	6.5E-01	9.3E-01	1.21	0.97	1.51	8.6E-02	3.8E-01	1.21	0.96	1.52	1.0E-01	3.2E-01
442	CR2	19	Weighted median	1.06	0.95	1.18	2.9E-01	3.9E-01	0.98	0.87	1.12	8.0E-01	8.0E-01	0.97	0.78	1.21	8.0E-01	9.3E-01	1.37	1.01	1.85	4.4E-02	3.8E-01	1.3	0.96	1.76	8.7E-02	3.2E-01
442	CR2	19	Weighted mode	1.06	0.93	1.22	3.6E-01	3.9E-01	1.02	0.87	1.2	8.3E-01	8.0E-01	0.96	0.73	1.26	7.7E-01	9.3E-01	1.35	0.95	1.92	9.1E-02	3.8E-01	1.36	0.94	1.97	9.3E-02	3.2E-01
442	CR2	19	MR Egger	1.08	0.93	1.26	3.0E-01	3.9E-01	1	0.86	1.17	9.7E-01	8.0E-01	1.03	0.75	1.41	8.5E-01	9.3E-01	1.39	0.93	2.06	9.9E-02	3.8E-01	1.42	0.94	2.15	9.0E-02	3.2E-01
443	DNAJB1	5	Inverse variance weighted	1.07	0.83	1.37	6.2E-01	9.0E-01	1.03	0.81	1.31	8.0E-01	7.7E-01	1.47	1.01	2.14	4.4E-02	3.4E-01	1.06	0.63	1.78	8.4E-01	6.3E-01	1.37	0.71	2.61	3.5E-01	5.6E-01
443	DNAJB1	5	Weighted median	0.99	0.77	1.28	9.5E-01	9.0E-01	1	0.76	1.32	9.8E-01	7.7E-01	1.56	0.99	2.46	5.4E-02	3.4E-01	0.87	0.44	1.7	6.8E-01	6.3E-01	1.04	0.51	2.11	9.2E-01	5.6E-01
443	DNAJB1	5	Weighted mode	0.89	0.47	1.71	6.5E-01	9.0E-01	0.91	0.49	1.71	7.0E-01	7.7E-01	1.92	0.74	4.95	1.3E-01	3.4E-01	0.72	0.16	3.22	5.7E-01	6.3E-01	0.74	0.1	5.67	7.0E-01	5.6E-01
443	DNAJB1	5	MR Egger	1.18	0.1	14.42	8.4E-01	9.0E-01	1.3	0.12	13.68	7.5E-01	7.7E-01	0.47	0.02	11.9	5.1E-01	3.4E-01	2.23	0.02	202.76	6.1E-01	6.3E-01	4.58	0.01	1880.39	4.8E-01	5.6E-01
444	MME	12	Inverse variance weighted	1	0.95	1.05	9.7E-01	1.0E-01	1.02	0.96	1.07	5.6E-01	2.6E-01	1.05	0.93	1.17	4.3E-01	6.7E-01	0.87	0.75	1.02	8.9E-02	9.9E-01	1.1	0.96	1.27	1.7E-01	8.4E-02
444	MME	12	Weighted median	1.01	0.96	1.07	5.8E-01	1.0E-01	1.02	0.97	1.08	4.1E-01	2.6E-01	1.07	0.96	1.19	2.1E-01	6.7E-01	0.87	0.75	1.01	6.2E-02	9.9E-01	1.14	0.99	1.31	7.9E-02	8.4E-02
444	MME	12	Weighted mode	1.02	0.96	1.07	5.6E-01	1.0E-01	1.03	0.96	1.09	3.8E-01	2.6E-01	1.06	0.94	1.19	2.9E-01	6.7E-01	0.86	0.73	1.02	7.3E-02	9.9E-01	1.15	0.98	1.35	8.8E-02	8.4E-02
444	MME	12	MR Egger	1.04	0.97	1.11	2.9E-01	1.0E-01	1.04	0.96	1.13	2.5E-01	2.6E-01	1.07	0.9	1.28	4.1E-01	6.7E-01	0.87	0.68	1.11	2.4E-01	9.9E-01	1.24	1.01	1.52	4.4E-02	8.4E-02
445	GLRX5	3	Inverse variance weighted	0.99	0.79	1.23	9.0E-01	4.6E-01	0.88	0.69	1.13	3.1E-01	3.9E-01	1.55	1.01	2.38	4.4E-02	4.3E-01	1.08	0.62	1.89	7.7E-01	8.8E-01	0.97	0.56	1.68	9.1E-01	9.2E-01
445	GLRX5	3	Weighted median	0.93	0.76	1.14	4.8E-01	4.6E-01	0.84	0.67	1.06	1.4E-01	3.9E-01	1.43	0.93	2.21	1.1E-01	4.3E-01	1.04	0.56	1.96	8.9E-01	8.8E-01	0.95	0.53	1.7	8.5E-01	9.2E-01
445	GLRX5	3	Weighted mode	0.92	0.57	1.48	5.2E-01	4.6E-01	0.81	0.45	1.47	2.7E-01	3.9E-01	1.34	0.5	3.59	3.3E-01	4.3E-01	1.01	0.22	4.66	9.7E-01	8.8E-01	0.94	0.22	4.06	8.6E-01	9.2E-01
445	GLRX5	3	MR Egger	0.77	0.04	16.46	4.8E-01	4.6E-01	0.64	0.03	15.14	3.2E-01	3.9E-01	0.92	0	332.89	8.8E-01	4.3E-01	0.97	0	3926.05	9.7E-01	8.8E-01	0.9	0	3175.09	9.0E-01	9.2E-01
446	GRAP2	3	Inverse variance weighted	1	0.77	1.31	9.7E-01	5.6E-01	1.16	0.81	1.67	4.2E-01	7.3E-01	0.95	0.53	1.7	8.6E-01	4.3E-01	0.43	0.19	0.98	4.4E-02	5.9E-01	0.52	0.23	1.15	1.1E-01	8.9E-01
446	GRAP2	3	Weighted median	1.08	0.79	1.48	6.2E-01	5.6E-01	1.27	0.87	1.87	2.2E-01	7.3E-01	0.93	0.46	1.86	8.3E-01	4.3E-01	0.54	0.2	1.44	2.2E-01	5.9E-01	0.53	0.21	1.31	1.7E-01	8.9E-01
446	GRAP2	3	Weighted mode	1.1	0.46	2.66	6.8E-01	5.6E-01	1.35	0.48	3.75	3.4E-01	7.3E-01	0.83	0.12	5.85	7.2E-01	4.3E-01	0.54	0.04	7.37	4.2E-01	5.9E-01	0.55	0.05	5.9	3.9E-01	8.9E-01
446	GRAP2	3	MR Egger	1.21	0.05	32.08	6.0E-01	5.6E-01	1.38	0	446.29	6.1E-01	7.3E-01	1.7	0	1678.35	5.1E-01	4.3E-01	0.24	0	14061.17	3.5E-01	5.9E-01	0.58	0	14207.22	6.2E-01	8.9E-01
447	PVR	3	Inverse variance weighted	1	0.97	1.04	8.6E-01	7.8E-01	1	0.96	1.05	8.9E-01	6.6E-01	0.99	0.91	1.07	7.5E-01	8.9E-01	0.94	0.78	1.13	5.0E-01	1.0E+00	1.12	1	1.26	4.9E-02	5.2E-01
447	PVR	3	Weighted median	1	0.97	1.04	8.9E-01	7.8E-01	1	0.96	1.05	8.9E-01	6.6E-01	0.99	0.91	1.08	7.6E-01	8.9E-01	0.93	0.83	1.04	2.2E-01	1.0E+00	1.12	1	1.26	4.4E-02	5.2E-01
447	PVR	3	Weighted mode	1	0.93	1.09	8.7E-01	7.8E-01	1	0.92	1.09	9.7E-01	6.6E-01	0.99	0.83	1.17	7.8E-01	8.9E-01	0.93	0.72	1.21	3.5E-01	1.0E+00	1.12	0.87	1.44	2.0E-01	5.2E-01
447	PVR	3	MR Egger	1.02	0.6	1.72	7.7E-01	7.8E-01	1.02	0.6	1.75	6.7E-01	6.6E-01	1	0.37	2.73	9.9E-01	8.9E-01	0.94	0.04	22.55	8.4E-01	1.0E+00	1.03	0.25	4.18	8.3E-01	5.2E-01
448	PNLIP	16	Inverse variance weighted	1.03	0.93	1.14	5.9E-01	1.2E-01	1	0.88	1.15	9.7E-01	3.3E-01	1.05	0.87	1.26	6.2E-01	2.4E-01	1.14	0.9	1.45	2.8E-01	8.2E-01	1	0.82	1.21	9.6E-01	1.8E-02

448	PNLIP	16	Weighted median	1.01	0.93	1.1	7.8E-01	1.2E-01	0.98	0.9	1.07	6.7E-01	3.3E-01	0.96	0.8	1.15	6.5E-01	2.4E-01	1	0.77	1.28	9.8E-01	8.2E-01	0.98	0.75	1.29	9.0E-01	1.8E-02
448	PNLIP	16	Weighted mode	1	0.91	1.1	1.0E+00	1.2E-01	0.99	0.89	1.1	8.0E-01	3.3E-01	0.95	0.78	1.16	6.0E-01	2.4E-01	1.03	0.76	1.4	8.5E-01	8.2E-01	0.76	0.55	1.06	9.8E-02	1.8E-02
448	PNLIP	16	MR Egger	0.92	0.77	1.1	3.4E-01	1.2E-01	0.91	0.72	1.17	4.4E-01	3.3E-01	0.9	0.64	1.26	5.1E-01	2.4E-01	1.1	0.7	1.72	6.6E-01	8.2E-01	0.71	0.5	0.99	4.4E-02	1.8E-02
449	COX5B	3	Inverse variance weighted	0.75	0.56	0.99	4.5E-02	7.5E-01	0.81	0.58	1.12	2.1E-01	5.7E-01	0.96	0.51	1.81	9.0E-01	9.8E-01	0.38	0.09	1.6	1.9E-01	3.7E-01	0.9	0.38	2.11	8.1E-01	7.9E-01
449	COX5B	3	Weighted median	0.83	0.58	1.18	2.9E-01	7.5E-01	0.85	0.58	1.25	4.0E-01	5.7E-01	0.97	0.48	1.97	9.4E-01	9.8E-01	0.42	0.14	1.23	1.1E-01	3.7E-01	0.83	0.31	2.23	7.2E-01	7.9E-01
449	COX5B	3	Weighted mode	0.87	0.31	2.43	6.2E-01	7.5E-01	0.93	0.32	2.7	7.9E-01	5.7E-01	1.01	0.16	6.28	9.9E-01	9.8E-01	0.43	0.02	8.82	3.5E-01	3.7E-01	0.82	0.07	10.07	7.6E-01	7.9E-01
449	COX5B	3	MR Egger	0.7	0.03	14.89	3.8E-01	7.5E-01	0.72	0.04	12.26	3.8E-01	5.7E-01	0.95	0	245.43	9.2E-01	9.8E-01	0.8	0	11203.5	8.2E-01	3.7E-01	0.79	0	1303.93	7.5E-01	7.9E-01
450	C1S	2	Inverse variance weighted	0.83	0.69	1	4.5E-02	NA	0.87	0.7	1.07	1.9E-01	NA	0.71	0.48	1.05	8.4E-02	NA	0.69	0.4	1.19	1.8E-01	NA	0.84	0.45	1.58	5.9E-01	NA
451	MDK	11	Inverse variance weighted	1.14	1	1.3	4.6E-02	9.3E-01	1.12	0.96	1.3	1.5E-01	8.4E-01	1.23	0.93	1.64	1.5E-01	7.9E-01	1.49	0.99	2.23	5.7E-02	9.8E-01	1.18	0.79	1.75	4.1E-01	2.6E-01
451	MDK	11	Weighted median	1.16	0.98	1.37	7.8E-02	9.3E-01	1.13	0.93	1.37	2.1E-01	8.4E-01	1.14	0.77	1.69	5.2E-01	7.9E-01	1.74	1.01	2.99	4.5E-02	9.8E-01	1.13	0.66	1.94	6.6E-01	2.6E-01
451	MDK	11	Weighted mode	1.16	0.89	1.5	2.4E-01	9.3E-01	1.17	0.86	1.6	2.7E-01	8.4E-01	1.16	0.59	2.29	6.3E-01	7.9E-01	1.83	0.79	4.22	1.4E-01	9.8E-01	1.37	0.52	3.58	4.9E-01	2.6E-01
451	MDK	11	MR Egger	1.17	0.63	2.16	5.8E-01	9.3E-01	1.05	0.52	2.12	8.8E-01	8.4E-01	1.05	0.25	4.31	9.4E-01	7.9E-01	1.52	0.18	13.08	6.7E-01	9.8E-01	3.23	0.46	22.5	2.1E-01	2.6E-01
452	VAMP8	1	Wald ratio	1.17	0.99	1.38	6.8E-02	NA	1.08	0.89	1.31	4.1E-01	NA	1.45	1.01	2.09	4.5E-02	NA	1.28	0.77	2.13	3.4E-01	NA	1.56	0.95	2.58	8.1E-02	NA
453	NBL1	3	Inverse variance weighted	1.08	0.88	1.32	4.8E-01	8.0E-01	1.25	0.99	1.59	6.4E-02	9.2E-01	0.97	0.62	1.51	8.9E-01	6.9E-01	0.82	0.44	1.51	5.2E-01	8.8E-01	0.7	0.38	1.28	2.4E-01	5.7E-01
453	NBL1	3	Weighted median	1.06	0.85	1.34	5.9E-01	8.0E-01	1.31	1.01	1.71	4.5E-02	9.2E-01	0.99	0.59	1.64	9.6E-01	6.9E-01	0.82	0.43	1.6	5.7E-01	8.8E-01	0.61	0.32	1.18	1.4E-01	5.7E-01
453	NBL1	3	Weighted mode	1.06	0.59	1.91	7.2E-01	8.0E-01	1.33	0.69	2.57	2.0E-01	9.2E-01	0.88	0.28	2.76	6.7E-01	6.9E-01	0.77	0.16	3.71	5.6E-01	8.8E-01	0.61	0.14	2.75	2.9E-01	5.7E-01
453	NBL1	3	MR Egger	0.98	0.02	49.67	9.6E-01	8.0E-01	1.31	0.01	182.17	6.1E-01	9.2E-01	0.7	0	3306.4	6.8E-01	6.9E-01	0.69	0	89881.17	7.5E-01	8.8E-01	0.35	0	39949.25	4.6E-01	5.7E-01
454	PDIA5	4	Inverse variance weighted	1.08	0.91	1.28	3.7E-01	9.4E-01	1.11	0.96	1.3	1.6E-01	9.7E-01	0.96	0.73	1.27	7.8E-01	5.5E-01	1.14	0.8	1.63	4.6E-01	7.4E-01	1.09	0.72	1.63	6.9E-01	6.3E-01
454	PDIA5	4	Weighted median	1.08	0.99	1.18	8.6E-02	9.4E-01	1.11	1	1.23	4.5E-02	9.7E-01	1	0.82	1.21	9.7E-01	5.5E-01	1.1	0.85	1.43	4.7E-01	7.4E-01	1.04	0.79	1.37	7.7E-01	6.3E-01
454	PDIA5	4	Weighted mode	1.08	0.94	1.25	1.7E-01	9.4E-01	1.11	0.93	1.33	1.5E-01	9.7E-01	1	0.74	1.36	9.9E-01	5.5E-01	1.1	0.69	1.73	5.7E-01	7.4E-01	1.17	0.69	1.98	4.3E-01	6.3E-01
454	PDIA5	4	MR Egger	1.1	0.45	2.66	7.0E-01	9.4E-01	1.12	0.51	2.46	5.9E-01	9.7E-01	1.15	0.32	4.14	6.8E-01	5.5E-01	1	0.17	6.01	1.0E+00	7.4E-01	0.87	0.12	6.17	7.9E-01	6.3E-01
455	ATP6V1G2	1	Wald ratio	1.01	0.85	1.19	9.4E-01	NA	1.16	0.96	1.4	1.3E-01	NA	0.69	0.48	0.99	4.5E-02	NA	0.67	0.4	1.12	1.2E-01	NA	1.34	0.81	2.2	2.5E-01	NA
456	EBAG9	4	Inverse variance weighted	0.91	0.75	1.12	3.8E-01	9.4E-01	0.95	0.7	1.29	7.4E-01	6.0E-01	0.84	0.55	1.3	4.4E-01	6.6E-01	0.73	0.4	1.33	3.0E-01	6.1E-01	0.55	0.3	0.99	4.5E-02	9.7E-01
456	EBAG9	4	Weighted median	0.89	0.7	1.13	3.3E-01	9.4E-01	0.93	0.71	1.23	6.2E-01	6.0E-01	0.86	0.52	1.43	5.7E-01	6.6E-01	0.7	0.35	1.37	2.9E-01	6.1E-01	0.58	0.3	1.13	1.1E-01	9.7E-01
456	EBAG9	4	Weighted mode	0.87	0.57	1.34	3.9E-01	9.4E-01	0.91	0.47	1.77	6.8E-01	6.0E-01	0.87	0.33	2.3	6.7E-01	6.6E-01	0.67	0.16	2.76	4.4E-01	6.1E-01	0.63	0.16	2.52	3.6E-01	9.7E-01
456	EBAG9	4	MR Egger	0.86	0.05	14.88	8.5E-01	9.4E-01	1.9	0.01	248.66	6.3E-01	6.0E-01	0.41	0	192.17	6.6E-01	6.6E-01	0.22	0	1159.5	5.3E-01	6.1E-01	0.59	0	2821.23	8.1E-01	9.7E-01
457	MTHFSD	3	Inverse variance weighted	1.06	1	1.12	6.6E-02	3.8E-01	1.06	0.96	1.18	2.5E-01	2.7E-01	1.01	0.89	1.14	8.9E-01	9.6E-01	1.09	0.92	1.29	3.4E-01	9.9E-01	0.98	0.82	1.18	8.5E-01	3.7E-01
457	MTHFSD	3	Weighted median	1.06	1	1.12	5.7E-02	3.8E-01	1.07	1	1.14	4.6E-02	2.7E-01	1.01	0.89	1.14	8.9E-01	9.6E-01	1.08	0.91	1.29	3.5E-01	9.9E-01	0.99	0.83	1.18	9.0E-01	3.7E-01
457	MTHFSD	3	Weighted mode	1.06	0.95	1.2	1.5E-01	3.8E-01	1.07	0.92	1.24	1.9E-01	2.7E-01	1.01	0.78	1.31	8.7E-01	9.6E-01	1.09	0.74	1.6	4.3E-01	9.9E-01	1	0.68	1.47	9.7E-01	3.7E-01
457	MTHFSD	3	MR Egger	1.1	0.66	1.85	2.5E-01	3.8E-01	1.14	0.63	2.07	2.2E-01	2.7E-01	1.01	0.33	3.09	9.1E-01	9.6E-01	1.08	0.14	8.2	7.0E-01	9.9E-01	1.12	0.24	5.2	5.2E-01	3.7E-01
458	INSL3	2	Inverse variance weighted	0.63	0.4	0.99	4.6E-02	NA	0.61	0.36	1.03	6.6E-02	NA	0.7	0.21	2.35	5.7E-01	NA	0.33	0.08	1.3	1.1E-01	NA	0.39	0.1	1.51	1.7E-01	NA
459	CRELD2	9	Inverse variance weighted	0.96	0.88	1.05	3.6E-01	5.6E-01	0.96	0.88	1.05	3.7E-01	3.7E-01	0.93	0.78	1.09	3.7E-01	6.6E-01	0.88	0.68	1.13	3.0E-01	5.8E-01	0.82	0.66	1.01	6.1E-02	4.4E-01
459	CRELD2	9	Weighted median	0.94	0.87	1.02	1.6E-01	5.6E-01	0.94	0.85	1.03	1.8E-01	3.7E-01	0.96	0.8	1.15	6.3E-01	6.6E-01	0.92	0.7	1.2	5.3E-01	5.8E-01	0.78	0.61	1	4.6E-02	4.4E-01
459	CRELD2	9	Weighted mode	0.94	0.85	1.03	1.7E-01	5.6E-01	0.93	0.83	1.05	2.0E-01	3.7E-01	0.95	0.78	1.17	6.0E-01	6.6E-01	0.9	0.65	1.24	4.7E-01	5.8E-01	0.77	0.58	1.01	5.9E-02	4.4E-01
459	CRELD2	9	MR Egger	0.94	0.81	1.08	3.2E-01	5.6E-01	0.92	0.8	1.07	2.3E-01	3.7E-01	0.96	0.72	1.28	7.5E-01	6.6E-01	0.82	0.54	1.25	3.0E-01	5.8E-01	0.76	0.54	1.06	9.3E-02	4.4E-01
460	RARRES1	1	Wald ratio	0.94	0.89	1	4.6E-02	NA	0.96	0.9	1.02	1.8E-01	NA	0.99	0.88	1.12	9.0E-01	NA	0.9	0.76	1.07	2.2E-01	NA	0.85	0.72	1	5.4E-02	NA
461	CST1	8	Inverse variance weighted	1.03	0.98	1.09	2.0E-01	4.7E-01	1.04	0.99	1.1	1.2E-01	9.1E-02	0.96	0.85	1.07	4.4E-01	3.3E-01	0.97	0.8	1.17	7.3E-01	8.5E-01	1.05	0.88	1.27	5.7E-01	9.0E-01
461	CST1	8	Weighted median	1.04	0.99	1.1	9.5E-02	4.7E-01	1.06	1	1.12	4.6E-02	9.1E-02	0.94	0.84	1.04	2.5E-01	3.3E-01	0.96	0.83	1.12	6.4E-01	8.5E-01	1.06	0.91	1.23	4.8E-01	9.0E-01
461	CST1	8	Weighted mode	1.04	0.98	1.11	1.4E-01	4.7E-01	1.06	0.99	1.14	9.0E-02	9.1E-02	0.94	0.82	1.07	2.8E-01	3.3E-01	0.97	0.8	1.17	7.0E-01	8.5E-01	1.05	0.88	1.25	5.5E-01	9.0E-01
461	CST1	8	MR Egger	1.05	0.97	1.15	2.0E-01	4.7E-01	1.1	1	1.2	4.8E-02	9.1E-02	0.91	0.75	1.09	2.5E-01	3.3E-01	0.95	0.68	1.33	7.2E-01	8.5E-01	1.04	0.76	1.44	7.6E-01	9.0E-01
462	TPR	1	Wald ratio	0.62	0.39	0.99	4.6E-02	NA	0.64	0.37	1.1	1.1E-01	NA	0.57	0.21	1.6	2.9E-01	NA	0.62	0.15	2.59	5.2E-01	NA	0.55	0.14	2.18	3.9E-01	NA
463	PIK3IP1	2	Inverse variance weighted	0.96	0.87	1.05	3.3E-01	NA	0.99	0.89	1.1	8.3E-01	NA	0.9	0.74	1.09	3.0E-01	NA	0.89	0.68	1.16	3.9E-01	NA	0.76	0.59	1	4.6E-02	NA
464	FOXO3	1	Wald ratio	0.99	0.62	1.6	9.8E-01	NA	0.74	0.43	1.28	2.8E-01	NA	1.7	0.61	4.76	3.1E-01	NA	1.65	0.39	6.96	4.9E-01	NA	4.23	1.02	17.49	4.6E-02	NA
465	MERTK	14	Inverse variance weighted	0.96	0.88	1.05	3.8E-01	5.8E-01	0.92	0.83	1.02	1.3E-01	6.3E-01	1.15	0.97	1.37	1.1E-01	4.2E-01	1.01	0.81	1.27	9.1E-01	8.3E-01	0.98	0.78	1.22	8.3E-01	6.1E-01
465	MERTK	14	Weighted median	0.92	0.84	1.02	1.1E-01	5.8E-01	0.9	0.81	1	4.6E-02	6.3E-01	1.11	0.91	1.36	3.2E-01	4.2E-01	1.01	0.76	1.34	9.6E-01	8.3E-01	0.98	0.74	1.3	9.0E-01	6.1E-01
465	MERTK	14	Weighted mode	0.92	0.83	1.03	1.3E-01	5.8E-01	0.9	0.79	1.02	9.2E-02	6.3E-01	1.1	0.87	1.37	3.9E-01	4.2E-01	1.02	0.71	1.46	9.1E-01	8.3E-01	0.98	0.72	1.35	9.2E-01	6.1E-01
465	MERTK	14	MR Egger	0.92	0.77	1.11	3.6E-01	5.8E-01	0.89	0.72	1.1	2.4E-01	6.3E-01	1.03	0.72	1.47	8.6E-01	4.2E-01	1.05	0.66	1.69	8.1E-01	8.3E-01	1.07	0.68	1.67	7.6E-01	6.1E-01
466	CFI	3	Inverse variance weighted	1.1	0.98	1.23	1.0E-01	5.6E-01	1.09	0.93	1.29	2.9E-01	6.9E-01	1.14	0.9	1.46	2.7E-01	6.8E-01	0.9	0.57	1.42	6.5E-01	4.9E-01	0.74	0.53	1.03	7.8E-02	4.1E-01
466	CFI	3	Weighted median	1.12	1	1.26	5.0E-02	5.6E-01	1.11	0.96	1.28	1.5E-01	6.9E-01	1.17	0.91	1.5	2.3E-01	6.8E-01	0.96	0.68	1.37	8.4E-01	4.9E-01	0.71	0.51	0.99	4.7E-02	4.1E-01
466	CFI	3	Weighted mode	1.13	0.85	1.51	2.0E-01	5.6E-01	1.15	0.82	1.61	2.2E-01	6.9E-01	1.19	0.66	2.15	3.3E-01	6.8E-01	0.96	0.44	2.12	8.6E-01	4.9E-01	0.7	0.32	1.54	1.9E-01	4.1E-01
466	CFI	3	MR Egger	1.19	0.27	5.34	3.8E-01	5.6E-01	1.21	0.08	17.65	5.4E-01	6.9E-01	1.29	0.06	29.04	4.9E-01	6.8E-01	1.35	0	440.29	6.3E-01	4.9E-01	0.5	0.01	37.34	2.9E-01	4.1E-01
467	PCBD1	5	Inverse variance weighted	0.88	0.77	1	4.7E-02	6.1E-01	0.87	0.75	1.01	6.9E-02	6.3E-01	0.88	0.67	1.16	3.5E-01	5.5E-01	0.98	0.67	1.44	9.1E-01	4.0E-01	0.94	0.64	1.37	7.5E-01	9.4E-01
467	PCBD1	5	Weighted median	0.88	0.76	1.01	7.5E-02	6.1E-01	0.88	0.75	1.04	1.3E-01	6.3E-01	0.84	0.63	1.14	2.6E-01	5.5E-01	0.97	0.63	1.51	9.0E-01	4.0E-01	0.91	0.6	1.39	6.6E-01	9.4E-01
467	PCBD1	5	Weighted mode	0.88	0.7	1.09	1.7E-01	6.1E-01	0.88	0.68	1.15	2.6E-01	6.3E-01	0.83	0.51	1.34	3.4E-01	5.5E-01	1.02	0.5	2.05	9.5E-01	4.0E-01	0.91	0.48	1.7	6.9E-01	9.4E-01
467	PCBD1	5	MR Egger	0.95	0.59	1.53	7.5E-01	6.1E-01	0.95	0.54	1.68	8.0E-01	6.3E-01	0.72	0.26	1.99	3.9E-01	5.5E-01	1.45	0.35	5.95	4.6E-01	4.0E-01	0.91	0.23	3.66	8.5E-01	9.4E-01
468	TIMP1	5	Inverse variance weighted	1.09	0.86	1.37	4.7E-01	9.9E-01	1.19	0.95	1.5	1.4E-01	8.6E-01	0.96	0.7	1.31	8.1E-01	4.3E-01	0.9	0.5	1.63	7.3E-01	6.9E-01	0.92	0.46	1.84	8.1E-01	3.9E-01
468	TIMP1	5	Weighted median	1.07	0.88	1.29	5.1E-01	9.9E-01	1.25	1	1.55	4.7E-02	8.6E-01	0.95	0.64	1.39	7.9E-01	4.3E-01	0.85	0.47	1.54	5.9E-01	6.9E-01	0.87	0.47	1.58	6.4E-01	3.9E-01
468	TIMP1	5	Weighted mode	1.1	0.8	1.51	4.7E-01	9.9E-01	1.23	0.88	1.72	1.6E-01	8.6E-01	0.81	0.38	1.74	4.8E-01	4.3E-01	0.84	0.3	2.34	6.7E-01	6.9E-01	0.66	0.17	2.62	4.9E-01	3.9E-01
468	TIMP1	5	MR Egger	1.1	0.13	9.55	9.0E-01	9.9E-01	1.05	0.12	8.98	9.5E-01	8.6E-01	0.47	0.04	5.97	4.1E-01	4.3E-01	1.88	0.01	408.81	7.3E-01	6.9E-01	5.31	0.02	1480.66	4.1E-01	3.9E-01
469	SSC5D	7	Inverse variance weighted	1.03	0.98	1.08	2.7E-01	9.3E-01	1.01	0.96	1.07	6.5E-01	8.2E-01	1.11	1	1.24	4.7E-02	7.1E-01	0.99	0.79	1.23	8.9E-01	6.9E-01	0.99	0.85	1.15	9.0E-01	8.7E-01
469	SSC5D	7	Weighted median	1.02	0.97	1.08	4.5E-01	9.3E-01	1.01	0.95	1.07	7.7E-01	8.2E-01	1.12	1	1.25	5.9E-02	7.1E-01	0.97	0.82	1.14	7.0E-01	6.9E-01	1.02	0.86	1.2	8.4E-01	8.7E-01
469	SSC5D	7	Weighted mode	1.02	0.95	1.1	5.5E-01	9.3E-01	1.01	0.94	1.08	7.4E-01	8.2E-01	1.12	0.96	1.3	1.2E-01	7.1E-01	0.94	0.76	1.16	5.1E-01	6.9E-01	1.02	0.82	1.26	8.4E-01	8.7E-01
469	SSC5D	7	MR Egger	1.03	0.93	1.14	5.6E-01	9.3E-01	1.02	0.92	1.14	6.4E-01	8.2E-01	1.14	0.93	1.39	1.6E-01	7.1E-01	1.04	0.66	1.62	8.4E-01	6.9E-01	0.98	0.73	1.3	8.4E-01	8.7E-01
470	SPRR3	3	Inverse variance weighted	1	0.9	1.11	1.0E+00	8.0E-01	0.96	0.83	1.12	6.0E-01	3.7E-01	1.26	1	1.58	4.7E-02	4.4E-01	0.82	0.6	1.12	2.1E-01	4.2E-01	0.95	0.7	1.3	7.6E-01	7.8E-01
470	SPRR3	3	Weighted median	1	0.9	1.11	9.8E-01	8.0E-01	0.97	0.86	1.1	6.8E-01	3.7E-01	1.23	0.98	1.54	6.9E-02	4.4E-01	0.85	0.63	1.16	3.1E-01	4.2E-01	0.95	0.7	1.3	7.5E-01	7.8E-01
470	SPRR3	3	Weighted mode	1.01	0.8	1.29	9.2E-01	8.0E-01	0.99	0.75	1.29	9.4E-01	3.7E-01	1.23	0.73	2.07	3.2E-01	4.4E-01	0.86	0.44	1.67	6.7E-01	4.2E-01	0.93	0.46	1.88	8.6E-01	7.8E-01
470	SPRR3	3	MR Egger	1.03	0.29	3.67	8.9E-01	8.0E-01	1.12	0.26	4.87	5.1E-01	3.7E-01	1.01	0.06	15.75	9.8E-01	4.4E-01	1.14	0.02	52.95	7.5E-01	4.2E-01	0.87	0.02	38.56	7.3E-01	7.8E-01
471	SMPD1	15	Inverse variance weighted	0.95	0.88	1.03	2.2E-01	1.8E-01	0.97	0.89	1.05	4.5E-01	5.3E-01	0.97	0.83	1.13	7.0E-01	2.1E-01	0.82	0.67	1	4.7E-02	3.4E-01	0.99	0.8	1.22	9.1E-01	5.0E-01
471	SMPD1	15	Weighted median	0.97	0.9	1.05	4.3E-01	1.8E-01	0.97	0.89	1.06	4.9E-01	5.3E-01	1.05	0.91	1.23	4.9E-01	2.1E-01	0.85	0.68	1.05	1.3E-01	3.4E-01	0.97	0.78	1.2	7.5E-01	5.0E-01
471	SMPD1	15	Weighted mode	0.97	0.9	1.05	4.4E-01	1.8E-01	0.97	0.88	1.07	5.5E-01	5.3E-01	1.04	0.87	1.25	6.2E-01	2.1E-01	0.85	0.65	1.1	2.0E-01	3.4E-01	0.98	0.76	1.26	8.6E-01	5.0E-01
471	SMPD1	15	MR Egger	1	0.9	1.12	9.4E-01	1.8E-01	1	0.87	1.13	9.4E-01	5.3E-01	1.07	0.85	1.34	5.5E-01	2.1E-01	0.9	0.67	1.22	4.7E-01	3.4E-01	1.06	0.77	1.47	6.9E-01	5.0E-01
472	ANKRD54	1	Wald ratio	0.66	0.44	0.99	4.7E-02	NA	0.7	0.44	1.12	1.4E-01	NA	0.58	0.24	1.41	2.3E-01	NA	0.49	0.14	1.68	2.5E-01	NA	0.85	0.26	2.82	7.9E-01	NA
473	COL1A1	8	Inverse variance weighted	1.02	0.84	1.24	8.4E-01	9.4E-01	1.01	0.84	1.21	9.3E-01	6.7E-01	0.84	0.58	1.23	3.7E-01	9.6E-01	1.54	0.89	2.64	1.2E-01	3.9E-01	1.01	0.63	1.63	9.7E-01	9.7E-01
473	COL1A1	8	Weighted median	1	0.84	1.19	1.0E+00	9.4E-01	1.03	0.83	1.26	8.1E-01	6.7E-01	0.82	0.56	1.19	3.0E-01	9.6E-01	1.69	1.01	2.85	4.7E-02	3.9E-01	1.23	0.72	2.1	4.6E-01	9.7E-01
473	COL1A1	8	Weighted mode	0.95	0.69	1.31	7.2E-01	9.4E-01	0.84	0.58	1.23	3.2E-01	6.7E-01	0.82	0.46	1.46	4.5E-01	9.6E-01	1.61	0.79	3.25	1.5E-01	3.9E-01	1.46	0.68	3.14	2.8E-01	9.7E-01
473	COL1A1	8	MR Egger	1	0.58	1.72	9.9E-01	9.4E-01	0.93	0.55	1.57	7.8E-01	6.7E-01	0.82	0.27	2.53	7.4E-01	9.6E-01	2.34	0.63	8.66	2.2E-01	3.9E-01	0.99	0.25	3.89	9.9E-01	9.7E-01
474	GAL	18	Inverse variance weighted	1.01	0.93	1.1	7.8E-01	9.6E-01	1.01	0.91	1.12	8.8E-01	6.3E-01	1.06	0.89	1.28	5.0E-01	3.6E-01	1.08	0.86	1.35	5.1E-01	1.5E-01	0.85	0.7	1.04	1.2E-01	1.6E-01
474	GAL	18	Weighted median	0.97	0.89	1.06	5.7E-01	9.6E-01	0.99	0.9	1.1	9.2E-01	6.3E-01	0.96	0.78	1.2	7.3E-01	3.6E-01	1.05	0.79	1.39	7.4E-01	1.5E-01	0.77	0.6	1	4.7E-02	1.6E-01
474	GAL	18	Weighted mode	0.97	0.89	1.07	5.6E-01	9.6E-01	0.99	0.89	1.11	9.1E-01	6.3E-01	0.93	0.73	1.18	5.2E-01	3.6E-01	0.99	0.73	1.35	9.5E-01	1.5E-01	0.75	0.57	1	5.1E-02	1.6E-01
474	GAL	18	MR Egger	1.01	0.87	1.17	9.0E-01	9.6E-01	1.04	0.87	1.25	6.4E-01	6.3E-01	0.95	0.69	1.31	7.5E-01	3.6E-01	0.87	0.59	1.27	4.4E-01	1.5E-01	0.7	0.49	1	5.1E-02	1.6E-01
475	CD200	8	Inverse variance weighted	1.1	0.98	1.22	1.1E-01	9.6E-01	1.12	1	1.26	4.7E-02	9.9E-01	1.03	0.83	1.27	7.8E-01	4.5E-01	1.04	0.77	1.4	8.0E-01	5.8E-01	0.86	0.64	1.16	3.3E-01	4.2E-01
475	CD200	8	Weighted median	1.12	0.99	1.27	7.7E-02	9.6E-01	1.13	0.98	1.29	8.9E-02	9.9E-01	0.99	0.76	1.28	9.2E-01	4.5E-01	1.08	0.75	1.56	6.9E-01	5.8E-01	0.85	0.58	1.25	4.2E-01	4.2E-01
475	CD200	8	Weighted mode	1.09	0.93	1.28	2.5E-01	9.6E-01	1.12	0.94	1.35	1.8E-01	9.9E-01	0.99	0.71	1.38	9.2E-01	4.5E-01	1.19	0.76	1.85	3.9E-01	5.8E-01	0.85	0.48	1.51	5.2E-01	4.2E-01
475	CD200	8	MR Egger	1.09	0.84	1.41	4.4E-01	9.6E-01	1.12	0.86	1.46	3.3E-01	9.9E-01	0.91	0.58	1.44	6.4E-01	4.5E-01	1.18	0.62	2.23	5.5E-01	5.8E-01	0.71	0.37	1.37	2.5E-01	4.2E-01
476	CCL16	5	Inverse variance weighted	0.97	0.94	1.01	1.6E-01	3.2E-01	0.98	0.93	1.03	3.8E-01	1.4E-01	0.92	0.85	1	4.8E-02	9.8E-01	1.09	0.97	1.22	1.6E-01	7.6E-01	1.03	0.88	1.19	7.3E-01	2.3E-01
476	CCL16	5	Weighted median	0.98	0.94	1.01	1.8E-01	3.2E-01	0.98	0.94	1.02	3.8E-01	1.4E-01	0.92	0.85	1	4.7E-02	9.8E-01	1.09	0.97	1.22	1.4E-01	7.6E-01	1.02	0.91	1.14	7.3E-01	2.3E-01
476	CCL16	5	Weighted mode	0.98	0.93	1.03	2.6E-01	3.2E-01	0.99	0.92	1.05	5.6E-01	1.4E-01	0.92	0.83	1.03	1.8E-01	9.8E-01	1.09	0.92	1.28	2.3E-01	7.6E-01	1.02	0.87	1.19	8.0E-01	2.3E-01
476	CCL16	5	MR Egger	0.98	0.92	1.05	5.0E-01	3.2E-01	1	0.92	1.08	9.6E-01	1.4E-01	0.92	0.8	1.06	1.7E-01	9.8E-01	1.1	0.89	1.35	2.5E-01	7.6E-01	0.98	0.78	1.24	8.0E-01	2.3E-01
477	ITGAV	4	Inverse variance weighted	1.13	0.98	1.3	8.7E-02	9.6E-01	1.18	1	1.38	4.8E-02	8.4E-01	0.86	0.63	1.16	3.2E-01	8.8E-01	0.99	0.65	1.49	9.4E-01	3.1E-01	1.25	0.83	1.87	2.9E-01	7.4E-01
477	ITGAV	4	Weighted median	1.12	0.96	1.31	1.4E-01	9.6E-01	1.15	0.96	1.38	1.3E-01	8.4E-01	0.83	0.6	1.15	2.7E-01	8.8E-01	1.14	0.72	1.81	5.7E-01	3.1E-01	1.31	0.85	2.02	2.3E-01	7.4E-01
477	ITGAV	4	Weighted mode	1.16	0.86	1.55	2.1E-01	9.6E-01	1.24	0.88	1.75	1.4E-01	8.4E-01	0.82	0.46	1.47	3.6E-01	8.8E-01	1.14	0.5	2.59	6.6E-01	3.1E-01	1.31	0.58	2.95	3.6E-01	7.4E-01
477	ITGAV	4	MR Egger	1.12	0.69	1.84	4.2E-01	9.6E-01	1.14	0.59	2.21	4.7E-01	8.4E-01	0.89	0.24	3.32	7.4E-01	8.8E-01	1.4	0.33	5.9	4.2E-01	3.1E-01	1.38	0.33	5.72	4.4E-01	7.4E-01
478	APOBR	2	Inverse variance weighted	1	0.97	1.02	6.9E-01	NA	1.01	0.96	1.06	6.4E-01	NA	0.94	0.89	1	6.8E-02	NA	0.94	0.73	1.21	6.3E-01	NA	0.93	0.87	1	4.8E-02	NA
479	TIE1	11	Inverse variance weighted	1.03	0.98	1.08	2.0E-01	3.7E-01	1.03	0.98	1.08	2.6E-01	9.6E-01	1.01	0.89	1.15	8.4E-01	2.7E-01	1.03	0.9	1.17	7.2E-01	7.7E-01	0.94	0.81	1.08	3.6E-01	2.8E-01
479	TIE1	11	Weighted median	1.02	0.97	1.07	5.1E-01	3.7E-01	1.03	0.97	1.08	3.1E-01	9.6E-01	0.99	0.89	1.1	8.9E-01	2.7E-01	1.02	0.87	1.18	8.5E-01	7.7E-01	0.85	0.71	1	4.8E-02	2.8E-01
479	TIE1	11	Weighted mode	1.02	0.96	1.08	5.4E-01	3.7E-01	1.03	0.97	1.09	3.1E-01	9.6E-01	0.99	0.88	1.12	9.0E-01	2.7E-01	1	0.84	1.2	9.6E-01	7.7E-01	0.85	0.7	1.03	7.9E-02	2.8E-01
479	TIE1	11	MR Egger	1.01	0.94	1.08	7.3E-01	3.7E-01	1.03	0.95	1.11	4.4E-01	9.6E-01	0.95	0.79	1.15	5.5E-01	2.7E-01	1.01	0.82	1.24	9.4E-01	7.7E-01	0.87	0.7	1.08	1.8E-01	2.8E-01
480	GLA	3	Inverse variance weighted	0.93	0.84	1.02	1.4E-01	5.8E-01	0.91	0.79	1.05	1.8E-01	7.5E-01	0.9	0.74	1.1	2.9E-01	9.6E-01	0.96	0.74	1.25	7.6E-01	8.2E-01	0.84	0.64	1.1	2.2E-01	5.7E-01
480	GLA	3	Weighted median	0.91	0.83	1	5.5E-02	5.8E-01	0.9	0.81	1	4.8E-02	7.5E-01	0.9	0.73	1.11	3.1E-01	9.6E-01	0.97	0.74	1.27	8.4E-01	8.2E-01	0.82	0.62	1.07	1.4E-01	5.7E-01
480	GLA	3	Weighted mode	0.91	0.74	1.12	1.9E-01	5.8E-01	0.89	0.71	1.13	1.7E-01	7.5E-01	0.9	0.56	1.45	4.3E-01	9.6E-01	0.97	0.54	1.77	8.7E-01	8.2E-01	0.82	0.44	1.51	2.9E-01	5.7E-01
480	GLA	3	MR Egger	0.91	0.4	2.04	3.7E-01	5.8E-01	0.89	0.24	3.33	4.7E-01	7.5E-01	0.9	0.22	3.68	5.1E-01	9.6E-01	0.98	0.15	6.26	9.1E-01	8.2E-01	0.8	0.12	5.54	3.8E-01	5.7E-01
481	KLB	9	Inverse variance weighted	0.96	0.92	1	6.7E-02	1.1E-01	0.96	0.92	1.01	1.0E-01	1.6E-01	0.98	0.91	1.05	6.1E-01	3.5E-01	0.94	0.85	1.04	2.2E-01	6.0E-01	0.92	0.8	1.06	2.7E-01	2.6E-01
481	KLB	9	Weighted median	0.97	0.93	1	4.8E-02	1.1E-01	0.97	0.93	1.01	1.4E-01	1.6E-01	0.98	0.91	1.06	6.4E-01	3.5E-01	0.94	0.85	1.05	3.0E-01	6.0E-01	0.97	0.87	1.07	5.4E-01	2.6E-01
481	KLB	9	Weighted mode	0.97	0.93	1.01	9.3E-02	1.1E-01	0.97	0.93	1.02	2.3E-01	1.6E-01	0.99	0.91	1.07	7.5E-01	3.5E-01	0.94	0.83	1.07	2.9E-01	6.0E-01	0.95	0.84	1.08	3.8E-01	2.6E-01
481	KLB	9	MR Egger	0.99	0.93	1.05	7.7E-01	1.1E-01	0.99	0.92	1.07	8.0E-01	1.6E-01	1.01	0.9	1.14	7.8E-01	3.5E-01	0.95	0.81	1.15	6.3E-01	6.0E-01	1	0.8	1.25	9.9E-01	2.6E-01
482	KAZALD1	5	Inverse variance weighted	1.02	0.9	1.15	7.7E-01	4.1E-01	1.01	0.86	1.18	9.2E-01	5.6E-01	1.02	0.91	1.14	7.2E-01	4.1E-01	1.12	0.99	1.27	6.9E-02	2.2E-01	1.03	0.87	1.22	7.2E-01	7.3E-01
482	KAZALD1	5	Weighted median	1.03	0.99	1.07	1.5E-01	4.1E-01	1.02	0.97	1.07	4.3E-01	5.6E-01	1.03	0.94	1.12	5.1E-01	4.1E-01	1.14	1	1.29	4.8E-02	2.2E-01	1.04	0.92	1.17	5.3E-01	7.3E-01
482	KAZALD1	5	Weighted mode	1.03	0.97	1.09	2.7E-01	4.1E-01	1.02	0.96	1.09	4.4E-01	5.6E-01	1.03	0.9	1.18	5.9E-01	4.1E-01	1.14	0.95	1.37	1.1E-01	2.2E-01	1.04	0.88	1.23	5.3E-01	7.3E-01
482	KAZALD1	5	MR Egger	1.05	0.84	1.3	5.4E-01	4.1E-01	1.04	0.76	1.4	7.4E-01	5.6E-01	1.05	0.85	1.29	5.2E-01	4.1E-01	1.18	0.94	1.48	1.0E-01	2.2E-01	1.05	0.74	1.49	6.8E-01	7.3E-01
483	SDHB	5	Inverse variance weighted	0.9	0.76	1.06	2.1E-01	1.8E-01	0.94	0.78	1.14	5.5E-01	7.7E-01	0.9	0.49	1.65	7.2E-01	6.7E-02	0.64	0.39	1.05	7.6E-02	5.4E-01	0.86	0.42	1.77	6.8E-01	5.5E-01
483	SDHB	5	Weighted median	0.84	0.69	1.03	9.7E-02	1.8E-01	0.93	0.74	1.16	4.9E-01	7.7E-01	0.63	0.4	1	4.8E-02	6.7E-02	0.66	0.37	1.19	1.7E-01	5.4E-01	0.73	0.4	1.34	3.1E-01	5.5E-01
483	SDHB	5	Weighted mode	0.82	0.6	1.11	1.4E-01	1.8E-01	0.92	0.65	1.3	5.4E-01	7.7E-01	0.64	0.33	1.25	1.4E-01	6.7E-02	0.69	0.25	1.92	3.7E-01	5.4E-01	0.75	0.32	1.77	4.1E-01	5.5E-01
483	SDHB	5	MR Egger	0.62	0.3	1.28	1.3E-01	1.8E-01	0.87	0.38	2	6.4E-01	7.7E-01	0.23	0.05	1.19	6.6E-02	6.7E-02	0.99	0.11	8.85	9.9E-01	5.4E-01	0.44	0.01	13.44	5.0E-01	5.5E-01
484	VCAM1	9	Inverse variance weighted	0.95	0.75	1.2	6.5E-01	1.5E-01	0.99	0.74	1.33	9.5E-01	2.6E-01	0.82	0.63	1.07	1.4E-01	2.9E-01	1.34	0.94	1.9	1.0E-01	7.9E-01	0.73	0.49	1.09	1.3E-01	3.8E-01
484	VCAM1	9	Weighted median	1.05	0.9	1.23	5.6E-01	1.5E-01	1.14	0.96	1.36	1.3E-01	2.6E-01	0.84	0.6	1.16	2.9E-01	2.9E-01	1.28	0.81	2.01	2.9E-01	7.9E-01	0.6	0.37	1	4.8E-02	3.8E-01
484	VCAM1	9	Weighted mode	1.05	0.88	1.26	5.4E-01	1.5E-01	1.14	0.92	1.42	2.1E-01	2.6E-01	0.83	0.54	1.27	3.4E-01	2.9E-01	1.19	0.63	2.24	5.9E-01	7.9E-01	0.49	0.2	1.22	1.4E-01	3.8E-01
484	VCAM1	9	MR Egger	1.27	0.78	2.09	2.9E-01	1.5E-01	1.34	0.68	2.61	3.4E-01	2.6E-01	1.05	0.57	1.94	8.4E-01	2.9E-01	1.45	0.65	3.25	3.1E-01	7.9E-01	1	0.4	2.5	9.9E-01	3.8E-01
485	FCRL5	11	Inverse variance weighted	0.91	0.82	1.02	1.1E-01	6.2E-01	0.88	0.78	1	4.9E-02	9.5E-01	0.95	0.71	1.27	7.1E-01	7.1E-01	0.93	0.64	1.35	7.0E-01	6.5E-01	0.97	0.7	1.35	8.6E-01	7.5E-01
485	FCRL5	11	Weighted median	0.96	0.83	1.11	5.9E-01	6.2E-01	0.87	0.74	1.02	9.2E-02	9.5E-01	0.94	0.68	1.3	7.2E-01	7.1E-01	0.83	0.54	1.29	4.2E-01	6.5E-01	1.01	0.67	1.53	9.5E-01	7.5E-01
485	FCRL5	11	Weighted mode	0.96	0.77	1.19	6.7E-01	6.2E-01	0.86	0.69	1.07	1.6E-01	9.5E-01	1.04	0.67	1.59	8.6E-01	7.1E-01	0.74	0.49	1.49	5.5E-01	6.5E-01	1.15	0.62	2.12	6.2E-01	7.5E-01
485	FCRL5	11	MR Egger	0.87	0.69	1.1	2.3E-01	6.2E-01	0.87	0.67	1.15	2.9E-01	9.5E-01	1.04	0.54	1.99	9.0E-01	7.1E-01	0.8	0.35	1.84	5.6E-01	6.5E-01	0.89	0.45	1.78	7.2E-01	7.5E-01
486	FOXO1	3	Inverse variance weighted	0.98	0.81	1.19	8.7E-01	4.8E-01	0.95	0.8	1.14	6.0E-01	4.3E-01	0.98	0.65	1.49	9.3E-01	8.7E-01	1.17	0.72	1.92	5.3E-01	9.5E-01	1.57	1	2.47	4.9E-02	6.3E-01
486	FOXO1	3	Weighted median	0.92	0.77	1.11	3.9E-01	4.8E-01	0.91	0.73	1.12	3.7E-01	4.3E-01	1	0.66	1.52	9.9E-01	8.7E-01	1.09	0.63	1.91	7.5E-01	9.5E-01	1.71	0.99	2.95	5.4E-02	6.3E-01
486	FOXO1	3	Weighted mode	0.91	0.59	1.41	4.6E-01	4.8E-01	0.89	0.51	1.56	4.7E-01	4.3E-01	1.07	0.3	3.87	8.4E-01	8.7E-01	0.92	0.16	5.25	8.6E-01	9.5E-01	1.81	0.42	7.77	2.2E-01	6.3E-01
486	FOXO1	3	MR Egger	0.78	0.04	15.23	4.9E-01	4.8E-01	0.74	0.04	12.31	4.1E-01	4.3E-01	0.86	0	7717.5	8.7E-01	8.7E-01	1.25	0	67358.03	8.4E-01	9.5E-01	2.29	0	6039.03	4.1E-01	6.3E-01
487	YAP1	4	Inverse variance weighted	0.9	0.79	1.04	1.6E-01	3.8E-01	0.91	0.78	1.06	2.4E-01	5.5E-01	0.8	0.59	1.07	1.3E-01	3.3E-01	0.96	0.65	1.42	8.3E-01	4.1E-01	0.87	0.59	1.29	5.0E-01	8.7E-01
487	YAP1	4	Weighted median	0.87	0.75	1	5.3E-02	3.8E-01	0.88	0.74	1.04	1.3E-01	5.5E-01	0.73	0.53	1	4.9E-02	3.3E-01	1.2	0.74	1.94	4.7E-01	4.1E-01	0.89	0.58	1.36	5.8E-01	8.7E-01
487	YAP1	4	Weighted mode	0.87	0.68	1.11	1.6E-01	3.8E-01	0.87	0.65	1.16	2.1E-01	5.5E-01	0.74	0.44	1.25	1.7E-01	3.3E-01	1.2	0.47	3.03	5.8E-01	4.1E-01	0.9	0.4	2.02	7.0E-01	8.7E-01
487	YAP1	4	MR Egger	0.81	0.5	1.34	2.2E-01	3.8E-01	0.84	0.44	1.58	3.5E-01	5.5E-01	0.62	0.22	1.75	1.9E-01	3.3E-01	0.73	0.17	3.06	4.4E-01	4.1E-01	0.92	0.22	3.85	8.3E-01	8.7E-01
488	PROK1	4	Inverse variance weighted	1.02	0.95	1.09	6.1E-01	9.1E-01	0.98	0.9	1.07	6.5E-01	8.3E-01	1.16	0.99	1.35	5.8E-02	7.4E-01	1.11	0.89	1.38	3.6E-01	3.3E-01	1.18	0.92	1.52	1.8E-01	2.3E-01
[truncated: 47,050 more chars]
